# Lysozyme-Induced Transcriptional Regulation of TNF-α Pathway Genes in Cells of the Monocyte Lineage

**DOI:** 10.3390/ijms20215502

**Published:** 2019-11-05

**Authors:** Alberta Bergamo, Marco Gerdol, Alberto Pallavicini, Samuele Greco, Isabelle Schepens, Romain Hamelin, Florence Armand, Paul J. Dyson, Gianni Sava

**Affiliations:** 1 Callerio Foundation Onlus, 34127 Trieste, Italy; gsava@units.it; 2 Department of Life Sciences, University of Trieste, 34127 Trieste, Italy; mgerdol@units.it (M.G.); pallavic@units.it (A.P.); samuele.greco@phd.units.it (S.G.); 3 Proteomics Core Facility, Ecole Polytechnique Fédérale de Lausanne (EPFL), CH-1015 Lausanne, Switzerland; isaschepens@gmail.com (I.S.); romain.hamelin@epfl.ch (R.H.); florence.armand@epfl.ch (F.A.); 4 Institut des Sciences et Ingénierie Chimique, Ecole Polytechnique Fédérale de Lausanne (EPFL), CH-1015 Lausanne, Switzerland; paul.dyson@epfl.ch

**Keywords:** lysozyme, inflammation, RNA-sequencing, whole transcriptome profiling, proteome profiling, TNF-α, IL-1β

## Abstract

Lysozyme is one of the most important anti-bacterial effectors in the innate immune system of animals. Besides its direct antibacterial enzymatic activity, lysozyme displays other biological properties, pointing toward a significant anti-inflammatory effect, many aspects of which are still elusive. Here we investigate the perturbation of gene expression profiles induced by lysozyme in a monocyte cell line in vitro considering a perspective as broad as the whole transcriptome profiling. The results of the RNA-seq experiment show that lysozyme induces transcriptional modulation of the TNF-α/IL-1β pathway genes in U937 monocytes. The analysis of transcriptomic profiles with IPA^®^ identified a simple but robust molecular network of genes, in which the regulation trends are fully consistent with the anti-inflammatory activity of lysozyme. This study provides the first evidence in support of the anti-inflammatory action of lysozyme on the basis of transcriptomic regulation data resulting from the broad perspective of a whole-transcriptome profiling. Such important effects can be achieved with the supplementation of relatively low concentrations of lysozyme, for a short time of exposure. These new insights allow the potential of lysozyme in pharmacological applications to be better exploited.

## 1. Introduction

Almost a century after its discovery by sir Alexander Fleming [1], lysozyme is now widely recognized as one of the most important anti-bacterial effectors in the innate immune system of animals [2,3], where it plays a key role in response to infection, by digesting peptidoglycan and destabilizing the cell wall of invading Gram-positive bacteria [4]. Over the years, lysozyme has become more and more important for studies in the fields of protein chemistry, enzymology, crystallography and molecular biology [5], and it is widely used as a preservative/maturation inductor in foods and pharmaceuticals [6,7,8].

The lysozyme muramidasic enzymatic activity is provided by three different types of evolutionarily unrelated gene-encoded proteins, named chicken (C)-, goose (G)- and invertebrate (I)-type lysozymes, which display a broad and partly overlapping taxonomic distribution [5]. Lysozymes are produced by a variety of cell types and tissues, and are highly abundant in saliva, mucus and other secretions. The human protein, encoded by the *LYZ* gene, pertains to the conventional C-type class and shares high homology (58% identity) with the chicken lysozyme. 

The oral administration of lysozymes is an attractive proposition, as it could help to suppress the spread of gastrointestinal pathogens, or to modulate the composition of the entire gut microbiota [9,10]. Besides its direct antibacterial enzymatic activity, lysozyme displays other promising biological properties, which may enable its therapeutic use as an immune-modulating agent in the near future. For example, lysozyme has remarkable nucleic acid-binding properties, which may explain its antiviral activity toward HIV-1 [11,12,13]. Moreover, the ability to stimulate the recovery of lymphocyte CD4+/CD8+ ratio in patients in remission after chemotherapy for malignant diseases [14], and to beneficially act in the context of human ageing [15], further highlights the multifaceted immune-modulating properties of this natural product. Lysozyme supplementation can also reduce the concentration of serum advanced glycation end products (AGEs) and their deposition in the kidneys of early-stage diabetic rats [16,17], preventing the development of glomerular and renal hypertrophy as well as the overexpression of AGE receptors (RAGEs) [18]. 

Recently, a series of immune-histochemical studies have indicated that lysozyme is up-regulated in the gastrointestinal (GI) tract of patients affected by pathologies characterized by chronic inflammation. These include Barrett’s oesophagitis, chronic gastritis, gluten-induced atrophic duodenitis (coeliac disease), collagenous colitis, lymphocytic colitis, ulcerative colitis (UC) and Crohn’s colitis [19,20,21,22]. This suggests that this enzyme may be somehow involved in counteracting the development of the hostile gut microenvironment typical of these pathologies. In support to these observations, the treatment with exogenous hen egg-white lysozyme (HEWL) can significantly reduce the local expression of pro-inflammatory cytokines and increase the expression of anti-inflammatory mediators in a porcine model of colitis, induced by dextran sodium sulphate (DSS), suggesting a role in the restoration of gut homeostasis [23]. Moreover, the supplementation of exogenous HEWL has a significant anti-inflammatory effect in subjects affected by inflammatory bowel disease [23].

This behaviour may be also linked to the lipopolysaccharide (LPS)-binding properties of lysozyme, which have been reported to abrogate TNF-α (tumour necrosis factor alpha) production and to reduce LPS-related mortality rates in an endotoxin shock murine model [24,25]. Other studies have evidenced that the anti-inflammatory effect of lysozyme is linked with an inhibition of serum complement activity [26]. Anti-septic effects have also been reported for PEGylated lysozyme in high mobility group B1-mediated inflammatory response, both in vitro and in vivo [27]. Moreover, the application of a charge-engineered lysozyme variant in a murine model of mucoid *Pseudomonas aeruginosa* lung infection was able to contrast both the burden of infection and the degree of tissue inflammation [28]. 

The wide number of biological roles of lysozyme and the significant effects against bacterial and viral infections, depression of immune response and phlogosis, led to the commercialization and employment of HEWL in a number of human pathologies, in which its use is consolidated and safe, since the 1950 (in addition to the indications for which marketing in various countries is authorized, see also: [29,30,31]). The therapeutic effectiveness of lysozyme to control the growth of susceptible bacteria is based on a generally well-known mechanism of action although some aspects, such as the impact of the peptidoglycan fragments released by the catalytic action, and the muramidase-independent properties, remain elusive [32,33]. The immune-regulating and anti-inflammatory properties of lysozyme, its ability to beneficially act in the context of human ageing [15], or to contrast the complications of chronic diseases like diabetes [16,17,18], to cite a few, are more recent indications of its importance as they highlight the potential use of lysozyme as a natural complement to other previously established pharmacological interventions. How lysozyme work to carry out these corollary activities is much less known. Regardless of the potential application of lysozyme, a deeper understanding of the cellular and molecular mechanisms implemented by this molecule is fundamental to improve the appropriateness of its use. 

The interaction of lysozyme with immune system cells is pivotal to all its biological and pharmacological effects. Although numerous in vitro and in vivo studies have analysed the mechanisms of action of lysozyme at the molecular level, to the best of our knowledge none has so far considered a perspective as broad as whole transcriptome profiling. Such an approach would ensure a fuller overview on the interactions between lysozyme and target cells, allowing the mechanisms of action and regulatory networks not easily predictable on the basis of current knowledge to be established. With this study, we aim to fill this gap, investigating the perturbation of gene expression profiles induced by *in vitro* lysozyme supplementation in a monocyte cell line, one of the main types of immune cells which interact with lysozyme. The chosen cellular model is the monocyte U937 cell line, which still expresses many of the monocytic-like characteristics and is at the same time genetically homogeneous and easily manageable. In addition, the possibility to differentiate U937 cells to macrophages enables the effects of lysozyme on two maturation stages of the same cellular lineage to be studied [34]. Through an in-depth analysis of the genes altered in response to the treatment, we expect to provide a rationale for an improved understanding of the molecular mechanisms underpinning the diverse pharmacological activities of lysozyme.

## 2. Results

The highest number of differentially expressed genes (DEGs) in the U937 monocyte cell line was detected at the end of the treatment with lysozyme for 1 h (3 up- and 22 down-regulated genes, Table 1).

The number of DEGs, compared to parallel control samples declined after a further 2 h in a lysozyme-free culture medium (2 up- and 1 down-regulated genes), and was similar to that observed in response to the non-stop treatment for 24 h (1 up- and 5 down-regulated genes). Overall lysozyme affected the expression level of few genes, although some very meaningful fold change values indicated the possibility of prominent consequences at a functional level. The complete list of DEGs and their relative fold change values are reported in Table 2.

**Figure 1:**
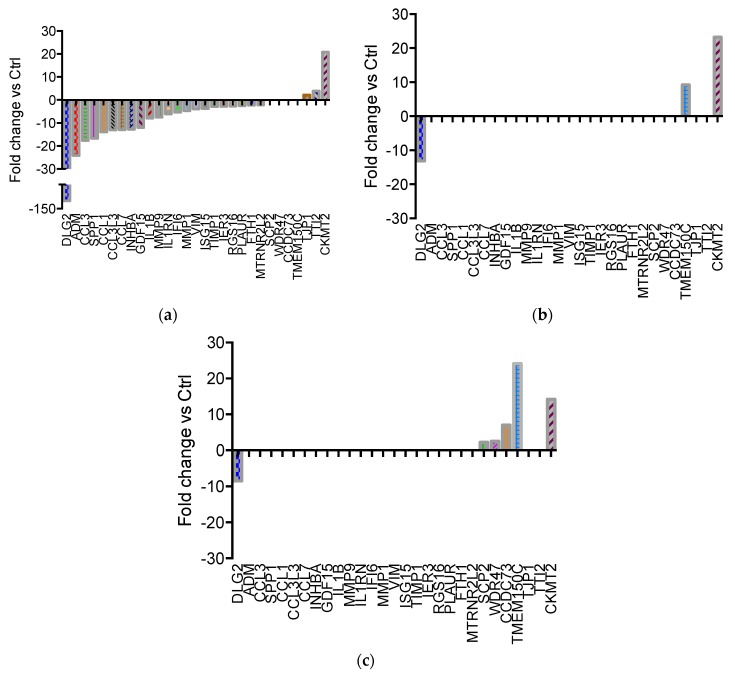
Differentially expressed genes in U937 cells treated with lysozyme 15 μg/mL for 1 h and analysed immediately at the end of the treatment (1 h, **a**) or 2 h after the end of the treatment (1 h + 2, **b**), or treated for 24 h and analysed immediately at the end of the treatment (24 h, **c**). Fold change values in respect of parallel control samples are shown in ascending order. Complete gene expression data is available in Supplementary Table S1.

Overall, the functions of most of the DEGs detected at the end of the exposure to lysozyme for 1 h were linked with immune system activity (26.5%), signal transduction (16.3%), metabolism (12.2%) and extracellular matrix organisation (8.2%) (Figure S1, Supplementary Material). At this time point, the majority of DEGs (88%) were down-regulated (Figure 1a; Figures S2 and S3, Supplementary Material).

**Figure 2:**
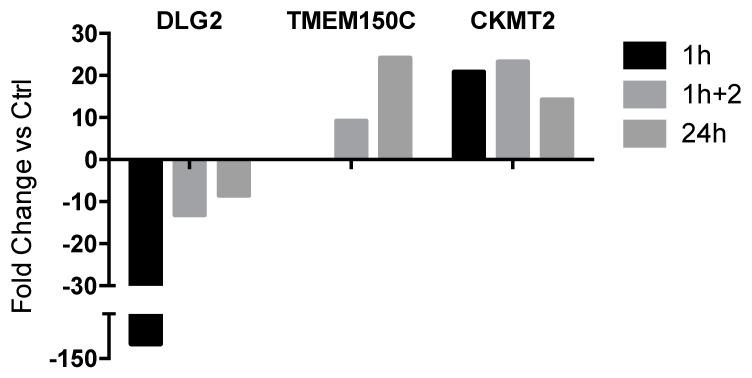
Time-trends of the three genes differentially expressed recurring at any time of analysis in U937 cells.

Many genes showed, compared to control samples, relevant fold change values that were in turn no longer detectable in cells kept for an additional 2 h in a lysozyme-free culture medium (Figure 1b; Figures S4 and S5, Supplementary Material), or in cells subjected to the non-stop 24 h treatment (Figure 1c; Figures S6 and S7, Supplementary Material), highlighting the transient nature of the alterations observed. The only genes that maintained a significant variation of expression at all times of investigation were *DLG2* (Discs Large MAGUK Scaffold Protein 2) and *CKMT2* (Creatine Kinase, Mitochondrial 2), which remained down- and up-regulated, respectively, even though the magnitude of the alteration of *DLG2* decreased over time (Figure 2). Another relevant DEG, *TMEM150C* (Transmembrane Protein 150C), showed a delayed over-expression, undetectable at the first time point of analysis, but highly significant in the later ones, regardless of the presence of lysozyme in the cell culture medium (Figure 2).

Ingenuity pathway analysis (IPA^®^) was then used to investigate the possible mode of action and physiological effects of lysozyme treatment in U937 cells, focusing in particular on the analysis of DEGs detected at 1 h, since this was the experimental time point displaying the most relevant alterations of gene expression profiles.

**Figure 3:**
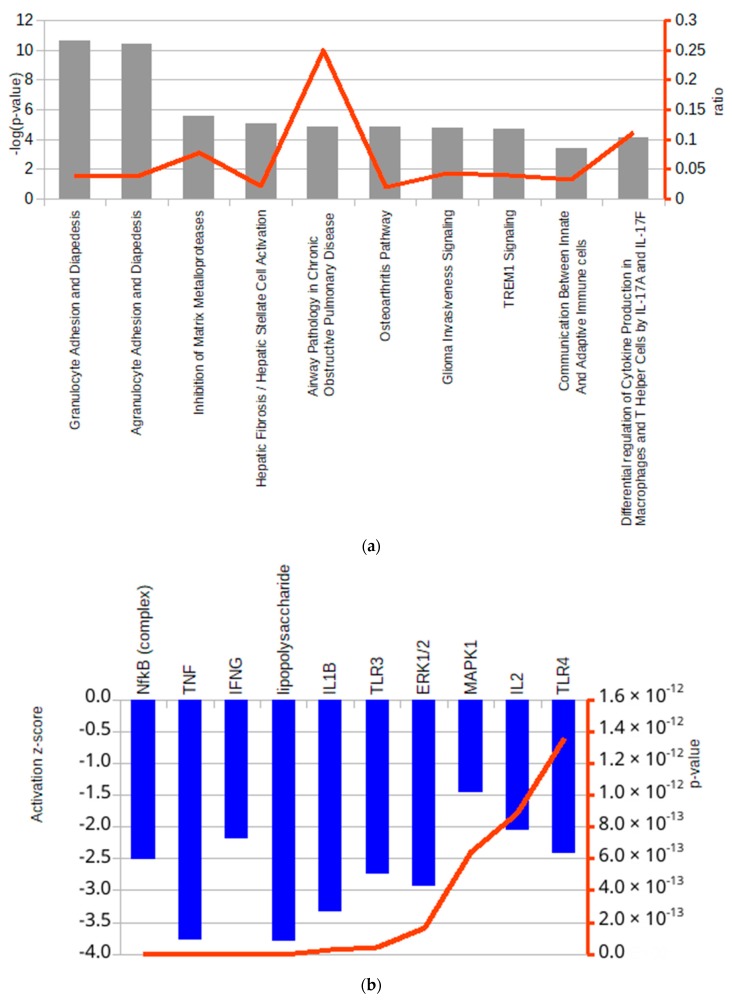
Top ten canonical pathways (**a**) and top ten up-stream regulators (**b**) detected as altered by IPA^®^ analysis. In (**a**) the ‘ratio’ value is the ratio between altered genes and total genes in the pathway, and in (**b**) bars represent the pathway z-score, the red line indicates the *p*-value of the prediction.

The pathway analysis showed significant alterations of canonical pathways that are involved in adhesion and diapedesis of granulocytes and agranulocytes (Figure 3a). The analysis of upstream regulators, which may possibly underpin the observed alterations, identified a strong correlation between the transcriptional profiles and the inhibition of *NF-κB* (nuclear factor kappa-light-chain-enhancer of activated B cells), *TNF-α* (tumour necrosis factor alpha), *IFNG* (interferon gamma), *IL-1* (interleukin 1) and also of other genes involved in the regulation of inflammatory processes (Figure 3b), suggesting that the anti-inflammatory effect exerted by lysozyme might be mediated by these proteins. The most likely affected downstream biological pathways were predicted to act in an inhibitory manner in the immune cell trafficking and inflammatory response. Conversely, a single process, i.e., cell death, was predicted to be slightly up-regulated (not shown). It is important to note that, based on the list of DEGs, no significant toxic effect was predicted by IPA^®^ neither at the level of the whole organism nor of a specific tissue. 

In order to explore in greater detail the possible physiological effects of lysozyme treatment, a custom gene regulatory network was built using *CKMT2* and *DLG2* as probes, i.e., the two genes displaying the most significant and long-lasting alterations. The two genes were used to search, within the ingenuity knowledge base, any possible connecting path to the other detected DEGs.

**Figure 4:**
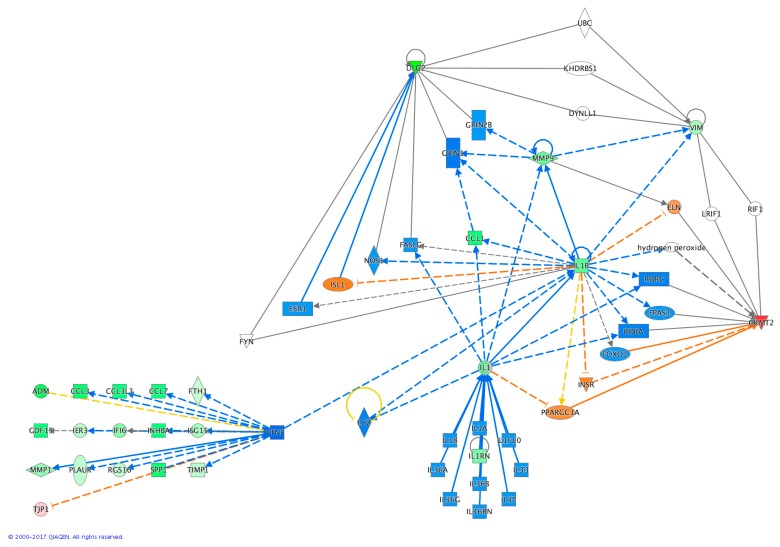
IPA^®^ analysis of signalling pathways of the genes differentially expressed upon lysozyme treatment. U937 cells were treated with lysozyme 15 μg/mL for 1 h and analysed immediately at the end of the treatment (1 h). Down-regulated genes by lysozyme treatment are marked in green, up-regulated genes are marked in red, orange colour indicates the prediction of an activation, blue colour the prediction of an inhibition; continuous line indicates direct relationships, dashed line indicates an indirect relationship (one or more intermediates), yellow colour indicates relationships non-consistent with the state of the down-stream molecule, grey colour indicates an unpredicted effect.

The resulting gene regulatory network clearly showed a central role for *TNF*-α and *IL-1*, while *CKMT2* and *DLG2* were placed in peripheral regions, suggesting that these two genes have a marginal role in the direct response to lysozyme. However, their marked and long-lasting differential expression indicates that these two genes may be used as potential molecular markers to track the effect of lysozyme treatment, at least within the first 24 h (Figure 4). 

It is worth noting that *TNF*-α was not found among the significantly down-regulated DEGs in the RNA-seq experiment, even though IPA^®^ predicted its inhibition with high confidence because of the observed down-regulation of several genes under direct positive transcriptional control by *TNF*-α itself. However, while the levels of *TNF*-α mRNA remained steady throughout the experimental timeline, the inhibition of *TNF*-α activity could possibly occur at the protein level (e.g., by increased protein turnover or by post-translational modifications). Moreover, *TNF*-α may be a highly responsive gene, whose expression levels vary very rapidly in response to the treatment with lysozyme, vanishing within a few minutes after the treatment and becoming undetectable at the time of analysis. In contrast to *TNF*-α, whose levels do not vary, *IL*-1β (interleukin 1 beta) displays a strong transcriptional repression with a −7.99-fold change value at 1 h in comparison to untreated cells (Figure 1a and Table 2).

LC-MS/MS (liquid chromatography-tandem mass spectrometry) analysis identified and quantified proteins in U937 cells, collected 24 h after exposure to lysozyme 15 μg/mL for 1 h (hereafter referred to as 1 h + 24) or for consecutive 24 h (hereafter referred to as 24 h + 24). The differences of protein abundance comparing the two conditions and of their statistical significance are shown by volcano plots constructed by plotting the Log2 ratio on x-axis for each quantified protein and the significance of the calculated difference as ‘−log10(*p*-value)’ on y-axis. Dashed lines mark different thresholds of statistical significance of the difference. Protein spots represented above the dashed lines show a significant difference in the two samples.

**Figure 5:**
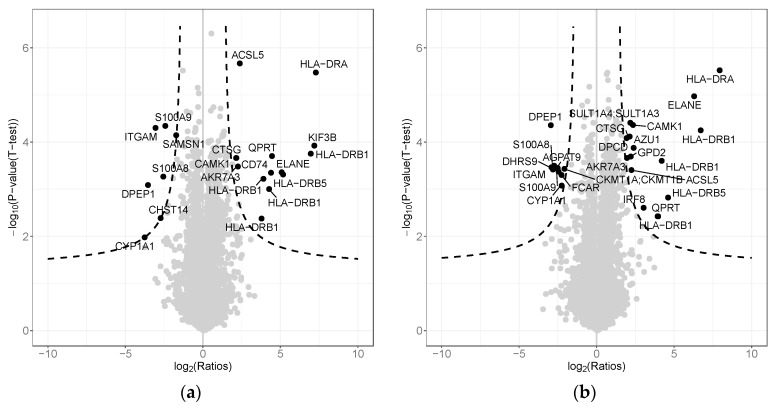
LC-MS/MS (liquid chromatography-tandem mass spectrometry) analysis, volcano plots show the differences of protein abundance comparing the control cells at 24 h + 24 vs. 1 h + 24 (**a**), and lysozyme-treated cells at 24 h + 24 vs. 1 h + 24 (**b**). Dashed lines mark the threshold of statistical significance with FDR < 0.05.

**Figure 6:**
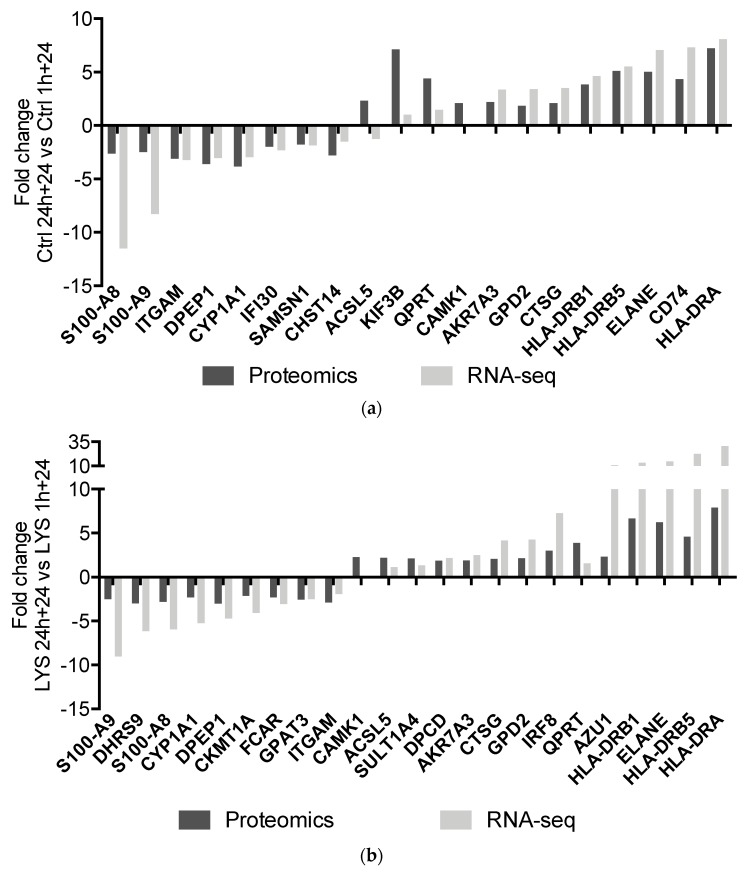
Correlation between the differential expression of proteins and of mRNAs in the control 24 h + 24 versus control 1 h + 24 (**a**) and in LYS 24 h + 24 versus LYS 1 h + 24 (**b**) in U937 cells.

No significant differences were observed between the lysozyme-treated and the relevant controls in both experimental conditions of treatment (1 h + 24 or 24 h + 24) (Figures S8 and S9 in the Supplementary Material). Conversely, significant different protein levels were observed when comparing the two experimental schemes in the controls (Table 3 and Figure 5a) or in the treated cells (Table 4 and Figure 5b), probably because of the different growth time of the cells in the two experimental settings. Table 3 compares control 24 h + 24 versus control 1 h + 24; 8 proteins are significantly down-regulated and 15 proteins are significantly up-regulated. A similar finding characterizes the comparison between lysozyme 24 h + 24 versus lysozyme 1 h + 24 (Table 4), where the proteins down- and up-regulated are 9 and 16, respectively. Most of the proteins differentially expressed are common to control and lysozyme-treated cells and are marked with S (=shared) in Table 3 and Table 4. That is likely a result related to the time chosen for the samples collection after the end of treatment, more than an effect of the treatment itself. The comparison of the results of the proteomic and the RNA-seq analyses highlighted a good correlation between the expression of mRNAs and the detected abundance of encoded proteins both in controls (Figure 6a) and in lysozyme-treated U937 cells (Figure 6b). The vast majority of down- or up-regulated proteins showed an identical trend of expression at the mRNA level, even though the fold change values observed in RNA-seq experiments were often more pronounced than their detected fold change at the proteomic level.

## 3. Discussion

Almost a century after its discovery, lysozyme still attracts considerable interest, and remains the subject of many research projects. Because of the well-established role of lysozyme in anti-bacterial defence, it has become a paradigm for the functioning of the innate immune system. Nevertheless, the precise contribution of lysozyme to antibacterial defence in different tissues is still lacking and many aspects concerning its non-catalytic biological role are not completely understood. An interesting development concerns the possibility to attribute to lysozyme additional functions, besides antibacterial defence, such as a potent anti-inflammatory activity [29,35,36]. Lysozyme significantly suppresses the production of the inflammatory cytokines TNF-α, IL-1β and IL-6 (Interleukin 6) [37,38,39], independently from muramidasic activity, because of the peptide motifs found in the N-terminal region of the protein [40]. Furthermore, Gallo et al. [41] showed that lysozyme is able to prevent the production and release of inflammatory mediators, such as IL-6, and to reduce macrophage recruitment in the inflammatory site induced by AGE (advanced glycation endproducts) in HK-2 proximal tubular epithelial cells. However, the detailed mechanism of this action and the exact molecular basis underpinning this behaviour are still undefined.

To gain an improved molecular basis for the many activities of this multi-tasking protein, we decided to investigate the transcriptional effects of the supplementation of very low concentrations of HEWL (15 μg/mL ≈ 1 μM) on a monocyte cell line. Our approach takes advantage of the exploratory capacity of whole RNA-sequencing analysis, integrating transcriptome analysis with proteome profiling, and allowing a comprehensive overview of the molecular alterations occurring in this experimental system.

The results of the RNA-seq analysis showed a limited effect on gene expression, with a small number of DEGs detected at all time points, which generally displayed modest fold change values. The only exceptions were the *DLG2* and *CKMT2* genes, which were differentially expressed in all the experimental conditions tested and displayed a particularly high fold change value in U937 cells collected immediately after the end of exposure to lysozyme (1 h). *DLG2* (discs large MAGUK scaffold protein 2), encoding a member of the MAGUK family of membrane-associated guanylate kinases with a role in protein–protein interactions at synapses and in tight junctions pathways was significantly down-regulated in response to HEWL treatment. By contrast, *CKMT2*, encoding a mitochondrial creatine kinase responsible for the high-energy phosphate transfer from mitochondria to the cytosol carrier creatine, was significantly over-expressed. The significant changes of the expression of these two genes do not permit, by themselves, to establish a functional hypothesis on the biological activity of lysozyme on monocyte cells. We opted for an in-depth analysis of the set of DEGs and of their modulation at 1-h exposure to lysozyme, which allowed us to formulate biological hypotheses consistent with the strong modulation of *DLG2* and *CKMT2*. Indeed, at this time point, a number of DEGs were significantly altered in addition to *DLG2* and *CKMT2*, but these transient regulatory effects were practically eliminated after 2 and 24 h. 

Data from the literature show that significant alterations of *TNF-α* and *IL-6* gene expression can be detected by RT-PCR after treatment with lysozyme in LPS-activated peritoneal macrophages at 500 μg/mL (approximately 35 μM) [38]. The effective anti-inflammatory concentrations of lysozyme-derived peptides are in the range 25–50 μM, a concentration in line with the levels of lysozyme in breast milk [40]. Therefore, the limited intensity and the low persistence of the effects on the transcriptome of the treated cells might depend on the much lower concentration of lysozyme used in this study (1 μM). We may hypothesize that the transcriptional and proteomic alterations detected may involve a greater number of genes, persist for a longer time span or affect with higher intensity cells exposed to higher HEWL doses. However, the present study highlights that a concentration as low as 1 μM can already exert a transient response in treated cells, suggesting a wide spectrum of active doses for this natural product. 

The DEGs were then subjected to IPA^®^ to build a regulatory network based on the literature data contained in the IPA^®^ Knowledge Base. The highly significant findings that emerged from the comparison between treated and untreated U937 cells after 1 h, revealed a series of canonical pathways whose activity was predicted as ‘altered’. From these, some showed a clear directionality, while others were discarded because they were not relevant for the biological context examined in this study. The regulatory network built with the IPA^®^ was based on *CKMT2* and *DLG2* as hinge points because of their extremely significant fold change values and the persistence of their alteration at 1 h and up to 24 h. The RNA-seq results show that *MMP-9* (matrix metallopeptidase 9), *CCL1* (chemokine ligand 1), *IL-1B*, and *IL-1* genes, belonging to the regulation networks of *DLG2* and *CKMT2*, were also down-regulated at 1 h, suggesting a very rapid onset of the cell response to lysozyme. *IL-1* and *IL-1B*, among the up-stream regulators predicted to be most likely involved in response to lysozyme form a central node in the network because of their connections with several other differentially expressed genes. Despite its prediction as a key repressed upstream regulator in lysozyme response, the *TNF-α* gene was not differentially expressed at the transcriptional level. The important role of *TNF-α* at the crossroads of regulation of a number of the other DEGs detected in this study is strongly supported by the high number of relationships in the IPA^®^ regulatory network (Figure 4). Moreover, literature data have previously demonstrated that lysozyme can strongly inhibit the transcription of *TNF-α* and *IL-1β* [24,25,42]. It is noteworthy that many of the down-regulated genes under the transcriptional control of *TNF-α* are involved in inflammatory processes. *CCL3* (chemokine ligand 3), known also as macrophage inflammatory protein 1-alpha, is a cytokine that activates polymorphonucleate leucocytes, which is also involved in the acute phase of inflammation [43]. CCL7 (chemokine ligand 7) is a chemokine that regulates macrophage function [44]. CCL3L3 (chemokine ligand 3-like 3), a cytokine with high similarity to CCL3 is implicated in the regulation of immune cell functions and is up-regulated during an inflammatory response [45,46]. Immediate early response 3 (IER3) acts in the immediate response mechanism and protects cells from Fas and TNF-α-induced apoptosis [47]. ISG15 (interferon-stimulated gene 15) is a ubiquitin-like protein with chemotactic function that recruits neutrophils in the sites of inflammation [48]. Regarding the lack of regulation of the *TNF-α* gene, it is essential to stress that RNA-seq data can only provide indications on the biological layer related to transcription. However, another order of dynamicity is added by post-transcriptional and post-translational regulation of gene expression. Several other factors like the regulation of translation, protein turnover and the regulation of its activity by post-translational modifications, including the interaction with co-factors, can together modulate the biological activity of a protein without the appreciable involvement of modifications at the mRNA level. Furthermore, a very recent study conducted in mice with LPS-induced systemic inflammation reports the significant suppression of TNF-α protein levels in serum and spleen after oral administration of lysozyme but, in contrast *TNF-α* gene expression resulted correspondingly not affected [39].

Overall, the analysis of the RNA-seq data suggests that, independently of the rapid onset and fading of the anti-inflammatory activity exerted by lysozyme, HEWL treatment results in a long-lasting alteration of the *DLG2* and *CKMT2* genes, which could be considered as molecular signature of the lysozyme activity that takes place. Further support of this hypothesis is given by the analysis of the effects of lysozyme on U937 cells differentiated to macrophages. Indeed, the whole RNA-seq experiment was specifically designed considering both monocytes and macrophages as cellular models, in order to investigate the effects of lysozyme on two maturation stages of the same cellular lineage. Accordingly, the experiments were conducted both with U937 monocytes as such, and after their differentiation into macrophages upon treatment with PMA (phorbol-12-myristate-13-acetate) 50 ng/mL for 72 h before the exposure to lysozyme. Surprisingly, lysozyme induced in macrophages results in the transcriptional modulation of very few genes (data not shown). It is, however, remarkable that these differentially expressed genes also included the very same genes that were also differentially expressed, at all time points, in monocytes, i.e., *DLG2* and *CKMT2*.

The functional hypotheses and the molecular networks highlighted in the present study are in good agreement with the results of a recent paper [38], which reports a study carried out with mouse macrophages stimulated with LPS to induce an inflammatory state. In that experimental model, lysozyme acted as a potent inhibitor of IL-6 and TNF-α in a dose-dependent manner, both at the protein and at the mRNA levels. At the level of the molecular pathway, lysozyme seems to act by reducing the phosphorylation of JNK (c-Jun N-terminal kinase), a member of the MAPK (mitogen activated protein kinase) family, which is also involved in the cell inflammatory response [49]. Indeed, phosphorylation of JNK induces the expression of many inflammatory cytokines, including TNF-α itself [50]. Moreover, the study of Tagashira and co-workers, similar to ours, did not reveal any toxic effect of lysozyme for the treated cells, further confirming the reliability of our observations and predictions.

In our attempts to understand the effects of lysozyme on U937 cells, by integrating transcriptomic and proteomic data, we were unable to detect any significantly differentially expressed protein in lysozyme-treated compared to untreated cells. Overall, literature on the integrative transcriptomic-proteomic analyses revealed only a poor correlation between the quantities of mRNAs and the corresponding proteins [51,52], which is indicative of a complex regulatory mechanism, controlling the expression mechanism at both the RNA and protein levels, and involves multiple layers of gene regulation like genomic variations and gene expression. Another order of dynamicity is added by post-transcriptional and post-translational regulations of gene expression. These include, but are not limited to, alternative splicing [53] and editing of expressed transcripts and post-translational addition of covalent modifications to proteins. Thus, several isoforms or proteoforms, with distinct structural and functional attributes, may originate from a given gene. The biological uncoupling between the levels of mRNA and proteins may be the consequence of half-life difference between proteins and mRNAs, these latter being five-times less stable than proteins in mammalian cells [54]. Furthermore, ubiquitination, phosphorylation and cellular localization are some of the post-translational regulations that can affect the protein half-lives and thus, their detection [51]. Studies have reported that mRNA abundance can predict protein abundance only partially, i.e., for approximately 40% of genes [52,55]. Also, proteomics lacks the sensitivity to detect poorly abundant proteins and it is limited in its ability to identify novel proteoforms resulting from alternative splicing or SNPs (single nucleotide polymorphisms). The fractionation approach used in this proteomic study, although not having the ability of other techniques, such as targeted proteomics [56], to detect the expression level of low abundance proteins, allowed us to provide the relative quantification of more than 6100 protein groups and to obtain a general overview on the translational molecular layer. Our study shows differentially expressed protein levels in samples collected at 24 h + 24 vs. 1 h + 24 in both controls and lysozyme-treated cells. This seems to be related to the growth time features of the cells being used, rather than to the pharmacological treatment. The differentially expressed protein levels follow the same trend of their relevant mRNAs (Figure 6a,b), albeit with minor shifts in expression, confirming the greater sensitivity of the RNA-seq analysis over the analysis of proteomic data.

## 4. Materials and Methods

### 4.1. Materials

All materials were purchased from Sigma-Aldrich (St. Louis, MO, USA), unless otherwise indicated. HEWL was purchased from Fluka (Honeywell Fluka™, Charlotte, NC, USA).

### 4.2. Cell Line and Treatment

The U937 cell line is a human immortalized monocyte cell line, originally obtained from pleural diffusions of a patient affected by histiocytic lymphoma [34]. It was kindly supplied by Dr. S. Pacor (Dept. of Life Sciences, University of Trieste) and maintained in RPMI-1640 supplemented with 10% foetal bovine serum (FBS, Gibco, Invitrogen™, Grand Island, NY, USA), 2 mM l-glutamine (EuroClone™, Devon, UK), 100 IU/mL penicillin and 100 μg/mL streptomycin (EuroClone™). Cells were kept in an incubator with 5% CO_2_ and 100% relative humidity at 37 °C. Cells were grown in suspension in 25 cm^2^ flasks; cell viability was checked by the trypan blue dye exclusion test. 

For experimental purposes 3 × 10^6^ cells were sown in T25 flasks in 9 mL of their complete culture medium and let grow 72 h before pharmacological treatment. The exposure to lysozyme 15 μg/mL was carried out by adding to each flask 1 mL of complete culture medium containing 150 μg/mL of lysozyme (treated cells) or 1 mL of complete culture medium (controls). The treatment was conducted according to two experimental schemes: (i) In the first one, the cells were exposed to lysozyme for 1 h at 37 °C, 5% CO_2_ and 100% relative humidity. The samples to be processed by RNA-sequencing were prepared immediately at the end of the treatment (hereafter referred to as 1h), and at 2 h after the end of treatment (hereafter referred to as 1 h + 2); in the latter case cells were left in fresh complete culture medium for an additional 2 h time in an incubator at 37 °C, 5% CO_2_, and 100% relative humidity after the removal of the lysozyme treatment solution by centrifugation for 7 min (300× *g*) at 4 °C. A supplementary time point analysis at 24 h after the end of treatment was employed for proteomic analysis only. In this case, cells were left in fresh complete culture medium for further 24 h in an incubator before collection (hereafter referred to as 1 h + 24) after the removal of the lysozyme treatment solution by centrifugation for 7 min (300× *g*) at 4 °C; (ii) in the second experimental scheme, the cells were exposed to lysozyme for 24 h nonstop and processed for RNA-sequencing immediately at the end of the treatment (hereafter referred to as 24 h); the proteomic analysis was conducted after further 24 h in fresh complete medium after the removal of the lysozyme treatment solution by centrifugation at the conditions indicated above (hereafter referred to as 24 h + 24). Each experimental condition corresponds to a biological triplicate.

### 4.3. Whole Transcriptome Expression Profiling via RNA-seq

At the end of each experimental treatment the cells were collected and centrifuged for 7 min (300× *g*) at 4 °C, the supernatant was discarded and the cell pellet transferred in an Eppendorf tube, washed with 1 mL of cold PBS (phosphate buffered saline) and centrifuged again for 7 min (800× *g*) at 4 °C. The supernatant was then discarded and the cell pellet re-suspended in an appropriate volume of the lysis buffer of the extraction kit. Total RNA was isolated using the Agilent Absolutely RNA Miniprep Kit (Agilent Technologies, Santa Clara, CA, USA) according to the manufacturer’s protocol. The RNA concentration and purity were calculated using spectrophotometer measurements (evaluating the ratio of absorbance at 260 and 280 nm as a measure of protein contamination, and the ratio of absorbance at 260 and 230 nm as a measure of carbohydrate contamination). RNA integrity was examined by capillary electrophoresis (BioAnalyzer 2100, Agilent Technologies) to ensure the achievements of a RNA integrity number (28S to 18S ribosomal RNA) >9, required for the preparation of the libraries for RNA-sequencing.

### 4.4. Library Preparation and RNA-Sequencing

Barcoded sequencing libraries for all samples selected for RNA-sequencing were prepared using QuantSeq 3′ mRNA-Seq Library prep kit produced by Lexogen (Wien, Austria), according to the manufacturer’s instructions. Briefly, this strategy generates sequencing libraries enriched in regions close to the 3′ end of polyadenylated RNAs, to enable cost-saving multiplexing with reduced sequencing depth compared to conventional RNA-seq protocols, while maintaining the possibility to accurately calculate gene expression levels with no need of a normalization step based on transcript length.

Following quality evaluation with an Agilent Bioanalyzer instrument, the libraries obtained were opportunely pooled in equimolar concentrations and sent to the sequencing centre at the Genomics Core of the Oklahoma Medical Research Foundation, where they were sequenced on a single lane of an Illumina NextSeq 500 platform, run in High Output mode with a single-end 75 base pair strategy. Raw sequence data have been deposited in the NCBI SRA database under the accession ID SRP124220, linked to BioProject PRJNA417221.

### 4.5. Sequencing Data Analysis

Raw demultiplexed sequencing data were provided from the sequencing centre and imported in the CLC Genomics Workbench 10 (Qiagen, Hilden, Germany) environment, where the trimming procedure was carried out using the following parameters: first, reads were trimmed by quality, setting the quality score threshold to 0.05 and removing ambiguous nucleotides. In addition, the 12 nucleotides at the 3′ terminus of the reads were discarded to remove a compositional nucleotide bias observed in a preliminary screening, and residual sequencing adapters (ACACTCTTTCCCTACACGACGCTCTTCCGATCT, GATCGGAAGAGCACACGTCTGAACTCCAGTCAC and GTGACTGGAGTTCAGACGTGTGCTCTTCCGATCT) were detected and removed, setting the ‘mismatch_cost’, ‘gap_cost’, ‘minimum_internal_score’ and ‘minimum_score_at_ends’ to 3, 3, 10 and 4. In addition, poly(A) stretches were removed because of the expected lack of match to the genome sequence. Similarly, poly(G) sequence stretches were discarded as these are recognized as possible artefacts linked to the lack of signal in the Illumina NextSeq sequencing chemistry.

### 4.6. Differential Gene Expression Analysis

Trimmed reads from each library were mapped on the annotated coding regions of the reference human genome (version Hg38) from the UCSC Genome Browser with the ‘RNA-seq analysis’ tool included in the CLC Genomics Workbench. The parameters set to allow a non-ambiguous read mapping were set as follows: mismatch cost = 2, insertion cost = 3, deletion cost = 3, length fraction = 0.98, similarity fraction = 0.98. Tables containing raw read counts for each gene and each sequencing library generated by the mapping process were grouped based on replicates and analysed with the CLC Genomics Workbench via the ‘differential expression for RNA-seq’ tool, comparing each experimental condition with the paired control at the same experimental time point. The generalized linear model (GLM) used for this statistical analysis assumes that read counts follow a negative binomial distribution. To identify significant differentially expressed genes (DEGs), only results with *p*-value ≤ 0.05 and fold change ≥ |2| were considered as meaningful. 

### 4.7. Ingenuity Pathway Analysis (IPA^®^)

Functional and pathway predictions were performed using Ingenuity Pathway Analysis (IPA^®^) (Qiagen, Hilden, Germany), software based on a database (the Ingenuity knowledgebase) which includes all the updated available information concerning gene regulatory networks in human. Significant DEGs identified in the previous step of analysis with an emphasis on 1 h, the time point with the most significant alterations, were used as input to identify the canonical pathways most significantly affected by the lysozyme treatment and infer the upstream regulators and downstream effects most likely to be involved in the process.

To identify the pathway specifically responsive to lysozyme, we took into account the most significant DEGs to build a custom pathway based on the shortest relation sub-pathways between the identified DEGs and other key genes predicted as likely to be significantly altered in a core IPA analysis.

### 4.8. Proteomic Analysis

Cells intended for proteomic analysis were collected 24 h after the end of each treatment, centrifuged for 7 min (300× *g*) at 4 °C, washed with phosphate buffered saline (PBS) and centrifuged again. The PBS was removed until approximately 50 μL of liquid remained. Cell pellets were snap frozen in liquid N_2_ and stored at −80 °C until analysis.

Each sample was digested by filter aided sample preparation (FASP) [57] with minor modifications. Dithiothreitol (DTT) was replaced by tris (2-carboxyethyl)phosphine (TCEP) as the reducing agent and iodoacetamide by chloracetamide as the alkylating agent. A combined proteolytic digestion was performed using endoproteinase Lys-C and trypsin. Peptides were desalted on C18 StageTips [58] and dried down by vacuum centrifugation. Samples were then fractionated into six fractions by strong cation exchange (SCX) chromatography on stage tips [59] and dried down again by vacuum centrifugation. For LC MS/MS analysis, peptides were resuspended and then separated by reversed-phase chromatography using a Dionex Ultimate 3000 RSLC nanoUPLC system on a home-made 75 µm ID × 50 cm C18 capillary column (Reprosil-Pur AQ 120 Å, 1.9 µm) in-line connected with an Orbitrap Q Exactive HF Mass-Spectrometer (Thermo Fischer Scientific, Waltham, MA, USA). Database search was performed using MaxQuant 1.6.0.1 [60] against the Human Uniprot database (Last Modified: 2017-05-23, 71567 canonical and isoform sequences) where the hen egg white lysozyme sequence (AFA52017.1, from Uniprot, 2018-04-09) was added. Carbamidomethylation was set as fixed modification, whereas oxidation (M), phosphorylation (S,T,Y), acetylation (protein N-term) and glutamine to pyroglutamate were considered as variable modifications. A maximum of two missed cleavages were allowed for the search and ‘match between runs’ option was selected. A minimum of two peptides were allowed for protein identification and the false discovery rate (FDR) cut-off for both peptides and proteins was set to 0.01. The MaxLFQ algorithm was used by MaxQuant for the label-free quantification (LFQ), with the standard settings [61]. Perseus, the statistical validation tool embedded in MaxQuant, was used for further data validation [62]. Reverse proteins, contaminants and proteins only identified by sites were filtered out. Biological replicates were grouped together and protein groups containing a minimum of two LFQ values in at least one group were conserved. Missing values were imputed with random numbers from a normal distribution (width = 0.4, down-shift = 1.8). Two-samples *t*-test coupled with permutation-based correction (false discovery rate) was performed to identify the differentially expressed proteins. Significant hits were determined by a volcano plot-based strategy, combining *t*-test p-values with ratio information [63]. Significance curves in the volcano plot corresponded to a S0 value of 0.5 and a FDR cut-off of 0.05. Further graphical displays were generated using homemade programs written in R [64]. 

## 5. Conclusions

Herein, we bring new insights into the origin of the non-catalytic anti-inflammatory properties of lysozyme. Because of the broad perspective of the RNA-sequencing analysis approach, this study shows that the anti-inflammatory activity of lysozyme occurs owing to mechanisms of gene regulation involving the TNF-α/IL-1β pathway modulation. Our findings, besides confirming the alteration of proteins of the inflammatory pathway such as TNF-α and IL-1β, previously described as affected by lysozyme treatment in scientific literature, enable their inclusion in a more expanded and comprehensive picture, under the form of a functional network, where they are related to many interactors. Furthermore, this approach demonstrates that such important, albeit transient, effects of lysozyme can be achieved with the supplementation of relatively low concentrations of HEWL for a short time of exposure. To the best of our knowledge, this is the first report to offer such a broad overview of the anti-inflammatory effects of lysozyme at the gene expression level. The novel data we report contributes to understanding the role of lysozyme at a molecular level in the context of inflammatory processes, potentially allowing its potential in pharmacological applications to be exploited.

## Figures and Tables

**Table 1:** Comparison of the number of genes differentially expressed in U937 cells following lysozyme treatment.

Duration of Exposure to Lysozyme (h)	Analysis after the End of Exposure (h)	Up-Regulated Genes	Down-Regulated Genes
1	Immediate	3/25	22/25
	2	2/25	1/25
24	Immediate	1/25	5/25

Tests of differential expression were performed on the complete human genome (version Hg38), assessing the gene expression levels of 53,816 annotated features. DEGs were considered significant when *p*-value ≤ 0.05 and modulus (fold change) ≥2. The table shows the number of up- or down-regulated genes against the total number of DEGs in each condition.

**Table 2:** Differentially expressed genes in U937 cells. Whenever significant, fold change values are shown. Only genes detected as up-or down-regulated in respect of parallel control samples are shown.

Official Symbol	Official Full Name ^	Compartment ^	Main Functions ^	Fold Change at Different Analysis Time Points		
				1 h	1 h + 2	24 h
**Up-regulated**						
*CCDC73*	Coiled-coil domain containing 73	Intracellular (predicted)	Unknown	-	-	7.00
*CKMT2*	Creatine kinase, mitochondrial 2	Mitochondrial inner membrane	Metabolism	20.80	23.25	14.20
*SCP2*	Sterol carrier protein 2	Peroxisomal matrix	Metabolism	-	-	2.25
*TMEM150C*	Transmembrane protein 150C	Plasma membrane	Mechanotransduction	-	9.20	24.14
*TTI2*	TELO2 interacting protein 2	Cytosol	DNA damage response	3.86	-	-
*WDR47*	WD repeat domain 47	Intracellular	Development biology	-	-	2.56
**Down-regulated**						
*ADM*	Adrenomedullin	Extracellular region	Signal transduction	−24.14	-	-
*CCL1*	C-C motif chemokine ligand 1	Extracellular region	Signal transduction	−13.91	-	-
*CCL3*	C-C motif chemokine ligand 3	Extracellular region	Immune system	−17.61	-	-
*CCL3L3*	C-C motif chemokine ligand 3 like 3	Extracellular region	Immune system	−12.98	-	-
*CCL7*	C-C motif chemokine ligand 7	Extracellular region	Immune system	−12.95	-	-
*DLG2*	Discs large MAGUK scaffold protein 2	Cytosol	Neuronal system	−110.73	−13.15	−8.48
*FTH1*	Ferritin heavy chain 1	Extracellular region; Cytosol	Immune system; Vesicle-mediated transport; Transport of small molecules	−2.28	-	-
*GDF15*	Growth differentiation factor 15	Extracellular region		−11.97	-	-
*IER3*	Immediate early response 3	Cytosol	Signal transduction	−2.82	-	-
*IFI6*	Interferon alpha inducible protein 6	Plasma membrane	Immune system	−5.40	-	-
*IL1B*	Interleukin 1 beta	Extracellular region; Cytosol	Immune system	−7.99	-	-
*IL1RN*	Interleukin 1 receptor antagonist	Plasma membrane	Immune system	−6.06	-	-
*INHBA*	Inhibin beta A subunit	Extracellular region	Metabolism of proteins; Signal transduction	−12.72	-	-
*ISG15*	ISG15 ubiquitin-like modifier	Cytosol; Nucleoplasm	Disease; Immune system; DNA repair	−3.73	-	-
*MMP1*	Matrix metallopeptidase 1	Extracellular region	Extracellular matrix organisation; Haemostasis; Metabolism of proteins; Immune system	−4.63	-	-
*MMP9*	Matrix metallopeptidase 9	Extracellular region	Development biology; Extracellular matrix organisation; Immune system; Signal transduction	−7.52	-	-
*MTRNR2L2*	MT-RNR2 like 2	Extracellular region	Programmed cell death	−2.14	-	-
*PLAUR*	Plasminogen activator, urokinase receptor	Plasma membrane; Endoplasmic reticulum membrane; Specific granule membrane; Endoplasmic reticulum lumen	Immune system; Metabolism of proteins	−2.55	-	-
*RGS16*	Regulator of G protein signalling 16	Plasma membrane	Signal transduction	−2.69	-	-
*SPP1*	Secreted phosphoprotein 1	Extracellular region; Endoplasmic reticulum lumen	Extracellular matrix organisation; Gene expression; Signal transduction	−16.71	-	-
*TIMP1*	TIMP metallopeptidase inhibitor 1	Extracellular region; Platelet alpha granule lumen; Endoplasmic reticulum lumen	Extracellular matrix organisation; Haemostasis; Immune system; Metabolism of proteins	−2.93	-	-
*TJP1*	Tight junction protein 1	Cell junctions; Plasma membrane; Cytosol; Golgi-associated vesicle membrane	Gene expression (transcription); Programmed cell death; Vesicle-mediated transport; Signal transduction	2.14	-	-
*VIM*	Vimentin	Cytosol	Immune system; Muscle contraction; Programmed cell death	−3.88	-	-

^ www.genenames.org; www.genecards.org; www.reactome.org; www.uniprot.org; www.proteinatlas.org.

**Table 3:** LC-MS/MS identification results of differentially expressed protein spots in U937 control cells upon the two experimental schemes (Ctrl 24 h + 24 versus Ctrl 1 h + 24).

Protein Name	Gene Name	Accession No. ^	Intensity Mean Difference	−log_10_(*p*-Value)	Subcellular Location ^	#
Cytochrome P450	*CYP1A1*	E7EMT5	−3.814	1.977	ER	S
Dipeptidase 1	*DPEP1*	P16444	−3.595	3.086	PM	S
Integrin alpha-M	*ITGAM*	P11215	−3.111	4.298	PM, OL	S
Carbohydrate sulfotransferase 14	*CHST14*	Q8NCH0	−2.780	2.386	GA	-
Protein S100-A8	*S100A8*	P05109	−2.607	3.267	PM, ExR/S, Cy, OL	S
Protein S100-A9	*S100A9*	P06702	−2.481	4.341	PM, ExR/S, Cy	S
Gamma-interferon-inducible lysosomal thiol reductase	*IFI30*	P13284	−1.971	3.228	ExR/S, OL	-
SAM domain-containing protein SAMSN-1	*SAMSN1*	Q9NSI8	−1.778	4.143	N	-
Glycerol-3-phosphate dehydrogenase, mitochondrial	*GPD2*	P43304	1.835	3.529	M	S
Calcium/calmodulin-dependent protein kinase type 1	*CAMK1*	Q14012	2.088	3.662	N, OL	S
Cathepsin G	*CTSG*	P08311	2.103	3.661	OL (cell surface)	S
Aflatoxin B1 aldehyde reductase member 3	*AKR7A3*	O95154	2.203	3.480	OL (cytoplasm)	S
Long-chain-fatty-acid—CoA ligase 5	*ACSL5*	Q9ULC5	2.327	5.667	ER, M	S
HLA class II histocompatibility antigen, DRB1-15 beta chain	*HLA-DRB1*	P01911	3.731	2.377	PM, E, L, GA, ER	S
HLA class II histocompatibility antigen, DRB1-14 beta chain	*HLA-DRB1*	Q9GIY3	3.851	3.219	PM, E, L, GA, ER	-
HLA class II histocompatibility antigen, DRB1-9 beta chain	*HLA-DRB1*	Q9TQE0	4.216	3.003	PM, E, L, GA, ER	S
HLA class II histocompatibility antigen, gamma chain	*CD74*	P04233	4.338	3.349	PM, GA, E, L, ER	-
Nicotinate-nucleotide pyrophosphorylase [carboxylating]	*QPRT*	Q15274	4.405	3.701	C, ExR/S, OL	S
Neutrophil elastase	*ELANE*	P08246	5.024	3.358	ExR/S, L, OL	S
HLA class II histocompatibility antigen, DR beta 5 chain	*HLA-DRB5*	Q30154	5.123	3.312	PM, E, L, GA, ER	S
HLA class II histocompatibility antigen, DRB1-16 beta chain	*HLA-DRB1*	Q29974	6.902	3.752	PM, E, L, GA, ER	S
Kinesin-like protein KIF3B	*KIF3B*	O15066	7.129	3.922	Cy, OL	-
HLA class II histocompatibility antigen, DR alpha chain	*HLA-DRA*	A0A0G2JMH6	7.236	5.473	PM, OL	S

^ Source: www.uniprot.org; C = cytosol, Cy = cytoskeleton, E = endosome, ER = endoplasmic reticulum, ExR/S = extracellular region or secreted, GA = Golgi apparatus, L = lysosome, M = mitochondrion, N = nucleus, OL = other locations, PM = plasma membrane. #: S (shared) indicated proteins differentially expressed both in controls and in lysozyme-treated U937 cells (see for comparison Table 4).

**Table 4:** LC-MS/MS identification results of differentially expressed protein spots in U937 lysozyme-treated cells upon the two experimental schemes (LYS 24 h+24 versus LYS 1 h+24).

Protein Name	Gene Name	Accession No. ^	Intensity Mean Difference	−log10(*p*-Value)	Subcellular Location ^	#
Dipeptidase 1	*DPEP1*	P16444	−3.010	4.354	PM	S
Dehydrogenase/reductase SDR family member 9	*DHRS9*	Q9BPW9	−2.967	3.462	ER	-
Integrin alpha-M	*ITGAM*	P11215	−2.872	3.416	PM, OL	S
Protein S100-A8	*S100A8*	P05109	−2.784	3.495	PM, ExR/S, Cy, OL	S
Glycerol-3-phosphate acyltransferase 3	*GPAT3*	Q53EU6	−2.564	3.446	ER	-
Protein S100-A9	*S100A9*	P06702	−2.506	3.373	PM, ExR/S, Cy, OL	S
Immunoglobulin alpha Fc receptor	*FCAR*	P24071	−2.314	3.301	PM, ExR/S	-
Cytochrome P450	*CYP1A1*	E7EMT5	−2.307	3.075	ER	S
Creatine kinase U-type, mitochondrial	*CKMT1A*	P12532	−2.129	3.430	M	-
Protein DPCD	*DPCD*	Q9BVM2	1.884	4.083	N	-
Aflatoxin B1 aldehyde reductase member 3	*AKR7A3*	O95154	1.906	3.661	OL (cytoplasm)	S
Cathepsin G	*CTSG*	P08311	2.084	4.115	OL (cell surface)	S
Sulfotransferase 1A4	*SULT1A4*	P0DMN0	2.118	4.406	OL (cytoplasm)	-
Glycerol-3-phosphate dehydrogenase, mitochondrial	*GPD2*	P43304	2.154	3.692	M	S
Long-chain-fatty-acid- -CoA ligase 5	*ACSL5*	Q9ULC5	2.193	3.405	ER, M	S
Calcium/calmodulin-dependent protein kinase type 1	*CAMK1*	Q14012	2.292	4.360	N, OL	S
Azurocidin	*AZU1*	P20160	2.332	3.876	OL (cytoplasmic granule membrane)	-
Interferon regulatory factor 8	*IRF8*	Q02556	2.980	2.604	N, OL	-
Nicotinate-nucleotide pyrophosphorylase [carboxylating]	*QPRT*	Q15274	3.881	2.424	C, ExR/S, OL	S
HLA class II histocompatibility antigen, DRB1-15 beta chain	*HLA-DRB1*	P01911	3.917	2.424	PM, E, L, GA, ER	S
HLA class II histocompatibility antigen, DRB1-9 beta chain	*HLA-DRB1*	Q9TQE0	4.142	3.599	PM, E, L, GA, ER	S
HLA class II histocompatibility antigen, DR beta 5 chain	*HLA-DRB5*	Q30154	4.556	2.820	PM, E, L, GA, ER	S
Neutrophil elastase	*ELANE*	P08246	6.238	4.969	ExR/S, L, OL	S
HLA class II histocompatibility antigen, DRB1-16 beta chain	*HLA-DRB1*	Q29974	6.658	4.247	PM, E, L, GA, ER	S
HLA class II histocompatibility antigen, DR alpha chain	*HLA-DRA*	A0A0G2JMH6	7.888	5.519	PM, OL	S

^ Source: www.uniprot.org; C = cytosol, Cy = cytoskeleton, E = endosome, ER = endoplasmic reticulum, ExR/S = extracellular region or secreted, GA = Golgi apparatus, L = lysosome, M = mitochondrion, N = nucleus, OL = other locations, PM = plasma membrane. #: S (shared) indicated proteins differentially expressed both in controls and in lysozyme-treated U937 cells (see for comparison Table 3).

## Supplementary Materials

Supplementary materials can be found at https://www.mdpi.com/1422-0067/20/21/5502/s1.

## Abbreviations

AGEs	Advanced Glycation End Products
CCL1	Chemokine Ligand 1
CCL3	Chemokine Ligand 3
CCL3L3	Chemokine Ligand 3-like 3
CCL7	Chemokine Ligand 7
CKMT2	Creatine Kinase Mitochondrial 2
DEGs	Differentially Expressed Genes
DLG2	Discs Large MAGUK Scaffold Protein 2
DSS	Dextran Sodium Sulphate
DTT	Dithiothreitol
FASP	Filter Aided Sample Preparation
FBS	Foetal Bovine Serum
FDR	False Discovery Rate
GI	Gastrointestinal
GLM	Generalized Linear Model
HEWL	Hen Egg-White Lysozyme
IER3	Immediate Early Response 3
IFNG	Interferon Gamma
IL-1	Interleukin 1
IL-6	Interleukin 6
IPA	Ingenuity Pathway Analysis
ISG15	Interferon-Stimulated Gene 15
JNK	c-Jun N-terminal Kinase
LC-MS/MS	Liquid Chromatography-tandem Mass Spectrometry
LFQ	Label-Free Quantification
LPS	Lipopolysaccharide
MAPK	Mitogen Activated Protein Kinase
MMP-9	Matrix Metallopeptidase 9
NF-κB	Nuclear Factor Kappa-light-chain-enhancer of activated B cells
PBS	Phosphate Buffered Saline
PMA	Phorbol-12-myristate 13-acetate
RAGE	Receptor of AGE
SCX	Strong Cation Exchange Chromatography
SNPs	Single Nucleotide Polymorphisms
UC	Ulcerative Colitis
TCEP	Tris (2-carboxyethyl)phosphine
TMEM150C	Transmembrane Protein 150C
TNF-α	Tumour Necrosis factor alpha
