# Supplementary material for: Lysozyme-Induced Transcriptional Regulation of TNF-α Pathway Genes in Cells of the Monocyte Lineage
Source: Int J Mol Sci. 2019 Nov 5;20(21):5502. doi: 10.3390/ijms20215502 (PMC6862675; doi:10.3390/ijms20215502)
Supplement: Supplementary file 1 [file ijms-20-05502-s001.zip › Supplementary material.pdf]

Supplementary Material

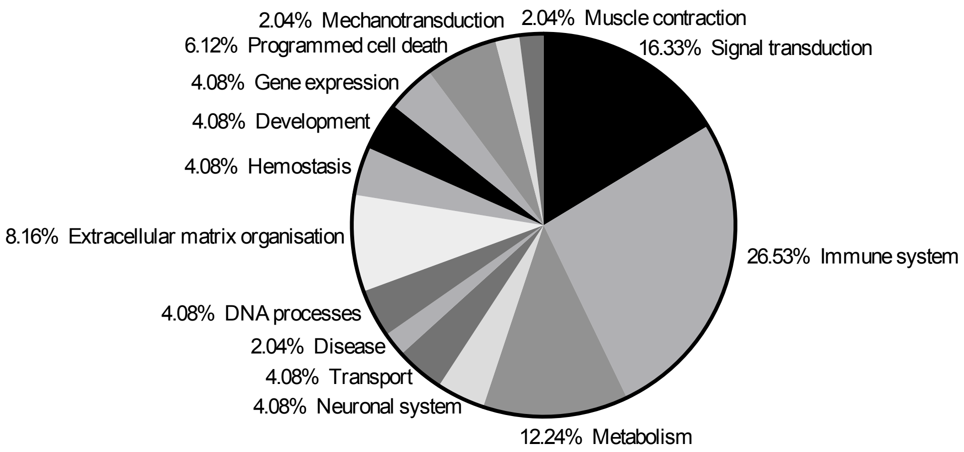

Figure S1. Representation of the main functions of the genes differentially expressed in the monocyte U937 cell line treated with 15 μg/ml lysozyme for 1 h and analysed immediately at the end of the treatment (1h). Every single gene can possess more than one function.

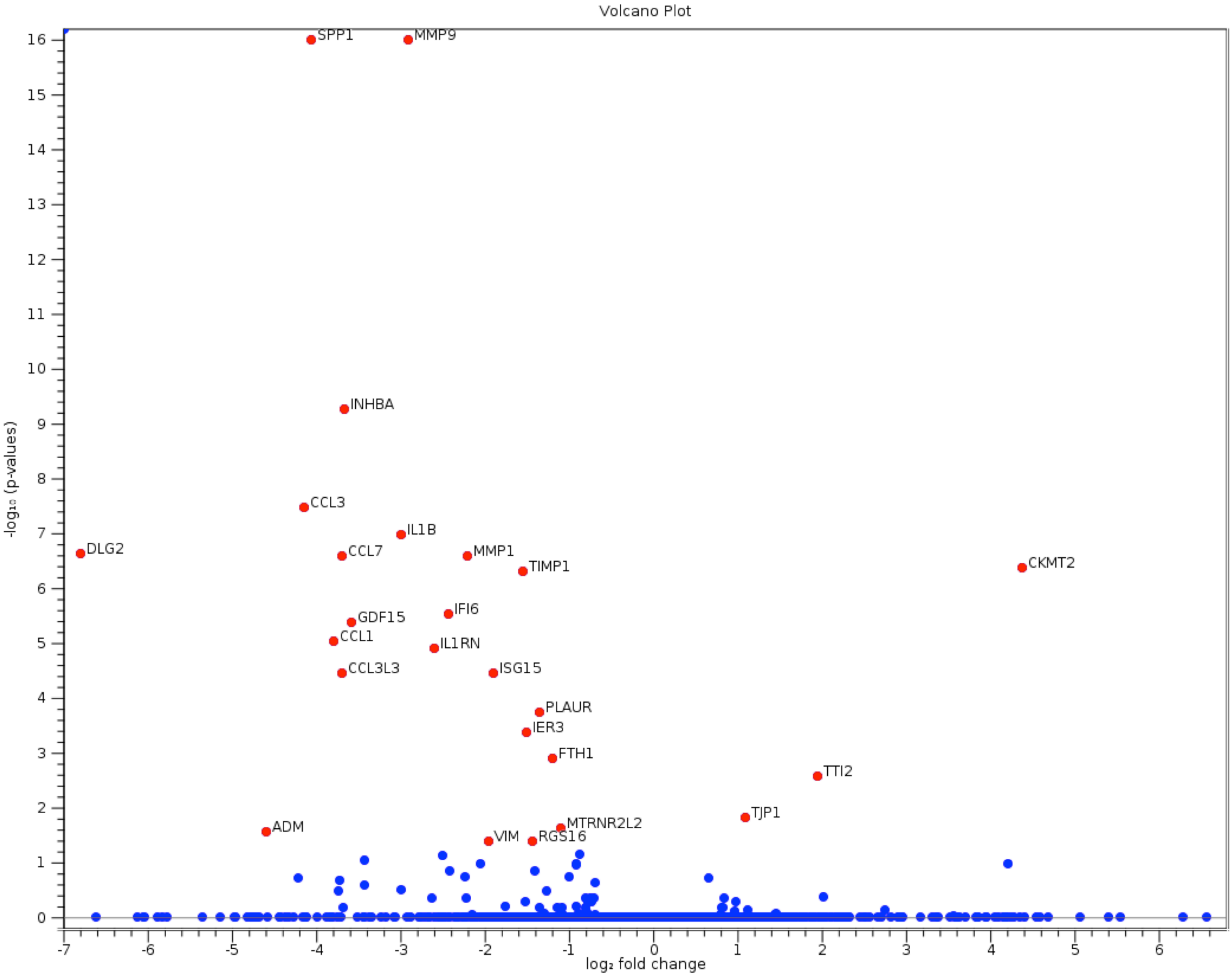

Figure S2: Volcano plot of differential expression between LZ and C at 1h. DEGs are plotted as red dots and labeled with names

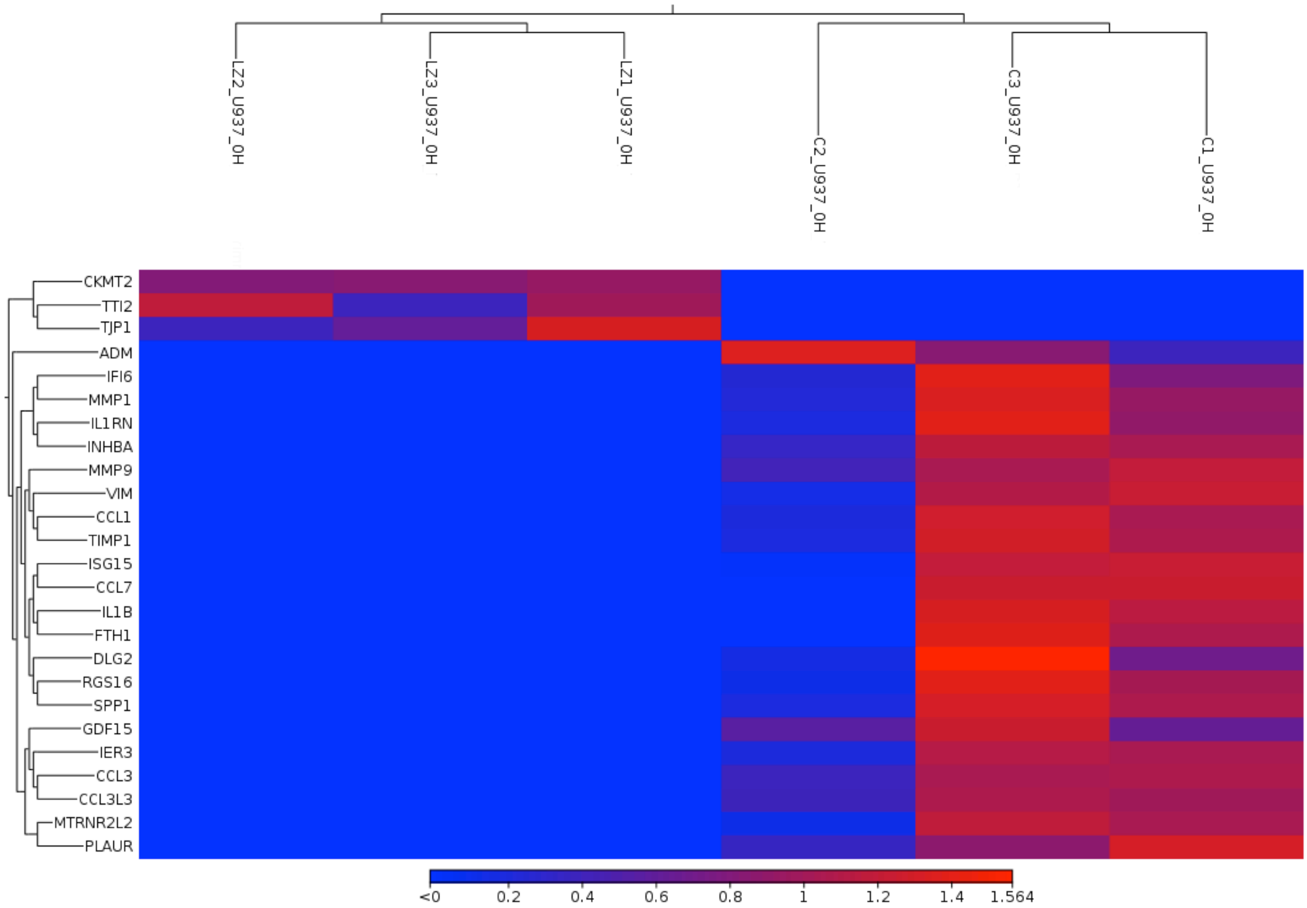

Figure S3: Heatmap of DEGs between LZ and C at 1h. Clusters from both rows and columns are merged by the maximum euclidean distance of their members.

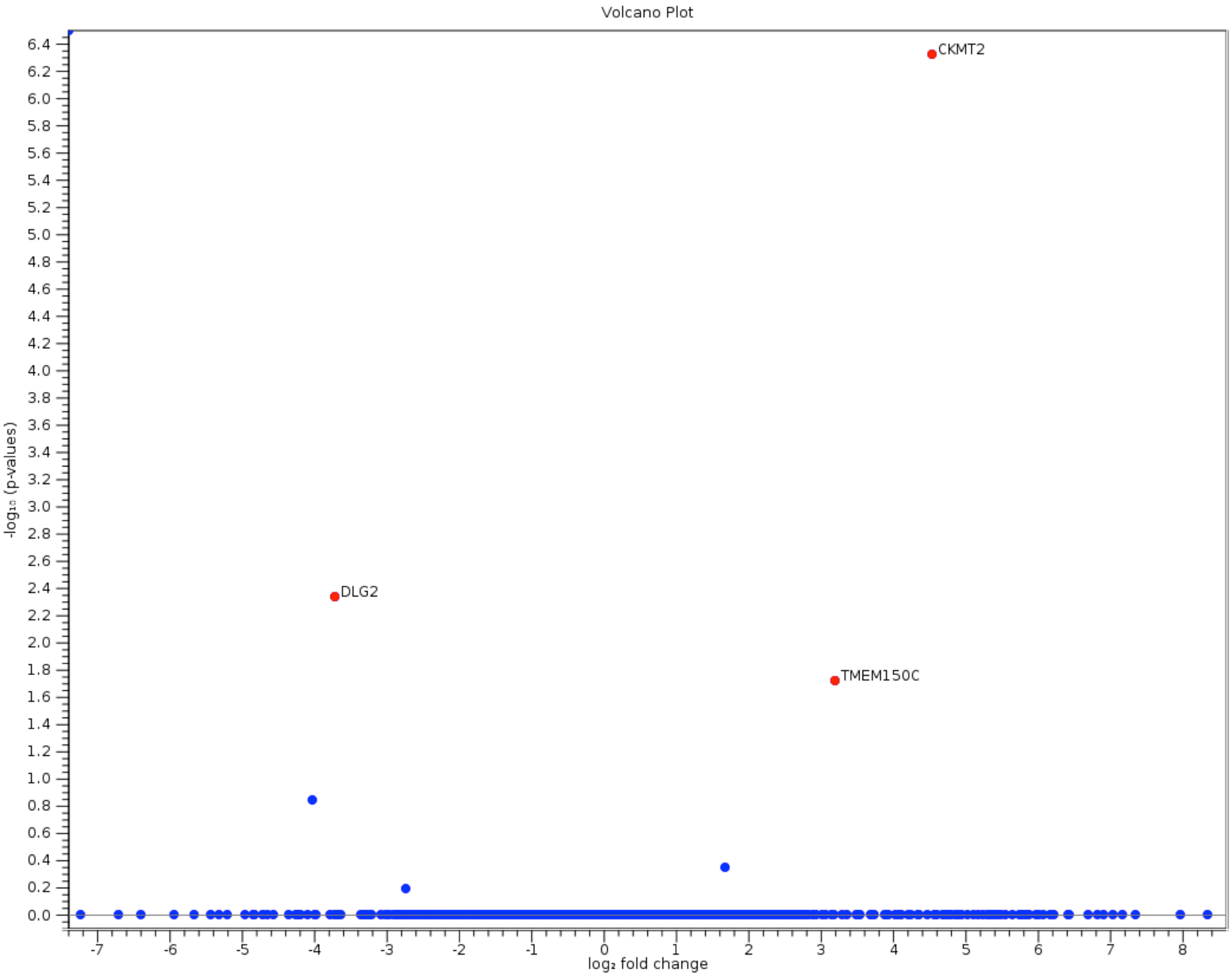

Figure S4: Volcano plot of differential expression between LZ and C at 1h+2. DEGs are plotted as red dots and labeled with names

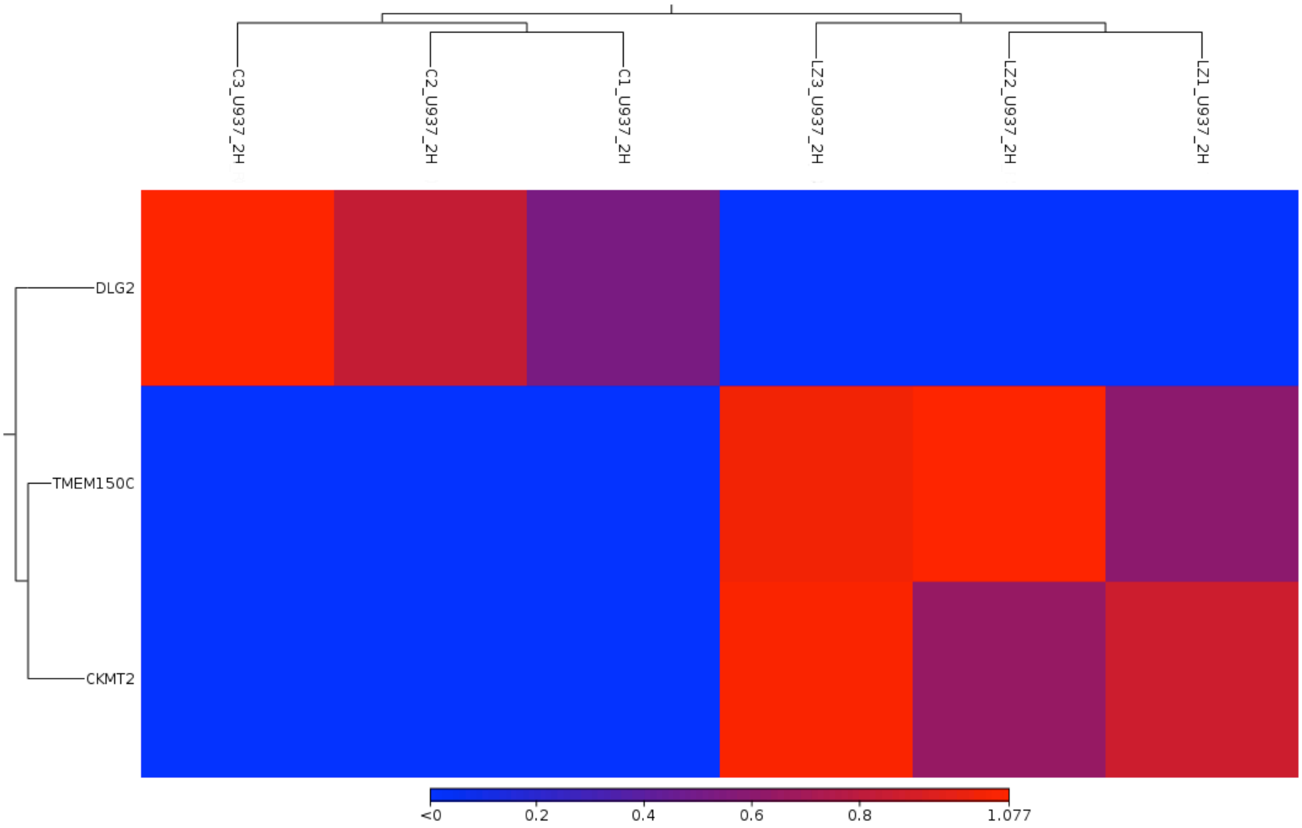

Figure S5: Heatmap of DEGs between LZ and C at 1h+2. Clusters from both rows and columns are merged by the maximum euclidean distance of their members.

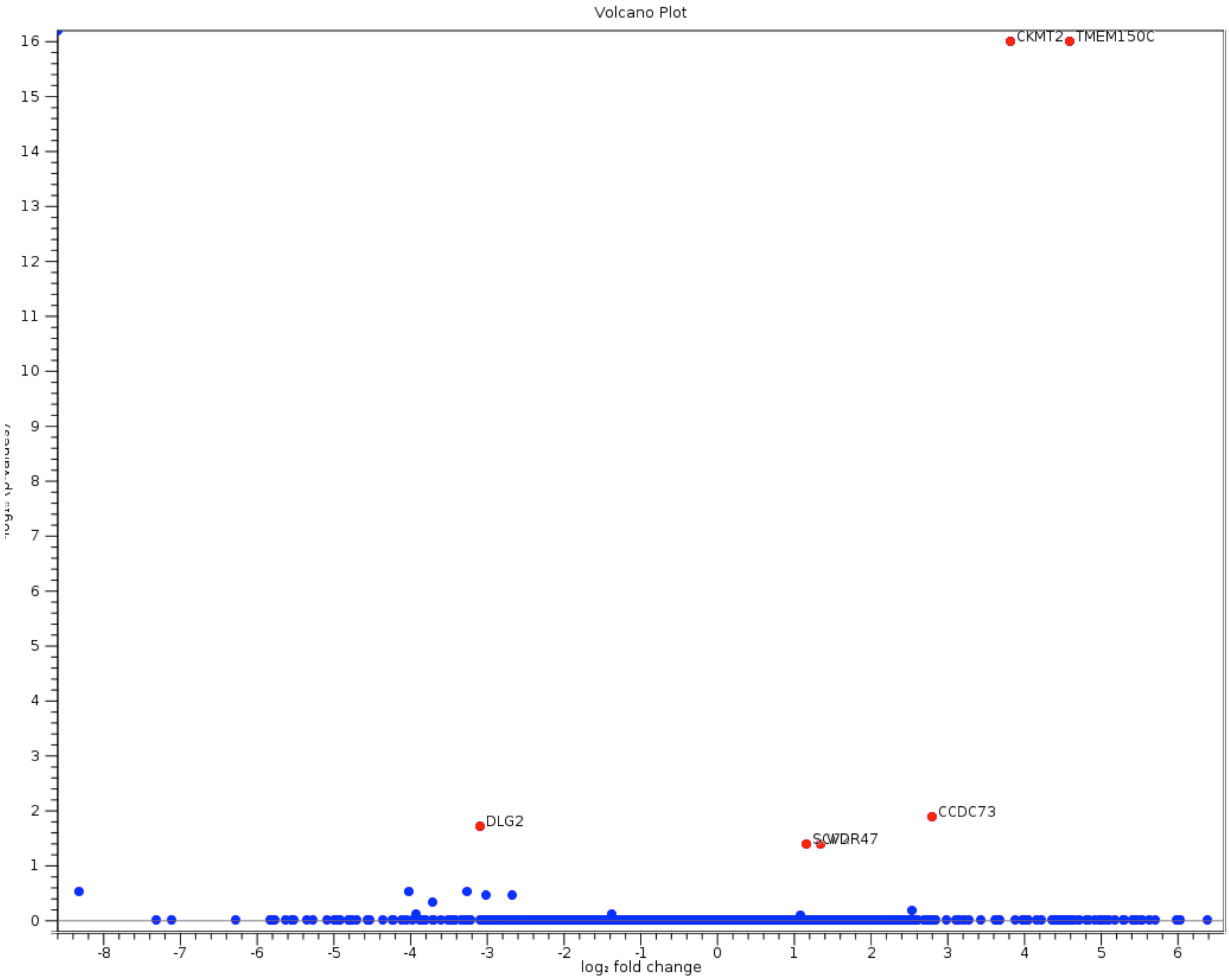

Figure S6: Volcano plot of differential expression between LZ and C at 24h. DEGs are plotted as red dots and labeled with names

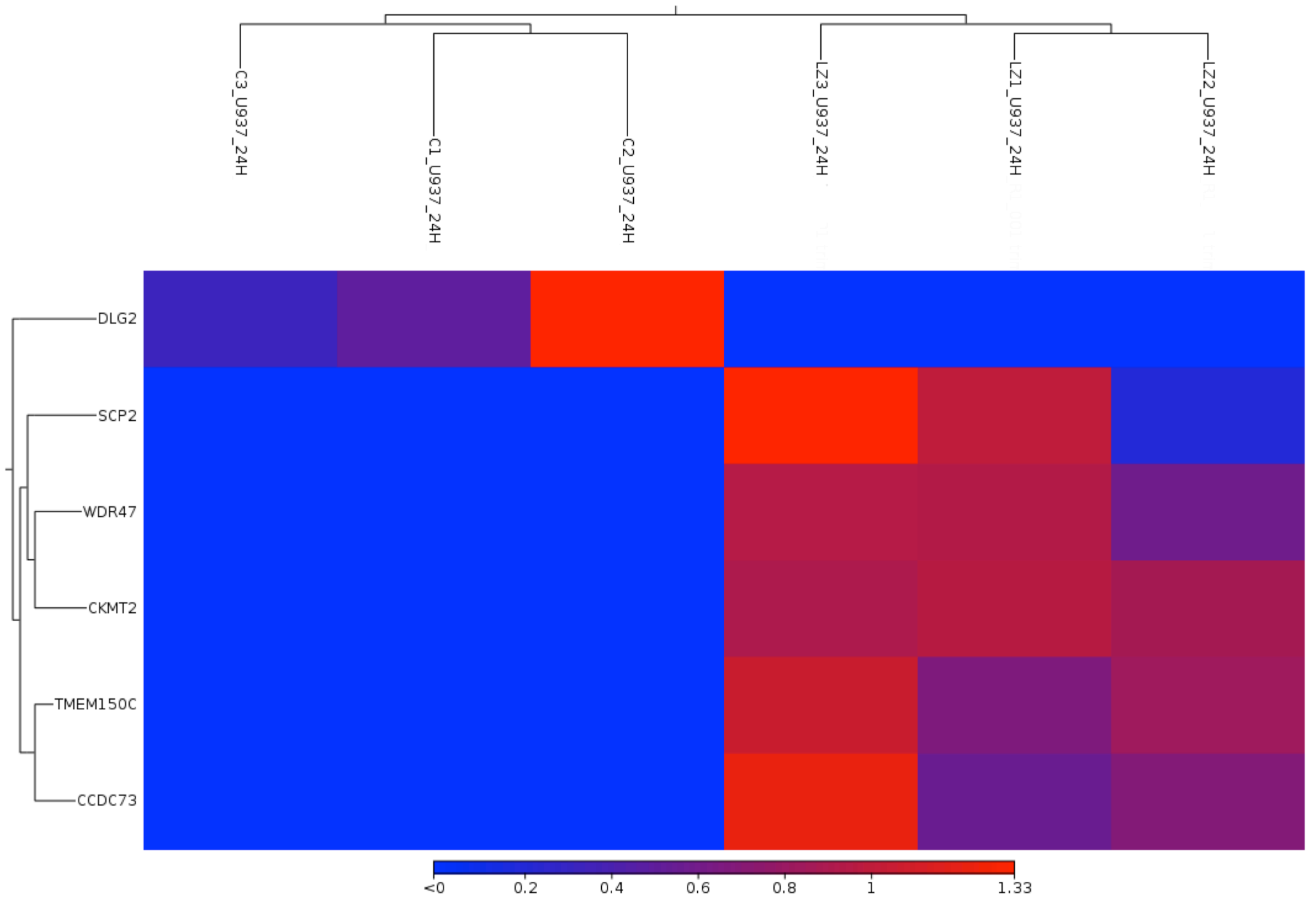

Figure S7: Heatmap of DEGs between LZ and C at 24h. Clusters from both rows and columns are merged by the maximum euclidean distance of their members.

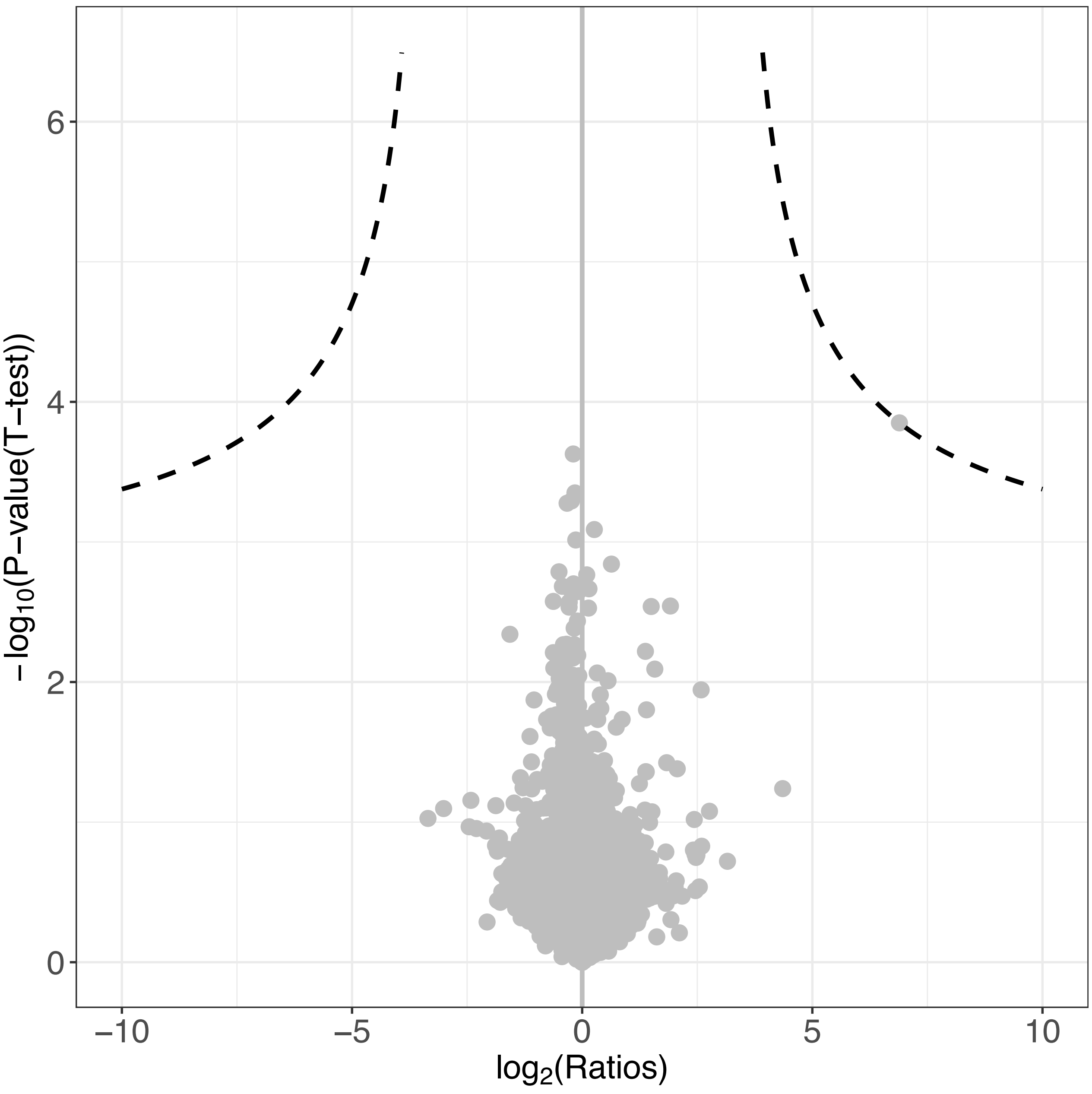

Figure S8. LC-MS/MS analysis, volcano plot shows the differences of protein abundance comparing Lysozyme-treated cells at 1h+24 vs Control cells at 1h+24. The dashed line marks the threshold of statistical significance with FDR < 0.05.

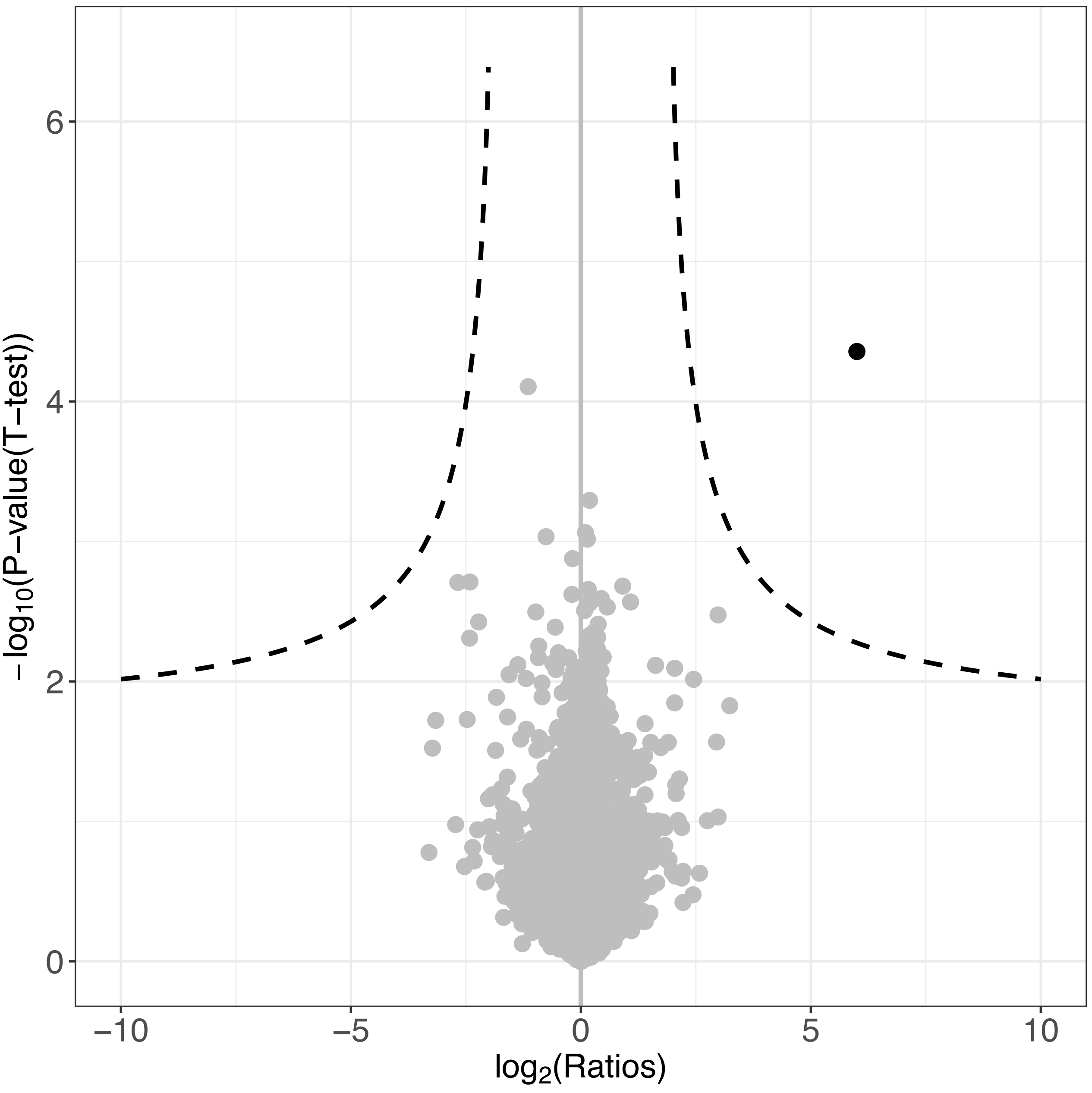

Figure S9. LC-MS/MS analysis, volcano plot shows the differences of protein abundance comparing Lysozyme-treated cells at 24h+24 vs Control cells at 24h+24. The dashed line marks the threshold of statistical significance with FDR < 0.05.
