# Supplementary material for: Lysozyme-Induced Transcriptional Regulation of TNF-α Pathway Genes in Cells of the Monocyte Lineage
Source: Int J Mol Sci. 2019 Nov 5;20(21):5502. doi: 10.3390/ijms20215502 (PMC6862675; doi:10.3390/ijms20215502)
Supplement: Supplementary file 1 [file Supplementary Table 1.pdf › Supplementary Table 1.pdf]

Name	LZ vs. C 0H - Fold change	LZ vs. C 0H - P-value	LZ vs. C 0H - FDR p-value
LOC105378947	3.2353357581	0.554787377	0.9999763851
OR4F16	1.174700458	0.5991193406	0.9999763851
LOC107984841	3.2361532718	0.5546934395	0.9999763851
SAMD11	7.7488609396	0.3054535756	0.9999763851
NOC2L	1.5461618678	0.0791181993	0.9999763851
KLHL17	-1.1145079958	0.8163643571	0.9999763851
PERM1	9.9312452783	0.2485636994	0.9999763851
HES4	1.7587700475	0.436247796	0.9999763851
ISG15	-3.730383726	1.05E-08	3.57E-05
AGRN	1.0390605202	0.8508273187	0.9999763851
RNF223	-2.2178496717	0.4263037525	0.9999763851
C1orf159	-1.0178513312	0.9703924314	0.9999763851
TTLL10	3.2356557963	0.5547505991	0.9999763851
SDF4	-1.0082437213	0.9556899836	0.9999763851
B3GALT6	1.2217146279	0.2396664651	0.9999763851
FAM132A	-4.2849908816	0.4028453076	0.9999763851
UBE2J2	1.2163737243	0.2109203609	0.9999763851
SCNN1D	-1.3487875373	0.588516679	0.9999763851
ACAP3	1.0463571199	0.9151483756	0.9999763851
PUSL1	1.1732893185	0.6030477562	0.9999763851
CPSF3L	-1.1939441443	0.3842183421	0.9999763851
CPTP	1.1414167913	0.7163034955	0.9999763851
TAS1R3	0	0	0
DVL1	1.0143284427	0.94371939	0.9999763851
MXRA8	0	0	0
AURKAIP1	-1.0056458463	0.9706011664	0.9999763851
CCNL2	-1.1774100633	0.2956263159	0.9999763851
MRPL20	1.0319266771	0.8137183017	0.9999763851
ATAD3C	3.2356557963	0.5547505991	0.9999763851
ATAD3B	1.0281248046	0.9174971078	0.9999763851
ATAD3A	1.3772262623	0.0715036014	0.9999763851
TMEM240	1.8132233098	0.5634960726	0.9999763851
SSU72	1.2013887752	0.3122483153	0.9999763851
C1orf233	-2.1176139011	0.1457079401	0.9999763851
MIB2	-1.6523757442	0.2606090588	0.9999763851
MMP23B	3.2353357581	0.554787377	0.9999763851
CDK11B	1.1587195806	0.5282351157	0.9999763851
SLC35E2B	-1.7746476327	0.301503033	0.9999763851
CDK11A	1.0055874539	0.9906962358	0.9999763851
SLC35E2	-1.2702338265	0.1431285802	0.9999763851
NADK	1.2954686403	0.149975455	0.9999763851
GNB1	1.3165781373	0.039505316	0.9999763851
LOC105378949	3.2356557963	0.5547505991	0.9999763851
CALML6	0	0	0
TMEM52	-7.5647903763	0.2176246281	0.9999763851
CFAP74	3.2353357581	0.554787377	0.9999763851
PRKCZ	-1.0852711617	0.9285820278	0.9999763851
LOC100506504	10.4285443305	0.2415083907	0.9999763851

FAAP20	-1.0802270554	0.724048885	0.9999763851
SKI	-1.0114378384	0.9536114695	0.9999763851
MORN1	-1.2600768367	0.752558555	0.9999763851
RER1	1.0968467061	0.5191661191	0.9999763851
PEX10	-1.3154339304	0.8205576571	0.9999763851
PLCH2	0	0	0
PANK4	-1.1295257451	0.8772514809	0.9999763851
TNFRSF14	-1.1848431621	0.5975706626	0.9999763851
FAM213B	1.370603997	0.2344892638	0.9999763851
PRDM16	3.2361532718	0.5546934395	0.9999763851
MEGF6	-4.2849908816	0.4028453076	0.9999763851
TPRG1L	-1.0618961302	0.7344983515	0.9999763851
WRAP73	-1.2617491889	0.1324104744	0.9999763851
TP73	-1.2791513211	0.6734222451	0.9999763851
CCDC27	-1.3154339304	0.8205576571	0.9999763851
LRRC47	-1.3847160182	0.0318397468	0.9999763851
CEP104	-1.0095793631	0.9761424763	0.9999763851
DFFB	-1.4837504829	0.0442488539	0.9999763851
C1orf174	1.2573545516	0.2718368116	0.9999763851
NPHP4	-1.0111546758	0.9714146213	0.9999763851
KCNAB2	-1.2811364388	0.1046163373	0.9999763851
CHD5	3.2353357581	0.554787377	0.9999763851
RPL22	-1.088625411	0.4753557957	0.9999763851
RNF207	-1.3154339304	0.8205576571	0.9999763851
ICMT	1.0740827468	0.6777485154	0.9999763851
GPR153	1.1425651427	0.7870991548	0.9999763851
ACOT7	1.0781361188	0.6851325867	0.9999763851
NOL9	-1.0051786172	0.9736009758	0.9999763851
TAS1R1	0	0	0
ZBTB48	-1.0625597214	0.8949548817	0.9999763851
KLHL21	-1.0686103144	0.7084214503	0.9999763851
PHF13	-1.00008624	0.9996854632	0.9999763851
THAP3	-1.1555612359	0.4216100473	0.9999763851
DNAJC11	-1.5155260696	0.3099023206	0.9999763851
CAMTA1	1.3088858435	0.1900563935	0.9999763851
VAMP3	1.2869549966	0.2759914382	0.9999763851
PER3	-1.4580055137	0.1291807939	0.9999763851
UTS2	11.4763420597	0.2057475773	0.9999763851
TNFRSF9	-1.3709710942	0.748951574	0.9999763851
PARK7	1.0810796221	0.5295730195	0.9999763851
ERRFI1	-1.0166702806	0.977057038	0.9999763851
RERE	-1.2241734493	0.2028619646	0.9999763851
ENO1	-1.0128188539	0.9191809382	0.9999763851
CA6	7.7192231741	0.2780352706	0.9999763851
SLC2A7	3.2353357581	0.554787377	0.9999763851
SLC2A5	1.0597414331	0.7730105805	0.9999763851
GPR157	18.9337530925	0.1183250341	0.9999763851
H6PD	-1.1730435764	0.4602718919	0.9999763851
SPSB1	1.077972134	0.8912753648	0.9999763851

SLC25A33	-1.3986516995	0.1838661971	0.9999763851
TMEM201	-1.2085686918	0.4349202455	0.9999763851
LOC107985734	1.4896325716	0.2380463107	0.9999763851
PIK3CD	-1.3567501826	0.0268320016	0.9999763851
CLSTN1	1.0392187447	0.8232239638	0.9999763851
CTNNBIP1	-1.0507736543	0.7828352545	0.9999763851
LZIC	1.1023423199	0.6141908316	0.9999763851
NMNAT1	-1.264629083	0.5312243894	0.9999763851
RBP7	-4.2849908816	0.4028453076	0.9999763851
UBE4B	1.0657174402	0.8010091842	0.9999763851
KIF1B	1.2219776479	0.3899456608	0.9999763851
PGD	-1.0302013907	0.8711931508	0.9999763851
APITD1	-1.2067848304	0.4417597325	0.9999763851
APITD1-CORT	0	0	0
CORT	-1.0022999659	0.9974194811	0.9999763851
DFFA	-1.2616288061	0.3871490765	0.9999763851
PEX14	-1.4814491832	0.1410820361	0.9999763851
CASZ1	-1.0771227858	0.7558744199	0.9999763851
TARDBP	-1.1914952151	0.1783366818	0.9999763851
MASP2	-4.2849908816	0.4028453076	0.9999763851
SRM	1.2535065026	0.0819309754	0.9999763851
EXOSC10	1.2586203042	0.2628603303	0.9999763851
MTOR	-1.089050219	0.6808115178	0.9999763851
UBIAD1	1.4441434306	0.0682871743	0.9999763851
DISP3	2.1749090749	0.2902963936	0.9999763851
FBXO44	1.0270472827	0.9776236632	0.9999763851
FBXO6	-2.0938791425	0.1311548144	0.9999763851
MAD2L2	1.1817117671	0.4247699789	0.9999763851
DRAXIN	1.0302158107	0.9680755921	0.9999763851
AGTRAP	-1.0450736439	0.7908901697	0.9999763851
MTHFR	1.5899501076	0.1846224876	0.9999763851
CLCN6	1.1567081039	0.665179057	0.9999763851
KIAA2013	-1.1227694078	0.4101468624	0.9999763851
PLOD1	-1.2090169908	0.2501471455	0.9999763851
MFN2	-1.0513352565	0.7880695764	0.9999763851
MIIP	-1.200174099	0.5534347562	0.9999763851
TNFRSF1B	-1.1703221131	0.2908432668	0.9999763851
VPS13D	1.1666597452	0.5375589315	0.9999763851
DHRS3	-1.3715341606	0.4900407406	0.9999763851
HNRNPCL1	-1.0092282152	0.9905053495	0.9999763851
PRDM2	-1.2222613113	0.2975697473	0.9999763851
KAZN	-1.6835365895	0.1330391167	0.9999763851
TMEM51	-1.1492735539	0.8452962127	0.9999763851
C1orf195	0	0	0
EFHD2	1.0048361664	0.977147235	0.9999763851
CTRC	3.2361532718	0.5546934395	0.9999763851
CELA2A	-4.2864494036	0.4027150576	0.9999763851
CELA2B	-1.6822287229	0.4686804752	0.9999763851
CASP9	1.1639808388	0.7442533189	0.9999763851

DNAJC16	-1.0451501088	0.9190510566	0.9999763851
AGMAT	0	0	0
DDI2	1.3406186202	0.7024599618	0.9999763851
RSC1A1	12.4427966444	0.1868322323	0.9999763851
PLEKHM2	-1.3446329162	0.2043512162	0.9999763851
SLC25A34	3.2353357581	0.554787377	0.9999763851
TMEM82	0	0	0
FBLIM1	-4.2849908816	0.4028453076	0.9999763851
UQCRHL	1.3474731584	0.0879372367	0.9999763851
SPEN	1.3087159369	0.2867057139	0.9999763851
ZBTB17	-1.191194284	0.673835896	0.9999763851
ARHGEF19	1.1341393112	0.6975739546	0.9999763851
ANO7P1	3.2361532718	0.5546934395	0.9999763851
RSG1	3.2353357581	0.554787377	0.9999763851
FBXO42	1.5304620398	0.1495632736	0.9999763851
SZRD1	1.2764111599	0.1836186681	0.9999763851
NECAP2	1.0358674562	0.8632038033	0.9999763851
FAM231B	0	0	0
NBPF1	1.2362886503	0.3311611848	0.9999763851
LOC102723383	0	0	0
MST1L	-1.4020601044	0.285441333	0.9999763851
CROCC	-2.6293060006	0.0591438487	0.9999763851
ATP13A2	3.2449000269	0.0177855534	0.9999763851
SDHB	1.1094444485	0.5304868464	0.9999763851
PADI2	-1.2522894312	0.6244386287	0.9999763851
PADI3	-2.3217011239	0.4357700933	0.9999763851
PADI4	1.8137824633	0.5633863058	0.9999763851
RCC2	-1.2273911715	0.1541266858	0.9999763851
ARHGEF10L	-1.3074921711	0.3449107834	0.9999763851
ACTL8	1.5358098925	0.2074089023	0.9999763851
IGSF21	0	0	0
ALDH4A1	1.1910339965	0.3546468207	0.9999763851
IFFO2	1.099610368	0.8314390123	0.9999763851
UBR4	-1.081994277	0.5950480478	0.9999763851
EMC1	-1.1620024768	0.340940226	0.9999763851
MRTO4	1.3227616372	0.1165370528	0.9999763851
AKR7A3	1.3516202149	0.6130596441	0.9999763851
AKR7A2	1.0906266199	0.5924502318	0.9999763851
PQLC2	-1.0610345935	0.8808049213	0.9999763851
CAPZB	-1.3282602261	0.031202376	0.9999763851
MINOS1	1.2877439777	0.0582233431	0.9999763851
MINOS1-NBL1	0	0	0
NBL1	1.0275312208	0.9771627006	0.9999763851
TMCO4	1.2509797067	0.5190719194	0.9999763851
OTUD3	2.1319137281	0.1718552059	0.9999763851
PLA2G2D	0	0	0
PLA2G2C	-1.3063941	0.6034609742	0.9999763851
UBXN10	-2.8642315427	0.0005256	0.5206160142
VWA5B1	-7.5622178268	0.2177145568	0.9999763851

CAMK2N1	1.3668805525	0.4890888903	0.9999763851
MUL1	1.4013617921	0.2512066017	0.9999763851
CDA	1.3549226673	0.2405985083	0.9999763851
PINK1	1.1993785606	0.3627658435	0.9999763851
DDOST	-1.2065714781	0.3230165015	0.9999763851
KIF17	-1.0849160377	0.8968604788	0.9999763851
HP1BP3	1.1671146963	0.2902501932	0.9999763851
EIF4G3	1.0937346032	0.6906345008	0.9999763851
ECE1	1.0466511912	0.8285864559	0.9999763851
NBPF3	-1.866246204	0.0555079382	0.9999763851
RAP1GAP	0	0	0
USP48	-1.1251461385	0.4340374529	0.9999763851
LDLRAD2	1.0279042192	0.9761267789	0.9999763851
HSPG2	0	0	0
CELA3A	5.4781708352	0.3745527127	0.9999763851
CDC42	1.1351419722	0.3345931252	0.9999763851
ZBTB40	-1.1166057691	0.7355956481	0.9999763851
EPHA8	0	0	0
C1QA	1.6117722432	0.3344605921	0.9999763851
C1QB	0	0	0
EPHB2	-1.3158816768	0.8203229797	0.9999763851
KDM1A	-1.098581633	0.684524642	0.9999763851
LUZP1	-1.3038212819	0.2940734353	0.9999763851
HNRNPR	1.2304240465	0.1665833315	0.9999763851
ZNF436	-1.4307993596	0.494865237	0.9999763851
TCEA3	-1.0611824601	0.9005410891	0.9999763851
ASAP3	-1.3154339304	0.8205576571	0.9999763851
E2F2	-1.0347668092	0.8660790216	0.9999763851
ID3	1.2661450094	0.7597475913	0.9999763851
RPL11	1.0194037102	0.8690248342	0.9999763851
TCEB3	-1.0689686945	0.744931492	0.9999763851
PITHD1	1.2581761367	0.166452002	0.9999763851
LYPLA2	1.1268043874	0.4344546813	0.9999763851
GALE	-1.0200757478	0.9389769545	0.9999763851
HMGCL	-1.2336977324	0.5102408997	0.9999763851
FUCA1	-1.0817369301	0.6043949421	0.9999763851
CNR2	1.6099279025	0.1016389644	0.9999763851
PNRC2	1.0542540952	0.7679180619	0.9999763851
SRSF10	1.0157418012	0.9159352384	0.9999763851
MYOM3	0	0	0
IL22RA1	0	0	0
IFNLR1	0	0	0
GRHL3	1.2867653957	0.8303027073	0.9999763851
STPG1	1.3261442362	0.7481308662	0.9999763851
NIPAL3	1.7498218804	0.1302972877	0.9999763851
RCAN3	1.4670354898	0.498132032	0.9999763851
NCMAP	0	0	0
SRRM1	1.0310513695	0.8284256726	0.9999763851
CLIC4	-1.2421295315	0.3306171329	0.9999763851

RUNX3	1.0523960626	0.6920371863	0.9999763851
SYF2	-1.0332529439	0.849509142	0.9999763851
RSRP1	-1.0968404282	0.6244630843	0.9999763851
TMEM50A	1.1663825021	0.4320550433	0.9999763851
RHCE	-1.3475331976	0.7688178297	0.9999763851
TMEM57	1.3751821414	0.2330431253	0.9999763851
LDLRAP1	1.1806295713	0.4113780214	0.9999763851
MAN1C1	1.1326643262	0.8792464483	0.9999763851
SEPN1	1.4404315282	0.1589759444	0.9999763851
MTFR1L	-40.9972410401	0.0218231458	0.9999763851
AUNIP	1.3913138705	0.1063272315	0.9999763851
PAQR7	3.2353357581	0.554787377	0.9999763851
STMN1	1.0636664556	0.5933236956	0.9999763851
PAFAH2	-1.0056922468	0.9879745456	0.9999763851
PDIK1L	-1.2574156331	0.7171083699	0.9999763851
FAM110D	-1.0351013771	0.9593129508	0.9999763851
ZNF593	1.1818495503	0.3186987545	0.9999763851
CNKSR1	3.2356557963	0.5547505991	0.9999763851
CATSPER4	0	0	0
CEP85	-1.0822680815	0.7944456243	0.9999763851
SH3BGRL3	1.2286048015	0.1992119706	0.9999763851
UBXN11	1.027554661	0.9756517998	0.9999763851
CD52	-1.0404139104	0.8243362932	0.9999763851
LIN28A	-4.2849908816	0.4028453076	0.9999763851
DHDDS	-1.6797920108	0.0670160399	0.9999763851
HMGN2	1.4199846747	0.004526	0.9999763851
RPS6KA1	1.0096851677	0.9590364726	0.9999763851
ARID1A	1.0309196656	0.8583610318	0.9999763851
PIGV	1.511699786	0.4786618003	0.9999763851
ZDHHC18	1.2242987547	0.4016810075	0.9999763851
SFN	-1.1491729646	0.847779038	0.9999763851
GPN2	1.0648972176	0.8542674712	0.9999763851
GPATCH3	1.1565287774	0.6517534415	0.9999763851
NUDC	1.2108105044	0.2338512031	0.9999763851
KDF1	0	0	0
TRNP1	1.62522264	0.5744236099	0.9999763851
SLC9A1	1.2006389141	0.6175123491	0.9999763851
WDTC1	1.12167016	0.7327891125	0.9999763851
TMEM222	-1.0957233907	0.7450479096	0.9999763851
SYTL1	1.2448173907	0.6109771672	0.9999763851
MAP3K6	-1.6685488022	0.2499416412	0.9999763851
FCN3	0	0	0
GPR3	3.2361532718	0.5546934395	0.9999763851
WASF2	1.0901609584	0.5925482224	0.9999763851
AHDC1	1.101622642	0.7783063855	0.9999763851
FGR	1.0677232935	0.7730948165	0.9999763851
IFI6	-5.4025441211	6.16E-10	3.01E-06
FAM76A	-2.2349198472	0.0703768246	0.9999763851
STX12	-1.2304642165	0.428059802	0.9999763851

PPP1R8	1.5680697646	0.009504	0.9999763851
THEMIS2	1.2339528129	0.2922779136	0.9999763851
RPA2	1.1365826064	0.3799677251	0.9999763851
SMPDL3B	-7.5622178268	0.2177145568	0.9999763851
XKR8	1.3706686629	0.3674901675	0.9999763851
EYA3	-1.2688574231	0.1806205706	0.9999763851
PTAFR	-1.7460333819	0.00886	0.9999763851
DNAJC8	1.0010121842	0.9948902754	0.9999763851
ATPIF1	-1.0168057009	0.8978314225	0.9999763851
SESN2	-1.513336191	0.1914034435	0.9999763851
MED18	1.5329222667	0.1264405606	0.9999763851
PHACTR4	1.1823043678	0.40033852	0.9999763851
RCC1	-1.033461336	0.8088548783	0.9999763851
TRNAU1AP	1.0648769255	0.7827119148	0.9999763851
TAF12	-1.1813360361	0.3721629073	0.9999763851
GMEB1	-1.0587083132	0.8560791701	0.9999763851
YTHDF2	1.2607475794	0.1057631326	0.9999763851
OPRD1	0	0	0
EPB41	-1.203763665	0.4854706396	0.9999763851
TMEM200B	1.0834237965	0.6104609929	0.9999763851
SRSF4	-1.1570466299	0.4136713489	0.9999763851
MECR	-1.4173138948	0.1886477124	0.9999763851
PTPRU	1.1107539683	0.8060895868	0.9999763851
MATN1	-1.9577848643	0.3993333243	0.9999763851
LAPTM5	-1.0607557082	0.6493937013	0.9999763851
PUM1	1.060730575	0.7073312768	0.9999763851
NKAIN1	-4.2849908816	0.4028453076	0.9999763851
SNRNP40	-1.0562260042	0.8655201862	0.9999763851
ZCCHC17	1.5213857738	0.3095286202	0.9999763851
FABP3	1.2254478425	0.348313789	0.9999763851
SERINC2	-1.081459568	0.7503780051	0.9999763851
TINAGL1	0	0	0
HCRTR1	0	0	0
PEF1	1.1734555506	0.4645705727	0.9999763851
COL16A1	0	0	0
ADGRB2	0	0	0
SPOCD1	-15.8725894304	0.00719	0.9999763851
PTP4A2	-1.0889136629	0.4986668648	0.9999763851
KHDRBS1	1.2996534026	0.0558781468	0.9999763851
TMEM39B	-1.3277520943	0.2270795529	0.9999763851
KPNA6	1.3806841363	0.0727999418	0.9999763851
TXLNA	1.5717321995	0.0306217739	0.9999763851
CCDC28B	1.0169984421	0.9311680911	0.9999763851
TMEM234	3.2361532718	0.5546934395	0.9999763851
IQCC	1.1338949431	0.8799751866	0.9999763851
DCDC2B	0	0	0
EIF3I	1.489220349	0.0129344258	0.9999763851
LCK	-1.9657157813	0.458714207	0.9999763851
HDAC1	1.1106681006	0.4844432564	0.9999763851

MARCKSL1	-1.4443312498	0.0298793785	0.9999763851
FAM229A	-1.3942010814	0.6863515839	0.9999763851
TSSK3	0	0	0
BSDC1	-1.8123280412	0.0752509865	0.9999763851
ZBTB8A	1.2272294314	0.6566691172	0.9999763851
ZBTB8OS	1.1013490248	0.7307733939	0.9999763851
RBBP4	1.8465089398	0.0737870877	0.9999763851
SYNC	1.0343561594	0.8545529821	0.9999763851
KIAA1522	1.0301493879	0.9655000661	0.9999763851
YARS	1.2456231905	0.1417942902	0.9999763851
S100PBP	-1.0241720764	0.9340862931	0.9999763851
FNDC5	-7.5647903763	0.2176246281	0.9999763851
TMEM54	1.9215420498	0.4502798488	0.9999763851
RNF19B	1.3712054902	0.2544792138	0.9999763851
AK2	-1.0332253788	0.8009381203	0.9999763851
AZIN2	-1.3713173847	0.744373445	0.9999763851
TRIM62	-1.1974053127	0.8020098371	0.9999763851
ZNF362	1.2507927833	0.5167684076	0.9999763851
A3GALT2	-4.2849908816	0.4028453076	0.9999763851
PHC2	-1.3864284638	0.1488605724	0.9999763851
ZSCAN20	1.8139617088	0.5768737227	0.9999763851
CSMD2	-1.315303824	0.8206171563	0.9999763851
SMIM12	1.0429840383	0.8268966976	0.9999763851
DLGAP3	0	0	0
TMEM35B	-1.1045245155	0.8011295043	0.9999763851
ZMYM6	-1.1850181137	0.5348878489	0.9999763851
ZMYM1	-1.0288429291	0.9342748108	0.9999763851
SFPQ	-1.2135663739	0.123071045	0.9999763851
ZMYM4	-1.0484473863	0.7235662879	0.9999763851
KIAA0319L	-1.0634223685	0.7145210223	0.9999763851
NCDN	1.3072634951	0.6713947708	0.9999763851
TFAP2E	1.1465380583	0.5868284037	0.9999763851
PSMB2	1.1440482277	0.3652391144	0.9999763851
C1orf216	1.0577637794	0.8743426099	0.9999763851
CLSPN	1.2867573272	0.1587778789	0.9999763851
AGO4	-1.419097851	0.1563229254	0.9999763851
AGO1	1.0977887122	0.6986308333	0.9999763851
AGO3	-1.3423274056	0.3017980552	0.9999763851
ADPRHL2	-1.4063529219	0.0502653695	0.9999763851
COL8A2	3.2361532718	0.5546934395	0.9999763851
TRAPPC3	1.0402064676	0.8071220241	0.9999763851
MAP7D1	1.3630876688	0.1107116129	0.9999763851
THRAP3	1.1953128571	0.1736719219	0.9999763851
SH3D21	2.3406309168	0.4061106601	0.9999763851
EVA1B	-2.3222877795	0.4499253493	0.9999763851
STK40	1.3627588291	0.0800004676	0.9999763851
LSM10	1.2707950058	0.1873159281	0.9999763851
OSCP1	-1.1229725452	0.8410441136	0.9999763851
MRPS15	1.0072888836	0.9800625082	0.9999763851

CSF3R	1.1980054699	0.496096585	0.9999763851
GRIK3	-1.8670181425	0.1635603156	0.9999763851
ZC3H12A	1.1363282285	0.4620162506	0.9999763851
MEAF6	-1.4783698659	0.0473098713	0.9999763851
SNIP1	-1.4838422186	0.175033542	0.9999763851
DNALI1	0	0	0
GNL2	-1.3070153459	0.1727987443	0.9999763851
C1orf109	-1.1159279385	0.8336084609	0.9999763851
CDCA8	-1.2162283908	0.1301918354	0.9999763851
EPHA10	0	0	0
MANEAL	-1.2336255799	0.4117282158	0.9999763851
YRDC	-1.2254727571	0.4229349423	0.9999763851
C1orf122	1.0639161696	0.7284146051	0.9999763851
MTF1	-1.3756051828	0.2744568868	0.9999763851
INPP5B	1.2824126562	0.2131103937	0.9999763851
SF3A3	-1.1678705615	0.2763331792	0.9999763851
FHL3	1.1752618031	0.4351565841	0.9999763851
UTP11	1.2816916059	0.1137338877	0.9999763851
POU3F1	0	0	0
RRAGC	-1.0850088641	0.6351472896	0.9999763851
MYCBP	1.4733430206	0.0302198045	0.9999763851
GJA9	7.722359209	0.2874653569	0.9999763851
RHBDL2	1.9234638322	0.4244885585	0.9999763851
AKIRIN1	-1.1223776694	0.5739319236	0.9999763851
NDUFS5	-1.0370693717	0.7759800332	0.9999763851
MACF1	-1.2899927641	0.1050251605	0.9999763851
BMP8A	1.3264062359	0.7554980028	0.9999763851
PABPC4	-1.4659153476	0.0153432966	0.9999763851
HEYL	0	0	0
HPCAL4	0	0	0
PPIE	1.0454151045	0.8214937213	0.9999763851
BMP8B	7.4933530102	0.2989996747	0.9999763851
OXCT2	3.2356557963	0.5547505991	0.9999763851
TRIT1	-1.204003756	0.4414480557	0.9999763851
MYCL	2.7978015099	0.1325958573	0.9999763851
MFSD2A	1.0516550026	0.8337114477	0.9999763851
CAP1	1.2786951759	0.0901760968	0.9999763851
PPT1	-1.0827602096	0.5718902911	0.9999763851
RLF	-1.2041312042	0.4283639205	0.9999763851
ZMPSTE24	1.3774094353	0.1978727565	0.9999763851
COL9A2	1.2654869437	0.5583906881	0.9999763851
SMAP2	1.1673937482	0.4425049331	0.9999763851
ZFP69B	2.8203441424	0.209650288	0.9999763851
ZFP69	-1.4222660396	0.6158386455	0.9999763851
EXO5	2.1067706904	0.1124684758	0.9999763851
ZNF684	-2.321702064	0.1782047152	0.9999763851
RIMS3	1.018375304	0.95694932	0.9999763851
NFYC	-1.0897062364	0.7564935629	0.9999763851
KCNQ4	3.2356557963	0.5547505991	0.9999763851

CITED4	1.1033113336	0.6464066241	0.9999763851
CTPS1	1.179096483	0.343188561	0.9999763851
SLFNL1	1.3576108079	0.7959116151	0.9999763851
SCMH1	-1.1459226781	0.4879558582	0.9999763851
FOXO6	1.1084459858	0.8163228227	0.9999763851
HIVEP3	-3.2405822385	0.0483564757	0.9999763851
FOXJ3	-1.1273761182	0.5244907001	0.9999763851
RIMKLA	0	0	0
ZMYND12	-4.2849908816	0.4028453076	0.9999763851
PPCS	1.0657023898	0.7686289001	0.9999763851
CCDC30	-3.1526523138	0.2046958923	0.9999763851
PPIH	-1.0749600167	0.6116493447	0.9999763851
YBX1	1.0126449093	0.9394509667	0.9999763851
P3H1	-1.0094521501	0.9668420056	0.9999763851
C1orf50	1.2131929708	0.6325554469	0.9999763851
LOC100129924	0	0	0
SVBP	1.7286417065	0.2511120207	0.9999763851
ERMAP	-1.4348894959	0.4650630281	0.9999763851
ZNF691	-1.8628321812	0.3658551233	0.9999763851
SLC2A1	1.261520985	0.1727866701	0.9999763851
EBNA1BP2	1.204286912	0.2517569072	0.9999763851
CFAP57	-4.2849908816	0.4028453076	0.9999763851
C1orf210	0	0	0
TIE1	0	0	0
MPL	0	0	0
CDC20	1.3981109525	0.0714615304	0.9999763851
ELOVL1	1.0688798244	0.8221522541	0.9999763851
MED8	1.0334395702	0.8694987083	0.9999763851
SZT2	-2.712424071	0.1658745147	0.9999763851
HYI	1.2418628383	0.3905338457	0.9999763851
PTPRF	-1.9096097289	0.1145861384	0.9999763851
KDM4A	1.1138255623	0.5474097204	0.9999763851
ST3GAL3	1.0012855074	0.9970513974	0.9999763851
IPO13	1.9412048835	0.043864734	0.9999763851
DPH2	1.4523595612	0.1355191493	0.9999763851
ATP6V0B	-1.2734918491	0.0805539925	0.9999763851
B4GALT2	-1.0930164211	0.6262146172	0.9999763851
CCDC24	-2.2413309057	0.3242360431	0.9999763851
SLC6A9	0	0	0
KLF17	1.0243527553	0.93160954	0.9999763851
DMAP1	2.014637565	0.176361245	0.9999763851
ERI3	-1.301206318	0.3350862144	0.9999763851
RNF220	-1.0263134702	0.8769070476	0.9999763851
TMEM53	1.3683883835	0.4355491148	0.9999763851
C1orf228	-1.2441005715	0.2063254001	0.9999763851
KIF2C	1.0938915703	0.5434066598	0.9999763851
RPS8	-1.0546302799	0.6763404266	0.9999763851
BEST4	-7.5622178268	0.2177145568	0.9999763851
PLK3	-1.1806494914	0.6083184779	0.9999763851

TCTEX1D4	6.368054416	0.3491336461	0.9999763851
BTBD19	-4.2849908816	0.4028453076	0.9999763851
PTCH2	2.3403997491	0.4168427477	0.9999763851
EIF2B3	1.234625829	0.3840771048	0.9999763851
HECTD3	-1.3079652595	0.5952824969	0.9999763851
UROD	1.3983273592	0.02344798	0.9999763851
ZSWIM5	9.9624059448	0.2315301533	0.9999763851
HPDL	0	0	0
MUTYH	1.001554667	0.997215672	0.9999763851
TOE1	1.4418110646	0.1201378768	0.9999763851
TESK2	-2.4944383406	0.1879532499	0.9999763851
MMACHC	1.1233250589	0.7732186807	0.9999763851
PRDX1	1.0040527974	0.976366907	0.9999763851
AKR1A1	-1.0867381317	0.6362519242	0.9999763851
NASP	1.1405989486	0.309707639	0.9999763851
GPBP1L1	-1.0987944205	0.5975313729	0.9999763851
TMEM69	-1.138064785	0.6629594767	0.9999763851
IPP	1.1934007193	0.7476980061	0.9999763851
MAST2	1.171017994	0.5779643661	0.9999763851
PIK3R3	-1.4249978991	0.1616800594	0.9999763851
TSPAN1	0	0	0
POMGNT1	1.5197618288	0.3428943336	0.9999763851
RAD54L	1.0174672472	0.9475685472	0.9999763851
LRRC41	-1.0209425382	0.9263620071	0.9999763851
UQCRH	1.2039942633	0.1173091837	0.9999763851
NSUN4	-1.0544361942	0.8460527835	0.9999763851
FAAH	1.7492118798	0.2028231509	0.9999763851
DMBX1	0	0	0
MKNK1	1.1287633192	0.5908690192	0.9999763851
MOB3C	1.0314251651	0.9600071573	0.9999763851
ATPAF1	-1.0958025414	0.7522087972	0.9999763851
TEX38	0	0	0
EFCAB14	1.2778015554	0.2316317792	0.9999763851
CYP4Z1	5.4781708352	0.3745527127	0.9999763851
STIL	-1.2712084615	0.2304664548	0.9999763851
CMPK1	-1.1278467171	0.4998078876	0.9999763851
FOXE3	3.2356557963	0.5547505991	0.9999763851
FOXD2	2.3920809821	0.1294240634	0.9999763851
TRABD2B	0	0	0
SPATA6	-1.6825599095	0.4644371097	0.9999763851
AGBL4	-1.3475331976	0.7683386714	0.9999763851
ELAVL4	-4.2864494036	0.4027150576	0.9999763851
FAF1	-1.1424742321	0.5069642762	0.9999763851
CDKN2C	1.2604632305	0.3387733237	0.9999763851
RNF11	1.6221515261	0.0257843808	0.9999763851
TTC39A	3.2353357581	0.554787377	0.9999763851
EPS15	1.1509760211	0.4572438922	0.9999763851
OSBPL9	-1.1619456025	0.3883086625	0.9999763851
NRDC	1.3577275062	0.0760404766	0.9999763851

RAB3B	0	0	0
TXNDC12	1.2125014789	0.162345766	0.9999763851
KTI12	1.3780698625	0.3836035132	0.9999763851
BTF3L4	-1.4670098702	0.0315139786	0.9999763851
ZFYVE9	-1.1288786827	0.7329015124	0.9999763851
CC2D1B	-1.1353715088	0.7631190466	0.9999763851
ORC1	1.1653216603	0.449537265	0.9999763851
PRPF38A	1.0465644099	0.765633682	0.9999763851
ZCCHC11	-1.1705331575	0.4652149051	0.9999763851
GPX7	0	0	0
COA7	1.241435741	0.5504933027	0.9999763851
ZYG11B	1.1218493062	0.4150157654	0.9999763851
ZYG11A	2.1492013829	0.2180858772	0.9999763851
ECHDC2	-1.4083241465	0.6544677626	0.9999763851
SCP2	-1.1459447402	0.5342818151	0.9999763851
SLC1A7	-4.2849908816	0.4028453076	0.9999763851
CPT2	1.3898267733	0.3157861028	0.9999763851
C1orf123	1.282769805	0.2398022004	0.9999763851
MAGOH	-1.3661819099	0.0848880741	0.9999763851
LRP8	-1.1599241494	0.2844723797	0.9999763851
GLIS1	-3.3295429242	0.2525387851	0.9999763851
NDC1	-1.0295957802	0.8586732177	0.9999763851
YIPF1	1.4693104963	0.2202750165	0.9999763851
DIO1	-1.3155492632	0.8262553345	0.9999763851
HSPB11	-1.2880687492	0.1808757109	0.9999763851
LRRC42	-1.1254239821	0.4771152926	0.9999763851
TMEM59	-1.0319373506	0.8294880011	0.9999763851
TCEANC2	1.61881294	0.1082493465	0.9999763851
CYB5RL	-1.6268130118	0.27816549	0.9999763851
MRPL37	-1.076789284	0.6325273204	0.9999763851
SSBP3	-1.0999915452	0.5245488907	0.9999763851
ACOT11	-2.6351957541	0.2411378651	0.9999763851
FAM151A	1.2306513941	0.5770694621	0.9999763851
TTC4	1.056290461	0.7649869104	0.9999763851
PARS2	1.4989395884	0.3648968522	0.9999763851
TTC22	0	0	0
LEXM	-1.3157515262	0.8228795024	0.9999763851
DHCR24	-1.4167981913	0.0100058065	0.9999763851
TMEM61	0	0	0
USP24	1.1359023621	0.6007514195	0.9999763851
PLPP3	1.0779215982	0.8988615317	0.9999763851
C1orf168	0	0	0
C8B	3.2356557963	0.5547505991	0.9999763851
DAB1	4.4484168982	0.1372823053	0.9999763851
OMA1	-1.1263510553	0.7560129484	0.9999763851
MYSM1	-1.1929248172	0.6000005937	0.9999763851
JUN	-1.6499275215	0.0107187099	0.9999763851
FGGY	1.1254081046	0.7375101646	0.9999763851
HOOK1	3.2353357581	0.554787377	0.9999763851

NFIA	1.6796325003	0.0765620578	0.9999763851
TM2D1	-1.4714785237	0.0383601022	0.9999763851
PATJ	1.2869631997	0.8233366737	0.9999763851
KANK4	1.4171435615	0.3670631318	0.9999763851
USP1	1.006724314	0.9628074921	0.9999763851
DOCK7	-1.5066556591	0.4259607502	0.9999763851
ATG4C	-1.1876800422	0.7267025893	0.9999763851
ALG6	1.1598967152	0.6272186165	0.9999763851
ITGB3BP	-1.038488582	0.8522071856	0.9999763851
EFCAB7	-1.6179658945	0.2563496448	0.9999763851
PGM1	1.5159143692	0.003727	0.9999763851
ROR1	0	0	0
CACHD1	-4.2864494036	0.4027150576	0.9999763851
RAVER2	-1.277986891	0.6565646065	0.9999763851
JAK1	-1.0607228959	0.750178617	0.9999763851
LOC107983962	1.2872739558	0.823346684	0.9999763851
AK4	1.1160385574	0.6016049397	0.9999763851
DNAJC6	1.0277462901	0.9757638168	0.9999763851
LEPROT	-1.4411210315	0.1934824586	0.9999763851
PDE4B	1.1549158921	0.6282365768	0.9999763851
SGIP1	0	0	0
WDR78	1.6251513227	0.5896897148	0.9999763851
MIER1	-1.1526540481	0.6110127086	0.9999763851
SLC35D1	-1.0052802913	0.9920420512	0.9999763851
IL12RB2	2.3403997491	0.4169269104	0.9999763851
SERBP1	1.06332528	0.6012211798	0.9999763851
GADD45A	-2.6444442696	0.0290133933	0.9999763851
GNG12	1.0452961372	0.893151198	0.9999763851
DIRAS3	0	0	0
DEPDC1	1.0812620512	0.8113653217	0.9999763851
LRRC7	-1.080916289	0.9219620637	0.9999763851
LRRC40	-1.1592062522	0.5341309734	0.9999763851
SRSF11	-1.0835638365	0.5394004115	0.9999763851
ANKRD13C	-1.136622131	0.6466843829	0.9999763851
HHLA3	1.6688831311	0.4451512916	0.9999763851
CTH	-1.7399553654	0.2241587259	0.9999763851
PTGER3	0	0	0
ZRANB2	1.0551770608	0.6951717105	0.9999763851
NEGR1	-1.0156680097	0.9726446607	0.9999763851
FPGT	-1.2875792692	0.7315004988	0.9999763851
FPGT-TNNI3K	4.3099016468	0.2319860646	0.9999763851
TNNI3K	3.2353357581	0.554787377	0.9999763851
LOC105378803	0	0	0
CRYZ	-1.1821581157	0.7825378475	0.9999763851
TYW3	-1.1379034424	0.5983812248	0.9999763851
ACADM	-1.0665218744	0.7246986445	0.9999763851
RABGGTB	1.2812219559	0.5185707431	0.9999763851
MSH4	0	0	0
ST6GALNAC3	-1.7080263853	0.0927863622	0.9999763851

PIGK	-1.0256958331	0.8668538253	0.9999763851
AK5	-1.0810230665	0.9184042055	0.9999763851
ZZZ3	-1.0771341689	0.6715152479	0.9999763851
USP33	-1.0123051483	0.9515305478	0.9999763851
FAM73A	-1.1489484111	0.6822387202	0.9999763851
NEXN	-2.5591355727	0.2873473728	0.9999763851
FUBP1	-1.1215377691	0.4133570466	0.9999763851
DNAJB4	1.0329089223	0.9460111055	0.9999763851
IFI44L	0	0	0
IFI44	-7.5690339649	0.2288949944	0.9999763851
ADGRL4	0	0	0
ADGRL2	-8.342239448	0.2168368514	0.9999763851
PRKACB	1.8752000307	0.2887564352	0.9999763851
SAMD13	1.5491073841	0.5508222679	0.9999763851
RPF1	-1.007125149	0.970882643	0.9999763851
GNG5	1.2197277957	0.171195485	0.9999763851
SPATA1	-1.901207257	0.1080420508	0.9999763851
CTBS	-1.2618921825	0.5401062119	0.9999763851
SSX2IP	1.4848798847	0.2478331301	0.9999763851
WDR63	3.2361532718	0.5546934395	0.9999763851
SYDE2	1.0920005331	0.8613118991	0.9999763851
C1orf52	1.0934518004	0.5945647388	0.9999763851
BCL10	-1.6542940914	0.0665958873	0.9999763851
DDAH1	0	0	0
ZNHIT6	-1.066036495	0.7796831474	0.9999763851
COL24A1	0	0	0
ODF2L	3.21452506	0.0699367023	0.9999763851
CLCA4	0	0	0
SH3GLB1	1.0482768737	0.8253941624	0.9999763851
SEP15	1.0120557381	0.9456939363	0.9999763851
HS2ST1	-1.5058913433	0.0345192174	0.9999763851
LMO4	1.252266097	0.3549176189	0.9999763851
PKN2	1.1983007114	0.5365931953	0.9999763851
GTF2B	1.2675988651	0.2998947978	0.9999763851
KYAT3	-1.0257173553	0.9250827778	0.9999763851
RBMXL1	1.7264313563	0.1579628373	0.9999763851
GBP3	5.4770877341	0.3746135179	0.9999763851
GBP1	-4.2864494036	0.4027150576	0.9999763851
GBP2	0	0	0
GBP5	-1.1747890416	0.8269564911	0.9999763851
LRRC8B	1.0307744197	0.9406823856	0.9999763851
LRRC8C	-1.3444874888	0.3005306634	0.9999763851
LRRC8D	-1.7006561141	0.034361671	0.9999763851
ZNF326	1.0671236887	0.7940501348	0.9999763851
ZNF644	1.0881377686	0.6718445569	0.9999763851
HFM1	-1.5886842618	0.3607312972	0.9999763851
CDC7	-1.0652127259	0.8215513268	0.9999763851
TGFBR3	-1.327843339	0.3520694664	0.9999763851
BRDT	0	0	0

EPHX4	3.2361532718	0.5546934395	0.9999763851
SETSIP	5.4784711963	0.3745358536	0.9999763851
C1orf146	0	0	0
GLMN	1.1493689076	0.4326194013	0.9999763851
RPAP2	-1.3158393667	0.828121228	0.9999763851
GFI1	1.0169248846	0.9055430262	0.9999763851
EVI5	1.1146588378	0.8394716519	0.9999763851
RPL5	1.0325206885	0.7873666158	0.9999763851
FAM69A	2.1485439616	0.2194572952	0.9999763851
MTF2	1.0775093189	0.6902472954	0.9999763851
TMED5	1.0629953743	0.7259689147	0.9999763851
CCDC18	-1.2954278811	0.2412495494	0.9999763851
DR1	-1.0875125239	0.6995979514	0.9999763851
FNBP1L	-1.1957618347	0.7958145403	0.9999763851
BCAR3	-1.0157357434	0.9794065735	0.9999763851
DNTTIP2	1.1951412336	0.3656962456	0.9999763851
GCLM	1.0355595807	0.8747065604	0.9999763851
ABCD3	-1.1382090997	0.5825470763	0.9999763851
F3	-1.3219738921	0.6269807691	0.9999763851
SLC44A3	3.2353357581	0.554787377	0.9999763851
ALG14	1.0332174016	0.9436058602	0.9999763851
TMEM56	-4.2849908816	0.4028453076	0.9999763851
RWDD3	-1.2041751285	0.6660799448	0.9999763851
PTBP2	1.0050067075	0.9857920479	0.9999763851
DPYD	-1.4193729064	0.1627447451	0.9999763851
PLPPR4	3.2356557963	0.5547505991	0.9999763851
AGL	1.2737544387	0.5539509627	0.9999763851
SLC35A3	-1.4144790023	0.2146920306	0.9999763851
MFSD14A	-1.4875632814	0.3719236359	0.9999763851
SASS6	1.3874498252	0.1484160027	0.9999763851
TRMT13	1.2653379663	0.5521026142	0.9999763851
LRRC39	2.5213873366	0.2621812916	0.9999763851
DBT	-1.0851002247	0.6817333818	0.9999763851
RTCA	-1.0780913449	0.7094130514	0.9999763851
CDC14A	1.255251492	0.6084776913	0.9999763851
EXTL2	1.7126026959	0.0716652317	0.9999763851
SLC30A7	1.1072566221	0.631074171	0.9999763851
DPH5	-1.1273829776	0.7965249122	0.9999763851
OLFM3	0	0	0
COL11A1	-1.3157515262	0.822832452	0.9999763851
LOC101928436	3.2353357581	0.554787377	0.9999763851
RNPC3	4.9062743278	0.003441	0.9999763851
AMY2B	-1.2118231382	0.7205584961	0.9999763851
AMY1B	0	0	0
PRMT6	1.0581252742	0.8759791608	0.9999763851
NTNG1	0	0	0
VAV3	1.35378055	0.1921197091	0.9999763851
SLC25A24	-1.406703979	0.06204141	0.9999763851
FAM102B	-1.564234202	0.438695862	0.9999763851

HENMT1	-1.1099481984	0.6063269117	0.9999763851
PRPF38B	-1.2074534887	0.2608145278	0.9999763851
FNDC7	0	0	0
STXBP3	-1.387737892	0.3483584401	0.9999763851
AKNAD1	0	0	0
GPSM2	-1.1207577255	0.7357632184	0.9999763851
CLCC1	-1.3415817296	0.1625407549	0.9999763851
WDR47	-1.079137627	0.8148515104	0.9999763851
TAF13	5.4770877341	0.3746135179	0.9999763851
TMEM167B	-1.9447695232	0.019663035	0.9999763851
SARS	1.3065974407	0.1868383053	0.9999763851
CELSR2	-1.1960247227	0.7898838677	0.9999763851
PSRC1	1.0883895327	0.7267532753	0.9999763851
SORT1	0	0	0
PSMA5	1.1071728435	0.6246323091	0.9999763851
ATXN7L2	-1.1492864126	0.8527946758	0.9999763851
CYB561D1	-1.0633232478	0.8526468959	0.9999763851
AMIGO1	3.2356557963	0.5547505991	0.9999763851
GNAI3	1.1565285708	0.3068819808	0.9999763851
GNAT2	-1.3950272772	0.6764095329	0.9999763851
AMPD2	-1.2319178835	0.322793879	0.9999763851
GSTM1	-1.3154339304	0.8205576571	0.9999763851
CSF1	0	0	0
AHCYL1	-1.2230577047	0.1326607278	0.9999763851
STRIP1	1.0657902922	0.7369663567	0.9999763851
ALX3	-1.5727227257	0.376861244	0.9999763851
KCNC4	1.127376782	0.6971269255	0.9999763851
RBM15	-1.0788728479	0.6948006004	0.9999763851
SLC16A4	1.2000422762	0.7007775812	0.9999763851
LAMTOR5	1.221469121	0.188506146	0.9999763851
KCNA3	-7.5622178268	0.2177145568	0.9999763851
CD53	1.8396376641	0.0682631385	0.9999763851
LRIF1	-1.1976607876	0.4442197914	0.9999763851
DRAM2	-1.2428847538	0.344709214	0.9999763851
CEPT1	-1.0065185197	0.9791000939	0.9999763851
DENND2D	1.287490194	0.2178410691	0.9999763851
CHI3L2	0	0	0
PIFO	3.2353357581	0.554787377	0.9999763851
OVGP1	7.7204093521	0.288300348	0.9999763851
WDR77	1.1733753465	0.4284249163	0.9999763851
ATP5F1	1.0709323043	0.5713702809	0.9999763851
C1orf162	-1.4016562451	0.2060864647	0.9999763851
TMIGD3	1.813344484	0.5819592412	0.9999763851
ADORA3	3.2353357581	0.554787377	0.9999763851
RAP1A	-1.1672483763	0.2994057901	0.9999763851
LOC107985184	3.2353357581	0.554787377	0.9999763851
FAM212B	1.9703420756	0.2529395872	0.9999763851
DDX20	1.0908848551	0.7194808803	0.9999763851
CTTNBP2NL	-1.7346211034	0.444497219	0.9999763851

WNT2B	0	0	0
ST7L	-1.2978410352	0.4227121455	0.9999763851
CAPZA1	1.0675562467	0.6370398968	0.9999763851
MOV10	1.2334603554	0.4237737167	0.9999763851
RHOC	1.2656255628	0.4144301139	0.9999763851
PPM1J	1.0277578074	0.9761746374	0.9999763851
FAM19A3	0	0	0
SLC16A1	1.2339578655	0.1610249189	0.9999763851
LRIG2	-1.8322554312	0.071015689	0.9999763851
MAGI3	-1.0366595916	0.9301123367	0.9999763851
PHTF1	1.0706784256	0.7988772108	0.9999763851
RSBN1	-1.9102531985	0.0161900996	0.9999763851
PTPN22	-2.0518164499	0.0106006161	0.9999763851
BCL2L15	1.027554661	0.975638146	0.9999763851
AP4B1	1.1972041652	0.4885946472	0.9999763851
DCLRE1B	-1.2078395962	0.4969751633	0.9999763851
HIPK1	-1.0513870735	0.7797007743	0.9999763851
OLFML3	-7.5664585246	0.2266693929	0.9999763851
TRIM33	-1.1154023967	0.4992088404	0.9999763851
BCAS2	1.2332193498	0.3129737184	0.9999763851
DENND2C	-1.315303824	0.8206171563	0.9999763851
NRAS	-1.0838610897	0.7169438978	0.9999763851
CSDE1	-1.3512044182	0.0128127024	0.9999763851
SIKE1	1.0155076801	0.9463013852	0.9999763851
SYCP1	2.6096308531	0.3725895477	0.9999763851
TSPAN2	-1.0576047247	0.8260322152	0.9999763851
VANGL1	-1.0154795398	0.9374804827	0.9999763851
SLC22A15	-1.7136463658	0.086579135	0.9999763851
MAB21L3	1.8144198156	0.5860619697	0.9999763851
ATP1A1	1.0324660122	0.8323123889	0.9999763851
CD58	-1.2302268426	0.3546395784	0.9999763851
IGSF3	-2.062085586	0.325289347	0.9999763851
CD2	-7.5622178268	0.2177145568	0.9999763851
CD101	-1.3259774455	0.7080148362	0.9999763851
TTF2	1.1213178826	0.5499800545	0.9999763851
TRIM45	-1.4294697437	0.5411731667	0.9999763851
MAN1A2	-1.2502135669	0.2668853316	0.9999763851
FAM46C	1.0518616614	0.7611607995	0.9999763851
GDAP2	-1.4482036688	0.272343121	0.9999763851
WDR3	1.0838966575	0.6590437066	0.9999763851
SPAG17	2.2691674219	0.0879064108	0.9999763851
TBX15	0	0	0
WARS2	1.2193952735	0.5383942732	0.9999763851
ZNF697	-1.1540493772	0.7927618033	0.9999763851
PHGDH	1.2342417119	0.1751241068	0.9999763851
NOTCH2	-1.3037390021	0.1556512424	0.9999763851
SEC22B	-1.0160529687	0.9342810764	0.9999763851
NBPF8	1.3332507287	0.4500770998	0.9999763851
LOC100996724	-1.8944365684	0.4327296728	0.9999763851

LOC101929796	-2.5587422769	0.2940369625	0.9999763851
NBPF26	-12.8287151494	0.0007027	0.6510516988
PPIAL4A	-1.9555716929	0.4957436191	0.9999763851
LOC100996720	1.8136032532	0.5955006303	0.9999763851
LOC105369199	1.3256045991	0.7561119736	0.9999763851
LOC100996721	-2.2382543016	0.2851758488	0.9999763851
LOC100996318	1.6568794408	0.5804676196	0.9999763851
FAM72B	-1.381021336	0.3273191067	0.9999763851
SRGAP2C	1.1016093311	0.6891633823	0.9999763851
LOC107985108	-1.6205404778	0.4233854166	0.9999763851
FAM72C	1.2671394648	0.7711183844	0.9999763851
PPIAL4E	0	0	0
NBPF15	-1.432833698	0.1306402296	0.9999763851
PPIAL4F	-4.2849908816	0.4028453076	0.9999763851
SRGAP2B	1.0686036807	0.8111207848	0.9999763851
FAM72D	1.8974640918	0.2189315318	0.9999763851
LOC101929805	2.3407592913	0.4273028978	0.9999763851
NBPF20	1.1336215097	0.8697170658	0.9999763851
LOC105371303	1.5317288444	0.3936174128	0.9999763851
GPR89A	1.1914041795	0.4211765317	0.9999763851
PDZK1	-1.2936836943	0.6069785979	0.9999763851
CD160	-1.435564605	0.4659176213	0.9999763851
RNF115	1.3555294217	0.1142880866	0.9999763851
POLR3C	1.315789257	0.2645690455	0.9999763851
NUDT17	-1.4308433661	0.1249730051	0.9999763851
ANKRD35	1.0424794224	0.8550773258	0.9999763851
ITGA10	0	0	0
PEX11B	1.6273535305	0.0377111398	0.9999763851
RBM8A	1.0422702584	0.7451489387	0.9999763851
LIX1L	-1.2426114623	0.2366273813	0.9999763851
POLR3GL	-1.490738166	0.1044646868	0.9999763851
ANKRD34A	2.34099053	0.4001141468	0.9999763851
TXNIP	-1.1741521134	0.5096025456	0.9999763851
NBPF10	1.2867653957	0.8302601326	0.9999763851
NOTCH2NL	-1.3155492632	0.8262133195	0.9999763851
LOC105371242	-4.2864494036	0.4027150576	0.9999763851
NBPF12	-1.7851710282	0.1761059492	0.9999763851
PRKAB2	-2.2729676677	0.0319256079	0.9999763851
CHD1L	1.0718297596	0.6339408405	0.9999763851
BCL9	1.1246242259	0.7969567034	0.9999763851
GPR89B	-1.1153704227	0.7073520298	0.9999763851
NBPF11	1.1272587345	0.6884538008	0.9999763851
PPIAL4G	3.9290176215	0.1118265154	0.9999763851
NBPF14	-1.1641941032	0.3600061734	0.9999763851
LOC100996763	-1.1271073854	0.7416745505	0.9999763851
LOC100996517	42.193091832	0.05359176	0.9999763851
PDE4DIP	-1.3788695055	0.3876950863	0.9999763851
NBPF9	-1.2506571049	0.8039287911	0.9999763851
LOC100996716	1.2117822176	0.6110682655	0.9999763851

LOC653513	-4.3331566429	0.1395575407	0.9999763851
LOC100996717	-1.44239004	0.2668790172	0.9999763851
NBPF19	1.9447930831	0.1563583428	0.9999763851
PPIAL4C	0	0	0
FCGR1A	1.0527400853	0.857589463	0.9999763851
HIST2H2BF	-4.3424739515	0.0811973008	0.9999763851
HIST2H3D	-1.3161718537	0.8277563775	0.9999763851
HIST2H4A	1.813344484	0.5819964267	0.9999763851
HIST2H3C	-2.3220580825	0.4429616903	0.9999763851
HIST2H2AA3	1.0909116947	0.8271879995	0.9999763851
HIST2H2AA4	1.2747271793	0.5556263711	0.9999763851
HIST2H3A	-3.3292136073	0.2382175538	0.9999763851
HIST2H4B	-1.5918873484	0.6446118987	0.9999763851
HIST2H2BE	1.6366750224	0.4805828117	0.9999763851
HIST2H2AC	1.5954768462	0.4014275276	0.9999763851
HIST2H2AB	1.2874012553	0.8192055827	0.9999763851
BOLA1	1.4570229755	0.4933567456	0.9999763851
SF3B4	1.2617130229	0.2401744871	0.9999763851
MTMR11	-1.6467400147	0.4408765834	0.9999763851
OTUD7B	-2.4486332546	0.1552205728	0.9999763851
VPS45	-1.0198978496	0.9515542242	0.9999763851
PLEKHO1	-1.0619217124	0.7465447405	0.9999763851
ANP32E	1.0521507098	0.7054046205	0.9999763851
CA14	1.6062903958	0.6466460568	0.9999763851
APH1A	-1.1977140902	0.2076445163	0.9999763851
C1orf54	-17.4061108707	0.0751382059	0.9999763851
CIART	-7.5647903763	0.2176246281	0.9999763851
MRPS21	1.0460951848	0.7857895266	0.9999763851
PRPF3	-1.0147482064	0.938560045	0.9999763851
RPRD2	-1.3757368245	0.0530965646	0.9999763851
TARS2	-1.3060327519	0.4337745949	0.9999763851
ECM1	-1.4251442267	0.5941988919	0.9999763851
ADAMTSL4	-7.5622178268	0.2177145568	0.9999763851
LOC107983947	0	0	0
MCL1	-1.1177766841	0.3855973826	0.9999763851
ENSA	1.0866647576	0.587518744	0.9999763851
GOLPH3L	1.2236057104	0.3715179841	0.9999763851
CTSS	-1.3930122343	0.1940977269	0.9999763851
CTSK	1.5508263305	0.5741530212	0.9999763851
ARNT	-1.8420317982	0.008088	0.9999763851
SETDB1	-1.4704594162	0.0580222203	0.9999763851
CERS2	-1.4511143597	0.1470051795	0.9999763851
ANXA9	1.624741281	0.5901758802	0.9999763851
FAM63A	-1.2032443692	0.74471745	0.9999763851
PRUNE	-1.24957653	0.2427886769	0.9999763851
BNIPL	-2.3220580825	0.4412254448	0.9999763851
C1orf56	-1.0011870397	0.9936264848	0.9999763851
CDC42SE1	-1.3730016089	0.2290611368	0.9999763851
MLLT11	3.2356557963	0.5547505991	0.9999763851

GABPB2	-1.0131874908	0.9474173243	0.9999763851
SEMA6C	-2.5519849925	0.366972053	0.9999763851
LYSMD1	-2.388225611	0.2016075703	0.9999763851
TNFAIP8L2	-1.2303481196	0.7580222184	0.9999763851
TNFAIP8L2-SCNM1	0	0	0
SCNM1	1.151571266	0.4685611914	0.9999763851
TMOD4	9.9619682616	0.2312959428	0.9999763851
VPS72	1.0138573928	0.9294384901	0.9999763851
PIP5K1A	-1.3872536611	0.1473567212	0.9999763851
PSMD4	1.0324928049	0.8358314618	0.9999763851
ZNF687	-1.4504358563	0.1594826336	0.9999763851
PI4KB	1.2897562968	0.2204211482	0.9999763851
RFX5	1.3230819179	0.2680304145	0.9999763851
SELENBP1	0	0	0
PSMB4	1.2120455538	0.1878660556	0.9999763851
POGZ	-1.2269093491	0.282728933	0.9999763851
CGN	-5.3414526768	0.0966968323	0.9999763851
TUFT1	-1.1088558938	0.8900763784	0.9999763851
SNX27	1.2812339433	0.2137679447	0.9999763851
CELF3	0	0	0
MRPL9	-1.2148020154	0.2439102425	0.9999763851
OAZ3	3.2353357581	0.554787377	0.9999763851
TDRKH	1.3707143557	0.4404910355	0.9999763851
RORC	0	0	0
THEM5	0	0	0
THEM4	1.1048224175	0.5912607219	0.9999763851
S100A10	1.0349587196	0.8665300516	0.9999763851
S100A11	1.0782019856	0.6133634613	0.9999763851
TCHH	3.7682243222	0.2257640701	0.9999763851
FLG	0	0	0
PGLYRP4	-2.5611869689	0.2935474297	0.9999763851
S100A9	1.1849574673	0.2771293344	0.9999763851
S100A12	-4.2849908816	0.4028453076	0.9999763851
S100A8	-1.0498598655	0.8034566104	0.9999763851
S100A6	-1.1009403755	0.4437381118	0.9999763851
S100A5	0	0	0
S100A4	-1.0587914147	0.6480528822	0.9999763851
S100A3	0	0	0
S100A2	1.0956757311	0.8956196498	0.9999763851
S100A16	5.4781708352	0.3745527127	0.9999763851
S100A13	1.0987760401	0.8356121536	0.9999763851
S100A1	4.6902702261	0.1352949237	0.9999763851
CHTOP	1.360206339	0.0327114551	0.9999763851
SNAPIN	-1.1655260263	0.3318378667	0.9999763851
ILF2	1.0443466865	0.7883855495	0.9999763851
INTS3	-1.7727038833	0.275983452	0.9999763851
SLC27A3	1.059382013	0.8733050322	0.9999763851
GATAD2B	-1.0441594867	0.7905859451	0.9999763851
DENND4B	-1.081161521	0.72225433	0.9999763851

CRTC2	2.0582329403	0.1817524752	0.9999763851
SLC39A1	-1.1296923632	0.5507989956	0.9999763851
CREB3L4	-1.299823342	0.5266325284	0.9999763851
JTB	-1.0307804305	0.8084407147	0.9999763851
RAB13	-1.3410807493	0.1992601122	0.9999763851
RPS27	1.2816087907	0.0579259837	0.9999763851
NUP210L	-2.1357363271	0.00456	0.9999763851
TPM3	1.0410958636	0.7381717874	0.9999763851
C1orf189	0	0	0
C1orf43	-1.0180047575	0.8983178138	0.9999763851
UBAP2L	-1.0001601104	0.9992219996	0.9999763851
HAX1	1.1334518983	0.410724924	0.9999763851
ATP8B2	-1.3979355019	0.2824956428	0.9999763851
IL6R	-1.150599819	0.3026044363	0.9999763851
SHE	2.3389220976	0.0332783348	0.9999763851
TDRD10	-4.2864494036	0.4027150576	0.9999763851
UBE2Q1	1.2723874063	0.1608155356	0.9999763851
CHRNB2	0	0	0
ADAR	1.1469920068	0.3130373548	0.9999763851
KCNN3	-1.3158816768	0.8203229797	0.9999763851
PMVK	-1.1767724419	0.2540503034	0.9999763851
PBXIP1	-1.8284886844	0.0134326638	0.9999763851
PYGO2	1.2089373577	0.4224194502	0.9999763851
LOC101928120	1.2033560084	0.4308440614	0.9999763851
SHC1	1.4565287385	0.4871473964	0.9999763851
CKS1B	-1.1445129826	0.3343695485	0.9999763851
FLAD1	1.1811669064	0.3552341173	0.9999763851
ZBTB7B	-1.0803368274	0.7999695782	0.9999763851
DCST2	-4.287394647	0.4026306792	0.9999763851
DCST1	1.9253836052	0.2060004179	0.9999763851
ADAM15	1.1348667598	0.4333609897	0.9999763851
EFNA4	-1.3667385249	0.4315127815	0.9999763851
EFNA3	1.2454645608	0.6986114833	0.9999763851
EFNA1	1.2808976007	0.7495758492	0.9999763851
SLC50A1	1.0921361189	0.6617028498	0.9999763851
DPM3	-1.0946344766	0.577172513	0.9999763851
KRTCAP2	-1.0063815542	0.9639357508	0.9999763851
TRIM46	2.8674942058	0.3052941215	0.9999763851
MUC1	-1.1593995914	0.7091879533	0.9999763851
THBS3	-3.1528628322	0.2026298137	0.9999763851
MTX1	-1.1399972765	0.4987001827	0.9999763851
GBA	1.097511022	0.7704556429	0.9999763851
FAM189B	1.2719719588	0.4841774416	0.9999763851
SCAMP3	1.0350486681	0.835797729	0.9999763851
CLK2	1.2166669437	0.4445591562	0.9999763851
HCN3	2.2215959932	0.3502437171	0.9999763851
FDPS	-1.0051346616	0.9778254532	0.9999763851
RUSC1-AS1	1.3934050331	0.0665473821	0.9999763851
RUSC1	1.1553988663	0.6157186158	0.9999763851

ASH1L	-1.1759361663	0.3699420076	0.9999763851
MSTO1	1.0515966867	0.7872245284	0.9999763851
YY1AP1	1.1987507809	0.2208076114	0.9999763851
DAP3	-1.070187162	0.6541279283	0.9999763851
GON4L	-1.2174335513	0.4677682675	0.9999763851
SYT11	-1.3715126673	0.7510413575	0.9999763851
RIT1	-1.0061298774	0.9795546526	0.9999763851
KIAA0907	-1.0617987621	0.7752451873	0.9999763851
ARHGEF2	1.1198784675	0.5569123317	0.9999763851
SSR2	1.1258418768	0.3608695518	0.9999763851
UBQLN4	1.1093269548	0.7030113309	0.9999763851
LAMTOR2	-1.1606592508	0.3508997143	0.9999763851
MEX3A	-1.0545686913	0.9163560124	0.9999763851
LMNA	1.0306480629	0.8466898109	0.9999763851
SEMA4A	-1.640520605	0.1665268943	0.9999763851
SLC25A44	1.0632475349	0.7757036579	0.9999763851
PMF1	-1.1898802798	0.3906576286	0.9999763851
BGLAP	1.0276562285	0.9749720028	0.9999763851
PAQR6	1.2259289984	0.5874846482	0.9999763851
SMG5	1.1510937597	0.520984932	0.9999763851
TMEM79	1.6659695968	0.3787706685	0.9999763851
GLMP	-1.3181851579	0.2203204729	0.9999763851
CCT3	1.3798226018	0.0112582558	0.9999763851
TSACC	1.0281638254	0.9765136019	0.9999763851
C1orf61	-1.6468494889	0.4444559941	0.9999763851
MEF2D	-1.4108285042	0.0543069272	0.9999763851
IQGAP3	1.047488889	0.8870817548	0.9999763851
NAXE	1.1620797367	0.3044645567	0.9999763851
GPATCH4	-2.3023833833	0.1951728721	0.9999763851
HAPLN2	-2.3217011239	0.4357748964	0.9999763851
BCAN	-2.4281122475	0.277146081	0.9999763851
CRABP2	-2.2372730517	0.3099480393	0.9999763851
ISG20L2	-1.0927570266	0.6161501789	0.9999763851
RRNAD1	1.1457251594	0.6857144331	0.9999763851
MRPL24	1.0384978731	0.8207499167	0.9999763851
HDGF	1.0571650153	0.6440761861	0.9999763851
PRCC	-1.8059244363	0.0294598564	0.9999763851
SH2D2A	1.0499328592	0.8871855803	0.9999763851
NTRK1	0	0	0
PEAR1	7.7488609396	0.3055345795	0.9999763851
LRRC71	0	0	0
ARHGEF11	1.6241172672	0.5986866126	0.9999763851
ETV3	-1.0177490021	0.9686309973	0.9999763851
FCRL2	0	0	0
FCRL1	0	0	0
CD1D	1.5499082437	0.5571928434	0.9999763851
CD1A	12.2062282412	0.1875343457	0.9999763851
CD1B	3.2356557963	0.5547505991	0.9999763851
CD1C	-2.3401094535	0.2613324175	0.9999763851

SPTA1	3.2356557963	0.5547505991	0.9999763851
MNDA	1.0011503061	0.9943464011	0.9999763851
PYHIN1	1.5573674027	0.4354502129	0.9999763851
IFI16	-1.0780941428	0.6423337524	0.9999763851
AIM2	-3.1438286037	0.1493990602	0.9999763851
DUSP23	1.2206979703	0.3209792572	0.9999763851
FCRL6	0	0	0
SLAMF8	-1.946482244	0.1341665652	0.9999763851
C1orf204	5.4770877341	0.3746135179	0.9999763851
VSIG8	3.2356557963	0.5547505991	0.9999763851
TAGLN2	1.0591641584	0.6981495535	0.9999763851
SLAMF9	0	0	0
PIGM	1.0142445777	0.94067109	0.9999763851
IGSF8	1.2115524546	0.6553109416	0.9999763851
CASQ1	3.2353357581	0.554787377	0.9999763851
PEA15	1.2817772252	0.2703759705	0.9999763851
DCAF8	-1.0928480136	0.5933599548	0.9999763851
PEX19	1.232474398	0.3653255314	0.9999763851
COPA	-1.0516051792	0.7582572358	0.9999763851
NCSTN	1.0572259099	0.8328655425	0.9999763851
NHLH1	-1.5918873484	0.6448733186	0.9999763851
CD84	0	0	0
SLAMF1	3.2353357581	0.554787377	0.9999763851
CD48	1.1665406493	0.6052866289	0.9999763851
SLAMF7	-3.7496649203	0.1128297175	0.9999763851
LY9	1.3261442362	0.7480478279	0.9999763851
CD244	-1.2733695535	0.3533546471	0.9999763851
ITLN1	-4.2849908816	0.4028453076	0.9999763851
F11R	1.0177856078	0.9165515984	0.9999763851
TSTD1	-4.2849908816	0.4028453076	0.9999763851
USF1	-1.3158816768	0.8203229797	0.9999763851
ARHGAP30	-1.2819455835	0.1303717753	0.9999763851
NECTIN4	0	0	0
PFDN2	-1.2105738492	0.196330021	0.9999763851
NIT1	-1.0667169133	0.7941114271	0.9999763851
DEDD	0	0	0
UFC1	1.0070992749	0.9643749311	0.9999763851
USP21	-1.2460931847	0.7218799605	0.9999763851
PPOX	-1.3047889611	0.2980399979	0.9999763851
B4GALT3	-1.0561811682	0.8875509022	0.9999763851
ADAMTS4	0	0	0
NDUFS2	-1.4961899163	0.002888	0.9999763851
FCER1G	-1.8635681125	0.002735	0.9999763851
TOMM40L	2.7543183162	0.001166	0.8594669785
NR1I3	-4.2939733029	0.005433	0.9999763851
PCP4L1	1.325955166	0.7487022553	0.9999763851
MPZ	1.433664499	0.1228399261	0.9999763851
SDHC	-1.0125083909	0.9257897888	0.9999763851
CFAP126	-4.2849908816	0.4028453076	0.9999763851

FCGR2A	-1.1998575617	0.4876434681	0.9999763851
HSPA6	-2.4817290922	0.1994228953	0.9999763851
FCGR3A	-4.0107040788	0.1999292346	0.9999763851
FCGR3B	3.2356557963	0.5547505991	0.9999763851
FCGR2B	-2.8046993736	0.044452776	0.9999763851
FCRLA	-5.7462213657	0.0147566938	0.9999763851
FCRLB	-2.06934004	0.1151622813	0.9999763851
DUSP12	1.0872914566	0.6643265468	0.9999763851
ATF6	-1.5448398882	0.1334097308	0.9999763851
OLFML2B	-1.3154339304	0.8205576571	0.9999763851
NOS1AP	1.0002576586	0.9997940268	0.9999763851
C1orf226	-7.5622178268	0.2177145568	0.9999763851
UHMK1	1.0363787593	0.8783556344	0.9999763851
UAP1	-1.3128199543	0.376621597	0.9999763851
RGS5	-1.0196127275	0.9752972769	0.9999763851
NUF2	-1.0104977114	0.9533214524	0.9999763851
RXRG	0	0	0
MGST3	-1.449187843	0.005327	0.9999763851
ALDH9A1	1.3886283382	0.0249627168	0.9999763851
TMCO1	1.2002852189	0.1950250832	0.9999763851
UCK2	-1.1715683759	0.3128621271	0.9999763851
FAM78B	-4.2864494036	0.4027150576	0.9999763851
POGK	1.1026165978	0.6048159013	0.9999763851
TADA1	1.1316554702	0.5781979615	0.9999763851
POU2F1	-1.3770720542	0.0902995786	0.9999763851
CD247	-1.406657056	0.6694280141	0.9999763851
CREG1	-1.0427441967	0.8341435116	0.9999763851
RCSD1	1.1007389606	0.5145787712	0.9999763851
MPZL1	-1.0634584883	0.7392593604	0.9999763851
ADCY10	-3.1534488363	0.1717799846	0.9999763851
MPC2	1.0512709282	0.7314038067	0.9999763851
DCAF6	-1.219777617	0.2267813009	0.9999763851
GPR161	-4.2849908816	0.4028453076	0.9999763851
TIPRL	-1.1209102894	0.4175596054	0.9999763851
SFT2D2	2.0141755765	0.1774053269	0.9999763851
TBX19	-3.9223203142	0.0711702913	0.9999763851
ATP1B1	1.2499200512	0.6597977783	0.9999763851
NME7	-1.4810140327	0.3352740582	0.9999763851
BLZF1	1.032823483	0.9442529827	0.9999763851
CCDC181	0	0	0
SLC19A2	1.0889112855	0.9196655925	0.9999763851
SELL	2.6282277398	0.1112294005	0.9999763851
METTL18	-1.4285462622	0.5393339392	0.9999763851
C1orf112	1.298994176	0.1781216973	0.9999763851
SCYL3	-2.2601303969	0.0751994704	0.9999763851
KIFAP3	1.348590037	0.4348126546	0.9999763851
GORAB	-1.2380211518	0.6426875883	0.9999763851
PRRX1	0	0	0
FMO2	0	0	0

FMO1	-1.1235515614	0.8524922458	0.9999763851
FMO4	-1.1815471621	0.2524743197	0.9999763851
PRRC2C	1.0879156039	0.5129525653	0.9999763851
MYOC	3.2353357581	0.554787377	0.9999763851
VAMP4	-1.5458910777	0.2129737492	0.9999763851
METTL13	1.3189756584	0.1672034303	0.9999763851
DNM3	-1.0535819566	0.9017469924	0.9999763851
C1orf105	1.3254881051	0.7679649235	0.9999763851
PIGC	-1.5811033866	0.0388202535	0.9999763851
SUCO	1.0448720241	0.8193921626	0.9999763851
FASLG	3.2353357581	0.554787377	0.9999763851
TNFSF4	1.696196843	0.2721274453	0.9999763851
PRDX6	1.0749207081	0.6117682336	0.9999763851
LOC730159	0	0	0
ANKRD45	7.7488609396	0.3056154965	0.9999763851
KLHL20	-1.1216698915	0.6558540889	0.9999763851
CENPL	-1.1689979606	0.5412170814	0.9999763851
DARS2	1.3459980149	0.1412117905	0.9999763851
ZBTB37	1.0281638254	0.9765424521	0.9999763851
RC3H1	-1.2068734287	0.4474521286	0.9999763851
RABGAP1L	-1.411561274	0.1277954236	0.9999763851
CACYBP	1.0778622106	0.5398075088	0.9999763851
MRPS14	-1.0961453291	0.5570606017	0.9999763851
TNN	-1.5049537523	0.0711845732	0.9999763851
TNR	4.9725786265	0.1100739501	0.9999763851
RFWD2	-1.0847246266	0.7091649829	0.9999763851
PAPPA2	0	0	0
SEC16B	0	0	0
RASAL2	-1.3290629654	0.5483041713	0.9999763851
LINC00083	0	0	0
RALGPS2	-1.5504730795	0.2468906418	0.9999763851
FAM20B	1.0859065772	0.6795751696	0.9999763851
TOR3A	-1.2128092255	0.2217872839	0.9999763851
ABL2	-1.4286184811	0.0886931974	0.9999763851
SOAT1	1.0701273451	0.7836750489	0.9999763851
AXDND1	5.4790129495	0.3751913962	0.9999763851
FAM163A	0	0	0
TOR1AIP2	1.2808915888	0.1280935883	0.9999763851
TOR1AIP1	1.0819500062	0.6996881387	0.9999763851
CEP350	-1.1621534905	0.4027792184	0.9999763851
QSOX1	-1.1759727425	0.2117954646	0.9999763851
LHX4	-2.0108457684	0.4190952835	0.9999763851
ACBD6	1.1477221495	0.4222962398	0.9999763851
XPR1	1.5655091706	0.0757111	0.9999763851
KIAA1614	-1.598713419	0.5554914697	0.9999763851
STX6	-1.4493760166	0.0545227574	0.9999763851
MR1	1.1839036608	0.5809118468	0.9999763851
IER5	-1.1867712704	0.2349762053	0.9999763851
CACNA1E	0	0	0

ZNF648	3.2353357581	0.554787377	0.9999763851
GLUL	-1.1103304053	0.5250161469	0.9999763851
RGSL1	0	0	0
RNASEL	-1.000061298	0.999862539	0.9999763851
RGS16	-2.6933726902	1.80E-05	0.0402963288
NPL	1.9244931186	0.0831740148	0.9999763851
DHX9	1.3773725349	0.110261246	0.9999763851
SHCBP1L	1.3417088146	0.6972953714	0.9999763851
LAMC1	-1.5078702112	0.0613848314	0.9999763851
LAMC2	3.2353357581	0.554787377	0.9999763851
NMNAT2	15.5019390839	0.162998722	0.9999763851
SMG7	-1.0623036171	0.6570357744	0.9999763851
NCF2	-2.1541375242	0.018365499	0.9999763851
ARPC5	-1.0265235593	0.8404490161	0.9999763851
RGL1	4.1545939766	0.1790719693	0.9999763851
COLGALT2	1.4381534126	0.433731487	0.9999763851
TSEN15	-1.2188813587	0.2995574386	0.9999763851
C1orf21	-1.7456384649	0.0007429	0.6510516988
EDEM3	1.1575905138	0.3971292374	0.9999763851
FAM129A	1.1675930346	0.3403237227	0.9999763851
RNF2	1.4201559333	0.1265199169	0.9999763851
TRMT1L	-1.2581748746	0.1239985224	0.9999763851
SWT1	1.0788104453	0.8830712373	0.9999763851
IVNS1ABP	1.1180427667	0.4399408406	0.9999763851
HMCN1	0	0	0
PRG4	-1.1445217789	0.6113903606	0.9999763851
TPR	-1.0272553864	0.872898569	0.9999763851
C1orf27	1.6366304021	0.0990731554	0.9999763851
PTGS2	-2.9315971648	0.0345590853	0.9999763851
PLA2G4A	-1.4138717488	0.1831602796	0.9999763851
RGS18	1.2625516817	0.2781151034	0.9999763851
RGS1	-1.5972028858	0.2288061555	0.9999763851
RGS13	0	0	0
RGS2	-1.9501691009	0.3588530544	0.9999763851
UCHL5	1.1626307567	0.3017458324	0.9999763851
TROVE2	1.0575460082	0.735311562	0.9999763851
GLRX2	1.2778100101	0.2450076348	0.9999763851
CDC73	1.2602792118	0.2668129261	0.9999763851
CFHR4	0	0	0
ASPM	-1.0838321223	0.7015612959	0.9999763851
ZBTB41	-1.414852501	0.6315218082	0.9999763851
CRB1	0	0	0
DENND1B	1.1291582731	0.662256638	0.9999763851
C1orf53	2.631623025	0.1149645872	0.9999763851
NEK7	1.1004396935	0.7364959663	0.9999763851
PTPRC	-1.3626087866	0.0458200668	0.9999763851
ZNF281	-1.1272112548	0.4769125955	0.9999763851
KIF14	1.2534434094	0.1568403366	0.9999763851
DDX59	1.2490800189	0.7079294704	0.9999763851

CAMSAP2	-2.5419532854	0.054070927	0.9999763851
C1orf106	1.168655229	0.4892841527	0.9999763851
KIF21B	1.4156601674	0.0765734249	0.9999763851
CACNA1S	-4.2864494036	0.4027150576	0.9999763851
ASCL5	0	0	0
TMEM9	-1.168852567	0.3973565477	0.9999763851
LAD1	0	0	0
PHLDA3	1.2869488883	0.8189667132	0.9999763851
CSRP1	1.2798555106	0.2331649683	0.9999763851
LOC107985246	2.3401939765	0.4037104303	0.9999763851
NAV1	1.0955803262	0.6516967525	0.9999763851
IPO9	1.2396492614	0.1955247659	0.9999763851
SHISA4	-4.3365422435	0.1769061646	0.9999763851
LMOD1	-3.3280817062	0.2437290808	0.9999763851
TIMM17A	1.1058392345	0.4994751163	0.9999763851
RNPEP	1.0797245704	0.6534081615	0.9999763851
ELF3	3.2356557963	0.5547505991	0.9999763851
GPR37L1	0	0	0
ARL8A	-1.2431881205	0.1153912965	0.9999763851
PTPN7	-1.0502172995	0.7607465022	0.9999763851
LGR6	0	0	0
UBE2T	1.0967698139	0.5804527335	0.9999763851
PPP1R12B	1.1488737757	0.2706204971	0.9999763851
SYT2	1.0276562285	0.9749568208	0.9999763851
KDM5B	1.0426538087	0.7704619553	0.9999763851
RABIF	1.079866816	0.7200055693	0.9999763851
KLHL12	-1.0920485491	0.6398074437	0.9999763851
ADIPOR1	-1.0452420823	0.8556728463	0.9999763851
CYB5R1	-1.0359668383	0.931906619	0.9999763851
TMEM183A	1.1142324753	0.5983817408	0.9999763851
PPFIA4	-1.1483163584	0.8581191922	0.9999763851
MYBPH	2.8663267584	0.3071313305	0.9999763851
CHI3L1	-1.1902618325	0.2920722028	0.9999763851
CHIT1	-10.7999546676	0.0001991	0.2613243706
BTG2	-1.4483197087	0.2851760483	0.9999763851
ATP2B4	1.2449335534	0.1832625618	0.9999763851
LAX1	2.2088270228	0.002833	0.9999763851
ZC3H11A	1.0081972115	0.9608644287	0.9999763851
ZBED6	-1.256908463	0.7027795348	0.9999763851
SNRPE	1.1537080499	0.2768452146	0.9999763851
SOX13	-4.9419266265	0.0417233463	0.9999763851
ETNK2	0	0	0
REN	3.2361532718	0.5546934395	0.9999763851
PLEKHA6	1.624741281	0.5898390997	0.9999763851
PPP1R15B	-1.0839517004	0.6690937	0.9999763851
PIK3C2B	1.6764913239	0.0078	0.9999763851
MDM4	-1.0010211801	0.9958483063	0.9999763851
LRRN2	0	0	0
NFASC	3.2353357581	0.554787377	0.9999763851

TMEM81	-1.9665380586	0.4647627012	0.9999763851
RBBP5	1.1516433606	0.4775540633	0.9999763851
DSTYK	-1.2350002556	0.2638177343	0.9999763851
TMCC2	-1.4448633361	0.31005423	0.9999763851
KLHDC8A	-1.6419444001	0.001779	0.9999763851
LEMD1	0	0	0
CDK18	-1.7346410473	0.4883019977	0.9999763851
MFSD4A	1.1334347738	0.8813011476	0.9999763851
ELK4	-1.1313222959	0.6971522345	0.9999763851
SLC45A3	-1.2014834037	0.5249356123	0.9999763851
NUCKS1	1.0288183624	0.8124869535	0.9999763851
RAB29	1.0553415902	0.7752434901	0.9999763851
SLC41A1	1.1427311309	0.5814567219	0.9999763851
PM20D1	4.3099016468	0.231928679	0.9999763851
SLC26A9	1.0614829508	0.7341409358	0.9999763851
RAB7B	-1.5319719579	0.147279759	0.9999763851
C1orf186	-2.0614985279	0.2954510093	0.9999763851
FAM72A	-1.4595440907	0.6537626087	0.9999763851
SRGAP2	-1.0409909061	0.8175823864	0.9999763851
IKBKE	1.1962459603	0.3316463109	0.9999763851
RASSF5	-1.2064303847	0.6510917841	0.9999763851
EIF2D	-1.0163651427	0.9038504193	0.9999763851
DYRK3	-1.0577757904	0.8009561548	0.9999763851
MAPKAPK2	-1.0605113394	0.673189447	0.9999763851
IL10	-3.3284109188	0.2423554398	0.9999763851
IL19	2.3406309168	0.4060494981	0.9999763851
IL24	-1.2301452062	0.7476243508	0.9999763851
FCMR	1.3952230027	0.1116801928	0.9999763851
PIGR	-7.5647903763	0.2176246281	0.9999763851
YOD1	1.5501101194	0.1555917508	0.9999763851
CD55	-1.3081529372	0.2536038116	0.9999763851
CR1	-1.071695521	0.8570760425	0.9999763851
CD46	1.1512012964	0.4145784119	0.9999763851
CD34	0	0	0
LOC105372883	0	0	0
PLXNA2	-4.2849908816	0.4028453076	0.9999763851
LAMB3	-1.4391263345	0.6925824661	0.9999763851
HSD11B1	0	0	0
TRAF3IP3	-1.4304387769	0.48482272	0.9999763851
C1orf74	-1.0118087356	0.9460965592	0.9999763851
DIEXF	1.6305411606	0.1130488079	0.9999763851
SYT14	0	0	0
HHAT	-2.11701454	0.1724029672	0.9999763851
RCOR3	1.0463504491	0.837460115	0.9999763851
TRAF5	2.3883691069	0.0442017374	0.9999763851
SLC30A1	-1.7201161798	0.1914105944	0.9999763851
NEK2	-1.4315960423	0.1010601875	0.9999763851
LPGAT1	-1.3132800332	0.1155179091	0.9999763851
INTS7	1.618255245	0.0299742801	0.9999763851

DTL	1.024994917	0.8589823777	0.9999763851
PPP2R5A	1.056946182	0.6926406858	0.9999763851
TMEM206	1.2467177837	0.4633648067	0.9999763851
NENF	1.0730706436	0.637505344	0.9999763851
ATF3	-2.1966220146	0.0637902335	0.9999763851
BATF3	1.6979164668	0.1449685579	0.9999763851
NSL1	-1.0778368634	0.7259114948	0.9999763851
TATDN3	1.2217548231	0.4880921233	0.9999763851
FLVCR1	1.0114994304	0.9555864417	0.9999763851
VASH2	1.2872739558	0.8232731242	0.9999763851
ANGEL2	1.0213697067	0.9414633104	0.9999763851
RPS6KC1	1.1429294821	0.7794310758	0.9999763851
PROX1	0	0	0
SMYD2	-1.5649573985	0.0186269288	0.9999763851
PTPN14	1.3280268994	0.7331737794	0.9999763851
CENPF	1.1141466881	0.4020519802	0.9999763851
KCTD3	-1.4872814561	0.2268210138	0.9999763851
USH2A	-4.2864494036	0.4027150576	0.9999763851
ESRRG	3.2356557963	0.5547505991	0.9999763851
GPATCH2	-1.2258173831	0.4615179634	0.9999763851
SPATA17	3.2353357581	0.554787377	0.9999763851
RRP15	1.1767648256	0.4291484025	0.9999763851
TGFB2	-1.3713018672	0.7312784555	0.9999763851
LYPLAL1	1.0106720339	0.9645302671	0.9999763851
SLC30A10	0	0	0
EPRS	1.2169531166	0.1671111236	0.9999763851
BPNT1	1.1279574795	0.6134933238	0.9999763851
IARS2	-1.2337935557	0.2374234932	0.9999763851
RAB3GAP2	1.1982468803	0.2362284553	0.9999763851
MARK1	1.0275312208	0.9771806264	0.9999763851
MARC1	0	0	0
HLX	1.0925602859	0.6794112757	0.9999763851
DUSP10	-1.7332721092	0.4770374529	0.9999763851
HHIPL2	3.2353357581	0.554787377	0.9999763851
TAF1A	1.2432181721	0.5174690828	0.9999763851
MIA3	1.2906152316	0.2851097062	0.9999763851
AIDA	1.118693876	0.5014806366	0.9999763851
BROX	1.0200402595	0.9326223815	0.9999763851
FAM177B	5.4781708352	0.3745527127	0.9999763851
DISP1	-1.6891760355	0.1459623624	0.9999763851
CAPN2	1.5024948974	0.009317	0.9999763851
TP53BP2	-1.0408820879	0.8140846113	0.9999763851
LOC107985330	-1.0852711617	0.9285760414	0.9999763851
FBXO28	1.1548797206	0.4949216235	0.9999763851
DEGS1	1.6004476434	0.0388445229	0.9999763851
NVL	-1.1411080156	0.5927089375	0.9999763851
CNIH4	1.1753729208	0.2677476371	0.9999763851
WDR26	1.0915398418	0.6214804081	0.9999763851
CNIH3	-1.6429934142	0.0649179273	0.9999763851

DNAH14	-1.0264930697	0.9336981446	0.9999763851
LBR	1.0217827645	0.8619584671	0.9999763851
ENAH	1.1681948128	0.6628724957	0.9999763851
SRP9	1.3828532625	0.0908672978	0.9999763851
EPHX1	-1.1149875749	0.6500373451	0.9999763851
TMEM63A	-1.7141788209	0.071758446	0.9999763851
PYCR2	1.1743207251	0.3006305166	0.9999763851
SDE2	-1.0882525933	0.7137821784	0.9999763851
H3F3A	1.1165098578	0.3493484825	0.9999763851
ACBD3	-1.4223894497	0.0250536071	0.9999763851
LIN9	1.0710612774	0.7613163191	0.9999763851
PARP1	-1.3548878514	0.0452081041	0.9999763851
ITPKB	1.7186508518	0.0917581235	0.9999763851
PSEN2	1.1278326735	0.5789056635	0.9999763851
ADCK3	1.0584649725	0.6882782063	0.9999763851
CDC42BPA	-1.8158058555	0.4784760064	0.9999763851
ZNF678	0	0	0
SNAP47	1.3589559912	0.1813217562	0.9999763851
JMJD4	1.06300796	0.8123367972	0.9999763851
PRSS38	0	0	0
LOC107985355	0	0	0
WNT3A	0	0	0
ARF1	1.0929279738	0.4959848822	0.9999763851
C1orf35	1.1098589773	0.6114560887	0.9999763851
MRPL55	1.2718929491	0.1934389216	0.9999763851
GUK1	-1.0018643492	0.9900826968	0.9999763851
GJC2	3.2353357581	0.554787377	0.9999763851
IBA57	1.6947657343	0.2800415757	0.9999763851
OBSCN	1.3583735382	0.598178681	0.9999763851
LOC101927401	1.8145193361	0.579007064	0.9999763851
TRIM11	-1.058196852	0.7068371326	0.9999763851
TRIM17	-7.5647903763	0.2176246281	0.9999763851
HIST3H2A	0	0	0
RNF187	1.1158267107	0.4320846438	0.9999763851
BTNL10	-1.7347162507	0.4694691399	0.9999763851
RHOU	1.4234022233	0.0182922463	0.9999763851
LOC105373132	-3.3750646091	0.0271745729	0.9999763851
RAB4A	-1.1029128504	0.578361165	0.9999763851
SPHAR	1.0383925775	0.9074925649	0.9999763851
CCSAP	-1.2037347462	0.3477775283	0.9999763851
ACTA1	0	0	0
NUP133	1.2140038275	0.2090820467	0.9999763851
ABCB10	1.1992225944	0.3934831978	0.9999763851
TAF5L	1.6091859184	0.0820436865	0.9999763851
URB2	-1.2079157725	0.4573281787	0.9999763851
GALNT2	1.1570605444	0.3201250166	0.9999763851
PGBD5	-1.4414448586	0.3384827533	0.9999763851
COG2	1.2289912372	0.3887726781	0.9999763851
CAPN9	0	0	0

C1orf198	1.0573262353	0.7991512635	0.9999763851
TTC13	-2.9617156352	0.0160361979	0.9999763851
ARV1	1.414230029	0.1191092526	0.9999763851
FAM89A	1.3163089146	0.2760554353	0.9999763851
TRIM67	0	0	0
C1orf131	1.1569822016	0.4248689219	0.9999763851
GNPAT	1.2681250064	0.1928786541	0.9999763851
EXOC8	1.4595704504	0.1210403826	0.9999763851
SPRTN	1.2380260214	0.394735285	0.9999763851
EGLN1	-1.072455917	0.7666156583	0.9999763851
TSNAX	-1.1774992262	0.2699754598	0.9999763851
DISC1	-1.3306149506	0.2054468284	0.9999763851
SIPA1L2	1.5348527306	0.1388688108	0.9999763851
MAP10	1.2195107938	0.5649982765	0.9999763851
NTPCR	1.0592817209	0.8117393738	0.9999763851
PCNX2	1.3413311511	0.3632694601	0.9999763851
KIAA1804	-1.0829099927	0.862226659	0.9999763851
SLC35F3	2.5912551305	0.1773652625	0.9999763851
COA6	1.2994728289	0.1747938436	0.9999763851
TARBP1	-1.0236855949	0.9346355457	0.9999763851
IRF2BP2	-1.0979092043	0.4668852771	0.9999763851
TOMM20	-1.3073814928	0.0604089754	0.9999763851
RBM34	-1.3940363338	0.095976249	0.9999763851
ARID4B	-1.0221273458	0.9044635958	0.9999763851
GGPS1	1.4191432578	0.1669939237	0.9999763851
TBCE	-1.9671220856	0.0322880083	0.9999763851
B3GALNT2	-1.0814806951	0.5909540923	0.9999763851
GNG4	0	0	0
LYST	-1.0726706566	0.6693160007	0.9999763851
NID1	3.2356557963	0.5547505991	0.9999763851
GPR137B	-3.039585867	0.1308351339	0.9999763851
ERO1B	-2.300598904	0.0637079481	0.9999763851
EDARADD	1.3690402154	0.360362666	0.9999763851
LGALS8	-1.1066431886	0.5325778836	0.9999763851
HEATR1	-1.6266040895	0.1634986334	0.9999763851
ACTN2	0	0	0
MTR	1.0750887389	0.7516076956	0.9999763851
RYR2	1.577482471	0.4114816587	0.9999763851
CHRM3	1.0306913768	0.9663586235	0.9999763851
FMN2	-1.1305053662	0.8413969899	0.9999763851
RGS7	-1.2640353805	0.3011901278	0.9999763851
FH	-1.1660451657	0.4258885275	0.9999763851
KMO	1.695765772	0.4035122561	0.9999763851
OPN3	-1.266178856	0.252669258	0.9999763851
CHML	2.010067585	0.1553250476	0.9999763851
EXO1	1.0992796121	0.7054169536	0.9999763851
PLD5	0	0	0
CEP170	-1.3176287362	0.1607315415	0.9999763851
SDCCAG8	1.0600258414	0.7712726603	0.9999763851

AKT3	-1.222737104	0.5946752517	0.9999763851
ZBTB18	-1.0997549815	0.8019385282	0.9999763851
C1orf100	-1.3151016298	0.8209191532	0.9999763851
ADSS	-1.0787092763	0.6830259621	0.9999763851
C1orf101	1.3261589136	0.7392619172	0.9999763851
DESI2	1.0395005483	0.8599576227	0.9999763851
COX20	1.0330195	0.8794952146	0.9999763851
HNRNPU	1.0418939195	0.7263311312	0.9999763851
EFCAB2	-1.4258863983	0.1013598931	0.9999763851
KIF26B	-1.3124898922	0.6379235716	0.9999763851
SMYD3	-1.0379322718	0.791102053	0.9999763851
TFB2M	1.5576026172	0.0808448455	0.9999763851
CNST	1.0393581549	0.8869990845	0.9999763851
SCCPDH	-1.0031275293	0.9811424175	0.9999763851
AHCTF1	-1.2851628739	0.2145846429	0.9999763851
ZNF670	1.1912917425	0.4624319641	0.9999763851
ZNF669	-2.66053263	0.1881799047	0.9999763851
ZNF124	1.5543571304	0.0691458767	0.9999763851
ZNF496	-1.4157066625	0.6339385014	0.9999763851
LOC107985115	5.4781708352	0.3745527127	0.9999763851
NLRP3	-1.8121833565	0.0257981329	0.9999763851
GCSAML	0	0	0
TRIM58	1.3885242882	0.636859239	0.9999763851
OR2T8	0	0	0
OR2L13	0	0	0
SH3BP5L	1.1403516318	0.5034566375	0.9999763851
ZNF672	1.338613774	0.2727406526	0.9999763851
ZNF692	1.5269799671	0.4164224723	0.9999763851
PGBD2	-1.2571676518	0.7062574259	0.9999763851
SH3YL1	-1.6881725486	0.1959582488	0.9999763851
ACP1	1.0084972055	0.9546866715	0.9999763851
FAM150B	-19.2975026899	0.0747390423	0.9999763851
LOC101927262	1.432536289	0.7056197696	0.9999763851
TMEM18	1.0376183753	0.8441603075	0.9999763851
TPO	0	0	0
LOC102723730	0	0	0
PXDN	0	0	0
TSSC1	-1.189270716	0.3908551375	0.9999763851
TRAPPC12	1.0971314572	0.6775834857	0.9999763851
ADI1	1.0476070101	0.7454619571	0.9999763851
RNASEH1	1.4055190025	0.0443460047	0.9999763851
RPS7	1.0399635508	0.747004298	0.9999763851
COLEC11	1.6242600155	0.5622221146	0.9999763851
ALLC	0	0	0
CMPK2	-1.762719092	0.1891342668	0.9999763851
RSAD2	-5.3416241096	0.0827512079	0.9999763851
RNF144A	1.1237411547	0.5598252903	0.9999763851
ID2	1.0055770466	0.9672544026	0.9999763851
KIDINS220	-1.5874002138	0.0183525391	0.9999763851

MBOAT2	-4.2864494036	0.4027150576	0.9999763851
ITGB1BP1	1.4799748357	0.008451	0.9999763851
CPSF3	-1.0301695395	0.8472081564	0.9999763851
IAH1	1.2572725474	0.264329027	0.9999763851
ADAM17	-1.1245407305	0.4115700557	0.9999763851
YWHAQ	1.1596965101	0.2715541254	0.9999763851
TAF1B	1.0900979943	0.7426858041	0.9999763851
GRHL1	1.5500442495	0.5675474939	0.9999763851
KLF11	0	0	0
RRM2	1.1767022222	0.1921767341	0.9999763851
C2orf48	-1.0882443234	0.6920370257	0.9999763851
HPCAL1	-1.2012811567	0.1993491558	0.9999763851
ODC1	-1.0870956003	0.5514138426	0.9999763851
NOL10	-1.0538603756	0.7706399034	0.9999763851
ATP6V1C2	-2.3308983668	0.1742927847	0.9999763851
PDIA6	-1.098722879	0.4388309972	0.9999763851
C2orf50	0	0	0
PQLC3	-1.2498228987	0.3393359442	0.9999763851
ROCK2	-1.0501075553	0.7955645025	0.9999763851
E2F6	-1.081783891	0.654903945	0.9999763851
GREB1	1.1439155847	0.660903732	0.9999763851
LPIN1	1.2615875172	0.1462667779	0.9999763851
TRIB2	1.3527663374	0.0712842355	0.9999763851
NBAS	1.0262684822	0.9051213462	0.9999763851
DDX1	1.2602488997	0.1727901949	0.9999763851
FAM49A	-2.0639259657	0.0425306027	0.9999763851
RAD51AP2	3.2361532718	0.5546934395	0.9999763851
VSNL1	7.7488609396	0.3056963296	0.9999763851
SMC6	1.1060688479	0.6376889314	0.9999763851
GEN1	-1.1501165544	0.5895348389	0.9999763851
RDH14	-1.3489467621	0.2302414953	0.9999763851
NT5C1B-RDH14	0	0	0
TTC32	-1.438772044	0.4126556588	0.9999763851
LAPTM4A	-1.2838017296	0.0521825831	0.9999763851
PUM2	1.1236476402	0.4235149182	0.9999763851
LOC107985856	0	0	0
HS1BP3	1.3736731313	0.102802268	0.9999763851
LDAH	1.3850261424	0.0952750193	0.9999763851
LOC107985792	0	0	0
KLHL29	-1.3714374731	0.7337777464	0.9999763851
ATAD2B	-1.2034981149	0.4052927491	0.9999763851
UBXN2A	1.0101496188	0.9770806976	0.9999763851
MFSD2B	2.0155685261	0.4138816669	0.9999763851
WDCP	-1.0420774881	0.8526568339	0.9999763851
FKBP1B	2.2534767074	0.3735854402	0.9999763851
SF3B6	1.115079315	0.4636735404	0.9999763851
FAM228B	-1.6633350468	0.056527654	0.9999763851
TP53I3	0	0	0
FAM228A	-1.315303824	0.8206171563	0.9999763851

ITSN2	1.087711955	0.6295278702	0.9999763851
NCOA1	-1.4097336706	0.2129215729	0.9999763851
PTRHD1	-1.5176931502	0.037686896	0.9999763851
CENPO	1.2771421293	0.1157550345	0.9999763851
ADCY3	-1.4216025334	0.1746366735	0.9999763851
DNAJC27	1.1436674817	0.7911446084	0.9999763851
EFR3B	1.278970045	0.374590486	0.9999763851
DNMT3A	-1.0424540121	0.8888622534	0.9999763851
DTNB	1.0337751987	0.8942763355	0.9999763851
ASXL2	1.1406881434	0.4535134815	0.9999763851
KIF3C	1.1783065881	0.7005898276	0.9999763851
RAB10	1.279311693	0.0986402696	0.9999763851
GAREM2	1.3843154403	0.0444336695	0.9999763851
HADHA	1.0139543316	0.9437541629	0.9999763851
HADHB	1.0583173815	0.7297702511	0.9999763851
ADGRF3	-1.394553145	0.6912186114	0.9999763851
EPT1	1.1855795817	0.4539996747	0.9999763851
DRC1	-1.3154339304	0.8205576571	0.9999763851
CIB4	3.2353357581	0.554787377	0.9999763851
SLC35F6	-1.0333725574	0.8662299831	0.9999763851
CENPA	1.0060876646	0.9766024825	0.9999763851
DPYSL5	7.7488609396	0.305777082	0.9999763851
MAPRE3	7.7192231741	0.2780352706	0.9999763851
TMEM214	1.5562072344	0.0869868067	0.9999763851
AGBL5	1.2255088861	0.3258256701	0.9999763851
OST4	-1.0191982216	0.9111057126	0.9999763851
EMILIN1	1.325955166	0.748945782	0.9999763851
KHK	1.3067868184	0.5351088923	0.9999763851
ABHD1	16.6918753579	0.1320442202	0.9999763851
PREB	1.0802539431	0.6285313659	0.9999763851
SLC5A6	1.0640729196	0.7431571875	0.9999763851
ATRAID	-1.148780957	0.458098647	0.9999763851
CAD	1.1168840181	0.5394414575	0.9999763851
TRIM54	0	0	0
UCN	-1.1116250788	0.8542790178	0.9999763851
MPV17	1.3189486249	0.234051062	0.9999763851
GTF3C2	1.0597187345	0.8011712825	0.9999763851
EIF2B4	-1.0217395644	0.92804605	0.9999763851
SNX17	1.1064823609	0.586503082	0.9999763851
ZNF513	1.8136032532	0.5951921074	0.9999763851
PPM1G	1.0195653192	0.881329213	0.9999763851
NRBP1	-1.1087404898	0.560966312	0.9999763851
KRTCAP3	3.2356557963	0.5547505991	0.9999763851
IFT172	3.2356557963	0.5547505991	0.9999763851
C2orf16	1.2869488883	0.8189909969	0.9999763851
ZNF512	1.308229032	0.1543179029	0.9999763851
CCDC121	-10.8422096127	0.1439257484	0.9999763851
GPN1	-1.0931885671	0.5997138692	0.9999763851
SUPT7L	1.3027543022	0.2552113773	0.9999763851

SLC4A1AP	-1.1184967074	0.5306043995	0.9999763851
MRPL33	-1.2612029286	0.2585476449	0.9999763851
RBKS	1.03638979	0.9611342334	0.9999763851
BRE	-1.2138532658	0.3133597188	0.9999763851
FOSL2	1.0527006686	0.7833717672	0.9999763851
PLB1	-1.3708355312	0.7478119744	0.9999763851
PPP1CB	1.1678127183	0.3044319711	0.9999763851
SPDYA	1.2257229004	0.7466143562	0.9999763851
TRMT61B	1.2062310354	0.3750512419	0.9999763851
WDR43	1.0818430411	0.5810376127	0.9999763851
FAM179A	-1.6465635173	0.4724225492	0.9999763851
CLIP4	1.0505152305	0.9609042588	0.9999763851
ALK	0	0	0
YPEL5	-1.1309096567	0.5777044456	0.9999763851
LBH	2.5195872738	0.2430923148	0.9999763851
LCLAT1	1.5487639993	0.0576017967	0.9999763851
CAPN13	-4.287394647	0.4026306792	0.9999763851
GALNT14	1.3316041862	0.1598189001	0.9999763851
XDH	0	0	0
SRD5A2	0	0	0
MEMO1	1.0003142026	0.998711558	0.9999763851
DPY30	1.3330703024	0.1419905517	0.9999763851
SPAST	-1.0786321364	0.7288880156	0.9999763851
SLC30A6	1.3653786981	0.1562459732	0.9999763851
NLRC4	-1.8153473988	0.4783188166	0.9999763851
YIPF4	-1.0164732355	0.9431206982	0.9999763851
BIRC6	1.1083718614	0.5349948639	0.9999763851
TTC27	1.0527325707	0.8665672744	0.9999763851
LTBP1	1.3263859237	0.4507635577	0.9999763851
RASGRP3	1.8131652995	0.5813691012	0.9999763851
FAM98A	1.1869076495	0.3398289187	0.9999763851
CRIM1	-1.8157062896	0.4699720344	0.9999763851
FEZ2	-1.0344478115	0.8529754667	0.9999763851
VIT	-1.2927005519	0.5318125783	0.9999763851
STRN	-1.3286155222	0.0848989372	0.9999763851
HEATR5B	1.0716936802	0.8218470713	0.9999763851
GPATCH11	1.1540913993	0.6117349577	0.9999763851
EIF2AK2	-1.0599626053	0.7687682823	0.9999763851
CEBPZOS	1.1617193387	0.2973171983	0.9999763851
CEBPZ	-1.0407036996	0.854007645	0.9999763851
NDUFAF7	1.1018352216	0.7182562241	0.9999763851
PRKD3	-1.4356236793	0.4435231656	0.9999763851
CDC42EP3	1.0531272985	0.7211362939	0.9999763851
RMDN2	3.2353357581	0.554787377	0.9999763851
CYP1B1	-1.6824870848	0.4555260885	0.9999763851
ATL2	-1.1145686507	0.5730443113	0.9999763851
HNRNPLL	-4.2849908816	0.4028453076	0.9999763851
GALM	1.0272717886	0.9763520096	0.9999763851
SRSF7	1.1896566698	0.1722171844	0.9999763851

GEMIN6	-1.110001124	0.6825055789	0.9999763851
DHX57	-1.6912821635	0.1835810695	0.9999763851
MORN2	7.5959889452	0.005376	0.9999763851
SOS1	1.1436337525	0.4797284352	0.9999763851
CDKL4	0	0	0
MAP4K3	1.4088536585	0.2964281978	0.9999763851
TMEM178A	0	0	0
THUMPD2	1.1575074525	0.6078914394	0.9999763851
SLC8A1	-4.2864494036	0.4027150576	0.9999763851
EML4	-1.6764906029	0.0003754	0.439186721
COX7A2L	-1.1043007756	0.4546553042	0.9999763851
KCNG3	-2.3209115874	0.4622161811	0.9999763851
MTA3	-1.3883071659	0.1446450142	0.9999763851
OXER1	-1.9659101454	0.4308207028	0.9999763851
HAAO	1.0277462901	0.9757625931	0.9999763851
ZFP36L2	1.0336416409	0.7869635463	0.9999763851
THADA	1.0434821601	0.8306360382	0.9999763851
DYNC2LI1	1.8723955021	0.5237072844	0.9999763851
LRPPRC	1.1304115511	0.4405393036	0.9999763851
PPM1B	1.0651626276	0.680338186	0.9999763851
PREPL	1.2380366674	0.1933582431	0.9999763851
CAMKMT	-1.971292034	0.001782	0.9999763851
SRBD1	-1.5928709463	0.0799034067	0.9999763851
LOC102724965	5.4776292235	0.3745831165	0.9999763851
PRKCE	-1.0292749024	0.9083540727	0.9999763851
EPAS1	1.3439762932	0.443528971	0.9999763851
ATP6V1E2	1.3458151177	0.2684008386	0.9999763851
RHOQ	-2.9512094836	0.0180535395	0.9999763851
PIGF	1.4643959653	0.0863784067	0.9999763851
CRIPT	1.3775819513	0.1746016123	0.9999763851
SOCS5	-1.285616125	0.3294421071	0.9999763851
MCFD2	1.1000968395	0.5772400204	0.9999763851
TTC7A	-1.1659152008	0.4673408271	0.9999763851
C2orf61	-1.7354771749	0.4610815461	0.9999763851
CALM2	-1.0137813509	0.9189882775	0.9999763851
MSH2	-1.1357998396	0.5160744001	0.9999763851
KCNK12	1.8139019215	0.5818413221	0.9999763851
MSH6	1.0853944235	0.5724917196	0.9999763851
FBXO11	-1.0214232282	0.9106956777	0.9999763851
FOXN2	1.054745941	0.7991630709	0.9999763851
PPP1R21	1.131179799	0.681863498	0.9999763851
STON1	1.027644744	0.9774807983	0.9999763851
LHCGR	0	0	0
ASB3	-1.1067714597	0.7095820506	0.9999763851
GPR75-ASB3	1.342772839	0.4505185335	0.9999763851
CHAC2	1.321110215	0.5060978172	0.9999763851
ERLEC1	1.0636953367	0.7931369705	0.9999763851
PSME4	-1.0784706666	0.629606575	0.9999763851
ACYP2	-1.4483300894	0.2096416084	0.9999763851

SPTBN1	1.50164491	0.00754	0.9999763851
EML6	-1.6869571388	0.2831814493	0.9999763851
RTN4	1.1568404118	0.2513290308	0.9999763851
CLHC1	-1.2507021559	0.6167086561	0.9999763851
RPS27A	-1.235077644	0.0989980096	0.9999763851
MTIF2	-1.0047395273	0.9784041219	0.9999763851
CCDC88A	-1.281720043	0.093791524	0.9999763851
CFAP36	-1.5423634354	0.2139785669	0.9999763851
PPP4R3B	-1.0308706825	0.8310822389	0.9999763851
PNPT1	1.0173325989	0.92544304	0.9999763851
CCDC85A	0	0	0
VRK2	1.1257957434	0.653220876	0.9999763851
LOC107984043	1.1322528341	0.8825188119	0.9999763851
FANCL	1.144404358	0.5296900122	0.9999763851
BCL11A	-1.0721956879	0.6453183373	0.9999763851
PAPOLG	1.1811939394	0.5558612063	0.9999763851
REL	-1.5214001104	0.0415106114	0.9999763851
PUS10	-1.3015109125	0.1397802069	0.9999763851
PEX13	-1.5429143958	0.1967210093	0.9999763851
KIAA1841	1.2223646681	0.6866069743	0.9999763851
AHSA2	1.106877086	0.5277167343	0.9999763851
USP34	-1.1224957927	0.6703546068	0.9999763851
XPO1	1.3113273552	0.0351216098	0.9999763851
FAM161A	-1.1385539513	0.70245366	0.9999763851
CCT4	-1.0134428003	0.9120268644	0.9999763851
COMMD1	-1.268751871	0.1707268122	0.9999763851
B3GNT2	-1.4274545135	0.0809286414	0.9999763851
TMEM17	5.4776292235	0.3745831165	0.9999763851
EHBP1	-1.2337115932	0.3395307119	0.9999763851
OTX1	1.2263565036	0.6714721801	0.9999763851
WDPCP	-1.1568317067	0.6161114894	0.9999763851
MDH1	1.0624159527	0.6458166987	0.9999763851
UGP2	-1.1276651119	0.4460470039	0.9999763851
VPS54	1.0867395771	0.7522772309	0.9999763851
AFTPH	-1.2890781143	0.1979181162	0.9999763851
SERTAD2	-1.0226514326	0.8899303859	0.9999763851
SLC1A4	1.0607169423	0.6867381001	0.9999763851
CEP68	1.5248785194	0.274759176	0.9999763851
RAB1A	-1.0237616723	0.8555427974	0.9999763851
ACTR2	1.0987856392	0.4684845082	0.9999763851
SPRED2	-1.1294902392	0.647145588	0.9999763851
MEIS1	-1.0896004414	0.5987921936	0.9999763851
ETAA1	-1.7858228363	0.204721115	0.9999763851
C1D	1.0450761103	0.7875441959	0.9999763851
WDR92	1.1168545673	0.6569235823	0.9999763851
PNO1	1.1789633401	0.3582884595	0.9999763851
PPP3R1	-1.0151243697	0.9143244234	0.9999763851
CNRIP1	1.0268207743	0.9771394108	0.9999763851
PLEK	1.1867012473	0.2317231772	0.9999763851

FBXO48	-1.1228875834	0.8547702516	0.9999763851
APLF	-1.1550995243	0.7941305324	0.9999763851
ARHGAP25	1.3637935162	0.1189643085	0.9999763851
ANTXR1	3.2356557963	0.5547505991	0.9999763851
GFPT1	-1.3922766146	0.0878597507	0.9999763851
NFU1	-1.339815096	0.1550560841	0.9999763851
AAK1	1.1415752274	0.4217329832	0.9999763851
ANXA4	-1.0959420662	0.6324399369	0.9999763851
GMCL1	1.1076539834	0.5489596845	0.9999763851
SNRNP27	1.1870286079	0.403856724	0.9999763851
MXD1	-1.1976738389	0.4575237098	0.9999763851
ASPRV1	2.8683753118	0.2970239536	0.9999763851
PCBP1	1.0246180889	0.8535331483	0.9999763851
C2orf42	1.1647633948	0.7495638197	0.9999763851
TIA1	1.1763165918	0.4261515316	0.9999763851
PCYOX1	-1.3743857604	0.0267636196	0.9999763851
SNRPG	-1.0671039586	0.5880833828	0.9999763851
FAM136A	1.1257171013	0.501554028	0.9999763851
TGFA	1.6241887583	0.5813605053	0.9999763851
CLEC4F	0	0	0
VAX2	-1.315303824	0.8206171563	0.9999763851
ATP6V1B1	1.7955828734	0.2867099268	0.9999763851
TEX261	1.4270854773	0.0327520538	0.9999763851
NAGK	1.3952870539	0.2431710997	0.9999763851
MCEE	-1.3822405422	0.3766611981	0.9999763851
MPHOSPH10	-1.2249881759	0.3581147062	0.9999763851
ZNF638	-1.1775845548	0.3990530189	0.9999763851
DYSF	1.3635876576	0.1732447172	0.9999763851
EXOC6B	1.2867653957	0.8302200816	0.9999763851
EMX1	0	0	0
SFXN5	1.1679083758	0.5812122433	0.9999763851
RAB11FIP5	-1.2468168198	0.2477623091	0.9999763851
NOTO	0	0	0
SMYD5	-1.3620636421	0.6368377281	0.9999763851
PRADC1	1.0129300777	0.9656951153	0.9999763851
CCT7	-1.0350100581	0.7999910039	0.9999763851
FBXO41	1.5968276379	0.1651166133	0.9999763851
ALMS1	1.8157003876	0.1214582642	0.9999763851
TPRKB	1.0307255886	0.8819738451	0.9999763851
DUSP11	1.0036050462	0.9885961386	0.9999763851
STAMBP	-1.1061510203	0.7018111654	0.9999763851
ACTG2	0	0	0
DGUOK	-1.0167055867	0.9207469825	0.9999763851
TET3	-1.1325057796	0.5958103997	0.9999763851
BOLA3	1.2286026936	0.2597593711	0.9999763851
MOB1A	1.4238131745	0.0349642558	0.9999763851
MTHFD2	-1.1211063026	0.4420067432	0.9999763851
SLC4A5	-1.281853878	0.3481963662	0.9999763851
DCTN1	1.2889258401	0.2646920084	0.9999763851

C2orf81	-1.080916289	0.9218494159	0.9999763851
WDR54	-1.0382665908	0.8834081126	0.9999763851
RTKN	0	0	0
INO80B	1.2172563436	0.7055631978	0.9999763851
WBP1	-1.2907936775	0.3098200842	0.9999763851
MOGS	1.0005382248	0.9979450567	0.9999763851
MRPL53	1.4765991151	0.0229825898	0.9999763851
CCDC142	-2.9937056245	0.1615965695	0.9999763851
TTC31	1.6242712064	0.1898982261	0.9999763851
LBX2	3.2356557963	0.5547505991	0.9999763851
PCGF1	-2.0110792645	0.2231009582	0.9999763851
DQX1	0	0	0
AUP1	1.2152265407	0.2409094809	0.9999763851
HTRA2	1.3308698118	0.2479591739	0.9999763851
LOXL3	-2.3763485512	0.4067077572	0.9999763851
DOK1	-1.2433211656	0.1477399957	0.9999763851
M1AP	-1.4153036866	0.6387328106	0.9999763851
SEMA4F	0	0	0
HK2	-1.2476028608	0.2130856504	0.9999763851
POLE4	1.0018790596	0.9915204116	0.9999763851
TACR1	1.1892050826	0.8083244611	0.9999763851
MRPL19	-1.5786340388	0.0156258948	0.9999763851
GCFC2	-1.0639563986	0.8601929054	0.9999763851
LRRTM4	3.2361532718	0.5546934395	0.9999763851
CTNNA2	5.4770877341	0.3746135179	0.9999763851
SUCLG1	1.1029527461	0.4957967366	0.9999763851
DNAH6	0	0	0
TRABD2A	-4.2849908816	0.4028453076	0.9999763851
TMSB10	-1.1337601534	0.3770608755	0.9999763851
KCMF1	1.2377568161	0.285959735	0.9999763851
TCF7L1	-2.2383770602	0.3301996687	0.9999763851
TGOLN2	-1.2689429059	0.0854042447	0.9999763851
RETSAT	1.1130819889	0.563271458	0.9999763851
ELMOD3	1.128763695	0.7570667636	0.9999763851
CAPG	1.1359314632	0.4406142068	0.9999763851
SH2D6	0	0	0
MAT2A	1.0198804115	0.8823139831	0.9999763851
GGCX	-1.0186141143	0.9440969484	0.9999763851
VAMP8	-1.1317356375	0.3551138233	0.9999763851
VAMP5	-1.0157801405	0.9771486462	0.9999763851
RNF181	-1.0672732615	0.7511287799	0.9999763851
TMEM150A	-1.0356386932	0.9396850031	0.9999763851
USP39	1.2216049891	0.2704184296	0.9999763851
C2orf68	-1.4699587079	0.1567987807	0.9999763851
ATOH8	2.8682179169	0.3019372198	0.9999763851
ST3GAL5	1.3036963729	0.5592653088	0.9999763851
POLR1A	1.1181992119	0.7093815731	0.9999763851
PTCD3	1.0643579535	0.7044545482	0.9999763851
IMMT	1.3018007913	0.0732684497	0.9999763851

MRPL35	1.2305812311	0.2547223322	0.9999763851
KDM3A	-2.210488049	0.0007811	0.6568370458
CHMP3	-1.0513119976	0.7577621645	0.9999763851
RNF103-CHMP3	-1.9451744431	0.4383692894	0.9999763851
RNF103	-1.9877309057	0.1505008152	0.9999763851
RMND5A	-1.2177526697	0.318928986	0.9999763851
CD8A	0	0	0
CD8B	0	0	0
PLGLB1	7.7204093521	0.2881896654	0.9999763851
LOC730268	1.0631873881	0.8974318562	0.9999763851
RGPD2	-1.6534026074	0.2581321001	0.9999763851
KRCC1	-1.1737646797	0.5211092162	0.9999763851
EIF2AK3	-1.6666198591	0.0671035089	0.9999763851
RPIA	-1.2420000559	0.1976195011	0.9999763851
LOC107985911	-1.3214631849	0.5690647226	0.9999763851
TEKT4	7.7488609396	0.3058577563	0.9999763851
MRPS5	1.2426220328	0.1750842263	0.9999763851
ZNF514	-4.3327792956	0.1654925197	0.9999763851
ZNF2	-1.7344697458	0.454054257	0.9999763851
KCNIP3	0	0	0
FAHD2A	-1.0322542091	0.8987337781	0.9999763851
ANKRD36C	-1.1187044772	0.6616498032	0.9999763851
LOC107984110	0	0	0
GPAT2	1.0408311207	0.959749795	0.9999763851
ASTL	7.7599005912	0.2898575514	0.9999763851
DUSP2	1.1112905062	0.6216869168	0.9999763851
STARD7	-1.0730594048	0.5530232333	0.9999763851
TMEM127	1.4376745279	0.1318752406	0.9999763851
CIAO1	1.1019880542	0.5405328236	0.9999763851
SNRNP200	1.3307224643	0.0460934643	0.9999763851
NCAPH	1.1259600006	0.4641526807	0.9999763851
ARID5A	1.4461813237	0.0549773423	0.9999763851
KANSL3	-1.0997603701	0.60625043	0.9999763851
FER1L5	0	0	0
LMAN2L	1.5467827042	0.1189191726	0.9999763851
CNNM4	-1.8992688232	0.055017229	0.9999763851
CNNM3	-1.0433072276	0.8545354838	0.9999763851
ANKRD23	-1.0537130233	0.9182419571	0.9999763851
ANKRD39	1.1609452035	0.3835696884	0.9999763851
SEMA4C	1.2064374145	0.2169368647	0.9999763851
FAM178B	-3.3302773804	0.2299524791	0.9999763851
FAHD2B	-1.1015850729	0.7001300645	0.9999763851
ANKRD36	-1.6663798667	0.3061143595	0.9999763851
ANKRD36B	-1.2214116021	0.3761733749	0.9999763851
COX5B	1.0102030401	0.9347982024	0.9999763851
ACTR1B	1.0094778478	0.946718742	0.9999763851
ZAP70	1.813344484	0.5820336358	0.9999763851
TMEM131	1.0562564288	0.7335566298	0.9999763851
VWA3B	3.2353357581	0.554787377	0.9999763851

INPP4A	1.0192312552	0.9077453102	0.9999763851
COA5	-1.1046072051	0.6118765378	0.9999763851
UNC50	1.4380021782	0.2331515865	0.9999763851
MGAT4A	1.3297552504	0.1786145587	0.9999763851
KIAA1211L	-1.0755082442	0.7977674856	0.9999763851
TSGA10	1.5807548893	0.1818240065	0.9999763851
C2orf15	-1.6219931477	0.4436846426	0.9999763851
LIPT1	4.1100123697	0.1007263803	0.9999763851
MITD1	-1.3563111278	0.1552092989	0.9999763851
MRPL30	-1.1650567664	0.3717197832	0.9999763851
LYG2	1.0270472827	0.9776204387	0.9999763851
TXNDC9	1.3286524472	0.1935058476	0.9999763851
EIF5B	-1.0060512258	0.9608784015	0.9999763851
REV1	-1.1475183994	0.4777509799	0.9999763851
AFF3	-1.1483793728	0.8593637268	0.9999763851
LONRF2	3.2356557963	0.5547505991	0.9999763851
CHST10	1.7771737389	0.030601334	0.9999763851
PDCL3	-2.0596204881	0.0698354965	0.9999763851
NPAS2	0	0	0
RPL31	-1.035186772	0.769726902	0.9999763851
TBC1D8	-1.0041110489	0.9923308766	0.9999763851
CNOT11	-1.3388081756	0.0666895219	0.9999763851
RNF149	-1.3010473315	0.1946273161	0.9999763851
CREG2	1.1294335392	0.8449647978	0.9999763851
RFX8	-1.7171302182	0.1205232019	0.9999763851
MAP4K4	-1.0639167558	0.681890303	0.9999763851
IL1R2	0	0	0
IL1R1	1.3262899408	0.7594254998	0.9999763851
IL18R1	1.4203047017	0.2820066814	0.9999763851
IL18RAP	1.8612147697	0.2010349174	0.9999763851
SLC9A4	0	0	0
SLC9A2	-2.4440355816	0.4439069782	0.9999763851
MFSD9	-1.4813240664	0.1789293071	0.9999763851
TMEM182	1.2100357382	0.6008460605	0.9999763851
MRPS9	-1.0610741291	0.7292454941	0.9999763851
TGFBRAP1	1.0368474459	0.8230817278	0.9999763851
C2orf49	-1.1195121669	0.6561072983	0.9999763851
FHL2	-1.3714374731	0.7336360884	0.9999763851
NCK2	1.0219431309	0.9125964542	0.9999763851
C2orf40	-4.2864494036	0.4027150576	0.9999763851
UXS1	1.023158894	0.8808816156	0.9999763851
RGPD3	0	0	0
LOC107985931	0	0	0
SULT1C2	0	0	0
GCC2	-1.6142246857	0.0451607739	0.9999763851
LIMS1	-1.213314902	0.1828073918	0.9999763851
RANBP2	1.0214071416	0.8908219343	0.9999763851
CCDC138	1.053416032	0.8452734528	0.9999763851
EDAR	0	0	0

SH3RF3	1.0734923781	0.7994345489	0.9999763851
SEPT10	1.9834686116	0.1499094332	0.9999763851
SOWAHC	-1.0756553267	0.8347383746	0.9999763851
RGPD5	-14.1139127893	0.1018289369	0.9999763851
LIMS3	0	0	0
MALL	0	0	0
NPHP1	0	0	0
LIMS4	0	0	0
RGPD6	-1.045438992	0.9610160069	0.9999763851
BUB1	-1.2480313723	0.2305172057	0.9999763851
ACOXL	1.2512644887	0.521863797	0.9999763851
BCL2L11	1.2733425569	0.1685161822	0.9999763851
ANAPC1	1.1031592173	0.505694661	0.9999763851
MERTK	-1.0270070658	0.9057698921	0.9999763851
TMEM87B	-1.1405864677	0.5050447268	0.9999763851
FBLN7	-1.0971100786	0.8676021818	0.9999763851
ZC3H8	-1.2219743294	0.3066724101	0.9999763851
ZC3H6	-1.2718349738	0.5098842696	0.9999763851
RGPD8	-1.4249254444	0.6101019289	0.9999763851
TTL	-1.1345145882	0.4141526962	0.9999763851
POLR1B	1.2236250052	0.2745990447	0.9999763851
CHCHD5	2.587886895	0.0429304897	0.9999763851
SLC20A1	-1.0658843322	0.6320687838	0.9999763851
NT5DC4	-1.4461138135	0.1880286321	0.9999763851
CKAP2L	1.1274883687	0.5454925737	0.9999763851
IL1B	-7.9941504099	9.66E-12	1.04E-07
IL36G	5.4770877341	0.3746135179	0.9999763851
IL36A	0	0	0
IL36B	0	0	0
IL36RN	-7.5647903763	0.2176246281	0.9999763851
IL1RN	-6.062531604	3.23E-09	1.24E-05
PSD4	1.3316955254	0.1109782799	0.9999763851
PAX8	2.2197442392	0.3827732759	0.9999763851
CBWD2	-1.1501557732	0.6625856998	0.9999763851
FOXD4L1	-1.8252752883	0.4396499131	0.9999763851
RABL2A	-1.4112614935	0.2843419853	0.9999763851
SLC35F5	1.3593234382	0.167721857	0.9999763851
ACTR3	1.2241783698	0.1476125658	0.9999763851
DDX18	-1.1369028626	0.3894054282	0.9999763851
LOC107985939	1.1429815926	0.7565703052	0.9999763851
CCDC93	-1.3974597658	0.0786853912	0.9999763851
INSIG2	-1.6497102849	0.1883267835	0.9999763851
MARCO	-1.7494132661	0.1069887274	0.9999763851
STEAP3	1.3251796054	0.2872637719	0.9999763851
C2orf76	-2.0486799378	0.2151357512	0.9999763851
DBI	-1.190950259	0.1530585697	0.9999763851
SCTR	0	0	0
CFAP221	0	0	0
TMEM177	-1.5728911116	0.1233092937	0.9999763851

PTPN4	-1.2533885642	0.5262666626	0.9999763851
EPB41L5	-1.3158816768	0.8203229797	0.9999763851
TMEM185B	1.2800922138	0.1307734764	0.9999763851
RALB	-1.5204433696	0.004249	0.9999763851
INHBB	0	0	0
TFCP2L1	0	0	0
CLASP1	-1.1491943618	0.3402246172	0.9999763851
NIFK	1.2405136054	0.1587750247	0.9999763851
TSN	1.1175265115	0.4609286173	0.9999763851
CNTNAP5	5.4770877341	0.3746135179	0.9999763851
GYPC	-1.1108671394	0.4195399387	0.9999763851
BIN1	1.3638024281	0.2213738185	0.9999763851
ERCC3	1.0087546418	0.9611343498	0.9999763851
MAP3K2	1.1124101115	0.5801403512	0.9999763851
PROC	-1.3154339304	0.8205576571	0.9999763851
IWS1	1.1484238619	0.3896505443	0.9999763851
LIMS2	1.0542383865	0.8046896137	0.9999763851
GPR17	-1.3157515262	0.8227855444	0.9999763851
SFT2D3	-1.0886417021	0.7739156355	0.9999763851
WDR33	1.0659363653	0.6970446659	0.9999763851
POLR2D	-1.137911618	0.5116338305	0.9999763851
LOC107985803	1.6096975159	0.204839876	0.9999763851
AMMECR1L	1.4456391063	0.1457521704	0.9999763851
SAP130	1.0274861459	0.8800199645	0.9999763851
UGGT1	1.0489172896	0.7656211274	0.9999763851
HS6ST1	1.2803615879	0.1751980264	0.9999763851
RAB6C	-3.1528628322	0.202248388	0.9999763851
CCDC74B	-4.2849908816	0.4028453076	0.9999763851
SMPD4	-1.1526450932	0.2565084101	0.9999763851
MZT2B	-1.0128241802	0.9255979016	0.9999763851
TUBA3E	3.2361532718	0.5546934395	0.9999763851
CCDC115	-1.7253992275	0.001068	0.833154348
IMP4	1.092733122	0.6042809177	0.9999763851
PTPN18	1.4586197561	0.071081198	0.9999763851
POTEI	-1.2279078578	0.1645039133	0.9999763851
FAM168B	1.3143457626	0.0976177827	0.9999763851
PLEKHB2	1.3164726749	0.0656147391	0.9999763851
WTH3DI	-3.7978639386	0.2119439624	0.9999763851
MZT2A	1.1691105424	0.3199047164	0.9999763851
CCDC74A	-1.0550245006	0.9552374595	0.9999763851
C2orf27A	-1.113460946	0.6186113448	0.9999763851
MGAT5	-1.0268694645	0.8598628634	0.9999763851
TMEM163	-1.1122312474	0.4507727924	0.9999763851
ACMSD	1.5102429321	0.5414379252	0.9999763851
CCNT2	1.2442592626	0.3602406347	0.9999763851
RAB3GAP1	-1.2865543081	0.1785395959	0.9999763851
ZRANB3	-1.2125368491	0.2925639616	0.9999763851
R3HDM1	1.1278613382	0.4888598206	0.9999763851
LOC107985946	0	0	0

UBXN4	-1.1537219212	0.483133874	0.9999763851
LCT	-1.3151016298	0.8209556205	0.9999763851
MCM6	1.3733382774	0.0110091128	0.9999763851
DARS	1.1007945623	0.4757582746	0.9999763851
CXCR4	-1.089537404	0.4998023687	0.9999763851
THSD7B	-7.5664585246	0.2266167716	0.9999763851
HNMT	1.3647763335	0.5423491451	0.9999763851
SPOPL	-1.5834751417	0.1194871292	0.9999763851
NXPH2	0	0	0
ARHGAP15	-1.0502905104	0.7400504591	0.9999763851
GTDC1	1.2910500905	0.4148260026	0.9999763851
ZEB2	-1.6915579332	0.008295	0.9999763851
ACVR2A	-5.7918925833	0.1509002625	0.9999763851
ORC4	1.0887046696	0.6813109391	0.9999763851
MBD5	-1.056370503	0.8435271623	0.9999763851
EPC2	-1.0118928759	0.9529805139	0.9999763851
KIF5C	-1.5352827146	0.00169	0.9999763851
LYPD6B	-1.0453655538	0.9385241153	0.9999763851
MMADHC	1.0682028691	0.6608349488	0.9999763851
RBM43	-1.6160275616	0.2644448311	0.9999763851
NMI	1.177517447	0.3299443549	0.9999763851
TNFAIP6	-4.2864494036	0.4027150576	0.9999763851
RIF1	-1.0448133566	0.7835605872	0.9999763851
NEB	1.1971594869	0.7106462339	0.9999763851
ARL5A	1.1408676765	0.4028961212	0.9999763851
CACNB4	-1.2401979461	0.4448675194	0.9999763851
STAM2	-1.1744975129	0.5989277265	0.9999763851
FMNL2	-1.2521389154	0.4524624584	0.9999763851
PRPF40A	1.0531539214	0.7183131685	0.9999763851
ARL6IP6	-1.1879572039	0.3936072265	0.9999763851
GALNT13	-4.2864494036	0.4027150576	0.9999763851
KCNJ3	0	0	0
NR4A2	-1.6831517933	0.4363769469	0.9999763851
GPD2	-1.4037781742	0.2895274188	0.9999763851
GALNT5	0	0	0
ERMN	0	0	0
CYTIP	1.3658340055	0.4683579203	0.9999763851
ACVR1	-2.2165260254	0.2363025112	0.9999763851
UPP2	2.3401939765	0.403890843	0.9999763851
PKP4	-1.3404489624	0.2351458445	0.9999763851
TANC1	-1.5510068859	0.3690648191	0.9999763851
WDSUB1	-1.5504586825	0.1018525871	0.9999763851
BAZ2B	-1.1715748754	0.6496607764	0.9999763851
MARCH7	1.0024187022	0.9892143563	0.9999763851
CD302	-2.6629764642	0.0123545902	0.9999763851
LY75-CD302	33.3819854938	0.0772464494	0.9999763851
LY75	1.4755288217	0.4708784779	0.9999763851
ITGB6	0	0	0
RBMS1	1.2158649977	0.2165533968	0.9999763851

TANK	-1.5510086139	0.0540655241	0.9999763851
PSMD14	1.2651599853	0.0900390766	0.9999763851
TBR1	1.287203384	0.8304822833	0.9999763851
FAP	-4.0702668798	0.0155904502	0.9999763851
IFIH1	-1.1548451273	0.5123944174	0.9999763851
GCA	1.5676312967	0.1129655165	0.9999763851
GRB14	-1.1078669959	0.53137739	0.9999763851
COBLL1	0	0	0
SCN2A	-4.2849908816	0.4028453076	0.9999763851
GALNT3	-1.345634794	0.5007276973	0.9999763851
TTC21B	-2.2104919443	0.008274	0.9999763851
B3GALT1	0	0	0
STK39	1.2875826832	0.0974493574	0.9999763851
CERS6	-1.2153364155	0.2347303616	0.9999763851
NOSTRIN	-1.6880138705	0.2499161077	0.9999763851
SPC25	1.0586218826	0.7829050059	0.9999763851
DHRS9	-1.0299800453	0.9016944456	0.9999763851
LRP2	3.2356557963	0.5547505991	0.9999763851
BBS5	1.3627548182	0.5243377697	0.9999763851
KLHL41	-1.407939759	0.6698538146	0.9999763851
FASTKD1	1.0378822123	0.8743439619	0.9999763851
PPIG	1.1485763478	0.3616544547	0.9999763851
PHOSPHO2	1.255717164	0.5914567131	0.9999763851
PHOSPHO2-KLHL23	-1.6593349313	0.3346611771	0.9999763851
KLHL23	-1.0552853955	0.7725668301	0.9999763851
SSB	1.1266577587	0.4001946605	0.9999763851
METTL5	-1.4010851323	0.2483041405	0.9999763851
UBR3	-1.1840710714	0.4030694268	0.9999763851
MYO3B	1.0991008447	0.8399132411	0.9999763851
ERICH2	-1.0860476531	0.8728611828	0.9999763851
GAD1	-1.0889276106	0.8415788258	0.9999763851
GORASP2	1.0155828628	0.9151323953	0.9999763851
TLK1	1.1770333151	0.3405868971	0.9999763851
METTL8	1.0957312771	0.6429496946	0.9999763851
DCAF17	-1.0650188182	0.8374081718	0.9999763851
CYBRD1	-1.183723567	0.4902051974	0.9999763851
DYNC1I2	-1.028831282	0.8156466637	0.9999763851
SLC25A12	-1.0201591107	0.9060279096	0.9999763851
HAT1	1.329659192	0.082784236	0.9999763851
METAP1D	1.0672187123	0.8133199352	0.9999763851
DLX1	1.5006771831	0.0125623929	0.9999763851
DLX2	1.0557951289	0.8466659238	0.9999763851
ITGA6	-1.0963829722	0.484725696	0.9999763851
PDK1	-1.1244532323	0.3302215589	0.9999763851
RAPGEF4	-1.3711818216	0.7358439975	0.9999763851
ZAK	1.2430807307	0.2580011987	0.9999763851
CDCA7	1.0585635182	0.7389662898	0.9999763851
SP3	-1.3790486573	0.0745906272	0.9999763851
OLA1	1.3155003919	0.0427314064	0.9999763851

CIR1	-1.3157650748	0.1959790288	0.9999763851
SCRN3	-1.1759179026	0.5792347872	0.9999763851
GPR155	-3.368395211	0.0626175119	0.9999763851
WIPF1	-1.0459841812	0.7421501978	0.9999763851
CHN1	-1.7334433524	0.4693833927	0.9999763851
ATF2	-1.094572815	0.6598390343	0.9999763851
ATP5G3	-1.0858167284	0.4631874557	0.9999763851
KIAA1715	-1.0431332864	0.7871694096	0.9999763851
HOXD11	3.2356557963	0.5547505991	0.9999763851
MTX2	1.0816473494	0.6751414187	0.9999763851
LOC105373759	5.4776292235	0.3745831165	0.9999763851
HNRNPA3	1.0520386633	0.6869630563	0.9999763851
NFE2L2	1.2743260254	0.2368101713	0.9999763851
AGPS	1.0773193477	0.6428684649	0.9999763851
TTC30B	-1.3157515262	0.822738687	0.9999763851
TTC30A	1.2866381786	0.8226816388	0.9999763851
PDE11A	3.2353357581	0.554787377	0.9999763851
RBM45	1.1350834036	0.8010610213	0.9999763851
PRKRA	-1.0089241456	0.9571466869	0.9999763851
DFNB59	-1.8847490183	0.0375268866	0.9999763851
FKBP7	1.3680439253	0.4843730687	0.9999763851
PLEKHA3	-1.0210413177	0.9171599352	0.9999763851
TTN	-1.4418754866	0.3607696841	0.9999763851
SESTD1	1.0188638266	0.9697764847	0.9999763851
CWC22	1.371359466	0.1401334365	0.9999763851
UBE2E3	-1.0164499562	0.9286090016	0.9999763851
ITGA4	1.0050723152	0.9724437888	0.9999763851
CERKL	-1.8582989466	0.1078815235	0.9999763851
SSFA2	1.2656507543	0.641213252	0.9999763851
PDE1A	1.0833376575	0.9038766628	0.9999763851
DNAJC10	1.136151586	0.4359589748	0.9999763851
DUSP19	5.4770877341	0.3746135179	0.9999763851
NUP35	1.1574604991	0.5652056993	0.9999763851
LOC105373780	-2.5584892659	0.2979878675	0.9999763851
ZNF804A	1.0785132654	0.8845443462	0.9999763851
FSIP2	0	0	0
ZC3H15	1.3546550011	0.0502096382	0.9999763851
ITGAV	-1.0439366194	0.9212761022	0.9999763851
GULP1	0	0	0
COL5A2	0	0	0
WDR75	-1.0988918807	0.6047422117	0.9999763851
SLC40A1	1.6802495696	0.185189774	0.9999763851
ASNSD1	1.1753507171	0.5574068427	0.9999763851
ANKAR	-1.2839721658	0.5580995611	0.9999763851
OSGEPL1	-1.0451537772	0.9050379132	0.9999763851
ORMDL1	1.0319372792	0.8294712036	0.9999763851
PMS1	1.3217197514	0.1879895107	0.9999763851
C2orf88	-1.2811588713	0.3624489837	0.9999763851
HIBCH	1.1349096448	0.5116731198	0.9999763851

INPP1	1.4611209985	0.2043350135	0.9999763851
MFSD6	-1.1656630398	0.7871895855	0.9999763851
NEMP2	1.0631248897	0.7625183607	0.9999763851
NAB1	-1.20097097	0.5442048614	0.9999763851
GLS	1.203461595	0.1926900557	0.9999763851
STAT1	-1.1953625942	0.2761892204	0.9999763851
MYO1B	0	0	0
NABP1	1.3226010982	0.2475407877	0.9999763851
SLC39A10	1.0064679317	0.9818166269	0.9999763851
LOC107985971	-2.3501290511	0.2085925271	0.9999763851
DNAH7	9.9604381148	0.22157015	0.9999763851
STK17B	-1.5977058749	0.0159562928	0.9999763851
HECW2	-2.5039523457	0.34035271	0.9999763851
CCDC150	1.0744721392	0.8507730122	0.9999763851
GTF3C3	-1.2046045428	0.3390577206	0.9999763851
PGAP1	1.3586119758	0.5599282744	0.9999763851
ANKRD44	-1.3433371022	0.1160359233	0.9999763851
SF3B1	1.101481233	0.4974251565	0.9999763851
COQ10B	1.0617070171	0.8280451738	0.9999763851
HSPD1	1.2161936565	0.0912274092	0.9999763851
HSPE1	1.0887192882	0.4934485097	0.9999763851
MOB4	-1.0505769986	0.7500889766	0.9999763851
RFTN2	-1.1626873995	0.8207088244	0.9999763851
MARS2	1.0337698243	0.8920860869	0.9999763851
SATB2	-1.5936448944	0.1454563281	0.9999763851
C2orf69	1.1881869609	0.337079388	0.9999763851
TYW5	-1.5149198995	0.1991055342	0.9999763851
C2orf47	1.147649169	0.5194294567	0.9999763851
SPATS2L	1.0318857931	0.9238246089	0.9999763851
KCTD18	1.9017460936	0.1555647612	0.9999763851
SGO2	1.0596181592	0.7477135576	0.9999763851
BZW1	1.211907776	0.1950992502	0.9999763851
CLK1	-1.1544409364	0.5488455555	0.9999763851
PPIL3	1.1455048655	0.7071168376	0.9999763851
NIF3L1	1.228196225	0.2937299599	0.9999763851
ORC2	1.2499423679	0.292056781	0.9999763851
FAM126B	-1.235272467	0.4290910986	0.9999763851
NDUFB3	1.1427828103	0.4727724289	0.9999763851
CFLAR	-1.1019682055	0.592961849	0.9999763851
CASP10	-1.1572308395	0.7637527567	0.9999763851
CASP8	1.0254997488	0.9161144297	0.9999763851
ALS2CR12	3.2356557963	0.5547505991	0.9999763851
TRAK2	1.082142696	0.7706218423	0.9999763851
STRADB	1.2576512613	0.4504591554	0.9999763851
ALS2CR11	-2.3220580825	0.4395237051	0.9999763851
TMEM237	1.0129802543	0.9785239909	0.9999763851
MPP4	0	0	0
ALS2	-1.6085366287	0.2740261171	0.9999763851
FZD7	-1.4076905562	0.6701866575	0.9999763851

SUMO1	1.029316589	0.8781096652	0.9999763851
NOP58	1.0919372119	0.5823471407	0.9999763851
BMPR2	-1.4215924811	0.1991912045	0.9999763851
FAM117B	2.1367227261	0.0676758763	0.9999763851
ICA1L	1.1852358057	0.7409840788	0.9999763851
WDR12	-1.171679205	0.4404440694	0.9999763851
CARF	-2.5593885838	0.3082814576	0.9999763851
NBEAL1	1.0905719519	0.5039409136	0.9999763851
CYP20A1	-1.6052227925	0.0562555368	0.9999763851
ABI2	-1.7145036848	0.073039098	0.9999763851
NRP2	-2.289781123	0.1126782447	0.9999763851
INO80D	-1.8487364466	0.00746	0.9999763851
NDUFS1	1.3394131029	0.1283652978	0.9999763851
EEF1B2	1.0474872267	0.714454433	0.9999763851
ZDBF2	-1.0709997525	0.8763967921	0.9999763851
MDH1B	3.2356557963	0.5547505991	0.9999763851
FASTKD2	1.1412278598	0.4930903934	0.9999763851
KLF7	-1.022452828	0.9568913447	0.9999763851
CREB1	-1.3921878447	0.3511713107	0.9999763851
METTL21A	1.1061644715	0.5519756905	0.9999763851
CCNYL1	-1.9225301651	0.0304967662	0.9999763851
FZD5	-1.393561973	0.3028240493	0.9999763851
PLEKHM3	1.1604187905	0.7882500505	0.9999763851
IDH1	-1.1942428059	0.2184182577	0.9999763851
PIKFYVE	-1.0591188143	0.8397599606	0.9999763851
PTH2R	0	0	0
RPE	-1.0520036112	0.8239077838	0.9999763851
KANSL1L	-1.6389769298	0.2548164182	0.9999763851
LANCL1	1.2060033109	0.2709701544	0.9999763851
CPS1	1.6142457716	0.4217179675	0.9999763851
ERBB4	1.6250621554	0.550833575	0.9999763851
BARD1	1.0811184434	0.6718522546	0.9999763851
ABCA12	1.030747947	0.9668202631	0.9999763851
ATIC	-1.0172597613	0.9050531181	0.9999763851
FN1	-1.1230477663	0.8441633752	0.9999763851
MREG	-1.0239678005	0.9571046039	0.9999763851
PECR	-1.0065652436	0.9901000633	0.9999763851
TMEM169	3.2353357581	0.554787377	0.9999763851
XRCC5	1.0736427223	0.5830282156	0.9999763851
MARCH4	-1.3161718537	0.8279629082	0.9999763851
SMARCAL1	1.1233166013	0.7599740781	0.9999763851
RPL37A	1.0139632833	0.9066603815	0.9999763851
IGFBP2	-1.0941830822	0.5904810871	0.9999763851
IGFBP5	3.2361532718	0.5546934395	0.9999763851
TNS1	-1.3160416743	0.8274241686	0.9999763851
CXCR2	1.1585942996	0.5959401862	0.9999763851
ARPC2	1.2127283019	0.130647845	0.9999763851
GPBAR1	0	0	0
AAMP	-1.31624394	0.0565075456	0.9999763851

PNKD	1.0807595273	0.6446703911	0.9999763851
TMBIM1	-1.608883212	0.2429873729	0.9999763851
CATIP	-1.3950272772	0.67648818	0.9999763851
SLC11A1	-6.346613468	0.0588664936	0.9999763851
CTDSP1	-1.2279642169	0.181960746	0.9999763851
VIL1	0	0	0
USP37	-1.6746698715	0.0125361703	0.9999763851
CNOT9	1.1348935546	0.4411261718	0.9999763851
PLCD4	3.478538239	0.2573416581	0.9999763851
ZNF142	1.2586333958	0.4738039921	0.9999763851
BCS1L	1.2647652031	0.4211559409	0.9999763851
RNF25	1.6062903958	0.6464772155	0.9999763851
STK36	1.0512676278	0.8447935887	0.9999763851
TTLL4	-1.2577809428	0.2525127221	0.9999763851
CYP27A1	0	0	0
WNT6	1.2809090962	0.6171266537	0.9999763851
WNT10A	2.2669099672	0.4734597612	0.9999763851
CDK5R2	-2.3222877795	0.4500648964	0.9999763851
NHEJ1	1.058833886	0.903003388	0.9999763851
SLC23A3	2.3404251821	0.4102399801	0.9999763851
CNPPD1	1.0265087714	0.8691456693	0.9999763851
FAM134A	1.2411878366	0.2514461794	0.9999763851
ZFAND2B	-1.0994281092	0.586056761	0.9999763851
ABCB6	1.2445757792	0.6423741543	0.9999763851
ATG9A	-1.1896060824	0.4069822469	0.9999763851
ANKZF1	1.2631552229	0.3054530456	0.9999763851
GLB1L	1.3421210769	0.701026043	0.9999763851
STK16	1.0349392099	0.8933431834	0.9999763851
TUBA4A	-1.0059257707	0.9628133189	0.9999763851
DNAJB2	-1.0064810351	0.9872717574	0.9999763851
PTPRN	0	0	0
DNPEP	1.4141411733	0.0603376325	0.9999763851
DES	0	0	0
SPEG	-1.3154339304	0.8205576571	0.9999763851
LOC100996693	0	0	0
GMPPA	-1.2861708228	0.548629652	0.9999763851
ASIC4	0	0	0
CHPF	1.5089755748	0.0670160627	0.9999763851
TMEM198	4.4488556079	0.1488038074	0.9999763851
INHA	0	0	0
STK11IP	1.3518913178	0.3256132792	0.9999763851
SGPP2	-1.4083241465	0.6545786505	0.9999763851
FARSB	1.0019542801	0.9911622876	0.9999763851
ACSL3	-1.151837056	0.4114028002	0.9999763851
AP1S3	1.413938195	0.3455269105	0.9999763851
WDFY1	-1.6115323606	0.0330033867	0.9999763851
MRPL44	1.1190298388	0.5980882635	0.9999763851
SERPINE2	-1.2930574702	0.0697667096	0.9999763851
CUL3	1.2897414016	0.186417693	0.9999763851

DOCK10	-1.1927869136	0.4791008504	0.9999763851
NYAP2	3.2353357581	0.554787377	0.9999763851
IRS1	-1.4328446441	0.5056254598	0.9999763851
RHBDD1	-1.4455661865	0.2533291841	0.9999763851
COL4A4	1.8881414096	0.5402431434	0.9999763851
COL4A3	-1.3157515262	0.8226830038	0.9999763851
MFF	-1.0751054924	0.6957496572	0.9999763851
AGFG1	1.2721291976	0.2531649773	0.9999763851
CCL20	-3.2612224403	0.0246048323	0.9999763851
DAW1	0	0	0
DNER	0	0	0
TRIP12	1.0569083798	0.7581450531	0.9999763851
SLC16A14	3.2353357581	0.554787377	0.9999763851
SP110	-1.1005813323	0.7165116417	0.9999763851
SP140	1.8138024348	0.5770921647	0.9999763851
SP140L	1.7589657787	0.1679799387	0.9999763851
SP100	-1.5910363362	0.0290113406	0.9999763851
CAB39	-1.0491007222	0.840068039	0.9999763851
ITM2C	-1.0381525376	0.8730539	0.9999763851
GPR55	-1.2612794495	0.3938336729	0.9999763851
PSMD1	1.1855203404	0.2633463644	0.9999763851
ARMC9	-1.003472981	0.9782275909	0.9999763851
B3GNT7	0	0	0
NCL	1.0618950076	0.6135822398	0.9999763851
PTMA	-1.4152922392	0.006595	0.9999763851
PDE6D	-1.3646188803	0.2776171639	0.9999763851
COPS7B	-1.0053745708	0.9817081137	0.9999763851
DIS3L2	1.2228272837	0.5122888671	0.9999763851
CHRND	0	0	0
TIGD1	-1.3644955467	0.6906903137	0.9999763851
EIF4E2	1.0298798404	0.8372276761	0.9999763851
EFHD1	-4.2849908816	0.4028453076	0.9999763851
GIGYF2	1.0075672987	0.9690259828	0.9999763851
KCNJ13	3.2361532718	0.5546934395	0.9999763851
INPP5D	-1.0656500116	0.6724686222	0.9999763851
ATG16L1	-1.1259116752	0.790438015	0.9999763851
SAG	1.2869488883	0.8190152004	0.9999763851
DGKD	1.168029501	0.3217599549	0.9999763851
USP40	1.1372511472	0.6940439831	0.9999763851
UGT1A8	0	0	0
HJURP	-1.1317195226	0.5104703586	0.9999763851
TRPM8	0	0	0
ARL4C	1.0426561918	0.7768635251	0.9999763851
SH3BP4	0	0	0
AGAP1	-4.3331566429	0.1395554976	0.9999763851
ASB18	0	0	0
ACKR3	-1.2958166054	0.7397560477	0.9999763851
COPS8	1.4653084177	0.0639875658	0.9999763851
COL6A3	-7.5622178268	0.2177145568	0.9999763851

MLPH	-2.0420845165	0.0483724655	0.9999763851
RAB17	-1.334991364	0.6555336911	0.9999763851
LRRFIP1	1.0107219472	0.9316701231	0.9999763851
RBM44	1.924244766	0.44965329	0.9999763851
UBE2F	1.1264957133	0.5053587244	0.9999763851
SCLY	1.5877250627	0.02648611	0.9999763851
ESPNL	3.2353357581	0.554787377	0.9999763851
KLHL30	-1.3715126673	0.7509237272	0.9999763851
FAM132B	-1.4346570644	0.2611773109	0.9999763851
ILKAP	-1.0952838523	0.5230096299	0.9999763851
HES6	1.0760954497	0.6423186805	0.9999763851
PER2	1.1173494516	0.7024880715	0.9999763851
TRAF3IP1	-1.3413723037	0.3009655949	0.9999763851
ASB1	-1.0247048659	0.9251589075	0.9999763851
TWIST2	-2.3222877795	0.4502048392	0.9999763851
HDAC4	0	0	0
NDUFA10	-1.0365361364	0.8281832422	0.9999763851
COPS9	1.7919480141	0.0004264	0.4545015308
GPC1	-1.5401845921	0.3097298624	0.9999763851
ANKMY1	1.7773630804	0.2370355448	0.9999763851
DUSP28	-1.3200992251	0.5541112764	0.9999763851
RNPEPL1	-1.0957050206	0.713383221	0.9999763851
CAPN10	1.0051659711	0.9905851628	0.9999763851
GPR35	-2.0858069141	0.1110354187	0.9999763851
KIF1A	0	0	0
SNED1	3.2356557963	0.5547505991	0.9999763851
MTERF4	1.3243738256	0.4543828446	0.9999763851
PASK	1.098391833	0.8417049788	0.9999763851
PPP1R7	1.1411371994	0.5658770134	0.9999763851
ANO7	0	0	0
HDLBP	1.0155421423	0.9266311192	0.9999763851
LOC101927509	0	0	0
SEPT2	1.0293414913	0.8592843763	0.9999763851
FARP2	1.7104570823	0.1386774286	0.9999763851
STK25	-1.1210164156	0.5152197405	0.9999763851
THAP4	1.106299355	0.6326684507	0.9999763851
ATG4B	-1.0592140668	0.7616685109	0.9999763851
DTYMK	1.1493513229	0.4284809433	0.9999763851
ING5	1.1430300728	0.6366830987	0.9999763851
D2HGDH	1.1642255242	0.4520376843	0.9999763851
NEU4	-4.2849908816	0.4028453076	0.9999763851
LOC101927174	0	0	0
CNTN6	0	0	0
CNTN4	0	0	0
TRNT1	1.1477712501	0.5295638966	0.9999763851
CRBN	-1.0727123653	0.7566466531	0.9999763851
LRRN1	3.2353357581	0.554787377	0.9999763851
SUMF1	-1.1221714937	0.6320226751	0.9999763851
SETMAR	1.8056665272	0.2099207542	0.9999763851

ITPR1	-1.1523439634	0.5318456503	0.9999763851
BHLHE40	-1.3000491284	0.282705716	0.9999763851
ARL8B	-1.0762454222	0.6173299236	0.9999763851
EDEM1	-1.2668210846	0.4765169547	0.9999763851
GRM7	3.2356557963	0.5547505991	0.9999763851
CAV3	0	0	0
OXTR	-4.3327792956	0.1655012474	0.9999763851
RAD18	-1.0456554272	0.8370740494	0.9999763851
SRGAP3	-4.018630071	0.0332148527	0.9999763851
THUMPD3	1.1862630021	0.4075195454	0.9999763851
SETD5	-1.181070644	0.218698143	0.9999763851
LHFPL4	3.2361532718	0.5546934395	0.9999763851
MTMR14	-1.0558994764	0.7685427489	0.9999763851
CPNE9	2.9534611339	0.194219602	0.9999763851
BRPF1	1.0314917624	0.8506219087	0.9999763851
OGG1	-1.2909260049	0.1369812943	0.9999763851
CAMK1	0	0	0
TADA3	1.091662145	0.6866751692	0.9999763851
ARPC4	1.1375239018	0.4419371741	0.9999763851
ARPC4-TTLL3	0	0	0
TTLL3	-1.0019250308	0.9939583944	0.9999763851
RPUSD3	-1.1410465573	0.4485492182	0.9999763851
CIDEC	0	0	0
JAGN1	-1.0576824332	0.7330036675	0.9999763851
IL17RE	1.0304093918	0.9664855273	0.9999763851
IL17RC	-1.8173414279	0.4639470588	0.9999763851
CRELD1	1.1959239111	0.7311287311	0.9999763851
PRRT3	-7.5622178268	0.2177145568	0.9999763851
EMC3	-1.0437297612	0.7728017795	0.9999763851
LOC401052	1.2044957046	0.4531537824	0.9999763851
FANCD2	-1.4162910309	0.2544841531	0.9999763851
FANCD2OS	1.5322833884	0.0118674101	0.9999763851
BRK1	1.1184858665	0.3854711568	0.9999763851
VHL	-1.3974022382	0.0290985738	0.9999763851
IRAK2	-1.5730923026	0.130151282	0.9999763851
TATDN2	-1.1225303251	0.4648594309	0.9999763851
GHRL	-1.1488069744	0.8543538549	0.9999763851
SEC13	1.1066405779	0.4749992385	0.9999763851
ATP2B2	-1.5447668635	0.5555166043	0.9999763851
SLC6A11	0	0	0
HRH1	1.0004240003	0.9986444717	0.9999763851
ATG7	-1.1267348979	0.490798587	0.9999763851
VGLL4	-1.0080901155	0.9615569158	0.9999763851
TAMM41	1.040875015	0.8496813845	0.9999763851
TIMP4	-4.2864494036	0.4027150576	0.9999763851
PPARG	1.0623332917	0.8119056555	0.9999763851
TSEN2	1.4672224812	0.1710384784	0.9999763851
MKRN2OS	-1.4081850562	0.65013235	0.9999763851
MKRN2	1.3127130603	0.2395461498	0.9999763851

RAF1	-1.1071698394	0.6542091221	0.9999763851
TMEM40	0	0	0
CAND2	7.4933530102	0.2989396898	0.9999763851
RPL32	1.0287151038	0.8081143108	0.9999763851
IQSEC1	1.0332635126	0.8869647729	0.9999763851
NUP210	1.0453770233	0.730295552	0.9999763851
HDAC11	-2.7940687382	0.0568493373	0.9999763851
FBLN2	0	0	0
CHCHD4	1.1102781544	0.5206172992	0.9999763851
TMEM43	-1.1752097473	0.4680949095	0.9999763851
XPC	-1.0883473716	0.7627548108	0.9999763851
LSM3	1.1397783878	0.3148169451	0.9999763851
SLC6A6	-1.3415713902	0.0665190302	0.9999763851
CCDC174	1.004888712	0.987330815	0.9999763851
C3orf20	1.0686473141	0.8890315687	0.9999763851
FGD5	-4.2849908816	0.4028453076	0.9999763851
NR2C2	-1.3464551322	0.2280016849	0.9999763851
MRPS25	1.0012684488	0.9932513762	0.9999763851
RBSN	-1.2003374826	0.4292591695	0.9999763851
CAPN7	1.2553257784	0.2869455725	0.9999763851
SH3BP5	1.007076005	0.9686154191	0.9999763851
METTL6	1.3382482306	0.2792513666	0.9999763851
EAF1	-1.1759955159	0.4516963407	0.9999763851
COLQ	-1.4840867037	0.2713885716	0.9999763851
HACL1	-1.349716291	0.2570146444	0.9999763851
BTD	1.0922041155	0.8273107258	0.9999763851
ANKRD28	-1.3767791716	0.0460540382	0.9999763851
GALNT15	3.2353357581	0.554787377	0.9999763851
DPH3	1.2691034552	0.2568972474	0.9999763851
OXNAD1	1.322243449	0.0932391268	0.9999763851
RFTN1	-1.3428126404	0.3782957564	0.9999763851
PLCL2	1.0075327263	0.9863553957	0.9999763851
TBC1D5	1.2357949024	0.2108171523	0.9999763851
LOC339862	-1.8164522609	0.1477357667	0.9999763851
SATB1	-1.1595432777	0.3049584518	0.9999763851
RAB5A	1.1738981864	0.3164260599	0.9999763851
PP2D1	16.6918753579	0.1320521391	0.9999763851
KAT2B	-1.2473636661	0.4846479579	0.9999763851
SGO1	-1.0475916265	0.8019187081	0.9999763851
ZNF385D	7.7488609396	0.3059383556	0.9999763851
UBE2E2	1.4054848718	0.2996660234	0.9999763851
UBE2E1	-1.0940932977	0.7230490882	0.9999763851
NKIRAS1	1.1325197708	0.3350055184	0.9999763851
RPL15	1.0589733125	0.6269142279	0.9999763851
NR1D2	-1.4410787947	0.4050751534	0.9999763851
RARB	1.3264789913	0.7365958046	0.9999763851
TOP2B	1.0478263509	0.7016755118	0.9999763851
NGLY1	-1.0834540683	0.6362328654	0.9999763851
OXSM	-1.0426858592	0.8795744584	0.9999763851

NEK10	2.3090331185	0.1558297642	0.9999763851
SLC4A7	1.067768791	0.7609120272	0.9999763851
EOMES	5.4784711963	0.3745358536	0.9999763851
CMC1	1.1195603727	0.5863939411	0.9999763851
AZI2	1.1276762637	0.499121037	0.9999763851
ZCWPW2	2.8345356849	0.3327756039	0.9999763851
TGFBR2	1.0055675201	0.9822270647	0.9999763851
STT3B	1.0045017876	0.9749817907	0.9999763851
OSBPL10	0	0	0
GPD1L	-1.4487308991	0.0744995686	0.9999763851
CMTM8	-1.0214219889	0.9548364502	0.9999763851
CMTM7	1.1406010161	0.5341857646	0.9999763851
CMTM6	-1.250469408	0.0703500523	0.9999763851
DYNC1LI1	1.3843067166	0.1118360057	0.9999763851
CNOT10	-1.2619950065	0.4048294927	0.9999763851
TRIM71	-1.9429501577	0.2004535376	0.9999763851
GLB1	-1.0504794572	0.7365524079	0.9999763851
CRTAP	-1.2736912009	0.0758954065	0.9999763851
FBXL2	2.8676515213	0.2915761253	0.9999763851
UBP1	-1.0752055631	0.6291853231	0.9999763851
CLASP2	-1.1610091359	0.4320930931	0.9999763851
PDCD6IP	-1.1983893958	0.2820790897	0.9999763851
STAC	-1.0269784107	0.9486325388	0.9999763851
EPM2AIP1	1.0717816676	0.7242224313	0.9999763851
MLH1	1.3299277551	0.1409753694	0.9999763851
LRRFIP2	1.0785932459	0.706945258	0.9999763851
GOLGA4	-1.3905315185	0.0219224215	0.9999763851
C3orf35	-1.8717794426	0.3395007389	0.9999763851
ITGA9	-1.5733616565	0.3774394479	0.9999763851
CTDSPL	1.1539201667	0.4311058932	0.9999763851
VILL	-1.6039579189	0.1805367231	0.9999763851
PLCD1	18.0173590016	0.1388117476	0.9999763851
DLEC1	1.6246521587	0.5746381271	0.9999763851
ACAA1	-1.0295529533	0.8650338596	0.9999763851
MYD88	1.5289821076	0.0585515538	0.9999763851
OXSR1	-1.0330184437	0.8712528786	0.9999763851
SLC22A14	3.2353357581	0.554787377	0.9999763851
XYLB	-1.2174112535	0.2692664988	0.9999763851
ACVR2B	1.0625449955	0.8540412275	0.9999763851
EXOG	1.9363876517	0.048611527	0.9999763851
SCN5A	-1.8164818713	0.4779791507	0.9999763851
SCN10A	0	0	0
SCN11A	-4.2849908816	0.4028453076	0.9999763851
WDR48	1.0897050618	0.7209698274	0.9999763851
GORASP1	1.3839674353	0.3978612769	0.9999763851
TTC21A	3.2353357581	0.554787377	0.9999763851
CSRNP1	-1.7191583623	0.0501804363	0.9999763851
XIRP1	-8.342239448	0.2163081661	0.9999763851
CX3CR1	2.4012906669	0.0538026956	0.9999763851

SLC25A38	-1.334034802	0.0815532523	0.9999763851
RPSA	1.0717623891	0.5628741385	0.9999763851
MYRIP	-1.1953557556	0.7975410711	0.9999763851
EIF1B	1.0185260796	0.9108009824	0.9999763851
ENTPD3	0	0	0
RPL14	1.0177508349	0.8910703578	0.9999763851
ZNF619	-1.1548225619	0.7833257766	0.9999763851
ZNF620	2.3405535777	0.4308645521	0.9999763851
ZNF621	1.1170664285	0.7693185044	0.9999763851
CTNNB1	-1.0215252336	0.8811382445	0.9999763851
ULK4	1.6845986357	0.1416753386	0.9999763851
TRAK1	-1.1775891752	0.3463772208	0.9999763851
SEC22C	1.1174244077	0.5507149401	0.9999763851
SS18L2	-1.2641030885	0.1406550747	0.9999763851
NKTR	-1.2681687265	0.1638393158	0.9999763851
ZBTB47	1.8415859751	0.0332598878	0.9999763851
CCDC13	2.9291342347	0.3283490844	0.9999763851
HIGD1A	1.3143274591	0.0651441084	0.9999763851
ACKR2	1.1723339317	0.7697497117	0.9999763851
FAM198A	-2.7031708163	0.0244698715	0.9999763851
POMGNT2	1.0190202406	0.9128029301	0.9999763851
SNRK	-1.1832772497	0.4216762857	0.9999763851
ANO10	-1.23763864	0.4053514189	0.9999763851
ABHD5	1.3958741906	0.2346355976	0.9999763851
TOPAZ1	0	0	0
TCAIM	-1.0866861808	0.6977980858	0.9999763851
ZNF445	-1.4319310951	0.2119951858	0.9999763851
ZNF852	1.566866561	0.3081323845	0.9999763851
ZKSCAN7	0	0	0
ZNF197	-1.0292467562	0.9407412209	0.9999763851
ZNF35	1.1897545502	0.7989394451	0.9999763851
KIAA1143	1.009163893	0.9483005362	0.9999763851
KIF15	1.1225444413	0.5877998829	0.9999763851
TMEM42	-1.1598170686	0.6114323215	0.9999763851
TGM4	-1.3717398996	0.7338113203	0.9999763851
ZDHHC3	-1.1136408878	0.474869207	0.9999763851
EXOSC7	1.0500351774	0.7351020077	0.9999763851
CLEC3B	1.3409143441	0.7126222688	0.9999763851
CDCP1	-1.4290145507	0.5263961287	0.9999763851
TMEM158	-1.8381419039	3.47E-05	0.0718176673
LARS2	1.0405342131	0.8746924846	0.9999763851
LIMD1	-1.3513996155	0.2846240039	0.9999763851
SACM1L	-1.0646522032	0.6458156236	0.9999763851
SLC6A20	0	0	0
LZTFL1	-1.0503551024	0.7992238996	0.9999763851
FYCO1	1.2055907042	0.6104066983	0.9999763851
CCR1	-1.6849455268	0.0983348929	0.9999763851
CCR3	1.286354513	0.820733428	0.9999763851
CCR2	16.6960867465	0.1348328872	0.9999763851

CCRL2	-1.3721150467	0.7447608683	0.9999763851
LTF	-1.9507065805	0.3146843414	0.9999763851
LRRC2	0	0	0
TDGF1	0	0	0
PTH1R	0	0	0
CCDC12	1.3325320271	0.0922206878	0.9999763851
NBEAL2	1.1951278832	0.3510620049	0.9999763851
SETD2	-1.0570765965	0.7364368405	0.9999763851
KIF9	-1.055371679	0.7914033392	0.9999763851
KLHL18	1.6965654779	0.0306084394	0.9999763851
PTPN23	1.2425710678	0.3065395238	0.9999763851
SCAP	1.0427310614	0.786892453	0.9999763851
ELP6	-1.1362490776	0.5673381854	0.9999763851
CSPG5	-7.5664585246	0.2265643942	0.9999763851
SMARCC1	-1.1643628995	0.2304893968	0.9999763851
DHX30	1.2050419075	0.2011843909	0.9999763851
MAP4	1.1084478407	0.4663627025	0.9999763851
CDC25A	1.0680911555	0.6642944652	0.9999763851
CAMP	1.1601789857	0.8601794734	0.9999763851
ZNF589	-1.2603689241	0.5057108829	0.9999763851
NME6	-1.053044499	0.8407376939	0.9999763851
SPINK8	3.2353357581	0.554787377	0.9999763851
FBXW12	0	0	0
PLXNB1	-1.294661912	0.6305398827	0.9999763851
CCDC51	1.2013557477	0.2786685372	0.9999763851
TMA7	-1.0154187393	0.8974134317	0.9999763851
ATRIP	1.0089601549	0.9763413814	0.9999763851
TREX1	1.5022661609	0.1618609865	0.9999763851
SHISA5	-1.3364532799	0.0440299301	0.9999763851
PFKFB4	1.0207868011	0.9556409858	0.9999763851
COL7A1	1.079433243	0.8576945853	0.9999763851
UQCRC1	1.2064796723	0.2104636208	0.9999763851
TMEM89	3.2361532718	0.5546934395	0.9999763851
SLC26A6	1.118477166	0.693116269	0.9999763851
CELSR3	-1.6218005948	0.4141172723	0.9999763851
NCKIPSD	1.5065159811	0.1258889398	0.9999763851
IP6K2	-1.2586364913	0.1681478956	0.9999763851
PRKAR2A	1.0183801972	0.8878884122	0.9999763851
SLC25A20	-1.4461583372	0.2018691835	0.9999763851
ARIH2OS	-1.3151016298	0.8209848177	0.9999763851
ARIH2	-1.0512004785	0.7166486665	0.9999763851
P4HTM	0	0	0
WDR6	1.2044969422	0.7915381575	0.9999763851
DALRD3	1.0216360171	0.8901636728	0.9999763851
NDUFAF3	1.1935092929	0.3256037592	0.9999763851
IMPDH2	-1.0868893997	0.6039213461	0.9999763851
QRICH1	1.1450961859	0.5364572909	0.9999763851
QARS	1.1297647221	0.4522697408	0.9999763851
USP19	-1.2730732952	0.1945546773	0.9999763851

CCDC71	1.112968273	0.5667916624	0.9999763851
KLHDC8B	-4.287394647	0.4026306792	0.9999763851
C3orf84	0	0	0
CCDC36	5.4770877341	0.3746135179	0.9999763851
C3orf62	-1.1344077061	0.7464283734	0.9999763851
USP4	1.4407884265	0.0439216841	0.9999763851
LOC107986084	1.2866381786	0.8226168136	0.9999763851
GPX1	1.0181788103	0.8841901527	0.9999763851
RHOA	1.0813294127	0.5118091751	0.9999763851
TCTA	1.2073106237	0.5647292456	0.9999763851
AMT	1.3652112359	0.5197398213	0.9999763851
NICN1	-1.086531101	0.7732150215	0.9999763851
DAG1	1.0816065448	0.7410029171	0.9999763851
APEH	1.2019178786	0.2176038003	0.9999763851
MST1	-1.2662554053	0.5405902545	0.9999763851
RNF123	-1.1094899287	0.6124824654	0.9999763851
AMIGO3	-1.571868157	0.3997137873	0.9999763851
GMPPB	-1.3360123905	0.5553974268	0.9999763851
IP6K1	1.2458110077	0.3273545291	0.9999763851
CDHR4	0	0	0
FAM212A	-1.4492943105	0.1819169842	0.9999763851
UBA7	1.4029474039	0.5112435416	0.9999763851
TRAIP	1.4269612769	0.2948756568	0.9999763851
CAMKV	-7.5622178268	0.2177145568	0.9999763851
MST1R	0	0	0
MON1A	1.2570816648	0.4967978932	0.9999763851
RBM6	1.10637051	0.5831500528	0.9999763851
RBM5	1.1745119895	0.3176497104	0.9999763851
SEMA3F	1.2668875868	0.6492200187	0.9999763851
GNAI2	1.1859683028	0.2025705929	0.9999763851
SEMA3B	1.3258241993	0.7378409434	0.9999763851
IFRD2	1.1505585744	0.3909206296	0.9999763851
HYAL3	1.4232167389	0.2290981588	0.9999763851
NAT6	-2.4060847021	0.025561888	0.9999763851
HYAL1	5.4784711963	0.3745358536	0.9999763851
HYAL2	-1.2022077587	0.5648776522	0.9999763851
TUSC2	-1.099290443	0.622775354	0.9999763851
RASSF1	-1.4364298205	0.0525935931	0.9999763851
ZMYND10	-1.148669206	0.8578196038	0.9999763851
NPRL2	1.3267267255	0.1078827133	0.9999763851
CYB561D2	1.3623915418	0.2598492146	0.9999763851
TMEM115	1.1385385199	0.565983322	0.9999763851
CACNA2D2	1.8685954806	0.5480020904	0.9999763851
C3orf18	1.7330034736	0.1931047234	0.9999763851
HEMK1	1.008711577	0.9781373666	0.9999763851
CISH	-1.0607911621	0.8243468088	0.9999763851
MAPKAPK3	1.0674408861	0.6857148619	0.9999763851
MANF	-1.2842339611	0.1088014636	0.9999763851
RBM15B	-1.1465585236	0.3686786284	0.9999763851

VPRBP	-1.1215421365	0.628602949	0.9999763851
RAD54L2	-1.035563939	0.8512324164	0.9999763851
TEX264	1.1010967543	0.6584734852	0.9999763851
RRP9	1.4528148907	0.0646924354	0.9999763851
PARP3	1.1536660933	0.6311394132	0.9999763851
PCBP4	1.0630267301	0.8302649895	0.9999763851
ABHD14B	1.5036434156	0.020672614	0.9999763851
ABHD14A	1.206409269	0.5178664215	0.9999763851
ABHD14A-ACY1	-31.0891925927	0.0379638326	0.9999763851
ACY1	-1.1714401286	0.4653538541	0.9999763851
RPL29	1.1348291983	0.2860303677	0.9999763851
DUSP7	-1.2655429917	0.2810170373	0.9999763851
POC1A	-1.3870331004	0.0909580128	0.9999763851
ALAS1	-1.3431951387	0.1602276443	0.9999763851
TLR9	2.3918191881	0.1134620566	0.9999763851
TWF2	-1.2528705581	0.1915441461	0.9999763851
PPM1M	-1.2909116169	0.5669662911	0.9999763851
WDR82	1.1168592529	0.4062727201	0.9999763851
GLYCTK	1.6712807673	0.4611016166	0.9999763851
DNAH1	-1.318758176	0.5780306891	0.9999763851
BAP1	1.2092857934	0.2657621708	0.9999763851
PHF7	-1.3824878834	0.0760843585	0.9999763851
SEMA3G	1.3716875655	0.3001268341	0.9999763851
NISCH	1.0744379843	0.7406030408	0.9999763851
NT5DC2	1.062034588	0.8439791066	0.9999763851
SMIM4	1.4403855545	0.0195705699	0.9999763851
PBRM1	-1.0202071526	0.9018105178	0.9999763851
GNL3	1.1423785752	0.3490961165	0.9999763851
GLT8D1	-1.1432929983	0.4786635641	0.9999763851
SPCS1	1.2652883548	0.1175221587	0.9999763851
NEK4	1.25484259	0.4035220135	0.9999763851
ITIH4	1.5114252137	0.3657064773	0.9999763851
MUSTN1	7.7199860138	0.2826011338	0.9999763851
TMEM110	-1.2981616433	0.2819922572	0.9999763851
SFMBT1	1.1516812757	0.6214011268	0.9999763851
LOC105377135	1.0274983165	0.9757481446	0.9999763851
RFT1	-1.3809935403	0.0745629145	0.9999763851
PRKCD	-1.0970845745	0.6140752348	0.9999763851
TKT	1.0544997161	0.7493719153	0.9999763851
DCP1A	-1.0449757749	0.8223454459	0.9999763851
CACNA1D	3.2361532718	0.5546934395	0.9999763851
IL17RB	-1.4386322103	0.4485935632	0.9999763851
ACTR8	-1.0560428932	0.8092423462	0.9999763851
SELK	-1.0709099804	0.7311887482	0.9999763851
CACNA2D3	1.5012317313	0.007362	0.9999763851
WNT5A	0	0	0
CCDC66	1.2447981031	0.2946166604	0.9999763851
FAM208A	-1.2808206717	0.2766327271	0.9999763851
ARHGEF3	-1.433835654	0.0754697903	0.9999763851

SPATA12	-1.1495270598	0.8432431454	0.9999763851
HESX1	-1.3709710942	0.7487098914	0.9999763851
APPL1	-1.2954282256	0.3557036608	0.9999763851
ASB14	-1.2474039722	0.2092845849	0.9999763851
PDE12	-1.3268415177	0.0543962715	0.9999763851
ARF4	-1.029760818	0.8261470739	0.9999763851
DENND6A	-1.0179271407	0.9712458391	0.9999763851
SLMAP	-1.2023597996	0.4089025179	0.9999763851
FLNB	1.0106445256	0.9438235523	0.9999763851
LOC105377650	-4.2864494036	0.4027150576	0.9999763851
DNASE1L3	0	0	0
ABHD6	-1.3020370284	0.3743615908	0.9999763851
RPP14	1.1808778314	0.4742222503	0.9999763851
PXK	-1.0936020386	0.5419470112	0.9999763851
PDHB	1.1283951438	0.5021333572	0.9999763851
KCTD6	2.3090611935	0.1771782426	0.9999763851
ACOX2	2.5225909253	0.0990722959	0.9999763851
FAM107A	0	0	0
FAM3D	-1.3154339304	0.8205576571	0.9999763851
C3orf67	0	0	0
FHIT	-1.4331262132	0.4996837349	0.9999763851
PTPRG	-1.0523028827	0.7128055231	0.9999763851
CADPS	-1.3151016298	0.821010945	0.9999763851
SYNPR	3.2353357581	0.554787377	0.9999763851
THOC7	1.3001255069	0.0709121042	0.9999763851
ATXN7	-1.6156840739	0.0142518415	0.9999763851
PSMD6	1.5431751623	0.0168415862	0.9999763851
PRICKLE2	0	0	0
ADAMTS9	-1.5367359325	0.4414005628	0.9999763851
MAGI1	5.4781708352	0.3745527127	0.9999763851
SLC25A26	1.2230601644	0.340329827	0.9999763851
LRIG1	-1.0356699342	0.9588830561	0.9999763851
KBTBD8	2.0914187992	0.0461059561	0.9999763851
SUCLG2	-1.321776646	0.1167072645	0.9999763851
EOGT	1.1614567738	0.7167575371	0.9999763851
TMF1	-1.2398489956	0.4365061081	0.9999763851
UBA3	1.1289396586	0.4682721371	0.9999763851
ARL6IP5	-1.0240098	0.873655646	0.9999763851
FRMD4B	-1.2848784482	0.4453886241	0.9999763851
MITF	-1.559876047	0.3820186292	0.9999763851
FOXP1	1.0863499396	0.5825911636	0.9999763851
EIF4E3	1.2015752383	0.3079674235	0.9999763851
PROK2	1.0986927862	0.7362073779	0.9999763851
RYBP	-1.0672101765	0.6896426489	0.9999763851
SHQ1	1.1158188329	0.6031230408	0.9999763851
GXYLT2	-2.9105919729	0.0649478678	0.9999763851
PPP4R2	1.0114932557	0.9371914059	0.9999763851
CNTN3	0	0	0
ZNF717	7.7211724672	0.2779709379	0.9999763851

ROBO1	0	0	0
GBE1	-1.0061687211	0.9703935322	0.9999763851
CADM2	12.2055584829	0.17807181	0.9999763851
CHMP2B	1.1959537026	0.2915277672	0.9999763851
POU1F1	0	0	0
HTR1F	0	0	0
CGGBP1	1.0077751846	0.9610768337	0.9999763851
ZNF654	1.0452015979	0.8444188116	0.9999763851
C3orf38	1.2441935159	0.2768198796	0.9999763851
EPHA3	0	0	0
PROS1	1.1271106302	0.7655154619	0.9999763851
ARL13B	-1.0579648884	0.8583771357	0.9999763851
DHFRL1	-1.0103031309	0.9790520265	0.9999763851
NSUN3	-1.9110287029	0.1338317742	0.9999763851
EPHA6	1.1717179916	0.7699833094	0.9999763851
ARL6	-1.2948054853	0.60878962	0.9999763851
CRYBG3	-1.2204840837	0.7215920718	0.9999763851
MINA	1.150341172	0.5158985821	0.9999763851
GABRR3	3.2356557963	0.5547505991	0.9999763851
CLDND1	1.2304480623	0.4507309247	0.9999763851
CPOX	1.1445562619	0.4733118136	0.9999763851
ST3GAL6	-1.3531219507	0.2112117614	0.9999763851
DCBLD2	-1.6239009366	0.072955016	0.9999763851
COL8A1	-4.287394647	0.4026306792	0.9999763851
CMSS1	1.3274399103	0.0535222942	0.9999763851
TBC1D23	-1.2544461108	0.3660767516	0.9999763851
NIT2	-1.05396332	0.7731786445	0.9999763851
TOMM70	-1.3228420713	0.0348044416	0.9999763851
LNP1	-1.3722068069	0.7436936864	0.9999763851
TMEM45A	-1.6814919341	0.4672141193	0.9999763851
ADGRG7	5.4781708352	0.3745527127	0.9999763851
TFG	-1.086508216	0.5711392284	0.9999763851
IMPG2	1.2865522343	0.8248288601	0.9999763851
SENP7	-1.8524248627	0.0111026412	0.9999763851
TRMT10C	1.4460357287	0.0271500582	0.9999763851
PCNP	1.0521476734	0.6747860109	0.9999763851
ZBTB11	1.4213218371	0.0854192528	0.9999763851
RPL24	-1.0655664024	0.6235275006	0.9999763851
LOC105374013	-2.0607746097	0.3328245492	0.9999763851
CEP97	-1.1899313222	0.5113865898	0.9999763851
NXPE3	1.1300497749	0.3896473501	0.9999763851
NFKBIZ	1.0752693155	0.8002211296	0.9999763851
ALCAM	-3.0163924595	0.0577613048	0.9999763851
CBLB	-2.2903861361	0.0965124574	0.9999763851
BBX	-1.1940754517	0.3334027285	0.9999763851
CD47	1.1333650593	0.4622578721	0.9999763851
IFT57	1.034785049	0.8568061014	0.9999763851
MYH15	1.10776511	0.7764920144	0.9999763851
KIAA1524	1.2830248059	0.2986100436	0.9999763851

DZIP3	1.2497919335	0.6951384701	0.9999763851
GUCA1C	-4.2849908816	0.4028453076	0.9999763851
MORC1	5.4770877341	0.3746135179	0.9999763851
DPPA2	0	0	0
DPPA4	0	0	0
NECTIN3	0	0	0
CD96	1.3280783098	0.1538805362	0.9999763851
PLCXD2	-3.3275701064	0.2301333816	0.9999763851
PHLDB2	0	0	0
ABHD10	1.1996908227	0.3461019033	0.9999763851
SLC9C1	5.4776292235	0.3745831165	0.9999763851
BTLA	-1.3158816768	0.8203229797	0.9999763851
ATG3	1.2067566644	0.3131054586	0.9999763851
SLC35A5	-1.1471946769	0.6895664555	0.9999763851
GTPBP8	-1.1901389799	0.4768102251	0.9999763851
NEPRO	-1.0424583423	0.7924047644	0.9999763851
LOC107986113	0	0	0
CFAP44	1.0986161065	0.8500854026	0.9999763851
SPICE1	-1.3149226732	0.6056882455	0.9999763851
SIDT1	-1.3154339304	0.8205576571	0.9999763851
USF3	-1.2483957135	0.589932625	0.9999763851
NAA50	-1.029028059	0.8480829196	0.9999763851
ATP6V1A	1.1213851828	0.5049916193	0.9999763851
ZDHHC23	1.3612513664	0.567051184	0.9999763851
CCDC191	1.1338327296	0.8700834379	0.9999763851
QTRT2	1.0752573285	0.7653207601	0.9999763851
DRD3	0	0	0
ZBTB20	-1.5352362153	0.3730103917	0.9999763851
IGSF11	0	0	0
B4GALT4	1.0344040943	0.9256505017	0.9999763851
ARHGAP31	-1.04266864	0.8914542338	0.9999763851
TMEM39A	1.0558861887	0.8335660469	0.9999763851
POGLUT1	-1.0375027647	0.8633055254	0.9999763851
TIMMDC1	1.0354266142	0.8270546839	0.9999763851
POPDC2	-4.2849908816	0.4028453076	0.9999763851
COX17	-1.1591526319	0.3560511583	0.9999763851
NR1I2	5.4776292235	0.3745831165	0.9999763851
GSK3B	-1.0913111269	0.6286214537	0.9999763851
GPR156	-2.0621986833	0.3113130119	0.9999763851
LRRC58	1.0922279939	0.6373266579	0.9999763851
NDUFB4	1.1544829114	0.3671804974	0.9999763851
HGD	-2.3222877795	0.4503451913	0.9999763851
RABL3	1.3551844819	0.2129861142	0.9999763851
GTF2E1	1.2314720159	0.4066519983	0.9999763851
STXBP5L	-1.8141758814	0.1955903771	0.9999763851
POLQ	1.1865803144	0.3321813005	0.9999763851
ARGFX	0	0	0
FBXO40	0	0	0
HCLS1	-1.1975317537	0.3144297203	0.9999763851

GOLGB1	-1.364060152	0.1669265564	0.9999763851
IQCB1	1.4605806774	0.0747851665	0.9999763851
EAF2	-1.2382236431	0.371051894	0.9999763851
SLC15A2	-2.0436668011	0.1732534927	0.9999763851
ILDR1	-1.8156851364	0.4778610011	0.9999763851
CD86	-3.3292136073	0.2379956236	0.9999763851
CASR	1.2867653957	0.8301799774	0.9999763851
CSTA	1.1763287397	0.2789094277	0.9999763851
CCDC58	-1.2451012674	0.3933266204	0.9999763851
FAM162A	-1.418614005	0.0405247107	0.9999763851
WDR5B	-1.5839476518	0.3957262794	0.9999763851
KPNA1	1.3584532803	0.1302371068	0.9999763851
PARP9	1.1244495853	0.7324308501	0.9999763851
DTX3L	1.0136044232	0.9410879452	0.9999763851
PARP14	-1.3806837706	0.2038033385	0.9999763851
HSPBAP1	-2.0856679879	0.0822658967	0.9999763851
DIRC2	1.1978957411	0.7182803215	0.9999763851
PDIA5	1.0343358777	0.9022552323	0.9999763851
SEC22A	-1.3178798201	0.2921924024	0.9999763851
ADCY5	0	0	0
HACD2	1.8992761065	0.0432932506	0.9999763851
MYLK	-2.2165521716	0.2490218059	0.9999763851
CCDC14	-1.2821340801	0.2870973127	0.9999763851
KALRN	0	0	0
UMPS	-1.0449918993	0.811453882	0.9999763851
ITGB5	-1.9345822491	0.0714793578	0.9999763851
HEG1	-1.2987563215	0.4604260968	0.9999763851
SLC12A8	-5.7100371403	0.0616082413	0.9999763851
ZNF148	-1.1150045063	0.5639239983	0.9999763851
SNX4	-1.3367762236	0.1520496104	0.9999763851
OSBPL11	1.0145080908	0.9817435881	0.9999763851
LOC105374312	-5.3392819425	0.0876453287	0.9999763851
ALG1L	1.9200533293	0.0254666832	0.9999763851
SLC41A3	-1.3313219867	0.3308205911	0.9999763851
ALDH1L1	7.0806012919	0.0454991489	0.9999763851
KLF15	2.5193387991	0.2487840621	0.9999763851
CFAP100	7.4933530102	0.2988796184	0.9999763851
LOC105374089	3.2361532718	0.5546934395	0.9999763851
ZXDC	-1.4433762618	0.0582713305	0.9999763851
UROC1	1.0021081365	0.994793735	0.9999763851
CHST13	1.193773795	0.5217511196	0.9999763851
C3orf22	0	0	0
TXNRD3NB	1.9041554506	0.0443729241	0.9999763851
TXNRD3	-2.8993041198	0.1243070475	0.9999763851
CHCHD6	-1.123333201	0.7310221809	0.9999763851
PLXNA1	1.2850063457	0.3656600284	0.9999763851
TPRA1	1.0897055243	0.696900668	0.9999763851
MCM2	1.0032946365	0.9797291721	0.9999763851
PODXL2	-1.7352888029	0.3006003262	0.9999763851

ABTB1	1.4979084202	0.3744068123	0.9999763851
MGLL	-1.0288739368	0.8730314715	0.9999763851
SEC61A1	-1.3613514316	0.2141122329	0.9999763851
RUVBL1	1.5091163311	0.006531	0.9999763851
EEFSEC	-1.043499337	0.9016183649	0.9999763851
GATA2	-1.1960111917	0.7988338311	0.9999763851
RPN1	-1.889485087	6.15E-05	0.1067663916
RAB7A	1.1416529658	0.2978133377	0.9999763851
ACAD9	1.5001230251	0.0349171674	0.9999763851
KIAA1257	-1.329606002	0.2995934432	0.9999763851
EFCC1	3.2361532718	0.5546934395	0.9999763851
RAB43	-1.1754554033	0.8029741723	0.9999763851
ISY1-RAB43	-1.9389267434	0.0582569698	0.9999763851
ISY1	1.5596843249	0.026964423	0.9999763851
CNBP	1.1533170375	0.3805088729	0.9999763851
COPG1	1.2715838875	0.3820797756	0.9999763851
HMCES	-1.1817352051	0.439209796	0.9999763851
H1FX	1.0703540063	0.6869977005	0.9999763851
EFCAB12	3.2361532718	0.5546934395	0.9999763851
MBD4	-1.2057059345	0.3256494347	0.9999763851
IFT122	-1.1004004339	0.7633567856	0.9999763851
RHO	0	0	0
H1FOO	-4.287394647	0.4026306792	0.9999763851
PLXND1	-1.0908978969	0.6386477162	0.9999763851
TMCC1	1.0758950116	0.8118089378	0.9999763851
ALG1L2	-1.9507065805	0.3140694428	0.9999763851
COL6A6	0	0	0
PIK3R4	-1.1384797168	0.7089230503	0.9999763851
ATP2C1	-1.3173035742	0.196566555	0.9999763851
ASTE1	2.8283024368	0.1159001738	0.9999763851
NEK11	1.1330495498	0.8752853443	0.9999763851
NUDT16	-1.2790937307	0.459461889	0.9999763851
MRPL3	1.0877945579	0.5511625589	0.9999763851
CPNE4	1.133559305	0.8722117747	0.9999763851
ACPP	1.2874012553	0.8191714702	0.9999763851
DNAJC13	1.7008710294	0.1181825924	0.9999763851
ACAD11	1.0421519038	0.9130657926	0.9999763851
UBA5	1.4485955787	0.1556588297	0.9999763851
NPHP3	1.2261550526	0.6297653084	0.9999763851
BFSP2	0	0	0
CDV3	-1.2095416571	0.1468645937	0.9999763851
TOPBP1	-1.000571662	0.9974872961	0.9999763851
SRPRB	1.1203203707	0.4407196439	0.9999763851
RAB6B	-1.4154271261	0.6105519133	0.9999763851
RYK	-1.4196169208	0.1985608003	0.9999763851
ANAPC13	1.2937699588	0.2322744005	0.9999763851
CEP63	-1.1206833447	0.6919882854	0.9999763851
KY	5.4770877341	0.3746135179	0.9999763851
EPHB1	3.2356557963	0.5547505991	0.9999763851

PPP2R3A	3.2353357581	0.554787377	0.9999763851
MSL2	-1.0290040451	0.8948327409	0.9999763851
PCCB	-1.3703195315	0.1804244061	0.9999763851
STAG1	-1.0990950867	0.6835688835	0.9999763851
SLC35G2	-1.0115279726	0.9793242514	0.9999763851
NCK1	1.325472434	0.3244849119	0.9999763851
CLDN18	3.0263279955	0.3115123768	0.9999763851
DZIP1L	-1.1182596865	0.8416090494	0.9999763851
DBR1	1.36433666	0.3165024618	0.9999763851
ARMC8	-1.1206637347	0.6995450574	0.9999763851
NME9	2.2039386922	0.0871165417	0.9999763851
MRAS	1.1215631341	0.736840376	0.9999763851
ESYT3	3.2361532718	0.5546934395	0.9999763851
CEP70	-2.3214980365	0.4340847305	0.9999763851
FAIM	0	0	0
PIK3CB	-1.0044235021	0.98014716	0.9999763851
MRPS22	3.0817853726	0.0812207485	0.9999763851
COPB2	-1.0905174599	0.5833727256	0.9999763851
RBP1	0	0	0
NMNAT3	1.1586670295	0.6787876709	0.9999763851
CLSTN2	7.7488609396	0.3060188822	0.9999763851
SLC25A36	-1.1477604482	0.4262419206	0.9999763851
PXYLP1	1.0954376344	0.8124072421	0.9999763851
ZBTB38	1.0783381642	0.8865784378	0.9999763851
RASA2	-1.0031005329	0.9909261704	0.9999763851
RNF7	1.5115371712	0.011745776	0.9999763851
ATP1B3	1.1065199984	0.4558271206	0.9999763851
TFDP2	-1.0032631199	0.989578547	0.9999763851
GK5	-1.2491605722	0.5052323166	0.9999763851
XRN1	-1.0079813763	0.973906207	0.9999763851
ATR	1.1163191522	0.7491071181	0.9999763851
PLS1	2.9500515526	0.0661112868	0.9999763851
TRPC1	-10.183182718	0.0189055694	0.9999763851
PCOLCE2	1.015287222	0.9606934461	0.9999763851
U2SURP	1.1699132347	0.2551432936	0.9999763851
CHST2	-1.2766016143	0.2113230031	0.9999763851
SLC9A9	1.3140287705	0.2896217013	0.9999763851
C3orf58	-1.4175084152	0.1762733961	0.9999763851
PLSCR2	-4.2864494036	0.4027150576	0.9999763851
PLSCR1	1.4160330642	0.3696201134	0.9999763851
AGTR1	0	0	0
GYG1	1.4916053228	0.0230472735	0.9999763851
HLTF	1.2411653478	0.4243373204	0.9999763851
HPS3	-1.0122062592	0.9576506294	0.9999763851
TM4SF18	3.2353357581	0.554787377	0.9999763851
TM4SF1	-20.6759688086	0.0533591965	0.9999763851
WWTR1	1.1334755146	0.7159284901	0.9999763851
COMMD2	1.1169392129	0.5695515988	0.9999763851
ANKUB1	0	0	0

RNF13	-1.0705624897	0.7909327981	0.9999763851
PFN2	-14.1139127893	0.1026741279	0.9999763851
TMEM183B	1.1866562053	0.243979103	0.9999763851
TSC22D2	-1.5040659276	0.1812830095	0.9999763851
SERP1	1.2480385638	0.0783297525	0.9999763851
EIF2A	-1.3491447492	0.2107029877	0.9999763851
SELT	-1.1466450818	0.3333040997	0.9999763851
ERICH6	3.2353357581	0.554787377	0.9999763851
SIAH2	-1.193068942	0.3118797431	0.9999763851
MED12L	1.03080245	0.9607462247	0.9999763851
GPR171	-4.2849908816	0.4028453076	0.9999763851
P2RY14	3.2361532718	0.5546934395	0.9999763851
IGSF10	1.0739559714	0.8227247301	0.9999763851
AADAC	1.287203384	0.8307715124	0.9999763851
SUCNR1	1.1828012293	0.2904246611	0.9999763851
MBNL1	-1.0816252979	0.5449949587	0.9999763851
RAP2B	-1.0646947091	0.6899067043	0.9999763851
DHX36	1.2891496731	0.3239619076	0.9999763851
GPR149	3.2353357581	0.554787377	0.9999763851
C3orf33	-1.4368678031	0.4210711557	0.9999763851
SLC33A1	-1.1014309721	0.7431639924	0.9999763851
GMPS	1.0778815819	0.6460767614	0.9999763851
KCNAB1	1.266669579	0.4830414795	0.9999763851
SSR3	1.0031182999	0.9802115072	0.9999763851
TIPARP	1.121767671	0.6120785446	0.9999763851
LEKR1	-1.1935389971	0.5020187721	0.9999763851
CCNL1	1.0844142186	0.7118782283	0.9999763851
VEPH1	-2.237494204	0.2906043448	0.9999763851
PTX3	-2.5458658279	0.1259180064	0.9999763851
SHOX2	-1.4285626083	0.2374738367	0.9999763851
RSRC1	1.2213200779	0.3659981823	0.9999763851
MLF1	-8.342239448	0.2161333573	0.9999763851
GFM1	1.044277047	0.7945210606	0.9999763851
MFSD1	1.4222314307	0.0411856249	0.9999763851
SCHIP1	-1.3740925027	0.4403496329	0.9999763851
IL12A	7.7211724672	0.2779709379	0.9999763851
C3orf80	-1.1773255455	0.5368400348	0.9999763851
IFT80	1.176064011	0.6340745022	0.9999763851
SMC4	1.1073248362	0.4071640736	0.9999763851
TRIM59	1.039782197	0.8555909553	0.9999763851
KPNA4	1.0626062953	0.7526470014	0.9999763851
PPM1L	-1.3867362463	0.41941401	0.9999763851
NMD3	-1.1785338781	0.3598328091	0.9999763851
WDR49	-4.2849908816	0.4028453076	0.9999763851
PDCD10	1.5868598332	0.0212106476	0.9999763851
SERPINI1	-2.6622871132	0.2012933849	0.9999763851
GOLIM4	-1.0831537958	0.714500085	0.9999763851
MECOM	-1.1354342752	0.4132114098	0.9999763851
ACTRT3	1.62522264	0.574692917	0.9999763851

MYNN	1.2525246941	0.5371126958	0.9999763851
LRRC34	3.2353357581	0.554787377	0.9999763851
SAMD7	0	0	0
SEC62	-1.0738961241	0.6228552324	0.9999763851
GPR160	1.3872744966	0.0519239027	0.9999763851
PHC3	-1.4869040765	0.0487453151	0.9999763851
PRKCI	-1.0399940248	0.9309503545	0.9999763851
SKIL	-1.3405418309	0.4923153716	0.9999763851
RPL22L1	1.2295312824	0.1628626064	0.9999763851
EIF5A2	1.5243873974	0.3198487405	0.9999763851
TNIK	-1.30841373	0.1515852228	0.9999763851
PLD1	1.0598758974	0.8008150816	0.9999763851
TMEM212	0	0	0
FNDC3B	-1.186582602	0.3294220045	0.9999763851
TNFSF10	1.012169218	0.9748507091	0.9999763851
NCEH1	-1.5412829136	0.4023437467	0.9999763851
ECT2	1.0407242035	0.8542573809	0.9999763851
NLGN1	-1.315303824	0.8206171563	0.9999763851
NAALADL2	3.2353357581	0.554787377	0.9999763851
TBL1XR1	-1.1941899888	0.2452560275	0.9999763851
ZMAT3	-1.1573184836	0.7542163964	0.9999763851
PIK3CA	-1.0988824076	0.7458773327	0.9999763851
KCNMB3	1.9656656116	0.3707760737	0.9999763851
ZNF639	-1.1822404165	0.5781405411	0.9999763851
MFN1	-1.2774889986	0.3888802003	0.9999763851
GNB4	1.3072620977	0.115035686	0.9999763851
ACTL6A	1.2852637101	0.1888240541	0.9999763851
MRPL47	1.3908277742	0.1720275416	0.9999763851
NDUFB5	-1.1469082456	0.4251682611	0.9999763851
USP13	-1.0921580628	0.6582349969	0.9999763851
PEX5L	-1.9659101454	0.4317898946	0.9999763851
TTC14	1.2351177415	0.3909414474	0.9999763851
FXR1	1.0250748735	0.9109887346	0.9999763851
DNAJC19	1.0343371087	0.8698879757	0.9999763851
ATP11B	-1.0134592009	0.9537656599	0.9999763851
DCUN1D1	1.0678398268	0.7957640667	0.9999763851
MCCC1	-1.101569338	0.6518870931	0.9999763851
LAMP3	0	0	0
MCF2L2	2.1957575791	0.307600681	0.9999763851
B3GNT5	1.1352440294	0.5624485733	0.9999763851
KLHL6	1.1028784299	0.8243955637	0.9999763851
KLHL24	-1.636255078	0.0689647976	0.9999763851
YEATS2	-1.7284583682	0.029756778	0.9999763851
MAP6D1	-2.7902229124	0.0442875052	0.9999763851
PARL	-1.0668614564	0.6852256418	0.9999763851
ABCC5	-1.0502418533	0.8323059812	0.9999763851
EIF2B5	-1.3384084533	0.0659253294	0.9999763851
DVL3	-1.7586402079	0.006934	0.9999763851
AP2M1	1.2252323556	0.1759188177	0.9999763851

ABCF3	-1.0276468447	0.9457563316	0.9999763851
ALG3	1.1381009579	0.4609074001	0.9999763851
ECE2	1.6353343936	0.0428548569	0.9999763851
CAMK2N2	-1.4347038381	0.3393964851	0.9999763851
PSMD2	1.030245285	0.8610167877	0.9999763851
EIF4G1	1.0204520903	0.878519736	0.9999763851
CLCN2	1.8742558907	0.2632551888	0.9999763851
POLR2H	-1.2823382844	0.1178527899	0.9999763851
CHRD	0	0	0
EPHB3	9.9312452783	0.2485677018	0.9999763851
MAGEF1	-1.0969243478	0.7325117665	0.9999763851
VPS8	-1.3055160532	0.2489096725	0.9999763851
C3orf70	-1.315303824	0.8206171563	0.9999763851
EHHADH	-1.2562050205	0.7082883295	0.9999763851
MAP3K13	-1.1142281527	0.5627279432	0.9999763851
TMEM41A	-1.0972966182	0.734342475	0.9999763851
SENP2	1.1891614677	0.5408643692	0.9999763851
IGF2BP2	-1.1600855189	0.5748796538	0.9999763851
TRA2B	1.1231176457	0.4382280626	0.9999763851
ETV5	-1.1521590587	0.3433829977	0.9999763851
DGKG	-2.1421506715	0.005164	0.9999763851
CRYGS	-7.5664585246	0.2265122542	0.9999763851
TBCCD1	1.1997581502	0.6805700871	0.9999763851
DNAJB11	-1.0589844835	0.7322090031	0.9999763851
HRG	0	0	0
KNG1	3.2361532718	0.5546934395	0.9999763851
EIF4A2	-1.3590588899	0.0433253619	0.9999763851
RFC4	-1.029944895	0.9121645386	0.9999763851
ST6GAL1	-1.0769217109	0.613285779	0.9999763851
RPL39L	-1.0233066419	0.9368943067	0.9999763851
BCL6	-1.7096752905	0.1029711582	0.9999763851
LPP	-1.3594690425	0.1796405858	0.9999763851
TP63	0	0	0
CLDN16	0	0	0
TMEM207	0	0	0
IL1RAP	1.2005852893	0.6046810427	0.9999763851
UTS2B	0	0	0
FGF12	0	0	0
MB21D2	1.2321174685	0.6710419103	0.9999763851
ATP13A4	1.0271815929	0.978028051	0.9999763851
OPA1	-1.0590971327	0.7642667327	0.9999763851
HES1	-13.1418879802	0.000152	0.2098020317
ATP13A3	1.2003249358	0.2994160788	0.9999763851
TMEM44	2.1124281032	0.1409457607	0.9999763851
LSG1	-1.1598792625	0.4208147841	0.9999763851
FAM43A	-1.3120672656	0.1291656231	0.9999763851
XXYLT1	-1.0331871839	0.8762068886	0.9999763851
ACAP2	1.1732183574	0.4030260011	0.9999763851
PPP1R2	1.0620475561	0.7886863846	0.9999763851

MUC20	1.4701226961	0.6325676268	0.9999763851
TNK2	1.0430366211	0.831492427	0.9999763851
LOC105374301	0	0	0
TFRC	1.2755563165	0.0555491828	0.9999763851
SLC51A	-1.2944792306	0.6141973559	0.9999763851
PCYT1A	1.1227331014	0.8019848471	0.9999763851
TCTEX1D2	1.3898325242	0.2021808174	0.9999763851
TM4SF19	-5.1487074295	0.003751	0.9999763851
UBXN7	-1.3785889735	0.0451035592	0.9999763851
RNF168	1.1934114606	0.4951632227	0.9999763851
SMCO1	3.2356557963	0.5547505991	0.9999763851
WDR53	1.0737815799	0.8607630547	0.9999763851
FBXO45	-1.0203955058	0.9069217783	0.9999763851
NRROS	-1.6244294592	0.0578579901	0.9999763851
CEP19	-1.2942163544	0.5121840697	0.9999763851
PIGX	-1.1644522501	0.4389351766	0.9999763851
PAK2	-1.0007072127	0.996346109	0.9999763851
SENP5	-1.061542734	0.7831165342	0.9999763851
NCBP2	1.0344049594	0.8640970697	0.9999763851
PIGZ	-1.6914686964	0.1719905867	0.9999763851
MELTF	-1.3381931295	0.1455118097	0.9999763851
DLG1	-1.0832744062	0.7179523204	0.9999763851
BDH1	1.216484259	0.2257162803	0.9999763851
RUBCN	-1.4213561069	0.1591309257	0.9999763851
FYTTD1	1.1521391975	0.4291728741	0.9999763851
LRCH3	-1.1538483885	0.5987491325	0.9999763851
IQCG	-1.05311448	0.8495209267	0.9999763851
RPL35A	-1.106422405	0.3859488717	0.9999763851
LMLN	1.6105560359	0.1658522727	0.9999763851
FAM157A	-4.7792357159	0.0182542221	0.9999763851
ZNF718	0	0	0
ZNF732	0	0	0
ZNF141	1.1264088673	0.3321538719	0.9999763851
ZNF721	-1.2705567171	0.503971729	0.9999763851
PIGG	1.1760872649	0.5968489971	0.9999763851
PDE6B	-1.315303824	0.8206171563	0.9999763851
ATP5I	1.0180072294	0.9028366698	0.9999763851
MYL5	-2.7217225437	0.0141203559	0.9999763851
MFSD7	0	0	0
PCGF3	-1.3527976385	0.0550932875	0.9999763851
CPLX1	0	0	0
GAK	1.2247927757	0.222768965	0.9999763851
TMEM175	1.1414405581	0.7946078199	0.9999763851
DGKQ	-1.0010232133	0.9970016742	0.9999763851
SLC26A1	1.1329115851	0.8742573041	0.9999763851
IDUA	1.8888297566	0.593759522	0.9999763851
FGFRL1	1.2630517183	0.4944535967	0.9999763851
SPON2	-1.2823592232	0.1829867057	0.9999763851
CTBP1	-1.0338563786	0.7988251259	0.9999763851

MAEA	1.4620014463	0.0440905242	0.9999763851
UVSSA	-1.9881850467	0.1813232557	0.9999763851
CRIPAK	-1.4349511833	0.4484662141	0.9999763851
FAM53A	-1.5697725216	0.2307806184	0.9999763851
SLBP	1.0268424454	0.8889614077	0.9999763851
TMEM129	-1.2134925058	0.2325885259	0.9999763851
TACC3	1.1129078934	0.4531742802	0.9999763851
FGFR3	9.9624059448	0.2315386396	0.9999763851
LETM1	1.0000573328	0.999733374	0.9999763851
WHSC1	-1.0820767385	0.6094193642	0.9999763851
NELFA	-1.2494520054	0.1591542191	0.9999763851
C4orf48	1.0876136086	0.8466977609	0.9999763851
NAT8L	-1.0866106505	0.7985619139	0.9999763851
POLN	1.5810707843	0.2137478199	0.9999763851
HAUS3	1.9298099233	0.2579707582	0.9999763851
MXD4	-1.1825399502	0.565673035	0.9999763851
ZFYVE28	-1.0485303181	0.8957156613	0.9999763851
CFAP99	0	0	0
RNF4	-1.2341676555	0.4532977448	0.9999763851
FAM193A	1.3731783823	0.23345049	0.9999763851
TNIP2	1.5040785766	0.1541308447	0.9999763851
SH3BP2	1.2770137422	0.2211383312	0.9999763851
ADD1	1.0956544562	0.5659602973	0.9999763851
MFSD10	1.0557483755	0.6696105047	0.9999763851
NOP14	-1.0687504927	0.6653018209	0.9999763851
GRK4	1.4693815047	0.3916238203	0.9999763851
HTT	-1.2426012352	0.2009990566	0.9999763851
MSANTD1	-1.6817502083	0.4746188271	0.9999763851
RGS12	-1.2443191648	0.3731727382	0.9999763851
LRPAP1	1.1870726488	0.1694177919	0.9999763851
ADRA2C	-1.315303824	0.8206171563	0.9999763851
TMEM128	1.2336156806	0.6360815995	0.9999763851
LYAR	1.0634526933	0.7140384016	0.9999763851
ZBTB49	-1.085116324	0.8529919374	0.9999763851
NSG1	-4.2864494036	0.4027150576	0.9999763851
STX18	1.2850675239	0.3721312582	0.9999763851
MSX1	1.0325803783	0.9229054902	0.9999763851
CYTL1	-1.0361486706	0.9389324599	0.9999763851
STK32B	-3.3272796812	0.2460356765	0.9999763851
EVC2	0	0	0
C4orf50	-2.5767557147	0.3126719162	0.9999763851
WFS1	0	0	0
PPP2R2C	-1.6268327642	0.2591915247	0.9999763851
MAN2B2	1.1650254167	0.3460393532	0.9999763851
MRFAP1	1.0727636309	0.6243406112	0.9999763851
S100P	1.1836417259	0.1878709638	0.9999763851
MRFAP1L1	1.3237198661	0.0784371357	0.9999763851
BLOC1S4	-1.1567051375	0.4875607336	0.9999763851
KIAA0232	-1.2797987247	0.3763859493	0.9999763851

TBC1D14	1.1772628259	0.3291238858	0.9999763851
CCDC96	6.368054416	0.3491474795	0.9999763851
TADA2B	1.1392776459	0.6517671668	0.9999763851
GRPEL1	1.1638151777	0.3916176955	0.9999763851
SORCS2	1.0463114448	0.8471144732	0.9999763851
AFAP1	3.2356557963	0.5547505991	0.9999763851
ABLIM2	3.6908783983	0.155915947	0.9999763851
SH3TC1	-2.3367314853	0.0123156375	0.9999763851
HTRA3	5.4776292235	0.3745831165	0.9999763851
ACOX3	-1.4161766541	0.209930935	0.9999763851
TRMT44	1.9562973384	0.1408713812	0.9999763851
HMX1	3.2356557963	0.5547505991	0.9999763851
FAM86KP	0	0	0
SLC2A9	-1.2422489176	0.4410732374	0.9999763851
WDR1	1.1012128055	0.4818630942	0.9999763851
ZNF518B	-1.7724666178	0.2815466456	0.9999763851
CLNK	0	0	0
RAB28	-1.25527926	0.3015839939	0.9999763851
NKX3-2	0	0	0
BOD1L1	1.010821934	0.9544491091	0.9999763851
CPEB2	-1.7410046481	0.157277291	0.9999763851
C1QTNF7	1.9668088044	0.3527516592	0.9999763851
CC2D2A	1.5100940589	0.5369787292	0.9999763851
FBXL5	1.1717067714	0.4154238468	0.9999763851
FAM200B	1.5176220905	0.2974567623	0.9999763851
BST1	-1.107597204	0.6321073823	0.9999763851
CD38	1.2202990301	0.2994968427	0.9999763851
FGFBP2	-1.082138348	0.7500888453	0.9999763851
PROM1	-1.7994722457	0.518998306	0.9999763851
TAPT1	-1.1587327924	0.4504971733	0.9999763851
QDPR	1.1327645257	0.5241970159	0.9999763851
LAP3	1.0186243512	0.9406683522	0.9999763851
MED28	-1.028441362	0.8699929784	0.9999763851
FAM184B	3.0102857067	0.108772236	0.9999763851
DCAF16	-1.0549102842	0.7512258104	0.9999763851
NCAPG	-1.0639280345	0.7504094943	0.9999763851
LCORL	-1.4910940494	0.0734346397	0.9999763851
PACRGL	1.7579989224	0.4436784171	0.9999763851
ADGRA3	1.0318771274	0.9421514932	0.9999763851
PPARGC1A	-2.1007484104	0.002553	0.9999763851
DHX15	1.2938164663	0.0711236451	0.9999763851
CCDC149	-1.3158816768	0.8203229797	0.9999763851
SEPSECS	-1.1374647481	0.8003136121	0.9999763851
PI4K2B	-1.0550790696	0.8220284989	0.9999763851
ZCCHC4	-1.0628052168	0.8947986973	0.9999763851
ANAPC4	1.0480911223	0.8509241908	0.9999763851
SEL1L3	-1.8455485512	0.0198729108	0.9999763851
SMIM20	1.2988912559	0.5035502254	0.9999763851
RBPJ	-1.1501262422	0.3568885261	0.9999763851

TBC1D19	-1.3955017315	0.6890604737	0.9999763851
STIM2	-1.4188455255	0.1787927395	0.9999763851
PCDH7	-1.3158816768	0.8203229797	0.9999763851
ARAP2	1.3659539746	0.4785017248	0.9999763851
DTHD1	0	0	0
C4orf19	-1.3375066151	0.5148613622	0.9999763851
RELL1	1.5915896242	0.0252829066	0.9999763851
PGM2	-1.0738016629	0.7424676907	0.9999763851
TBC1D1	-1.1208674434	0.4882228262	0.9999763851
PTTG2	-2.3214980365	0.434695234	0.9999763851
KLF3	-1.2874031024	0.436757811	0.9999763851
TLR1	-7.5647903763	0.2176246281	0.9999763851
FAM114A1	-1.0546608592	0.9396385026	0.9999763851
TMEM156	1.4845744246	0.5084253436	0.9999763851
KLHL5	1.0085894724	0.9714825084	0.9999763851
WDR19	-1.3199842814	0.5569193553	0.9999763851
RFC1	-1.0504740903	0.7470811106	0.9999763851
KLB	-4.287394647	0.4026306792	0.9999763851
RPL9	1.0360877857	0.807099414	0.9999763851
LIAS	-1.3366791839	0.3096603661	0.9999763851
UGDH	1.1022334604	0.7447771372	0.9999763851
SMIM14	-1.4865693181	0.1906690922	0.9999763851
UBE2K	-1.0759389286	0.6531413881	0.9999763851
PDS5A	-1.4348399905	0.012791086	0.9999763851
N4BP2	1.3139349046	0.5244614682	0.9999763851
RHOH	-1.6218880674	0.1070363003	0.9999763851
RBM47	1.0433636009	0.8769365528	0.9999763851
NSUN7	-7.5664585246	0.2264603451	0.9999763851
APBB2	1.1726446997	0.4911039614	0.9999763851
LIMCH1	0	0	0
TMEM33	1.1394892856	0.4086242246	0.9999763851
DCAF4L1	-3.5156187543	0.1697255493	0.9999763851
SLC30A9	1.2029382445	0.436962168	0.9999763851
ATP8A1	-1.560273095	0.2229754011	0.9999763851
YIPF7	-4.2849908816	0.4028453076	0.9999763851
GUF1	2.282706334	0.0125693986	0.9999763851
GNPDA2	2.7408203244	0.1806493815	0.9999763851
GABRB1	-4.2849908816	0.4028453076	0.9999763851
COMMD8	1.4383799478	0.1698610411	0.9999763851
ATP10D	1.1735024689	0.7592627571	0.9999763851
CORIN	1.8140611791	0.5846585145	0.9999763851
NFXL1	-1.2595156937	0.4879085401	0.9999763851
TXK	-3.1552159335	0.1779442196	0.9999763851
TEC	1.6245095224	0.5657710166	0.9999763851
SLAIN2	1.0117354529	0.9624421511	0.9999763851
FRYL	1.0003405083	0.9985850864	0.9999763851
OCIAD1	-1.2445861868	0.1079809318	0.9999763851
DCUN1D4	1.1127324824	0.7334314148	0.9999763851
USP46	1.1337334788	0.8709612797	0.9999763851

RASL11B	-1.315303824	0.8206171563	0.9999763851
SCFD2	1.0352267643	0.9145642358	0.9999763851
FIP1L1	-1.2162236182	0.2801527742	0.9999763851
LNX1	1.2800950129	0.6390483545	0.9999763851
CHIC2	-1.5345073782	0.1656900135	0.9999763851
KIT	1.3257077688	0.7618555768	0.9999763851
KDR	0	0	0
SRD5A3	1.2326051615	0.2608156415	0.9999763851
TMEM165	1.1911375557	0.3989151165	0.9999763851
CLOCK	-1.0136140271	0.983743704	0.9999763851
EXOC1	1.3049558338	0.3278150342	0.9999763851
CEP135	1.1878095634	0.6238044249	0.9999763851
AASDH	-1.2403869811	0.4373511546	0.9999763851
PPAT	-1.083581999	0.7360581786	0.9999763851
PAICS	1.2790418563	0.0787847729	0.9999763851
SRP72	1.1878680166	0.1863320189	0.9999763851
ARL9	0	0	0
REST	1.3166583482	0.2169957535	0.9999763851
NOA1	-1.2890966877	0.3299452005	0.9999763851
POLR2B	-1.1852990369	0.273914481	0.9999763851
IGFBP7	-1.0935440221	0.6856998492	0.9999763851
ADGRL3	-1.1950744547	0.6368039176	0.9999763851
EPHA5	1.068546911	0.6524476546	0.9999763851
CENPC	-1.0921731205	0.7828224972	0.9999763851
STAP1	1.1649666292	0.7558170958	0.9999763851
UBA6	-1.1643901678	0.4727495754	0.9999763851
GNRHR	0	0	0
TMPRSS11D	3.2353357581	0.554787377	0.9999763851
TMPRSS11F	0	0	0
YTHDC1	1.0876836535	0.6025775433	0.9999763851
UGT2A2	-4.2849908816	0.4028453076	0.9999763851
SULT1B1	3.2353357581	0.554787377	0.9999763851
UTP3	1.1171911976	0.5044975576	0.9999763851
RUFY3	-1.470438136	0.297146182	0.9999763851
GRSF1	-1.2509995584	0.1261100335	0.9999763851
MOB1B	-1.3618151029	0.4393753445	0.9999763851
DCK	1.2040053286	0.5182777697	0.9999763851
SLC4A4	0	0	0
COX18	-1.1072678687	0.6856011223	0.9999763851
ANKRD17	1.0610594126	0.7211217424	0.9999763851
CXCL8	-2.1226426784	0.0011	0.8381590036
CXCL6	1.2866381786	0.8225514932	0.9999763851
PF4V1	5.4781708352	0.3745527127	0.9999763851
PPBP	-4.3381560983	0.1571039335	0.9999763851
CXCL5	0	0	0
CXCL3	0	0	0
CXCL2	1.0299009028	0.9680669521	0.9999763851
MTHFD2L	1.4242050291	0.2197531218	0.9999763851
RCHY1	1.3443063725	0.2261165134	0.9999763851

THAP6	-3.1539335425	0.1560971771	0.9999763851
C4orf26	0	0	0
CDKL2	0	0	0
G3BP2	1.1920571081	0.301988519	0.9999763851
USO1	-1.2914170303	0.3077726524	0.9999763851
NAAA	1.0571697919	0.770543817	0.9999763851
SDAD1	1.1171301611	0.6348697452	0.9999763851
ART3	-2.3512645813	0.0806570148	0.9999763851
CXCL10	-1.1067104994	0.7535841099	0.9999763851
CXCL11	-4.2864494036	0.4027150576	0.9999763851
NUP54	-1.1348538065	0.6781680871	0.9999763851
SCARB2	-1.2410951018	0.3072402823	0.9999763851
FAM47E	-1.5416912797	0.6826008231	0.9999763851
FAM47E-STBD1	1.1902374898	0.8038783922	0.9999763851
STBD1	-1.7449096743	0.2147488318	0.9999763851
CCDC158	-5.7918925833	0.1508437595	0.9999763851
SHROOM3	0	0	0
SEPT11	1.2042940426	0.5447857733	0.9999763851
CCNI	1.1396213147	0.3001120811	0.9999763851
CCNG2	-1.5493290245	0.1869844655	0.9999763851
CXCL13	0	0	0
CNOT6L	1.2494804394	0.3805480153	0.9999763851
MRPL1	-1.155042445	0.4327751427	0.9999763851
FRAS1	1.0492709541	0.7843690435	0.9999763851
BMP2K	-1.1352818852	0.5825657649	0.9999763851
PAQR3	-1.0235316757	0.893631629	0.9999763851
NAA11	1.2051753167	0.2519354577	0.9999763851
ANTXR2	-1.0348695615	0.8736248246	0.9999763851
PRDM8	1.2552092302	0.3931284801	0.9999763851
LOC107986215	-1.3154339304	0.8205576571	0.9999763851
HNRNPD	1.1480102407	0.2732581953	0.9999763851
HNRNPDL	1.0698288225	0.5856906499	0.9999763851
ENOPH1	1.1130584483	0.6066227194	0.9999763851
TMEM150C	18.5663370642	5.83E-05	0.1067663916
SCD5	-1.4848881099	0.1400528121	0.9999763851
SEC31A	1.069516196	0.7680668318	0.9999763851
THAP9	2.027155953	0.4565858217	0.9999763851
LIN54	-1.2071175708	0.447080185	0.9999763851
COPS4	1.2106908088	0.4003582732	0.9999763851
PLAC8	-1.2759525062	0.4342237833	0.9999763851
COQ2	-1.2158285418	0.2668167718	0.9999763851
HPSE	1.5501349378	0.3909125046	0.9999763851
HELQ	2.7811444689	0.0480747177	0.9999763851
MRPS18C	-1.1384524923	0.4519739357	0.9999763851
FAM175A	-1.0833082856	0.8045678854	0.9999763851
GPAT3	1.202593002	0.5375763439	0.9999763851
CDS1	-1.3161718537	0.8283869414	0.9999763851
WDFY3	-1.5673900329	0.2105407982	0.9999763851
ARHGAP24	-1.8915159376	0.2432791975	0.9999763851

SLC10A6	0	0	0
C4orf36	7.7219356627	0.2871728309	0.9999763851
AFF1	1.0328722823	0.845197565	0.9999763851
KLHL8	1.0671989501	0.7980100737	0.9999763851
HSD17B13	-2.5600051511	0.2551953091	0.9999763851
HSD17B11	1.0001160746	0.9996121197	0.9999763851
NUDT9	1.3710545995	0.2990601602	0.9999763851
SPARCL1	0	0	0
SPP1	-16.7128947179	0	0
PKD2	1.2018106325	0.6260982749	0.9999763851
PPM1K	-1.2658500736	0.3573069364	0.9999763851
HERC6	-4.7486078443	0.0859010109	0.9999763851
HERC5	-2.1074911394	0.0553427893	0.9999763851
PIGY	1.5226701091	0.055863487	0.9999763851
PYURF	1.0462207029	0.8199302568	0.9999763851
HERC3	-1.3209946069	0.5352547278	0.9999763851
FAM13A	-1.1490595167	0.8521121188	0.9999763851
TIGD2	1.6427550276	0.3265931446	0.9999763851
GPRIN3	-1.1491729646	0.8479881239	0.9999763851
CCSER1	-1.3199491909	0.0790718867	0.9999763851
GRID2	3.2356557963	0.5547505991	0.9999763851
SMARCAD1	1.2186632612	0.4506048862	0.9999763851
HPGDS	0	0	0
PDLIM5	1.0147533103	0.9478754046	0.9999763851
UNC5C	-1.2271284135	0.530077268	0.9999763851
STPG2	1.0754508071	0.926308326	0.9999763851
RAP1GDS1	1.573611224	0.009066	0.9999763851
TSPAN5	-1.0982545278	0.7119501816	0.9999763851
EIF4E	1.1171100019	0.4669030604	0.9999763851
METAP1	1.0823874698	0.6635511912	0.9999763851
ADH5	-1.2813552179	0.1889398215	0.9999763851
TRMT10A	1.0984285422	0.8461403166	0.9999763851
DAPP1	-1.331698876	0.6378999261	0.9999763851
LAMTOR3	1.2140064845	0.5307303547	0.9999763851
DNAJB14	1.1161396164	0.703527385	0.9999763851
H2AFZ	-1.1518569535	0.2200050751	0.9999763851
EMCN	0	0	0
PPP3CA	-1.0837749694	0.6335918541	0.9999763851
BANK1	0	0	0
SLC39A8	1.422515618	0.0638353265	0.9999763851
NFKB1	1.2556376591	0.2039447703	0.9999763851
MANBA	-1.647097176	0.0180444493	0.9999763851
UBE2D3	-1.1299094468	0.30702579	0.9999763851
CISD2	-1.1096324395	0.5345519789	0.9999763851
SLC9B1	-2.3222877795	0.4504859661	0.9999763851
SLC9B2	1.077543515	0.7672214132	0.9999763851
BDH2	-1.013117539	0.9701845889	0.9999763851
CENPE	1.407373725	0.0708888024	0.9999763851
TACR3	3.2353357581	0.554787377	0.9999763851

TET2	-1.1960569267	0.6353434242	0.9999763851
PPA2	-1.0015539194	0.9925581341	0.9999763851
ARHGEF38	-1.3157515262	0.8225133873	0.9999763851
INTS12	-1.3406620238	0.3127239279	0.9999763851
GSTCD	1.0226914481	0.9437369346	0.9999763851
NPNT	0	0	0
TBCK	-1.2565638732	0.4464824629	0.9999763851
AIMP1	-1.0013722044	0.9935607105	0.9999763851
LOC107986216	0	0	0
PAPSS1	1.030038545	0.896396286	0.9999763851
SGMS2	-1.0356181086	0.9414035386	0.9999763851
CYP2U1	0	0	0
HADH	1.2582845488	0.1484634146	0.9999763851
LEF1	-4.2864494036	0.4027150576	0.9999763851
RPL34	-1.052130748	0.689442608	0.9999763851
OSTC	-1.0379316479	0.8081053725	0.9999763851
SEC24B	1.0928228087	0.7180987483	0.9999763851
CCDC109B	1.1571373806	0.5533393134	0.9999763851
CASP6	1.0853809703	0.6944615366	0.9999763851
PLA2G12A	1.2730221868	0.3544196166	0.9999763851
GAR1	1.2670246486	0.1080875721	0.9999763851
RRH	0	0	0
LRIT3	-4.287394647	0.4026306792	0.9999763851
ELOVL6	0	0	0
C4orf32	1.6822464147	0.163738542	0.9999763851
AP1AR	-1.2232674253	0.4292608793	0.9999763851
TIFA	1.1685062055	0.5938619413	0.9999763851
ALPK1	-1.5014218323	0.2037492959	0.9999763851
ZGRF1	-1.386022444	0.3394022199	0.9999763851
LARP7	1.401158557	0.1090562262	0.9999763851
ANK2	1.7768474191	0.0007473	0.6510516988
CAMK2D	1.1089323737	0.8152634383	0.9999763851
UGT8	6.368054416	0.3491544048	0.9999763851
NDST3	1.2869488883	0.8190393436	0.9999763851
PRSS12	1.3549794337	0.6408840014	0.9999763851
METTL14	1.0625563436	0.8542849361	0.9999763851
SEC24D	-1.1021096772	0.6056123818	0.9999763851
SYNPO2	1.286354513	0.820736494	0.9999763851
MYOZ2	0	0	0
USP53	1.1165323446	0.8615979356	0.9999763851
C4orf3	1.256153715	0.1781326305	0.9999763851
PDE5A	1.1617715381	0.7178733882	0.9999763851
MAD2L1	1.390514629	0.0563123885	0.9999763851
ANXA5	1.080888217	0.6411877792	0.9999763851
EXOSC9	1.0688402276	0.7336745199	0.9999763851
CCNA2	1.0067022434	0.9621296337	0.9999763851
BBS7	-1.0624089353	0.9009500062	0.9999763851
TRPC3	0	0	0
KIAA1109	-1.6192143192	0.1252442052	0.9999763851

BBS12	2.8919248679	0.3275957387	0.9999763851
FGF2	-2.5593885838	0.3085129336	0.9999763851
NUDT6	-2.293109698	0.0921057855	0.9999763851
SPATA5	1.0002073169	0.99944365	0.9999763851
SPRY1	1.9894954061	0.3034692612	0.9999763851
ANKRD50	-1.6253771944	0.5661731301	0.9999763851
FAT4	3.2353357581	0.554787377	0.9999763851
INTU	0	0	0
SLC25A31	0	0	0
HSPA4L	-1.1111893603	0.7045620482	0.9999763851
PLK4	1.3119415123	0.4298756636	0.9999763851
MFSD8	-1.5339127666	0.3597857662	0.9999763851
ABHD18	1.2522910089	0.265633347	0.9999763851
LARP1B	-1.4668342783	0.0964994245	0.9999763851
PGRMC2	1.0229148277	0.9002087051	0.9999763851
JADE1	1.2557321103	0.1974887377	0.9999763851
SCLT1	-1.1068523862	0.6387781806	0.9999763851
C4orf33	-1.0597336095	0.8940004901	0.9999763851
SLC7A11	-1.6032197253	0.0125000975	0.9999763851
NOCT	1.1226966385	0.7977141362	0.9999763851
ELF2	1.0848895405	0.7139498408	0.9999763851
MGARP	5.4784711963	0.3745358536	0.9999763851
NDUFC1	1.3936693387	0.0458513825	0.9999763851
NAA15	-1.2427274364	0.2199414353	0.9999763851
RAB33B	-1.2728224451	0.5872567352	0.9999763851
SETD7	-1.1060288371	0.6023401121	0.9999763851
MGST2	1.1033112629	0.4466290835	0.9999763851
MAML3	-1.22639426	0.4717370387	0.9999763851
SCOC	-1.1248296681	0.6581272647	0.9999763851
CLGN	2.1743121231	0.288220291	0.9999763851
ELMOD2	-1.4522487716	0.352541687	0.9999763851
RNF150	0	0	0
ZNF330	1.0574806748	0.7738404283	0.9999763851
INPP4B	-1.0013292194	0.9946423531	0.9999763851
USP38	-1.2852492726	0.3127201804	0.9999763851
GAB1	-1.2384147505	0.5737159702	0.9999763851
SMARCA5	1.1890487834	0.2807054778	0.9999763851
FREM3	0	0	0
HHIP	5.4776292235	0.3745831165	0.9999763851
ANAPC10	1.6643198842	0.2778787977	0.9999763851
ABCE1	1.1446474202	0.3478345501	0.9999763851
OTUD4	2.0340623597	0.006858	0.9999763851
SMAD1	0	0	0
MMAA	1.9538319373	0.3873463274	0.9999763851
C4orf51	5.4770877341	0.3746135179	0.9999763851
LSM6	-1.3517755293	0.1687594151	0.9999763851
SLC10A7	-1.1772401109	0.6801211517	0.9999763851
TMEM184C	1.0995934927	0.8356547924	0.9999763851
PRMT9	1.2792150955	0.3409378111	0.9999763851

ARHGAP10	-1.229834886	0.7597812191	0.9999763851
NR3C2	-2.3214980365	0.4351998275	0.9999763851
DCLK2	-4.2849908816	0.4028453076	0.9999763851
LRBA	-1.1376206353	0.7126403037	0.9999763851
RPS3A	-1.0858190464	0.5246830263	0.9999763851
SH3D19	9.9614219348	0.2203097956	0.9999763851
GATB	-1.6166137326	0.2594721822	0.9999763851
FBXW7	-1.2376108889	0.2890719164	0.9999763851
TMEM154	-1.0759294333	0.8469343959	0.9999763851
TIGD4	0	0	0
ARFIP1	2.5601600361	0.0710306602	0.9999763851
FHDC1	-1.3977001462	0.4854329951	0.9999763851
MND1	-1.0839831739	0.6826872775	0.9999763851
KIAA0922	-1.4032483146	0.1130334153	0.9999763851
TLR2	-1.8444638303	0.2881950487	0.9999763851
RNF175	0	0	0
SFRP2	0	0	0
PLRG1	-1.1239428	0.637306906	0.9999763851
FGB	5.4790129495	0.3753326899	0.9999763851
RBM46	3.2353357581	0.554787377	0.9999763851
ASIC5	5.4781708352	0.3745527127	0.9999763851
TDO2	-6.1820830156	0.0004033	0.4429482063
CTSO	-1.4336216598	0.5431179172	0.9999763851
GLRB	3.2353357581	0.554787377	0.9999763851
GRIA2	0	0	0
TMEM144	-1.2942500023	0.6174680893	0.9999763851
RXFP1	0	0	0
C4orf46	-1.13185205	0.5212886628	0.9999763851
ETFDH	-1.9154384599	0.0616699513	0.9999763851
PPID	1.0042502086	0.9834845588	0.9999763851
FNIP2	-1.8571734693	0.001165	0.8594669785
C4orf45	6.5213929566	0.0192650197	0.9999763851
RAPGEF2	-1.9857368557	0.1230135899	0.9999763851
FSTL5	0	0	0
NAF1	-1.1561682569	0.5338976513	0.9999763851
TMA16	-1.4350137047	0.0636087362	0.9999763851
MARCH1	-1.2620299418	0.7409721149	0.9999763851
ANP32C	-1.3717838846	0.7498319246	0.9999763851
APELA	0	0	0
TMEM192	1.1684511279	0.4778300295	0.9999763851
KLHL2	-1.3105381531	0.4324501055	0.9999763851
MSMO1	-1.2390044602	0.314149743	0.9999763851
CPE	-2.6603009385	0.1945638402	0.9999763851
SPOCK3	0	0	0
DDX60	1.2815256224	0.7940112845	0.9999763851
DDX60L	-1.4482994521	0.2640877781	0.9999763851
CBR4	-1.5537816711	0.0550063097	0.9999763851
SH3RF1	-1.5561198097	0.1915994258	0.9999763851
NEK1	-1.0448320985	0.9068158747	0.9999763851

CLCN3	-1.2291183436	0.3639434238	0.9999763851
HPF1	-1.204412426	0.3682537872	0.9999763851
MFAP3L	-1.4217670568	0.5902939428	0.9999763851
GALNTL6	5.4770877341	0.3746135179	0.9999763851
GALNT7	1.0901802494	0.70501677	0.9999763851
HMGB2	-1.235449581	0.3058774826	0.9999763851
SAP30	-1.5138803647	0.163723602	0.9999763851
SCRG1	-3.0146678557	0.0810158537	0.9999763851
HAND2	0	0	0
FBXO8	-1.4079846504	0.6793411967	0.9999763851
CEP44	-1.0444677949	0.8938225661	0.9999763851
GPM6A	0	0	0
ASB5	0	0	0
SPCS3	-1.1068315597	0.4561985166	0.9999763851
NEIL3	-1.0819801774	0.7506692006	0.9999763851
AGA	-1.2903424981	0.2583268971	0.9999763851
LOC285500	-1.1966292775	0.7920922107	0.9999763851
TENM3	-1.3709710942	0.7484705277	0.9999763851
DCTD	1.4951146823	0.0614194283	0.9999763851
WWC2	2.8687666286	0.1459114632	0.9999763851
CLDN22	1.349683529	0.6749312302	0.9999763851
LOC107986217	0	0	0
CDKN2AIP	1.1702761325	0.5474426319	0.9999763851
ING2	1.1453428599	0.4792088443	0.9999763851
RWDD4	1.021524123	0.9093606898	0.9999763851
TRAPPC11	-1.0309606102	0.8972493591	0.9999763851
STOX2	1.1747070055	0.5236993701	0.9999763851
ENPP6	0	0	0
IRF2	-1.3759070126	0.4143580367	0.9999763851
CASP3	1.3235654395	0.2802419009	0.9999763851
PRIMPOL	1.5491924319	0.5377707716	0.9999763851
CENPU	1.2233564881	0.2474440965	0.9999763851
ACSL1	1.1139512805	0.5444566976	0.9999763851
CFAP97	1.5372837252	0.0444207809	0.9999763851
SNX25	1.0845209046	0.8701279009	0.9999763851
LRP2BP	1.5489906133	0.5725077297	0.9999763851
ANKRD37	-2.5290144326	0.0834805787	0.9999763851
UFSP2	-1.0163884814	0.9529698758	0.9999763851
C4orf47	-1.3713173847	0.744040857	0.9999763851
FAM149A	0	0	0
CYP4V2	1.208087406	0.3524931907	0.9999763851
KLKB1	1.6257221546	0.5967441092	0.9999763851
F11	5.4790129495	0.3754356866	0.9999763851
FAT1	3.2356557963	0.5547505991	0.9999763851
ZFP42	-4.2849908816	0.4028453076	0.9999763851
FRG1	-1.0710314888	0.6471719935	0.9999763851
CCDC127	1.363881472	0.5474380469	0.9999763851
SDHA	-1.028194942	0.8403896637	0.9999763851
PDCD6	1.1652379177	0.3237611231	0.9999763851

AHRR	-1.1906058445	0.3642471873	0.9999763851
EXOC3	-1.0737605579	0.6813380552	0.9999763851
SLC9A3	-1.1016049459	0.7710587737	0.9999763851
CEP72	1.5470780217	0.1582432918	0.9999763851
LOC101929895	9.9619682616	0.2312787469	0.9999763851
ZDHHC11	3.1165674937	0.1715472926	0.9999763851
BRD9	1.1095046498	0.5737361322	0.9999763851
TRIP13	1.2253692208	0.3142024972	0.9999763851
SLC12A7	1.3788134048	0.7084343816	0.9999763851
TERT	1.0272952748	0.9775187538	0.9999763851
CLPTM1L	-1.0944523267	0.5059698834	0.9999763851
LPCAT1	1.2314482354	0.2028154931	0.9999763851
MRPL36	-1.2391296706	0.2302305204	0.9999763851
NDUFS6	1.1268306312	0.385312181	0.9999763851
ADAMTS16	0	0	0
ICE1	1.1338173263	0.6085113608	0.9999763851
MED10	-1.1746505679	0.4358773797	0.9999763851
NSUN2	-1.2586099463	0.1836930704	0.9999763851
SRD5A1	-1.5464300369	0.172284816	0.9999763851
PAPD7	1.3272140271	0.3278855255	0.9999763851
ADCY2	18.2947739705	0.150179073	0.9999763851
FASTKD3	1.1079745143	0.7944069785	0.9999763851
MTRR	1.1425979009	0.6241310009	0.9999763851
SEMA5A	3.2353357581	0.554787377	0.9999763851
FAM173B	1.0320974006	0.9221007787	0.9999763851
CCT5	1.2508132988	0.0701522824	0.9999763851
MARCH6	1.0028277798	0.9881600434	0.9999763851
ROPN1L	1.2451878652	0.6413505763	0.9999763851
LOC105369214	0	0	0
ANKRD33B	1.1928599878	0.4755675729	0.9999763851
DAP	1.020277823	0.8982707271	0.9999763851
DNAH5	0	0	0
TRIO	-1.8391085221	0.1241848701	0.9999763851
FAM105A	-1.3579731069	0.6947105643	0.9999763851
OTULIN	1.0967807893	0.6426899807	0.9999763851
ANKH	-1.6720999618	0.1251828667	0.9999763851
FBXL7	3.2353357581	0.554787377	0.9999763851
ZNF622	-1.0124149774	0.9682488784	0.9999763851
FAM134B	-1.0942721156	0.5574644958	0.9999763851
MYO10	1.6701562626	0.2405779072	0.9999763851
BASP1	1.2138388766	0.2025633959	0.9999763851
CDH18	-1.315303824	0.8206171563	0.9999763851
CDH12	-1.2473421265	0.4846981226	0.9999763851
CDH10	3.2356557963	0.5547505991	0.9999763851
CDH6	3.2353357581	0.554787377	0.9999763851
DROSHA	-1.0083131701	0.9700519136	0.9999763851
C5orf22	1.3519899252	0.1392657729	0.9999763851
PDZD2	3.3745826092	0.002649	0.9999763851
GOLPH3	-1.0064794072	0.9706343362	0.9999763851

MTMR12	-1.1128186202	0.7044269244	0.9999763851
ZFR	1.1805519355	0.4154627842	0.9999763851
SUB1	-1.035908532	0.7791023536	0.9999763851
NPR3	0	0	0
TARS	-1.1688223867	0.2768165296	0.9999763851
ADAMTS12	-1.0365000139	0.939724395	0.9999763851
SLC45A2	5.4784711963	0.3745358536	0.9999763851
AMACR	-1.122607055	0.6172844992	0.9999763851
C1QTNF3	1.1139743991	0.825894664	0.9999763851
RAI14	7.7204093521	0.2880799703	0.9999763851
TTC23L	1.0277462901	0.9757593063	0.9999763851
RAD1	-1.0065558416	0.974974658	0.9999763851
BRIX1	1.0475222536	0.8147204312	0.9999763851
DNAJC21	1.0648239295	0.7388007403	0.9999763851
SPEF2	3.2353357581	0.554787377	0.9999763851
CAPSL	0	0	0
UGT3A1	0	0	0
UGT3A2	0	0	0
LMBRD2	1.019178451	0.9511885408	0.9999763851
SKP2	1.010106987	0.9537645527	0.9999763851
NADK2	1.1890743034	0.4077011875	0.9999763851
RANBP3L	0	0	0
SLC1A3	1.0313460152	0.9425337514	0.9999763851
NIPBL	1.0028494494	0.9854846891	0.9999763851
C5orf42	1.194342259	0.3611314359	0.9999763851
NUP155	-1.1465963961	0.6331928248	0.9999763851
WDR70	-1.1528282791	0.5245421344	0.9999763851
EGFLAM	0	0	0
RICTOR	-1.3800150214	0.2139514793	0.9999763851
FYB	1.163794998	0.5359784372	0.9999763851
C9	-4.2864494036	0.4027150576	0.9999763851
DAB2	-1.7714603521	0.2988191245	0.9999763851
PTGER4	-1.6990889299	0.0431805404	0.9999763851
TTC33	-3.6747177393	0.1931151166	0.9999763851
PRKAA1	-1.1577391151	0.5453531994	0.9999763851
RPL37	1.0793623142	0.5292698612	0.9999763851
CARD6	1.0615520261	0.898558141	0.9999763851
C7	-7.5622178268	0.2177145568	0.9999763851
PLCXD3	0	0	0
OXCT1	1.5016088246	0.133359043	0.9999763851
C5orf51	1.253548887	0.4615056856	0.9999763851
FBXO4	1.4212629034	0.1916695825	0.9999763851
GHR	1.2866381786	0.8224897331	0.9999763851
SEPP1	0	0	0
ANXA2R	-1.3372592868	0.0699291494	0.9999763851
ZNF131	1.086898254	0.7323244428	0.9999763851
NIM1K	-1.3154339304	0.8205576571	0.9999763851
HMGCS1	-1.0917094462	0.6237411636	0.9999763851
TMEM267	-1.2572901881	0.4107223017	0.9999763851

C5orf34	1.3611910537	0.5505174976	0.9999763851
PAIP1	1.1162284714	0.6223733884	0.9999763851
NNT	-1.2785787609	0.1311952866	0.9999763851
MRPS30	-1.4633034756	0.0754729668	0.9999763851
HCN1	0	0	0
EMB	1.2765785972	0.2753771178	0.9999763851
PARP8	1.0322659531	0.9462243091	0.9999763851
PELO	1.2457531369	0.3859198919	0.9999763851
ITGA1	1.1447333993	0.8765821901	0.9999763851
ITGA2	3.2356557963	0.5547505991	0.9999763851
MOCS2	-1.300942886	0.4931808278	0.9999763851
FST	-4.287394647	0.4026306792	0.9999763851
NDUFS4	1.1611500398	0.4229890686	0.9999763851
ARL15	-1.0264098069	0.9305384066	0.9999763851
SNX18	-1.4050843109	0.2694386048	0.9999763851
CDC20B	0	0	0
CCNO	3.3940373312	0.2284542701	0.9999763851
DHX29	1.0279249515	0.8980545797	0.9999763851
SKIV2L2	1.0908364731	0.6182025513	0.9999763851
PLPP1	3.3222963726	0.0159383191	0.9999763851
SLC38A9	1.1284190029	0.6940396891	0.9999763851
DDX4	0	0	0
IL31RA	1.0721046145	0.8895376753	0.9999763851
IL6ST	1.4730422263	0.3935397287	0.9999763851
MAP3K1	-1.8657528234	0.0470704441	0.9999763851
SETD9	-1.1576691309	0.6617655499	0.9999763851
MIER3	1.027644744	0.9775449549	0.9999763851
GPBP1	-1.2729873009	0.1316691284	0.9999763851
PLK2	-1.3154339304	0.8205576571	0.9999763851
GAPT	1.2370774927	0.4176638119	0.9999763851
RAB3C	1.3496981678	0.6618895999	0.9999763851
PDE4D	1.0494437221	0.8349144389	0.9999763851
DEPDC1B	-1.1801724753	0.38976722	0.9999763851
ELOVL7	-5.3416241096	0.0832549721	0.9999763851
ERCC8	2.0297988877	0.1270784268	0.9999763851
NDUFAF2	1.2760462595	0.2630941422	0.9999763851
SMIM15	-1.003471392	0.9903336711	0.9999763851
ZSWIM6	-1.1615828914	0.5838043997	0.9999763851
KIF2A	1.2063524007	0.3405315704	0.9999763851
DIMT1	1.1026851294	0.5822798633	0.9999763851
IPO11	1.0168309472	0.9509490403	0.9999763851
LRRC70	-4.2864494036	0.4027150576	0.9999763851
RGS7BP	0	0	0
FAM159B	-7.5622178268	0.2177145568	0.9999763851
SREK1IP1	-1.0674789252	0.72359263	0.9999763851
CWC27	1.0036494938	0.9855311691	0.9999763851
ADAMTS6	3.2353357581	0.554787377	0.9999763851
CENPK	-1.0260147302	0.9065100995	0.9999763851
PPWD1	-1.0436959252	0.8957371606	0.9999763851

TRIM23	1.2793384004	0.6434907424	0.9999763851
TRAPPC13	1.1767575576	0.553476984	0.9999763851
SGTB	1.0315755169	0.9467751201	0.9999763851
NLN	-1.0172690578	0.9313873694	0.9999763851
ERBIN	1.0371371172	0.8566336588	0.9999763851
SREK1	1.0194482272	0.9090055451	0.9999763851
MAST4	1.8136032532	0.5948809763	0.9999763851
PIK3R1	1.1848553405	0.2567139726	0.9999763851
SLC30A5	1.0317057902	0.9012887237	0.9999763851
CCNB1	1.0936217148	0.5557234296	0.9999763851
CENPH	-1.1698024954	0.5034810836	0.9999763851
MRPS36	-1.0139998568	0.9301881695	0.9999763851
CDK7	-1.775327643	0.3002198612	0.9999763851
CCDC125	1.6650197809	0.0734706097	0.9999763851
AK6	1.0056001956	0.974843088	0.9999763851
TAF9	1.2022376639	0.2241238402	0.9999763851
RAD17	-1.2417177958	0.2654276525	0.9999763851
MARVELD2	-4.2849908816	0.4028453076	0.9999763851
GTF2H2C	-1.4837348679	0.3693808373	0.9999763851
SERF1B	1.5675103224	0.4616650372	0.9999763851
SMN2	-1.7201309463	0.1211048637	0.9999763851
LOC107986354	0	0	0
SERF1A	1.198709426	0.7216013186	0.9999763851
SMN1	-1.0931170377	0.8312051539	0.9999763851
NAIP	1.032631767	0.902739453	0.9999763851
GTF2H2	-1.0587047992	0.8148951894	0.9999763851
LOC107986352	-10.8422096127	0.1434763784	0.9999763851
LOC107986353	-3.7489679912	0.1057436943	0.9999763851
BDP1	1.0536761547	0.8340660496	0.9999763851
MCCC2	1.3544926024	0.0874318893	0.9999763851
MAP1B	1.8142404796	0.5680221692	0.9999763851
MRPS27	-1.1771582637	0.2597252541	0.9999763851
PTCD2	-1.3986233071	0.3120962747	0.9999763851
TNPO1	1.0698079969	0.6642165571	0.9999763851
FCHO2	1.8137824633	0.5633863058	0.9999763851
FOXD1	-1.940446983	0.1183048709	0.9999763851
BTF3	1.0287149299	0.8237335446	0.9999763851
ANKRA2	-1.968664074	0.0977539434	0.9999763851
UTP15	1.2121834804	0.474552359	0.9999763851
ARHGEF28	5.6508551585	0.0443799389	0.9999763851
ENC1	-1.7343545975	0.4566121889	0.9999763851
HEXB	-1.1317733674	0.4088478275	0.9999763851
GFM2	1.2632069501	0.4608574497	0.9999763851
NSA2	1.0721873083	0.5898381939	0.9999763851
FAM169A	-3.3284109188	0.2422458152	0.9999763851
ANKRD31	-7.5647903763	0.2176246281	0.9999763851
HMGCR	-1.2071024166	0.2598485477	0.9999763851
COL4A3BP	-1.2299962021	0.3244120804	0.9999763851
POLK	-1.2483936913	0.4250880288	0.9999763851

ANKDD1B	1.2315138108	0.7930333038	0.9999763851
POC5	-1.7864048017	0.1955388735	0.9999763851
SV2C	-1.8719268659	0.3710114725	0.9999763851
IQGAP2	1.0666330679	0.757162646	0.9999763851
F2R	1.5540089775	0.5615744147	0.9999763851
CRHBP	-4.2864494036	0.4027150576	0.9999763851
AGGF1	1.105637555	0.6509809011	0.9999763851
ZBED3	1.6038585578	0.307708966	0.9999763851
PDE8B	-3.0806189565	0.1290820471	0.9999763851
WDR41	-1.308169079	0.2189957088	0.9999763851
OTP	3.2353357581	0.554787377	0.9999763851
TBCA	-1.2756512989	0.0476454889	0.9999763851
AP3B1	-1.1307099154	0.5665352894	0.9999763851
SCAMP1	-1.1416259037	0.408209981	0.9999763851
ARSB	-1.0181071179	0.9565283684	0.9999763851
JMY	1.0300270506	0.8498549792	0.9999763851
HOMER1	-1.2025267623	0.6484810084	0.9999763851
PAPD4	1.3750879473	0.2654900227	0.9999763851
CMYA5	0	0	0
MTX3	0	0	0
THBS4	-1.7337096776	0.4786781179	0.9999763851
SERINC5	-1.4066552203	0.2082326005	0.9999763851
ZFYVE16	1.0519851191	0.8564203476	0.9999763851
FAM151B	-1.0418588519	0.944162312	0.9999763851
DHFR	1.0683887893	0.6009708296	0.9999763851
MTRNR2L2	-2.1365060939	9.63E-06	0.0235467048
MSH3	-1.2990694178	0.3508257282	0.9999763851
RASGRF2	9.9624059448	0.2315471014	0.9999763851
CKMT2	20.799085697	7.21E-11	4.31E-07
ZCCHC9	1.0013621484	0.9957054878	0.9999763851
ACOT12	2.3407592913	0.4269677734	0.9999763851
SSBP2	-1.5447339161	0.2028156496	0.9999763851
ATG10	1.7530520053	0.0163448595	0.9999763851
RPS23	1.0825437927	0.4978635748	0.9999763851
ATP6AP1L	-3.0164295697	0.0800525572	0.9999763851
TMEM167A	-1.1887439211	0.2806827082	0.9999763851
XRCC4	1.2755036466	0.5430916698	0.9999763851
VCAN	3.2353357581	0.554787377	0.9999763851
COX7C	1.154623255	0.244525171	0.9999763851
RASA1	-1.5937874697	0.0907235579	0.9999763851
CCNH	-1.0970873269	0.6955009708	0.9999763851
TMEM161B	2.0025381574	0.1552880968	0.9999763851
MEF2C	1.4052763861	0.2520103609	0.9999763851
CETN3	1.0282352085	0.8573127588	0.9999763851
MBLAC2	2.0927749596	0.0915461587	0.9999763851
POLR3G	1.0956731458	0.7904871151	0.9999763851
LYSMD3	-1.1170247957	0.6941083451	0.9999763851
ARRDC3	1.5099452149	0.542638918	0.9999763851
NR2F1	1.3406186202	0.7026896893	0.9999763851

FAM172A	1.0878740233	0.6223693281	0.9999763851
KIAA0825	1.0945342559	0.8965971969	0.9999763851
SLF1	-1.6512078915	0.0798476454	0.9999763851
MCTP1	-1.2858772868	0.1933390603	0.9999763851
FAM81B	-4.2849908816	0.4028453076	0.9999763851
TTC37	-1.1706789818	0.5502880119	0.9999763851
ARSK	-1.9373716923	0.0482348772	0.9999763851
RFESD	-3.3272796812	0.2456402057	0.9999763851
SPATA9	0	0	0
RHOBTB3	0	0	0
GLRX	1.0234550894	0.8946808773	0.9999763851
LOC102724720	-1.3154339304	0.8205576571	0.9999763851
ELL2	-2.9675189681	0.0611094455	0.9999763851
CAST	1.5891505389	0.2868055959	0.9999763851
ERAP1	-1.1635943169	0.4892676851	0.9999763851
ERAP2	-1.5848281994	0.3611434329	0.9999763851
LOC101929747	3.2356557963	0.5547505991	0.9999763851
LNPEP	-1.1481507873	0.6748774685	0.9999763851
LIX1	-1.3948894265	0.6799644931	0.9999763851
RIOK2	1.1693005131	0.5511569783	0.9999763851
CHD1	1.3195610312	0.1631124018	0.9999763851
LOC100652833	0	0	0
FAM174A	-1.6309957392	0.1952230627	0.9999763851
ST8SIA4	1.3341592963	0.1362095382	0.9999763851
SLCO4C1	-1.2848160912	0.5743594087	0.9999763851
PAM	-1.5798006741	0.0661296139	0.9999763851
GIN1	-1.8165815049	0.4746912067	0.9999763851
PPIP5K2	-1.3580247762	0.1168063605	0.9999763851
C5orf30	-1.0545833002	0.8734296562	0.9999763851
EFNA5	3.2356557963	0.5547505991	0.9999763851
FBXL17	1.5469427682	0.053419978	0.9999763851
FER	1.0322800741	0.9510622492	0.9999763851
PJA2	-1.157385705	0.3961537472	0.9999763851
MAN2A1	-1.263116436	0.2274879757	0.9999763851
TMEM232	0	0	0
SLC25A46	1.1893696271	0.4200689215	0.9999763851
WDR36	-1.0854751162	0.6770445866	0.9999763851
STARD4	-1.9064802739	0.0369870394	0.9999763851
NREP	1.411495399	0.0381969471	0.9999763851
EPB41L4A	-1.564279968	0.015274556	0.9999763851
APC	-1.2546908028	0.3456481161	0.9999763851
SRP19	1.0765281899	0.6607126881	0.9999763851
REEP5	-1.5015911137	0.2427780701	0.9999763851
DCP2	1.1431060207	0.5050676797	0.9999763851
MCC	3.2353357581	0.554787377	0.9999763851
YTHDC2	-1.41969129	0.1700302102	0.9999763851
KCNN2	-1.7520744477	0.2065543168	0.9999763851
TRIM36	1.3407820359	0.7147820854	0.9999763851
PGGT1B	1.0490856426	0.8565738431	0.9999763851

CCDC112	-1.1273823203	0.6968775075	0.9999763851
FEM1C	1.0732627226	0.8480605384	0.9999763851
TICAM2	0	0	0
TMED7-TICAM2	-1.2498986999	0.4865361796	0.9999763851
TMED7	-1.0335201779	0.905858675	0.9999763851
ATG12	-1.2075932983	0.3513252039	0.9999763851
AP3S1	-1.1764626148	0.3327530666	0.9999763851
LVRN	3.2353357581	0.554787377	0.9999763851
ARL14EPL	1.0009947738	0.9940214408	0.9999763851
COMMD10	1.2799856445	0.3387271038	0.9999763851
SEMA6A	-2.5487783651	0.1140509541	0.9999763851
DTWD2	-1.0700717465	0.6288295893	0.9999763851
DMXL1	-1.3990154195	0.3414084987	0.9999763851
TNFAIP8	-1.3817809126	0.034347209	0.9999763851
HSD17B4	-1.0881652793	0.69641269	0.9999763851
PRR16	0	0	0
SRFBP1	-1.3517268404	0.343509771	0.9999763851
LOX	0	0	0
ZNF474	0	0	0
SNX2	-1.0389108698	0.8057363678	0.9999763851
SNX24	-4.3352072989	0.1515507502	0.9999763851
PPIC	0	0	0
CEP120	-1.1045657066	0.6851057233	0.9999763851
CSNK1G3	-1.1486121256	0.5657606046	0.9999763851
GRAMD3	0	0	0
PHAX	1.7051924843	0.1482362717	0.9999763851
TEX43	0	0	0
LMNB1	-1.1163235083	0.4713965397	0.9999763851
MARCH3	-2.1912989202	0.0189101366	0.9999763851
MEGF10	-4.287394647	0.4026306792	0.9999763851
PRRC1	-1.3776938236	0.0555987375	0.9999763851
CCDC192	-1.1820879394	0.7421986139	0.9999763851
SLC12A2	1.2199487697	0.3917108225	0.9999763851
ISOC1	1.1722292081	0.5300619524	0.9999763851
ADAMTS19	0	0	0
HINT1	-1.1747433422	0.1739616219	0.9999763851
LYRM7	1.1260650562	0.6332086168	0.9999763851
CDC42SE2	-1.0157492495	0.9468599796	0.9999763851
FNIP1	-1.3844811081	0.1705498344	0.9999763851
ACSL6	1.7580757543	0.4309464653	0.9999763851
IL3	0	0	0
SLC22A4	-1.2636518444	0.5000518047	0.9999763851
SLC22A5	-1.0877958255	0.769428697	0.9999763851
IRF1	-1.1458979993	0.6335117999	0.9999763851
RAD50	-1.0947723928	0.7086616672	0.9999763851
IL4	0	0	0
KIF3A	1.031230197	0.9525578979	0.9999763851
CCNI2	0	0	0
SEPT8	-1.2160661565	0.3434123335	0.9999763851

GDF9	0	0	0
UQCRQ	1.1078720448	0.4129865986	0.9999763851
LEAP2	1.8136032532	0.594590364	0.9999763851
AFF4	-1.6099145059	0.1200311409	0.9999763851
ZCCHC10	1.1412099429	0.6668671236	0.9999763851
HSPA4	-1.198795388	0.3507582851	0.9999763851
C5orf15	-1.3161865823	0.1707117165	0.9999763851
VDAC1	-1.1002831775	0.5306268282	0.9999763851
TCF7	3.2353357581	0.554787377	0.9999763851
SKP1	1.0374739805	0.7844187868	0.9999763851
PPP2CA	1.2530993626	0.1147117146	0.9999763851
CDKL3	-1.1541631479	0.7852744789	0.9999763851
UBE2B	-1.3230199474	0.1606651347	0.9999763851
CDKN2AIPNL	-1.1619180137	0.5861487389	0.9999763851
JADE2	1.4821235331	0.0844989709	0.9999763851
SAR1B	1.0055313518	0.9813904477	0.9999763851
SEC24A	-1.0237789293	0.9318003261	0.9999763851
CAMLG	-1.2464569194	0.1903344522	0.9999763851
DDX46	1.2181127865	0.2271641123	0.9999763851
C5orf24	-1.1840119095	0.4169757847	0.9999763851
TXNDC15	1.3361019637	0.1100119051	0.9999763851
PCBD2	-1.075784922	0.5570560047	0.9999763851
CATSPER3	-2.071297876	0.1061464698	0.9999763851
PITX1	2.1616088585	0.006889	0.9999763851
C5orf66	1.3428586414	0.5071091904	0.9999763851
H2AFY	1.0469595388	0.7385907231	0.9999763851
CXCL14	0	0	0
SLC25A48	-1.0803829108	0.921672941	0.9999763851
TGFBI	-10.8458971147	0.1433597877	0.9999763851
SMAD5	1.0095363977	0.9693953821	0.9999763851
SPOCK1	-1.0874546536	0.8051376623	0.9999763851
KLHL3	-4.2864494036	0.4027150576	0.9999763851
HNRNPA0	-1.1135184955	0.3858589531	0.9999763851
MYOT	-1.3946909469	0.71478671	0.9999763851
PKD2L2	1.730302978	0.1650268848	0.9999763851
FAM13B	1.283581429	0.4393518731	0.9999763851
NME5	0	0	0
BRD8	1.1637986196	0.5086804596	0.9999763851
KIF20A	1.0388101443	0.8671636894	0.9999763851
CDC23	1.0007795933	0.9973445443	0.9999763851
GFRA3	0	0	0
CDC25C	-1.6340413022	0.1591878748	0.9999763851
FAM53C	-1.2778879422	0.1912500561	0.9999763851
KDM3B	-1.8138472326	0.0330953316	0.9999763851
EGR1	-1.3655134517	0.3345336318	0.9999763851
ETF1	1.0436404415	0.7605969815	0.9999763851
HSPA9	-1.0050831432	0.9732525418	0.9999763851
CTNNA1	1.2229704509	0.3479516401	0.9999763851
SIL1	-1.6230240837	0.0445087979	0.9999763851

MATR3	1.1295049835	0.4293294823	0.9999763851
PAIP2	1.0645334165	0.8485470867	0.9999763851
SLC23A1	-1.1880898117	0.3460184105	0.9999763851
MZB1	1.3085209175	0.6210520445	0.9999763851
SPATA24	1.1327761444	0.8747914308	0.9999763851
DNAJC18	1.6857129034	0.3148839628	0.9999763851
ECSCR	-1.3716482783	0.7377245996	0.9999763851
TMEM173	1.1150224288	0.585410117	0.9999763851
UBE2D2	1.0212756737	0.8867828949	0.9999763851
CXXC5	1.0888926823	0.6395867376	0.9999763851
PSD2	3.2356557963	0.5547505991	0.9999763851
NRG2	3.2353357581	0.554787377	0.9999763851
PURA	-1.3440683414	0.1409325343	0.9999763851
IGIP	-1.5114413735	0.3258384346	0.9999763851
CYSTM1	-3.3685676065	0.0940787265	0.9999763851
PFDN1	1.2199860856	0.2187000662	0.9999763851
HBEGF	-1.9673293586	0.0282493532	0.9999763851
ANKHD1	-1.0430474576	0.8377020979	0.9999763851
ANKHD1-EIF4EBP3	1.4411002879	0.458942252	0.9999763851
EIF4EBP3	-1.0105516683	0.9751040845	0.9999763851
SRA1	1.5287513252	0.1539988889	0.9999763851
APBB3	1.7581722929	0.4280050507	0.9999763851
SLC35A4	-1.0134756743	0.9544118717	0.9999763851
TMCO6	-1.4325783176	0.4765600414	0.9999763851
CD14	-7.5622178268	0.2177145568	0.9999763851
NDUFA2	1.1719785289	0.3026992074	0.9999763851
IK	-1.1769459506	0.359337595	0.9999763851
WDR55	-1.7278081733	0.0296743137	0.9999763851
DND1	1.1505384589	0.7314979266	0.9999763851
HARS	1.0665594668	0.8154698205	0.9999763851
HARS2	-1.0208088603	0.9405243558	0.9999763851
ZMAT2	-1.3075549877	0.0499064585	0.9999763851
PCDHA1	0	0	0
PCDHB2	1.7500801512	0.5399738241	0.9999763851
PCDHB3	0	0	0
PCDHB16	0	0	0
PCDHB9	0	0	0
PCDHB10	0	0	0
PCDHB14	0	0	0
TAF7	1.2120035213	0.2753184362	0.9999763851
PCDHGA1	0	0	0
PCDHGB2	-1.3151016298	0.8210358465	0.9999763851
PCDHGA5	3.2356557963	0.5547505991	0.9999763851
PCDHGA6	-1.5416912797	0.6822921782	0.9999763851
DIAPH1	1.2540398548	0.1131610425	0.9999763851
HDAC3	-1.2688316816	0.1673193195	0.9999763851
RELL2	-3.5263270662	0.0226684974	0.9999763851
FCHSD1	2.2692722146	0.175986875	0.9999763851
ARAP3	-1.4447343307	0.2591314051	0.9999763851

KIAA0141	-1.0745995006	0.7492213718	0.9999763851
PCDH12	-1.0168655014	0.9689401748	0.9999763851
RNF14	-1.3805816571	0.2125795669	0.9999763851
GNPDA1	-1.0268806897	0.8876999037	0.9999763851
NDFIP1	-1.0153876591	0.9275471863	0.9999763851
SPRY4	-1.3714374731	0.7335244774	0.9999763851
FGF1	-4.2864494036	0.4027150576	0.9999763851
ARHGAP26	-1.4049714896	0.1254477339	0.9999763851
NR3C1	1.0197893251	0.9155713797	0.9999763851
YIPF5	-1.2425334927	0.2547564212	0.9999763851
KCTD16	-4.287394647	0.4026306792	0.9999763851
GRXCR2	0	0	0
SH3RF2	0	0	0
PLAC8L1	3.2353357581	0.554787377	0.9999763851
LARS	1.0255270847	0.8661933871	0.9999763851
RBM27	1.2695716025	0.2822370486	0.9999763851
TCERG1	1.0135270151	0.9329030201	0.9999763851
DPYSL3	-4.3514532582	0.0205389788	0.9999763851
JAKMIP2	-20.6921206198	0.0599099101	0.9999763851
SPINK14	3.2356557963	0.5547505991	0.9999763851
SPINK13	0	0	0
FBXO38	-1.1012212704	0.7729249184	0.9999763851
HTR4	-4.2849908816	0.4028453076	0.9999763851
ADRB2	-1.7770936465	0.0227716217	0.9999763851
GRPEL2	1.1697704486	0.5224381191	0.9999763851
PCYOX1L	-1.1044549699	0.6334647319	0.9999763851
IL17B	1.8145193361	0.5777749766	0.9999763851
CSNK1A1	1.2677264113	0.1585535003	0.9999763851
ARHGEF37	-2.3209115874	0.4620416282	0.9999763851
PPARGC1B	1.4026020849	0.2965096614	0.9999763851
PDE6A	-1.3154339304	0.8205576571	0.9999763851
SLC26A2	1.0600218663	0.7818659059	0.9999763851
TIGD6	1.2869488883	0.8192981379	0.9999763851
HMGXB3	1.0377449105	0.9001776168	0.9999763851
CSF1R	1.1136314063	0.3597254529	0.9999763851
CDX1	0	0	0
SLC6A7	0	0	0
CAMK2A	0	0	0
TCOF1	1.3051780921	0.2084530638	0.9999763851
CD74	-2.1595611468	0.007857	0.9999763851
RPS14	-1.1371811063	0.3279137754	0.9999763851
NDST1	-1.0489280572	0.7622470796	0.9999763851
SYNPO	3.2356557963	0.5547505991	0.9999763851
RBM22	-1.3939909523	0.0526193041	0.9999763851
DCTN4	-1.0081483343	0.97911102	0.9999763851
SMIM3	-1.0312351099	0.8490962339	0.9999763851
IRGM	-1.315303824	0.8206171563	0.9999763851
GPX3	3.2361532718	0.5546934395	0.9999763851
TNIP1	1.085406994	0.6739486537	0.9999763851

ANXA6	1.0113708098	0.9319255361	0.9999763851
CCDC69	-1.2418316932	0.2395884883	0.9999763851
LOC105378230	3.2353357581	0.554787377	0.9999763851
GM2A	-1.2252411179	0.2915648772	0.9999763851
SLC36A3	3.2361532718	0.5546934395	0.9999763851
SLC36A1	-1.0490453212	0.8754747555	0.9999763851
SPARC	-7.5647903763	0.2176246281	0.9999763851
ATOX1	-1.0064278152	0.9758739643	0.9999763851
G3BP1	-1.0473718592	0.7498732515	0.9999763851
GLRA1	3.0263279955	0.3115700401	0.9999763851
FAM114A2	1.0893432568	0.7985961817	0.9999763851
MFAP3	1.1968019865	0.6235588644	0.9999763851
GALNT10	-1.1693746321	0.4747997409	0.9999763851
SAP30L	-1.0323602148	0.8687921959	0.9999763851
LARP1	1.1248374295	0.4808724868	0.9999763851
FAXDC2	1.5670546705	0.2795090808	0.9999763851
CNOT8	1.0460582615	0.8458151004	0.9999763851
GEMIN5	1.5023703383	0.0768621916	0.9999763851
MRPL22	-1.0697514124	0.7497981712	0.9999763851
KIF4B	-1.1168215278	0.8600835236	0.9999763851
SGCD	3.2356557963	0.5547505991	0.9999763851
TIMD4	0	0	0
HAVCR2	-7.5664585246	0.2263571921	0.9999763851
MED7	-1.7539069202	0.3004110086	0.9999763851
CYFIP2	1.1632338587	0.444099768	0.9999763851
SOX30	0	0	0
THG1L	-1.1499816039	0.5079728324	0.9999763851
LSM11	1.25862468	0.5218580594	0.9999763851
CLINT1	-1.1486301191	0.5029575071	0.9999763851
EBF1	-1.0569678118	0.8933020074	0.9999763851
RNF145	1.2318567466	0.2489003337	0.9999763851
UBLCP1	1.202397692	0.474101566	0.9999763851
ADRA1B	-1.0847550188	0.6224205807	0.9999763851
TTC1	1.0002501273	0.9988195572	0.9999763851
PWWP2A	-1.0089122074	0.9677389195	0.9999763851
FABP6	-1.3154339304	0.8205576571	0.9999763851
CCNJL	1.0438732601	0.8432223354	0.9999763851
ZBED8	-3.3264391549	0.2740318683	0.9999763851
SLU7	1.0378378892	0.851262215	0.9999763851
PTTG1	1.3030244098	0.1028561638	0.9999763851
ATP10B	-2.3227998731	0.4384090875	0.9999763851
GABRB2	0	0	0
CCNG1	1.0702745978	0.7213632317	0.9999763851
NUDCD2	-1.0352930687	0.8853099154	0.9999763851
HMMR	1.1151479677	0.5936879568	0.9999763851
MAT2B	1.2100435965	0.3622352782	0.9999763851
WWC1	-1.315303824	0.8206171563	0.9999763851
RARS	1.2289263869	0.2399183768	0.9999763851
PANK3	1.1255489526	0.7150289392	0.9999763851

SLIT3	-1.3154339304	0.8205576571	0.9999763851
SPDL1	1.0380266976	0.8470102679	0.9999763851
DOCK2	-1.0014383892	0.9937981797	0.9999763851
C5orf58	-1.4298152945	0.5324548037	0.9999763851
LCP2	1.0890640737	0.5932705599	0.9999763851
KCNIP1	1.0278478563	0.9759371063	0.9999763851
KCNMB1	1.8309300162	0.2403158441	0.9999763851
RANBP17	0	0	0
NPM1	1.1127709422	0.4420663976	0.9999763851
FGF18	0	0	0
FBXW11	1.3007573377	0.3293280918	0.9999763851
STK10	1.292463118	0.104516222	0.9999763851
EFCAB9	7.7488609396	0.306063442	0.9999763851
UBTD2	-1.0734606083	0.8199035404	0.9999763851
SH3PXD2B	-1.5899635701	0.1421405993	0.9999763851
NEURL1B	-1.3145826607	0.0799435306	0.9999763851
DUSP1	-1.0636117229	0.8984347309	0.9999763851
ERGIC1	1.112369021	0.6167791956	0.9999763851
RPL26L1	-1.1166789592	0.5356225617	0.9999763851
ATP6V0E1	-1.1378254862	0.4000076312	0.9999763851
CREBRF	-1.2287093703	0.6650442501	0.9999763851
BNIP1	1.1821588963	0.640152621	0.9999763851
STC2	0	0	0
BOD1	1.1279816408	0.6138282389	0.9999763851
CPEB4	-1.0113323872	0.9785550984	0.9999763851
HMP19	0	0	0
SFXN1	-1.0813967429	0.6306584392	0.9999763851
HRH2	-1.0158926982	0.9611368643	0.9999763851
CPLX2	0	0	0
THOC3	-1.2862780529	0.225665919	0.9999763851
FAM153B	0	0	0
SIMC1	-1.3517402332	0.2709176894	0.9999763851
KIAA1191	1.1231753977	0.598199386	0.9999763851
ARL10	2.1898530381	0.0932715052	0.9999763851
NOP16	-1.0828370655	0.6266816572	0.9999763851
HIGD2A	1.0107336606	0.9481618855	0.9999763851
CLTB	1.3064627426	0.1917166162	0.9999763851
FAF2	1.1605017343	0.5240843689	0.9999763851
RNF44	-1.2278833135	0.3606905001	0.9999763851
CDHR2	9.9312452783	0.248571704	0.9999763851
GPRIN1	1.2872739558	0.823199828	0.9999763851
TSPAN17	1.3865306171	0.1616943158	0.9999763851
UNC5A	-2.4722032711	0.0162714699	0.9999763851
HK3	2.5918265342	0.1720915532	0.9999763851
UIMC1	-1.2219585324	0.480139307	0.9999763851
ZNF346	-1.165637376	0.4845047742	0.9999763851
FGFR4	0	0	0
NSD1	-1.0066831822	0.9795199421	0.9999763851
RAB24	1.191827495	0.4384099002	0.9999763851

PRELID1	1.2392551038	0.1132141269	0.9999763851
MXD3	-1.2734465399	0.2867942235	0.9999763851
LMAN2	-1.329736006	0.0224027058	0.9999763851
RGS14	1.1248505837	0.6625029083	0.9999763851
SLC34A1	0	0	0
F12	1.5448092247	0.0468022512	0.9999763851
GRK6	1.1578445421	0.3950917926	0.9999763851
PRR7	-1.1364457606	0.5640940914	0.9999763851
DBN1	1.0330706281	0.901026743	0.9999763851
PDLIM7	2.375646122	0.0438465364	0.9999763851
DOK3	-1.1268218746	0.5099106524	0.9999763851
DDX41	1.0613932891	0.7599829569	0.9999763851
FAM193B	-2.9860395318	0.0314884637	0.9999763851
TMED9	-1.1146119307	0.3950343941	0.9999763851
B4GALT7	1.1793587329	0.412755337	0.9999763851
FAM153A	1.1402814242	0.8191118827	0.9999763851
N4BP3	3.0864801482	0.3123107395	0.9999763851
RMND5B	-1.1241424001	0.724707939	0.9999763851
NHP2	1.1497750015	0.3576628199	0.9999763851
HNRNPAB	1.2071105457	0.1895377802	0.9999763851
PHYKPL	-1.6351434787	0.138894839	0.9999763851
COL23A1	1.0558437182	0.8263950877	0.9999763851
CLK4	-1.4449771506	0.3141887912	0.9999763851
LOC102724657	5.4781708352	0.3745527127	0.9999763851
ZNF354A	1.1432178119	0.7644331663	0.9999763851
ZFP2	1.0156708	0.9843163845	0.9999763851
ZNF454	-4.2864494036	0.4027150576	0.9999763851
ZNF354C	-1.0281042328	0.9660991904	0.9999763851
RUFY1	1.1668053172	0.4476650866	0.9999763851
HNRNPH1	-1.1739393047	0.2084249518	0.9999763851
CANX	-1.0105851451	0.93661336	0.9999763851
MAML1	-1.0644122754	0.7593711873	0.9999763851
LTC4S	-1.9665380586	0.4649954536	0.9999763851
MGAT4B	1.1547107643	0.3081534752	0.9999763851
SQSTM1	-1.100891526	0.5100785141	0.9999763851
C5orf45	1.9234847933	0.4409858345	0.9999763851
TBC1D9B	-1.0198859634	0.9104713834	0.9999763851
RNF130	1.0480085305	0.8089449377	0.9999763851
MAPK9	-1.7039899964	0.0566909813	0.9999763851
CNOT6	-1.1670010485	0.3422330604	0.9999763851
MGAT1	1.0201442584	0.899787473	0.9999763851
ZFP62	-1.5630381647	0.1367644137	0.9999763851
BTNL8	7.7192231741	0.2782707334	0.9999763851
BTNL3	1.1235489843	0.8887158854	0.9999763851
BTNL9	0	0	0
TRIM7	1.3309109743	0.4419099056	0.9999763851
TRIM41	1.0310899617	0.9154024576	0.9999763851
RACK1	1.1363422888	0.2932821553	0.9999763851
TRIM52	1.0331774172	0.9315504759	0.9999763851

OR4F3	7.4933530102	0.2988194544	0.9999763851
DUSP22	-1.3334843025	0.0689783311	0.9999763851
IRF4	0	0	0
EXOC2	1.0527962553	0.8135231698	0.9999763851
HUS1B	0	0	0
FOXC1	-1.002854239	0.98390514	0.9999763851
GMDS	1.0633471786	0.7264081501	0.9999763851
MYLK4	-2.3212684229	0.4457081023	0.9999763851
WRNIP1	-1.6255183566	0.0522188792	0.9999763851
SERPINB1	1.1174187187	0.3762945995	0.9999763851
SERPINB9	0	0	0
SERPINB6	1.0614402741	0.8085620198	0.9999763851
NQO2	1.871095762	0.005377	0.9999763851
RIPK1	-1.3600967295	0.1470196257	0.9999763851
BPHL	-1.2633222574	0.6187620644	0.9999763851
TUBB2A	1.4471291231	0.1673804881	0.9999763851
PSMG4	1.199115031	0.3299313822	0.9999763851
PRPF4B	1.1448490863	0.431145989	0.9999763851
FAM217A	0	0	0
C6orf201	0	0	0
ECI2	1.1867934925	0.4095264875	0.9999763851
CDYL	1.1937141994	0.413022128	0.9999763851
RPP40	1.373180136	0.2046216572	0.9999763851
LYRM4	-1.0951263794	0.5874755201	0.9999763851
FARS2	1.0110918591	0.9538133653	0.9999763851
LY86	1.168799638	0.7851988158	0.9999763851
RREB1	1.1399080677	0.5174439093	0.9999763851
SSR1	-1.0034439005	0.9817877547	0.9999763851
CAGE1	-1.7728569902	0.2878106055	0.9999763851
RIOK1	1.0829986487	0.7000332669	0.9999763851
SNRNP48	1.1958741207	0.4912068544	0.9999763851
TXNDC5	-1.0418038076	0.830530858	0.9999763851
BLOC1S5	1.2073832161	0.5073841549	0.9999763851
EEF1E1	1.2015479138	0.3983350906	0.9999763851
SLC35B3	-1.5572663444	0.1843255065	0.9999763851
LOC107983965	3.2353357581	0.554787377	0.9999763851
GCNT2	1.7960129086	0.2738372475	0.9999763851
C6orf52	1.080806403	0.9300751612	0.9999763851
PAK1IP1	1.1011194953	0.731507482	0.9999763851
TMEM14C	1.0902346985	0.5833302326	0.9999763851
TMEM14B	1.0368765127	0.8294347783	0.9999763851
MAK	1.0309914225	0.9539298003	0.9999763851
SYCP2L	0	0	0
SMIM13	1.102970576	0.7296526583	0.9999763851
NEDD9	-1.0015502022	0.9941218394	0.9999763851
TMEM170B	-1.2031435942	0.629981179	0.9999763851
ADTRP	-1.6862542947	0.5029729576	0.9999763851
HIVEP1	-1.7965983223	0.1075879351	0.9999763851
EDN1	-4.287394647	0.4026306792	0.9999763851

PHACTR1	1.048909848	0.8971040141	0.9999763851
TBC1D7-LOC100130357	-2.8954939448	0.1031374145	0.9999763851
LOC100130357	-1.9358492725	0.1794595207	0.9999763851
TBC1D7	-1.0964044636	0.7291248559	0.9999763851
GFOD1	1.1204546568	0.5692277502	0.9999763851
SIRT5	-1.8154850617	0.0370015592	0.9999763851
NOL7	1.2867658583	0.1527092405	0.9999763851
RANBP9	-2.0435742997	0.0767853163	0.9999763851
MCUR1	1.1265670181	0.6302894636	0.9999763851
CD83	1.0900573575	0.6909963875	0.9999763851
JARID2	-1.0281380524	0.8364772847	0.9999763851
DTNBP1	-1.0625573106	0.7385454519	0.9999763851
MYLIP	-1.2423939185	0.2372608349	0.9999763851
GMPR	-1.2291531336	0.4275012611	0.9999763851
ATXN1	-2.1107222195	0.0007695	0.6568370458
CAP2	0	0	0
FAM8A1	-1.3710217679	0.4272488728	0.9999763851
NUP153	1.0077032902	0.9627955799	0.9999763851
TPMT	-1.0274776969	0.9212508016	0.9999763851
KDM1B	-1.1662637326	0.6355381457	0.9999763851
DEK	1.0735137278	0.6380575792	0.9999763851
MBOAT1	-1.3652390417	0.2618926622	0.9999763851
E2F3	-1.0800786676	0.6719351348	0.9999763851
CDKAL1	-1.3710069214	0.1967909662	0.9999763851
SOX4	1.1329926733	0.5112785847	0.9999763851
MRS2	1.4059691433	0.334629559	0.9999763851
GPLD1	-1.9649399627	0.4633510527	0.9999763851
ALDH5A1	1.0550720662	0.8182683786	0.9999763851
KIAA0319	0	0	0
TDP2	1.1097167271	0.716812405	0.9999763851
ACOT13	-1.6500534154	0.0288478327	0.9999763851
C6orf62	1.290500015	0.175119709	0.9999763851
GMNN	1.2040262067	0.2992667262	0.9999763851
FAM65B	-1.0810584893	0.9277710023	0.9999763851
LRRC16A	1.3371096606	0.5606154336	0.9999763851
SLC17A2	0	0	0
TRIM38	-1.3296395049	0.3308849748	0.9999763851
HIST1H3A	3.2353357581	0.554787377	0.9999763851
HIST1H4B	0	0	0
HIST1H3B	0	0	0
HIST1H2AB	-2.3214980365	0.4356694687	0.9999763851
HIST1H2BB	3.2353357581	0.554787377	0.9999763851
HIST1H3C	5.5007639251	0.0820165818	0.9999763851
HIST1H1C	1.3840473097	0.19617271	0.9999763851
HIST1H4C	-1.1424830125	0.3490995262	0.9999763851
HIST1H2BC	-1.3158816768	0.8203229797	0.9999763851
HIST1H2AC	-2.3227998731	0.4393018063	0.9999763851
HIST1H1E	-2.6922490556	0.2894499135	0.9999763851
HIST1H2BD	1.9439386629	0.1725410944	0.9999763851

HIST1H4D	0	0	0
HIST1H3D	1.02953687	0.9674511753	0.9999763851
HIST1H2BF	12.2036838333	0.1842407399	0.9999763851
HIST1H4E	1.3931546432	0.780500185	0.9999763851
HIST1H1D	1.3017937938	0.5827569262	0.9999763851
HIST1H3F	1.432536289	0.7055682726	0.9999763851
HIST1H2BH	3.395936765	0.2436383267	0.9999763851
HIST1H4H	-4.287394647	0.4026306792	0.9999763851
BTN3A2	-1.3739855382	0.2993391487	0.9999763851
BTN2A2	1.1893748374	0.7992261052	0.9999763851
BTN3A1	-1.0361602273	0.9386077569	0.9999763851
BTN3A3	2.7932054058	0.2348296718	0.9999763851
BTN2A1	-1.4425936126	0.3116159148	0.9999763851
HMGN4	1.1620728604	0.4304138487	0.9999763851
ABT1	-1.2492280853	0.1212026377	0.9999763851
ZNF322	0	0	0
HIST1H2BJ	-1.3715126673	0.7508069165	0.9999763851
HIST1H2AG	1.027554661	0.9756512103	0.9999763851
HIST1H2BK	1.3733021616	0.356493371	0.9999763851
HIST1H4I	-4.3471027681	0.0736681408	0.9999763851
HIST1H2AH	2.8679346912	0.2853554691	0.9999763851
ZNF391	-14.1139127893	0.1029631119	0.9999763851
ZNF184	1.5874882539	0.3330291155	0.9999763851
HIST1H2BL	-2.3222877795	0.4506271768	0.9999763851
HIST1H2AI	1.1594180032	0.8885293561	0.9999763851
HIST1H3H	-7.5647903763	0.2176246281	0.9999763851
HIST1H2AJ	12.2185410059	0.2032706859	0.9999763851
HIST1H2BM	0	0	0
HIST1H4J	-1.2763412932	0.5497413118	0.9999763851
HIST1H4K	1.3330083285	0.6870523879	0.9999763851
HIST1H2AK	0	0	0
HIST1H2BN	0	0	0
HIST1H2AL	13.0486682238	0.1811889602	0.9999763851
HIST1H1B	-1.3714374731	0.7334000329	0.9999763851
HIST1H3J	-3.1553169002	0.1647503595	0.9999763851
HIST1H2AM	1.9659465739	0.3446433874	0.9999763851
HIST1H2BO	1.2869488883	0.8194288953	0.9999763851
OR2B2	-4.2849908816	0.4028453076	0.9999763851
OR2B6	0	0	0
ZNF165	1.1695260761	0.7891522264	0.9999763851
ZSCAN16	5.7467514146	0.0890156596	0.9999763851
ZKSCAN8	1.0486314085	0.8629341143	0.9999763851
ZSCAN9	1.3988502013	0.2439725787	0.9999763851
ZKSCAN4	-1.0125657412	0.9674572788	0.9999763851
NKAPL	0	0	0
ZSCAN26	-1.7344697458	0.454110681	0.9999763851
PGBD1	-1.158513231	0.7301951407	0.9999763851
ZSCAN31	1.0949541325	0.8930596511	0.9999763851
ZKSCAN3	1.292477824	0.4934975649	0.9999763851

GPX6	0	0	0
TRIM27	-1.2204826048	0.2263320891	0.9999763851
OR2H2	0	0	0
GABBR1	1.1675190011	0.7088251579	0.9999763851
MOG	0	0	0
HLA-F	-1.7096486986	0.273465867	0.9999763851
HLA-G	0	0	0
HLA-A	-1.079111361	0.5855363408	0.9999763851
ZNRD1	-1.0388863158	0.8253915168	0.9999763851
PPP1R11	1.0827162259	0.7203155703	0.9999763851
RNF39	3.7441009688	0.2476895772	0.9999763851
TRIM31	0	0	0
TRIM26	1.3544335938	0.1375912662	0.9999763851
TRIM39	-20.0378342472	0.0892204583	0.9999763851
TRIM39-RPP21	-1.1573686005	0.7427884485	0.9999763851
RPP21	-1.1127851704	0.6539898097	0.9999763851
HLA-E	-1.5072059691	0.003625	0.9999763851
GNL1	1.0987763497	0.638703301	0.9999763851
PRR3	1.1456993984	0.6225473516	0.9999763851
ABCF1	1.2335808128	0.1481646686	0.9999763851
PPP1R10	-1.1770030549	0.3539269016	0.9999763851
MRPS18B	-1.0313267437	0.8165273578	0.9999763851
ATAT1	1.4842005632	0.5232350533	0.9999763851
C6orf136	1.0117350175	0.9743631906	0.9999763851
DHX16	1.0155177103	0.9481413597	0.9999763851
PPP1R18	1.1202586693	0.425575773	0.9999763851
NRM	1.090831491	0.680838766	0.9999763851
MDC1	-1.1108862188	0.5410051403	0.9999763851
TUBB	1.1120620968	0.3826158046	0.9999763851
FLOT1	1.1453415904	0.4769329628	0.9999763851
IER3	-2.8235114951	1.43E-07	0.0004266
DDR1	0	0	0
GTF2H4	-1.0921363389	0.6628029503	0.9999763851
VARS2	1.232147775	0.4308935742	0.9999763851
PSORS1C1	3.2361532718	0.5546934395	0.9999763851
CCHCR1	-1.1396682755	0.6330207147	0.9999763851
TCF19	1.1880288265	0.3771669073	0.9999763851
POU5F1	-2.3901671273	0.2049653457	0.9999763851
HLA-C	1.0139129072	0.9083474318	0.9999763851
HLA-B	-1.1130410705	0.3529404656	0.9999763851
MICA	-5.3300550044	0.00568	0.9999763851
MICB	-1.2485856028	0.3767962661	0.9999763851
DDX39B	1.316538806	0.2442027782	0.9999763851
ATP6V1G2	-1.315303824	0.8206171563	0.9999763851
NFKBIL1	1.039116412	0.9507855786	0.9999763851
LTA	-3.3275701064	0.2296160779	0.9999763851
TNF	-1.3565048605	0.3938437439	0.9999763851
LTB	-1.2716043255	0.3954933343	0.9999763851
LST1	1.0178406016	0.9304903466	0.9999763851

NCR3	0	0	0
AIF1	-1.0313053768	0.8219982288	0.9999763851
PRRC2A	-1.0436765172	0.8548639305	0.9999763851
BAG6	1.2622328658	0.1482369216	0.9999763851
APOM	1.1162737823	0.8189082035	0.9999763851
C6orf47	-1.2526089319	0.4444352123	0.9999763851
GPANK1	1.1262935268	0.6061673408	0.9999763851
CSNK2B	-1.111051353	0.6178038402	0.9999763851
LY6G5C	-4.1323543353	0.2116983892	0.9999763851
ABHD16A	-1.2511027301	0.1489326732	0.9999763851
LY6G6F	0	0	0
LY6G6D	0	0	0
LY6G6C	-4.2864494036	0.4027150576	0.9999763851
C6orf25	3.2353357581	0.554787377	0.9999763851
DDAH2	-1.209428745	0.4606087591	0.9999763851
CLIC1	-1.0255940494	0.887545273	0.9999763851
MSH5	1.5038533918	0.3206245341	0.9999763851
SAPCD1	-1.5993699949	0.2386112253	0.9999763851
VWA7	3.2361532718	0.5546934395	0.9999763851
VARS	1.4004270135	0.0715753125	0.9999763851
LSM2	-1.2445964545	0.1173032334	0.9999763851
HSPA1L	1.4536849862	0.5998516948	0.9999763851
HSPA1A	-1.3068858933	0.1533762596	0.9999763851
HSPA1B	1.0307397793	0.9394662322	0.9999763851
C6orf48	-1.0036979326	0.9766038038	0.9999763851
NEU1	1.1802597002	0.2414554302	0.9999763851
SLC44A4	-4.3342515471	0.1407355628	0.9999763851
EHMT2	1.1977690051	0.377919572	0.9999763851
C2	-1.3448369019	0.3702703197	0.9999763851
CFB	0	0	0
NELFE	-1.1432636923	0.4716488323	0.9999763851
SKIV2L	1.1158867863	0.6433037083	0.9999763851
DXO	1.4193087527	0.3955382139	0.9999763851
STK19	1.1877151069	0.4730321971	0.9999763851
C4A	3.2361532718	0.5546934395	0.9999763851
C4B	0	0	0
CYP21A2	1.2869488883	0.8195618665	0.9999763851
TNXB	-1.3708355312	0.7476295757	0.9999763851
ATF6B	-1.0317851214	0.8790885174	0.9999763851
FKBPL	1.0820364507	0.8681455525	0.9999763851
PRRT1	7.7211724672	0.2779709379	0.9999763851
PPT2	1.601609162	0.048671121	0.9999763851
EGFL8	1.2445072841	0.6554110332	0.9999763851
AGPAT1	1.1555723305	0.4667118456	0.9999763851
RNF5	-1.0408365126	0.7988439409	0.9999763851
AGER	1.2552297886	0.328727511	0.9999763851
PBX2	-1.2677708339	0.4558133494	0.9999763851
GPSM3	-1.3571603628	0.6496251316	0.9999763851
BTNL2	0	0	0

HLA-DRA	-4.1569222306	6.13E-05	0.1067663916
HLA-DRB5	-3.8874746295	0.004145	0.9999763851
HLA-DRB1	-3.0580329935	0.004268	0.9999763851
HLA-DQA1	0	0	0
HLA-DQB1	-4.2849908816	0.4028453076	0.9999763851
HLA-DQB2	0	0	0
HLA-DOB	1.3544447817	0.6246627528	0.9999763851
TAP2	1.0806491141	0.6128763048	0.9999763851
PSMB8	-1.0863811535	0.5579720761	0.9999763851
TAP1	1.0674921463	0.6976855195	0.9999763851
PSMB9	1.0041462236	0.9818447128	0.9999763851
HLA-DMB	-1.3717838846	0.7497288712	0.9999763851
HLA-DMA	-4.439622732	0.1610722081	0.9999763851
BRD2	1.1324951578	0.4733483943	0.9999763851
HLA-DPA1	-6.873511443	0.0740539612	0.9999763851
HLA-DPB1	-4.2849908816	0.4028453076	0.9999763851
COL11A2	-2.3217011239	0.4355680547	0.9999763851
RXRB	1.0290642613	0.8855348643	0.9999763851
SLC39A7	-1.3069194868	0.1694861383	0.9999763851
HSD17B8	-1.3154339304	0.8205576571	0.9999763851
RING1	-1.2346253125	0.3997639897	0.9999763851
VPS52	1.4078728668	0.1823690597	0.9999763851
RPS18	-1.0246697185	0.8325409311	0.9999763851
WDR46	1.4420574504	0.0258098785	0.9999763851
PFDN6	1.171836825	0.6616807212	0.9999763851
RGL2	1.5673863565	0.1058453692	0.9999763851
TAPBP	1.0795239262	0.6016790093	0.9999763851
ZBTB22	1.8271023597	0.2382582789	0.9999763851
DAXX	1.0468507633	0.7994633165	0.9999763851
KIFC1	-1.1693818425	0.2718539388	0.9999763851
PHF1	-2.1335533219	0.1103124001	0.9999763851
CUTA	1.047381073	0.7572631763	0.9999763851
SYNGAP1	1.3500680352	0.6817324792	0.9999763851
ZBTB9	2.0077272611	0.2076621845	0.9999763851
BAK1	-1.4058129006	0.1640587645	0.9999763851
ITPR3	1.0653352255	0.7259616133	0.9999763851
UQCC2	1.7306862161	0.001235	0.8877123975
LEMD2	-1.1584054121	0.4768064393	0.9999763851
GRM4	0	0	0
HMGA1	1.0240936374	0.865206954	0.9999763851
C6orf1	-1.0969881584	0.5768730555	0.9999763851
NUDT3	-1.8915302495	6.62E-05	0.1114052542
RPS10-NUDT3	6.7289516799	0.0008724	0.7222990014
RPS10	1.0659986387	0.6139187632	0.9999763851
PACSIN1	0	0	0
C6orf106	-1.1409810007	0.3744386594	0.9999763851
SNRPC	1.0701711077	0.599406673	0.9999763851
UHRF1BP1	1.0129025062	0.9650812812	0.9999763851
TAF11	1.5347618451	0.0127948793	0.9999763851

ANKS1A	-1.412609226	0.0276593207	0.9999763851
SCUBE3	-1.6613257169	0.5372589806	0.9999763851
ZNF76	-1.5785323773	0.111021089	0.9999763851
DEF6	1.3921969005	0.0728931032	0.9999763851
PPARD	1.0121633381	0.9653127521	0.9999763851
FANCE	-1.0869160903	0.6355340917	0.9999763851
RPL10A	-1.1555252134	0.2019472043	0.9999763851
TEAD3	7.7204093521	0.2879712462	0.9999763851
TULP1	1.3260278971	0.7356363825	0.9999763851
FKBP5	1.2577852996	0.1622877808	0.9999763851
ARMC12	3.2353357581	0.554787377	0.9999763851
CLPSL2	0	0	0
CLPSL1	3.2356557963	0.5547505991	0.9999763851
SRPK1	1.2287548547	0.2372923813	0.9999763851
SLC26A8	1.1332235671	0.8775145921	0.9999763851
MAPK14	1.0206219905	0.8948870152	0.9999763851
MAPK13	-1.4158302751	0.6302808572	0.9999763851
BRPF3	1.9281764229	0.0605497017	0.9999763851
PNPLA1	1.6226464637	0.2844609798	0.9999763851
ETV7	1.1665343232	0.748393306	0.9999763851
PXT1	0	0	0
KCTD20	1.0763906677	0.6726939536	0.9999763851
STK38	-1.0910421376	0.6644928954	0.9999763851
SRSF3	1.1055702909	0.4004525456	0.9999763851
CDKN1A	-1.7193195151	0.0431703437	0.9999763851
RAB44	1.3657705797	0.0656729029	0.9999763851
CPNE5	-1.1638622622	0.6504706901	0.9999763851
C6orf89	-1.0203370123	0.8871502959	0.9999763851
PPIL1	1.3584065155	0.038170088	0.9999763851
PI16	3.2356557963	0.5547505991	0.9999763851
MTCH1	1.0525178827	0.7000245797	0.9999763851
PIM1	-1.2918127437	0.1320409694	0.9999763851
TMEM217	2.407854628	0.146589779	0.9999763851
TBC1D22B	-1.021394029	0.9457581476	0.9999763851
RNF8	1.2377771851	0.2972919164	0.9999763851
LOC107986531	0	0	0
CMTR1	-1.1712090826	0.4576166623	0.9999763851
CCDC167	-1.0139481072	0.9317216983	0.9999763851
MDGA1	-1.6479730166	0.4712945128	0.9999763851
ZFAND3	1.1812153012	0.2468949758	0.9999763851
BTBD9	1.3691236703	0.416749668	0.9999763851
GLO1	1.1217509757	0.3444200061	0.9999763851
DNAH8	-4.3338228569	0.1404318024	0.9999763851
SAYSD1	-1.1493738841	0.5012672948	0.9999763851
KCNK5	1.1462649934	0.5537859876	0.9999763851
KIF6	-4.2864494036	0.4027150576	0.9999763851
DAAM2	0	0	0
MOCS1	0	0	0
UNC5CL	3.2353357581	0.554787377	0.9999763851

APOBEC2	1.1334347738	0.8813103257	0.9999763851
OARD1	-1.0803406925	0.7693396242	0.9999763851
NFYA	-1.0384695716	0.8530541423	0.9999763851
TREM2	-3.3295429242	0.2522859573	0.9999763851
TREML2	1.1659487415	0.6571782306	0.9999763851
TREML4	0	0	0
TREM1	-5.6775738349	3.69E-05	0.0734702713
NCR2	-1.8514073702	0.0758420951	0.9999763851
FOXP4	1.1826038557	0.5376432012	0.9999763851
TFEB	1.012545413	0.9493078966	0.9999763851
FRS3	-1.0358731694	0.9588091495	0.9999763851
PRICKLE4	1.8140611791	0.5849939348	0.9999763851
TOMM6	1.1196341116	0.3260014588	0.9999763851
USP49	1.0692698325	0.7290472565	0.9999763851
MED20	-1.0119640774	0.9465615314	0.9999763851
BYSL	1.2030940444	0.2394192144	0.9999763851
CCND3	1.2972618728	0.050878361	0.9999763851
TAF8	1.4941732541	0.0815937503	0.9999763851
C6orf132	3.2353357581	0.554787377	0.9999763851
GUCA1A	0	0	0
GUCA1B	-1.4822028091	0.4890505289	0.9999763851
MRPS10	1.0792021131	0.5563499708	0.9999763851
TRERF1	-1.0028561598	0.9864764537	0.9999763851
LOC107986596	-1.949721077	0.3508375077	0.9999763851
UBR2	-1.1188966184	0.5133662104	0.9999763851
PRPH2	0	0	0
TBCC	1.0059763525	0.969748239	0.9999763851
GLTSCR1L	-1.220312238	0.2954314148	0.9999763851
RPL7L1	1.1217284398	0.361294495	0.9999763851
C6orf226	1.7574593324	0.442324695	0.9999763851
PTCRA	-4.2864494036	0.4027150576	0.9999763851
CNPY3	1.1589763219	0.4578530195	0.9999763851
CNPY3-GNMT	1.0056447524	0.9727034618	0.9999763851
PEX6	-1.0562128771	0.800251561	0.9999763851
MEA1	1.0436369579	0.7367193442	0.9999763851
PPP2R5D	2.3116608094	0.0797852139	0.9999763851
KLHDC3	1.0467323482	0.7267949098	0.9999763851
RRP36	1.1788412298	0.2715892533	0.9999763851
CUL7	-1.0685767374	0.8222644228	0.9999763851
MRPL2	1.3151480869	0.1066079709	0.9999763851
KLC4	-1.0778855142	0.7994648555	0.9999763851
SRF	-1.1869425882	0.1643204028	0.9999763851
CUL9	-1.2441265444	0.277649362	0.9999763851
DNPH1	1.0601421455	0.6478598197	0.9999763851
TTBK1	7.7211724672	0.2779709379	0.9999763851
CRIP3	1.4463034795	0.0533500486	0.9999763851
ZNF318	-1.0671797592	0.6841960465	0.9999763851
ABCC10	-1.0869186916	0.8214477469	0.9999763851
DLK2	1.0314934075	0.9611515549	0.9999763851

TJAP1	-1.2076376767	0.273368086	0.9999763851
LRRC73	-1.3721150467	0.7444800139	0.9999763851
YIPF3	1.5628800426	0.0202834091	0.9999763851
POLR1C	1.0481548662	0.6929190638	0.9999763851
XPO5	-3.4503326209	0.0233320685	0.9999763851
POLH	-1.0214360731	0.8934943258	0.9999763851
GTPBP2	1.0093072281	0.9720846375	0.9999763851
MAD2L1BP	-1.0081116646	0.9640559747	0.9999763851
RSPH9	1.325955166	0.7492862409	0.9999763851
MRPS18A	1.1316574259	0.4280038823	0.9999763851
VEGFA	-1.0049625357	0.9782906021	0.9999763851
C6orf223	1.2096806648	0.3944290945	0.9999763851
MRPL14	1.1221021697	0.3559307774	0.9999763851
TMEM63B	1.0070404208	0.972303037	0.9999763851
CAPN11	3.2356557963	0.5547505991	0.9999763851
SLC29A1	1.0124401578	0.92423441	0.9999763851
HSP90AB1	-1.049514962	0.6665379601	0.9999763851
SLC35B2	-1.0260010535	0.882233389	0.9999763851
NFKBIE	1.1334837816	0.4408688126	0.9999763851
TMEM151B	-1.3955017315	0.689342747	0.9999763851
TCTE1	2.4514020909	0.3850422181	0.9999763851
AARS2	1.3657065372	0.1184646056	0.9999763851
SPATS1	5.4784711963	0.3745358536	0.9999763851
CDC5L	1.037109002	0.8187987601	0.9999763851
SUPT3H	1.250643843	0.3978619388	0.9999763851
RUNX2	-1.0492086453	0.7813075978	0.9999763851
RCAN2	0	0	0
TDRD6	-1.315303824	0.8206171563	0.9999763851
MEP1A	0	0	0
ADGRF1	0	0	0
TNFRSF21	-1.6084677558	0.0400999668	0.9999763851
CD2AP	1.0792387885	0.7070903433	0.9999763851
ADGRF2	1.1896371946	0.7988015917	0.9999763851
ADGRF4	-1.7084677273	0.3890526785	0.9999763851
MUT	-1.1910037127	0.4583296213	0.9999763851
CENPQ	-1.2984387629	0.3833928946	0.9999763851
C6orf141	0	0	0
RHAG	0	0	0
PKHD1	-1.3158816768	0.8203229797	0.9999763851
MCM3	1.2133019234	0.130235486	0.9999763851
PAQR8	1.1379272057	0.5451547124	0.9999763851
EFHC1	-1.1227774568	0.8508022187	0.9999763851
TRAM2	-1.4191632377	0.0419417383	0.9999763851
TMEM14A	-1.0437533583	0.8221339067	0.9999763851
GSTA3	3.2353357581	0.554787377	0.9999763851
GSTA4	-1.0717596186	0.8633478991	0.9999763851
ICK	-1.1203610564	0.7759966146	0.9999763851
FBXO9	1.5105593683	0.006789	0.9999763851
GCM1	-1.7711401125	0.2157917524	0.9999763851

ELOVL5	-1.0474148973	0.7215473631	0.9999763851
GCLC	1.1079033769	0.5507004006	0.9999763851
LRRC1	-1.8368912216	0.0971180894	0.9999763851
MLIP	-7.5647903763	0.2176246281	0.9999763851
TINAG	0	0	0
FAM83B	0	0	0
HMGCLL1	0	0	0
BMP5	0	0	0
COL21A1	-3.3269505881	0.2580204562	0.9999763851
DST	-1.2129285898	0.328418692	0.9999763851
BEND6	1.169174563	0.3982542401	0.9999763851
KIAA1586	-1.0427592776	0.823235729	0.9999763851
ZNF451	-1.0977682612	0.5258135868	0.9999763851
BAG2	1.2760186269	0.468259409	0.9999763851
RAB23	1.1867635619	0.5968835092	0.9999763851
PRIM2	-1.1471719155	0.505948824	0.9999763851
MTRNR2L9	-1.2670579504	0.3402691062	0.9999763851
PTP4A1	-1.1568619607	0.2706848226	0.9999763851
PHF3	-1.0914976412	0.6324546205	0.9999763851
EYS	1.0999689805	0.7689957749	0.9999763851
LOC441155	18.9347928613	0.114046703	0.9999763851
ADGRB3	0	0	0
LMBRD1	-1.4763678669	0.1231893088	0.9999763851
FAM135A	-1.703596772	0.1626616082	0.9999763851
SDHAF4	-2.010880934	0.2148009337	0.9999763851
SMAP1	1.3216296414	0.143461253	0.9999763851
OGFRL1	1.2291366728	0.473155489	0.9999763851
RIMS1	-4.2849908816	0.4028453076	0.9999763851
KCNQ5	-1.6123994012	0.0119665963	0.9999763851
KHDC1L	3.2356557963	0.5547505991	0.9999763851
KHDC1	-1.0306365966	0.9303566738	0.9999763851
DDX43	0	0	0
MB21D1	-1.9610914961	0.0155348571	0.9999763851
MTO1	1.7074391115	0.0901637141	0.9999763851
EEF1A1	-1.1994300987	0.166763282	0.9999763851
SLC17A5	1.2207096619	0.207676873	0.9999763851
CD109	-10.8385246611	0.1453015101	0.9999763851
COX7A2	1.2358646627	0.10241246	0.9999763851
TMEM30A	1.0126600474	0.930727631	0.9999763851
FILIP1	1.8140812535	0.5878146406	0.9999763851
SENP6	-1.8111234909	0.0127677396	0.9999763851
MYO6	0	0	0
MEI4	3.2356557963	0.5547505991	0.9999763851
IRAK1BP1	-1.0815224328	0.9262924998	0.9999763851
PHIP	-1.2061432484	0.2384960355	0.9999763851
TTK	1.0583253094	0.7893276165	0.9999763851
BCKDHB	1.1378874951	0.7093366969	0.9999763851
FAM46A	1.0892111776	0.6887004191	0.9999763851
IBTK	-1.1084036985	0.5998623255	0.9999763851

TPBG	-4.2849908816	0.4028453076	0.9999763851
UBE3D	1.0351109789	0.9296290841	0.9999763851
DOPEY1	-1.2525198743	0.6385929001	0.9999763851
PGM3	-1.4479946306	0.1338797839	0.9999763851
RWDD2A	1.8888297566	0.5937927916	0.9999763851
ME1	1.3703087869	0.3489766819	0.9999763851
CYB5R4	1.730418368	0.1664685948	0.9999763851
MRAP2	0	0	0
CEP162	-1.4341321573	0.4685405061	0.9999763851
SNX14	-1.1942361256	0.3294395799	0.9999763851
SYNCRIP	1.2176558038	0.1223751758	0.9999763851
LOC107986532	-1.0807156495	0.9291988419	0.9999763851
ZNF292	-1.4301043057	0.0703579246	0.9999763851
GJB7	0	0	0
SMIM8	-1.2527940068	0.5262642636	0.9999763851
C6orf163	7.7211724672	0.2779709379	0.9999763851
SLC35A1	-1.0663356269	0.7735933849	0.9999763851
RARS2	1.4931995045	0.1702026761	0.9999763851
ORC3	1.0218846004	0.9245136806	0.9999763851
AKIRIN2	-1.241328187	0.4635220948	0.9999763851
RNGTT	1.350549265	0.1759765583	0.9999763851
PNRC1	-1.5399169699	0.0861251539	0.9999763851
PM20D2	1.2243377737	0.1977508311	0.9999763851
GABRR1	0	0	0
UBE2J1	1.0836087475	0.6198539499	0.9999763851
RRAGD	-1.0165675422	0.933353489	0.9999763851
ANKRD6	-4.0207005432	0.0447300815	0.9999763851
LYRM2	1.0053063086	0.979472967	0.9999763851
MDN1	1.0800354728	0.6316413815	0.9999763851
CASP8AP2	-1.5492547216	0.109530628	0.9999763851
BACH2	-2.8987629076	0.0620455048	0.9999763851
MAP3K7	-1.2838378668	0.1556178771	0.9999763851
EPHA7	0	0	0
MANEA	1.4124334722	0.4083499387	0.9999763851
UFL1	-1.5846674331	0.1647542822	0.9999763851
GPR63	1.3580191471	0.5962668121	0.9999763851
NDUFAF4	1.351638631	0.3319358051	0.9999763851
KLHL32	2.8679346912	0.2849790884	0.9999763851
MMS22L	-1.2287749538	0.4726297733	0.9999763851
FBXL4	-1.3081859926	0.4237864019	0.9999763851
COQ3	1.9932608239	0.0194599068	0.9999763851
PNISR	-1.3079541648	0.0974398984	0.9999763851
TSTD3	-1.131876121	0.7218770577	0.9999763851
USP45	-1.4638331383	0.624152621	0.9999763851
CCNC	1.2788335761	0.1941539081	0.9999763851
PRDM13	0	0	0
ASCC3	1.2143531641	0.3617331803	0.9999763851
LOC102724422	0	0	0
GRIK2	5.4776292235	0.3745831165	0.9999763851

HACE1	1.2473684242	0.6794463821	0.9999763851
LIN28B	-4.2849908816	0.4028453076	0.9999763851
BVES	3.2356557963	0.5547505991	0.9999763851
PREP	1.1059939742	0.6058563353	0.9999763851
PRDM1	-2.3530139268	0.0663961605	0.9999763851
ATG5	-1.0158620999	0.9529249312	0.9999763851
AIM1	1.3137035423	0.1784261115	0.9999763851
RTN4IP1	1.0279179327	0.8974401616	0.9999763851
QRSL1	1.6330003008	0.1622756277	0.9999763851
CD24	1.1285928346	0.7693268702	0.9999763851
C6orf203	1.0500438305	0.8255166149	0.9999763851
BEND3	1.1299140175	0.7763637691	0.9999763851
PDSS2	1.3120687954	0.167728114	0.9999763851
SOBP	1.6316087781	0.1573700897	0.9999763851
SCML4	-4.2849908816	0.4028453076	0.9999763851
SEC63	-1.2515762906	0.1529774511	0.9999763851
OSTM1	-1.1486603698	0.6148770227	0.9999763851
SNX3	1.196106392	0.3964735143	0.9999763851
LACE1	1.1727168467	0.7269856526	0.9999763851
FOXO3	1.0888147867	0.64250281	0.9999763851
ARMC2	1.0749377199	0.7937397887	0.9999763851
SESN1	-1.1319081309	0.8237118329	0.9999763851
CEP57L1	1.3441803356	0.356975107	0.9999763851
LOC105377934	1.2921998209	0.6032579091	0.9999763851
LOC100996634	1.8140611791	0.5846844351	0.9999763851
CD164	1.2208525338	0.1733465654	0.9999763851
PPIL6	1.2179289398	0.6929640669	0.9999763851
SMPD2	-1.2647120687	0.5909564232	0.9999763851
MICAL1	1.226230989	0.3713420352	0.9999763851
ZBTB24	-1.6013245408	0.3751386787	0.9999763851
AK9	-1.4742186188	0.513609135	0.9999763851
FIG4	-1.4359415136	0.3878882242	0.9999763851
WASF1	-1.2341042692	0.345177248	0.9999763851
CDC40	1.3142478017	0.254109296	0.9999763851
METTL24	-4.2849908816	0.4028453076	0.9999763851
DDO	-1.2297276897	0.7514173651	0.9999763851
SLC22A16	1.0962555457	0.7395877126	0.9999763851
CDK19	-1.0952444576	0.5979874212	0.9999763851
AMD1	1.205527965	0.2001795839	0.9999763851
GTF3C6	1.0169736326	0.9053530751	0.9999763851
RPF2	1.0976697574	0.6317361314	0.9999763851
SLC16A10	1.6215399586	0.1097487974	0.9999763851
MFSD4B	-2.0967354172	0.2624298551	0.9999763851
REV3L	-1.1948108063	0.5074012391	0.9999763851
TRAF3IP2	1.1518009378	0.591536374	0.9999763851
FYN	1.0046315933	0.9920383047	0.9999763851
TUBE1	1.7657721624	0.172729343	0.9999763851
FAM229B	-1.3280998313	0.5340536917	0.9999763851
MARCKS	1.1308916209	0.3407246766	0.9999763851

HDAC2	1.1501019878	0.3584023448	0.9999763851
FRK	-4.2864494036	0.4027150577	0.9999763851
NT5DC1	1.238749935	0.4887214752	0.9999763851
COL10A1	0	0	0
TSPYL4	-1.2207995474	0.6968644478	0.9999763851
DSE	1.4469538193	0.1036669902	0.9999763851
TSPYL1	1.2409039469	0.306831724	0.9999763851
FAM26D	0	0	0
RWDD1	-1.1886723202	0.3079024293	0.9999763851
RSPH4A	-1.3154339304	0.8205576571	0.9999763851
ZUFSP	-1.2213614463	0.663616369	0.9999763851
KPNA5	1.6082661877	0.1743862546	0.9999763851
ROS1	-1.966276531	0.4565057531	0.9999763851
DCBLD1	-1.3760918252	0.4448069427	0.9999763851
GOPC	-1.0987264323	0.6162387525	0.9999763851
NUS1	1.1627120146	0.4332822575	0.9999763851
SLC35F1	1.3848774703	0.1911237801	0.9999763851
CEP85L	1.4881741755	0.3488928894	0.9999763851
PLN	0	0	0
MCM9	1.7037661383	0.1886410815	0.9999763851
ASF1A	1.1852952967	0.5581023769	0.9999763851
TBC1D32	-1.0476390497	0.9474907295	0.9999763851
GJA1	0	0	0
HSF2	-1.2812372033	0.2723198375	0.9999763851
SERINC1	-1.0847337247	0.713795971	0.9999763851
PKIB	7.7211724672	0.2779709379	0.9999763851
FABP7	0	0	0
SMPDL3A	-3.3295429242	0.2520819079	0.9999763851
TRDN	10.4285443305	0.2414635521	0.9999763851
NKAIN2	-1.3157515262	0.8223941627	0.9999763851
RNF217	0	0	0
TPD52L1	-4.2864494036	0.4027150577	0.9999763851
HDDC2	1.1686767847	0.3538930219	0.9999763851
NCOA7	1.2756840241	0.2476794175	0.9999763851
HINT3	-1.0191924291	0.9268747613	0.9999763851
TRMT11	-1.2922129281	0.4208201658	0.9999763851
CENPW	1.1005936778	0.5162379684	0.9999763851
RSPO3	0	0	0
RNF146	1.1293715103	0.6040782753	0.9999763851
ECHDC1	-1.3867458064	0.0791697995	0.9999763851
KIAA0408	-20.6735088573	0.0596784778	0.9999763851
SOGA3	-1.3158816768	0.8203229797	0.9999763851
C6orf58	0	0	0
LAMA2	0	0	0
ARHGAP18	1.0875837793	0.7428036023	0.9999763851
L3MBTL3	2.6536751413	0.0846132643	0.9999763851
SAMD3	0	0	0
EPB41L2	0	0	0
ARG1	-1.3945372452	0.6923954461	0.9999763851

MED23	-1.2888772595	0.4000088184	0.9999763851
ENPP3	0	0	0
CTAGE9	0	0	0
ENPP1	0	0	0
STX7	-1.0264157338	0.9180024705	0.9999763851
VNN2	0	0	0
SLC18B1	0	0	0
RPS12	-1.0908391691	0.4604119943	0.9999763851
EYA4	1.3576108079	0.7958575571	0.9999763851
TBPL1	-1.0019981827	0.9953135518	0.9999763851
SLC2A12	-1.0801101658	0.9305702272	0.9999763851
SGK1	1.2245528207	0.6379606866	0.9999763851
ALDH8A1	1.7868451227	0.5987183051	0.9999763851
HBS1L	1.1917185729	0.3810967979	0.9999763851
MYB	-1.4143075971	0.008165	0.9999763851
AHI1	-1.1943993967	0.5035170024	0.9999763851
PDE7B	0	0	0
MTFR2	1.2473224675	0.3075865547	0.9999763851
BCLAF1	1.1728448645	0.2132735595	0.9999763851
MAP7	-1.3304807702	0.3284699732	0.9999763851
MAP3K5	-1.003712937	0.9819582226	0.9999763851
PEX7	-1.2156517911	0.5966166723	0.9999763851
IFNGR1	1.3454004999	0.434598124	0.9999763851
TNFAIP3	-1.7230252927	0.0608266532	0.9999763851
ARFGEF3	-1.035203499	0.9620562309	0.9999763851
HEBP2	-1.0707222049	0.6705591536	0.9999763851
NHSL1	-1.0884255882	0.8126876408	0.9999763851
GVQW2	-1.3717838846	0.749694777	0.9999763851
CCDC28A	-1.4969762645	0.0508750907	0.9999763851
ECT2L	1.3500680352	0.6816657837	0.9999763851
REPS1	1.0208529483	0.9142969021	0.9999763851
ABRACL	-1.1634888863	0.1996821567	0.9999763851
HECA	-1.2751658844	0.1906799201	0.9999763851
TXLNB	-2.6583180448	0.2228894538	0.9999763851
CITED2	-1.0361213583	0.8533535631	0.9999763851
NMBR	5.4770877341	0.3746135179	0.9999763851
VTA1	1.0777272804	0.6880954873	0.9999763851
HIVEP2	-1.0724400783	0.7179205181	0.9999763851
AIG1	1.6862503526	0.003915	0.9999763851
ADAT2	1.0057349704	0.9793023517	0.9999763851
PEX3	-1.0946458236	0.6967839079	0.9999763851
FUCA2	1.207460313	0.3549507106	0.9999763851
PHACTR2	-1.3068227128	0.3061450378	0.9999763851
LTV1	1.3896833392	0.1858319533	0.9999763851
PLAGL1	-1.4466165318	0.2011689131	0.9999763851
SF3B5	1.0474717302	0.7537655678	0.9999763851
STX11	-1.1789827383	0.6645183531	0.9999763851
UTRN	-1.0010697271	0.9959173065	0.9999763851
EPM2A	1.3411938723	0.7190877752	0.9999763851

FBXO30	1.2803327132	0.4753208115	0.9999763851
SHPRH	-1.3730257125	0.3371568481	0.9999763851
GRM1	0	0	0
RAB32	1.6716607621	0.007951	0.9999763851
ADGB	-2.2172342137	0.240633903	0.9999763851
STXBP5	1.039232723	0.8070730387	0.9999763851
SASH1	-1.506459725	0.2560554472	0.9999763851
UST	1.2280506827	0.7913354969	0.9999763851
TAB2	-1.3662940011	0.1165347147	0.9999763851
ZC3H12D	-1.2488104335	0.6000278683	0.9999763851
PPIL4	1.4585017579	0.1649458145	0.9999763851
GINM1	-1.4379340561	0.4507102514	0.9999763851
KATNA1	-1.5371062944	0.0369350778	0.9999763851
LATS1	-1.2120781	0.5065755597	0.9999763851
NUP43	1.5618587269	0.0174950989	0.9999763851
PCMT1	1.0190438243	0.8808168542	0.9999763851
LRP11	1.087401112	0.6699597928	0.9999763851
RAET1E	1.3258969121	0.7482148046	0.9999763851
RAET1G	-1.9647456674	0.4667580718	0.9999763851
ULBP2	17.8309472943	0.1311012957	0.9999763851
ULBP1	0	0	0
ULBP3	3.2353357581	0.554787377	0.9999763851
PPP1R14C	-1.3535347227	0.1211577436	0.9999763851
PLEKHG1	-1.3713018672	0.7312784555	0.9999763851
MTHFD1L	1.1677072628	0.3444221413	0.9999763851
AKAP12	3.2353357581	0.554787377	0.9999763851
ZBTB2	1.0178172013	0.9584263534	0.9999763851
RMND1	1.6082665467	0.0989129975	0.9999763851
ARMT1	1.7995737228	0.0784539531	0.9999763851
CCDC170	10.1794135333	0.2227082573	0.9999763851
SYNE1	-2.387679463	0.250963581	0.9999763851
MYCT1	0	0	0
FBXO5	-1.1059803881	0.6735589113	0.9999763851
MTRF1L	1.3858279321	0.1012554968	0.9999763851
RGS17	0	0	0
IPCEF1	-1.196159321	0.5447872011	0.9999763851
CNKSR3	1.1458418283	0.7335384756	0.9999763851
SCAF8	1.0455429258	0.8122677138	0.9999763851
TFB1M	-1.033779693	0.8784563299	0.9999763851
NOX3	0	0	0
ARID1B	1.3263303579	0.1116515092	0.9999763851
TMEM242	1.2404845018	0.3656965958	0.9999763851
ZDHHC14	-1.1359847401	0.537617409	0.9999763851
SNX9	-1.5825973022	0.2432679778	0.9999763851
SYNJ2	1.1304465917	0.5089536125	0.9999763851
SERAC1	1.2400199432	0.6259876835	0.9999763851
GTF2H5	-1.0605960473	0.7971014152	0.9999763851
TULP4	-1.027905502	0.8978640112	0.9999763851
TMEM181	-1.0214938428	0.9367116225	0.9999763851

DYNLT1	1.3591633902	0.1281995316	0.9999763851
SYTL3	2.5943187527	0.4291761521	0.9999763851
EZR	1.0799653326	0.5813778945	0.9999763851
C6orf99	-3.6747177393	0.1929122622	0.9999763851
RSPH3	1.7077281375	0.1198766276	0.9999763851
TAGAP	1.2403990118	0.274098919	0.9999763851
SOD2	1.0165210628	0.9067569597	0.9999763851
WTAP	1.2837840517	0.0862048231	0.9999763851
ACAT2	-1.5236417268	0.1878059385	0.9999763851
TCP1	1.1035520587	0.400917535	0.9999763851
MRPL18	1.0386082935	0.7974875655	0.9999763851
PNLDC1	-2.5588825616	0.3182945072	0.9999763851
IGF2R	-1.4328453811	0.0191503346	0.9999763851
SLC22A1	1.3264789913	0.7365304439	0.9999763851
SLC22A3	-4.2849908816	0.4028453076	0.9999763851
MAP3K4	-1.3281093608	0.1304341216	0.9999763851
AGPAT4	-1.2101498166	0.3928323733	0.9999763851
PARK2	-1.4073512753	0.6477548675	0.9999763851
PACRG	-2.3214980365	0.4361267576	0.9999763851
QKI	-1.2236603551	0.1707430916	0.9999763851
C6orf118	0	0	0
PDE10A	0	0	0
PRR18	0	0	0
SFT2D1	1.0909647162	0.574051668	0.9999763851
MPC1	1.1037081178	0.5712580278	0.9999763851
RPS6KA2	1.088224954	0.7776854976	0.9999763851
RNASET2	1.0139044383	0.9279685604	0.9999763851
FGFR1OP	-1.106691354	0.6922730782	0.9999763851
TCP10L2	0	0	0
THBS2	3.7878555937	0.1335689782	0.9999763851
WDR27	-1.0435173128	0.900681031	0.9999763851
C6orf120	1.4222302241	0.0854055231	0.9999763851
PHF10	-1.2552056841	0.3395919942	0.9999763851
TCTE3	1.6852133521	0.3567744131	0.9999763851
ERMARD	-1.0217536457	0.9461462251	0.9999763851
DLL1	-1.0159162816	0.9563944047	0.9999763851
FAM120B	1.2077284526	0.3975677018	0.9999763851
PSMB1	-1.2229389378	0.1098652498	0.9999763851
TBP	-1.1755004017	0.5748029008	0.9999763851
PDCD2	1.2266835514	0.1787451049	0.9999763851
LOC101929692	-1.3943388833	0.6978395358	0.9999763851
LOC105375112	-2.6392692278	0.2037510509	0.9999763851
FAM20C	-1.3746319532	0.1420402549	0.9999763851
PDGFA	1.4677562844	0.4921005175	0.9999763851
PRKAR1B	1.304572789	0.2529036119	0.9999763851
DNAAF5	-1.4620686098	0.0444845339	0.9999763851
SUN1	-1.229310616	0.442398656	0.9999763851
GET4	-1.0495872442	0.8049029128	0.9999763851
ADAP1	1.2006607582	0.4051123297	0.9999763851

COX19	1.1490664863	0.3852750456	0.9999763851
C7orf50	-1.0286649341	0.8446517921	0.9999763851
GPR146	-1.3285230121	0.5504873399	0.9999763851
GPER1	1.2865109866	0.8332890582	0.9999763851
ZFAND2A	1.0465430187	0.8788128453	0.9999763851
UNCX	3.2353357581	0.554787377	0.9999763851
MICALL2	1.1205508003	0.7371148509	0.9999763851
INTS1	1.0893398421	0.7909095996	0.9999763851
MAFK	-1.0353512704	0.8998782062	0.9999763851
PSMG3	-1.4213609899	0.0115694198	0.9999763851
ELFN1	1.0302684927	0.9328343505	0.9999763851
MAD1L1	1.1134167364	0.5000522479	0.9999763851
MRM2	-1.1423661451	0.5782057211	0.9999763851
NUDT1	1.2627479314	0.1641686371	0.9999763851
SNX8	1.2429741178	0.2389987227	0.9999763851
EIF3B	1.1494704623	0.2993822495	0.9999763851
CHST12	-1.2147667984	0.3694418207	0.9999763851
LFNG	-1.2863371215	0.4744010428	0.9999763851
BRAT1	1.1811938198	0.3962921104	0.9999763851
IQCE	1.5950137501	0.1926779545	0.9999763851
TTYH3	-1.1838460142	0.3147666812	0.9999763851
AMZ1	-1.6845306815	0.1404340331	0.9999763851
GNA12	-1.1564270927	0.3101802877	0.9999763851
SDK1	-1.6135797963	0.0444410753	0.9999763851
LOC105375131	0	0	0
FOXK1	1.0460439162	0.7643410799	0.9999763851
AP5Z1	1.1356374427	0.4557332701	0.9999763851
RADIL	1.2659578115	0.5728551857	0.9999763851
RBAK	1.1107271141	0.8344933529	0.9999763851
RBAK-RBAKDN	-1.1271483256	0.8699978408	0.9999763851
WIPI2	1.0790050059	0.6138945473	0.9999763851
SLC29A4	3.392655372	0.2312814813	0.9999763851
TNRC18	-1.0497135041	0.7659396233	0.9999763851
FBXL18	-1.0221272571	0.9279937042	0.9999763851
ACTB	1.0680961438	0.5753255288	0.9999763851
FSCN1	-2.5593885838	0.3087159846	0.9999763851
LOC107986762	1.2121405506	0.6313524744	0.9999763851
RNF216	1.0532468783	0.7443640488	0.9999763851
OCM	-4.2864494036	0.4027150576	0.9999763851
CCZ1	1.204740328	0.3199103008	0.9999763851
PMS2	1.0272428791	0.9139102564	0.9999763851
AIMP2	-1.5847923668	0.0117694623	0.9999763851
EIF2AK1	1.0335605711	0.8253487333	0.9999763851
USP42	1.1266788205	0.6918528206	0.9999763851
CYTH3	-1.0059429769	0.9810185437	0.9999763851
FAM220A	1.0398188398	0.8531052781	0.9999763851
RAC1	1.2437852244	0.0718189011	0.9999763851
DAGLB	-1.329805302	0.212746387	0.9999763851
KDELR2	-1.0618250649	0.6749785842	0.9999763851

GRID2IP	0	0	0
ZDHHC4	1.374991703	0.2481159832	0.9999763851
C7orf26	1.0080017172	0.9727504268	0.9999763851
ZNF853	1.8888297566	0.5937856039	0.9999763851
ZNF316	-1.119083406	0.5223625992	0.9999763851
ZNF12	-1.2047687689	0.389945581	0.9999763851
RSPH10B2	0	0	0
CCZ1B	-1.127945212	0.4879294808	0.9999763851
C1GALT1	-1.2410766168	0.2994965975	0.9999763851
MIOS	-1.0571491483	0.8365334947	0.9999763851
RPA3	1.1247752168	0.4124502373	0.9999763851
UMAD1	-1.1047346439	0.7098880836	0.9999763851
GLCCI1	1.1152747909	0.5213669277	0.9999763851
ICA1	-2.4959539324	0.0206445382	0.9999763851
NXPH1	0	0	0
NDUFA4	1.3973154398	0.0152409492	0.9999763851
PHF14	1.0299579949	0.8602793657	0.9999763851
THSD7A	3.2353357581	0.554787377	0.9999763851
TMEM106B	-1.3886499572	0.3428994851	0.9999763851
ARL4A	1.024184787	0.921639489	0.9999763851
SOSTDC1	0	0	0
ANKMY2	-1.1152182159	0.7211177889	0.9999763851
BZW2	-1.1080828312	0.5498094987	0.9999763851
TSPAN13	0	0	0
AHR	-1.4850263799	0.1724200511	0.9999763851
SNX13	-1.051947998	0.8829972058	0.9999763851
HDAC9	0	0	0
TWISTNB	1.4045408894	0.0406468179	0.9999763851
MACC1	1.5486451741	0.3619847107	0.9999763851
SP4	1.313059784	0.5043302541	0.9999763851
DNAH11	-1.133152361	0.7575687991	0.9999763851
CDCA7L	1.0389006805	0.7973914388	0.9999763851
RAPGEF5	0	0	0
TOMM7	-1.231791851	0.0941943248	0.9999763851
FAM126A	1.3220487992	0.221827423	0.9999763851
KLHL7	-1.1347366538	0.6363949736	0.9999763851
NUPL2	-1.6433117329	0.0703872288	0.9999763851
GPNMB	-1.6201312029	0.003014	0.9999763851
MALSU1	1.2938253468	0.132130534	0.9999763851
IGF2BP3	1.1438678081	0.5157725994	0.9999763851
TRA2A	-1.215350831	0.1669181876	0.9999763851
CCDC126	1.0866224378	0.8474746166	0.9999763851
FAM221A	0	0	0
STK31	0	0	0
LOC107986777	0	0	0
MPP6	-1.7727550014	0.1821757109	0.9999763851
DFNA5	3.2353357581	0.554787377	0.9999763851
OSBPL3	-1.3015993756	0.3137132845	0.9999763851
CYCS	1.348888814	0.0217302403	0.9999763851

C7orf31	-1.086337313	0.8518103479	0.9999763851
LOC646588	-1.5326255354	0.3273559566	0.9999763851
NFE2L3	-1.2680859889	0.4626954618	0.9999763851
HNRNPA2B1	1.1624967127	0.3188248218	0.9999763851
CBX3	1.0330378287	0.7837102051	0.9999763851
SNX10	1.2962543332	0.0746438689	0.9999763851
C7orf71	0	0	0
SKAP2	1.0850232822	0.6231097707	0.9999763851
HOXA1	-2.0624023724	0.3159041639	0.9999763851
HOXA2	1.8139617088	0.5773955095	0.9999763851
HOXA3	1.1979476908	0.7131292907	0.9999763851
HOXA4	-1.6817502083	0.4753426566	0.9999763851
HOXA5	1.4284463338	0.3964082208	0.9999763851
HOXA6	0	0	0
HOXA7	1.5678512442	0.2011211293	0.9999763851
HOXA9	-1.4231209207	0.0732854132	0.9999763851
HOXA10	-1.1047881842	0.4954656902	0.9999763851
HOXA11	-1.0806221785	0.6663107186	0.9999763851
HOXA13	-1.0593234938	0.8755017111	0.9999763851
EVX1	1.2869488883	0.8196978268	0.9999763851
HIBADH	-1.4119049299	0.217173095	0.9999763851
TAX1BP1	1.0579613571	0.6476879451	0.9999763851
JAZF1	-1.3902764042	0.1283912589	0.9999763851
CREB5	-2.3225701307	0.4434188202	0.9999763851
CPVL	-1.0966514822	0.7040599468	0.9999763851
CHN2	1.1179927641	0.7683746224	0.9999763851
PRR15	3.2356557963	0.5547505991	0.9999763851
LOC101928208	0	0	0
WIPF3	3.2353357581	0.554787377	0.9999763851
FKBP14	-1.2170633691	0.641733647	0.9999763851
PLEKHA8	1.2993674237	0.5924896239	0.9999763851
MTURN	-1.1603790588	0.4485720602	0.9999763851
ZNRF2	-1.5631097096	0.0865154286	0.9999763851
NOD1	1.2710994888	0.4072166317	0.9999763851
LOC101928268	2.8946978995	0.3713424171	0.9999763851
GGCT	-1.1084568192	0.4477798508	0.9999763851
GARS	1.1059617356	0.5053268989	0.9999763851
FAM188B	3.2356557963	0.5547505991	0.9999763851
CCDC129	3.2353357581	0.554787377	0.9999763851
LSM5	1.0098540477	0.9434868349	0.9999763851
AVL9	-1.0979690673	0.5600040563	0.9999763851
KBTBD2	-1.1128294411	0.6244685223	0.9999763851
FKBP9	2.5194489141	0.2710522815	0.9999763851
NT5C3A	-1.0314720559	0.8536062362	0.9999763851
RP9	-1.210295034	0.375377743	0.9999763851
BBS9	-1.0967436785	0.8470930984	0.9999763851
DPY19L1	-1.198464454	0.4522853041	0.9999763851
TBX20	3.2353357581	0.554787377	0.9999763851
HERPUD2	-1.1662609782	0.5603819431	0.9999763851

SEPT7	1.0682474528	0.6057566305	0.9999763851
EEPD1	1.057563572	0.7859715397	0.9999763851
KIAA0895	-3.0826121034	0.104637516	0.9999763851
ANLN	-1.5004737976	0.0575964775	0.9999763851
AOAH	1.0337794545	0.9247821455	0.9999763851
ELMO1	-1.8383664782	0.009758	0.9999763851
GPR141	1.6845813354	0.1489837267	0.9999763851
NME8	4.9736751744	0.1121871596	0.9999763851
SFRP4	3.2353357581	0.554787377	0.9999763851
EPDR1	1.1458237419	0.4732568993	0.9999763851
STARD3NL	-1.1599549494	0.4407167629	0.9999763851
TARP	1.2730653865	0.408096528	0.9999763851
AMPH	1.224539777	0.758670947	0.9999763851
VPS41	-1.1282015585	0.573760314	0.9999763851
POU6F2	-4.2849908816	0.4028453076	0.9999763851
YAE1D1	-1.0196651264	0.9501900046	0.9999763851
RALA	1.095492144	0.5900677156	0.9999763851
CDK13	1.2752800796	0.3063835027	0.9999763851
MPLKIP	1.3939085476	0.0981958103	0.9999763851
SUGCT	-2.0001755787	0.100419344	0.9999763851
INHBA	-12.7231911949	3.09E-14	5.54E-10
GLI3	3.2353357581	0.554787377	0.9999763851
C7orf25	-1.1378395056	0.7828604638	0.9999763851
PSMA2	1.1706611118	0.2380284777	0.9999763851
MRPL32	1.0334710135	0.8854176074	0.9999763851
HECW1	3.9179071451	0.1947667958	0.9999763851
STK17A	-1.2191700914	0.4746847413	0.9999763851
COA1	1.0054088224	0.9721329502	0.9999763851
BLVRA	1.1789463547	0.4026653141	0.9999763851
MRPS24	1.0664325442	0.6743878102	0.9999763851
URGCP-MRPS24	17.0964048721	0.1497629194	0.9999763851
URGCP	1.0468936009	0.8888735338	0.9999763851
LOC107986791	3.1108254173	0.2256589065	0.9999763851
UBE2D4	2.3705921744	0.005659	0.9999763851
SPDYE1	25.6678580218	0.0811866116	0.9999763851
DBNL	1.1531973685	0.5346928854	0.9999763851
PGAM2	-14.1218219134	0.1013475713	0.9999763851
POLM	2.0266506433	0.1473179989	0.9999763851
POLD2	1.0026928218	0.9867681316	0.9999763851
GCK	5.4770877341	0.3746135179	0.9999763851
YKT6	1.143714748	0.4549882942	0.9999763851
NUDCD3	1.0417061798	0.7322824367	0.9999763851
NPC1L1	0	0	0
DDX56	1.0123323431	0.948168058	0.9999763851
TMED4	1.0759285031	0.6529610637	0.9999763851
OGDH	1.0926219429	0.658103219	0.9999763851
ZMIZ2	1.0275830709	0.8842294661	0.9999763851
PPIA	1.1314256047	0.2861199226	0.9999763851
H2AFV	1.097995633	0.4858648736	0.9999763851

PURB	1.0884674369	0.6642499686	0.9999763851
MYO1G	-1.1744664923	0.4828918037	0.9999763851
CCM2	1.1374873956	0.4906748608	0.9999763851
NACAD	0	0	0
TBRG4	1.2109207131	0.2419167053	0.9999763851
ADCY1	1.4854101668	0.2569611734	0.9999763851
TNS3	-1.0646944547	0.7501007794	0.9999763851
PKD1L1	-1.3012214104	0.6239224869	0.9999763851
HUS1	1.1335363412	0.64352396	0.9999763851
SUN3	1.9239491811	0.433246221	0.9999763851
UPP1	-1.8524539147	0.0517927113	0.9999763851
ABCA13	1.4214641047	0.6299148796	0.9999763851
IKZF1	1.1803764552	0.2181061867	0.9999763851
FIGNL1	-1.0359786311	0.8407326173	0.9999763851
DDC	1.6253118249	0.5750177155	0.9999763851
GRB10	1.4136037747	0.0214759489	0.9999763851
COBL	1.6243491221	0.5664322505	0.9999763851
SEC61G	-1.5986312416	0.004749	0.9999763851
LANCL2	1.7083674139	0.0790142082	0.9999763851
VOPP1	-1.1258158404	0.5576034844	0.9999763851
LOC101060341	0	0	0
SEPT14	0	0	0
ZNF713	2.4170276959	0.1758547997	0.9999763851
MRPS17	1.2128111819	0.4116369404	0.9999763851
GBAS	1.1194606158	0.4824879695	0.9999763851
PSPH	1.1382185409	0.5371136662	0.9999763851
CCT6A	1.1947851875	0.1711798835	0.9999763851
SUMF2	-1.6872825634	0.2054476845	0.9999763851
PHKG1	1.2669721097	0.2491935427	0.9999763851
CHCHD2	-1.1479022718	0.2643242381	0.9999763851
LOC100287294	0	0	0
ZNF479	0	0	0
LOC107986805	2.3406309168	0.4060501105	0.9999763851
ZNF735	0	0	0
ZNF679	0	0	0
ZNF736	-4.2849908816	0.4028453076	0.9999763851
ZNF680	-1.4888352741	0.2069827013	0.9999763851
ZNF107	-1.0508956747	0.84290331	0.9999763851
ZNF138	-1.0729018924	0.8690422057	0.9999763851
ZNF273	-1.2547157149	0.3981182387	0.9999763851
ZNF117	0	0	0
LOC107986705	-2.1156977606	0.1655422058	0.9999763851
ZNF92	-1.8291300434	0.0855494284	0.9999763851
LOC101929322	-1.9480990641	0.3316726962	0.9999763851
VKORC1L1	1.013816593	0.9363214399	0.9999763851
GUSB	1.3010864341	0.0814449706	0.9999763851
ASL	1.5460012317	0.0920076971	0.9999763851
CRCP	1.1654884662	0.3399902737	0.9999763851
TPST1	-5.3347590109	8.82E-05	0.1438827417

KCTD7	-1.1240552884	0.5484778446	0.9999763851
RABGEF1	1.0680353064	0.7216677111	0.9999763851
TMEM248	1.034763318	0.8205642058	0.9999763851
SBDS	1.1482245208	0.6236361747	0.9999763851
TYW1	-1.052980845	0.7977673666	0.9999763851
AUTS2	3.2361532718	0.5546934395	0.9999763851
CALN1	2.1743612496	0.3142134991	0.9999763851
TYW1B	1.1916049594	0.6783498221	0.9999763851
POM121	1.2883535175	0.0535239316	0.9999763851
TRIM74	1.0116836987	0.9385654264	0.9999763851
SPDYE11	0	0	0
NSUN5	1.028682763	0.8356754949	0.9999763851
FKBP6	-7.5647903763	0.2176246281	0.9999763851
FZD9	1.0300813919	0.9662262396	0.9999763851
BAZ1B	1.2721596092	0.1191175671	0.9999763851
BCL7B	1.5568038595	0.0638081998	0.9999763851
TBL2	-1.2027232664	0.3902448502	0.9999763851
VPS37D	-14.1235077517	0.0960269008	0.9999763851
DNAJC30	1.2421084522	0.6190204294	0.9999763851
WBSCR22	-1.0271873135	0.8701588163	0.9999763851
STX1A	-1.7346211034	0.4442349129	0.9999763851
ABHD11	-1.1490291748	0.6727019035	0.9999763851
CLDN4	1.2869488883	0.8198374627	0.9999763851
WBSCR27	-4.2849908816	0.4028453076	0.9999763851
LIMK1	-1.0486645347	0.8593613004	0.9999763851
EIF4H	1.2164068878	0.1381167362	0.9999763851
LAT2	1.1224522092	0.3680278282	0.9999763851
RFC2	1.0855980361	0.6541270068	0.9999763851
CLIP2	-1.8616301137	0.2899918013	0.9999763851
GTF2I	1.3724467001	0.0124781066	0.9999763851
NCF1	1.2302557502	0.4636179342	0.9999763851
GTF2IRD2	1.2403673619	0.3196941717	0.9999763851
GATSL2	-1.0897331625	0.8620339786	0.9999763851
RCC1L	1.4676966812	0.017320005	0.9999763851
GTF2IRD2B	-1.9054160201	0.2181824354	0.9999763851
TRIM73	1.3652112359	0.519848658	0.9999763851
POM121C	-1.1312570689	0.4750681582	0.9999763851
SPDYE5	6.368054416	0.3491682726	0.9999763851
HIP1	-1.3376808546	0.0871746469	0.9999763851
CCL26	1.287203384	0.8310555568	0.9999763851
CCL24	0	0	0
RHBDD2	-1.0263862488	0.9112103021	0.9999763851
POR	-1.0156303811	0.9311682612	0.9999763851
TMEM120A	-1.3926190429	0.0593785979	0.9999763851
STYXL1	1.2550338396	0.3200630987	0.9999763851
MDH2	-1.2823587699	0.0614451096	0.9999763851
LOC107986711	3.2353357581	0.554787377	0.9999763851
SRRM3	-2.2378380635	0.2934725787	0.9999763851
HSPB1	1.2215632519	0.1217406919	0.9999763851

YWHAG	-1.0092469091	0.9413345488	0.9999763851
SSC4D	-1.3155492632	0.8261713847	0.9999763851
ZP3	78.1389315045	0.0227503897	0.9999763851
DTX2	-1.00749963	0.9754354981	0.9999763851
LOC105375355	-1.3948129396	0.6825391405	0.9999763851
SPDYE16	-2.5519849925	0.3670892633	0.9999763851
POMZP3	-1.1602744916	0.4049682728	0.9999763851
SPDYE17	-1.3956396272	0.7086142632	0.9999763851
SPDYE18	3.2356557963	0.5547505991	0.9999763851
CCDC146	-1.1227023204	0.8439040104	0.9999763851
FGL2	-1.3158816768	0.8203229797	0.9999763851
GSAP	-1.2927829929	0.3357304937	0.9999763851
PTPN12	1.1186151785	0.4853938825	0.9999763851
RSBN1L	1.495303933	0.1401550032	0.9999763851
TMEM60	1.300878672	0.2308215209	0.9999763851
PHTF2	-1.488029285	0.0342954132	0.9999763851
CD36	1.2042995172	0.224660613	0.9999763851
SEMA3C	0	0	0
PCLO	0	0	0
SEMA3E	0	0	0
KIAA1324L	3.2356557963	0.5547505991	0.9999763851
DMTF1	-1.0475929348	0.9175870281	0.9999763851
TMEM243	1.7681732715	0.1531744259	0.9999763851
CROT	0	0	0
ABCB4	0	0	0
SLC25A40	1.2114651276	0.4263832093	0.9999763851
DBF4	1.0088100305	0.9696729819	0.9999763851
ADAM22	1.2271473258	0.5400016229	0.9999763851
SRI	1.1317656442	0.39818441	0.9999763851
ZNF804B	0	0	0
STEAP1	-1.4247848768	0.5842374704	0.9999763851
CFAP69	0	0	0
GTPBP10	1.1948858339	0.5410311155	0.9999763851
CLDN12	1.2869488883	0.8199814146	0.9999763851
LOC102723899	-2.3222877795	0.4507688368	0.9999763851
CDK14	-1.0297650955	0.8965629522	0.9999763851
FZD1	1.4689483778	0.5007779952	0.9999763851
MTERF1	1.2022777405	0.7558869724	0.9999763851
AKAP9	-1.3851411871	0.0898086519	0.9999763851
CYP51A1	-1.1684489699	0.2822574634	0.9999763851
KRIT1	1.3468981445	0.0998064985	0.9999763851
ANKIB1	-1.6019220054	0.0423115949	0.9999763851
GATAD1	-1.1016397833	0.7259426403	0.9999763851
ERVW-1	0	0	0
PEX1	1.265567881	0.580122472	0.9999763851
RBM48	2.3003736834	0.041799148	0.9999763851
FAM133B	-1.0321585418	0.8685596857	0.9999763851
CDK6	1.0507650045	0.7172731672	0.9999763851
SAMD9	-1.7541501495	0.1177032502	0.9999763851

SAMD9L	-4.6971544391	0.0748617416	0.9999763851
VPS50	-1.062560424	0.8606597789	0.9999763851
BET1	-1.2331400996	0.4891558517	0.9999763851
CASD1	-1.366696748	0.4199395349	0.9999763851
PPP1R9A	0	0	0
PON3	0	0	0
PON2	1.7551621564	0.0646017307	0.9999763851
DYNC1I1	-1.4268484256	0.5780095417	0.9999763851
SLC25A13	1.0609732377	0.7664321594	0.9999763851
SHFM1	-1.0540032366	0.7017784396	0.9999763851
SDHAF3	1.2847060092	0.3281059859	0.9999763851
ASNS	-1.779055394	0.042180879	0.9999763851
LOC107986827	1.7592136256	0.4457276065	0.9999763851
OCM2	3.2353357581	0.554787377	0.9999763851
LMTK2	-1.2713648927	0.5536422756	0.9999763851
TECPR1	-1.0270065693	0.9262083892	0.9999763851
BRI3	1.0285699665	0.8192591687	0.9999763851
BAIAP2L1	-1.1912102323	0.2492142708	0.9999763851
NPTX2	-1.3710462586	0.7615898281	0.9999763851
TRRAP	-1.5469149503	0.139033385	0.9999763851
SMURF1	1.1467407327	0.6764053913	0.9999763851
KPNA7	-4.2849908816	0.4028453076	0.9999763851
ARPC1A	-1.2608322605	0.2784577983	0.9999763851
ARPC1B	1.0390450041	0.7913334311	0.9999763851
PDAP1	1.0415028014	0.7828712571	0.9999763851
BUD31	1.1262173631	0.4676405344	0.9999763851
PTCD1	14.446008687	0.1577541434	0.9999763851
ATP5J2-PTCD1	0	0	0
CPSF4	-1.1626148587	0.4717407775	0.9999763851
ATP5J2	1.0890001278	0.5409414341	0.9999763851
ZNF789	1.0989839006	0.8128183266	0.9999763851
ZNF394	-1.0766156499	0.7531241496	0.9999763851
ZKSCAN5	-1.009302712	0.9713197197	0.9999763851
FAM200A	-1.6199909674	0.4517141176	0.9999763851
ZNF655	1.9719302285	0.0004979	0.5153264972
GS1-259H13.2	3.1346807871	0.2968391969	0.9999763851
ZSCAN25	1.345907943	0.3601442194	0.9999763851
CYP3A5	2.591684032	0.1693979146	0.9999763851
CYP3A7	9.9624059448	0.2315099272	0.9999763851
CYP3A43	0	0	0
TRIM4	1.1671803253	0.445693855	0.9999763851
ZKSCAN1	-1.0438058947	0.7965140985	0.9999763851
ZSCAN21	3.2161501486	0.0897292495	0.9999763851
ZNF3	1.1594331036	0.6129651644	0.9999763851
COPS6	-1.0168255986	0.9180326247	0.9999763851
MCM7	1.1447565675	0.2967441159	0.9999763851
AP4M1	1.7913131739	0.0464373172	0.9999763851
TAF6	1.6488217526	0.0902934773	0.9999763851
CNPY4	1.0802618785	0.7805255726	0.9999763851

MBLAC1	12.2024790685	0.1819162385	0.9999763851
LAMTOR4	1.1614854217	0.3510749749	0.9999763851
C7orf43	1.0305490671	0.9501192505	0.9999763851
GAL3ST4	3.1150297783	0.1784429497	0.9999763851
GPC2	1.5020206484	0.595518	0.9999763851
STAG3	-1.209301103	0.5145811454	0.9999763851
GATS	-1.9654362824	0.4778430398	0.9999763851
SPDYE3	1.2799008528	0.6133680451	0.9999763851
PILRB	1.4257727745	0.0570378154	0.9999763851
PILRA	-1.2053868316	0.6690828323	0.9999763851
ZCWPW1	-1.4290643539	0.5224140734	0.9999763851
MEPCE	-1.1489303376	0.4903438998	0.9999763851
PPP1R35	-1.0341414086	0.8638163751	0.9999763851
C7orf61	-1.3944607594	0.697549577	0.9999763851
TSC22D4	1.1034161578	0.5566377809	0.9999763851
AGFG2	1.4666968614	0.5099418282	0.9999763851
SAP25	1.2812270507	0.6328575526	0.9999763851
LRCH4	-1.6735246496	0.0315148035	0.9999763851
ZASP	1.2505648436	0.4210039892	0.9999763851
FBXO24	1.1334347738	0.8812595716	0.9999763851
PCOLCE	1.7343484554	0.2271428026	0.9999763851
MOSPD3	1.3403479028	0.1258704939	0.9999763851
TFR2	1.4361889031	0.2566163307	0.9999763851
GNB2	-1.1244168047	0.3667707991	0.9999763851
GIGYF1	-1.092116278	0.7211343967	0.9999763851
POP7	-1.0237684779	0.8586887118	0.9999763851
EPO	3.2356557963	0.5547505991	0.9999763851
EPHB4	1.1513000895	0.4670878805	0.9999763851
SLC12A9	1.3978130107	0.1806262012	0.9999763851
TRIP6	-1.1234645008	0.8363557165	0.9999763851
SRRT	1.0449515701	0.7589269472	0.9999763851
UFSP1	2.2212549951	0.3556854729	0.9999763851
MUC3A	3.2356557963	0.5547505991	0.9999763851
MUC12	0	0	0
TRIM56	-1.053427523	0.7410472063	0.9999763851
SERPINE1	-18.4802551288	0.0001291	0.1878439983
AP1S1	1.0067196653	0.9891548453	0.9999763851
VGF	-1.1962228143	0.7972904931	0.9999763851
MOGAT3	0	0	0
PLOD3	-1.1057018411	0.5865985675	0.9999763851
ZNHIT1	1.0217857268	0.9036715459	0.9999763851
CLDN15	-1.2134598188	0.5747275998	0.9999763851
FIS1	-1.2561509121	0.2010315273	0.9999763851
IFT22	2.7880133726	0.0908064682	0.9999763851
COL26A1	5.4781708352	0.3745527127	0.9999763851
CUX1	1.0163759511	0.9166410007	0.9999763851
SPDYE6	9.9312452783	0.2486353486	0.9999763851
LOC100289561	1.673619509	0.4236672576	0.9999763851
PRKRIP1	1.0328374856	0.9235876721	0.9999763851

ORAI2	1.0392534547	0.8109607281	0.9999763851
ALKBH4	1.4888225777	0.2744189662	0.9999763851
LRWD1	-2.6603009385	0.1946738494	0.9999763851
POLR2J	-1.2145813177	0.3064877079	0.9999763851
RASA4B	0	0	0
LOC107983993	0	0	0
POLR2J3	1.0089954394	0.9652881497	0.9999763851
LOC102724229	-1.1196184556	0.8189927681	0.9999763851
RASA4	-1.8159853009	0.478604594	0.9999763851
POLR2J2	1.4449856502	0.5449034009	0.9999763851
UPK3BL	-1.371091068	0.7484873287	0.9999763851
FBXL13	1.4318936151	0.1082619162	0.9999763851
FAM185A	-1.0892180603	0.7506760557	0.9999763851
NFE4	1.3317707718	0.6601972729	0.9999763851
ARMC10	1.0656971818	0.7206636175	0.9999763851
NAPEPLD	-1.9256800829	0.0647040826	0.9999763851
PMPCB	1.1238791348	0.5156746575	0.9999763851
DNAJC2	1.0549212145	0.8446306158	0.9999763851
PSMC2	-1.19656883	0.1540966075	0.9999763851
SLC26A5	5.4781708352	0.3745527127	0.9999763851
RELN	0	0	0
ORC5	-1.0338762551	0.9100251593	0.9999763851
LHFPL3	-1.8524439232	0.2062397362	0.9999763851
KMT2E	-1.1346058734	0.5080522737	0.9999763851
SRPK2	-1.303337299	0.101628381	0.9999763851
PUS7	1.2618104364	0.3397138619	0.9999763851
RINT1	1.4461902254	0.2752129056	0.9999763851
ATXN7L1	-1.1269100093	0.7069992646	0.9999763851
CDHR3	1.9230607285	0.4470996142	0.9999763851
SYPL1	1.1629346815	0.2881039119	0.9999763851
NAMPT	-1.0105440606	0.9493115781	0.9999763851
CCDC71L	1.3688597115	0.4078431595	0.9999763851
PIK3CG	-1.2234812458	0.3581719499	0.9999763851
PRKAR2B	-1.014356189	0.9623156199	0.9999763851
HBP1	-1.2093542669	0.4685871111	0.9999763851
COG5	1.4531138015	0.2063591417	0.9999763851
GPR22	-4.2864494036	0.4027150576	0.9999763851
DUS4L	-1.2346199706	0.524190447	0.9999763851
BCAP29	-1.1969289209	0.4592612032	0.9999763851
SLC26A4	-4.5995992229	0.1605548602	0.9999763851
CBLL1	-1.2569069293	0.4003054319	0.9999763851
DLD	1.5495543508	0.003463	0.9999763851
NRCAM	0	0	0
PNPLA8	-1.550326225	0.2890871085	0.9999763851
THAP5	-1.3718527825	0.2133293136	0.9999763851
DNAJB9	-1.7577873142	0.038087293	0.9999763851
IMMP2L	-1.1779074577	0.5451659866	0.9999763851
DOCK4	1.1438127432	0.8674029573	0.9999763851
ZNF277	-1.4424144392	0.3384422939	0.9999763851

IFRD1	1.2605333674	0.4029606176	0.9999763851
LSMEM1	3.2353357581	0.554787377	0.9999763851
TMEM168	1.1089275909	0.7904350518	0.9999763851
BMT2	-1.3211198904	0.3618786663	0.9999763851
MDFIC	1.3022525694	0.3481098137	0.9999763851
TFEC	1.8138024348	0.5768982502	0.9999763851
TES	1.296628076	0.3136043733	0.9999763851
CAPZA2	1.0510457015	0.8179629542	0.9999763851
ST7	-1.3819268034	0.3761256608	0.9999763851
CFTR	0	0	0
CTTNBP2	5.4781708352	0.3745527127	0.9999763851
LSM8	1.040225237	0.8196280048	0.9999763851
ING3	-1.2769585099	0.328284084	0.9999763851
CPED1	0	0	0
FAM3C	1.1932462846	0.4543505732	0.9999763851
CADPS2	1.0262948724	0.9788500541	0.9999763851
NDUFA5	-1.1607463535	0.3695663698	0.9999763851
ASB15	0	0	0
WASL	-1.8540910445	0.4097533821	0.9999763851
POT1	-1.6455958656	0.0570632832	0.9999763851
ZNF800	1.2826424203	0.3638767268	0.9999763851
GCC1	1.260495218	0.2679199795	0.9999763851
ARF5	-1.0588299491	0.7717288313	0.9999763851
SND1	1.0987437621	0.5139869387	0.9999763851
RBM28	-1.0094952909	0.9652033447	0.9999763851
PRRT4	1.8693032305	0.1326026597	0.9999763851
IMPDH1	1.0685166136	0.7302868034	0.9999763851
HILPDA	-1.945838168	0.003028	0.9999763851
METTL2B	-1.2993229644	0.4976832443	0.9999763851
FAM71F2	-1.9663843334	0.476508184	0.9999763851
FAM71F1	3.2353357581	0.554787377	0.9999763851
CALU	-1.2109437025	0.2147955128	0.9999763851
OPN1SW	-4.3316847633	0.1687825633	0.9999763851
CCDC136	-1.0679378543	0.7910914553	0.9999763851
ATP6V1F	-1.1260258665	0.4702532794	0.9999763851
LOC100130705	1.5495665418	0.5563644089	0.9999763851
IRF5	1.0277365203	0.9239947893	0.9999763851
TNPO3	-1.2219893577	0.1657249651	0.9999763851
TSPAN33	-1.2256426962	0.6433721869	0.9999763851
SMO	1.2866381786	0.8224275873	0.9999763851
AHCYL2	-2.9688820569	0.0310987832	0.9999763851
STRIP2	1.2249691435	0.7524637155	0.9999763851
SMKR1	-4.2849908816	0.4028453076	0.9999763851
NRF1	1.5538406436	0.0755770827	0.9999763851
UBE2H	-1.115986649	0.612123744	0.9999763851
ZC3HC1	-1.2698792734	0.2823146177	0.9999763851
KLHDC10	1.0927383015	0.7174448363	0.9999763851
TMEM209	1.2660009925	0.4310092531	0.9999763851
CPA5	0	0	0

CEP41	-1.2622708615	0.4113883407	0.9999763851
MEST	0	0	0
COPG2	-1.3772969212	0.4325882662	0.9999763851
TSGA13	0	0	0
MKLN1	1.0071973984	0.9672636541	0.9999763851
PODXL	-1.2699511628	0.2729241891	0.9999763851
PLXNA4	0	0	0
CHCHD3	1.4018184941	0.0120684538	0.9999763851
EXOC4	-1.1373139244	0.4745637935	0.9999763851
LRGUK	0	0	0
SLC35B4	1.144676811	0.5437955191	0.9999763851
AKR1B15	0	0	0
BPGM	-1.1044788179	0.8049647853	0.9999763851
CALD1	-4.287394647	0.4026306792	0.9999763851
AGBL3	1.0954359883	0.892881712	0.9999763851
C7orf49	1.0032161556	0.9895938427	0.9999763851
TMEM140	-2.7550513428	0.095252233	0.9999763851
WDR91	-1.98808452	0.0655207501	0.9999763851
CNOT4	1.645595549	0.1278190377	0.9999763851
NUP205	1.1654208446	0.5260626212	0.9999763851
C7orf73	1.2936396508	0.1222371871	0.9999763851
SLC13A4	-1.8254196192	0.4539876142	0.9999763851
LUZP6	-1.1084024167	0.4837885847	0.9999763851
MTPN	1.0481197273	0.7526529306	0.9999763851
CHRM2	0	0	0
PTN	3.2356557963	0.5547505991	0.9999763851
DGKI	-1.0264773286	0.8406414173	0.9999763851
CREB3L2	-1.1906675204	0.3174455083	0.9999763851
LOC100130880	3.2361532718	0.5546934395	0.9999763851
AKR1D1	0	0	0
TRIM24	-1.4214426375	0.170576949	0.9999763851
SVOPL	0	0	0
ATP6V0A4	0	0	0
KIAA1549	-1.430050834	0.5394927005	0.9999763851
ZC3HAV1	-1.1436195888	0.3979275	0.9999763851
TTC26	1.0417717796	0.9336885605	0.9999763851
UBN2	1.0178058951	0.9616027636	0.9999763851
LUC7L2	-1.3445014557	0.0431168178	0.9999763851
C7orf55	-1.2220450386	0.4047334616	0.9999763851
C7orf55-LUC7L2	1.2664941716	0.7819606336	0.9999763851
HIPK2	1.0038952002	0.9808706516	0.9999763851
TBXAS1	1.039632449	0.7973534339	0.9999763851
PARP12	-1.076056544	0.8459356546	0.9999763851
KDM7A	1.6448847496	0.3382553044	0.9999763851
SLC37A3	1.5007464133	0.3573145612	0.9999763851
RAB19	0	0	0
MKRN1	-1.0568706076	0.7876241559	0.9999763851
ADCK2	-1.1149911171	0.5813856853	0.9999763851
NDUFB2	1.0282568252	0.8639422449	0.9999763851

BRAF	-1.1885728102	0.4631981259	0.9999763851
MRPS33	1.077191044	0.6935135282	0.9999763851
TMEM178B	-4.2864494036	0.4027150576	0.9999763851
AGK	-1.0378467481	0.9215653342	0.9999763851
KIAA1147	1.045784423	0.8522787339	0.9999763851
WEE2	3.2353357581	0.554787377	0.9999763851
SSBP1	-1.1390383599	0.3762204388	0.9999763851
PRSS37	0	0	0
CLEC5A	-1.1429477392	0.5482043743	0.9999763851
MGAM	1.7952300153	0.2592030523	0.9999763851
MGAM2	5.4781708352	0.3745527127	0.9999763851
EPHB6	-1.251972694	0.6214264958	0.9999763851
TRPV6	0	0	0
KEL	1.4852250107	0.5071786273	0.9999763851
OR6V1	0	0	0
PIP	0	0	0
GSTK1	-1.0986039579	0.6267835153	0.9999763851
CASP2	-1.0817185402	0.5923620908	0.9999763851
CLCN1	3.2356557963	0.5547505991	0.9999763851
FAM131B	2.8345356849	0.3324761417	0.9999763851
LOC100507507	1.0879252635	0.8520900292	0.9999763851
ZYX	1.2154052789	0.2744682412	0.9999763851
EPHA1	1.0953952354	0.724211736	0.9999763851
LOC101928422	6.368054416	0.3491752152	0.9999763851
TCAF2	-1.9498743893	0.3211403515	0.9999763851
TCAF1	-1.0146583012	0.9650716657	0.9999763851
TPK1	-2.3437421063	0.0286777084	0.9999763851
CNTNAP2	-1.8919466972	0.2216852657	0.9999763851
CUL1	1.1559033425	0.3616115311	0.9999763851
EZH2	1.3751208806	0.1184798313	0.9999763851
PDIA4	-1.249551815	0.1283609228	0.9999763851
ZNF786	2.3406309168	0.4064998765	0.9999763851
ZNF425	-3.3284109188	0.2422780785	0.9999763851
ZNF398	-1.1505850001	0.6757588464	0.9999763851
ZNF282	-1.1279750704	0.6271694393	0.9999763851
ZNF212	-1.0716946815	0.8422101461	0.9999763851
ZNF783	-2.7177561303	0.1581637869	0.9999763851
ZNF777	1.2997010458	0.2582767168	0.9999763851
ZNF746	2.2676908633	0.0156067308	0.9999763851
KRBA1	1.005759094	0.9891590912	0.9999763851
ZNF467	2.3399628165	0.4287847126	0.9999763851
SSPO	0	0	0
ZNF862	2.0392704743	0.0786456448	0.9999763851
ATP6V0E2	1.5076098516	0.1523718194	0.9999763851
ACTR3C	5.4790129495	0.3755300282	0.9999763851
REPIN1	1.0425666032	0.8105158122	0.9999763851
ZNF775	1.2357073156	0.5353976021	0.9999763851
GIMAP6	0	0	0
GIMAP2	-2.2386708426	0.3228075893	0.9999763851

GIMAP1	3.1181383987	0.1644481717	0.9999763851
GIMAP5	-1.2301452062	0.7475757252	0.9999763851
TMEM176B	0	0	0
TMEM176A	0	0	0
AOC1	4.2952862008	0.0174898113	0.9999763851
KCNH2	-1.148769585	0.8524480909	0.9999763851
NOS3	1.4700672986	0.6282958928	0.9999763851
ATG9B	1.3642607159	0.2404378931	0.9999763851
ABCB8	1.5224190013	0.2953660822	0.9999763851
ASIC3	-4.2849908816	0.4028453076	0.9999763851
CDK5	1.6414863076	0.1283550728	0.9999763851
SLC4A2	1.1097280139	0.7211673493	0.9999763851
FASTK	1.2134624697	0.3099068408	0.9999763851
TMUB1	1.034986563	0.9166283544	0.9999763851
AGAP3	-1.2645526064	0.1622459227	0.9999763851
ASB10	3.2353357581	0.554787377	0.9999763851
ABCF2	1.162485388	0.444492397	0.9999763851
CHPF2	1.2230015943	0.6314072868	0.9999763851
SMARCD3	-1.1561688231	0.5338962639	0.9999763851
NUB1	1.003230485	0.9865390006	0.9999763851
WDR86	0	0	0
CRYGN	3.2361532718	0.5546934395	0.9999763851
RHEB	1.1198816058	0.4395619225	0.9999763851
PRKAG2	-1.5042685252	0.1263404086	0.9999763851
GALNTL5	0	0	0
GALNT11	-1.5151731585	0.3392634407	0.9999763851
KMT2C	1.0081522651	0.9652181705	0.9999763851
XRCC2	-2.2009269002	0.1330182492	0.9999763851
ACTR3B	1.420167376	0.37771521	0.9999763851
DPP6	-4.287394647	0.4026306792	0.9999763851
PAXIP1	-1.0921052317	0.7191404986	0.9999763851
INSIG1	1.098043046	0.566422573	0.9999763851
CNPY1	0	0	0
RBM33	-1.040247496	0.8216489381	0.9999763851
SHH	-1.1960111917	0.7986160897	0.9999763851
RNF32	1.3712615059	0.3506300527	0.9999763851
LMBR1	-1.1549860672	0.5772522782	0.9999763851
NOM1	1.0061963107	0.9837036735	0.9999763851
MNX1	0	0	0
UBE3C	-1.2362450796	0.2723486973	0.9999763851
DNAJB6	1.0649713633	0.6581283139	0.9999763851
PTPRN2	1.2238256159	0.1940897266	0.9999763851
NCAPG2	1.227705825	0.2841848852	0.9999763851
ESYT2	1.0842470507	0.5446453596	0.9999763851
WDR60	-1.4462633224	0.1898811196	0.9999763851
LOC101927506	-1.3032413687	0.4237502393	0.9999763851
ZNF596	1.0603640252	0.9187222914	0.9999763851
FBXO25	-1.1787543677	0.417556954	0.9999763851
ERICH1	1.0429366413	0.8073255904	0.9999763851

LOC105377774	0	0	0
CLN8	1.1435750234	0.5165600216	0.9999763851
ARHGEF10	-1.152788508	0.3383661556	0.9999763851
KBTBD11	1.0579831489	0.6714738048	0.9999763851
MYOM2	1.2167155364	0.6934042691	0.9999763851
CSMD1	-1.1494263678	0.8551670286	0.9999763851
MCPH1	-1.0092042184	0.9737700029	0.9999763851
AGPAT5	1.0604882267	0.7265222185	0.9999763851
DEFB1	-1.1362077083	0.7906079227	0.9999763851
DEFA6	3.2356557963	0.5547505991	0.9999763851
DEFA3	0	0	0
LOC101928095	3.2356557963	0.5547505991	0.9999763851
DEFB107B	0	0	0
SGK223	1.6240330447	0.1740344477	0.9999763851
MFHAS1	1.019818381	0.9198398018	0.9999763851
ERI1	1.009192244	0.9613795581	0.9999763851
PPP1R3B	-1.2274062003	0.5252792829	0.9999763851
TNKS	-1.1677562568	0.4657381213	0.9999763851
MSRA	1.0771462741	0.6565629718	0.9999763851
PRSS55	0	0	0
RP1L1	-7.5664585246	0.2262040232	0.9999763851
C8orf74	0	0	0
SOX7	-4.287394647	0.4026306792	0.9999763851
PINX1	1.5534800774	0.0546158456	0.9999763851
XKR6	0	0	0
MTMR9	1.0440636445	0.8223917365	0.9999763851
BLK	0	0	0
GATA4	3.2361532718	0.5546934395	0.9999763851
NEIL2	-1.1043267839	0.6072977078	0.9999763851
FDFT1	1.1087028495	0.441346208	0.9999763851
CTSB	-1.4385375411	0.0220313104	0.9999763851
FAM86B1	1.6938437942	0.6482137545	0.9999763851
FAM86B2	2.7888075642	0.0791380525	0.9999763851
LONRF1	1.0576498276	0.7963966868	0.9999763851
SGCZ	3.2361532718	0.5546934395	0.9999763851
TUSC3	0	0	0
ZDHHC2	1.1706923952	0.3751755433	0.9999763851
CNOT7	-1.0880305422	0.5614371017	0.9999763851
VPS37A	-1.005026464	0.9854952552	0.9999763851
MTMR7	-1.2775131623	0.645953223	0.9999763851
SLC7A2	0	0	0
PDGFRL	2.3718951865	0.4298080056	0.9999763851
MTUS1	6.5527883443	0.0604654102	0.9999763851
FGL1	-2.9214815332	0.2255412548	0.9999763851
PCM1	-1.1754375874	0.2744839348	0.9999763851
ASAH1	-1.1648416212	0.3038487466	0.9999763851
NAT1	1.5792356417	0.2523822491	0.9999763851
PSD3	-6.7190164239	0.0168710208	0.9999763851
CSGALNACT1	-4.2849908816	0.4028453076	0.9999763851

INTS10	1.0289547713	0.8477526861	0.9999763851
ATP6V1B2	-1.163518063	0.2776621727	0.9999763851
LZTS1	-1.0993155783	0.8549022551	0.9999763851
GFRA2	3.2353357581	0.554787377	0.9999763851
DOK2	1.2227263711	0.3518472625	0.9999763851
XPO7	1.3646850532	0.0396533149	0.9999763851
NPM2	0	0	0
FGF17	3.2361532718	0.5546934395	0.9999763851
DMTN	0	0	0
FAM160B2	1.0341944798	0.904489878	0.9999763851
NUDT18	1.3411938723	0.7188283026	0.9999763851
REEP4	1.2189329004	0.3119568966	0.9999763851
BMP1	-1.1425383389	0.604674118	0.9999763851
PHYHIP	-1.315303824	0.8206171563	0.9999763851
POLR3D	-1.1574171561	0.6074902132	0.9999763851
PIWIL2	-1.3154339304	0.8205576571	0.9999763851
SLC39A14	1.0259840595	0.9056486958	0.9999763851
PPP3CC	1.0817588787	0.8298605094	0.9999763851
SORBS3	1.4373308199	0.0586194251	0.9999763851
PDLIM2	1.0991104274	0.624111947	0.9999763851
LOC107986876	-1.2303879832	0.7501769833	0.9999763851
CCAR2	1.0446393582	0.878752623	0.9999763851
BIN3	-1.1213769521	0.6508656537	0.9999763851
EGR3	-2.4274267559	0.444512626	0.9999763851
PEBP4	-2.3220580825	0.4379866635	0.9999763851
LOC107984124	-3.6013971471	0.1475950682	0.9999763851
RHOBTB2	-1.1017419755	0.6450123638	0.9999763851
TNFRSF10B	1.0597962099	0.771675869	0.9999763851
TNFRSF10C	1.3416940469	0.7101625849	0.9999763851
TNFRSF10D	1.4332638114	0.1928969018	0.9999763851
TNFRSF10A	-2.5600051511	0.2551919212	0.9999763851
CHMP7	-1.2478132897	0.3684863237	0.9999763851
R3HCC1	1.3985996961	0.2603718541	0.9999763851
LOXL2	-2.2684489947	0.0897041859	0.9999763851
ENTPD4	1.0592170798	0.8465746069	0.9999763851
SLC25A37	1.0064786111	0.9644771447	0.9999763851
NKX3-1	1.9229787277	0.4494377664	0.9999763851
STC1	1.2867653957	0.8301398163	0.9999763851
ADAMDEC1	-6.3107594234	0.001443	0.9833300676
DOCK5	-1.2786711062	0.2517035205	0.9999763851
GNRH1	-1.817658111	0.4893544708	0.9999763851
KCTD9	1.3212186742	0.2122866179	0.9999763851
CDCA2	1.206659786	0.2650265567	0.9999763851
PPP2R2A	1.0282154786	0.8516664593	0.9999763851
BNIP3L	1.0724375763	0.709035667	0.9999763851
PNMA2	3.2353357581	0.554787377	0.9999763851
DPYSL2	1.0729100739	0.6424729382	0.9999763851
ADRA1A	3.2353357581	0.554787377	0.9999763851
TRIM35	-1.0233682613	0.9191432708	0.9999763851

PTK2B	1.0834284135	0.6129108127	0.9999763851
CHRNA2	0	0	0
CLU	1.3414047442	0.1384967216	0.9999763851
SCARA3	-1.0726805543	0.8582000601	0.9999763851
CCDC25	-1.0067341735	0.9676799004	0.9999763851
ESCO2	1.9672305522	0.0352105008	0.9999763851
PBK	1.2619692003	0.1990293938	0.9999763851
ELP3	-1.0154903507	0.9348709302	0.9999763851
PNOC	0	0	0
ZNF395	-1.099623388	0.5585524064	0.9999763851
FBXO16	0	0	0
EXTL3	1.3288910258	0.3258158961	0.9999763851
INTS9	1.2121485014	0.4134545908	0.9999763851
HMBOX1	-1.3448864747	0.2448941456	0.9999763851
KIF13B	1.0300839327	0.9518122402	0.9999763851
DUSP4	1.3255544809	0.1763344276	0.9999763851
SARAF	1.0171353809	0.9133091191	0.9999763851
LEPROTL1	-1.0061328951	0.968936976	0.9999763851
MBOAT4	1.6248125624	0.5711456773	0.9999763851
DCTN6	1.1969183505	0.4548920034	0.9999763851
GTF2E2	1.0500526515	0.7634339484	0.9999763851
GSR	1.0793188762	0.6139673865	0.9999763851
UBXN8	-1.0243023348	0.9126350169	0.9999763851
PPP2CB	-1.3341345691	0.1760758379	0.9999763851
PURG	0	0	0
WRN	1.1521303174	0.5762933101	0.9999763851
NRG1	-1.3708355312	0.7474480451	0.9999763851
FUT10	1.0129043461	0.9639044789	0.9999763851
MAK16	-2.6620159038	9.14E-05	0.1447401432
TTI2	3.8630893811	1.00E-06	0.002699
RNF122	1.0280058166	0.9750561227	0.9999763851
DUSP26	0	0	0
UNC5D	3.2356557963	0.5547505991	0.9999763851
ZNF703	1.0856559564	0.6970820605	0.9999763851
ERLIN2	-1.3153234859	0.2186008144	0.9999763851
PROSC	1.2267447587	0.2460114131	0.9999763851
ADGRA2	-1.066062659	0.6775410842	0.9999763851
BRF2	-1.0638730528	0.7084096568	0.9999763851
RAB11FIP1	1.0149166326	0.9326850055	0.9999763851
EIF4EBP1	-1.0890685398	0.663186681	0.9999763851
ASH2L	1.0885071947	0.5946804104	0.9999763851
STAR	-1.8294751893	0.0793776121	0.9999763851
LSM1	1.2040323987	0.365498686	0.9999763851
BAG4	1.0557350448	0.7838883256	0.9999763851
DDHD2	1.2128575914	0.3794195116	0.9999763851
PLPP5	1.0081284723	0.9782103242	0.9999763851
WHSC1L1	-1.0880417053	0.6735051696	0.9999763851
LETM2	1.1406059108	0.8268710036	0.9999763851
FGFR1	1.3241974546	0.4559237843	0.9999763851

TACC1	1.0425537857	0.7448355208	0.9999763851
PLEKHA2	1.0385359501	0.7952760373	0.9999763851
TM2D2	1.6068927566	0.0282422178	0.9999763851
ADAM9	1.4550699438	0.3461120508	0.9999763851
ADAM32	-1.6348888287	0.3753617822	0.9999763851
ADAM18	0	0	0
IDO2	3.2356557963	0.5547505991	0.9999763851
ZMAT4	0	0	0
GOLGA7	-1.2706863904	0.1464848051	0.9999763851
GINS4	2.0478219626	0.0217006917	0.9999763851
GPAT4	1.0174088714	0.9106729016	0.9999763851
ANK1	1.7349366192	0.230323362	0.9999763851
KAT6A	-1.0290856422	0.8549017917	0.9999763851
AP3M2	1.3131380334	0.3250121124	0.9999763851
PLAT	0	0	0
IKBKB	-1.3820034023	0.1146948094	0.9999763851
POLB	1.3407709621	0.2667210306	0.9999763851
DKK4	-4.2864494036	0.4027150576	0.9999763851
VDAC3	1.0838697098	0.5462600802	0.9999763851
SLC20A2	-1.0479340919	0.8138877403	0.9999763851
SMIM19	1.540076391	0.0704941204	0.9999763851
THAP1	1.746702179	0.1162530926	0.9999763851
RNF170	-1.1018826496	0.6477426669	0.9999763851
HOOK3	1.0133405051	0.9346183523	0.9999763851
FNTA	1.1619438452	0.3590933086	0.9999763851
POMK	2.34099053	0.3999111465	0.9999763851
HGSNAT	1.3053388029	0.2113487013	0.9999763851
SPIDR	-1.6034251166	0.0332215248	0.9999763851
CEBPD	7.7192231741	0.2782707334	0.9999763851
PRKDC	1.0266437769	0.8754379185	0.9999763851
MCM4	1.1825452925	0.2142825582	0.9999763851
UBE2V2	-1.1835284581	0.1925514982	0.9999763851
SNTG1	0	0	0
PXDNL	5.4781708352	0.3745527127	0.9999763851
PCMTD1	-1.0867524902	0.7576084892	0.9999763851
ST18	3.2361532718	0.5546934395	0.9999763851
FAM150A	0	0	0
RB1CC1	-1.2105452699	0.4012354925	0.9999763851
OPRK1	1.1346379968	0.5902408663	0.9999763851
ATP6V1H	1.2709322457	0.270087636	0.9999763851
RGS20	-4.2849908816	0.4028453076	0.9999763851
TCEA1	-1.0269224857	0.8879403369	0.9999763851
LYPLA1	1.4487581206	0.0192311475	0.9999763851
MRPL15	1.0616357253	0.6880334228	0.9999763851
RP1	6.368054416	0.3491821635	0.9999763851
TMEM68	-1.0477853461	0.8781669371	0.9999763851
TGS1	1.1521810797	0.4395768762	0.9999763851
LYN	-1.1105043527	0.4766837322	0.9999763851
RPS20	1.0479476681	0.7012615761	0.9999763851

PLAG1	1.0172688829	0.9498968131	0.9999763851
CHCHD7	1.1891295795	0.238004314	0.9999763851
IMPAD1	-1.1111356375	0.5548146279	0.9999763851
UBXN2B	-1.0448300484	0.8049453653	0.9999763851
SDCBP	-1.8682211847	0.1439379878	0.9999763851
NSMAF	-1.2484053101	0.0958118738	0.9999763851
TOX	-2.5596117216	0.2615742465	0.9999763851
RAB2A	-1.2201517894	0.1539852589	0.9999763851
CHD7	-1.394553145	0.6912352536	0.9999763851
LOC105375938	1.2869488883	0.8201303114	0.9999763851
CLVS1	3.6822803285	0.1937397534	0.9999763851
ASPH	-1.6101603492	0.1061890958	0.9999763851
NKAIN3	5.4790129495	0.3756234837	0.9999763851
YTHDF3	-1.2618751124	0.1142120203	0.9999763851
CYP7B1	0	0	0
ARMC1	1.1259473867	0.4485182965	0.9999763851
MTFR1	1.1978861101	0.3712977355	0.9999763851
PDE7A	1.3092091275	0.558097201	0.9999763851
DNAJC5B	-1.3158816768	0.8203229797	0.9999763851
TRIM55	7.4933530102	0.2987077671	0.9999763851
RRS1	-1.0326179662	0.8398968301	0.9999763851
ADHFE1	0	0	0
MYBL1	2.1540433933	0.1038866927	0.9999763851
VCPIP1	-1.53186198	0.0806246417	0.9999763851
C8orf44	3.7682243222	0.2257682304	0.9999763851
C8orf44-SGK3	-1.5947065317	0.077697772	0.9999763851
SGK3	1.2505118907	0.5118545335	0.9999763851
MCMDC2	-1.0522684077	0.7175096505	0.9999763851
TCF24	-4.2849908816	0.4028453076	0.9999763851
COPS5	1.3733825717	0.1043460423	0.9999763851
CSPP1	-1.0297929625	0.9062599159	0.9999763851
ARFGEF1	-1.1062204244	0.6552764523	0.9999763851
CPA6	1.027554661	0.9756568037	0.9999763851
SULF1	0	0	0
PRDM14	0	0	0
NCOA2	-1.1471955195	0.6081182073	0.9999763851
TRAM1	-1.0172530996	0.916051658	0.9999763851
LACTB2	1.0729406712	0.801816672	0.9999763851
XKR9	-4.2849908816	0.4028453076	0.9999763851
KCNB2	3.2356557963	0.5547505991	0.9999763851
TERF1	1.0302418787	0.8731524169	0.9999763851
SBSPON	2.8699786574	0.2275014857	0.9999763851
C8orf89	0	0	0
RPL7	1.2483960303	0.0612657832	0.9999763851
RDH10	-1.0372532667	0.9142791095	0.9999763851
STAU2	1.0091927785	0.9542264913	0.9999763851
UBE2W	-2.1045879989	0.0178730665	0.9999763851
TCEB1	1.0367425103	0.8083411912	0.9999763851
TMEM70	-1.3196262818	0.1409588848	0.9999763851

LY96	1.2870761236	0.8160709833	0.9999763851
GDAP1	3.6824427513	0.0711644253	0.9999763851
ZFHX4	0	0	0
PEX2	-1.005099002	0.9856968383	0.9999763851
LOC105375907	-10.8458971147	0.143297758	0.9999763851
ZC2HC1A	0	0	0
IL7	-3.0625500992	0.1535337395	0.9999763851
MRPS28	1.1529148827	0.6011280599	0.9999763851
TPD52	1.959384623	0.0154744288	0.9999763851
ZNF704	3.2353357581	0.554787377	0.9999763851
PAG1	-1.1514079534	0.3952447469	0.9999763851
FABP5	-1.1228160801	0.4309918747	0.9999763851
IMPA1	1.5593807263	0.0164639935	0.9999763851
SLC10A5	-4.2849908816	0.4028453076	0.9999763851
ZFAND1	-1.237777719	0.6465559301	0.9999763851
SNX16	1.4995339414	0.4512081041	0.9999763851
LRRCC1	1.2804626953	0.3557880341	0.9999763851
E2F5	1.6147583029	0.4303877657	0.9999763851
C8orf59	-1.128203331	0.4498807463	0.9999763851
CA13	0	0	0
CA3	0	0	0
ATP6V0D2	-7.5647903763	0.2176246281	0.9999763851
WWP1	-1.3066821489	0.6164945661	0.9999763851
RMDN1	-1.1742706176	0.4235470363	0.9999763851
CPNE3	1.3169713144	0.0476031897	0.9999763851
CNBD1	10.4285443305	0.2414187314	0.9999763851
MMP16	3.2353357581	0.554787377	0.9999763851
RIPK2	-1.5845031786	0.3782776676	0.9999763851
OSGIN2	1.0044678214	0.9815058608	0.9999763851
NBN	-1.1336121546	0.5705107675	0.9999763851
DECR1	1.1632906229	0.4317237135	0.9999763851
CALB1	3.2361532718	0.5546934395	0.9999763851
TMEM64	-1.0107884713	0.9720612535	0.9999763851
NECAB1	0	0	0
C8orf88	-4.2849908816	0.4028453076	0.9999763851
TMEM55A	-1.4513062802	0.3941834571	0.9999763851
OTUD6B	-1.1455204108	0.5938638676	0.9999763851
LRRC69	-1.0377913173	0.943433887	0.9999763851
SLC26A7	0	0	0
TRIQK	3.2361532718	0.5546934395	0.9999763851
FAM92A1	16.68931238	0.1282237357	0.9999763851
RBM12B	1.2355188726	0.4673562656	0.9999763851
TMEM67	-2.5616116111	0.3180401137	0.9999763851
PDP1	1.1347062888	0.6199933211	0.9999763851
CDH17	-1.3158816768	0.8203229797	0.9999763851
RAD54B	-1.2409726533	0.5254821756	0.9999763851
FSBP	-2.5595294703	0.3313983831	0.9999763851
KIAA1429	1.0150765162	0.9279112938	0.9999763851
DPY19L4	1.0196018649	0.9455722808	0.9999763851

INTS8	1.3364022371	0.3745241867	0.9999763851
CCNE2	3.7488601547	0.0213945604	0.9999763851
NDUFAF6	-1.367640106	0.1216497897	0.9999763851
PLEKHF2	-1.0305481475	0.8805727142	0.9999763851
C8orf37	2.1695825873	0.4532423473	0.9999763851
UQCRB	-1.0483086239	0.7090369211	0.9999763851
MTERF3	2.0140400713	0.1958351475	0.9999763851
PTDSS1	-1.0738019293	0.580977574	0.9999763851
CPQ	-1.3250400972	0.1359969495	0.9999763851
MTDH	-1.0591801378	0.6299636056	0.9999763851
LAPTM4B	5.4770877341	0.3746135179	0.9999763851
MATN2	3.2356557963	0.5547505991	0.9999763851
RPL30	-1.0172902628	0.8971506862	0.9999763851
ERICH5	3.2356557963	0.5547505991	0.9999763851
RIDA	1.1500645434	0.5086081001	0.9999763851
POP1	1.2372070932	0.3241224367	0.9999763851
NIPAL2	1.1107555331	0.6982254012	0.9999763851
STK3	-1.4241948029	0.193440807	0.9999763851
VPS13B	1.0554937377	0.80476688	0.9999763851
COX6C	-1.1737227755	0.1959688688	0.9999763851
RGS22	3.2353357581	0.554787377	0.9999763851
FBXO43	-2.8354075942	0.0152263282	0.9999763851
POLR2K	1.0563097794	0.7956891145	0.9999763851
SPAG1	1.1421174847	0.789795773	0.9999763851
RNF19A	-1.0386571315	0.8942369549	0.9999763851
ANKRD46	-1.3160897641	0.2444144846	0.9999763851
PABPC1	1.1562436235	0.273846531	0.9999763851
YWHAZ	1.1136845338	0.3568112561	0.9999763851
ZNF706	1.4611536178	0.0248054797	0.9999763851
GRHL2	-1.3154339304	0.8205576571	0.9999763851
NCALD	1.2607119075	0.4622572671	0.9999763851
RRM2B	-1.3689541823	0.2076157972	0.9999763851
UBR5	-1.0569893753	0.7381709571	0.9999763851
KLF10	1.0369944912	0.8856566578	0.9999763851
LOC101927245	3.2356557963	0.5547505991	0.9999763851
LOC107986873	1.9410444986	0.0577707054	0.9999763851
AZIN1	-1.0125047424	0.9336375479	0.9999763851
ATP6V1C1	-1.0318091797	0.8768324791	0.9999763851
BAALC	5.4776292235	0.3745831165	0.9999763851
CTHRC1	1.7889997486	0.4430561517	0.9999763851
SLC25A32	1.1731002443	0.3832294477	0.9999763851
DCAF13	1.157540306	0.3801294601	0.9999763851
DCSTAMP	-7.348280118	0.003043	0.9999763851
DPYS	3.2356557963	0.5547505991	0.9999763851
ZFPM2	1.2869488883	0.820284766	0.9999763851
OXR1	-1.3160416743	0.8273188224	0.9999763851
ANGPT1	0	0	0
RSPO2	-1.3154339304	0.8205576571	0.9999763851
EIF3E	-1.0654213796	0.6733477971	0.9999763851

EMC2	1.0828516477	0.6858123481	0.9999763851
TRHR	3.2353357581	0.554787377	0.9999763851
NUDCD1	1.0453846255	0.8572309215	0.9999763851
ENY2	1.1077136231	0.5404840797	0.9999763851
PKHD1L1	0	0	0
EBAG9	1.1684835695	0.5487226903	0.9999763851
CSMD3	0	0	0
TRPS1	0	0	0
EIF3H	1.0852775113	0.5860029758	0.9999763851
UTP23	1.4160905498	0.2158903567	0.9999763851
RAD21	1.171139379	0.2246521849	0.9999763851
SLC30A8	0	0	0
MED30	-1.3483595022	0.1766711155	0.9999763851
EXT1	-1.3158816768	0.8203229797	0.9999763851
SAMD12	-1.315303824	0.8206171563	0.9999763851
MAL2	0	0	0
NOV	-1.3184224589	0.4570199892	0.9999763851
TAF2	-1.0847163728	0.6750476981	0.9999763851
DSCC1	1.128139477	0.4696858834	0.9999763851
DEPTOR	-2.0577216265	0.0432314028	0.9999763851
COL14A1	0	0	0
MRPL13	1.0724863516	0.6673766183	0.9999763851
MTBP	1.242084178	0.5683162366	0.9999763851
SNTB1	-1.1102883523	0.5197689663	0.9999763851
ZHX2	-1.3821220995	0.3949950573	0.9999763851
DERL1	1.1422082637	0.3856336652	0.9999763851
TBC1D31	1.0190916226	0.9543427582	0.9999763851
C8orf76	1.015377145	0.9390940245	0.9999763851
ZHX1-C8orf76	-3.5755910691	0.1288494926	0.9999763851
ZHX1	1.2488043423	0.2292352643	0.9999763851
ATAD2	-1.0851010578	0.6136224313	0.9999763851
WDYHV1	1.6947153005	0.0924906518	0.9999763851
FBXO32	-4.4559126662	0.001263	0.894101927
ANXA13	0	0	0
FAM91A1	1.1496297567	0.4295061752	0.9999763851
FER1L6	0	0	0
TMEM65	1.0960345728	0.5101694289	0.9999763851
TRMT12	1.2294531996	0.5598157328	0.9999763851
RNF139	1.4771475472	0.0272855884	0.9999763851
TATDN1	-1.1878366718	0.2288021274	0.9999763851
NDUFB9	1.0463204234	0.7095143924	0.9999763851
MTSS1	-1.0066325552	0.9897651172	0.9999763851
SQLE	-1.0280527015	0.8654880425	0.9999763851
KIAA0196	1.5787178173	0.0290788851	0.9999763851
NSMCE2	-1.2904312553	0.0617165053	0.9999763851
TRIB1	-2.0623786617	0.3152408669	0.9999763851
MYC	1.1649185816	0.5167043664	0.9999763851
GSDMC	0	0	0
FAM49B	-1.1121637699	0.427671201	0.9999763851

ASAP1	-1.2473570434	0.2416942349	0.9999763851
EFR3A	1.0539072798	0.8051795113	0.9999763851
HHLA1	0	0	0
KCNQ3	1.6240996663	0.5566551786	0.9999763851
LRRC6	-1.3710462586	0.7617555962	0.9999763851
TMEM71	2.0313258944	0.0979654213	0.9999763851
PHF20L1	1.331258793	0.1001838341	0.9999763851
TG	1.595494579	0.2109856831	0.9999763851
SLA	1.1846698446	0.278134793	0.9999763851
WISP1	0	0	0
NDRG1	1.2539587871	0.1571652665	0.9999763851
ST3GAL1	-1.0634767112	0.670490098	0.9999763851
ZFAT	-1.0254325963	0.937538564	0.9999763851
FAM135B	1.0310184702	0.9596632217	0.9999763851
COL22A1	-3.3001189282	0.0758440668	0.9999763851
TRAPPC9	1.0870429928	0.6894414329	0.9999763851
CHRAC1	-1.1099034481	0.6823227514	0.9999763851
AGO2	-1.3248386508	0.1193676222	0.9999763851
DENND3	-1.2502031159	0.1606714613	0.9999763851
SLC45A4	1.2561837095	0.1904482544	0.9999763851
GPR20	-4.2864494036	0.4027150576	0.9999763851
PTP4A3	1.3400821203	0.3853351754	0.9999763851
TSNARE1	-1.1453118218	0.5273941892	0.9999763851
ADGRB1	-1.0300371575	0.9610439186	0.9999763851
JRK	1.4100026603	0.2957597358	0.9999763851
PSCA	-14.1187086092	0.0979429	0.9999763851
LY6K	0	0	0
THEM6	1.0281954075	0.8848886009	0.9999763851
GML	-4.019862227	0.0281217507	0.9999763851
LY6E	-1.4890278981	0.0140810221	0.9999763851
C8orf31	-4.2849908816	0.4028453076	0.9999763851
GPIHBP1	0	0	0
ZFP41	1.1480677451	0.7784327362	0.9999763851
GLI4	1.3406186202	0.7026521051	0.9999763851
ZNF696	1.3704689566	0.3587884482	0.9999763851
TOP1MT	-1.1048386768	0.5748891036	0.9999763851
ZC3H3	1.1020710098	0.6660130065	0.9999763851
GSDMD	-1.1750175617	0.3355162369	0.9999763851
MROH6	-1.079686599	0.8233658746	0.9999763851
NAPRT	1.2473808269	0.2317447378	0.9999763851
EEF1D	-1.0934180838	0.5021319197	0.9999763851
TIGD5	1.570638626	0.2660133584	0.9999763851
PYCRL	1.7379490089	0.2866993556	0.9999763851
TSTA3	1.3481552047	0.1853450499	0.9999763851
ZNF623	-1.3646033691	0.444383834	0.9999763851
ZNF707	1.1234425176	0.7923370859	0.9999763851
SCRIB	1.0172954087	0.9503202189	0.9999763851
PUF60	-1.0827258833	0.6748521137	0.9999763851
NRBP2	-2.3534778273	0.0821357113	0.9999763851

PLEC	-1.0229379022	0.9116160073	0.9999763851
PARP10	-1.8746606282	0.3553885246	0.9999763851
GRINA	-1.0846213695	0.60885892	0.9999763851
OPLAH	1.1900936369	0.8126829972	0.9999763851
EXOSC4	1.0610821823	0.7210151355	0.9999763851
GPAA1	-1.0703091748	0.6587602306	0.9999763851
CYC1	1.1622858615	0.2605545515	0.9999763851
SHARPIN	-1.7190876755	0.0974960092	0.9999763851
MAF1	1.2351562318	0.227698311	0.9999763851
WDR97	-4.2849908816	0.4028453076	0.9999763851
HGH1	1.4220413028	0.2094064918	0.9999763851
MROH1	-1.0196891832	0.9394992958	0.9999763851
BOP1	1.2585413352	0.1649874542	0.9999763851
SCX	-1.9440624723	0.1375665664	0.9999763851
HSF1	-1.0584767853	0.7310300543	0.9999763851
DGAT1	-1.1483201326	0.5596457856	0.9999763851
SCRT1	3.2356557963	0.5547505991	0.9999763851
TMEM249	1.0102638454	0.9884365864	0.9999763851
FBXL6	2.1397134371	0.0435901977	0.9999763851
SLC52A2	1.0979850407	0.5727483592	0.9999763851
ADCK5	1.8999924656	0.1425456128	0.9999763851
CPSF1	1.8688830099	0.007991	0.9999763851
SLC39A4	1.0305225036	0.9051048931	0.9999763851
VPS28	1.144903607	0.4802348406	0.9999763851
TONSL	1.0974633436	0.7240552053	0.9999763851
CYHR1	-1.0305864516	0.8674914018	0.9999763851
KIFC2	0	0	0
FOXH1	1.1130684164	0.6644326683	0.9999763851
PPP1R16A	1.4238206429	0.287474715	0.9999763851
MFSD3	1.1314138894	0.6065094511	0.9999763851
RECQL4	-1.0083886279	0.9589103237	0.9999763851
LRRC14	1.133785151	0.702221855	0.9999763851
LRRC24	1.4321406453	0.2484986505	0.9999763851
C8orf82	1.0229226905	0.9695079924	0.9999763851
ARHGAP39	-1.3157515262	0.8223957341	0.9999763851
ZNF251	-1.6880094949	0.0619954524	0.9999763851
ZNF34	-1.1956050279	0.7982698734	0.9999763851
RPL8	1.0666206141	0.5893552547	0.9999763851
ZNF517	-1.2212653632	0.664385966	0.9999763851
ZNF7	-1.0068064973	0.9856165711	0.9999763851
COMMD5	-1.3639574501	0.0528740479	0.9999763851
ZNF250	-1.0482436097	0.8789975961	0.9999763851
ZNF16	-1.0561204258	0.8442608948	0.9999763851
LOC107986987	0	0	0
C8orf33	1.0413633888	0.8384075092	0.9999763851
LOC107983994	5.4770877341	0.3746135179	0.9999763851
WASH1	1.113945423	0.5537330394	0.9999763851
FOXD4	7.7488609396	0.3060538318	0.9999763851
CBWD1	1.7572156823	0.001936	0.9999763851

DOCK8	1.0675372946	0.8059410437	0.9999763851
C9orf66	2.8791826771	0.2412543662	0.9999763851
KANK1	1.0799577951	0.6993337387	0.9999763851
DMRT1	0	0	0
SMARCA2	1.2894108424	0.137443788	0.9999763851
VLDLR	-1.286676189	0.4723572827	0.9999763851
PUM3	-1.2385201276	0.1627017927	0.9999763851
RFX3	-2.0558572505	0.0830436661	0.9999763851
GLIS3	3.2353357581	0.554787377	0.9999763851
PLPP6	1.3620056689	0.5501900276	0.9999763851
CDC37L1	-1.0602463444	0.8734924755	0.9999763851
AK3	1.254055931	0.2667370385	0.9999763851
RCL1	-1.3349517858	0.1589117894	0.9999763851
JAK2	-1.0635819984	0.858200087	0.9999763851
INSL6	1.4853712947	0.5117403326	0.9999763851
RLN1	0	0	0
PLGRKT	-1.3449836187	0.5040923813	0.9999763851
CD274	-1.3151016298	0.8210852002	0.9999763851
PDCD1LG2	3.2353357581	0.554787377	0.9999763851
RIC1	-1.0361301795	0.9055663952	0.9999763851
ERMP1	-1.1451616851	0.545899785	0.9999763851
KIAA2026	-1.188449319	0.5327026192	0.9999763851
MLANA	-1.7654496905	0.5421321503	0.9999763851
RANBP6	-1.1104141706	0.6890721437	0.9999763851
UHRF2	-1.0726883997	0.7340796006	0.9999763851
GLDC	-1.3158816768	0.8203229797	0.9999763851
KDM4C	1.0512338861	0.780697094	0.9999763851
TMEM261	1.4010109169	0.1370926971	0.9999763851
PTPRD	-1.30626541	0.6126738185	0.9999763851
LURAP1L	-2.3217011239	0.4355766872	0.9999763851
FREM1	0	0	0
TTC39B	0	0	0
SNAPC3	-1.1917196451	0.4498228765	0.9999763851
PSIP1	-1.1465956252	0.4414994755	0.9999763851
CCDC171	1.359700539	0.5933881588	0.9999763851
BNC2	-1.3297810912	0.3509675909	0.9999763851
CNTLN	-1.4693925787	0.0653489038	0.9999763851
SH3GL2	-4.2849908816	0.4028453076	0.9999763851
ADAMTSL1	-4.2849908816	0.4028453076	0.9999763851
SAXO1	-1.2843945718	0.6674649296	0.9999763851
RRAGA	-1.1564715988	0.3528735611	0.9999763851
HAUS6	-1.019084379	0.9413109249	0.9999763851
PLIN2	-1.6275512937	0.000384	0.4396020528
DENND4C	-1.503999038	0.1061842909	0.9999763851
RPS6	-1.074565803	0.5474539716	0.9999763851
ACER2	-1.0805963894	0.9257141641	0.9999763851
SLC24A2	1.4200447571	0.6745299961	0.9999763851
MLLT3	1.2162627286	0.6949209112	0.9999763851
FOCAD	-1.0361594383	0.8584237894	0.9999763851

HACD4	-1.0609572332	0.8538375093	0.9999763851
KLHL9	-1.0913605166	0.6904713634	0.9999763851
MTAP	-1.2430941661	0.3280349121	0.9999763851
CDKN2A	1.0073527891	0.9616884703	0.9999763851
CDKN2B	1.0951597789	0.8919882384	0.9999763851
CAAP1	1.0916188512	0.7738700686	0.9999763851
PLAA	-1.0963055653	0.7327621294	0.9999763851
IFT74	-1.0560434138	0.9108691523	0.9999763851
LRRC19	5.4781708352	0.3745527127	0.9999763851
MOB3B	-1.1173334845	0.822305597	0.9999763851
IFNK	0	0	0
C9orf72	-1.1255766836	0.7168536833	0.9999763851
ACO1	-1.2808471111	0.2080097038	0.9999763851
DDX58	-1.462352467	0.4737739818	0.9999763851
TOPORS	-1.2032631612	0.4928868525	0.9999763851
NDUFB6	1.1247594267	0.5844688715	0.9999763851
TAF1L	1.2868359435	0.8211331271	0.9999763851
APTX	1.3672617029	0.1035447404	0.9999763851
DNAJA1	1.0322507328	0.8329630152	0.9999763851
SMU1	1.2776277247	0.1747514848	0.9999763851
B4GALT1	-1.2728642678	0.0989527069	0.9999763851
SPINK4	1.0273967864	0.9753446573	0.9999763851
BAG1	1.0303231931	0.8537297507	0.9999763851
CHMP5	-1.3687833855	0.0339452197	0.9999763851
NFX1	-1.0981250715	0.6616282509	0.9999763851
AQP3	9.9614219348	0.2204168573	0.9999763851
NOL6	1.3645800931	0.1184296003	0.9999763851
UBE2R2	-1.2075737288	0.2456451681	0.9999763851
UBAP2	1.2805906892	0.255651132	0.9999763851
DCAF12	-1.324226696	0.0856354568	0.9999763851
UBAP1	1.2327121093	0.1614126723	0.9999763851
KIF24	1.0271487798	0.9777810704	0.9999763851
NUDT2	-1.332579958	0.3420838718	0.9999763851
KIAA1161	-1.1896333359	0.5976767418	0.9999763851
C9orf24	-1.5066055279	0.2624813462	0.9999763851
FAM219A	2.6539157614	0.2942499482	0.9999763851
ENHO	1.027644744	0.9776324385	0.9999763851
RPP25L	1.0423548825	0.842720602	0.9999763851
DCTN3	1.3148100676	0.1579803451	0.9999763851
ARID3C	3.2353357581	0.554787377	0.9999763851
SIGMAR1	1.1152701893	0.5920838885	0.9999763851
GALT	1.3046695472	0.5404700004	0.9999763851
IL11RA	-1.1025877923	0.7334223836	0.9999763851
CCL27	0	0	0
LOC730098	-4.2849908816	0.4028453076	0.9999763851
PHF24	1.0274983165	0.9757328476	0.9999763851
FAM205C	0	0	0
DNAJB5	-1.9496141069	0.3129399936	0.9999763851
VCP	1.1012850307	0.455182609	0.9999763851

FANCG	-1.2097506569	0.3369959682	0.9999763851
PIGO	1.6101837194	0.2473061134	0.9999763851
STOML2	-1.1471538264	0.2794893894	0.9999763851
FAM214B	-2.1173141197	0.1342190419	0.9999763851
UNC13B	1.4398911185	0.2865945989	0.9999763851
RUSC2	-1.6382503713	0.2993683659	0.9999763851
FAM166B	0	0	0
TESK1	1.6940282912	0.2714177434	0.9999763851
CD72	1.0272717886	0.9763454022	0.9999763851
SIT1	0	0	0
CCDC107	-1.0202292189	0.9353385008	0.9999763851
ARHGEF39	1.0534203474	0.8892972107	0.9999763851
TPM2	-5.5810225657	0.1050200998	0.9999763851
TLN1	1.092304729	0.6344824079	0.9999763851
CREB3	-1.1130833213	0.7104055241	0.9999763851
GBA2	-1.2656798021	0.6073026954	0.9999763851
RGP1	3.2361532718	0.5546934395	0.9999763851
NPR2	-1.4086684429	0.1064340838	0.9999763851
SPAG8	-14.1187086092	0.0980455964	0.9999763851
HINT2	1.1083919763	0.6224783917	0.9999763851
TMEM8B	1.2493247399	0.7057007986	0.9999763851
RECK	1.2841082437	0.3623663114	0.9999763851
GLIPR2	-1.1127550279	0.5342069247	0.9999763851
CLTA	1.0929897714	0.4987387607	0.9999763851
GNE	-1.0508762794	0.8590682033	0.9999763851
RNF38	1.1325554197	0.6552862676	0.9999763851
MELK	1.0582336841	0.8026796482	0.9999763851
PAX5	1.4704803692	0.3438352433	0.9999763851
ZCCHC7	-1.6270953275	0.0386594921	0.9999763851
GRHPR	1.1346084002	0.5347723412	0.9999763851
ZBTB5	1.1527925122	0.6317775019	0.9999763851
POLR1E	1.1031467499	0.700662701	0.9999763851
FBXO10	-1.4337189649	0.0661498201	0.9999763851
TOMM5	1.2489976691	0.0840916173	0.9999763851
FRMPD1	-1.2075354762	0.7804751596	0.9999763851
TRMT10B	1.0520558225	0.8564409238	0.9999763851
EXOSC3	1.0314823313	0.8879004606	0.9999763851
DCAF10	1.1330332019	0.4546308502	0.9999763851
SLC25A51	1.6703467261	0.4627637299	0.9999763851
SHB	1.1967819733	0.7262616107	0.9999763851
ALDH1B1	1.1627563179	0.5187027347	0.9999763851
CNTNAP3	-1.3158816768	0.8203229797	0.9999763851
ANKRD20A2	0	0	0
LOC101929959	3.2361532718	0.5546934395	0.9999763851
CBWD6	-11.3553837208	0.0264067104	0.9999763851
CNTNAP3B	1.5763434412	0.4248543391	0.9999763851
LOC107984126	7.4933530102	0.2985955846	0.9999763851
LOC100289279	0	0	0
LOC107987001	-1.8920890108	0.2402718518	0.9999763851

LOC107987020	1.7450428903	0.6325021146	0.9999763851
LOC100996643	1.2869488883	0.8204454189	0.9999763851
ANKRD20A4	0	0	0
LOC107987067	-4.2864494036	0.4027150576	0.9999763851
LOC105379443	0	0	0
CBWD5	1.2138491204	0.2400487238	0.9999763851
ZNF658	-1.3154339304	0.8205576571	0.9999763851
ANKRD20A1	1.0279042192	0.9761167157	0.9999763851
CBWD3	-1.9401132992	0.003241	0.9999763851
PIP5K1B	-1.1899619792	0.5994777923	0.9999763851
FAM122A	1.3122760622	0.5156070774	0.9999763851
FXN	1.1300369432	0.5176900858	0.9999763851
TJP2	-1.4153779564	0.0902885729	0.9999763851
PTAR1	-1.1401087448	0.6650630895	0.9999763851
C9orf135	0	0	0
MAMDC2	1.0088696185	0.9438432389	0.9999763851
SMC5	-1.0463368722	0.812350064	0.9999763851
KLF9	-1.6070866372	0.2530441111	0.9999763851
TRPM3	3.2361532718	0.5546934395	0.9999763851
TMEM2	-1.6853462283	0.0339196004	0.9999763851
ABHD17B	1.1368964633	0.6891996259	0.9999763851
C9orf85	-1.3681500532	0.427184063	0.9999763851
GDA	-1.6472991724	0.452777616	0.9999763851
ZFAND5	1.0784688248	0.5985702405	0.9999763851
TMC1	-2.6922490556	0.2893265166	0.9999763851
ALDH1A1	5.4776292235	0.3745831165	0.9999763851
ANXA1	1.0779464342	0.7785584415	0.9999763851
RORB	-1.4385420949	0.4289731639	0.9999763851
C9orf40	1.4134133827	0.0492885953	0.9999763851
CARNMT1	1.4932725115	0.0940573791	0.9999763851
NMRK1	-1.3521723279	0.4955567366	0.9999763851
OSTF1	-1.0296781401	0.9102486598	0.9999763851
PCSK5	2.7026796819	0.199886923	0.9999763851
RFK	-1.362654174	0.0423335258	0.9999763851
GCNT1	-1.0988339395	0.7054074501	0.9999763851
PRUNE2	-2.5042299531	0.0698735021	0.9999763851
VPS13A	-1.1420159437	0.5941254024	0.9999763851
GNAQ	-1.0863454815	0.6173534542	0.9999763851
CEP78	1.3945438682	0.1724688251	0.9999763851
PSAT1	-1.0128039403	0.9301406448	0.9999763851
TLE4	-1.1630806134	0.4963753339	0.9999763851
FRMD3	-1.1818290031	0.3042318996	0.9999763851
IDNK	1.5304942689	0.379398497	0.9999763851
UBQLN1	-1.067817395	0.6322523689	0.9999763851
GKAP1	-1.2032710508	0.7133251699	0.9999763851
KIF27	-1.4338155876	0.4815054425	0.9999763851
C9orf64	1.6862854291	0.3095196926	0.9999763851
HNRNPK	-1.4293757942	0.003499	0.9999763851
RMI1	1.0680407692	0.8195597189	0.9999763851

SLC28A3	-4.2849908816	0.4028453076	0.9999763851
NTRK2	3.2356557963	0.5547505991	0.9999763851
AGTPBP1	-1.1742719589	0.4001740998	0.9999763851
NAA35	-1.0621056295	0.7044102318	0.9999763851
GOLM1	1.4207068373	0.0516695317	0.9999763851
C9orf153	0	0	0
ISCA1	1.4032643055	0.127331769	0.9999763851
ZCCHC6	-1.0297707304	0.8964874425	0.9999763851
GAS1	1.8145193361	0.5774836285	0.9999763851
DAPK1	-1.0460465895	0.757358437	0.9999763851
CTSL	-4.287394647	0.4026306792	0.9999763851
CDK20	-1.3954727771	0.7076496332	0.9999763851
SPIN1	-1.2196894768	0.2477990573	0.9999763851
NXNL2	0	0	0
S1PR3	1.4793732198	0.3346515661	0.9999763851
SHC3	1.3575315598	0.6157286415	0.9999763851
CKS2	-1.0406973934	0.8282783276	0.9999763851
SECISBP2	-1.0687514163	0.7957017286	0.9999763851
SEMA4D	1.1427203064	0.4388256282	0.9999763851
SYK	1.1313540501	0.3965117539	0.9999763851
AUH	-1.190381716	0.5349905664	0.9999763851
NFIL3	-1.2043991989	0.4937630668	0.9999763851
ROR2	1.2869631997	0.8235334545	0.9999763851
SPTLC1	-1.4657871867	0.0149521168	0.9999763851
IARS	1.103030641	0.5906097314	0.9999763851
NOL8	1.436064063	0.0916866956	0.9999763851
CENPP	-1.3204363091	0.1744050445	0.9999763851
OGN	5.4781708352	0.3745527127	0.9999763851
OMD	3.2361532718	0.5546934395	0.9999763851
ASPN	5.4781708352	0.3745527127	0.9999763851
ECM2	-2.3893004408	0.2387173544	0.9999763851
IPPK	1.4991995774	0.3648071119	0.9999763851
BICD2	-1.2916019732	0.4281712414	0.9999763851
ZNF484	-1.9665380586	0.4655882819	0.9999763851
FGD3	-1.2295235471	0.5969595849	0.9999763851
CARD19	1.2158417811	0.3275374437	0.9999763851
NINJ1	-1.2170866783	0.578778076	0.9999763851
WNK2	0	0	0
FAM120AOS	1.3578926719	0.2379449237	0.9999763851
FAM120A	1.0710973731	0.6078472425	0.9999763851
PHF2	1.2282530599	0.4613503359	0.9999763851
BARX1	2.3819299027	0.2314664695	0.9999763851
PTPDC1	1.1543701575	0.7890880015	0.9999763851
ZNF169	-1.1616341521	0.7783152214	0.9999763851
NUTM2F	-2.4668543404	0.4373911847	0.9999763851
LOC728026	-1.1718061449	0.7025896338	0.9999763851
MFSD14B	1.1121773183	0.5872157249	0.9999763851
FBP1	0	0	0
C9orf3	-1.1546673765	0.5890268125	0.9999763851

FANCC	-1.6549455077	0.1909639688	0.9999763851
PTCH1	-1.357946363	0.4686064683	0.9999763851
ERCC6L2	1.2950503608	0.3950369837	0.9999763851
HSD17B3	0	0	0
SLC35D2	0	0	0
ZNF367	-1.0281705436	0.8990507777	0.9999763851
HABP4	-1.3619727376	0.322693256	0.9999763851
CDC14B	-1.7346410473	0.4884043883	0.9999763851
AAED1	-1.8929385914	0.2099174747	0.9999763851
ZNF510	-1.7663530955	0.3353361767	0.9999763851
ZNF782	1.3667310916	0.5973777972	0.9999763851
NUTM2G	-2.3222130951	0.4504474497	0.9999763851
CTSV	1.2869488883	0.8206129236	0.9999763851
TDRD7	-2.148568609	0.0408512148	0.9999763851
TMOD1	-1.1240435479	0.8529602621	0.9999763851
TSTD2	1.7446573669	0.0781923322	0.9999763851
NCBP1	1.4897704265	0.5576337855	0.9999763851
XPA	1.1162406597	0.5360741526	0.9999763851
TRMO	1.3142360065	0.3210206791	0.9999763851
ANP32B	1.0117423553	0.9217857533	0.9999763851
NANS	1.1140620628	0.5009822794	0.9999763851
TRIM14	1.014874258	0.9392974891	0.9999763851
CORO2A	-1.0232790478	0.9082262173	0.9999763851
TBC1D2	1.0164134504	0.935990877	0.9999763851
GABBR2	1.0631544433	0.894611596	0.9999763851
ANKS6	1.2107734211	0.4076721036	0.9999763851
COL15A1	16.6876660904	0.1287829899	0.9999763851
TGFBR1	-1.1401824273	0.3219450074	0.9999763851
ALG2	-1.0416911523	0.8561765936	0.9999763851
SEC61B	1.2267343354	0.1534653	0.9999763851
NR4A3	0	0	0
STX17	1.8463964425	0.026081221	0.9999763851
ERP44	1.1087897422	0.6618038501	0.9999763851
INVS	-1.3136773018	0.2531361402	0.9999763851
TEX10	1.2288383165	0.3550916404	0.9999763851
MSANTD3	1.0746461843	0.7648923409	0.9999763851
MSANTD3-TMEFF1	-1.4718977929	0.5502694686	0.9999763851
MURC	0	0	0
PLPPR1	0	0	0
MRPL50	-1.0543064498	0.8359422044	0.9999763851
ZNF189	3.1348667383	0.076064056	0.9999763851
ALDOB	0	0	0
RNF20	-1.2852122563	0.2580008927	0.9999763851
GRIN3A	-1.8165815049	0.4742437528	0.9999763851
SMC2	1.0683906703	0.6369647491	0.9999763851
NIPSNAP3A	-1.0317174994	0.8563318859	0.9999763851
NIPSNAP3B	-1.0054744133	0.9910384289	0.9999763851
ABCA1	-7.5622178268	0.2177145568	0.9999763851
SLC44A1	-1.085702025	0.6285100873	0.9999763851

FSD1L	1.2153836787	0.6151676368	0.9999763851
FKTN	1.3302725252	0.4390117041	0.9999763851
TAL2	0	0	0
TMEM38B	-1.2736952199	0.1639780788	0.9999763851
ZNF462	0	0	0
RAD23B	1.0481662113	0.743501764	0.9999763851
KLF4	1.600981085	0.4582199418	0.9999763851
IKBKAP	1.4303035818	0.0676989456	0.9999763851
FAM206A	-1.011503618	0.9497869076	0.9999763851
CTNNAL1	-1.044395343	0.8759427304	0.9999763851
TMEM245	-1.1104836154	0.6441189726	0.9999763851
EPB41L4B	0	0	0
PTPN3	1.0078917917	0.9793671819	0.9999763851
AKAP2	-1.1527631095	0.3194495306	0.9999763851
C9orf152	0	0	0
TXN	1.0090728589	0.9612274003	0.9999763851
TXNDC8	-1.0351581998	0.9578046258	0.9999763851
SVEP1	1.6609402791	0.2713423199	0.9999763851
MUSK	0	0	0
LPAR1	-1.8172614764	0.4924033287	0.9999763851
KIAA0368	-1.3042631738	0.1040416369	0.9999763851
PTGR1	0	0	0
DNAJC25	2.2397267087	0.1155137259	0.9999763851
DNAJC25-GNG10	-4.4737841935	0.0703526802	0.9999763851
GNG10	1.0608389507	0.722136205	0.9999763851
C9orf84	-1.427780369	0.5512477001	0.9999763851
UGCG	-1.1321426257	0.7134735977	0.9999763851
SUSD1	1.9876202062	0.3043609346	0.9999763851
PTBP3	-1.0924213881	0.6406366305	0.9999763851
HSDL2	1.0524349057	0.8118941317	0.9999763851
KIAA1958	1.0058249855	0.9780622566	0.9999763851
INIP	1.0768822303	0.8271359522	0.9999763851
SNX30	1.5651177242	0.3531167973	0.9999763851
SLC46A2	0	0	0
ZNF883	0	0	0
SLC31A2	-1.7559775848	0.1403278028	0.9999763851
FKBP15	1.013790067	0.9690571009	0.9999763851
SLC31A1	1.1464104408	0.4677542534	0.9999763851
CDC26	1.0982423741	0.6993308942	0.9999763851
PRPF4	1.3186822302	0.2162940327	0.9999763851
RNF183	3.2356557963	0.5547505991	0.9999763851
WDR31	-1.9492754797	0.3439396048	0.9999763851
BSPRY	1.1329024962	0.756123277	0.9999763851
HDHD3	1.1333354589	0.8698393542	0.9999763851
ALAD	-1.284404498	0.5357906094	0.9999763851
POLE3	-1.0561884249	0.6611029933	0.9999763851
C9orf43	-4.2864494036	0.4027150576	0.9999763851
RGS3	1.6249017024	0.5570296953	0.9999763851
ZNF618	-1.0594156207	0.8654115327	0.9999763851

COL27A1	2.8197880227	0.1994971414	0.9999763851
ORM1	-1.3154339304	0.8205576571	0.9999763851
ORM2	3.2361532718	0.5546934395	0.9999763851
AKNA	1.224487139	0.3359902349	0.9999763851
WHRN	-1.6481169983	0.4665104648	0.9999763851
ATP6V1G1	-1.1423900637	0.3052842433	0.9999763851
C9orf91	-1.6250767384	0.1158699236	0.9999763851
TNFSF15	-14.1218219134	0.1012622274	0.9999763851
DEC1	0	0	0
PAPPA	1.2868359435	0.821136059	0.9999763851
ASTN2	1.6401870795	0.1024958053	0.9999763851
TRIM32	-1.818898251	0.2492423032	0.9999763851
TLR4	1.75912153	0.0341797958	0.9999763851
BRINP1	0	0	0
CDK5RAP2	-1.4112567957	0.081580631	0.9999763851
MEGF9	-1.1602953579	0.6474487167	0.9999763851
FBXW2	1.1486772943	0.4468640067	0.9999763851
PSMD5	-1.1887559249	0.3860863664	0.9999763851
PHF19	1.4530850141	0.0286678342	0.9999763851
TRAF1	-1.3719041759	0.7491476502	0.9999763851
C5	2.309949263	0.1695128747	0.9999763851
CNTRL	-1.058947545	0.7968771668	0.9999763851
RAB14	-1.6153811884	0.001304	0.9116837145
GSN	-1.8319891166	0.0955440429	0.9999763851
STOM	1.4673073682	0.0246239673	0.9999763851
DAB2IP	-1.9668180936	0.4711036877	0.9999763851
TTLL11	1.7726991575	0.0563574559	0.9999763851
NDUFA8	-1.0104669066	0.9448903897	0.9999763851
MORN5	-4.2849908816	0.4028453076	0.9999763851
RBM18	1.3235099296	0.2969998803	0.9999763851
MRRF	1.6409889624	0.0983889626	0.9999763851
PTGS1	-1.4368458787	0.041749375	0.9999763851
OR1J2	0	0	0
PDCL	-1.0352849975	0.8662318594	0.9999763851
RC3H2	-1.1951838885	0.6286076329	0.9999763851
ZBTB6	1.7018174596	0.259281846	0.9999763851
ZBTB26	-1.9659101454	0.4330083403	0.9999763851
RABGAP1	1.0734628279	0.7638854644	0.9999763851
STRBP	-1.2457557502	0.2543073657	0.9999763851
CRB2	0	0	0
DENND1A	1.0019349318	0.9925034682	0.9999763851
LHX2	-2.4601618341	0.0682953778	0.9999763851
NEK6	-1.2586818316	0.1200994363	0.9999763851
PSMB7	1.0854624161	0.5135004734	0.9999763851
NR5A1	0	0	0
NR6A1	1.0180746967	0.9419269609	0.9999763851
OLFML2A	1.1066314536	0.862106592	0.9999763851
WDR38	3.2353357581	0.554787377	0.9999763851
RPL35	1.2920617182	0.0417266139	0.9999763851

ARPC5L	-1.0511098053	0.7333350909	0.9999763851
GOLGA1	1.6446863712	0.3175854061	0.9999763851
SCAI	-1.1755828149	0.6067591909	0.9999763851
PPP6C	-1.0293860456	0.8702081381	0.9999763851
RABEPK	1.0599298826	0.7616606244	0.9999763851
HSPA5	-1.1930980902	0.172358332	0.9999763851
GAPVD1	1.1529852506	0.5841194324	0.9999763851
MAPKAP1	1.4446388626	0.0569625996	0.9999763851
PBX3	-1.3109147982	0.086707122	0.9999763851
MVB12B	-1.0124255437	0.9354910183	0.9999763851
ZBTB43	-1.0228293514	0.927577496	0.9999763851
ZBTB34	-1.0718982213	0.8649662694	0.9999763851
RALGPS1	-1.1138651893	0.8187207939	0.9999763851
GARNL3	-1.8725992231	0.3205258278	0.9999763851
ZNF79	2.799028425	0.126607932	0.9999763851
RPL12	1.0534319819	0.6930457963	0.9999763851
LRSAM1	-1.2403239507	0.5756742367	0.9999763851
FAM129B	1.236026333	0.2724398645	0.9999763851
STXBP1	1.1627248867	0.5274164534	0.9999763851
PTRH1	1.0275809886	0.9576395509	0.9999763851
TTC16	0	0	0
TOR2A	1.044169403	0.8803191232	0.9999763851
SH2D3C	1.3775907027	0.4834760416	0.9999763851
CDK9	1.127554366	0.7641117508	0.9999763851
FPGS	-1.0150951592	0.9320822655	0.9999763851
ENG	1.0031215334	0.9884980291	0.9999763851
AK1	1.3262752232	0.7420374029	0.9999763851
ST6GALNAC6	1.4843598846	0.2442921497	0.9999763851
ST6GALNAC4	-1.4018717905	0.2821044931	0.9999763851
PIP5KL1	-1.1245500639	0.8352721746	0.9999763851
DPM2	1.67547459	0.0913757914	0.9999763851
FAM102A	1.1111395401	0.7250182304	0.9999763851
NAIF1	1.4028425303	0.2595311101	0.9999763851
SLC25A25	1.3978085319	0.2397968853	0.9999763851
PTGES2	-1.0906524881	0.7648119467	0.9999763851
C9orf16	-1.2547198293	0.4846212878	0.9999763851
CIZ1	-1.0096991286	0.9619064114	0.9999763851
DNM1	1.6601904867	0.3624463587	0.9999763851
GOLGA2	-1.4981538449	0.0513578247	0.9999763851
SWI5	-1.1951432965	0.5387780167	0.9999763851
TRUB2	1.1760665768	0.5338993292	0.9999763851
COQ4	1.1589198719	0.5460528317	0.9999763851
SLC27A4	1.1348932798	0.7056260352	0.9999763851
URM1	-1.2397061803	0.1691425109	0.9999763851
CERCAM	1.0855646724	0.7807757616	0.9999763851
ODF2	1.1424552908	0.5704958561	0.9999763851
GLE1	-1.5193340799	0.00374	0.9999763851
SPTAN1	-1.3166771546	0.2829826359	0.9999763851
WDR34	1.3051552326	0.0994079688	0.9999763851

SET	1.1230899437	0.3075123262	0.9999763851
PKN3	1.7669460201	0.1885514336	0.9999763851
ZDHHC12	1.4758880504	0.267943328	0.9999763851
ZER1	-1.71960772	0.1148946035	0.9999763851
TBC1D13	1.8247620725	0.0389215737	0.9999763851
ENDOG	1.1025993542	0.5697620297	0.9999763851
C9orf114	1.6621407841	0.1633260743	0.9999763851
KYAT1	-1.9803152966	0.0498709869	0.9999763851
LRRC8A	1.1089656077	0.6728515864	0.9999763851
PHYHD1	0	0	0
DOLK	1.8301434462	0.1860975807	0.9999763851
NUP188	-1.039693804	0.8085143696	0.9999763851
SH3GLB2	-1.0099838896	0.9781795044	0.9999763851
FAM73B	-1.4060752702	0.2335662843	0.9999763851
DOLPP1	1.4209833715	0.452821417	0.9999763851
CRAT	-1.1387519898	0.6993705861	0.9999763851
PPP2R4	1.3989763488	0.1164584117	0.9999763851
IER5L	1.0475909463	0.8401625156	0.9999763851
NTMT1	7.5217611033	0.0411721632	0.9999763851
ASB6	1.0120460414	0.9442584918	0.9999763851
PRRX2	0	0	0
PTGES	-3.0819688013	0.1350901342	0.9999763851
TOR1B	1.518254932	0.0972609679	0.9999763851
TOR1A	1.0263693285	0.8898699628	0.9999763851
C9orf78	-1.0614348868	0.6402072989	0.9999763851
USP20	-1.1081496276	0.5764788525	0.9999763851
FNBP1	1.0210297633	0.8679828672	0.9999763851
GPR107	-1.4025319731	0.0782784902	0.9999763851
NCS1	-1.0337384718	0.9007806609	0.9999763851
HMCN2	0	0	0
ASS1	-1.6107004734	0.0542474448	0.9999763851
FUBP3	-1.4195844395	0.0789679395	0.9999763851
PRDM12	0	0	0
EXOSC2	1.3291888385	0.2333034164	0.9999763851
ABL1	-1.0077983939	0.9678482478	0.9999763851
QRFP	-4.2849908816	0.4028453076	0.9999763851
FIBCD1	1.4043593911	0.3255833197	0.9999763851
AIF1L	0	0	0
NUP214	1.1156137814	0.5879924611	0.9999763851
FAM78A	1.1782453432	0.4984769062	0.9999763851
PRRC2B	-1.3382310599	0.1421058016	0.9999763851
POMT1	13.0486682238	0.181312288	0.9999763851
UCK1	1.1825037047	0.6281291508	0.9999763851
LOC642515	0	0	0
RAPGEF1	1.1473032841	0.54928026	0.9999763851
MED27	1.0463616764	0.7730314365	0.9999763851
NTNG2	-1.3001131679	0.3424686551	0.9999763851
SETX	1.2787987097	0.3227772159	0.9999763851
TTF1	1.2772068104	0.1985636284	0.9999763851

CFAP77	-4.2864494036	0.4027150576	0.9999763851
DDX31	1.0859188704	0.7535187093	0.9999763851
GTF3C4	-1.1043339428	0.5721874532	0.9999763851
AK8	-3.3294360969	0.2395755018	0.9999763851
SPACA9	2.7885870269	0.0921679679	0.9999763851
TSC1	1.1470839235	0.632873079	0.9999763851
GTF3C5	-1.0260264395	0.8743203179	0.9999763851
CEL	-4.3342515471	0.1405566338	0.9999763851
RALGDS	-1.2842198819	0.1381662173	0.9999763851
GBGT1	1.9906522869	0.2910050433	0.9999763851
SURF6	1.1486704957	0.3956251896	0.9999763851
MED22	-1.0395883013	0.880718955	0.9999763851
RPL7A	-1.06939035	0.5627175207	0.9999763851
SURF1	-1.1735182106	0.5484984984	0.9999763851
SURF2	1.2672731558	0.5708994064	0.9999763851
SURF4	1.0453219074	0.7688279804	0.9999763851
STKLD1	1.1336585844	0.8772375055	0.9999763851
REXO4	1.4370362158	0.0542609243	0.9999763851
ADAMTS13	1.8136032532	0.5941211288	0.9999763851
CACFD1	3.4758470146	0.0468823228	0.9999763851
SLC2A6	7.7211724672	0.2779709379	0.9999763851
ADAMTSL2	0	0	0
SARDH	0	0	0
VAV2	-1.3107985259	0.1528647798	0.9999763851
BRD3	1.0074820752	0.9626297635	0.9999763851
WDR5	-1.0076796777	0.9592606505	0.9999763851
RXRA	1.3758142483	0.0328517095	0.9999763851
COL5A1	0	0	0
FCN1	1.0064028117	0.9866277677	0.9999763851
PPP1R26	1.6870764756	0.332925959	0.9999763851
C9orf116	-1.0853454036	0.8981327655	0.9999763851
MRPS2	1.0291485821	0.8439241733	0.9999763851
GLT6D1	0	0	0
KCNT1	0	0	0
CAMSAP1	-1.1555210169	0.6472058107	0.9999763851
UBAC1	1.005018407	0.9719600287	0.9999763851
NACC2	1.5039737695	0.0761470461	0.9999763851
C9orf69	1.0955650147	0.6156175497	0.9999763851
LOC107987142	1.287203384	0.8313351269	0.9999763851
LHX3	3.2353357581	0.554787377	0.9999763851
QSOX2	1.3451472929	0.3482153789	0.9999763851
CCDC187	0	0	0
GPSM1	0	0	0
DNLZ	-1.2523681884	0.4381207828	0.9999763851
CARD9	-1.8497396327	0.1951733951	0.9999763851
SNAPC4	-1.0017726746	0.9960124783	0.9999763851
SDCCAG3	-1.2440123885	0.2282967124	0.9999763851
PMPCA	1.0754244855	0.6940360869	0.9999763851
INPP5E	2.3255510872	0.0835396976	0.9999763851

SEC16A	-1.0597433903	0.7677601567	0.9999763851
NOTCH1	1.2893689256	0.1882271126	0.9999763851
EGFL7	-1.7420007258	0.2244504392	0.9999763851
AGPAT2	1.1852434809	0.3513087217	0.9999763851
FAM69B	-1.2593896088	0.320775637	0.9999763851
LCN10	-4.2849908816	0.4028453076	0.9999763851
LCN6	0	0	0
TMEM141	-1.0402064156	0.8530510203	0.9999763851
CCDC183	1.2671911569	0.6528299093	0.9999763851
RABL6	1.2440132253	0.1873741489	0.9999763851
PHPT1	-1.0474055508	0.8062161965	0.9999763851
MAMDC4	3.3952287445	0.2424412637	0.9999763851
EDF1	1.1087027575	0.4566736237	0.9999763851
TRAF2	1.0153505021	0.9648317337	0.9999763851
FBXW5	1.0155705136	0.9325273408	0.9999763851
C8G	-2.4440355816	0.4436151611	0.9999763851
C9orf142	-1.0759936984	0.8209212899	0.9999763851
CLIC3	0	0	0
ABCA2	20.2949411759	0.1207307807	0.9999763851
C9orf139	1.2865109866	0.8334383935	0.9999763851
FUT7	1.0479999936	0.8870749341	0.9999763851
NPDC1	-1.0144903535	0.9555278816	0.9999763851
ENTPD2	0	0	0
SAPCD2	1.2757054906	0.1412237608	0.9999763851
UAP1L1	1.3997613402	0.118011653	0.9999763851
MAN1B1	1.1165054798	0.6475210862	0.9999763851
DPP7	-1.2678493068	0.1869493223	0.9999763851
LRRC26	0	0	0
ANAPC2	0	0	0
SSNA1	1.1546260337	0.3875936719	0.9999763851
TPRN	1.462610597	0.2135108633	0.9999763851
TMEM203	1.1370696386	0.5282271295	0.9999763851
NDOR1	1.0391995142	0.928692599	0.9999763851
RNF208	1.2865109866	0.8335873235	0.9999763851
CYSRT1	1.8139617088	0.5779223724	0.9999763851
SLC34A3	0	0	0
TUBB4B	1.003098129	0.9831891842	0.9999763851
FAM166A	-1.9663843334	0.4764302308	0.9999763851
NELFB	1.0030753002	0.9852280831	0.9999763851
TOR4A	1.2403195898	0.2842222953	0.9999763851
NRARP	-1.2870893299	0.3998958579	0.9999763851
EXD3	1.242910695	0.5512190762	0.9999763851
NOXA1	4.9748777347	0.1018700777	0.9999763851
NSMF	1.8997470176	0.0465844676	0.9999763851
PNPLA7	1.2868359435	0.8210968834	0.9999763851
MRPL41	1.1355272914	0.4394940949	0.9999763851
DPH7	-1.1310936908	0.573783197	0.9999763851
ZMYND19	-1.2817765109	0.3274005272	0.9999763851
ARRDC1	1.2604856478	0.3505011265	0.9999763851

EHMT1	-1.2238136449	0.3049522544	0.9999763851
FAM157B	1.5276737168	0.2570181449	0.9999763851
ZMYND11	-1.1467792511	0.4538335489	0.9999763851
DIP2C	-2.6587266395	0.1997709661	0.9999763851
LARP4B	-1.1317061308	0.3867909827	0.9999763851
GTPBP4	-1.038136083	0.7918829786	0.9999763851
IDI2	2.4770826173	0.0421920504	0.9999763851
IDI1	-1.1330304916	0.5075727367	0.9999763851
WDR37	-1.3960041553	0.2025955797	0.9999763851
ADARB2	1.8846717628	0.1103997922	0.9999763851
LOC101927824	7.7192231741	0.2782707334	0.9999763851
PFKP	-1.0509401828	0.7860960012	0.9999763851
PITRM1	1.51766418	0.0362139051	0.9999763851
KLF6	-1.5574004387	0.007303	0.9999763851
AKR1C1	7.4933530102	0.2985445178	0.9999763851
AKR1C2	7.7488609396	0.30604422	0.9999763851
AKR1C3	5.4770877341	0.3746135179	0.9999763851
NET1	-1.185198129	0.4235625601	0.9999763851
ASB13	-1.0975922777	0.7453325089	0.9999763851
FAM208B	-1.335126453	0.2428587568	0.9999763851
GDI2	-1.191919218	0.1585779604	0.9999763851
ANKRD16	1.6491085401	0.2178624659	0.9999763851
FBXO18	-1.4457112944	0.2258347345	0.9999763851
IL15RA	-1.2719915409	0.5189030354	0.9999763851
IL2RA	0	0	0
RBM17	-1.0116073204	0.9407391283	0.9999763851
PFKFB3	-1.0828318755	0.635565173	0.9999763851
PRKCQ	-1.3319546752	0.3272350104	0.9999763851
SFMBT2	-1.3929953701	0.1035154592	0.9999763851
KIN	-1.1539615691	0.7449715033	0.9999763851
ATP5C1	-1.2505841073	0.1826801474	0.9999763851
TAF3	1.2865833997	0.2698252271	0.9999763851
GATA3	0	0	0
CELF2	1.1107563488	0.4276428832	0.9999763851
USP6NL	-1.4271299095	0.5799896856	0.9999763851
LOC107984208	0	0	0
ECHDC3	0	0	0
PROSER2	-1.3020932657	0.4581893591	0.9999763851
UPF2	-1.0841753676	0.6902596447	0.9999763851
DHTKD1	-1.0947309863	0.6737401064	0.9999763851
SEC61A2	-1.1078402714	0.7368966347	0.9999763851
NUDT5	1.4483644655	0.0628063733	0.9999763851
CDC123	1.334279998	0.0526424446	0.9999763851
CAMK1D	-1.0898785616	0.7022830768	0.9999763851
CCDC3	0	0	0
OPTN	-1.1879022687	0.4334745196	0.9999763851
MCM10	1.1989046599	0.3912352954	0.9999763851
PHYH	-1.2592876644	0.4164111012	0.9999763851
SEPHS1	1.1534900846	0.3718938703	0.9999763851

BEND7	0	0	0
PRPF18	-1.3038500007	0.3582810102	0.9999763851
FRMD4A	-3.7489679912	0.1056863984	0.9999763851
FAM107B	1.1226709472	0.4339217475	0.9999763851
CDNF	3.2361532718	0.5546934395	0.9999763851
HSPA14	1.2758823837	0.1353027984	0.9999763851
SUV39H2	1.7689525345	0.4240876668	0.9999763851
DCLRE1C	1.0136592644	0.9412542664	0.9999763851
ACBD7	1.2870761236	0.8160709833	0.9999763851
RPP38	-1.0649133204	0.846531529	0.9999763851
NMT2	1.0535512441	0.8326925426	0.9999763851
FAM188A	1.2016035043	0.6436702891	0.9999763851
PTER	-1.2157970631	0.4292372864	0.9999763851
C1QL3	1.7969999196	0.2765017484	0.9999763851
RSU1	1.0898782563	0.5583775796	0.9999763851
TRDMT1	1.7579580407	0.4338806613	0.9999763851
VIM	-3.8760288064	1.92E-05	0.0412638911
HACD1	1.4891726905	0.2330267312	0.9999763851
STAM	-1.282054125	0.4037090904	0.9999763851
TMEM236	3.2361532718	0.5546934395	0.9999763851
MRC1	0	0	0
CACNB2	-1.333277728	0.4114947162	0.9999763851
NSUN6	1.1237523584	0.791579165	0.9999763851
ARL5B	-1.1171101007	0.6392106948	0.9999763851
PLXDC2	-1.9661045084	0.453457533	0.9999763851
SKIDA1	0	0	0
MLLT10	1.6525164228	0.1300049447	0.9999763851
DNAJC1	-1.2946572915	0.1107792618	0.9999763851
COMMD3	-1.007704891	0.9552525588	0.9999763851
COMMD3-BMI1	-1.0687042921	0.9325672115	0.9999763851
BMI1	1.0309157868	0.8033689869	0.9999763851
SPAG6	1.193263566	0.4963395463	0.9999763851
PIP4K2A	1.0571655908	0.7287877592	0.9999763851
ARMC3	0	0	0
MSRB2	1.6000355819	0.0356264605	0.9999763851
OTUD1	2.8672419672	0.3103828854	0.9999763851
KIAA1217	-3.2837744538	0.0545169772	0.9999763851
ARHGAP21	1.1483926048	0.5886486088	0.9999763851
THNSL1	-1.4073673926	0.67478989	0.9999763851
ENKUR	3.2353357581	0.554787377	0.9999763851
APBB1IP	1.2725130525	0.1324931821	0.9999763851
PDSS1	1.3643389243	0.3477032333	0.9999763851
ABI1	1.0542103453	0.7694977714	0.9999763851
ANKRD26	1.0337843191	0.9302757929	0.9999763851
YME1L1	1.2254692216	0.1543758403	0.9999763851
MASTL	-1.5540399947	0.218113573	0.9999763851
ACBD5	2.224467511	0.0228452651	0.9999763851
RAB18	-1.3075363155	0.1008226144	0.9999763851
MKX	0	0	0

ARMC4	-7.5622178268	0.2177145568	0.9999763851
MPP7	1.1411132031	0.5969112488	0.9999763851
WAC	-1.2121870701	0.1944490521	0.9999763851
BAMBI	-1.2190011384	0.4884821042	0.9999763851
SVIL	1.5359848111	0.1301318376	0.9999763851
KIAA1462	1.8778891881	0.1664208711	0.9999763851
MTPAP	-1.0886024732	0.6590545118	0.9999763851
MAP3K8	1.0504056933	0.8161953317	0.9999763851
ZNF438	1.0462705393	0.8101000529	0.9999763851
ZEB1	2.8182416066	0.2019120972	0.9999763851
ARHGAP12	-1.2535571721	0.3563367205	0.9999763851
KIF5B	1.1854652447	0.227901118	0.9999763851
EPC1	-1.0137090768	0.9451838337	0.9999763851
CCDC7	-1.7847353177	0.0671684177	0.9999763851
ITGB1	1.1378174453	0.4582031549	0.9999763851
NRP1	-4.04068853	0.1312233848	0.9999763851
LOC105376488	-4.1323543353	0.2115526896	0.9999763851
PARD3	2.3398344857	0.4133387514	0.9999763851
CUL2	-1.4488995931	0.2113688004	0.9999763851
CREM	1.0235181839	0.929365868	0.9999763851
CCNY	-1.0090830796	0.9485705656	0.9999763851
ANKRD30A	3.2353357581	0.554787377	0.9999763851
ZNF248	-1.107052137	0.7205077955	0.9999763851
ZNF25	-2.2197236441	0.0108170859	0.9999763851
ZNF33A	-1.3621433495	0.0975384407	0.9999763851
ZNF37A	1.486460666	0.0287063449	0.9999763851
ZNF33B	1.2547049557	0.5570972114	0.9999763851
BMS1	-1.1047261569	0.5265509378	0.9999763851
RET	-4.2849908816	0.4028453076	0.9999763851
CSGALNACT2	-1.2636109227	0.2088557988	0.9999763851
RASGEF1A	0	0	0
HNRNPF	-1.0204285988	0.8710719746	0.9999763851
ZNF32	-1.181723761	0.7474101105	0.9999763851
TMEM72	-1.1491729646	0.8481357365	0.9999763851
RASSF4	1.4035550145	0.0728845761	0.9999763851
C10orf25	-4.3342515471	0.1403801422	0.9999763851
ZNF22	1.0526325354	0.7812774213	0.9999763851
ALOX5	3.2356557963	0.5547505991	0.9999763851
MARCH8	-1.3404654384	0.1452398255	0.9999763851
ZFAND4	-1.053758897	0.9198673225	0.9999763851
FAM21C	-1.3285885305	0.2204816431	0.9999763851
FAM25E	3.2353357581	0.554787377	0.9999763851
AGAP4	-1.0052361772	0.9919615135	0.9999763851
TIMM23	1.2998059111	0.1040693291	0.9999763851
NCOA4	1.2686654285	0.0812398639	0.9999763851
ANTXRL	3.2361532718	0.5546934395	0.9999763851
LOC107984026	7.7878683154	0.0271494815	0.9999763851
SYT15	-1.1490595167	0.8521920567	0.9999763851
ANXA8	-1.0987792284	0.7584237601	0.9999763851

AGAP9	1.6243668162	0.5953234889	0.9999763851
FRMPD2	0	0	0
MAPK8	-1.0159569792	0.9514185105	0.9999763851
WDFY4	1.2647554825	0.2852483678	0.9999763851
LRRC18	0	0	0
VSTM4	0	0	0
C10orf128	1.689326647	0.0156308726	0.9999763851
ERCC6	-1.5977494888	0.1395141053	0.9999763851
PGBD3	1.9665069869	0.380756122	0.9999763851
CHAT	0	0	0
C10orf53	3.2353357581	0.554787377	0.9999763851
OGDHL	1.2869488883	0.8207674385	0.9999763851
PARG	1.2001585789	0.6352968538	0.9999763851
TIMM23B	-1.21717731	0.4666456168	0.9999763851
AGAP6	-1.3504062993	0.3579932544	0.9999763851
FAM21A	1.5840102541	0.0434169723	0.9999763851
SGMS1	-1.504530746	0.250601656	0.9999763851
PRKG1	-1.1491729646	0.8482307178	0.9999763851
CSTF2T	1.5072276666	0.0652160916	0.9999763851
ZWINT	1.0778039014	0.611368984	0.9999763851
IPMK	-1.9244394993	0.0125198203	0.9999763851
CISD1	1.2114560708	0.3535086302	0.9999763851
UBE2D1	1.1389064505	0.6711499294	0.9999763851
TFAM	-1.0771487817	0.6554544661	0.9999763851
BICC1	-1.0263302305	0.9539385027	0.9999763851
PHYHIPL	3.2356557963	0.5547505991	0.9999763851
SLC16A9	1.0177424686	0.9586743554	0.9999763851
CCDC6	-1.4483030344	0.0196719726	0.9999763851
CDK1	1.0104413471	0.9420320722	0.9999763851
ARID5B	-3.3284109188	0.2427071832	0.9999763851
RTKN2	0	0	0
ZNF365	0	0	0
ADO	-1.0659611257	0.7579370665	0.9999763851
EGR2	-3.1559763085	0.1811594967	0.9999763851
NRBF2	-1.133777439	0.6141919325	0.9999763851
JMJD1C	-1.2599703224	0.4132101017	0.9999763851
REEP3	0	0	0
DNAJC12	-3.7464240212	0.1398552477	0.9999763851
SIRT1	-1.1258785493	0.630055791	0.9999763851
HERC4	-1.2209854762	0.3604791486	0.9999763851
MYPN	3.2353357581	0.554787377	0.9999763851
PBLD	5.4790129495	0.3757193507	0.9999763851
HNRNPH3	1.0447619908	0.7887177146	0.9999763851
RUFY2	-1.5530955003	0.3276351892	0.9999763851
DNA2	-1.9923980812	0.037019664	0.9999763851
SLC25A16	1.3161558074	0.5299451961	0.9999763851
TET1	-1.7928551696	0.063252743	0.9999763851
CCAR1	-1.024622964	0.8737690131	0.9999763851
STOX1	3.2356557963	0.5547505991	0.9999763851

DDX50	1.0592118339	0.7414076852	0.9999763851
DDX21	1.0939992895	0.4993144937	0.9999763851
KIF1BP	-1.0486347201	0.9170936101	0.9999763851
SRGN	1.1523264374	0.2513396275	0.9999763851
VPS26A	1.358921533	0.096552737	0.9999763851
SUPV3L1	-1.6328194669	0.1075970386	0.9999763851
HKDC1	-7.5647903763	0.2176246281	0.9999763851
HK1	1.005304992	0.9701636763	0.9999763851
TACR2	-1.3154339304	0.8205576571	0.9999763851
TSPAN15	-1.0803829108	0.9216984918	0.9999763851
H2AFY2	0	0	0
AIFM2	1.9216708403	0.4666539967	0.9999763851
TYSND1	-1.0998248599	0.7466721725	0.9999763851
SAR1A	-1.0848474349	0.6150097605	0.9999763851
PPA1	1.3763695697	0.009301	0.9999763851
LRRC20	1.3952985806	0.2593190867	0.9999763851
EIF4EBP2	1.0746257704	0.6374883429	0.9999763851
NODAL	0	0	0
PALD1	-2.4840175982	0.1707209957	0.9999763851
PRF1	0	0	0
ADAMTS14	-1.3710462586	0.7619212495	0.9999763851
SGPL1	-1.508703732	0.2318572489	0.9999763851
PCBD1	1.4287486362	0.0570351191	0.9999763851
SLC29A3	1.1848656123	0.5788389069	0.9999763851
CDH23	1.3259726427	0.7105573285	0.9999763851
C10orf105	2.3407592913	0.42663657	0.9999763851
C10orf54	1.1365701103	0.5863658994	0.9999763851
PSAP	-1.4748597948	0.00178	0.9999763851
SPOCK2	0	0	0
ASCC1	-1.245203271	0.4300244976	0.9999763851
ANAPC16	1.1986417738	0.2601839774	0.9999763851
DDIT4	-1.289757765	0.146270355	0.9999763851
DNAJB12	1.7865457543	0.0545411025	0.9999763851
MICU1	-1.0911841987	0.6327325137	0.9999763851
MCU	-1.4376367353	0.1077307178	0.9999763851
OIT3	1.2169952033	0.8019634658	0.9999763851
P4HA1	-1.1594101938	0.427739862	0.9999763851
NUDT13	1.6110435152	0.2048689835	0.9999763851
ECD	-1.3999454175	0.292157649	0.9999763851
FAM149B1	-1.3822046165	0.3565793379	0.9999763851
DNAJC9	1.2852163259	0.0751107473	0.9999763851
MRPS16	1.4185518436	0.0178177661	0.9999763851
CFAP70	1.2493677488	0.6915260791	0.9999763851
ANXA7	1.0226223617	0.8886032217	0.9999763851
MSS51	-1.9647456674	0.4670688857	0.9999763851
PPP3CB	1.5216261928	0.0274740326	0.9999763851
USP54	-1.1126860973	0.7088829528	0.9999763851
AGAP5	-1.2528461868	0.8154925613	0.9999763851
SEC24C	-1.0922285977	0.6558070164	0.9999763851

FUT11	1.0594535678	0.8501549536	0.9999763851
CHCHD1	1.1007104448	0.5792875639	0.9999763851
ZSWIM8	1.2784112794	0.5009386897	0.9999763851
NDST2	1.1646865819	0.5678188543	0.9999763851
CAMK2G	1.0887092959	0.6588724435	0.9999763851
PLAU	-1.0900485969	0.7008877174	0.9999763851
C10orf55	0	0	0
VCL	-1.2830650381	0.2542161428	0.9999763851
AP3M1	1.5065079695	0.0598650881	0.9999763851
ADK	-1.1084855186	0.5032096961	0.9999763851
KAT6B	1.2912962828	0.5928581364	0.9999763851
DUPD1	1.0803193328	0.8198617506	0.9999763851
DUSP13	0	0	0
SAMD8	-1.0301645798	0.9624348605	0.9999763851
VDAC2	1.1143064241	0.4540496737	0.9999763851
COMTD1	1.5279303089	0.0614057097	0.9999763851
ZNF503	1.0798750465	0.6902751972	0.9999763851
C10orf11	-1.2343803304	0.3107329971	0.9999763851
KCNMA1	3.2361532718	0.5546934395	0.9999763851
DLG5	1.028073281	0.8918154264	0.9999763851
POLR3A	1.2261821008	0.4333704546	0.9999763851
RPS24	1.0077024216	0.952436144	0.9999763851
ZMIZ1	-1.1741355171	0.4127135422	0.9999763851
PPIF	1.1422698771	0.3047606579	0.9999763851
ZCCHC24	-1.4198124442	0.1517123757	0.9999763851
EIF5AL1	1.3761556368	0.1566694688	0.9999763851
NUTM2B	0	0	0
TMEM254	-1.0353098561	0.9275926056	0.9999763851
PLAC9	-2.7159660136	0.1427636224	0.9999763851
ANXA11	1.2224360938	0.2123435703	0.9999763851
FAM213A	-1.9482682667	0.3554043225	0.9999763851
TSPAN14	1.046014878	0.7710395168	0.9999763851
SH2D4B	1.2862273491	0.8293392712	0.9999763851
GHITM	1.2405429994	0.0927058676	0.9999763851
C10orf99	0	0	0
CCSER2	1.0490795055	0.8908703838	0.9999763851
GRID1	-4.2864494036	0.4027150576	0.9999763851
WAPL	1.1934222148	0.2651006538	0.9999763851
LDB3	7.7488609396	0.3060229829	0.9999763851
BMPR1A	1.8462191135	0.2318500436	0.9999763851
GLUD1	-1.056571541	0.7781739575	0.9999763851
FAM35A	-1.0874718771	0.5902706491	0.9999763851
NUTM2A	3.2356557963	0.5547505991	0.9999763851
MINPP1	1.0136112833	0.9711043879	0.9999763851
PAPSS2	0	0	0
ATAD1	1.1625351611	0.5030624858	0.9999763851
KLLN	3.2353357581	0.554787377	0.9999763851
PTEN	-1.064913514	0.6212703293	0.9999763851
RNLS	-1.3305441684	0.531944401	0.9999763851

LIPJ	0	0	0
LIPM	3.2361532718	0.5546934395	0.9999763851
ANKRD22	3.922469612	0.1728578129	0.9999763851
STAMBPL1	-1.7016367315	0.146041691	0.9999763851
ACTA2	-1.4166688595	0.6478521083	0.9999763851
FAS	1.078437726	0.8560118111	0.9999763851
LIPA	-1.0437490334	0.8370651979	0.9999763851
IFIT2	-4.9389863757	0.0459126109	0.9999763851
IFIT3	-4.7124369261	0.0001219	0.1822932891
IFIT1	-4.452374454	0.0149907392	0.9999763851
IFIT5	1.8928577306	0.4866844079	0.9999763851
PANK1	-1.4473774847	0.2217989422	0.9999763851
KIF20B	1.1200770599	0.5400115407	0.9999763851
HTR7	3.2353357581	0.554787377	0.9999763851
RPP30	-1.244019471	0.5010159461	0.9999763851
ANKRD1	0	0	0
PCGF5	-1.419280569	0.1487350531	0.9999763851
TNKS2	1.1371914808	0.5937903876	0.9999763851
FGFBP3	-1.4386212672	0.3966570688	0.9999763851
BTAF1	-1.5341924225	0.1099965165	0.9999763851
CPEB3	1.2643855079	0.5000954591	0.9999763851
MARCH5	-1.5865819262	0.0171344252	0.9999763851
IDE	-1.2752003243	0.3848552952	0.9999763851
KIF11	1.3497019207	0.1713366676	0.9999763851
HHEX	1.1944099268	0.4900875819	0.9999763851
EXOC6	1.7729620821	0.0305741279	0.9999763851
CYP26A1	-4.2849908816	0.4028453076	0.9999763851
MYOF	-1.3957650729	0.7120029752	0.9999763851
CEP55	1.3716572599	0.1873187194	0.9999763851
FRA10AC1	1.0500790048	0.8551031113	0.9999763851
PLCE1	1.9257563044	0.2026850018	0.9999763851
NOC3L	1.1070848958	0.653830896	0.9999763851
TBC1D12	1.2867653957	0.8300995955	0.9999763851
HELLS	1.1175121923	0.5587524477	0.9999763851
CYP2C18	-4.2849908816	0.4028453076	0.9999763851
CYP2C19	0	0	0
CYP2C9	3.2353357581	0.554787377	0.9999763851
CYP2C8	0	0	0
ALDH18A1	-1.354670874	0.0657091427	0.9999763851
TCTN3	1.1547106538	0.4485831446	0.9999763851
ENTPD1	1.1957618756	0.4440006431	0.9999763851
C10orf131	0	0	0
CC2D2B	1.966636009	0.3499667665	0.9999763851
CCNJ	1.1333904196	0.6536258018	0.9999763851
ZNF518A	1.0086801481	0.9666510518	0.9999763851
BLNK	-1.2491748198	0.5871102564	0.9999763851
DNTT	-2.3214980365	0.4365300349	0.9999763851
TLL2	-1.394935021	0.7122094033	0.9999763851
TM9SF3	-1.173085336	0.1987102481	0.9999763851

PIK3AP1	-1.1393698337	0.5166310479	0.9999763851
LCOR	-1.0395907431	0.8539804282	0.9999763851
C10orf12	-1.0163160609	0.9782973355	0.9999763851
SLIT1	1.0299460308	0.9688152998	0.9999763851
ARHGAP19	-1.3825162976	0.0535015858	0.9999763851
FRAT1	1.2592440571	0.3901549251	0.9999763851
FRAT2	1.5836787245	0.004868	0.9999763851
RRP12	1.1988454122	0.3551580394	0.9999763851
LOC644215	2.3406309168	0.4065662218	0.9999763851
PGAM1	1.2847351348	0.0877709456	0.9999763851
EXOSC1	1.152391092	0.5362749721	0.9999763851
ZDHHC16	1.4379219091	0.2356373814	0.9999763851
MMS19	1.0674529083	0.6771594704	0.9999763851
UBTD1	-1.0384170457	0.839000135	0.9999763851
ANKRD2	0	0	0
HOGA1	0	0	0
PI4K2A	1.0523050855	0.8328712017	0.9999763851
AVPI1	1.0306913768	0.9663663879	0.9999763851
MARVELD1	0	0	0
ZFYVE27	1.2431771806	0.2973992945	0.9999763851
SFRP5	0	0	0
GOLGA7B	-1.1865044235	0.5515338829	0.9999763851
R3HCC1L	-1.4340983205	0.1989575907	0.9999763851
LOXL4	-4.2849908816	0.4028453076	0.9999763851
PYROXD2	1.1987778498	0.3065170022	0.9999763851
HPS1	1.0784631874	0.6127871606	0.9999763851
HPSE2	-1.3713173847	0.7437037807	0.9999763851
GOT1	1.0989769021	0.6311506662	0.9999763851
SLC25A28	1.1085477988	0.7619249141	0.9999763851
ENTPD7	-3.7493386361	0.1114123703	0.9999763851
COX15	1.1765319427	0.3336806202	0.9999763851
CUTC	1.0393879572	0.8510021628	0.9999763851
ABCC2	-1.0298310449	0.92879698	0.9999763851
DNMBP	-1.5623913641	0.2016233652	0.9999763851
CPN1	0	0	0
ERLIN1	-1.1681908088	0.2593090935	0.9999763851
CHUK	-1.0667777682	0.7451813205	0.9999763851
CWF19L1	1.2480955274	0.3709117718	0.9999763851
BLOC1S2	-1.0568180935	0.7701392613	0.9999763851
SCD	-1.1723026395	0.2568613703	0.9999763851
WNT8B	-1.1491729646	0.8484018332	0.9999763851
SEC31B	1.1224441006	0.6935659246	0.9999763851
NDUFB8	1.1621214334	0.2355714474	0.9999763851
HIF1AN	-1.0552464916	0.8017134908	0.9999763851
PAX2	0	0	0
SLF2	1.1505430053	0.5419633901	0.9999763851
MRPL43	1.13191723	0.4075944082	0.9999763851
C10orf2	-1.1480002274	0.4779069042	0.9999763851
LZTS2	-1.1334715548	0.7102451389	0.9999763851

PDZD7	-1.4249413061	0.6087749825	0.9999763851
SFXN3	-1.2627502236	0.2829993355	0.9999763851
KAZALD1	1.5970252596	0.0243955597	0.9999763851
BTRC	1.0694951511	0.8231249914	0.9999763851
POLL	1.2917088661	0.4009072931	0.9999763851
DPCD	-1.104724475	0.7954280428	0.9999763851
FBXW4	-1.2236048847	0.3894734867	0.9999763851
NPM3	-1.3895268265	0.009395	0.9999763851
MGEA5	1.0687200677	0.6261721583	0.9999763851
KCNIP2	1.0894780223	0.9290979185	0.9999763851
C10orf76	1.0671472725	0.7944278412	0.9999763851
HPS6	-1.255871624	0.3129234296	0.9999763851
LDB1	1.001481805	0.9956830128	0.9999763851
PPRC1	1.8869872272	0.015668474	0.9999763851
NOLC1	1.3350947101	0.025876258	0.9999763851
ELOVL3	1.5208572074	0.1457369553	0.9999763851
PITX3	0	0	0
GBF1	1.1306696009	0.518959133	0.9999763851
NFKB2	2.4754293524	0.2005573491	0.9999763851
PSD	-1.3154339304	0.8205576571	0.9999763851
FBXL15	1.4691642448	0.2407783418	0.9999763851
CUEDC2	1.3040091908	0.2121442201	0.9999763851
C10orf95	0	0	0
MFSD13A	-1.1313021227	0.7099064585	0.9999763851
ACTR1A	1.1895251048	0.518586387	0.9999763851
SUFU	-1.1649728581	0.5337437037	0.9999763851
TRIM8	1.3286628829	0.2845881786	0.9999763851
ARL3	1.2431180238	0.2592383122	0.9999763851
SFXN2	1.3614039826	0.3289098684	0.9999763851
WBP1L	-1.1123632911	0.5316387304	0.9999763851
CYP17A1	-4.2849908816	0.4028453076	0.9999763851
BORCS7	-1.2806983362	0.2742832874	0.9999763851
AS3MT	0	0	0
CNNM2	-1.253349311	0.4446273867	0.9999763851
NT5C2	-1.1401409178	0.4060446003	0.9999763851
RPEL1	-19.2174470604	0.0691494629	0.9999763851
PCGF6	1.3237528899	0.3664647692	0.9999763851
TAF5	-1.0006454179	0.9979289417	0.9999763851
USMG5	1.2370923304	0.1556486924	0.9999763851
PDCD11	1.1976709788	0.2970417419	0.9999763851
CALHM2	1.6912538201	0.0567271979	0.9999763851
CALHM1	0	0	0
NEURL1	1.509580932	0.5403772662	0.9999763851
SH3PXD2A	-1.1200215877	0.5249262738	0.9999763851
OBFC1	1.7469303317	0.1046832561	0.9999763851
SLK	1.0270816425	0.8492695324	0.9999763851
COL17A1	-1.966276531	0.4562735265	0.9999763851
SFR1	-1.4410781675	0.3220354368	0.9999763851
CFAP43	9.9639367786	0.2203391531	0.9999763851

GSTO1	-1.1693669463	0.2583460039	0.9999763851
GSTO2	-1.3704303691	0.42335635	0.9999763851
ITPRIP	-1.0383711009	0.8425706535	0.9999763851
SORCS3	-3.5156187543	0.1696872133	0.9999763851
SORCS1	1.287203384	0.8316108013	0.9999763851
XPNPEP1	1.0374644315	0.81373403	0.9999763851
ADD3	1.1601255181	0.4265415229	0.9999763851
LOC105378479	5.4770877341	0.3746135179	0.9999763851
MXI1	-1.3145386162	0.1901122825	0.9999763851
SMNDC1	1.0137814032	0.9314654447	0.9999763851
DUSP5	-1.5736822895	0.228659524	0.9999763851
SMC3	1.1101083649	0.4461678897	0.9999763851
RBM20	0	0	0
PDCD4	-1.2032165598	0.2011368689	0.9999763851
BBIP1	1.909761605	0.0465382379	0.9999763851
SHOC2	-1.1939461008	0.3051331512	0.9999763851
GPAM	-1.0550268381	0.8628568019	0.9999763851
ACSL5	-2.7131089336	0.1511136884	0.9999763851
ZDHHC6	1.2925506417	0.1935362265	0.9999763851
VTI1A	-1.0459042202	0.8636298478	0.9999763851
TCF7L2	1.8003661677	0.0401618153	0.9999763851
NRAP	0	0	0
CASP7	-1.0084930073	0.9786837489	0.9999763851
DCLRE1A	2.1487114209	0.2253505247	0.9999763851
NHLRC2	-1.3134935995	0.4466365282	0.9999763851
ADRB1	1.1570040918	0.8865554867	0.9999763851
CCDC186	-1.0165392043	0.9692226474	0.9999763851
TDRD1	7.7488609396	0.3060017416	0.9999763851
AFAP1L2	3.2356557963	0.5547505991	0.9999763851
ABLIM1	-1.0833074967	0.7458504106	0.9999763851
FAM160B1	1.3575565792	0.2471779291	0.9999763851
TRUB1	-1.1552182069	0.4835673199	0.9999763851
HSPA12A	0	0	0
ENO4	0	0	0
SHTN1	0	0	0
SLC18A2	2.0598070501	0.0310544561	0.9999763851
PDZD8	-1.2935336416	0.1433020953	0.9999763851
EMX2	-4.2849908816	0.4028453076	0.9999763851
RAB11FIP2	-1.2315422062	0.5740405512	0.9999763851
FAM204A	1.0793839346	0.7421577046	0.9999763851
CACUL1	-1.0468966333	0.8115821677	0.9999763851
EIF3A	-1.0733947284	0.6561447284	0.9999763851
FAM45A	1.2982530607	0.2534492093	0.9999763851
SFXN4	1.2545927603	0.1662559672	0.9999763851
PRDX3	1.3579765755	0.0943848805	0.9999763851
GRK5	1.2449460941	0.5883260389	0.9999763851
RGS10	1.1545167366	0.398882808	0.9999763851
TIAL1	-1.2930018493	0.1179030625	0.9999763851
BAG3	-1.2339926131	0.5033839092	0.9999763851

INPP5F	-1.4578013109	0.259403341	0.9999763851
MCMBP	1.2160915711	0.311028016	0.9999763851
SEC23IP	1.1910306666	0.4409067385	0.9999763851
PLPP4	0	0	0
WDR11	1.3239127919	0.3440404769	0.9999763851
ATE1	1.1574661856	0.5386966345	0.9999763851
NSMCE4A	1.479035653	0.1176146611	0.9999763851
TACC2	3.2353357581	0.554787377	0.9999763851
BTBD16	0	0	0
PLEKHA1	-1.0368629227	0.9388126276	0.9999763851
HTRA1	-3.8382377092	0.005404	0.9999763851
C10orf120	0	0	0
FAM24B	-1.2915514768	0.5587208434	0.9999763851
C10orf88	2.2745276088	0.0824298643	0.9999763851
PSTK	-1.3948129396	0.6824769794	0.9999763851
IKZF5	-1.1804274504	0.5476954981	0.9999763851
ACADSB	-1.0381811629	0.8916542795	0.9999763851
BUB3	1.1945919005	0.190719034	0.9999763851
CHST15	0	0	0
OAT	1.2863960258	0.1887505941	0.9999763851
LHPP	1.1572257991	0.660649247	0.9999763851
LOC107984159	0	0	0
FAM53B	-1.2727850804	0.2472960051	0.9999763851
METTL10	-1.2182464339	0.3121793757	0.9999763851
FAM175B	1.0239585932	0.9326154759	0.9999763851
ZRANB1	1.0306998984	0.9118162733	0.9999763851
CTBP2	1.1688695279	0.420889897	0.9999763851
TEX36	0	0	0
EDRF1	-1.3291942657	0.445696718	0.9999763851
UROS	1.2691519355	0.2897264179	0.9999763851
BCCIP	1.1177244679	0.5261388951	0.9999763851
DHX32	-4.2864494036	0.4027150576	0.9999763851
FANK1	-4.2849908816	0.4028453076	0.9999763851
ADAM12	-7.5622178268	0.2177145568	0.9999763851
PTPRE	-1.0753601417	0.733139309	0.9999763851
MKI67	1.3150506741	0.0492587852	0.9999763851
MGMT	0	0	0
EBF3	1.1993473325	0.5393512812	0.9999763851
GLRX3	1.2315647751	0.2304673085	0.9999763851
PPP2R2D	-1.1300206787	0.5681161829	0.9999763851
BNIP3	1.285973213	0.2971185531	0.9999763851
STK32C	1.0591614067	0.7513998596	0.9999763851
LRRC27	1.4551263046	0.4624594753	0.9999763851
PWWP2B	1.0959404905	0.6828947506	0.9999763851
C10orf91	0	0	0
LOC107984282	5.4770877341	0.3746135179	0.9999763851
INPP5A	-1.4457183815	0.2844367719	0.9999763851
NKX6-2	-1.4503491785	0.0677222243	0.9999763851
VENTX	1.4561621084	0.4733776806	0.9999763851

ADAM8	1.0733608887	0.8573672073	0.9999763851
TUBGCP2	1.0597857097	0.7340972913	0.9999763851
ZNF511	-1.0235615146	0.9032418088	0.9999763851
PRAP1	0	0	0
FUOM	1.0509768657	0.8906527617	0.9999763851
ECHS1	-1.0401881261	0.7888610762	0.9999763851
MTG1	-1.1837340823	0.6066189755	0.9999763851
SPRN	2.5202262643	0.2772382374	0.9999763851
CYP2E1	9.9624059448	0.2314727708	0.9999763851
SCGB1C1	3.2353357581	0.554787377	0.9999763851
BET1L	1.2641209224	0.2186164612	0.9999763851
RIC8A	1.5811781315	0.008492	0.9999763851
SIRT3	1.0595963625	0.8474528594	0.9999763851
PSMD13	-1.1017898316	0.4780456203	0.9999763851
NLRP6	-2.3209115874	0.4617202599	0.9999763851
ATHL1	-1.1346497424	0.7413817045	0.9999763851
IFITM2	1.0888384964	0.5042228972	0.9999763851
IFITM1	1.3670163388	0.1280306777	0.9999763851
IFITM3	-1.3154339304	0.8205576571	0.9999763851
SIGIRR	1.3878502127	0.1838736393	0.9999763851
ANO9	0	0	0
PTDSS2	-1.0954654781	0.666017894	0.9999763851
RNH1	1.1651701115	0.3100347603	0.9999763851
LRRC56	5.4776292235	0.3745831165	0.9999763851
HRAS	1.523820577	0.1948253469	0.9999763851
LMNTD2	-1.4079687435	0.6448100812	0.9999763851
RASSF7	-1.1562405362	0.6261986804	0.9999763851
PHRF1	1.1315867871	0.4903034469	0.9999763851
IRF7	-1.1598159168	0.7754581207	0.9999763851
CDHR5	0	0	0
DRD4	0	0	0
DEAF1	-1.1271597983	0.4593684757	0.9999763851
TMEM80	2.0537095626	0.006206	0.9999763851
EPS8L2	3.2353357581	0.554787377	0.9999763851
TALDO1	1.2117818625	0.1235572561	0.9999763851
PDDC1	-1.0355604617	0.8662461958	0.9999763851
SLC25A22	-1.2682881137	0.5703197371	0.9999763851
PANO1	-1.0810230665	0.9183118938	0.9999763851
PIDD1	-1.6011851216	0.3896990657	0.9999763851
RPLP2	1.0079880607	0.948595219	0.9999763851
PNPLA2	1.3337636591	0.2142017887	0.9999763851
CRACR2B	1.8002526059	0.1132156194	0.9999763851
CD151	1.218879019	0.3600766079	0.9999763851
POLR2L	1.3006552709	0.07276114	0.9999763851
TSPAN4	1.7537362318	0.1570043559	0.9999763851
CHID1	-1.0080532677	0.9560065999	0.9999763851
AP2A2	-1.2432139538	0.3182778851	0.9999763851
MUC6	0	0	0
LOC101927503	0	0	0

MUC5B	0	0	0
TOLLIP	-1.2320387137	0.1987810928	0.9999763851
BRSK2	0	0	0
MOB2	1.1200052521	0.7152710928	0.9999763851
DUSP8	3.2361532718	0.5546934395	0.9999763851
IFITM10	1.0294704161	0.9669274527	0.9999763851
CTSD	-1.1666172837	0.2610024919	0.9999763851
TNNI2	-4.2864494036	0.4027150576	0.9999763851
LSP1	1.2294887479	0.2479387883	0.9999763851
TNNT3	0	0	0
MRPL23	1.325623298	0.1834944224	0.9999763851
HOTS	-7.5622178268	0.2177145568	0.9999763851
ASCL2	2.2137747159	0.0216549649	0.9999763851
C11orf21	1.2870761236	0.8160709833	0.9999763851
TSPAN32	-1.1002038147	0.7584032646	0.9999763851
TSSC4	1.2046996235	0.484118437	0.9999763851
TRPM5	0	0	0
KCNQ1	-1.0412222277	0.8367513609	0.9999763851
CDKN1C	1.050070356	0.9028401825	0.9999763851
SLC22A18AS	-1.2562050205	0.7085481797	0.9999763851
SLC22A18	-1.3861836028	0.3325300008	0.9999763851
PHLDA2	1.0681016286	0.7613114134	0.9999763851
NAP1L4	1.1295773766	0.3820027785	0.9999763851
CARS	1.1568626616	0.4015394939	0.9999763851
OSBPL5	-1.4548833948	0.0868266071	0.9999763851
ZNF195	-1.1270820338	0.6497152799	0.9999763851
LOC105376525	-1.3158816768	0.8203229797	0.9999763851
ART1	-1.3157515262	0.8223973021	0.9999763851
CHRNA10	1.0281638254	0.9765707018	0.9999763851
NUP98	-1.0386768445	0.8494487214	0.9999763851
PGAP2	1.0874064405	0.7723709221	0.9999763851
RHOG	1.2231846012	0.1578576604	0.9999763851
STIM1	-1.2073858184	0.2732160055	0.9999763851
RRM1	-1.0236619168	0.8493632702	0.9999763851
TRIM21	-1.3876816948	0.360685681	0.9999763851
TRIM68	-2.3212684229	0.447354629	0.9999763851
TRIM5	1.1209121282	0.7603390199	0.9999763851
TRIM34	1.3407820359	0.7146293923	0.9999763851
TRIM22	-3.0368738636	0.1019843322	0.9999763851
C11orf42	-10.8385246611	0.1453473185	0.9999763851
FAM160A2	1.5170758868	0.0986661746	0.9999763851
CNGA4	-2.6604789227	0.1932206488	0.9999763851
SMPD1	1.0175127479	0.9363584697	0.9999763851
APBB1	-4.2864494036	0.4027150576	0.9999763851
HPX	0	0	0
TRIM3	1.6938437942	0.6482542272	0.9999763851
ARFIP2	1.1284664006	0.7747265309	0.9999763851
TIMM10B	-1.0395982519	0.8570873922	0.9999763851
DNHD1	1.2936707046	0.5572557448	0.9999763851

RRP8	1.1956970232	0.4908605823	0.9999763851
ILK	1.4064341007	0.0592223874	0.9999763851
TAF10	-1.2249366693	0.5354806869	0.9999763851
TPP1	-1.023417204	0.8791117141	0.9999763851
DCHS1	-1.3158393667	0.8280718157	0.9999763851
MRPL17	1.1287492713	0.4468674425	0.9999763851
NLRP14	-4.2849908816	0.4028453076	0.9999763851
SYT9	0	0	0
OLFML1	1.2866381786	0.8225176192	0.9999763851
PPFIBP2	-1.3334061201	0.3468883623	0.9999763851
CYB5R2	0	0	0
EIF3F	1.0359637597	0.8141368002	0.9999763851
TUB	0	0	0
RIC3	3.2353357581	0.554787377	0.9999763851
LMO1	1.0198511656	0.9456704056	0.9999763851
STK33	0	0	0
TRIM66	-1.5293856892	0.1499616007	0.9999763851
RPL27A	-1.0560971285	0.684715362	0.9999763851
ST5	5.4784711963	0.3745358536	0.9999763851
AKIP1	1.02817935	0.9075040951	0.9999763851
C11orf16	-4.2864494036	0.4027150576	0.9999763851
TMEM9B	-1.2843448044	0.2487869682	0.9999763851
NRIP3	-2.0988867482	0.0120910656	0.9999763851
SCUBE2	-2.3209115874	0.4613944243	0.9999763851
DENND5A	-1.6998322356	0.0275945356	0.9999763851
TMEM41B	-1.2097148337	0.3817491183	0.9999763851
IPO7	1.0585444115	0.7080832625	0.9999763851
ZNF143	-1.4874685974	0.1565095185	0.9999763851
WEE1	1.1564633722	0.5194280829	0.9999763851
SWAP70	-1.4446435678	0.110727556	0.9999763851
SBF2	-1.6412232356	0.0831806815	0.9999763851
ADM	-24.1442657935	1.19E-05	0.027763203
AMPD3	-1.1094127078	0.647280633	0.9999763851
MTRNR2L8	-1.4210747916	0.0268134487	0.9999763851
RNF141	-1.1136249807	0.5842196965	0.9999763851
MRVI1	-1.3263815267	0.4218136692	0.9999763851
CTR9	1.1295338724	0.4836051763	0.9999763851
EIF4G2	1.1004750933	0.5145828946	0.9999763851
ZBED5	-1.0075228815	0.9812611408	0.9999763851
CSNK2A3	1.1552768729	0.7987874843	0.9999763851
USP47	-1.1825623229	0.362358175	0.9999763851
MICAL2	-1.5874464891	0.147499296	0.9999763851
MICALCL	-5.6080522719	0.1117474342	0.9999763851
TEAD1	0	0	0
ARNTL	-1.3906985905	0.3840620169	0.9999763851
BTBD10	-1.2123166596	0.54104405	0.9999763851
PTH	-1.0281765664	0.8886340452	0.9999763851
FAR1	1.170973175	0.5371579558	0.9999763851
SPON1	0	0	0

COPB1	1.0387497685	0.8209013173	0.9999763851
PSMA1	-1.0283233684	0.8700105943	0.9999763851
PDE3B	-1.6544378424	0.2576446952	0.9999763851
CYP2R1	1.0419785874	0.8833410107	0.9999763851
LOC107984314	3.2361532718	0.5546934395	0.9999763851
INSC	0	0	0
SOX6	-4.2849908816	0.4028453076	0.9999763851
C11orf58	-1.1096952991	0.4953584327	0.9999763851
PLEKHA7	-1.1559070418	0.2374189885	0.9999763851
RPS13	1.1176245987	0.3727566394	0.9999763851
PIK3C2A	-1.158687391	0.5810024371	0.9999763851
NUCB2	-1.0088886763	0.9470185718	0.9999763851
NCR3LG1	-2.774230463	0.0784695365	0.9999763851
KCNJ11	1.5490136109	0.3237788062	0.9999763851
KCNC1	-1.3160416743	0.8272147023	0.9999763851
SERGEF	-1.390733637	0.2005380556	0.9999763851
SAAL1	1.3108294194	0.1804650067	0.9999763851
MRGPRX3	0	0	0
SAA4	0	0	0
HPS5	-1.0487490121	0.915093015	0.9999763851
GTF2H1	-1.1353098397	0.5200919978	0.9999763851
LDHA	1.5713581681	0.0001381	0.1956409862
LDHAL6A	1.7868451227	0.5989310412	0.9999763851
TSG101	1.8143602283	0.0568853602	0.9999763851
UEVLD	1.2424325408	0.4407166508	0.9999763851
SPTY2D1	1.2028307756	0.4742560896	0.9999763851
TMEM86A	0	0	0
IGSF22	3.2353357581	0.554787377	0.9999763851
ZDHHC13	-2.2647966062	0.1228626797	0.9999763851
E2F8	1.2261666389	0.4776066832	0.9999763851
NAV2	-1.263684942	0.2803075134	0.9999763851
HTATIP2	1.5387792626	0.0139263845	0.9999763851
PRMT3	-1.000165457	0.999582439	0.9999763851
NELL1	-1.4157843229	0.612753461	0.9999763851
FANCF	-1.9412091951	0.1494134001	0.9999763851
LUZP2	3.2353357581	0.554787377	0.9999763851
LGR4	-4.2849908816	0.4028453076	0.9999763851
LIN7C	1.2200237807	0.4585956717	0.9999763851
BDNF	0	0	0
KIF18A	-1.2210442353	0.5418002616	0.9999763851
METTL15	1.1983325423	0.3851182292	0.9999763851
ARL14EP	-1.1461350495	0.5320824206	0.9999763851
DCDC5	0	0	0
DCDC1	-4.2849908816	0.4028453076	0.9999763851
DNAJC24	-1.5190729878	0.4050723199	0.9999763851
IMMP1L	1.0251276472	0.9037885592	0.9999763851
ELP4	-1.2796723791	0.416406905	0.9999763851
PAX6	2.382974333	0.2355539459	0.9999763851
RCN1	1.488474938	0.1777323214	0.9999763851

WT1	1.0170204453	0.9465335782	0.9999763851
EIF3M	1.2040348813	0.2283869647	0.9999763851
CCDC73	1.4187907312	0.4508305076	0.9999763851
PRRG4	1.1779207	0.686760044	0.9999763851
QSER1	1.3643514649	0.1703496707	0.9999763851
TCP11L1	-1.0150102882	0.9725549011	0.9999763851
CSTF3	-1.3996472136	0.0767666209	0.9999763851
HIPK3	-1.0283086345	0.8875270884	0.9999763851
FBXO3	1.0576664542	0.8561291336	0.9999763851
LMO2	1.1819567914	0.4101371485	0.9999763851
CAPRIN1	1.0452498843	0.7899218348	0.9999763851
NAT10	1.0520468466	0.7430810511	0.9999763851
ABTB2	1.0304020284	0.9504726675	0.9999763851
CAT	1.1484747071	0.3628374604	0.9999763851
APIP	-1.2470971516	0.453648454	0.9999763851
PDHX	1.482476071	0.1893539301	0.9999763851
CD44	-1.1770998506	0.2418331237	0.9999763851
SLC1A2	0	0	0
FJX1	-2.5604817607	0.2731422845	0.9999763851
TRIM44	-1.0225749403	0.8893578303	0.9999763851
LDLRAD3	1.040469927	0.8630793116	0.9999763851
COMMD9	-1.27223215	0.3787309123	0.9999763851
PRR5L	1.8525489671	0.1467431427	0.9999763851
TRAF6	1.1859266318	0.7056408559	0.9999763851
C11orf74	1.6338700832	0.2739925035	0.9999763851
API5	1.1176637559	0.5592458002	0.9999763851
TTC17	-1.0343656598	0.8641413251	0.9999763851
HSD17B12	2.4405833224	0.0449227536	0.9999763851
ALKBH3	-1.0569601447	0.761313524	0.9999763851
ACCSL	-1.3151016298	0.8211947154	0.9999763851
C11orf96	-1.0447361553	0.9067119433	0.9999763851
EXT2	-1.3204355981	0.1823691007	0.9999763851
ALX4	-3.3275701064	0.2296160779	0.9999763851
CD82	-2.0078462284	0.0001175	0.1806818129
TSPAN18	1.316696459	0.2777835441	0.9999763851
TP53I11	1.0324850193	0.8408422484	0.9999763851
LOC101060179	-1.3715126673	0.7506909141	0.9999763851
CHST1	1.8139617088	0.5784350384	0.9999763851
SLC35C1	-1.0904200057	0.6873227931	0.9999763851
CRY2	-1.2702381915	0.4208823877	0.9999763851
MAPK8IP1	-3.3280817062	0.2431049726	0.9999763851
PEX16	1.9471619863	0.2454515101	0.9999763851
PHF21A	-1.5313052123	0.1246518302	0.9999763851
CREB3L1	3.2361532718	0.5546934395	0.9999763851
DGKZ	1.2486886879	0.1986642592	0.9999763851
MDK	1.0773444223	0.8936517727	0.9999763851
AMBRA1	-1.018873575	0.8726534988	0.9999763851
HARBI1	3.3654696961	0.1580529345	0.9999763851
ATG13	1.392797841	0.1804657778	0.9999763851

ARHGAP1	1.162729018	0.4140895538	0.9999763851
ZNF408	1.3349646538	0.3931084199	0.9999763851
F2	3.2353357581	0.554787377	0.9999763851
CKAP5	1.3288398066	0.1193279371	0.9999763851
LRP4	-7.5647903763	0.2176246281	0.9999763851
C11orf49	1.0989324126	0.8396180905	0.9999763851
ARFGAP2	1.2803920328	0.208225975	0.9999763851
PACSIN3	-1.0497023957	0.8402180243	0.9999763851
DDB2	1.1591585179	0.5222403341	0.9999763851
ACP2	1.0885190141	0.8438358517	0.9999763851
NR1H3	-2.2152323827	0.2441191925	0.9999763851
MADD	-1.1812179649	0.5193928253	0.9999763851
MYBPC3	-1.816422855	0.4359989028	0.9999763851
SPI1	1.1622116163	0.5667893654	0.9999763851
SLC39A13	1.1637306491	0.6418948832	0.9999763851
PSMC3	1.0446675582	0.82361331	0.9999763851
RAPSN	0	0	0
CELF1	-1.0489536745	0.7996311246	0.9999763851
PTPMT1	1.119984549	0.5056619521	0.9999763851
KBTBD4	1.3745641786	0.7399429411	0.9999763851
NDUFS3	-1.4833912503	0.0104770122	0.9999763851
FAM180B	-3.3269505881	0.2594254525	0.9999763851
C1QTNF4	0	0	0
MTCH2	-1.3530215083	0.0180224222	0.9999763851
AGBL2	-2.3209115874	0.4610635141	0.9999763851
FNBP4	-1.336060578	0.1220682127	0.9999763851
NUP160	1.3271788544	0.2561392257	0.9999763851
PTPRJ	-1.5591949695	0.1271272522	0.9999763851
TNKS1BP1	2.0526865509	0.1738510117	0.9999763851
SSRP1	-1.0274583285	0.8439148387	0.9999763851
P2RX3	0	0	0
PRG2	-2.0609782294	0.3008190586	0.9999763851
SLC43A3	-1.004859436	0.9706806319	0.9999763851
RTN4RL2	-1.3124628314	0.6128228316	0.9999763851
SLC43A1	-1.0501628322	0.7664175936	0.9999763851
TIMM10	1.2403251906	0.2278733561	0.9999763851
UBE2L6	-1.1707722522	0.4530285376	0.9999763851
CLP1	-1.3082371081	0.4746835972	0.9999763851
ZDHHC5	-1.0716252208	0.6507863493	0.9999763851
MED19	1.0476662048	0.8517039531	0.9999763851
TMX2	-1.2002581092	0.2444365504	0.9999763851
C11orf31	1.1732042399	0.2213765479	0.9999763851
BTBD18	9.9629524019	0.2192284853	0.9999763851
CTNND1	-1.1802901363	0.7540192034	0.9999763851
OR9Q1	0	0	0
LPXN	1.0118617639	0.9387642623	0.9999763851
ZFP91	1.0478979516	0.7670009228	0.9999763851
GLYATL1	0	0	0
FAM111A	1.243090902	0.1632047737	0.9999763851

DTX4	-2.0660393506	0.0553880684	0.9999763851
MPEG1	0	0	0
OSBP	1.1302014727	0.666022616	0.9999763851
PATL1	-1.3238132256	0.1007266607	0.9999763851
STX3	1.099030712	0.5184429641	0.9999763851
MRPL16	1.10502152	0.5189140645	0.9999763851
GIF	-4.2864494036	0.4027150576	0.9999763851
TCN1	-1.3154339304	0.8205576571	0.9999763851
MS4A3	-1.2231442609	0.5024608206	0.9999763851
MS4A2	-4.287394647	0.4026306792	0.9999763851
MS4A6A	1.349505569	0.6824616559	0.9999763851
MS4A4E	0	0	0
MS4A4A	-4.2849908816	0.4028453076	0.9999763851
MS4A7	-1.0193796434	0.9441413143	0.9999763851
MS4A5	0	0	0
CCDC86	1.0144334144	0.9675855466	0.9999763851
PTGDR2	1.0797026578	0.8772102504	0.9999763851
ZP1	0	0	0
PRPF19	1.2999227171	0.087974938	0.9999763851
TMEM109	-1.1626081622	0.3378465537	0.9999763851
TMEM132A	-1.3154339304	0.8205576571	0.9999763851
SLC15A3	-1.3158816768	0.8203229797	0.9999763851
CD6	-5.3422739143	0.0885609822	0.9999763851
CD5	1.287203384	0.8318830578	0.9999763851
VPS37C	1.1042445014	0.6201542937	0.9999763851
VWCE	1.1331614061	0.8702271379	0.9999763851
DDB1	-1.3087696554	0.02950501	0.9999763851
TKFC	2.1868632022	0.0242118047	0.9999763851
CYB561A3	1.2228637109	0.2854554761	0.9999763851
TMEM138	1.06677647	0.8350843861	0.9999763851
TMEM216	-1.1195868461	0.7725248488	0.9999763851
CPSF7	-1.290889535	0.1979234541	0.9999763851
SDHAF2	1.2966040974	0.1979898362	0.9999763851
PPP1R32	-3.3272796812	0.2440049033	0.9999763851
DAGLA	1.8135020099	0.5902512871	0.9999763851
MYRF	1.1817745558	0.6268599799	0.9999763851
TMEM258	1.1209639972	0.4565439604	0.9999763851
FEN1	-1.1625104956	0.2635633954	0.9999763851
FADS1	1.118350171	0.4943443193	0.9999763851
FADS2	-1.050270132	0.7757088393	0.9999763851
FADS3	-2.6618053695	0.0503244689	0.9999763851
RAB3IL1	-1.1277510391	0.8131505558	0.9999763851
BEST1	5.4784711963	0.3745358536	0.9999763851
FTH1	-2.279345415	4.43E-07	0.001254
INCENP	-1.0131210356	0.9450268714	0.9999763851
SCGB2A1	0	0	0
ASRGL1	1.2645294143	0.2440199209	0.9999763851
AHNAK	-1.5090075488	0.00967	0.9999763851
EEF1G	1.1141529289	0.372690679	0.9999763851

TUT1	-1.1819730229	0.6274702415	0.9999763851
MTA2	1.2709066462	0.1972937268	0.9999763851
EML3	1.6969887163	0.1401414116	0.9999763851
ROM1	1.0953517363	0.8964715001	0.9999763851
B3GAT3	-1.2174601429	0.4496248384	0.9999763851
GANAB	-1.2572940375	0.1532480796	0.9999763851
INTS5	1.2400381557	0.508332947	0.9999763851
C11orf98	-1.0407593017	0.8570174429	0.9999763851
LBHD1	12.2062282412	0.1876867146	0.9999763851
METTL12	-1.2208277452	0.6759019168	0.9999763851
UQCC3	1.1313479325	0.4995760156	0.9999763851
UBXN1	1.0239201175	0.8871859951	0.9999763851
LRRN4CL	0	0	0
BSCL2	-1.0882385856	0.8122003297	0.9999763851
HNRNPUL2	1.1113384765	0.5575867619	0.9999763851
TTC9C	1.835448846	0.1134222441	0.9999763851
ZBTB3	-1.8177798391	0.4637711422	0.9999763851
POLR2G	-1.0872916886	0.606231998	0.9999763851
TAF6L	-1.406657056	0.6693786986	0.9999763851
TMEM179B	1.1108116713	0.5654030997	0.9999763851
TMEM223	1.6134707631	0.0775449336	0.9999763851
NXF1	1.2095247063	0.433484436	0.9999763851
STX5	1.6229824428	0.3231204292	0.9999763851
WDR74	-1.5232345967	0.0411637389	0.9999763851
SLC3A2	1.1685887597	0.3426477096	0.9999763851
HRASLS5	0	0	0
LGALS12	1.483694972	0.0202108271	0.9999763851
RARRES3	12.2012745411	0.188290858	0.9999763851
ATL3	1.0492488921	0.7789970683	0.9999763851
RTN3	-1.1139640448	0.4446882499	0.9999763851
C11orf95	-1.3026553232	0.3179510519	0.9999763851
C11orf84	1.1501624171	0.6310977453	0.9999763851
MARK2	1.6196175664	0.1049143762	0.9999763851
RCOR2	-7.5664585246	0.2260541296	0.9999763851
NAA40	2.0135785869	0.1691796767	0.9999763851
COX8A	1.1195958257	0.3829647666	0.9999763851
OTUB1	-1.0720661067	0.7191594043	0.9999763851
MACROD1	1.0819762035	0.7414101895	0.9999763851
FLRT1	2.5294022043	0.2955322964	0.9999763851
STIP1	1.2411612936	0.1501942945	0.9999763851
FERMT3	-1.1324365637	0.3646140369	0.9999763851
TRPT1	-1.5856150365	0.4018879357	0.9999763851
NUDT22	1.3214281743	0.4025691961	0.9999763851
DNAJC4	-1.346752514	0.5056951645	0.9999763851
VEGFB	-2.5602582481	0.2739893701	0.9999763851
FKBP2	1.1520967501	0.5149599608	0.9999763851
PPP1R14B	-1.031208036	0.8102260922	0.9999763851
PLCB3	1.1352532707	0.5624501511	0.9999763851
BAD	1.0561382742	0.88337991	0.9999763851

GPR137	1.030012456	0.966792439	0.9999763851
KCNK4	3.2353357581	0.554787377	0.9999763851
TEX40	3.2353357581	0.554787377	0.9999763851
ESRRA	1.1241949748	0.7983541789	0.9999763851
TRMT112	-1.3172769959	0.0228606395	0.9999763851
PRDX5	-1.0746649281	0.7870951223	0.9999763851
CCDC88B	-1.201883171	0.5527224229	0.9999763851
RPS6KA4	1.7619379809	0.0554205974	0.9999763851
RASGRP2	-1.0638637113	0.7350710645	0.9999763851
PYGM	1.6242600155	0.561953739	0.9999763851
SF1	1.1571592486	0.2834119323	0.9999763851
MAP4K2	-1.7212327308	0.0959479782	0.9999763851
MEN1	1.0045798836	0.982123976	0.9999763851
EHD1	-1.0071407265	0.9625461786	0.9999763851
ATG2A	1.1026640234	0.6659339055	0.9999763851
PPP2R5B	-1.2565073761	0.7040724342	0.9999763851
MAJIN	3.2356557963	0.5547505991	0.9999763851
BATF2	-1.9661492502	0.4737530821	0.9999763851
ARL2	-1.1296263162	0.5981508201	0.9999763851
SNX15	-1.3449153029	0.3350817122	0.9999763851
SAC3D1	1.0285804126	0.8703648878	0.9999763851
NAALADL1	0	0	0
CDCA5	1.717724697	0.002834	0.9999763851
ZFPL1	-2.2442957359	0.063759107	0.9999763851
TMEM262	0	0	0
VPS51	-1.675207249	0.0533977071	0.9999763851
TM7SF2	-1.7376064016	0.2806654257	0.9999763851
ZNHIT2	-1.4244255272	0.5877023505	0.9999763851
FAU	-1.2402466359	0.0909018905	0.9999763851
MRPL49	1.120445842	0.6790432479	0.9999763851
SYVN1	1.5158388998	0.0791004365	0.9999763851
CAPN1	1.176352579	0.3381612643	0.9999763851
SLC22A20	1.9220906279	0.4463275739	0.9999763851
POLA2	-1.0309871549	0.8768981943	0.9999763851
CDC42EP2	-2.5595294703	0.3316288706	0.9999763851
DPF2	1.4482677287	0.0455477238	0.9999763851
TIGD3	-1.6460380751	0.4436071582	0.9999763851
SLC25A45	1.5505881706	0.5536185115	0.9999763851
FRMD8	-1.2770177567	0.1547288856	0.9999763851
LOC101927789	-1.1464451114	0.8080570339	0.9999763851
SCYL1	-2.1914873857	0.006676	0.9999763851
LTBP3	2.6108942912	0.0423947525	0.9999763851
SSSCA1	1.1681740725	0.5956630871	0.9999763851
FAM89B	1.0122830567	0.9662129723	0.9999763851
EHBP1L1	1.0867556325	0.8528223935	0.9999763851
MAP3K11	-1.0384701228	0.8872745145	0.9999763851
PCNX3	1.5783118254	0.4063594651	0.9999763851
SIPA1	2.914697366	0.0755996307	0.9999763851
RELA	-1.0412708593	0.801957789	0.9999763851

KAT5	1.7574593324	0.4419655219	0.9999763851
RNASEH2C	1.216537129	0.2221093648	0.9999763851
AP5B1	1.1005314116	0.6056491187	0.9999763851
OVOL1	0	0	0
SNX32	0	0	0
CFL1	-1.0284267113	0.8112191676	0.9999763851
MUS81	1.1773342207	0.5498543999	0.9999763851
EFEMP2	-2.3295566515	0.3229213845	0.9999763851
CTSW	0	0	0
FIBP	1.1343712798	0.5651212583	0.9999763851
CCDC85B	1.0009834747	0.9982609096	0.9999763851
FOSL1	-1.1159645743	0.7692835156	0.9999763851
C11orf68	1.6555746083	0.2286995038	0.9999763851
DRAP1	1.0174493166	0.9478094132	0.9999763851
TSGA10IP	5.4770877341	0.3746135179	0.9999763851
SART1	-1.5541105705	0.3569856994	0.9999763851
EIF1AD	1.0858778812	0.7209433125	0.9999763851
BANF1	1.1104025442	0.5366630071	0.9999763851
CATSPER1	0	0	0
SF3B2	1.0653078201	0.6559165097	0.9999763851
PACS1	1.3261442362	0.7479785331	0.9999763851
KLC2	1.5428214719	0.539053522	0.9999763851
RAB1B	1.0615112096	0.7188503301	0.9999763851
CNIH2	1.0204530064	0.983670156	0.9999763851
YIF1A	1.0384514402	0.8624629334	0.9999763851
TMEM151A	5.4781708352	0.3745527127	0.9999763851
RIN1	1.2721844354	0.4077144764	0.9999763851
BRMS1	1.1741240448	0.4930003948	0.9999763851
B4GAT1	-1.3763682718	0.4338678011	0.9999763851
SLC29A2	-1.3154339304	0.8205576571	0.9999763851
NPAS4	1.2872739558	0.8231267639	0.9999763851
MRPL11	-1.0124176423	0.9390797451	0.9999763851
PELI3	-1.4443368534	0.3582238424	0.9999763851
DPP3	1.4919907328	0.0769017581	0.9999763851
BBS1	1.7450428903	0.632553386	0.9999763851
ZDHHC24	-1.0314839288	0.9137022144	0.9999763851
CCDC87	0	0	0
CCS	1.198960756	0.6444355609	0.9999763851
RBM14	1.3396031948	0.171748147	0.9999763851
RBM14-RBM4	-3.5443870889	0.0127432346	0.9999763851
RBM4	-1.2445372745	0.1212344514	0.9999763851
RBM4B	-1.0214433455	0.9141390128	0.9999763851
SPTBN2	1.8137824633	0.5636614767	0.9999763851
C11orf80	1.135845862	0.819803762	0.9999763851
RCE1	1.5501971516	0.5508474977	0.9999763851
PC	1.7451114492	0.3459884553	0.9999763851
LRFN4	-1.3063735095	0.1822042325	0.9999763851
SYT12	-2.5519849925	0.3672751297	0.9999763851
LOC107984341	2.8674942058	0.3051338712	0.9999763851

KDM2A	-1.1057036686	0.5706638956	0.9999763851
GRK2	1.3466320652	0.0410543501	0.9999763851
ANKRD13D	-1.0654807703	0.7887927614	0.9999763851
SSH3	1.5488017649	0.5478215345	0.9999763851
POLD4	-1.3022440858	0.3748337604	0.9999763851
CLCF1	-2.3210912367	0.1860179279	0.9999763851
RAD9A	1.2522196733	0.546983369	0.9999763851
PPP1CA	-1.0426140781	0.7923625911	0.9999763851
TBC1D10C	1.6485236883	0.0987545195	0.9999763851
CARNS1	0	0	0
RPS6KB2	-1.245622969	0.1619822784	0.9999763851
PTPRCAP	-4.2849908816	0.4028453076	0.9999763851
CORO1B	1.3413703944	0.7016505406	0.9999763851
CABP4	3.2361532718	0.5546934395	0.9999763851
TMEM134	-1.2356356493	0.4507195652	0.9999763851
AIP	1.1887057207	0.4954655157	0.9999763851
PITPNM1	3.1090798703	0.1420690117	0.9999763851
CDK2AP2	-1.0206858404	0.9155979113	0.9999763851
GSTP1	1.0443950044	0.7642498787	0.9999763851
NDUFV1	-1.0936144335	0.625458558	0.9999763851
NUDT8	1.2120602209	0.4614977122	0.9999763851
TBX10	0	0	0
UNC93B1	1.315783129	0.2568295293	0.9999763851
ALDH3B1	1.536652845	0.082465148	0.9999763851
NDUFS8	1.508349381	0.0392652091	0.9999763851
TCIRG1	-1.7431796887	0.0003921	0.4396020528
CHKA	1.0558715428	0.7908767651	0.9999763851
KMT5B	1.2729661331	0.3206196012	0.9999763851
C11orf24	1.1585371987	0.5211890806	0.9999763851
LRP5	-1.1926402477	0.6140596541	0.9999763851
PPP6R3	-1.1282258727	0.4296692974	0.9999763851
GAL	-1.4080455631	0.236041005	0.9999763851
TESMIN	1.1238503373	0.707273313	0.9999763851
CPT1A	-1.1784113049	0.2680978126	0.9999763851
MRPL21	1.111918143	0.6006196884	0.9999763851
IGHMBP2	-1.0450733255	0.9283650227	0.9999763851
TPCN2	1.1257162644	0.6175410505	0.9999763851
MYEOV	1.9925962985	0.4276660305	0.9999763851
CCND1	-1.4210949265	0.5923516988	0.9999763851
ORAOV1	-1.1373337057	0.490458352	0.9999763851
ANO1	-4.2864494036	0.4027150576	0.9999763851
FADD	1.2007616417	0.2626466184	0.9999763851
PPFIA1	1.0858056468	0.685984558	0.9999763851
CTTN	7.722359209	0.2874446962	0.9999763851
SHANK2	1.0277462901	0.9757572405	0.9999763851
DHCR7	1.2881789078	0.129147185	0.9999763851
NADSYN1	-1.0448563285	0.8323539553	0.9999763851
KRTAP5-10	-4.2849908816	0.4028453076	0.9999763851
FAM86C1	1.2442206333	0.643528236	0.9999763851

ZNF705E	-4.2864494036	0.4027150576	0.9999763851
LOC100129216	5.4784711963	0.3745358536	0.9999763851
RNF121	1.7953519338	0.0648212335	0.9999763851
IL18BP	-4.9394901244	0.0454676904	0.9999763851
NUMA1	-1.0437112545	0.8196585342	0.9999763851
LRTOMT	-2.4191812103	0.1185371269	0.9999763851
LAMTOR1	-1.036557434	0.8180444347	0.9999763851
ANAPC15	-1.0001841089	0.9994775231	0.9999763851
FOLR3	-4.2849908816	0.4028453076	0.9999763851
FOLR2	0	0	0
INPPL1	1.2014172385	0.3201764713	0.9999763851
PHOX2A	0	0	0
CLPB	-1.0735973567	0.8678854197	0.9999763851
PDE2A	2.5098011754	0.4395977304	0.9999763851
ARAP1	-1.024471907	0.8826217268	0.9999763851
STARD10	-3.7476955756	0.1101822092	0.9999763851
ATG16L2	-1.0711396247	0.6604686682	0.9999763851
FCHSD2	-1.0989347282	0.706004538	0.9999763851
P2RY2	1.2889734854	0.320717372	0.9999763851
P2RY6	1.0147626614	0.9568770254	0.9999763851
ARHGEF17	1.1570600642	0.6838099751	0.9999763851
RELT	-1.6129557959	0.0863037936	0.9999763851
FAM168A	1.1804954333	0.6621406232	0.9999763851
PLEKHB1	0	0	0
RAB6A	-1.2536742049	0.1858333075	0.9999763851
MRPL48	1.3353298256	0.1864873624	0.9999763851
COA4	1.3436378606	0.0794314062	0.9999763851
PAAF1	1.724050441	0.0790463794	0.9999763851
DNAJB13	-1.3161718537	0.8286049067	0.9999763851
UCP2	-1.1067838923	0.7190849319	0.9999763851
UCP3	1.027644744	0.9776658337	0.9999763851
C2CD3	-1.1270203652	0.7805359844	0.9999763851
PPME1	-1.1992385941	0.5189184882	0.9999763851
PGM2L1	-1.0597661417	0.8798597939	0.9999763851
KCNE3	-1.5761220861	0.0698976886	0.9999763851
LIPT2	1.2363030365	0.7851059401	0.9999763851
LOC100287896	1.461772119	0.5972841681	0.9999763851
POLD3	-1.3631566548	0.1466381893	0.9999763851
CHRDL2	0	0	0
RNF169	-1.3952982372	0.1621821523	0.9999763851
XRRA1	1.6676353015	0.092474176	0.9999763851
SPCS2	-1.1645322612	0.2955976028	0.9999763851
NEU3	-3.5693441777	0.0997686245	0.9999763851
SLCO2B1	-4.2849908816	0.4028453076	0.9999763851
ARRB1	1.0185070741	0.8936942159	0.9999763851
RPS3	1.0111740709	0.9297611455	0.9999763851
KLHL35	1.4836611752	0.510002522	0.9999763851
GDPD5	1.1155755303	0.7498090188	0.9999763851
SERPINH1	-3.1563891683	0.1985369756	0.9999763851

DGAT2	1.0993384034	0.750459544	0.9999763851
UVRAG	1.1154461669	0.5791322435	0.9999763851
THAP12	-1.2359220512	0.2861987592	0.9999763851
LOC100506127	1.7574012207	0.4563628961	0.9999763851
EMSY	1.0047624586	0.9882887708	0.9999763851
LRRC32	0	0	0
TSKU	1.293138255	0.5080209778	0.9999763851
ACER3	1.1676214644	0.5806179135	0.9999763851
CAPN5	-1.1959066992	0.7977185273	0.9999763851
MYO7A	-1.4889425408	0.3691873014	0.9999763851
PAK1	-1.1811552572	0.3813519639	0.9999763851
LOC107987163	-1.7842319031	0.2668247326	0.9999763851
AQP11	1.922830455	0.4619641046	0.9999763851
CLNS1A	-1.1857376123	0.26743614	0.9999763851
RSF1	-1.0702066481	0.7036354525	0.9999763851
AAMDC	1.1774367818	0.5510614692	0.9999763851
INTS4	1.2897962377	0.6135164093	0.9999763851
NDUFC2-KCTD14	-1.1494500921	0.8539263637	0.9999763851
NDUFC2	1.5004646008	0.004831	0.9999763851
ALG8	1.5427277387	0.0174339231	0.9999763851
KCTD21	1.2792966707	0.6250374225	0.9999763851
USP35	-1.9143611748	0.003703	0.9999763851
GAB2	1.0321351827	0.9341974879	0.9999763851
NARS2	1.2978009956	0.4811750543	0.9999763851
PRCP	-1.0761678674	0.6082538354	0.9999763851
DDIAS	1.8132200733	0.0498565486	0.9999763851
RAB30	-1.0097592345	0.9913143167	0.9999763851
PCF11	-1.0581617769	0.7446219406	0.9999763851
ANKRD42	1.1766460192	0.7119344815	0.9999763851
CCDC90B	-1.2840642084	0.2795022152	0.9999763851
TMEM126B	1.3174874926	0.1687338702	0.9999763851
TMEM126A	1.2989286428	0.2398859338	0.9999763851
CREBZF	-1.0001158534	0.9994640303	0.9999763851
CCDC83	-4.2864494036	0.4027150576	0.9999763851
PICALM	1.0005461492	0.9970919231	0.9999763851
EED	-1.4436888609	0.0417242824	0.9999763851
C11orf73	1.3506954796	0.3006647502	0.9999763851
CCDC81	-4.2849908816	0.4028453076	0.9999763851
ME3	1.2490665649	0.6891501225	0.9999763851
FZD4	-3.3294360969	0.2401993702	0.9999763851
TMEM135	1.1557101781	0.6821822268	0.9999763851
RAB38	-2.5616934468	0.2767599224	0.9999763851
CTSC	1.0529294202	0.7863925464	0.9999763851
GRM5	0	0	0
NOX4	0	0	0
FOLH1B	0	0	0
NAALAD2	1.1398942771	0.8201346513	0.9999763851
CHORDC1	1.082445148	0.7312055905	0.9999763851
FAT3	-1.3154339304	0.8205576571	0.9999763851

SLC36A4	2.2449332928	0.0274954344	0.9999763851
SMCO4	-1.2832893042	0.309391455	0.9999763851
CEP295	-1.2810113349	0.2416677928	0.9999763851
TAF1D	1.6442860233	0.0226397121	0.9999763851
C11orf54	1.1134501195	0.7722426636	0.9999763851
MED17	1.1201543217	0.6875551788	0.9999763851
HEPHL1	0	0	0
PANX1	-1.1307821804	0.6848715402	0.9999763851
MRE11A	-1.1720410399	0.475510112	0.9999763851
ANKRD49	-1.4469657545	0.1857358243	0.9999763851
C11orf97	-2.3209115874	0.4607268102	0.9999763851
FUT4	1.364818085	0.0870329021	0.9999763851
PIWIL4	-1.2173951742	0.6272534066	0.9999763851
AMOTL1	1.5801325271	0.1846220677	0.9999763851
CWC15	1.0179464936	0.9232834958	0.9999763851
SRSF8	-1.1109891469	0.5744700623	0.9999763851
ENDOD1	-1.3217747202	0.4735220445	0.9999763851
SESN3	1.0208478557	0.9691053071	0.9999763851
FAM76B	1.2875518292	0.3218643288	0.9999763851
CEP57	-1.1503577323	0.4700765328	0.9999763851
MTMR2	1.0018974266	0.9937247305	0.9999763851
MAML2	-1.627682253	0.2615210651	0.9999763851
CCDC82	1.460456132	0.3203838581	0.9999763851
JRKL	-1.6207139327	0.4424263779	0.9999763851
CNTN5	5.4781708352	0.3745527127	0.9999763851
ARHGAP42	7.7211724672	0.2779709379	0.9999763851
TMEM133	5.4770877341	0.3746135179	0.9999763851
TRPC6	0	0	0
C11orf70	-4.2849908816	0.4028453076	0.9999763851
BIRC3	1.4098853934	0.4301552738	0.9999763851
BIRC2	-1.1298355859	0.5423583039	0.9999763851
TMEM123	1.0097609095	0.9536972714	0.9999763851
MMP7	-3.3291448741	0.2509160445	0.9999763851
MMP20	0	0	0
MMP8	1.1333715873	0.8726005559	0.9999763851
MMP10	-8.342239448	0.2154408104	0.9999763851
MMP1	-4.6343491481	3.92E-11	2.64E-07
MMP3	-5.3420894951	0.107104171	0.9999763851
DCUN1D5	-1.4863914839	0.0303605823	0.9999763851
DYNC2H1	-1.4398891606	0.4515666575	0.9999763851
CASP4	-1.2754335354	0.5673738748	0.9999763851
CASP1	-1.6289638637	0.2468401906	0.9999763851
CARD16	-1.0416486765	0.9634135134	0.9999763851
MSANTD4	-1.5497302023	0.2020454122	0.9999763851
KBTBD3	-1.1443525594	0.8866090056	0.9999763851
AASDHPPT	1.0654617698	0.8000769253	0.9999763851
GUCY1A2	-1.1904273562	0.594903681	0.9999763851
CWF19L2	-1.0781169176	0.798977013	0.9999763851
ALKBH8	-1.7727596799	0.3006990432	0.9999763851

SLC35F2	1.1620365995	0.7158638779	0.9999763851
RAB39A	0	0	0
CUL5	-1.2667161869	0.2121395773	0.9999763851
ACAT1	1.0170073898	0.91480362	0.9999763851
NPAT	1.1267828244	0.7201433059	0.9999763851
ATM	-1.311395496	0.2791372587	0.9999763851
KDELC2	-3.5820989397	0.005343	0.9999763851
EXPH5	1.2803213157	0.6466122346	0.9999763851
DDX10	1.2524836573	0.2188734688	0.9999763851
ZC3H12C	-1.0440126181	0.9266218969	0.9999763851
RDX	1.0003973295	0.9984608595	0.9999763851
FDX1	-1.108353712	0.5738217796	0.9999763851
ARHGAP20	3.2353357581	0.554787377	0.9999763851
SIK2	1.1826785705	0.6468623915	0.9999763851
PPP2R1B	1.1205983644	0.4842055193	0.9999763851
ALG9	-1.2304465284	0.538375643	0.9999763851
FDXACB1	1.6466739695	0.2373638424	0.9999763851
C11orf1	-1.3504460039	0.3665520678	0.9999763851
HSPB2	0	0	0
C11orf52	0	0	0
DIXDC1	-2.3222877795	0.450910957	0.9999763851
DLAT	-8.3604661647	0.0290780452	0.9999763851
PIH1D2	1.4568251364	0.0835611223	0.9999763851
C11orf57	-1.2811951234	0.3293782385	0.9999763851
TIMM8B	1.0344259866	0.8206773495	0.9999763851
SDHD	1.8432973052	0.0369855774	0.9999763851
IL18	-1.3946909469	0.7147517481	0.9999763851
TEX12	0	0	0
BCO2	2.3398344857	0.4135656902	0.9999763851
PTS	1.0568629461	0.829886905	0.9999763851
PLET1	3.2361532718	0.5546934395	0.9999763851
NCAM1	-5.0519831528	0.0413915934	0.9999763851
TTC12	-1.3953638359	0.6936906344	0.9999763851
TMPRSS5	0	0	0
ZW10	1.1380744274	0.4597575193	0.9999763851
USP28	1.5423203699	0.1200665698	0.9999763851
HTR3B	0	0	0
ZBTB16	-2.6615286456	0.1869435458	0.9999763851
C11orf71	1.4658599468	0.5109208968	0.9999763851
RBM7	-1.7522679011	0.0918201139	0.9999763851
REXO2	1.1059719989	0.671617501	0.9999763851
NXPE2	-2.3214980365	0.4366467708	0.9999763851
BUD13	-1.0548760322	0.8471293087	0.9999763851
ZPR1	1.2120087295	0.4392411754	0.9999763851
SIK3	-1.6215814123	0.0345749104	0.9999763851
PAFAH1B2	-1.0965839776	0.5029567447	0.9999763851
SIDT2	1.0231141341	0.9373311592	0.9999763851
TAGLN	-2.2178496717	0.4267261952	0.9999763851
PCSK7	1.0317365674	0.8978952417	0.9999763851

RNF214	-1.2329246863	0.5735694382	0.9999763851
BACE1	-1.4284678387	0.5770606471	0.9999763851
CEP164	1.2949616389	0.3771936769	0.9999763851
FXYD2	3.2361532718	0.5546934395	0.9999763851
FXYD6	2.3403997491	0.4171851616	0.9999763851
IL10RA	-1.0268089327	0.9294099452	0.9999763851
JAML	3.2353357581	0.554787377	0.9999763851
MPZL3	0	0	0
CD3E	2.1016747882	0.5172996432	0.9999763851
CD3G	0	0	0
UBE4A	1.3550670981	0.113295973	0.9999763851
ATP5L	-1.3052322202	0.0256513894	0.9999763851
KMT2A	-1.2884919816	0.3224336452	0.9999763851
TMEM25	-1.1334584708	0.7593433652	0.9999763851
IFT46	9.0182935529	0.2608495149	0.9999763851
ARCN1	-1.3166740593	0.1600939323	0.9999763851
PHLDB1	1.0271487798	0.9777882605	0.9999763851
DDX6	-1.2180204013	0.1587119066	0.9999763851
CXCR5	-2.9861951639	0.0442663886	0.9999763851
BCL9L	-1.0351698453	0.9623707877	0.9999763851
UPK2	0	0	0
FOXR1	0	0	0
CCDC84	-1.3505206804	0.4984231448	0.9999763851
RPS25	1.0540873508	0.7656647951	0.9999763851
TRAPPC4	1.0298276653	0.8607142902	0.9999763851
SLC37A4	1.6398301766	0.0580339511	0.9999763851
HYOU1	1.0077941852	0.9614315505	0.9999763851
VPS11	1.3234492558	0.4296193548	0.9999763851
HMBS	1.6570081571	0.0137998607	0.9999763851
H2AFX	-1.0345128081	0.8461772579	0.9999763851
DPAGT1	1.4031041064	0.177508555	0.9999763851
C2CD2L	-1.029742369	0.9289993328	0.9999763851
HINFP	1.0688575366	0.8915966954	0.9999763851
NLRX1	1.252607192	0.5278707508	0.9999763851
CBL	-1.2935150851	0.174295431	0.9999763851
RNF26	1.0117842166	0.9547439063	0.9999763851
USP2	-1.4422814889	0.6418633521	0.9999763851
NECTIN1	1.1753784252	0.577019469	0.9999763851
OAF	-1.0371590333	0.8420324712	0.9999763851
TMEM136	0	0	0
ARHGEF12	4.4613963453	0.02510871	0.9999763851
SC5D	-1.0051991567	0.9868206056	0.9999763851
SORL1	1.0533635738	0.7488602299	0.9999763851
UBASH3B	1.0750676819	0.6133015735	0.9999763851
CRTAM	3.2361532718	0.5546934395	0.9999763851
C11orf63	1.7584422599	0.448089874	0.9999763851
HSPA8	1.1210857482	0.3318252021	0.9999763851
GRAMD1B	-1.0405076573	0.833776492	0.9999763851
SCN3B	12.1150575073	0.1935053692	0.9999763851

ZNF202	-1.1268821418	0.7938470845	0.9999763851
VWA5A	-2.1283157091	0.1193169916	0.9999763851
OR8G5	3.2353357581	0.554787377	0.9999763851
TBRG1	1.2002398463	0.3742189224	0.9999763851
SIAE	1.0883507656	0.8430905565	0.9999763851
SPA17	4.9743874554	0.1013229557	0.9999763851
NRGN	1.5680568372	0.0659097285	0.9999763851
VSIG2	1.7447664027	0.3572062657	0.9999763851
ESAM	0	0	0
MSANTD2	-1.1485931013	0.8489951402	0.9999763851
ROBO3	-1.4294246668	0.5387190379	0.9999763851
CCDC15	-1.5666222009	0.2291863852	0.9999763851
SLC37A2	-1.2133026633	0.4043635041	0.9999763851
TMEM218	-1.1527732967	0.4737511048	0.9999763851
PKNOX2	-1.3709710942	0.7480489583	0.9999763851
FEZ1	3.2353357581	0.554787377	0.9999763851
EI24	1.0651277587	0.6279704872	0.9999763851
STT3A	-1.0509716589	0.8045400931	0.9999763851
CHEK1	-1.1979059207	0.4538173584	0.9999763851
ACRV1	3.3300007086	0.101203885	0.9999763851
PATE4	0	0	0
HYLS1	1.3427855291	0.766209566	0.9999763851
PUS3	1.9505880649	0.0167025056	0.9999763851
CDON	-1.0477046426	0.9130827507	0.9999763851
LOC105369591	0	0	0
RPUSD4	1.1025683607	0.6939179025	0.9999763851
FAM118B	3.2356557963	0.5547505991	0.9999763851
SRPRA	-1.04045024	0.8407572776	0.9999763851
FOXRED1	-1.0198393273	0.9321438735	0.9999763851
TIRAP	-1.8170402834	0.4612648103	0.9999763851
DCPS	-1.2034088468	0.2592990255	0.9999763851
ST3GAL4	-1.1872475607	0.331043967	0.9999763851
ETS1	-1.1432044445	0.4427971187	0.9999763851
FLI1	-1.2856701017	0.1108252676	0.9999763851
KCNJ1	-2.387415242	0.1994751072	0.9999763851
C11orf45	1.281021076	0.6278819723	0.9999763851
ARHGAP32	1.2796805414	0.3342992563	0.9999763851
BARX2	0	0	0
TMEM45B	-1.4159079662	0.6106094087	0.9999763851
NFRKB	-1.1125046297	0.6395885715	0.9999763851
PRDM10	1.1735024689	0.7592989575	0.9999763851
APLP2	1.0600948959	0.645071191	0.9999763851
ST14	0	0	0
ZBTB44	-1.0170394511	0.9061432836	0.9999763851
JAM3	1.4018110974	0.2629076277	0.9999763851
NCAPD3	1.6093199957	0.1088499801	0.9999763851
VPS26B	1.2106199021	0.2598928117	0.9999763851
THYN1	1.126765711	0.519327967	0.9999763851
ACAD8	-1.0506770252	0.8888489299	0.9999763851

IQSEC3	-2.037919908	0.4308742935	0.9999763851
SLC6A12	-1.3154339304	0.8205576571	0.9999763851
SLC6A13	0	0	0
KDM5A	1.1556593628	0.4731910774	0.9999763851
CCDC77	1.0206647194	0.9526668595	0.9999763851
B4GALNT3	1.3582418801	0.5651099155	0.9999763851
NINJ2	1.0411710409	0.8957462119	0.9999763851
WNK1	1.2506531947	0.1722724379	0.9999763851
RAD52	-1.0809624948	0.6607869766	0.9999763851
ERC1	-1.263705764	0.3060291146	0.9999763851
FBXL14	1.2870167985	0.3085679203	0.9999763851
WNT5B	3.2353357581	0.554787377	0.9999763851
ADIPOR2	1.0344256097	0.8224577966	0.9999763851
CACNA2D4	-1.4159103415	0.1049750955	0.9999763851
DCP1B	1.3130992724	0.6476834362	0.9999763851
CACNA1C	1.3174677035	0.4905105285	0.9999763851
FKBP4	1.0189482716	0.9074544403	0.9999763851
ITFG2	-1.1264604962	0.7393438531	0.9999763851
NRIP2	-1.9649399627	0.4624713245	0.9999763851
LOC101929469	1.8136032532	0.5936433508	0.9999763851
FOXM1	-1.0803009863	0.6574656063	0.9999763851
RHNO1	-1.0116385133	0.9610742502	0.9999763851
TULP3	-2.1386792405	0.0443763238	0.9999763851
TEAD4	-4.2849908816	0.4028453076	0.9999763851
TSPAN9	1.1303300756	0.2946827138	0.9999763851
CRACR2A	-1.5034858819	0.2304274565	0.9999763851
PARP11	1.2810764603	0.4454070136	0.9999763851
CCND2	-1.0022657819	0.9901131285	0.9999763851
TIGAR	-1.3835329262	0.3576012447	0.9999763851
C12orf4	-1.6665665338	0.0894600368	0.9999763851
RAD51AP1	-1.093125409	0.6801551931	0.9999763851
DYRK4	1.2897802709	0.4083818954	0.9999763851
AKAP3	-2.3222877795	0.4510535509	0.9999763851
NDUFA9	1.3551043853	0.0791316537	0.9999763851
KCNA6	-1.4078456073	0.6523815848	0.9999763851
VWF	-1.3154339304	0.8205576571	0.9999763851
CD9	-1.4889549815	0.1013083694	0.9999763851
PLEKHG6	1.0276562285	0.9749404666	0.9999763851
TNFRSF1A	-1.278099635	0.1719141242	0.9999763851
LTBR	-1.3962107668	0.1588013625	0.9999763851
CD27	-1.9607753243	0.3468462156	0.9999763851
TAPBPL	1.2212990596	0.7249164228	0.9999763851
VAMP1	1.854443779	0.1451917865	0.9999763851
MRPL51	1.0601963822	0.6847359567	0.9999763851
NCAPD2	1.3349188643	0.0907123617	0.9999763851
GAPDH	1.162567248	0.2096633372	0.9999763851
IFFO1	-1.8419078132	0.0361636907	0.9999763851
NOP2	1.1182161592	0.5837093852	0.9999763851
CHD4	-1.0648360591	0.648614847	0.9999763851

LPAR5	0	0	0
ING4	-1.5316049682	0.106134764	0.9999763851
ZNF384	-1.0806909421	0.9257208946	0.9999763851
PIANP	0	0	0
COPS7A	1.1361341026	0.5585706901	0.9999763851
MLF2	1.0043605061	0.9796557739	0.9999763851
PTMS	-3.3707866895	0.0760156811	0.9999763851
CD4	-1.9583625423	0.002616	0.9999763851
GPR162	4.0100732174	0.092674205	0.9999763851
P3H3	0	0	0
GNB3	1.2068244943	0.7388347796	0.9999763851
CDCA3	1.0509855951	0.805758824	0.9999763851
USP5	1.1599005311	0.5760346524	0.9999763851
TPI1	1.0736395399	0.605573557	0.9999763851
SPSB2	1.8308492597	0.2221102442	0.9999763851
LRRC23	1.7937734078	0.2742282374	0.9999763851
ENO2	-1.2066415907	0.6077059447	0.9999763851
ATN1	-1.3876013709	0.5592854581	0.9999763851
C12orf57	1.0608841114	0.6960711895	0.9999763851
PTPN6	1.2299091006	0.3333270635	0.9999763851
PHB2	1.0029521756	0.9820812625	0.9999763851
EMG1	1.0265346575	0.8984703422	0.9999763851
LPCAT3	1.0145103141	0.949205554	0.9999763851
C1R	-4.3338228569	0.1406162149	0.9999763851
C1RL	1.1838848411	0.6976778895	0.9999763851
RBP5	0	0	0
CLSTN3	5.4790129495	0.3759263024	0.9999763851
PEX5	-1.2050188027	0.6578286764	0.9999763851
CD163L1	1.0158315147	0.9640692406	0.9999763851
APOBEC1	-7.5622178268	0.2177145568	0.9999763851
NANOG	1.2867653957	0.8300593121	0.9999763851
SLC2A14	-2.3233606776	0.4606540202	0.9999763851
SLC2A3	-1.5839517417	0.009095	0.9999763851
FOXJ2	1.2358838774	0.5359166854	0.9999763851
C3AR1	1.0572168065	0.907348269	0.9999763851
NECAP1	1.500566265	0.3840509599	0.9999763851
CLEC4A	-4.2864494036	0.4027150576	0.9999763851
FAM90A1	1.6889837257	0.5257211958	0.9999763851
CLEC6A	3.2353357581	0.554787377	0.9999763851
CLEC4D	0	0	0
CLEC4E	3.2353357581	0.554787377	0.9999763851
AICDA	0	0	0
RIMKLB	-1.4307207903	0.5121677191	0.9999763851
PHC1	-1.5219491751	0.3405771983	0.9999763851
M6PR	-1.0307072292	0.8932054804	0.9999763851
KLRG1	-1.5222481543	0.3262697284	0.9999763851
LOC728715	-1.3716482783	0.7379337699	0.9999763851
LOC105376678	-2.3222877795	0.4511966308	0.9999763851
KLRB1	-1.3942010814	0.6866801413	0.9999763851

LOC105369728	-14.1218219134	0.1012694921	0.9999763851
CLEC2D	-1.3773671343	0.2576906435	0.9999763851
CLECL1	1.223014665	0.1714336947	0.9999763851
CD69	-1.2947353761	0.3574718524	0.9999763851
KLRF1	2.3396033966	0.4202464014	0.9999763851
CLEC2B	-1.1053561302	0.4270481667	0.9999763851
KLRF2	-4.2849908816	0.4028453076	0.9999763851
CLEC2A	-1.1451177614	0.4176092349	0.9999763851
CLEC12A	-1.179741572	0.3186594552	0.9999763851
CLEC1B	1.9569793291	0.1261980914	0.9999763851
CLEC12B	1.0686406341	0.9156239439	0.9999763851
CLEC1A	0	0	0
CLEC7A	-4.2864494036	0.4027150576	0.9999763851
OLR1	-1.9665787608	0.4507082304	0.9999763851
TMEM52B	2.4673165638	0.3081930627	0.9999763851
GABARAPL1	-1.3813335983	0.5942230549	0.9999763851
MAGOHB	-1.0657812115	0.7720234114	0.9999763851
STYK1	0	0	0
PRR4	9.0182935529	0.2609092261	0.9999763851
PRH1	-1.4872057783	0.1999842305	0.9999763851
PRH2	3.2353357581	0.554787377	0.9999763851
PRH1-TAS2R14	2.0522850196	0.2163751342	0.9999763851
TAS2R20	-1.3713018672	0.7312784555	0.9999763851
SMIM10L1	-1.1035941466	0.6722274371	0.9999763851
TAS2R42	3.2356557963	0.5547505991	0.9999763851
PRB2	0	0	0
ETV6	-1.7110359294	0.0165517854	0.9999763851
BCL2L14	-3.3287018261	0.2432318723	0.9999763851
LOC100506248	-1.0267611171	0.8620071521	0.9999763851
LRP6	-1.3160416743	0.827111756	0.9999763851
BORCS5	-1.7105841649	0.2855292439	0.9999763851
DUSP16	-1.3660704831	0.096177093	0.9999763851
CREBL2	-1.27741639	0.4661415069	0.9999763851
GPR19	1.1979463287	0.7308991062	0.9999763851
CDKN1B	-1.5311947631	0.003942	0.9999763851
APOLD1	-1.8621602614	0.0109143542	0.9999763851
DDX47	1.1741180746	0.6935564891	0.9999763851
FAM234B	-1.2791784011	0.6198824746	0.9999763851
GPRC5D	0	0	0
HEBP1	1.0749218048	0.6761717498	0.9999763851
GSG1	-2.5600051511	0.2551885332	0.9999763851
EMP1	-2.2449641806	0.004633	0.9999763851
GRIN2B	3.2356557963	0.5547505991	0.9999763851
ATF7IP	-1.5606018786	0.0790300641	0.9999763851
C12orf60	-1.6000437015	0.4089217587	0.9999763851
H2AFJ	-1.3158816768	0.8203229797	0.9999763851
WBP11	-1.3808917577	0.0891101928	0.9999763851
SMCO3	0	0	0
ART4	0	0	0

ERP27	1.8308244325	0.3752642869	0.9999763851
ARHGDIB	1.2675477183	0.0422967967	0.9999763851
PDE6H	3.2356557963	0.5547505991	0.9999763851
PTPRO	-4.2849908816	0.4028453076	0.9999763851
EPS8	1.3262899408	0.7595844133	0.9999763851
STRAP	1.078410246	0.6412728349	0.9999763851
DERA	-1.1809434159	0.5149571525	0.9999763851
SLC15A5	0	0	0
MGST1	1.1864304412	0.3221895407	0.9999763851
PLCZ1	1.01706669	0.9418734652	0.9999763851
AEBP2	1.0244775335	0.9241828573	0.9999763851
PDE3A	1.2587826646	0.4498440593	0.9999763851
SLCO1B7	3.2353357581	0.554787377	0.9999763851
SLCO1B1	0	0	0
PYROXD1	1.374321878	0.103971806	0.9999763851
RECQL	-2.1907380469	0.077650129	0.9999763851
GOLT1B	1.1641908516	0.4852191293	0.9999763851
GYS2	6.368054416	0.3491891175	0.9999763851
LDHB	1.1941797877	0.1368990723	0.9999763851
ABCC9	0	0	0
CMAS	-1.2696767654	0.220574821	0.9999763851
ST8SIA1	0	0	0
C2CD5	-1.317121473	0.3896799217	0.9999763851
ETNK1	-1.0934677069	0.617939118	0.9999763851
SOX5	-1.3157515262	0.8223988689	0.9999763851
BCAT1	-1.0339624653	0.8485265576	0.9999763851
LRMP	-1.9057962763	0.1369813077	0.9999763851
CASC1	1.2454866827	0.8305954337	0.9999763851
LYRM5	2.2525498699	0.0835794197	0.9999763851
KRAS	1.1116076138	0.5570289451	0.9999763851
LMNTD1	3.2361532718	0.5546934395	0.9999763851
BHLHE41	1.6182325762	0.4523455056	0.9999763851
SSPN	-2.3217011239	0.435585292	0.9999763851
ITPR2	1.3118767628	0.3033206988	0.9999763851
ASUN	1.0280155056	0.8903136334	0.9999763851
FGFR1OP2	-1.4339256786	0.070077276	0.9999763851
TM7SF3	-1.0982844089	0.5097843669	0.9999763851
MED21	1.4543830584	0.1417851921	0.9999763851
C12orf71	0	0	0
STK38L	1.1115930993	0.7220522451	0.9999763851
ARNTL2	1.185296182	0.7165145745	0.9999763851
SMCO2	0	0	0
PPFIBP1	-3.088460367	0.007789	0.9999763851
REP15	0	0	0
MRPS35	1.3639118056	0.0571773411	0.9999763851
KLHL42	-1.1919161296	0.3327528336	0.9999763851
CCDC91	1.0733932509	0.7940682375	0.9999763851
FAR2	-1.1271144273	0.6708875755	0.9999763851
ERGIC2	-1.0723959035	0.7459784794	0.9999763851

IPO8	1.3495129021	0.1520583297	0.9999763851
CAPRIN2	1.9673046429	0.3556155176	0.9999763851
DDX11	1.2411278332	0.4966667032	0.9999763851
OVOS2	7.7488609396	0.3059804961	0.9999763851
FAM60A	1.0874294147	0.6629174683	0.9999763851
DENND5B	0	0	0
ETFBKMT	-10.3797341339	0.0137668691	0.9999763851
AMN1	-1.3778718463	0.4532522613	0.9999763851
H3F3C	0	0	0
KIAA1551	-1.1307508007	0.6838135318	0.9999763851
BICD1	-1.2257825366	0.4174886108	0.9999763851
FGD4	1.0097257706	0.954525635	0.9999763851
DNM1L	1.0744190344	0.6525097427	0.9999763851
YARS2	-1.0413307662	0.8863415275	0.9999763851
SYT10	0	0	0
ALG10	1.1142028814	0.8847026385	0.9999763851
ALG10B	-1.2914969252	0.3981572677	0.9999763851
CPNE8	1.3729073284	0.3903695724	0.9999763851
KIF21A	-1.0670397333	0.8462441559	0.9999763851
SLC2A13	-1.3506115695	0.4924534057	0.9999763851
LRRK2	0	0	0
MUC19	3.2361532718	0.5546934395	0.9999763851
CNTN1	3.2356557963	0.5547505991	0.9999763851
PDZRN4	-1.887160399	0.0605198417	0.9999763851
GXYLT1	-1.5006822338	0.0378881136	0.9999763851
YAF2	1.0983589586	0.6185831095	0.9999763851
ZCRB1	-1.1094492781	0.5710826962	0.9999763851
PPHLN1	1.0429105735	0.7747715166	0.9999763851
PRICKLE1	1.9672084963	0.4361707133	0.9999763851
ADAMTS20	3.2356557963	0.5547505991	0.9999763851
PUS7L	-1.3012745614	0.3726395662	0.9999763851
IRAK4	1.1740900496	0.5594751174	0.9999763851
TWF1	1.020188711	0.9458097593	0.9999763851
TMEM117	-2.560793319	0.2842140824	0.9999763851
NELL2	3.2356557963	0.5547505991	0.9999763851
ANO6	-1.1578274269	0.6302608396	0.9999763851
ARID2	-1.1506197858	0.5768500868	0.9999763851
SCAF11	-1.0110574562	0.9370486954	0.9999763851
SLC38A1	1.2066671239	0.2369485443	0.9999763851
SLC38A2	1.2271925499	0.1707606731	0.9999763851
SLC38A4	0	0	0
AMIGO2	5.4790129495	0.3762270444	0.9999763851
PCED1B	-1.0863345041	0.8632896557	0.9999763851
RPAP3	1.4539082266	0.1176291472	0.9999763851
ENDOU	0	0	0
RAPGEF3	-7.5622178268	0.2177145568	0.9999763851
SLC48A1	1.2642745034	0.4892678972	0.9999763851
HDAC7	-1.2883792986	0.165515083	0.9999763851
VDR	-1.2140099331	0.3267171635	0.9999763851

TMEM106C	-1.2760280268	0.1449685733	0.9999763851
SENP1	-1.3326566059	0.2713811414	0.9999763851
PFKM	14.4482274128	0.15092542	0.9999763851
ASB8	-1.1184497767	0.6613156813	0.9999763851
ZNF641	1.2251858726	0.7478337156	0.9999763851
C12orf54	6.368054416	0.3491960774	0.9999763851
ANP32D	0	0	0
KANSL2	1.1693806602	0.4646005284	0.9999763851
CCNT1	1.4443049625	0.0932298187	0.9999763851
CACNB3	1.0019060314	0.9946243066	0.9999763851
DDX23	1.2732764948	0.1559279644	0.9999763851
RND1	3.3680355205	0.2626171715	0.9999763851
CCDC65	-1.9647456674	0.4668002959	0.9999763851
FKBP11	1.325955166	0.7493014348	0.9999763851
ARF3	1.0153077251	0.9178805863	0.9999763851
WNT10B	1.3662413441	0.5183024283	0.9999763851
WNT1	0	0	0
DDN	0	0	0
PRKAG1	-1.3164821438	0.226568568	0.9999763851
KMT2D	-1.1975892914	0.4882034401	0.9999763851
RHEBL1	-1.1136064003	0.6382350518	0.9999763851
LMBR1L	-1.4402112053	0.3931295054	0.9999763851
TUBA1B	-1.0971762785	0.4190869824	0.9999763851
TUBA1A	-1.0480101495	0.7425101697	0.9999763851
TUBA1C	-1.1091508177	0.3682329793	0.9999763851
PRPH	-1.9654362824	0.4778548266	0.9999763851
TROAP	-1.3220726355	0.4043267527	0.9999763851
C1QL4	3.2356557963	0.5547505991	0.9999763851
SPATS2	1.3989121072	0.2941693859	0.9999763851
MCRS1	1.054919057	0.7947388225	0.9999763851
FAM186B	1.5498231645	0.5451352725	0.9999763851
PRPF40B	-1.4351206863	0.449626059	0.9999763851
FMNL3	1.011351558	0.9767684969	0.9999763851
TMBIM6	1.1369336709	0.3288446411	0.9999763851
NCKAP5L	-1.1973379604	0.8034421863	0.9999763851
BCDIN3D	-1.5606624867	0.3482060469	0.9999763851
FAIM2	0	0	0
AQP6	-4.2849908816	0.4028453076	0.9999763851
RACGAP1	1.0750443486	0.6963764069	0.9999763851
ASIC1	-1.2608756966	0.2925182096	0.9999763851
SMARCD1	1.274453323	0.4652088953	0.9999763851
COX14	1.1479511909	0.3275518091	0.9999763851
CERS5	1.0226958662	0.9239027888	0.9999763851
LIMA1	-1.1482733712	0.5269366991	0.9999763851
LARP4	-1.0492208539	0.7807706192	0.9999763851
DIP2B	-1.2860764275	0.3418098884	0.9999763851
ATF1	-1.0198107596	0.9264464811	0.9999763851
TMPRSS12	0	0	0
METTL7A	1.4014822113	0.2305499446	0.9999763851

HIGD1C	12.2055584829	0.1780614767	0.9999763851
SLC11A2	1.1759996854	0.5595462097	0.9999763851
LETMD1	1.2696519671	0.2717558203	0.9999763851
CSRNP2	-1.3196367789	0.5563539322	0.9999763851
TFCP2	1.0783264607	0.6502619906	0.9999763851
POU6F1	1.6426539897	0.366230928	0.9999763851
DAZAP2	-1.079708293	0.5982328885	0.9999763851
SMAGP	1.2815256224	0.7943925444	0.9999763851
BIN2	-1.2054559085	0.3225942626	0.9999763851
CELA1	-1.0658620272	0.9360605674	0.9999763851
GALNT6	-1.2171767658	0.3401276819	0.9999763851
SLC4A8	-2.3227182272	0.1433892385	0.9999763851
SCN8A	-2.5766131218	0.0564204456	0.9999763851
FIGNL2	2.027155953	0.4561872529	0.9999763851
ACVRL1	-5.5367104188	0.0318782444	0.9999763851
ACVR1B	-2.2277517054	0.007771	0.9999763851
GRASP	-2.4440355816	0.4434695289	0.9999763851
NR4A1	1.1390433155	0.8350984278	0.9999763851
ATG101	-1.2342403926	0.2560601265	0.9999763851
KRT80	0	0	0
KRT7	-1.7802290875	0.520804732	0.9999763851
KRT86	1.0560111589	0.716729178	0.9999763851
KRT81	1.5956463682	0.1870072494	0.9999763851
KRT2	-1.0346478895	0.9604887723	0.9999763851
KRT1	-20.6829910744	0.056372274	0.9999763851
KRT77	0	0	0
KRT3	-1.0629193228	0.9429506554	0.9999763851
KRT4	0	0	0
KRT79	-9.0566712722	0.0551899426	0.9999763851
KRT78	-4.2864494036	0.4027150576	0.9999763851
EIF4B	-1.1054358986	0.5092774729	0.9999763851
TNS2	5.4784711963	0.3745358536	0.9999763851
SPRYD3	1.3009924456	0.4589672075	0.9999763851
IGFBP6	1.9229787277	0.4494470579	0.9999763851
SOAT2	1.8142404796	0.5678116976	0.9999763851
CSAD	-2.5610465578	0.2827494718	0.9999763851
ZNF740	-1.1804194403	0.5869645893	0.9999763851
ITGB7	-4.6450398006	0.0004307	0.4545015308
RARG	-1.0164253063	0.9779732893	0.9999763851
MFSD5	1.4563531	0.089265473	0.9999763851
ESPL1	1.6291323892	0.0911363827	0.9999763851
PFDN5	-1.3010829267	0.1250406317	0.9999763851
C12orf10	-1.3673585473	0.2630428083	0.9999763851
AAAS	-1.4940668448	0.2843531942	0.9999763851
SP1	-1.2939628123	0.2875548805	0.9999763851
PRR13	-1.0760630455	0.5866095563	0.9999763851
PCBP2	-1.0241398896	0.8498098253	0.9999763851
MAP3K12	-4.2864494036	0.4027150576	0.9999763851
TARBP2	1.1455776172	0.6239438968	0.9999763851

NPFF	1.8137824633	0.5639230692	0.9999763851
ATF7	1.4172000507	0.4687992903	0.9999763851
ATP5G2	-1.0327621783	0.7923230233	0.9999763851
CALCOCO1	-3.7510687687	0.10461698	0.9999763851
HOXC11	3.2353357581	0.554787377	0.9999763851
HOXC10	-1.2264847888	0.4964468664	0.9999763851
HOXC9	-1.0806556559	0.9243074408	0.9999763851
LOC107984512	0	0	0
HOXC8	-2.384593258	0.1959859761	0.9999763851
HOXC6	-1.3907093864	0.3144885423	0.9999763851
HOXC5	-14.1266231044	0.0934860779	0.9999763851
HOXC4	-1.2179546502	0.6362964223	0.9999763851
SMUG1	-1.2176171242	0.5871840894	0.9999763851
CBX5	1.1357829648	0.4181835842	0.9999763851
HNRNPA1	1.2358746064	0.0976169469	0.9999763851
NFE2	1.1709907292	0.4735997042	0.9999763851
COPZ1	-1.2347514516	0.2387026811	0.9999763851
GPR84	1.2600857935	0.4046665229	0.9999763851
ZNF385A	5.0439373197	0.1373919139	0.9999763851
ITGA5	1.3252539819	0.1349715915	0.9999763851
GTSF1	1.4019998954	0.0711194924	0.9999763851
NCKAP1L	1.0605476193	0.6453733337	0.9999763851
PDE1B	-1.3016311333	0.5086680194	0.9999763851
METTL7B	-1.9661045084	0.4534785671	0.9999763851
ITGA7	-1.2364244735	0.6561766678	0.9999763851
BLOC1S1	1.3847052766	0.088155645	0.9999763851
RDH5	2.3396033966	0.4195336873	0.9999763851
CD63	-1.4427490365	0.004669	0.9999763851
GDF11	1.1649936507	0.5930395516	0.9999763851
SARNP	1.2341142649	0.1696493315	0.9999763851
ORMDL2	-1.1024527533	0.6981427948	0.9999763851
DNAJC14	1.2052932884	0.7140409236	0.9999763851
MMP19	-1.6009524639	0.3424507204	0.9999763851
PYM1	-1.2968803677	0.2360833161	0.9999763851
DGKA	-1.3400248288	0.3759060896	0.9999763851
PMEL	-2.4440355816	0.4432184348	0.9999763851
CDK2	-1.2664749725	0.1505215264	0.9999763851
RAB5B	-1.7263628306	0.001008	0.7976857137
SUOX	-2.3882538214	0.2039514464	0.9999763851
IKZF4	-1.3815626988	0.7462525959	0.9999763851
RPS26	1.1818099431	0.4896654199	0.9999763851
PA2G4	-1.0368902275	0.7638635184	0.9999763851
RPL41	-1.0450875133	0.7046968234	0.9999763851
ZC3H10	-1.5856089735	0.3884915118	0.9999763851
ESYT1	1.2538702881	0.1867489324	0.9999763851
LOC107987175	0	0	0
MYL6B	-1.1176748757	0.6285168017	0.9999763851
MYL6	1.0096359395	0.9454915792	0.9999763851
SMARCC2	1.0861791589	0.6764776978	0.9999763851

RNF41	-1.1178255036	0.6804172692	0.9999763851
NABP2	1.2369280863	0.2819900412	0.9999763851
ANKRD52	1.129642016	0.6330471908	0.9999763851
COQ10A	-1.1256157506	0.781398033	0.9999763851
CS	1.1962129382	0.199235243	0.9999763851
CNPY2	1.0058470155	0.9713834079	0.9999763851
PAN2	-1.4422794878	0.3329084101	0.9999763851
IL23A	-2.3884896926	0.2362464309	0.9999763851
STAT2	-2.237104103	0.014089415	0.9999763851
TIMELESS	-1.0448018256	0.8036760493	0.9999763851
SPRYD4	-1.0502065902	0.8893673825	0.9999763851
GLS2	19.4295281995	0.1168822197	0.9999763851
BAZ2A	-1.3990320541	0.1674366198	0.9999763851
ATP5B	1.1551663802	0.230680047	0.9999763851
PTGES3	-1.0249118322	0.8434892508	0.9999763851
NACA	1.0847532938	0.5290258523	0.9999763851
PRIM1	-1.3753060378	0.3879855354	0.9999763851
HSD17B6	-1.015368255	0.9782471204	0.9999763851
RDH16	0	0	0
GPR182	0	0	0
ZBTB39	1.6738056472	0.2335313431	0.9999763851
TAC3	-4.2864494036	0.4027150576	0.9999763851
MYO1A	1.2867653957	0.8300189633	0.9999763851
NEMP1	1.0334617858	0.8952091416	0.9999763851
NAB2	1.0738088626	0.8206363691	0.9999763851
STAT6	1.0708001324	0.7693418495	0.9999763851
LRP1	0	0	0
NXPH4	-1.0810230665	0.9182586474	0.9999763851
SHMT2	1.0210378276	0.8942781235	0.9999763851
NDUFA4L2	-1.3155492632	0.8261295197	0.9999763851
STAC3	1.9661403391	0.3468098204	0.9999763851
R3HDM2	-1.1171701541	0.6989810798	0.9999763851
INHBE	-1.3710462586	0.7620868046	0.9999763851
GLI1	1.7432383105	0.3363935773	0.9999763851
ARHGAP9	1.1273757736	0.3953737558	0.9999763851
MARS	1.2245044181	0.2325078268	0.9999763851
DDIT3	-1.0130599365	0.9616924742	0.9999763851
MBD6	-1.4784390749	0.1564422424	0.9999763851
DCTN2	1.0351143167	0.8112585338	0.9999763851
KIF5A	0	0	0
PIP4K2C	-1.0511522002	0.8059590378	0.9999763851
DTX3	3.2353357581	0.554787377	0.9999763851
ARHGEF25	1.3166612325	0.512196322	0.9999763851
B4GALNT1	1.4035461069	0.5107049853	0.9999763851
OS9	-1.1125850509	0.5684800048	0.9999763851
AGAP2	2.0014523426	0.058932647	0.9999763851
TSPAN31	-1.3158393667	0.828022737	0.9999763851
CDK4	-1.0653580613	0.6790328936	0.9999763851
MARCH9	-1.4522402311	0.1891823743	0.9999763851

CYP27B1	1.8300010925	0.3755956347	0.9999763851
METTL1	-1.2111696858	0.5156002855	0.9999763851
METTL21B	-1.0440247144	0.8712518178	0.9999763851
TSFM	-1.1222150249	0.6026250864	0.9999763851
AVIL	1.1395802074	0.8210180636	0.9999763851
CTDSP2	-1.0524694221	0.7251525612	0.9999763851
ATP23	1.8155966892	0.0401856557	0.9999763851
FAM19A2	-1.0438195622	0.8468584009	0.9999763851
USP15	-1.4663283799	0.0757193091	0.9999763851
MON2	-1.2057748068	0.5189394406	0.9999763851
PPM1H	-1.1585895879	0.7098807152	0.9999763851
TMEM5	-1.0327801934	0.9381799147	0.9999763851
SRGAP1	-1.2038877456	0.7029617524	0.9999763851
C12orf66	1.1987700037	0.7039884516	0.9999763851
XPOT	1.7539496929	0.0007286	0.6510516988
TBK1	-1.4376052732	0.4083939747	0.9999763851
RASSF3	-1.0744961362	0.751900866	0.9999763851
GNS	-1.0866715991	0.5966824599	0.9999763851
TBC1D30	1.3415763937	0.6951087905	0.9999763851
WIF1	0	0	0
LEMD3	1.4179080198	0.1985431086	0.9999763851
MSRB3	1.1186632532	0.4824272368	0.9999763851
HMGA2	3.2353357581	0.554787377	0.9999763851
LOC100129940	0	0	0
LLPH	1.153222613	0.3783481134	0.9999763851
TMBIM4	-1.0367084778	0.8192895079	0.9999763851
IRAK3	1.1703613646	0.346671282	0.9999763851
HELB	1.3764516987	0.6092752561	0.9999763851
GRIP1	-1.9611742755	0.0609968596	0.9999763851
CAND1	-1.1321526656	0.4267968118	0.9999763851
DYRK2	-1.4682883541	0.1386160072	0.9999763851
MDM1	1.3253189807	0.4924627294	0.9999763851
RAP1B	-1.1818597803	0.2354564477	0.9999763851
NUP107	1.0091781346	0.9691932706	0.9999763851
SLC35E3	-1.5601557122	0.1121021493	0.9999763851
MDM2	1.2219979853	0.4369192603	0.9999763851
CPM	1.1765662098	0.6376044304	0.9999763851
CPSF6	1.3215883416	0.0494016788	0.9999763851
LYZ	-1.3669658748	0.0869199127	0.9999763851
YEATS4	-1.1637343363	0.4386705026	0.9999763851
FRS2	-1.3214047712	0.5063138756	0.9999763851
CCT2	-1.0219004506	0.8689292733	0.9999763851
BEST3	3.2353357581	0.554787377	0.9999763851
RAB3IP	2.2435235174	0.0108368454	0.9999763851
MYRFL	5.4781708352	0.3745527127	0.9999763851
CNOT2	1.1823793977	0.3504903407	0.9999763851
KCNMB4	-3.3272796812	0.2432983936	0.9999763851
PTPRB	-1.1390506844	0.7292068483	0.9999763851
PTPRR	0	0	0

ZFC3H1	-1.2632087649	0.5037280418	0.9999763851
THAP2	-2.0471526469	0.1532216967	0.9999763851
TMEM19	1.4494567783	0.2834248967	0.9999763851
RAB21	1.0617035472	0.7532999984	0.9999763851
TBC1D15	1.0841369938	0.6538755465	0.9999763851
TPH2	0	0	0
TRHDE	-4.2849908816	0.4028453076	0.9999763851
ATXN7L3B	1.2859308211	0.2188961716	0.9999763851
GLIPR1	1.2330585255	0.2632767943	0.9999763851
KRR1	-1.4396652144	0.3151871719	0.9999763851
PHLDA1	-1.7445027335	0.116735103	0.9999763851
NAP1L1	-1.1900657307	0.1831690929	0.9999763851
BBS10	-1.3948129396	0.6824171443	0.9999763851
OSBPL8	-1.0773507625	0.6470274596	0.9999763851
ZDHHC17	-1.8142908552	0.1961389679	0.9999763851
CSRP2	0	0	0
SYT1	3.2353357581	0.554787377	0.9999763851
PAWR	0	0	0
PPP1R12A	-1.1098482068	0.5982727939	0.9999763851
LOC107984534	0	0	0
OTOGL	0	0	0
PTPRQ	0	0	0
PPFIA2	3.2356557963	0.5547505991	0.9999763851
CCDC59	1.1313126825	0.5336763931	0.9999763851
METTL25	-1.4361212023	0.4463144691	0.9999763851
TMTC2	1.245188991	0.5656833032	0.9999763851
LRRIQ1	0	0	0
MGAT4C	1.3264789913	0.7364653153	0.9999763851
C12orf50	0	0	0
C12orf29	1.1284689447	0.7015390269	0.9999763851
CEP290	-1.507367245	0.2995486116	0.9999763851
TMTC3	1.8338024858	0.1622993667	0.9999763851
DUSP6	-1.0772985462	0.6125684594	0.9999763851
POC1B	1.0728240527	0.75944187	0.9999763851
GALNT4	4.8791443919	0.0119753603	0.9999763851
ATP2B1	-1.2319005127	0.2625366812	0.9999763851
DCN	0	0	0
BTG1	-1.3762231099	0.0238101753	0.9999763851
CLLU1OS	0	0	0
PLEKHG7	1.8888297566	0.5937424439	0.9999763851
EEA1	-1.1609678916	0.5744726564	0.9999763851
NUDT4	1.1257780379	0.4252355397	0.9999763851
UBE2N	1.071059093	0.6610136971	0.9999763851
MRPL42	1.2055858703	0.2870388985	0.9999763851
SOCS2	4.4659480018	0.0189331185	0.9999763851
CRADD	1.0532517376	0.8918397042	0.9999763851
PLXNC1	1.3766957264	0.221495003	0.9999763851
CEP83	-1.5866901523	0.1330849648	0.9999763851
NDUFA12	1.388199735	0.027171701	0.9999763851

NR2C1	1.056695501	0.8471069127	0.9999763851
FGD6	-1.3373750721	0.5214808288	0.9999763851
VEZT	1.3226203449	0.2275108441	0.9999763851
METAP2	-1.0403892141	0.7612254046	0.9999763851
USP44	1.6372041402	0.0125820513	0.9999763851
NTN4	3.2356557963	0.5547505991	0.9999763851
SNRPF	1.1424641012	0.3630883163	0.9999763851
CCDC38	-1.9672070343	0.4589649407	0.9999763851
AMDHD1	1.8145193361	0.5769212622	0.9999763851
HAL	-1.0400495026	0.952350152	0.9999763851
LTA4H	-1.3406994067	0.1626335555	0.9999763851
ELK3	-1.0767776346	0.6899154922	0.9999763851
CDK17	1.0535408318	0.8563018775	0.9999763851
NEDD1	-1.2534975641	0.2250237113	0.9999763851
TMPO	-1.1082455856	0.4114642247	0.9999763851
SLC25A3	1.4802807089	0.00155	0.9999763851
IKBIP	1.0855182472	0.7216795562	0.9999763851
APAF1	-1.4110698006	0.2843397327	0.9999763851
ANKS1B	-4.3327792956	0.1655099731	0.9999763851
UHRF1BP1L	1.0690837976	0.7971214744	0.9999763851
ACTR6	-1.3342619073	0.3206561677	0.9999763851
DEPDC4	-1.3157515262	0.8224004334	0.9999763851
SCYL2	1.2857852807	0.3732899472	0.9999763851
NR1H4	0	0	0
GAS2L3	-1.6899157383	0.2658794665	0.9999763851
UTP20	-1.0218852586	0.937424121	0.9999763851
ARL1	-1.063374598	0.7105963071	0.9999763851
CHPT1	1.1639389253	0.4515621415	0.9999763851
SYCP3	9.9312452783	0.2486349035	0.9999763851
GNPTAB	-1.0658243062	0.7538044472	0.9999763851
DRAM1	-1.1393632939	0.5544736177	0.9999763851
CCDC53	-1.1577250277	0.7454565085	0.9999763851
NUP37	1.1234130511	0.5998784207	0.9999763851
PARPBP	1.0057682772	0.9897441132	0.9999763851
PMCH	-1.0799166688	0.691230467	0.9999763851
IGF1	5.4770877341	0.3746135179	0.9999763851
STAB2	-3.9253679416	0.057276238	0.9999763851
NT5DC3	1.0274719886	0.916105305	0.9999763851
HSP90B1	-1.3835376718	0.0128406001	0.9999763851
C12orf73	-1.1832343782	0.5566733626	0.9999763851
TDG	1.2487174004	0.2029189865	0.9999763851
GLT8D2	3.7152157043	0.1014605005	0.9999763851
HCFC2	1.7347411924	0.2343756956	0.9999763851
NFYB	-1.5496772939	0.0669699932	0.9999763851
TXNRD1	1.1047394712	0.4427414818	0.9999763851
CHST11	-1.0946474727	0.5565379744	0.9999763851
SLC41A2	1.3502752571	0.6713058002	0.9999763851
C12orf45	-1.1060650023	0.700456703	0.9999763851
ALDH1L2	9.0182935529	0.2609690465	0.9999763851

KIAA1033	1.2605271666	0.3660854302	0.9999763851
APPL2	1.0978401185	0.7858816265	0.9999763851
C12orf75	1.2222459419	0.2783078122	0.9999763851
CKAP4	1.0717099893	0.609348928	0.9999763851
TCP11L2	5.4781708352	0.3745527127	0.9999763851
POLR3B	-1.2960787455	0.3371190281	0.9999763851
RFX4	-1.315303824	0.8206171563	0.9999763851
RIC8B	1.3878896577	0.4715255896	0.9999763851
TMEM263	-1.6311976455	0.1008366958	0.9999763851
MTERF2	-1.1551770576	0.7825692367	0.9999763851
CRY1	1.0319839448	0.9030242979	0.9999763851
BTBD11	1.0187933879	0.961267209	0.9999763851
PWP1	1.1453893163	0.4790782747	0.9999763851
PRDM4	-1.012037051	0.9604775799	0.9999763851
WSCD2	0	0	0
FICD	1.8134439204	0.5785866709	0.9999763851
SART3	-1.0951297196	0.6336299882	0.9999763851
ISCU	1.0672279869	0.7642579399	0.9999763851
SELPLG	-1.0369584988	0.8367484486	0.9999763851
CORO1C	-1.1209327294	0.5810036114	0.9999763851
SSH1	-1.1009962177	0.7761576304	0.9999763851
USP30	-1.4422318051	0.3485687063	0.9999763851
ALKBH2	1.1183908697	0.7113590959	0.9999763851
UNG	-1.0871539234	0.6014385169	0.9999763851
ACACB	1.0884716508	0.7372743599	0.9999763851
FOXN4	1.8139617088	0.5795007922	0.9999763851
MYO1H	-1.3154339304	0.8205576571	0.9999763851
KCTD10	1.0410285939	0.8397078125	0.9999763851
UBE3B	-1.3429483816	0.2109646488	0.9999763851
MMAB	1.1228686272	0.5937001865	0.9999763851
MVK	1.0410147721	0.869302523	0.9999763851
FAM222A	1.1618338782	0.7127844588	0.9999763851
GLTP	-1.3191119898	0.1554094666	0.9999763851
TCHP	-1.0264093798	0.926849083	0.9999763851
GIT2	1.3374276907	0.2080345625	0.9999763851
ANKRD13A	1.0041735482	0.9839454475	0.9999763851
C12orf76	1.4286150572	0.3980598449	0.9999763851
IFT81	-1.0855005302	0.8789912013	0.9999763851
ATP2A2	1.2481138143	0.1192868588	0.9999763851
ANAPC7	1.1176796956	0.5696110091	0.9999763851
ARPC3	-1.5145216783	0.006513	0.9999763851
GPN3	-1.0448127287	0.8584853047	0.9999763851
FAM216A	-1.0690174217	0.6456271283	0.9999763851
VPS29	1.0443776619	0.8154696192	0.9999763851
RAD9B	-1.1608121394	0.5428138779	0.9999763851
PPTC7	-3.3295429242	0.2520233306	0.9999763851
TCTN1	-1.0763452518	0.8848488471	0.9999763851
HVCN1	1.4500352271	0.5019378363	0.9999763851
PPP1CC	-1.0764171224	0.5859565	0.9999763851

CCDC63	0	0	0
CUX2	1.0836209381	0.7374389153	0.9999763851
FAM109A	-1.0368101062	0.9376592811	0.9999763851
SH2B3	-1.0144670007	0.9500456182	0.9999763851
ATXN2	1.1144554766	0.6043080282	0.9999763851
BRAP	2.2325830412	0.0324054358	0.9999763851
ACAD10	-1.0052070851	0.9888218496	0.9999763851
ALDH2	1.8142404796	0.56760804	0.9999763851
MAPKAPK5	-1.0918749052	0.7285806186	0.9999763851
TMEM116	-1.157574336	0.7091335357	0.9999763851
ERP29	1.0902688123	0.4962121647	0.9999763851
NAA25	1.0402846135	0.87424391	0.9999763851
TRAFD1	1.2372833671	0.5658177748	0.9999763851
HECTD4	-1.1349790134	0.642618607	0.9999763851
RPL6	1.0597824479	0.6227854658	0.9999763851
PTPN11	1.1460229687	0.5122241492	0.9999763851
OAS1	-4.7786096117	0.00586	0.9999763851
OAS3	-1.2557903015	0.5052812826	0.9999763851
OAS2	-2.4031966624	0.1541753007	0.9999763851
RASAL1	0	0	0
CFAP73	-1.0481261601	0.7500331978	0.9999763851
DDX54	-1.2497188948	0.6323760728	0.9999763851
RITA1	-1.0680559507	0.7541438809	0.9999763851
IQCD	1.0275875807	0.9774412141	0.9999763851
TPCN1	1.7422300925	0.0799334549	0.9999763851
SLC8B1	1.2819157687	0.2626933217	0.9999763851
PLBD2	1.2823000855	0.3587497204	0.9999763851
SDSL	2.0638935339	0.254686542	0.9999763851
RBM19	-1.0556518783	0.8271309921	0.9999763851
MED13L	-1.2404714034	0.2254952353	0.9999763851
MAP1LC3B2	-1.6140694975	0.4640180831	0.9999763851
C12orf49	-1.0245979387	0.8809297881	0.9999763851
RNFT2	-1.0628405718	0.9007521036	0.9999763851
HRK	-1.1069884702	0.8174866028	0.9999763851
FBXW8	-1.2763875162	0.3122437392	0.9999763851
TESC	1.2001886431	0.1696135094	0.9999763851
FBXO21	-1.7282325433	0.114830198	0.9999763851
NOS1	0	0	0
RFC5	1.0203532753	0.9011063185	0.9999763851
WSB2	-1.7085584318	0.2506228776	0.9999763851
VSIG10	1.503528631	0.1676503262	0.9999763851
PEBP1	1.0892677477	0.4614154807	0.9999763851
TAOK3	1.1575928138	0.2192893245	0.9999763851
SUDS3	-2.1039798632	0.0201684054	0.9999763851
SRRM4	0	0	0
TMEM233	0	0	0
PRKAB1	1.0141263359	0.9598232867	0.9999763851
CIT	1.1823059654	0.5104306755	0.9999763851
BICDL1	-1.026798645	0.9628770982	0.9999763851

RAB35	-1.1366364673	0.4510806144	0.9999763851
GCN1	1.138519209	0.3815926602	0.9999763851
RPLP0	1.0813457709	0.5189791923	0.9999763851
PXN	-1.4466819936	0.0708873162	0.9999763851
SIRT4	-1.0806648985	0.9257079518	0.9999763851
PLA2G1B	-7.5622178268	0.2177145568	0.9999763851
MSI1	3.2356557963	0.5547505991	0.9999763851
COX6A1	1.2219243676	0.1359002484	0.9999763851
TRIAP1	-1.190675255	0.2137084274	0.9999763851
GATC	1.6117798265	0.2679978835	0.9999763851
SRSF9	-1.1875976429	0.1840011289	0.9999763851
DYNLL1	1.1645600498	0.214676498	0.9999763851
COQ5	-1.1452480954	0.479100134	0.9999763851
RNF10	-1.0523059729	0.740277968	0.9999763851
POP5	1.4117881067	0.109476915	0.9999763851
CABP1	1.027554661	0.975654119	0.9999763851
MLEC	-1.0416825998	0.7839491997	0.9999763851
UNC119B	1.2424415035	0.5165805344	0.9999763851
ACADS	-1.2933471009	0.1892659263	0.9999763851
SPPL3	1.225645404	0.3709628013	0.9999763851
HNF1A	1.6236002864	0.5936648968	0.9999763851
C12orf43	-1.2017092396	0.5050439407	0.9999763851
OASL	-14.7252461469	0.0978119437	0.9999763851
P2RX7	1.5159317688	0.2988363527	0.9999763851
P2RX4	-1.1541456714	0.6218300844	0.9999763851
CAMKK2	1.0161620368	0.9260761685	0.9999763851
ANAPC5	1.2681176469	0.1081127059	0.9999763851
RNF34	1.101646536	0.6038113897	0.9999763851
KDM2B	1.0607665261	0.754714381	0.9999763851
ORAI1	-1.0305119758	0.8753577549	0.9999763851
MORN3	3.2353357581	0.554787377	0.9999763851
TMEM120B	-1.090584448	0.7774021473	0.9999763851
RHOF	1.9067530143	0.002617	0.9999763851
SETD1B	-1.0516260059	0.775411655	0.9999763851
HPD	2.5371732676	0.2688076764	0.9999763851
PSMD9	1.0214233139	0.8847302376	0.9999763851
WDR66	-3.5004921496	0.1012598367	0.9999763851
BCL7A	1.1789171499	0.5574991189	0.9999763851
MLXIP	-1.0970278948	0.6339495902	0.9999763851
LRRC43	7.7211724672	0.2779709379	0.9999763851
B3GNT4	1.1884456013	0.3306919682	0.9999763851
DIABLO	1.0549969765	0.8897010051	0.9999763851
VPS33A	1.1535425065	0.5839633502	0.9999763851
CLIP1	1.1983501327	0.5626449986	0.9999763851
ZCCHC8	-1.2612930663	0.3918867923	0.9999763851
RSRC2	-1.347470579	0.0971267375	0.9999763851
KNTC1	-1.0862933446	0.7829949159	0.9999763851
HCAR2	0	0	0
DENR	-1.0428348499	0.7957657472	0.9999763851

CCDC62	-2.0624499785	0.3046934717	0.9999763851
HIP1R	1.0798895836	0.8002348375	0.9999763851
VPS37B	1.1004919326	0.7641276814	0.9999763851
ABCB9	-1.246996882	0.6156584494	0.9999763851
OGFOD2	1.4588583651	0.2001928054	0.9999763851
ARL6IP4	1.0843644845	0.567252205	0.9999763851
PITPNM2	-1.1364685749	0.7981193492	0.9999763851
MPHOSPH9	1.195495662	0.3326091313	0.9999763851
C12orf65	-1.3579189174	0.1215615475	0.9999763851
CDK2AP1	1.2928593571	0.2573384603	0.9999763851
SBNO1	-1.0937421358	0.615806713	0.9999763851
KMT5A	1.9012661905	0.1217582485	0.9999763851
RILPL2	-1.0836191629	0.5609031408	0.9999763851
SNRNP35	-1.1045077582	0.8043455073	0.9999763851
RILPL1	1.0306012855	0.9665926947	0.9999763851
TMED2	-1.0597390377	0.7568405968	0.9999763851
DDX55	-1.0334437111	0.8410842808	0.9999763851
EIF2B1	1.1899582372	0.2619259105	0.9999763851
GTF2H3	-1.0544547337	0.8613149399	0.9999763851
ATP6V0A2	-1.2761605512	0.3347914284	0.9999763851
DNAH10	-1.415563406	0.4334008684	0.9999763851
CCDC92	1.0622977031	0.8356107745	0.9999763851
ZNF664	1.1909153774	0.2328393866	0.9999763851
ZNF664-FAM101A	-1.491584767	0.295555348	0.9999763851
NCOR2	1.1646795964	0.3749150096	0.9999763851
SCARB1	1.2733310231	0.2787266884	0.9999763851
UBC	1.0807084084	0.5879865337	0.9999763851
DHX37	1.1057595541	0.7360000724	0.9999763851
BRI3BP	-1.2676671308	0.1313234577	0.9999763851
AACS	1.014844671	0.9596813234	0.9999763851
TMEM132B	-6.3085154939	0.001787	0.9999763851
LOC107984465	3.2353357581	0.554787377	0.9999763851
LOC107984449	0	0	0
TMEM132C	0	0	0
SLC15A4	1.2675769726	0.2957534025	0.9999763851
LOC101929984	0	0	0
TMEM132D	-1.3955017315	0.6896676877	0.9999763851
RIMBP2	0	0	0
STX2	1.2591650677	0.4480026073	0.9999763851
RAN	1.0148911031	0.8974478844	0.9999763851
SFSWAP	1.3112729362	0.1337957609	0.9999763851
MMP17	1.1607221631	0.8158246639	0.9999763851
ULK1	-1.0209429437	0.9127082848	0.9999763851
PUS1	1.0411308625	0.847819404	0.9999763851
EP400	1.0134138078	0.9472839842	0.9999763851
DDX51	1.1237782774	0.6001416683	0.9999763851
NOC4L	1.2472114703	0.5183944052	0.9999763851
GALNT9	4.0584869584	0.0356586285	0.9999763851
MUC8	0	0	0

FBRSL1	1.0085281906	0.9682465698	0.9999763851
LRCOL1	7.7488609396	0.3059592464	0.9999763851
P2RX2	0	0	0
POLE	-1.1635256109	0.5728404307	0.9999763851
PXMP2	1.6152919211	0.0433596475	0.9999763851
PGAM5	1.1589002785	0.4614945051	0.9999763851
ANKLE2	1.0865010662	0.669668886	0.9999763851
GOLGA3	-1.0169128737	0.9113980396	0.9999763851
ZNF605	-1.0159154378	0.9783684179	0.9999763851
ZNF26	-1.2310110463	0.5996281408	0.9999763851
ZNF84	-1.1746984042	0.5592761201	0.9999763851
ZNF140	-1.3720072732	0.4144057991	0.9999763851
ZNF891	-1.3151016298	0.8212266933	0.9999763851
ZNF10	1.2869488883	0.8209286526	0.9999763851
ZNF268	1.0994425743	0.8848880562	0.9999763851
TPTE2	-4.2849908816	0.4028453076	0.9999763851
MPHOSPH8	1.1423760863	0.4372619512	0.9999763851
PSPC1	1.145763669	0.4981021878	0.9999763851
ZMYM5	-1.3457828145	0.4997430838	0.9999763851
ZMYM2	-1.4656083347	0.0979763531	0.9999763851
GJA3	1.8136231599	0.5627419009	0.9999763851
GJB2	1.7600058205	0.2482309662	0.9999763851
GJB6	3.2353357581	0.554787377	0.9999763851
CRYL1	1.0884036112	0.7414526211	0.9999763851
IFT88	1.1434559725	0.7547759294	0.9999763851
IL17D	1.126568796	0.7219221313	0.9999763851
EEF1AKMT1	-1.0314719684	0.9123771591	0.9999763851
XPO4	-1.1968115817	0.481090372	0.9999763851
LATS2	1.0218216658	0.9395944826	0.9999763851
SAP18	-1.0437256997	0.7499741727	0.9999763851
SKA3	1.0493857185	0.8138879907	0.9999763851
MRPL57	1.2912938345	0.0887115288	0.9999763851
LOC101928764	3.2361532718	0.5546934395	0.9999763851
ZDHHC20	-1.0556732778	0.7545260836	0.9999763851
MICU2	-1.2192858978	0.4043661341	0.9999763851
FGF9	-4.2864494036	0.4027150576	0.9999763851
SGCG	0	0	0
SACS	1.3620951349	0.0885838447	0.9999763851
TNFRSF19	1.0300362649	0.9688504826	0.9999763851
MIPEP	1.7137045638	0.0386979418	0.9999763851
C1QTNF9B-AS1	2.3839656649	0.225884284	0.9999763851
SPATA13	-1.0974604745	0.7854000248	0.9999763851
PARP4	1.075309685	0.7515271404	0.9999763851
ATP12A	6.368054416	0.349203043	0.9999763851
RNF17	0	0	0
CENPJ	-1.3955890483	0.1926448071	0.9999763851
PABPC3	1.2179591309	0.3001180125	0.9999763851
MTMR6	1.6581580088	0.0182499863	0.9999763851
NUP58	1.1454053591	0.5255336929	0.9999763851

ATP8A2	-1.3233953713	0.7047340731	0.9999763851
SHISA2	1.7928823177	0.0880219463	0.9999763851
RNF6	1.0425678762	0.8824054789	0.9999763851
CDK8	1.1273898284	0.7404569468	0.9999763851
WASF3	-1.4391263345	0.6925013455	0.9999763851
GPR12	0	0	0
USP12	-1.0128593849	0.9518920232	0.9999763851
RPL21	1.1931911716	0.2799459693	0.9999763851
RASL11A	0	0	0
GTF3A	1.2630896172	0.130004301	0.9999763851
MTIF3	1.004947455	0.9842628975	0.9999763851
LNX2	-1.0785835147	0.7077263006	0.9999763851
POLR1D	-1.0099746801	0.9439944002	0.9999763851
PDX1	3.2361532718	0.5546934395	0.9999763851
CDX2	0	0	0
FLT3	-1.3161718537	0.8288271375	0.9999763851
PAN3	-1.2502869806	0.2378163287	0.9999763851
FLT1	1.6702739263	0.4623279285	0.9999763851
POMP	1.0884109285	0.5035844696	0.9999763851
SLC46A3	0	0	0
MTUS2	1.3422974682	0.7193093082	0.9999763851
SLC7A1	1.1517298807	0.4539754429	0.9999763851
UBL3	-1.3737975077	0.1960099766	0.9999763851
KATNAL1	-1.9154154343	0.0628503988	0.9999763851
HMGB1	-1.1235385483	0.3230932378	0.9999763851
USPL1	-1.1395736879	0.6980017986	0.9999763851
ALOX5AP	-1.1246689653	0.4417971728	0.9999763851
MEDAG	0	0	0
HSPH1	1.0630232672	0.6740765585	0.9999763851
B3GLCT	1.0614218676	0.8944605946	0.9999763851
FRY	-1.0880251013	0.754941191	0.9999763851
ZAR1L	-1.3154339304	0.8205576571	0.9999763851
BRCA2	-1.8573895885	0.1218912923	0.9999763851
N4BP2L1	-1.937382965	0.0562632522	0.9999763851
N4BP2L2	1.027866861	0.8811981838	0.9999763851
PDS5B	-1.2165168527	0.3442953778	0.9999763851
KL	0	0	0
STARD13	1.3576108079	0.7957172822	0.9999763851
RFC3	-1.0091083545	0.9657303546	0.9999763851
NBEA	-1.3158816768	0.8203229796	0.9999763851
DCLK1	0	0	0
CCDC169-SOHLH2	-2.3270898917	0.1874348492	0.9999763851
CCDC169	5.4979928157	0.0934985193	0.9999763851
SPG20	-1.1376847162	0.3815221552	0.9999763851
CCNA1	1.1326662498	0.5729201338	0.9999763851
RFXAP	1.0703924237	0.8059306183	0.9999763851
SMAD9	-3.3295429242	0.2519647292	0.9999763851
ALG5	1.2361899045	0.1734213601	0.9999763851
EXOSC8	-1.0149354528	0.9418089327	0.9999763851

SUPT20H	1.1452447525	0.8090056193	0.9999763851
UFM1	-1.3486509972	0.1512987889	0.9999763851
PROSER1	-1.0268141656	0.9147612162	0.9999763851
NHLRC3	-1.2795221109	0.4846602832	0.9999763851
LHFP	-1.3296842173	0.5342529675	0.9999763851
COG6	-1.1462578978	0.7191773116	0.9999763851
FOXO1	1.1945087061	0.5767013586	0.9999763851
MRPS31	1.3930160408	0.1966536592	0.9999763851
SLC25A15	-1.4928849167	0.197944303	0.9999763851
ELF1	-1.4483263347	0.0692645525	0.9999763851
WBP4	-1.2937870376	0.363677588	0.9999763851
KBTBD6	-1.0710159452	0.7512032046	0.9999763851
KBTBD7	-1.770321806	0.1730201304	0.9999763851
MTRF1	1.113847379	0.7558948988	0.9999763851
NAA16	-1.3199689805	0.3588627852	0.9999763851
RGCC	-1.3673305384	0.4401552175	0.9999763851
VWA8	1.314194154	0.3758172082	0.9999763851
DGKH	-1.0082790795	0.9719073785	0.9999763851
AKAP11	1.3702889139	0.3015148917	0.9999763851
EPSTI1	-4.2864494036	0.4027150576	0.9999763851
DNAJC15	-1.1955812749	0.6464561724	0.9999763851
ENOX1	0	0	0
CCDC122	-2.3214980365	0.4368799648	0.9999763851
LACC1	-1.8878800522	0.1713064801	0.9999763851
SERP2	0	0	0
TSC22D1	1.5888029347	0.1137816275	0.9999763851
NUFIP1	1.0162348354	0.9317992587	0.9999763851
GPALPP1	1.0798342924	0.6814382523	0.9999763851
GTF2F2	-1.0625295572	0.708051814	0.9999763851
TPT1	1.0167674024	0.8829407859	0.9999763851
SLC25A30	1.1297420685	0.6989120703	0.9999763851
COG3	-1.4338022674	0.1330826482	0.9999763851
ZC3H13	-1.0444324469	0.7933962088	0.9999763851
CPB2	-10.844600477	0.1422232425	0.9999763851
LCP1	-1.0101167137	0.9366000634	0.9999763851
LRRC63	-2.3877824956	0.2390456433	0.9999763851
LRCH1	1.0082861712	0.9687828568	0.9999763851
ESD	1.1103681811	0.549549017	0.9999763851
SUCLA2	1.7572405781	0.0108544453	0.9999763851
NUDT15	1.2147270957	0.3966575855	0.9999763851
MED4	1.0969401126	0.5340958542	0.9999763851
ITM2B	1.0077968007	0.9549010499	0.9999763851
RB1	-1.1959122532	0.2692407392	0.9999763851
RCBTB2	1.3017810321	0.0707400095	0.9999763851
CYSLTR2	0	0	0
FNDC3A	-1.2089911247	0.3205067299	0.9999763851
CDADC1	-1.0516063636	0.8300852381	0.9999763851
CAB39L	-1.7772896743	0.009375	0.9999763851
SETDB2	1.0849467892	0.7027568597	0.9999763851

SETDB2-PHF11	0	0	0
PHF11	-1.1126545412	0.5845450654	0.9999763851
RCBTB1	-1.0880884245	0.7281816933	0.9999763851
ARL11	-1.1891643579	0.5427997699	0.9999763851
EBPL	-1.0767780729	0.6304190019	0.9999763851
KPNA3	1.0591418317	0.7214188649	0.9999763851
SPRYD7	1.1720926153	0.4444115968	0.9999763851
TRIM13	-1.0846376079	0.6482651794	0.9999763851
KCNRG	-1.0800633574	0.7967473884	0.9999763851
DLEU7	1.276674184	0.505066503	0.9999763851
RNASEH2B	1.1982174179	0.2157644497	0.9999763851
FAM124A	-1.9197578446	0.1150025293	0.9999763851
SERPINE3	-7.5622178268	0.2177145568	0.9999763851
INTS6	1.2695153484	0.1950715216	0.9999763851
WDFY2	-1.013445227	0.9470864202	0.9999763851
DHRS12	1.250998045	0.5111871576	0.9999763851
CCDC70	-7.5622178268	0.2177145568	0.9999763851
ATP7B	1.3593776586	0.5989975472	0.9999763851
ALG11	-1.217570241	0.5957587584	0.9999763851
UTP14C	1.0456607091	0.8293307722	0.9999763851
LOC107984562	-6.7239400998	0.0177063447	0.9999763851
NEK5	-4.2849908816	0.4028453076	0.9999763851
NEK3	-1.0503595166	0.8544249519	0.9999763851
THSD1	1.9436648833	0.1470473319	0.9999763851
VPS36	-1.1953617845	0.3471006689	0.9999763851
CKAP2	1.0764817955	0.6481334293	0.9999763851
HNRNPA1L2	1.0703167922	0.7884464097	0.9999763851
SUGT1	1.1252938069	0.4254095138	0.9999763851
LECT1	7.7192231741	0.2782707334	0.9999763851
OLFM4	0	0	0
DIAPH3	1.0390231012	0.8174100331	0.9999763851
TDRD3	1.2801490406	0.3640315375	0.9999763851
MZT1	1.067831973	0.6849034664	0.9999763851
BORA	-1.2841586617	0.3195032063	0.9999763851
DIS3	1.089589204	0.5174703835	0.9999763851
PIBF1	-1.2015293973	0.4481151845	0.9999763851
KLF5	-2.2051397677	0.1670732594	0.9999763851
KLF12	0	0	0
TBC1D4	-1.2806176768	0.2068507237	0.9999763851
COMMD6	-1.2265628773	0.1270575063	0.9999763851
UCHL3	1.5125917048	0.00455	0.9999763851
LMO7	-1.4009298347	0.1516180504	0.9999763851
KCTD12	-4.2849908816	0.4028453076	0.9999763851
ACOD1	-1.3715126673	0.7507171393	0.9999763851
CLN5	1.7772431003	0.0147107803	0.9999763851
FBXL3	1.0789760474	0.672435692	0.9999763851
MYCBP2	1.0940161879	0.4857284112	0.9999763851
SCEL	0	0	0
SLAIN1	1.5099452149	0.5429892632	0.9999763851

LOC107987188	-1.5020071852	0.3209561268	0.9999763851
POU4F1	1.3320004752	0.7386776771	0.9999763851
RNF219	1.2770512181	0.1116327523	0.9999763851
RBM26	1.1738165351	0.3042246082	0.9999763851
NDFIP2	-1.4072122324	0.6699289508	0.9999763851
SPRY2	1.3935957345	0.2579501105	0.9999763851
GPC6	-2.5608753354	0.262256475	0.9999763851
DCT	-3.3295429242	0.2518232852	0.9999763851
TGDS	-1.2847157431	0.4615239888	0.9999763851
GPR180	-1.1608669276	0.5143063616	0.9999763851
ABCC4	-1.1115315794	0.6464088186	0.9999763851
CLDN10	-1.315303824	0.8206171563	0.9999763851
DZIP1	1.3260278971	0.7357146261	0.9999763851
DNAJC3	-1.138293383	0.439974785	0.9999763851
UGGT2	-1.1363688399	0.6404466807	0.9999763851
HS6ST3	-4.2864494036	0.4027150576	0.9999763851
MBNL2	-1.3951205161	0.694452287	0.9999763851
RAP2A	-1.1256573413	0.581430869	0.9999763851
IPO5	1.0238008028	0.8777670836	0.9999763851
FARP1	1.2867653957	0.8300426537	0.9999763851
STK24	1.0470901044	0.7590561231	0.9999763851
SLC15A1	3.2353357581	0.554787377	0.9999763851
DOCK9	-1.6634732451	0.1271076274	0.9999763851
UBAC2	1.0327717731	0.8475389058	0.9999763851
GPR18	1.9286482047	0.2100163897	0.9999763851
GPR183	-1.202523987	0.6225277988	0.9999763851
TM9SF2	1.0198308211	0.9040258773	0.9999763851
CLYBL	1.1737421692	0.539826704	0.9999763851
PCCA	-1.1810196669	0.3854033594	0.9999763851
GGACT	1.2310267785	0.1343875497	0.9999763851
TMTC4	1.0240005833	0.900166536	0.9999763851
ITGBL1	-13.1048806834	0.1324481376	0.9999763851
FGF14	-1.9660821036	0.4622956112	0.9999763851
TPP2	1.1522778798	0.4703453159	0.9999763851
TEX30	1.705862946	0.0629855538	0.9999763851
BIVM-ERCC5	18.9271138591	0.1177496117	0.9999763851
ERCC5	-1.2634201006	0.3622742119	0.9999763851
EFNB2	9.9604381148	0.2218973023	0.9999763851
ARGLU1	-1.1576207106	0.3334622764	0.9999763851
FAM155A	1.2718841722	0.7771465425	0.9999763851
LIG4	1.8974116751	0.2923483741	0.9999763851
ABHD13	1.2521208762	0.480551666	0.9999763851
TNFSF13B	1.127484244	0.3421445943	0.9999763851
MYO16	1.1526791883	0.7910763709	0.9999763851
IRS2	1.0363210839	0.7780630521	0.9999763851
COL4A1	3.4147527346	0.1247694125	0.9999763851
COL4A2	-1.0682221617	0.6889316246	0.9999763851
RAB20	1.1246744277	0.4434178406	0.9999763851
NAXD	-1.208052497	0.5721950269	0.9999763851

CARS2	1.1625313792	0.3056443968	0.9999763851
LOC105376684	-4.2864494036	0.4027150576	0.9999763851
ING1	1.3896920299	0.2144012331	0.9999763851
LOC105370362	3.2361532718	0.5546934395	0.9999763851
ANKRD10	-1.1372659035	0.4599805023	0.9999763851
ARHGEF7	1.6052778367	0.0258682061	0.9999763851
TEX29	-1.3710462586	0.7622082741	0.9999763851
SPACA7	3.2353357581	0.554787377	0.9999763851
TUBGCP3	-1.3367834488	0.0500123711	0.9999763851
ATP11AUN	3.2353357581	0.554787377	0.9999763851
ATP11A	-1.2351865397	0.29494355	0.9999763851
MCF2L	-1.3154339304	0.8205576571	0.9999763851
F10	2.5202262643	0.2771054268	0.9999763851
LOC102724474	5.4776292235	0.3745831165	0.9999763851
PROZ	-1.3721150467	0.7444453804	0.9999763851
PCID2	1.0130262065	0.9394839179	0.9999763851
CUL4A	-1.1707755843	0.3046859359	0.9999763851
LAMP1	-1.0532020886	0.6714159566	0.9999763851
GRTP1	2.8037355282	0.1605777343	0.9999763851
LOC101928841	1.8139019215	0.5819978038	0.9999763851
ADPRHL1	1.5493286353	0.556702986	0.9999763851
DCUN1D2	-1.6044120687	0.1583964223	0.9999763851
TMCO3	1.0346779978	0.8949365969	0.9999763851
TFDP1	-1.2002953361	0.1523776575	0.9999763851
ATP4B	0	0	0
LOC105377805	1.8131652995	0.5810759404	0.9999763851
TMEM255B	-1.1404788704	0.7098194	0.9999763851
GAS6	1.1124412839	0.8250699041	0.9999763851
LINC00452	0	0	0
RASA3	-1.027898279	0.8496882704	0.9999763851
LOC101929355	3.2353357581	0.554787377	0.9999763851
CDC16	1.2208437067	0.3065209345	0.9999763851
UPF3A	-1.1128576611	0.5566860618	0.9999763851
CHAMP1	1.4230506103	0.1140013589	0.9999763851
TTC5	1.3691429619	0.2866337295	0.9999763851
CCNB1IP1	-1.1722109164	0.4963152086	0.9999763851
PARP2	-1.4978774039	0.241503862	0.9999763851
TEP1	-1.2209578689	0.6677290967	0.9999763851
KLHL33	0	0	0
OSGEP	-1.9134730607	0.0199619857	0.9999763851
APEX1	-1.1768784529	0.4858985716	0.9999763851
TMEM55B	-1.4496322545	0.1345557388	0.9999763851
PNP	1.4207030268	0.005714	0.9999763851
RNASE11	-1.4167465726	0.6306109352	0.9999763851
ANG	1.4562362196	0.6629487923	0.9999763851
RNASE4	-3.3704537804	0.0730760137	0.9999763851
RNASE6	0	0	0
RNASE1	0	0	0
RNASE3	1.0138863098	0.946175862	0.9999763851

RNASE2	1.1527565413	0.3331128879	0.9999763851
METTL17	1.0702560619	0.7526430115	0.9999763851
NDRG2	0	0	0
ARHGEF40	-1.0807630082	0.8016790503	0.9999763851
ZNF219	-1.1477187905	0.6931031218	0.9999763851
TMEM253	0	0	0
HNRNPC	1.0890903032	0.4712844896	0.9999763851
RPGRIP1	1.5497553448	0.5828862717	0.9999763851
SUPT16H	-1.2045469432	0.189072318	0.9999763851
CHD8	-1.2105311477	0.4783325129	0.9999763851
RAB2B	-1.0606772872	0.8403229304	0.9999763851
TOX4	1.0426880755	0.8158640679	0.9999763851
METTL3	-1.1459068635	0.7176043985	0.9999763851
DAD1	1.0571415312	0.7236253161	0.9999763851
ABHD4	-2.0107465889	0.1983251074	0.9999763851
OXA1L	1.3656499798	0.0982982244	0.9999763851
SLC7A7	-3.1926989958	0.009916	0.9999763851
MRPL52	1.0241282711	0.867682537	0.9999763851
MMP14	-7.5622178268	0.2177145568	0.9999763851
LRP10	-1.1204316341	0.6282036435	0.9999763851
REM2	5.4770877341	0.3746135179	0.9999763851
RBM23	-1.0730725531	0.8034091347	0.9999763851
PRMT5	1.1037018694	0.5430444404	0.9999763851
HAUS4	1.1222409733	0.6036359946	0.9999763851
AJUBA	-1.0848090731	0.8949558776	0.9999763851
C14orf93	4.9781511567	0.1090416082	0.9999763851
PSMB5	-1.0539860337	0.7485843686	0.9999763851
CDH24	1.1333354589	0.8697279169	0.9999763851
LOC105370705	0	0	0
ACIN1	-1.1065482002	0.6265992053	0.9999763851
C14orf119	1.2335039717	0.2567110236	0.9999763851
CEBPE	1.3580658612	0.0697396595	0.9999763851
SLC7A8	1.5848328097	0.1932995148	0.9999763851
RNF212B	-3.3288155965	0.2424150695	0.9999763851
HOMEZ	-1.2606665903	0.5594715839	0.9999763851
PPP1R3E	-1.6083734375	0.1389760744	0.9999763851
BCL2L2	-2.3214980365	0.4369965837	0.9999763851
BCL2L2-PABPN1	-1.5833626518	0.6002463987	0.9999763851
PABPN1	1.1462970993	0.4151708635	0.9999763851
NGDN	-1.201163443	0.234509199	0.9999763851
THTPA	-1.3158393667	0.8279642648	0.9999763851
ZFHX2	1.2870761236	0.8160709833	0.9999763851
AP1G2	-1.0198206672	0.9500443818	0.9999763851
DHRS4	1.0947700809	0.6119871589	0.9999763851
DHRS4L2	1.1783128022	0.6101609051	0.9999763851
DHRS4L1	1.0268535045	0.9784766606	0.9999763851
NRL	3.7126144389	0.1266038921	0.9999763851
PCK2	-1.1165025049	0.6604029116	0.9999763851
DCAF11	-1.3107333445	0.093686061	0.9999763851

FITM1	3.2353357581	0.554787377	0.9999763851
PSME1	1.5007227872	0.0152458755	0.9999763851
EMC9	-1.309406502	0.4731994394	0.9999763851
PSME2	1.2594192156	0.1739214409	0.9999763851
RNF31	-1.1070836643	0.8119518588	0.9999763851
IRF9	3.3716563396	0.2594998905	0.9999763851
REC8	1.0058242696	0.9918215703	0.9999763851
IPO4	1.1158617614	0.6974426322	0.9999763851
TM9SF1	-1.2361716764	0.3985835177	0.9999763851
TSSK4	3.2353357581	0.554787377	0.9999763851
CHMP4A	1.313620252	0.1285289898	0.9999763851
MDP1	-1.2398810344	0.7239925434	0.9999763851
NEDD8-MDP1	-3.1011527746	0.0547646753	0.9999763851
NEDD8	1.0319351712	0.8497598073	0.9999763851
GMPR2	-1.1917603061	0.3219898871	0.9999763851
TINF2	1.9767289733	0.007579	0.9999763851
TGM1	1.3421649772	0.7036281394	0.9999763851
RABGGTA	1.0116223261	0.9733368601	0.9999763851
DHRS1	-1.0098810639	0.9471036536	0.9999763851
NOP9	1.1535378754	0.5453141247	0.9999763851
CIDEB	1.5503890537	0.1526643051	0.9999763851
LTB4R	-1.0497563294	0.7852464657	0.9999763851
ADCY4	1.0319749808	0.951164983	0.9999763851
RIPK3	1.2241909054	0.7566147316	0.9999763851
KHNYN	-1.1472914366	0.7167256897	0.9999763851
CBLN3	1.0272952748	0.9775286953	0.9999763851
SDR39U1	1.1750370259	0.4042078615	0.9999763851
CMA1	1.2869488883	0.8209826444	0.9999763851
CTSG	-1.1490343211	0.3023759425	0.9999763851
GZMB	3.2356557963	0.5547505991	0.9999763851
STXBP6	3.2356557963	0.5547505991	0.9999763851
PRKD1	0	0	0
G2E3	1.1677360944	0.4900317109	0.9999763851
SCFD1	1.2069165106	0.4730583567	0.9999763851
COCH	0	0	0
STRN3	1.1246066681	0.5453176177	0.9999763851
AP4S1	1.6141925788	0.4103037139	0.9999763851
HECTD1	-1.0209899056	0.9210810726	0.9999763851
HEATR5A	-1.3247786941	0.4298367639	0.9999763851
DTD2	1.4213972935	0.1072269762	0.9999763851
NUBPL	1.0264058989	0.9153675379	0.9999763851
ARHGAP5	-1.9393868411	0.0694479439	0.9999763851
AKAP6	1.5501834829	0.2836250088	0.9999763851
SPTSSA	-1.1004679569	0.6136804115	0.9999763851
EAPP	-1.1997132288	0.4493304483	0.9999763851
SNX6	1.3842227211	0.0444905077	0.9999763851
CFL2	1.3672691571	0.5165783555	0.9999763851
BAZ1A	1.0501471126	0.8101214168	0.9999763851
SRP54	-1.1320106821	0.558227719	0.9999763851

FAM177A1	1.0863932551	0.7801205452	0.9999763851
PPP2R3C	1.1996428849	0.4130826932	0.9999763851
KIAA0391	-1.1441982339	0.5883429011	0.9999763851
PSMA6	1.0862837101	0.5875886124	0.9999763851
NFKBIA	-1.2800045647	0.1035756334	0.9999763851
RALGAPA1	-1.0447196859	0.898346144	0.9999763851
BRMS1L	1.1525850614	0.7602440885	0.9999763851
MBIP	-2.1294557464	0.1039780616	0.9999763851
SEC23A	-1.0765067747	0.7597682469	0.9999763851
GEMIN2	-1.5451294964	0.3654252861	0.9999763851
TRAPPC6B	1.6667841079	0.1314481423	0.9999763851
PNN	-1.0578444442	0.7489333966	0.9999763851
MIA2	0	0	0
CTAGE5	-1.2740539157	0.3412698915	0.9999763851
FBXO33	1.1003612957	0.6947338477	0.9999763851
LRFN5	0	0	0
C14orf28	-1.7332721092	0.4777599329	0.9999763851
KLHL28	-1.702782182	0.1455679255	0.9999763851
FAM179B	-1.0583554569	0.9135167201	0.9999763851
PRPF39	1.0346199249	0.8926827061	0.9999763851
FKBP3	1.2537137095	0.3058901387	0.9999763851
FANCM	-1.4329170107	0.510622977	0.9999763851
MIS18BP1	-1.2379865672	0.2601157231	0.9999763851
RPL10L	0	0	0
RPS29	-1.1800301485	0.1643516854	0.9999763851
LRR1	1.0331292858	0.8330660597	0.9999763851
RPL36AL	1.0231039924	0.863854277	0.9999763851
MGAT2	-1.5207716133	0.1051578036	0.9999763851
DNAAF2	-1.2794884001	0.465138609	0.9999763851
POLE2	-1.2999820927	0.3303263415	0.9999763851
KLHDC1	-1.7016113555	0.5931451629	0.9999763851
KLHDC2	-1.0023968826	0.9909206364	0.9999763851
NEMF	1.3905846548	0.1313459178	0.9999763851
ARF6	1.0917989334	0.5027545646	0.9999763851
VCPKMT	1.6314611786	0.1700931061	0.9999763851
SOS2	-1.4720055868	0.1299994561	0.9999763851
L2HGDH	-1.1647574798	0.6506114682	0.9999763851
ATP5S	1.4953438031	0.1780203159	0.9999763851
CDKL1	-1.3226524418	0.5085876018	0.9999763851
MAP4K5	-1.0321679061	0.9245985775	0.9999763851
ATL1	2.5893856813	0.1954557046	0.9999763851
SAV1	-1.2657156961	0.6556004234	0.9999763851
NIN	1.0687392552	0.7124274438	0.9999763851
ABHD12B	1.5501971516	0.5511432317	0.9999763851
PYGL	1.0765112361	0.6879872194	0.9999763851
TRIM9	-1.1820732547	0.7637413137	0.9999763851
TMX1	1.2518879514	0.1862764341	0.9999763851
FRMD6	-3.3284109188	0.2428427922	0.9999763851
GNG2	1.511213481	0.0409084038	0.9999763851

C14orf166	1.2148103366	0.1916802534	0.9999763851
NID2	-1.1243143183	0.8470262754	0.9999763851
PTGER2	-1.1213042985	0.6335665291	0.9999763851
TXNDC16	-1.7222722635	0.1494111666	0.9999763851
GPR137C	9.9624059448	0.2314356309	0.9999763851
ERO1A	1.0201846462	0.900966848	0.9999763851
PSMC6	1.0693474516	0.6709955777	0.9999763851
STYX	1.007908665	0.9842396563	0.9999763851
GNPNAT1	1.0021613767	0.9930641806	0.9999763851
DDHD1	-1.2316149889	0.4385556438	0.9999763851
CDKN3	1.0240424827	0.8908720197	0.9999763851
CNIH1	-1.1436271977	0.4242379924	0.9999763851
GMFB	1.4997469069	0.0546788206	0.9999763851
CGRRF1	2.0277299818	0.1423376925	0.9999763851
SAMD4A	-1.0807623952	0.9264695345	0.9999763851
GCH1	1.2626044358	0.3008476076	0.9999763851
WDHD1	1.335243219	0.2501600254	0.9999763851
SOCS4	-1.1951072689	0.5251057439	0.9999763851
MAPK1IP1L	1.1776942056	0.3267971866	0.9999763851
LGALS3	-1.1634326155	0.498469466	0.9999763851
DLGAP5	-1.2691218387	0.2803533398	0.9999763851
FBXO34	-1.5614029831	0.2135085909	0.9999763851
ATG14	-1.8127308044	0.1757977154	0.9999763851
TBPL2	3.2361532718	0.5546934395	0.9999763851
KTN1	1.3075799796	0.06729228	0.9999763851
PELI2	-1.428657825	0.373030472	0.9999763851
TMEM260	-1.0297308187	0.9352899765	0.9999763851
EXOC5	1.4539583699	0.0885441514	0.9999763851
AP5M1	1.5782605759	0.1269109295	0.9999763851
NAA30	1.4356543638	0.2351201898	0.9999763851
C14orf37	0	0	0
ACTR10	1.1511706914	0.4606048501	0.9999763851
PSMA3	1.0234588834	0.9011701073	0.9999763851
ARID4A	1.3329057256	0.2468966278	0.9999763851
TOMM20L	-7.9575607668	0.0002467	0.3161474386
TIMM9	1.6813332878	0.0108531779	0.9999763851
KIAA0586	1.0227249762	0.9198227172	0.9999763851
DACT1	-4.2849908816	0.4028453076	0.9999763851
DAAM1	1.4143009003	0.3616523408	0.9999763851
GPR135	-1.8158646428	0.4740903721	0.9999763851
L3HYPDH	-1.1363427917	0.7769987364	0.9999763851
JKAMP	1.0478567807	0.875379534	0.9999763851
RTN1	0	0	0
PCNX4	-1.0904900687	0.750827833	0.9999763851
DHRS7	-1.5118023216	0.0354534664	0.9999763851
PPM1A	-1.1754685973	0.4437331243	0.9999763851
LOC105378189	0	0	0
MNAT1	-2.0055683193	0.0942932127	0.9999763851
TRMT5	1.9031476545	0.004128	0.9999763851

SLC38A6	-1.6401961624	0.2690018137	0.9999763851
PRKCH	3.2356557963	0.5547505991	0.9999763851
HIF1A	-1.1518373097	0.5931302381	0.9999763851
SNAPC1	-1.0749243009	0.8622463986	0.9999763851
KCNH5	0	0	0
RHOJ	0	0	0
GPHB5	0	0	0
PPP2R5E	1.2076049774	0.3539292112	0.9999763851
WDR89	1.0629801063	0.7831745382	0.9999763851
SYNE2	-1.203081765	0.7269933713	0.9999763851
MTHFD1	1.1020669887	0.8616357511	0.9999763851
ZBTB25	-1.0569206728	0.7313549487	0.9999763851
ZBTB1	1.0124752921	0.962031826	0.9999763851
HSPA2	-1.3157515262	0.8224035558	0.9999763851
PPP1R36	-4.2864494036	0.4027150576	0.9999763851
PLEKHG3	-1.185778748	0.7418801732	0.9999763851
SPTB	2.0270981464	0.1179245092	0.9999763851
CHURC1	-1.1206877067	0.4819131192	0.9999763851
CHURC1-FNTB	1.0407242355	0.9323516262	0.9999763851
GPX2	-4.2849908816	0.4028453076	0.9999763851
FNTB	-1.0407883986	0.9419681682	0.9999763851
MAX	-1.1461162834	0.281107642	0.9999763851
FUT8	-1.3178189483	0.6015254756	0.9999763851
GPHN	-1.3357278221	0.2903238619	0.9999763851
MPP5	1.0417389841	0.8688580388	0.9999763851
ATP6V1D	-1.3169916224	0.1662882389	0.9999763851
EIF2S1	1.1362322023	0.3895203774	0.9999763851
TMEM229B	1.1892052954	0.423430025	0.9999763851
PLEKHH1	3.3925476883	0.2301995251	0.9999763851
PIGH	1.4679150796	0.2307880839	0.9999763851
ARG2	1.2478968186	0.3234753689	0.9999763851
VTI1B	-1.750250261	0.1521541198	0.9999763851
RDH11	-1.1391097737	0.4629414406	0.9999763851
RDH12	-1.3944607594	0.6978930708	0.9999763851
ZFYVE26	1.0038295049	0.9872274157	0.9999763851
RAD51B	-1.1705912028	0.5650171054	0.9999763851
ZFP36L1	-3.1662181208	0.003627	0.9999763851
ACTN1	-1.0456842589	0.7366392887	0.9999763851
DCAF5	-1.2535287924	0.2833305957	0.9999763851
EXD2	-1.0359312355	0.9576186513	0.9999763851
GALNT16	7.7204093521	0.2878634691	0.9999763851
ERH	1.0162091537	0.8971454522	0.9999763851
SLC39A9	-1.3635425342	0.1150242361	0.9999763851
SUSD6	1.2157404572	0.4100785312	0.9999763851
SRSF5	-1.0330865123	0.8219109625	0.9999763851
SMOC1	1.0206841205	0.8686086455	0.9999763851
SLC8A3	0	0	0
COX16	1.2054760487	0.5647951731	0.9999763851
SYNJ2BP	0	0	0

ADAM20	3.2353357581	0.554787377	0.9999763851
MED6	1.0199759989	0.9250893073	0.9999763851
TTC9	-1.0804548487	0.7631158124	0.9999763851
MAP3K9	1.0968516109	0.8800685302	0.9999763851
PCNX1	1.1253050877	0.5214364492	0.9999763851
SIPA1L1	-1.1369142867	0.6876653804	0.9999763851
RGS6	-4.287394647	0.4026306792	0.9999763851
DCAF4	-1.4415258552	0.4021861259	0.9999763851
ZFYVE1	-1.445019819	0.1656985214	0.9999763851
RBM25	1.069422282	0.6971617675	0.9999763851
PSEN1	-1.1293259948	0.527924022	0.9999763851
PAPLN	-1.3077628127	0.5117873797	0.9999763851
NUMB	-1.1911377986	0.3987313101	0.9999763851
HEATR4	-3.1563891683	0.1984612855	0.9999763851
C14orf169	1.3049487892	0.1284646981	0.9999763851
ACOT1	3.2353357581	0.554787377	0.9999763851
ACOT4	0	0	0
DNAL1	1.4855176074	0.5232243796	0.9999763851
PNMA1	-2.4128397145	0.0391017463	0.9999763851
ELMSAN1	-1.0818943607	0.7436330002	0.9999763851
PTGR2	-1.1126938801	0.8229495989	0.9999763851
ZNF410	1.1286188094	0.7560178809	0.9999763851
FAM161B	-1.2987553962	0.4747507686	0.9999763851
COQ6	-1.1483402483	0.8095036785	0.9999763851
ENTPD5	1.225790229	0.7596250559	0.9999763851
BBOF1	2.4644821133	0.2157217278	0.9999763851
ALDH6A1	-1.9917023999	0.009553	0.9999763851
LIN52	-1.1530202597	0.563991113	0.9999763851
ABCD4	1.5941751628	0.2482873459	0.9999763851
SYNDIG1L	-1.966276531	0.4562605965	0.9999763851
NPC2	-1.1667725453	0.3030871551	0.9999763851
ISCA2	-1.1642555862	0.4970732755	0.9999763851
LTBP2	-1.4078779033	0.6705865709	0.9999763851
AREL1	-1.2662499317	0.3852683626	0.9999763851
FCF1	1.4298848901	0.0271950435	0.9999763851
YLPM1	1.3849210421	0.0669287796	0.9999763851
PROX2	-3.5515374526	0.0239735744	0.9999763851
DLST	1.2137455894	0.4689433068	0.9999763851
RPS6KL1	-7.5622178268	0.2177145568	0.9999763851
PGF	3.2361532718	0.5546934395	0.9999763851
EIF2B2	1.2704114302	0.2687437385	0.9999763851
ACYP1	1.1043386127	0.7389587014	0.9999763851
ZC2HC1C	0	0	0
NEK9	-1.1178767928	0.561374169	0.9999763851
TMED10	-1.0110647419	0.9341625779	0.9999763851
FOS	-1.9940432718	0.003145	0.9999763851
JDP2	1.1180428059	0.4867551132	0.9999763851
BATF	1.1992822003	0.2467520832	0.9999763851
FLVCR2	-1.0375898469	0.8118000791	0.9999763851

C14orf1	1.3048477792	0.122434486	0.9999763851
TTLL5	-1.1841105081	0.5597943887	0.9999763851
TGFB3	-1.1428539459	0.8692422886	0.9999763851
IFT43	1.140469041	0.8246909778	0.9999763851
GPATCH2L	-1.2344097505	0.2336781602	0.9999763851
ESRRB	6.368054416	0.3492100145	0.9999763851
VASH1	1.0712510693	0.7827542291	0.9999763851
ANGEL1	-1.1757067823	0.5866332573	0.9999763851
LRRC74A	-4.2849908816	0.4028453076	0.9999763851
IRF2BPL	-1.1618014716	0.3856275204	0.9999763851
LOC107984638	3.3962720959	0.2487408509	0.9999763851
LOC105370579	-1.3713018672	0.7312784555	0.9999763851
CIPC	-1.0580576228	0.8550714343	0.9999763851
ZDHHC22	-1.3154339304	0.8205576571	0.9999763851
TMEM63C	3.0369471101	0.0561625942	0.9999763851
POMT2	1.253371753	0.4286488574	0.9999763851
GSTZ1	-1.3297796194	0.4325604954	0.9999763851
TMED8	1.1691735018	0.4005888637	0.9999763851
SAMD15	0	0	0
NOXRED1	-1.8155268442	0.4541459751	0.9999763851
VIPAS39	-1.2157281827	0.5556524596	0.9999763851
AHSA1	1.0621606248	0.6917742413	0.9999763851
SPTLC2	-1.0766039449	0.7020797058	0.9999763851
ALKBH1	1.5933038533	0.2086715079	0.9999763851
SLIRP	1.0850229077	0.5131100829	0.9999763851
SNW1	-1.1223119469	0.4599898777	0.9999763851
C14orf178	-1.3955017315	0.6893712014	0.9999763851
ADCK1	1.1618795222	0.7432025849	0.9999763851
DIO2	3.2353357581	0.554787377	0.9999763851
CEP128	1.0247946641	0.9319968898	0.9999763851
TSHR	-2.3227998731	0.4412873135	0.9999763851
GTF2A1	-1.0495842426	0.8284045633	0.9999763851
SEL1L	1.2039481119	0.3018510768	0.9999763851
FLRT2	0	0	0
GALC	1.1845842897	0.6399514638	0.9999763851
GPR65	-2.0618162662	0.2890546301	0.9999763851
SPATA7	-2.5596117216	0.2619061677	0.9999763851
PTPN21	3.2353357581	0.554787377	0.9999763851
ZC3H14	1.2903767883	0.255127638	0.9999763851
EML5	1.77355136	0.3504963136	0.9999763851
TTC8	-1.1275107155	0.7955702427	0.9999763851
FOXN3	1.0964283051	0.5427838305	0.9999763851
EFCAB11	-1.2759764368	0.5680131961	0.9999763851
TDP1	1.0772430571	0.690036859	0.9999763851
PSMC1	-1.1608491844	0.3161498401	0.9999763851
NRDE2	1.3352783103	0.4981655874	0.9999763851
CALM1	1.094122918	0.5096106212	0.9999763851
TTC7B	-1.3087201242	0.0287706073	0.9999763851
RPS6KA5	-1.3849626133	0.1522951946	0.9999763851

C14orf159	-1.0049876255	0.9845396301	0.9999763851
GPR68	-1.5213785253	0.1236563295	0.9999763851
CCDC88C	-1.1175268014	0.5211530474	0.9999763851
PPP4R3A	1.1224576486	0.5249201912	0.9999763851
CATSPERB	0	0	0
TC2N	1.0692787428	0.8883308497	0.9999763851
TRIP11	-1.2378789805	0.3765916494	0.9999763851
ATXN3	-1.0115922948	0.958513672	0.9999763851
NDUFB1	1.3576675492	0.023313147	0.9999763851
CPSF2	1.0674670837	0.7248906416	0.9999763851
SLC24A4	7.7199860138	0.2827668552	0.9999763851
RIN3	1.1817431331	0.6461993912	0.9999763851
LGMN	5.4781708352	0.3745527127	0.9999763851
GOLGA5	-2.2246364432	0.0170812706	0.9999763851
ITPK1	1.2976657101	0.2073266597	0.9999763851
MOAP1	1.0674639794	0.8032700415	0.9999763851
TMEM251	-1.1325204832	0.704940744	0.9999763851
C14orf142	1.315643376	0.1708421086	0.9999763851
UBR7	1.4740366494	0.0707631629	0.9999763851
BTBD7	1.0278739796	0.8706606071	0.9999763851
UNC79	-1.2836274026	0.198231551	0.9999763851
COX8C	1.0335261549	0.8557657131	0.9999763851
ASB2	1.0511307685	0.8628037794	0.9999763851
OTUB2	-1.1044064817	0.7029547957	0.9999763851
DDX24	1.0325919958	0.8667395332	0.9999763851
IFI27L1	1.0280982828	0.9044647402	0.9999763851
IFI27	-6.5395499284	0.0715111971	0.9999763851
IFI27L2	-1.0992163363	0.5986522715	0.9999763851
PPP4R4	-1.0246989039	0.9749672939	0.9999763851
SERPINA1	-1.108707669	0.8100578236	0.9999763851
DICER1	1.2942199142	0.2289826149	0.9999763851
CLMN	-1.3677549179	0.3948519917	0.9999763851
SYNE3	1.5133967596	0.3969241141	0.9999763851
LOC105370641	0	0	0
GLRX5	1.2172729524	0.1852724744	0.9999763851
ATG2B	1.394769684	0.2867195369	0.9999763851
GSKIP	1.3943642907	0.2198523295	0.9999763851
AK7	3.2353357581	0.554787377	0.9999763851
PAPOLA	-1.0180835712	0.8967296884	0.9999763851
VRK1	1.0536756255	0.7480691266	0.9999763851
BCL11B	-1.1484927822	0.8521482357	0.9999763851
SETD3	-1.0854165674	0.6110688692	0.9999763851
CCNK	1.0720321805	0.7889561101	0.9999763851
CCDC85C	1.3718298627	0.3005497654	0.9999763851
HHIPL1	-7.5622178268	0.2177145568	0.9999763851
CYP46A1	-1.3661346698	0.1688078642	0.9999763851
EML1	2.3413502536	0.4226333491	0.9999763851
EVL	1.1848555144	0.313605435	0.9999763851
DEGS2	0	0	0

YY1	1.0486176224	0.7710142475	0.9999763851
SLC25A29	-1.0282824703	0.8927303433	0.9999763851
WARS	1.0417389437	0.8495607048	0.9999763851
WDR25	1.0993343646	0.7035438152	0.9999763851
BEGAIN	-1.0849160377	0.8968117556	0.9999763851
DLK1	-1.0170263897	0.9761886005	0.9999763851
DIO3	0	0	0
PPP2R5C	1.2151784139	0.1841789101	0.9999763851
DYNC1H1	-1.0496332211	0.7177116537	0.9999763851
HSP90AA1	1.2139586818	0.0922553605	0.9999763851
WDR20	-1.4464015966	0.209170409	0.9999763851
MOK	-1.5611789752	0.3779139874	0.9999763851
ZNF839	1.0481449855	0.8628926118	0.9999763851
CINP	1.1588701408	0.6016683475	0.9999763851
TECPR2	-1.361400044	0.2369778653	0.9999763851
ANKRD9	-1.3248028807	0.4333211325	0.9999763851
RCOR1	-1.0192896859	0.9044059243	0.9999763851
TRAF3	-1.0568947635	0.7945691727	0.9999763851
CDC42BPB	-1.2313879396	0.7534607881	0.9999763851
TNFAIP2	-1.2391850792	0.188693506	0.9999763851
LOC105370687	3.2353357581	0.554787377	0.9999763851
EIF5	1.1968001626	0.2060968784	0.9999763851
MARK3	1.0468550507	0.749500943	0.9999763851
CKB	-1.8508980143	0.2114411285	0.9999763851
TRMT61A	1.0374283598	0.873652458	0.9999763851
BAG5	-1.0068434975	0.9698822471	0.9999763851
APOPT1	1.1489241552	0.4835441661	0.9999763851
KLC1	1.3242871579	0.0560895019	0.9999763851
XRCC3	-1.0552694049	0.8061165559	0.9999763851
ZFYVE21	1.0032978266	0.9850350721	0.9999763851
PPP1R13B	1.0311420768	0.9411968039	0.9999763851
C14orf2	1.2069243396	0.1459850737	0.9999763851
TDRD9	-1.6184601085	0.2518078696	0.9999763851
INF2	1.385537808	0.1212786272	0.9999763851
ADSSL1	-7.5664585246	0.2259050528	0.9999763851
SIVA1	1.1700916619	0.215816606	0.9999763851
AKT1	-1.036920636	0.8134654133	0.9999763851
ZBTB42	1.1584377343	0.7061349718	0.9999763851
CEP170B	3.2356557963	0.5547505991	0.9999763851
PLD4	-7.5622178268	0.2177145568	0.9999763851
C14orf79	1.1719216794	0.7691029435	0.9999763851
CDCA4	-1.1102896237	0.4864017597	0.9999763851
GPR132	1.0118504824	0.9761482386	0.9999763851
JAG2	-1.7344697458	0.4549805961	0.9999763851
NUDT14	-1.2197310707	0.5872713466	0.9999763851
BRF1	-1.4458167332	0.1751336033	0.9999763851
BTBD6	1.2533673879	0.2519341463	0.9999763851
PACS2	1.1468523263	0.4990745253	0.9999763851
TEX22	1.1739877281	0.7950675747	0.9999763851

MTA1	-1.0646540693	0.7026327636	0.9999763851
CRIP2	0	0	0
CRIP1	1.0480191054	0.9202174948	0.9999763851
C14orf80	-1.0393995099	0.8968977105	0.9999763851
TMEM121	-1.0795865172	0.8110368543	0.9999763851
LOC105369274	0	0	0
POTEB	7.7599005912	0.2899043981	0.9999763851
NIPA2	1.4071729286	0.0357478972	0.9999763851
CYFIP1	1.0149206188	0.9409662282	0.9999763851
TUBGCP5	1.1940219778	0.504030799	0.9999763851
SNRPN	1.4130640769	0.0653569086	0.9999763851
SNURF	1.0175453049	0.9322869323	0.9999763851
UBE3A	1.0471442782	0.7736064532	0.9999763851
ATP10A	0	0	0
GABRB3	4.5321181295	0.0803767201	0.9999763851
GABRG3	3.2361532718	0.5546934395	0.9999763851
HERC2	-1.2118257861	0.2305135007	0.9999763851
HERC2P11	9.9312452783	0.2486169813	0.9999763851
GOLGA8M	1.3496267716	0.3969554083	0.9999763851
APBA2	1.0906194713	0.7899790954	0.9999763851
FAM189A1	-3.3291448741	0.2508455485	0.9999763851
NSMCE3	1.3974216759	0.2388207023	0.9999763851
TJP1	2.1351997131	5.83E-06	0.0149348367
GOLGA8J	-1.2619776428	0.2219031399	0.9999763851
CHRFAM7A	-2.2393906731	0.0637349995	0.9999763851
LOC101060569	-1.0810230665	0.9182800245	0.9999763851
LOC101927579	-1.5745091213	0.2435473376	0.9999763851
LOC101930434	3.2361532718	0.5546934395	0.9999763851
FAN1	-1.7543527075	0.0248244023	0.9999763851
MTMR10	3.5495567446	0.0218975379	0.9999763851
TRPM1	3.2356557963	0.5547505991	0.9999763851
LOC283710	-7.5647903763	0.2176246281	0.9999763851
KLF13	1.2592326539	0.2311073856	0.9999763851
OTUD7A	-1.976218429	0.1378958941	0.9999763851
GOLGA8K	-3.6368469092	0.1554927842	0.9999763851
GOLGA8O	11.8381114083	0.001365	0.941889539
LOC100653133	0	0	0
GOLGA8N	1.113102083	0.5433411917	0.9999763851
ARHGAP11A	1.084768125	0.5898444544	0.9999763851
SCG5	-1.8910486008	0.0157103928	0.9999763851
FMN1	0	0	0
RYR3	1.9654718743	0.3595065335	0.9999763851
AVEN	1.2931182987	0.3619190869	0.9999763851
EMC7	1.1780301381	0.2854961034	0.9999763851
PGBD4	1.8142404796	0.5674129376	0.9999763851
KATNBL1	1.3238575552	0.216929908	0.9999763851
EMC4	-1.2161981917	0.2895942999	0.9999763851
SLC12A6	-1.1125219664	0.7618550212	0.9999763851
NOP10	1.1747329187	0.2136104567	0.9999763851

NUTM1	0	0	0
LPCAT4	1.0141869896	0.9762975438	0.9999763851
GOLGA8A	-1.3743618633	0.2964119494	0.9999763851
GOLGA8B	1.2688210612	0.3975334954	0.9999763851
AQR	1.0098447889	0.9608453386	0.9999763851
ZNF770	-1.0996962667	0.5544166066	0.9999763851
DPH6	-1.0439398283	0.8968513524	0.9999763851
C15orf41	-1.2509530446	0.3582324957	0.9999763851
MEIS2	1.1900007014	0.5621461544	0.9999763851
SPRED1	-1.4430537563	0.3465959521	0.9999763851
FAM98B	1.0700841631	0.7195468982	0.9999763851
RASGRP1	1.3258969121	0.7484675255	0.9999763851
C15orf53	5.4781708352	0.3745527127	0.9999763851
THBS1	0	0	0
FSIP1	-1.8165442169	0.4624811618	0.9999763851
EIF2AK4	1.0605477037	0.7277965219	0.9999763851
SRP14	1.1264795055	0.3387693795	0.9999763851
BMF	1.1180839932	0.6892792366	0.9999763851
BUB1B	1.511547826	0.1288416318	0.9999763851
BUB1B-PAK6	-1.2204694913	0.5005382317	0.9999763851
ANKRD63	1.8324140901	0.2228966347	0.9999763851
PLCB2	1.2848014778	0.1976645379	0.9999763851
INAFM2	1.195187054	0.4240531193	0.9999763851
C15orf52	-1.1555457336	0.7962874948	0.9999763851
DISP2	-1.6471937774	0.4537137196	0.9999763851
KNSTRN	-1.0614259536	0.7311179555	0.9999763851
IVD	-1.0974068487	0.6197037695	0.9999763851
BAHD1	1.0122519739	0.9424247162	0.9999763851
CHST14	-1.3702525313	0.1697687576	0.9999763851
C15orf57	-1.1410620197	0.5496241715	0.9999763851
RPUSD2	-1.1131288859	0.5630430192	0.9999763851
CASC5	-1.1448917158	0.4424627929	0.9999763851
RAD51	-1.089837143	0.5581883376	0.9999763851
RMDN3	-1.4350911753	0.0585268741	0.9999763851
GCHFR	1.1924470622	0.3358074592	0.9999763851
DNAJC17	1.3306699864	0.5030739621	0.9999763851
C15orf62	-7.5622178268	0.2177145568	0.9999763851
ZFYVE19	-1.0801789211	0.7455842843	0.9999763851
PPP1R14D	0	0	0
SPINT1	1.2967961371	0.5425933948	0.9999763851
RHOV	1.6045400131	0.2024907551	0.9999763851
VPS18	1.2621565212	0.346436461	0.9999763851
DLL4	3.2356557963	0.5547505991	0.9999763851
CHAC1	1.3101246791	0.5448501399	0.9999763851
INO80	1.1103208773	0.600121524	0.9999763851
CHP1	-1.1584784781	0.4439124906	0.9999763851
OIP5	1.0054354677	0.9715069188	0.9999763851
NUSAP1	1.2257056745	0.1259725679	0.9999763851
NDUFAF1	1.1699036908	0.4392382153	0.9999763851

RTF1	-1.1998248253	0.2042816524	0.9999763851
ITPKA	1.2033287128	0.4484124519	0.9999763851
RPAP1	-1.0386994409	0.8765390845	0.9999763851
TYRO3	1.0947740043	0.5555333148	0.9999763851
MGA	-1.4838154859	0.2202135214	0.9999763851
MAPKBP1	-1.0092518449	0.9799958602	0.9999763851
JMJD7	1.3415898104	0.1838731273	0.9999763851
PLA2G4B	1.0326480616	0.9473537026	0.9999763851
SPTBN5	-1.9667102674	0.4562426721	0.9999763851
EHD4	-1.074281164	0.8490042051	0.9999763851
PLA2G4D	2.027155953	0.4566921088	0.9999763851
PLA2G4F	0	0	0
VPS39	1.2753525472	0.3459292581	0.9999763851
TMEM87A	-1.3200894816	0.0502886223	0.9999763851
GANC	-2.0942123789	0.0323628423	0.9999763851
CAPN3	3.0070207342	0.0977427145	0.9999763851
ZNF106	-1.0938068302	0.5752899308	0.9999763851
SNAP23	-1.1609792616	0.8263604482	0.9999763851
LRRC57	1.1664480927	0.3258770286	0.9999763851
HAUS2	1.3826180953	0.0629863809	0.9999763851
STARD9	-1.4825451185	0.1412892508	0.9999763851
CDAN1	1.4510492157	0.2770559418	0.9999763851
TTBK2	-1.4857512099	0.2497441979	0.9999763851
UBR1	-1.3889793987	0.1371681461	0.9999763851
TMEM62	1.1471782791	0.5997731008	0.9999763851
CCNDBP1	1.0779928553	0.6533026495	0.9999763851
EPB42	0	0	0
TGM5	1.6139567595	0.0628517449	0.9999763851
TGM7	0	0	0
LCMT2	1.5022389329	0.2879370514	0.9999763851
ADAL	1.5617218108	0.242415469	0.9999763851
ZSCAN29	-1.4156852347	0.200059224	0.9999763851
TUBGCP4	-1.0730342888	0.6849125558	0.9999763851
TP53BP1	-1.0105262219	0.9788288289	0.9999763851
MAP1A	-1.3157515262	0.822405115	0.9999763851
PPIP5K1	-1.2952959251	0.571130903	0.9999763851
CATSPER2	1.2306977958	0.5592351738	0.9999763851
CKMT1A	-1.4214535124	0.5872146233	0.9999763851
PDIA3	-1.149166722	0.4177319432	0.9999763851
ELL3	1.0787893643	0.8503745427	0.9999763851
SERF2	-1.0763217179	0.5735296156	0.9999763851
SERINC4	0	0	0
HYPK	1.2075143774	0.2312081274	0.9999763851
MFAP1	1.0479773392	0.8009967266	0.9999763851
WDR76	1.2272267104	0.2370309686	0.9999763851
FRMD5	-1.8164974932	0.2877322169	0.9999763851
CASC4	1.4922170707	0.046406942	0.9999763851
CTDSPL2	1.2960489645	0.2325709765	0.9999763851
EIF3J	-1.0459879141	0.7350514867	0.9999763851

SPG11	1.3799735952	0.1431856016	0.9999763851
PATL2	-1.2571014048	0.7135055873	0.9999763851
B2M	1.062627034	0.624183536	0.9999763851
SORD	-1.1512425569	0.3239825866	0.9999763851
DUOXA1	-7.5622178268	0.2177145568	0.9999763851
DUOX1	3.2356557963	0.5547505991	0.9999763851
SHF	1.7450428903	0.6326046324	0.9999763851
SLC28A2	-10.8385246611	0.1452344072	0.9999763851
GATM	-1.3161718537	0.8290538512	0.9999763851
SPATA5L1	-1.0826106007	0.8422581788	0.9999763851
C15orf48	-1.3940632796	0.7033607529	0.9999763851
LOC105376714	1.2865109866	0.8337358825	0.9999763851
SLC30A4	-7.3573348511	0.0415312621	0.9999763851
BLOC1S6	1.1247503106	0.4446884243	0.9999763851
SQRDL	1.0588702751	0.6604545531	0.9999763851
SEMA6D	1.2067719715	0.3192996386	0.9999763851
SLC24A5	0	0	0
SLC12A1	-7.5647903763	0.2176246281	0.9999763851
DUT	1.3399467374	0.0270343383	0.9999763851
FBN1	-2.0616365036	0.2870938773	0.9999763851
CEP152	-1.1526293349	0.3869675404	0.9999763851
EID1	1.0951354147	0.4877543507	0.9999763851
SECISBP2L	-1.3255552882	0.163874075	0.9999763851
COPS2	-1.0241836036	0.8691070942	0.9999763851
GALK2	1.0282123421	0.8633282678	0.9999763851
FAM227B	-1.679491499	0.1787567505	0.9999763851
DTWD1	-1.0924569781	0.7111827754	0.9999763851
ATP8B4	1.2969524787	0.0750553594	0.9999763851
SLC27A2	2.6245799343	0.0203770122	0.9999763851
HDC	-4.2849908816	0.4028453076	0.9999763851
GABPB1	-1.145621223	0.4553928255	0.9999763851
USP8	-1.1302296627	0.5370921077	0.9999763851
USP50	3.4795886266	0.1215585104	0.9999763851
TRPM7	-1.2388429003	0.1456686646	0.9999763851
SPPL2A	1.0936801666	0.6784871508	0.9999763851
AP4E1	-1.1568220072	0.4513266463	0.9999763851
CYP19A1	-4.2864494036	0.4027150576	0.9999763851
GLDN	-1.3154339304	0.8205576571	0.9999763851
DMXL2	-1.0229663256	0.8740061105	0.9999763851
SCG3	-2.3214980365	0.4371133126	0.9999763851
LYSMD2	1.0226860115	0.906161627	0.9999763851
TMOD2	-1.1761801462	0.5917656762	0.9999763851
TMOD3	-1.0009590706	0.9947474184	0.9999763851
LEO1	1.0071616295	0.9754310277	0.9999763851
MAPK6	1.0091413333	0.951825807	0.9999763851
GNB5	-1.0066914591	0.9784347992	0.9999763851
MYO5C	1.853680583	0.4438768915	0.9999763851
MYO5A	-1.234391415	0.5153683471	0.9999763851
ARPP19	1.2137378175	0.2233467991	0.9999763851

FAM214A	-1.5005740005	0.2072645796	0.9999763851
LOC107983981	1.1886434711	0.769483385	0.9999763851
UNC13C	0	0	0
RSL24D1	-1.0844241121	0.5537846483	0.9999763851
RAB27A	-1.049161985	0.7198137458	0.9999763851
PIGBOS1	1.079324942	0.7558661512	0.9999763851
PIGB	-1.6179934434	0.0966603458	0.9999763851
CCPG1	1.0989471035	0.4972607038	0.9999763851
C15orf65	-1.3717838846	0.7496607128	0.9999763851
DYX1C1	1.3886971814	0.6551591573	0.9999763851
PYGO1	0	0	0
PRTG	-2.3214980365	0.437230213	0.9999763851
NEDD4	1.032850494	0.8942378607	0.9999763851
RFX7	-1.3302461797	0.1817786519	0.9999763851
TEX9	1.7590401103	0.4576017216	0.9999763851
MNS1	-1.1115570433	0.8819103505	0.9999763851
ZNF280D	1.0084786864	0.9758946843	0.9999763851
TCF12	-1.4140854878	0.0160689497	0.9999763851
CGNL1	1.2491760998	0.6936243595	0.9999763851
POLR2M	1.0043673644	0.9838257035	0.9999763851
ALDH1A2	0	0	0
LOC101928635	0	0	0
AQP9	-8.9954408168	0.0160073831	0.9999763851
LIPC	0	0	0
ADAM10	1.0007608381	0.9967265596	0.9999763851
FAM63B	1.0793425334	0.6772356069	0.9999763851
SLTM	1.0531369254	0.7149306498	0.9999763851
RNF111	1.2372563758	0.4414508672	0.9999763851
CCNB2	-1.0174106925	0.8917698565	0.9999763851
MYO1E	-1.4431270474	0.3279646756	0.9999763851
FAM81A	-1.1420017683	0.6705647982	0.9999763851
GTF2A2	1.1586460456	0.3605510977	0.9999763851
BNIP2	1.0440213206	0.7594356327	0.9999763851
ANXA2	1.2006487618	0.1967999219	0.9999763851
ICE2	1.36812103	0.1075580053	0.9999763851
RORA	-2.5948264407	0.0765471993	0.9999763851
VPS13C	1.0300095272	0.8637009754	0.9999763851
LOC105370849	0	0	0
TLN2	-1.8023222043	0.0492829908	0.9999763851
TPM1	1.0832154117	0.6942982566	0.9999763851
LACTB	-1.3248116562	0.1557426696	0.9999763851
RPS27L	1.0682656678	0.6447126875	0.9999763851
RAB8B	-1.2468571291	0.302567835	0.9999763851
APH1B	1.0073115753	0.9726643106	0.9999763851
CA12	3.2353357581	0.554787377	0.9999763851
USP3	1.2103649667	0.2592892866	0.9999763851
FBXL22	1.0301153598	0.8963998083	0.9999763851
HERC1	-1.1645223241	0.5351442161	0.9999763851
DAPK2	1.4837238256	0.4464315257	0.9999763851

FAM96A	-1.1166570594	0.3879828081	0.9999763851
SNX1	-1.2475149636	0.1448785147	0.9999763851
SNX22	-1.4579219457	0.0221519135	0.9999763851
PPIB	-21.5726182549	0.0723492168	0.9999763851
CSNK1G1	-1.2252929639	0.3555901115	0.9999763851
KIAA0101	1.0379948138	0.7663458906	0.9999763851
TRIP4	-1.1359188837	0.6537441782	0.9999763851
ZNF609	-1.4355233392	0.0410162379	0.9999763851
OAZ2	1.0051234332	0.9745274458	0.9999763851
RBPMS2	3.2356557963	0.5547505991	0.9999763851
PIF1	1.1306282344	0.5490600858	0.9999763851
PLEKHO2	1.2494013776	0.2961304378	0.9999763851
ANKDD1A	1.0327525483	0.9025477575	0.9999763851
SPG21	1.0486410616	0.7154900168	0.9999763851
MTFMT	-1.3583788028	0.1090665212	0.9999763851
RASL12	0	0	0
UBAP1L	-2.2160441003	0.225803791	0.9999763851
PDCD7	-1.1445984222	0.47040392	0.9999763851
CLPX	1.1727195968	0.3632793369	0.9999763851
PARP16	1.0459919078	0.835276476	0.9999763851
IGDCC3	3.2356557963	0.5547505991	0.9999763851
IGDCC4	-1.371091068	0.7484030603	0.9999763851
DPP8	-1.1544653224	0.4278024043	0.9999763851
HACD3	1.2788600355	0.0980768489	0.9999763851
VWA9	1.1109841579	0.6316752037	0.9999763851
SLC24A1	1.4753751177	0.0806294451	0.9999763851
DENND4A	-1.0111700098	0.9559498323	0.9999763851
RAB11A	-1.019789901	0.8946769817	0.9999763851
MEGF11	-4.2849908816	0.4028453076	0.9999763851
DIS3L	1.0489947442	0.8027102094	0.9999763851
TIPIN	-1.0451623442	0.823648412	0.9999763851
MAP2K1	-1.8426234914	0.0434699276	0.9999763851
SNAPC5	1.0814280189	0.6184750146	0.9999763851
RPL4	-1.1048625948	0.4340406693	0.9999763851
ZWILCH	-1.295372828	0.4335069502	0.9999763851
LCTL	1.0084462472	0.9570932293	0.9999763851
SMAD6	-1.1730080493	0.5423427694	0.9999763851
SMAD3	-1.4309809053	0.1047715548	0.9999763851
AAGAB	-1.395080981	0.0184062468	0.9999763851
IQCH	1.2112706036	0.571660306	0.9999763851
C15orf61	-1.0268933593	0.8744327438	0.9999763851
MAP2K5	1.064375052	0.7592522728	0.9999763851
SKOR1	1.4987063639	0.3640738393	0.9999763851
PIAS1	-1.1665509386	0.3338070866	0.9999763851
CALML4	-1.1291675761	0.5887793628	0.9999763851
CLN6	1.1940407882	0.3081585047	0.9999763851
FEM1B	1.2392646243	0.2165279896	0.9999763851
ITGA11	1.0949541325	0.8930561542	0.9999763851
CORO2B	-1.8155805633	0.2919182654	0.9999763851

ANP32A	1.0239196929	0.8574375183	0.9999763851
SPESP1	1.0273967864	0.9753624609	0.9999763851
NOX5	0	0	0
GLCE	1.2177422228	0.681030015	0.9999763851
PAQR5	-2.5519849925	0.3673796027	0.9999763851
KIF23	1.0075853624	0.9681040816	0.9999763851
RPLP1	1.0622378963	0.6126181498	0.9999763851
TLE3	1.0318258355	0.824824701	0.9999763851
UACA	-1.0005497817	0.9979871265	0.9999763851
LARP6	-1.3154339304	0.8205576571	0.9999763851
LRRC49	0	0	0
THAP10	0	0	0
THSD4	1.0186740864	0.9080875182	0.9999763851
MYO9A	-1.0212240708	0.9147966941	0.9999763851
SENP8	-2.3220580825	0.4365515537	0.9999763851
GRAMD2	0	0	0
PKM	1.1063172187	0.4136400498	0.9999763851
PARP6	-1.2673098767	0.2889158182	0.9999763851
CELF6	-1.434036104	0.4692753993	0.9999763851
HEXA	-1.0235875123	0.8838604896	0.9999763851
ARIH1	-1.0971502195	0.5517805853	0.9999763851
GOLGA6B	0	0	0
BBS4	1.2026743796	0.6438688769	0.9999763851
ADPGK	1.0483710119	0.805453605	0.9999763851
NEO1	1.3035167051	0.3107391718	0.9999763851
LOC107984728	-4.2864494036	0.4027150577	0.9999763851
REC114	-4.0939088663	0.196705311	0.9999763851
NPTN	-1.0873050083	0.5706403584	0.9999763851
CD276	-1.5100843857	0.2789106089	0.9999763851
STOML1	-1.079446406	0.7824961939	0.9999763851
PML	1.0684569094	0.7194653408	0.9999763851
LOC732265	0	0	0
STRA6	1.875820524	0.4834469148	0.9999763851
CCDC33	14.4496536579	0.1576050684	0.9999763851
SEMA7A	-1.4680969778	0.3638904739	0.9999763851
UBL7	1.3718132832	0.2001899047	0.9999763851
ARID3B	1.1245591249	0.7402789812	0.9999763851
CLK3	-1.2765509237	0.3099807964	0.9999763851
EDC3	1.290012244	0.3313009307	0.9999763851
CYP1A1	1.9603274464	0.006903	0.9999763851
CSK	-1.055654525	0.729802855	0.9999763851
CPLX3	0	0	0
ULK3	-1.0826950941	0.5845690224	0.9999763851
SCAMP2	-1.5085140934	0.005593	0.9999763851
MPI	-1.3689345067	0.1300770921	0.9999763851
FAM219B	1.0130167433	0.9445768499	0.9999763851
COX5A	1.3308810339	0.0158460388	0.9999763851
RPP25	1.0282463515	0.9031117762	0.9999763851
SCAMP5	1.237058262	0.4045081618	0.9999763851

PPCDC	-1.336207849	0.3935569671	0.9999763851
C15orf39	-1.0276284391	0.9031742446	0.9999763851
LOC105376731	0	0	0
COMMD4	1.2354737932	0.2196721879	0.9999763851
NEIL1	1.8815691358	0.3797756536	0.9999763851
MAN2C1	1.3306386159	0.2972555662	0.9999763851
SIN3A	-1.0154983511	0.9392003039	0.9999763851
PTPN9	1.0047304414	0.98242446	0.9999763851
SNUPN	1.3129650794	0.1596982012	0.9999763851
IMP3	-1.2389673855	0.1063924595	0.9999763851
SNX33	-2.5474362455	0.0486548944	0.9999763851
UBE2Q2	-1.1044439159	0.5703283971	0.9999763851
FBXO22	1.2003633378	0.3157640966	0.9999763851
NRG4	-1.131206398	0.6707792487	0.9999763851
TMEM266	3.410933624	0.1443048838	0.9999763851
ETFA	-1.1151466065	0.3866265057	0.9999763851
ISL2	1.6697244135	0.436121507	0.9999763851
SCAPER	-1.1722008032	0.4930456041	0.9999763851
RCN2	-1.0595820767	0.740207631	0.9999763851
PSTPIP1	1.3973120488	0.1532124175	0.9999763851
TSPAN3	-1.1119662725	0.4633977341	0.9999763851
PEAK1	-1.3057613625	0.1772178881	0.9999763851
HMG20A	-1.2104808074	0.2272736955	0.9999763851
LINGO1	-4.2864494036	0.4027150577	0.9999763851
TBC1D2B	-1.0206279598	0.9062753052	0.9999763851
CIB2	-1.762890411	0.2557075219	0.9999763851
IDH3A	-1.0599201751	0.7538849088	0.9999763851
ACSBG1	-2.0620617764	0.3255177884	0.9999763851
DNAJA4	1.1392672551	0.6610125897	0.9999763851
WDR61	1.0687175096	0.6348249763	0.9999763851
IREB2	-1.032437139	0.8294422191	0.9999763851
HYKK	-4.2864494036	0.4027150576	0.9999763851
PSMA4	1.0549534316	0.6665092124	0.9999763851
CHRNA5	1.3752710391	0.2825872603	0.9999763851
CHRNA3	-1.0808570019	0.9213116299	0.9999763851
CHRNB4	0	0	0
ADAMTS7	-1.734127143	0.4720132604	0.9999763851
MORF4L1	-1.2399341761	0.0770376873	0.9999763851
CTSH	-1.2456126596	0.2182140867	0.9999763851
RASGRF1	-1.9647456674	0.4666785999	0.9999763851
ANKRD34C	0	0	0
TMED3	-1.138806241	0.3024300581	0.9999763851
KIAA1024	-1.0346922203	0.9416203726	0.9999763851
MTHFS	1.1762027674	0.3344714158	0.9999763851
ST20-MTHFS	0	0	0
ST20	1.0948940003	0.5558530845	0.9999763851
BCL2A1	-1.2144856475	0.1381299707	0.9999763851
ZFAND6	-1.0615944308	0.6908514317	0.9999763851
FAH	1.1159820333	0.4452241914	0.9999763851

ARNT2	3.2361532718	0.5546934395	0.9999763851
ABHD17C	-1.6190833591	0.0001712	0.2302753662
CEMIP	-1.3470658739	0.5372310133	0.9999763851
MESDC2	1.0810427116	0.5674264197	0.9999763851
MESDC1	-1.4733843402	0.007659	0.9999763851
CFAP161	0	0	0
IL16	1.395399928	0.0546502364	0.9999763851
STARD5	-1.7611771642	0.2386080919	0.9999763851
TMC3	-1.3048572565	0.581285498	0.9999763851
MEX3B	-1.4251277271	0.5598666089	0.9999763851
EFL1	1.1282205658	0.6034177634	0.9999763851
SAXO2	0	0	0
GOLGA6L10	-1.3151016298	0.821261495	0.9999763851
GOLGA6L9	-1.2388924391	0.5817515287	0.9999763851
RPS17	1.0283278139	0.8092452057	0.9999763851
AP3B2	3.2356557963	0.5547505991	0.9999763851
FSD2	-1.3703523424	0.1675439246	0.9999763851
WHAMM	1.0801136599	0.7729873551	0.9999763851
HOMER2	-1.0850230024	0.9014494212	0.9999763851
FAM103A1	1.0557268552	0.7737321306	0.9999763851
C15orf40	1.2582131845	0.4055015639	0.9999763851
BTBD1	-1.4950721211	0.2282978279	0.9999763851
TM6SF1	-1.1274970118	0.6292121797	0.9999763851
HDGFRP3	1.548733729	0.4184307244	0.9999763851
SH3GL3	0	0	0
ADAMTSL3	-1.3157515262	0.8224529522	0.9999763851
UBE2Q2L	-1.315303824	0.8206171563	0.9999763851
GOLGA6L4	1.8137824633	0.5647228959	0.9999763851
LOC105376722	0	0	0
LOC102724135	2.7613468677	0.1587819227	0.9999763851
ZSCAN2	1.4960066429	0.2590935642	0.9999763851
WDR73	-1.1658181548	0.5828766852	0.9999763851
NMB	1.3057634074	0.1031565035	0.9999763851
SEC11A	-1.2592941295	0.0773616049	0.9999763851
ZNF592	1.1489701094	0.3388460237	0.9999763851
SLC28A1	3.2361532718	0.5546934395	0.9999763851
PDE8A	-1.5582407316	0.2085754345	0.9999763851
LOC101929479	-19.2975026899	0.0745445842	0.9999763851
AKAP13	-1.0288844925	0.8577265686	0.9999763851
KLHL25	-1.2518394342	0.4801689335	0.9999763851
NTRK3	1.1566956761	0.4512497748	0.9999763851
MRPL46	1.3380795409	0.0967193209	0.9999763851
MRPS11	1.3920473231	0.0930551005	0.9999763851
DET1	-1.2381588911	0.6404330018	0.9999763851
AEN	1.1946929691	0.2416118021	0.9999763851
ISG20	-2.9373390517	0.0646454926	0.9999763851
ACAN	0	0	0
HAPLN3	3.2356557963	0.5547505991	0.9999763851
MFGE8	-1.0296977446	0.9241841833	0.9999763851

ABHD2	-1.1455530671	0.3567952458	0.9999763851
FANCI	1.2835677552	0.4233776346	0.9999763851
POLG	-1.06220127	0.6804538248	0.9999763851
TICRR	-1.0585400835	0.8158426695	0.9999763851
KIF7	-1.4286541955	0.5388886767	0.9999763851
PLIN1	0	0	0
PEX11A	1.8131652995	0.5810863128	0.9999763851
WDR93	0	0	0
MESP1	12.4427966444	0.1869118792	0.9999763851
MESP2	-2.5604817607	0.2733447017	0.9999763851
ANPEP	1.0661557403	0.6502601026	0.9999763851
AP3S2	-1.03283249	0.8696375071	0.9999763851
C15orf38-AP3S2	-10.8385246611	0.1451971321	0.9999763851
ZNF710	-1.0136292866	0.9564018768	0.9999763851
IDH2	-1.1783266727	0.2634830123	0.9999763851
SEMA4B	1.110619294	0.8084943371	0.9999763851
CIB1	1.2575008902	0.1898120354	0.9999763851
GDPGP1	5.4770877341	0.3746135179	0.9999763851
NGRN	1.3937291778	0.0480453227	0.9999763851
ZNF774	9.9624059448	0.2313849172	0.9999763851
IQGAP1	-1.0116379513	0.9351477093	0.9999763851
CRTC3	1.1970287763	0.1762923345	0.9999763851
BLM	1.1012603346	0.6469031147	0.9999763851
FURIN	-1.1145076094	0.4588390473	0.9999763851
FES	1.3053348228	0.2238833712	0.9999763851
MAN2A2	-1.0464334895	0.8526298425	0.9999763851
UNC45A	-1.0985448978	0.5592072739	0.9999763851
HDDC3	1.2034611299	0.3140553555	0.9999763851
RCCD1	-1.1361094306	0.433365491	0.9999763851
PRC1	1.3195044057	0.1089111675	0.9999763851
VPS33B	2.0325084624	0.0978903991	0.9999763851
SV2B	1.4057119189	0.332556999	0.9999763851
SLCO3A1	1.1586293796	0.3517347177	0.9999763851
C15orf32	0	0	0
FAM174B	-3.3667365964	0.0822007356	0.9999763851
CHD2	-1.0365736049	0.8430556635	0.9999763851
RGMA	3.2356557963	0.5547505991	0.9999763851
MCTP2	-1.0579204029	0.8617249193	0.9999763851
NR2F2	1.2801664493	0.2726140955	0.9999763851
IGF1R	1.2115166815	0.3382240344	0.9999763851
LRRC28	-2.2834115981	0.0122441296	0.9999763851
MEF2A	-1.2621672826	0.3073621695	0.9999763851
LYSMD4	2.0798362004	0.0162703516	0.9999763851
ADAMTS17	1.2726068991	0.4108725946	0.9999763851
CERS3	-1.1484927822	0.8523925759	0.9999763851
LINS1	-1.0471396859	0.8899055053	0.9999763851
ASB7	-1.059137913	0.8533191585	0.9999763851
ALDH1A3	-4.2849908816	0.4028453076	0.9999763851
LRRK1	1.1448917872	0.6931854778	0.9999763851

CHSY1	1.0577187938	0.7046736059	0.9999763851
VIMP	-1.0571620308	0.7473591819	0.9999763851
SNRPA1	-1.0767428149	0.5999943248	0.9999763851
TM2D3	1.0764353056	0.672074307	0.9999763851
TARSL2	-1.0136338617	0.9550842088	0.9999763851
LOC100128108	-1.3943388833	0.6976327539	0.9999763851
POLR3K	-1.0165584321	0.907206148	0.9999763851
SNRNP25	1.3905933568	0.0851033157	0.9999763851
RHBDF1	3.2356557963	0.5547505991	0.9999763851
MPG	1.0330846715	0.8303231701	0.9999763851
NPRL3	1.2030850339	0.4987779448	0.9999763851
LUC7L	1.9186905814	0.002195	0.9999763851
FAM234A	1.1519953019	0.6665568051	0.9999763851
RGS11	0	0	0
PDIA2	0	0	0
AXIN1	1.5160490518	0.0356339568	0.9999763851
MRPL28	-1.1241765729	0.6294902255	0.9999763851
TMEM8A	1.0831728164	0.7426384802	0.9999763851
NME4	1.1296753348	0.5195929392	0.9999763851
DECR2	2.1439120143	0.0155517712	0.9999763851
RAB11FIP3	1.0805876287	0.8189984717	0.9999763851
CAPN15	1.2643818596	0.3496774341	0.9999763851
NHLRC4	-1.3158816768	0.8203229796	0.9999763851
PIGQ	-2.0848233926	0.085346169	0.9999763851
RAB40C	1.0864762822	0.7471396997	0.9999763851
WFIKKN1	18.9337530925	0.1183773971	0.9999763851
C16orf13	-1.0822646173	0.7107952525	0.9999763851
MCRIP2	-1.410614508	0.1195210882	0.9999763851
WDR90	-1.1779247292	0.3855296921	0.9999763851
RHOT2	1.0097721214	0.9588582037	0.9999763851
RHBDL1	-1.258591527	0.7129985657	0.9999763851
STUB1	1.3197891344	0.1162558364	0.9999763851
JMJD8	-2.9668522646	0.0870408888	0.9999763851
WDR24	1.1981701765	0.7240252433	0.9999763851
FBXL16	-1.2483867832	0.553550747	0.9999763851
METRN	1.278271816	0.6339068788	0.9999763851
FAM173A	-1.1163994476	0.705052052	0.9999763851
CCDC78	0	0	0
HAGHL	1.0081732941	0.9782212905	0.9999763851
NARFL	-1.1819151916	0.6365281905	0.9999763851
RPUSD1	-1.0094307391	0.9715711343	0.9999763851
CHTF18	1.0093574677	0.9730114563	0.9999763851
PRR25	-4.2849908816	0.4028453076	0.9999763851
LMF1	-1.0576923102	0.8949890405	0.9999763851
SSTR5	-4.2849908816	0.4028453076	0.9999763851
CACNA1H	0	0	0
TPSB2	-4.2864494036	0.4027150576	0.9999763851
TPSAB1	1.2303080856	0.1909914731	0.9999763851
UBE2I	1.1214247916	0.4717530393	0.9999763851

TSR3	-1.04213887	0.7861851537	0.9999763851
GNPTG	-1.1917560214	0.4150418815	0.9999763851
UNKL	-1.4417042689	0.3230376368	0.9999763851
C16orf91	-1.1191339506	0.6659416977	0.9999763851
CCDC154	1.1134374935	0.8328927841	0.9999763851
CLCN7	1.1054136073	0.5610658448	0.9999763851
TELO2	1.2528458085	0.3791536574	0.9999763851
IFT140	-2.5785151148	0.0485244425	0.9999763851
TMEM204	5.4776292235	0.3745831165	0.9999763851
CRAMP1	-1.0557782062	0.8464320726	0.9999763851
HN1L	1.2966353491	0.0904506016	0.9999763851
MAPK8IP3	1.2904932202	0.3620531615	0.9999763851
NME3	-1.6821549226	0.4452894633	0.9999763851
MRPS34	1.154830355	0.3175641198	0.9999763851
EME2	0	0	0
SPSB3	1.0921832798	0.7162252386	0.9999763851
NUBP2	1.2248948424	0.3102937786	0.9999763851
HAGH	-1.4095429277	0.1359648251	0.9999763851
FAHD1	1.0138046614	0.9591203708	0.9999763851
MEIOB	0	0	0
HS3ST6	0	0	0
MSRB1	1.2680704053	0.1013902321	0.9999763851
RPL3L	0	0	0
NDUFB10	1.1205990837	0.4729709851	0.9999763851
RPS2	1.3593533714	0.0126147678	0.9999763851
TBL3	1.0020475844	0.9963509966	0.9999763851
NOXO1	-1.3711818216	0.7359931333	0.9999763851
GFER	1.0320381057	0.9368389146	0.9999763851
ZNF598	1.2495188393	0.1168687306	0.9999763851
NPW	0	0	0
NTHL1	1.366244644	0.2286111654	0.9999763851
TSC2	-1.3584939481	0.4661881996	0.9999763851
PKD1	-1.4435428726	0.28968819	0.9999763851
TRAF7	1.1272947192	0.5636399778	0.9999763851
MLST8	-1.1204673359	0.6612891712	0.9999763851
BRICD5	-1.3022505632	0.640124484	0.9999763851
PGP	1.5781216412	0.007961	0.9999763851
E4F1	1.2645775038	0.4059302504	0.9999763851
DNASE1L2	7.7219356627	0.2870682832	0.9999763851
ECI1	1.3182201719	0.1230785016	0.9999763851
RNPS1	1.0701364561	0.6654009185	0.9999763851
ABCA3	1.2896441358	0.3852406677	0.9999763851
CCNF	-1.1142264841	0.5192506212	0.9999763851
C16orf59	1.5854863787	0.0774005772	0.9999763851
TBC1D24	1.0873909459	0.7111290583	0.9999763851
ATP6V0C	1.0273033991	0.839666264	0.9999763851
AMDHD2	1.3252610453	0.3276124255	0.9999763851
CEMP1	1.3281447959	0.3027725563	0.9999763851
PDPK1	-1.1914516084	0.4456902708	0.9999763851

KCTD5	-1.1312103628	0.520868247	0.9999763851
PRSS27	-1.3708355312	0.7473175529	0.9999763851
SRRM2	-1.3582543752	0.0188234188	0.9999763851
TCEB2	1.1094274353	0.4753478849	0.9999763851
PRSS41	0	0	0
ZG16B	-2.3209115874	0.4600323819	0.9999763851
PRSS22	0	0	0
FLYWCH2	1.7592136256	0.4466700096	0.9999763851
FLYWCH1	-1.547818952	0.2126747191	0.9999763851
KREMEN2	1.4605855953	0.2388093381	0.9999763851
PAQR4	1.0753630423	0.6569532651	0.9999763851
PKMYT1	18.2197362899	0.1335428821	0.9999763851
CLDN9	7.4933530102	0.2984933196	0.9999763851
TNFRSF12A	-1.3948299892	0.3367272453	0.9999763851
HCFC1R1	-1.0477997223	0.8490439345	0.9999763851
THOC6	-1.4148090745	0.6667649706	0.9999763851
BICDL2	-1.3158816768	0.8203229796	0.9999763851
MMP25	1.3133007328	0.0515846602	0.9999763851
IL32	1.1193375794	0.4635950604	0.9999763851
ZNF213	1.4676600125	0.4961183188	0.9999763851
OR1F1	1.3931546432	0.7805583477	0.9999763851
ZNF200	-1.028224716	0.9367976837	0.9999763851
MEFV	3.2353357581	0.554787377	0.9999763851
ZNF263	1.2751690848	0.465035147	0.9999763851
TIGD7	-1.946360247	0.2368441498	0.9999763851
ZNF75A	1.4444279444	0.2394998052	0.9999763851
ZSCAN32	1.4360936694	0.5558711813	0.9999763851
ZNF174	1.4172262368	0.3767007479	0.9999763851
ZNF597	2.5233645174	0.101584876	0.9999763851
NAA60	1.6011212398	0.0813784656	0.9999763851
CLUAP1	1.3698099455	0.4101006846	0.9999763851
NLRC3	1.6670781462	0.0144887982	0.9999763851
SLX4	1.7360868867	0.2780065091	0.9999763851
DNASE1	1.3150705446	0.0897501219	0.9999763851
TRAP1	2.0878543187	0.240335612	0.9999763851
CREBBP	1.1399191197	0.540262033	0.9999763851
ADCY9	-1.4454723025	0.2757415412	0.9999763851
TFAP4	1.1460204305	0.6640595	0.9999763851
GLIS2	-1.0785703051	0.801579469	0.9999763851
PAM16	-1.1509405224	0.6326677604	0.9999763851
CORO7-PAM16	-54.8867774144	0.0136746322	0.9999763851
CORO7	2.7211475822	0.001237	0.8877123975
DNAJA3	1.1166241625	0.5073435087	0.9999763851
NMRAL1	2.0357182782	0.007023	0.9999763851
HMOX2	1.2626919311	0.1937348116	0.9999763851
CDIP1	1.1912725459	0.4857217854	0.9999763851
C16orf96	-1.1634453187	0.7150725687	0.9999763851
UBALD1	1.2881944537	0.4983693459	0.9999763851
MGRN1	1.2210769515	0.3781611324	0.9999763851

NUDT16L1	-1.2657109448	0.1575239908	0.9999763851
ANKS3	-1.6534620495	0.2495103305	0.9999763851
ZNF500	1.9573813386	0.1320207215	0.9999763851
SEPT12	0	0	0
SMIM22	5.4770877341	0.3746135179	0.9999763851
ROGDI	-1.2208847101	0.367380131	0.9999763851
GLYR1	1.1740992552	0.3671448411	0.9999763851
UBN1	-1.0270039832	0.8572584245	0.9999763851
SEC14L5	0	0	0
NAGPA	-1.5235019618	0.0804947756	0.9999763851
C16orf89	0	0	0
ALG1	46.8233069212	0.0377462812	0.9999763851
EEF2KMT	-1.1753371232	0.4673938232	0.9999763851
TMEM114	0	0	0
METTL22	-1.8398035576	0.1470699994	0.9999763851
ABAT	-1.0352603217	0.9579358385	0.9999763851
TMEM186	1.0191054807	0.937777144	0.9999763851
PMM2	-1.0970809583	0.6556786632	0.9999763851
CARHSP1	1.0903596638	0.6321925872	0.9999763851
USP7	1.1221343066	0.4258602963	0.9999763851
C16orf72	-1.1933340555	0.2699064436	0.9999763851
ATF7IP2	1.0716525358	0.8072306839	0.9999763851
EMP2	-1.2848646031	0.1926745537	0.9999763851
TEKT5	-7.5622178268	0.2177145568	0.9999763851
NUBP1	1.0435669252	0.8771334749	0.9999763851
TVP23A	0	0	0
CIITA	-1.4077065642	0.6444410636	0.9999763851
DEXI	1.0727743412	0.6180717715	0.9999763851
CLEC16A	1.1514611162	0.592927736	0.9999763851
LOC107984859	3.2353357581	0.554787377	0.9999763851
SOCS1	1.5887888751	0.3277158303	0.9999763851
RMI2	1.0790026953	0.6573504288	0.9999763851
LOC400499	-1.7351751251	0.4560530772	0.9999763851
LITAF	1.1406332842	0.2983596491	0.9999763851
SNN	1.3072077413	0.2359624007	0.9999763851
TXNDC11	1.0787329428	0.8376633331	0.9999763851
ZC3H7A	1.0695218942	0.8214219227	0.9999763851
RSL1D1	1.116342662	0.4532424427	0.9999763851
GSPT1	1.1939219773	0.194238728	0.9999763851
SNX29	-1.0433654426	0.9000943839	0.9999763851
CPPED1	-1.0783837494	0.8137191823	0.9999763851
SHISA9	1.8140611791	0.5843782687	0.9999763851
ERCC4	-1.3808683643	0.378826146	0.9999763851
MKL2	1.0919788133	0.8096981335	0.9999763851
PARN	-1.2092362333	0.403591255	0.9999763851
BFAR	1.0278734383	0.894417184	0.9999763851
PLA2G10	3.3937384616	0.2324742559	0.9999763851
NOMO1	1.039203756	0.8465774872	0.9999763851
NPIPA1	-1.249350536	0.6464093256	0.9999763851

PDXDC1	-1.709503123	0.0005209	0.5206160142
NTAN1	-1.0198854673	0.930979823	0.9999763851
RRN3	-1.4127622903	0.048840002	0.9999763851
NPIPA5	-7.5647903763	0.2176246281	0.9999763851
C16orf45	-1.4300348335	0.5354813571	0.9999763851
KIAA0430	-1.8681323242	0.002616	0.9999763851
NDE1	1.1390346241	0.4109388104	0.9999763851
MYH11	-1.4261611759	0.5857172933	0.9999763851
FOPNL	-1.250215949	0.2119122471	0.9999763851
ABCC1	-1.1417350748	0.6237147033	0.9999763851
NOMO3	2.3818235376	0.2353385649	0.9999763851
NPIPA7	-1.4129914544	0.6387901956	0.9999763851
XYLT1	1.3957375972	0.228882788	0.9999763851
NPIPA8	7.7488609396	0.3059379923	0.9999763851
LOC102723728	1.0222936935	0.8636181296	0.9999763851
NOMO2	1.027644744	0.977726264	0.9999763851
RPS15A	1.0053673729	0.963149077	0.9999763851
ARL6IP1	1.1783908529	0.3065891607	0.9999763851
SMG1	1.0123230705	0.9597420893	0.9999763851
COQ7	-1.8158263248	0.2088892072	0.9999763851
ITPRIPL2	-1.1242300002	0.8483581398	0.9999763851
SYT17	-4.2864494036	0.4027150576	0.9999763851
TMC5	1.6243491221	0.5671682172	0.9999763851
GDE1	1.0658568849	0.6668607039	0.9999763851
CCP110	-1.0468046711	0.8372220359	0.9999763851
C16orf62	-1.2572963427	0.3142199126	0.9999763851
KNOP1	-1.0974585998	0.6550976306	0.9999763851
IQCK	1.1908637081	0.8036880892	0.9999763851
GPRC5B	2.3407592913	0.4264645427	0.9999763851
ACSM5	0	0	0
ACSM1	-4.2849908816	0.4028453076	0.9999763851
THUMPD1	1.236387244	0.265591576	0.9999763851
ACSM3	0	0	0
ERI2	1.0035685331	0.9893075266	0.9999763851
LOC81691	1.1443480656	0.7389554061	0.9999763851
DCUN1D3	-1.3622162529	0.437935214	0.9999763851
LYRM1	1.0632691158	0.8219976702	0.9999763851
TMEM159	-1.6303129344	0.293061738	0.9999763851
CRYM	0	0	0
NPIPB3	0	0	0
METTL9	1.4043451064	0.0190263261	0.9999763851
OTOA	-1.0351581998	0.9578210637	0.9999763851
UQCRC2	-1.0060444589	0.9684350326	0.9999763851
PDZD9	5.4770877341	0.3746135179	0.9999763851
C16orf52	1.2556219454	0.4527397015	0.9999763851
VWA3A	-4.2849908816	0.4028453076	0.9999763851
SDR42E2	0	0	0
EEF2K	-1.0750102471	0.6986637985	0.9999763851
POLR3E	1.3607879792	0.182809921	0.9999763851

CDR2	1.6377558733	0.0507530284	0.9999763851
NPIPB5	-1.5561499223	0.2237663546	0.9999763851
LOC107984851	10.231382017	0.2261759961	0.9999763851
HS3ST2	1.0623586752	0.7971252751	0.9999763851
USP31	-1.2346620905	0.4159489864	0.9999763851
COG7	-1.0865877167	0.8072712281	0.9999763851
GGA2	-1.1851574011	0.3421985886	0.9999763851
EARS2	-1.4014576459	0.321075952	0.9999763851
UBFD1	-1.5901305471	0.0651096642	0.9999763851
NDUFAB1	1.0617126774	0.6474379872	0.9999763851
PALB2	-1.1107403175	0.719691592	0.9999763851
DCTN5	-1.052809328	0.7991346401	0.9999763851
PLK1	-1.0498230818	0.7704871316	0.9999763851
PRKCB	1.0512573724	0.6919716922	0.9999763851
RBBP6	-1.2086489254	0.1983663808	0.9999763851
TNRC6A	1.0365424575	0.8379281788	0.9999763851
SLC5A11	-4.3352959826	0.1433550293	0.9999763851
ARHGAP17	1.141277847	0.6111652593	0.9999763851
LCMT1	1.2792434359	0.365211968	0.9999763851
ZKSCAN2	-2.0563269266	0.3431899436	0.9999763851
HS3ST4	1.0271487798	0.9778343721	0.9999763851
KDM8	-1.0543030667	0.9163413891	0.9999763851
NSMCE1	1.0284273312	0.8858595052	0.9999763851
IL4R	1.0651160478	0.688300917	0.9999763851
IL21R	-1.5329190378	0.5267840422	0.9999763851
GTF3C1	-1.012673157	0.9493973703	0.9999763851
KIAA0556	-1.2781210127	0.7682070384	0.9999763851
GSG1L	0	0	0
XPO6	1.0710867194	0.7037754626	0.9999763851
SBK1	2.2735484122	0.0757901906	0.9999763851
NPIPB6	14.3882523344	0.1834756444	0.9999763851
EIF3CL	1.0002235821	0.9991794598	0.9999763851
CLN3	1.1643219308	0.4508234884	0.9999763851
APOBR	-1.2335633808	0.4575807767	0.9999763851
IL27	5.4790129495	0.376569852	0.9999763851
NUPR1	3.2356557963	0.5547505991	0.9999763851
SGF29	-1.1984251502	0.5241615395	0.9999763851
SULT1A2	0	0	0
SULT1A1	-1.2627066896	0.1517417558	0.9999763851
NPIPB8	0	0	0
EIF3C	1.9803336519	0.007139	0.9999763851
NPIPB9	1.2396974231	0.7289048746	0.9999763851
ATXN2L	-1.1419585324	0.5345814203	0.9999763851
TUFM	-1.1133651351	0.4104598314	0.9999763851
SH2B1	1.1765503364	0.4654682002	0.9999763851
ATP2A1	1.2744858108	0.4980689778	0.9999763851
RABEP2	1.1445056083	0.7561803815	0.9999763851
CD19	1.0757570521	0.8767986127	0.9999763851
NFATC2IP	-1.2533248505	0.2699235424	0.9999763851

SPNS1	1.4652718213	0.3390014976	0.9999763851
LAT	-1.405180248	0.1043476394	0.9999763851
LOC107984832	-1.0874314895	0.8222602559	0.9999763851
NPIPB11	-1.3581901144	0.5199396722	0.9999763851
BOLA2-SMG1P6	-1.0046112753	0.9808492816	0.9999763851
BOLA2	1.2905287366	0.2590561076	0.9999763851
SLX1B	1.158290244	0.5743977522	0.9999763851
SULT1A4	-2.1237124164	0.1138711062	0.9999763851
SPN	-1.007605927	0.9639982709	0.9999763851
QPRT	-1.0334370292	0.8304858742	0.9999763851
C16orf54	1.471094976	0.2137934358	0.9999763851
KIF22	1.2275450497	0.3010181947	0.9999763851
MAZ	-1.2125697723	0.2437650282	0.9999763851
PRRT2	3.2356557963	0.5547505991	0.9999763851
PAGR1	-1.2002296341	0.2380425159	0.9999763851
MVP	-1.2837924324	0.7540149713	0.9999763851
CDIPT	1.1521562548	0.5019422052	0.9999763851
SEZ6L2	-1.7051915203	0.020358759	0.9999763851
ASPHD1	-1.0053000015	0.9917008245	0.9999763851
KCTD13	1.1061700908	0.7684962146	0.9999763851
TMEM219	-1.3309675751	0.1176341303	0.9999763851
TAOK2	-1.2582927336	0.5581329347	0.9999763851
HIRIP3	1.6501236438	0.0996907815	0.9999763851
INO80E	1.7161788927	0.2779766859	0.9999763851
DOC2A	-1.1616645801	0.5846330003	0.9999763851
C16orf92	0	0	0
ALDOA	-1.0692696351	0.6253106041	0.9999763851
PPP4C	-1.4405208139	0.182202088	0.9999763851
TBX6	1.8136032532	0.5931965901	0.9999763851
YPEL3	1.0875204518	0.8524940683	0.9999763851
GDPD3	1.1707681772	0.6575169146	0.9999763851
MAPK3	1.1039763567	0.6879721207	0.9999763851
CORO1A	1.2938710133	0.0404992847	0.9999763851
BOLA2B	1.3977108366	0.1349232907	0.9999763851
SLX1A	1.1399060968	0.6403113486	0.9999763851
SULT1A3	2.8642644956	0.0574470559	0.9999763851
LOC613037	-3.2709536258	0.1436203394	0.9999763851
CD2BP2	-1.0017322143	0.993774963	0.9999763851
TBC1D10B	1.0741491662	0.7935623449	0.9999763851
MYLPF	-1.0167501853	0.9768439533	0.9999763851
SEPT1	-1.7725705758	0.2892585841	0.9999763851
ZNF48	-1.1072837095	0.7074698057	0.9999763851
ZNF771	-1.9776151526	0.1205691429	0.9999763851
DCTPP1	1.3647147032	0.0416363252	0.9999763851
SEPHS2	1.2780659236	0.0830553176	0.9999763851
ITGAL	1.0732754703	0.7658399804	0.9999763851
ZNF768	-1.0579207406	0.7868301349	0.9999763851
ZNF747	-1.1650784505	0.8440812259	0.9999763851
ZNF764	-1.0861299175	0.8714928775	0.9999763851

ZNF688	-1.4318485396	0.5083015153	0.9999763851
ZNF785	-2.2054783554	0.1352122226	0.9999763851
ZNF689	1.2046670653	0.5122284892	0.9999763851
PRR14	1.3066994432	0.3350393634	0.9999763851
FBRS	1.7218443259	0.0217179631	0.9999763851
LOC730183	-2.2385982899	0.2960934568	0.9999763851
SRCAP	1.0752626087	0.8651516287	0.9999763851
TMEM265	2.3083799844	0.1549062382	0.9999763851
PHKG2	1.1678783373	0.7064204423	0.9999763851
CCDC189	-1.4910755477	0.2852526381	0.9999763851
RNF40	1.60132526	0.0383232778	0.9999763851
ZNF629	-1.0296909279	0.9336641585	0.9999763851
BCL7C	-1.5281672709	0.1395671118	0.9999763851
CTF1	0	0	0
FBXL19	-1.1774901235	0.5689535814	0.9999763851
ORAI3	-1.2465002924	0.3229889699	0.9999763851
SETD1A	-1.4289230219	0.1010017143	0.9999763851
HSD3B7	-4.2849908816	0.4028453076	0.9999763851
STX4	1.0228608943	0.912623003	0.9999763851
ZNF668	-1.1247852448	0.7773221481	0.9999763851
ZNF646	1.3241637928	0.4750385688	0.9999763851
PRSS53	1.2867653957	0.8300179538	0.9999763851
VKORC1	1.0027937231	0.9857212161	0.9999763851
BCKDK	1.3153126416	0.2809663169	0.9999763851
KAT8	1.0492086771	0.8197177097	0.9999763851
PRSS36	3.2361532718	0.5546934395	0.9999763851
FUS	1.3212793553	0.0390303837	0.9999763851
PYCARD	-1.1131203687	0.6188824606	0.9999763851
ITGAM	1.0291407439	0.926893828	0.9999763851
ITGAX	-2.8468892561	0.0268812909	0.9999763851
ITGAD	-4.2849908816	0.4028453076	0.9999763851
ARMC5	1.3124769122	0.1393597552	0.9999763851
TGFB1I1	-1.2767981988	0.6536366834	0.9999763851
SLC5A2	-1.4185994118	0.1694242056	0.9999763851
C16orf58	7.6089345734	0.0381452559	0.9999763851
ZNF720	1.5251117717	0.2776037019	0.9999763851
ZNF267	-1.6621400061	0.0647704893	0.9999763851
SHCBP1	-1.0568975437	0.7643891364	0.9999763851
VPS35	1.3143900426	0.0630622114	0.9999763851
ORC6	1.06593017	0.7552700535	0.9999763851
C16orf87	1.8604390903	0.1907744327	0.9999763851
GPT2	-1.2132523032	0.2222077888	0.9999763851
DNAJA2	1.2692492838	0.2802859784	0.9999763851
NETO2	-1.3001100519	0.29413876	0.9999763851
ITFG1	-1.1835122402	0.328392921	0.9999763851
PHKB	1.4679355433	0.1131607932	0.9999763851
ABCC11	-4.2849908816	0.4028453076	0.9999763851
LONP2	1.2716409777	0.2289855293	0.9999763851
SIAH1	-1.3042104128	0.3015585315	0.9999763851

N4BP1	1.148449662	0.5655659713	0.9999763851
CNEP1R1	-1.0355551732	0.9614598426	0.9999763851
HEATR3	1.2828776604	0.1838657094	0.9999763851
PAPD5	1.0613876049	0.7642755019	0.9999763851
ADCY7	1.0212958619	0.9319620858	0.9999763851
BRD7	1.0090391145	0.9517377319	0.9999763851
NKD1	-1.5730895081	0.49983691	0.9999763851
SNX20	-1.3570916218	0.4839550286	0.9999763851
NOD2	1.3420325123	0.6975998781	0.9999763851
CYLD	-1.0644237681	0.8225274063	0.9999763851
CHD9	-1.0385032728	0.8727509631	0.9999763851
LOC643802	3.2356557963	0.5547505991	0.9999763851
RBL2	-1.0983280931	0.7759953984	0.9999763851
AKTIP	-1.1966685147	0.6178015989	0.9999763851
RPGRIP1L	1.7962512653	0.2964238608	0.9999763851
FTO	1.1076976324	0.6107027743	0.9999763851
IRX3	-1.2670355964	0.2116743639	0.9999763851
CRNDE	1.0177227776	0.9257644365	0.9999763851
IRX5	1.0625249051	0.897368082	0.9999763851
MMP2	-1.6884118522	0.2189759046	0.9999763851
LPCAT2	-1.2131434911	0.3433990505	0.9999763851
CES1	-1.2519275495	0.2353195657	0.9999763851
AMFR	-1.0501479855	0.8498767415	0.9999763851
NUDT21	-1.2409161599	0.2490331997	0.9999763851
OGFOD1	1.5550603304	0.0397643384	0.9999763851
BBS2	1.2047897185	0.446038062	0.9999763851
MT2A	1.112490146	0.4849809333	0.9999763851
MT1E	-1.317161512	0.0845050407	0.9999763851
MT1F	1.0299718578	0.9137329146	0.9999763851
MT1G	1.132070048	0.4667125107	0.9999763851
MT1X	-1.3393974676	0.2532686854	0.9999763851
NUP93	1.1158054623	0.4148978972	0.9999763851
HERPUD1	-1.0280014612	0.8896518339	0.9999763851
CETP	0	0	0
NLRC5	-1.2394438584	0.2840613238	0.9999763851
CPNE2	1.0264769189	0.9022498335	0.9999763851
FAM192A	1.1289990301	0.5710400258	0.9999763851
RSPRY1	1.4028746246	0.2961345335	0.9999763851
ARL2BP	-1.6968818127	0.032004934	0.9999763851
CCL22	3.2356557963	0.5547505991	0.9999763851
CCL17	0	0	0
CIAPIN1	1.1319494705	0.597012216	0.9999763851
COQ9	1.1159689206	0.7712865204	0.9999763851
POLR2C	-1.1053704912	0.5915616434	0.9999763851
DOK4	-1.3625919741	0.4346640864	0.9999763851
CCDC102A	1.4559388088	0.4565764204	0.9999763851
ADGRG5	1.2254274439	0.7467288428	0.9999763851
ADGRG1	0	0	0
ADGRG3	-3.0007254032	0.0622552446	0.9999763851

KATNB1	1.3766737223	0.2284758319	0.9999763851
KIFC3	16.6918753579	0.1320089484	0.9999763851
CNGB1	5.4781708352	0.3745527127	0.9999763851
ZNF319	1.6276641846	0.2246328247	0.9999763851
USB1	1.1773118019	0.3920911698	0.9999763851
MMP15	1.367312239	0.467678077	0.9999763851
CFAP20	1.0338112889	0.8412422474	0.9999763851
CSNK2A2	-1.1869734549	0.3030477501	0.9999763851
CCDC113	1.2869488883	0.821092998	0.9999763851
PRSS54	-1.3714374731	0.7332709577	0.9999763851
GINS3	1.2689990134	0.2909128651	0.9999763851
NDRG4	3.1827248461	0.1129407392	0.9999763851
SETD6	-1.0684586477	0.7544828105	0.9999763851
CNOT1	1.0845163416	0.6179082097	0.9999763851
SLC38A7	1.5890074071	0.4231488651	0.9999763851
GOT2	1.1739130499	0.2831761744	0.9999763851
BEAN1	-4.2849908816	0.4028453076	0.9999763851
TK2	-1.4148698791	0.4021213002	0.9999763851
CKLF	-1.2739843359	0.1066837763	0.9999763851
CMTM1	-1.6644409499	0.0611960983	0.9999763851
CMTM2	3.2356557963	0.5547505991	0.9999763851
CMTM4	-1.2509437445	0.4500849279	0.9999763851
CMTM3	1.4596957905	0.0215056788	0.9999763851
DYNC1LI2	1.0277188775	0.9053577794	0.9999763851
TERB1	0	0	0
NAE1	1.1491538314	0.3591858936	0.9999763851
PDP2	1.1276351151	0.5607630168	0.9999763851
RRAD	0	0	0
FAM96B	1.1893246022	0.3036379211	0.9999763851
CES2	1.1652777522	0.5260152044	0.9999763851
CES3	1.164363665	0.7454379348	0.9999763851
CES4A	3.2353357581	0.554787377	0.9999763851
CBFB	1.2792720509	0.1608033682	0.9999763851
C16orf70	-1.4276067727	0.5506213428	0.9999763851
B3GNT9	1.0159553944	0.9674854292	0.9999763851
TRADD	1.1186688471	0.725177035	0.9999763851
FBXL8	5.4781708352	0.3745527127	0.9999763851
NOL3	-1.0872048538	0.8636920486	0.9999763851
KIAA0895L	2.1207130064	0.1438653947	0.9999763851
EXOC3L1	0	0	0
E2F4	1.1476882968	0.4056008269	0.9999763851
ELMO3	-1.0362251892	0.9602994275	0.9999763851
LRRC29	0	0	0
LOC105369155	-3.3272796812	0.2424955191	0.9999763851
TMEM208	1.0594206605	0.7820243101	0.9999763851
FHOD1	-1.1158591381	0.4692712154	0.9999763851
SLC9A5	1.0955921063	0.8993695964	0.9999763851
PLEKHG4	1.054664986	0.8204331037	0.9999763851
KCTD19	-8.342239448	0.2147985685	0.9999763851

LRRC36	3.2356557963	0.5547505991	0.9999763851
TPPP3	3.2356557963	0.5547505991	0.9999763851
ATP6V0D1	-1.2368112955	0.4975663831	0.9999763851
AGRP	-4.2849908816	0.4028453076	0.9999763851
FAM65A	-1.1355325181	0.6262530807	0.9999763851
LOC107984813	0	0	0
CTCF	1.1783612825	0.3774134623	0.9999763851
CARMIL2	-4.2849908816	0.4028453076	0.9999763851
ACD	-1.1036621065	0.7235580681	0.9999763851
PARD6A	1.3829444468	0.6360363249	0.9999763851
ENKD1	-1.1461750681	0.6420011317	0.9999763851
C16orf86	-1.3154339304	0.8205576571	0.9999763851
GFOD2	1.0594645413	0.8311543128	0.9999763851
RANBP10	-1.3408992778	0.1871054102	0.9999763851
TSNAXIP1	-1.1982896796	0.2170494683	0.9999763851
CENPT	-1.5141799262	0.3173209968	0.9999763851
THAP11	-1.0406045361	0.8065582985	0.9999763851
NUTF2	-1.0779157431	0.7059793302	0.9999763851
EDC4	-1.0809745187	0.6156161	0.9999763851
NRN1L	1.2872739558	0.8229961387	0.9999763851
PSKH1	-1.2446907532	0.3015177265	0.9999763851
CTRL	1.1081639824	0.7318903874	0.9999763851
PSMB10	1.0724592139	0.6905621261	0.9999763851
LCAT	-2.3209115874	0.4596723125	0.9999763851
SLC12A4	-1.4304003311	0.569588286	0.9999763851
DPEP2	-1.9306444188	0.3794725725	0.9999763851
DDX28	1.2702395184	0.2802455181	0.9999763851
DUS2	1.4070953692	0.3175209247	0.9999763851
NFATC3	-1.1139346016	0.4683715147	0.9999763851
ESRP2	-1.0208803598	0.9124381823	0.9999763851
PLA2G15	-1.6496225342	0.0884417053	0.9999763851
SLC7A6	1.4286998782	0.2351809258	0.9999763851
SLC7A6OS	-1.0460045128	0.8744731485	0.9999763851
PRMT7	-1.0275940386	0.9217410347	0.9999763851
SMPD3	1.041027979	0.9582907678	0.9999763851
ZFP90	1.4060713105	0.3666968526	0.9999763851
CDH3	9.0182935529	0.2609674114	0.9999763851
CDH1	0	0	0
TANGO6	-1.0877352973	0.8348999467	0.9999763851
HAS3	1.1562207354	0.3747441924	0.9999763851
CHTF8	-2.2066914541	0.3421276514	0.9999763851
UTP4	1.8725298335	0.003185	0.9999763851
SNTB2	-1.7037707755	0.2002314089	0.9999763851
VPS4A	-1.127281704	0.4877933072	0.9999763851
PDF	1.084201425	0.6517830251	0.9999763851
COG8	1.512982915	0.3749393992	0.9999763851
NIP7	1.2150079594	0.4030261025	0.9999763851
TMED6	-1.7383115404	0.2659013851	0.9999763851
TERF2	1.0075482427	0.9662086143	0.9999763851

CYB5B	1.0300475451	0.8775579876	0.9999763851
NFAT5	-1.1474882311	0.5644548571	0.9999763851
NQO1	1.1104434052	0.5333787769	0.9999763851
NOB1	1.0917219753	0.6235660186	0.9999763851
WWP2	-1.0528262407	0.7736859843	0.9999763851
PDPR	-1.0371000208	0.8685895746	0.9999763851
CLEC18C	5.4776292235	0.3745831165	0.9999763851
LOC107984138	1.2847127328	0.3830454116	0.9999763851
EXOSC6	1.0337016061	0.8905187403	0.9999763851
AARS	1.0218976378	0.9212748614	0.9999763851
DDX19B	1.9282328564	0.044682014	0.9999763851
DDX19A	1.3915109285	0.1063380268	0.9999763851
ST3GAL2	-1.5861199845	0.0630002649	0.9999763851
FUK	1.4582509317	0.3118333737	0.9999763851
COG4	-1.2354058875	0.587670106	0.9999763851
SF3B3	1.1521447994	0.385888672	0.9999763851
IL34	3.2353357581	0.554787377	0.9999763851
MTSS1L	5.4776292235	0.3745831165	0.9999763851
VAC14	-1.3977241544	0.3241088061	0.9999763851
HYDIN	-2.9413009608	0.1738340989	0.9999763851
CMTR2	1.3298922017	0.3447550053	0.9999763851
ZNF23	-2.4440355816	0.4429677948	0.9999763851
TAT	0	0	0
MARVELD3	0	0	0
PHLPP2	1.0882934963	0.8549309515	0.9999763851
AP1G1	-1.4542722784	0.0761787373	0.9999763851
ATXN1L	-1.0432573948	0.8964668994	0.9999763851
ZNF821	-2.1524685389	0.0416364764	0.9999763851
IST1	1.0485181386	0.7481576976	0.9999763851
PKD1L3	5.4770877341	0.3746135179	0.9999763851
DHODH	1.2405841432	0.3982637473	0.9999763851
TXNL4B	1.0337503277	0.9353428307	0.9999763851
HP	-1.620272684	0.4305429354	0.9999763851
HPR	0	0	0
DHX38	-1.1306646719	0.5021436339	0.9999763851
PMFBP1	1.027554661	0.9756370564	0.9999763851
LOC107984814	-7.5622178268	0.2177145568	0.9999763851
ZFHX3	-1.1945980561	0.2965049	0.9999763851
PSMD7	1.0866327228	0.5756408936	0.9999763851
NPIPB15	-1.9285516304	0.4974475048	0.9999763851
CLEC18B	0	0	0
GLG1	-1.0630873983	0.6628587521	0.9999763851
RFWD3	-1.1222519806	0.5285949729	0.9999763851
MLKL	1.4444564486	0.2010852286	0.9999763851
FA2H	5.4776292235	0.3745831165	0.9999763851
WDR59	-1.5758582453	0.1001297294	0.9999763851
ZNRF1	1.2331864921	0.2985334421	0.9999763851
LDHD	-4.2849908816	0.4028453076	0.9999763851
ZFP1	2.5902663417	0.1883429208	0.9999763851

CFDP1	-1.1065122731	0.6185594296	0.9999763851
TMEM170A	1.2016783547	0.4905421418	0.9999763851
CHST5	-1.3158816768	0.8203229797	0.9999763851
TMEM231	-2.2871333017	0.1127327287	0.9999763851
GABARAPL2	-1.0880431678	0.5799621223	0.9999763851
ADAT1	1.1201594825	0.6872012934	0.9999763851
KARS	1.1477100849	0.3407732386	0.9999763851
TERF2IP	-1.0002069302	0.9988252984	0.9999763851
CNTNAP4	1.0981889172	0.7314521061	0.9999763851
MON1B	2.6013599918	0.2508681234	0.9999763851
SYCE1L	1.0350683151	0.8534258609	0.9999763851
ADAMTS18	0	0	0
NUDT7	1.0895551508	0.8552430632	0.9999763851
VAT1L	0	0	0
WWOX	-1.1440753219	0.4223930498	0.9999763851
DYNLRB2	0	0	0
CDYL2	-1.2838111839	0.3620566672	0.9999763851
CMC2	1.3325414652	0.2186226989	0.9999763851
CENPN	-1.1122291502	0.7345284208	0.9999763851
ATMIN	1.2744846658	0.2684639782	0.9999763851
C16orf46	1.3505403582	0.6843337746	0.9999763851
GCSH	1.0947926752	0.6365138581	0.9999763851
PKD1L2	0	0	0
BCO1	3.2353357581	0.554787377	0.9999763851
GAN	-2.196793761	0.0988727129	0.9999763851
CMIP	-1.1129042172	0.5341615109	0.9999763851
PLCG2	-1.1751889714	0.3603480324	0.9999763851
HSD17B2	0	0	0
MPHOSPH6	1.0630453121	0.6805983626	0.9999763851
HSBP1	1.09707018	0.5160321205	0.9999763851
MLYCD	1.0645599416	0.839359949	0.9999763851
LOC105371371	-1.3154339304	0.8205576571	0.9999763851
OSGIN1	1.1853645663	0.7830833345	0.9999763851
NECAB2	0	0	0
MBTPS1	1.1844574891	0.3504294451	0.9999763851
HSDL1	1.0312683987	0.9048830426	0.9999763851
DNAAF1	3.2353357581	0.554787377	0.9999763851
TAF1C	-1.0108914973	0.9619176432	0.9999763851
KCNG4	0	0	0
WFDC1	-1.2865931055	0.7364990454	0.9999763851
ATP2C2	-2.3893004408	0.2387205476	0.9999763851
TLDC1	-1.8724972875	0.1626913659	0.9999763851
COTL1	-1.1180082884	0.3495379183	0.9999763851
KLHL36	-1.0578326959	0.7688221933	0.9999763851
USP10	1.0207891179	0.9019607456	0.9999763851
ZDHHC7	1.0752308913	0.6251397585	0.9999763851
KIAA0513	-1.554288553	0.3423427355	0.9999763851
GSE1	-1.2811436126	0.2763045468	0.9999763851
GINS2	1.0647384453	0.6896858851	0.9999763851

C16orf74	-1.1999599755	0.2540986074	0.9999763851
EMC8	1.1746422046	0.339171823	0.9999763851
COX4I1	1.2609797481	0.0718006453	0.9999763851
IRF8	-1.8692701989	0.102096509	0.9999763851
FOXF1	-1.2762952295	0.669588532	0.9999763851
MTHFSD	1.1818304825	0.5301502246	0.9999763851
FOXL1	0	0	0
C16orf95	-1.21567076	0.5518037188	0.9999763851
FBXO31	-1.3566039499	0.2427074331	0.9999763851
MAP1LC3B	1.0182129829	0.9148772218	0.9999763851
ZCCHC14	-1.368381082	0.2511384251	0.9999763851
KLHDC4	1.1529184678	0.5882618517	0.9999763851
LOC105371397	0	0	0
SLC7A5	-1.0806116659	0.7009602738	0.9999763851
CA5A	-4.2849908816	0.4028453076	0.9999763851
BANP	-1.5221452646	0.1348481309	0.9999763851
LOC107984862	0	0	0
ZNF469	-1.8727248054	0.3026340166	0.9999763851
ZFPM1	5.8993332406	0.0789660619	0.9999763851
ZC3H18	1.2228940656	0.361211411	0.9999763851
CYBA	1.5142168573	0.1690943423	0.9999763851
MVD	1.1058210883	0.615690914	0.9999763851
SNAI3	-1.047573144	0.8822422776	0.9999763851
RNF166	-1.3385991307	0.1353656807	0.9999763851
CTU2	1.0561722097	0.8522571444	0.9999763851
PIEZO1	-1.202551081	0.3527691277	0.9999763851
CDT1	1.0430994381	0.8177790142	0.9999763851
APRT	1.0517268197	0.7356044131	0.9999763851
GALNS	-1.3550833716	0.2535111902	0.9999763851
TRAPPC2L	1.0511554219	0.7926974051	0.9999763851
PABPN1L	-7.5664585246	0.2257565985	0.9999763851
CBFA2T3	1.0347578143	0.8906405406	0.9999763851
LOC100129697	0	0	0
ACSF3	2.0365389983	0.0966431959	0.9999763851
CDH15	-4.2864494036	0.4027150576	0.9999763851
ZNF778	1.1833479949	0.7221336636	0.9999763851
ANKRD11	1.0935009085	0.5305311692	0.9999763851
LOC100287036	-4.2849908816	0.4028453076	0.9999763851
SPG7	1.1649467242	0.4438074421	0.9999763851
RPL13	-1.0331751359	0.7834245229	0.9999763851
CPNE7	-1.3765878235	0.2430668564	0.9999763851
DPEP1	1.6135829349	0.0231730331	0.9999763851
CHMP1A	-1.2107723729	0.2697813648	0.9999763851
SPATA33	-1.008394933	0.9806441135	0.9999763851
CDK10	-1.0434279787	0.8531638765	0.9999763851
SPATA2L	1.027554661	0.9756469146	0.9999763851
VPS9D1	1.4260670756	0.4259126361	0.9999763851
ZNF276	1.0563761465	0.804768624	0.9999763851
FANCA	1.2344847171	0.4670237474	0.9999763851

SPIRE2	-1.9652419871	0.4566429162	0.9999763851
LOC105371419	3.2353357581	0.554787377	0.9999763851
TCF25	-1.0452663996	0.7443533772	0.9999763851
MC1R	1.1805116415	0.8039812948	0.9999763851
TUBB3	1.1202669996	0.6967485496	0.9999763851
DEF8	1.0034782893	0.9863238104	0.9999763851
CENPBD1	1.6049013466	0.4086307377	0.9999763851
DBNDD1	1.6703467261	0.4625480201	0.9999763851
GAS8	1.8750770526	0.25604441	0.9999763851
RPH3AL	-2.1310892017	0.1502195489	0.9999763851
C17orf97	-1.3155492632	0.8260459548	0.9999763851
FAM101B	1.2449563557	0.0848566964	0.9999763851
VPS53	-1.0357656165	0.8557482009	0.9999763851
FAM57A	1.166470252	0.4854253603	0.9999763851
GEMIN4	1.4043185766	0.0531386179	0.9999763851
GLOD4	-1.0378436564	0.8799919122	0.9999763851
MRM3	1.4163066528	0.2780719106	0.9999763851
NXN	-1.0356702413	0.8705022513	0.9999763851
TIMM22	1.1659395787	0.5507184626	0.9999763851
ABR	1.2889550051	0.1144442003	0.9999763851
TUSC5	0	0	0
YWHAE	1.1377347436	0.3268336829	0.9999763851
CRK	1.2938234034	0.2997366351	0.9999763851
MYO1C	1.2382051676	0.610298108	0.9999763851
INPP5K	1.4795907957	0.3450579579	0.9999763851
PITPNA	-1.2016837152	0.2384417492	0.9999763851
SLC43A2	1.3417513497	0.1007281925	0.9999763851
SCARF1	1.2910285137	0.5964736895	0.9999763851
RILP	-3.9975329737	0.1323617233	0.9999763851
PRPF8	-1.0898250667	0.5755031603	0.9999763851
TLCD2	3.2353357581	0.554787377	0.9999763851
WDR81	2.4020525162	0.0450870124	0.9999763851
SERPINF1	1.5509282945	0.6031372669	0.9999763851
SMYD4	-1.0469642615	0.9029572353	0.9999763851
RPA1	1.1043769593	0.4875694741	0.9999763851
RTN4RL1	-2.2967284599	0.279282876	0.9999763851
DPH1	1.8136231599	0.5627419009	0.9999763851
OVCA2	1.6213132362	0.004871	0.9999763851
HIC1	1.0295394469	0.9676292791	0.9999763851
SMG6	1.1884806434	0.4201353894	0.9999763851
LOC107984987	5.4784711963	0.3745358536	0.9999763851
SRR	1.1220936442	0.4406922342	0.9999763851
TSR1	-1.6616773163	0.0659998913	0.9999763851
SGSM2	1.2062089301	0.3646613886	0.9999763851
MNT	1.5047239003	0.1228827216	0.9999763851
METTL16	1.0307646113	0.91040169	0.9999763851
PAFAH1B1	1.0055129112	0.9691172034	0.9999763851
CLUH	-1.0873310146	0.6014845777	0.9999763851
RAP1GAP2	-1.0400675265	0.8700522583	0.9999763851

OR3A2	0	0	0
OR3A1	-4.2849908816	0.4028453076	0.9999763851
TRPV3	7.7192231741	0.2782707334	0.9999763851
TRPV1	2.3578986999	0.3419975027	0.9999763851
SHPK	-1.7263681129	0.3312162762	0.9999763851
CTNS	1.2726668757	0.5634864189	0.9999763851
TAX1BP3	-1.0623828521	0.8352751541	0.9999763851
EMC6	1.1224892719	0.4445429565	0.9999763851
P2RX5	1.9045919035	0.0182425713	0.9999763851
ITGAE	-1.0371829675	0.8363048652	0.9999763851
GSG2	25.650992429	0.0841903384	0.9999763851
NCBP3	1.1467551549	0.5236532407	0.9999763851
CAMKK1	-1.3608741329	0.3253583551	0.9999763851
P2RX1	-1.1599215708	0.5869484827	0.9999763851
ATP2A3	1.3184397896	0.0806087399	0.9999763851
ZZEF1	-1.0322618059	0.9097578017	0.9999763851
CYB5D2	1.7806067509	0.2556717637	0.9999763851
ANKFY1	-1.2710074703	0.1573456451	0.9999763851
UBE2G1	1.3458858674	0.0435697936	0.9999763851
SPNS3	-1.3332616972	0.2529690736	0.9999763851
SPNS2	-1.587874259	0.1841104925	0.9999763851
MYBBP1A	1.5177144553	0.021705639	0.9999763851
GGT6	0	0	0
ALOX15	0	0	0
PELP1	1.2444255102	0.3915263939	0.9999763851
ARRB2	1.067660055	0.6437414324	0.9999763851
MED11	2.7752654288	0.0358051927	0.9999763851
CXCL16	-4.3450459626	0.0778003224	0.9999763851
VMO1	2.2182046909	0.3722715638	0.9999763851
GLTPD2	0	0	0
PSMB6	1.3700853553	0.0620544435	0.9999763851
LOC107984973	3.2356557963	0.5547505991	0.9999763851
PLD2	-1.1962473193	0.7840961715	0.9999763851
MINK1	-1.4168415622	0.6037121917	0.9999763851
CHRNE	3.2356557963	0.5547505991	0.9999763851
GP1BA	0	0	0
SLC25A11	1.4034914671	0.0709150967	0.9999763851
RNF167	-1.1076181067	0.6567974638	0.9999763851
PFN1	-1.1117907055	0.3985500119	0.9999763851
ENO3	-1.2573057306	0.7191499136	0.9999763851
SPAG7	-1.494661231	0.007849	0.9999763851
CAMTA2	-1.315575163	0.3235854627	0.9999763851
INCA1	-1.7867779362	0.2303716025	0.9999763851
KIF1C	-1.1943066477	0.2027356947	0.9999763851
RABEP1	1.1063010599	0.6249159724	0.9999763851
NUP88	1.1182429931	0.5858219518	0.9999763851
RPAIN	1.001790201	0.9929530304	0.9999763851
C1QBP	1.0748440441	0.656471164	0.9999763851
DHX33	-1.208778901	0.3953986611	0.9999763851

DERL2	1.2309232496	0.4047618484	0.9999763851
MIS12	1.8154932902	0.0428756917	0.9999763851
LOC728392	0	0	0
NLRP1	-7.5622178268	0.2177145568	0.9999763851
FAM64A	1.0251087726	0.9030937961	0.9999763851
PITPNM3	3.2353357581	0.554787377	0.9999763851
KIAA0753	-2.2682534019	0.0752763358	0.9999763851
TXNDC17	1.149510245	0.3435900249	0.9999763851
MED31	2.3487446974	0.0230111655	0.9999763851
C17orf100	-1.3158816768	0.8203229797	0.9999763851
XAF1	-4.2864494036	0.4027150576	0.9999763851
ALOX12	1.6252006195	0.2416235007	0.9999763851
RNASEK	-1.0839492302	0.6334789237	0.9999763851
C17orf49	1.2299905445	0.3179648694	0.9999763851
SLC16A13	3.2356557963	0.5547505991	0.9999763851
CLEC10A	0	0	0
ASGR2	1.028107434	0.9764144245	0.9999763851
ASGR1	-10.8385246611	0.1451600336	0.9999763851
DLG4	-1.26626789	0.2478183086	0.9999763851
ACADVL	-1.1007634179	0.528710155	0.9999763851
DVL2	-1.09493033	0.6965881004	0.9999763851
PHF23	1.1559790358	0.5260044895	0.9999763851
GABARAP	-1.0007085033	0.9960303032	0.9999763851
CTDNEP1	-1.0314894316	0.8097361546	0.9999763851
ELP5	-1.6351635971	0.0392673368	0.9999763851
CLDN7	3.2356557963	0.5547505991	0.9999763851
SLC2A4	2.8674942058	0.3049740122	0.9999763851
EIF5A	1.1361544233	0.3698292759	0.9999763851
GPS2	1.6250860079	0.0426969959	0.9999763851
NEURL4	-1.1431502971	0.7678763112	0.9999763851
ACAP1	-1.058631225	0.806391799	0.9999763851
KCTD11	1.1334473729	0.8684459396	0.9999763851
TNK1	3.2361532718	0.5546934395	0.9999763851
PLSCR3	1.2315392431	0.2426822386	0.9999763851
TMEM256	-1.1798269106	0.3714173155	0.9999763851
NLGN2	-3.0813597757	0.123465347	0.9999763851
TMEM102	0	0	0
FGF11	3.3950799507	0.2318810884	0.9999763851
CHRNB1	-1.276380235	0.6853195504	0.9999763851
ZBTB4	-1.2535687009	0.4368303274	0.9999763851
POLR2A	-1.185551348	0.2570515677	0.9999763851
TNFSF12	0	0	0
TNFSF13	1.6148394316	0.0505309222	0.9999763851
SENP3	-1.4499772591	0.5897886748	0.9999763851
EIF4A1	1.180118723	0.186423255	0.9999763851
CD68	1.5778108037	0.4331455547	0.9999763851
LOC100996842	-4.2864494036	0.4027150576	0.9999763851
MPDU1	-1.0978650545	0.6488320838	0.9999763851
SOX15	-2.3225701307	0.4434167901	0.9999763851

FXR2	-1.3334910313	0.168964667	0.9999763851
SHBG	-4.2864494036	0.4027150576	0.9999763851
SAT2	1.031218952	0.8633971849	0.9999763851
TP53	1.5604866468	0.2706534128	0.9999763851
WRAP53	-1.0813907267	0.924225171	0.9999763851
DNAH2	0	0	0
KDM6B	1.1452440163	0.6895147262	0.9999763851
NAA38	1.019950292	0.8835780975	0.9999763851
CYB5D1	1.2609309139	0.4475463014	0.9999763851
CHD3	-1.0186765896	0.9077291841	0.9999763851
KCNAB3	1.2872739558	0.8230180306	0.9999763851
TRAPPC1	1.5540792477	0.11862788	0.9999763851
CNTROB	1.633055077	0.1360843272	0.9999763851
GUCY2D	7.7488609396	0.3059167339	0.9999763851
ALOX12B	1.9234638322	0.4260239582	0.9999763851
HES7	1.0115359816	0.9884109369	0.9999763851
PER1	1.0520225176	0.8433671935	0.9999763851
VAMP2	-2.5355197317	0.0120780553	0.9999763851
TMEM107	-1.0760002437	0.8180053101	0.9999763851
BORCS6	1.3356309462	0.2582181506	0.9999763851
AURKB	-1.0056498774	0.9712301663	0.9999763851
CTC1	1.4674853957	0.4791134648	0.9999763851
PFAS	1.7213613948	0.004664	0.9999763851
RANGRF	-1.0959549601	0.5375995608	0.9999763851
SLC25A35	-2.0606615507	0.2983602424	0.9999763851
KRBA2	-1.3158816768	0.8203229797	0.9999763851
RPL26	-1.0349100946	0.7844978127	0.9999763851
NDEL1	1.1545372228	0.5135332867	0.9999763851
MYH10	-1.8146129166	0.140684327	0.9999763851
CCDC42	3.2361532718	0.5546934395	0.9999763851
PIK3R6	-2.387886984	0.2112259006	0.9999763851
PIK3R5	-1.0036279234	0.9844814841	0.9999763851
NTN1	-1.6698105334	0.2616609649	0.9999763851
STX8	1.3310953909	0.2114730191	0.9999763851
CFAP52	0	0	0
DHRS7C	0	0	0
GSG1L2	3.2353357581	0.554787377	0.9999763851
GAS7	-1.1341222204	0.4394662756	0.9999763851
MYH4	0	0	0
MYH3	-1.0156237173	0.9578991223	0.9999763851
SCO1	1.2228422576	0.2454025338	0.9999763851
ADPRM	-1.5708165304	0.3885522438	0.9999763851
TMEM220	-1.1957366748	0.804418173	0.9999763851
SHISA6	1.2869488883	0.8211494027	0.9999763851
DNAH9	-3.3264391549	0.2738511788	0.9999763851
ZNF18	1.4229779878	0.3967872523	0.9999763851
MAP2K4	-1.0739578835	0.732437054	0.9999763851
ARHGAP44	-1.3944153431	0.6946124935	0.9999763851
ELAC2	1.138559278	0.5309129731	0.9999763851

COX10	1.0567705373	0.8390352295	0.9999763851
CDRT4	0	0	0
CDRT1	-7.5622178268	0.2177145568	0.9999763851
TRIM16	1.2718294823	0.4389567361	0.9999763851
ZNF286A	1.2174484142	0.2092501952	0.9999763851
ADORA2B	1.9228097209	0.4268417327	0.9999763851
ZSWIM7	-1.0091597961	0.9656406783	0.9999763851
TTC19	-1.121568196	0.4223356804	0.9999763851
NCOR1	-1.0308489887	0.8830282425	0.9999763851
PIGL	-1.1963614536	0.5297679889	0.9999763851
CENPV	-1.1244193605	0.4593569342	0.9999763851
UBB	1.0497786882	0.6972290186	0.9999763851
TRPV2	1.3261522439	0.3097807283	0.9999763851
LRRC75A	-3.2225510108	0.001582	0.9999763851
ZNF287	0	0	0
CCDC144A	-1.0118618877	0.9600691232	0.9999763851
MPRIP	1.0836002603	0.6652340073	0.9999763851
PLD6	1.3620430594	0.137892508	0.9999763851
FLCN	-1.302442452	0.1543508838	0.9999763851
COPS3	-1.1043562603	0.5361168892	0.9999763851
NT5M	1.2786230139	0.4325388468	0.9999763851
MED9	1.0598196992	0.7919596393	0.9999763851
PEMT	1.016846405	0.9624176122	0.9999763851
RAI1	1.258116452	0.2899616324	0.9999763851
SREBF1	1.0468481076	0.8490145467	0.9999763851
TOM1L2	-1.414491976	0.3141241806	0.9999763851
DRC3	1.2355274324	0.7159989804	0.9999763851
ATPAF2	1.2800234422	0.4857267176	0.9999763851
GID4	-2.7035192643	0.0410183252	0.9999763851
DRG2	2.1110724278	0.0131352871	0.9999763851
MYO15A	-1.3716482783	0.7378342759	0.9999763851
ALKBH5	1.036729118	0.8332761405	0.9999763851
LLGL1	-1.4162370021	0.1292888555	0.9999763851
FLII	1.3033011074	0.1700273162	0.9999763851
MIEF2	3.4224740954	0.0615853663	0.9999763851
TOP3A	1.2667035848	0.3674613794	0.9999763851
SMCR8	1.6827913576	0.0890406028	0.9999763851
SHMT1	-1.854712782	0.0531111933	0.9999763851
LGALS9C	1.3585372872	0.569561054	0.9999763851
ZNF286B	1.5964182648	0.3573082571	0.9999763851
TRIM16L	-1.1934908034	0.51785316	0.9999763851
FBXW10	3.2353357581	0.554787377	0.9999763851
TVP23B	-1.1860931861	0.5867283512	0.9999763851
LOC101929141	3.2356557963	0.5547505991	0.9999763851
PRPSAP2	-1.9491322084	0.006352	0.9999763851
FAM83G	-1.4082372984	0.500276742	0.9999763851
GRAP	7.7199860138	0.2831153382	0.9999763851
B9D1	1.0019491055	0.9965746579	0.9999763851
MAPK7	2.1739784176	0.2850503355	0.9999763851

MFAP4	-1.3154339304	0.8205576571	0.9999763851
RNF112	3.2356557963	0.5547505991	0.9999763851
ALDH3A2	3.2353357581	0.554787377	0.9999763851
SLC47A2	-4.2849908816	0.4028453076	0.9999763851
AKAP10	1.0869995648	0.8147815169	0.9999763851
SPECC1	-1.3904212186	0.3233314668	0.9999763851
LGALS9B	0	0	0
CDRT15L2	-4.2849908816	0.4028453076	0.9999763851
LOC105369205	-1.1501339038	0.2929746317	0.9999763851
USP22	-1.6196855671	0.4334704695	0.9999763851
DHRS7B	1.530068871	0.1011956651	0.9999763851
TMEM11	1.0282854549	0.8619597267	0.9999763851
NATD1	-1.8172198407	0.4513898593	0.9999763851
MAP2K3	-1.3054097359	0.0493622288	0.9999763851
C17orf51	2.34099053	0.3997152508	0.9999763851
MTRNR2L1	-1.4490890341	0.006544	0.9999763851
WSB1	-1.1169360047	0.4942771814	0.9999763851
KSR1	-1.1454514418	0.7172311881	0.9999763851
LGALS9	1.1221804086	0.499802388	0.9999763851
LYRM9	2.4683190578	0.1241697105	0.9999763851
NLK	1.0998128519	0.7715430396	0.9999763851
LOC105371930	3.2356557963	0.5547505991	0.9999763851
TMEM97	1.1299880241	0.5083016692	0.9999763851
IFT20	-1.5445282614	0.3855316116	0.9999763851
TNFAIP1	1.8995141861	0.2648648392	0.9999763851
POLDIP2	1.0399044543	0.8303986863	0.9999763851
TMEM199	-1.0697739241	0.8160846839	0.9999763851
SARM1	1.1336585844	0.8771625718	0.9999763851
SLC46A1	-1.4267084394	0.623413526	0.9999763851
UNC119	1.1090242499	0.6163488351	0.9999763851
PIGS	1.1134395574	0.4493189701	0.9999763851
ALDOC	-1.0499879077	0.9360410035	0.9999763851
SPAG5	-1.0906009033	0.735093317	0.9999763851
SGK494	1.09599993	0.8958262722	0.9999763851
KIAA0100	1.266002603	0.167246694	0.9999763851
SDF2	-1.1400302879	0.4891071303	0.9999763851
SUPT6H	1.0035345536	0.9894233975	0.9999763851
PROCA1	-1.6376683773	0.2791192326	0.9999763851
RPL23A	-1.2672124045	0.1601284757	0.9999763851
TLCD1	2.8676515213	0.2915201778	0.9999763851
NEK8	1.508933517	0.5739834686	0.9999763851
TRAF4	-1.0694967919	0.8116685143	0.9999763851
FAM222B	-1.0271989054	0.9309187743	0.9999763851
ERAL1	1.2206570197	0.5027602187	0.9999763851
FLOT2	-1.0211215605	0.9185266363	0.9999763851
DHRS13	-1.5030417283	0.223833105	0.9999763851
PHF12	1.2438927194	0.5906089796	0.9999763851
PIPOX	-7.5647903763	0.2176246281	0.9999763851
MYO18A	-1.2944792306	0.6141463268	0.9999763851

TIAF1	1.0031999999	0.9886334311	0.9999763851
CRYBA1	0	0	0
NUFIP2	1.0551027384	0.7651691101	0.9999763851
TAOK1	1.0519294002	0.7753879062	0.9999763851
ABHD15	1.7034063944	0.1955093224	0.9999763851
TP53I13	1.1639313698	0.6520939686	0.9999763851
GIT1	1.369503506	0.259641286	0.9999763851
ANKRD13B	1.3615191234	0.5601183957	0.9999763851
CORO6	1.8136032532	0.5927398692	0.9999763851
SSH2	-1.3777067009	0.0892763601	0.9999763851
NSRP1	1.3089906873	0.2554373386	0.9999763851
SLC6A4	0	0	0
CPD	-1.0073353425	0.9606768812	0.9999763851
GOSR1	-1.0526190304	0.8197410088	0.9999763851
TBC1D29	-1.3714374731	0.7332684715	0.9999763851
CRLF3	-1.2656425068	0.3582812988	0.9999763851
ATAD5	-1.0351154379	0.8976354938	0.9999763851
TEFM	1.0818349382	0.7936794896	0.9999763851
RNF135	1.036671475	0.8654883948	0.9999763851
LOC107984974	1.3253731754	0.7579223892	0.9999763851
NF1	1.4440222127	0.1564929694	0.9999763851
EVI2B	1.6339767567	0.2552096059	0.9999763851
EVI2A	-2.0785652782	0.0130283404	0.9999763851
RAB11FIP4	-1.0339674486	0.8737993194	0.9999763851
COPRS	1.2267026038	0.1659983693	0.9999763851
UTP6	1.0941886947	0.6533387653	0.9999763851
SUZ12	1.1358684156	0.3797163899	0.9999763851
LOC105371727	0	0	0
LRRC37B	1.1122687244	0.6688190726	0.9999763851
LOC107985082	-4.2849908816	0.4028453076	0.9999763851
RHOT1	-1.0403633808	0.8460095901	0.9999763851
RHBDL3	-2.3225701307	0.443414764	0.9999763851
C17orf75	1.4337164916	0.4195789468	0.9999763851
ZNF207	1.1681281935	0.2881130147	0.9999763851
PSMD11	-1.1434687784	0.4314210548	0.9999763851
CDK5R1	1.0036282455	0.986925407	0.9999763851
MYO1D	1.6249017024	0.5572207845	0.9999763851
SPACA3	0	0	0
ASIC2	-1.467826005	0.6619417583	0.9999763851
CCL2	-1.2431650002	0.1158390549	0.9999763851
CCL7	-12.9532379654	3.51E-11	2.64E-07
CCL8	-14.164430006	0.0118012851	0.9999763851
CCL1	-13.9077333422	2.28E-09	9.43E-06
CCT6B	1.0259413652	0.9598924492	0.9999763851
ZNF830	1.1835489739	0.5825606899	0.9999763851
LIG3	-1.3586906184	0.1481826135	0.9999763851
RFFL	-1.1800351275	0.5292762177	0.9999763851
RAD51D	-1.4372601129	0.4195901531	0.9999763851
FNDC8	-1.7348876098	0.4534177332	0.9999763851

NLE1	1.6324231799	0.094434495	0.9999763851
LOC105371932	-2.3235904983	0.4581477541	0.9999763851
SLFN5	-3.166158432	0.1247178772	0.9999763851
SLFN11	1.0573998774	0.7811898902	0.9999763851
LOC105371933	-2.3214980365	0.4373473522	0.9999763851
SLFN12	-1.2494129491	0.5649197672	0.9999763851
SLFN13	-1.7868207301	0.1977253734	0.9999763851
PEX12	1.8129446953	0.5691176486	0.9999763851
AP2B1	1.2247064781	0.3074840832	0.9999763851
C17orf50	-1.3154339304	0.8205576571	0.9999763851
TAF15	1.4418883205	0.0137563014	0.9999763851
HEATR9	3.3940373312	0.2270373552	0.9999763851
CCL5	-1.1008057266	0.5450032139	0.9999763851
RDM1	-1.6049296996	0.2582909529	0.9999763851
CCL23	2.3398344857	0.4133791656	0.9999763851
CCL18	0	0	0
CCL3	-17.6103194045	2.57E-12	3.46E-08
CCL4	-5.4163258644	0.001709	0.9999763851
CCL3L3	-12.9839108308	1.06E-08	3.57E-05
CCL4L2	-14.7252461469	0.0977604827	0.9999763851
TBC1D3H	3.2361532718	0.5546934395	0.9999763851
ZNHIT3	1.1059604441	0.5283470985	0.9999763851
MYO19	-1.4488052739	0.118354408	0.9999763851
PIGW	1.0742851968	0.7351507398	0.9999763851
GGNBP2	1.1005958916	0.5800821903	0.9999763851
DHRS11	1.3718703568	0.413193369	0.9999763851
MRM1	1.0733799428	0.8218783912	0.9999763851
AATF	-1.048396059	0.7483766532	0.9999763851
ACACA	1.4011565592	0.0775677589	0.9999763851
TADA2A	1.1799018915	0.5626929604	0.9999763851
DUSP14	-1.1003088294	0.7330855418	0.9999763851
SYNRG	1.0209056748	0.9166391119	0.9999763851
DDX52	1.9499859725	0.005227	0.9999763851
HNF1B	0	0	0
LOC101929950	-2.6922490556	0.2893715837	0.9999763851
TBC1D3E	1.4698296658	0.3354510495	0.9999763851
TBC1D3	0	0	0
MRPL45	-1.207217016	0.3464195062	0.9999763851
SOCS7	-1.1815679737	0.4217915766	0.9999763851
ARHGAP23	-1.0707153004	0.8626449646	0.9999763851
SRCIN1	2.8679346912	0.2849286157	0.9999763851
C17orf96	1.0100106634	0.9509923982	0.9999763851
LOC105371763	1.1889819001	0.6619838725	0.9999763851
MLLT6	-1.1346159767	0.5502563098	0.9999763851
CISD3	1.0148091922	0.9372065718	0.9999763851
PCGF2	5.4770877341	0.3746135179	0.9999763851
PSMB3	1.1557818862	0.5369166203	0.9999763851
PIP4K2B	1.2610997688	0.2073659094	0.9999763851
CWC25	1.0791614234	0.7629704486	0.9999763851

C17orf98	0	0	0
RPL23	-1.0215666948	0.8696214561	0.9999763851
LASP1	-1.2708239979	0.0533396306	0.9999763851
FBXO47	3.2356557963	0.5547505991	0.9999763851
PLXDC1	1.0045143155	0.9957708879	0.9999763851
ARL5C	5.4770877341	0.3746135179	0.9999763851
CACNB1	-10.8458971147	0.1433027679	0.9999763851
RPL19	1.0764230667	0.5414152114	0.9999763851
FBXL20	1.1700961976	0.6048180548	0.9999763851
MED1	-1.2324529637	0.2071331913	0.9999763851
CDK12	1.0352802988	0.8722802606	0.9999763851
NEUROD2	0	0	0
STARD3	1.0499909904	0.7675908889	0.9999763851
TCAP	0	0	0
PGAP3	-1.6609687825	0.225525288	0.9999763851
MIEN1	1.1091700675	0.6840660913	0.9999763851
GRB7	0	0	0
IKZF3	-4.2849908816	0.4028453076	0.9999763851
GSDMB	-14.1139127893	0.1033851165	0.9999763851
ORMDL3	1.1076665828	0.6293113138	0.9999763851
LRRC3C	-4.287394647	0.4026306792	0.9999763851
GSDMA	3.2356557963	0.5547505991	0.9999763851
PSMD3	1.0395211043	0.7777450577	0.9999763851
CSF3	0	0	0
MED24	1.0435275421	0.7618615459	0.9999763851
THRA	-1.2365090914	0.1320108594	0.9999763851
MSL1	-1.0751710674	0.6470181117	0.9999763851
CASC3	-1.0960367898	0.6103525429	0.9999763851
RAPGEFL1	-1.315303824	0.8206171563	0.9999763851
WIPF2	-1.3628222188	0.0752083329	0.9999763851
CDC6	-1.003623917	0.9844097	0.9999763851
RARA	1.1169451538	0.7702722309	0.9999763851
TOP2A	-1.0640367134	0.6071274505	0.9999763851
IGFBP4	-2.3401094535	0.2609856653	0.9999763851
TNS4	0	0	0
CCR7	1.2869488883	0.8212066554	0.9999763851
SMARCE1	-1.3833508898	0.0500687425	0.9999763851
KRT222	0	0	0
KRT25	0	0	0
KRT10	1.0237809329	0.8768576682	0.9999763851
TMEM99	-1.7952455218	0.1427630397	0.9999763851
KRT19	0	0	0
KRT9	-1.2461435291	0.4620754759	0.9999763851
KRT17	-2.06231313	0.3313568767	0.9999763851
EIF1	1.0399823804	0.7502798809	0.9999763851
JUP	-1.0399161003	0.8452131433	0.9999763851
P3H4	1.0542635479	0.7848359029	0.9999763851
FKBP10	0	0	0
NT5C3B	1.4119559467	0.1997901694	0.9999763851

KLHL10	6.368054416	0.3492169917	0.9999763851
KLHL11	-1.170772176	0.5645557816	0.9999763851
ACLY	1.1987262702	0.1727408773	0.9999763851
TTC25	-1.7358601187	0.4443221278	0.9999763851
CNP	1.0357298083	0.8188519096	0.9999763851
DNAJC7	1.0886084528	0.8452955492	0.9999763851
NKIRAS2	1.0646073048	0.802415491	0.9999763851
DHX58	0	0	0
KAT2A	1.1054298992	0.6218793988	0.9999763851
HSPB9	0	0	0
RAB5C	-1.2256506757	0.1603709551	0.9999763851
KCNH4	0	0	0
HCRT	-4.2849908816	0.4028453076	0.9999763851
GHDC	1.4202922713	0.3947032046	0.9999763851
STAT5B	-1.3123575227	0.2752368204	0.9999763851
STAT5A	-1.2743490152	0.244807045	0.9999763851
STAT3	-1.1752124293	0.5042935906	0.9999763851
ATP6V0A1	7.7488609396	0.3058954712	0.9999763851
NAGLU	1.3416512829	0.1501168336	0.9999763851
HSD17B1	1.3012534193	0.4826225785	0.9999763851
COASY	1.3066405269	0.1443738879	0.9999763851
MLX	-1.1340166139	0.6741374227	0.9999763851
PSMC3IP	1.1526628378	0.8652492972	0.9999763851
FAM134C	-1.1945402121	0.5431208618	0.9999763851
TUBG1	1.1397126095	0.626047407	0.9999763851
TUBG2	0	0	0
PLEKHH3	1.1450830729	0.756697014	0.9999763851
CCR10	-14.1139127893	0.1035179751	0.9999763851
CNTNAP1	1.1095153459	0.8982805878	0.9999763851
EZH1	-1.2571363067	0.5678241157	0.9999763851
VPS25	1.0203774656	0.9168909617	0.9999763851
WNK4	2.3408621302	0.428191081	0.9999763851
COA3	1.5335957987	0.0354154381	0.9999763851
CNTD1	-1.191042134	0.3739164963	0.9999763851
BECN1	95.2169782749	0.020473733	0.9999763851
PSME3	1.1723721653	0.306301255	0.9999763851
AOC2	7.7204093521	0.287657111	0.9999763851
AOC3	-1.9652419871	0.4561425173	0.9999763851
G6PC	0	0	0
AARSD1	-1.3767562248	0.1640118821	0.9999763851
PTGES3L-AARSD1	3.2361532718	0.5546934395	0.9999763851
PTGES3L	-1.4166067094	0.6341659675	0.9999763851
RUNDC1	-1.1754889166	0.5789881894	0.9999763851
RPL27	1.0589976191	0.6396559735	0.9999763851
IFI35	-10.8422096127	0.1421302158	0.9999763851
VAT1	-1.05767269	0.6506037656	0.9999763851
BRCA1	1.0894002801	0.6109947754	0.9999763851
NBR1	-1.3802187527	0.1525368497	0.9999763851
TMEM106A	1.1166943097	0.7788160019	0.9999763851

ARL4D	-2.0102635635	0.2120053943	0.9999763851
DHX8	-1.3079890236	0.3410844044	0.9999763851
ETV4	1.6192525572	0.1267673744	0.9999763851
DUSP3	-1.3382225061	0.196368155	0.9999763851
C17orf105	-4.2849908816	0.4028453076	0.9999763851
MPP3	-3.3269505881	0.2608501707	0.9999763851
PYY	-1.5455442631	0.3604848352	0.9999763851
TMEM101	-1.0233608878	0.9332403775	0.9999763851
LSM12	-1.5960709051	0.004057	0.9999763851
G6PC3	1.2564042319	0.594724224	0.9999763851
HDAC5	-2.6440243018	0.0142538131	0.9999763851
C17orf53	1.3319720943	0.5089082667	0.9999763851
ASB16	-1.1241910911	0.8432827727	0.9999763851
TMUB2	-1.0897317093	0.7907810642	0.9999763851
ATXN7L3	-1.3374394544	0.069450966	0.9999763851
UBTF	1.0367267784	0.8113230865	0.9999763851
RUNDC3A	0	0	0
SLC25A39	1.0804394103	0.6201121307	0.9999763851
GRN	-1.0024513187	0.9881060716	0.9999763851
FAM171A2	1.222945565	0.5694149912	0.9999763851
GPATCH8	1.3501414444	0.1338150361	0.9999763851
FZD2	-2.5587132929	0.0933655759	0.9999763851
MEIOC	2.8186446591	0.1926593969	0.9999763851
CCDC43	-1.2875371507	0.3032849826	0.9999763851
DBF4B	1.3153052107	0.3508845456	0.9999763851
ADAM11	-1.8150683874	0.5018445411	0.9999763851
GJC1	0	0	0
HIGD1B	3.4162861017	0.1308425662	0.9999763851
EFTUD2	1.0326193658	0.8369687972	0.9999763851
CCDC103	1.6246521587	0.5738699216	0.9999763851
GFAP	0	0	0
KIF18B	-1.1903616839	0.4151608807	0.9999763851
C1QL1	-1.3157515262	0.8224985603	0.9999763851
LOC107987243	1.523256191	0.5847125344	0.9999763851
DCAKD	-1.1094327092	0.7763014869	0.9999763851
NMT1	1.2712449208	0.1504294082	0.9999763851
PLCD3	-1.2887895783	0.5908308782	0.9999763851
ACBD4	-1.406518047	0.6841405251	0.9999763851
HEXIM1	-1.2788649105	0.2356832065	0.9999763851
HEXIM2	1.2865109866	0.8338841024	0.9999763851
FMNL1	-1.0948916497	0.7261203456	0.9999763851
SPATA32	0	0	0
MAP3K14	1.3585694454	0.5622502806	0.9999763851
ARHGAP27	1.3000097871	0.2265919269	0.9999763851
PLEKHM1	-1.1585461795	0.719978444	0.9999763851
MGC57346-CRHR1	-1.0802533536	0.7829779462	0.9999763851
MAPT	1.0615241038	0.8137644691	0.9999763851
KANSL1	-1.196791613	0.3631340361	0.9999763851
LRRC37A	-1.3103107816	0.272793767	0.9999763851

ARL17B	-10.7322924268	4.82E-05	0.0927102426
ARL17A	1.5503523123	0.0356333545	0.9999763851
LRRC37A2	-66.4111511063	0.007734	0.9999763851
NSF	1.0152855734	0.9000823576	0.9999763851
WNT3	1.2868359435	0.8210197937	0.9999763851
GOSR2	1.0159371566	0.9385881466	0.9999763851
RPRML	0	0	0
CDC27	-1.1683393756	0.3334248928	0.9999763851
ITGB3	3.2356557963	0.5547505991	0.9999763851
EFCAB13	-1.0873921633	0.8201629532	0.9999763851
NPEPPS	1.1075626917	0.5334323518	0.9999763851
KPNB1	1.0036295374	0.9763370992	0.9999763851
TBKBP1	-1.5558662382	0.2038210095	0.9999763851
TBX21	5.4770877341	0.3746135179	0.9999763851
OSBPL7	-1.0599217033	0.8801588024	0.9999763851
MRPL10	-1.2697754829	0.3495224202	0.9999763851
LRRC46	-1.3154339304	0.8205576571	0.9999763851
SCRN2	-1.1188213207	0.7006464392	0.9999763851
SP2	1.0867964178	0.820319231	0.9999763851
PNPO	-1.1913010768	0.2529779258	0.9999763851
CDK5RAP3	-1.1426043575	0.4527221952	0.9999763851
NFE2L1	-1.0451053376	0.8224873622	0.9999763851
CBX1	1.2252250926	0.2602942312	0.9999763851
SNX11	1.4359829547	0.2102083709	0.9999763851
SKAP1	5.4790129495	0.3768260133	0.9999763851
HOXB3	3.2356557963	0.5547505991	0.9999763851
HOXB4	0	0	0
HOXB6	0	0	0
TTLL6	-4.2849908816	0.4028453076	0.9999763851
CALCOCO2	1.0321021876	0.8580290964	0.9999763851
ATP5G1	-1.0438725929	0.7516376007	0.9999763851
UBE2Z	-1.0034275075	0.9866267474	0.9999763851
SNF8	-1.3350818357	0.1477940669	0.9999763851
GIP	0	0	0
IGF2BP1	3.2353357581	0.554787377	0.9999763851
B4GALNT2	0	0	0
GNGT2	0	0	0
ABI3	-1.7526708531	0.4695612548	0.9999763851
PHOSPHO1	0	0	0
ZNF652	-1.0841997218	0.5347893593	0.9999763851
PHB	1.1572248427	0.3422185415	0.9999763851
SPOP	-1.4701792421	0.0156184295	0.9999763851
SLC35B1	1.1517103588	0.5151933111	0.9999763851
FAM117A	-1.6421689323	0.1126963516	0.9999763851
KAT7	1.4238860596	0.1457870429	0.9999763851
TAC4	0	0	0
FLJ45513	3.2356557963	0.5547505991	0.9999763851
DLX4	-1.3471391661	0.527115492	0.9999763851
ITGA3	-2.5593586246	0.2687178723	0.9999763851

PDK2	1.4166727518	0.3927535453	0.9999763851
SAMD14	-1.3157515262	0.8225209718	0.9999763851
PPP1R9B	1.4728802061	0.1859053176	0.9999763851
SGCA	3.2361532718	0.5546934395	0.9999763851
COL1A1	1.0277462901	0.9757606161	0.9999763851
TMEM92	-1.4076805062	0.6614174686	0.9999763851
XYLT2	-1.1596625696	0.5741192914	0.9999763851
MRPL27	1.0791781399	0.6374391291	0.9999763851
EME1	1.4194606792	0.1208147082	0.9999763851
LRRC59	1.0792194461	0.6986870827	0.9999763851
ACSF2	-1.3618862658	0.2957116187	0.9999763851
RSAD1	1.0424672271	0.8357512347	0.9999763851
EPN3	0	0	0
SPATA20	1.5338540238	0.3341716736	0.9999763851
ABCC3	-4.439622732	0.1608087207	0.9999763851
ANKRD40	-1.0761702759	0.8158323987	0.9999763851
LUC7L3	-1.1791702156	0.2627328648	0.9999763851
TOB1	1.0194321817	0.9740724285	0.9999763851
SPAG9	-1.1166419185	0.5957712143	0.9999763851
NME1-NME2	3.6490389733	0.005738	0.9999763851
NME1	1.031271857	0.8227417022	0.9999763851
NME2	-1.0390559375	0.7557857132	0.9999763851
MBTD1	-1.6335310538	0.0786078153	0.9999763851
UTP18	1.1488545714	0.4994847656	0.9999763851
CA10	3.2353357581	0.554787377	0.9999763851
COX11	1.0635918753	0.728393867	0.9999763851
STXBP4	-1.8159051241	0.1283322844	0.9999763851
HLF	14.4518735001	0.1558556665	0.9999763851
MMD	1.1213013009	0.6953673088	0.9999763851
TMEM100	0	0	0
PCTP	-1.1958756174	0.4285990759	0.9999763851
ANKFN1	3.2353357581	0.554787377	0.9999763851
NOG	1.0625615059	0.8992436519	0.9999763851
C17orf67	-1.4388607299	0.4662668392	0.9999763851
DGKE	-1.3460110406	0.2035289537	0.9999763851
TRIM25	-1.2574523527	0.1327616845	0.9999763851
COIL	-1.3955957376	0.0751667733	0.9999763851
SCPEP1	1.8085324452	0.1811903705	0.9999763851
AKAP1	1.2116960743	0.2739390077	0.9999763851
MSI2	-1.7304294588	0.003928	0.9999763851
MRPS23	1.2320422494	0.2025225315	0.9999763851
CUEDC1	1.3357167892	0.5014721262	0.9999763851
VEZF1	1.2194007659	0.26652107	0.9999763851
SRSF1	-1.385474219	0.013870896	0.9999763851
DYNLL2	-1.0807084955	0.7965937641	0.9999763851
EPX	-1.3943388833	0.6974268411	0.9999763851
MKS1	1.0037564108	0.9931419805	0.9999763851
LPO	5.4784711963	0.3745358536	0.9999763851
MPO	-4.2849908816	0.4028453076	0.9999763851

TSPOAP1	-4.2849908816	0.4028453076	0.9999763851
SUPT4H1	-1.2170614071	0.1335504044	0.9999763851
RNF43	1.0738836724	0.7664621396	0.9999763851
HSF5	0	0	0
MTMR4	-1.170513748	0.3151713619	0.9999763851
TEX14	-4.2864494036	0.4027150576	0.9999763851
RAD51C	-1.1091664288	0.6191959388	0.9999763851
PPM1E	3.2353357581	0.554787377	0.9999763851
TRIM37	1.1416446224	0.5484804353	0.9999763851
SKA2	1.0027181747	0.9873525805	0.9999763851
PRR11	1.0009019956	0.9951755513	0.9999763851
SMG8	-1.2863920245	0.1722477791	0.9999763851
GDPD1	-1.4076293613	0.6576393166	0.9999763851
YPEL2	-3.3900767704	0.0006754	0.6376579103
DHX40	1.1721161655	0.4458883532	0.9999763851
CLTC	-1.0763256042	0.5504841057	0.9999763851
PTRH2	1.5378949554	0.072808	0.9999763851
VMP1	-1.0413773281	0.8774799168	0.9999763851
TUBD1	1.1411653845	0.5554011251	0.9999763851
RPS6KB1	1.0620306757	0.813678936	0.9999763851
RNFT1	1.6681561475	0.4996074955	0.9999763851
HEATR6	1.1146484645	0.6933103203	0.9999763851
USP32	-1.3048347316	0.2244717033	0.9999763851
C17orf64	-1.4281695322	0.5302130795	0.9999763851
APPBP2	-1.1322703841	0.6624606562	0.9999763851
PPM1D	1.3257277328	0.2966120482	0.9999763851
BCAS3	1.0314357805	0.8675629671	0.9999763851
TBX4	0	0	0
NACA2	1.0500641291	0.7410342079	0.9999763851
BRIP1	1.0783690276	0.7851707421	0.9999763851
INTS2	1.611485542	0.2537316629	0.9999763851
MED13	1.1438871918	0.5825867542	0.9999763851
EFCAB3	3.2353357581	0.554787377	0.9999763851
METTL2A	-1.007937192	0.9780050303	0.9999763851
TLK2	-1.1872516466	0.3546495846	0.9999763851
MRC2	-1.2706018552	0.1609364621	0.9999763851
TANC2	1.1450832573	0.7437900081	0.9999763851
KCNH6	0	0	0
DCAF7	-1.1685069018	0.3381330906	0.9999763851
TACO1	-1.0507861217	0.8173164314	0.9999763851
MAP3K3	-1.5395862987	0.1816499329	0.9999763851
LIMD2	1.2764977549	0.2569277466	0.9999763851
STRADA	1.7466959631	0.1068332325	0.9999763851
CCDC47	1.261568972	0.1591795738	0.9999763851
DDX42	1.3351148823	0.1764650744	0.9999763851
FTSJ3	-1.181734384	0.538495871	0.9999763851
PSMC5	1.1056608389	0.5703133009	0.9999763851
SMARCD2	1.0082469566	0.9561715307	0.9999763851
CD79B	1.094680312	0.9298789058	0.9999763851

PRR29	-2.4384604498	0.004352	0.9999763851
ICAM2	2.8677508622	0.0260718734	0.9999763851
ERN1	-1.7163870119	0.0573970097	0.9999763851
TEX2	1.2859765675	0.2162367541	0.9999763851
PECAM1	-2.4085187422	0.0002638	0.3301211415
MILR1	-1.9665787608	0.450663293	0.9999763851
POLG2	-2.5014873462	0.0658656921	0.9999763851
DDX5	1.1752632563	0.2169227488	0.9999763851
CEP95	1.0985384731	0.748821251	0.9999763851
SMURF2	-1.4800104493	0.1651715063	0.9999763851
LRRC37A3	-1.5778537154	0.1526225869	0.9999763851
GNA13	-1.3101417765	0.2005730372	0.9999763851
RGS9	1.8139617088	0.5798476583	0.9999763851
AXIN2	12.2012745411	0.1883263981	0.9999763851
PRKCA	-4.2849908816	0.4028453076	0.9999763851
CACNG1	0	0	0
HELZ	-1.1156903389	0.6127510599	0.9999763851
PSMD12	1.2573073716	0.1823343025	0.9999763851
PITPNC1	-1.2593300907	0.1526737039	0.9999763851
NOL11	1.1244027211	0.449704627	0.9999763851
BPTF	-1.1588585304	0.4248513281	0.9999763851
C17orf58	1.2185643604	0.450905043	0.9999763851
KPNA2	1.2242515848	0.1151816203	0.9999763851
AMZ2	-1.1086294121	0.5285429094	0.9999763851
ARSG	1.3631350465	0.5236987364	0.9999763851
SLC16A6	-1.2785467372	0.6689930072	0.9999763851
PRKAR1A	-1.167505528	0.3208269551	0.9999763851
WIPI1	1.4169878943	0.3696390264	0.9999763851
FAM20A	0	0	0
ABCA8	0	0	0
ABCA9	0	0	0
MAP2K6	-3.0838658458	0.1375299849	0.9999763851
SLC39A11	-1.1386598353	0.3613927247	0.9999763851
SSTR2	1.8139019215	0.5824613813	0.9999763851
COG1	1.2515691339	0.6278785591	0.9999763851
FAM104A	-1.135602	0.5350106964	0.9999763851
C17orf80	1.0694478451	0.8833536893	0.9999763851
CPSF4L	-1.5229205493	0.4617274286	0.9999763851
CDC42EP4	2.3029443907	0.1002902997	0.9999763851
SDK2	0	0	0
RPL38	-1.0143018709	0.9098905487	0.9999763851
TTYH2	-6.3450869775	0.0583047746	0.9999763851
KIF19	3.2353357581	0.554787377	0.9999763851
GPRC5C	1.1293468666	0.6309874511	0.9999763851
CD300A	1.1012193558	0.7145045659	0.9999763851
CD300LB	1.3676001091	0.4638766938	0.9999763851
CD300C	-1.8805051931	0.066916264	0.9999763851
LOC100130520	3.2353357581	0.554787377	0.9999763851
CD300E	0	0	0

RAB37	1.3621691	0.1164686567	0.9999763851
CD300LF	-1.2384709082	0.2280057426	0.9999763851
SLC9A3R1	1.0936977062	0.5309851788	0.9999763851
NAT9	-1.1720017138	0.6901013187	0.9999763851
TMEM104	1.139610238	0.4889235223	0.9999763851
GRIN2C	1.7554673993	0.479297055	0.9999763851
FDXR	-1.119831722	0.6801991635	0.9999763851
CDR2L	-1.450325375	0.1052699842	0.9999763851
ICT1	1.2047320057	0.3297505937	0.9999763851
KCTD2	-1.1216682835	0.6518896821	0.9999763851
ATP5H	1.0051076379	0.9785483619	0.9999763851
SLC16A5	-1.1258156018	0.7808757233	0.9999763851
ARMC7	1.1275157128	0.7666537206	0.9999763851
NT5C	-1.2201487575	0.4844217624	0.9999763851
HN1	1.3178170654	0.0387620258	0.9999763851
SUMO2	1.2155486113	0.1495463053	0.9999763851
NUP85	-1.5784688329	0.1951245379	0.9999763851
GGA3	-1.0219114763	0.8977765063	0.9999763851
MRPS7	1.1631272425	0.3079149475	0.9999763851
MIF4GD	-1.0781049508	0.7448054521	0.9999763851
SLC25A19	1.0368491199	0.876805636	0.9999763851
GRB2	1.2279305246	0.1102982332	0.9999763851
TMEM94	-1.6545598694	0.0760921455	0.9999763851
CASKIN2	-2.481317684	0.1590172943	0.9999763851
TSEN54	1.0356058542	0.8559899728	0.9999763851
LLGL2	-1.4393973942	0.4211652208	0.9999763851
MYO15B	-1.0470082974	0.9199051466	0.9999763851
RECQL5	-1.5856285156	0.3670025194	0.9999763851
SMIM5	0	0	0
SMIM6	1.2865109866	0.8339574889	0.9999763851
SAP30BP	-1.2436491145	0.3468366601	0.9999763851
ITGB4	-10.371672165	0.0142517641	0.9999763851
GALK1	-1.248361254	0.3579667898	0.9999763851
H3F3B	1.0022949672	0.9841393382	0.9999763851
UNK	-1.248061582	0.2977937211	0.9999763851
UNC13D	-1.2346984267	0.3622146135	0.9999763851
WBP2	1.0074425751	0.9628863593	0.9999763851
TRIM47	0	0	0
TRIM65	1.432567934	0.0829948425	0.9999763851
MRPL38	-1.7987138531	0.0144475727	0.9999763851
FBF1	-2.2143932241	0.24856486	0.9999763851
ACOX1	-1.115204261	0.6066359367	0.9999763851
TEN1	1.1756185533	0.6547039784	0.9999763851
CDK3	1.0324501895	0.9523751626	0.9999763851
SRP68	1.0229648789	0.8944932124	0.9999763851
GALR2	-1.3161718537	0.8292852722	0.9999763851
ZACN	-1.0656213907	0.7332831655	0.9999763851
EXOC7	-1.4401565523	0.3983523357	0.9999763851
RNF157	-1.8129967537	0.4216496909	0.9999763851

UBALD2	-1.2313785434	0.1381059307	0.9999763851
QRICH2	1.5602490925	0.6804593908	0.9999763851
PRPSAP1	1.1646300979	0.4438327987	0.9999763851
SPHK1	-1.0857981264	0.8001436871	0.9999763851
UBE2O	1.0509444478	0.8166301874	0.9999763851
AANAT	3.2353357581	0.554787377	0.9999763851
RHBDF2	1.0132034058	0.9568305091	0.9999763851
CYGB	0	0	0
PRCD	2.3408621302	0.4283701132	0.9999763851
ST6GALNAC2	-1.3344418569	0.7451003495	0.9999763851
MXRA7	-1.294055056	0.6171079393	0.9999763851
JMJD6	-1.6554357091	0.0130886671	0.9999763851
METTL23	1.1090294691	0.6625603247	0.9999763851
SRSF2	1.1650504353	0.2155742584	0.9999763851
MFSD11	-1.134041525	0.7130988011	0.9999763851
MGAT5B	0	0	0
SEC14L1	1.2179077413	0.5425987287	0.9999763851
SEPT9	1.010028779	0.9492735519	0.9999763851
TNRC6C	1.4004060892	0.2361249462	0.9999763851
TMC6	1.1883889727	0.4044046286	0.9999763851
TMC8	1.2540889313	0.2759664525	0.9999763851
C17orf99	1.3406186202	0.7024074684	0.9999763851
SYNGR2	1.0007922426	0.9962191031	0.9999763851
TK1	1.5988984277	0.0481773985	0.9999763851
AFMID	1.0540431582	0.8983311216	0.9999763851
BIRC5	-1.2664221316	0.1486772135	0.9999763851
SOCS3	5.4790129495	0.3770544328	0.9999763851
PGS1	1.0592807813	0.8490445845	0.9999763851
DNAH17	3.3937024411	0.2365744837	0.9999763851
CYTH1	-1.4837669675	0.1794407997	0.9999763851
USP36	1.5747810022	0.0491538128	0.9999763851
TIMP2	-1.3715658618	0.0636813925	0.9999763851
LGALS3BP	0	0	0
CANT1	1.5073404876	0.1093228765	0.9999763851
ENGASE	1.3417868536	0.3539514453	0.9999763851
RBFOX3	-2.5587422769	0.2939152544	0.9999763851
CBX2	-1.2167281174	0.2464040924	0.9999763851
CBX8	1.027554661	0.9756499449	0.9999763851
CBX4	1.4818259365	0.0194434151	0.9999763851
TBC1D16	-1.4318010494	0.1293841081	0.9999763851
CCDC40	-1.1494135484	0.8479372539	0.9999763851
GAA	-1.2228681114	0.2674177744	0.9999763851
EIF4A3	-1.0747790408	0.5812168489	0.9999763851
SGSH	-4.2849908816	0.4028453076	0.9999763851
SLC26A11	-2.3220580825	0.4361528034	0.9999763851
RNF213	-1.2988776157	0.1424568851	0.9999763851
ENDOV	1.031881869	0.9342107461	0.9999763851
RPTOR	1.7213189457	0.0237191973	0.9999763851
LOC105371921	3.7137105359	0.127513383	0.9999763851

CHMP6	1.0502262877	0.9153431807	0.9999763851
BAIAP2	-1.4160477835	0.6149260578	0.9999763851
AATK	5.4770877341	0.3746135179	0.9999763851
CEP131	1.0983992073	0.8445066911	0.9999763851
ENTHD2	1.0524293328	0.8249979255	0.9999763851
C17orf89	-1.0511468366	0.6997333266	0.9999763851
SLC38A10	1.1989902512	0.1724453134	0.9999763851
TMEM105	-3.3269505881	0.2614707944	0.9999763851
LOC100130370	1.7659093218	0.1645744979	0.9999763851
BAHCC1	-1.3105086293	0.1641695256	0.9999763851
ACTG1	-1.1965139403	0.1387789246	0.9999763851
FSCN2	6.368054416	0.3491635634	0.9999763851
FAAP100	1.311894018	0.3445888282	0.9999763851
NPLOC4	-1.2326254162	0.2720037118	0.9999763851
TSPAN10	-1.9740380889	0.280981329	0.9999763851
PDE6G	-1.3158816768	0.8203229796	0.9999763851
OXLD1	1.7530922662	0.0522775349	0.9999763851
CCDC137	1.3913813201	0.1438165004	0.9999763851
ARL16	1.0694712425	0.8224209407	0.9999763851
HGS	1.1379232316	0.5454124149	0.9999763851
MRPL12	1.0377233846	0.8583253283	0.9999763851
SLC25A10	-1.0445055821	0.8559792649	0.9999763851
GCGR	0	0	0
MCRIP1	-1.1638773997	0.5146295439	0.9999763851
PPP1R27	-1.1862097654	0.6911970683	0.9999763851
P4HB	1.1816681558	0.1722579276	0.9999763851
ARHGDIA	1.109153248	0.5297948035	0.9999763851
ALYREF	1.2291377558	0.2354199433	0.9999763851
ANAPC11	1.23351879	0.1267009386	0.9999763851
PCYT2	-1.0057481359	0.9782906303	0.9999763851
SIRT7	1.2490378222	0.2785227466	0.9999763851
MAFG	-1.0785559128	0.6068514886	0.9999763851
MYADML2	0	0	0
NOTUM	0	0	0
ASPSCR1	1.5237627223	0.27691641	0.9999763851
STRA13	1.0903504173	0.6824079806	0.9999763851
LRRC45	-1.3409801979	0.4893586368	0.9999763851
RAC3	-1.0195222327	0.9715494501	0.9999763851
DCXR	1.1505292304	0.4123407491	0.9999763851
RFNG	1.0778949265	0.8632727738	0.9999763851
GPS1	1.1056930974	0.6638533059	0.9999763851
DUS1L	1.5196477277	0.011241128	0.9999763851
FASN	-1.0190819804	0.8908694471	0.9999763851
CCDC57	-1.2537635832	0.3659778794	0.9999763851
SLC16A3	1.007412379	0.9636829309	0.9999763851
CSNK1D	1.2293362349	0.1987391202	0.9999763851
TEX19	1.1427172643	0.7752356948	0.9999763851
OGFOD3	1.4492553209	0.1934123457	0.9999763851
HEXDC	1.4950776813	0.2107898692	0.9999763851

C17orf62	1.2184108832	0.4295393158	0.9999763851
NARF	1.1729331475	0.5131037997	0.9999763851
FOXK2	-1.0753705697	0.6522633226	0.9999763851
WDR45B	-1.1889874864	0.2852939405	0.9999763851
RAB40B	1.190830385	0.7249087679	0.9999763851
FN3KRP	-1.0708183273	0.7729172705	0.9999763851
FN3K	0	0	0
TBCD	-1.9158695132	0.0801179606	0.9999763851
LOC105371944	3.2356557963	0.5547505991	0.9999763851
B3GNTL1	1.1602988526	0.4118026422	0.9999763851
METRNL	-1.6699253022	0.0168594392	0.9999763851
USP14	1.268719576	0.1617850482	0.9999763851
THOC1	-1.1850965868	0.486945675	0.9999763851
COLEC12	-4.2849908816	0.4028453076	0.9999763851
CLUL1	4.9761319165	0.1044956469	0.9999763851
TYMSOS	-1.0030965355	0.9882143083	0.9999763851
TYMS	1.3445571224	0.133731681	0.9999763851
ENOSF1	1.2594240891	0.2226723187	0.9999763851
YES1	-1.1266057749	0.7238896945	0.9999763851
METTL4	1.4276679728	0.4182629513	0.9999763851
NDC80	1.5368360324	0.0666069778	0.9999763851
SMCHD1	1.222222408	0.2418909853	0.9999763851
EMILIN2	-1.7376472043	0.0246840727	0.9999763851
LPIN2	-1.2305055086	0.5110075629	0.9999763851
MYL12A	-1.039244956	0.8229916289	0.9999763851
MYL12B	-1.2767519225	0.1330881155	0.9999763851
TGIF1	-1.1113430264	0.8259028597	0.9999763851
DLGAP1	2.3833649494	0.2136734025	0.9999763851
ZBTB14	-1.5731112195	0.1155256579	0.9999763851
EPB41L3	1.3858577527	0.1166141524	0.9999763851
ARHGAP28	3.2356557963	0.5547505991	0.9999763851
LAMA1	-4.2864494036	0.4027150576	0.9999763851
RAB12	-1.0857545116	0.6997866977	0.9999763851
MTCL1	-1.4265959713	0.5889879194	0.9999763851
NDUFV2	1.0985417743	0.5927695475	0.9999763851
ANKRD12	-1.7024590316	0.0150631528	0.9999763851
TWSG1	-1.1989352035	0.4643356999	0.9999763851
RALBP1	1.0625244586	0.6946807271	0.9999763851
PPP4R1	1.1750457763	0.3638509778	0.9999763851
RAB31	-1.3123459729	0.0559937365	0.9999763851
TXNDC2	0	0	0
VAPA	1.1434807562	0.2976169952	0.9999763851
APCDD1	7.722359209	0.2874240873	0.9999763851
NAPG	1.0844005072	0.7240639501	0.9999763851
GNAL	-1.4073673926	0.6744285455	0.9999763851
CHMP1B	-1.472549039	0.1532825515	0.9999763851
MPPE1	1.0538144491	0.8651115928	0.9999763851
IMPA2	1.2467875593	0.1314080006	0.9999763851
CIDEA	-1.9661045084	0.4534996017	0.9999763851

TUBB6	1.0528456853	0.7054818371	0.9999763851
AFG3L2	-2.2613004035	0.0180473284	0.9999763851
PRELID3A	2.011636301	0.1831048152	0.9999763851
SPIRE1	2.2799218304	0.0424500369	0.9999763851
PSMG2	1.1289216452	0.5260274705	0.9999763851
CEP76	-1.125788792	0.8101302279	0.9999763851
PTPN2	-1.1066674826	0.6447097725	0.9999763851
SEH1L	1.1553499034	0.4350136921	0.9999763851
CEP192	-1.6465320858	0.1210643586	0.9999763851
LDLRAD4	1.1785905591	0.577841858	0.9999763851
FAM210A	1.0415522553	0.8450658861	0.9999763851
RNMT	1.0393627572	0.8248916887	0.9999763851
ZNF519	1.4145351204	0.4102711148	0.9999763851
ANKRD30B	0	0	0
ROCK1	1.1302097523	0.4852404621	0.9999763851
ESCO1	1.013979731	0.9590659246	0.9999763851
SNRPD1	-1.0301930549	0.8146288958	0.9999763851
ABHD3	1.0076240997	0.9807605031	0.9999763851
MIB1	-1.4842623491	0.0641970462	0.9999763851
GATA6	-2.3227998731	0.4423442872	0.9999763851
CTAGE1	-4.2864494036	0.4027150576	0.9999763851
RBBP8	1.0316188723	0.8978489437	0.9999763851
LOC105372022	0	0	0
CABLES1	-1.4049149076	0.4270897107	0.9999763851
TMEM241	1.1313222753	0.6266838642	0.9999763851
RIOK3	-1.1719432075	0.5624205679	0.9999763851
C18orf8	1.0530606975	0.8285526709	0.9999763851
NPC1	-1.5368605507	0.1620602488	0.9999763851
ANKRD29	-4.2849908816	0.4028453076	0.9999763851
LAMA3	0	0	0
TTC39C	-1.0644101204	0.7436789616	0.9999763851
OSBPL1A	1.3583153059	0.2177960888	0.9999763851
IMPACT	1.8331084374	0.0784332203	0.9999763851
SS18	1.0320900741	0.8759787169	0.9999763851
PSMA8	4.337646665	0.005906	0.9999763851
TAF4B	-1.2941113162	0.4923770922	0.9999763851
KCTD1	1.1314940385	0.748535491	0.9999763851
DSG3	0	0	0
DSG2	0	0	0
SLC25A52	-1.3178224963	0.7617796282	0.9999763851
TRAPPC8	1.0051706883	0.9871178963	0.9999763851
RNF125	1.294438746	0.4097490779	0.9999763851
RNF138	-1.1097586732	0.6715422085	0.9999763851
GAREM1	4.6124114429	0.0497052645	0.9999763851
DTNA	0	0	0
MAPRE2	1.4182977911	0.2135128463	0.9999763851
ZNF397	-1.0390719457	0.9459961495	0.9999763851
ZSCAN30	-1.7384828959	0.2722560013	0.9999763851
ZNF24	1.0615050333	0.734345652	0.9999763851

ZNF396	0	0	0
INO80C	1.1763088418	0.7037208211	0.9999763851
GALNT1	1.2587168859	0.1740068173	0.9999763851
C18orf21	-1.0772491272	0.8038099843	0.9999763851
RPRD1A	1.3277535968	0.1740396859	0.9999763851
SLC39A6	-1.0099910827	0.9599779856	0.9999763851
ELP2	-1.2623910018	0.2748664257	0.9999763851
FHOD3	1.2869488883	0.8212647797	0.9999763851
TPGS2	1.1570498	0.3686347809	0.9999763851
KIAA1328	-1.0106205677	0.979228462	0.9999763851
PIK3C3	1.3099658605	0.2022104335	0.9999763851
SLC14A2	-1.4211113077	0.5992730278	0.9999763851
SLC14A1	1.1393200909	0.836754531	0.9999763851
EPG5	-1.1871152842	0.3221336632	0.9999763851
SIGLEC15	-1.2347505946	0.7212635075	0.9999763851
PSTPIP2	1.3531784651	0.0886505475	0.9999763851
ATP5A1	1.1623316224	0.2073866185	0.9999763851
HAUS1	1.347668138	0.1522736807	0.9999763851
C18orf25	-1.1680439785	0.4908845841	0.9999763851
RNF165	-1.5911835926	0.2688940412	0.9999763851
LOXHD1	0	0	0
PIAS2	-1.046240234	0.8520608296	0.9999763851
KATNAL2	-1.677905058	0.1789147043	0.9999763851
HDHD2	-1.0564198249	0.8278121123	0.9999763851
IER3IP1	1.0116081812	0.9435816828	0.9999763851
SMAD2	1.1129968444	0.4112374721	0.9999763851
ZBTB7C	1.8137824633	0.5650708093	0.9999763851
CTIF	-1.6263145503	0.2845371714	0.9999763851
SMAD7	1.5931922204	0.475646022	0.9999763851
DYM	1.3021913696	0.1918979724	0.9999763851
C18orf32	-1.5045639136	0.6135235448	0.9999763851
RPL17-C18orf32	-1.046758537	0.9431787071	0.9999763851
RPL17	-1.0680504711	0.6098631586	0.9999763851
LIPG	0	0	0
ACAA2	1.3473501545	0.1604902033	0.9999763851
MYO5B	-1.3157515262	0.8225431686	0.9999763851
CFAP53	-1.1960912581	0.7932911701	0.9999763851
MBD1	-1.1366589468	0.5274264358	0.9999763851
CXXC1	1.290381635	0.2956972014	0.9999763851
SKA1	1.0758157455	0.6904055391	0.9999763851
MRO	1.2869488883	0.8213966802	0.9999763851
ME2	1.3162081797	0.1377623823	0.9999763851
ELAC1	-1.2501416505	0.6234664112	0.9999763851
SMAD4	-1.1840754211	0.3995692433	0.9999763851
MEX3C	1.0956963208	0.7198710167	0.9999763851
DCC	-1.3157515262	0.8226406568	0.9999763851
MBD2	1.1496545935	0.4344302282	0.9999763851
POLI	-1.4275443527	0.5508169969	0.9999763851
STARD6	0	0	0

C18orf54	-1.0613861353	0.8766861319	0.9999763851
RAB27B	1.1809081663	0.431212455	0.9999763851
CCDC68	0	0	0
TCF4	1.460070465	0.1651055916	0.9999763851
TXNL1	1.4664524438	0.0192607815	0.9999763851
WDR7	1.1649170552	0.5869194555	0.9999763851
BOD1L2	3.2353357581	0.554787377	0.9999763851
ONECUT2	1.5665550185	0.1461142335	0.9999763851
FECH	1.0079974426	0.9689864487	0.9999763851
NARS	1.0834630824	0.5637073624	0.9999763851
LOC100505549	1.0316918329	0.9422244948	0.9999763851
ATP8B1	1.0959397657	0.8987293012	0.9999763851
NEDD4L	-1.4490644074	0.177761689	0.9999763851
LOC101927322	3.2353357581	0.554787377	0.9999763851
MALT1	-1.0002638517	0.9991526872	0.9999763851
ZNF532	0	0	0
SEC11C	-1.4603990861	0.0575076048	0.9999763851
LMAN1	-1.4232019761	0.0456366823	0.9999763851
PMAIP1	-1.1675544153	0.632017776	0.9999763851
CDH20	3.2356557963	0.5547505991	0.9999763851
PIGN	1.3981943276	0.2234987455	0.9999763851
KIAA1468	-1.6410991858	0.1216969411	0.9999763851
TNFRSF11A	-1.3157515262	0.8227364894	0.9999763851
ZCCHC2	1.0943213612	0.6805532992	0.9999763851
PHLPP1	-1.2210442353	0.5416895447	0.9999763851
BCL2	1.3574611945	0.1219747567	0.9999763851
KDSR	1.4319835927	0.1120552441	0.9999763851
VPS4B	1.0191525346	0.9163300125	0.9999763851
SERPINB13	0	0	0
SERPINB4	5.4781708352	0.3745527127	0.9999763851
SERPINB2	-1.2487160765	0.2249693702	0.9999763851
SERPINB10	-1.1487065491	0.8497509362	0.9999763851
HMSD	-4.2849908816	0.4028453076	0.9999763851
SERPINB8	1.1662324787	0.660964801	0.9999763851
TMX3	1.038674887	0.8440882348	0.9999763851
CCDC102B	-4.7674026393	0.0254957192	0.9999763851
DOK6	0	0	0
CD226	0	0	0
RTTN	-1.431094597	0.3617603982	0.9999763851
SOCS6	1.2649613881	0.4833458416	0.9999763851
CBLN2	0	0	0
FBXO15	-1.3151016298	0.8212997976	0.9999763851
TIMM21	1.2615674681	0.2687467046	0.9999763851
CYB5A	-1.1806901819	0.7419498692	0.9999763851
CNDP2	1.43710671	0.0785497062	0.9999763851
CNDP1	0	0	0
ZNF407	1.3806944824	0.3061357917	0.9999763851
ZADH2	1.605074042	0.0205974713	0.9999763851
TSHZ1	-1.0058215633	0.9805488346	0.9999763851

SMIM21	0	0	0
ZNF516	1.1029598534	0.6378174891	0.9999763851
C18orf65	0	0	0
ZNF236	2.2652956169	0.0436937484	0.9999763851
MBP	-1.1565182767	0.2359212865	0.9999763851
LOC107987260	-4.3498975359	0.0551187273	0.9999763851
ATP9B	1.4461746901	0.3026275879	0.9999763851
LOC100996274	-4.2849908816	0.4028453076	0.9999763851
NFATC1	1.2991507835	0.2754794045	0.9999763851
CTDP1	1.0170868483	0.9361862445	0.9999763851
LOC105376875	0	0	0
KCNG2	1.7333395294	0.2445560354	0.9999763851
PQLC1	-1.0916230486	0.5315989856	0.9999763851
HSBP1L1	-1.4740283994	0.4768688405	0.9999763851
TXNL4A	1.3412242596	0.0248043144	0.9999763851
RBFA	-1.1952419538	0.3053420021	0.9999763851
ADNP2	-1.2341261477	0.3627906561	0.9999763851
PARD6G	1.1796097153	0.7225421517	0.9999763851
PLPP2	0	0	0
LOC101929060	0	0	0
MIER2	1.290970743	0.4946227036	0.9999763851
C2CD4C	-1.3151016298	0.8212381876	0.9999763851
ODF3L2	-1.3154339304	0.8205576571	0.9999763851
MADCAM1	1.8139617088	0.5800559797	0.9999763851
TPGS1	-1.3943229106	0.7064421931	0.9999763851
CDC34	1.2772717163	0.1456358752	0.9999763851
BSG	-1.1266048093	0.4916397366	0.9999763851
HCN2	5.4790129495	0.3772418585	0.9999763851
POLRMT	1.413178442	0.3283936249	0.9999763851
RNF126	1.0364439616	0.7970954411	0.9999763851
FSTL3	-1.231512585	0.5748721099	0.9999763851
PRSS57	-1.1495398408	0.8598004411	0.9999763851
PALM	-1.0192343692	0.9691073227	0.9999763851
PTBP1	1.131922505	0.3209385984	0.9999763851
PLPPR3	-1.0358048925	0.9599204069	0.9999763851
AZU1	-2.0972707676	0.0146336224	0.9999763851
PRTN3	-2.7315023848	0.002801	0.9999763851
ELANE	-2.1559272076	0.008335	0.9999763851
CFD	-1.8839942601	0.0006626	0.6368063985
MED16	1.2786596453	0.4722063616	0.9999763851
R3HDM4	1.2088484948	0.5032060441	0.9999763851
ARID3A	-1.0714448023	0.645970121	0.9999763851
WDR18	-1.1125770258	0.5322724397	0.9999763851
GRIN3B	1.7666556068	0.007222	0.9999763851
TMEM259	-1.1613439262	0.3946440167	0.9999763851
CNN2	1.2475563951	0.2287581546	0.9999763851
ABCA7	1.1367329528	0.6845222225	0.9999763851
HMHA1	-1.1482188025	0.3292999748	0.9999763851
POLR2E	1.1010004561	0.4868334108	0.9999763851

GPX4	-1.0317008389	0.8395631037	0.9999763851
SBNO2	1.0209730818	0.9207863291	0.9999763851
LOC102725180	-3.3272796812	0.2422722016	0.9999763851
STK11	1.4167691791	0.1564333106	0.9999763851
CBARP	1.1833354309	0.6986106397	0.9999763851
ATP5D	1.0671525712	0.7385254444	0.9999763851
MIDN	1.3387894598	0.0886668652	0.9999763851
CIRBP	1.035292992	0.8057141232	0.9999763851
C19orf24	-1.05909459	0.7524654517	0.9999763851
MUM1	1.2512259867	0.2673091583	0.9999763851
NDUFS7	-1.0454754495	0.8339337701	0.9999763851
GAMT	-1.0936291488	0.6504607401	0.9999763851
DAZAP1	-1.1454902037	0.2527936722	0.9999763851
RPS15	1.1976131177	0.1265152381	0.9999763851
APC2	1.1829955191	0.4173601781	0.9999763851
C19orf25	1.2238657423	0.5731691936	0.9999763851
PCSK4	-1.1080029864	0.8633017572	0.9999763851
REEP6	1.0786099006	0.8879346195	0.9999763851
ADAMTSL5	1.6233493708	0.594412743	0.9999763851
PLK5	3.2353357581	0.554787377	0.9999763851
MEX3D	-1.1860170558	0.3976578489	0.9999763851
MBD3	1.0816737879	0.697655724	0.9999763851
UQCR11	1.2165253877	0.1154014256	0.9999763851
TCF3	-1.0484094273	0.7397398038	0.9999763851
ATP8B3	1.1517721744	0.6032066345	0.9999763851
REXO1	-1.1777373612	0.6950323536	0.9999763851
KLF16	1.0037582097	0.9833317709	0.9999763851
ABHD17A	-1.07702174	0.7362004703	0.9999763851
ADAT3	5.9192858611	0.1688273823	0.9999763851
SCAMP4	2.814770943	0.0765236739	0.9999763851
CSNK1G2	-1.0266551666	0.8691250895	0.9999763851
BTBD2	1.1577339402	0.6058809204	0.9999763851
MKNK2	1.3208484269	0.2400526405	0.9999763851
MOB3A	1.182977171	0.4092961741	0.9999763851
IZUMO4	1.1352686914	0.7008053866	0.9999763851
AP3D1	1.1605087322	0.385082447	0.9999763851
DOT1L	1.0322896598	0.9103597557	0.9999763851
PLEKHJ1	1.1479919186	0.339120879	0.9999763851
SF3A2	-1.4282645744	0.5366645992	0.9999763851
AMH	1.4570861054	0.4848855715	0.9999763851
JSRP1	0	0	0
OAZ1	1.0384096329	0.7585799008	0.9999763851
C19orf35	-1.9659101454	0.4336378235	0.9999763851
LINGO3	1.0637644931	0.8356010693	0.9999763851
LSM7	1.0879769411	0.5705777505	0.9999763851
SPPL2B	-1.2361579023	0.2926920122	0.9999763851
TMPRSS9	-4.3357248284	0.1409480754	0.9999763851
TIMM13	-1.1606820567	0.294971482	0.9999763851
LMNB2	-1.1248319312	0.4387481828	0.9999763851

GADD45B	-1.1858930738	0.3094010067	0.9999763851
LOC101929097	-1.4010538105	0.179624853	0.9999763851
GNG7	-1.4533319337	0.1285468438	0.9999763851
DIRAS1	1.1729244398	0.7554942151	0.9999763851
SLC39A3	2.1659783213	0.0009005	0.734233805
SGTA	1.0814295429	0.6759542052	0.9999763851
THOP1	1.1248383721	0.4361454396	0.9999763851
ZNF554	1.7226814704	0.2677187242	0.9999763851
ZNF555	-1.2382563075	0.6308946879	0.9999763851
ZNF556	-4.2849908816	0.4028453076	0.9999763851
ZNF57	3.3957503398	0.239352767	0.9999763851
ZNF77	2.8184855207	0.1996329781	0.9999763851
TLE6	-1.0049708175	0.9922834184	0.9999763851
TLE2	1.478582848	0.5996274859	0.9999763851
AES	1.0509218159	0.7448172235	0.9999763851
GNA11	-1.3297681187	0.6010806461	0.9999763851
GNA15	-1.0462218838	0.8122999357	0.9999763851
S1PR4	-1.2386892921	0.2310374059	0.9999763851
NCLN	-1.0977440504	0.556341226	0.9999763851
CELF5	5.4781708352	0.3745527127	0.9999763851
NFIC	1.0934320093	0.5678219705	0.9999763851
SMIM24	-1.6887668142	0.1688496532	0.9999763851
DOHH	-1.5228866999	0.1204790783	0.9999763851
FZR1	-1.2032459726	0.5815483738	0.9999763851
MFSD12	-1.2002212613	0.2216139358	0.9999763851
HMG20B	-1.1246151404	0.470963387	0.9999763851
GIPC3	-1.3160416743	0.8270099247	0.9999763851
TBXA2R	1.1232485485	0.4547549211	0.9999763851
CACTIN	1.0685418362	0.7862644062	0.9999763851
PIP5K1C	1.1317338022	0.7210439827	0.9999763851
TJP3	1.287203384	0.8320172331	0.9999763851
APBA3	1.3288018935	0.417699301	0.9999763851
MRPL54	-1.1073647831	0.5648144307	0.9999763851
MATK	-2.2387936332	0.3206210108	0.9999763851
ZFR2	-1.7352105782	0.4494410497	0.9999763851
ATCAY	-2.3220580825	0.4353552009	0.9999763851
DAPK3	-1.2242067767	0.3826988383	0.9999763851
EEF2	1.0680643246	0.5814156193	0.9999763851
PIAS4	1.1874204229	0.5241937705	0.9999763851
ZBTB7A	-1.0054081079	0.9724444475	0.9999763851
MAP2K2	1.028587371	0.8371433472	0.9999763851
CREB3L3	0	0	0
SIRT6	1.0312790402	0.8901782785	0.9999763851
ANKRD24	-4.2849908816	0.4028453076	0.9999763851
EBI3	0	0	0
CCDC94	-1.0328207578	0.9052833085	0.9999763851
TMIGD2	-4.2849908816	0.4028453076	0.9999763851
FSD1	-4.2849908816	0.4028453076	0.9999763851
STAP2	1.8134025452	0.5839701294	0.9999763851

MPND	-1.1745625425	0.5847105787	0.9999763851
SH3GL1	1.3625725331	0.0659705702	0.9999763851
CHAF1A	1.1875376103	0.2359816683	0.9999763851
UBXN6	1.0719831242	0.6449033605	0.9999763851
HDGFRP2	-1.1384030018	0.4773255691	0.9999763851
PLIN4	-1.9661492502	0.4738452784	0.9999763851
PLIN5	-2.5608228084	0.2884816449	0.9999763851
LRG1	-1.1144840443	0.4936927508	0.9999763851
SEMA6B	-2.011562566	0.2172147759	0.9999763851
TNFAIP8L1	1.170567477	0.4898465667	0.9999763851
MYDGF	1.0103185382	0.9393700802	0.9999763851
DPP9	1.2911107955	0.2503707114	0.9999763851
FEM1A	1.2106712876	0.5211185411	0.9999763851
TICAM1	-1.3380251034	0.3209490074	0.9999763851
PLIN3	-1.1070113067	0.4377086886	0.9999763851
ARRDC5	0	0	0
UHRF1	1.2429191645	0.2356182332	0.9999763851
KDM4B	1.0180068408	0.9156153488	0.9999763851
PTPRS	3.2353357581	0.554787377	0.9999763851
SAFB2	-1.5592662614	0.0311889825	0.9999763851
SAFB	-1.1755084641	0.3731025313	0.9999763851
LOC107985320	3.2361532718	0.5546934395	0.9999763851
C19orf70	1.1281916477	0.4789422159	0.9999763851
HSD11B1L	-1.2955873989	0.6211568569	0.9999763851
RPL36	1.1167669333	0.3595710433	0.9999763851
LONP1	1.1654789411	0.336149732	0.9999763851
CATSPERD	0	0	0
PRR22	5.4784711963	0.3745358536	0.9999763851
DUS3L	1.1602605742	0.5932537302	0.9999763851
NRTN	-1.816422855	0.4360212244	0.9999763851
FUT6	0	0	0
FUT5	0	0	0
NDUFA11	1.2405297017	0.1624475673	0.9999763851
VMAC	1.0156708	0.9843174313	0.9999763851
CAPS	1.9083992729	0.0863873725	0.9999763851
RANBP3	1.026674448	0.9010747607	0.9999763851
RFX2	-1.2460230533	0.3258045947	0.9999763851
ACSBG2	-1.1716993339	0.6820520076	0.9999763851
MLLT1	1.4801516776	0.027575056	0.9999763851
ACER1	-7.5647903763	0.2176246281	0.9999763851
CLPP	1.0564096296	0.6836389937	0.9999763851
ALKBH7	-1.3360448093	0.214090064	0.9999763851
PSPN	0	0	0
GTF2F1	1.2075004634	0.3388979875	0.9999763851
KHSRP	-1.060771175	0.6908056133	0.9999763851
SLC25A41	-1.6975288129	0.2822628035	0.9999763851
SLC25A23	1.2303930058	0.290532441	0.9999763851
CRB3	0	0	0
DENND1C	1.3736469229	0.2725320222	0.9999763851

TNFSF9	-5.4150770802	0.002095	0.9999763851
CD70	-1.3190802586	0.2023685913	0.9999763851
TNFSF14	-1.3783209169	0.2941263468	0.9999763851
C3	-3.3614101997	0.002049	0.9999763851
GPR108	1.2443704681	0.3561351474	0.9999763851
TRIP10	-1.9893343261	0.144428183	0.9999763851
SH2D3A	-1.5725867351	0.3749408036	0.9999763851
VAV1	-1.0433573602	0.7871238427	0.9999763851
ADGRE1	1.8309908604	0.2318677919	0.9999763851
MBD3L2	0	0	0
ZNF557	1.0956619805	0.6776500152	0.9999763851
INSR	1.0207636983	0.9051154068	0.9999763851
LOC107985283	-1.3157515262	0.822831887	0.9999763851
ARHGEF18	1.1051313581	0.5479029135	0.9999763851
PEX11G	-1.4150863318	0.6298602669	0.9999763851
C19orf45	0	0	0
ZNF358	0	0	0
PNPLA6	-1.4319521272	0.50340234	0.9999763851
XAB2	-1.3158816768	0.8203229797	0.9999763851
PET100	1.2761522657	0.1958612507	0.9999763851
PCP2	-2.5596117216	0.2621352285	0.9999763851
STXBP2	-1.1910501548	0.621473838	0.9999763851
RETN	1.2066055503	0.3030269637	0.9999763851
MCEMP1	-1.2671942003	0.0842823326	0.9999763851
TRAPPC5	1.1048722787	0.5167319418	0.9999763851
FCER2	-3.3693112949	0.0664786679	0.9999763851
CLEC4G	0	0	0
CD209	-10.7444032429	0.016846655	0.9999763851
CLEC4M	0	0	0
EVI5L	1.0065551119	0.9854709919	0.9999763851
LRRC8E	0	0	0
MAP2K7	1.0997097912	0.6742067383	0.9999763851
TGFBR3L	3.2353357581	0.554787377	0.9999763851
SNAPC2	1.1753816022	0.4974522329	0.9999763851
CTXN1	3.6309026086	0.0492490545	0.9999763851
TIMM44	-1.0300544651	0.899763472	0.9999763851
ELAVL1	1.2558480381	0.0857132023	0.9999763851
FBN3	3.2353357581	0.554787377	0.9999763851
CERS4	-1.0856424323	0.8747587319	0.9999763851
CD320	1.2384680585	0.4061169286	0.9999763851
NDUFA7	1.2000714918	0.3031412615	0.9999763851
RPS28	-1.0949611177	0.5270022575	0.9999763851
KANK3	3.7612496746	0.008217	0.9999763851
ANGPTL4	1.2868359435	0.8210199768	0.9999763851
RAB11B	1.0751482894	0.662666647	0.9999763851
MARCH2	1.2746699714	0.6347625844	0.9999763851
HNRNPM	-1.0876045946	0.4975784655	0.9999763851
PRAM1	1.5530451128	0.0288885423	0.9999763851
ZNF414	1.6418746427	0.3227716463	0.9999763851

MYO1F	1.2321566976	0.2492267318	0.9999763851
ZNF558	-1.3077795393	0.6073560301	0.9999763851
ZNF317	1.8437472423	0.0200195762	0.9999763851
OR7D2	3.2353357581	0.554787377	0.9999763851
ZNF699	-1.3154339304	0.8205576571	0.9999763851
ZNF559	-1.9412937271	0.1535158704	0.9999763851
ZNF559-ZNF177	0	0	0
ZNF177	-1.395165126	0.7000541941	0.9999763851
ZNF266	-1.5855899351	0.1208081883	0.9999763851
LOC105372269	3.2361532718	0.5546934395	0.9999763851
ZNF560	0	0	0
ZNF121	2.3097697104	0.1647584067	0.9999763851
ZNF561	-1.3625760305	0.4332846605	0.9999763851
ZNF562	1.0278478563	0.9759411348	0.9999763851
ZNF846	1.3489007316	0.6910545606	0.9999763851
FBXL12	-2.0146318623	0.0314833149	0.9999763851
UBL5	1.1453890918	0.3043822708	0.9999763851
PIN1	-1.0113289418	0.9481848371	0.9999763851
COL5A3	5.4770877341	0.3746135179	0.9999763851
RDH8	0	0	0
C19orf66	-3.9563917548	0.006909	0.9999763851
PPAN	-1.0434661196	0.9034271688	0.9999763851
P2RY11	1.3088342267	0.293462589	0.9999763851
EIF3G	1.3807552255	0.0630799767	0.9999763851
DNMT1	-1.0341768888	0.799917453	0.9999763851
S1PR2	1.1701717412	0.6593832046	0.9999763851
MRPL4	1.3778703189	0.0608340738	0.9999763851
ICAM1	1.1042375223	0.7878914631	0.9999763851
ICAM4	1.1596047671	0.7293259112	0.9999763851
ICAM5	-2.3217011239	0.4355938696	0.9999763851
ZGLP1	1.6254723588	0.5800651146	0.9999763851
FDX1L	1.277141552	0.463559857	0.9999763851
RAVER1	1.209729158	0.5925394386	0.9999763851
ICAM3	1.2560548125	0.1061860828	0.9999763851
TYK2	-1.172192962	0.5083594564	0.9999763851
CDC37	-1.0583853697	0.6754962068	0.9999763851
PDE4A	-10.8385246611	0.1451469791	0.9999763851
KEAP1	1.0024246813	0.987380383	0.9999763851
ATG4D	-3.6710485044	0.006046	0.9999763851
KRI1	1.9514877687	0.000978	0.7855409274
CDKN2D	-1.0176416257	0.9228580931	0.9999763851
AP1M2	-17.4476623799	0.0923403128	0.9999763851
SLC44A2	-1.6013360249	0.0529265119	0.9999763851
ILF3	-1.1506875844	0.2699617103	0.9999763851
QTRT1	1.2255213	0.7789922607	0.9999763851
DNM2	1.0783684779	0.6418444086	0.9999763851
TMED1	1.1600511197	0.4801875769	0.9999763851
C19orf38	-1.0031126323	0.9878742355	0.9999763851
CARM1	-4.3338228569	0.1410030633	0.9999763851

YIPF2	-1.0838099088	0.5363923334	0.9999763851
C19orf52	1.1815738793	0.43406435	0.9999763851
SMARCA4	1.2931038207	0.0756208693	0.9999763851
LDLR	-1.1245609235	0.4149174191	0.9999763851
SPC24	-1.0396375606	0.8393911704	0.9999763851
KANK2	1.5438777797	0.1118546573	0.9999763851
DOCK6	1.2432087164	0.5809991689	0.9999763851
TSPAN16	1.8239500745	0.2601331824	0.9999763851
RAB3D	1.0344029654	0.8288713433	0.9999763851
TMEM205	1.266972032	0.2214455334	0.9999763851
CCDC159	1.1331614061	0.8698784785	0.9999763851
PLPPR2	-1.0255207638	0.9242784129	0.9999763851
SWSAP1	1.8944409591	0.0741989658	0.9999763851
EPOR	-1.1030553504	0.8681350083	0.9999763851
RGL3	0	0	0
CCDC151	3.2353357581	0.554787377	0.9999763851
PRKCSH	1.7086746023	0.0809371027	0.9999763851
ELAVL3	3.2353357581	0.554787377	0.9999763851
ZNF653	2.3408621302	0.4285500168	0.9999763851
ECSIT	-1.2447180094	0.3320723652	0.9999763851
ELOF1	-1.1749043917	0.4502472849	0.9999763851
ZNF627	1.3673111203	0.4519520586	0.9999763851
ZNF823	0	0	0
ZNF491	0	0	0
ZNF440	1.7348614386	0.2550741511	0.9999763851
ZNF439	0	0	0
ZNF69	-1.5616830306	0.3459208614	0.9999763851
ZNF700	-1.2394518952	0.4439605209	0.9999763851
ZNF763	-4.2864494036	0.4027150576	0.9999763851
ZNF433	0	0	0
ZNF878	0	0	0
ZNF136	1.4848975885	0.5142317695	0.9999763851
ZNF442	0	0	0
ZNF799	1.2798565264	0.6274032214	0.9999763851
ZNF443	-1.1237477727	0.8346766123	0.9999763851
ZNF709	0	0	0
ZNF564	1.1953634879	0.7390137721	0.9999763851
ZNF490	-2.7526009596	0.1364593139	0.9999763851
ZNF791	1.6406834893	0.0765534114	0.9999763851
MAN2B1	-1.2473871228	0.1554489174	0.9999763851
WDR83	2.2534767074	0.3736000139	0.9999763851
WDR83OS	1.1213011145	0.4683338982	0.9999763851
DHPS	-1.1374078352	0.5531377708	0.9999763851
FBXW9	1.4641838996	0.2172858013	0.9999763851
TNPO2	-1.0068922956	0.9723402126	0.9999763851
C19orf43	1.188518324	0.3187821323	0.9999763851
ASNA1	1.044930903	0.8414235456	0.9999763851
HOOK2	-1.236041506	0.5072788673	0.9999763851
JUNB	-1.2871882981	0.1950891011	0.9999763851

PRDX2	1.5911808653	0.0175541418	0.9999763851
RNASEH2A	1.0491006103	0.7682562439	0.9999763851
RTBDN	7.7488609396	0.3058742041	0.9999763851
MAST1	-1.203182252	0.5063658999	0.9999763851
DNASE2	1.1011143421	0.5434091808	0.9999763851
KLF1	-1.4282479823	0.5509849511	0.9999763851
GCDH	-1.4401864441	0.0272734717	0.9999763851
SYCE2	-2.3209115874	0.459126456	0.9999763851
FARSA	1.1962938132	0.2495377331	0.9999763851
CALR	-1.0665205261	0.5879431423	0.9999763851
RAD23A	1.0564124502	0.7454474222	0.9999763851
GADD45GIP1	1.2748575742	0.0935895955	0.9999763851
DAND5	1.2869631997	0.8237304791	0.9999763851
NFIX	1.7539848782	0.002223	0.9999763851
LYL1	-1.14176961	0.4342653465	0.9999763851
TRMT1	1.1820551979	0.5101565895	0.9999763851
NACC1	1.179963469	0.4371382585	0.9999763851
STX10	-1.2654641634	0.1215484044	0.9999763851
IER2	1.1068412426	0.443562674	0.9999763851
CACNA1A	-5.8625844673	0.1006263162	0.9999763851
CCDC130	-1.329968573	0.3901617482	0.9999763851
MRI1	1.2276033781	0.3161152738	0.9999763851
C19orf53	1.0875709451	0.5955944596	0.9999763851
ZSWIM4	-2.0977020549	0.2415937482	0.9999763851
NANOS3	0	0	0
C19orf57	-1.3142040285	0.6183104986	0.9999763851
CC2D1A	-1.4329683871	0.4727581532	0.9999763851
PODNL1	-6.3477930347	0.0669450725	0.9999763851
DCAF15	1.0299222035	0.9650667014	0.9999763851
RFX1	1.1232814079	0.6696878922	0.9999763851
RLN3	-4.2864494036	0.4027150576	0.9999763851
IL27RA	-1.1665910418	0.3793536522	0.9999763851
LOC113230	-1.3154339304	0.8205576571	0.9999763851
C19orf67	0	0	0
SAMD1	1.350674756	0.681377295	0.9999763851
PRKACA	1.0771122766	0.6877791902	0.9999763851
ASF1B	1.0409118144	0.7898115151	0.9999763851
ADGRL1	1.2204170136	0.2161385347	0.9999763851
ADGRE5	1.1371801064	0.3738363588	0.9999763851
DDX39A	1.4214963773	0.0156647105	0.9999763851
PKN1	1.2652342951	0.5624099629	0.9999763851
GIPC1	1.9447136431	0.00812	0.9999763851
DNAJB1	-1.3798612032	0.0380106382	0.9999763851
TECR	-1.4904620976	0.2746231921	0.9999763851
NDUFB7	-1.0527236871	0.7684223425	0.9999763851
CLEC17A	-4.2849908816	0.4028453076	0.9999763851
ADGRE3	-1.8803545563	0.1642579369	0.9999763851
ZNF333	1.3185653742	0.3728589747	0.9999763851
ADGRE2	1.0144236072	0.9599128542	0.9999763851

OR7A5	0	0	0
SYDE1	3.2353357581	0.554787377	0.9999763851
ILVBL	1.1800650254	0.494192187	0.9999763851
NOTCH3	3.2353357581	0.554787377	0.9999763851
EPHX3	0	0	0
BRD4	-1.1031183158	0.5316358708	0.9999763851
AKAP8	1.0434406395	0.827607578	0.9999763851
AKAP8L	-1.4132215589	0.3460136155	0.9999763851
WIZ	1.2040232924	0.3609816264	0.9999763851
RASAL3	-1.08690536	0.6076477091	0.9999763851
PGLYRP2	0	0	0
CYP4F22	0	0	0
CYP4F3	3.2353357581	0.554787377	0.9999763851
CYP4F12	0	0	0
LOC102724279	0	0	0
TPM4	1.0047475783	0.9794641917	0.9999763851
RAB8A	-1.2147621074	0.318678006	0.9999763851
HSH2D	1.0000956864	0.9996720747	0.9999763851
CIB3	1.1331739081	0.8751456835	0.9999763851
FAM32A	1.0449403751	0.7430281327	0.9999763851
AP1M1	1.0957025428	0.5918646668	0.9999763851
KLF2	-1.3716482783	0.7377357014	0.9999763851
EPS15L1	-1.104850146	0.7032618746	0.9999763851
CALR3	0	0	0
C19orf44	1.2060563862	0.3770068219	0.9999763851
CHERP	-1.2840865018	0.3432705245	0.9999763851
SLC35E1	-1.1171876943	0.5338398185	0.9999763851
MED26	1.0920421038	0.6579561913	0.9999763851
SMIM7	1.2710635424	0.1292625443	0.9999763851
TMEM38A	1.1853704077	0.6685983586	0.9999763851
NWD1	1.3418641175	0.4166434705	0.9999763851
SIN3B	1.0651272837	0.7300792166	0.9999763851
F2RL3	-1.4588494716	0.1731350692	0.9999763851
CPAMD8	1.1163773708	0.8392195656	0.9999763851
HAUS8	1.4539838934	0.0949511196	0.9999763851
MYO9B	1.0575862861	0.7597026515	0.9999763851
USE1	-1.2589321822	0.4099483841	0.9999763851
OCEL1	-1.0360786651	0.9201574857	0.9999763851
NR2F6	-1.0434390209	0.7778087456	0.9999763851
USHBP1	-1.315303824	0.8206171563	0.9999763851
BABAM1	1.0023535744	0.9878339644	0.9999763851
ANKLE1	1.1928462817	0.413396744	0.9999763851
ABHD8	1.2164115278	0.7007122607	0.9999763851
MRPL34	1.168192583	0.3066185549	0.9999763851
DDA1	-1.0342436049	0.8390360265	0.9999763851
ANO8	-2.0965536249	0.2332674201	0.9999763851
GTPBP3	-1.0696056131	0.7130433817	0.9999763851
BST2	-1.3682671155	0.0351949524	0.9999763851
MVB12A	1.0221977985	0.918086836	0.9999763851

TMEM221	0	0	0
SLC27A1	-1.4155138243	0.205309565	0.9999763851
PGLS	1.1772694094	0.4346931168	0.9999763851
FAM129C	1.813344484	0.5820708689	0.9999763851
COLGALT1	-1.1645773907	0.2105388261	0.9999763851
UNC13A	-4.2849908816	0.4028453076	0.9999763851
MAP1S	1.1152142222	0.6120908768	0.9999763851
FCHO1	-1.4825300559	0.3569365986	0.9999763851
B3GNT3	0	0	0
INSL3	-1.3158816768	0.8203229797	0.9999763851
JAK3	1.2479568125	0.2852848647	0.9999763851
RPL18A	1.0362622849	0.7655787262	0.9999763851
SLC5A5	1.3931546432	0.7807621158	0.9999763851
CCDC124	-1.7573998886	0.1404676335	0.9999763851
KCNN1	-4.2849908816	0.4028453076	0.9999763851
ARRDC2	-1.0420578029	0.8290474805	0.9999763851
IL12RB1	-1.3304326759	0.4803856778	0.9999763851
MAST3	-1.3902431567	0.2202203948	0.9999763851
PIK3R2	1.1391041232	0.503317144	0.9999763851
IFI30	1.2213401507	0.1093755748	0.9999763851
MPV17L2	2.006731798	0.0374239167	0.9999763851
RAB3A	3.393068638	0.2345545336	0.9999763851
PDE4C	0	0	0
JUND	-1.2975605905	0.1754813527	0.9999763851
LSM4	1.2314882538	0.1507983672	0.9999763851
PGPEP1	-1.0672426289	0.7225522779	0.9999763851
GDF15	-11.974701451	9.30E-10	4.17E-06
LRRC25	3.2353357581	0.554787377	0.9999763851
SSBP4	1.3691595692	0.123852906	0.9999763851
ISYNA1	-1.0850039112	0.7070786812	0.9999763851
ELL	-1.0851973974	0.6523613288	0.9999763851
FKBP8	1.2327525168	0.6438618149	0.9999763851
KXD1	0	0	0
UBA52	1.258996948	0.0603720892	0.9999763851
C19orf60	1.0678509819	0.7346196341	0.9999763851
CRLF1	-7.5622178268	0.2177145568	0.9999763851
TMEM59L	-1.3938490178	0.711903528	0.9999763851
KLHL26	-1.7743705246	0.0716707166	0.9999763851
CRTC1	1.3715789216	0.3429331257	0.9999763851
UPF1	1.7209669239	0.002821	0.9999763851
GDF1	-2.2884562891	0.1044500762	0.9999763851
CERS1	1.2663175364	0.6611603511	0.9999763851
COPE	-1.118457215	0.6186259207	0.9999763851
DDX49	1.5584694926	0.0337442047	0.9999763851
HOMER3	1.1356334013	0.3498093619	0.9999763851
SUGP2	-1.220507883	0.2297594156	0.9999763851
ARMC6	1.2359134521	0.2974950734	0.9999763851
SLC25A42	1.1334971085	0.6441541083	0.9999763851
TMEM161A	1.0323564321	0.8817325157	0.9999763851

MEF2B	-1.4284672166	0.4088004202	0.9999763851
BORCS8-MEF2B	2.1483330058	0.3686913481	0.9999763851
BORCS8	-1.5426848551	0.1903027911	0.9999763851
RFXANK	1.974984593	0.2028549091	0.9999763851
NR2C2AP	1.2679337948	0.1847407279	0.9999763851
HAPLN4	-4.2849908816	0.4028453076	0.9999763851
SUGP1	-1.0161600208	0.9586017219	0.9999763851
MAU2	-1.2724378149	0.1797417092	0.9999763851
GATAD2A	1.0414624372	0.7734147895	0.9999763851
TSSK6	0	0	0
NDUFA13	-1.036523514	0.8005878088	0.9999763851
YJEFN3	-4.3420972042	0.0743571444	0.9999763851
CILP2	1.0308468109	0.9442482056	0.9999763851
PBX4	-1.4083241465	0.6552973937	0.9999763851
LPAR2	-1.9932034713	0.0716191759	0.9999763851
GMIP	-1.0000082075	0.9999763851	0.9999763851
ATP13A1	1.1508529835	0.5603698158	0.9999763851
ZNF101	1.0968099086	0.7165769868	0.9999763851
ZNF14	0	0	0
ZNF506	-1.6383542693	0.0609889926	0.9999763851
ZNF253	-1.174829898	0.8464488482	0.9999763851
ZNF93	0	0	0
ZNF486	0	0	0
ZNF430	-1.0050487102	0.9848865971	0.9999763851
ZNF714	1.1450199936	0.7659512768	0.9999763851
ZNF431	-1.3508186367	0.5022449639	0.9999763851
ZNF708	1.0588106662	0.8515586677	0.9999763851
ZNF493	0	0	0
ZNF429	0	0	0
ZNF100	-1.1844868221	0.700286769	0.9999763851
ZNF43	3.2353357581	0.554787377	0.9999763851
ZNF729	0	0	0
ZNF730	1.287203384	0.8324480351	0.9999763851
ZNF675	-1.0152663785	0.9641430436	0.9999763851
ZNF254	-1.0200593627	0.9586956875	0.9999763851
UQCRFS1	1.1772909376	0.1689067453	0.9999763851
VSTM2B	0	0	0
POP4	-1.1287755297	0.4340563848	0.9999763851
PLEKHF1	-1.1529109595	0.4840961211	0.9999763851
C19orf12	1.3028919319	0.1954862848	0.9999763851
CCNE1	1.2599541703	0.3200018237	0.9999763851
URI1	-1.0612629508	0.6993543851	0.9999763851
ZNF536	1.7605129081	0.0739351419	0.9999763851
TSHZ3	-1.1778040165	0.5042247148	0.9999763851
ZNF507	-1.4210999367	0.1800575937	0.9999763851
DPY19L3	1.0458725953	0.8575604443	0.9999763851
PDCD5	1.1122836288	0.4877638429	0.9999763851
ANKRD27	-1.0550973845	0.7256109675	0.9999763851
RGS9BP	-5.5346559238	0.0330569473	0.9999763851

NUDT19	1.5375750441	0.0296016008	0.9999763851
TDRD12	3.2353357581	0.554787377	0.9999763851
CEP89	1.550549155	0.2040435653	0.9999763851
FAAP24	1.2712477602	0.5291976717	0.9999763851
RHPN2	2.5210001938	0.2515222021	0.9999763851
GPATCH1	1.0620510624	0.8793102262	0.9999763851
WDR88	1.4731942122	0.5491208182	0.9999763851
LRP3	1.1183780443	0.5624901374	0.9999763851
CEBPA	-1.0187671465	0.8758094332	0.9999763851
CEBPG	-1.126382668	0.506193469	0.9999763851
PEPD	1.1744697588	0.3271218182	0.9999763851
KCTD15	1.7165250546	0.1627603716	0.9999763851
LSM14A	-1.4114598045	0.0347094855	0.9999763851
KIAA0355	-1.4409190307	0.4015704955	0.9999763851
GPI	1.1169672958	0.3933933461	0.9999763851
PDCD2L	-1.0521270905	0.8432000573	0.9999763851
UBA2	1.140056611	0.3031816718	0.9999763851
WTIP	-1.3708355312	0.7472226321	0.9999763851
ZNF302	-1.3065945973	0.6074229245	0.9999763851
ZNF181	-1.0852370907	0.9019261501	0.9999763851
ZNF792	-7.5622178268	0.2177145568	0.9999763851
GRAMD1A	1.1988354147	0.3962738541	0.9999763851
SCN1B	1.7450428903	0.6326558576	0.9999763851
LGI4	0	0	0
FXYD7	5.4776292235	0.3745831165	0.9999763851
FXYD5	1.3897175581	0.0440841643	0.9999763851
LSR	-1.6780739121	0.4519648594	0.9999763851
USF2	1.018900114	0.8874526422	0.9999763851
CD22	1.1262394215	0.7189758098	0.9999763851
FFAR3	-17.404035928	0.0706253351	0.9999763851
FFAR2	2.1587484894	0.0613231554	0.9999763851
SBSN	3.2353357581	0.554787377	0.9999763851
GAPDHS	2.7870243652	0.0983219931	0.9999763851
TMEM147	1.2486827984	0.1181700194	0.9999763851
ATP4A	0	0	0
HAUS5	-1.0607880166	0.826834255	0.9999763851
RBM42	1.1316878188	0.4859249812	0.9999763851
ETV2	2.3404251821	0.4104012847	0.9999763851
COX6B1	1.2649078108	0.0803501159	0.9999763851
UPK1A	0	0	0
ZBTB32	-1.9663843334	0.4762692525	0.9999763851
KMT2B	1.2401084497	0.258106832	0.9999763851
IGFLR1	-1.3392406917	0.2349802551	0.9999763851
U2AF1L4	-2.3871793711	0.2311721631	0.9999763851
PSENEN	1.7168524355	0.0226245132	0.9999763851
LIN37	4.770728625	0.1333582974	0.9999763851
HSPB6	0	0	0
PROSER3	-1.9656080095	0.4359316876	0.9999763851
ARHGAP33	-1.1607449466	0.6901124672	0.9999763851

KIRREL2	2.1108802346	0.1328099687	0.9999763851
APLP1	1.8888297566	0.5936992478	0.9999763851
NFKBID	1.4591297179	0.3162321115	0.9999763851
HCST	1.2162778238	0.1773945926	0.9999763851
TYROBP	-1.0698344336	0.8759333206	0.9999763851
LRFN3	2.782915368	0.0598035455	0.9999763851
SDHAF1	-1.0812139872	0.7547851136	0.9999763851
SYNE4	5.4790129495	0.3774309405	0.9999763851
ALKBH6	1.0854964152	0.7792558418	0.9999763851
LOC101927572	-1.0273204946	0.9450611489	0.9999763851
CLIP3	15.3448820323	0.1557484147	0.9999763851
THAP8	-1.1087957893	0.8313746422	0.9999763851
WDR62	1.5228955501	0.1205806125	0.9999763851
OVOL3	0	0	0
POLR2I	1.0813004963	0.680927184	0.9999763851
TBCB	1.008595631	0.9566565273	0.9999763851
CAPNS1	1.0059641127	0.9706914368	0.9999763851
COX7A1	0	0	0
ZNF565	1.1716150989	0.7789193114	0.9999763851
ZNF146	-1.282320363	0.2028167784	0.9999763851
ZFP14	-1.942139423	0.1365790865	0.9999763851
ZFP82	3.2353357581	0.554787377	0.9999763851
ZNF566	-1.1576370621	0.746501348	0.9999763851
ZNF260	1.3021448763	0.3474622915	0.9999763851
ZNF529	1.0454950652	0.8789054797	0.9999763851
ZNF461	-2.0611818492	0.296684002	0.9999763851
ZNF567	-1.0030988635	0.9907191506	0.9999763851
ZNF850	1.2311394327	0.4950012034	0.9999763851
LOC728485	0	0	0
ZNF790	0	0	0
ZNF345	2.3412218143	0.4038852691	0.9999763851
ZNF829	-2.3214980365	0.437464777	0.9999763851
ZNF568	0	0	0
ZNF420	-1.4428110141	0.297842089	0.9999763851
ZNF585A	24.0213517934	0.0971698279	0.9999763851
ZNF585B	5.4784711963	0.3745358536	0.9999763851
ZNF383	-1.087491429	0.8275230563	0.9999763851
HKR1	-1.9857123279	0.1461566059	0.9999763851
ZNF527	1.9110845344	0.2046502702	0.9999763851
ZNF569	-1.0537249313	0.9204722266	0.9999763851
ZNF570	1.9661194815	0.3814700456	0.9999763851
ZNF793	0	0	0
ZNF540	3.2356557963	0.5547505991	0.9999763851
ZNF571	5.4790129495	0.3776249757	0.9999763851
ZFP30	0	0	0
ZNF607	0	0	0
SIPA1L3	-1.194843005	0.5213605425	0.9999763851
DPF1	1.2120222182	0.5137053162	0.9999763851
SPINT2	1.624741281	0.5893738657	0.9999763851

YIF1B	1.0025147799	0.9921767598	0.9999763851
C19orf33	0	0	0
KCNK6	-1.039143791	0.8670370476	0.9999763851
CATSPERG	-2.8431696679	0.2709621775	0.9999763851
PSMD8	-1.0192178655	0.8945608085	0.9999763851
SPRED3	1.1536776807	0.8524553733	0.9999763851
FAM98C	1.022245923	0.9501898781	0.9999763851
RASGRP4	2.2027818203	0.3663459935	0.9999763851
RYR1	-7.5622178268	0.2177145568	0.9999763851
MAP4K1	1.1209913177	0.6223630361	0.9999763851
EIF3K	1.0383768195	0.7603377663	0.9999763851
ACTN4	1.2976809049	0.1145552614	0.9999763851
CAPN12	-1.3796902431	0.012665087	0.9999763851
LGALS4	0	0	0
ECH1	1.1721289439	0.2799094086	0.9999763851
HNRNPL	-1.2733889453	0.0931311678	0.9999763851
RINL	1.0802422362	0.8512795235	0.9999763851
SIRT2	-1.1697994047	0.3073362798	0.9999763851
NFKBIB	1.0200362961	0.924682609	0.9999763851
CCER2	-2.5616934468	0.2769879973	0.9999763851
SARS2	-1.3222868533	0.2987108518	0.9999763851
MRPS12	1.1190552069	0.4674276995	0.9999763851
FBXO17	0	0	0
ACP7	0	0	0
PAK4	-7.5622178268	0.2177145568	0.9999763851
LRFN1	-3.9243769581	0.0578185971	0.9999763851
GMFG	1.1419539468	0.2943796043	0.9999763851
SAMD4B	1.4744681007	0.0652897265	0.9999763851
PAF1	-1.0365571328	0.877355226	0.9999763851
MED29	1.1056219059	0.7161951057	0.9999763851
ZFP36	-1.0677426957	0.9246624071	0.9999763851
PLEKHG2	1.1732088536	0.6045118843	0.9999763851
RPS16	1.1132725498	0.3724981715	0.9999763851
SUPT5H	-1.0265546878	0.8641094878	0.9999763851
TIMM50	1.3030867946	0.2430070654	0.9999763851
DLL3	-1.0182668643	0.9338421726	0.9999763851
EID2B	-1.5846035454	0.3623022762	0.9999763851
EID2	1.0050574537	0.9831775339	0.9999763851
CLC	0	0	0
DYRK1B	1.2870761236	0.8160709833	0.9999763851
FBL	1.2141608841	0.1396279423	0.9999763851
FCGBP	3.2353357581	0.554787377	0.9999763851
PSMC4	1.1812636775	0.4169669492	0.9999763851
ZNF546	-1.3438516515	0.5337433512	0.9999763851
ZNF780B	1.0312722972	0.9328013402	0.9999763851
ZNF780A	-1.3748759807	0.4110451153	0.9999763851
MAP3K10	1.0274983165	0.9757178065	0.9999763851
TTC9B	0	0	0
AKT2	-1.0366528986	0.7824341187	0.9999763851

C19orf47	1.3953182585	0.0790198063	0.9999763851
PLD3	1.1743261678	0.5845949101	0.9999763851
HIPK4	3.2356557963	0.5547505991	0.9999763851
PRX	1.0617559242	0.8941938323	0.9999763851
SERTAD1	-1.1190427674	0.6521815279	0.9999763851
SERTAD3	-1.9475004969	0.3568459862	0.9999763851
BLVRB	-1.103208099	0.5474918928	0.9999763851
SPTBN4	-3.1553169002	0.1652074601	0.9999763851
SHKBP1	1.3015885853	0.1676048204	0.9999763851
LTBP4	-1.0873265326	0.8073260356	0.9999763851
NUMBL	-1.1144198402	0.6051521208	0.9999763851
ADCK4	1.3541179499	0.6294672651	0.9999763851
ITPKC	1.0765594593	0.8962739866	0.9999763851
C19orf54	1.3673573351	0.4965025997	0.9999763851
SNRPA	-1.0429569508	0.8030501225	0.9999763851
MIA	-4.2849908816	0.4028453076	0.9999763851
RAB4B	1.3303015586	0.4237052938	0.9999763851
EGLN2	-1.0672456891	0.6909986646	0.9999763851
CYP2A6	-7.5647903763	0.2176246281	0.9999763851
CYP2F1	0	0	0
CYP2S1	-2.3560083799	0.0641377222	0.9999763851
AXL	0	0	0
HNRNPUL1	1.109375286	0.4258483918	0.9999763851
CCDC97	1.4894233582	0.1695499826	0.9999763851
TGFB1	1.2275260648	0.4393872213	0.9999763851
B9D2	1.4262436006	0.2773051974	0.9999763851
TMEM91	-1.4336294793	0.4716756365	0.9999763851
EXOSC5	1.2381162237	0.327317703	0.9999763851
BCKDHA	-1.0446883288	0.7808739272	0.9999763851
B3GNT8	2.1044296444	0.1878333908	0.9999763851
ATP5SL	1.0180386352	0.9356427133	0.9999763851
CEACAM21	-1.0674131201	0.8158938724	0.9999763851
CEACAM4	-1.0988817322	0.741850741	0.9999763851
CEACAM5	3.9201049863	0.1888099258	0.9999763851
CEACAM6	1.366958711	0.0159119563	0.9999763851
CEACAM3	3.2356557963	0.5547505991	0.9999763851
LYPD4	3.2353357581	0.554787377	0.9999763851
RPS19	-1.1704762672	0.1862745414	0.9999763851
CD79A	1.0381604432	0.8810816681	0.9999763851
ARHGEF1	1.0595436806	0.797740665	0.9999763851
LOC390937	-2.6249844518	0.2433007306	0.9999763851
RABAC1	1.0887455155	0.6769241019	0.9999763851
GRIK5	3.2361532718	0.5546934395	0.9999763851
ZNF574	-1.2596555089	0.5258323709	0.9999763851
POU2F2	1.1864798477	0.5293596068	0.9999763851
DEDD2	1.3836654999	0.1331275035	0.9999763851
ZNF526	3.7495951467	0.0246731341	0.9999763851
GSK3A	1.2241412544	0.3701637809	0.9999763851
ERF	-1.0657115305	0.7837117845	0.9999763851

CIC	1.5005838045	0.2791406121	0.9999763851
PAFAH1B3	1.2248115336	0.2011585062	0.9999763851
PRR19	2.2305031351	0.3590599981	0.9999763851
TMEM145	1.6242600155	0.5618938402	0.9999763851
MEGF8	1.0140526777	0.9698364098	0.9999763851
CNFN	1.455553068	0.4718050591	0.9999763851
LIPE	1.0763878196	0.9030624767	0.9999763851
CXCL17	-4.2849908816	0.4028453076	0.9999763851
CEACAM1	1.2869488883	0.821524196	0.9999763851
CEACAM8	0	0	0
TEX101	1.1427172643	0.7752940779	0.9999763851
LYPD3	1.0277462901	0.9757639819	0.9999763851
PHLDB3	2.0209619459	0.1693862273	0.9999763851
ETHE1	1.2030676682	0.3063933712	0.9999763851
ZNF575	-1.4420864275	0.3613565856	0.9999763851
XRCC1	-1.5486171134	0.4223561277	0.9999763851
PINLYP	-4.0879703482	0.0149316273	0.9999763851
IRGQ	-1.4980248305	0.2369980283	0.9999763851
ZNF576	-1.1802836473	0.4536333322	0.9999763851
ZNF428	-1.1463823201	0.4346741804	0.9999763851
CADM4	1.0273967864	0.9753772823	0.9999763851
PLAUR	-2.5494303999	5.84E-08	0.0001849
SMG9	1.0643686479	0.8602521217	0.9999763851
KCNN4	-1.3414473019	0.0782650218	0.9999763851
LYPD5	0	0	0
ZNF283	0	0	0
ZNF45	-1.0416330641	0.9063517637	0.9999763851
ZNF221	-4.2849908816	0.4028453076	0.9999763851
ZNF155	-1.5437509479	0.349932048	0.9999763851
ZNF230	-1.063351306	0.8895821798	0.9999763851
ZNF222	5.4770877341	0.3746135179	0.9999763851
ZNF284	0	0	0
ZNF224	-1.0099162218	0.9847656349	0.9999763851
ZNF225	1.225293246	0.7587253944	0.9999763851
ZNF226	-1.7095938638	0.0699030905	0.9999763851
ZNF227	-1.2068284073	0.4516690809	0.9999763851
ZNF233	2.3412218143	0.403714452	0.9999763851
ZNF235	16.6918753579	0.1319658723	0.9999763851
ZNF229	0	0	0
ZNF180	1.1121894999	0.7504108903	0.9999763851
CEACAM20	0	0	0
PVR	-1.1416513844	0.4669493586	0.9999763851
CEACAM19	2.3390875834	0.423386324	0.9999763851
BCL3	1.8670053592	0.0541134572	0.9999763851
BCAM	0	0	0
NECTIN2	1.3272955974	0.4503217028	0.9999763851
TOMM40	1.2365766771	0.2393519921	0.9999763851
APOE	-1.3151016298	0.8211851345	0.9999763851
APOC4	0	0	0

APOC2	-1.068702738	0.8808334266	0.9999763851
CLPTM1	1.4290402066	0.4325616374	0.9999763851
RELB	1.0026756297	0.9904703416	0.9999763851
CLASRP	-1.002857596	0.9914675148	0.9999763851
ZNF296	1.9583043179	0.1676626037	0.9999763851
GEMIN7	1.1834480789	0.6448037116	0.9999763851
PPP1R37	-1.01653746	0.9690241408	0.9999763851
NKPD1	-1.9649399627	0.4616023013	0.9999763851
TRAPPC6A	-1.1606377885	0.3749032272	0.9999763851
BLOC1S3	1.0689667084	0.8563521145	0.9999763851
MARK4	-1.5635414544	0.0708552079	0.9999763851
KLC3	-1.5912207724	0.2297503593	0.9999763851
ERCC2	1.2457882067	0.5862528268	0.9999763851
PPP1R13L	1.5485442135	0.4096446861	0.9999763851
CD3EAP	1.1013421799	0.5666060588	0.9999763851
ERCC1	-1.0111807915	0.9752343262	0.9999763851
FOSB	1.8535308482	0.2746503873	0.9999763851
RTN2	-1.2692341856	0.5758578917	0.9999763851
PPM1N	-1.5528893325	0.6370113367	0.9999763851
VASP	-1.4327478803	0.0125423453	0.9999763851
OPA3	1.5617030755	0.0391911105	0.9999763851
GPR4	-4.2864494036	0.4027150576	0.9999763851
EML2	-1.0687677289	0.7416380977	0.9999763851
GIPR	-1.3944153431	0.6946684156	0.9999763851
SNRPD2	1.113381032	0.3721558782	0.9999763851
QPCTL	-1.2932039271	0.6293083934	0.9999763851
FBXO46	-1.2590527892	0.2699124358	0.9999763851
BHMG1	0	0	0
SIX5	-1.3939868197	0.7079482006	0.9999763851
DMPK	0	0	0
DMWD	-1.1490733186	0.858679221	0.9999763851
SYMPK	-1.0767178987	0.7216211299	0.9999763851
FOXA3	0	0	0
MYPOP	-1.3960016768	0.1607620593	0.9999763851
NANOS2	0	0	0
CCDC61	1.4538178923	0.4747774916	0.9999763851
HIF3A	3.2353357581	0.554787377	0.9999763851
PPP5C	1.0740835145	0.8202663644	0.9999763851
PPP5D1	-4.3494677458	0.0470903699	0.9999763851
CALM3	-1.0411206757	0.7552019338	0.9999763851
PTGIR	-1.3161718537	0.8295213067	0.9999763851
GNG8	0	0	0
DACT3	-14.1139127893	0.1036616799	0.9999763851
PRKD2	-1.0583648998	0.8091138058	0.9999763851
LOC107987269	0	0	0
STRN4	1.3423800262	0.2162409437	0.9999763851
FKRP	2.6298086381	0.0977192164	0.9999763851
SLC1A5	-1.1994240326	0.1760075585	0.9999763851
AP2S1	1.0393096086	0.8169524361	0.9999763851

ARHGAP35	1.1967377613	0.3890986999	0.9999763851
NPAS1	-1.6524072588	0.2843434323	0.9999763851
TMEM160	1.1249404561	0.623597631	0.9999763851
ZC3H4	1.0812500535	0.6385480359	0.9999763851
SAE1	1.0464893113	0.7145275517	0.9999763851
BBC3	-1.661239928	0.0209794549	0.9999763851
CCDC9	-2.5516036663	0.1117933054	0.9999763851
INAFM1	2.0227082105	0.0683638421	0.9999763851
C5AR1	-1.3951494284	0.6924416979	0.9999763851
C5AR2	3.2353357581	0.554787377	0.9999763851
DHX34	1.3096884437	0.3858037405	0.9999763851
MEIS3	-5.3420894951	0.1069892491	0.9999763851
SLC8A2	0	0	0
KPTN	-1.1095493821	0.8171788564	0.9999763851
NAPA	1.4176889383	0.0627529124	0.9999763851
ZNF541	1.321204212	0.6513570406	0.9999763851
GLTSCR1	1.1671718	0.7542152158	0.9999763851
EHD2	-1.1712402541	0.7053738199	0.9999763851
GLTSCR2	1.1616039842	0.6158765615	0.9999763851
SEPW1	-1.1272745011	0.5108096469	0.9999763851
SULT2A1	0	0	0
BSPH1	0	0	0
ELSPBP1	0	0	0
PLA2G4C	3.2353357581	0.554787377	0.9999763851
LIG1	-1.0675033587	0.7061260964	0.9999763851
C19orf68	-1.0723377799	0.8278574481	0.9999763851
CARD8	-1.1064731205	0.5296619953	0.9999763851
ZNF114	-1.136490973	0.7996290466	0.9999763851
CCDC114	1.8888297566	0.5936560099	0.9999763851
EMP3	1.0400848455	0.8336817388	0.9999763851
TMEM143	-1.2216551083	0.6572253979	0.9999763851
SYNGR4	-1.3161718537	0.8297458114	0.9999763851
KDELR1	-1.1123007939	0.5025335538	0.9999763851
GRIN2D	-1.3751873623	0.3821126918	0.9999763851
GRWD1	1.0279248939	0.8519975167	0.9999763851
KCNJ14	1.0477755851	0.8933885045	0.9999763851
CYTH2	1.1145325251	0.6188708424	0.9999763851
LMTK3	1.0308108187	0.9549662693	0.9999763851
SULT2B1	-4.287394647	0.4026306792	0.9999763851
FAM83E	1.2872739558	0.8230399183	0.9999763851
RPL18	1.0198195528	0.8788417354	0.9999763851
SPHK2	-1.3493136485	0.3861514809	0.9999763851
DBP	-1.0785712517	0.7543578431	0.9999763851
CA11	-1.6023531879	0.1434093561	0.9999763851
NTN5	-7.5664585246	0.2254051562	0.9999763851
FUT2	-1.4081078364	0.6612397999	0.9999763851
MAMSTR	-2.5591355727	0.2872197658	0.9999763851
RASIP1	-1.0343869119	0.9624580852	0.9999763851
IZUMO1	0	0	0

FUT1	0	0	0
BCAT2	1.0105452535	0.9599240277	0.9999763851
HSD17B14	-4.3352959826	0.1432739397	0.9999763851
PLEKHA4	-7.5977820511	0.002836	0.9999763851
PPP1R15A	-1.2745292358	0.1461808215	0.9999763851
TULP2	-4.287394647	0.4026306792	0.9999763851
NUCB1	1.3190344221	0.2414531077	0.9999763851
DHDH	5.4790129495	0.3778264384	0.9999763851
BAX	-1.629117792	0.3238138062	0.9999763851
FTL	-1.0051865821	0.9647772864	0.9999763851
GYS1	1.1448717847	0.4899095965	0.9999763851
RUVBL2	-1.1077362151	0.5325007457	0.9999763851
CGB7	-4.287394647	0.4026306792	0.9999763851
SNRNP70	1.1732365625	0.2275842871	0.9999763851
LIN7B	1.2887116085	0.6524852855	0.9999763851
C19orf73	7.7204093521	0.2874544417	0.9999763851
PPFIA3	-1.4417878993	0.4169973529	0.9999763851
TRPM4	-1.1262199596	0.567473403	0.9999763851
SLC6A16	1.0605024554	0.9032518638	0.9999763851
CD37	-1.0431939783	0.7776884927	0.9999763851
TEAD2	-2.2372730517	0.3101430576	0.9999763851
DKKL1	-1.9496368441	0.3265476433	0.9999763851
CCDC155	1.287203384	0.8328960235	0.9999763851
SLC17A7	0	0	0
PIH1D1	-1.0011084901	0.9951409641	0.9999763851
ALDH16A1	1.1355003882	0.4593998874	0.9999763851
FLT3LG	-1.3968316357	0.6231752741	0.9999763851
RPL13A	-1.0518943864	0.6629629285	0.9999763851
RPS11	1.1678348468	0.2146361635	0.9999763851
FCGRT	-1.0356988155	0.8341891284	0.9999763851
RCN3	5.4776292235	0.3745831165	0.9999763851
NOSIP	1.3080638019	0.2155709172	0.9999763851
PRRG2	2.3396033966	0.4194162393	0.9999763851
PRR12	1.0728812783	0.8309405961	0.9999763851
RRAS	-1.3871978116	0.3767950736	0.9999763851
SCAF1	-2.6584638529	0.2215162537	0.9999763851
IRF3	1.049196881	0.8252494282	0.9999763851
BCL2L12	1.0591129027	0.7106976928	0.9999763851
PRMT1	-1.0998424179	0.546788991	0.9999763851
ADM5	1.4251505961	0.4231625047	0.9999763851
TSKS	-1.3154339304	0.8205576571	0.9999763851
AP2A1	-1.6388669327	0.2788528738	0.9999763851
FUZ	1.5105242113	0.5630238503	0.9999763851
MED25	-1.7854938727	0.003728	0.9999763851
PTOV1	1.1442499687	0.4675560649	0.9999763851
PNKP	1.2255983844	0.5128723529	0.9999763851
AKT1S1	-2.4060941143	0.0543292565	0.9999763851
TBC1D17	-1.2770352695	0.6501125273	0.9999763851
IL4I1	-1.1513118791	0.7190317952	0.9999763851

NUP62	1.1903578148	0.1943777625	0.9999763851
ATF5	-1.0057960537	0.9748064755	0.9999763851
VRK3	-1.2229879211	0.3565817158	0.9999763851
ZNF473	1.2067235764	0.4878643051	0.9999763851
MYH14	-1.315303824	0.8206171563	0.9999763851
NAPSA	0	0	0
NR1H2	2.1303847456	0.0203213037	0.9999763851
POLD1	1.0289925255	0.8999166998	0.9999763851
FAM71E1	-4.2849908816	0.4028453076	0.9999763851
EMC10	1.1349048304	0.5110679374	0.9999763851
JOSD2	-1.2556552222	0.7305609006	0.9999763851
ASPDH	0	0	0
LRRC4B	5.4784711963	0.3745358536	0.9999763851
SYT3	14.4482274128	0.150899916	0.9999763851
C19orf81	1.3262316595	0.7525282844	0.9999763851
SHANK1	1.178525712	0.6299590747	0.9999763851
CLEC11A	1.0727650825	0.6812810122	0.9999763851
ACPT	2.5187299588	0.2457619387	0.9999763851
C19orf48	1.0717669009	0.7770421264	0.9999763851
KLK1	0	0	0
KLK6	3.2353357581	0.554787377	0.9999763851
CTU1	1.9904122338	0.287607171	0.9999763851
SIGLEC9	1.8144198156	0.5860132782	0.9999763851
SIGLEC7	-1.443902089	0.3292552504	0.9999763851
CD33	1.049246854	0.7644264115	0.9999763851
SIGLECL1	0	0	0
IGLON5	0	0	0
VSIG10L	-2.461315534	0.2088619118	0.9999763851
ETFB	1.2487341727	0.1826772009	0.9999763851
CLDND2	-2.156860942	0.0197790359	0.9999763851
NKG7	1.079839729	0.6225749946	0.9999763851
SIGLEC10	1.2033077928	0.4789055168	0.9999763851
SIGLEC8	-2.4274267559	0.4444039917	0.9999763851
SIGLEC12	1.0869683407	0.7276379404	0.9999763851
SIGLEC6	-1.2763103583	0.1994636503	0.9999763851
ZNF175	2.3109853843	0.1780900813	0.9999763851
SIGLEC5	1.0715381111	0.6991735065	0.9999763851
SIGLEC14	1.1985595417	0.4650412219	0.9999763851
SPACA6	-1.1907355565	0.533735572	0.9999763851
HAS1	0	0	0
FPR1	-4.3342515471	0.1410261823	0.9999763851
FPR2	-1.0944884231	0.7128019938	0.9999763851
FPR3	-4.2849908816	0.4028453076	0.9999763851
ZNF577	2.9478558986	0.0691812863	0.9999763851
ZNF649	-2.2030645698	0.1688809464	0.9999763851
ZNF613	3.3935897477	0.2591478647	0.9999763851
ZNF614	-1.5930784051	0.2579458416	0.9999763851
ZNF432	3.6398918228	0.0416544689	0.9999763851
ZNF616	1.5493902735	0.3957245407	0.9999763851

PPP2R1A	1.0748334908	0.6200826654	0.9999763851
ZNF766	-1.2626839641	0.538898979	0.9999763851
ZNF480	1.174316906	0.4675532091	0.9999763851
ZNF528	0	0	0
ZNF578	0	0	0
ZNF808	-4.2864494036	0.4027150576	0.9999763851
ZNF701	-4.2849908816	0.4028453076	0.9999763851
ZNF83	1.2196138221	0.4346787527	0.9999763851
ZNF611	1.07432532	0.8555485013	0.9999763851
ZNF28	1.0296736943	0.9675240215	0.9999763851
ZNF468	1.4334329897	0.4259407477	0.9999763851
ZNF320	-4.2864494036	0.4027150576	0.9999763851
ZNF888	1.1341380884	0.6968534675	0.9999763851
ZNF816-ZNF321P	-1.0853248114	0.8761045508	0.9999763851
ZNF816	3.2353357581	0.554787377	0.9999763851
ZNF160	-1.0571636065	0.8736442425	0.9999763851
ZNF415	3.2353357581	0.554787377	0.9999763851
ZNF347	0	0	0
ZNF665	-1.3154339304	0.8205576571	0.9999763851
ZNF677	0	0	0
ZNF845	1.6724224215	0.3465125467	0.9999763851
ZNF761	-1.2058523068	0.6360230421	0.9999763851
ZNF813	0	0	0
ZNF331	0	0	0
NLRP12	1.9656449183	0.36154752	0.9999763851
MYADM	-1.0236776646	0.8771176713	0.9999763851
CACNG6	0	0	0
OSCAR	1.6411409338	0.0360841278	0.9999763851
NDUFA3	-1.1704791342	0.3323736151	0.9999763851
TFPT	1.0755760503	0.737005642	0.9999763851
PRPF31	1.3970834559	0.060304803	0.9999763851
CNOT3	1.0615476676	0.8537583953	0.9999763851
LENG1	1.339639958	0.6059754243	0.9999763851
TMC4	-20.6829910744	0.0563788217	0.9999763851
MBOAT7	-1.0718011147	0.7027683832	0.9999763851
TSEN34	1.0371678772	0.8136438245	0.9999763851
RPS9	-1.106439253	0.4327492923	0.9999763851
LILRB3	-1.8163232553	0.4523888332	0.9999763851
LILRB2	1.6933529607	0.2997621671	0.9999763851
LILRA5	0	0	0
LILRA4	0	0	0
LAIR1	-1.09273269	0.5987598898	0.9999763851
TTYH1	-6.0681194476	0.0987663897	0.9999763851
LENG8	1.3410024408	0.7175063525	0.9999763851
LENG9	-1.0590040249	0.8394594159	0.9999763851
LAIR2	-2.3214980365	0.4375012815	0.9999763851
LILRA2	1.2596261728	0.1273875514	0.9999763851
LILRA1	1.2872739558	0.8230618026	0.9999763851
LILRB1	-4.3497596498	0.0424561003	0.9999763851

LILRB4	-4.2864494036	0.4027150576	0.9999763851
KIR2DL3	-4.2864494036	0.4027150576	0.9999763851
KIR2DL1	0	0	0
KIR2DL4	7.7488609396	0.3058529326	0.9999763851
KIR3DL2	1.6245986645	0.5891121414	0.9999763851
FCAR	1.3987124065	0.058184229	0.9999763851
NCR1	-1.7405372638	0.3492323171	0.9999763851
NLRP7	1.2335574408	0.4277296328	0.9999763851
NLRP2	-1.4204723584	0.6090314739	0.9999763851
GP6	-1.817658111	0.4899045682	0.9999763851
RDH13	-1.0610519349	0.7618954391	0.9999763851
EPS8L1	-2.0459777417	0.1652741858	0.9999763851
PPP1R12C	-1.0332644239	0.8711410271	0.9999763851
TNNT1	-1.1123683504	0.5554711241	0.9999763851
TNNI3	1.4680150042	0.4822226802	0.9999763851
DNAAF3	1.4352142164	0.2459729858	0.9999763851
SYT5	-1.3161718537	0.8299542244	0.9999763851
PTPRH	-1.3946909469	0.7145899032	0.9999763851
TMEM86B	1.4728639419	0.256351474	0.9999763851
PPP6R1	1.022828758	0.8732357292	0.9999763851
HSPBP1	1.1403359424	0.545350267	0.9999763851
BRSK1	0	0	0
TMEM150B	-1.0552520483	0.9183054086	0.9999763851
KMT5C	-1.4424774004	0.0865423284	0.9999763851
COX6B2	0	0	0
IL11	-1.5420290394	0.6711533512	0.9999763851
TMEM238	-1.0854185322	0.8773319942	0.9999763851
RPL28	-1.0069314725	0.9582969321	0.9999763851
UBE2S	-1.2228123551	0.2270779935	0.9999763851
ISOC2	1.6565793297	0.1624366642	0.9999763851
ZNF628	-1.2489704587	0.5602733251	0.9999763851
NAT14	1.1994236376	0.6790810627	0.9999763851
SBK2	0	0	0
ZNF579	1.0457585792	0.8944063195	0.9999763851
FIZ1	1.0312908337	0.9224493132	0.9999763851
ZNF524	1.6222611763	0.1741476053	0.9999763851
ZNF865	1.2505247135	0.5434864404	0.9999763851
ZNF784	1.2371469701	0.5696714321	0.9999763851
LOC107983998	-1.3711818216	0.7361262837	0.9999763851
ZNF581	-1.1358401809	0.5545121912	0.9999763851
ZNF580	-1.1333762761	0.6534102773	0.9999763851
CCDC106	3.3942235682	0.2227323437	0.9999763851
U2AF2	-1.1546023052	0.3280070159	0.9999763851
EPN1	1.3485884002	0.1818976801	0.9999763851
NLRP9	-2.3212684229	0.4489510253	0.9999763851
NLRP5	0	0	0
ZNF787	-1.0417738745	0.8614618061	0.9999763851
ZNF444	-1.2681082708	0.2830006094	0.9999763851
ZSCAN5A	-1.008604596	0.9643922311	0.9999763851

ZNF583	-1.3962845548	0.7156673328	0.9999763851
ZNF667	0	0	0
ZNF471	0	0	0
ZNF470	1.6243491221	0.5675493249	0.9999763851
ZNF71	-1.4897297417	0.2193031866	0.9999763851
ZIM2	0	0	0
DUXA	0	0	0
ZNF805	-1.1000773412	0.7917505987	0.9999763851
ZNF460	1.3942139156	0.1134600985	0.9999763851
ZNF547	-1.2730325932	0.584169862	0.9999763851
ZNF548	-1.4714438827	0.4307362296	0.9999763851
ZNF17	-1.2518413874	0.6243462905	0.9999763851
ZNF749	1.2910602585	0.408323903	0.9999763851
VN1R1	-4.2864494036	0.4027150576	0.9999763851
ZNF772	0	0	0
ZNF419	-1.2721074713	0.3475195954	0.9999763851
ZNF773	1.1042539188	0.6749893818	0.9999763851
ZNF549	-1.4392727316	0.4399516201	0.9999763851
ZNF550	1.0338216758	0.9015762845	0.9999763851
ZNF416	3.1161214639	0.1563424467	0.9999763851
ZNF530	-1.0880053136	0.8594853871	0.9999763851
ZNF134	1.1436909671	0.7539542115	0.9999763851
ZNF211	-2.3251540141	0.0862439704	0.9999763851
ZNF551	3.0394080913	0.0469339881	0.9999763851
ZNF154	3.9208783318	0.1839380568	0.9999763851
ZNF671	9.9614219348	0.2209163234	0.9999763851
ZNF776	3.4241220592	0.0665997038	0.9999763851
ZNF586	3.2353357581	0.554787377	0.9999763851
ZNF552	1.4324468288	0.4169058051	0.9999763851
ZNF587B	-1.1006912859	0.7868786466	0.9999763851
ZNF587	-1.0103676967	0.978955517	0.9999763851
ZNF814	-1.7069221799	0.3519969811	0.9999763851
ZNF417	-1.3948129396	0.6826169491	0.9999763851
C19orf18	0	0	0
ZNF606	0	0	0
ZNF135	3.2361532718	0.5546934395	0.9999763851
ZSCAN18	0	0	0
ZNF274	-1.2473340862	0.5298238395	0.9999763851
ZNF544	-1.4430036527	0.3345368996	0.9999763851
ZNF8	2.2219096964	0.3519102996	0.9999763851
ZSCAN22	1.35901596	0.6150212849	0.9999763851
A1BG	-1.4906022544	0.1757249258	0.9999763851
ZNF497	-1.5528755355	0.3187380754	0.9999763851
ZNF837	0	0	0
RPS5	1.1272394736	0.3247648756	0.9999763851
RNF225	0	0	0
ZNF584	1.3715279496	0.2177673837	0.9999763851
ZNF132	1.2869488883	0.8215749962	0.9999763851
ZNF324B	-3.5020254147	0.0785511656	0.9999763851

ZNF324	1.0121024616	0.9756497553	0.9999763851
ZNF446	-1.5299337314	0.4579746563	0.9999763851
SLC27A5	1.2133349159	0.5104526526	0.9999763851
ZBTB45	2.0283988866	0.0206459548	0.9999763851
TRIM28	1.1694042773	0.2976039341	0.9999763851
CHMP2A	-1.061457915	0.674060491	0.9999763851
UBE2M	-1.1966910128	0.2538782382	0.9999763851
MZF1	1.0953709777	0.7953458226	0.9999763851
C20orf96	1.0256422978	0.937069879	0.9999763851
ZCCHC3	1.0784531001	0.623441848	0.9999763851
SOX12	-1.0366341859	0.8128296771	0.9999763851
LOC101929937	3.2356557963	0.5547505991	0.9999763851
NRSN2	-4.2864494036	0.4027150576	0.9999763851
TRIB3	1.116888451	0.4577910289	0.9999763851
RBCK1	1.1017473261	0.5508620874	0.9999763851
TBC1D20	-1.4286529559	0.0449845336	0.9999763851
CSNK2A1	-1.2219251045	0.2591121791	0.9999763851
SRXN1	-1.0726498826	0.7283908537	0.9999763851
SCRT2	0	0	0
FAM110A	-1.0191324298	0.9351060089	0.9999763851
ANGPT4	-1.3716482783	0.7374997556	0.9999763851
PSMF1	1.1571878611	0.2996215233	0.9999763851
TMEM74B	-2.3220580825	0.4349418702	0.9999763851
C20orf202	3.2353357581	0.554787377	0.9999763851
SNPH	3.2353357581	0.554787377	0.9999763851
SDCBP2	-1.0054744133	0.9910361459	0.9999763851
FKBP1A	-1.0612378916	0.6560949646	0.9999763851
NSFL1C	1.2049561558	0.2304930763	0.9999763851
SIRPB2	-2.8635001401	0.2946223333	0.9999763851
SIRPD	1.7501499392	0.0303892363	0.9999763851
SIRPB1	1.22555435	0.6127810454	0.9999763851
SIRPG	1.5892415096	0.4866236646	0.9999763851
LOC107984104	1.3583656505	0.6871858489	0.9999763851
SIRPA	1.0446830517	0.7677106636	0.9999763851
PDYN	-26.1866591561	0.0435410495	0.9999763851
STK35	1.0015682699	0.9919422365	0.9999763851
LOC388780	1.0451823316	0.8975258825	0.9999763851
TGM3	1.3974818312	0.2716342407	0.9999763851
TGM6	0	0	0
SNRPB	1.0840197895	0.5229016055	0.9999763851
ZNF343	1.0554341485	0.9046100388	0.9999763851
NOP56	1.0411506962	0.7537898185	0.9999763851
IDH3B	-1.2437448903	0.1412179723	0.9999763851
EBF4	-1.2296063424	0.7622382116	0.9999763851
CPXM1	0	0	0
PCED1A	-1.0916357762	0.7085210892	0.9999763851
VPS16	-1.062309553	0.8223844034	0.9999763851
PTPRA	-1.0111486878	0.9562062612	0.9999763851
GNRH2	5.4781708352	0.3745527127	0.9999763851

MRPS26	1.3269750954	0.1118834174	0.9999763851
UBOX5	1.1614912412	0.6158719692	0.9999763851
FASTKD5	1.2957928355	0.0968531147	0.9999763851
LZTS3	-1.0810230665	0.9183369404	0.9999763851
DDRGK1	1.1965780279	0.3746792109	0.9999763851
ITPA	1.0009770077	0.9942557113	0.9999763851
SLC4A11	-2.8024905865	0.0364495397	0.9999763851
C20orf194	-1.2918426844	0.511986203	0.9999763851
ATRN	-1.080288086	0.7005342013	0.9999763851
SIGLEC1	-14.2871033531	0.0106283846	0.9999763851
HSPA12B	3.9191576211	0.181765289	0.9999763851
C20orf27	1.3899390486	0.0438140024	0.9999763851
SPEF1	3.2356557963	0.5547505991	0.9999763851
CENPB	1.3159185192	0.0788341143	0.9999763851
CDC25B	1.3171512711	0.0997053685	0.9999763851
AP5S1	-1.407612475	0.2383897358	0.9999763851
MAVS	1.0303869215	0.8396320527	0.9999763851
PANK2	1.200549583	0.2700036359	0.9999763851
RNF24	1.0572840473	0.7358725403	0.9999763851
SMOX	1.07406911	0.8180216435	0.9999763851
ADRA1D	3.2356557963	0.5547505991	0.9999763851
PRNP	1.0670917926	0.6793827102	0.9999763851
RASSF2	1.4166075663	0.013662753	0.9999763851
SLC23A2	-1.3527418065	0.1389924147	0.9999763851
TMEM230	1.1108441324	0.4572856505	0.9999763851
PCNA	1.4489432893	0.0129445846	0.9999763851
CDS2	1.0130674744	0.9525234843	0.9999763851
GPCPD1	1.0685866958	0.7087445891	0.9999763851
C20orf196	1.7838351596	0.0709210323	0.9999763851
CHGB	-7.5622178268	0.2177145568	0.9999763851
TRMT6	1.1376079985	0.4947552548	0.9999763851
MCM8	1.1989709456	0.3519837345	0.9999763851
CRLS1	1.0986725977	0.547173851	0.9999763851
FERMT1	1.4099229642	0.1654068036	0.9999763851
TMX4	1.215319382	0.2769394075	0.9999763851
PLCB1	-1.1596074999	0.509645488	0.9999763851
PLCB4	0	0	0
LAMP5	-1.5257315481	0.1164897177	0.9999763851
ANKEF1	5.4781708352	0.3745527127	0.9999763851
SNAP25	0	0	0
MKKS	1.1881064672	0.2908156771	0.9999763851
SLX4IP	1.4784574538	0.1633205261	0.9999763851
JAG1	-1.0879031548	0.8007426145	0.9999763851
BTBD3	-4.2849908816	0.4028453076	0.9999763851
SPTLC3	5.4770877341	0.3746135179	0.9999763851
TASP1	1.4883386233	0.334512757	0.9999763851
ISM1	0	0	0
ESF1	1.0302504836	0.9018399481	0.9999763851
NDUFAF5	-1.3090423502	0.5914447829	0.9999763851

SEL1L2	3.2356557963	0.5547505991	0.9999763851
MACROD2	1.0954104339	0.8960124723	0.9999763851
KIF16B	1.2157962998	0.6812111224	0.9999763851
SNRPB2	-1.0708924923	0.5995085938	0.9999763851
PCSK2	-7.5622178268	0.2177145568	0.9999763851
BFSP1	-3.1523782906	0.1933609522	0.9999763851
DSTN	-1.0355869681	0.8023879703	0.9999763851
RRBP1	1.0358863196	0.9021231549	0.9999763851
SNX5	1.0304607309	0.856974194	0.9999763851
MGME1	1.0534674235	0.8236855672	0.9999763851
OVOL2	0	0	0
PET117	-1.1556427038	0.7845857376	0.9999763851
KAT14	-1.0208707959	0.9466423894	0.9999763851
ZNF133	1.4463480413	0.4529675933	0.9999763851
DZANK1	-7.5622178268	0.2177145568	0.9999763851
POLR3F	1.2789709507	0.5242367122	0.9999763851
RBBP9	1.0620746528	0.8961151778	0.9999763851
SEC23B	1.5661772776	0.0173458118	0.9999763851
DTD1	-1.4298969304	0.0456113532	0.9999763851
C20orf78	0	0	0
SLC24A3	1.5335035479	0.3134676029	0.9999763851
RIN2	1.0316990044	0.9492598485	0.9999763851
NAA20	1.0460663147	0.7787468358	0.9999763851
CRNKL1	1.1675028992	0.5870344399	0.9999763851
CFAP61	-1.0808570019	0.9213873901	0.9999763851
INSM1	3.3950799507	0.2315706881	0.9999763851
RALGAPA2	1.3021022452	0.1568302323	0.9999763851
KIZ	-1.0042382655	0.9852303231	0.9999763851
XRN2	1.3171858897	0.1821054418	0.9999763851
THBD	1.1118569034	0.8274124065	0.9999763851
CD93	1.0389040775	0.7576888968	0.9999763851
NXT1	1.0853299105	0.5918975948	0.9999763851
GZF1	1.6360339425	0.1163372759	0.9999763851
NAPB	1.4995000169	0.3604758121	0.9999763851
CST3	-1.0653081877	0.7308630639	0.9999763851
GGTLC1	-2.3214980365	0.4375379017	0.9999763851
SYNDIG1	-4.2864494036	0.4027150576	0.9999763851
CST7	1.0556368149	0.6636173989	0.9999763851
APMAP	1.0235598933	0.8596327074	0.9999763851
ACSS1	-1.2777479351	0.0583911194	0.9999763851
ENTPD6	-1.430063291	0.1322543628	0.9999763851
PYGB	1.6164170941	0.2317298459	0.9999763851
ABHD12	1.1940108392	0.2609348743	0.9999763851
GINS1	1.230499516	0.1490164818	0.9999763851
NINL	-1.3157515262	0.8229277089	0.9999763851
NANP	-1.0039427715	0.9854289261	0.9999763851
ZNF337	-1.1006548278	0.6840985452	0.9999763851
HM13	1.0725757393	0.6287624317	0.9999763851
LOC107985416	3.3940373312	0.2264967686	0.9999763851

ID1	-1.988561407	0.1904042371	0.9999763851
COX4I2	3.2353357581	0.554787377	0.9999763851
BCL2L1	1.1368774686	0.6475552384	0.9999763851
TPX2	-1.0006839168	0.9963313624	0.9999763851
MYLK2	-1.3943388833	0.6971518111	0.9999763851
DUSP15	0	0	0
TTLL9	14.1936656463	0.168558271	0.9999763851
PDRG1	1.1385815663	0.4555346055	0.9999763851
XKR7	0	0	0
CCM2L	-3.3294360969	0.2408463107	0.9999763851
HCK	-1.3568889329	0.069900331	0.9999763851
TM9SF4	-1.4499037543	0.1203715588	0.9999763851
PLAGL2	1.6385235135	0.0567128116	0.9999763851
POFUT1	1.0228900071	0.8997446512	0.9999763851
KIF3B	1.5876959961	0.0712535843	0.9999763851
ASXL1	-1.1499791126	0.4038358742	0.9999763851
NOL4L	1.2180183377	0.3894944596	0.9999763851
COMMD7	1.2889595363	0.0799404458	0.9999763851
DNMT3B	-1.1855578399	0.6515370995	0.9999763851
MAPRE1	1.033997266	0.839878763	0.9999763851
BPIFB4	0	0	0
CDK5RAP1	1.1376729952	0.5058799588	0.9999763851
SNTA1	-1.1947172969	0.5155327608	0.9999763851
CBFA2T2	-1.471044569	0.1780424858	0.9999763851
NECAB3	1.1355685265	0.829331427	0.9999763851
ACTL10	3.3962720959	0.248861434	0.9999763851
E2F1	-1.3220817815	0.2718403798	0.9999763851
PXMP4	1.4097521379	0.3608655966	0.9999763851
ZNF341	1.0790800989	0.6090245382	0.9999763851
CHMP4B	1.0950306294	0.5385479998	0.9999763851
RALY	1.0523149088	0.7908923464	0.9999763851
EIF2S2	1.0307326447	0.7974595655	0.9999763851
ASIP	-1.0384617903	0.8842878605	0.9999763851
AHCY	-1.2108400369	0.2246609137	0.9999763851
ITCH	-1.9328035682	0.0850308536	0.9999763851
LOC107985381	1.0002576586	0.9997940293	0.9999763851
DYNLRB1	1.3718734691	0.0927620699	0.9999763851
MAP1LC3A	1.8685954806	0.5481434699	0.9999763851
PIGU	-1.5343845509	0.0934089445	0.9999763851
TP53INP2	-1.4413164169	0.3310309941	0.9999763851
NCOA6	1.4323329492	0.1143867707	0.9999763851
ACSS2	-1.1707536276	0.677090725	0.9999763851
GSS	1.0232916298	0.9267478192	0.9999763851
TRPC4AP	-1.1308297127	0.5292549112	0.9999763851
EDEM2	-1.4826249465	0.1669351594	0.9999763851
PROCR	-1.76987712	0.0653950088	0.9999763851
MMP24	-1.0533860414	0.8847040156	0.9999763851
EIF6	1.1256319169	0.4325214836	0.9999763851
UQCC1	1.0355552074	0.8702827928	0.9999763851

GDF5	3.2356557963	0.5547505991	0.9999763851
CEP250	-1.0857485418	0.7060900175	0.9999763851
ERGIC3	-1.0174397783	0.9242972359	0.9999763851
SPAG4	-2.4272000607	0.2274781555	0.9999763851
CPNE1	-1.0074795398	0.9598025715	0.9999763851
RBM12	-1.2587402587	0.4678005783	0.9999763851
NFS1	-1.0479299084	0.8208008865	0.9999763851
ROMO1	-1.040362966	0.7599323551	0.9999763851
RBM39	-1.0880061346	0.5475084317	0.9999763851
PHF20	1.0683529549	0.6829303654	0.9999763851
SCAND1	-1.0132667933	0.9591188074	0.9999763851
CNBD2	-2.3214980365	0.4375930761	0.9999763851
EPB41L1	1.0317869295	0.9593518629	0.9999763851
AAR2	1.2412696033	0.2937144985	0.9999763851
DLGAP4	-1.0805706915	0.7851856618	0.9999763851
TGIF2	-1.092567478	0.6706215333	0.9999763851
C20orf24	1.4484483818	0.003656	0.9999763851
SLA2	-1.1160928733	0.7724379585	0.9999763851
NDRG3	-1.1173695294	0.509890285	0.9999763851
DSN1	-1.133609719	0.4360390472	0.9999763851
SOGA1	1.0298836313	0.9358147356	0.9999763851
TLDC2	-1.2789550668	0.6662995471	0.9999763851
SAMHD1	-1.3154339304	0.8205576571	0.9999763851
RBL1	-1.0745700941	0.7314424875	0.9999763851
MROH8	-2.7748204488	0.1723035535	0.9999763851
RPN2	1.2028409537	0.1765718693	0.9999763851
MANBAL	1.4056374044	0.4824275485	0.9999763851
BLCAP	1.0985607688	0.6343437099	0.9999763851
CTNNBL1	1.2661914927	0.2754819065	0.9999763851
VSTM2L	-4.2849908816	0.4028453076	0.9999763851
TTI1	1.060209841	0.8667985886	0.9999763851
RPRD1B	1.0789847674	0.7734304457	0.9999763851
TGM2	-4.2864494036	0.4027150576	0.9999763851
KIAA1755	0	0	0
BPI	1.0313773182	0.9285585262	0.9999763851
LBP	0	0	0
RALGAPB	-1.1443901739	0.6146783214	0.9999763851
ACTR5	-1.4088516573	0.3861805437	0.9999763851
PPP1R16B	-1.0159689854	0.97668512	0.9999763851
FAM83D	1.3074494948	0.1557222086	0.9999763851
DHX35	-1.0290561094	0.9402925561	0.9999763851
MAFB	-2.6885892031	0.0346890225	0.9999763851
TOP1	1.2205880993	0.2507078017	0.9999763851
PLCG1	1.2198907174	0.4967548468	0.9999763851
ZHX3	-1.3476313236	0.2225587741	0.9999763851
CHD6	-1.055131914	0.8462298464	0.9999763851
SRSF6	1.1735274425	0.2321975923	0.9999763851
L3MBTL1	-1.075487785	0.8806084986	0.9999763851
SGK2	-4.2864494036	0.4027150576	0.9999763851

IFT52	-1.2178827002	0.506527468	0.9999763851
MYBL2	1.1493453876	0.4929544872	0.9999763851
TOX2	1.9664421512	0.3428506238	0.9999763851
JPH2	-1.9650476918	0.4868473848	0.9999763851
OSER1	-1.0215415652	0.9461052224	0.9999763851
GDAP1L1	-8.342239448	0.2145009236	0.9999763851
FITM2	-1.9661045084	0.4536538719	0.9999763851
HNF4A	0	0	0
TTPAL	1.057088054	0.8858669249	0.9999763851
SERINC3	-1.0375895714	0.8577233098	0.9999763851
PKIG	-2.2015243791	0.1346705763	0.9999763851
ADA	-1.1577290956	0.3343739689	0.9999763851
WISP2	0	0	0
YWHAB	1.2971550882	0.0377864355	0.9999763851
PABPC1L	5.4776292235	0.3745831165	0.9999763851
TOMM34	-1.0634129928	0.7080758565	0.9999763851
STK4	1.10847478	0.5256344995	0.9999763851
SLPI	-1.3808899025	0.2173360256	0.9999763851
MATN4	-1.9652419871	0.4557873831	0.9999763851
SDC4	-1.0825129847	0.7110844884	0.9999763851
SYS1	-1.0190651418	0.939760473	0.9999763851
TP53TG5	-1.3157515262	0.8230246091	0.9999763851
DBNDD2	3.3951164687	0.250814793	0.9999763851
PIGT	-1.0005435824	0.9973709744	0.9999763851
WFDC8	0	0	0
WFDC11	0	0	0
WFDC3	0	0	0
DNTTIP1	1.0423402332	0.839044261	0.9999763851
UBE2C	-1.8913295165	0.223853939	0.9999763851
SNX21	1.0352194987	0.9042103676	0.9999763851
ACOT8	1.3648471011	0.5034311008	0.9999763851
ZSWIM3	-1.0417840134	0.9051070896	0.9999763851
ZSWIM1	-1.278603095	0.3089307697	0.9999763851
NEURL2	0	0	0
CTSA	1.1501739715	0.4237781181	0.9999763851
PLTP	-2.5222693939	0.1001615339	0.9999763851
LOC107985388	-7.5622178268	0.2177145568	0.9999763851
PCIF1	1.3220696995	0.3781846522	0.9999763851
ZNF335	-1.2580875227	0.4449435508	0.9999763851
MMP9	-7.515816106	0	0
SLC12A5	1.2196928187	0.5868763933	0.9999763851
NCOA5	-1.0255741017	0.9171644381	0.9999763851
CD40	0	0	0
CDH22	3.2356557963	0.5547505991	0.9999763851
SLC35C2	1.2188030094	0.2366815023	0.9999763851
ELMO2	-1.7836060192	0.1060817513	0.9999763851
OCSTAMP	-3.5359384325	0.1861728921	0.9999763851
SLC13A3	4.607561061	0.0514509885	0.9999763851
TP53RK	1.293202823	0.2499456712	0.9999763851

EYA2	-1.8170402834	0.4617275345	0.9999763851
ZMYND8	-1.1741161294	0.4148906947	0.9999763851
NCOA3	-1.2778458906	0.5560304168	0.9999763851
SULF2	1.9461790648	0.422502182	0.9999763851
PREX1	-1.6706232709	0.003913	0.9999763851
ARFGEF2	-1.1228736987	0.7300216397	0.9999763851
CSE1L	1.2266119642	0.1600706383	0.9999763851
STAU1	1.0842553943	0.5715264743	0.9999763851
DDX27	1.245343101	0.1353144034	0.9999763851
ZNFX1	-1.3063804818	0.2170648607	0.9999763851
KCNB1	0	0	0
B4GALT5	1.334312072	0.0704176141	0.9999763851
SLC9A8	1.0541480648	0.8431050993	0.9999763851
SPATA2	1.4154915891	0.3707018573	0.9999763851
RNF114	-1.0554330156	0.7419161538	0.9999763851
SNAI1	1.257031282	0.5160393245	0.9999763851
UBE2V1	-1.1475927864	0.4355118933	0.9999763851
TMEM189-UBE2V1	-1.0545930339	0.8480191659	0.9999763851
TMEM189	-1.3070874458	0.6188949756	0.9999763851
CEBPB	-1.2340630687	0.2871778085	0.9999763851
LINC01272	-4.3357248284	0.1410436389	0.9999763851
PTPN1	-1.2455608123	0.1526214449	0.9999763851
FAM65C	7.7204093521	0.2872553038	0.9999763851
PARD6B	1.7588469196	0.4594883115	0.9999763851
BCAS4	-1.3158816768	0.820532939	0.9999763851
ADNP	-1.0051021492	0.9747203598	0.9999763851
DPM1	-1.1551731281	0.4579438118	0.9999763851
MOCS3	1.3533910037	0.6436402884	0.9999763851
NFATC2	-1.0836376607	0.646534018	0.9999763851
ATP9A	-1.4872724642	0.1542745197	0.9999763851
SALL4	0	0	0
ZFP64	1.1805583337	0.5591178466	0.9999763851
TSHZ2	-1.3158816768	0.820532939	0.9999763851
ZNF217	-1.1867922133	0.529066932	0.9999763851
CYP24A1	0	0	0
PFDN4	-1.39304807	0.1176612983	0.9999763851
FAM210B	1.5224020367	0.1074926087	0.9999763851
AURKA	-1.0186839686	0.9171622059	0.9999763851
CSTF1	1.0843707723	0.7389716391	0.9999763851
CASS4	-1.0540706169	0.9535654663	0.9999763851
RTFDC1	1.5002365221	0.5923684537	0.9999763851
GCNT7	-1.1237631643	0.4685298653	0.9999763851
FAM209A	-10.8422096127	0.1418941141	0.9999763851
FAM209B	0	0	0
BMP7	-3.7194282732	0.1374523325	0.9999763851
RAE1	-1.1773640812	0.2952765814	0.9999763851
RBM38	-1.0264348637	0.8322773352	0.9999763851
CTCFL	0	0	0
PMEPA1	1.6188017753	0.3109079449	0.9999763851

RAB22A	-1.1878104357	0.4637705007	0.9999763851
VAPB	-1.2302677144	0.2633824892	0.9999763851
STX16	-1.329955166	0.180029792	0.9999763851
NPEPL1	1.2558075435	0.2708324772	0.9999763851
GNAS	-1.0636438486	0.7488884005	0.9999763851
NELFCD	1.3329703122	0.110678819	0.9999763851
CTSZ	1.031779777	0.9463482361	0.9999763851
TUBB1	-3.1542453588	0.1721503405	0.9999763851
ATP5E	1.2773622546	0.045895283	0.9999763851
PRELID3B	1.3171909144	0.2649934396	0.9999763851
ZNF831	3.2361532718	0.5546934395	0.9999763851
PHACTR3	1.0867698084	0.6815501374	0.9999763851
SYCP2	1.360595239	0.5391119927	0.9999763851
FAM217B	1.2206321529	0.2351384296	0.9999763851
PPP1R3D	1.9261319652	0.199694235	0.9999763851
CDH26	1.2872739558	0.8230836847	0.9999763851
C20orf197	-1.4072456792	0.2330339439	0.9999763851
CDH4	3.2361532718	0.5546934395	0.9999763851
TAF4	1.0325951904	0.9229895065	0.9999763851
LSM14B	-1.0123107948	0.955580793	0.9999763851
PSMA7	1.0557480938	0.670826388	0.9999763851
SS18L1	1.132487444	0.7751963279	0.9999763851
MTG2	1.0328236685	0.9126370522	0.9999763851
OSBPL2	-1.0590068471	0.8483254192	0.9999763851
ADRM1	1.2409078837	0.1896510143	0.9999763851
LAMA5	-2.5616934468	0.2771584414	0.9999763851
RPS21	1.0889440024	0.4936244383	0.9999763851
CABLES2	1.9041578618	0.0390924499	0.9999763851
SLCO4A1	1.1142963709	0.5537064495	0.9999763851
NTSR1	-1.3158816768	0.820532939	0.9999763851
LOC107987285	-1.7345857276	0.4906929473	0.9999763851
LOC107985428	-1.3715126673	0.7505409261	0.9999763851
MRGBP	-1.1011310344	0.6301788725	0.9999763851
OGFR	-1.0457825007	0.8483560572	0.9999763851
COL9A3	-1.4098410659	0.2179733166	0.9999763851
TCFL5	1.025678665	0.874569862	0.9999763851
DPH3P1	-4.287394647	0.4026306792	0.9999763851
DIDO1	-1.2541308198	0.3202283377	0.9999763851
GID8	-1.331103818	0.0290034687	0.9999763851
SLC17A9	1.1599235797	0.3439333349	0.9999763851
YTHDF1	1.1601027661	0.3833997709	0.9999763851
BIRC7	1.4010222013	0.0786425879	0.9999763851
NKAIN4	1.0311943882	0.9508738918	0.9999763851
ARFGAP1	1.543529674	0.0749499097	0.9999763851
KCNQ2	-4.2849908816	0.4028453076	0.9999763851
PPDPF	-1.1038034742	0.5793328199	0.9999763851
PTK6	-1.2296063424	0.7622194614	0.9999763851
C20orf195	-4.2849908816	0.4028453076	0.9999763851
HELZ2	-1.1399034669	0.648278145	0.9999763851

GMEB2	-1.2141623974	0.3192112308	0.9999763851
STMN3	1.4069142603	0.0447093106	0.9999763851
RTEL1	-8.3586475111	0.0312400138	0.9999763851
TNFRSF6B	1.3645396519	0.5215317269	0.9999763851
ARFRP1	1.1579167623	0.4989001583	0.9999763851
ZGPAT	-1.9647456674	0.4666595891	0.9999763851
LIME1	1.397925865	0.2336542961	0.9999763851
SLC2A4RG	-1.165811203	0.3093951913	0.9999763851
ZBTB46	-2.2867821077	0.1388049159	0.9999763851
ABHD16B	-1.4069959864	0.6661843062	0.9999763851
TPD52L2	1.0238634633	0.8902381226	0.9999763851
DNAJC5	-1.1533740954	0.3604125784	0.9999763851
UCKL1	-1.1288593904	0.7017024739	0.9999763851
ZNF512B	3.2356557963	0.5547505991	0.9999763851
SAMD10	1.1283065639	0.706449465	0.9999763851
PRPF6	-1.0108270299	0.9487806427	0.9999763851
TCEA2	-2.5759987075	0.0346611161	0.9999763851
RGS19	1.180907932	0.3365904544	0.9999763851
OPRL1	-1.1959917806	0.5228072037	0.9999763851
MYT1	1.0582319078	0.9023674713	0.9999763851
PCMTD2	-1.8459111238	0.0392992975	0.9999763851
LOC102723996	-1.1220082471	0.8898761875	0.9999763851
LOC102724023	-1.0324117823	0.8431342162	0.9999763851
LOC102724159	1.3774830511	0.1757310753	0.9999763851
LOC102724200	1.1516136901	0.5703676768	0.9999763851
LOC102724219	1.6246521587	0.5734298714	0.9999763851
LOC102724334	-14.1139127893	0.1038075351	0.9999763851
CH507-42P11.6	3.2361532718	0.5546934395	0.9999763851
LOC102724428	-4.3342515471	0.1408338982	0.9999763851
LOC102724843	-1.1961689377	0.3566200864	0.9999763851
LOC107987290	1.8134025452	0.5842204249	0.9999763851
U2AF1L5	-2.1539887829	0.0523423104	0.9999763851
LOC102724951	1.6254723588	0.5801222467	0.9999763851
LOC102723360	3.7829747665	0.1145712259	0.9999763851
SMIM11B	-1.5334266392	0.3112532932	0.9999763851
KCNE1B	-13.1048806834	0.1324873564	0.9999763851
LOC101927345	0	0	0
LIPI	2.341581609	0.4128010673	0.9999763851
HSPA13	-1.0055793843	0.9787250768	0.9999763851
SAMSN1	-1.11035574	0.6065388383	0.9999763851
NRIP1	-4.2849908816	0.4028453076	0.9999763851
USP25	1.1825933735	0.6286268408	0.9999763851
C21orf91	2.1611354447	0.004407	0.9999763851
CHODL	1.3931546432	0.7809653897	0.9999763851
MRPL39	-1.1570162597	0.6622522899	0.9999763851
ATP5J	1.2754837142	0.0488674881	0.9999763851
GABPA	-1.4815908761	0.1655130883	0.9999763851
APP	-1.0476791637	0.7848585187	0.9999763851
CYYR1	0	0	0

N6AMT1	1.7588469196	0.4594325773	0.9999763851
LTN1	-1.1537916894	0.5728466639	0.9999763851
RWDD2B	-1.3258492561	0.4692371946	0.9999763851
USP16	-1.1805467293	0.3753239492	0.9999763851
CCT8	1.0284058661	0.8226240061	0.9999763851
MAP3K7CL	0	0	0
BACH1	1.3224351089	0.3884551295	0.9999763851
GRIK1	1.6245986645	0.5892525377	0.9999763851
TIAM1	1.2869488883	0.8216243495	0.9999763851
SOD1	1.1536618555	0.2328843825	0.9999763851
SCAF4	-1.0154604628	0.9618775074	0.9999763851
MIS18A	-1.233642533	0.199604931	0.9999763851
MRAP	1.1698689513	0.5257570165	0.9999763851
URB1	1.2581017049	0.6547290519	0.9999763851
EVA1C	-1.3717838846	0.7496266785	0.9999763851
TCP10L	-1.1967195072	0.8052198585	0.9999763851
C21orf59	1.0731868423	0.7052539411	0.9999763851
SYNJ1	-1.5173134124	0.2966770655	0.9999763851
PAXBP1	1.03195848	0.9039927485	0.9999763851
C21orf62	11.9390382099	0.2001680618	0.9999763851
OLIG1	-1.1251191584	0.4633262155	0.9999763851
IFNAR2	-1.2566195273	0.2496207451	0.9999763851
IL10RB	1.0942674598	0.549184108	0.9999763851
IFNAR1	1.2306496411	0.2336590993	0.9999763851
IFNGR2	-1.2665026552	0.1555169615	0.9999763851
TMEM50B	1.1623646707	0.4800180909	0.9999763851
GART	1.0782239703	0.6433105957	0.9999763851
SON	1.151474296	0.2815044595	0.9999763851
DONSON	1.242926043	0.3648895161	0.9999763851
CRYZL1	1.2249261486	0.4960839847	0.9999763851
ITSN1	-1.3896472144	0.1504948208	0.9999763851
ATP5O	1.0662572636	0.6028727549	0.9999763851
MRPS6	-1.0276074985	0.8466813976	0.9999763851
KCNE2	1.8136032532	0.5922712384	0.9999763851
SMIM11A	-1.1093913073	0.6959843054	0.9999763851
LOC388820	0	0	0
KCNE1	-2.0111262383	0.2195294197	0.9999763851
RCAN1	-1.0847871666	0.6248114469	0.9999763851
RUNX1	-1.3516027245	0.0885977207	0.9999763851
SETD4	-1.2368303279	0.4872744417	0.9999763851
CBR1	1.2796825913	0.2423757922	0.9999763851
CBR3	0	0	0
DOPEY2	1.0159123018	0.929363298	0.9999763851
MORC3	-1.7958863753	0.0261246072	0.9999763851
CHAF1B	1.3867343779	0.1029179278	0.9999763851
CLDN14	0	0	0
SIM2	21.1691860784	0.1051441446	0.9999763851
HLCS	-1.0655137537	0.8232026858	0.9999763851
PIGP	1.1448191152	0.686778718	0.9999763851

TTC3	1.0165320361	0.9037221903	0.9999763851
DSCR3	-1.6417419452	0.0440783505	0.9999763851
DYRK1A	1.5954539035	0.007749	0.9999763851
LOC105372798	1.2872739558	0.8231055655	0.9999763851
KCNJ6	0	0	0
KCNJ15	-1.2791513211	0.6735153603	0.9999763851
ERG	1.0332411218	0.9299948202	0.9999763851
ETS2	-1.3343600057	0.0555350586	0.9999763851
PSMG1	1.0049782818	0.9759802125	0.9999763851
BRWD1	-1.0782803538	0.6671705655	0.9999763851
HMGN1	1.1658637217	0.2167850472	0.9999763851
WRB	1.3685450424	0.0993869812	0.9999763851
LCA5L	1.3412806227	0.6979357883	0.9999763851
SH3BGR	1.8138024348	0.5774527952	0.9999763851
IGSF5	3.2356557963	0.5547505991	0.9999763851
DSCAM	-1.0806434532	0.9277094092	0.9999763851
MX2	-1.2607263966	0.1452225198	0.9999763851
MX1	-3.3302773804	0.2305772477	0.9999763851
TMPRSS2	0	0	0
PRDM15	1.373208934	0.3894483419	0.9999763851
C2CD2	1.0954045989	0.6593166633	0.9999763851
ZBTB21	-1.1547200932	0.6393713624	0.9999763851
UMODL1	-2.3882538214	0.204206133	0.9999763851
ABCG1	-1.9040212742	0.18967379	0.9999763851
TFF3	1.4301853332	0.034562222	0.9999763851
TFF1	-4.2864494036	0.4027150576	0.9999763851
UBASH3A	3.2353357581	0.554787377	0.9999763851
SLC37A1	1.0849477094	0.741429701	0.9999763851
LOC101928212	1.1342454438	0.7257525569	0.9999763851
PDE9A	1.176424468	0.4157050996	0.9999763851
WDR4	-1.0207179885	0.8922757868	0.9999763851
NDUFV3	1.3309263306	0.1462744326	0.9999763851
ERVH48-1	0	0	0
PKNOX1	1.2114370586	0.3114319325	0.9999763851
CBS	1.6544532591	0.0876159115	0.9999763851
U2AF1	1.7695738058	0.018485891	0.9999763851
SIK1	-13.3922837871	0.00611	0.9999763851
HSF2BP	1.0965527729	0.8946753135	0.9999763851
RRP1B	-1.0015620478	0.991440235	0.9999763851
PDXK	1.0433335781	0.7759724306	0.9999763851
CSTB	-1.1198149861	0.3353180891	0.9999763851
RRP1	1.1219841073	0.5387692471	0.9999763851
AGPAT3	1.3217407808	0.05389175	0.9999763851
TRAPPC10	1.8249407711	0.0224357858	0.9999763851
PWP2	1.2061491632	0.3142083008	0.9999763851
ICOSLG	1.5837920266	0.0906188721	0.9999763851
DNMT3L	2.3404251821	0.4098945743	0.9999763851
PFKL	1.0109228692	0.9416829029	0.9999763851
C21orf2	-1.1105690639	0.6387702414	0.9999763851

TRPM2	1.0210379017	0.8990319483	0.9999763851
LRRC3	1.1404564402	0.821417007	0.9999763851
TSPEAR	-1.1235515614	0.852529858	0.9999763851
KRTAP10-11	0	0	0
UBE2G2	1.1878490596	0.2297451872	0.9999763851
SUMO3	-1.0202440932	0.8797889868	0.9999763851
PTTG1IP	1.2978916291	0.0914339077	0.9999763851
ITGB2	-1.0669645412	0.581032251	0.9999763851
FAM207A	1.0670824278	0.7304797413	0.9999763851
LOC105372836	14.4540940263	0.1606114182	0.9999763851
ADARB1	-1.1316611385	0.5062115624	0.9999763851
POFUT2	-1.5201577695	0.1302765052	0.9999763851
COL18A1	-2.2370783531	0.3352356858	0.9999763851
SLC19A1	1.1159079845	0.5151687785	0.9999763851
PCBP3	-1.3868448209	0.2512103113	0.9999763851
COL6A1	-1.3157515262	0.8232237083	0.9999763851
SPATC1L	1.4856971374	0.5241682844	0.9999763851
LSS	1.2691254053	0.2636540069	0.9999763851
MCM3AP	-1.0244878678	0.8839471721	0.9999763851
YBEY	-3.3198541437	0.0123775863	0.9999763851
C21orf58	1.4368933492	0.1991026849	0.9999763851
PCNT	1.019927783	0.934381039	0.9999763851
DIP2A	-1.0073484616	0.9700458138	0.9999763851
S100B	1.7616937791	0.2136840547	0.9999763851
PRMT2	-1.0281121354	0.8484078682	0.9999763851
POTEH	0	0	0
IL17RA	1.1621180655	0.3313259784	0.9999763851
CECR6	2.5928204807	0.1892694029	0.9999763851
CECR5	-1.2918033289	0.1186873312	0.9999763851
CECR1	1.1961053826	0.4022162446	0.9999763851
CECR2	0	0	0
SLC25A18	1.0280058166	0.9750429901	0.9999763851
LOC101929372	5.4770877341	0.3746135179	0.9999763851
ATP6V1E1	1.0336797504	0.8157284294	0.9999763851
BCL2L13	-1.0631368843	0.766618491	0.9999763851
BID	1.2813878356	0.1122085109	0.9999763851
MICAL3	-1.6226762749	0.1346566479	0.9999763851
PEX26	1.1100595557	0.5558816994	0.9999763851
TUBA8	-3.0655574101	0.2538022597	0.9999763851
USP18	-1.2775602755	0.2639765813	0.9999763851
LOC100996401	0	0	0
TMEM191B	-4.2849908816	0.4028453076	0.9999763851
DGCR6	1.1103525767	0.8045197664	0.9999763851
PRODH	-4.3331566429	0.1396076612	0.9999763851
DGCR2	-1.0066336582	0.9684138072	0.9999763851
DGCR14	-1.5166423721	0.2736089451	0.9999763851
SLC25A1	1.02951383	0.8832063722	0.9999763851
CLTCL1	1.225991271	0.2869385855	0.9999763851
HIRA	1.3386261381	0.4973719322	0.9999763851

MRPL40	1.4198884904	0.1388794262	0.9999763851
C22orf39	1.1715041281	0.3568604322	0.9999763851
UFD1L	1.242368087	0.2481952816	0.9999763851
CDC45	-1.1427825327	0.6101251141	0.9999763851
CLDN5	-1.0496579118	0.8781381517	0.9999763851
SEPT5	-1.3151016298	0.8211380418	0.9999763851
GP1BB	-2.3209115874	0.4585844086	0.9999763851
TBX1	1.6249017024	0.5574533205	0.9999763851
GNB1L	-1.2327202297	0.3708768376	0.9999763851
C22orf29	4.0460575546	0.0003538	0.4231276475
TXNRD2	1.5922095737	0.2057621794	0.9999763851
COMT	1.332032552	0.0806837995	0.9999763851
ARVCF	-1.4196636908	0.3002805286	0.9999763851
TANGO2	-1.0867034227	0.8417134414	0.9999763851
DGCR8	-1.1160460844	0.6977643515	0.9999763851
TRMT2A	-1.0333830348	0.9204827412	0.9999763851
RANBP1	1.2816208413	0.1333792794	0.9999763851
ZDHHC8	-1.0080160342	0.9755248585	0.9999763851
CCDC188	0	0	0
RTN4R	1.0849786238	0.7049234604	0.9999763851
DGCR6L	-1.0501057288	0.8557900637	0.9999763851
USP41	-1.5420290394	0.6710947271	0.9999763851
ZNF74	-1.3052610132	0.4390029348	0.9999763851
SCARF2	1.2872739558	0.8230839604	0.9999763851
KLHL22	-1.4238553434	0.2101465195	0.9999763851
MED15	1.4434210903	0.2860976023	0.9999763851
PI4KA	1.3751756206	0.033094635	0.9999763851
SNAP29	-1.1041047545	0.7483112989	0.9999763851
CRKL	-1.262028694	0.3076036479	0.9999763851
AIFM3	3.2353357581	0.554787377	0.9999763851
LZTR1	1.0012007891	0.9971231092	0.9999763851
THAP7	1.5803548206	0.0643356451	0.9999763851
P2RX6	0	0	0
RIMBP3B	1.3540269312	0.6268938733	0.9999763851
HIC2	-1.0180678453	0.9407902102	0.9999763851
TMEM191C	9.9654679573	0.2182826153	0.9999763851
RIMBP3C	0	0	0
UBE2L3	1.1512595308	0.3480360927	0.9999763851
YDJC	1.416167499	0.0807881266	0.9999763851
CCDC116	9.9629524019	0.2191087173	0.9999763851
SDF2L1	-1.0225419001	0.9026703595	0.9999763851
LOC107985532	1.6856724774	0.3227139203	0.9999763851
PPIL2	1.1728787543	0.5076821275	0.9999763851
YPEL1	-1.0109314135	0.9847491886	0.9999763851
MAPK1	1.0443695456	0.7992524031	0.9999763851
PPM1F	1.0850500344	0.5623520503	0.9999763851
TOP3B	1.0005367574	0.9991274089	0.9999763851
ZNF280B	-1.440961515	0.3787440936	0.9999763851
ZNF280A	3.2353357581	0.554787377	0.9999763851

PRAME	1.3575838719	0.4688216522	0.9999763851
GGTLC2	-1.176860274	0.6031774786	0.9999763851
GNAZ	1.5103258976	0.5452528327	0.9999763851
RAB36	3.3952287445	0.2427003854	0.9999763851
BCR	1.589439761	0.1268157977	0.9999763851
IGLL1	-4.3327792956	0.165617213	0.9999763851
DRICH1	1.1905633731	0.8015533761	0.9999763851
RGL4	1.28729267	0.1632989598	0.9999763851
ZNF70	3.2353357581	0.554787377	0.9999763851
VPREB3	3.2356557963	0.5547505991	0.9999763851
C22orf15	-4.2864494036	0.4027150576	0.9999763851
CHCHD10	1.0847786614	0.5634093838	0.9999763851
MMP11	0	0	0
SMARCB1	1.053464371	0.8092436238	0.9999763851
DERL3	-1.2354893189	0.6320368895	0.9999763851
SLC2A11	1.2681733251	0.5754041855	0.9999763851
MIF	1.224671875	0.1048315514	0.9999763851
DDTL	0	0	0
DDT	1.1024885098	0.4392928165	0.9999763851
CABIN1	1.0664386336	0.7300427552	0.9999763851
SUSD2	0	0	0
GGT5	1.6711160385	0.4628395196	0.9999763851
SPECC1L	1.0753361728	0.7953969655	0.9999763851
ADORA2A	1.2613917301	0.174480077	0.9999763851
UPB1	-1.228925712	0.5134987392	0.9999763851
GUCD1	1.0655988812	0.6986782554	0.9999763851
SNRPD3	-1.0620943846	0.6529606522	0.9999763851
GGT1	-1.1864535933	0.6577945118	0.9999763851
LRRC75B	-1.0821042168	0.9291660534	0.9999763851
SGSM1	-1.0379770968	0.9645627254	0.9999763851
TMEM211	0	0	0
KIAA1671	-4.2864494036	0.4027150576	0.9999763851
LOC100652901	-1.4853639298	0.6492324819	0.9999763851
LRP5L	-1.740998567	0.298355622	0.9999763851
GRK3	-1.0571630207	0.8103972169	0.9999763851
MYO18B	0	0	0
SEZ6L	-4.2864494036	0.4027150576	0.9999763851
ASPHD2	-1.5754456673	0.352421028	0.9999763851
HPS4	-1.2930344642	0.2051108142	0.9999763851
SRRD	1.210352153	0.3199767214	0.9999763851
TFIP11	-2.486429952	0.1376534043	0.9999763851
TPST2	1.0051892445	0.9705981581	0.9999763851
CRYBA4	0	0	0
LOC105372977	0	0	0
MN1	-3.3284109188	0.2432170002	0.9999763851
PITPNB	1.12431465	0.4023267585	0.9999763851
TTC28	-1.1961292548	0.7881574773	0.9999763851
CHEK2	-1.6071211529	0.007679	0.9999763851
HSCB	1.0568582013	0.9025496521	0.9999763851

CCDC117	1.1693011825	0.4312848402	0.9999763851
XBP1	1.0166377545	0.9056491539	0.9999763851
ZNRF3	-1.105669915	0.6432252193	0.9999763851
KREMEN1	1.1397793504	0.7462971379	0.9999763851
EMID1	-1.5712347939	0.2148007785	0.9999763851
RHBDD3	1.3720115748	0.3582141635	0.9999763851
EWSR1	1.2219120443	0.2709789689	0.9999763851
GAS2L1	-1.0661499473	0.8694831307	0.9999763851
AP1B1	1.063711204	0.6556796011	0.9999763851
RFPL1	0	0	0
THOC5	-1.4482233792	0.1757426109	0.9999763851
NIPSNAP1	-2.5598805278	0.0007501	0.6510516988
NF2	1.1968970453	0.3835758366	0.9999763851
CABP7	-1.0039253265	0.9900222704	0.9999763851
ZMAT5	-10.844600477	0.1421279131	0.9999763851
UQCR10	1.5087757119	0.0133115864	0.9999763851
ASCC2	-1.0530745232	0.8381953766	0.9999763851
MTMR3	-1.1571307401	0.6077219702	0.9999763851
HORMAD2	3.2356557963	0.5547505991	0.9999763851
OSM	1.6708777748	0.4564705348	0.9999763851
GATSL3	-1.5839258669	0.2683865757	0.9999763851
TBC1D10A	1.0573616162	0.8985497238	0.9999763851
SF3A1	1.0568313029	0.6884028828	0.9999763851
CCDC157	-2.5593885838	0.3089102914	0.9999763851
RNF215	0	0	0
SEC14L2	1.0775103844	0.9223202328	0.9999763851
MTFP1	-1.1719786845	0.325927273	0.9999763851
SEC14L3	-4.2864494036	0.4027150576	0.9999763851
SEC14L6	5.4770877341	0.3746135179	0.9999763851
PES1	1.6448674626	0.0678102288	0.9999763851
TCN2	-1.4075675212	0.657907102	0.9999763851
SLC35E4	1.3502901104	0.656953267	0.9999763851
DUSP18	1.0308701261	0.9416535156	0.9999763851
OSBP2	-1.6834103876	0.4266347168	0.9999763851
LOC107985544	-1.3983009058	0.6830199456	0.9999763851
MORC2	1.1593247404	0.6006386107	0.9999763851
SMTN	-2.5599229866	0.2927760335	0.9999763851
SELM	-1.6490543479	0.4721736735	0.9999763851
RNF185	1.3482172868	0.3687080386	0.9999763851
LIMK2	1.1627482163	0.4514885285	0.9999763851
PIK3IP1	-2.7791015202	0.003956	0.9999763851
PATZ1	-1.1060868578	0.7306235546	0.9999763851
DRG1	1.0339579293	0.8363106962	0.9999763851
EIF4ENIF1	-1.1055176445	0.6740892965	0.9999763851
SFI1	1.2337801177	0.5940542865	0.9999763851
PISD	1.1009230701	0.6629571519	0.9999763851
PRR14L	-1.527465681	0.1033867687	0.9999763851
DEPDC5	-1.1282581934	0.7204868691	0.9999763851
C22orf24	0	0	0

YWHAH	1.2712772621	0.1364472651	0.9999763851
C22orf42	0	0	0
RFPL2	1.3262752232	0.7419297927	0.9999763851
SLC5A4	-1.8933556971	0.1856677614	0.9999763851
RTCB	1.0735168783	0.607729719	0.9999763851
FBXO7	1.1260305787	0.4046334418	0.9999763851
SYN3	-3.5008381699	0.0885110757	0.9999763851
TIMP3	3.2353357581	0.554787377	0.9999763851
LARGE	-1.966276531	0.4563825937	0.9999763851
HMGXB4	-1.0711530789	0.7768493216	0.9999763851
TOM1	-1.1993015321	0.5528693809	0.9999763851
HMOX1	-1.5548498442	0.1631854599	0.9999763851
MCM5	1.1533589205	0.314600495	0.9999763851
RASD2	4.9068606068	0.1990447136	0.9999763851
MB	0	0	0
APOL6	-1.0216426756	0.9155711207	0.9999763851
APOL5	0	0	0
RBFOX2	1.2080915232	0.3004552883	0.9999763851
APOL2	-1.4294980372	0.5308157876	0.9999763851
APOL1	-4.2849908816	0.4028453076	0.9999763851
MYH9	1.0944786979	0.5287318245	0.9999763851
TXN2	-1.0867480293	0.6041605125	0.9999763851
FOXRED2	1.3151366651	0.1270580259	0.9999763851
EIF3D	-1.0398701271	0.7707063741	0.9999763851
IFT27	-1.1153189913	0.6928890073	0.9999763851
NCF4	1.2221616177	0.1286259626	0.9999763851
CSF2RB	-1.3346926667	0.3192625075	0.9999763851
TST	1.1559244369	0.3488014528	0.9999763851
MPST	1.0681536231	0.6546621002	0.9999763851
KCTD17	-1.0681864798	0.8802635065	0.9999763851
C1QTNF6	-2.3222877795	0.4514842962	0.9999763851
RAC2	1.0686070718	0.6119330422	0.9999763851
CYTH4	1.4682328874	0.1116309835	0.9999763851
ELFN2	-3.1531745435	0.2210901857	0.9999763851
MFNG	-1.0911124916	0.5330970695	0.9999763851
LGALS2	0	0	0
GGA1	-1.2378036562	0.2351874891	0.9999763851
SH3BP1	1.0426616343	0.8074303692	0.9999763851
PDXP	-1.0203738825	0.9252842105	0.9999763851
LGALS1	1.2208778044	0.1357541363	0.9999763851
NOL12	-1.0553736989	0.7832424084	0.9999763851
TRIOBP	-1.3910046339	0.1207861289	0.9999763851
LOC102724378	7.4933530102	0.2984419804	0.9999763851
H1F0	1.1334835159	0.6774454371	0.9999763851
GCAT	1.2286469138	0.3679031864	0.9999763851
GALR3	0	0	0
ANKRD54	1.1751885467	0.6107314678	0.9999763851
EIF3L	-1.031332557	0.8137159737	0.9999763851
MICALL1	1.9443929147	0.0995067197	0.9999763851

C22orf23	1.0313900409	0.9427220287	0.9999763851
POLR2F	-1.0279214349	0.8579823038	0.9999763851
PICK1	3.2356557963	0.5547505991	0.9999763851
BAIAP2L2	0	0	0
PLA2G6	-2.2395803318	0.3062314467	0.9999763851
MAFF	1.0621674542	0.8956921362	0.9999763851
TMEM184B	-1.3812835672	0.2222454904	0.9999763851
CSNK1E	-1.4804530749	0.0723734828	0.9999763851
LOC400927-CSNK1E	1.1889211165	0.6679673895	0.9999763851
KCNJ4	0	0	0
KDELR3	-1.3157515262	0.8233267857	0.9999763851
DDX17	-1.0432079039	0.7309838838	0.9999763851
DMC1	1.6800044611	0.1906206682	0.9999763851
FAM227A	7.4933530102	0.2983904924	0.9999763851
CBY1	-1.3020121131	0.5131236587	0.9999763851
TOMM22	-1.3264968198	0.0338067359	0.9999763851
JOSD1	1.2485112258	0.4074223837	0.9999763851
GTPBP1	1.4335777985	0.0377936253	0.9999763851
SUN2	-1.1155761924	0.4783362558	0.9999763851
DNAL4	-1.2910873964	0.3160716536	0.9999763851
NPTXR	-1.0601026445	0.6507103748	0.9999763851
CBX6	-1.0346953986	0.7905961016	0.9999763851
APOBEC3A	0	0	0
APOBEC3B	1.0212074832	0.9205277089	0.9999763851
APOBEC3C	1.1840645706	0.412945062	0.9999763851
APOBEC3F	-1.1056047606	0.8033358193	0.9999763851
APOBEC3D	-1.9503202885	0.2950377969	0.9999763851
APOBEC3G	-1.23791485	0.6360466842	0.9999763851
APOBEC3H	-1.3948129396	0.6828350831	0.9999763851
CBX7	-1.3628298901	0.2512550173	0.9999763851
PDGFB	0	0	0
RPL3	1.1172886736	0.3669239743	0.9999763851
SYNGR1	-1.0413103756	0.8411846811	0.9999763851
TAB1	1.0676863702	0.7945622493	0.9999763851
MGAT3	-1.0469262829	0.917570701	0.9999763851
MIEF1	-1.0086618248	0.9526795762	0.9999763851
ATF4	-1.0516629968	0.7232804805	0.9999763851
RPS19BP1	-1.0433803243	0.772921139	0.9999763851
CACNA1I	1.1896618529	0.8096233854	0.9999763851
ENTHD1	2.8686586245	0.3031425829	0.9999763851
GRAP2	1.2355372155	0.458658358	0.9999763851
FAM83F	2.5158674433	0.157762887	0.9999763851
TNRC6B	1.007383414	0.9703379696	0.9999763851
ADSL	1.4337817249	0.0178858648	0.9999763851
SGSM3	1.6805963267	0.1672597429	0.9999763851
MKL1	-1.0366126104	0.8675734786	0.9999763851
SLC25A17	-1.0950505939	0.7002368511	0.9999763851
ST13	-1.0165098397	0.9086444847	0.9999763851
XPNPEP3	1.4738212105	0.1235664607	0.9999763851

RBX1	1.0879696103	0.5543935494	0.9999763851
EP300	1.2458920932	0.4853699538	0.9999763851
L3MBTL2	1.1425429394	0.8468216772	0.9999763851
CHADL	1.675672972	0.1992527799	0.9999763851
RANGAP1	-1.0449684496	0.8165412017	0.9999763851
ZC3H7B	1.1074712133	0.5713951209	0.9999763851
TEF	1.6062903958	0.6462291901	0.9999763851
LOC100996598	0	0	0
TOB2	-1.0163866231	0.9379008137	0.9999763851
PHF5A	1.1192951819	0.5165027058	0.9999763851
ACO2	-1.1310533061	0.6637395659	0.9999763851
POLR3H	-1.059536567	0.6736770758	0.9999763851
CSDC2	7.4933530102	0.2983388468	0.9999763851
PMM1	-1.3180690451	0.2954619332	0.9999763851
DESI1	1.4704692409	0.2121482623	0.9999763851
XRCC6	1.1541600817	0.2874148707	0.9999763851
SNU13	1.2473872153	0.0909433768	0.9999763851
C22orf46	-2.4822462884	0.1569326898	0.9999763851
MEI1	1.8863723168	0.0959387322	0.9999763851
CCDC134	1.1679003387	0.6388780108	0.9999763851
SREBF2	-1.1988116812	0.2666975686	0.9999763851
SHISA8	-1.262094928	0.2295584231	0.9999763851
TNFRSF13C	-7.5647903763	0.2176246281	0.9999763851
CENPM	1.4431448518	0.0564245271	0.9999763851
SEPT3	1.5214888853	0.0564992374	0.9999763851
WBP2NL	-1.8155268442	0.4542227514	0.9999763851
NAGA	1.1385810305	0.6428075238	0.9999763851
SMDT1	1.2025671134	0.3763004298	0.9999763851
NDUFA6	1.017206874	0.9033584437	0.9999763851
CYP2D6	-1.3158816768	0.8205329391	0.9999763851
LOC102723722	-2.3230035206	0.462320266	0.9999763851
TCF20	1.3282486672	0.2837281499	0.9999763851
NFAM1	1.1672834816	0.5410858652	0.9999763851
RRP7A	1.0391021844	0.8501169727	0.9999763851
SERHL2	-1.1196180126	0.76905871	0.9999763851
POLDIP3	-1.132217337	0.452088566	0.9999763851
CYB5R3	1.1934246816	0.3088881027	0.9999763851
A4GALT	0	0	0
ARFGAP3	-1.1060582033	0.6818267113	0.9999763851
PACSIN2	1.1911388687	0.3600545686	0.9999763851
TTLL1	1.3494005179	0.6621559438	0.9999763851
BIK	-1.0881697047	0.8423426344	0.9999763851
MCAT	2.1009570993	0.0902992804	0.9999763851
TSPO	1.3767377756	0.0574725303	0.9999763851
TTLL12	1.5340128447	0.105066722	0.9999763851
EFCAB6	1.0881489642	0.8452895084	0.9999763851
SAMM50	1.2278546593	0.36851581	0.9999763851
PARVB	-1.3638422295	0.2555882281	0.9999763851
PARVG	1.1959420702	0.4492195362	0.9999763851

LDOC1L	1.2146725126	0.4874343753	0.9999763851
PRR5	-1.149117102	0.7163947097	0.9999763851
PRR5-ARHGAP8	-4.2864494036	0.4027150576	0.9999763851
ARHGAP8	-2.3214980365	0.4376484453	0.9999763851
NUP50	1.1313848445	0.5102161154	0.9999763851
KIAA0930	1.0095036664	0.9457634885	0.9999763851
FAM118A	-1.4358159967	0.4502072849	0.9999763851
SMC1B	-1.7406929207	0.1883844221	0.9999763851
RIBC2	1.6249017024	0.5574174975	0.9999763851
ATXN10	1.2931446075	0.1183895626	0.9999763851
WNT7B	0	0	0
PPARA	-1.1675397055	0.6042606352	0.9999763851
CDPF1	1.2503531424	0.6998770054	0.9999763851
PKDREJ	7.7488609396	0.3058740553	0.9999763851
TTC38	-1.2799544286	0.490546675	0.9999763851
GTSE1	-1.0564668116	0.7844519175	0.9999763851
TRMU	-1.5080411851	0.2716472836	0.9999763851
CELSR1	3.2361532718	0.5546934395	0.9999763851
GRAMD4	1.3972470118	0.2479953678	0.9999763851
CERK	1.0575377111	0.8384601251	0.9999763851
TBC1D22A	-1.3365054582	0.3857800219	0.9999763851
FAM19A5	0	0	0
C22orf34	-1.0536799076	0.9207877986	0.9999763851
BRD1	-3.1534488363	0.1726148086	0.9999763851
ZBED4	1.0947078904	0.872092506	0.9999763851
ALG12	-1.1250614384	0.6398282793	0.9999763851
CRELD2	1.0396072898	0.8145817295	0.9999763851
PIM3	-1.1492299585	0.3071829584	0.9999763851
MLC1	1.3259325514	0.0636234178	0.9999763851
MOV10L1	10.4285443305	0.2413352043	0.9999763851
PANX2	3.2356557963	0.5547505991	0.9999763851
TRABD	1.2171461068	0.193906849	0.9999763851
SELO	1.5114898576	0.1473539696	0.9999763851
TUBGCP6	-2.5225832327	0.0714787553	0.9999763851
HDAC10	-1.0634814851	0.8910740731	0.9999763851
MAPK12	1.2477316029	0.2910394858	0.9999763851
MAPK11	-1.1463904141	0.7409911536	0.9999763851
DENND6B	1.6136604673	0.3343111555	0.9999763851
PPP6R2	1.0187412987	0.9311301651	0.9999763851
SBF1	-1.1597014588	0.510354082	0.9999763851
ADM2	3.2356557963	0.5547505991	0.9999763851
LMF2	1.4950048559	0.215327416	0.9999763851
NCAPH2	-1.2852096485	0.3384142635	0.9999763851
SCO2	1.4389747352	0.0750882995	0.9999763851
TYMP	-2.8684846696	0.0818181769	0.9999763851
ODF3B	-2.5598350099	0.3143812462	0.9999763851
SYCE3	0	0	0
CPT1B	-1.2084536663	0.5627729793	0.9999763851
CHKB	2.3039938442	0.0989903022	0.9999763851

MAPK8IP2	-1.3711818216	0.7361521743	0.9999763851
ARSA	-1.0720105362	0.8577050025	0.9999763851
RABL2B	1.03619961	0.8724541221	0.9999763851
PLCXD1_1	1.1311294149	0.6518213332	0.9999763851
GTPBP6_1	-1.4867671956	0.160446421	0.9999763851
PPP2R3B_1	-1.0539454417	0.9183616523	0.9999763851
CRLF2_1	0	0	0
CSF2RA_1	1.1446563693	0.7083051758	0.9999763851
IL3RA_1	-2.097264704	0.2347984707	0.9999763851
SLC25A6_1	-1.0571640456	0.6811821107	0.9999763851
LOC105373102_1	0	0	0
ASMTL_1	1.6073771667	0.1314342295	0.9999763851
P2RY8_1	1.0095074247	0.9570299003	0.9999763851
AKAP17A_1	-1.0760820216	0.7888689209	0.9999763851
ASMT_1	-4.2849908816	0.4028453076	0.9999763851
DHRSX_1	1.134000037	0.7541445376	0.9999763851
ZBED1_1	1.822945724	0.0794997653	0.9999763851
CD99_1	1.042613259	0.8058234196	0.9999763851
ARSD	1.2455307469	0.3117246051	0.9999763851
ARSE	3.2356557963	0.5547505991	0.9999763851
ARSF	0	0	0
MXRA5	0	0	0
PRKX	1.0833955382	0.660523238	0.9999763851
NLGN4X	3.2353357581	0.554787377	0.9999763851
VCX3A	0	0	0
PUDP	1.128515276	0.5686320015	0.9999763851
STS	1.3155286451	0.5176698159	0.9999763851
VCX	-1.9660821036	0.4630669119	0.9999763851
PNPLA4	-4.2849908816	0.4028453076	0.9999763851
VCX3B	-10.8385246611	0.1452076922	0.9999763851
ANOS1	1.5505881706	0.5541323877	0.9999763851
FAM9B	-4.2849908816	0.4028453076	0.9999763851
TBL1X	1.3809138387	0.0717732399	0.9999763851
GPR143	-1.0842744172	0.8974869046	0.9999763851
SHROOM2	1.2859100748	0.2742592518	0.9999763851
CLDN34	-4.2849908816	0.4028453076	0.9999763851
WWC3	-1.0460646669	0.7897701338	0.9999763851
CLCN4	3.2353357581	0.554787377	0.9999763851
MID1	-1.4326576759	0.4756486663	0.9999763851
HCCS	-1.1607103098	0.4526398879	0.9999763851
MSL3	1.7045734286	0.0192832661	0.9999763851
FRMPD4	-10.8385246611	0.1452685011	0.9999763851
PRPS2	1.2494007127	0.1747641272	0.9999763851
TLR8	0	0	0
TMSB4X	-1.5056544639	0.002174	0.9999763851
FAM9C	0	0	0
LOC105373133	5.4776292235	0.3745831165	0.9999763851
TCEANC	-2.0689192087	0.1013965962	0.9999763851
RAB9A	-1.7162371334	0.1148952661	0.9999763851

TRAPPC2	1.119416785	0.5839056537	0.9999763851
OFD1	-1.3598335189	0.260084491	0.9999763851
GPM6B	-1.1489598494	0.8503578071	0.9999763851
GEMIN8	1.0066443544	0.9804165752	0.9999763851
GLRA2	-1.318394896	0.60246829	0.9999763851
FANCB	-1.0589551369	0.7932186849	0.9999763851
MOSPD2	-1.3837692955	0.2431220168	0.9999763851
PIGA	-1.1038372572	0.7356939141	0.9999763851
FIGF	15.5019390839	0.1629420389	0.9999763851
PIR	-1.1018674108	0.6496071707	0.9999763851
ACE2	3.2353357581	0.554787377	0.9999763851
BMX	-1.1157749871	0.668282009	0.9999763851
TMEM27	-1.0249053994	0.957183673	0.9999763851
CA5B	-1.1474192383	0.614553273	0.9999763851
ZRSR2	-1.2094954552	0.5406004034	0.9999763851
AP1S2	1.0213235579	0.8888782926	0.9999763851
CTPS2	-1.3389300992	0.3987070039	0.9999763851
S100G	3.2353357581	0.554787377	0.9999763851
SYAP1	-1.0320918397	0.8735467269	0.9999763851
TXLNG	-1.0385063033	0.8270516867	0.9999763851
RBBP7	1.0017946772	0.9890855247	0.9999763851
REPS2	-2.983726772	0.0354693453	0.9999763851
NHS	-1.3717838846	0.7496003097	0.9999763851
SCML1	-1.9790947981	0.0515532886	0.9999763851
BEND2	0	0	0
SCML2	1.0952111839	0.837065563	0.9999763851
CDKL5	-1.4348551497	0.4400003091	0.9999763851
RS1	0	0	0
PHKA2	-1.0034718473	0.9855471784	0.9999763851
ADGRG2	1.5436836647	0.5637612112	0.9999763851
PDHA1	-1.0848299514	0.5704497557	0.9999763851
MAP3K15	1.5292328987	0.2305004082	0.9999763851
SH3KBP1	1.2086968483	0.1581233116	0.9999763851
CXorf23	-1.5993594647	0.181695333	0.9999763851
MAP7D2	0	0	0
EIF1AX	1.094668985	0.4817197775	0.9999763851
RPS6KA3	-1.0990299475	0.6083335724	0.9999763851
MBTPS2	1.2890041542	0.2873325522	0.9999763851
YY2	-1.1984126895	0.7609390165	0.9999763851
SMS	-1.3570592004	0.0382322825	0.9999763851
PHEX	-1.6282722959	0.2526250928	0.9999763851
PRDX4	-1.075865927	0.7127671848	0.9999763851
ACOT9	1.1180930905	0.6374854616	0.9999763851
SAT1	-1.6624997722	0.0005321	0.5206160142
APOO	-1.0055907798	0.9643834886	0.9999763851
CXorf58	3.2353357581	0.554787377	0.9999763851
KLHL15	-1.4455952043	0.2681444881	0.9999763851
EIF2S3	1.1202569008	0.3657615292	0.9999763851
ZFX	-1.2867418298	0.1268163096	0.9999763851

PDK3	1.0573199452	0.7613061534	0.9999763851
PCYT1B	-7.5622178268	0.2177145568	0.9999763851
POLA1	-1.1017919972	0.4178851601	0.9999763851
ARX	3.9195442002	0.1876409732	0.9999763851
IL1RAPL1	-2.3860318298	0.2391398476	0.9999763851
CXorf21	-2.1609178192	0.1196034727	0.9999763851
GK	-1.1022660321	0.7220627014	0.9999763851
TAB3	-1.0251334554	0.9407856827	0.9999763851
DMD	1.7826260038	0.0484565649	0.9999763851
CFAP47	0	0	0
XK	-1.4464778568	0.2106237091	0.9999763851
CYBB	-4.4381516454	0.003279	0.9999763851
DYNLT3	1.2917773094	0.6056405669	0.9999763851
SYTL5	0	0	0
SRPX	-7.5647903763	0.2176246281	0.9999763851
RPGR	1.4729385521	0.3315413034	0.9999763851
MID1IP1	-1.0927733793	0.6278044557	0.9999763851
BCOR	-1.1986675416	0.274819153	0.9999763851
LOC107985687	3.2356557963	0.5547505991	0.9999763851
ATP6AP2	-1.0063039776	0.9600119427	0.9999763851
CXorf38	-1.285420026	0.3244092484	0.9999763851
MED14	1.2098211433	0.3297304702	0.9999763851
MED14OS	1.813344484	0.5819972383	0.9999763851
USP9X	1.1373338302	0.5275548472	0.9999763851
DDX3X	-1.0194013297	0.9092170139	0.9999763851
CASK	-1.5814483139	0.1178729032	0.9999763851
GPR34	3.9208783318	0.1835694973	0.9999763851
GPR82	-56.6417269775	0.0116180255	0.9999763851
MAOA	0	0	0
EFHC2	3.2356557963	0.5547505991	0.9999763851
FUNDC1	-1.0057623416	0.9855317908	0.9999763851
KDM6A	-1.3043012555	0.4121862023	0.9999763851
KRBOX4	-1.0100414917	0.9740026485	0.9999763851
ZNF674	-1.1898705397	0.7496636016	0.9999763851
CHST7	-2.1905898287	0.0537971722	0.9999763851
SLC9A7	-1.5313558062	0.3457396277	0.9999763851
RP2	1.1516625217	0.4851378572	0.9999763851
JADE3	-1.1668940991	0.61819947	0.9999763851
RGN	3.2356557963	0.5547505991	0.9999763851
NDUFB11	1.1666773004	0.2859619363	0.9999763851
RBM10	1.4681982282	0.016592973	0.9999763851
UBA1	1.0822906735	0.5820354218	0.9999763851
CDK16	1.0371381181	0.8819394442	0.9999763851
USP11	-1.9734077647	0.0150664542	0.9999763851
ZNF157	0	0	0
ZNF41	1.2710064579	0.3951267144	0.9999763851
ARAF	1.1873113369	0.3779878685	0.9999763851
SYN1	0	0	0
TIMP1	-2.9292922021	9.26E-11	4.98E-07

CFP	1.7327347029	0.1944854808	0.9999763851
ELK1	-1.1034538003	0.5317006375	0.9999763851
UXT	1.1365976374	0.4566229418	0.9999763851
ZNF81	1.265515385	0.5275979612	0.9999763851
ZNF182	1.1726118417	0.6762422962	0.9999763851
FTSJ1	-1.1228139951	0.53275104	0.9999763851
PORCN	1.1937721849	0.5072892414	0.9999763851
EBP	1.0532635744	0.6930512132	0.9999763851
TBC1D25	1.0801640314	0.772598819	0.9999763851
RBM3	-1.1456664847	0.3479583121	0.9999763851
WDR13	1.1894999221	0.4483517272	0.9999763851
WAS	1.0113940998	0.9417965002	0.9999763851
SUV39H1	1.1084857721	0.6563537554	0.9999763851
HDAC6	-1.4740248466	0.1216141712	0.9999763851
ERAS	0	0	0
TIMM17B	-1.0439968087	0.8364343658	0.9999763851
PQBP1	1.7308642843	0.0291387859	0.9999763851
SLC35A2	-1.5296575463	0.0160544618	0.9999763851
PIM2	-1.4242351556	0.0243578384	0.9999763851
OTUD5	-1.2019691455	0.3707584189	0.9999763851
KCND1	1.813344484	0.5819236562	0.9999763851
GRIPAP1	-1.0757115787	0.7129901085	0.9999763851
TFE3	-1.3229023523	0.2577149599	0.9999763851
PRAF2	-1.2498178925	0.5783042473	0.9999763851
WDR45	1.030183431	0.9306181922	0.9999763851
GPKOW	1.6752798993	0.0680289676	0.9999763851
MAGIX	3.2361532718	0.5546934395	0.9999763851
PLP2	1.0175007395	0.8966206179	0.9999763851
PRICKLE3	1.3493579391	0.6657471906	0.9999763851
SYP	-14.1187086092	0.0981884247	0.9999763851
CACNA1F	0	0	0
CCDC22	-1.2095485068	0.3528383372	0.9999763851
FOXP3	-1.1443525594	0.88651452	0.9999763851
PPP1R3F	1.2630745238	0.5564103824	0.9999763851
GAGE10	0	0	0
GAGE2A	3.2353357581	0.554787377	0.9999763851
GAGE1	-4.287394647	0.4026306792	0.9999763851
USP27X	1.3130800431	0.5193394846	0.9999763851
CLCN5	-1.270340952	0.4306348779	0.9999763851
MAGED1	-1.3652157337	0.1777369426	0.9999763851
XAGE1B	-1.1153322386	0.498682315	0.9999763851
XAGE1E	1.0331815147	0.8529676334	0.9999763851
FAM156B	1.1411003116	0.5302311957	0.9999763851
GPR173	0	0	0
TSPYL2	1.3046067945	0.4726343397	0.9999763851
KDM5C	1.0654156949	0.7868026413	0.9999763851
IQSEC2	1.2158504019	0.6836004413	0.9999763851
SMC1A	1.3480668306	0.0271862024	0.9999763851
RIBC1	3.2356557963	0.5547505991	0.9999763851

HSD17B10	1.1447236157	0.4257266926	0.9999763851
HUWE1	1.1568384718	0.3121167377	0.9999763851
PHF8	-1.3728106496	0.0921834502	0.9999763851
FAM120C	1.1062009175	0.7236563425	0.9999763851
WNK3	7.4933530102	0.2982870353	0.9999763851
TSR2	1.5511434682	0.0618220748	0.9999763851
FGD1	-1.7174926864	0.0232842252	0.9999763851
GNL3L	1.4559428502	0.0324699126	0.9999763851
MAGED2	-1.0984728665	0.6307774502	0.9999763851
PFKFB1	-4.2849908816	0.4028453076	0.9999763851
APEX2	-1.4637539616	0.1202832486	0.9999763851
PAGE2B	-4.2849908816	0.4028453076	0.9999763851
PAGE2	0	0	0
FAM104B	1.1792692551	0.4812955265	0.9999763851
MTRNR2L10	-1.4397976359	0.4306287756	0.9999763851
MAGEH1	-2.2671808911	0.0902515536	0.9999763851
USP51	-1.8168761427	0.3007818583	0.9999763851
RRAGB	-1.5918761572	0.231940438	0.9999763851
UBQLN2	1.2341421734	0.3128576808	0.9999763851
SPIN2B	-1.1129358595	0.7040360096	0.9999763851
FAAH2	1.3490603578	0.6657647894	0.9999763851
ZXDB	-1.1689839273	0.6495162263	0.9999763851
ZXDA	2.2216198074	0.3240427491	0.9999763851
SPIN4	-1.2438403447	0.1681593589	0.9999763851
ARHGEF9	1.4725467939	0.3296874274	0.9999763851
AMER1	1.2790370317	0.4608373594	0.9999763851
MTMR8	0	0	0
ZC3H12B	1.7458374716	0.3348613795	0.9999763851
LAS1L	1.0196799831	0.9067297654	0.9999763851
MSN	-1.2843222569	0.0664172284	0.9999763851
VSIG4	-20.6875548266	0.0567446204	0.9999763851
YIPF6	-1.0715462119	0.7232305364	0.9999763851
STARD8	1.3436913572	0.3301168171	0.9999763851
EFNB1	-1.075653075	0.831742049	0.9999763851
PJA1	1.1419432367	0.7760394597	0.9999763851
EDA	-2.0621569129	0.3088988963	0.9999763851
IGBP1	-1.0680588629	0.6835952361	0.9999763851
DGAT2L6	0	0	0
ARR3	1.491541136	0.6993320602	0.9999763851
RAB41	0	0	0
PDZD11	-1.1721434719	0.555027666	0.9999763851
KIF4A	1.1765063059	0.282560093	0.9999763851
TEX11	3.2356557963	0.5547505991	0.9999763851
SNX12	1.0167372062	0.9163847774	0.9999763851
FOXO4	-1.266255247	0.4512138479	0.9999763851
CXorf65	1.3267411246	0.7616152189	0.9999763851
IL2RG	-1.1559770095	0.3870204995	0.9999763851
MED12	1.06119807	0.8616004195	0.9999763851
NLGN3	-1.0534934212	0.9209994765	0.9999763851

ZMYM3	-1.083841854	0.6298970506	0.9999763851
NONO	1.1247539228	0.3372949029	0.9999763851
ITGB1BP2	2.1857290676	0.3706467231	0.9999763851
TAF1	-1.2744307092	0.1151573619	0.9999763851
OGT	-1.4144525282	0.0355723334	0.9999763851
ACRC	1.0947156806	0.8968067763	0.9999763851
CXCR3	1.464733964	0.3322664835	0.9999763851
LOC101059915	0	0	0
NHSL2	1.0903512237	0.794042671	0.9999763851
RGAG4	-8.342239448	0.2143036092	0.9999763851
FLJ44635	1.0871322184	0.6078940919	0.9999763851
PIN4	1.2117683575	0.2423115497	0.9999763851
ERCC6L	1.2603768829	0.3627645311	0.9999763851
RPS4X	1.0537926544	0.6794475115	0.9999763851
CITED1	2.5183171819	0.2738196453	0.9999763851
HDAC8	-1.1476311659	0.4822478769	0.9999763851
SLC16A2	0	0	0
RLIM	1.0939646876	0.5616057234	0.9999763851
ABCB7	-1.2146369422	0.325065146	0.9999763851
UPRT	1.1042699293	0.6957662062	0.9999763851
ZDHHC15	-1.3714374731	0.7327118287	0.9999763851
PBDC1	1.0766307602	0.6011759312	0.9999763851
MAGEE1	-1.3154339304	0.8205576571	0.9999763851
ATRX	1.00824822	0.9539055808	0.9999763851
MAGT1	1.3640202715	0.0563202957	0.9999763851
COX7B	1.1326327774	0.3287217994	0.9999763851
ATP7A	-1.7917981288	0.2394004801	0.9999763851
PGAM4	-2.4563440357	0.001106	0.8381590036
PGK1	1.3155914723	0.0175422094	0.9999763851
TAF9B	1.1628145501	0.4293903183	0.9999763851
CYSLTR1	1.2320939056	0.4115800563	0.9999763851
LPAR4	1.6637897627	0.5538972415	0.9999763851
P2RY10	0	0	0
BRWD3	1.1575592079	0.6660558757	0.9999763851
HMGN5	-1.0635429505	0.8509476692	0.9999763851
SH3BGRL	-1.0465589908	0.7740906806	0.9999763851
APOOL	-1.1709556645	0.5662046408	0.9999763851
SATL1	-1.3969420996	0.1931312729	0.9999763851
CHM	-1.0494133147	0.8522243885	0.9999763851
DACH2	3.2353357581	0.554787377	0.9999763851
PCDH11X	-1.1936051126	0.7086869192	0.9999763851
DIAPH2	-1.1970875105	0.3345913465	0.9999763851
RPA4	-3.3272796812	0.2416806948	0.9999763851
TSPAN6	0	0	0
SRPX2	0	0	0
CSTF2	1.2221953461	0.4478723682	0.9999763851
NOX1	-1.4379838511	0.2130291302	0.9999763851
XKRX	2.9232244853	0.2442102849	0.9999763851
ARL13A	-4.2849908816	0.4028453076	0.9999763851

TRMT2B	1.6303232337	0.0342620234	0.9999763851
CENPI	-1.0919815161	0.6201048971	0.9999763851
DRP2	3.2353357581	0.554787377	0.9999763851
TAF7L	9.0182935529	0.2609657796	0.9999763851
TIMM8A	1.2812091185	0.2272376241	0.9999763851
BTK	-1.3888691604	0.3330595002	0.9999763851
RPL36A	-1.0420715634	0.7363288338	0.9999763851
RPL36A-HNRNPH2	2.544562497	0.0387463177	0.9999763851
GLA	-1.1422652531	0.6335434355	0.9999763851
HNRNPH2	1.0411480659	0.7862114063	0.9999763851
ZMAT1	1.3264789913	0.7365132275	0.9999763851
NXF2	3.2353357581	0.554787377	0.9999763851
ARMCX5	-1.6284449418	0.1078910947	0.9999763851
ARMCX5-GPRASP2	-1.545717287	0.473734045	0.9999763851
GPRASP1	3.2356557963	0.5547505991	0.9999763851
GPRASP2	2.2221563097	0.3483409618	0.9999763851
BHLHB9	2.2210080778	0.3400283051	0.9999763851
BEX1	1.1959576961	0.1321389594	0.9999763851
NXF3	1.1844904925	0.3044025267	0.9999763851
TCEAL8	-1.0090719407	0.9741041649	0.9999763851
MORF4L2	-1.1498286819	0.2537412253	0.9999763851
TMEM31	7.7204093521	0.2871636946	0.9999763851
SLC25A53	1.1539476483	0.6061023964	0.9999763851
FAM199X	1.1701032242	0.4467382393	0.9999763851
NRK	0	0	0
RNF128	1.5203268921	0.0428728861	0.9999763851
CLDN2	0	0	0
RBM41	-1.8993910992	0.018435713	0.9999763851
NUP62CL	-1.3709710942	0.7479558959	0.9999763851
FRMPD3	0	0	0
PRPS1	1.0170642475	0.8949629157	0.9999763851
TSC22D3	-1.3625562525	0.1711797479	0.9999763851
MID2	0	0	0
PSMD10	-1.2786988487	0.172711177	0.9999763851
ATG4A	1.2363127965	0.4536382615	0.9999763851
GUCY2F	0	0	0
NXT2	-1.0074371304	0.9726342134	0.9999763851
KCNE5	-1.3154339304	0.8205576571	0.9999763851
ACSL4	-1.3531337689	0.0534029084	0.9999763851
LOC105373311	0	0	0
TMEM164	-2.3256462279	0.0279298985	0.9999763851
LOC101928589	1.0128923067	0.9405360741	0.9999763851
AMMECR1	1.1163284952	0.4706754767	0.9999763851
PAK3	0	0	0
ALG13	1.2832457643	0.3277979776	0.9999763851
TRPC5	-4.2849908816	0.4028453076	0.9999763851
ZCCHC16	0	0	0
AMOT	0	0	0
HTR2C	0	0	0

SLC6A14	0	0	0
KLHL13	-7.5622178268	0.2177145568	0.9999763851
WDR44	-1.2867962094	0.4216882584	0.9999763851
DOCK11	1.0252970311	0.8907222698	0.9999763851
IL13RA1	-1.140370259	0.7119758921	0.9999763851
LONRF3	1.0549477691	0.8449792278	0.9999763851
KIAA1210	3.2353357581	0.554787377	0.9999763851
PGRMC1	1.1265560212	0.4379595853	0.9999763851
LOC107985692	1.028107434	0.9764068387	0.9999763851
SLC25A43	1.1844585705	0.5170993692	0.9999763851
SLC25A5	1.0670132311	0.6288575696	0.9999763851
CXorf56	-1.0144012522	0.9369106824	0.9999763851
UBE2A	1.2553873273	0.1365634671	0.9999763851
NKRF	1.1989286777	0.4182014241	0.9999763851
SEPT6	1.0542922729	0.7479085908	0.9999763851
SOWAHD	-1.5574413096	0.1094395301	0.9999763851
RPL39	-1.2206888271	0.0903692017	0.9999763851
UPF3B	-1.1147038553	0.5051723384	0.9999763851
RNF113A	-1.1380165846	0.5922713725	0.9999763851
NDUFA1	-1.0640445523	0.6382188041	0.9999763851
NKAP	-1.290596591	0.2886927211	0.9999763851
RHOXF2B	1.0931859836	0.6239408594	0.9999763851
RHOXF1	-2.3212684229	0.4490660804	0.9999763851
RHOXF2	1.0356249387	0.8658316457	0.9999763851
ZBTB33	-1.0534764424	0.7911118114	0.9999763851
TMEM255A	-1.421366223	0.7027283292	0.9999763851
LAMP2	-1.2294740586	0.0886505595	0.9999763851
CUL4B	-1.5418605154	0.009505	0.9999763851
MCTS1	1.0320054111	0.8377654909	0.9999763851
C1GALT1C1	-1.2815186629	0.2053470938	0.9999763851
GLUD2	0	0	0
GRIA3	0	0	0
THOC2	-1.0780940073	0.62766397	0.9999763851
XIAP	-1.030253522	0.885780927	0.9999763851
STAG2	-1.2475689796	0.1254940216	0.9999763851
SH2D1A	0	0	0
TENM1	0	0	0
SMARCA1	-4.2849908816	0.4028453076	0.9999763851
OCRL	1.1667515589	0.6057142129	0.9999763851
APLN	1.0739483694	0.8527518242	0.9999763851
SASH3	-1.1955019714	0.5180133536	0.9999763851
ZDHHC9	-1.4522850717	0.2901047194	0.9999763851
UTP14A	1.0636156443	0.7935158949	0.9999763851
BCORL1	-1.5857351591	0.1498111938	0.9999763851
ELF4	-1.0208225032	0.93052989	0.9999763851
RAB33A	-1.3636445218	0.2348444116	0.9999763851
AIFM1	1.0638062388	0.7269319748	0.9999763851
ZNF280C	-1.0159473794	0.9785897534	0.9999763851
SLC25A14	1.6753475703	0.3450928518	0.9999763851

RBMX2	-1.2748094198	0.2547520794	0.9999763851
ENOX2	-2.132117667	0.007662	0.9999763851
STK26	1.2177057538	0.3532860046	0.9999763851
RAP2C	-1.0959609813	0.7825573475	0.9999763851
MBNL3	1.4759716183	0.1791659182	0.9999763851
HS6ST2	3.2356557963	0.5547505991	0.9999763851
USP26	0	0	0
GPC4	0	0	0
GPC3	-1.196198281	0.3323894559	0.9999763851
PHF6	-1.1200584376	0.5596286482	0.9999763851
HPRT1	-1.0944752509	0.5993886396	0.9999763851
PLAC1	-4.2849908816	0.4028453076	0.9999763851
FAM122B	-1.1834007807	0.4315408975	0.9999763851
FAM122C	1.5501971516	0.5514360035	0.9999763851
MOSPD1	-1.0389294895	0.8903987234	0.9999763851
ZNF75D	1.0656407304	0.8623107089	0.9999763851
ZNF449	1.3625577098	0.5439964944	0.9999763851
INTS6L	-1.1792156438	0.6405103661	0.9999763851
MMGT1	-1.379267999	0.0470949682	0.9999763851
SLC9A6	-1.1118685855	0.6594975818	0.9999763851
FHL1	-2.5596117216	0.2622579937	0.9999763851
MAP7D3	1.2140077333	0.3476429432	0.9999763851
HTATSF1	1.1878277556	0.3271124364	0.9999763851
CD40LG	0	0	0
ARHGEF6	1.3823840123	0.0663173568	0.9999763851
RBMX	1.1894441661	0.212235094	0.9999763851
ATP11C	-1.1623857971	0.6491903299	0.9999763851
FMR1	1.0531286057	0.8070604233	0.9999763851
AFF2	-1.2496200521	0.1576433464	0.9999763851
IDS	-1.1600717323	0.3504041917	0.9999763851
CXorf40A	1.0650515411	0.6562378832	0.9999763851
LOC101928917	-1.2440466269	0.8062696907	0.9999763851
HSFX2	0	0	0
TMEM185A	-1.0836737653	0.7482294461	0.9999763851
CXorf40B	1.1605570989	0.7098520795	0.9999763851
LOC101927685	10.4285443305	0.2412517111	0.9999763851
MAMLD1	-1.2505104404	0.6167819305	0.9999763851
MTM1	-1.0979035237	0.7888241439	0.9999763851
MTMR1	1.0798575008	0.7011637079	0.9999763851
CD99L2	-1.1589734621	0.3935521396	0.9999763851
HMGB3	1.0968298592	0.5834890602	0.9999763851
VMA21	-1.072787977	0.5599800554	0.9999763851
CETN2	1.2244124268	0.3564465786	0.9999763851
NSDHL	1.3420805834	0.0867692213	0.9999763851
ZNF185	-1.1480107667	0.7088226083	0.9999763851
PNMA5	-3.5025636081	0.0814357562	0.9999763851
PNMA6A	-1.035933364	0.9119548403	0.9999763851
LOC105373377	0	0	0
ZNF275	1.1667838182	0.3709045723	0.9999763851

ZFP92	-1.1006359229	0.8004925966	0.9999763851
TREX2	0	0	0
HAUS7	1.0447737834	0.8115390768	0.9999763851
ATP2B3	1.6016752259	0.048130337	0.9999763851
FAM58A	-1.089243938	0.5875825455	0.9999763851
DUSP9	0	0	0
PNCK	0	0	0
SLC6A8	1.0690172838	0.8559098113	0.9999763851
BCAP31	-1.1792743487	0.2009318969	0.9999763851
ABCD1	-1.190711552	0.7392376829	0.9999763851
PLXNB3	-4.2849908816	0.4028453076	0.9999763851
SRPK3	3.2353357581	0.554787377	0.9999763851
IDH3G	1.1346603404	0.5355827764	0.9999763851
SSR4	-1.0155381757	0.9059964588	0.9999763851
L1CAM	-4.2849908816	0.4028453076	0.9999763851
ARHGAP4	1.0471879359	0.7858131542	0.9999763851
NAA10	1.0864836414	0.6338615596	0.9999763851
RENBP	-1.3478377477	0.70763595	0.9999763851
HCFC1	-1.087419549	0.5290397413	0.9999763851
TMEM187	-1.1766249893	0.5839295293	0.9999763851
IRAK1	-1.0701854491	0.5928609473	0.9999763851
MECP2	1.0386967751	0.8373976116	0.9999763851
OPN1MW3	0	0	0
TEX28	-4.2864494036	0.4027150576	0.9999763851
TKTL1	0	0	0
FLNA	1.113066242	0.4517748001	0.9999763851
EMD	-1.1266188698	0.3739418889	0.9999763851
LOC107987332	-1.0805489166	0.9266273402	0.9999763851
RPL10	1.007566759	0.9508925814	0.9999763851
DNASE1L1	25.7318302623	0.086611497	0.9999763851
TAZ	1.3416866188	0.250956068	0.9999763851
ATP6AP1	-1.290725549	0.0931001185	0.9999763851
GDI1	-1.0578991638	0.8141885679	0.9999763851
FAM50A	-1.0997381288	0.6697718491	0.9999763851
PLXNA3	-1.7100795486	0.2800622542	0.9999763851
LAGE3	1.1097842427	0.4491767082	0.9999763851
UBL4A	1.117272202	0.4908774701	0.9999763851
SLC10A3	1.0102115797	0.9712896597	0.9999763851
FAM3A	-1.1759623533	0.5227866729	0.9999763851
G6PD	1.061669991	0.791531188	0.9999763851
IKBKG	1.1892372608	0.3855290077	0.9999763851
CTAG1A	0	0	0
CTAG2	0	0	0
GAB3	-1.126520263	0.7606676546	0.9999763851
DKC1	-1.019636857	0.8746029938	0.9999763851
MPP1	-1.0174105478	0.9204934252	0.9999763851
F8	-1.6000763874	0.1136264493	0.9999763851
F8A1	1.1705011255	0.3793251097	0.9999763851
FUNDC2	1.3538261043	0.0450860948	0.9999763851

CMC4	-1.1608255751	0.5863480437	0.9999763851
BRCC3	1.3096431394	0.0551580228	0.9999763851
VBP1	1.235780371	0.1441397678	0.9999763851
RAB39B	-10.8385246611	0.1454069559	0.9999763851
CLIC2	5.4776292235	0.3745831165	0.9999763851
H2AFB3	0	0	0
TMLHE	-1.5706816925	0.0753798139	0.9999763851
VAMP7_1	-1.141054452	0.5454661057	0.9999763851
IL9R_1	1.6938437942	0.6481572941	0.9999763851
PLCXD1_2	1.2905586889	0.3873702766	0.9999763851
GTPBP6_2	-1.3046636917	0.3468079464	0.9999763851
PPP2R3B_2	1.5771203822	0.4036766773	0.9999763851
CRLF2_2	3.2361532718	0.5546934395	0.9999763851
CSF2RA_2	1.2803114561	0.5266534532	0.9999763851
IL3RA_2	-3.6968212056	0.0403926302	0.9999763851
SLC25A6_2	-1.0665690999	0.6273739961	0.9999763851
LOC105373102_2	0	0	0
ASMTL_2	1.0643864794	0.846950869	0.9999763851
P2RY8_2	1.1317377513	0.4851385916	0.9999763851
AKAP17A_2	-1.4204208174	0.1312113196	0.9999763851
DHRSX_2	1.6720530332	0.2414991074	0.9999763851
ZBED1_2	1.3579048027	0.3925305766	0.9999763851
CD99_2	1.3642399858	0.0567801736	0.9999763851
PCDH11Y	1.2978756447	0.6533023971	0.9999763851
TBL1Y	3.2356557963	0.5547505991	0.9999763851
UTY	-1.0446740863	0.8391423821	0.9999763851
VAMP7_2	-1.0257561175	0.9060544063	0.9999763851
IL9R_2	-4.2864494036	0.4027150576	0.9999763851

LZ vs. C 2H - Fold change	LZ vs. C 2H - P-value	LZ vs. C 2H - FDR p-value	LZ vs. C 24H - Fold change
1.10528199	0.9276332648	0.9999537652	1.8767838835
-1.5860648294	0.2531574376	0.9999537652	1.0136550264
-2.6446103845	0.1562354099	0.9999537652	3.0003884395
-5.4085287896	0.1678820504	0.9999537652	2.6680086435
-1.1605807802	0.5166447141	0.9999537652	-1.2193980307
-1.8463159225	0.3754329048	0.9999537652	1.9767548276
-1.1614998763	0.8765266921	0.9999537652	-7.959096917
43.1761895246	0.138814462	0.9999537652	-1.4457524838
1.3831342462	0.1347081514	0.9999537652	1.3681076502
2.0510528871	0.014024137	0.9999537652	-1.720458724
-2.2358596109	0.3474892464	0.9999537652	-1.7815563324
1.4934216573	0.322078157	0.9999537652	-1.4542840694
2.7933974408	0.6948713406	0.9999537652	2.996475627
1.1516354134	0.2960876305	0.9999537652	-1.0196853503
-1.1773928401	0.3695770411	0.9999537652	-1.0465296466
6.3130691196	0.4797718667	0.9999537652	-2.579624099
1.2399533326	0.150158816	0.9999537652	-1.4706241177
1.6505943575	0.3275001729	0.9999537652	-1.076260814
1.9844400563	0.0837345008	0.9999537652	1.198439035
1.3482604893	0.3884706256	0.9999537652	1.1773892926
-1.1914214817	0.3862039382	0.9999537652	-1.2309828877
-1.0385845306	0.9212851175	0.9999537652	-1.1931526885
0	0	0	4.9527501949
-1.1029431029	0.6938649941	0.9999537652	-1.5093905438
2.7957660672	0.7053686484	0.9999537652	-2.2688699986
-1.0305638735	0.8451673338	0.9999537652	-1.1442878229
1.1365504914	0.4660309926	0.9999537652	-1.373255798
-1.3692914198	0.0129226026	0.9999537652	-1.0358353977
0	0	0	0
-1.1906603306	0.3566322876	0.9999537652	-1.0552255407
1.1094478586	0.5676066705	0.9999537652	1.1828806805
4.5545995984	0.5696849358	0.9999537652	15.9659633994
1.1381578178	0.4220729377	0.9999537652	-1.0934581933
-2.045920934	0.2680651636	0.9999537652	1.194084372
2.4053922493	0.072197911	0.9999537652	1.0603766475
4.5505976667	0.5602947896	0.9999537652	2.0426682045
-1.0303073468	0.9092333091	0.9999537652	1.0525858406
-1.3668253196	0.6211065807	0.9999537652	-1.5695393714
1.6383451237	0.1764597898	0.9999537652	-1.1745673469
-1.0958096873	0.6506193145	0.9999537652	-1.3134652331
1.1496401381	0.3645879395	0.9999537652	1.1582559275
1.0562323577	0.6839483801	0.9999537652	-1.0596568104
0	0	0	0
0	0	0	2.996475627
1.8448460942	0.5998814491	0.9999537652	-1.1444432957
9.8205505243	0.3808420729	0.9999537652	-2.1294907556
3.4733637305	0.1323277297	0.9999537652	-4.1628315719
-1.3161399115	0.6818520939	0.9999537652	-2.7336138579

1.2331805204	0.3514761239	0.9999537652	1.1099148212
1.2203996378	0.3274799992	0.9999537652	1.0600268824
1.0808480048	0.9081655184	0.9999537652	1.1856012743
-1.1292443617	0.4351211423	0.9999537652	1.0374261822
-1.0573626032	0.9485462547	0.9999537652	1.2648328658
2.7933974408	0.6948713406	0.9999537652	2.996475627
-9.978837909	0.0402225068	0.9999537652	4.3673319332
1.464268315	0.1950663647	0.9999537652	-1.2477442156
1.0052819665	0.9854632738	0.9999537652	-1.3577034746
3.9205610751	0.3357966134	0.9999537652	-2.2890929548
11.5766527773	0.3453182652	0.9999537652	-1.0416326979
-1.110189331	0.5774271764	0.9999537652	1.0930564242
1.1575446714	0.3998498426	0.9999537652	-1.101093941
-1.3400297652	0.6616825679	0.9999537652	1.1651206374
0	0	0	1.4002700172
1.172424593	0.3590082646	0.9999537652	1.1175147103
-1.7351800694	0.1234754864	0.9999537652	1.4313839154
-1.6349152306	0.0389941583	0.9999537652	-1.2850581633
-1.2060989687	0.3846621475	0.9999537652	1.2070699866
1.2346183256	0.5355491746	0.9999537652	-1.3840773989
1.0608210445	0.6741671183	0.9999537652	-1.1317017104
8.064174035	0.4217332484	0.9999537652	8.8639046058
1.0332721989	0.7796012859	0.9999537652	1.1051144085
0	0	0	-1.8579915692
-1.0279853933	0.8620338982	0.9999537652	-1.2607518155
1.6090500367	0.3625909589	0.9999537652	-1.0678039382
-1.2532594199	0.1940100651	0.9999537652	-1.1878739819
-1.0015629939	0.9929026379	0.9999537652	-1.0560370856
0	0	0	2.996475627
-1.0470191668	0.9220269612	0.9999537652	-1.4349942283
-1.0708604909	0.7170251155	0.9999537652	-1.0960965781
-1.20855776	0.4169736539	0.9999537652	-1.4736426865
1.0841005925	0.6635011713	0.9999537652	-1.1848744921
2.6978201187	0.2074092412	0.9999537652	1.2941165299
1.1196684085	0.5745829099	0.9999537652	1.1240225664
-1.0370558735	0.8737506926	0.9999537652	-1.2077559626
-1.5820364396	0.0373170244	0.9999537652	1.2640773099
-4.7509653312	0.0676923003	0.9999537652	5.5797416422
-1.474256113	0.6746833091	0.9999537652	-2.1299895658
1.1296005396	0.3323816266	0.9999537652	-1.0167862486
1.2673417318	0.6154243016	0.9999537652	-1.0470597281
-1.0983845365	0.5738426571	0.9999537652	-1.1525448434
1.0206140979	0.8581335656	0.9999537652	-1.1767681806
11.5766527773	0.3453795744	0.9999537652	-1.2612704763
4.5505976667	0.5602519568	0.9999537652	1.8368734345
1.0763770131	0.7359935714	0.9999537652	-1.2326808857
-2.1325963601	0.4603080994	0.9999537652	1.3143074801
-1.0726620988	0.8037005364	0.9999537652	-1.0191594585
-1.1189982814	0.8120281354	0.9999537652	1.1860886022

1.3118920539	0.34727647	0.9999537652	1.4880588297
1.0914623784	0.7930215925	0.9999537652	-1.5205147039
1.5291298825	0.3532551279	0.9999537652	-1.1537126741
-1.2015263683	0.2364451284	0.9999537652	-1.2282585625
1.0637999368	0.741521951	0.9999537652	-1.2000274238
1.1704047011	0.4190084799	0.9999537652	1.0368675479
1.0034416048	0.9848842656	0.9999537652	1.0392791098
1.044734083	0.9162329306	0.9999537652	1.7317352387
0	0	0	4.9527501949
-1.0993527828	0.6900911222	0.9999537652	1.137517165
1.2564260193	0.3591803667	0.9999537652	-1.1771672068
1.0855263342	0.6130272174	0.9999537652	-1.1258264514
1.1048804532	0.6482951209	0.9999537652	1.8265018786
16.8433158146	0.2732521726	0.9999537652	1.1908762699
1.8176665782	0.2803966492	0.9999537652	1.1917871104
1.0818727716	0.782196633	0.9999537652	-1.3224092277
1.3064529969	0.3466226355	0.9999537652	-1.0220704003
-1.2931976772	0.4084820216	0.9999537652	-1.3262834747
-1.2019892207	0.1829553932	0.9999537652	-1.0139778938
-6.9764533263	0.2589868856	0.9999537652	10.8262784008
1.1126634716	0.4317679094	0.9999537652	1.0143094048
1.2796759192	0.2868486277	0.9999537652	1.205614606
1.4342270601	0.0959307236	0.9999537652	-1.1242533918
-1.2647715161	0.2021858668	0.9999537652	1.1426264459
1.0737844037	0.9174636831	0.9999537652	-1.0755220967
-4.8154268017	0.1712535923	0.9999537652	1.401081723
-1.1760439694	0.7322879508	0.9999537652	1.9159733225
-1.1675671268	0.4305517183	0.9999537652	-1.0775371582
1.1168108737	0.9283095311	0.9999537652	-2.2407182823
1.5217239176	0.065183155	0.9999537652	-1.4261172168
1.5129672515	0.2592466998	0.9999537652	-1.641094014
1.0813404924	0.8356929949	0.9999537652	-1.5563677034
1.0717488148	0.6398508845	0.9999537652	-1.1509258264
1.4597061306	0.11471059	0.9999537652	-1.1890517545
1.0269468359	0.8816704934	0.9999537652	1.1342587074
-1.8964662446	0.0626215067	0.9999537652	-1.1429488648
1.4322794364	0.0121417135	0.9999537652	-1.2295595384
-1.1897798958	0.5568564682	0.9999537652	1.0043705722
1.3115617248	0.6180786908	0.9999537652	-1.1773184466
-7.8036543138	0.0001877	0.9999537652	-1.537349674
-1.0310062934	0.8832622147	0.9999537652	1.1414587399
-1.4596913105	0.2218197026	0.9999537652	-1.1037459052
1.3048687591	0.7280138725	0.9999537652	-1.4238795843
0	0	0	2.996475627
-1.0744629625	0.703009882	0.9999537652	-1.1382543897
0	0	0	-5.3589986948
2.7933974408	0.6948713406	0.9999537652	-1.1517639145
2.2973085201	0.3347705566	0.9999537652	-1.0001429436
-1.3015793855	0.6212406659	0.9999537652	1.0473903176

1.5529110445	0.3265626359	0.9999537652	-1.4669246696
4.5505976667	0.5601667107	0.9999537652	-2.0477227478
-1.7258641834	0.4572354644	0.9999537652	-1.7043700168
1.1260783533	0.874823167	0.9999537652	1.2400291296
-1.1411794826	0.6103272282	0.9999537652	-1.1000749884
0	0	0	4.9561842702
0	0	0	2.996475627
0	0	0	10.818785458
-1.6975052657	0.004311	0.9999537652	-1.4734484598
-1.1620087829	0.465133506	0.9999537652	-1.2963520819
-1.0464740425	0.9164900533	0.9999537652	1.0218716297
1.7746691972	0.0920574714	0.9999537652	1.177682036
-1.5881166222	0.7258793166	0.9999537652	-2.8769815942
0	0	0	-4.1123558411
1.551024778	0.2155505763	0.9999537652	1.0389279812
-1.0971375911	0.5913987171	0.9999537652	1.0588529503
-1.0957145392	0.6815053152	0.9999537652	-1.2452416615
9.8205505243	0.3808419294	0.9999537652	0
-1.0419294728	0.8643541431	0.9999537652	-1.1981180197
-1.1614010521	0.8859841055	0.9999537652	-1.859280336
1.5692454894	0.3144870947	0.9999537652	-1.272799313
2.4246765564	0.3289726365	0.9999537652	-1.6166104906
-1.7991827318	0.1599768416	0.9999537652	1.6631648689
1.1721069104	0.4231487407	0.9999537652	1.5818326671
-1.0116689235	0.981072017	0.9999537652	-1.1184437609
11.5864583426	0.3448356866	0.9999537652	-9.7598809705
1.1158583321	0.9297364246	0.9999537652	4.9527501949
-1.0570844474	0.7048086156	0.9999537652	1.1865151013
-1.2654738527	0.4412787449	0.9999537652	-1.5584993668
1.0678890188	0.8326439865	0.9999537652	4.5786566845
0	0	0	4.9527501949
1.0750163095	0.7314637246	0.9999537652	1.1198036744
1.2056652288	0.5924413045	0.9999537652	-1.5260706903
-1.0154268928	0.9222589647	0.9999537652	-1.229336511
-1.1331646408	0.5215903731	0.9999537652	-1.209742527
1.1994744877	0.215167938	0.9999537652	1.3527275514
1.403011356	0.5100676788	0.9999537652	-1.0175257376
1.0762862679	0.7100499246	0.9999537652	-1.318299503
-1.1218301713	0.7886031365	0.9999537652	1.4979993531
-1.1764280082	0.2798347826	0.9999537652	-1.0561374651
1.1448492661	0.2887277473	0.9999537652	1.0527462452
0	0	0	6.908559683
9.8205505243	0.3808417859	0.9999537652	0
1.6993243546	0.1953859618	0.9999537652	-1.2411843512
-1.7534006362	0.3972637108	0.9999537652	-1.3017571381
0	0	0	-1.859280336
-1.2944263568	0.7117037764	0.9999537652	1.0391615581
1.2489712975	0.5694639771	0.9999537652	-1.1788432134
9.8205505243	0.3808416424	0.9999537652	-1.2450158522

-1.2009319295	0.702008548	0.9999537652	-1.0661298805
1.6470826326	0.1223015051	0.9999537652	1.3581885365
1.1320933008	0.684023517	0.9999537652	1.061240163
-1.0049166968	0.9823524039	0.9999537652	-1.1797894605
-1.1496649418	0.3198611941	0.9999537652	-1.0360183373
2.4133372013	0.2023508426	0.9999537652	-1.3071878875
-1.19172018	0.2315250344	0.9999537652	1.0446619467
-1.3434418218	0.1583998081	0.9999537652	1.0484512629
1.3732135791	0.2617531941	0.9999537652	-1.1576880251
1.3937045222	0.4388371092	0.9999537652	1.0053122895
0	0	0	-1.4671686592
-1.2218032769	0.2053928402	0.9999537652	-1.1103354284
2.8197560167	0.3542734986	0.9999537652	-2.4486849086
2.7933974408	0.6948713406	0.9999537652	-2.763189328
0	0	0	0
1.0672621842	0.6327788678	0.9999537652	1.2169611695
-1.590255273	0.1879166969	0.9999537652	-1.1405754084
0	0	0	1.0667457716
-3.4373511952	0.0248626078	0.9999537652	-1.2771771246
0	0	0	2.9985540909
8.064174035	0.4218818154	0.9999537652	8.8761957907
-1.0791207813	0.7440224237	0.9999537652	-1.2810451512
1.4965788567	0.2852845556	0.9999537652	1.1254891831
1.0750942371	0.6200566068	0.9999537652	1.3066423756
1.2537376893	0.8095955294	0.9999537652	1.1040524007
-1.9361116524	0.2794128531	0.9999537652	-1.0667363766
-1.1447852916	0.915393807	0.9999537652	-1.5086090391
-1.3400152954	0.1900421589	0.9999537652	-1.1868502474
2.5569228903	0.2905718207	0.9999537652	-1.2376612364
-1.141509172	0.263860757	0.9999537652	1.0430214279
-1.2429792146	0.4555557552	0.9999537652	-1.2236459029
1.0181442537	0.9193517276	0.9999537652	1.0139763625
1.5261191208	0.0201939316	0.9999537652	-1.2672325335
1.3039775859	0.2559723514	0.9999537652	1.4952908799
1.8332330904	0.0594152722	0.9999537652	-1.1496850827
1.0277663838	0.8580232464	0.9999537652	-1.1057368774
1.4016970427	0.1746374643	0.9999537652	-1.0260127158
1.0218616277	0.9130820929	0.9999537652	1.3753063988
-1.1288878214	0.3808223911	0.9999537652	1.0738389115
29.8698312703	0.2177267878	0.9999537652	0
0	0	0	2.996475627
0	0	0	6.908559683
-2.5849372227	0.5263317694	0.9999537652	1.1566640911
32.6858196958	0.1751819618	0.9999537652	-14.149476906
-1.0184593498	0.9668655544	0.9999537652	-1.2561727436
3.1790945967	0.1209938425	0.9999537652	1.0675523501
0	0	0	-1.8577842586
-1.3447452208	0.0387185886	0.9999537652	1.1200722131
1.0665167775	0.7412067047	0.9999537652	1.1528263681

-1.1354078557	0.350677279	0.9999537652	-1.2444105602
-1.0524246233	0.7887770687	0.9999537652	1.3386813366
1.1155148055	0.5701359842	0.9999537652	1.2266753676
1.2477586312	0.2155490651	0.9999537652	1.3979704468
-2.9523355565	0.3410999344	0.9999537652	1.1381503565
1.3149096804	0.3082638591	0.9999537652	-1.3075866405
1.5808525587	0.0449636379	0.9999537652	-1.1278235195
1.3092729054	0.6816045262	0.9999537652	-1.5035806696
1.2283025042	0.4718104648	0.9999537652	-1.202902615
-4.3600564455	0.1220241376	0.9999537652	-1.1706134134
1.0339753574	0.875351026	0.9999537652	1.548465534
-1.0076821978	0.9935464972	0.9999537652	-6.5476410741
-1.1314437449	0.2611415892	0.9999537652	-1.0046649058
1.543349394	0.3251748101	0.9999537652	-1.2209089991
-1.4982318958	0.3290218254	0.9999537652	1.2518282786
-1.1325176624	0.8544192897	0.9999537652	-2.9099961801
1.0204847908	0.9060053277	0.9999537652	-1.0554711626
0	0	0	-5.3589986948
0	0	0	2.9985540909
-1.1289689692	0.7157749477	0.9999537652	1.0063338304
-1.0229372541	0.880544896	0.9999537652	-1.2601794007
-2.1280553469	0.2609296482	0.9999537652	-1.6419022999
-1.0860630806	0.6751624839	0.9999537652	1.3340777614
0	0	0	6.908559683
-1.0447383068	0.880831421	0.9999537652	-1.2223702414
-1.3009386067	0.0208704337	0.9999537652	-1.1377342995
1.0032331827	0.98524775	0.9999537652	-1.376033252
-1.0437177504	0.8484588017	0.9999537652	-1.0149433916
-1.0096484376	0.9873212096	0.9999537652	1.2389534888
1.5024583949	0.1887806579	0.9999537652	-1.1483432976
-5.6856811034	0.0707662592	0.9999537652	-1.859280336
-1.789269507	0.1059902484	0.9999537652	-1.4988235641
1.0730433706	0.8369116387	0.9999537652	1.0545907089
1.1714077321	0.3400891644	0.9999537652	1.3070615412
2.7933974408	0.6948713406	0.9999537652	0
-1.0100340266	0.9887471041	0.9999537652	-1.0989904503
1.6704883732	0.3479647645	0.9999537652	1.8969815978
1.604666205	0.3082122442	0.9999537652	-1.2613931533
-1.2242279135	0.5269571422	0.9999537652	-1.248448714
-1.7618136108	0.1949034723	0.9999537652	-1.6606015239
-1.562511822	0.373223776	0.9999537652	1.5393291474
-1.5881570201	0.7146301739	0.9999537652	0
0	0	0	0
-1.1545963256	0.3370611736	0.9999537652	-1.3559302201
-1.3227196977	0.4984757332	0.9999537652	1.1792892539
-1.1088351144	0.5955295795	0.9999537652	1.1536883034
3.2033390901	5.05E-05	0.4532897791	1.3419260067
-1.5912011739	0.2421582398	0.9999537652	1.2540059547
-1.0751790306	0.8180323213	0.9999537652	1.0203647951

1.1438931627	0.4516663143	0.9999537652	1.1980908138
1.0856236913	0.6891805068	0.9999537652	1.3373138363
1.1427767498	0.3570358765	0.9999537652	-1.1329318032
11.5766527773	0.3454410623	0.9999537652	-1.2787310716
1.1365121976	0.767701082	0.9999537652	-1.270468274
1.2254326777	0.2857157666	0.9999537652	1.1232950943
1.3741714424	0.1038410379	0.9999537652	-1.1627235497
-1.1497536519	0.2915725961	0.9999537652	-1.3279684207
-1.1207502931	0.382197607	0.9999537652	1.0223549505
1.1496921257	0.6873342003	0.9999537652	-1.1303011748
1.1443001377	0.5328957798	0.9999537652	1.0911481205
1.3978582255	0.1147921926	0.9999537652	-1.2111656156
-1.0766524571	0.5890138713	0.9999537652	1.0948628877
1.0273494929	0.8966956411	0.9999537652	-1.0214419977
-1.0607912724	0.7598743747	0.9999537652	-1.1333879622
-1.1967624622	0.6007272064	0.9999537652	-1.2272270493
1.0714346035	0.6355078051	0.9999537652	1.2043980651
0	0	0	2.9985540909
-1.3548532324	0.2887876617	0.9999537652	1.0907258221
1.2171458216	0.2944296163	0.9999537652	1.1681252601
1.1051145612	0.6637526669	0.9999537652	1.2098581886
-1.1185944805	0.7185550791	0.9999537652	-1.0593186558
-1.5799266646	0.3938777432	0.9999537652	-1.1569776719
2.2857336825	0.2833602445	0.9999537652	-2.1545257517
1.0332333907	0.8094728176	0.9999537652	-1.0207763969
-1.1073437342	0.6042753505	0.9999537652	-1.1423640102
0	0	0	8.8639046058
2.3442980505	0.00678	0.9999537652	2.201778415
1.7979931789	0.0944765429	0.9999537652	1.1609100073
-1.038894833	0.888402065	0.9999537652	-1.1696206489
1.1040648097	0.7883568421	0.9999537652	-1.3353650969
18.5983228161	0.2580928751	0.9999537652	2.2945277226
4.5505976667	0.5601242665	0.9999537652	0
1.1254792007	0.6211463848	0.9999537652	-1.0753446658
0	0	0	10.818785458
0	0	0	-1.1254032646
55.4372391663	0.1147996924	0.9999537652	1.1338344759
1.0144837009	0.9168845455	0.9999537652	-1.1454522933
1.0243749863	0.8503847803	0.9999537652	1.2949812292
1.1872085626	0.5238286834	0.9999537652	1.2110972878
1.0781358157	0.677317153	0.9999537652	1.0248871388
1.4406217787	0.1031887252	0.9999537652	-1.2466835892
1.4466893048	0.1039928749	0.9999537652	1.0285208533
11.5766527773	0.3454528878	0.9999537652	4.9529970125
-2.4387448463	0.3485738865	0.9999537652	1.4541273836
-6.9736190158	0.2250321147	0.9999537652	2.9988886918
1.2900400603	0.0877242979	0.9999537652	1.189817362
2.7957660672	0.7053709654	0.9999537652	-5.3583704783
1.2533830106	0.142365015	0.9999537652	-1.175992956

-1.3614238431	0.2480889334	0.9999537652	-1.1189333769
-1.1457555741	0.9107267231	0.9999537652	-1.9566789748
1.3594099105	0.7971743526	0.9999537652	-2.173958308
1.2259155854	0.5337441509	0.9999537652	1.0911177817
-1.5320841189	0.24150329	0.9999537652	1.3911553586
1.4423177973	0.1062936902	0.9999537652	1.2017934321
-1.7227220134	0.0807320056	0.9999537652	-1.2254275527
1.3032749865	0.1319847735	0.9999537652	1.5729208749
-2.4906115235	0.156990793	0.9999537652	-1.1551081672
1.12057142	0.4635478989	0.9999537652	1.0784595307
-1.0043704115	0.9854537652	0.9999537652	-1.3371247115
0	0	0	6.908559683
1.0711012009	0.9181830574	0.9999537652	-1.1397615944
-1.3685209014	0.2178779165	0.9999537652	-1.265422587
1.1134695966	0.4014261559	0.9999537652	1.1724782094
3.9929885588	0.117094833	0.9999537652	-1.3490488064
-1.5354924971	0.6039487219	0.9999537652	-3.8549258508
-1.1280025391	0.773650983	0.9999537652	-1.0928761359
0	0	0	2.996475627
1.0420087918	0.8664534931	0.9999537652	-1.3763380209
-1.5875118025	0.720728186	0.9999537652	7.2225086035
0	0	0	1.8381437152
1.0926154152	0.6231511643	0.9999537652	-1.0232628409
4.5505976667	0.5600819198	0.9999537652	-5.3583704783
1.0794045774	0.8424035836	0.9999537652	1.4482711617
1.6330634729	0.0927260061	0.9999537652	1.223678401
1.044906612	0.9071823808	0.9999537652	-1.1100066722
-1.3279488045	0.0299032448	0.9999537652	1.0338097128
1.2475158226	0.0869075837	0.9999537652	1.0902649432
1.2396532016	0.2434787162	0.9999537652	1.0545114666
2.0450110053	0.2143930889	0.9999537652	-1.7084103116
1.1713242174	0.5403593713	0.9999537652	-1.2957489773
1.1261680185	0.3943093522	0.9999537652	-1.0089336775
1.1797801492	0.6227367526	0.9999537652	1.4777834233
1.3473079473	0.1031447121	0.9999537652	1.0576756121
-1.2045611581	0.5317745731	0.9999537652	1.2305376207
-1.3451777133	0.2621448914	0.9999537652	-1.0010144402
-1.2318295872	0.4112273969	0.9999537652	-1.1568211817
-1.0393161861	0.8395802028	0.9999537652	-1.2343426546
0	0	0	2.9985540909
-1.1115581008	0.4755648918	0.9999537652	-1.3980882715
-1.4181452512	0.0469945874	0.9999537652	-1.1004426199
-1.1006844411	0.4688745958	0.9999537652	-1.0285751786
-1.008417887	0.9935361022	0.9999537652	1.3117500655
20.3702769952	0.2388376207	0.9999537652	3.3380115123
-1.0583085622	0.7502207169	0.9999537652	1.0530619383
1.3080531364	0.1782914917	0.9999537652	-1.1920628602
1.1212430665	0.9127384187	0.9999537652	1.6203860779
1.2687304249	0.2900395142	0.9999537652	1.0874274909

-1.035902411	0.9039002051	0.9999537652	-1.0780384492
1.9974748864	0.2104286387	0.9999537652	-1.1628609811
-1.0139359282	0.9416100496	0.9999537652	-1.0868570947
-1.1570114275	0.4345759868	0.9999537652	1.064483668
1.5856903433	0.1504032706	0.9999537652	1.3255064867
4.5505976667	0.5600396528	0.9999537652	2.996475627
1.1002538735	0.5686891582	0.9999537652	1.0656291512
1.0286795814	0.9375929988	0.9999537652	1.0450332033
-1.3171866006	0.0791307732	0.9999537652	-1.3755108522
0	0	0	2.9936870021
-1.172656166	0.641383722	0.9999537652	-1.4127324621
-1.2416590917	0.3215959406	0.9999537652	-1.000767484
1.1450755216	0.4266514545	0.9999537652	-1.1910688372
-1.1679888826	0.625579704	0.9999537652	-1.1716453936
1.0675496976	0.7670807905	0.9999537652	-1.1795037312
-1.1420896173	0.3481934473	0.9999537652	-1.1526321072
1.5530248508	0.0909486209	0.9999537652	-1.224470308
-1.2254108314	0.221572877	0.9999537652	1.1774012103
8.064174035	0.4220879833	0.9999537652	-5.3583704783
1.1548106548	0.3772606293	0.9999537652	-1.0853578586
1.220469677	0.2516044354	0.9999537652	1.2547296735
-1.1605169044	0.87724665	0.9999537652	22.5539272529
1.3832388859	0.7303129975	0.9999537652	2.6927776414
1.1038170718	0.6220664145	0.9999537652	1.431180021
1.0477021779	0.7251321517	0.9999537652	1.1145577554
-1.269291824	0.1712494738	0.9999537652	-1.0730936685
-1.3668802709	0.7626139546	0.9999537652	1.7753008822
1.0809129028	0.6625002902	0.9999537652	-1.0300908234
6.3075231393	0.4870565495	0.9999537652	-2.9512799199
0	0	0	1.2400291296
1.1908963242	0.3754914071	0.9999537652	-1.3050063441
-6.9764533263	0.259011687	0.9999537652	-5.3589986948
3.8622037496	0.6197833075	0.9999537652	0
1.1279421177	0.5948685733	0.9999537652	1.2853061507
1.452960249	0.6330455447	0.9999537652	-1.4474945519
-1.2223452557	0.3896042942	0.9999537652	-1.2425261009
1.0263353284	0.8369840022	0.9999537652	-1.0721422223
-1.0685789358	0.6156289222	0.9999537652	1.0660829201
1.3259854412	0.1474313202	0.9999537652	-1.1009279041
1.1803283106	0.5624303581	0.9999537652	-1.0146938145
1.3442080467	0.4379190427	0.9999537652	-1.212009577
-1.0151320554	0.9378540944	0.9999537652	-1.0111186892
1.1236950163	0.8892568623	0.9999537652	-1.2310805544
1.2269824241	0.6860838188	0.9999537652	1.1284441498
-1.2353103691	0.613909627	0.9999537652	1.067496081
1.8122239907	0.3026711123	0.9999537652	-1.3616350993
1.6277441819	0.3379156267	0.9999537652	1.0452464871
-1.4228319157	0.1225655692	0.9999537652	-1.2826769754
0	0	0	4.9527501949

1.270931815	0.2810925714	0.9999537652	-1.0733539803
1.0872001045	0.6275433545	0.9999537652	1.0731629585
0	0	0	-5.3583704783
-1.2690293214	0.2143339979	0.9999537652	-1.2140979582
2.650087649	0.2276274606	0.9999537652	1.0662842542
-1.0409426418	0.9348906388	0.9999537652	1.6551813817
-1.075154381	0.7086016326	0.9999537652	1.1466305304
0	0	0	-1.1441751338
2.7933974408	0.6948713406	0.9999537652	1.1396966605
1.2951345093	0.1642799924	0.9999537652	1.1277171289
1.6455794001	0.5875096234	0.9999537652	-1.5809021445
-1.2183641759	0.2048828625	0.9999537652	-1.409190966
1.0398978461	0.7786895577	0.9999537652	1.4443700226
-1.2146479491	0.4365184718	0.9999537652	-1.2963167315
1.1565716118	0.7656411555	0.9999537652	-1.0160919311
0	0	0	1.2681859003
-1.0495582422	0.8967050307	0.9999537652	1.02194066
-1.4557262146	0.4433082788	0.9999537652	-1.0323778846
25.6372777234	0.20637664	0.9999537652	-1.5694163036
1.1997782402	0.3658540217	0.9999537652	-1.1822331645
-1.028547072	0.8374286041	0.9999537652	1.2281193937
4.5505976667	0.5599974468	0.9999537652	-3.382249888
2.7957660672	0.7053732832	0.9999537652	0
2.7933974408	0.6948713406	0.9999537652	0
4.5505976667	0.559955282	0.9999537652	1.5908108123
-1.1528972112	0.4614226755	0.9999537652	1.242496389
2.0222242291	0.0522194841	0.9999537652	1.3950796117
-1.2169205422	0.3215619449	0.9999537652	-1.1211764494
-1.5015849846	0.4870187897	0.9999537652	1.8439942031
-1.374129716	0.2142538533	0.9999537652	-1.6992005533
-2.5145521224	0.2123343195	0.9999537652	-1.248570001
1.110865361	0.6351870135	0.9999537652	-1.2007690487
-1.0877084206	0.836841528	0.9999537652	1.1388267452
-1.022930803	0.9356959145	0.9999537652	-1.1965536073
1.1737643413	0.5257982872	0.9999537652	1.6395947974
-1.1750224663	0.2181997378	0.9999537652	-1.1450381121
-1.2562244706	0.1913821449	0.9999537652	-1.3259678528
2.5559496274	0.2823303722	0.9999537652	-1.412904361
2.7933974408	0.6948713406	0.9999537652	0
-1.3032890847	0.2283528612	0.9999537652	-1.3823684937
-1.0466496129	0.9269454828	0.9999537652	-1.6804592274
1.1944930214	0.561824779	0.9999537652	1.2531283311
1.1208167739	0.4310563753	0.9999537652	-1.0912911915
2.2667759576	0.1565270749	0.9999537652	-1.1440764699
1.1400871961	0.486887258	0.9999537652	1.1171236468
-1.0484758332	0.7404669371	0.9999537652	-1.158712681
1.2024692751	0.11488698	0.9999537652	1.1012093621
-1.586167124	0.7106933692	0.9999537652	-1.9383054326
1.5208253654	0.2188386781	0.9999537652	-1.7889873273

0	0	0	0
2.7933974408	0.6948713406	0.9999537652	4.9527501949
1.1262768711	0.8820374785	0.9999537652	2.4214437991
-1.0971760565	0.7240935216	0.9999537652	1.0002705924
-1.3302776424	0.5715852522	0.9999537652	1.0812629779
1.0476525566	0.7661785389	0.9999537652	-1.2516077308
0	0	0	20.5862458385
-2.5838710423	0.5189022168	0.9999537652	12.7732027325
-2.1702133002	0.2400058676	0.9999537652	-1.0178965903
-1.5381976543	0.0248062011	0.9999537652	-1.2029817889
-1.8543734073	0.327231276	0.9999537652	-1.0655113791
1.9813161731	0.1878091287	0.9999537652	-1.0010652393
1.1173036187	0.3838712762	0.9999537652	-1.1383641311
1.3099269631	0.1614494217	0.9999537652	1.0211011786
-1.0412221126	0.7492152322	0.9999537652	-1.1574600751
1.0891709124	0.6429823572	0.9999537652	1.1310125259
1.1112840053	0.7419993669	0.9999537652	-1.0393822825
1.2450906471	0.7134929081	0.9999537652	-1.2573486479
-1.2208413918	0.5088838225	0.9999537652	-2.3103658431
1.1791628127	0.5775372983	0.9999537652	-1.7749289475
4.5505976667	0.5599131367	0.9999537652	-6.4329947745
-1.1927063612	0.6486131355	0.9999537652	-1.6373785342
-1.3102553506	0.2911977325	0.9999537652	-1.059205827
1.2837029658	0.2452452891	0.9999537652	1.0131599781
-1.0217868365	0.8505900797	0.9999537652	1.0014588759
1.3124976847	0.3256530702	0.9999537652	-1.0257219525
1.0178731443	0.9716553156	0.9999537652	-1.2930061459
8.064174035	0.4222958674	0.9999537652	-3.3849925178
1.2580470129	0.3730274099	0.9999537652	-1.0192383179
-1.1431736341	0.7926921836	0.9999537652	-1.2007824255
-2.1526207025	0.009106	0.9999537652	1.2158092611
4.5505976667	0.5598588711	0.9999537652	1.6293799659
-1.2033988235	0.4615907512	0.9999537652	1.1024083936
2.7933974408	0.6948713406	0.9999537652	4.7563522994
-1.1537703343	0.4412044283	0.9999537652	-1.1555825015
-1.2590448765	0.2134816883	0.9999537652	1.0937933066
0	0	0	0
1.8608089771	0.2514551438	0.9999537652	-1.1223546752
0	0	0	1.2400291296
1.569331264	0.5622617354	0.9999537652	1.5212920711
2.7933974408	0.6948713406	0.9999537652	-1.4289359629
2.7957660672	0.705375602	0.9999537652	-1.0503901552
-1.4167725316	0.1108109033	0.9999537652	-1.0253319425
-1.0664434244	0.8017966411	0.9999537652	1.0715429283
1.2252166591	0.3519119722	0.9999537652	1.4412840417
2.7957660672	0.7053779218	0.9999537652	1.64586198
-1.2632342045	0.1912828238	0.9999537652	1.2232620225
-1.0725350922	0.7075375353	0.9999537652	1.3341992583
-1.007893027	0.9634127891	0.9999537652	-1.1284028615

2.7933974408	0.6948713406	0.9999537652	1.4519459565
1.0176403375	0.9014011939	0.9999537652	1.1104674798
-1.2537904993	0.4492640358	0.9999537652	-1.1309414623
1.3212001599	0.1271431747	0.9999537652	1.1729183131
-2.4874599804	0.0985140367	0.9999537652	-1.1127358558
1.147804498	0.7481271203	0.9999537652	1.3282167267
-1.0469105869	0.8214398455	0.9999537652	1.3425712678
1.4382653442	0.0412710827	0.9999537652	-1.1858911589
-1.1180612904	0.6580019736	0.9999537652	-1.1473667065
8.0712632467	0.4224823202	0.9999537652	-14.1527912683
-1.3963189647	0.2797786231	0.9999537652	-1.0903061024
1.1029877261	0.4859699052	0.9999537652	1.2700656458
-1.276159903	0.6660435593	0.9999537652	1.2107640399
1.8760194999	0.3716979044	0.9999537652	-1.3224225408
1.302434794	0.2099218319	0.9999537652	2.2528915636
0	0	0	10.818785458
-1.5890368905	0.1561935953	0.9999537652	-1.1105438951
1.1823230351	0.4804014385	0.9999537652	1.2614173336
1.1016992685	0.5982343613	0.9999537652	1.2210499789
-1.3393078441	0.1083477069	0.9999537652	1.1315225758
9.8291821523	0.3811038069	0.9999537652	1.9220782632
-1.0140128546	0.9248168754	0.9999537652	-1.0445368591
-1.0194394823	0.9535232858	0.9999537652	-1.0005203963
11.5766527773	0.3454647247	0.9999537652	-1.8664768557
-1.1298429156	0.5422616178	0.9999537652	-1.2990942919
1.4874702342	0.0354925444	0.9999537652	1.0848162035
-1.1304640754	0.4530955876	0.9999537652	1.2689520799
-1.015023794	0.9617424478	0.9999537652	-1.071818415
-1.2518750322	0.5895440668	0.9999537652	-1.2489737278
-1.2066482828	0.1977150416	0.9999537652	-1.1795680277
-1.1680330631	0.3161034777	0.9999537652	1.1945559699
1.8649006196	0.620506401	0.9999537652	1.2040945368
1.1614348574	0.7337831187	0.9999537652	1.0130851806
1.3799976028	0.0844200459	0.9999537652	1.2311569557
-1.1615355103	0.8156655765	0.9999537652	1.3253294644
0	0	0	2.9988886918
4.5505976667	0.5598045854	0.9999537652	1.0266237419
-1.4199866313	0.009704	0.9999537652	-1.6156900919
0	0	0	6.914120153
-1.2836946567	0.3876729156	0.9999537652	1.051284174
13.0741687784	0.0107627561	0.9999537652	1.5297892232
0	0	0	2.996475627
4.5544554512	0.5691573201	0.9999537652	1.6460628488
29.1226294179	0.1908491632	0.9999537652	1.2349846686
1.0085580205	0.9719014407	0.9999537652	-1.0211049779
1.2432971499	0.4441913921	0.9999537652	1.7959624207
1.0783948971	0.6653394751	0.9999537652	-1.3531986202
1.006042197	0.9872041664	0.9999537652	-1.2786189483
-2.587194901	0.5008071247	0.9999537652	0

-1.2414788382	0.5283065092	0.9999537652	-1.1212858988
-1.0755885071	0.7603678095	0.9999537652	1.0859322026
-1.1670984831	0.8533071017	0.9999537652	-1.1427246333
1.7803338323	0.2993613603	0.9999537652	-1.3205536323
1.0479669741	0.7275551741	0.9999537652	-1.0087746951
-1.1736612603	0.7212980005	0.9999537652	-1.0416271711
-1.7093706328	0.2551301996	0.9999537652	1.0985139818
-1.1747320085	0.5771403015	0.9999537652	-1.3375701837
-1.110274191	0.6839699653	0.9999537652	1.1559901259
1.0260974091	0.9540061191	0.9999537652	1.4652110309
1.4726411111	0.015535084	0.9999537652	-1.06727129
6.3075231393	0.4870548453	0.9999537652	2.996475627
0	0	0	1.5919129963
2.8260140473	0.18078623	0.9999537652	2.8650010279
-1.3144441883	0.1866894606	0.9999537652	-1.2337913027
1.3821044394	0.7594382286	0.9999537652	-1.2450880572
-1.0790260414	0.7381417187	0.9999537652	-1.0581826463
1.5149376505	0.6512923256	0.9999537652	1.082190931
1.2827463514	0.3970537723	0.9999537652	1.3313894322
1.4765643835	0.2267293522	0.9999537652	-1.0124565469
2.7933974408	0.6948713406	0.9999537652	1.5908108123
2.2953539498	0.3499733738	0.9999537652	-1.8255939325
1.030874123	0.9031610281	0.9999537652	-1.4270185394
-1.2682498223	0.5784049935	0.9999537652	1.4132821047
1.3826309223	0.7323555863	0.9999537652	-1.2199128373
1.0537173575	0.6503272345	0.9999537652	1.1467844088
1.2984446767	0.5053935968	0.9999537652	1.1011764849
1.6921527638	0.0934683774	0.9999537652	-1.8626091451
4.5505976667	0.559750219	0.9999537652	0
1.0619127924	0.8439416306	0.9999537652	-1.1273830213
13.3441949841	0.3177049549	0.9999537652	1.6285845991
1.8436995528	0.0169463878	0.9999537652	1.4672774015
1.032470546	0.8174750124	0.9999537652	1.0444101517
-1.1557546193	0.5610505449	0.9999537652	1.0202904168
2.2828048949	0.2761585922	0.9999537652	-1.1323477624
-2.4356048515	0.0424513725	0.9999537652	1.6033706197
1.3617152416	0.8015870432	0.9999537652	0
-1.0481376142	0.7351715364	0.9999537652	1.0325833828
-1.5106136434	0.304074111	0.9999537652	-1.0589727686
2.7616190797	0.1168811574	0.9999537652	-1.1172080901
-4.8154268017	0.1712456043	0.9999537652	-1.3406866991
0	0	0	0
0	0	0	6.908559683
1.2092875995	0.7354335025	0.9999537652	1.4431959468
-1.026130559	0.9086434867	0.9999537652	1.0916197813
-1.0045671267	0.9794658065	0.9999537652	1.2874756047
-1.0658187115	0.8343305658	0.9999537652	2.6558491542
-1.1614998763	0.877263814	0.9999537652	-1.3460625967
-1.0619892888	0.8547593956	0.9999537652	1.0508833362

1.3098888734	0.0310859266	0.9999537652	1.5811779411
1.1953624891	0.8063911122	0.9999537652	1.0028935245
1.1964050001	0.3551592326	0.9999537652	1.0995627442
1.6189298858	0.007904	0.9999537652	1.1241562773
1.3954871691	0.3158928431	0.9999537652	1.2532053475
23.8818846228	0.2168921793	0.9999537652	1.0850143694
-1.0739118031	0.6291266811	0.9999537652	1.0805055683
1.2497322474	0.6610116033	0.9999537652	-1.287783599
0	0	0	2.996475627
-2.587194901	0.5007977106	0.9999537652	2.996475627
0	0	0	2.9985540909
6.3075231393	0.4870531411	0.9999537652	1.0673595208
2.4919468484	0.1073556459	0.9999537652	1.1687969718
1.6448587922	0.5768369835	0.9999537652	-1.2169768823
1.0409914384	0.8170632415	0.9999537652	-1.0784019888
-1.0319370591	0.8437590749	0.9999537652	1.2431826382
2.204523133	0.0941049187	0.9999537652	-1.0589575765
1.4619973739	0.2420335956	0.9999537652	1.5346385069
-1.1089990494	0.7578364363	0.9999537652	1.2528476294
0	0	0	4.9527501949
1.1584236488	0.7979322158	0.9999537652	-1.2550752451
1.1465825723	0.4454769103	0.9999537652	1.0269561388
1.1834575741	0.6054658473	0.9999537652	1.2470262599
-6.9736190158	0.2250321147	0.9999537652	24.5408193011
-1.084625365	0.6566362132	0.9999537652	1.2689613162
2.7933974408	0.6948713406	0.9999537652	-5.3583704783
-1.4446988347	0.3958219378	0.9999537652	-1.0374074348
6.3075231393	0.4870514369	0.9999537652	2.996475627
1.4935445839	0.0576373984	0.9999537652	-1.0849732258
1.41861628	0.0676243914	0.9999537652	1.5218081115
1.1846288744	0.3558529695	0.9999537652	1.0662368354
-1.0270975892	0.9060033342	0.9999537652	1.3078443889
-1.2897101956	0.4485967688	0.9999537652	1.0024869783
1.3607552436	0.2140643887	0.9999537652	1.4048944916
-1.2856389565	0.3258523473	0.9999537652	1.1971273964
-1.7024984242	0.1940920662	0.9999537652	-9.7565542884
-1.3050033308	0.7824877417	0.9999537652	-1.5084403566
2.7933974408	0.6948713406	0.9999537652	0
2.7933974408	0.6948713406	0.9999537652	14.7271569203
-2.4915576559	0.1318344601	0.9999537652	1.6945134434
1.6142330545	0.2135737425	0.9999537652	2.5413342366
-1.7279722953	0.0640447823	0.9999537652	1.1307280711
-1.2318853072	0.4318401572	0.9999537652	1.4703966859
-1.280251943	0.2995270005	0.9999537652	1.0181675417
-1.0168196879	0.9312519871	0.9999537652	1.5591512593
1.1695753773	0.6659147485	0.9999537652	1.3731133983
1.0711768141	0.8051215872	0.9999537652	1.1204654083
1.5144001303	0.3007443421	0.9999537652	1.2645869512
2.7933974408	0.6948713406	0.9999537652	-1.1248877899

0	0	0	0
7.4739040237	0.1008834747	0.9999537652	1.4385379898
2.7957660672	0.705382564	0.9999537652	2.996475627
1.1354804683	0.4678744308	0.9999537652	1.1849753758
1.5942609845	0.6106378183	0.9999537652	-1.414059405
-1.0257731718	0.8506330264	0.9999537652	1.018622023
1.6428845456	0.6267602266	0.9999537652	1.0586381389
1.0694480413	0.5489916955	0.9999537652	1.1008047072
1.6759772247	0.3694953536	0.9999537652	1.6298275237
-1.115698638	0.5350801453	0.9999537652	-1.0726029878
1.2014721249	0.3256934717	0.9999537652	1.2227983415
-1.2145611367	0.4411336854	0.9999537652	-1.0425735623
1.0879572536	0.6210504912	0.9999537652	1.4424972597
2.1954222178	0.2920136428	0.9999537652	1.4794741436
-1.7146188266	0.4972625953	0.9999537652	-2.1976334226
1.0410102632	0.8230558291	0.9999537652	-1.050878895
1.2221108119	0.2390877421	0.9999537652	1.4841516106
-1.0784794388	0.7736953054	0.9999537652	1.1381874685
1.4552917288	0.3968888521	0.9999537652	2.1180141103
0	0	0	0
1.4679613063	0.4730028445	0.9999537652	1.5086458818
0	0	0	8.8639046058
-1.2483357725	0.4737498379	0.9999537652	2.5023645444
1.0141721246	0.9650035863	0.9999537652	1.0777669409
1.1958263013	0.5048745871	0.9999537652	1.1374552277
0	0	0	14.7271569203
-1.030824928	0.9252365898	0.9999537652	1.6709039948
-1.2397868203	0.4499344227	0.9999537652	1.7624090547
-1.2531250398	0.5262415523	0.9999537652	1.1339915098
-1.1672564259	0.5168900938	0.9999537652	1.4812202009
-1.184478038	0.6535250826	0.9999537652	2.0485684246
1.4798117838	0.4940719948	0.9999537652	-2.7981159234
-1.4898977885	0.0286162544	0.9999537652	1.0925445727
-1.0340229824	0.8661478714	0.9999537652	1.2094198834
-1.2663866006	0.5706163133	0.9999537652	1.2987140329
1.7095073648	0.1045661774	0.9999537652	-1.0391483365
-1.1118107884	0.6112349489	0.9999537652	1.2383859598
1.5895477496	0.3448386985	0.9999537652	1.1172165601
2.7933974408	0.6948713406	0.9999537652	0
0	0	0	0
2.2227236654	0.5576122896	0.9999537652	0
2.8768322695	0.0291807398	0.9999537652	-1.2255416612
-2.5976911622	0.1594346616	0.9999537652	1.3516549241
0	0	0	2.996475627
-1.2978421103	0.2894903171	0.9999537652	1.1495916509
4.5505976667	0.559695704	0.9999537652	-5.3583704783
-1.1747706321	0.3898511878	0.9999537652	-1.1894732148
1.0758834867	0.6972059346	0.9999537652	1.340161908
2.5517619419	0.2269258556	0.9999537652	1.0272257233

-1.0303494681	0.8884525785	0.9999537652	-1.0368403734
-1.0361955495	0.8319243115	0.9999537652	1.2208108843
0	0	0	2.9985540909
1.1871740561	0.5158939589	0.9999537652	1.4654860365
2.7933974408	0.6948713406	0.9999537652	0
-1.6557166718	0.1237841544	0.9999537652	1.0612621008
-1.2583961502	0.3373695513	0.9999537652	-1.0160670569
-1.0368692941	0.9058106607	0.9999537652	2.5600627597
2.8173722589	0.3577669978	0.9999537652	3.2657224158
1.292117439	0.3895562168	0.9999537652	-1.0873510868
-1.0579833113	0.8032974766	0.9999537652	-1.316764095
1.3463065368	0.6532398128	0.9999537652	1.0299959472
-1.6551555364	0.0376752247	0.9999537652	-1.2919935564
4.5505976667	0.5596409618	0.9999537652	8.8639046058
1.0818835795	0.6677691656	0.9999537652	1.8421840903
-1.7229869003	0.4597035638	0.9999537652	-2.6858283977
1.0827232528	0.8634039461	0.9999537652	1.3893212658
0	0	0	0
1.0093397137	0.9537317867	0.9999537652	-1.0217636661
2.3315363418	0.4580941801	0.9999537652	2.4453271239
1.0868403168	0.7234174556	0.9999537652	-1.2757967611
0	0	0	-1.859280336
0	0	0	2.996475627
1.1155984297	0.472272975	0.9999537652	-1.1354989886
-1.3199211939	0.2972133226	0.9999537652	-1.078347745
3.0589901989	0.3341132581	0.9999537652	-1.3488041533
1.1572993515	0.7071639474	0.9999537652	-1.1346529096
-1.382285147	0.0787101672	0.9999537652	-1.3001581672
-1.1422376038	0.8030979454	0.9999537652	1.1632513483
-1.1267842075	0.4323671983	0.9999537652	1.3593533866
4.5544554512	0.5691553252	0.9999537652	1.0358397119
-1.1604425563	0.6587895713	0.9999537652	1.7214224153
-1.1838720538	0.3588832457	0.9999537652	-1.0654018702
1.2690034822	0.2546383057	0.9999537652	1.0968004623
1.389810412	0.2593260076	0.9999537652	1.0281511403
1.1684024639	0.4104425792	0.9999537652	-1.0478873443
2.7957660672	0.7053848865	0.9999537652	10.818785458
2.7933974408	0.6948713406	0.9999537652	6.9133480437
-15.9195842901	0.1130415848	0.9999537652	-1.6167962027
1.0327599746	0.8531335432	0.9999537652	-1.016293876
-1.194665787	0.1308004239	0.9999537652	-1.0239909659
1.1044813232	0.7122981519	0.9999537652	-1.1346351671
8.064174035	0.4225054603	0.9999537652	2.9992223802
0	0	0	0
-1.1219188376	0.4504998474	0.9999537652	1.1542105478
2.7933974408	0.6948713406	0.9999537652	0
-1.7511065395	0.2293591058	0.9999537652	-1.960470516
-1.1395341593	0.5365984201	0.9999537652	1.3024494827
-2.6355394971	0.2445686686	0.9999537652	1.4629057285

9.8205505243	0.3808413552	0.9999537652	-1.1522914685
-1.1430737555	0.6687740403	0.9999537652	1.4717456185
1.1736721022	0.2398222703	0.9999537652	1.115669794
-1.2022466431	0.5416265927	0.9999537652	-1.4135047979
-1.0895171574	0.8001718146	0.9999537652	-1.0909179045
23.8818846228	0.2168824291	0.9999537652	4.1512667769
2.7933974408	0.6948713406	0.9999537652	-2.9690502285
-1.1709096732	0.3311014877	0.9999537652	1.1314176841
1.6399788653	0.3024962241	0.9999537652	-1.2676948642
-1.2510739195	0.5808061699	0.9999537652	-1.0545601298
-1.0088863782	0.9692620277	0.9999537652	1.0019225444
-1.5602271121	0.1472201228	0.9999537652	1.0845924443
1.4675489498	0.2685988532	0.9999537652	1.4115718519
15.1008094413	0.2915509966	0.9999537652	-2.2054639418
-1.0938584153	0.7165638662	0.9999537652	-1.0145832446
-1.2943981539	0.4663898718	0.9999537652	1.1915601161
-1.0214154392	0.9189433686	0.9999537652	1.0852678531
15.0880352532	0.2955162311	0.9999537652	-1.6597328868
-1.2270945	0.2026598256	0.9999537652	1.0185318626
-1.088957945	0.6606867548	0.9999537652	1.3566278423
-2.5838710423	0.5188528616	0.9999537652	2.0319787781
-1.4800942427	0.0736094667	0.9999537652	-1.0540937473
-1.1119527526	0.4396828756	0.9999537652	-1.1525763939
-1.6371610311	0.0550098302	0.9999537652	1.1773357628
2.6918713088	0.2731993713	0.9999537652	4.1560582013
1.0923581991	0.6955364554	0.9999537652	1.4620188002
-1.5310262465	0.0520589563	0.9999537652	-1.0507591584
-1.1005046685	0.7965256409	0.9999537652	1.2106318225
-3.1152970666	0.2480298036	0.9999537652	1.5350521141
1.2344205867	0.1914179599	0.9999537652	-1.1569312494
1.167723567	0.5308868582	0.9999537652	1.1592070029
1.9272397791	0.3803191445	0.9999537652	-1.3645532167
2.3315363418	0.4567770889	0.9999537652	-1.1248877899
1.9059795869	0.473972393	0.9999537652	-1.2595285394
-1.5873039391	0.0218276461	0.9999537652	-1.1106016836
2.3698151166	0.2853082968	0.9999537652	-1.4853269632
1.6976262464	0.009697	0.9999537652	1.0353252981
1.1703747636	0.3398281469	0.9999537652	1.3329693463
1.238159275	0.5551768418	0.9999537652	1.2532777712
-1.328381226	0.1224988626	0.9999537652	1.175568096
1.3511752896	0.5440306657	0.9999537652	-1.6015263356
2.7933974408	0.6948713406	0.9999537652	0
-1.0667442002	0.8033340283	0.9999537652	1.2413603202
1.6606903217	0.4967398022	0.9999537652	1.5872604488
-1.0073036916	0.9689431355	0.9999537652	1.1771766201
-1.2419142083	0.3819559565	0.9999537652	-1.0388671897
1.1513292902	0.4312894332	0.9999537652	1.245949871
-1.0933774438	0.8895755447	0.9999537652	-1.0678306391
-1.0685380045	0.9372864963	0.9999537652	1.3057138888

1.0341361492	0.9526081129	0.9999537652	-2.0885294094
112.9073396958	0.0649398267	0.9999537652	-1.8985187394
-3.2098360912	0.2200962863	0.9999537652	-1.2278585504
3.7919141877	0.228766703	0.9999537652	1.2373582818
-1.1594970587	0.8783085982	0.9999537652	1.6466246098
-2.4044862812	0.2648188345	0.9999537652	-3.7220275797
23.8826459185	0.2176242361	0.9999537652	-1.2906233813
1.1707538763	0.6226017438	0.9999537652	1.1378819462
-1.011833847	0.9616682405	0.9999537652	-1.1017259164
1.2048227046	0.7536216831	0.9999537652	-1.8697075327
-1.325824447	0.5096375692	0.9999537652	1.2994531956
0	0	0	-4.9108225367
-1.1437325324	0.6574904458	0.9999537652	1.17009039
0	0	0	-1.8594983184
1.2321987391	0.4591366571	0.9999537652	1.4796091919
1.2971825433	0.5670604537	0.9999537652	-1.1941032272
11.5766527773	0.3454765732	0.9999537652	-2.0119450672
11.5864583426	0.3448684039	0.9999537652	1.5859005182
1.0747047708	0.8914319431	0.9999537652	-1.4266907893
1.0394940749	0.8290192082	0.9999537652	1.0148320803
-2.3428050596	0.1474409311	0.9999537652	-1.718491221
-1.3143853396	0.6624878778	0.9999537652	1.9983008022
-1.3961564483	0.0767240403	0.9999537652	-1.0914937607
1.0478408518	0.839768706	0.9999537652	-1.1628200171
-1.1728577642	0.5775496791	0.9999537652	-1.4629399511
-1.0361505987	0.9017837679	0.9999537652	-1.1315328619
-3.120470419	0.2651849967	0.9999537652	9.7116702472
1.017951084	0.9418471051	0.9999537652	-1.0020795099
-1.0081523277	0.9537078973	0.9999537652	-1.135210397
-1.2318260166	0.403425163	0.9999537652	1.0618776247
1.5827734148	0.1191105714	0.9999537652	-1.0643980444
-1.3691511916	0.766059705	0.9999537652	1.6253455238
1.5935386712	0.0117085857	0.9999537652	-1.0147370719
6.3075231393	0.4870497328	0.9999537652	1.3991459716
6.3075231393	0.4870480286	0.9999537652	15.7262184124
0	0	0	-5.3608259368
-1.8340501382	0.1459517743	0.9999537652	-1.6536525323
1.0535144626	0.8961220952	0.9999537652	-1.1542382793
-1.0043977703	0.9769109598	0.9999537652	1.0190413348
-1.8204008393	0.2668342798	0.9999537652	-2.5827393065
1.0101997367	0.9762612135	0.9999537652	-1.3356925267
-1.02842598	0.9337745143	0.9999537652	1.0303129129
-1.2792313791	0.6879427774	0.9999537652	1.3671110933
1.092247477	0.6463548061	0.9999537652	-1.0156153632
1.0938517453	0.9120019487	0.9999537652	1.4272116761
-2.6401633109	0.1672261647	0.9999537652	2.1787326106
1.1786656655	0.7285781898	0.9999537652	-1.0305682403
2.9933797697	0.1706009253	0.9999537652	1.2743776568
-1.1785641661	0.6995857337	0.9999537652	-1.3583915205

1.0420921493	0.9687392597	0.9999537652	3.3963062689
1.2723625174	0.4909201602	0.9999537652	-1.3772097969
-1.3215743889	0.4783728684	0.9999537652	-1.2469959719
9.8205505243	0.3808412115	0.9999537652	-2.2636076395
1.0291362451	0.9155229121	0.9999537652	-1.0987470635
-1.7421110952	0.3631793379	0.9999537652	-1.5395536011
-1.5347763257	0.5933331253	0.9999537652	0
1.2542903488	0.8053864093	0.9999537652	0
1.1172293767	0.9254265261	0.9999537652	4.9527501949
-1.2491488311	0.5574291752	0.9999537652	-1.1842014001
-1.126648491	0.7431041456	0.9999537652	-1.3677157312
-3.119124118	0.2337220357	0.9999537652	4.9527501949
-1.4756779277	0.644761308	0.9999537652	2.9985540909
1.5422760074	0.4459778168	0.9999537652	-1.2092759065
-1.8321015168	0.1420815492	0.9999537652	-1.2888749872
11.5766527773	0.3455074627	0.9999537652	8.8700459102
1.3265655997	0.6051157694	0.9999537652	-1.17020599
1.0668745317	0.7743134951	0.9999537652	-1.1966071074
1.7745951127	0.53884114	0.9999537652	1.3243823553
-1.1780799409	0.7732742336	0.9999537652	2.0989270987
-1.2320688955	0.5270123505	0.9999537652	-1.010720354
1.2091276469	0.1795445599	0.9999537652	-1.0853110265
-1.0882457414	0.4934019698	0.9999537652	1.2248373959
30.8757261591	0.1833375354	0.9999537652	1.4455173915
1.4672043133	0.005657	0.9999537652	-1.2704860016
20.3530564257	0.2435958385	0.9999537652	1.1417431999
0	0	0	-1.6585837467
-1.0941118227	0.5832828179	0.9999537652	-1.0703149415
1.0765250201	0.7319412868	0.9999537652	-1.5799490508
-1.1865218343	0.3772879949	0.9999537652	1.0950008656
-1.2994208831	0.4773778757	0.9999537652	-1.0940315701
2.0381335646	0.4031621962	0.9999537652	-1.762193025
20.3709255077	0.2404988473	0.9999537652	-1.272423596
2.7933974408	0.6948713406	0.9999537652	0
-1.0840364949	0.5515923564	0.9999537652	-1.4468182126
1.1620873508	0.3028411669	0.9999537652	1.183621985
1.3481381979	0.3097278649	0.9999537652	1.1128731016
1.1216193189	0.6526605599	0.9999537652	1.5932899643
2.0885615936	0.5182581982	0.9999537652	2.1115841666
1.0143250012	0.9604728342	0.9999537652	-1.1943810977
1.207641835	0.4533883125	0.9999537652	-1.4676183345
-1.1477894728	0.6750100443	0.9999537652	1.4093216918
4.528904208	0.0699050533	0.9999537652	2.5265852226
-1.1727457626	0.813025756	0.9999537652	-1.8226439191
-1.3926047059	0.1506739222	0.9999537652	1.050923712
-3.2671212482	0.1585469027	0.9999537652	-1.1025970582
1.3276900376	0.1359833624	0.9999537652	-1.0093255066
1.1655980979	0.6097461559	0.9999537652	1.6285133308
8.064174035	0.4227167532	0.9999537652	-5.3608259368

-1.2859869419	0.3146373523	0.9999537652	1.0608129519
-2.10905182	0.4746266164	0.9999537652	-1.1627130392
1.7732441546	0.5688915525	0.9999537652	-27.7543447711
1.3039771927	0.649634093	0.9999537652	-1.4333628253
0	0	0	83.9572092248
1.1206202914	0.6547322374	0.9999537652	1.11154671
1.6018473574	0.6931155549	0.9999537652	-9.7542681481
1.1783339214	0.3175377245	0.9999537652	-1.171675518
-1.189138865	0.4224313395	0.9999537652	1.1288834106
1.0335374958	0.8379910159	0.9999537652	1.0963315098
-1.0089574802	0.9821922527	0.9999537652	-1.0692080637
-1.4955709411	0.0962424356	0.9999537652	-1.4392652199
-1.2555610833	0.5051807848	0.9999537652	-1.065818815
0	0	0	4.9527501949
1.3133995489	0.0470352353	0.9999537652	1.0467710429
1.0558685248	0.8227057087	0.9999537652	-1.2838626807
6.3075231393	0.4870463245	0.9999537652	1.569400479
1.8471118167	0.5844285823	0.9999537652	1.1338167742
-1.0751526887	0.7380383325	0.9999537652	-1.0425585607
0	0	0	2.996475627
1.3294970916	0.0735333983	0.9999537652	-1.1636290812
-5.5280856191	0.0261108481	0.9999537652	-2.7485376566
-1.0468430487	0.9362589568	0.9999537652	-1.0013269045
0	0	0	2.996475627
2.7957660672	0.7053872099	0.9999537652	0
1.051061108	0.804894	0.9999537652	-1.0390227343
-1.1782328014	0.3651811946	0.9999537652	-1.0338955768
1.1015508841	0.4355816496	0.9999537652	-1.1880585484
0	0	0	-3.3849925178
0	0	0	9.7116702472
-1.3917747917	0.6964805593	0.9999537652	6.1630209487
-1.3425706498	0.025780769	0.9999537652	1.1330308067
0	0	0	4.9527501949
1.0790848001	0.6737138597	0.9999537652	1.8534401227
1.0359384084	0.773673456	0.9999537652	-1.019125305
1.3826309223	0.7327639894	0.9999537652	-2.7873245155
1.0647461547	0.5992467491	0.9999537652	-1.2752052612
25.6380955075	0.2075470826	0.9999537652	1.9281410065
9.8291821523	0.3810915107	0.9999537652	-1.5098301937
-2.5861438062	0.4446475004	0.9999537652	1.2400291296
1.5089100509	0.4338951187	0.9999537652	1.1864416124
-1.9040183652	0.3839748904	0.9999537652	-1.0017830005
-1.3327742757	0.0387306432	0.9999537652	-1.3467134356
-1.0341074793	0.853337016	0.9999537652	-1.3965668626
1.0003591822	0.997883287	0.9999537652	1.2600550135
3.0794952153	0.1796259702	0.9999537652	-2.0354068982
1.1345301145	0.844341838	0.9999537652	-1.0496824516
1.1255502721	0.5301552925	0.9999537652	1.0852503975
-1.2300867032	0.3726619362	0.9999537652	-1.2389420537

1.0788623646	0.8794621638	0.9999537652	2.2980242085
1.3394887056	0.1102020151	0.9999537652	1.1391669937
-1.1415884534	0.7675508606	0.9999537652	-1.1328333233
1.0794225454	0.5672117116	0.9999537652	1.0064095132
1.6522734637	0.057183709	0.9999537652	1.0858691071
1.084383624	0.5121245755	0.9999537652	1.2692455646
-1.4153881263	0.1975966584	0.9999537652	1.0222409221
-1.0218756188	0.8556216969	0.9999537652	1.0039705875
11.5766527773	0.3455384703	0.9999537652	18.6336779883
1.0390043702	0.7710857835	0.9999537652	-1.0031324896
-1.0628412383	0.7264332049	0.9999537652	-1.048172647
1.0166008243	0.9282972033	0.9999537652	1.0325175065
-1.5855465301	0.3180011641	0.9999537652	1.1942361679
1.0409391126	0.768374111	0.9999537652	1.0453074768
1.579545244	0.2498326164	0.9999537652	1.0564115018
-2.5861438062	0.4501841952	0.9999537652	2.996475627
-1.0563241977	0.7591747823	0.9999537652	-1.155524654
0	0	0	2.9985540909
-1.0573600669	0.6662488922	0.9999537652	-1.1510141561
2.7957660672	0.7053895343	0.9999537652	-2.5807221064
-1.1107000559	0.4644028316	0.9999537652	-1.2892288294
1.3443356233	0.346104978	0.9999537652	1.105497907
-1.004909125	0.9835643911	0.9999537652	-1.3076347655
1.3484562641	0.2026024793	0.9999537652	-1.0983995869
1.8364642969	0.2597307992	0.9999537652	-1.452826495
1.2160518684	0.1192874401	0.9999537652	1.0476557467
1.2138738476	0.3051518693	0.9999537652	-1.338622924
-1.7812510556	0.0830064742	0.9999537652	-1.1225022772
2.7933974408	0.6948713406	0.9999537652	-3.7629410475
1.342790253	0.6796736288	0.9999537652	-1.4216390073
1.4284792189	0.0315027693	0.9999537652	-1.3989947955
1.2991846931	0.6306880951	0.9999537652	-1.0879012925
1.9981836368	0.1976536902	0.9999537652	1.0508398566
-3.1179374904	0.257589356	0.9999537652	1.0665314776
1.2618189434	0.3041374506	0.9999537652	1.2072502091
1.0970898789	0.5180068888	0.9999537652	-1.1744185741
1.1551208739	0.3288301413	0.9999537652	1.1924035104
-1.5367922295	0.6209588228	0.9999537652	1.3364870474
1.1946467681	0.680873746	0.9999537652	-1.2001404617
1.7475447163	0.468448536	0.9999537652	-1.5028658085
-1.1197848744	0.6104874384	0.9999537652	-1.1217392901
1.3390279474	0.4319407938	0.9999537652	-1.323104021
-1.0577719468	0.890274283	0.9999537652	-1.1567552283
-1.0272066817	0.8812683978	0.9999537652	-1.3622086292
-1.2636756559	0.3907945023	0.9999537652	-1.1097801682
1.7343571244	0.4255056415	0.9999537652	-1.1299049042
-1.2701996041	0.2045269255	0.9999537652	-1.4568672711
1.0352042491	0.8553562721	0.9999537652	1.0187834857
1.423187655	0.2793290155	0.9999537652	-1.1708696749

-1.1205933125	0.5379368142	0.9999537652	-1.0318918388
-1.0018218711	0.9929438781	0.9999537652	1.0236265405
-1.0474194693	0.7766556501	0.9999537652	-1.2597779457
-1.1136379061	0.4812646927	0.9999537652	1.0414318029
-1.1027274396	0.6936229977	0.9999537652	-1.0119300714
0	0	0	-2.0425384369
-1.0517744207	0.8718434304	0.9999537652	1.6527729294
-1.0887237807	0.6593208013	0.9999537652	-1.0007070338
-1.0787425903	0.6930038455	0.9999537652	1.0947865009
1.0206564818	0.8822022119	0.9999537652	-1.0244041443
1.0853228233	0.7939559592	0.9999537652	-1.1100074999
-1.1766524626	0.3728594536	0.9999537652	1.3536092118
-1.2805460531	0.7218219266	0.9999537652	1.1535823687
1.1156383778	0.4293971664	0.9999537652	-1.185562208
1.1289659924	0.8076712967	0.9999537652	-1.0676368212
-1.1066845452	0.6769625052	0.9999537652	-1.3386629358
-1.0730234155	0.7481589473	0.9999537652	1.0175085635
-1.6659978101	0.6171778111	0.9999537652	-1.3272544584
-1.2658245094	0.5610923058	0.9999537652	1.1141524631
-1.0649744804	0.7913926884	0.9999537652	-1.5631736917
1.1928884055	0.7919968788	0.9999537652	1.2554401713
1.0048203054	0.986131674	0.9999537652	-1.7854834287
-1.277130025	0.0613503549	0.9999537652	-1.1772301363
-1.5023225118	0.48592112	0.9999537652	1.402380423
2.5592477755	0.2796920273	0.9999537652	-1.4581849019
1.011238394	0.9599371787	0.9999537652	-1.1343988689
1.2131456072	0.678409366	0.9999537652	-1.2917506888
-1.1473356941	0.3283676764	0.9999537652	1.163987552
-1.2525738931	0.6840686714	0.9999537652	46.0227597074
13.3441949841	0.3177140183	0.9999537652	-1.9672204454
-1.1026217154	0.8826320874	0.9999537652	-1.0588964999
1.2516202231	0.8309576897	0.9999537652	-3.6575338339
-1.1354652942	0.3872190737	0.9999537652	1.1339114455
1.4883588481	0.3864822928	0.9999537652	-1.4035149583
-1.0094207738	0.9608138064	0.9999537652	-1.0804410877
1.0330887906	0.7934589992	0.9999537652	1.0072904498
-1.4146200267	0.2632389392	0.9999537652	1.1059138662
1.662563624	0.1222070965	0.9999537652	1.122450923
4.5505976667	0.5595859049	0.9999537652	-4.9102474202
0	0	0	6.914120153
0	0	0	1.2400291296
1.0169427395	0.979217399	0.9999537652	-1.6992246588
-2.0880518127	0.1338014647	0.9999537652	1.9068523787
3.8622037496	0.6197859473	0.9999537652	0
2.7933974408	0.6948713406	0.9999537652	0
2.0171694328	0.3557744633	0.9999537652	3.3948931521
13.3324809638	0.3187265067	0.9999537652	0
-2.5861438062	0.4554367588	0.9999537652	-1.859280336
1.1706961862	0.8143413385	0.9999537652	2.6430925766

0	0	0	0
1.1346775939	0.4071429061	0.9999537652	1.0740490608
1.1791283763	0.8306029758	0.9999537652	-1.8657462831
-1.0103033074	0.955704898	0.9999537652	1.2490703609
1.1612873439	0.7871092903	0.9999537652	-2.0479626835
-1.0184855781	0.9218813	0.9999537652	-1.3112054494
6.3128691894	0.4820668695	0.9999537652	2.9985540909
1.0963928122	0.8798714834	0.9999537652	1.1516208978
0	0	0	4.3957515041
-6.9764533263	0.2590282316	0.9999537652	-1.3598304575
1.3125770724	0.0462317594	0.9999537652	-1.443872876
0	0	0	2.9985540909
1.2117843243	0.4198468967	0.9999537652	1.0756332046
-1.2378656322	0.6962665807	0.9999537652	-1.3364717659
1.8447991669	0.6001207281	0.9999537652	8.8700459102
1.3747331495	0.1762744558	0.9999537652	1.0901315896
1.2382880807	0.2810921441	0.9999537652	-1.2362746027
-1.0944189448	0.6562795995	0.9999537652	-1.1160305038
1.1245065018	0.4359400628	0.9999537652	-1.0164026202
-1.0021713262	0.9951149022	0.9999537652	-1.1564485764
4.5505976667	0.5595304294	0.9999537652	8.8700459102
2.7933974408	0.6948713406	0.9999537652	1.4541197941
0	0	0	0
1.0057156055	0.9822925788	0.9999537652	1.3023085789
-1.0112984464	0.9875882006	0.9999537652	-2.0479626835
-1.1148499669	0.8711329204	0.9999537652	1.3444140054
-1.4229953559	0.2073983311	0.9999537652	-1.4021527887
2.7933974408	0.6948713406	0.9999537652	1.2601780493
-1.2436857194	0.2052434452	0.9999537652	-1.1391719477
4.5505976667	0.5594744114	0.9999537652	6.9133480437
11.5766527773	0.3455460644	0.9999537652	1.3991459716
1.0318399266	0.8402705031	0.9999537652	1.0579212841
0	0	0	2.996475627
-1.059627023	0.7093473883	0.9999537652	-1.0000316058
1.0500736843	0.858235831	0.9999537652	1.0803778267
13.3324809638	0.3187058729	0.9999537652	-1.9381826991
1.1371033445	0.4406018367	0.9999537652	-1.0679430452
1.6152476451	0.4916310712	0.9999537652	-1.2158215885
-1.5587785782	0.0871970404	0.9999537652	-1.5496897635
-1.2228034435	0.7432463395	0.9999537652	-1.4649359741
0	0	0	6.908559683
1.0052680514	0.9693836211	0.9999537652	-1.1977998706
1.4500104838	0.0374818495	0.9999537652	1.0783949697
1.0119014143	0.9521912004	0.9999537652	1.1497717777
-1.2644619883	0.6569145987	0.9999537652	2.4392766943
8.064174035	0.4229297384	0.9999537652	2.0329095384
1.7396072511	0.0807668753	0.9999537652	-1.0058986185
-1.4078332045	0.025262738	0.9999537652	-1.1012156767
-9.7926713908	0.0108577287	0.9999537652	2.0326275696

1.1504373392	0.5800249174	0.9999537652	1.0724442482
-2.3844120755	0.1843655285	0.9999537652	-1.4904107309
2.5734927579	0.4062055419	0.9999537652	8.8639046058
2.7957660672	0.7053918596	0.9999537652	0
1.4368859147	0.4772524049	0.9999537652	1.4141517698
16.9336769091	0.005624	0.9999537652	1.1633733544
3.1354156389	0.0431357447	0.9999537652	-2.8421015892
1.0545963744	0.7689896114	0.9999537652	-1.0642290523
-1.3018941027	0.386927218	0.9999537652	-1.1202970205
8.064174035	0.4232543184	0.9999537652	2.1856030494
1.1168392827	0.9281787834	0.9999537652	-1.964996944
2.7957660672	0.7053941858	0.9999537652	-1.1402057619
-1.0973129919	0.6668285208	0.9999537652	1.1880946258
-1.1018711685	0.7155771109	0.9999537652	1.3313386303
-3.8687254972	0.006959	0.9999537652	1.5218211422
1.425927401	0.0663130207	0.9999537652	1.2070870155
0	0	0	2.996475627
1.0284858559	0.8360404789	0.9999537652	-1.058857779
1.0561456518	0.7056800171	0.9999537652	1.0046529216
-1.1337240081	0.3712719252	0.9999537652	1.0021269717
-1.0800277357	0.6681371988	0.9999537652	-1.1256368387
0	0	0	1.591370391
1.0559250984	0.7956381467	0.9999537652	1.1451393879
-1.0490628311	0.8067449726	0.9999537652	1.3898089786
1.0226610922	0.9126645797	0.9999537652	-1.0314978039
2.087766184	0.5276159126	0.9999537652	1.6092914501
1.1963486671	0.2976563017	0.9999537652	1.7685341113
-1.0687311449	0.6470331988	0.9999537652	-1.133353719
-1.2490618236	0.2010915935	0.9999537652	1.0185248653
-1.668044468	0.613200015	0.9999537652	-1.0927947264
1.1803348591	0.2352286547	0.9999537652	1.442199925
-1.2036744258	0.3598586213	0.9999537652	1.1262490607
0	0	0	1.6443474089
-1.0357934313	0.8007612818	0.9999537652	1.2378541571
1.3007682274	0.6023285611	0.9999537652	1.3580474398
1.1929187497	0.7982408975	0.9999537652	-1.2509190994
1.3410567367	0.6064094859	0.9999537652	1.5897129105
-1.6410854145	0.1823609159	0.9999537652	-1.3437600795
1.0409285013	0.9282642155	0.9999537652	-1.0356815745
29.1965962496	0.1897407744	0.9999537652	-2.2219274269
1.2358806825	0.7447341246	0.9999537652	1.739826308
1.2513710769	0.7428089476	0.9999537652	-1.1301634184
1.3034912742	0.5835830106	0.9999537652	1.079071812
-1.1270718913	0.5500786548	0.9999537652	1.029856939
1.1267577468	0.8244880558	0.9999537652	1.2162076516
-1.497969427	0.2137848981	0.9999537652	-1.3210047164
-1.2891465825	0.5834743189	0.9999537652	1.7565333715
0	0	0	2.9985540909
0	0	0	2.9985540909

1.1049592869	0.8324870811	0.9999537652	1.3886271106
1.067189349	0.7121315897	0.9999537652	-1.2688692667
-1.1081466031	0.4300357093	0.9999537652	1.0396543764
0	0	0	0
-1.5194868928	0.3647504582	0.9999537652	-1.0867326727
-1.1804987656	0.3811697061	0.9999537652	-1.0812785503
1.2493564472	0.5957487921	0.9999537652	1.7142160183
1.8455489546	0.5921421893	0.9999537652	-1.7280477862
1.261593916	0.3665502443	0.9999537652	1.164525062
1.7935303623	0.0117736816	0.9999537652	1.2667641637
0	0	0	0
3.7646374853	0.0228507284	0.9999537652	1.3884398102
-1.0239344554	0.8611718804	0.9999537652	1.0477135298
0	0	0	-1.8582093966
0	0	0	-2.9704130837
-1.3561169077	0.1909000903	0.9999537652	1.1625223099
-1.45316555	0.2230144151	0.9999537652	-1.0550896307
1.0405262485	0.864440244	0.9999537652	1.1744377388
-3.1152970666	0.2481088807	0.9999537652	-2.126356481
-1.3596094348	0.2510083855	0.9999537652	-1.2121928516
-1.3211693577	0.2254660564	0.9999537652	-1.0124749309
-1.1106440131	0.403672345	0.9999537652	1.2363968483
1.0152238342	0.930706727	0.9999537652	1.074633242
1.7202185701	0.0267598672	0.9999537652	-1.0236147659
1.6032449232	0.6828998797	0.9999537652	2.158131531
1.2815614752	0.2297176104	0.9999537652	1.0666457282
4.5505976667	0.5594177039	0.9999537652	-4.9096724685
2.7933974408	0.6948713406	0.9999537652	21.234349681
-1.174138964	0.7443801568	0.9999537652	-1.1739024285
0	0	0	4.9527501949
1.6327986643	0.2113466903	0.9999537652	-1.4967026339
-1.1768475501	0.3861180789	0.9999537652	-1.2480001491
1.4573148427	0.0119265638	0.9999537652	1.016715098
1.2829850977	0.2081285342	0.9999537652	1.1000268711
-1.0928106607	0.77875253	0.9999537652	1.2572757741
1.6024984014	0.6863389077	0.9999537652	14.7271569203
2.7933974408	0.6948713406	0.9999537652	0
-1.1172314123	0.4462109268	0.9999537652	-1.0008887706
-1.0422849782	0.8476450124	0.9999537652	1.4078357959
-1.0798572356	0.6440905994	0.9999537652	1.0860257805
-1.0670711847	0.6100400886	0.9999537652	-1.3875733375
2.2993099004	0.3149778893	0.9999537652	1.9620527532
-1.1244190149	0.4391001937	0.9999537652	1.1887751235
-1.4230508496	0.2104815481	0.9999537652	1.1437272898
2.0894806265	0.5159631706	0.9999537652	-1.2906260171
-1.2636213218	0.4424650338	0.9999537652	-1.1569063145
1.5585739867	0.1947005617	0.9999537652	-1.0584967635
1.2941502612	0.090144737	0.9999537652	1.1751665493
4.5505976667	0.5593601265	0.9999537652	1.6439796197

6.3075231393	0.4870446204	0.9999537652	-1.1541540815
-1.0422566822	0.7946377073	0.9999537652	1.0659886612
13.3324809638	0.3186935307	0.9999537652	1.1187149872
1.149325511	0.7880674963	0.9999537652	1.2114560929
1.408607799	0.1251693305	0.9999537652	-1.1848550849
-1.1366402845	0.7689165733	0.9999537652	1.2224788667
-1.2070536782	0.1931908174	0.9999537652	-1.0636135027
-2.4674846869	0.2524478057	0.9999537652	3.7626699959
1.0538545495	0.8564552798	0.9999537652	-1.0548883304
-12.9849514806	0.1056623725	0.9999537652	8.8639046058
-6.9762758667	0.2487223524	0.9999537652	1.2739728139
-1.1550077361	0.3809793112	0.9999537652	-1.3102806234
3.1856931818	4.95E-05	0.4532897791	1.0340636297
1.1208685561	0.3809518414	0.9999537652	1.3430540455
1.6598659682	0.4911540959	0.9999537652	-1.5368923423
-2.4938987347	0.1900181964	0.9999537652	-1.3720581997
1.3005599152	0.1114667188	0.9999537652	1.2568193482
-1.21945187	0.3471125125	0.9999537652	-1.4808978312
1.0526092736	0.7268632801	0.9999537652	1.507783976
-1.2750105366	0.190210922	0.9999537652	-1.1727837651
1.0439653084	0.8158204993	0.9999537652	1.2720516154
1.006476955	0.9710736628	0.9999537652	-1.1863717922
-1.3801390768	0.4196278878	0.9999537652	-1.2981728992
1.1355300996	0.4037350801	0.9999537652	1.1581439775
2.7933974408	0.6948713406	0.9999537652	0
-1.261175982	0.2460875047	0.9999537652	2.0867795204
-1.0638676528	0.7391276928	0.9999537652	1.1085906144
1.2441481942	0.4908510853	0.9999537652	-1.0443582197
2.5837563296	0.0838860998	0.9999537652	2.2373565916
1.1182324784	0.75942276	0.9999537652	1.3657420951
1.0810172347	0.6884191059	0.9999537652	1.0196237367
2.136620456	0.0284153628	0.9999537652	2.3075221479
2.7933974408	0.6948713406	0.9999537652	0
-1.1296634247	0.8370702167	0.9999537652	1.0152345338
-1.0145556489	0.9245938269	0.9999537652	1.2370613877
-1.0851115432	0.5857613118	0.9999537652	1.1250065588
1.3297997404	0.1295369639	0.9999537652	1.4866181668
1.0815398013	0.6978904345	0.9999537652	1.236723965
0	0	0	2.9988886918
-1.1258242841	0.5590668938	0.9999537652	1.1936991856
3.0057842914	0.1568636952	0.9999537652	1.6440299384
2.7933974408	0.6948713406	0.9999537652	-4.9102474202
-1.1762225355	0.4981442106	0.9999537652	1.1721607493
2.225750868	0.0701005595	0.9999537652	-1.0614997003
1.2216323643	0.3871081962	0.9999537652	1.2126315391
-1.0185075916	0.9083503427	0.9999537652	1.0625430295
1.0792890732	0.6656008124	0.9999537652	-1.0664860119
-1.0186712293	0.9079175572	0.9999537652	1.2788524217
1.4469190838	0.5669759403	0.9999537652	-1.620066493

-1.1107856505	0.8176201251	0.9999537652	-1.5197168271
-1.1827407426	0.5091782206	0.9999537652	-1.2084761666
-1.1076099558	0.6739548388	0.9999537652	1.1596928614
9.8205505243	0.3808410679	0.9999537652	1.9416545697
2.7933974408	0.6948713406	0.9999537652	-5.3583704783
1.5407610335	0.0631060003	0.9999537652	-1.302835756
2.7933974408	0.6948713406	0.9999537652	-5.3608259368
-1.5875118025	0.7206459959	0.9999537652	1.0718708003
1.0306349546	0.8576504014	0.9999537652	1.1029027173
13.3324809638	0.3186812994	0.9999537652	-2.2244958821
-1.0419927409	0.858571396	0.9999537652	1.125590994
-1.0651429566	0.7125848956	0.9999537652	-1.1954732047
-12.9800088947	0.1123772008	0.9999537652	6.9133480437
20.3530564257	0.2435949783	0.9999537652	-1.3598304575
-1.0556179016	0.6795225303	0.9999537652	1.1625581206
1.0781105086	0.7215327017	0.9999537652	-1.3654618481
8.064174035	0.4235795291	0.9999537652	2.996475627
4.5505976667	0.559241397	0.9999537652	8.8639046058
1.0243225506	0.8677372128	0.9999537652	-1.8532649194
-1.1025173972	0.5059909083	0.9999537652	-1.3520766929
0	0	0	2.996475627
-1.5029932233	0.0239355884	0.9999537652	-1.1768184713
-1.0853641679	0.5351329194	0.9999537652	1.0232030332
9.8205505243	0.3808409242	0.9999537652	-1.4828709862
-1.2157703387	0.3179375665	0.9999537652	-1.2630399486
1.0264063574	0.9098886297	0.9999537652	1.0825252848
1.2396217867	0.3467601674	0.9999537652	-1.2436261599
1.8878043233	0.0431065753	0.9999537652	1.33978059
-1.504767939	0.2675219121	0.9999537652	-1.4267790697
1.7115283675	0.0264729974	0.9999537652	1.0794498339
1.8455489546	0.5921541081	0.9999537652	1.0399956057
18.5983228161	0.2580273756	0.9999537652	-1.7010011802
1.0748166427	0.6332530978	0.9999537652	-1.2423043616
4.152684212	0.0471604651	0.9999537652	2.13786569
-1.0795123299	0.8266873523	0.9999537652	-1.1899547796
1.1993246541	0.3273008897	0.9999537652	1.0794365166
1.500470071	0.1706460039	0.9999537652	-1.0186073977
1.0860005437	0.6382465296	0.9999537652	1.0319343329
2.1792111666	0.1674743493	0.9999537652	-1.1714044472
1.0596729611	0.6561800966	0.9999537652	1.2326305898
1.6449006282	0.5760768301	0.9999537652	-1.0742189171
0	0	0	2.996475627
0	0	0	9.7116702472
6.3075231393	0.4870429164	0.9999537652	-4.3143133277
-1.0681823752	0.7736434411	0.9999537652	1.4690948575
-1.1624994961	0.4300467313	0.9999537652	1.1507467722
1.257679243	0.2079784935	0.9999537652	1.0612141566
13.3324809638	0.3186715236	0.9999537652	0
-2.4710767924	0.5444392205	0.9999537652	-2.0465436769

1.1933821756	0.817979771	0.9999537652	-1.922437777
-1.1739623639	0.4151120079	0.9999537652	1.0203718815
1.2020281895	0.3980168969	0.9999537652	-1.2217034509
1.5440317143	0.421825462	0.9999537652	-1.2662240385
-1.1295275404	0.3670295432	0.9999537652	-1.1925801034
-1.5875118025	0.7205638289	0.9999537652	-1.6091899336
-1.9064656256	0.3786759995	0.9999537652	1.1948314323
-1.5875118025	0.7204816854	0.9999537652	-1.3220707163
-1.0465704874	0.8798522357	0.9999537652	1.1494551386
1.1382423994	0.6960768051	0.9999537652	1.3029181405
1.0251145015	0.8359169443	0.9999537652	1.040202433
1.0171245116	0.9292754456	0.9999537652	1.1101676875
1.4194152128	0.1391069198	0.9999537652	1.1650664198
-6.9736190158	0.2250321147	0.9999537652	-2.0479626835
-1.0881151135	0.6238823682	0.9999537652	-1.0774266526
1.0680000096	0.8358806912	0.9999537652	-1.0891785648
-1.735240127	0.4420027122	0.9999537652	1.7042814026
-104.7921208189	0.0106389139	0.9999537652	1.6515976785
1.1949787423	0.3151964271	0.9999537652	-1.3243542427
-1.1438485333	0.5208537995	0.9999537652	-1.7926358938
-3.1427187605	0.0438436144	0.9999537652	1.8561987564
1.1233786522	0.4211661078	0.9999537652	1.0298615413
1.0168678129	0.9416708699	0.9999537652	1.140742452
1.0475297386	0.7155264822	0.9999537652	-1.3853640178
-3.4796602944	0.1973678641	0.9999537652	-1.8594983184
1.846361394	0.588821929	0.9999537652	-1.859280336
15.1012892518	0.291421393	0.9999537652	1.7969381251
1.0420664329	0.8359187983	0.9999537652	-1.072040457
4.5505976667	0.5591795733	0.9999537652	-2.7915177751
1.057806085	0.8505958429	0.9999537652	-1.059822702
1.0862892331	0.697961073	0.9999537652	1.3508032289
1.2717623111	0.6271412318	0.9999537652	-1.0506119921
-1.0730744975	0.6857444107	0.9999537652	1.6237898951
0	0	0	6.908559683
0	0	0	8.8639046058
2.7933974408	0.6948713406	0.9999537652	0
5.7340522642	0.0946843495	0.9999537652	1.0674845818
0	0	0	4.9527501949
4.2587446027	0.0814831998	0.9999537652	-1.1200150326
-1.0761109553	0.7246465142	0.9999537652	-1.1839516159
-1.1953172574	0.562616778	0.9999537652	1.2294152116
4.5505976667	0.559115459	0.9999537652	-5.3608259368
2.0743013645	0.2947599427	0.9999537652	1.1151016283
-1.2217923647	0.3869602341	0.9999537652	1.0676160259
1.3314867457	0.5355407435	0.9999537652	1.1139889608
-1.0831636347	0.7915740255	0.9999537652	1.2039835117
1.1847101141	0.5213245331	0.9999537652	1.538629966
-1.0122937504	0.9520723841	0.9999537652	1.0645409019
-1.2397204368	0.2746178139	0.9999537652	-1.0019502581

-1.000750819	0.9954228661	0.9999537652	1.1549713914
-1.319714631	0.0924761322	0.9999537652	1.1439418427
1.0961472636	0.6651310615	0.9999537652	1.3363538012
1.1975410153	0.1902726246	0.9999537652	1.0359424051
-1.0079213105	0.9894727047	0.9999537652	1.0462989978
-1.0857238964	0.7296807432	0.9999537652	-1.243849799
1.1137677492	0.5392602992	0.9999537652	1.269742836
1.2150139978	0.5360984406	0.9999537652	1.1634117828
-1.25015895	0.2482011699	0.9999537652	1.4038076113
-1.1457847193	0.9109354302	0.9999537652	-1.3185744328
-1.1514399217	0.623922185	0.9999537652	-1.0136007675
-1.70011302	0.3157002504	0.9999537652	-1.2836563634
2.7933974408	0.6948713406	0.9999537652	0
1.1586479412	0.4254666531	0.9999537652	1.3205928426
4.6434843517	0.076244974	0.9999537652	1.2147889987
-1.0674124218	0.6010678542	0.9999537652	-1.0134345348
-1.5045637226	0.1532677429	0.9999537652	1.193086555
2.7933974408	0.6948713406	0.9999537652	1.5327000992
0	0	0	-1.1241083699
-1.4831540626	0.1744514667	0.9999537652	-1.1681097405
0	0	0	20.5862458385
-1.1284624793	0.484233811	0.9999537652	-1.0725899598
-1.1457555741	0.9107270194	0.9999537652	-2.6872709087
1.0911651902	0.7123611077	0.9999537652	1.353574307
2.7933974408	0.6948713406	0.9999537652	0
1.0356243286	0.7938883712	0.9999537652	1.1702757359
1.390432632	0.1671388614	0.9999537652	1.1991832054
-1.0158067418	0.9145872113	0.9999537652	1.3397861551
-1.0630360144	0.6860847327	0.9999537652	1.0209415656
1.8448460942	0.5998187605	0.9999537652	2.0331135065
0	0	0	4.9561842702
1.0264895578	0.9140608961	0.9999537652	-1.1111104911
1.2927487333	0.7024828618	0.9999537652	1.5215976716
-1.7155763775	0.5431827862	0.9999537652	2.0330936321
1.1907640094	0.6155005824	0.9999537652	1.0770235919
-1.027307705	0.9043358628	0.9999537652	1.0845826962
1.0399448041	0.8406668766	0.9999537652	-1.0317603866
-1.1285144383	0.498439232	0.9999537652	1.3691497691
2.087766184	0.5275423011	0.9999537652	24.5069156959
1.1489836539	0.7747280855	0.9999537652	-1.1207946905
1.0580357993	0.6995053861	0.9999537652	1.2495884702
1.0903297719	0.6523250313	0.9999537652	1.2931686431
8.0710074692	0.4203428021	0.9999537652	1.1669991985
1.0293890928	0.8864893973	0.9999537652	-1.0162846839
1.0194166936	0.9328802858	0.9999537652	1.6459334029
-1.1348424908	0.6087669905	0.9999537652	1.1915928618
1.1011815539	0.4892771649	0.9999537652	1.027994439
1.0295433344	0.8774264894	0.9999537652	-1.0132431938
1.1250986691	0.7314635333	0.9999537652	1.0325259814

-1.3165740693	0.3272788854	0.9999537652	1.1796960653
-1.2235251948	0.1296392655	0.9999537652	-1.2809927463
1.1583474915	0.6457917244	0.9999537652	1.2583079248
1.1392945479	0.3460640593	0.9999537652	1.6824702242
2.3338728054	0.0155652264	0.9999537652	1.0919614762
1.2139465006	0.5893642353	0.9999537652	-1.5555619072
-1.0127924418	0.9226695326	0.9999537652	-1.1659442745
1.696766011	0.0202283509	0.9999537652	1.1233584612
1.0476006043	0.6779968627	0.9999537652	1.1613988312
1.2378168121	0.2480250112	0.9999537652	-1.0225512667
-1.0041580036	0.9820951024	0.9999537652	1.0019678055
-1.2382693302	0.298825916	0.9999537652	-1.1299082346
1.0880616693	0.7911812044	0.9999537652	-1.0163915095
-1.0204329064	0.9281675221	0.9999537652	-1.0052037536
-1.1706813018	0.3686538719	0.9999537652	-1.2386701626
1.0353934819	0.9655076958	0.9999537652	-1.4671686592
2.7933974408	0.6948713406	0.9999537652	-5.3608259368
-1.5019578083	0.0820173058	0.9999537652	-1.6832006208
-1.3871019527	0.2168771481	0.9999537652	-1.1781799406
2.7933974408	0.6948713406	0.9999537652	0
0	0	0	2.996475627
2.7933974408	0.6948713406	0.9999537652	12.7820459093
1.1599020496	0.2218310044	0.9999537652	1.1032553757
1.0427352324	0.8159871845	0.9999537652	-1.2850605774
1.0018182841	0.9928716262	0.9999537652	-1.3683431323
1.055403819	0.7320478228	0.9999537652	1.0621316994
-1.5875118025	0.720509259	0.9999537652	-1.1250196696
-1.8295228655	0.0497775693	0.9999537652	-1.2126524817
-1.5307492972	0.4898296704	0.9999537652	1.3825160561
2.7933974408	0.6948713406	0.9999537652	-2.0075801108
1.0080763077	0.9615534062	0.9999537652	-1.741928766
-1.1453192235	0.9088481354	0.9999537652	-1.2441497291
18.9318466825	0.2781668755	0.9999537652	-3.3853891979
1.062468148	0.6761067646	0.9999537652	1.1038681736
-1.1680566799	0.8561027567	0.9999537652	3.2952516161
1.0007513483	0.9959043742	0.9999537652	-1.0920277825
1.7468796346	0.487334085	0.9999537652	1.3291630813
-1.0837115097	0.6534274825	0.9999537652	1.0536154066
-1.2008922436	0.6849233221	0.9999537652	1.4872367092
1.3221510186	0.2326967356	0.9999537652	1.2153938578
2.7957660672	0.7053965129	0.9999537652	-9.7542681481
-1.0586237724	0.7165771191	0.9999537652	1.0967139971
1.4925902484	0.0365036776	0.9999537652	1.3399624403
-1.3326607281	0.1716659141	0.9999537652	1.0404692053
-1.3664432468	0.2227248695	0.9999537652	1.0944410148
-1.0103424929	0.9513153457	0.9999537652	1.0529068814
-1.0319676128	0.9360625401	0.9999537652	-1.1621647533
-1.1865280291	0.491946888	0.9999537652	1.020720748
0	0	0	2.9985540909

1.180155548	0.5892672801	0.9999537652	-1.2842682395
-1.0590676079	0.9086161652	0.9999537652	-1.2107375927
-1.1155695121	0.6239147974	0.9999537652	1.0036041841
1.189950996	0.3773186703	0.9999537652	1.0339615657
2.7933974408	0.6948713406	0.9999537652	0
-1.1378228505	0.5203688277	0.9999537652	1.0597303391
1.1113891643	0.6307656027	0.9999537652	-1.102938353
1.0518185424	0.8222868357	0.9999537652	-1.2736473028
1.3109319137	0.2900199645	0.9999537652	1.1531328016
1.1038430652	0.682704485	0.9999537652	1.0888098614
1.2081853085	0.187725366	0.9999537652	1.3830564903
-1.4892185071	0.2498597357	0.9999537652	-1.2149661469
1.2692991505	0.5549790002	0.9999537652	1.061137471
1.053110248	0.8972447585	0.9999537652	-1.1165539028
1.5046268894	0.0987964606	0.9999537652	1.01880287
-1.2562680862	0.5608898904	0.9999537652	-1.0469148155
-1.5985141516	0.2600369468	0.9999537652	-1.1826445195
1.2662502143	0.6544549948	0.9999537652	7.2042928012
1.0048199391	0.9806344077	0.9999537652	1.7843249597
-1.5338379945	0.1647777463	0.9999537652	1.0366400567
-1.0419257066	0.7612193159	0.9999537652	1.0981208618
1.0371672258	0.7763549621	0.9999537652	1.2027667053
1.417833254	0.0794363432	0.9999537652	1.1271571482
-1.0398491032	0.8288781938	0.9999537652	1.2387278832
-1.0005954443	0.9980832029	0.9999537652	1.2078214629
1.530834222	0.4654560206	0.9999537652	-1.692973547
-1.0854686404	0.5717702772	0.9999537652	1.1153998112
-2.5871290907	0.4897520312	0.9999537652	2.0633900066
-1.029383396	0.8397245778	0.9999537652	1.1386900021
-5.6905011588	0.0697469629	0.9999537652	-2.5700155719
1.0653610172	0.9019323902	0.9999537652	-1.2999171773
1.2028657005	0.7012058367	0.9999537652	-1.0911032105
1.0067174661	0.9882976783	0.9999537652	-1.4550176959
-1.0050957921	0.9722358152	0.9999537652	-1.0529688048
-1.3904787857	0.3342694355	0.9999537652	-1.1647876254
0	0	0	9.7116702472
-1.0999155919	0.594486437	0.9999537652	1.001536589
1.0156970969	0.9746146524	0.9999537652	1.7985228477
-2.1317846121	0.4669919408	0.9999537652	1.4559140589
-1.3149940926	0.6578399284	0.9999537652	1.0037832326
-1.390716754	0.2347941135	0.9999537652	1.08068827
1.0861017494	0.6119790961	0.9999537652	1.1558320785
-2.1133488124	0.274544293	0.9999537652	-2.1076057036
1.0040153316	0.9881994885	0.9999537652	-1.0769998477
1.1047411091	0.7066467487	0.9999537652	3.0847523153
-1.1891753494	0.4693327568	0.9999537652	-1.1354529759
9.8205505243	0.3808407804	0.9999537652	-3.3849925178
1.3425821795	0.1295773693	0.9999537652	1.0958572906
1.0596930067	0.8067757227	0.9999537652	1.1148004664

-1.0649522349	0.8441457685	0.9999537652	-1.5171865159
1.8480785336	0.1117186477	0.9999537652	-1.0432953382
1.1216701798	0.9025715021	0.9999537652	0
-1.0911287448	0.5824191102	0.9999537652	1.1544815395
-7.0533847096	0.0431407853	0.9999537652	3.0951743987
-1.2132422548	0.2991238748	0.9999537652	1.1763814727
-1.4964478785	0.00722	0.9999537652	1.010300601
-1.0565749157	0.6466841917	0.9999537652	1.0349235454
1.0430970519	0.8798872452	0.9999537652	-1.1781087501
-1.4551235062	0.4379783324	0.9999537652	-1.8207449568
1.051464295	0.7641018888	0.9999537652	1.0465980141
1.1175790356	0.5747015969	0.9999537652	1.604209852
-1.2405205283	0.319274142	0.9999537652	1.3125232669
1.1115529823	0.415954332	0.9999537652	1.1689826805
-1.1349126235	0.5069756597	0.9999537652	1.0534576444
1.1954292806	0.5443823914	0.9999537652	-1.0984163235
-1.6562690281	0.4326602367	0.9999537652	-1.5397188061
-1.050090966	0.8044453571	0.9999537652	-1.5978340446
3.8758796457	0.1141530164	0.9999537652	-1.2216347795
0	0	0	1.6477648168
1.028906392	0.9392655355	0.9999537652	1.0965589323
8.064174035	0.4240401907	0.9999537652	4.9527501949
1.2616406433	0.7593024676	0.9999537652	5.1169828535
-6.9736190158	0.2250321147	0.9999537652	2.9985540909
0	0	0	2.9988886918
1.4611417641	0.0398133249	0.9999537652	-1.6681219016
-1.0144075024	0.9594694852	0.9999537652	-1.0916567783
2.408291804	0.1235001808	0.9999537652	-1.2991127731
1.2817804908	0.7138329158	0.9999537652	2.9461161017
1.1131917049	0.806142385	0.9999537652	1.1723221187
1.0559562349	0.69771185	0.9999537652	1.1769551818
9.8288704632	0.3801944294	0.9999537652	-1.3588677454
-1.1453192235	0.9088592382	0.9999537652	-1.4624596198
1.2286551847	0.2488486281	0.9999537652	1.2872391788
2.7933974408	0.6948713406	0.9999537652	0
2.7933974408	0.6948713406	0.9999537652	4.9527501949
0	0	0	4.9527501949
1.0166443047	0.9432815558	0.9999537652	-1.261237475
1.0802482415	0.7762235705	0.9999537652	-1.1385484184
-1.0665916386	0.6446559446	0.9999537652	1.013004461
1.1667175549	0.3939179647	0.9999537652	1.1577785237
1.1415403797	0.2415299997	0.9999537652	1.1966245047
-1.6582973663	0.4208402466	0.9999537652	-1.2147197311
6.3075231393	0.4870288231	0.9999537652	0
2.2844548067	0.0992600529	0.9999537652	1.4049865365
37.9815106936	0.1535136995	0.9999537652	1.2756108885
1.1044295112	0.6532091714	0.9999537652	1.0036338694
1.0272517307	0.8575189846	0.9999537652	1.1995238457
1.0976267088	0.6687072457	0.9999537652	-1.1352596594

0	0	0	6.908559683
-1.0337139684	0.8149369268	0.9999537652	-1.01294458
-1.117086213	0.5258611403	0.9999537652	-1.181915085
1.2784040264	0.2323748443	0.9999537652	-1.0961327023
1.1851317683	0.2482257728	0.9999537652	-1.0994918423
1.0976785224	0.4234822196	0.9999537652	1.2862529429
1.1156994743	0.6799390204	0.9999537652	-1.0771135169
1.4815638907	0.6277785519	0.9999537652	-2.2046899968
0	0	0	2.9985540909
-1.0482500809	0.7073671371	0.9999537652	-1.0505827324
-1.2195193132	0.4614762358	0.9999537652	-1.1028528742
-1.0629426453	0.6573503697	0.9999537652	-1.4202076863
-1.3275847878	0.0396141043	0.9999537652	-1.2133072679
-1.0725723485	0.7242401022	0.9999537652	-1.1874291173
-1.9882162322	0.2289444813	0.9999537652	1.2161260858
1.0936755407	0.4713842591	0.9999537652	1.0857371991
0	0	0	14.7475552392
1.0446736572	0.8994266017	0.9999537652	1.1738267509
-1.2508553793	0.3129076794	0.9999537652	-1.0223256556
1.2111997716	0.2894834368	0.9999537652	-1.0911071078
-1.2461965714	0.4856020931	0.9999537652	-1.0451356757
-1.1070325457	0.5717995717	0.9999537652	-1.0074196187
1.4460508616	0.0969432314	0.9999537652	-1.4453283606
1.1646788163	0.5425968321	0.9999537652	1.0306227887
1.0701732095	0.6538342866	0.9999537652	1.3238369069
-1.0322955143	0.9316792366	0.9999537652	1.0100047459
3.8622037496	0.6197885868	0.9999537652	0
2.7933974408	0.6948713406	0.9999537652	-9.7554110723
-1.2836554294	0.2230596134	0.9999537652	1.4305497067
1.1665586926	0.4982843779	0.9999537652	-1.1426381184
-1.1382805948	0.5853501149	0.9999537652	-1.0175284655
0	0	0	8.8639046058
1.9825471683	0.1667754613	0.9999537652	1.3283001114
1.1122087721	0.4135418043	0.9999537652	1.1330939217
1.0101964742	0.9431976813	0.9999537652	1.0575990195
1.0505262422	0.9641113376	0.9999537652	-2.7915177751
-1.1345273227	0.5279269254	0.9999537652	-1.447371804
1.159734916	0.5593021392	0.9999537652	1.0744819519
-1.5875118025	0.7205368862	0.9999537652	0
2.5734272962	0.4089959982	0.9999537652	0
-1.10880233	0.6195276406	0.9999537652	-1.041718219
1.0956092405	0.7739516499	0.9999537652	1.1724711819
2.0346773175	0.4381573916	0.9999537652	-1.1200974951
1.3295349533	0.2063689986	0.9999537652	-1.2779481972
1.4808270106	0.6343642752	0.9999537652	5.1060810775
1.2071571	0.1589138045	0.9999537652	1.2186107439
1.0770732551	0.8461116863	0.9999537652	1.2573415003
-2.4941151816	0.4492553881	0.9999537652	0
16.8433158146	0.2732251893	0.9999537652	-2.6352978387

1.1995245564	0.3185432324	0.9999537652	1.0794746026
-1.4737854567	0.1979946162	0.9999537652	-1.0562020033
1.1110412466	0.6296773011	0.9999537652	1.0175331845
-1.7127326186	0.001336	0.9999537652	-1.5632827783
-1.6775581998	0.0527380129	0.9999537652	-1.5594645273
1.4388088249	0.4949045114	0.9999537652	-1.1159525223
-1.001548889	0.9971040176	0.9999537652	-1.2423539692
1.0821115005	0.8268815401	0.9999537652	1.1845130728
1.423446877	0.1496932697	0.9999537652	-1.2675406798
-1.0284896134	0.8794509328	0.9999537652	1.117063615
-1.0033956753	0.993151457	0.9999537652	1.0692854103
1.0271125191	0.8364209498	0.9999537652	1.4398725432
1.0416399656	0.776998151	0.9999537652	1.3085993496
-1.0102775895	0.964357671	0.9999537652	-1.2215158908
-1.0711593783	0.6143655579	0.9999537652	1.2387940297
-4.098385977	0.1622357015	0.9999537652	1.671192618
-1.0037185932	0.986610555	0.9999537652	-1.0052956664
2.0885615936	0.5182136312	0.9999537652	-1.859280336
0	0	0	0
1.0242836376	0.9050904545	0.9999537652	1.2513219298
-1.0282648099	0.886268229	0.9999537652	1.0144701543
-1.6652199227	0.6285563437	0.9999537652	-4.5793149393
-2.8969127737	0.339811129	0.9999537652	-2.5775354782
1.5404849439	0.0761548279	0.9999537652	-1.1499873756
1.2605254131	0.30961344	0.9999537652	1.0448706416
-1.0878753682	0.583920027	0.9999537652	-1.0083171846
-1.0387419901	0.9527659097	0.9999537652	-6.4360166533
1.2802242621	0.5367765405	0.9999537652	1.7469894978
-3.8068935632	0.0377057973	0.9999537652	-1.2221582574
-1.2402015004	0.1520226315	0.9999537652	-1.1352138982
-1.1747327298	0.305095714	0.9999537652	-1.2030856568
1.0862608685	0.6772201657	0.9999537652	-1.1158007863
-1.081974055	0.7310334017	0.9999537652	-1.8715902532
2.7933974408	0.6948713406	0.9999537652	14.7373490367
2.9819103542	0.0646749425	0.9999537652	-1.0418570551
1.0844353317	0.7228765396	0.9999537652	-1.0185562526
1.0756929012	0.7031239524	0.9999537652	-1.2350535412
-1.0354432274	0.8703592214	0.9999537652	1.1048572939
-1.3227553882	0.1327159698	0.9999537652	-1.149866961
-4.8154268017	0.1712376447	0.9999537652	6.9133480437
-1.1634417496	0.2527358457	0.9999537652	-1.4051817751
1.6507269804	0.00334	0.9999537652	-1.4126926335
-7.0403382421	0.1013299562	0.9999537652	1.0283954772
4.5505976667	0.5590482994	0.9999537652	4.9561842702
0	0	0	-5.3583704783
1.4988385415	0.1000765999	0.9999537652	-1.0829831093
3.7885904228	0.2339824168	0.9999537652	-1.337530768
1.6527716635	0.007363	0.9999537652	1.0941081457
-1.2997076887	0.1891313776	0.9999537652	-1.0020501253

-1.1858615939	0.3360574621	0.9999537652	1.1511255918
-1.129950963	0.482216618	0.9999537652	1.1199725611
-1.3407975671	0.6539496193	0.9999537652	1.2166702826
-1.1470209572	0.5672248431	0.9999537652	-1.0595430673
-1.1213329976	0.6273172022	0.9999537652	1.0651971243
-2.9548384089	0.3317424324	0.9999537652	1.0845613577
-1.2366999846	0.1711900534	0.9999537652	1.259269689
-1.1767385784	0.7912839162	0.9999537652	1.1651752551
1.446336875	0.1393446943	0.9999537652	1.1478439863
1.1367761803	0.3825506238	0.9999537652	1.2240473471
1.6108138003	0.6452806171	0.9999537652	1.1031068191
-1.5881166222	0.7249115844	0.9999537652	14.759428837
2.7957660672	0.705398841	0.9999537652	-2.7336138579
1.5087199967	0.1529632749	0.9999537652	1.2073350352
3.7917931577	0.2239176224	0.9999537652	-1.0663370569
1.0672244872	0.7763311143	0.9999537652	1.1760313725
-4.2249492059	0.1250400043	0.9999537652	4.4079224032
1.4482995658	0.1186906269	0.9999537652	1.0379317943
0	0	0	-1.1250196696
4.5505976667	0.558976936	0.9999537652	0
1.1824783045	0.2907449004	0.9999537652	1.0666270089
-1.1011091173	0.6736130095	0.9999537652	1.2608174091
1.4926310182	0.091016644	0.9999537652	1.1389471866
-1.147880094	0.5297789204	0.9999537652	1.0098298752
2.0381854033	0.4032758863	0.9999537652	1.3119335805
1.4203878116	0.129538662	0.9999537652	1.3239181054
-1.1219739905	0.4891565813	0.9999537652	-1.1381751718
-1.2825267982	0.4198945116	0.9999537652	-1.1699896178
-1.1618692829	0.7343507238	0.9999537652	-1.2584883793
29.5568947391	0.1882827705	0.9999537652	1.8520499819
1.1924441742	0.3044727206	0.9999537652	-1.0881018621
1.1248274988	0.8663597643	0.9999537652	-1.237232726
1.0945334741	0.6191673329	0.9999537652	1.0766039014
-1.0809392341	0.8804092977	0.9999537652	-1.075323797
-1.0157496634	0.9444382047	0.9999537652	1.0539842754
-1.0595232108	0.8732730153	0.9999537652	-1.0082503917
1.0983740647	0.6764925561	0.9999537652	1.1395831622
1.1258669626	0.5202517206	0.9999537652	1.0143336599
1.1346597241	0.3577530268	0.9999537652	1.3885942677
-1.2975783546	0.2530639977	0.9999537652	-1.0642416929
2.3382720325	0.007936	0.9999537652	1.2479541116
1.5836224273	0.424393935	0.9999537652	-1.374499486
-1.1758687306	0.2461257478	0.9999537652	1.0250175212
4.5505976667	0.5588994102	0.9999537652	2.996475627
1.2844709255	0.6663174317	0.9999537652	10.818785458
1.0974495954	0.6514484385	0.9999537652	1.0972806759
20.3530564257	0.2435350409	0.9999537652	-1.1248877899
4.0331711848	0.2059780192	0.9999537652	1.2240749939
-1.081658322	0.5043968781	0.9999537652	1.1709029152

1.0177249572	0.9481907009	0.9999537652	1.0307535348
1.2392631105	0.510749715	0.9999537652	1.0839194019
-1.6410314086	0.1765155094	0.9999537652	-1.1808627664
-1.0150276475	0.9409864798	0.9999537652	1.0511396578
4.5505976667	0.5588175533	0.9999537652	2.2924151284
1.1941395572	0.6812722468	0.9999537652	1.3268903746
0	0	0	1.590997316
-1.0799225403	0.7947625901	0.9999537652	1.5621846161
1.8448460942	0.5998237605	0.9999537652	1.2408886006
-1.4605535668	0.0338895755	0.9999537652	1.1924577423
-1.1414441294	0.3058758729	0.9999537652	1.1295273274
1.6052154301	0.6856306879	0.9999537652	2.9999087555
1.4796429016	0.1079417343	0.9999537652	-1.2017351532
-4.7471147536	0.100390451	0.9999537652	-2.3087357349
-1.3934705832	0.7156183074	0.9999537652	-1.4335125437
-1.0196336813	0.8694049631	0.9999537652	1.1018112104
-1.193840142	0.420532216	0.9999537652	-1.0125776662
4.3358068607	0.0480613054	0.9999537652	1.7313438286
-1.1550920675	0.2965004508	0.9999537652	1.1471454071
-1.0676606513	0.6574594499	0.9999537652	-1.1745329622
-1.192568278	0.3278033392	0.9999537652	-1.0062679703
-1.1713471254	0.4584900772	0.9999537652	1.156139105
1.2216084395	0.5113503136	0.9999537652	-1.2269109072
-2.9548384089	0.3317265812	0.9999537652	2.5959757848
1.081613075	0.7482593112	0.9999537652	-1.073185368
1.138991558	0.8093688082	0.9999537652	-1.673676791
-1.5631133972	0.082691569	0.9999537652	1.1101290999
-1.3853586434	0.489141958	0.9999537652	-2.4582849266
-1.0434215101	0.8359922231	0.9999537652	1.1106114347
1.5802084855	0.0442204743	0.9999537652	1.1550894851
1.3350543109	0.2141469026	0.9999537652	-1.030322181
-1.1284395444	0.4249747513	0.9999537652	1.1370132042
1.0363695661	0.8493418368	0.9999537652	-1.0858768016
2.2953539498	0.349495545	0.9999537652	1.304860287
-1.0806091882	0.5768849485	0.9999537652	-1.0245944544
1.1882223702	0.2820198657	0.9999537652	1.5083170857
9.8205505243	0.3808406367	0.9999537652	1.588935368
1.0238002172	0.8680757698	0.9999537652	1.0417738994
-1.0383368576	0.8350030578	0.9999537652	-1.0459739401
-1.2009583878	0.4579088607	0.9999537652	-1.0703613529
1.3968621473	0.3075018376	0.9999537652	-1.2659218438
2.0913709129	0.5137428426	0.9999537652	-5.3430506873
-6.9736190158	0.2250321147	0.9999537652	4.9527501949
1.11528193	0.7704660283	0.9999537652	1.0897539889
1.1535694891	0.6820466608	0.9999537652	-1.0225040408
2.2621823855	0.0506747972	0.9999537652	1.9509380862
1.1164814744	0.6148923367	0.9999537652	1.1595196511
1.2758024572	0.138113074	0.9999537652	1.0422618977
1.2903280591	0.4852696395	0.9999537652	1.363905278

-1.1504170339	0.4602796512	0.9999537652	-1.1055976923
-1.2477064495	0.7454992784	0.9999537652	-1.0081791187
-1.0023783034	0.9848097252	0.9999537652	1.056564502
-1.0107771541	0.9816254756	0.9999537652	1.0056130278
1.0776947607	0.5283350487	0.9999537652	-1.0250974795
-1.1080247849	0.5699907126	0.9999537652	1.1935116032
-1.1006309869	0.6101853279	0.9999537652	1.0938472463
1.4084382019	0.176157438	0.9999537652	1.1866769623
-1.0469015548	0.7456748563	0.9999537652	-1.0912954657
-1.0083824309	0.9631793812	0.9999537652	1.4614030989
2.7933974408	0.6948713406	0.9999537652	-5.3589986948
-1.4331761012	0.1422959565	0.9999537652	1.3571316467
22.1637147731	0.2320658758	0.9999537652	-2.7873245155
1.4302608385	0.1841225353	0.9999537652	1.1222410032
-1.0672495073	0.6588876572	0.9999537652	-1.1490553244
-1.0423102182	0.8719911666	0.9999537652	1.0973775433
1.2348966906	0.3381613159	0.9999537652	-1.0790081451
-1.0924641211	0.6345656442	0.9999537652	-1.1898109537
1.046147328	0.8865917895	0.9999537652	1.461601044
-1.1043731566	0.8176763872	0.9999537652	-1.353566927
1.0044815238	0.9830250119	0.9999537652	-1.1959066293
-1.2640901764	0.4178413883	0.9999537652	-1.0513578167
1.1863311226	0.1858902285	0.9999537652	1.2489553775
-1.2792349814	0.5762420891	0.9999537652	1.4812228128
1.0008312617	0.9944426877	0.9999537652	1.2087635659
1.1276684763	0.5282196151	0.9999537652	-1.1144856634
1.1833883571	0.4085164661	0.9999537652	1.1163350546
-1.6674095463	0.6216697131	0.9999537652	-1.0352055009
-1.09519984	0.7014669087	0.9999537652	1.0242726638
1.1287717835	0.8170883049	0.9999537652	-1.1112342004
-1.4162789898	0.1422618517	0.9999537652	1.0635636782
1.2827259419	0.0554044628	0.9999537652	1.1975018884
-1.3445734548	0.0589631521	0.9999537652	1.3880366718
-1.2616007393	0.4543924045	0.9999537652	1.0807937202
-1.0174112379	0.9304315041	0.9999537652	1.2112235551
-1.0062813162	0.9738032556	0.9999537652	1.0569285196
-1.0225532015	0.875315734	0.9999537652	-1.1221491258
1.6733267759	0.3535639994	0.9999537652	-1.0803902304
-1.0457166013	0.7114214317	0.9999537652	1.2072968312
-1.1392184577	0.3315872055	0.9999537652	1.4657776848
-1.4766751143	0.1289481382	0.9999537652	-1.1071880313
-1.0641133638	0.7224596679	0.9999537652	1.0051893968
-1.0960247838	0.8188635375	0.9999537652	1.2183690585
1.0689288608	0.6810744377	0.9999537652	1.7082023842
1.2179721842	0.5210174987	0.9999537652	-1.2014138954
1.1559047076	0.4712539334	0.9999537652	1.0366002214
-1.0074619145	0.953649892	0.9999537652	-1.1205297027
-1.1609514984	0.8759353054	0.9999537652	1.0314779617
-1.0136156507	0.9227786195	0.9999537652	1.0537178219

1.6310542149	0.447638661	0.9999537652	1.1013488662
1.8572566386	0.2746114019	0.9999537652	-1.4143716384
1.1745681	0.4975919024	0.9999537652	-1.0052617088
-5.2081415539	0.1200752094	0.9999537652	31.6482701458
-1.0012328548	0.9955505496	0.9999537652	-1.1855146152
1.631109999	0.0132764326	0.9999537652	1.1749259263
1.0316496749	0.8579940459	0.9999537652	-1.069568083
-1.0703032235	0.7132170751	0.9999537652	1.4473744106
-1.1830558853	0.3693552596	0.9999537652	1.2588187514
1.0921987811	0.6419548404	0.9999537652	1.1699783717
1.1182309188	0.7210196154	0.9999537652	-1.1448969378
-1.1594602839	0.8873224608	0.9999537652	-1.0132535059
1.1299883427	0.3184330125	0.9999537652	1.369590135
-1.1344497867	0.8239755572	0.9999537652	1.0331293307
-1.0947947183	0.6021771455	0.9999537652	1.2340083662
1.163060446	0.3906541211	0.9999537652	1.004047103
-1.0567489886	0.6625260327	0.9999537652	-1.0008897953
1.005861914	0.9739146685	0.9999537652	1.1040274674
1.906705352	0.4815058269	0.9999537652	-1.7637225952
0	0	0	6.9133480437
1.6024984014	0.6863390106	0.9999537652	-1.1609740856
-1.1737692886	0.8014790843	0.9999537652	-1.4059521531
1.1292730045	0.5123858395	0.9999537652	1.2470851628
2.1619312741	0.0309488162	0.9999537652	-1.2460067808
-1.0551173092	0.88942853	0.9999537652	1.9672049126
-1.0860194698	0.6733001923	0.9999537652	1.1393348467
-1.0591106575	0.7534166064	0.9999537652	1.1804450321
1.2811235259	0.3297026695	0.9999537652	-1.0327783131
-1.3679958882	0.7463894949	0.9999537652	-1.0340216643
0	0	0	2.996475627
-1.0742417884	0.784719014	0.9999537652	-1.0424642958
-1.0307385525	0.8876769475	0.9999537652	-1.6237020337
2.7933974408	0.6948713406	0.9999537652	0
-1.3188045192	0.6344491193	0.9999537652	-1.3196481246
1.3664122026	0.2727582128	0.9999537652	1.6146658147
1.0201549666	0.8762936608	0.9999537652	-1.183172489
1.186255622	0.7061639179	0.9999537652	-1.003224592
1.1271331023	0.7232675099	0.9999537652	1.3803649771
1.7201856284	0.003523	0.9999537652	1.4778087278
-1.1608369906	0.496090571	0.9999537652	1.1449693265
1.0338075662	0.9025661541	0.9999537652	-1.0273104736
0	0	0	1.6470015103
1.6806509228	0.0110255051	0.9999537652	-1.0494927142
-1.5431926863	0.1381518764	0.9999537652	-1.0493105259
1.0746905425	0.6998849347	0.9999537652	-1.0677043653
1.0974242985	0.5711580141	0.9999537652	1.2515995961
1.1587010925	0.3399954314	0.9999537652	1.1170163834
-1.4106347582	0.1480537988	0.9999537652	1.351539716
-1.6168919992	0.0322781001	0.9999537652	-1.4913938484

-2.4397350298	0.3113408283	0.9999537652	-1.0534454052
1.6252975995	0.0876175722	0.9999537652	-1.0402281654
3.8622037496	0.6197912269	0.9999537652	0
2.0108427018	0.1261005778	0.9999537652	1.1403259038
1.6321617407	0.1248286651	0.9999537652	-1.0264356825
1.4407939588	0.0845190214	0.9999537652	-1.0104538447
-1.1268728768	0.4870888507	0.9999537652	-1.1896248241
3.3385228807	0.1727008687	0.9999537652	1.0691812528
-1.1287920639	0.7941713956	0.9999537652	-1.3834448758
-1.369198268	0.7570901059	0.9999537652	3.3816577698
2.0464985585	0.2111223347	0.9999537652	1.1305037387
0	0	0	2.996475627
1.3611047324	0.0410826256	0.9999537652	1.3696797105
1.4481777514	0.1509118261	0.9999537652	1.1684759897
-5.0934378954	0.0661078826	0.9999537652	14.7271569203
-1.1516369802	0.3249971728	0.9999537652	-1.1220629068
-1.1027145204	0.8889519758	0.9999537652	1.5745525552
2.7957660672	0.7054035	0.9999537652	0
1.012313686	0.9336752872	0.9999537652	1.0767276024
1.0013296842	0.9925106306	0.9999537652	-1.1018588721
-2.4640383069	0.1675453282	0.9999537652	-1.2592400625
-1.136454091	0.5865725953	0.9999537652	-1.2143172274
1.7587380237	0.0840270217	0.9999537652	-1.3228951124
4.5545995984	0.5696828575	0.9999537652	1.7042814026
8.064174035	0.4240385553	0.9999537652	-3.3838184763
1.0506030245	0.7550296352	0.9999537652	-1.2045333701
-2.5871290907	0.4901557076	0.9999537652	2.0101122192
0	0	0	1.590997316
1.0953061359	0.5044578433	0.9999537652	1.1542655677
-1.2248076078	0.2538596766	0.9999537652	1.0038060405
1.3588576256	0.8005871795	0.9999537652	-2.4446009962
1.1148127239	0.4386243598	0.9999537652	1.1586081984
1.3485758094	0.1556083153	0.9999537652	-1.1871464056
1.2335007002	0.6050764947	0.9999537652	-1.1292070952
1.0775890912	0.6480678701	0.9999537652	-1.2757278192
-2.5861438062	0.4593544485	0.9999537652	-1.0582880019
-1.2865014513	0.04741445	0.9999537652	-1.2722630121
1.8157951106	0.0524022788	0.9999537652	-1.1501541157
1.228785314	0.1366613878	0.9999537652	1.0595736936
-1.0503494032	0.9230439891	0.9999537652	-1.482390217
1.4768617964	0.1122135864	0.9999537652	-1.2294717239
1.9093672774	0.2765822602	0.9999537652	1.1142549194
-1.0484551083	0.8079858041	0.9999537652	-1.4580823973
1.9188725267	0.0706903846	0.9999537652	-1.0311814116
-4.815549291	0.172666663	0.9999537652	1.0674541829
1.0496155734	0.9014219216	0.9999537652	1.4774479455
-1.3483457532	0.4814624825	0.9999537652	-1.0345039466
-1.2679667006	0.1459203165	0.9999537652	-1.124919753
-1.0190276476	0.9036923916	0.9999537652	-1.0848463094

1.0023830937	0.9890161277	0.9999537652	1.3469223651
1.1663801151	0.5185778634	0.9999537652	1.0627811335
1.1266417136	0.4774726759	0.9999537652	-1.0514001977
-1.268557881	0.7787183379	0.9999537652	-1.2591665984
-2.0663635347	0.1296821437	0.9999537652	-1.001857738
-1.0381611335	0.8267893531	0.9999537652	1.3710339796
2.7933974408	0.6948713406	0.9999537652	0
0	0	0	10.818785458
-6.9736190158	0.2250321147	0.9999537652	-2.9955485583
-2.3849174722	0.1969103805	0.9999537652	-1.1614920563
1.3708516524	0.6090811611	0.9999537652	-1.3128566056
-1.53365015	0.1686510024	0.9999537652	1.4580296131
1.4513002414	0.2443454839	0.9999537652	1.2789673914
-1.1887483584	0.4582806237	0.9999537652	1.0179006565
1.325095935	0.6330361996	0.9999537652	-1.2262611854
0	0	0	-3.4711016225
-1.1821937148	0.2429865594	0.9999537652	-1.3273704064
2.1650257591	0.3956668774	0.9999537652	-1.0791732097
1.2163510872	0.7697170192	0.9999537652	1.2014591601
-6.9762758667	0.2509338461	0.9999537652	9.7116702472
-1.1222587459	0.6567889369	0.9999537652	-1.1828036329
-1.4055454552	0.2669334127	0.9999537652	1.2362136219
2.7957660672	0.7054058308	0.9999537652	22.5539272529
5.2531489146	0.1114715711	0.9999537652	1.6027802519
11.5766527773	0.345553673	0.9999537652	-3.3834218841
1.4377166642	0.1465003402	0.9999537652	-1.8131571573
-1.165837361	0.2284741822	0.9999537652	-1.0715002114
-1.0686488677	0.7820309837	0.9999537652	-1.0255458428
1.0532083082	0.7311186762	0.9999537652	1.1721017748
-1.0674006543	0.6187641486	0.9999537652	-1.1382260012
1.1445165456	0.4866412192	0.9999537652	-1.0121601074
1.1738583558	0.3499054667	0.9999537652	1.1536593207
1.305101859	0.1939792897	0.9999537652	-1.0715946945
0	0	0	1.4519459565
1.317944786	0.4035688708	0.9999537652	1.0327485006
1.2838219277	0.5319545894	0.9999537652	1.2131821607
-1.0291193109	0.8909520609	0.9999537652	-1.0318940327
2.4788672036	0.2123416138	0.9999537652	-1.2776942159
1.1771929735	0.4434471529	0.9999537652	1.0448125546
-1.0116973132	0.9426813799	0.9999537652	1.1130988947
4.5505976667	0.5587384693	0.9999537652	-1.1433829476
1.1186366265	0.6847109418	0.9999537652	1.0512404652
2.510672768	0.031400241	0.9999537652	-1.1121086812
-1.0345420863	0.8569486449	0.9999537652	1.3602123097
-1.0012147382	0.9923340503	0.9999537652	-1.1624628605
1.1275963333	0.3940734482	0.9999537652	-1.2827398931
-4.3250226376	0.1726258036	0.9999537652	-2.7918445703
-1.2901173657	0.1627769905	0.9999537652	-1.0101971699
0	0	0	0

-1.2710677205	0.224891964	0.9999537652	-1.1457991715
1.3044750886	0.1610519568	0.9999537652	1.1309671479
1.1455330763	0.5092854207	0.9999537652	1.5155070745
-1.0428047338	0.835711037	0.9999537652	-1.129949082
-1.0512817794	0.877079614	0.9999537652	-1.1122942121
1.5773324614	0.1255865088	0.9999537652	1.0371418293
46.8021225725	0.1292814098	0.9999537652	2.0042248033
104.2166042209	0.06765071	0.9999537652	5.8017888001
-1.3728449527	0.0825086113	0.9999537652	1.0794942816
1.3245600169	0.0692156514	0.9999537652	1.0275992757
23.8818846228	0.2168908833	0.9999537652	-2.5819763794
1.0376345914	0.842092064	0.9999537652	1.3473560483
-1.0483745719	0.7211899931	0.9999537652	1.0434600974
-1.187908183	0.40707579	0.9999537652	1.0985327076
3.5488407019	0.2472528439	0.9999537652	-1.2845492958
9.8288704632	0.3801844595	0.9999537652	-1.859280336
1.4997045445	0.2117943172	0.9999537652	-1.1105671173
-1.3515702809	0.3170017616	0.9999537652	1.4144924728
0	0	0	-1.1250196696
-1.0759947022	0.5026798888	0.9999537652	1.178005267
-2.1395079434	0.2271274032	0.9999537652	-1.172852367
-1.0789109711	0.6083221091	0.9999537652	-1.2043065154
1.2839510277	0.2225916562	0.9999537652	-1.1992656708
3.446135261	0.0885500567	0.9999537652	-1.013911182
-1.3397986347	0.4386969039	0.9999537652	1.3664789483
-1.0064379636	0.9666535515	0.9999537652	1.0137731937
4.5505976667	0.5586621239	0.9999537652	-1.1248877899
2.575670643	0.3941663361	0.9999537652	0
-1.0186884041	0.947520079	0.9999537652	-1.2021560064
-1.5569501361	0.2680036825	0.9999537652	-1.3884544962
11.5766527773	0.3455612973	0.9999537652	0
2.7933974408	0.6948713406	0.9999537652	3.0482656807
-1.0353751356	0.9229757744	0.9999537652	1.0364045702
-1.8294672119	0.1881205748	0.9999537652	-1.1557875368
1.1817900243	0.3795437847	0.9999537652	1.0914552284
1.382415244	0.0862349607	0.9999537652	1.0031106257
1.005424923	0.9824966244	0.9999537652	-1.1373502947
8.064174035	0.4240369201	0.9999537652	1.6470015103
1.1217899267	0.5723623488	0.9999537652	1.1629107527
-1.1457555741	0.9107273157	0.9999537652	-1.7494930838
1.2920645114	0.2086048738	0.9999537652	-1.0011836516
4.5505976667	0.5585883533	0.9999537652	0
4.5505976667	0.5585170036	0.9999537652	26.0746223853
6.3075231393	0.4870147668	0.9999537652	17.8369746551
-1.0144267182	0.9637982862	0.9999537652	1.1168832909
1.1842625607	0.2010796008	0.9999537652	1.1735734856
-1.1974308496	0.1898139472	0.9999537652	-1.0448176821
1.3373994913	0.1343966613	0.9999537652	1.5699530952
0	0	0	2.9985540909

1.2923908691	0.4077268937	0.9999537652	-1.4525757749
1.0230433426	0.9558263749	0.9999537652	2.5681060545
1.517710517	0.2956100222	0.9999537652	1.85994496
36.2262387855	0.1571274218	0.9999537652	1.5880461882
3.8622037496	0.6197938668	0.9999537652	0
4.5505976667	0.5584479292	0.9999537652	0
2.7957660672	0.7054081626	0.9999537652	-1.3243569371
2.7933974408	0.6948713406	0.9999537652	-2.8128331331
2.2076065015	0.2812119945	0.9999537652	3.1614536713
-1.0661223841	0.7136107442	0.9999537652	1.2699702945
-1.0639540243	0.8836502861	0.9999537652	1.1031211226
1.0276995588	0.8811142018	0.9999537652	-1.0876854922
1.0106616082	0.9451560717	0.9999537652	1.1292748305
1.2160807835	0.410684846	0.9999537652	1.728947716
1.4022354684	0.1189204562	0.9999537652	1.1521163836
1.5918525205	0.1754832731	0.9999537652	-1.0982471667
-1.2156742938	0.3437797691	0.9999537652	-1.2924883707
1.7911605773	0.2065499534	0.9999537652	1.9237392568
-4.5265719391	0.1070176165	0.9999537652	2.8725186868
1.0324918925	0.8383634528	0.9999537652	-1.0200286386
-1.2006203594	0.33783475	0.9999537652	1.2166111376
1.101472639	0.8451646504	0.9999537652	1.3212038641
-1.0044315522	0.9724543502	0.9999537652	-1.0358592993
-1.063392902	0.9063452941	0.9999537652	-1.7592853085
1.0360591358	0.860578447	0.9999537652	-1.0218909763
2.5092975614	0.001741	0.9999537652	1.0022032551
2.7933974408	0.6948713406	0.9999537652	-1.5065200998
6.3075231393	0.4870049067	0.9999537652	0
2.7933974408	0.6948713406	0.9999537652	0
0	0	0	2.996475627
3.230418959	0.0004293	0.9999537652	-1.0038932274
-1.3839218068	0.0441088945	0.9999537652	-1.1337820137
3.2070037624	0.1842513972	0.9999537652	2.8611270168
-1.1015288048	0.757551012	0.9999537652	1.4848438361
2.5581903582	0.2693857439	0.9999537652	-14.1559579336
1.3925460243	0.4701448106	0.9999537652	-1.2804657245
1.222500971	0.4176775896	0.9999537652	1.5757387169
1.0764438128	0.5763053497	0.9999537652	1.1931625413
-1.1748712185	0.2851947396	0.9999537652	1.0296425567
3.899557091	0.0454346083	0.9999537652	-1.179601593
-1.1166055497	0.5923615733	0.9999537652	-1.0467335517
1.2453719422	0.5866162136	0.9999537652	1.3022507594
1.3072022755	0.3815946085	0.9999537652	-1.2469968636
1.2275955918	0.4802504768	0.9999537652	-2.0159686276
-1.4926346254	0.3717455876	0.9999537652	1.9980400577
-1.0677893158	0.5928114977	0.9999537652	-1.2062845609
4.5545995984	0.5696807792	0.9999537652	0
2.7933974408	0.6948713406	0.9999537652	0
1.1187569927	0.6751923738	0.9999537652	1.0759626672

-1.0843112546	0.7887559903	0.9999537652	-1.299373743
0	0	0	1.5920996292
-1.2619015472	0.1901100734	0.9999537652	-1.0827480763
1.3112536333	0.0796239317	0.9999537652	1.1935826794
8.064174035	0.4240352852	0.9999537652	-3.4501420467
2.7933974408	0.6948713406	0.9999537652	1.590997316
-1.0602949127	0.6997599954	0.9999537652	-1.2214960137
-1.0488959901	0.7048196682	0.9999537652	1.2312389026
-1.1423603746	0.3435165941	0.9999537652	1.1958946217
-6.9736190158	0.2250321147	0.9999537652	-1.4739994041
-1.0182291284	0.8769334005	0.9999537652	-1.1915465113
-1.0380199861	0.8952084827	0.9999537652	1.0940970007
1.0202256494	0.9166452923	0.9999537652	1.1316997244
1.1458270613	0.5176624813	0.9999537652	1.108333056
0	0	0	2.294258748
-1.1726092784	0.3727005891	0.9999537652	-1.2508200746
1.1707605808	0.5379476895	0.9999537652	-1.4970747678
11.5766527773	0.3455689347	0.9999537652	-9.7542681481
1.2064361531	0.5350547081	0.9999537652	1.297983153
-1.435120256	0.0339141526	0.9999537652	-1.0139773506
1.0395434765	0.8432792198	0.9999537652	1.1210523177
-1.3434978242	0.4073829945	0.9999537652	1.3154989745
-1.051491947	0.8420411988	0.9999537652	-1.0440636349
1.0327559126	0.8697011307	0.9999537652	-1.0908243304
1.2719903785	0.1700499159	0.9999537652	-1.3628983638
1.0407302421	0.8036513922	0.9999537652	1.2691815865
1.4870011197	0.6638762022	0.9999537652	-1.3526296379
0	0	0	-2.1991066392
-1.4062733228	0.0544880716	0.9999537652	-1.2358178488
1.0604986262	0.7362959231	0.9999537652	-1.1009587873
0	0	0	0
-1.1962616753	0.3415749422	0.9999537652	-1.0229274783
-1.1926591704	0.3326831606	0.9999537652	-1.0185507825
-1.0196766622	0.9348522408	0.9999537652	-1.0337380773
-1.1175060453	0.4578610681	0.9999537652	-1.233058625
-1.1671519505	0.377727764	0.9999537652	-1.0012186838
-1.0115710622	0.9364327486	0.9999537652	1.0641310171
2.5571932577	0.2303310822	0.9999537652	-1.174998263
1.2931887438	0.0675507649	0.9999537652	-1.1337382177
16.8433158146	0.2733719414	0.9999537652	-1.3501019658
-1.4096239266	0.1047920512	0.9999537652	1.3368117001
-1.0018414411	0.9910663511	0.9999537652	-1.1589935475
1.087187886	0.5765108995	0.9999537652	1.0674459458
-4.1886437672	0.0186117221	0.9999537652	-1.0276765506
1.0110399122	0.9627302231	0.9999537652	1.1629265409
1.2179883202	0.2890981602	0.9999537652	1.0432374177
-1.3544063599	0.1796142567	0.9999537652	-1.1258047693
1.0700335683	0.7334322846	0.9999537652	-1.0970126568
6.3075231393	0.486995049	0.9999537652	0

1.2063715897	0.3024298548	0.9999537652	1.1439629319
-6.3379683005	0.1119058386	0.9999537652	1.0709925636
1.0717281897	0.5901231216	0.9999537652	1.1060222317
1.0197735878	0.8929436831	0.9999537652	1.418619578
1.0625917309	0.6358574856	0.9999537652	-1.0111699819
-2.1334087267	0.5175405488	0.9999537652	15.7262184124
2.3664678756	0.1245201961	0.9999537652	1.7623374623
1.0113135824	0.9638160788	0.9999537652	1.2321844265
0	0	0	2.9985540909
-1.1623826462	0.3336722447	0.9999537652	-1.0038493808
-1.2105057064	0.5220780342	0.9999537652	1.0064153785
1.413444022	0.0509080291	0.9999537652	-1.0542288639
1.6866556362	0.6359287998	0.9999537652	28.6068612613
-1.091376919	0.6388241446	0.9999537652	1.157579215
1.2902169021	0.3383846177	0.9999537652	1.096361896
1.0324564755	0.8574344515	0.9999537652	1.0018070987
1.0206510335	0.9073675912	0.9999537652	-1.171422604
1.478101785	0.433673781	0.9999537652	1.1699817492
1.088666893	0.6414089663	0.9999537652	1.359646203
3.5613076969	0.0440183865	0.9999537652	1.545158595
-1.1214471766	0.4754548331	0.9999537652	1.2549748625
6.3075231393	0.4869851934	0.9999537652	16.0748987101
1.0285995067	0.8470265337	0.9999537652	1.114013196
-1.7160826635	0.5343625296	0.9999537652	-1.4270110243
-1.0764497303	0.6376082386	0.9999537652	1.2465702795
1.0616140053	0.8518166904	0.9999537652	1.0494761943
1.2130956915	0.49783033	0.9999537652	1.3675882005
1.3872634786	0.4583705084	0.9999537652	-1.0258745523
-1.0257181198	0.8443252306	0.9999537652	1.0853660913
-1.0382490483	0.8590962711	0.9999537652	1.3286203085
4.5505976667	0.5583809928	0.9999537652	-3.7211335265
0	0	0	9.7116702472
16.872391439	0.2715769208	0.9999537652	-2.5339008304
-1.4332997873	0.369601374	0.9999537652	-1.1258049891
0	0	0	2.996475627
2.7933974408	0.6948713406	0.9999537652	2.996475627
-1.2423868056	0.6262708475	0.9999537652	2.0080890125
1.1250627958	0.8657063046	0.9999537652	1.2291839564
6.3128691894	0.4819949873	0.9999537652	3.9094828033
-1.2414140907	0.449320062	0.9999537652	-1.0900809808
-1.8357405856	0.3301157908	0.9999537652	-1.3999527387
1.4611506879	0.218634201	0.9999537652	1.1002582051
1.2965216442	0.5197173903	0.9999537652	1.5567359402
1.3029210265	0.1346135097	0.9999537652	1.0692392968
1.6342333843	0.2478608519	0.9999537652	1.2255247796
0	0	0	-1.2959348192
-1.4392737333	0.3642851052	0.9999537652	2.4815633475
0	0	0	2.996475627
1.0202468338	0.8999714141	0.9999537652	-1.0022339759

-1.1985756519	0.4249861985	0.9999537652	1.4631802027
1.0805576243	0.5644574288	0.9999537652	1.0659289352
9.8205505243	0.3808404929	0.9999537652	0
2.0660567426	0.2013704182	0.9999537652	1.5103206593
-1.0887621021	0.6399977841	0.9999537652	1.1428374642
-1.139075093	0.692583422	0.9999537652	1.0506932347
-1.0218515284	0.9043628656	0.9999537652	1.6373966832
0	0	0	2.996475627
3.8622037496	0.6197965079	0.9999537652	-3.3849925178
2.4010450491	0.0168777931	0.9999537652	1.3457310933
-1.0063159556	0.9837631252	0.9999537652	1.3508439475
13.3324809638	0.3187168751	0.9999537652	-1.3092040421
-1.2357865481	0.1543328556	0.9999537652	-1.027808602
-1.0254318685	0.8947088978	0.9999537652	-1.049373127
1.2922974312	0.623436882	0.9999537652	-1.5327036042
-1.096157347	0.6561527158	0.9999537652	-1.132448702
1.5689162209	0.2706849998	0.9999537652	1.1019812351
4.5505976667	0.5583160638	0.9999537652	-3.3741193575
-1.4839996981	0.4042717683	0.9999537652	-2.0484795942
2.3335098933	0.4437782776	0.9999537652	-1.7407464071
1.0619456367	0.7550902428	0.9999537652	1.1704445585
1.1752277064	0.2063495906	0.9999537652	1.2961039209
-2.1738281006	0.1445763008	0.9999537652	-1.2070737822
-1.1658031207	0.7514658487	0.9999537652	-1.3864382837
1.3868844561	0.0792359634	0.9999537652	1.4978801849
-1.0154374517	0.9170280963	0.9999537652	1.304002562
-1.1523625851	0.5059877632	0.9999537652	1.2252125759
-1.2444688318	0.2489349744	0.9999537652	-1.1877098512
2.0215345212	0.3443233919	0.9999537652	1.0748763975
3.6433090331	0.0212075534	0.9999537652	1.2329802688
-1.0307866248	0.9620463197	0.9999537652	1.0281463992
1.0295170701	0.8495341074	0.9999537652	1.0733745753
-1.0818792194	0.6664256033	0.9999537652	1.0942609207
1.1363165238	0.4993429781	0.9999537652	-1.1847139903
-1.272585976	0.4460948253	0.9999537652	1.1589797287
-1.051463187	0.814624741	0.9999537652	1.1024264461
-1.2274198939	0.1098533592	0.9999537652	-1.0867782361
-1.0304210628	0.8546703948	0.9999537652	1.2230812928
-1.000156736	0.9990642158	0.9999537652	1.4921326223
1.7634848916	0.0780312672	0.9999537652	-1.0275493049
-1.2517429193	0.114674507	0.9999537652	-1.3831684571
1.5320677021	0.2882698089	0.9999537652	1.7378131584
1.0001757323	0.9990483488	0.9999537652	1.0947110595
-1.0229584176	0.8463992578	0.9999537652	1.0239969943
13.3324809638	0.3187276287	0.9999537652	-1.3099965392
-1.030375307	0.8672486983	0.9999537652	1.059753782
-1.097664026	0.5502596136	0.9999537652	1.1736949744
-1.6411031698	0.0147356282	0.9999537652	1.1924288531
1.1973641932	0.1853613335	0.9999537652	1.2505805198

-1.2885050132	0.178517828	0.9999537652	1.0009128106
1.6853566275	0.2021282027	0.9999537652	1.7321858005
1.5403995586	0.4942526665	0.9999537652	-1.2656159229
1.0311205031	0.8235790876	0.9999537652	1.0218920299
3.3088597104	0.2907670152	0.9999537652	1.6471944927
-1.005190037	0.9786400993	0.9999537652	1.1801881468
-1.1911833595	0.1360568632	0.9999537652	-1.045717365
-1.003225651	0.9863250408	0.9999537652	1.2984302006
-1.5875118025	0.7205923019	0.9999537652	-1.1254032646
1.0482732744	0.7956280461	0.9999537652	1.442581964
4.5505976667	0.5582530147	0.9999537652	1.2593062028
1.0765326296	0.544855869	0.9999537652	1.2494807965
1.223819637	0.2291349325	0.9999537652	1.0911408377
1.0413081868	0.8287865988	0.9999537652	-1.0855091452
-1.161430603	0.8784010043	0.9999537652	-1.1562397956
-6.9736190158	0.2250321147	0.9999537652	-5.3583704783
9.8205505243	0.380840349	0.9999537652	1.5311196755
1.267115312	0.4711718241	0.9999537652	1.0103916121
-1.1525214631	0.4649789513	0.9999537652	1.2031386065
1.2791605412	0.5723921011	0.9999537652	-1.0348230647
1.3898813682	0.4008838065	0.9999537652	1.0383384988
1.1056911288	0.6254564422	0.9999537652	1.1825045398
1.251621044	0.6633633619	0.9999537652	1.0768129053
-1.0199071928	0.9641599725	0.9999537652	1.1296795182
1.0552787177	0.7617154161	0.9999537652	1.107176084
1.0453452172	0.8040567394	0.9999537652	1.1067505722
1.0361536905	0.8096831687	0.9999537652	1.0893051629
-1.1540333603	0.6980171334	0.9999537652	1.3341877854
-1.0794534027	0.8622227259	0.9999537652	1.2395256026
2.9455692854	0.2506589786	0.9999537652	1.1649025164
1.0359855881	0.8054575669	0.9999537652	1.2196941538
8.064174035	0.4240336506	0.9999537652	12.7732027325
-1.2380953167	0.375752925	0.9999537652	1.2516896556
3.3030568913	0.2924051351	0.9999537652	1.3524832521
-2.6939394783	0.055964976	0.9999537652	-1.9134251124
0	0	0	1.590997316
1.1677076967	0.2363691601	0.9999537652	-1.1803723473
1.1141438549	0.7739256145	0.9999537652	-1.0269431281
2.7957660672	0.7054104954	0.9999537652	0
-6.9736190158	0.2250321147	0.9999537652	6.9133480437
1.0101562266	0.9563857453	0.9999537652	1.172226521
1.1258807493	0.8274596914	0.9999537652	1.1904333214
1.2569286632	0.4708436512	0.9999537652	1.6437272007
-3.9215158983	0.008582	0.9999537652	1.0718602436
1.066045755	0.8628794928	0.9999537652	1.3262779288
-1.0733333034	0.6209825436	0.9999537652	1.0668806596
-1.078395208	0.758988149	0.9999537652	1.0297715698
1.1210965818	0.615269012	0.9999537652	-1.0588590735
1.045800646	0.7980389604	0.9999537652	1.189909816

-1.2123808693	0.634676617	0.9999537652	-1.3033454715
-3.5762417122	0.0565212365	0.9999537652	-1.9669634371
-1.0322777638	0.8837704544	0.9999537652	1.2300622311
1.5801796798	0.152917217	0.9999537652	-1.0198510119
-1.0824110166	0.570533428	0.9999537652	1.1302993515
1.14839599	0.3737651183	0.9999537652	1.0409062867
0	0	0	2.996475627
1.2640282506	0.3076123179	0.9999537652	1.0441811362
1.2810033213	0.3919221315	0.9999537652	1.0453848234
50.1857468784	0.1243843017	0.9999537652	2.1222754974
1.8630879566	0.6335231072	0.9999537652	1.2397607065
-1.1395639687	0.4888899096	0.9999537652	1.1972295133
27.3932715777	0.197482392	0.9999537652	2.6940349656
-1.3813923947	0.4839809798	0.9999537652	-1.3657603362
1.1788909975	0.3836650913	0.9999537652	-1.1434333717
-1.3391564615	0.6615202665	0.9999537652	1.2783607527
1.3092955734	0.1839755236	0.9999537652	1.0251623935
-1.0545696323	0.6901013043	0.9999537652	1.2377892869
1.3569373401	0.3223644107	0.9999537652	-1.0833288836
1.030004704	0.7938435542	0.9999537652	1.2340058613
-1.0583197908	0.6553839162	0.9999537652	1.1280225449
-1.1037584049	0.4819030095	0.9999537652	1.3240350194
1.3551194596	0.6392288335	0.9999537652	-1.5222509939
-1.1167245966	0.6358810949	0.9999537652	1.0706614599
-1.0667502471	0.8601191448	0.9999537652	1.0575599908
1.1303461451	0.5106746627	0.9999537652	1.2202302902
-1.1859709831	0.5771238034	0.9999537652	1.3473969921
1.058219814	0.7688706394	0.9999537652	1.1851543742
-1.0074216738	0.9789079253	0.9999537652	-1.6743503122
-1.3172599084	0.4594814912	0.9999537652	1.7769750169
-1.0092908548	0.9572458139	0.9999537652	1.2590998485
-1.1371677032	0.3594311832	0.9999537652	1.157767079
1.2287729562	0.4484940492	0.9999537652	1.0170875148
1.5090449182	0.3503203014	0.9999537652	1.2348393609
1.3150692508	0.1676436074	0.9999537652	1.1086181017
1.0138213848	0.944721706	0.9999537652	1.2202682297
1.0841390398	0.7912039592	0.9999537652	1.0132218493
-1.161001029	0.5451100332	0.9999537652	1.7401841879
-1.0329658574	0.8824878845	0.9999537652	-1.1727110236
1.0260370961	0.9581725913	0.9999537652	-1.3133336704
1.2039644173	0.4821575665	0.9999537652	1.1873279498
-1.1619717093	0.8742377907	0.9999537652	-1.1446993292
-1.6118021191	0.1271792192	0.9999537652	-1.1600083213
-1.2152885933	0.5840786338	0.9999537652	-1.1107648757
2.7933974408	0.6948713406	0.9999537652	-2.2257756135
-1.0998935066	0.7943292286	0.9999537652	1.6977523584
0	0	0	-1.1248877899
1.2226446453	0.6279738043	0.9999537652	1.5917227281
-5.4573937553	0.0302926077	0.9999537652	-1.3657600172

-1.3253156225	0.1065876722	0.9999537652	1.2623357232
-1.0116548238	0.9350114087	0.9999537652	1.0812438028
1.1567773451	0.561829627	0.9999537652	1.1737626996
1.0481015402	0.9024854325	0.9999537652	-1.0381057738
-1.9095359099	0.109736308	0.9999537652	1.4158562612
1.3354181433	0.1757843497	0.9999537652	-1.2267145309
-1.1332932171	0.8338881712	0.9999537652	-1.7032006509
1.003883175	0.9755488749	0.9999537652	1.1167538524
-1.0758170349	0.7634161678	0.9999537652	1.1874743817
1.1277691601	0.7674569686	0.9999537652	-1.2025447152
1.8400912182	0.3191027864	0.9999537652	1.3176900479
-1.4208363008	0.1579929172	0.9999537652	1.1505900379
1.1125683164	0.6295560649	0.9999537652	1.5340625254
1.1738319622	0.2047657435	0.9999537652	1.216866972
1.7002236452	0.281275669	0.9999537652	1.0309277703
6.3075231393	0.4869753402	0.9999537652	-1.3095105362
1.0860562686	0.6925009202	0.9999537652	1.1017263253
-1.0439858303	0.9249190988	0.9999537652	1.4103835193
-2.2249719371	0.0108973053	0.9999537652	-1.2731411559
1.2986022509	0.1515148823	0.9999537652	1.3544323277
1.8506577326	0.043698759	0.9999537652	-1.1037129679
1.0596933394	0.8779706269	0.9999537652	-1.2767601004
1.4820785793	0.6115468577	0.9999537652	-2.0137065482
-1.1940150405	0.3587783604	0.9999537652	1.1539226643
1.140553923	0.6227044993	0.9999537652	1.1734390491
0	0	0	12.7732027325
-1.0531838867	0.8162927034	0.9999537652	1.0366246806
1.4580514267	0.4302251493	0.9999537652	-1.0843710368
1.0105397644	0.9556398671	0.9999537652	1.3204262674
-1.1162443808	0.8322481261	0.9999537652	1.1758729474
-1.1757141981	0.8163603748	0.9999537652	1.5535398187
1.2045695872	0.3520233776	0.9999537652	-1.0579234063
1.3026120258	0.7419394862	0.9999537652	1.4921254839
1.0207704433	0.8971301718	0.9999537652	-1.1116659376
-1.1414188877	0.8119548858	0.9999537652	-1.3413401502
1.2765725207	0.4460767873	0.9999537652	1.9175744387
-1.3465581125	0.552974065	0.9999537652	-1.0766624679
0	0	0	0
1.03027242	0.8144767173	0.9999537652	1.0619488531
-6.9736190158	0.2250321147	0.9999537652	4.9527501949
-1.0271646873	0.948943424	0.9999537652	1.9870000726
-1.0135973112	0.9048691625	0.9999537652	1.0460668524
1.3903023218	0.1934120009	0.9999537652	-1.3897039857
4.5505976667	0.5581917296	0.9999537652	0
6.3128691894	0.4819234968	0.9999537652	-1.4728350147
-1.0727609084	0.8156721765	0.9999537652	1.0278217942
1.1282078904	0.3648449436	0.9999537652	1.1209291596
0	0	0	-1.4671767304
-1.2147312824	0.1369595011	0.9999537652	1.0331078259

1.0095636091	0.9502098008	0.9999537652	-1.1143538443
1.1087515229	0.8129797173	0.9999537652	1.038863527
4.0401294047	0.1919830592	0.9999537652	-2.5054827272
27.3923972417	0.1964503009	0.9999537652	-1.9178288323
1.035192206	0.8551984834	0.9999537652	1.1385985186
0	0	0	-2.1685158072
-1.2561586628	0.2892683759	0.9999537652	1.1787864514
1.0554694455	0.7464126477	0.9999537652	1.0280072859
1.1254904498	0.868834698	0.9999537652	1.3740474488
-1.2985324355	0.5416642819	0.9999537652	-1.0947578512
1.2958082392	0.3716357545	0.9999537652	-1.1288486095
1.5130808007	0.6581389655	0.9999537652	2.9978319446
1.2240090143	0.5382183742	0.9999537652	-1.7191465338
-1.2631157829	0.3151974122	0.9999537652	-1.2646640326
2.7933974408	0.6948713406	0.9999537652	-5.4409910544
-1.4094813119	0.4085009965	0.9999537652	-2.4330476915
-2.1317846121	0.464748747	0.9999537652	-1.5243363277
-4.1053393351	0.1096088376	0.9999537652	-1.7948605068
1.0817237071	0.8551786859	0.9999537652	1.2718037447
2.7933974408	0.6948713406	0.9999537652	0
1.1032793086	0.5968085246	0.9999537652	-1.2772393174
-1.0418792254	0.8418212978	0.9999537652	-1.1256928502
-1.1558758044	0.430189498	0.9999537652	-1.2114550843
2.9944437113	0.1614341308	0.9999537652	-1.1788803767
1.4860705144	0.1576785593	0.9999537652	-1.4926140709
2.3097704908	0.020711205	0.9999537652	1.0865197787
3.3114514989	0.2937601664	0.9999537652	1.8474131398
1.1204373452	0.6567437196	0.9999537652	1.0801961346
-1.0491370297	0.707772094	0.9999537652	-1.2229886814
-1.6733656555	0.1187970193	0.9999537652	-1.010526951
0	0	0	3.1913304638
1.1402567378	0.4912097448	0.9999537652	-1.2236670793
0	0	0	2.9988886918
-1.5875118025	0.7206200909	0.9999537652	0
-6.9764533263	0.259053074	0.9999537652	-1.5763711172
1.1750989557	0.7277280591	0.9999537652	-2.0649908954
9.8205505243	0.3808402052	0.9999537652	-1.144309199
-1.2893763188	0.3840196263	0.9999537652	1.1326198253
-1.0568432489	0.9498385621	0.9999537652	-2.5663005569
2.7933974408	0.6948713406	0.9999537652	0
-1.0260851178	0.9393087011	0.9999537652	-1.1445768905
-3.9839141918	0.0994854876	0.9999537652	1.9432271302
-1.1411720019	0.3984779253	0.9999537652	-1.1580868739
1.003274034	0.9868547831	0.9999537652	1.300981969
-1.2252746757	0.5669367796	0.9999537652	1.4159900243
1.3137214689	0.2660352841	0.9999537652	1.0407557179
1.2321037674	0.3502609252	0.9999537652	1.4126742545
1.0673525149	0.7268465479	0.9999537652	-1.0461598799
1.0632895484	0.7168778537	0.9999537652	1.1548697487

1.8494309744	0.0124988136	0.9999537652	1.0585565708
0	0	0	-3.1516923425
-1.8773533293	0.2045219025	0.9999537652	-3.2889322436
-1.3972192265	0.2512087309	0.9999537652	1.2490339579
0	0	0	-4.3195903374
20.4048055231	0.246079472	0.9999537652	-2.579624099
-1.0348720175	0.8426920372	0.9999537652	1.1864169507
-1.5014101828	0.0931797002	0.9999537652	1.2147010314
-1.2863901983	0.5895187731	0.9999537652	1.8978804472
0	0	0	2.996475627
2.4216796225	0.4425859126	0.9999537652	-5.3583704783
1.0933476234	0.5766174706	0.9999537652	1.1038848703
0	0	0	-5.0960079851
-1.0265069324	0.9366443631	0.9999537652	-1.1264295403
2.5766519373	0.3876342521	0.9999537652	-1.1613446338
1.0482613331	0.9171305993	0.9999537652	-1.7224004393
1.1262386404	0.5701295562	0.9999537652	1.1930563997
-1.1295449742	0.5760775667	0.9999537652	1.2501801898
1.2647301303	0.4768291636	0.9999537652	-1.1654303074
-1.1745200145	0.5143347864	0.9999537652	1.1660617042
-1.080358607	0.5984886236	0.9999537652	1.0090087368
1.0580476554	0.6408378458	0.9999537652	-1.2894960279
0	0	0	6.908559683
1.0100314058	0.9347893395	0.9999537652	-1.0160970018
-1.0237573864	0.8409906768	0.9999537652	1.079085824
1.1869395158	0.5858755113	0.9999537652	1.0178076744
-1.0011733583	0.9960520473	0.9999537652	-1.1211876898
-2.2920785558	0.0304938839	0.9999537652	-1.0036376062
0	0	0	2.996475627
-1.369107775	0.7698015353	0.9999537652	1.1933100121
-1.0720169699	0.6876417036	0.9999537652	1.1470758138
6.3075231393	0.4869556403	0.9999537652	1.2400291296
-1.2013925844	0.4362888778	0.9999537652	1.098002369
0	0	0	-1.3617381988
1.0967049244	0.5128419913	0.9999537652	-1.1643787204
-1.0506428809	0.8924886902	0.9999537652	-1.0455086903
2.3315363418	0.4566542164	0.9999537652	-3.382249888
1.2802408629	0.2017579673	0.9999537652	-1.1216823014
-1.0187949736	0.9569466613	0.9999537652	-1.3378731131
0	0	0	2.996475627
1.1885220664	0.3587791553	0.9999537652	-1.0155038661
2.7933974408	0.6948713406	0.9999537652	0
-1.0651579331	0.679000415	0.9999537652	1.0353632944
2.7933974408	0.6948713406	0.9999537652	-5.3589986948
1.3826309223	0.7324947448	0.9999537652	1.0669823991
2.7933974408	0.6948713406	0.9999537652	-5.3589986948
-2.9545470831	0.1381910896	0.9999537652	-2.2056994995
-1.0191619084	0.9357712783	0.9999537652	1.1468336262
2.3306483975	0.4666935134	0.9999537652	1.2763409465

1.3212876093	0.4589448151	0.9999537652	1.1469441772
1.2346032743	0.769881264	0.9999537652	1.5745887665
1.1426619303	0.281657303	0.9999537652	1.2945900793
-5.6856811034	0.0708024766	0.9999537652	1.0222616753
-1.3846532604	0.0390506095	0.9999537652	-1.0067949779
-1.1518064782	0.5433812731	0.9999537652	1.2336874045
-6.9764533263	0.2590613606	0.9999537652	1.9409927546
2.7933974408	0.6948713406	0.9999537652	-2.5222250873
1.0702762257	0.8655447696	0.9999537652	-1.3324877564
-1.1009757193	0.6143019146	0.9999537652	-1.1981424176
-1.16556239	0.2548259605	0.9999537652	-1.7839917098
-1.0055348611	0.9858555724	0.9999537652	-1.0540126521
-1.0503749378	0.8865261346	0.9999537652	1.7006901418
1.4626324078	0.1631155282	0.9999537652	-1.4027096298
-1.4730496058	0.6732334461	0.9999537652	-3.0614198881
0	0	0	14.7373490367
-1.0692287614	0.7633988418	0.9999537652	-1.2546382484
-1.0117429258	0.9349354386	0.9999537652	-1.1014096766
1.4445739763	0.381073472	0.9999537652	-1.1648456324
-1.076896491	0.8708644127	0.9999537652	-1.0890762164
1.2481337696	0.6688274541	0.9999537652	-1.8727008913
-1.0041911222	0.989330489	0.9999537652	-1.242686304
1.723299787	0.326882136	0.9999537652	-1.2662973836
1.1635326013	0.7271969569	0.9999537652	1.114585203
0	0	0	14.7475552392
2.7933974408	0.6948713406	0.9999537652	-5.3819632848
1.3477725595	0.4399269373	0.9999537652	-1.1879753381
-1.4925372765	0.465824438	0.9999537652	2.2593124731
1.1496195342	0.6033705128	0.9999537652	-1.4150328943
0	0	0	1.4387127179
-1.0435404671	0.8176269062	0.9999537652	-1.1798832126
1.8471118167	0.5852930979	0.9999537652	-1.8588429871
-1.3490631423	0.0978929328	0.9999537652	-1.2049946809
-1.2069889858	0.612065593	0.9999537652	1.3255711295
-1.3350370223	0.1253098265	0.9999537652	-1.2048231468
1.2874792726	0.3086108591	0.9999537652	1.0529403468
-1.2472792498	0.2521246042	0.9999537652	-1.6047681636
1.101516099	0.6396713219	0.9999537652	-1.3332393553
-1.1804683847	0.5356429508	0.9999537652	-1.2983501761
1.0300692006	0.9020707217	0.9999537652	-1.3337744313
27.5316084765	0.2201932174	0.9999537652	-1.4680135518
0	0	0	2.996475627
-6.9764533263	0.2590696501	0.9999537652	4.9527501949
4.5544554512	0.5691533303	0.9999537652	-3.8916086545
1.3309197109	0.1757473688	0.9999537652	-1.188297243
1.2633807446	0.1966684808	0.9999537652	1.5401176291
2.7957660672	0.705412829	0.9999537652	-1.859280336
-1.1548762608	0.5225638973	0.9999537652	1.2488812764
1.3243506448	0.5320346529	0.9999537652	-1.2247309377

1.533161959	0.0491946337	0.9999537652	1.1977619604
-1.0717904323	0.7889708395	0.9999537652	-1.064035082
-1.237796356	0.1126891461	0.9999537652	-1.015724449
-1.188360529	0.6232964391	0.9999537652	1.3461492645
-1.5875118025	0.7206479341	0.9999537652	-1.1441751338
0	0	0	6.9133480437
-2.0024109583	0.4495787895	0.9999537652	-1.4622935059
-1.1947261676	0.3466229975	0.9999537652	-1.0233594013
-1.1021391198	0.8877864867	0.9999537652	1.2747132884
1.1449402332	0.4824849835	0.9999537652	1.1188752579
-1.0051533246	0.9725527122	0.9999537652	-1.3630846845
4.5505976667	0.5581320915	0.9999537652	-6.0051456121
1.1295538257	0.4850482306	0.9999537652	-1.0983976852
1.1246169603	0.8844676345	0.9999537652	-1.391616432
1.1242038379	0.5457442046	0.9999537652	-1.0803109059
1.5578828389	0.0220292604	0.9999537652	-1.1843267308
-1.5881570201	0.7143327638	0.9999537652	0
-1.1717840466	0.4515170202	0.9999537652	1.3173342465
-1.0256145833	0.8499474304	0.9999537652	-1.3345891775
15.0880352532	0.2953486088	0.9999537652	-1.7798895855
-1.1157917445	0.7450228049	0.9999537652	-1.5048451516
1.0140479765	0.9434313142	0.9999537652	-1.437422123
0	0	0	6.908559683
1.3706606753	0.0598083558	0.9999537652	1.0923047088
-1.9066285216	0.4112774978	0.9999537652	-1.2777730283
-6.7517740937	0.0419513922	0.9999537652	2.5722386983
1.1319091165	0.7831001171	0.9999537652	-1.3777696496
4.5505976667	0.5580739888	0.9999537652	1.2400291296
1.0909783654	0.6274446261	0.9999537652	1.0670688866
1.4975734907	0.3458065418	0.9999537652	-1.5285117081
1.6829190363	0.1390886019	0.9999537652	-1.1919250463
-1.0869804889	0.6819309838	0.9999537652	1.1525254813
1.33173066	0.028576996	0.9999537652	-1.2691742637
-1.1747670113	0.2540224687	0.9999537652	-1.0663451385
-1.4275159746	0.2759763719	0.9999537652	1.18892017
1.1696360235	0.3088325449	0.9999537652	-1.2025900524
-1.1722508884	0.8428824786	0.9999537652	1.2713908921
1.0555799377	0.7241720264	0.9999537652	-1.0901859096
2.0147014689	0.3721795711	0.9999537652	-1.1556233835
2.7933974408	0.6948713406	0.9999537652	4.9527501949
-1.1380651639	0.6066000933	0.9999537652	-1.0496882456
1.062412966	0.7447679072	0.9999537652	1.0380839978
-1.0627347782	0.7600386745	0.9999537652	-1.1898303489
1.0519687938	0.8082980864	0.9999537652	-1.1414733898
9.8205505243	0.3808400613	0.9999537652	0
-1.0278953273	0.9000976283	0.9999537652	-1.0018371968
-1.4630808444	0.1782572544	0.9999537652	1.3181249798
1.2516520703	0.8156421472	0.9999537652	1.0400262591
1.2995690157	0.171542552	0.9999537652	1.1865783268

-1.0882400522	0.7076339672	0.9999537652	1.075252377
0	0	0	2.996475627
20.4054563206	0.2419036965	0.9999537652	2.1558362933
1.0801248131	0.491649292	0.9999537652	1.2768987782
-1.1738833733	0.5226578916	0.9999537652	1.0336256636
1.0878396345	0.5273168526	0.9999537652	1.0513353475
1.8432954066	0.2236075826	0.9999537652	-1.3442663154
2.7933974408	0.6948713406	0.9999537652	0
-1.2143691758	0.3544002851	0.9999537652	-1.1655745862
1.1662222053	0.4562854682	0.9999537652	1.2436089475
-1.401904019	0.2472442398	0.9999537652	1.0794803462
-1.0186711384	0.8971204062	0.9999537652	1.1264869246
-1.0069789698	0.9695716553	0.9999537652	-1.0317026069
1.255213919	0.4875133401	0.9999537652	-1.3362378512
-1.1017553008	0.8828498074	0.9999537652	-1.0717919967
2.7933974408	0.6948713406	0.9999537652	8.8710369702
1.1007577316	0.7192817647	0.9999537652	-1.0395103963
-1.074889252	0.6327375011	0.9999537652	-1.0638087584
1.0874974211	0.7221844391	0.9999537652	-1.1641973137
1.1835739482	0.4941725174	0.9999537652	1.1899200692
-1.3053434151	0.1550000765	0.9999537652	-1.1238045626
-1.0442477743	0.8885048732	0.9999537652	1.0859108159
-1.27257017	0.3013612787	0.9999537652	1.0769881468
1.1686557743	0.7253217997	0.9999537652	-1.081402095
1.3567051147	0.2934434383	0.9999537652	1.3529685916
1.2353666622	0.5651499799	0.9999537652	-1.0273076086
-1.24184085	0.1357825302	0.9999537652	1.0678206553
0	0	0	2.9985540909
1.6093235429	0.0132432862	0.9999537652	1.4655468751
-1.0787980506	0.6666700524	0.9999537652	-1.1044661758
-1.1798239495	0.7428940477	0.9999537652	1.0089175415
-1.3127867632	0.6062290758	0.9999537652	1.1626468016
-1.1914071896	0.3957176394	0.9999537652	-1.1015222444
1.2599271245	0.6972195229	0.9999537652	1.0786082953
1.3983587352	0.0576265418	0.9999537652	1.2213058754
1.1543733253	0.4741066448	0.9999537652	1.2213990352
8.064174035	0.4240320162	0.9999537652	1.1562511914
-1.3650719983	0.2778900758	0.9999537652	1.4806806294
-1.3675201086	0.0800610462	0.9999537652	-1.0172407608
22.1075168104	0.2294608814	0.9999537652	-2.1739698775
-1.1445309719	0.7097940718	0.9999537652	-1.2262159896
-2.1075975357	0.0321670009	0.9999537652	-1.4281142667
1.0920083237	0.530870447	0.9999537652	1.2753754649
1.0802548627	0.4878635771	0.9999537652	1.1535898994
-1.4423423054	0.2431163149	0.9999537652	1.3167123989
1.1914277133	0.8117858645	0.9999537652	-2.6746008127
-1.227317217	0.1188376775	0.9999537652	-1.2176496266
1.1739489549	0.3704706621	0.9999537652	1.1727729024
1.3185287977	0.4225721752	0.9999537652	1.24422459

-3.4177105345	0.0721308729	0.9999537652	-1.102767722
1.2921120854	0.2339903854	0.9999537652	1.2267060643
9.8205505243	0.3808399173	0.9999537652	0
1.3516771602	0.1607729428	0.9999537652	1.3293516924
1.0096129644	0.9562403855	0.9999537652	1.1304867999
2.5978004522	0.277637291	0.9999537652	-1.0841692731
1.0788599929	0.774192317	0.9999537652	-1.0163230861
1.2685621488	0.0863134473	0.9999537652	1.1718520249
-2.9572400577	0.3881281056	0.9999537652	1.2400291296
-1.440762573	0.0863682673	0.9999537652	1.3046555537
-1.2124067144	0.6333881725	0.9999537652	1.2092416821
1.1492128484	0.4478258965	0.9999537652	-1.0837443005
1.0998136985	0.4725090168	0.9999537652	-1.0782430267
1.1158451632	0.5531193435	0.9999537652	1.3819985049
-1.0012407479	0.9958769486	0.9999537652	-1.0616573598
-1.1290947109	0.8331932331	0.9999537652	1.1305195109
1.1244438725	0.4036630742	0.9999537652	-1.0784962119
-1.1466990632	0.3550906332	0.9999537652	-1.1604670357
4.7614107123	0.1436014112	0.9999537652	-4.7163364444
-1.1645887681	0.283792707	0.9999537652	1.0506505876
-1.1215916118	0.5345978191	0.9999537652	-1.0678385594
1.0453445941	0.7560709922	0.9999537652	1.4176145398
1.2768593149	0.6323521332	0.9999537652	1.317164407
1.0113371931	0.9552229391	0.9999537652	1.0483733949
-1.0683830416	0.7239610195	0.9999537652	-1.199627656
1.0442984967	0.8634975451	0.9999537652	-1.0345958421
1.1037494409	0.4771034464	0.9999537652	1.0778311073
6.2246906171	0.0773126051	0.9999537652	-1.3718108746
1.0564165883	0.92058328	0.9999537652	-1.0370232947
-1.081012484	0.7021624299	0.9999537652	-1.0460724035
-1.6377847339	0.3273846753	0.9999537652	-1.592297434
1.6246623472	0.6510231429	0.9999537652	-1.4429295936
2.3326953621	0.4514643039	0.9999537652	3.1865842129
1.0638877532	0.7576345508	0.9999537652	1.0612499777
1.1794321532	0.4258584282	0.9999537652	-1.0073229861
-1.1368516183	0.5569782897	0.9999537652	1.0147499216
0	0	0	2.2924151284
-1.2597567125	0.2704320998	0.9999537652	-1.0347217198
1.922991318	0.1255960091	0.9999537652	1.362158545
1.3553688224	0.2661285345	0.9999537652	1.2032425627
-1.4756779277	0.6448525036	0.9999537652	1.394244722
2.7933974408	0.6948713406	0.9999537652	-1.8594983184
2.7933974408	0.6948713406	0.9999537652	-1.6599272513
1.5051934407	0.1804255097	0.9999537652	1.1916968684
-1.0693000945	0.8288362771	0.9999537652	-1.1998618237
6.3075231393	0.4869457937	0.9999537652	-1.2686400182
-1.3445680738	0.372349338	0.9999537652	-1.1268479345
2.7933974408	0.6948713406	0.9999537652	2.996475627
-1.9092068046	0.2147738672	0.9999537652	1.9531401374

-1.031455842	0.8546947883	0.9999537652	-1.250079026
1.1616975475	0.2269343045	0.9999537652	1.0861474191
-2.1307903719	0.4720844383	0.9999537652	1.3545358667
1.0871941419	0.6866437355	0.9999537652	1.2317975363
0	0	0	-1.7821643145
-1.0807774387	0.5366111533	0.9999537652	1.0709330902
2.4330469542	0.3069239716	0.9999537652	1.4413839312
1.3588576256	0.8006552884	0.9999537652	2.0824386352
1.0016019147	0.9962278894	0.9999537652	1.4080056231
-1.107492687	0.4839843171	0.9999537652	1.0933086642
-1.1293058762	0.7122271104	0.9999537652	1.2596904875
-1.10949614	0.5902593743	0.9999537652	-1.0535176252
1.2221798058	0.3318411053	0.9999537652	1.2158060398
-1.0790790138	0.690051948	0.9999537652	1.131597295
1.1930433859	0.3104405349	0.9999537652	1.1410183531
1.3180633219	0.4151269929	0.9999537652	1.0933396906
41.4235246733	0.1435027196	0.9999537652	1.3236157985
1.1362156476	0.3349083945	0.9999537652	1.4850410783
1.7343571244	0.4255612591	0.9999537652	1.8954581254
-1.0085170112	0.991234555	0.9999537652	-2.377796608
1.3302832209	0.1780130593	0.9999537652	-1.0616604374
1.2414529377	0.3941776292	0.9999537652	1.0357921779
-1.2065250574	0.5754375187	0.9999537652	-1.2032930043
-1.5844926328	0.2928898311	0.9999537652	-1.0037720183
4.5544554512	0.5691513355	0.9999537652	2.996475627
1.066114904	0.7790125986	0.9999537652	1.1878512321
-1.4218851597	0.2021505194	0.9999537652	-1.3619244823
1.4125493175	0.47515701	0.9999537652	1.3870656775
8.064174035	0.4240303822	0.9999537652	4.126189409
1.0400521022	0.9177764795	0.9999537652	1.2276228864
-2.5802899275	0.1924459151	0.9999537652	1.2763856823
1.2527770819	0.1066714357	0.9999537652	1.1312379668
1.1224328358	0.5916307399	0.9999537652	1.0107264157
1.3713677664	0.3053737283	0.9999537652	-1.1459184691
0	0	0	2.2217890137
-1.1604679895	0.2940377987	0.9999537652	-1.1506336998
-1.1921357948	0.2759612186	0.9999537652	-1.1100726194
1.3941900765	0.6650574701	0.9999537652	2.2834391572
3.0768986443	0.1959607209	0.9999537652	1.0382391686
-1.1555924555	0.4341371267	0.9999537652	-1.184213977
1.0216276708	0.9346436078	0.9999537652	-1.0535711679
1.3457117447	0.2805936965	0.9999537652	-1.000820421
1.0267631725	0.8452280229	0.9999537652	-1.1514684104
0	0	0	8.8639046058
-1.1601290564	0.516889599	0.9999537652	-1.1278152979
1.3885632347	0.4929415852	0.9999537652	1.0063324498
2.2471394157	0.0189373217	0.9999537652	1.4150427547
2.7957660672	0.7054151637	0.9999537652	1.2588770129
-2.0025825252	0.4567145214	0.9999537652	15.7262184124

-2.0033446699	0.4339646293	0.9999537652	-1.144309199
2.5603529545	0.2532101709	0.9999537652	-1.8594983184
-1.5881166222	0.7239157085	0.9999537652	1.4517758292
2.7933974408	0.6948713406	0.9999537652	0
0	0	0	-1.7246537898
1.2647653865	0.1570837287	0.9999537652	1.0510027534
1.17415232	0.4340552989	0.9999537652	-1.1272227054
-1.1453283303	0.4586340933	0.9999537652	1.001533894
-1.0109410966	0.9645016042	0.9999537652	1.3736676544
1.0854325391	0.7432343103	0.9999537652	-1.1825488267
1.0157484293	0.9584759501	0.9999537652	-1.4315187639
-1.0584868311	0.7109160884	0.9999537652	-1.0332508069
-1.066643349	0.7879640303	0.9999537652	1.2679822722
-17.0527724669	0.1064674885	0.9999537652	23.5244756139
-1.0771318433	0.5961924429	0.9999537652	-1.1969186018
-1.0806525169	0.6186272643	0.9999537652	-1.1376608345
-1.0670395817	0.6906317793	0.9999537652	-1.0384683378
-1.0923861226	0.5300534522	0.9999537652	-1.2921474481
1.1162834585	0.9271558935	0.9999537652	-1.1635587355
1.0279946931	0.9485359441	0.9999537652	-1.2218815242
-1.2262938314	0.5088047563	0.9999537652	-1.3316817021
4.5505976667	0.5580173132	0.9999537652	-9.7554110723
0	0	0	1.5908108123
-1.0379260793	0.9585357071	0.9999537652	-1.3078946307
1.1185766111	0.5261353187	0.9999537652	1.2652835739
-1.0610340974	0.6352119047	0.9999537652	1.165299022
1.1183928359	0.7551947418	0.9999537652	-1.6297897138
1.3952142421	0.3136682314	0.9999537652	-1.1019946937
-1.0776652847	0.6300458984	0.9999537652	-1.4437596553
1.7532109063	0.115603547	0.9999537652	-1.238353717
-3.1291666749	0.0323045313	0.9999537652	-1.1738675494
-1.1761859055	0.2807690586	0.9999537652	-1.237028424
-6.9762758667	0.253866474	0.9999537652	0
1.2571214932	0.5072000291	0.9999537652	-1.1059553934
1.9050337421	0.4951423099	0.9999537652	-1.5885342441
-1.7667103191	0.0516754793	0.9999537652	-1.229096511
1.108572056	0.6372818569	0.9999537652	-1.2760778257
-1.0073959536	0.9552518798	0.9999537652	-1.1140168664
-1.0927147006	0.8102482266	0.9999537652	1.6895558503
-1.3917747917	0.6966176058	0.9999537652	1.2763409465
-1.1162201988	0.4428337072	0.9999537652	-1.2254009866
0	0	0	1.6460548351
74.7406392012	0.0902614905	0.9999537652	-2.331907554
1.121447126	0.5192161512	0.9999537652	-1.1111108703
1.7635124906	0.008027	0.9999537652	-1.13989203
-1.121495293	0.504863659	0.9999537652	-1.1147231155
1.0254725855	0.9116949242	0.9999537652	-1.041905817
1.0225186495	0.8799908906	0.9999537652	1.1147364043
-1.3365311834	0.2589043729	0.9999537652	-1.1252678765

1.5708934584	0.0639454711	0.9999537652	-1.0607646103
0	0	0	-1.1508383804
2.7933974408	0.6948713406	0.9999537652	4.9527501949
22.1075168104	0.2294502413	0.9999537652	-1.5297710554
1.3149506351	0.6126934083	0.9999537652	1.9360947879
-1.1202350469	0.5247359333	0.9999537652	1.1210033521
1.096561783	0.9223104206	0.9999537652	1.0991765388
-1.0032480039	0.9808188243	0.9999537652	1.089455734
1.0960528897	0.454923575	0.9999537652	-1.1091269249
1.1751994235	0.604872244	0.9999537652	-1.5168971076
2.3849251739	0.1342034118	0.9999537652	-1.8602423201
1.3865458338	0.3120228366	0.9999537652	-1.0956757346
-1.0687261513	0.8067809387	0.9999537652	-1.2195832731
1.1487009072	0.3427191929	0.9999537652	-1.2315562255
1.8883256627	0.2298542657	0.9999537652	-1.0781260401
1.3533738682	0.2072420923	0.9999537652	-1.2497470341
3.7523729726	0.0228427497	0.9999537652	-1.037692948
1.1297591131	0.7740804817	0.9999537652	1.1531578644
-1.0957129989	0.6679741783	0.9999537652	-1.1184962775
0	0	0	2.996475627
-1.2690039376	0.3510550613	0.9999537652	-1.564877706
1.3671044512	0.5766577112	0.9999537652	-1.3150685428
-1.4054122033	0.2746591465	0.9999537652	1.0105456338
-1.1614998763	0.8773192096	0.9999537652	1.4044603321
4.5505976667	0.5579619574	0.9999537652	2.996475627
2.034148039	0.0298009468	0.9999537652	1.8925242337
-1.0560631224	0.7876252281	0.9999537652	-1.4243489174
-1.0257731938	0.8832127477	0.9999537652	-1.396063674
-1.0819600142	0.9162018126	0.9999537652	1.8800448668
-1.059763157	0.6562260482	0.9999537652	-1.2091630533
2.7933974408	0.6948713406	0.9999537652	9.7116702472
-1.1665877442	0.3067627174	0.9999537652	-1.2715614061
-1.1736489462	0.5486352989	0.9999537652	-1.0066599369
5.3380256565	0.0156260469	0.9999537652	-1.2497649194
-7.9898319344	0.1016094332	0.9999537652	-1.6583880401
-1.3943722837	0.2810161615	0.9999537652	-1.3430625169
1.0923252707	0.6273565521	0.9999537652	-1.14433415
1.2295632006	0.2271871332	0.9999537652	-1.258471424
1.8448460942	0.5998287668	0.9999537652	1.1432021086
1.3739897277	0.068115637	0.9999537652	-1.0352370402
1.158980172	0.5161906027	0.9999537652	-1.1968846508
-1.2476608323	0.4103709005	0.9999537652	1.1698301311
1.0349118986	0.9686502377	0.9999537652	2.0333774675
-1.1282287082	0.809946751	0.9999537652	1.1754629295
1.7269704105	0.0883616283	0.9999537652	1.0253547832
-1.0219105051	0.9393650362	0.9999537652	1.007761306
-1.0952452944	0.5299035712	0.9999537652	-1.2113266497
-1.2001233705	0.2622684935	0.9999537652	-1.042434304
-1.0183979066	0.9260254173	0.9999537652	1.1566994192

-1.198195383	0.4959201598	0.9999537652	-1.0606110772
-1.4030428403	0.0724612686	0.9999537652	-1.2136864031
1.2296435103	0.3851019127	0.9999537652	-1.1438542276
1.3690053512	0.111283279	0.9999537652	-1.0525401628
1.3864669654	0.3394864491	0.9999537652	1.0642466534
1.0008393685	0.9982239774	0.9999537652	-1.3758941261
1.1270368536	0.5878964781	0.9999537652	-1.3781556017
-1.1413158663	0.5920147714	0.9999537652	-1.1157738555
37.88539235	0.1565996658	0.9999537652	26.4411849241
1.2156533336	0.5248574419	0.9999537652	-1.6081792972
-1.0619382556	0.6125019144	0.9999537652	-1.0643066081
-1.4577097056	0.1143646838	0.9999537652	-1.0517559564
1.1905159456	0.3655253456	0.9999537652	-1.1385879714
-1.1092580418	0.5965093906	0.9999537652	-1.354750432
2.9119618468	0.1763896963	0.9999537652	6.707469253
-1.0011916999	0.9949560529	0.9999537652	-1.0495031741
1.0479923404	0.9389039023	0.9999537652	-1.4906613952
-1.0677096669	0.609517423	0.9999537652	1.0663484326
1.0095442985	0.9845388759	0.9999537652	-1.5880330355
1.4429296921	0.5032933253	0.9999537652	-1.767251894
1.0554462378	0.7825083483	0.9999537652	-1.0443113605
1.0453838347	0.8271625761	0.9999537652	1.0328079488
-1.72984743	0.164721529	0.9999537652	-1.0523531675
-1.0874203217	0.6907193161	0.9999537652	-1.2412605831
1.1076035217	0.7647017847	0.9999537652	-1.265919561
-1.1762678581	0.2620300614	0.9999537652	1.0163970146
1.0061021724	0.9705245818	0.9999537652	1.0584477901
1.0990216257	0.4967736937	0.9999537652	1.2905892642
1.288858212	0.2150198825	0.9999537652	1.201666433
-1.085738098	0.5301478823	0.9999537652	1.1119377878
1.0394195103	0.8609192306	0.9999537652	1.2256613922
3.4767359435	0.0267163168	0.9999537652	1.3864139466
-12.9852817769	0.1147836011	0.9999537652	-1.8253078054
-1.1395883354	0.5081418135	0.9999537652	1.0399149366
1.270881016	0.4249577277	0.9999537652	-1.3025507027
-1.008469205	0.9935810449	0.9999537652	-6.0729310777
1.1398257343	0.5036738	0.9999537652	1.0034738992
1.0444695573	0.8610315331	0.9999537652	1.3187975868
1.5363100135	0.0008527	0.9999537652	-1.0181838004
-1.1459811318	0.5203393478	0.9999537652	1.1909725355
4.5505976667	0.5579078161	0.9999537652	10.8262784008
-1.2396398271	0.6548980824	0.9999537652	1.2080752003
-1.1435108721	0.4851994266	0.9999537652	1.4973084741
1.0473636192	0.8147014548	0.9999537652	1.380553022
-1.009681737	0.957387029	0.9999537652	1.1437374984
0	0	0	4.9527501949
1.3243004386	0.1840623748	0.9999537652	1.0620776654
-1.0016468537	0.9945574887	0.9999537652	-1.1331938418
1.4862493016	0.1305295567	0.9999537652	1.2167195052

1.2606170699	0.7467425389	0.9999537652	-1.2762710378
-1.0085355311	0.9930274221	0.9999537652	6.7469554847
-1.279760684	0.4417888989	0.9999537652	-1.1120738424
-1.1732230158	0.3698777128	0.9999537652	1.1294203839
1.1301468848	0.4878364901	0.9999537652	1.0509668345
-1.2783650587	0.0823788055	0.9999537652	1.1126129626
2.1376103502	0.2145050301	0.9999537652	1.8671030839
-1.1926358913	0.3914649458	0.9999537652	1.1601003761
1.1806192557	0.2654571496	0.9999537652	-1.2399849193
2.7933974408	0.6948713406	0.9999537652	0
2.7957660672	0.7054174994	0.9999537652	2.996475627
-1.0565714786	0.8363427098	0.9999537652	1.0828596188
1.0856152202	0.7281121749	0.9999537652	-1.1120640616
-1.1614097668	0.3485184741	0.9999537652	1.0631226698
-1.3621920484	0.0405352221	0.9999537652	1.3013555217
-2.1245975834	0.207144066	0.9999537652	1.1381268463
-1.0202683945	0.964543078	0.9999537652	1.0185827607
0	0	0	2.996475627
2.7933974408	0.6948713406	0.9999537652	-3.041999427
8.064174035	0.4240287484	0.9999537652	2.996475627
1.4007542765	0.6400421221	0.9999537652	1.5431835469
-1.2403987282	0.1333629274	0.9999537652	1.0755240756
1.1158583321	0.9297250264	0.9999537652	1.4067915718
0	0	0	0
-1.0160526977	0.9120035799	0.9999537652	1.3734949884
1.331919152	0.2319828673	0.9999537652	1.2423153014
1.1997507743	0.3364210972	0.9999537652	1.3706479508
8.064174035	0.4240271148	0.9999537652	10.8337816869
-1.5467714784	0.4110922294	0.9999537652	-1.3808948998
-1.3673139529	0.7635965496	0.9999537652	-1.5073881707
-1.4106961981	0.1051859254	0.9999537652	-1.0003425849
2.3737552332	0.2446495601	0.9999537652	1.7808322773
-1.0969197434	0.8159830747	0.9999537652	-1.0754168758
1.1904657605	0.3641545068	0.9999537652	1.3632184647
-1.391716287	0.3433888257	0.9999537652	1.160111376
1.9471359044	0.0148763596	0.9999537652	1.2568111805
1.0890761302	0.5985788091	0.9999537652	1.4249006961
1.1628621834	0.340014116	0.9999537652	1.3225988361
-1.3050140938	0.4739802318	0.9999537652	1.0285858458
-1.5743983764	0.3044546883	0.9999537652	1.4674995293
-1.1536291367	0.3713029403	0.9999537652	-1.1515336279
-1.0462278488	0.7965403152	0.9999537652	1.114382676
-1.2475151308	0.4540136069	0.9999537652	1.3834546127
-1.1155159907	0.5753425947	0.9999537652	1.05589636
-1.2856821655	0.1783973495	0.9999537652	-1.0006069154
-1.0142477981	0.9835465205	0.9999537652	-1.299636469
-1.037087901	0.7882868401	0.9999537652	1.1207970828
2.7933974408	0.6948713406	0.9999537652	2.996475627
1.603906631	0.6805122765	0.9999537652	1.9347708844

9.8205505243	0.3808397734	0.9999537652	-1.701433652
-1.0552948741	0.7089127195	0.9999537652	1.2113283328
-1.0089452257	0.9925445593	0.9999537652	-1.2358596711
1.0028817055	0.9883552948	0.9999537652	-1.0504011122
0	0	0	2.996475627
2.7933974408	0.6948713406	0.9999537652	-1.4686861114
-1.2190851278	0.2844719609	0.9999537652	-1.025860876
-1.4190701167	0.1412615685	0.9999537652	1.0628214622
-1.0410255504	0.8429548836	0.9999537652	1.2871328815
0	0	0	4.9527501949
1.5773769852	0.2543259556	0.9999537652	1.0041611919
1.4508486595	0.2584518573	0.9999537652	1.8984175443
-1.6425083755	0.1718528299	0.9999537652	-1.5635062569
-1.026120188	0.956984998	0.9999537652	1.1177948481
1.4358565683	0.4882930092	0.9999537652	-2.289224136
1.8841048031	0.3818416655	0.9999537652	-1.2810613201
1.153523318	0.7748544789	0.9999537652	1.4680647468
-1.0732711947	0.7150019683	0.9999537652	1.198283077
0	0	0	0
-1.0369526723	0.8794477607	0.9999537652	1.2378159073
1.1366214264	0.6229028616	0.9999537652	1.3233448108
-1.124642383	0.5898544516	0.9999537652	1.1127106533
-1.2443703945	0.3713577277	0.9999537652	-1.0861542894
0	0	0	9.7116702472
1.0523998499	0.7040782092	0.9999537652	1.2034554223
1.0936472057	0.7459326692	0.9999537652	1.2207818039
1.2469835681	0.2648557894	0.9999537652	1.4962757462
-1.1144665121	0.4828094965	0.9999537652	1.0525164226
8.064174035	0.4240254816	0.9999537652	-3.6579608117
11.5766527773	0.3455597772	0.9999537652	-1.0101500723
2.7933974408	0.6948713406	0.9999537652	6.914120153
1.0506286177	0.7301171223	0.9999537652	1.2546702541
4.5505976667	0.5578027471	0.9999537652	2.996475627
-1.0239566571	0.9304545966	0.9999537652	1.2083115563
1.1025661277	0.5327864216	0.9999537652	1.6175594145
1.0546290961	0.6357355779	0.9999537652	-1.0018920057
1.1042168676	0.628219332	0.9999537652	1.2848202536
1.3243181014	0.0323560678	0.9999537652	1.3574533881
1.570657352	0.541554012	0.9999537652	-2.858326524
-1.3784114725	0.4127458278	0.9999537652	1.1852540552
1.097881638	0.5072593192	0.9999537652	1.194308948
1.1457000343	0.6482659151	0.9999537652	-1.2269769112
-1.178850416	0.7079512524	0.9999537652	-1.0834206278
1.6097021916	0.374518625	0.9999537652	-1.1164222069
1.085205972	0.6741413386	0.9999537652	1.0545249725
1.0616020983	0.647917281	0.9999537652	1.0557093976
1.2521045812	0.2335091981	0.9999537652	1.3200420653
-1.3468525069	0.450839795	0.9999537652	1.0578168979
-1.3820549165	0.3129754329	0.9999537652	1.8628875485

-1.4047168188	0.6308422194	0.9999537652	1.9460841409
6.3075231393	0.4869314728	0.9999537652	6.908559683
0	0	0	2.9988886918
0	0	0	8.8639046058
0	0	0	2.9988886918
0	0	0	2.9985540909
1.4374492998	0.0479566374	0.9999537652	1.1306146945
13.3324809638	0.3187921671	0.9999537652	-6.43677014
6.3075231393	0.4869171328	0.9999537652	0
1.1172990819	0.6234749095	0.9999537652	-1.0820055751
-1.4768020436	0.6834230519	0.9999537652	-2.1290021891
1.3588921917	0.7999879612	0.9999537652	8.8639046058
1.1668522709	0.341842075	0.9999537652	1.3626947754
-1.2617840437	0.4076763673	0.9999537652	-1.0745562869
-1.2112341051	0.5188011103	0.9999537652	-1.1550769623
1.0249910597	0.8913507876	0.9999537652	-1.3156221067
0	0	0	-5.3608259368
-1.8388155927	0.3031935442	0.9999537652	1.0540033075
1.2750541257	0.7269643024	0.9999537652	1.2872098348
2.7957660672	0.7054198357	0.9999537652	-1.0487016324
-1.4184563758	0.2966975136	0.9999537652	-1.117146965
-1.2192458286	0.1190536578	0.9999537652	1.1503829113
-1.1054711699	0.5191917061	0.9999537652	1.028452815
1.1272506675	0.8145911962	0.9999537652	-1.4475676167
23.8617041368	0.2201356	0.9999537652	-2.0460434872
-1.805355848	0.0886677608	0.9999537652	-1.0581841245
8.064174035	0.4240238486	0.9999537652	0
-1.3509108602	0.53397695	0.9999537652	-1.0292208205
0	0	0	-1.1241083699
-1.6604569504	0.125188802	0.9999537652	-1.2048913056
-1.2826793645	0.5782811392	0.9999537652	-1.0565679627
-1.0934532261	0.6656456822	0.9999537652	1.0230531569
-1.5611997839	0.0468219528	0.9999537652	-1.1323269197
-1.4994755676	0.008111	0.9999537652	-1.0598687636
2.7933974408	0.6948713406	0.9999537652	-1.8579915692
-1.1202106492	0.4403463424	0.9999537652	1.3192202191
-12.9849514806	0.1075631758	0.9999537652	2.996475627
1.0046736267	0.9783308205	0.9999537652	1.0758365074
-4.7472651917	0.1001644684	0.9999537652	9.7116702472
-1.0346083795	0.8758056174	0.9999537652	1.1640706073
1.2907921107	0.1348007733	0.9999537652	1.0196228717
-1.1619717093	0.874250091	0.9999537652	1.1094441646
-1.1001857288	0.6578223073	0.9999537652	-1.0557292257
1.2336408201	0.4454709972	0.9999537652	-1.0515965497
1.0835518633	0.8597856819	0.9999537652	1.2940082969
-1.1650017951	0.4580178901	0.9999537652	-1.3198935578
0	0	0	14.7271569203
2.7933974408	0.6948713406	0.9999537652	-1.4680135518
-1.0018973682	0.9915574558	0.9999537652	-1.3619766871

-1.1639879069	0.5000166322	0.9999537652	1.0328289677
-1.4958408987	0.1268256912	0.9999537652	1.3841568482
1.2636997902	0.4769312577	0.9999537652	1.7028332523
1.3555910646	0.6328180489	0.9999537652	-1.0168197456
-1.5881570201	0.7141169061	0.9999537652	2.7978679986
0	0	0	8.8700459102
9.8205505243	0.3808396294	0.9999537652	-1.8680077128
1.4002317187	0.0349336644	0.9999537652	1.2377837467
-1.3186764966	0.1372763265	0.9999537652	1.0361673413
1.0308103059	0.876649923	0.9999537652	1.1261121939
2.2235544392	0.1676839488	0.9999537652	1.0722064199
1.1882440599	0.4368395155	0.9999537652	1.3034883583
-1.0633740706	0.8867774259	0.9999537652	1.1941105127
1.3881753996	0.0970312347	0.9999537652	1.0067252044
1.2621193273	0.4185580758	0.9999537652	-1.4367042876
-1.7518156055	0.2450454017	0.9999537652	1.3317722472
-1.369113427	0.5520806069	0.9999537652	-1.0746435454
1.7564707436	0.0926236485	0.9999537652	1.3362107554
-1.0518597919	0.8643254424	0.9999537652	1.0763732538
6.3075231393	0.4869027739	0.9999537652	-5.3583704783
1.2769351731	0.3728681407	0.9999537652	1.8632032351
-1.7279819296	0.4990673139	0.9999537652	1.2560473231
-1.1646731514	0.4898019399	0.9999537652	1.0147508311
1.1348984553	0.9169119154	0.9999537652	-1.5226286376
-1.6934349257	0.004637	0.9999537652	-1.3002035426
1.1898218348	0.7383238351	0.9999537652	-1.0868937382
1.6341257697	0.1918959912	0.9999537652	-1.1521491098
2.8197560167	0.354326402	0.9999537652	2.9983256599
1.0611819785	0.739021242	0.9999537652	1.043761478
-1.1490354002	0.4436559828	0.9999537652	-1.0718542709
-1.3417312931	0.470349407	0.9999537652	-1.4754797778
-2.4114503608	0.2961651996	0.9999537652	-2.314898282
2.8235674082	0.0978681259	0.9999537652	-1.6773238966
-1.4752136658	0.2658112389	0.9999537652	-1.454395254
-1.7063800139	0.4441716791	0.9999537652	4.1926556018
-1.7316657157	0.4577741195	0.9999537652	1.4584010041
-1.5875118025	0.7206758318	0.9999537652	-1.3884544962
0	0	0	6.908559683
1.0434520965	0.8234402572	0.9999537652	1.1458319212
1.7298869115	0.1577178278	0.9999537652	-1.5886586298
-1.0812271213	0.7826111778	0.9999537652	-1.1502930532
0	0	0	22.424363668
1.1033117594	0.7595856256	0.9999537652	-1.3296540485
-1.4920566811	0.5136528766	0.9999537652	1.241384699
1.2882164802	0.4132820401	0.9999537652	1.0373448136
1.0018615075	0.9952056803	0.9999537652	-1.143948355
-1.02760247	0.9101577628	0.9999537652	-1.3832648534
-1.19001074	0.2063291628	0.9999537652	-1.2700122278
1.5217013145	0.449353971	0.9999537652	-1.3959796701

-1.2321249909	0.7350882853	0.9999537652	1.1898778424
-1.1097332106	0.5128270969	0.9999537652	-1.0932120778
1.2915813014	0.2099713613	0.9999537652	1.2503345085
-1.0808922345	0.5831084768	0.9999537652	-1.0902816092
-1.2658372721	0.5601823836	0.9999537652	-1.1643439296
4.6415988335	0.0733894789	0.9999537652	2.0936238317
1.0488816194	0.8088733406	0.9999537652	-1.1246437894
-1.016615192	0.8994712105	0.9999537652	-1.0221184396
1.2670445632	0.2363032287	0.9999537652	-1.1770564973
1.0396384317	0.9173510855	0.9999537652	1.4194920823
-4.3251326698	0.1491179034	0.9999537652	-1.2074994903
-1.7020144588	0.3674739902	0.9999537652	-1.4855934874
325.0013340373	0.0217669979	0.9999537652	-1.9213028425
1.0383140723	0.8375300453	0.9999537652	1.3960268977
-1.0015038314	0.9912287402	0.9999537652	1.2376295816
1.2299264923	0.5370080757	0.9999537652	1.081433166
1.0532901703	0.8749556871	0.9999537652	1.2596920488
1.0712863643	0.6864735673	0.9999537652	-1.2906536651
-1.1448144121	0.9147526127	0.9999537652	-1.1592847852
1.5018264344	0.0423761195	0.9999537652	1.1118717605
-1.2368292141	0.5978223739	0.9999537652	-1.2641822333
0	0	0	4.9527501949
0	0	0	0
-1.1815702935	0.5122585302	0.9999537652	1.0159277583
1.1428524152	0.6448369303	0.9999537652	-1.5199923942
4.2494730656	0.1149727218	0.9999537652	1.1511394531
2.7933974408	0.6948713406	0.9999537652	6.908559683
1.514788658	0.2764513638	0.9999537652	1.1169723904
-1.1473244287	0.4876515394	0.9999537652	-1.1111702606
-1.1941506748	0.7699414064	0.9999537652	-1.0959134036
-1.0109137605	0.9884181605	0.9999537652	-1.1366231445
2.1372951012	0.0437574031	0.9999537652	1.245932158
1.1515629863	0.3141757134	0.9999537652	1.1586941576
1.1162834585	0.9271703652	0.9999537652	-1.6213283288
1.5719856594	0.5411479595	0.9999537652	-1.3582894632
-1.2846434244	0.3426871819	0.9999537652	1.2896108775
-1.0980091122	0.8297371405	0.9999537652	-1.1224899608
1.0466477925	0.8392974151	0.9999537652	1.3896430926
1.1754033384	0.7228069388	0.9999537652	1.1885750115
2.7933974408	0.6948713406	0.9999537652	4.9527501949
-1.0990278222	0.4786336867	0.9999537652	1.1717634946
1.1394224315	0.4648059622	0.9999537652	1.1897179107
-1.13806285	0.3440926076	0.9999537652	-1.0380334123
1.5727245322	0.5727884888	0.9999537652	-1.626869178
1.0629395588	0.8294812321	0.9999537652	1.1349667763
1.2396156377	0.4521721018	0.9999537652	1.1565809603
1.4857799254	0.1165239995	0.9999537652	1.1748773187
6.3130691196	0.479781225	0.9999537652	4.9527501949
2.7933974408	0.6948713406	0.9999537652	1.9416545697

1.2526375214	0.8112063186	0.9999537652	-1.5093307449
1.0395601932	0.8613372028	0.9999537652	-1.0576656324
1.5355940002	0.1361021634	0.9999537652	1.1064187527
-1.274390642	0.2293124134	0.9999537652	1.3072621753
-1.1746950837	0.7151220353	0.9999537652	1.0574694687
1.3092285135	0.3107915979	0.9999537652	1.0621093729
2.6478843491	0.4839523426	0.9999537652	1.6620430936
-1.5374498673	0.3643932466	0.9999537652	-1.3143165521
-1.1723855247	0.5330615417	0.9999537652	-1.0141056811
-1.4220832417	0.2374696045	0.9999537652	1.2471804733
1.1398230858	0.7799197742	0.9999537652	2.7532875246
-1.2013589017	0.6009400267	0.9999537652	-1.0425223956
-10.2105791297	0.0964986804	0.9999537652	1.2594537582
1.2526375214	0.8110779782	0.9999537652	1.1891564272
4.5505976667	0.5577515992	0.9999537652	1.2400291296
-1.1376671745	0.5399309518	0.9999537652	-1.0564385649
-1.8189146456	0.1019639261	0.9999537652	1.1966582884
1.2640003427	0.1546602684	0.9999537652	-1.0294899909
2.7933974408	0.6948713406	0.9999537652	0
1.4629074171	0.4454622236	0.9999537652	1.1466012146
-6.9762758667	0.2543312413	0.9999537652	1.9418822056
1.0519783983	0.8202323084	0.9999537652	1.1612218985
-1.4753668421	0.3068409055	0.9999537652	2.0690108874
-1.1556447285	0.7563453294	0.9999537652	1.1522248271
1.1209573618	0.6209824743	0.9999537652	1.2357807983
1.0618958585	0.7377986777	0.9999537652	1.6018531497
-1.0958421877	0.4865456432	0.9999537652	1.1540524872
1.1268625863	0.6209213197	0.9999537652	-1.0391478797
-1.5558976288	0.2829234496	0.9999537652	1.4741193153
-1.2592595272	0.4134185621	0.9999537652	-1.0796345363
-1.0218048715	0.9248445829	0.9999537652	-1.0427121652
1.362534054	0.5750086336	0.9999537652	1.5405640381
2.2962279822	0.3617930293	0.9999537652	1.7829918158
-1.6275497791	0.0722699822	0.9999537652	-1.5852382832
-1.1221013894	0.4199814739	0.9999537652	1.0176218088
1.0358097786	0.8694949735	0.9999537652	1.44015355
1.4660614269	0.1339236684	0.9999537652	1.5201318652
2.5206824432	0.007425	0.9999537652	1.6450331597
0	0	0	12.7732027325
1.2348958965	0.5147007124	0.9999537652	1.1535080144
0	0	0	8.8700459102
-1.0932906587	0.6225172159	0.9999537652	1.0140834814
1.3956385608	0.1717235821	0.9999537652	1.9714425293
-1.0844287504	0.7915575097	0.9999537652	1.1836091821
0	0	0	0
30.8757261591	0.1833133149	0.9999537652	-3.8929589845
-1.2923085997	0.6023372515	0.9999537652	-2.2694112962
-1.2099761873	0.2539006431	0.9999537652	-1.0416233259
0	0	0	2.9985540909

1.0873127571	0.702340663	0.9999537652	-1.0845273753
2.7933974408	0.6948713406	0.9999537652	-4.7285781921
-1.0128697188	0.9344790928	0.9999537652	-1.0588573523
-1.1511075236	0.5650664003	0.9999537652	1.0535317752
-1.0202071497	0.8795091522	0.9999537652	1.1351622757
1.0856187283	0.7112721406	0.9999537652	1.1808059798
-1.0345898309	0.7935053102	0.9999537652	1.0240564198
2.7933974408	0.6948713406	0.9999537652	-9.7542681481
-1.4657265584	0.0930455844	0.9999537652	-1.068127751
3.6196822224	0.0814577471	0.9999537652	-1.8355489271
4.5505976667	0.5577012236	0.9999537652	0
-2.5839527681	0.5183071693	0.9999537652	3.3441984921
-1.1253178567	0.6937263196	0.9999537652	1.3816692393
2.7933974408	0.6948713406	0.9999537652	-1.859280336
-1.1489611607	0.3693466201	0.9999537652	-1.0049356411
1.0562407945	0.6757795175	0.9999537652	1.1468341032
-1.0074064571	0.9619366858	0.9999537652	1.0750687188
-1.0892055415	0.6605419792	0.9999537652	1.5048172557
2.7933974408	0.6948713406	0.9999537652	-5.3583704783
-1.6498401319	0.2277349388	0.9999537652	-1.0107962671
1.2617131479	0.4309540234	0.9999537652	1.1188164018
-1.0287317069	0.8603258463	0.9999537652	1.1242181891
1.0821648394	0.818452559	0.9999537652	1.2486609772
-1.1437638024	0.285305938	0.9999537652	1.1685717749
-1.2266252847	0.3801539858	0.9999537652	1.1968610544
1.6485917849	0.1704046002	0.9999537652	-1.1212667352
1.1592313424	0.5102221654	0.9999537652	1.4169816582
11.5970171551	0.3502937215	0.9999537652	1.1696208368
1.309390581	0.665934907	0.9999537652	-1.130838319
1.6477221914	0.1991199259	0.9999537652	-1.0141886587
1.045400384	0.8420370358	0.9999537652	1.2202850714
6.3075231393	0.4868883961	0.9999537652	0
-1.1451704697	0.4880472376	0.9999537652	1.0138252452
-1.0978575892	0.5177003087	0.9999537652	1.1722033645
-1.716438562	0.2228354232	0.9999537652	-1.3306211756
-1.1600049263	0.8812951992	0.9999537652	-5.0960079851
-1.1291460463	0.6006015934	0.9999537652	1.4645561749
1.76873066	0.0753943829	0.9999537652	1.2264102027
1.0720580179	0.5478826827	0.9999537652	1.2254413111
1.1405427003	0.5022528838	0.9999537652	1.6475738629
1.158795471	0.3662614082	0.9999537652	1.1756952739
-2.3677331735	0.0169723416	0.9999537652	-1.224979783
-1.0670771203	0.7297574629	0.9999537652	1.0631299404
6.3128691894	0.481861816	0.9999537652	1.4534231615
-1.2528594162	0.3123764475	0.9999537652	1.2600320081
-1.9073536712	0.3866237965	0.9999537652	1.1380586761
-1.3737409188	0.1239436417	0.9999537652	1.5352045503
1.0253956216	0.878370439	0.9999537652	-1.0036749067
-1.011324172	0.9882060702	0.9999537652	-1.859280336

1.0511680412	0.8722168973	0.9999537652	1.5928924857
-3.211138696	0.1796576764	0.9999537652	4.398765428
0	0	0	2.996475627
1.0916003691	0.517450196	0.9999537652	1.2710171101
-1.0642455518	0.6833214822	0.9999537652	1.4841496024
1.1012257078	0.6561440393	0.9999537652	-1.3346958976
1.5868795429	0.314446012	0.9999537652	-1.1403455413
1.4551873183	0.1666428635	0.9999537652	1.8251029429
-1.2273289242	0.1280516413	0.9999537652	1.0166666861
-1.3127136138	0.4319534894	0.9999537652	1.0210994733
-1.1486265126	0.5266175683	0.9999537652	-1.1597153584
1.0643300638	0.8323213297	0.9999537652	1.1825391065
2.7957660672	0.7054221731	0.9999537652	0
1.00230527	0.9895414565	0.9999537652	1.0275549918
1.5908598212	0.3202414594	0.9999537652	1.8941137294
-1.4785983583	0.3579900557	0.9999537652	-1.1524390841
1.0413955461	0.8497535317	0.9999537652	-1.0829274777
8.064174035	0.4240222159	0.9999537652	1.0669957455
18.6146543426	0.2563481578	0.9999537652	-13.0161175857
1.0896909925	0.5737125951	0.9999537652	1.1512446747
1.7101327785	0.324666804	0.9999537652	1.0907555042
-1.3140713878	0.4094078676	0.9999537652	-1.0791316819
1.0337486417	0.9598947756	0.9999537652	1.1650284134
-1.0501977401	0.8537055785	0.9999537652	1.375868489
1.020806471	0.9494662306	0.9999537652	1.9824465554
-1.0085989693	0.9616334718	0.9999537652	-1.1903487432
1.328223045	0.1024179437	0.9999537652	1.815249296
-1.442184797	0.1088475838	0.9999537652	-1.1143071197
1.0568838	0.7297254104	0.9999537652	1.6527969371
-1.2740858477	0.2176388607	0.9999537652	1.3876787987
-1.1453192235	0.9088286671	0.9999537652	-1.3786406679
1.1170173403	0.6453146386	0.9999537652	1.6278305962
1.2034750756	0.3245807335	0.9999537652	1.1015874304
1.3115064824	0.1413113992	0.9999537652	1.0386673647
1.3553887312	0.1309610636	0.9999537652	1.309697898
1.0983482417	0.6590568401	0.9999537652	1.217504941
1.4996200024	0.1045064697	0.9999537652	-1.3002625694
8.064174035	0.4240205835	0.9999537652	0
1.0942194321	0.8686328358	0.9999537652	2.1544981479
1.0874334999	0.6834874152	0.9999537652	1.14041084
1.150984149	0.7760120724	0.9999537652	1.2597234857
1.505135715	0.3819904603	0.9999537652	1.0226831506
-1.0847830821	0.7751841997	0.9999537652	1.0481962118
-1.2421272489	0.6912872228	0.9999537652	-1.0266179774
1.0917258909	0.6335093651	0.9999537652	1.0150594856
-1.8938020412	0.0167804608	0.9999537652	-1.3088023773
1.0385910948	0.8256296728	0.9999537652	-1.0948424424
-1.4629126083	0.1049551923	0.9999537652	-1.0247038044
1.1746975725	0.2741743233	0.9999537652	-1.2357106507

-1.0511723584	0.9209357802	0.9999537652	1.1538557337
1.1429949461	0.4820282082	0.9999537652	-1.1672257916
-1.0248800347	0.90584246	0.9999537652	1.0814155746
1.4513773693	0.362124465	0.9999537652	-1.6040917952
-1.169745712	0.2782519686	0.9999537652	-1.1516564529
-1.1744398787	0.355121647	0.9999537652	-1.3302832635
1.4958687715	0.496904432	0.9999537652	-1.4629673466
1.1900002326	0.375305214	0.9999537652	-1.1144918816
0	0	0	9.7116702472
2.7933974408	0.6948713406	0.9999537652	-1.356070459
1.1766425009	0.5973362274	0.9999537652	1.076505587
1.0352399375	0.8985544878	0.9999537652	-1.153757751
2.7933974408	0.6948713406	0.9999537652	0
1.035374331	0.9606271499	0.9999537652	1.0112222041
-1.2020669635	0.4071065797	0.9999537652	-1.2136313295
-1.3052296421	0.2417564777	0.9999537652	1.0002975092
1.3164602335	0.3885740171	0.9999537652	1.352532263
1.519717953	0.1903331694	0.9999537652	1.0622908449
-1.1901517603	0.2053590623	0.9999537652	-1.1167649501
-1.189279638	0.3315082153	0.9999537652	-1.1032339807
1.2588461709	0.5601081094	0.9999537652	-1.0252542254
8.064174035	0.4239978731	0.9999537652	1.8383591193
1.3050983867	0.4831923024	0.9999537652	1.1080762564
1.1133075268	0.5432715388	0.9999537652	1.1400503692
0	0	0	2.996475627
6.3075231393	0.4868739992	0.9999537652	0
-1.08031947	0.6063059469	0.9999537652	1.24651194
1.2021764012	0.4843033549	0.9999537652	1.2662555378
-1.4019663325	0.0476335002	0.9999537652	-1.0215834625
-1.1427526036	0.6458042671	0.9999537652	-1.0177894517
2.4059761043	0.0402225208	0.9999537652	-1.0177868458
1.2986112034	0.3301623758	0.9999537652	-1.0353147614
6.3075231393	0.4868595833	0.9999537652	2.996475627
0	0	0	2.996475627
0	0	0	2.996475627
-1.1726921613	0.6262713869	0.9999537652	1.3445786505
0	0	0	2.996475627
0	0	0	3.3371876641
1.076994809	0.8787522402	0.9999537652	-1.6060544264
-2.1317846121	0.465033729	0.9999537652	-2.4832891211
1.1637651383	0.4530262829	0.9999537652	1.0199462791
3.7228204944	0.0195702251	0.9999537652	3.9136979278
-1.1104037121	0.59655728	0.9999537652	1.3240641201
1.6173170138	0.4881951629	0.9999537652	1.0477796599
1.0444164156	0.8358964811	0.9999537652	-1.1665378568
-1.1227911744	0.4681672298	0.9999537652	-1.3609204707
1.1870754472	0.4787749581	0.9999537652	1.0184224455
-1.2496447372	0.1495007075	0.9999537652	1.2097408287
1.073848037	0.7386670365	0.9999537652	1.4840125295

-1.1457847193	0.9109214482	0.9999537652	1.3626105447
-1.1264997606	0.6055307391	0.9999537652	-1.38164978
2.7933974408	0.6948713406	0.9999537652	-1.6016888038
-1.2441412193	0.087827701	0.9999537652	-1.175019344
-1.5023747081	0.5454951764	0.9999537652	-1.7099509994
1.6141510062	0.2508927353	0.9999537652	-1.0198881406
-1.226319784	0.4191284192	0.9999537652	1.2203332304
1.1497233216	0.8135557954	0.9999537652	4.7695885622
1.0713278051	0.7401835915	0.9999537652	-1.2230686499
-1.1549283906	0.5699118603	0.9999537652	1.0598025085
0	0	0	0
-1.0626935003	0.8749974338	0.9999537652	1.0431939855
-1.1180346051	0.5212107105	0.9999537652	1.0942593694
-1.0161374203	0.9559898734	0.9999537652	1.0159652145
1.2803598908	0.5980381504	0.9999537652	1.1908574208
-1.0562791674	0.7936647094	0.9999537652	1.3988993153
1.0068397368	0.9663845186	0.9999537652	1.0652516359
1.0582337724	0.8090665533	0.9999537652	-1.1538988809
-1.33324794	0.1210635042	0.9999537652	1.0133581482
-1.1213600596	0.8125948082	0.9999537652	-1.1636699835
-1.3198880592	0.2495391052	0.9999537652	-1.3663208889
-1.1748137342	0.488164946	0.9999537652	1.1396986193
1.0458462643	0.7656018246	0.9999537652	-1.076296232
-1.2374155669	0.4552737317	0.9999537652	1.0449121576
-1.0259719857	0.8956232325	0.9999537652	-1.1480129737
1.1324974701	0.6557070921	0.9999537652	-1.0539486489
-1.6388358136	0.131036403	0.9999537652	-1.1570501672
1.0160046865	0.8872339051	0.9999537652	1.1588879339
-1.1562840158	0.7699589651	0.9999537652	1.1457929503
1.2195533953	0.723759516	0.9999537652	-1.4910734584
2.7957660672	0.7054245114	0.9999537652	0
0	0	0	2.9985540909
1.1005825568	0.4459436823	0.9999537652	1.006683691
1.2889988575	0.5738423145	0.9999537652	1.07862799
-1.2084036764	0.4276486206	0.9999537652	1.2761736378
0	0	0	6.9189162209
1.0064752338	0.963208787	0.9999537652	1.2436561265
-1.5240520637	0.3496825185	0.9999537652	-1.7567895333
0	0	0	7.1143876568
-1.4015448726	0.0778871131	0.9999537652	-1.2757129396
0	0	0	4.9527501949
1.0174016281	0.9211170119	0.9999537652	-1.1772047529
1.5991448424	0.5357957999	0.9999537652	1.1688090495
-1.9021540937	0.037850383	0.9999537652	-1.2302364531
-2.0589994115	0.1862728649	0.9999537652	-1.0186071408
-1.7155763775	0.5432257671	0.9999537652	-2.0281652258
-1.2475563626	0.491694841	0.9999537652	-1.1771584984
1.498009066	0.0579485092	0.9999537652	-1.1040047039
1.2776773713	0.0671941206	0.9999537652	1.1319182604

1.0589267387	0.7599175222	0.9999537652	-1.0806326085
1.0538600751	0.9221992369	0.9999537652	1.0200923869
-1.4420675885	0.5481285907	0.9999537652	-1.207672864
2.2285006183	0.0986211377	0.9999537652	1.1632254461
1.0520850385	0.7294734493	0.9999537652	1.3786566794
1.037067994	0.844140845	0.9999537652	-1.1965148367
1.0560121516	0.7167480839	0.9999537652	-1.0061261619
1.1230473096	0.8987510784	0.9999537652	4.9527501949
-1.3239989074	0.1129322713	0.9999537652	-1.0002932914
1.1108432409	0.4768303412	0.9999537652	1.0210580016
1.204311661	0.3383383836	0.9999537652	-1.080425513
1.869534062	0.061134213	0.9999537652	1.0682839776
-1.4998778653	0.186608436	0.9999537652	1.0710804526
-1.3388995872	0.4526404876	0.9999537652	1.2105488934
1.0979970564	0.8179310374	0.9999537652	1.482011877
1.1744022705	0.6197450195	0.9999537652	-1.2584238334
1.2210507869	0.5452191408	0.9999537652	1.3417930919
0	0	0	-1.9327627911
-1.0442858003	0.8838833313	0.9999537652	1.3706649725
1.0247762442	0.9175730921	0.9999537652	-1.1781224712
-1.4037437325	0.158080739	0.9999537652	-2.477109613
-1.1210727303	0.5472728228	0.9999537652	-1.0756686032
-1.1536798624	0.4261263735	0.9999537652	-1.2344310368
1.2761421706	0.0710486915	0.9999537652	-1.2804465838
-1.1534283699	0.3789090504	0.9999537652	-1.0537584906
1.5539296366	0.3024131654	0.9999537652	-1.0189838092
-1.0627566641	0.7397392911	0.9999537652	-1.1568202852
4.6439223933	0.0923159429	0.9999537652	-1.4933246322
1.4109285177	0.2142267401	0.9999537652	-1.1195209627
-1.0733798751	0.5732282514	0.9999537652	1.0199562944
1.6455269788	0.5819748549	0.9999537652	-1.5238166904
1.2150155835	0.6083540341	0.9999537652	1.5335812826
1.2024834744	0.3160057651	0.9999537652	1.2470352738
-2.1522508354	0.0723138508	0.9999537652	-2.2069756414
-6.9736190158	0.2250321147	0.9999537652	6.908559683
1.1633389219	0.6642141551	0.9999537652	-1.0220830109
-1.2381922417	0.4943169404	0.9999537652	-1.1289441484
1.6024607202	0.5296952292	0.9999537652	1.4392655202
1.1158583321	0.9297136342	0.9999537652	-2.2019778712
2.7933974408	0.6948713406	0.9999537652	0
-1.0072378423	0.9939316374	0.9999537652	24.5069156959
0	0	0	2.996475627
-1.2131496116	0.6906057536	0.9999537652	1.3568020981
1.1915864663	0.377519174	0.9999537652	1.1089859259
-1.4202916206	0.023254002	0.9999537652	-1.1822845528
-1.0703118558	0.6036248977	0.9999537652	1.0198138373
1.3013038034	0.0874813483	0.9999537652	-1.47417274
-1.0249684676	0.899436258	0.9999537652	-1.0031616923
1.1195202445	0.7316821184	0.9999537652	-1.0246191345

-1.0493385585	0.7987195472	0.9999537652	-1.0703107444
9.8205505243	0.3808394853	0.9999537652	-18.8248240915
-1.375810396	0.3341088348	0.9999537652	-1.1753126331
-1.1411275992	0.4433807929	0.9999537652	-1.2222093388
-1.4310782011	0.1708376939	0.9999537652	-1.4219545387
8.064174035	0.4239752387	0.9999537652	0
-3.0103959855	0.113018508	0.9999537652	-1.3676887997
1.2292952208	0.6486208657	0.9999537652	1.0184491269
4.5505976667	0.5576922483	0.9999537652	-9.7542681481
-1.2034535928	0.5285939162	0.9999537652	-1.23587814
-1.3609572986	0.5044044733	0.9999537652	-1.5900728097
0	0	0	0
-2.9570895726	0.3902706139	0.9999537652	2.996475627
1.3477216605	0.4557063625	0.9999537652	-1.1585435023
1.1119615774	0.5092568092	0.9999537652	1.1211907233
1.5701097546	0.5500352572	0.9999537652	1.0537060663
0	0	0	6.908559683
1.1504647989	0.5620110266	0.9999537652	1.1433916657
0	0	0	6.908559683
-1.1613472761	0.4303450321	0.9999537652	1.398498165
1.1937906519	0.6881194648	0.9999537652	-1.1757086358
1.5694218508	0.5760214204	0.9999537652	-1.2102801718
-1.3998169344	0.6019956971	0.9999537652	1.191977693
1.5911703288	0.0184098181	0.9999537652	-1.0355545636
2.3210078398	0.0138403392	0.9999537652	1.0602090509
-1.0248974411	0.9010802185	0.9999537652	1.2473309637
1.0688350535	0.790826852	0.9999537652	1.200383551
1.3461558519	0.261825581	0.9999537652	-1.228241807
2.9970436837	0.1412286185	0.9999537652	1.1337017128
1.1418395671	0.4718124864	0.9999537652	-1.128011257
-1.0879922278	0.725174544	0.9999537652	1.0447083638
1.0725526372	0.780284787	0.9999537652	1.3400239211
-1.0467415704	0.7678274088	0.9999537652	1.1828803552
1.0904709062	0.888954656	0.9999537652	-1.6841458174
-1.5516098154	0.008209	0.9999537652	-1.2704449498
1.3773841198	0.0750974533	0.9999537652	1.1870741418
1.0649437907	0.7691497218	0.9999537652	-1.0691890671
-1.4072265381	0.4084547954	0.9999537652	-1.9274193478
-1.0760180298	0.8728234071	0.9999537652	-1.1838610992
1.173620467	0.5484838813	0.9999537652	-1.1021055527
1.0138924925	0.9172152722	0.9999537652	1.2198315865
1.0160501577	0.9818068531	0.9999537652	-2.6257240681
-1.2056234393	0.7216619522	0.9999537652	1.6363955728
1.3873670102	0.095139037	0.9999537652	1.2644536509
-1.0745764919	0.8779335339	0.9999537652	1.0645142396
1.3057821641	0.322197175	0.9999537652	1.136305171
-1.7960963046	0.0353999668	0.9999537652	-1.6383799161
-1.0824508832	0.8070829465	0.9999537652	1.1684426858
-1.0987247164	0.4891451095	0.9999537652	1.0130217641

-1.3133154702	0.6767621103	0.9999537652	-1.1606819021
-1.7439958368	0.0181843813	0.9999537652	-1.005234379
-2.5871290907	0.4905598287	0.9999537652	2.9985540909
1.1453117903	0.750102408	0.9999537652	-1.5011800753
4.5505976667	0.5576832976	0.9999537652	6.908559683
-1.8902736666	0.2230740538	0.9999537652	-1.457796445
1.2178229098	0.396837049	0.9999537652	1.0582750014
1.0390670679	0.8607139937	0.9999537652	1.2289441646
-1.171237928	0.4192912667	0.9999537652	-1.2667167793
-4.8113172456	0.1956605724	0.9999537652	-1.11577527
1.1286193068	0.7085293488	0.9999537652	1.6473660147
-2.770555493	0.2449195082	0.9999537652	2.996475627
1.1232102074	0.8949390203	0.9999537652	1.0327700015
1.3967109867	0.6231176573	0.9999537652	1.3928979747
1.1265794196	0.6559015827	0.9999537652	1.0249543837
-1.7001055654	0.2974767457	0.9999537652	-1.250150973
-1.2711181053	0.1671672404	0.9999537652	-1.0893427877
13.3324809638	0.3188556925	0.9999537652	-1.1248877899
-1.080766331	0.5207966622	0.9999537652	1.2629406651
-1.1653373542	0.6252658568	0.9999537652	1.6169019773
1.2991215087	0.2723357714	0.9999537652	1.1424057438
-1.1094275927	0.8535992931	0.9999537652	1.0615361821
-1.1214545535	0.3874840679	0.9999537652	1.076893704
-1.0243470512	0.8701255507	0.9999537652	1.1253801282
1.5626555917	0.2452518179	0.9999537652	-1.0390755631
1.499792306	0.2428020631	0.9999537652	1.0612790076
-1.1205725737	0.7069996167	0.9999537652	-1.1965830081
4.5545995984	0.569676623	0.9999537652	-1.1242401582
1.1427607173	0.6205412318	0.9999537652	1.4138937627
4.5505976667	0.5576743707	0.9999537652	0
-1.0680746196	0.6551967471	0.9999537652	1.1422735879
-2.6335352531	0.2724653065	0.9999537652	1.4735607458
1.1193653536	0.5990796234	0.9999537652	1.1666476618
1.2012386498	0.6891067262	0.9999537652	1.2666808338
0	0	0	4.9527501949
1.0697937879	0.8125693604	0.9999537652	1.300168505
2.1676257531	0.2057616268	0.9999537652	1.3034669708
6.3075231393	0.4868451483	0.9999537652	0
1.2401865025	0.365918224	0.9999537652	1.4903777703
1.1281260175	0.8393942768	0.9999537652	-1.0519534482
32.6837273262	0.1727736902	0.9999537652	-1.5278961905
1.1545303195	0.6541583145	0.9999537652	1.0341801366
1.1172293767	0.9254380029	0.9999537652	-1.1177863553
-1.312693991	0.6755329729	0.9999537652	1.2175748555
1.1250293197	0.6723403968	0.9999537652	1.2952947879
1.0248763208	0.9177075708	0.9999537652	-1.0743713943
-1.1266512601	0.379196072	0.9999537652	-1.1264753873
1.289655948	0.3729279532	0.9999537652	-1.1655942033
-1.4528331939	0.442685151	0.9999537652	1.3146074947

-15.9195842901	0.1128472012	0.9999537652	14.7271569203
-1.2895053411	0.2849709007	0.9999537652	-1.2578047364
-1.115795443	0.5888669656	0.9999537652	1.034521005
1.9435125307	0.30513947	0.9999537652	1.2348501603
-1.0426529243	0.8842622733	0.9999537652	-1.0057370137
2.1641462884	0.4097159943	0.9999537652	-1.9838489833
2.7933974408	0.6948713406	0.9999537652	0
-1.0403321647	0.8055030261	0.9999537652	1.2005352496
1.1704806069	0.452253772	0.9999537652	-1.0140817857
72.2737389183	0.0952283593	0.9999537652	1.0174335377
-1.0749487437	0.7485943297	0.9999537652	-1.3106851597
1.5475388881	0.2232908411	0.9999537652	1.2133852125
1.7034428179	0.1369328297	0.9999537652	1.2076177368
1.153390097	0.4712070511	0.9999537652	1.1314184705
-1.0627924055	0.6513445754	0.9999537652	1.1838317375
1.0454070479	0.7356454641	0.9999537652	1.2114212094
22.1637147731	0.232069292	0.9999537652	-2.9704130837
-1.0283793853	0.8848980537	0.9999537652	1.1838802961
-1.0653372444	0.8335414035	0.9999537652	-1.0381388704
-1.1140565098	0.4362450579	0.9999537652	1.0711807146
1.0798613785	0.7318149218	0.9999537652	1.4716633007
-1.8332588124	0.0520307048	0.9999537652	-1.6838698083
1.0343611962	0.8175589165	0.9999537652	1.1705276959
-1.3991734392	0.3044553083	0.9999537652	-1.1829732968
2.5433021181	0.1134282609	0.9999537652	-2.3029868242
1.5521007833	0.0240623009	0.9999537652	1.3236194061
2.7933974408	0.6948713406	0.9999537652	0
0	0	0	-1.0084773122
4.5505976667	0.5576654668	0.9999537652	-1.8577842586
1.0618073657	0.7329934494	0.9999537652	1.1956408773
0	0	0	2.4169633444
2.8162992884	0.3665164546	0.9999537652	0
-1.0711601088	0.6732498314	0.9999537652	1.168470637
1.176334431	0.6721143261	0.9999537652	1.1772574461
1.0003454956	0.9980277424	0.9999537652	1.1663109764
-1.1106270444	0.7898148094	0.9999537652	1.0011056049
1.6137547008	0.0339682191	0.9999537652	1.8510705847
0	0	0	-1.1246234874
1.3366122163	0.197512625	0.9999537652	1.1164893671
1.1730876003	0.4123037573	0.9999537652	-1.2132780891
1.013596976	0.9556122211	0.9999537652	2.6011217741
-1.1453192235	0.9087980493	0.9999537652	4.9527501949
0	0	0	0
7.441072047	0.0613606596	0.9999537652	1.0675791322
4.5505976667	0.557656585	0.9999537652	0
0	0	0	2.996475627
-1.1189664609	0.7970761536	0.9999537652	1.6656146362
-1.0066229223	0.9825912206	0.9999537652	-1.404547562
1.0031116066	0.9901061168	0.9999537652	1.4338063522

-1.0479970231	0.942250377	0.9999537652	-1.005481025
4.5505976667	0.5576477246	0.9999537652	0
1.8455489546	0.5922034177	0.9999537652	2.9985540909
-1.1159721327	0.4908292075	0.9999537652	1.2804490154
1.0140986119	0.9588003633	0.9999537652	1.0751211944
-1.0761763568	0.7194503308	0.9999537652	-1.0177688911
1.5442117167	0.091630784	0.9999537652	1.1849556214
1.6768394119	0.3631543028	0.9999537652	1.1061451365
-1.0445990748	0.8807280632	0.9999537652	1.371246993
4.5505976667	0.5576388846	0.9999537652	4.9527501949
-1.1475554583	0.6474593633	0.9999537652	-1.1488224766
1.5415125877	0.1158102842	0.9999537652	1.1053272374
-1.1453192235	0.9087673816	0.9999537652	1.400113184
-1.392221459	0.6739064548	0.9999537652	1.1687602177
1.1330532184	0.7750234861	0.9999537652	-1.0320814787
16.296263041	0.296224819	0.9999537652	-3.4788333565
2.7933974408	0.6948713406	0.9999537652	-2.0482026754
1.1465841484	0.6592560474	0.9999537652	1.0503291133
1.022428299	0.8648394789	0.9999537652	1.0131308351
1.0054097483	0.9899836708	0.9999537652	1.2263068423
-2.1317846121	0.4678509562	0.9999537652	16.6940563684
1.03746342	0.8722212938	0.9999537652	1.4745605849
1.1467689727	0.4019675033	0.9999537652	1.0051312602
-1.3037508243	0.084212867	0.9999537652	-1.3475445171
1.1347840865	0.610457031	0.9999537652	-1.1185136845
1.0180521385	0.9252341466	0.9999537652	-1.1304977299
1.1435506627	0.4162041729	0.9999537652	1.1906304787
-1.1140468122	0.6581012245	0.9999537652	-1.0119064276
1.0763501624	0.7435351129	0.9999537652	1.1031915719
0	0	0	6.908559683
-1.0869274837	0.532008322	0.9999537652	1.025240004
-1.0489830583	0.6894234885	0.9999537652	1.0230216711
-1.2351182932	0.2734582121	0.9999537652	1.4707706705
9.1990164803	1.06E-06	0.0189773051	24.143356737
-1.63410696	0.03723209	0.9999537652	1.3616638938
1.0179460471	0.9296751333	0.9999537652	1.3070137565
-2.2365969411	0.3843373066	0.9999537652	1.2335551469
-1.2193405642	0.3643378554	0.9999537652	1.469956191
1.0684375289	0.7748364665	0.9999537652	1.5714037494
-1.0269328778	0.9276292786	0.9999537652	1.2198405611
1.2490174178	0.1567640689	0.9999537652	1.586305387
1.2395265977	0.6640853447	0.9999537652	-1.7644830068
-1.3350320065	0.4914023021	0.9999537652	1.0594513172
1.1268391856	0.393344045	0.9999537652	-1.1750601984
1.1323967673	0.7017700152	0.9999537652	1.1232183178
-1.1900524362	0.5588072892	0.9999537652	1.2558227783
1.1220972931	0.9041283662	0.9999537652	-4.8176145137
-1.0391534682	0.9257707764	0.9999537652	-1.2230392117
-1.0798958047	0.8799381257	0.9999537652	-2.1736017414

0	0	0	8.8639046058
-2.6343761231	0.2907424951	0.9999537652	-1.5086090391
-1.1092422741	0.5596935199	0.9999537652	1.0538815882
1.5212036067	0.1434326634	0.9999537652	1.2055304698
6.3075231393	0.4868306943	0.9999537652	-1.2504304605
1.583406222	0.0408111628	0.9999537652	1.3796263994
1.035792188	0.9120676382	0.9999537652	1.6417851307
-1.5875118025	0.7207317913	0.9999537652	0
4.6453887007	0.0002512	0.9999537652	1.6929027962
1.5221698053	0.3377984522	0.9999537652	1.0640697182
1.0197307873	0.943631204	0.9999537652	1.0454720013
1.4581308732	0.7062389653	0.9999537652	-1.9277963491
-1.1238167931	0.8141386403	0.9999537652	1.0167624289
-1.0965061948	0.6512540573	0.9999537652	-1.0656734804
-1.0033260952	0.9851993224	0.9999537652	-1.0379887263
-2.0643077486	0.0965713158	0.9999537652	1.1065197263
3.5328411982	0.3047710221	0.9999537652	-1.0743752856
-1.4605783765	0.4748944014	0.9999537652	-1.2346788625
3.0602334741	0.328531917	0.9999537652	-1.1226656213
1.3699340379	0.0183804665	0.9999537652	1.3179373629
1.5130808007	0.6579655594	0.9999537652	1.2407431382
1.183461603	0.5515206837	0.9999537652	1.2773148379
0	0	0	4.9527501949
-1.0909766278	0.6783225896	0.9999537652	-1.0214064203
1.2793127563	0.5638582298	0.9999537652	-1.1498444917
-3.7593785559	0.002431	0.9999537652	-2.1285134593
1.0820344243	0.6759707952	0.9999537652	1.0820014852
-1.1779626091	0.594980742	0.9999537652	1.1423135253
-1.0230814332	0.8772607162	0.9999537652	1.3822325221
1.1153789105	0.5468932214	0.9999537652	1.4291118044
-1.0244254994	0.889177542	0.9999537652	-1.0177733019
1.3025382023	0.5397017064	0.9999537652	-1.0356082651
1.1285469221	0.8431951108	0.9999537652	1.0238096781
-1.1575422179	0.5369490546	0.9999537652	1.2182949399
-1.7015132167	0.0137077438	0.9999537652	1.4735783319
-1.0507148122	0.655743744	0.9999537652	1.1172664846
0	0	0	4.9527501949
-1.0835132333	0.5995928735	0.9999537652	1.0936003835
2.7957660672	0.705429191	0.9999537652	16.6806485101
-1.1216939351	0.5592132271	0.9999537652	1.1862172136
-1.1472085644	0.4614650073	0.9999537652	1.0353645359
1.4332074867	0.0998365847	0.9999537652	1.0828006422
-1.1214254558	0.3507938952	0.9999537652	-1.0243099247
1.0378161503	0.8417225015	0.9999537652	-1.0599889274
26.4578430969	0.207636342	0.9999537652	-1.0153549098
1.1060075337	0.6879695557	0.9999537652	1.181449813
-1.1679931418	0.6864116387	0.9999537652	1.519456354
-1.2145271299	0.2814843107	0.9999537652	1.1158347271
0	0	0	8.8700459102

-1.2008500321	0.6659626727	0.9999537652	1.2748737516
1.4382672885	0.0467766931	0.9999537652	1.1649330407
2.7933974408	0.6948713406	0.9999537652	-1.1248877899
2.0902635436	0.0265428439	0.9999537652	1.005803903
-1.2390439306	0.4507496108	0.9999537652	-1.1661798093
8.064174035	0.4239556197	0.9999537652	-5.3583704783
2.4292825457	0.0264783623	0.9999537652	-1.1585884938
1.113905848	0.5513491	0.9999537652	1.0018916725
0	0	0	2.996475627
1.3442212242	0.2458128722	0.9999537652	1.0863823731
2.4323134639	0.1518615326	0.9999537652	1.2786917525
2.7933974408	0.6948713406	0.9999537652	1.0704119569
1.0032989227	0.9822772737	0.9999537652	1.0681764334
0	0	0	6.908559683
1.1826141087	0.1275385942	0.9999537652	1.3718561716
1.0514685567	0.7329685116	0.9999537652	1.246988131
-1.2679518858	0.2883421052	0.9999537652	-1.1005419295
1.3147725167	0.2579142622	0.9999537652	1.0116122557
1.3306804405	0.1681169142	0.9999537652	1.3020017707
-1.2930855193	0.2201536123	0.9999537652	1.3563068377
-1.1750347589	0.2544426577	0.9999537652	1.0290303943
-6.9736190158	0.2250321147	0.9999537652	12.7820459093
0	0	0	2.9985540909
1.1158583321	0.9297022482	0.9999537652	2.7252039655
1.0135290913	0.9713416216	0.9999537652	-1.1000511018
-1.1260619216	0.591917528	0.9999537652	1.2361896133
-1.2845289784	0.4457821375	0.9999537652	-1.1157700333
1.1753506509	0.7378354607	0.9999537652	-1.1988273335
-1.0601803977	0.8727029628	0.9999537652	1.2723442537
1.0435800517	0.823711467	0.9999537652	1.119711375
-1.0630902122	0.7678207553	0.9999537652	-1.1005816045
1.0209414833	0.9659715487	0.9999537652	-1.1112031805
-2.5929763269	0.3852067027	0.9999537652	1.6675282515
8.064174035	0.4239360593	0.9999537652	-2.1302390584
-1.0886845112	0.8947382894	0.9999537652	-1.6542599977
1.055579126	0.8632089637	0.9999537652	1.4458781219
1.592047446	0.0507986798	0.9999537652	1.3093189581
8.064174035	0.4239165559	0.9999537652	5.0990322208
2.7933974408	0.6948713406	0.9999537652	0
1.6802698771	0.3095147362	0.9999537652	1.6474778295
-1.0424941752	0.821415831	0.9999537652	1.2145507737
-1.4337129396	0.3344601427	0.9999537652	1.1004698731
1.2052221669	0.228430723	0.9999537652	1.5048241012
1.0256765964	0.8781535785	0.9999537652	1.3863659661
1.3186512428	0.127507528	0.9999537652	1.1538928234
-1.0230304326	0.882520738	0.9999537652	1.473978586
-1.9648206137	0.156022751	0.9999537652	-1.183151052
0	0	0	4.9527501949
-1.048658627	0.8708153037	0.9999537652	1.035728673

-1.5490672502	0.6602358917	0.9999537652	-1.5322468652
-1.3767560855	0.6774317336	0.9999537652	1.9917172127
1.7129231458	0.2434027398	0.9999537652	-1.0636121131
-1.6229167897	0.0665915856	0.9999537652	1.0498462618
1.9273015337	0.3884096762	0.9999537652	2.3378233432
2.7322393205	0.1855349669	0.9999537652	-1.5672193861
0	0	0	-1.859280336
8.064174035	0.4238971112	0.9999537652	2.996475627
0	0	0	2.996475627
-1.3787811241	0.1454603146	0.9999537652	1.2907842745
-2.6298987489	0.0002685	0.9999537652	1.3331994943
1.0894495432	0.8597628417	0.9999537652	-1.0500316379
-1.0027759582	0.9904631883	0.9999537652	1.4781412465
-1.2000073527	0.3468350275	0.9999537652	-1.2288659474
1.2296406719	0.1783954715	0.9999537652	-1.085396602
-1.0585723933	0.7687145374	0.9999537652	1.102835841
-1.0475941333	0.8135417484	0.9999537652	1.1554697286
-1.4873781733	0.3400315784	0.9999537652	1.3122152917
1.1251728234	0.6014551014	0.9999537652	1.082641593
-1.132931259	0.786759906	0.9999537652	-1.111189816
-1.3328887579	0.1145849329	0.9999537652	1.1268232115
0	0	0	-2.7870276542
1.0868111736	0.5348770754	0.9999537652	1.6578317416
-1.1096700401	0.5519943915	0.9999537652	-1.0643715305
-1.3721203041	0.4409845728	0.9999537652	1.2058456617
1.2174591383	0.4086053811	0.9999537652	1.1110207582
-1.0590565585	0.6538576352	0.9999537652	-1.0647810106
1.5539708133	0.2807540937	0.9999537652	-1.0866002666
1.1344686964	0.5993021364	0.9999537652	1.1312329064
5.9742368043	0.0914512158	0.9999537652	1.4482006908
-1.2625255738	0.5773559197	0.9999537652	1.3022980794
2.7933974408	0.6948713406	0.9999537652	1.1335799376
1.0140684473	0.9487466178	0.9999537652	1.1338761835
-1.4768639477	0.0468497868	0.9999537652	-1.1153379709
-1.0521485075	0.8315491341	0.9999537652	1.2783074523
2.5884057377	0.0986538822	0.9999537652	-1.1260106122
1.2351741356	0.1740716067	0.9999537652	1.1730835714
2.7933974408	0.6948713406	0.9999537652	-5.3589986948
9.8205505243	0.3808393413	0.9999537652	-2.0465550316
1.0103406333	0.9743154328	0.9999537652	1.2324852636
1.07852598	0.5913526644	0.9999537652	1.4015804368
-1.3795667895	0.1106669476	0.9999537652	1.1409265097
0	0	0	8.8639046058
3.0168480578	0.1701374261	0.9999537652	1.5581596264
0	0	0	0
-1.0342337483	0.8569130339	0.9999537652	1.3897990347
-1.0216578084	0.9716443017	0.9999537652	1.2706722081
1.0922951157	0.8216377349	0.9999537652	1.4720681524
-1.0612751512	0.817353751	0.9999537652	1.4756100212

-1.2468662972	0.8025491941	0.9999537652	8.9512754559
0	0	0	12.7732027325
-1.5881166222	0.7228901076	0.9999537652	2.9985540909
1.1603377009	0.6262942504	0.9999537652	-1.125885481
1.0802573526	0.5139091549	0.9999537652	1.2559570221
-1.0093295716	0.9924640716	0.9999537652	-12.8832386883
1.0359781225	0.9271223662	0.9999537652	1.1415232136
-1.2084217749	0.3012527754	0.9999537652	1.107680005
1.2524620043	0.5894540632	0.9999537652	1.4721443024
2.7933974408	0.6948713406	0.9999537652	6.908559683
-1.0689372098	0.8489196481	0.9999537652	-1.3579476032
-1.4604501748	0.5443773721	0.9999537652	2.5840758705
1.2404159697	0.2544008467	0.9999537652	1.3294948616
-1.2321943066	0.353306342	0.9999537652	1.0572857182
1.3335143304	0.5939529995	0.9999537652	1.2896621072
2.7933974408	0.6948713406	0.9999537652	15.9061931258
6.3075231393	0.4867863046	0.9999537652	10.818785458
1.206749803	0.4132470242	0.9999537652	1.1683770586
-7.0403382421	0.1010692775	0.9999537652	2.9988886918
0	0	0	0
9.8288704632	0.3801744934	0.9999537652	2.996475627
11.2816220699	0.001898	0.9999537652	-1.4779643456
-1.1457279534	0.755679655	0.9999537652	1.6221650026
9.8205505243	0.3808391972	0.9999537652	0
2.7933974408	0.6948713406	0.9999537652	0
-1.4759347606	0.424165953	0.9999537652	-4.5467268531
0	0	0	-1.1250196696
1.1412592751	0.5132094973	0.9999537652	1.1130848926
1.3870101328	0.3998358273	0.9999537652	1.2190380209
-1.1046576488	0.6026013242	0.9999537652	1.1163328357
1.2658813585	0.2136408311	0.9999537652	-1.3261640779
1.1498219992	0.8550109014	0.9999537652	1.2574761276
-1.5565512955	0.3702761191	0.9999537652	-1.1455657765
0	0	0	1.5460384014
-1.1969021862	0.3155990084	0.9999537652	1.2522493324
-1.2188171528	0.3014042843	0.9999537652	-1.0832473793
2.0181702238	0.3286473557	0.9999537652	1.5988002519
-2.9522421189	0.3515372342	0.9999537652	6.1594296767
2.7933974408	0.6948713406	0.9999537652	0
1.0025811227	0.9905442596	0.9999537652	1.4169396562
-1.215335218	0.5392183488	0.9999537652	-1.3957459304
-1.0051359782	0.9786553861	0.9999537652	1.4020060926
-2.6367689659	0.2378468441	0.9999537652	1.5066785066
6.3075231393	0.4867420163	0.9999537652	1.2408886006
-1.507949153	0.462181297	0.9999537652	1.0701872957
1.0294181858	0.9213171184	0.9999537652	-1.0259995933
-1.2234021823	0.381420419	0.9999537652	1.0797672603
1.4309282923	0.2891186569	0.9999537652	-1.0199599076
-1.0207037322	0.9516214767	0.9999537652	-1.169289888

-1.2078158201	0.4644528997	0.9999537652	1.2113001521
1.0963707767	0.6215070788	0.9999537652	1.5153802379
-1.0845813758	0.9233789719	0.9999537652	4.0583010578
-2.9572400577	0.3880473155	0.9999537652	1.0151959523
-1.114021433	0.6488179502	0.9999537652	-1.1999202249
-1.3442764818	0.1095764648	0.9999537652	2.0014830204
1.068981042	0.8119307497	0.9999537652	1.2759049989
27.4155731128	0.1963840634	0.9999537652	-1.4585845073
9.8205505243	0.380839053	0.9999537652	0
-1.3933996626	0.6833647949	0.9999537652	-1.4745350277
1.1340135027	0.6502305977	0.9999537652	1.1232837734
4.5505976667	0.5576212623	0.9999537652	2.9985540909
0	0	0	2.996475627
-1.3958938496	0.009899	0.9999537652	-1.2229252019
1.052430575	0.8669026662	0.9999537652	1.0884938645
-1.0604967947	0.8058871106	0.9999537652	-1.1242890554
-2.2709544313	0.3092339471	0.9999537652	-1.7204385276
2.087766184	0.5274687709	0.9999537652	-3.1733746603
-1.2250923551	0.2954035657	0.9999537652	1.1482472774
-1.0103950142	0.98738051	0.9999537652	1.0589992167
1.2904566672	0.7268769739	0.9999537652	2.9156790983
0	0	0	4.9527501949
1.2953449135	0.2561067794	0.9999537652	1.2577862402
-1.1439240384	0.4924180072	0.9999537652	-1.1809097479
-1.1044242564	0.5792495377	0.9999537652	1.0607869006
1.1309803324	0.6763667569	0.9999537652	1.076027501
-1.1937159529	0.4553479776	0.9999537652	1.2324196296
0	0	0	-1.0560956114
2.7143120644	0.0837077054	0.9999537652	1.3525144016
1.4458709654	0.0932827305	0.9999537652	1.4944598535
-1.0084499073	0.9933947082	0.9999537652	2.2979606844
1.2761324148	0.1598173547	0.9999537652	1.1040489325
1.0614674141	0.7317133577	0.9999537652	1.1130194387
1.0722062209	0.7944033552	0.9999537652	1.0845877786
-2.1244367526	0.1391519762	0.9999537652	-1.3209139133
-1.2309701968	0.7621578392	0.9999537652	1.3825660474
2.2335761138	0.1422037167	0.9999537652	-1.3253616166
1.4216110432	0.3219195352	0.9999537652	1.0050815742
6.3128691894	0.4817622099	0.9999537652	-1.1359208141
0	0	0	2.9985540909
-1.1541081848	0.505860272	0.9999537652	1.132394351
1.2133599992	0.8165072914	0.9999537652	-1.0145369899
4.5505976667	0.5576124784	0.9999537652	2.996475627
0	0	0	2.996475627
0	0	0	8.8639046058
-1.0340849837	0.8236148257	0.9999537652	-1.1510236116
-1.224738827	0.6172814904	0.9999537652	1.1519216724
1.3009747194	0.0848805232	0.9999537652	1.0160184385
-1.1232444378	0.3736792655	0.9999537652	1.0397722754

1.0850169905	0.69459103	0.9999537652	1.053243598
-1.107335893	0.6092259068	0.9999537652	-1.1753083041
1.1407743692	0.7300957571	0.9999537652	-1.1130280866
-1.2617574407	0.4128257321	0.9999537652	-1.1190893491
1.9015109615	0.6236751871	0.9999537652	-1.3581071904
-2.2969122905	0.2159289273	0.9999537652	1.1354053555
1.0212342466	0.9210393278	0.9999537652	-1.218924531
-1.0523487265	0.8425083109	0.9999537652	-1.0873506494
-2.2765895442	0.2462975649	0.9999537652	1.1497024471
25.6156185703	0.2096921881	0.9999537652	-1.1948316779
-1.1953989914	0.2466577335	0.9999537652	-1.1524727856
1.0331640127	0.8454565489	0.9999537652	-1.0305428753
1.0808397513	0.6670436583	0.9999537652	-1.1570412398
-1.1506708574	0.2998208212	0.9999537652	1.0023214339
4.5505976667	0.5575511101	0.9999537652	1.6443474089
-1.45767569	0.0913714901	0.9999537652	1.0908194723
-1.2784842915	0.1527671027	0.9999537652	-1.0711461857
-1.1732921553	0.3394118716	0.9999537652	1.05509979
1.0714859567	0.849976859	0.9999537652	-1.2399569449
-1.7101749736	0.0822708948	0.9999537652	-1.0556161299
-12.9800088947	0.1123482096	0.9999537652	2.7994779515
1.0831038628	0.8428060021	0.9999537652	1.9430798386
1.0769378425	0.7710405733	0.9999537652	1.5734048295
-2.5871290907	0.4928985963	0.9999537652	-1.8601323346
1.1372688736	0.649009526	0.9999537652	-1.3293842842
1.0312051686	0.8090924854	0.9999537652	1.1695103465
1.0901963832	0.6530645632	0.9999537652	-1.0026139604
-2.5920268585	0.1101910039	0.9999537652	-1.795534612
0	0	0	2.996475627
-1.0842628572	0.7234045828	0.9999537652	1.2413255809
-1.055527626	0.716366754	0.9999537652	1.0029527295
2.7933974408	0.6948713406	0.9999537652	2.996475627
-2.0384167005	0.0395000183	0.9999537652	-1.2530192101
-1.6740570647	0.3668076726	0.9999537652	-1.0650092079
-1.1052957679	0.6848931253	0.9999537652	1.0730819615
-1.2323182674	0.6864795118	0.9999537652	1.1106407039
0	0	0	-2.0463152328
1.258140575	0.4398816245	0.9999537652	-1.2917716631
-1.0272464238	0.8830105017	0.9999537652	1.255679041
-1.794017352	0.0967672381	0.9999537652	-1.3933343071
-1.1204616853	0.4658533482	0.9999537652	1.0022793449
-2.587194901	0.5007694689	0.9999537652	2.6095282774
-1.2432283984	0.5440013819	0.9999537652	-1.0917020994
0	0	0	-5.3583704783
0	0	0	2.9985540909
1.2752591223	0.3301134063	0.9999537652	1.2937849956
-1.272582491	0.2850708423	0.9999537652	1.3516044956
-1.0836885942	0.847314717	0.9999537652	-1.0732972824
-1.1340529312	0.4095814907	0.9999537652	1.0179353422

1.0933374333	0.7809873883	0.9999537652	1.319874764
1.2510248049	0.2874277814	0.9999537652	1.3297704096
1.2061157042	0.1238133703	0.9999537652	1.2673923685
0	0	0	6.908559683
1.1023805338	0.5116524054	0.9999537652	1.4200470431
-1.0449919058	0.9092414739	0.9999537652	2.0350233904
0	0	0	-1.1254032646
-1.1631557214	0.5038406697	0.9999537652	1.1719373443
1.1795201921	0.6920312472	0.9999537652	1.8169626502
0	0	0	8.9512754559
1.1230473096	0.8987290504	0.9999537652	1.0675068228
-1.0608818821	0.7584672387	0.9999537652	1.0467540526
1.2740923875	0.2056797158	0.9999537652	1.2251074635
1.1968571056	0.3599671827	0.9999537652	1.0777197968
2.7933974408	0.6948713406	0.9999537652	4.9527501949
0	0	0	-1.8601323346
0	0	0	2.9985540909
4.5505976667	0.5574896519	0.9999537652	-1.4683576122
1.999501975	0.0455063022	0.9999537652	1.154760806
-1.1937981845	0.3933044161	0.9999537652	-1.0961097638
1.1006473838	0.6220474703	0.9999537652	1.4595436118
0	0	0	14.7271569203
1.0522221262	0.8989149056	0.9999537652	1.1884963553
-1.0201255269	0.9180314718	0.9999537652	1.0835182101
1.0106047485	0.9564398959	0.9999537652	-1.1705539547
1.7200622607	0.1460245339	0.9999537652	-1.2671891773
-1.4362615507	0.1150457108	0.9999537652	-1.1857709912
0	0	0	2.996475627
-1.1007163838	0.7447713359	0.9999537652	-1.3932485676
-1.0492674615	0.816901669	0.9999537652	1.3109830036
0	0	0	2.9985540909
1.1593172687	0.8154114746	0.9999537652	-1.0648790285
1.4933961252	0.2617123731	0.9999537652	1.068944881
1.6723076668	0.4779983403	0.9999537652	2.1360046216
-1.1076902759	0.6317857831	0.9999537652	1.0112953478
1.1107076023	0.3727902666	0.9999537652	1.1710321067
-1.2032766627	0.6669104148	0.9999537652	1.0756319973
0	0	0	10.818785458
0	0	0	2.9985540909
1.0223251278	0.9206302712	0.9999537652	1.5565371331
-1.4830351086	0.2886312421	0.9999537652	1.0703354919
1.4096764265	0.2480422075	0.9999537652	1.2624921042
3.3030568913	0.2922814234	0.9999537652	1.1337017128
2.7933974408	0.6948713406	0.9999537652	0
1.0329791895	0.8534836298	0.9999537652	-1.1955907641
1.1250409692	0.5260561978	0.9999537652	-1.008736381
11.5864583426	0.3448989522	0.9999537652	-1.1525615042
-1.0261571745	0.8559028342	0.9999537652	-1.0360406632
-1.1645046325	0.5275660774	0.9999537652	1.2981566449

2.9559514325	0.0497245117	0.9999537652	1.3298332913
-1.1014485384	0.6525217147	0.9999537652	1.1734590987
-1.0231405271	0.9014242143	0.9999537652	1.3085234465
1.4597150658	0.0606350298	0.9999537652	1.5993323501
0	0	0	4.9567375629
-1.068706652	0.718524576	0.9999537652	1.0860494603
1.6826048407	0.2145934198	0.9999537652	1.2014173431
1.3374001555	0.2433790531	0.9999537652	-1.0718400703
-1.1370538804	0.795723457	0.9999537652	1.6450849506
0	0	0	0
-1.5841803933	0.2569454649	0.9999537652	1.5301103929
1.3025872973	0.7465578565	0.9999537652	-1.6601216613
1.1016605519	0.6212140596	0.9999537652	1.1022734496
1.1599572917	0.6982256017	0.9999537652	-1.0428302909
1.0088184708	0.9752998498	0.9999537652	-1.14709072
2.7933974408	0.6948713406	0.9999537652	0
-2.1275749471	0.3790741644	0.9999537652	1.5181428274
1.6158500465	0.0416262393	0.9999537652	1.3404073402
-1.0201114532	0.9023577941	0.9999537652	-1.0218252842
-2.1645000652	0.0641189666	0.9999537652	1.7057482666
-1.0644408749	0.8404809776	0.9999537652	1.1325541061
0	0	0	4.048242769
2.2176218575	0.1893310212	0.9999537652	-1.0896495823
-1.7123365171	0.120720105	0.9999537652	-1.7662436608
2.3950619229	0.0506639864	0.9999537652	1.0843294558
-1.1034081465	0.7203818659	0.9999537652	1.1685706957
0	0	0	1.4521160839
1.112475741	0.5055515338	0.9999537652	1.1686664851
-18.9917210433	0.0490903146	0.9999537652	8.8700459102
1.0520335119	0.8321579136	0.9999537652	1.3516959922
1.010772682	0.9866084536	0.9999537652	-1.6806212191
-1.010254736	0.9639574161	0.9999537652	-1.0049208546
-1.1563128528	0.5087571193	0.9999537652	1.2893508596
1.0375432383	0.967468043	0.9999537652	1.0149761046
1.2519085621	0.5730684305	0.9999537652	1.2281292061
1.4536812284	0.0380408905	0.9999537652	1.0904750919
1.122949656	0.5676134548	0.9999537652	1.2794108582
-1.1181108671	0.6405192867	0.9999537652	-1.0020865701
-1.0862647357	0.6929761604	0.9999537652	1.1219709539
1.0248273979	0.8698455441	0.9999537652	-1.0420899896
1.0140672623	0.9495446152	0.9999537652	-1.0645575112
-2.223376117	0.587910807	0.9999537652	2.4158463203
9.8205505243	0.3808389089	0.9999537652	0
0	0	0	14.7271569203
-1.0598109345	0.7335471395	0.9999537652	1.1641161013
1.1289041984	0.5480571118	0.9999537652	1.3790612785
9.8288704632	0.3801645309	0.9999537652	-1.5231115313
1.0580157395	0.8332065448	0.9999537652	1.4956275721
1.1726872759	0.6624370035	0.9999537652	1.144168923

-1.4148364358	0.4118970207	0.9999537652	1.8437445921
1.2014766201	0.4653826523	0.9999537652	1.3119257997
-1.3654573513	0.5142709449	0.9999537652	1.0234215401
-1.1026704902	0.6680694212	0.9999537652	1.0348685957
-1.0573589437	0.8132744249	0.9999537652	-1.0482857127
1.221084889	0.2433360042	0.9999537652	1.2255914072
1.790983262	0.5810154013	0.9999537652	1.6031258163
-1.115172402	0.4822504437	0.9999537652	-1.1293715415
1.2406045165	0.314761841	0.9999537652	1.3716954177
1.0897697681	0.6282163481	0.9999537652	1.2166761858
1.0796615964	0.7462807995	0.9999537652	-1.2041599944
1.0787918696	0.7468563173	0.9999537652	1.2528440139
-1.3373886055	0.6533220837	0.9999537652	-1.1154666261
-1.2099276495	0.4450813128	0.9999537652	1.3028914884
1.6970882876	0.00214	0.9999537652	1.6598573373
1.2642861534	0.1719467817	0.9999537652	1.1760239978
-1.0497044992	0.8161384067	0.9999537652	-1.0956264652
0	0	0	5.5821463171
1.2246332021	0.7285532155	0.9999537652	-1.6431786337
1.3804849695	0.5835847231	0.9999537652	-1.5871952362
-1.9204725526	0.2581709262	0.9999537652	2.0108391727
9.8205505243	0.3808387647	0.9999537652	-15.1852849638
-1.4446620174	0.3865038973	0.9999537652	-1.0357939207
1.3559615247	0.5271371005	0.9999537652	1.7397737319
-1.2408513535	0.4696579109	0.9999537652	1.1942738533
1.0158675054	0.9430582274	0.9999537652	1.136827562
1.8455489546	0.5921428704	0.9999537652	-1.0913262364
-1.3680392696	0.7427855011	0.9999537652	-4.3763505365
-1.1948629276	0.4760149092	0.9999537652	1.052206256
-1.0467864486	0.7971432371	0.9999537652	1.2175123118
2.7933974408	0.6948713406	0.9999537652	8.8700459102
-1.1263817155	0.4887588236	0.9999537652	1.0119477924
-1.5504587635	0.3024925725	0.9999537652	1.5442891328
1.2130196921	0.1608944659	0.9999537652	-1.0140759481
29.1226294179	0.1908174588	0.9999537652	1.1534465819
-1.0868595418	0.8422012011	0.9999537652	1.1531984169
-1.0794237853	0.5959415775	0.9999537652	1.2048665088
-1.714960605	0.2395394467	0.9999537652	1.0655248182
-1.0876985364	0.6680540713	0.9999537652	1.1949098724
1.0892577239	0.8827742006	0.9999537652	1.2519252348
3.2124200058	0.1554806876	0.9999537652	-1.7615977248
-1.2636266608	0.0897353188	0.9999537652	1.0199796051
-1.1897443838	0.5000550479	0.9999537652	1.0169991694
-1.0846068082	0.5299013838	0.9999537652	-1.0386373114
1.2537728225	0.817040096	0.9999537652	2.8014102743
4.5505976667	0.5574276376	0.9999537652	-1.1606221547
1.0597806836	0.7574972691	0.9999537652	-1.1429791198
1.2734725309	0.1879772344	0.9999537652	1.2781978555
1.412667931	0.2690367016	0.9999537652	1.0380307731

2.7933974408	0.6948713406	0.9999537652	-2.6915507987
-1.0438861455	0.9233280057	0.9999537652	1.5377922323
2.9454946803	0.2263687608	0.9999537652	-1.1978482939
-1.0175223485	0.9346644876	0.9999537652	1.0728472883
1.8005303146	0.4603884802	0.9999537652	1.3007017019
2.7933974408	0.6948713406	0.9999537652	6.908559683
1.2550639593	0.3156236213	0.9999537652	1.3922420879
1.3912288555	0.4889987277	0.9999537652	1.2307849316
4.9998594995	0.1457418858	0.9999537652	1.0508512607
-1.1718367359	0.5194161077	0.9999537652	1.3592049979
4.5505976667	0.5573644116	0.9999537652	0
-1.1204980277	0.3659792872	0.9999537652	-1.0629265317
-1.3124071691	0.159373337	0.9999537652	-1.055219354
1.1245174259	0.5218494635	0.9999537652	1.0624286957
-1.0505312761	0.8532306115	0.9999537652	1.2520490625
1.1177795791	0.493950889	0.9999537652	1.226161162
1.1764408742	0.7468931854	0.9999537652	1.4532048688
1.2075578353	0.5581308423	0.9999537652	1.201183604
8.064174035	0.423894446	0.9999537652	-2.1297401315
-4.3180711894	0.1823926288	0.9999537652	2.6434024479
-1.9371601608	0.4342629151	0.9999537652	-1.7730044157
1.2661052625	0.3295038039	0.9999537652	1.0461907738
1.2611184766	0.4857720203	0.9999537652	-1.0341266844
-1.2687815271	0.7062232209	0.9999537652	1.0602346496
1.0631140495	0.6036503397	0.9999537652	1.1206146427
1.2078834908	0.2333369125	0.9999537652	1.5402573373
1.0038742847	0.9880852996	0.9999537652	1.2589181996
2.3315363418	0.4564183025	0.9999537652	-2.9984907247
23.2549975817	8.81E-12	4.74E-07	14.1962620371
-1.2303138128	0.3440796534	0.9999537652	1.3216696079
0	0	0	-1.7024124862
1.0365898415	0.912901337	0.9999537652	1.2575504785
1.3482326958	0.1306069852	0.9999537652	1.2646827089
-1.0411631314	0.7130224907	0.9999537652	1.1009305386
1.2894344649	0.7055208293	0.9999537652	-1.0719017482
1.3911022238	0.052133966	0.9999537652	1.205545871
1.2724276903	0.5123459585	0.9999537652	1.2165655949
0	0	0	8.8639046058
-1.2239085157	0.0746252929	0.9999537652	1.0048988569
1.0429821129	0.9104894848	0.9999537652	-1.3006417976
1.1529839918	0.5288032838	0.9999537652	1.0918123237
-1.7393750388	0.2117571489	0.9999537652	1.0306529987
-2.0760544151	0.009533	0.9999537652	1.6416773698
1.0166182688	0.9239272062	0.9999537652	-1.2827400313
1.068973822	0.8596566038	0.9999537652	-1.252720257
1.6191925625	0.1643804894	0.9999537652	1.6097966628
1.5101534214	0.2108339425	0.9999537652	1.0697537821
3.2874587288	0.0700612812	0.9999537652	1.7034812636
20.3875267508	0.2406688262	0.9999537652	-1.9046013323

1.1783184722	0.3538727321	0.9999537652	1.1518532945
1.725353503	0.3636004044	0.9999537652	-1.7244260891
1.000233608	0.999359058	0.9999537652	1.5114386081
1.0555534884	0.8090891909	0.9999537652	1.0957379155
0	0	0	1.2587294573
1.2137459782	0.4037685777	0.9999537652	1.2762699538
-1.8867485977	0.0377061994	0.9999537652	1.1033222553
2.1669248089	0.3744490528	0.9999537652	2.585368765
0	0	0	16.7037438866
2.7933974408	0.6948713406	0.9999537652	2.996475627
-1.1100297124	0.5741887904	0.9999537652	1.2194962493
1.1162834585	0.927170544	0.9999537652	5.3031285132
-1.5487724841	0.4732183263	0.9999537652	-1.3233173658
2.6669428532	0.1713276099	0.9999537652	-1.0489576146
-1.1458534393	0.5523751271	0.9999537652	1.0259801231
-1.172755391	0.8035177642	0.9999537652	-1.022317512
0	0	0	-13.9030117058
-1.2736522378	0.5225162932	0.9999537652	-1.1146097401
-2.9548384089	0.3317107331	0.9999537652	1.0009299851
1.6064145452	0.0519553099	0.9999537652	1.4298725333
-1.0197053463	0.9147021669	0.9999537652	-1.0569142754
0	0	0	1.5932034817
1.2626610726	0.558966444	0.9999537652	2.1141100707
1.1930586531	0.349163639	0.9999537652	1.1882020292
1.4551284208	0.4274154188	0.9999537652	2.1559839166
1.6443787287	0.2073011806	0.9999537652	1.2424369576
1.2385263514	0.773184025	0.9999537652	1.1741127097
1.4462436411	0.0402867675	0.9999537652	1.2474923818
-1.1831553223	0.6571225578	0.9999537652	1.0557707978
1.1172647827	0.9265701192	0.9999537652	-2.549728253
-1.1625448402	0.511822214	0.9999537652	1.3761837342
-1.8940726907	0.1684731647	0.9999537652	-1.1336296567
-1.1880616166	0.4344902106	0.9999537652	1.2034913241
-1.0118462754	0.9614286429	0.9999537652	1.068901338
1.1158583321	0.9296908682	0.9999537652	10.818785458
-1.0117844224	0.9525671991	0.9999537652	1.0414976458
-1.0570999302	0.7677317076	0.9999537652	1.3488766665
1.4533569907	0.2610890653	0.9999537652	1.5760573592
1.0692681437	0.6416608804	0.9999537652	-1.0917727204
1.1253337357	0.5284151147	0.9999537652	1.1919489117
-1.3368462221	0.2179361397	0.9999537652	1.2961450191
1.0716600511	0.6308363791	0.9999537652	1.3341437171
-1.82919341	0.1679343834	0.9999537652	-1.1008827529
-1.1641900256	0.3907313538	0.9999537652	1.1516989021
1.3600088454	0.794960303	0.9999537652	-2.1774581292
-1.0995721568	0.701073456	0.9999537652	1.2509746019
2.4133372013	0.2012359494	0.9999537652	-1.0396253615
1.6709396412	0.4771291076	0.9999537652	-1.140745521
-1.1168880873	0.647764417	0.9999537652	1.0386585445

-1.8216877188	0.0938769386	0.9999537652	1.553087122
1.6180171572	0.2244162569	0.9999537652	1.8461714854
0	0	0	65.3692964857
-1.4579253281	0.2242832179	0.9999537652	-2.9273712752
1.1174132796	0.610350854	0.9999537652	2.1215282689
1.1566874978	0.4612140025	0.9999537652	1.3585124789
1.1001378475	0.5630071992	0.9999537652	1.1094847396
0	0	0	-1.8594983184
1.2969713468	0.0388403627	0.9999537652	1.3938865503
1.046546455	0.8087380518	0.9999537652	1.6214163674
-2.7962024446	0.1324034876	0.9999537652	-1.0364027614
1.3864061192	0.0122430718	0.9999537652	1.0257783546
1.1437875311	0.6676054201	0.9999537652	1.2695698098
1.2007224403	0.2953454688	0.9999537652	-1.002306183
-1.0016946171	0.9957930074	0.9999537652	1.402481867
4.5505976667	0.5572989648	0.9999537652	4.9527501949
-1.2671957211	0.3881923883	0.9999537652	1.7495193904
3.8622037496	0.6197991502	0.9999537652	-5.3583704783
0	0	0	9.7116702472
1.240772164	0.2341844335	0.9999537652	1.2365655841
3.0589901989	0.3339458584	0.9999537652	-2.0201197513
2.7933974408	0.6948713406	0.9999537652	0
-1.3156526862	0.3059800019	0.9999537652	-1.1737729783
1.4314051501	0.1437222088	0.9999537652	1.2009705444
0	0	0	4.9527501949
1.0943928865	0.7395724313	0.9999537652	-1.239640147
0	0	0	6.9133480437
1.5353475041	0.002633	0.9999537652	-1.1328500552
-1.6652083331	0.0746765145	0.9999537652	1.2265076099
6.3075231393	0.4866978291	0.9999537652	-5.3608259368
1.1576311514	0.4255179187	0.9999537652	1.3419675087
-1.4922515188	0.4388240763	0.9999537652	1.2757385288
-1.1544072534	0.6168554253	0.9999537652	1.403195266
-1.4027130419	0.121962089	0.9999537652	1.1696068105
0	0	0	2.996475627
-1.0810826794	0.4886952822	0.9999537652	-1.1803354873
-1.0315208323	0.8827043884	0.9999537652	1.1297417728
-1.1873835869	0.4532487137	0.9999537652	1.0115470235
-1.007641984	0.9705733442	0.9999537652	1.2320200731
1.8528871235	0.623566799	0.9999537652	-1.3005386984
0	0	0	2.996475627
-1.4103611906	0.2448005634	0.9999537652	-1.236048459
1.2617955178	0.5675397955	0.9999537652	-1.2915690998
-1.113103362	0.7164743959	0.9999537652	1.0758616108
1.0105618114	0.9709214026	0.9999537652	1.3654235692
0	0	0	4.9527501949
1.7196588555	0.3209604026	0.9999537652	1.3337261284
0	0	0	42.778784484
-1.1289125015	0.6175221819	0.9999537652	-1.5285283537

0	0	0	-1.1248877899
-1.0692899082	0.6078224755	0.9999537652	1.0178781618
20.3888260912	0.2437822987	0.9999537652	2.9917767413
-1.2155057891	0.4364392793	0.9999537652	-1.0474323635
-1.0809529152	0.7523951116	0.9999537652	1.1290111144
1.1652657636	0.4491461466	0.9999537652	-1.0343391894
-1.1000310811	0.6125337027	0.9999537652	1.0398383468
-1.0880283862	0.6225071869	0.9999537652	-1.0281557023
0	0	0	2.996475627
1.0570167493	0.6589692863	0.9999537652	1.0624738029
-1.0625134934	0.6798114583	0.9999537652	-1.042159486
3.0361007025	0.0585367725	0.9999537652	-1.4198061614
1.0956682475	0.5923060717	0.9999537652	1.147316181
-1.1894823918	0.4803817728	0.9999537652	1.0131404587
-1.0342567252	0.9028969232	0.9999537652	-1.2650080359
-1.2504331937	0.3411905857	0.9999537652	1.1039507496
1.0483321523	0.8504863069	0.9999537652	1.1162593019
1.4809743415	0.0272652229	0.9999537652	1.2361365672
1.0031854705	0.9842674442	0.9999537652	1.2744221307
1.1078305751	0.6232202496	0.9999537652	1.377938166
-1.0881705995	0.6354225458	0.9999537652	-1.0461723246
-1.0507575627	0.7279786309	0.9999537652	-1.1869734717
-1.3728063405	0.5755811112	0.9999537652	-1.1294728419
-1.0678530174	0.7910048701	0.9999537652	-1.2475589188
-1.3666809895	0.5073919575	0.9999537652	-1.0372370362
-1.1607408997	0.2743559979	0.9999537652	-1.0206781456
0	0	0	8.8639046058
4.7592049098	0.1553248345	0.9999537652	-1.3639210451
2.7933974408	0.6948713406	0.9999537652	2.9985540909
-2.0529408058	0.0006611	0.9999537652	-1.0956770117
1.2302735751	0.7079163224	0.9999537652	-1.2458876685
11.5766527773	0.3455506293	0.9999537652	-1.8005840097
-1.1117907406	0.4249044388	0.9999537652	-1.0507437192
2.2973669506	0.3345846111	0.9999537652	-1.0867028168
-2.6954260115	0.0133172062	0.9999537652	1.1467439722
1.3836650888	0.3222165281	0.9999537652	-1.030170223
0	0	0	-2.0489010529
1.0761798764	0.7225600451	0.9999537652	1.0617376153
-1.185328559	0.5965075092	0.9999537652	-1.1159401933
1.0464340592	0.8311958812	0.9999537652	-1.2188554536
0	0	0	2.9988886918
1.7184007083	0.1276413844	0.9999537652	-1.3921181425
1.5402932757	0.0725975952	0.9999537652	1.0300175353
1.2078168898	0.6058998415	0.9999537652	-1.1431917398
1.3558346831	0.3360136566	0.9999537652	-1.6302697327
1.0848119754	0.5892831947	0.9999537652	1.5590080317
1.1157762506	0.4500693975	0.9999537652	-1.0939329145
1.1840269212	0.4406232453	0.9999537652	1.0390981295
1.0264851248	0.9163896212	0.9999537652	-1.0022600174

1.0180392536	0.8924319417	0.9999537652	1.2515340388
-1.3482051264	0.3390167359	0.9999537652	1.2090345282
1.047189223	0.8474981941	0.9999537652	1.5603054901
1.1929843318	0.7247799011	0.9999537652	-1.7983697847
1.0781067776	0.9185802503	0.9999537652	1.0454933714
2.9544506384	0.2198890622	0.9999537652	-1.0152221464
25.6156185703	0.2096755115	0.9999537652	2.6430925766
1.1352263809	0.5985792834	0.9999537652	-1.0595678782
-1.0644133438	0.6339380818	0.9999537652	1.1460701641
1.1365971553	0.4154548489	0.9999537652	-1.3521778748
9.8205505243	0.3808386205	0.9999537652	1.2398837679
-1.3697196358	0.7436289251	0.9999537652	-4.813150031
1.0967951028	0.6504743755	0.9999537652	-1.0302140091
2.3879510836	0.1223916363	0.9999537652	-1.2265242384
1.4938176592	0.5512187069	0.9999537652	1.2229210119
1.4042366201	0.0697548182	0.9999537652	1.1767129854
1.8682428625	0.0301557381	0.9999537652	1.2911772256
-1.135518731	0.4414149602	0.9999537652	1.0295811709
1.0944887125	0.9047793702	0.9999537652	-1.5497287574
1.3713640768	0.3651222943	0.9999537652	-1.0589411484
1.4179537639	0.285991108	0.9999537652	-1.2147376262
1.6441361883	0.5983009715	0.9999537652	-1.5124381096
1.4581319152	0.1044767013	0.9999537652	-1.2403745322
1.4007280226	0.5493936643	0.9999537652	-1.3259396095
23.9020991723	0.2167736487	0.9999537652	1.2408886006
-1.1979104008	0.2496866858	0.9999537652	-1.0068689533
1.0206413031	0.9183163186	0.9999537652	-1.0318383234
1.4862234682	0.2813589782	0.9999537652	-1.4173314434
-1.1276934383	0.7214944388	0.9999537652	1.295020922
-1.0495316972	0.8624702881	0.9999537652	-1.2547138577
-1.3578642254	0.411711825	0.9999537652	-1.101386667
-1.1319652264	0.4393482736	0.9999537652	-1.1880943607
0	0	0	2.9985540909
-9.2619917135	0.0528587024	0.9999537652	1.0384669137
2.7933974408	0.6948713406	0.9999537652	0
6.3075231393	0.4866537428	0.9999537652	0
0	0	0	2.996475627
0	0	0	15.7262184124
-1.5875118025	0.7207598535	0.9999537652	0
-1.0572952983	0.7606357168	0.9999537652	-1.0932825359
0	0	0	14.7271569203
8.064174035	0.4238931132	0.9999537652	-9.7542681481
17.3472938331	0.2975186309	0.9999537652	20.5862458385
-2.5838710423	0.5188035227	0.9999537652	52.5265523658
-1.4746252544	0.0166083758	0.9999537652	-1.4023610203
-1.1110347733	0.5816380193	0.9999537652	-1.453449175
-1.2153250435	0.7039222965	0.9999537652	1.2194341811
3.0807666776	0.1934544642	0.9999537652	-1.7929361642
-1.9485415231	0.1182328296	0.9999537652	-1.2727247161

1.1128591252	0.5877177857	0.9999537652	-1.1435111005
-1.1744040886	0.7846187136	0.9999537652	1.088442107
1.1225773715	0.6163209519	0.9999537652	1.3237792469
1.2473794605	0.1596811256	0.9999537652	-1.3766318986
1.4088786693	0.0277483684	0.9999537652	1.0919926119
9.8205505243	0.3808384762	0.9999537652	-1.1428585529
2.7933974408	0.6948713406	0.9999537652	0
1.0230073464	0.9178722265	0.9999537652	-1.1438602843
1.4986288452	0.0336209611	0.9999537652	1.0040404844
1.2836725962	0.2051284299	0.9999537652	1.2947317965
-1.5875118025	0.7207879709	0.9999537652	1.9445739043
0	0	0	4.9567375629
6.3075231393	0.4866097571	0.9999537652	0
-1.6674095463	0.6215916509	0.9999537652	1.2593062028
-1.2010889248	0.2018095525	0.9999537652	-1.020603936
-1.1576191994	0.5166553177	0.9999537652	1.1346327327
-1.0706122399	0.6286314075	0.9999537652	1.1081851589
2.464775932	0.241721847	0.9999537652	-2.1246849375
37.88539235	0.1565225563	0.9999537652	-1.0279151802
2.7957660672	0.7054338742	0.9999537652	0
0	0	0	4.9527501949
-1.0230767376	0.9402903993	0.9999537652	1.2030700015
2.7933974408	0.6948713406	0.9999537652	-1.859280336
1.0341241413	0.9263340948	0.9999537652	-1.0179158912
1.0475771934	0.8254654265	0.9999537652	-1.0323515718
1.2619027659	0.3281694584	0.9999537652	-1.1421431227
11.5962805185	0.3474878338	0.9999537652	4.3982723415
1.0505987295	0.7806694734	0.9999537652	1.1810618133
6.3075231393	0.4865658718	0.9999537652	-2.9687025352
-1.630148962	0.0793599071	0.9999537652	1.285164444
1.603855737	0.6771202434	0.9999537652	2.989954611
-1.1428820038	0.4799547132	0.9999537652	1.2856926805
1.3982968057	0.6380473753	0.9999537652	3.6032308871
-1.0989390372	0.775979211	0.9999537652	1.1572726584
1.045801626	0.7163432158	0.9999537652	1.092421817
0	0	0	2.996475627
0	0	0	9.7116702472
0	0	0	8.8639046058
-1.4834522794	0.1602756224	0.9999537652	-1.0449047584
1.4915752377	0.1893045585	0.9999537652	-1.2791983259
1.201660483	0.1197035248	0.9999537652	-1.2582723471
1.307662308	0.0558844005	0.9999537652	-1.3214829101
0	0	0	12.7820459093
1.2651060306	0.2042493533	0.9999537652	1.3260780833
-1.8798796919	0.0239084044	0.9999537652	-1.0300176988
1.1812966876	0.3464581067	0.9999537652	1.1330419982
2.7933974408	0.6948713406	0.9999537652	1.5908108123
2.7933974408	0.6948713406	0.9999537652	23.4460379037
-1.2007127004	0.3525953124	0.9999537652	-1.1333660957

-1.0914503499	0.4726861003	0.9999537652	-1.5005808652
1.2620379849	0.1983927198	0.9999537652	-1.1218436865
11.5766527773	0.34554149	0.9999537652	-5.3583704783
-1.0000124842	0.9999510716	0.9999537652	-1.1128095414
8.064174035	0.4238917803	0.9999537652	1.2593062028
1.3686888135	0.4442228891	0.9999537652	-1.6615004578
0	0	0	16.7056146828
1.1088986585	0.5971083209	0.9999537652	-1.0685185035
1.0632552373	0.7138776134	0.9999537652	1.1009716393
4.5505976667	0.5572295569	0.9999537652	1.9418822056
-1.5927776507	0.3470359586	0.9999537652	1.5473527806
-1.5978100653	0.1624820466	0.9999537652	-1.1312478619
1.015233579	0.955898366	0.9999537652	-1.3752204598
-1.1715242538	0.4957059291	0.9999537652	1.1041800899
-1.0671475729	0.6081123672	0.9999537652	1.0009250626
2.5214230046	0.0226527795	0.9999537652	2.6015290694
1.1203602583	0.6278355195	0.9999537652	1.2627491082
1.1087202775	0.7312191126	0.9999537652	1.2586670818
1.163807901	0.4023228062	0.9999537652	-1.0688480653
-1.6526009432	0.4830169255	0.9999537652	-1.9650340611
6.3128691894	0.4816632907	0.9999537652	-2.0465436769
0	0	0	4.9527501949
11.5864583426	0.3448799196	0.9999537652	-1.4287688341
-1.9021571549	0.2382410969	0.9999537652	1.1629965089
-1.2400178896	0.2582180547	0.9999537652	1.0399748301
8.064174035	0.4238904473	0.9999537652	0
1.1173386658	0.6113111419	0.9999537652	-1.2945052945
1.2479835075	0.5600170758	0.9999537652	-1.0562313793
-1.2981663134	0.1785567034	0.9999537652	1.4605405247
7.4603922969	0.0630497486	0.9999537652	1.5522360599
-1.1066390797	0.5924795738	0.9999537652	-1.0577386521
1.2843183796	0.2721112948	0.9999537652	1.9177846405
-1.413531519	0.0513771193	0.9999537652	-1.3539224495
1.0876776269	0.6430444424	0.9999537652	-1.2570679864
-1.2706465183	0.2259442406	0.9999537652	1.0247510359
0	0	0	-2.3124617434
-1.0094167007	0.9589879991	0.9999537652	-1.0036945121
1.5136569565	0.6634036298	0.9999537652	5.4963641385
-1.2272463128	0.3017957955	0.9999537652	1.0125876117
1.6850765316	0.0005041	0.9999537652	1.1868317555
-1.5875118025	0.7206984509	0.9999537652	1.4531133406
0	0	0	12.7732027325
-1.0237695497	0.8785019532	0.9999537652	1.3786817672
-1.2462090013	0.2460552055	0.9999537652	1.1639645484
1.201227144	0.4184330786	0.9999537652	-1.0581579587
-1.1270573621	0.5848386097	0.9999537652	-1.0162217574
2.8162992884	0.366711651	0.9999537652	1.0674845818
1.0192274061	0.9277706475	0.9999537652	-1.1806977151
1.9330108539	0.0469266979	0.9999537652	-1.0139243548

2.7933974408	0.6948713406	0.9999537652	8.8639046058
1.2192329692	0.284085464	0.9999537652	-1.0561222345
-1.1973802577	0.2954295151	0.9999537652	1.0100969024
3.1790945967	0.1208827335	0.9999537652	1.3604797255
-1.1110973787	0.5364931328	0.9999537652	1.1212896775
3.5458425332	0.2611345469	0.9999537652	-1.1360245985
1.0145797596	0.9783905603	0.9999537652	-1.0770459476
-1.1457555741	0.9107279124	0.9999537652	9.7116702472
1.0459648406	0.7030373068	0.9999537652	1.1630923518
4.5505976667	0.5571525373	0.9999537652	-1.8588429871
1.1217075242	0.5961942016	0.9999537652	1.3662981026
1.0987495796	0.6044009343	0.9999537652	-1.0639913339
-1.0085355311	0.9930286747	0.9999537652	3.7005872492
-1.4207399578	0.2102207842	0.9999537652	-1.1876845416
1.1533888548	0.7389699071	0.9999537652	1.0699633757
-1.1734800276	0.2544550374	0.9999537652	-1.1307002395
-5.2162291127	0.0147190029	0.9999537652	1.9764541971
1.049812312	0.8279994571	0.9999537652	1.2087333127
1.0033015556	0.9846064691	0.9999537652	1.0435541647
1.04645314	0.7796204264	0.9999537652	-1.0329867966
1.5614767778	0.4210866863	0.9999537652	-1.5770365571
1.5920970871	0.1660608162	0.9999537652	1.0532462097
9.8205505243	0.3808383319	0.9999537652	2.996475627
1.1795618635	0.551770623	0.9999537652	1.0779819142
-1.0362977598	0.9298507188	0.9999537652	-1.6083927275
4.5505976667	0.5570582932	0.9999537652	0
1.0786153018	0.6993284315	0.9999537652	1.1694072278
-1.0960753358	0.7575774429	0.9999537652	-1.0923038235
-3.3110661094	0.2931365506	0.9999537652	1.135404339
1.1119531472	0.6248940358	0.9999537652	1.3089997572
2.7933974408	0.6948713406	0.9999537652	0
1.0773205754	0.8153941806	0.9999537652	1.0136158815
-1.111148269	0.6671250447	0.9999537652	-1.0299332478
2.3790091494	0.2089509541	0.9999537652	1.7988939724
-1.1501724092	0.3240144388	0.9999537652	-1.0919011508
1.2991018857	0.088124634	0.9999537652	1.1488115036
-1.167348377	0.498502891	0.9999537652	-2.036936333
1.1743582038	0.5442372801	0.9999537652	-1.0861035764
-1.4092459178	0.1491112736	0.9999537652	1.0547377881
1.643510367	0.5932005263	0.9999537652	6.1502733747
0	0	0	0
1.2794027342	0.3245397678	0.9999537652	-1.3635732
-2.5006943961	0.2204003117	0.9999537652	-1.0787540783
1.5561082845	0.4495036593	0.9999537652	-2.2662643201
-1.336888917	0.344325085	0.9999537652	1.0476271914
1.1921859122	0.6162769501	0.9999537652	-1.0834843518
0	0	0	1.2400291296
1.262522021	0.3584304408	0.9999537652	1.115410668
1.2214173095	0.3886719093	0.9999537652	-1.0599570888

1.1321960974	0.3384355703	0.9999537652	-1.0937456196
1.1228908358	0.7092998575	0.9999537652	1.1732317338
-1.0263983145	0.8419186948	0.9999537652	-1.2779217837
-2.0029042714	0.0148460932	0.9999537652	-1.4008374892
0	0	0	1.6447327969
1.5281694116	0.148045007	0.9999537652	-1.3052482672
1.0266084653	0.8864352549	0.9999537652	-1.450069903
1.2895507939	0.370531601	0.9999537652	-1.0779759069
-1.0640418707	0.8208771626	0.9999537652	-1.3361678085
1.2877273268	0.5595665879	0.9999537652	1.1449820521
-1.1690605878	0.4282251654	0.9999537652	-1.0088598524
1.1618755293	0.5070219505	0.9999537652	-1.5060201569
-1.3910428484	0.548974263	0.9999537652	-1.2615639262
-1.1121733088	0.3779759811	0.9999537652	-1.3383494434
-1.1560239903	0.5337387881	0.9999537652	-1.0206054289
-4.9246171726	0.0160367423	0.9999537652	1.1283576093
8.064174035	0.4238891141	0.9999537652	-5.2664224014
1.2004471468	0.6384308557	0.9999537652	-1.7754312169
1.0119907617	0.9299615634	0.9999537652	1.0054194995
1.0851757923	0.6166899583	0.9999537652	1.1959947667
1.4005015264	0.4156323712	0.9999537652	-1.2504232996
-1.0309904128	0.9164295386	0.9999537652	-1.0464917544
-1.1020098136	0.7842688625	0.9999537652	1.3825778496
0	0	0	0
1.2435605641	0.7150506791	0.9999537652	-1.4391975388
13.3324809638	0.318861735	0.9999537652	2.0162086657
0	0	0	8.8639046058
-2.0444867044	0.2889852069	0.9999537652	-1.0829866837
-1.1615046801	0.4666954903	0.9999537652	-1.1340186059
1.0723185921	0.5797536219	0.9999537652	1.0738192242
1.0893016951	0.5287689342	0.9999537652	-1.0708010149
-1.1363553669	0.5655825258	0.9999537652	1.0074689419
-1.1619421561	0.8738173294	0.9999537652	1.3826538357
1.0285359703	0.8265079464	0.9999537652	-1.0654882021
-1.0128933798	0.9184869252	0.9999537652	-1.0514476481
-3.2135835145	0.1883770604	0.9999537652	1.336777274
-1.0221660434	0.9018416527	0.9999537652	-1.4393627314
-1.0318248652	0.8976434066	0.9999537652	1.494926664
-1.0532227046	0.8370985413	0.9999537652	1.087661788
-1.2755405754	0.1657315287	0.9999537652	1.0310755018
1.0682731901	0.6530677214	0.9999537652	1.032892103
1.0993426524	0.7609479835	0.9999537652	1.0669598816
2.6861488177	0.2572639209	0.9999537652	1.6021060248
8.064174035	0.4238877808	0.9999537652	-3.382249888
0	0	0	2.996475627
-1.0427325085	0.9275374657	0.9999537652	1.4517793168
-1.3061638095	0.4139376104	0.9999537652	1.2304521604
1.1659257209	0.1768059953	0.9999537652	1.1759280256
-1.2901514672	0.6027253403	0.9999537652	-1.1292156252

0	0	0	0
1.3035690226	0.1877054737	0.9999537652	-1.0582720996
0	0	0	2.996475627
-1.1187955526	0.6207354082	0.9999537652	-1.1868304815
-6.9736190158	0.2250321147	0.9999537652	8.8700459102
1.1184618766	0.4695960748	0.9999537652	-1.0067434559
-1.0825658782	0.6483631256	0.9999537652	-1.1612836244
1.385663464	0.7405957135	0.9999537652	1.5337599584
-1.1502575669	0.464086414	0.9999537652	1.0745711763
1.0079681429	0.9480144504	0.9999537652	1.0960060476
-6.9764533263	0.2591527081	0.9999537652	2.996475627
-1.1075853887	0.6403206776	0.9999537652	-1.0314963485
-1.190013612	0.4069965635	0.9999537652	-1.0209271148
-1.2078453842	0.4229067126	0.9999537652	-1.0826481551
2.637098445	0.0966374395	0.9999537652	-1.1202893487
1.097906629	0.6663708067	0.9999537652	-1.2134214314
1.3061023617	0.0952456961	0.9999537652	1.1627686083
-1.0158729707	0.9157053863	0.9999537652	1.2937215116
0	0	0	4.9527501949
0	0	0	-1.4683576122
-1.1981038122	0.3166691958	0.9999537652	1.0628253109
-1.0048017054	0.982109323	0.9999537652	-1.1171765848
1.1514532867	0.6458006393	0.9999537652	1.4168598407
-1.2115650054	0.3060469981	0.9999537652	-1.1804667828
-1.2101335528	0.3904605532	0.9999537652	-1.3684132159
-1.3222955607	0.5926978433	0.9999537652	-1.0725053996
1.1071458299	0.6334904392	0.9999537652	1.2634355722
-1.1030068143	0.4911369511	0.9999537652	-1.028598447
-1.9998864941	0.2337115107	0.9999537652	-1.8579406165
1.0353919069	0.8747778288	0.9999537652	-1.0980714093
-1.0127004261	0.9578286179	0.9999537652	1.1584793983
-1.0720413622	0.7359160667	0.9999537652	1.0956177841
1.5410453821	0.1325034642	0.9999537652	1.460984529
-1.4200868943	0.064751511	0.9999537652	1.0060084609
1.2252048389	0.5931262283	0.9999537652	1.0759551413
9.8205505243	0.3808381876	0.9999537652	-1.3598304575
2.1456333787	0.2686698026	0.9999537652	-1.5553070134
1.6032957979	0.6856291136	0.9999537652	-3.8938703542
1.0339032799	0.8858133141	0.9999537652	2.0909603275
1.0053488917	0.9763241364	0.9999537652	1.1386988599
-1.0071406522	0.9646468584	0.9999537652	1.1093031277
1.5094197345	0.5421031518	0.9999537652	-1.051661731
8.0712632467	0.4228070877	0.9999537652	6.8557843057
1.1194713798	0.6182158685	0.9999537652	-1.352185903
-1.7791703059	0.0189865663	0.9999537652	-1.4144082985
-1.3922626495	0.2908124458	0.9999537652	-1.0677157328
30.9018162087	0.1808244771	0.9999537652	2.4624880301
1.1686731033	0.7287411549	0.9999537652	-1.2070914674
-1.668764792	0.6524660488	0.9999537652	1.9418822056

-1.0160612632	0.9727557129	0.9999537652	1.2135193374
-2.9254107669	0.0742978173	0.9999537652	2.2865482986
-1.0733763588	0.8487857752	0.9999537652	1.0660970169
1.4498325328	0.2114994516	0.9999537652	1.205209037
-1.0921302295	0.6916131318	0.9999537652	-1.237865632
-1.2130208078	0.5486735336	0.9999537652	-1.1307495516
1.0605394327	0.6843551447	0.9999537652	1.5063914963
-1.0197299234	0.9592503149	0.9999537652	1.166233864
1.0381523107	0.8948682286	0.9999537652	1.2214458812
-1.2493308946	0.3030913026	0.9999537652	-1.303419522
-1.1086614441	0.4970131044	0.9999537652	-1.3218553704
1.0188456169	0.9222381186	0.9999537652	1.0250114612
1.3590871487	0.2067824681	0.9999537652	-1.2863860476
-1.0278977896	0.9389399672	0.9999537652	-1.0124132194
-1.37106644	0.1813753108	0.9999537652	1.0510366557
6.3075231393	0.4865220867	0.9999537652	1.2591586474
1.0709594809	0.8759281669	0.9999537652	1.0841218071
1.0789024281	0.6299049109	0.9999537652	1.1806563242
-1.2232107561	0.5290477151	0.9999537652	1.0471119896
-1.3527263764	0.264584807	0.9999537652	1.1800981769
1.0863825424	0.5263542355	0.9999537652	1.4996420382
-1.1279436419	0.6584387789	0.9999537652	1.058339823
-1.0080753521	0.9707702803	0.9999537652	1.1899518177
-1.2746918797	0.3149053553	0.9999537652	1.2959766283
-1.0365821876	0.8703971439	0.9999537652	-1.0756392781
1.5283877156	0.2078925759	0.9999537652	-1.0404628629
9.8205505243	0.3808380432	0.9999537652	-1.6435965623
-1.0837489199	0.7239104456	0.9999537652	1.0552917305
0	0	0	3.6255745018
-1.0536697353	0.7787692181	0.9999537652	1.2410901277
1.185023511	0.3644652508	0.9999537652	1.2016746579
1.065339183	0.7452987136	0.9999537652	-1.0636268881
-1.0466247305	0.7675908831	0.9999537652	1.2325282921
-2.4086999574	0.2737576869	0.9999537652	5.8001127949
-1.5423955481	0.328942826	0.9999537652	1.1192883622
0	0	0	4.9527501949
1.586170423	0.2209403175	0.9999537652	-1.0154949376
0	0	0	-5.3608259368
6.3130691196	0.4797905908	0.9999537652	10.818785458
0	0	0	12.7820459093
4.7573924101	0.1600870481	0.9999537652	4.9561842702
-12.9852817769	0.1147570896	0.9999537652	0
-1.1722807207	0.8571676859	0.9999537652	20.5862458385
1.1632726083	0.5051878508	0.9999537652	-1.2447273879
-1.1888187072	0.2580843085	0.9999537652	-1.374663208
2.7933974408	0.6948713406	0.9999537652	2.9985540909
-6.9736190158	0.2250321147	0.9999537652	4.9527501949
-1.3933642051	0.7077473685	0.9999537652	-1.462791692
-1.3472167972	0.5063197174	0.9999537652	-1.0455160135

-25.00673561	0.030469531	0.9999537652	0
1.9059187914	0.4671380118	0.9999537652	-1.8075300459
0	0	0	0
0	0	0	2.9985540909
3.2403799754	0.0189785748	0.9999537652	1.7597763749
-6.9736190158	0.2250321147	0.9999537652	6.908559683
2.0896001704	0.5218039645	0.9999537652	2.9985540909
9.8205505243	0.3808108655	0.9999537652	14.7271569203
1.4997369472	0.3164335185	0.9999537652	1.009365708
-2.2367676968	0.3913890677	0.9999537652	1.3152290032
3.2652031108	0.1135577923	0.9999537652	1.0201618369
1.3282415411	0.7111199514	0.9999537652	-1.6830699048
-1.5731601222	0.128622098	0.9999537652	1.4198708893
1.2785460571	0.2288957227	0.9999537652	1.0019917518
1.5513190246	0.003117	0.9999537652	1.1317263111
2.7933974408	0.6948713406	0.9999537652	9.7116702472
0	0	0	2.996475627
-2.956038745	0.3205463475	0.9999537652	3.6950602847
1.0597826207	0.8150868337	0.9999537652	-1.7191746425
-5.7694389001	0.1064318809	0.9999537652	0
-2.5435664515	0.2868407697	0.9999537652	0
9.8205505243	0.3807837837	0.9999537652	-1.0828846244
1.0835184344	0.8653904195	0.9999537652	1.2114093143
4.0347077664	0.2007388651	0.9999537652	2.9985540909
2.9478137269	0.2171695007	0.9999537652	2.996475627
2.0885615936	0.5184501642	0.9999537652	-5.3608259368
1.1256551335	0.8550932486	0.9999537652	2.9985540909
-6.9736190158	0.2250321147	0.9999537652	0
-1.4067833083	0.5016335852	0.9999537652	49.3647206798
2.3585309934	0.2363096842	0.9999537652	-2.3893631603
-6.9764533263	0.2591610297	0.9999537652	2.9985540909
0	0	0	-4.9108225367
-2.0025825252	0.4565366416	0.9999537652	4.9561842702
-4.3986109171	0.0647848049	0.9999537652	-5.3589986948
1.1172293767	0.9254471598	0.9999537652	2.9985540909
1.0362439076	0.9657000127	0.9999537652	-1.4145214232
-3.8100204382	0.0433819871	0.9999537652	-1.859280336
0	0	0	0
0	0	0	-1.3076992656
-1.0950954595	0.8432534259	0.9999537652	-1.1039775761
1.6463457326	0.5770783364	0.9999537652	1.1487875235
1.1821296961	0.6067513781	0.9999537652	-1.0241995621
1.1289037299	0.7578127575	0.9999537652	1.0843261094
1.1682841801	0.6940597841	0.9999537652	-1.0668168928
6.3075231393	0.4864784014	0.9999537652	0
1.8028740077	0.4327252229	0.9999537652	-1.39839735
1.0481614378	0.9224812408	0.9999537652	-1.7793027384
-1.4507123533	0.5771189461	0.9999537652	-1.0535935422
-1.5077992302	0.4103352239	0.9999537652	-1.4743534501

0	0	0	2.996475627
-1.0556351465	0.7401574136	0.9999537652	-1.0965162164
-6.9736190158	0.2250321147	0.9999537652	8.8639046058
-1.0254840392	0.9630956218	0.9999537652	-1.770694404
2.7933974408	0.6948713406	0.9999537652	0
-1.4751643279	0.4023864197	0.9999537652	1.0921666199
2.7933974408	0.6948713406	0.9999537652	0
1.1840195478	0.1996679028	0.9999537652	-1.0674789927
-1.1607833186	0.4971058233	0.9999537652	-1.1590401749
1.3040410104	0.2505478845	0.9999537652	1.0490292685
-1.3034475687	0.7660079242	0.9999537652	-1.0547761065
0	0	0	8.8639046058
-1.005295052	0.9797974733	0.9999537652	1.0574546485
-3.4215393891	0.1594796204	0.9999537652	1.0545339774
1.7340816349	0.456979915	0.9999537652	33.3754495834
-1.4007421347	0.2446117861	0.9999537652	1.1526333825
1.3068443743	0.0769983581	0.9999537652	1.0982129056
-1.0859085205	0.6521095782	0.9999537652	-1.4173032361
1.1681004938	0.5278603723	0.9999537652	-1.1786414434
-1.1173354264	0.4789760151	0.9999537652	-1.0468180094
-1.136341756	0.4842657152	0.9999537652	1.3778975001
1.0092984939	0.9439961935	0.9999537652	-1.1658213379
6.3075231393	0.4864348159	0.9999537652	1.4307087091
-1.0623230887	0.8946225583	0.9999537652	-1.1311590874
-1.4150256844	0.2172770523	0.9999537652	-1.2893654143
1.0147290952	0.9178855089	0.9999537652	-1.0741600643
-1.3329838577	0.2307030686	0.9999537652	-1.1203943854
-1.0048249416	0.9802525692	0.9999537652	-1.1590845563
-1.0342635618	0.780208478	0.9999537652	-1.2233396533
1.0011886599	0.9958371418	0.9999537652	-1.1872591097
1.7140509772	0.0233540187	0.9999537652	1.1399696131
2.7933974408	0.6948713406	0.9999537652	8.8639046058
1.678527217	0.0269993884	0.9999537652	-1.2099214114
1.0903560025	0.7011811117	0.9999537652	-1.1419323411
-6.9762758667	0.2574789448	0.9999537652	0
1.2987672615	0.5563426288	0.9999537652	1.4365560648
-1.2669294153	0.1885299475	0.9999537652	-1.0037164875
-1.5875118025	0.7206089826	0.9999537652	3.1840303018
1.0687364382	0.5873093811	0.9999537652	-1.0112879402
1.1590517798	0.2176231676	0.9999537652	-1.009716291
1.3324604159	0.5979840553	0.9999537652	1.0289648288
1.0741875757	0.8167989425	0.9999537652	-1.2710342057
1.1161480477	0.6165957258	0.9999537652	1.2356293295
1.1158583321	0.9296794942	0.9999537652	1.4875735439
2.5591644743	0.2765005285	0.9999537652	1.2315827612
8.064174035	0.4238851146	0.9999537652	-2.1734470428
-1.0254259049	0.9292244373	0.9999537652	1.1229749837
-1.610091706	0.0997622898	0.9999537652	-1.1109219045
1.3183638953	0.1153806567	0.9999537652	1.2215749773

2.7933974408	0.6948713406	0.9999537652	-1.1248877899
1.0259887574	0.8389620022	0.9999537652	1.2281404994
1.1595950732	0.5937345789	0.9999537652	1.2697639665
1.2913910109	0.1705841776	0.9999537652	-1.2611234932
1.278891521	0.6736448536	0.9999537652	1.9682972556
1.0458377827	0.8849353392	0.9999537652	-1.1351719003
1.1444741366	0.5029663632	0.9999537652	-1.1957596544
-1.0899268619	0.6262674875	0.9999537652	1.149928073
1.8471118167	0.5853667708	0.9999537652	-2.2389069466
-1.0066196254	0.971880842	0.9999537652	-1.373431707
0	0	0	9.7116702472
0	0	0	2.9988886918
0	0	0	2.996475627
0	0	0	4.9527501949
1.8194592678	0.0678513817	0.9999537652	1.3596326619
1.399194136	0.0205077282	0.9999537652	-1.0421982907
-1.8302136322	0.2181223483	0.9999537652	1.0300536047
-1.0256316245	0.9616368238	0.9999537652	-1.710761321
4.5544554512	0.5691493408	0.9999537652	2.9988886918
1.0415128524	0.8293254855	0.9999537652	-1.1557755125
1.1801090485	0.2585825597	0.9999537652	-1.0804762147
-1.5561044836	0.4814137536	0.9999537652	3.3933901003
-1.2772483432	0.229684108	0.9999537652	-1.4508343673
-1.1286633091	0.7943127178	0.9999537652	1.2132260292
1.0494886587	0.7105243268	0.9999537652	1.0206936996
1.0349698144	0.8098355697	0.9999537652	1.0238649754
1.3600434408	0.7948580329	0.9999537652	1.8289353302
1.292993688	0.2514867306	0.9999537652	-1.2723778944
1.8746187661	0.1363966058	0.9999537652	1.0105988956
-6.0768340904	0.0391945461	0.9999537652	-1.363546436
1.3257589065	0.1489058121	0.9999537652	1.0597719093
-1.3093027525	0.2076543837	0.9999537652	-1.2627050234
2.3677225249	0.0954742241	0.9999537652	-1.3555674202
-1.7236237864	0.0512494097	0.9999537652	-1.42560978
2.7957660672	0.7054362173	0.9999537652	-1.3601489831
2.7933974408	0.6948713406	0.9999537652	-1.1250196696
2.7957660672	0.7054385613	0.9999537652	0
-2.4416556705	0.3741597953	0.9999537652	-1.6785798811
2.3638535568	0.00335	0.9999537652	-1.5800538106
-1.1767301283	0.7356508434	0.9999537652	-1.2431995102
0	0	0	-1.4426061733
-1.0514502364	0.8521875302	0.9999537652	-1.2511268014
1.4398079274	0.6076968255	0.9999537652	-1.0661031289
-1.1574050327	0.5009366663	0.9999537652	-1.1390484015
1.0678935042	0.7082410069	0.9999537652	-1.1456049027
1.0621646818	0.8255575251	0.9999537652	-1.4128809283
2.2253482524	0.168155229	0.9999537652	-1.119419279
-2.0795578895	0.2303025552	0.9999537652	2.3464201963
2.7933974408	0.6948713406	0.9999537652	0

3.1394606969	0.0208986664	0.9999537652	-1.1377627016
3.6937166632	0.1516359407	0.9999537652	-1.0019440938
2.0461586227	0.0498893726	0.9999537652	-1.0038926288
0	0	0	1.5560296184
2.7933974408	0.6948713406	0.9999537652	-1.0952583848
0	0	0	9.7116702472
-1.7435834966	0.3751438191	0.9999537652	-1.0599737366
1.0271041699	0.8604223939	0.9999537652	1.1169811054
1.0674653943	0.6421591476	0.9999537652	1.0374757509
1.057075147	0.7685421702	0.9999537652	-1.0208487261
1.1126440841	0.6287412745	0.9999537652	-1.137428435
1.643510367	0.5932811336	0.9999537652	-2.2269755185
1.6024984014	0.6862123674	0.9999537652	1.0751932413
-1.112649727	0.5453055882	0.9999537652	-1.1804856425
1.6024984014	0.6863020091	0.9999537652	1.3224475497
18.5983228161	0.2579912666	0.9999537652	-1.0422531512
15.0880352532	0.2954313143	0.9999537652	1.1356707315
1.0588081849	0.8076807353	0.9999537652	1.0502267737
1.1268789908	0.6209917575	0.9999537652	-1.1763909563
2.7933974408	0.6948713406	0.9999537652	1.590997316
-1.0756177415	0.8277185279	0.9999537652	-1.2238171391
-1.3808293235	0.2507868229	0.9999537652	-1.0386188186
-1.1171933108	0.3383807421	0.9999537652	-1.0533887887
-1.1868411067	0.3373531855	0.9999537652	-1.1004635847
1.374894702	0.4249801248	0.9999537652	1.656499278
1.4659701501	0.2466513768	0.9999537652	1.2209111776
1.1331613376	0.3948453618	0.9999537652	-1.161147529
1.8531875067	0.2370570258	0.9999537652	-1.4584988872
-1.1394252305	0.4821147396	0.9999537652	-1.4222473021
1.1374046657	0.3826585234	0.9999537652	-1.2974135692
-1.3090139168	0.5492284828	0.9999537652	-1.2073079744
1.0204921805	0.8828548032	0.9999537652	1.1198468788
2.1658501647	0.4036988285	0.9999537652	1.1401808029
-1.334767918	0.5625675086	0.9999537652	-1.0576354638
1.3371354523	0.2455078369	0.9999537652	1.1314061565
-1.129145102	0.5679877426	0.9999537652	-1.0598062483
1.0010854785	0.9953532227	0.9999537652	-1.3977221883
1.4233266177	0.1624503722	0.9999537652	1.0955094207
2.7933974408	0.6948713406	0.9999537652	1.5318210718
1.0521732606	0.6591520173	0.9999537652	-1.6239152791
1.0791230727	0.7325999065	0.9999537652	-1.2126413694
-1.0444653896	0.8168363725	0.9999537652	-1.102248457
-1.5646820981	0.3843686079	0.9999537652	-1.4970321636
1.293233992	0.0481091914	0.9999537652	1.1042075406
-2.5871290907	0.4950945092	0.9999537652	4.9527501949
1.2121185951	0.2159519009	0.9999537652	-1.1392761998
1.0473634717	0.7153294189	0.9999537652	1.1147109755
1.2780162304	0.3191860887	0.9999537652	1.3098872242
-1.2273076528	0.2075118121	0.9999537652	1.0504211725

1.0336671984	0.8024098427	0.9999537652	1.0656090756
-1.1717451824	0.842242725	0.9999537652	-1.0363457245
1.5916509819	0.1687566686	0.9999537652	-1.0202639665
-1.3395903204	0.1040614364	0.9999537652	1.1809623962
1.0374602287	0.9152758405	0.9999537652	-1.0238013002
1.2611060645	0.2336393493	0.9999537652	1.0666556075
-1.0208090526	0.8537385697	0.9999537652	-1.0092038547
1.5815875305	0.6773602241	0.9999537652	2.5355690913
2.7933974408	0.6948713406	0.9999537652	1.1705662818
-1.2797682501	0.1744049023	0.9999537652	1.1639941059
-4.8135938501	0.1784471282	0.9999537652	6.9059398214
4.5545995984	0.5696724671	0.9999537652	-1.1613446338
0	0	0	0
-1.0381732196	0.8047062221	0.9999537652	-1.155280022
15.0880352532	0.2955141276	0.9999537652	-1.5088059915
-1.0493965567	0.7652737093	0.9999537652	-1.196490283
1.7512805614	0.4586763835	0.9999537652	-1.5377948106
-1.5748119492	0.1638121189	0.9999537652	1.1142934311
-2.3309889016	0.0582592716	0.9999537652	-1.1460036793
1.3629585105	0.4891027116	0.9999537652	-1.3645279797
0	0	0	1.4537866576
1.0012234717	0.9939819803	0.9999537652	1.0616512271
1.1988901647	0.3944443032	0.9999537652	-1.0669827063
-1.0212657651	0.8570355903	0.9999537652	1.1472153602
1.7496094965	0.0465398404	0.9999537652	1.0335171055
-1.0551684694	0.7902791308	0.9999537652	-1.0181945116
1.1887725976	0.7459692189	0.9999537652	-1.167242164
-1.1399317987	0.4036820198	0.9999537652	-1.0864216024
-1.0048262473	0.9785013941	0.9999537652	1.2069716752
-6.9764533263	0.2591693543	0.9999537652	-2.5408482902
-1.1279672712	0.3647144041	0.9999537652	-1.0842108578
-1.0638626403	0.7433583271	0.9999537652	-1.0123878144
1.4744829524	0.4679304542	0.9999537652	2.0125565823
-1.444237639	0.2134880837	0.9999537652	-1.0378827035
-1.0474416127	0.8090311684	0.9999537652	1.0809946696
-28.7225758273	0.0587692404	0.9999537652	0
-1.0349725645	0.8924276428	0.9999537652	-1.2406667354
1.1169928711	0.5592902785	0.9999537652	1.2551218789
3.5444019705	0.2680766623	0.9999537652	-2.2893450003
-1.4828085853	0.0222864868	0.9999537652	-1.0189585148
-1.0685222725	0.861801674	0.9999537652	-1.7000936341
1.1841787841	0.1448514514	0.9999537652	1.2317474762
-1.1623847729	0.887198882	0.9999537652	2.1874714972
-1.4090706138	0.1499911461	0.9999537652	-1.1504541598
-1.4927097311	0.0583006136	0.9999537652	1.1023356584
6.3075231393	0.4863913298	0.9999537652	0
0	0	0	12.7732027325
4.5505976667	0.556961481	0.9999537652	2.996475627
0	0	0	1.3687080685

2.5626013909	0.2583666894	0.9999537652	1.0386350901
-1.0583306168	0.8346524598	0.9999537652	1.3049298416
-1.0897087179	0.7013952254	0.9999537652	1.2890853677
2.7933974408	0.6948713406	0.9999537652	6.908559683
-1.3112478436	0.5797092353	0.9999537652	-3.0890541028
2.7933974408	0.6948713406	0.9999537652	0
-1.075354506	0.8812920875	0.9999537652	-1.3619468048
1.2187593053	0.646450408	0.9999537652	-2.1420558366
-1.1688020512	0.5543746649	0.9999537652	-1.0505422135
-1.119170163	0.5558366868	0.9999537652	-1.2361720544
1.0719361517	0.9204580613	0.9999537652	2.7558973556
15.2205810263	0.3052951562	0.9999537652	1.9307645037
1.0829215919	0.5234163166	0.9999537652	1.1523433234
1.0392987413	0.8614009226	0.9999537652	-1.01416649
-1.1141213666	0.5802525906	0.9999537652	-1.339061348
-1.0473748842	0.7587590104	0.9999537652	-1.0491877748
-1.0887426178	0.5074536948	0.9999537652	-1.0904939959
-1.0120882276	0.9554535527	0.9999537652	1.2867793222
0	0	0	-1.8579915692
0	0	0	4.9527501949
-2.5388858734	0.0182615408	0.9999537652	1.2359037312
-1.2172862773	0.1333151906	0.9999537652	-1.1949743817
1.1902886668	0.3171223424	0.9999537652	-1.0173166018
-2.0113664731	0.3218543358	0.9999537652	1.0235501953
1.1170390388	0.5307952327	0.9999537652	1.028231189
-6.9764533263	0.2591776818	0.9999537652	20.5862458385
1.0404306232	0.7984801853	0.9999537652	1.1330523478
-1.0872200494	0.6782048461	0.9999537652	1.0433514422
1.0882474284	0.4908888412	0.9999537652	1.302918515
1.5301326131	0.5572343884	0.9999537652	-1.1045292627
4.5505976667	0.556872722	0.9999537652	2.996475627
-1.1825442559	0.4389861437	0.9999537652	-1.9465370399
-1.0487971014	0.7291124127	0.9999537652	1.039390121
1.3218378061	0.186107566	0.9999537652	-1.0156737093
1.0290969881	0.8258427175	0.9999537652	-1.1129489101
1.5030896757	0.3450820806	0.9999537652	-1.1406774814
1.1929259108	0.1952989603	0.9999537652	-1.0695304886
1.1142649762	0.5396595954	0.9999537652	-1.1388724317
1.4596322495	0.2606154363	0.9999537652	-1.3129548488
-1.0867987676	0.6710401397	0.9999537652	-1.0528894191
1.0675221527	0.8038050871	0.9999537652	-1.2347594907
-1.3845458221	0.0187676015	0.9999537652	-1.3395646568
1.4311344656	0.2948198056	0.9999537652	1.2660913732
-1.0514867521	0.7114250838	0.9999537652	-1.0228056829
2.7933974408	0.6948713406	0.9999537652	4.0953564288
2.0520648891	0.0005044	0.9999537652	1.1155998797
-1.0113174282	0.9470273042	0.9999537652	1.0748613072
1.0227628079	0.9517928594	0.9999537652	1.4152745727
4.2550171442	0.0859870266	0.9999537652	-1.5790138432

-1.1405421203	0.565995331	0.9999537652	-1.3460667863
11.5766527773	0.3455323591	0.9999537652	-1.1248877899
-1.255307045	0.1493450762	0.9999537652	-1.2945666078
-1.1529341132	0.2656618028	0.9999537652	-1.1723540339
2.7946088374	0.1756383905	0.9999537652	-6.3801224602
-1.1642275307	0.4107069602	0.9999537652	1.0584521356
-1.0521391104	0.8303109305	0.9999537652	1.3302431142
-1.1064269778	0.5627949537	0.9999537652	1.0110815331
-1.0085355311	0.9930349942	0.9999537652	-1.2862623659
1.029457602	0.851337824	0.9999537652	-1.0660772917
-1.071649735	0.6894258955	0.9999537652	1.2376018931
1.1420472463	0.6879630567	0.9999537652	-1.3519027108
1.0594591997	0.6424424004	0.9999537652	-1.1263534136
-1.1272314551	0.6374508254	0.9999537652	-1.2767128556
0	0	0	0
-1.2739485345	0.0721671183	0.9999537652	-1.2598417836
1.0515587552	0.6458967168	0.9999537652	-1.046876222
-1.0308932783	0.8543778769	0.9999537652	1.0457109906
1.0587348009	0.7227233673	0.9999537652	-1.2965418811
3.3058503755	0.2782186392	0.9999537652	-1.4505064176
1.9281341265	0.3667314513	0.9999537652	-1.0505510228
1.0978374414	0.6529629789	0.9999537652	-1.0044429769
-17.0527724669	0.106444846	0.9999537652	30.2246886723
-1.309833681	0.0356913756	0.9999537652	-1.0975098507
1.0038035562	0.9896145132	0.9999537652	-1.0532964828
-1.0235213271	0.8978928968	0.9999537652	1.0364307717
2.7933974408	0.6948713406	0.9999537652	-5.3583704783
0	0	0	0
2.7957660672	0.7054409062	0.9999537652	4.9527501949
4.5505976667	0.5567907887	0.9999537652	4.9527501949
2.3294952774	0.0008986	0.9999537652	1.2335327471
-1.0186316221	0.9220811648	0.9999537652	1.7422701847
-3.9838128634	0.0782031329	0.9999537652	-1.6441794863
2.8499419597	0.1545766515	0.9999537652	1.5473225268
1.2443104586	0.3574086148	0.9999537652	1.4778927474
1.3171203617	0.2812537184	0.9999537652	1.514223496
0	0	0	4.9527501949
0	0	0	2.996475627
2.7933974408	0.6948713406	0.9999537652	15.9061931258
-1.0215828419	0.8612448068	0.9999537652	-1.0640829976
-1.2652071252	0.3200745781	0.9999537652	-1.3161997844
2.7489196894	0.1407602186	0.9999537652	-1.2539651059
-1.3677102863	0.0691775063	0.9999537652	-1.0174432075
-1.1458750359	0.4833158468	0.9999537652	1.4135717602
0	0	0	0
1.0000375767	0.999903597	0.9999537652	-1.0369015529
-1.0566585585	0.8604175972	0.9999537652	1.0421433702
1.0398470077	0.8048263006	0.9999537652	-1.1242384842
-1.2504644039	0.6603823288	0.9999537652	-1.1485257488

1.111316199	0.4134558422	0.9999537652	1.2413793697
-1.0115631292	0.9482760309	0.9999537652	1.3293785687
-1.0197059834	0.9552087155	0.9999537652	-1.0711934072
0	0	0	4.9527501949
29.8698312703	0.2177565601	0.9999537652	0
2.7933974408	0.6948713406	0.9999537652	0
4.5545995984	0.5696703893	0.9999537652	0
0	0	0	2.996475627
2.7933974408	0.6948713406	0.9999537652	-2.1280158161
-1.2855530795	0.1781343428	0.9999537652	1.1047955909
-1.0185848716	0.923356326	0.9999537652	1.3012202595
1.0304768702	0.8848312396	0.9999537652	1.2979368764
-1.052528547	0.7138151094	0.9999537652	1.0126705463
1.0376118341	0.8674868797	0.9999537652	-1.0460513082
1.5673682763	0.1384135688	0.9999537652	1.2029516336
-1.1213619883	0.5950546312	0.9999537652	-1.2153482059
1.1005503536	0.6646906634	0.9999537652	1.0246983759
1.0294957231	0.8293850167	0.9999537652	1.3264387446
-1.0792592098	0.6548938358	0.9999537652	1.1601827896
1.1156288919	0.7252031668	0.9999537652	1.5809792956
9.1195404325	0.0465133478	0.9999537652	-1.3118256695
0	0	0	1.4537866576
1.0954236713	0.7783717327	0.9999537652	1.8059698302
-1.6432252404	0.1338463006	0.9999537652	1.0704358607
1.2022589705	0.6620120933	0.9999537652	1.8734568509
-1.0403556337	0.8442048573	0.9999537652	1.1344855898
1.1454289347	0.5429727711	0.9999537652	1.2297170892
4.5505976667	0.5567145673	0.9999537652	-1.3579552885
-1.1682472802	0.4615953888	0.9999537652	1.1760157523
4.5505976667	0.5566836989	0.9999537652	0
-1.1817849279	0.6099194748	0.9999537652	-1.0541182149
-1.1447852916	0.9153207121	0.9999537652	-4.0167610368
-1.0052890785	0.986167464	0.9999537652	1.3082928694
1.9183025552	0.1044001112	0.9999537652	1.0537189289
-1.0262163333	0.8205748844	0.9999537652	1.2350319696
-1.1196564798	0.5467823594	0.9999537652	-1.0930472912
11.5864583426	0.3448609145	0.9999537652	-1.5255688471
-1.0148144681	0.9169069979	0.9999537652	1.1200680908
1.1855043684	0.2293123565	0.9999537652	1.0304883745
-4.3234865661	0.150015522	0.9999537652	12.7732027325
1.11360368	0.5694735292	0.9999537652	1.1669498724
0	0	0	6.9133480437
2.7933974408	0.6948713406	0.9999537652	-6.4352633827
4.7592049098	0.1553546697	0.9999537652	-1.166228783
1.3835687086	0.0787709866	0.9999537652	-1.0247952108
1.0371707186	0.8658934414	0.9999537652	1.1386081413
1.6141448781	0.1595822383	0.9999537652	1.084872138
1.0897630839	0.6412362873	0.9999537652	-1.0928615833
1.0579973875	0.7798809306	0.9999537652	-1.0262823766

-2.4710767924	0.5445220763	0.9999537652	0
-1.3150087148	0.3961402037	0.9999537652	1.045554902
1.3025325442	0.6131002717	0.9999537652	1.1353089796
1.194738735	0.5850453649	0.9999537652	1.0205162228
24.3870295947	0.2290228206	0.9999537652	-2.2935209839
-2.0942274998	0.046788865	0.9999537652	-1.8811465506
1.0145668119	0.9661089919	0.9999537652	1.3107735704
54.5793006814	0.1214851	0.9999537652	2.996475627
1.8957557082	0.4627928026	0.9999537652	-1.1611062701
1.1302840871	0.5038563542	0.9999537652	1.1030282654
-1.0872029594	0.5090928015	0.9999537652	1.0471350312
-2.6368360135	0.2392545506	0.9999537652	2.3077201453
1.103931787	0.6641471502	0.9999537652	1.1324635149
0	0	0	2.9985540909
2.9591626092	0.0108162484	0.9999537652	1.569417442
-1.1670613769	0.8550003358	0.9999537652	-4.7399721888
1.0280366057	0.9066135725	0.9999537652	1.3443231479
1.4706009574	0.2037058681	0.9999537652	1.1309302511
1.1901283357	0.4452666418	0.9999537652	1.355174895
1.4529708599	0.2207114612	0.9999537652	1.2560288234
-1.2733479599	0.3646705959	0.9999537652	1.1268172095
2.018188514	0.0372783068	0.9999537652	1.358659433
1.4208057684	0.0528385048	0.9999537652	1.235364176
6.3075231393	0.4863782724	0.9999537652	2.996475627
-1.034418822	0.8495020495	0.9999537652	-1.0781829996
-1.0815397743	0.7130259269	0.9999537652	-1.0576180928
2.1947124478	0.3162270937	0.9999537652	-1.0193908853
-1.1323086324	0.5141112952	0.9999537652	-1.0590834607
-1.2399604201	0.2380271471	0.9999537652	-1.0593257701
-1.2407663199	0.490270913	0.9999537652	1.3112449963
-1.0579755669	0.9334067448	0.9999537652	-1.5830173904
1.1244691651	0.579623143	0.9999537652	-1.2927168785
2.7933974408	0.6948713406	0.9999537652	0
1.2802584115	0.4810185133	0.9999537652	2.1539920158
1.1621627331	0.6143733589	0.9999537652	1.3155744671
-1.6575837066	0.4541293519	0.9999537652	1.2008979237
1.0186672449	0.9423122656	0.9999537652	1.0746233485
2.4277111557	0.2920079741	0.9999537652	2.9707177548
-1.0993267572	0.7808434325	0.9999537652	1.4401814165
1.6314647197	0.2169904676	0.9999537652	1.0260604864
1.5965657362	0.1136941818	0.9999537652	2.2018816799
1.3225465194	0.066854768	0.9999537652	1.1902884253
1.3387611711	0.4715960368	0.9999537652	-1.0084351443
-2.0908000446	0.4356910554	0.9999537652	-1.3783411646
-1.2162857015	0.1785273158	0.9999537652	1.101900263
2.7957660672	0.7054432521	0.9999537652	-3.3853891979
1.1175388732	0.6564915258	0.9999537652	1.0433462964
-2.8080850154	0.3318381258	0.9999537652	-5.5994415605
4.5505976667	0.5566538964	0.9999537652	10.8262784008

1.081436306	0.8487979596	0.9999537652	1.0096506788
2.7933974408	0.6948713406	0.9999537652	-1.1250196696
-2.5838710423	0.5187542002	0.9999537652	2.9985540909
-1.1680276447	0.3902595839	0.9999537652	1.018006364
-1.6422116369	0.4178088296	0.9999537652	-1.4035964637
-1.2391589495	0.3556718585	0.9999537652	1.5688532466
1.4725798242	0.0798828039	0.9999537652	1.0119272205
-1.0457369147	0.8574225033	0.9999537652	-1.0987906769
1.3003719044	0.4421645438	0.9999537652	1.0092712287
-1.4686464794	0.3913540107	0.9999537652	1.3753245199
-1.0433958077	0.8343560122	0.9999537652	-1.1488420713
-1.3922070018	0.4422614556	0.9999537652	1.6706106163
1.2319435881	0.3469881434	0.9999537652	-1.4656942709
-1.4137177797	0.398621742	0.9999537652	-1.027479335
6.3075231393	0.4863597406	0.9999537652	1.4537866576
-1.0758544827	0.6550740464	0.9999537652	-1.0418103549
1.0418460307	0.8641500961	0.9999537652	1.5841593768
1.0228776005	0.9000546292	0.9999537652	1.4612263206
-1.1097311508	0.8347230881	0.9999537652	-1.0466177644
1.202091047	0.3296256592	0.9999537652	1.059622733
-1.0250895366	0.9297349637	0.9999537652	-1.155975356
-1.5347269239	0.2658884195	0.9999537652	1.5107602448
2.6134824879	0.0171696081	0.9999537652	1.2938186044
3.859400354	0.1173269063	0.9999537652	-1.1280370244
-1.4762397703	0.6421141941	0.9999537652	16.6806485101
1.1552692485	0.3785925423	0.9999537652	1.0280880059
1.0844487454	0.8707989975	0.9999537652	-1.1662003378
-1.1994367768	0.6709214158	0.9999537652	1.321803727
-1.4263849482	0.1003895749	0.9999537652	-1.193849186
-1.8172508579	0.3615251308	0.9999537652	2.1851140688
1.0074744043	0.9912150162	0.9999537652	1.0386658977
2.0852494071	0.203685478	0.9999537652	1.0712963309
1.0473973226	0.8358952645	0.9999537652	1.3456319557
1.1307159098	0.6319491641	0.9999537652	1.0566125318
-5.855397775	0.1779668514	0.9999537652	6.908559683
1.4273126267	0.4333625821	0.9999537652	2.1612032567
-1.0492162905	0.8654427389	0.9999537652	-1.169586205
1.271436232	0.2602352117	0.9999537652	1.1475277752
1.0570403596	0.7140650462	0.9999537652	1.2503054055
-1.0647557993	0.6901321504	0.9999537652	1.131988253
1.2674088665	0.159934602	0.9999537652	1.2721374381
1.3640422474	0.3026206717	0.9999537652	1.3683146205
-3.2727308847	0.1982147524	0.9999537652	-1.4602971429
1.1156982492	0.7238181458	0.9999537652	1.2854220982
1.7114087897	0.0696450289	0.9999537652	-1.0364925567
-1.9041828432	0.1375057486	0.9999537652	-1.2468680136
1.3450284088	0.4646567808	0.9999537652	1.1560248777
2.9792351324	0.0743767023	0.9999537652	1.0300568638
1.0155282432	0.9077373176	0.9999537652	1.4089260639

-1.212233978	0.3270041685	0.9999537652	1.2680305681
-1.1453192235	0.908736665	0.9999537652	1.0674788249
1.0129420433	0.9674476493	0.9999537652	-1.362787283
0	0	0	2.9988886918
1.05232932	0.9175943991	0.9999537652	1.302851463
-1.6413044039	0.0385866709	0.9999537652	1.0441388886
-1.2299936391	0.3123070881	0.9999537652	1.0510593525
0	0	0	2.996475627
-1.1254649332	0.4924379603	0.9999537652	1.1171769112
1.846408361	0.5882299327	0.9999537652	2.9985540909
1.5051828897	0.4676346508	0.9999537652	1.4940516767
1.0385208736	0.9226902176	0.9999537652	1.2176542164
18.5983228161	0.2579759179	0.9999537652	-1.0469500538
1.2020388661	0.651531815	0.9999537652	-1.3763386703
-1.0833740364	0.6913476011	0.9999537652	-1.1335219385
1.0632335311	0.6713514206	0.9999537652	1.4066496785
1.7938786006	0.007712	0.9999537652	-1.7181032845
-1.2734834919	0.5805362738	0.9999537652	-1.3501592245
0	0	0	2.996475627
-1.4645225255	0.2887272322	0.9999537652	2.3222187522
1.3386362079	0.1616077745	0.9999537652	1.8358521658
-1.0877948079	0.8945772997	0.9999537652	-1.1093538102
0	0	0	2.9985540909
-1.0996825855	0.7348599497	0.9999537652	-1.0838017905
1.120080119	0.6034885487	0.9999537652	1.5156845621
-1.5875118025	0.7205195663	0.9999537652	2.3413262342
6.3075231393	0.4863412791	0.9999537652	0
-1.1309816678	0.8547260639	0.9999537652	-1.1544184423
-1.171252603	0.8507580726	0.9999537652	1.0682347371
2.7957660672	0.7054455989	0.9999537652	-1.1242401582
8.064174035	0.4238837802	0.9999537652	-1.8594983184
-6.9764533263	0.2592026819	0.9999537652	20.6004768562
1.0555020791	0.7096491587	0.9999537652	1.3446520506
-1.1348674605	0.6200995173	0.9999537652	-1.0655129099
1.4757537288	0.1303870459	0.9999537652	1.3839536241
2.0224217452	0.0542198543	0.9999537652	1.9047163923
1.0304230289	0.8147039122	0.9999537652	1.1782068679
4.5505976667	0.5566250533	0.9999537652	8.8700459102
1.1593773706	0.5267973225	0.9999537652	1.107856765
-1.1761541094	0.5060071174	0.9999537652	1.135571024
3.7902526731	0.2288683402	0.9999537652	1.2482398711
-1.6973123489	0.5505981895	0.9999537652	4.1864221648
0	0	0	2.9985540909
-1.854982839	0.669500012	0.9999537652	4.2599323069
-1.0382088849	0.8629596625	0.9999537652	-1.1261879802
-1.3784666702	0.5533441515	0.9999537652	-1.0624205355
4.5505976667	0.5565970628	0.9999537652	0
1.3588576256	0.8007235826	0.9999537652	0
-3.3110661094	0.2931306503	0.9999537652	5.9722067269

1.0797125592	0.8027923246	0.9999537652	1.5342866731
6.3075231393	0.4863228875	0.9999537652	1.2394611522
0	0	0	2.9985540909
4.5505976667	0.5565698204	0.9999537652	2.2253832076
1.1058644643	0.7071044163	0.9999537652	1.0448231237
0	0	0	2.996475627
2.7933974408	0.6948713406	0.9999537652	6.908559683
1.0613198825	0.6001880255	0.9999537652	1.0654411492
0	0	0	1.3166104106
-1.669821626	0.1178436899	0.9999537652	1.6154009151
-1.8455979539	0.3270696906	0.9999537652	1.087618212
-1.0210365629	0.963173373	0.9999537652	-1.0445158578
-2.5861438062	0.4665546927	0.9999537652	2.387778404
1.1156751249	0.5148974932	0.9999537652	1.4169368365
-1.1509029955	0.3076581429	0.9999537652	-1.2838067469
-1.5564897538	0.1292289349	0.9999537652	-1.0664889667
-2.587194901	0.5007506416	0.9999537652	1.2593062028
1.0246710853	0.9222381925	0.9999537652	1.452217877
-1.0573992956	0.6743936028	0.9999537652	1.118998532
-1.0949486505	0.781349341	0.9999537652	1.0367247291
-1.1581634302	0.4101430556	0.9999537652	-1.0147651787
1.3999031749	0.4019305723	0.9999537652	1.6450014292
1.0892911075	0.7405101077	0.9999537652	1.6072123027
-1.0104114485	0.9763773132	0.9999537652	1.5692729976
1.071066925	0.9164818364	0.9999537652	1.6860091478
1.3408749081	0.0381423508	0.9999537652	1.1739008283
-1.4571852392	0.3087031755	0.9999537652	1.0291548006
-1.1619421561	0.8737038912	0.9999537652	-2.044605925
1.6778054259	0.0179542736	0.9999537652	-1.0827464978
2.3306483975	0.4665651467	0.9999537652	-2.0901136056
1.1813435872	0.4619124265	0.9999537652	1.0676588949
-1.1292995288	0.3564097569	0.9999537652	1.2222969848
1.0898301836	0.6840130732	0.9999537652	1.1327328324
34.3811030879	0.1695431793	0.9999537652	2.0643077124
-1.0177924513	0.9263518423	0.9999537652	1.4387781797
0	0	0	-6.6628455824
-1.1345373546	0.4669317304	0.9999537652	1.1411328699
-1.2892908447	0.2419779638	0.9999537652	-1.1654630419
1.1008317228	0.5728724564	0.9999537652	1.0180949097
-1.3869431146	0.1763327198	0.9999537652	-1.275711308
-1.0007130659	0.9971624001	0.9999537652	1.6269911157
1.0194266775	0.9297249356	0.9999537652	1.014555652
-1.8199241288	0.0434154809	0.9999537652	-1.0808400157
-1.2189276647	0.3953523009	0.9999537652	1.1083496351
-1.027205394	0.9267001476	0.9999537652	1.1835915972
1.1919268115	0.2016712203	0.9999537652	-1.1354744873
-1.4940676537	0.3401568431	0.9999537652	-1.0744014923
1.1925582499	0.4779455974	0.9999537652	-1.0111001095
-1.9736273861	0.2288237199	0.9999537652	1.5462826785

1.2356860561	0.5617708085	0.9999537652	-1.1925159819
-1.5498131543	0.1001118269	0.9999537652	1.1434776067
2.7933974408	0.6948713406	0.9999537652	0
1.0880934088	0.6332809855	0.9999537652	1.0755730762
1.334054837	0.5906041102	0.9999537652	1.8861877352
-1.0645813667	0.6873220823	0.9999537652	-1.0197153102
-1.8277288365	0.1747969359	0.9999537652	1.3830625852
-2.1053066666	0.3146010489	0.9999537652	1.124343984
1.2707804646	0.3225906659	0.9999537652	1.2280067376
2.1141085505	0.1259521107	0.9999537652	-1.0301015622
-1.0135297804	0.954795723	0.9999537652	-1.0140608132
-2.0649459568	0.1648036282	0.9999537652	-1.2475746038
-1.094053711	0.6240083282	0.9999537652	-1.0744302441
-1.019770079	0.9419569073	0.9999537652	1.3352977848
1.0096552952	0.9592991208	0.9999537652	-1.0603835776
-1.0603878335	0.6512323981	0.9999537652	1.0689983623
1.0941338387	0.7012486721	0.9999537652	1.3292208293
-1.1453192235	0.9087196086	0.9999537652	-6.4352633827
1.643510367	0.5933618518	0.9999537652	12.7732027325
25.6156185703	0.2096653013	0.9999537652	2.1590507919
0	0	0	1.6470100299
0	0	0	0
-1.2171598465	0.387757276	0.9999537652	-1.2406306012
1.6270711323	0.5959701078	0.9999537652	-1.2180148517
-1.0748031098	0.6534606546	0.9999537652	1.0560712212
4.5544554512	0.5691473461	0.9999537652	-1.1250196696
1.1136602091	0.7303514282	0.9999537652	-1.7477970174
1.0833043499	0.7895153765	0.9999537652	1.5828434909
-1.0188106624	0.9445074881	0.9999537652	1.3209354107
-2.3601138181	0.4328865573	0.9999537652	0
8.0710074692	0.4203286965	0.9999537652	1.3973764147
0	0	0	2.9985540909
-1.0878470573	0.6684904877	0.9999537652	1.3506488583
-1.5876922998	0.0262284393	0.9999537652	-1.0774886362
2.7933974408	0.6948713406	0.9999537652	-2.9704130837
-1.2374416969	0.3777445303	0.9999537652	1.2197661357
-2.1805032503	0.047195031	0.9999537652	1.5847701151
-1.213640762	0.3241754139	0.9999537652	-1.0633959938
1.4185591585	0.1484039155	0.9999537652	-1.0718646352
0	0	0	1.0015079005
1.2103350963	0.3581476865	0.9999537652	1.0935701428
-1.0290766315	0.9193074995	0.9999537652	1.1786408586
-1.2068050655	0.2668208025	0.9999537652	-1.5304899078
1.1893568102	0.7037017453	0.9999537652	1.2340294199
1.0175625897	0.9309805471	0.9999537652	1.0398389702
1.0676227971	0.8827466108	0.9999537652	-1.2310789416
-1.2586068636	0.258780112	0.9999537652	-1.0152481348
-1.2540680512	0.3219385889	0.9999537652	1.0071704479
1.2160018552	0.4044533403	0.9999537652	1.3676474441

1.2642169253	0.1829870753	0.9999537652	-1.0898259795
2.0849718726	0.2968429513	0.9999537652	-1.3034241767
-1.0048342918	0.9754252246	0.9999537652	-1.2103983862
-2.0008239305	0.4558475686	0.9999537652	3.3511460746
-1.5726195369	0.1944030852	0.9999537652	1.3602978882
1.2563615157	0.2248972152	0.9999537652	1.3007454686
1.2001438777	0.1954563871	0.9999537652	1.0845293682
-1.032472774	0.8117670889	0.9999537652	-1.2110270751
-1.0525647226	0.8710276927	0.9999537652	-1.2986493178
1.0015906331	0.9892091757	0.9999537652	1.0887731188
1.2598784428	0.1315734194	0.9999537652	1.2119768401
-1.7000974549	0.5642294185	0.9999537652	2.3312237541
1.0275650038	0.8983695841	0.9999537652	1.0112621194
3.3030568913	0.2924636484	0.9999537652	-2.1744673066
4.5505976667	0.5565432176	0.9999537652	2.996475627
1.0929659304	0.6845021347	0.9999537652	-1.3948548231
1.1343245663	0.5981105235	0.9999537652	1.1199751632
-1.4750790135	0.6599060967	0.9999537652	-1.3945395231
-1.5881570201	0.7139018484	0.9999537652	1.9418822056
-1.0728948619	0.6417138291	0.9999537652	1.081137687
2.7957660672	0.7054479467	0.9999537652	-5.3589986948
-1.1448144121	0.9147429026	0.9999537652	39.208884251
2.7957660672	0.7054502954	0.9999537652	0
-1.203782886	0.2093480875	0.9999537652	1.1240571972
1.1674063569	0.4173859578	0.9999537652	1.5939666485
1.2867504688	0.4415127097	0.9999537652	-1.2729731543
1.0766311068	0.5998913689	0.9999537652	1.1442314663
1.0312883198	0.9260052049	0.9999537652	1.0405263134
0	0	0	2.996475627
34.4112440562	0.1677827362	0.9999537652	1.0206439538
1.0543870454	0.8777768431	0.9999537652	-1.3927131448
1.1731864831	0.4355602875	0.9999537652	1.5080072099
-1.0535398293	0.8201277133	0.9999537652	-1.0815428652
1.1286582069	0.8387514599	0.9999537652	-1.4857481814
-1.4090963591	0.2149387121	0.9999537652	1.5182472669
-1.1704736049	0.7124119409	0.9999537652	1.2233825687
1.0228019496	0.9241732804	0.9999537652	1.0197650366
-1.4753878392	0.004148	0.9999537652	-1.0778831524
-1.0730247126	0.8030696181	0.9999537652	1.3896895945
-1.1064940004	0.5009196079	0.9999537652	1.311422293
0	0	0	1.2601712954
-2.587194901	0.5007412281	0.9999537652	-2.7908644137
1.4648506267	0.0768711658	0.9999537652	1.1290124242
-1.6481429995	0.3099147217	0.9999537652	-1.1420150361
-1.1654633928	0.484681861	0.9999537652	1.0196647181
1.0203077888	0.9132529633	0.9999537652	1.0589277392
-1.5579775407	0.055264417	0.9999537652	1.3555671242
1.0844515221	0.6498784618	0.9999537652	-1.0500287798
1.1225079038	0.5377579969	0.9999537652	-1.4708376147

-1.179607106	0.3615426294	0.9999537652	-1.1313166983
-1.0753295372	0.6793817299	0.9999537652	-1.0225487295
-1.2528462971	0.5855036262	0.9999537652	-1.028198607
-5.692811393	0.0769573318	0.9999537652	-2.1754645223
1.3708361697	0.3174635236	0.9999537652	-1.1446198957
3.3046967148	0.286112132	0.9999537652	1.9366867128
4.3339220787	0.0345006863	0.9999537652	-1.8825220281
-1.3849169173	0.4227693234	0.9999537652	-1.270907783
1.0193491865	0.9480614523	0.9999537652	-1.6291921415
-1.0478190319	0.728785053	0.9999537652	-1.3145183095
1.5246050946	0.1820668124	0.9999537652	1.1566053577
1.0877270743	0.5485091464	0.9999537652	-1.1606695365
1.0065937959	0.98287441	0.9999537652	1.1123850928
1.0882764953	0.6039652676	0.9999537652	-1.2451937783
-1.1077631395	0.5427945262	0.9999537652	-1.394327004
-1.1728665519	0.288987112	0.9999537652	-1.1780960275
1.5139120855	0.1227803056	0.9999537652	-1.253593396
1.0394465243	0.9000030707	0.9999537652	1.3013077265
1.1433277037	0.5408728708	0.9999537652	1.0878402497
1.2644283007	0.4797334785	0.9999537652	-1.2651508969
1.0617714076	0.7650167901	0.9999537652	-1.1068137173
-1.199620006	0.5663163458	0.9999537652	1.2708100605
-1.1266305634	0.4092020898	0.9999537652	-1.0362496051
-1.0729553632	0.7472553945	0.9999537652	-1.0844668862
3.8622037496	0.6198017915	0.9999537652	0
1.0016714953	0.9929517886	0.9999537652	-1.0407167934
1.1982230535	0.261515817	0.9999537652	-1.3259104505
1.6758188804	0.349901153	0.9999537652	1.1008237951
-1.4885847327	0.2426288145	0.9999537652	6.763601952
55.3905770121	0.1166692559	0.9999537652	-2.4419375169
-1.3724562175	0.091724715	0.9999537652	-1.5847649862
2.3335098933	0.4432059046	0.9999537652	-1.8812654885
1.0981607799	0.5502859248	0.9999537652	-1.1719801697
1.1301002015	0.6527775333	0.9999537652	-1.4775736169
1.0606233766	0.6046784824	0.9999537652	1.018277346
74.4187556167	0.101011951	0.9999537652	3.9786486669
-1.1974130486	0.6991505805	0.9999537652	-1.1843678061
-1.1676089869	0.3780243873	0.9999537652	-1.0200488649
1.8816453506	0.5620798186	0.9999537652	-3.384595918
1.0898155678	0.5884080913	0.9999537652	1.4212368995
-1.0846110904	0.7899066679	0.9999537652	-1.1218684868
1.5578739567	0.1936445217	0.9999537652	-1.2752320921
-1.0693354128	0.6483007491	0.9999537652	-1.0564174525
-1.1088272453	0.6603549313	0.9999537652	-1.3029571861
1.1484131946	0.5783625936	0.9999537652	-1.1167341643
1.0028996321	0.9886672405	0.9999537652	1.2822660794
-1.0854147188	0.4976614732	0.9999537652	-1.1353172254
-1.283806923	0.294694549	0.9999537652	1.0839349136
1.037224176	0.7982783218	0.9999537652	1.0345324313

-1.5881570201	0.7136875907	0.9999537652	1.6458706287
1.2123375468	0.5161955262	0.9999537652	-1.4082057682
-1.3734734207	0.138372106	0.9999537652	-1.3353081179
-6.9736190158	0.2250321147	0.9999537652	1.2152209729
-1.3067013344	0.0829150461	0.9999537652	-1.6614124678
-1.0122485522	0.9507474156	0.9999537652	-1.0664409132
0	0	0	8.8639046058
-1.1279291834	0.476394892	0.9999537652	1.3744633733
1.3645322244	0.1640759156	0.9999537652	1.3057408509
1.1605171257	0.5577796385	0.9999537652	1.1793720802
1.0388568099	0.7899181207	0.9999537652	1.2140419356
1.2203853078	0.4626421935	0.9999537652	1.0651595895
-1.096520927	0.6501523335	0.9999537652	1.0757465337
-1.0703225841	0.8739244647	0.9999537652	-1.1105104235
2.7957660672	0.705452645	0.9999537652	9.7116702472
-1.0350587619	0.7930749776	0.9999537652	1.1901910042
1.0645242292	0.6608678193	0.9999537652	1.2170316649
1.8816453506	0.5619963272	0.9999537652	-2.9683549234
1.8198190335	0.1794672747	0.9999537652	1.3469801082
-1.4427560727	0.0407454827	0.9999537652	-1.0894493391
0	0	0	1.2587294573
1.0551495721	0.8332035447	0.9999537652	1.0396465114
1.0561276962	0.7636455676	0.9999537652	1.1490189803
0	0	0	1.2408886006
-1.1859130246	0.5676390436	0.9999537652	1.7707295391
-1.0998722319	0.7800692623	0.9999537652	-1.0254122857
4.5505976667	0.5565171389	0.9999537652	2.6083962368
1.0969812451	0.5380722762	0.9999537652	1.318832555
-1.6975651946	0.2544664462	0.9999537652	1.2680438217
-1.3354993644	0.493516444	0.9999537652	1.0783678942
1.7848781972	0.2092172409	0.9999537652	-1.2438746902
-1.1971456387	0.2135096655	0.9999537652	-1.2104349233
0	0	0	2.996475627
-1.1418375965	0.29140335	0.9999537652	-1.1158265266
-1.3548635538	0.1153136988	0.9999537652	1.2411446349
-1.1222860487	0.6667349904	0.9999537652	1.151303861
1.1861556027	0.4387432074	0.9999537652	1.3314618789
1.5889344781	0.007328	0.9999537652	1.1641477471
1.1392832044	0.4030831611	0.9999537652	1.0791187773
1.0387730132	0.8532050008	0.9999537652	-1.0340036787
-1.0411822654	0.769797375	0.9999537652	1.1388171606
-1.2352651412	0.5884145319	0.9999537652	-1.0148517306
0	0	0	4.9567375629
0	0	0	2.996475627
-2.5871290907	0.4971685309	0.9999537652	2.9988886918
-1.022950828	0.9568648422	0.9999537652	1.3610449496
4.5505976667	0.5564914553	0.9999537652	2.9957580451
1.0196170288	0.9331251824	0.9999537652	-1.0570531772
-1.0132488865	0.9139421382	0.9999537652	1.6039616802

5.5633222739	0.0326492049	0.9999537652	-1.448424821
-1.1240657836	0.8422577978	0.9999537652	-1.1113313989
1.1465573442	0.6607578092	0.9999537652	1.2877910214
-1.09011441	0.5423613771	0.9999537652	1.3622659139
-1.0245189834	0.8386898558	0.9999537652	1.0613079565
1.2793077919	0.0995763078	0.9999537652	1.1082217927
17.3472938331	0.2975374064	0.9999537652	-5.3583704783
-1.0002863038	0.9986568707	0.9999537652	1.20634567
1.0834633305	0.8769565316	0.9999537652	-1.6327386627
2.7933974408	0.6948713406	0.9999537652	5.4461692489
3.909066224	0.0443781561	0.9999537652	1.0713899155
3.3017989522	0.3007889978	0.9999537652	-1.0884300027
-1.0505456648	0.9100035885	0.9999537652	-1.1452805869
0	0	0	2.9985540909
1.6416093931	0.295188787	0.9999537652	-1.594099409
1.079659181	0.6757728699	0.9999537652	-1.0057445251
-1.1314343639	0.3971569952	0.9999537652	1.1203584451
1.3477925422	0.0874824933	0.9999537652	1.1698066237
-1.5872046595	0.2188016656	0.9999537652	1.0654521669
0	0	0	-1.5077410611
-1.0334204232	0.9072798662	0.9999537652	1.7675108519
1.0854333409	0.5216915842	0.9999537652	1.0995806642
-1.2903209516	0.2347076566	0.9999537652	-1.065971545
1.3588576256	0.800792063	0.9999537652	2.2955915087
-1.1587451003	0.5224638276	0.9999537652	1.2070573104
1.1102687236	0.7943688868	0.9999537652	-1.1201968599
8.064174035	0.4238824465	0.9999537652	-1.1164993979
0	0	0	1.7042814026
0	0	0	1.6293799659
1.056991956	0.9097113347	0.9999537652	1.1904503086
-1.6405494181	0.2724949146	0.9999537652	-1.0825975059
1.0339902911	0.8531700152	0.9999537652	1.1103543664
1.1232098075	0.6453651812	0.9999537652	1.1815223025
1.2695653613	0.4164423688	0.9999537652	-1.0737322285
-12.9800088947	0.1123076381	0.9999537652	-4.711036837
-1.1718672023	0.2647642012	0.9999537652	1.2157852356
-1.0028037445	0.9841422526	0.9999537652	1.0631525517
0	0	0	4.9527501949
-2.4710767924	0.5445182174	0.9999537652	-5.3583704783
1.0190498317	0.8861396084	0.9999537652	1.148231535
-1.2429119773	0.202055106	0.9999537652	-1.0223254671
-1.5161100861	0.0710077428	0.9999537652	-1.230765576
-2.6366769527	0.252572612	0.9999537652	1.0674788249
-1.1133089272	0.5476939577	0.9999537652	1.1258672774
1.4313972465	0.1423857367	0.9999537652	-1.0719928885
1.175793432	0.7443422654	0.9999537652	-1.0520061228
1.1910830518	0.3691458832	0.9999537652	1.0390730893
0	0	0	-5.3583704783
1.6496991901	0.0408504948	0.9999537652	1.6193994924

-1.0591807507	0.6480967425	0.9999537652	1.0710890116
1.2160198636	0.3337278084	0.9999537652	-1.1820115946
-1.0192337537	0.9832892044	0.9999537652	1.0004347069
-1.2713391982	0.4422648508	0.9999537652	1.1906441422
1.2142668303	0.6267114526	0.9999537652	-1.0145941688
-1.3162509082	0.2139251457	0.9999537652	-1.1254134366
-1.1851309897	0.658443918	0.9999537652	1.1308273013
-2.4376693931	0.323587534	0.9999537652	-4.8159020612
9.8288704632	0.3801545722	0.9999537652	4.9527501949
-1.2143331251	0.2497001133	0.9999537652	-1.0287425445
-1.096301567	0.6201226385	0.9999537652	-1.0920498285
-1.5679960926	0.1121056864	0.9999537652	1.06381961
-1.5301288162	0.5118136775	0.9999537652	-1.6786831267
1.3006914835	0.3423029556	0.9999537652	-1.2693689896
2.7933974408	0.6948713406	0.9999537652	-1.1241083699
-1.0115392237	0.9675010015	0.9999537652	1.1345066285
-1.0512864146	0.7782188972	0.9999537652	-1.100838278
-1.1188424386	0.6179152342	0.9999537652	-1.0550419797
-1.0186938213	0.9033218228	0.9999537652	1.3095173403
-1.068668045	0.8828973018	0.9999537652	1.3543104828
5.2576341863	0.0005838	0.9999537652	2.5976142664
-1.1457555741	0.9107282109	0.9999537652	10.8274885115
-1.1100034067	0.7940801804	0.9999537652	1.9637256609
-1.018666339	0.8863356846	0.9999537652	1.2502871892
1.0753946936	0.7034258603	0.9999537652	1.5642467132
11.5766527773	0.3455232366	0.9999537652	-1.7821607506
1.1310215839	0.6841290759	0.9999537652	-1.0959998497
-1.0368041366	0.8040193041	0.9999537652	1.1629226657
1.2140337697	0.311182045	0.9999537652	1.1147215251
-1.1736759301	0.2340185621	0.9999537652	-1.1939652751
3.0266471183	0.2195043824	0.9999537652	-1.491016894
-1.1497558295	0.6556228587	0.9999537652	-1.5080084849
-8.4262209803	0.0176813917	0.9999537652	-1.1555570895
-1.227781611	0.5052396273	0.9999537652	1.304390149
4.0330685933	0.2055801456	0.9999537652	1.227177999
-1.3155434431	0.2280107278	0.9999537652	1.0460298302
-1.5875118025	0.7203408911	0.9999537652	-1.0179604803
-1.1441752206	0.7518955569	0.9999537652	-1.3235543942
-1.0643374939	0.6797563259	0.9999537652	-1.2575597267
3.8622037496	0.6197712208	0.9999537652	0
1.2584736431	0.1498803748	0.9999537652	1.1522446748
-1.0904723968	0.4734250269	0.9999537652	1.205086186
0	0	0	2.996475627
-1.0424897253	0.8314324814	0.9999537652	1.1261918462
-1.0626556027	0.7275872025	0.9999537652	-1.067671253
-1.3062073106	0.2022991167	0.9999537652	-1.3480922959
-1.2984065896	0.2539048728	0.9999537652	-1.5584173753
1.1123634008	0.3459287975	0.9999537652	-1.0900407533
1.15516252	0.2592501606	0.9999537652	1.0870052084

-1.1378737193	0.5056482981	0.9999537652	1.0009451735
1.1036508172	0.7181471271	0.9999537652	-1.3286757515
1.2043349655	0.3048964761	0.9999537652	-1.0834119567
2.7933974408	0.6948713406	0.9999537652	0
-1.183701252	0.448589428	0.9999537652	-1.3721612747
-1.2431207827	0.4936304287	0.9999537652	1.5300205048
1.0242029872	0.9125172391	0.9999537652	-1.0986698231
-1.1932732931	0.726725701	0.9999537652	1.1165002124
1.0311996087	0.8994213472	0.9999537652	1.3283916125
16.8575730017	0.2721091126	0.9999537652	-1.0506331478
1.2061829176	0.5081781343	0.9999537652	1.0614006349
-2.4358985765	0.0649923735	0.9999537652	1.5193243617
-1.0418968141	0.7622517752	0.9999537652	-1.1034680751
1.0528083966	0.792875035	0.9999537652	-1.0528685767
8.064174035	0.4238811127	0.9999537652	-2.9704130837
-1.0386108215	0.8058503938	0.9999537652	-1.0134400192
6.9558210435	0.0673521558	0.9999537652	-1.1511018589
-1.177447641	0.330929195	0.9999537652	1.0757654896
-1.0819620975	0.8063563272	0.9999537652	-1.3202915744
-1.0122036701	0.9577190665	0.9999537652	-1.0437846675
3.8622037496	0.6197406321	0.9999537652	0
2.7933974408	0.6948713406	0.9999537652	6.908559683
-1.9975604569	0.2291259617	0.9999537652	1.1509395518
1.0836803219	0.6764368243	0.9999537652	1.4906387176
-1.0851416252	0.5941926214	0.9999537652	1.0313984331
-1.3515355345	0.1264184095	0.9999537652	1.4301329544
1.0150293136	0.9069332185	0.9999537652	1.2288493997
-1.0798002724	0.8319227147	0.9999537652	-1.0208195549
1.1053671313	0.5642732598	0.9999537652	-1.0930555456
1.0896463341	0.5676539534	0.9999537652	-1.1273394934
0	0	0	4.9527501949
0	0	0	2.9988886918
11.5766527773	0.3455141227	0.9999537652	2.3505428635
2.7933974408	0.6948713406	0.9999537652	0
0	0	0	4.9527501949
0	0	0	2.996475627
-1.3015602086	0.3499684268	0.9999537652	-1.0535739124
1.3011205276	0.3788507886	0.9999537652	-1.0134862908
1.1260948192	0.7265886182	0.9999537652	1.1821192204
1.7000556252	0.1610844117	0.9999537652	1.0167574856
8.064174035	0.4238797786	0.9999537652	1.2701719397
-1.5770193234	0.5220861338	0.9999537652	1.2582332362
1.8803596835	0.0410285348	0.9999537652	1.4944551242
1.2196511139	0.6729192742	0.9999537652	1.4575709774
-1.1243032004	0.4592279177	0.9999537652	-1.0325860981
1.304143254	0.0551809802	0.9999537652	1.0520267141
-1.124626603	0.6094313749	0.9999537652	1.2212572633
1.0997068575	0.6307950294	0.9999537652	-1.1065497994
1.4033096916	0.4546085013	0.9999537652	-1.2457309108

1.1579584104	0.4542404874	0.9999537652	-1.2846706518
-1.0738293836	0.6928813637	0.9999537652	-1.1209634421
-1.1653805024	0.3369306773	0.9999537652	-1.1289732603
-1.1426973033	0.6390844966	0.9999537652	2.09026542
-1.2768053901	0.1885157817	0.9999537652	1.0911970977
1.8447991669	0.5995956551	0.9999537652	1.2603257066
-1.2721835448	0.7612041976	0.9999537652	-1.1429632564
1.4230954532	0.4399067959	0.9999537652	-1.4471458091
-1.008919192	0.9465163914	0.9999537652	1.1420669117
-1.1707332864	0.2503597835	0.9999537652	-1.1954364983
-1.0395607898	0.9548034713	0.9999537652	-14.6584086407
1.0511705165	0.7160685638	0.9999537652	-1.061530527
0	0	0	9.7116702472
-1.3917747917	0.6970232734	0.9999537652	1.2400291296
1.008701994	0.951683209	0.9999537652	1.1064822986
1.4599072921	0.1104263299	0.9999537652	-1.0681921999
1.1749168429	0.4720964737	0.9999537652	1.1937907387
1.5156048348	0.6493157554	0.9999537652	2.0065549998
1.6823381434	0.27876534	0.9999537652	-1.7201879373
-1.0659956562	0.6591275295	0.9999537652	1.030361141
1.8471704681	0.5865330365	0.9999537652	-1.4289359629
-1.1762843017	0.6812149389	0.9999537652	1.0010073177
-1.1453192235	0.908702506	0.9999537652	-1.3890024963
0	0	0	2.0802791138
1.1314234599	0.5689214246	0.9999537652	-1.6425175533
1.0199039242	0.8731982539	0.9999537652	1.2894149211
1.1288210825	0.3472512723	0.9999537652	-1.1173975184
-1.1683691627	0.3134341259	0.9999537652	1.3600498468
-2.1259516533	0.2002071903	0.9999537652	-1.4357827083
-1.0889712648	0.50662197	0.9999537652	-1.0224559251
1.0904713562	0.6908440813	0.9999537652	1.2646493422
-1.1771668443	0.5939389089	0.9999537652	-1.6038905579
2.1589187397	0.1886471966	0.9999537652	-1.2301428514
1.0473340934	0.770959458	0.9999537652	-1.3287136988
1.5546442448	0.2971793516	0.9999537652	2.1173625742
-1.4283007528	0.4417574616	0.9999537652	1.0030454184
-1.2483094716	0.1531559889	0.9999537652	-1.4800010719
-1.5367922295	0.6205377515	0.9999537652	1.9794217644
-1.2637872473	0.2391554306	0.9999537652	-1.4932072141
6.3075231393	0.4863045655	0.9999537652	2.6452318482
8.064174035	0.4238784445	0.9999537652	2.996475627
1.0914985981	0.7708717765	0.9999537652	-1.5575025402
1.2530475337	0.2341075695	0.9999537652	-1.3590789545
-1.2037557411	0.3401294702	0.9999537652	-1.4252299804
1.7436048685	0.0227335717	0.9999537652	1.4096118677
-1.0188434964	0.884338135	0.9999537652	-1.1128226793
0	0	0	1.3757378992
-1.1030694751	0.8869158163	0.9999537652	1.1651145108
1.2257124861	0.1263379164	0.9999537652	-1.0974260901

-1.2219701176	0.1198705479	0.9999537652	-1.1090759583
-1.7148207449	0.4982811351	0.9999537652	-2.2064515952
-9.5927824577	0.0627300928	0.9999537652	1.8242145631
-1.2992327108	0.2485973151	0.9999537652	-1.4235023404
-1.6979583136	0.52708405	0.9999537652	-2.288586236
-6.9736190158	0.2250321147	0.9999537652	-1.3629566786
1.1561466881	0.4129033313	0.9999537652	-1.1087769401
3.4702345718	0.132094998	0.9999537652	5.1298002698
2.7933974408	0.6948713406	0.9999537652	-9.7542681481
1.2543302685	0.815380382	0.9999537652	-1.1712158574
-1.1457847193	0.9108794384	0.9999537652	-1.1428585529
-1.0245505194	0.9213534784	0.9999537652	1.0662883779
1.1011276872	0.5234153629	0.9999537652	-1.0035642263
-1.2309398501	0.3916807678	0.9999537652	1.1619574673
1.0780282601	0.7318162467	0.9999537652	1.3895796599
-1.1913290417	0.3692893047	0.9999537652	1.0479866055
-1.224757981	0.1652006614	0.9999537652	1.2041752613
0	0	0	6.908559683
0	0	0	2.9985540909
-1.5881570201	0.7123844934	0.9999537652	4.9527501949
4.5505976667	0.5564660232	0.9999537652	-1.3607721321
-1.384480384	0.4810037456	0.9999537652	1.748177722
1.7666151886	0.1578934708	0.9999537652	1.2945704833
0	0	0	2.996475627
2.7933974408	0.6948713406	0.9999537652	0
1.2081164684	0.3330701469	0.9999537652	1.1465227234
1.0046300162	0.9799202937	0.9999537652	1.1518011246
-2.4860479092	0.0127385304	0.9999537652	1.1168613791
1.0212820148	0.8857722916	0.9999537652	1.0000525651
4.5505976667	0.556440658	0.9999537652	-5.3583704783
2.0531936747	0.2756311904	0.9999537652	2.1987962985
0	0	0	-1.0736853543
-1.2567122496	0.3784351985	0.9999537652	1.1187960119
1.1160662736	0.9014358996	0.9999537652	-1.5895292203
0	0	0	15.7262184124
-1.172762946	0.5171201453	0.9999537652	1.2134830475
-1.207469403	0.6947648395	0.9999537652	1.0679265502
-1.3496490808	0.4935816941	0.9999537652	3.5378193056
-1.1300378481	0.5251245248	0.9999537652	1.4782955468
-1.2668701426	0.1444536657	0.9999537652	1.1240481985
-1.0451994804	0.8189126304	0.9999537652	1.0252393679
-1.1311287411	0.581547565	0.9999537652	-1.0126481109
1.7370284568	0.0631395562	0.9999537652	-1.1229324119
0	0	0	-1.1248877899
-1.0450419984	0.9176623542	0.9999537652	1.2676096077
1.5377935878	0.2805114482	0.9999537652	1.6015907506
-1.1970027991	0.2701321857	0.9999537652	-1.1328238368
1.0009753821	0.9942472877	0.9999537652	1.179766711
1.0304023397	0.9338371855	0.9999537652	1.805145171

-1.3911622898	0.6714764518	0.9999537652	1.2664395295
-1.0473198237	0.8921360615	0.9999537652	1.2190466787
-1.1448325836	0.6215951369	0.9999537652	1.949571098
-1.11834697	0.7687480154	0.9999537652	1.5103995078
0	0	0	4.9527501949
3.8622037496	0.619710026	0.9999537652	0
1.781465944	0.0425618473	0.9999537652	1.027718802
-1.1723373306	0.810906083	0.9999537652	-1.4793168818
-1.1172887652	0.6379113812	0.9999537652	-1.0277124968
-1.0597147941	0.6681975472	0.9999537652	1.1005643961
1.3243942908	0.3815867471	0.9999537652	1.2568189063
1.2387774014	0.5127373226	0.9999537652	1.0653716404
-1.3165593074	0.5880561172	0.9999537652	1.099518226
0	0	0	2.996475627
-1.2476752529	0.6099551648	0.9999537652	1.1449032728
1.4406035338	0.3106454122	0.9999537652	1.1727776509
-1.0210282393	0.8682649295	0.9999537652	1.0193911459
-1.1941256282	0.1917251978	0.9999537652	1.0534461771
15.0880352532	0.2955487272	0.9999537652	1.1907369359
-1.8083367302	0.0515238741	0.9999537652	-1.1170164808
-1.3439150003	0.4321137333	0.9999537652	-1.3005596012
2.7933974408	0.6948713406	0.9999537652	1.0664958154
-1.1041393144	0.604397662	0.9999537652	-1.0530450836
-1.6124745138	0.0006538	0.9999537652	-1.4719586316
1.0058857643	0.9679414999	0.9999537652	-1.1327934145
1.0223680143	0.8985487572	0.9999537652	-1.1838591443
2.7957660672	0.7054549956	0.9999537652	-1.1128220641
-1.1445321534	0.8756977135	0.9999537652	0
-1.1787770555	0.3919854824	0.9999537652	-1.5097430549
1.4852169389	0.007731	0.9999537652	1.5087426035
-1.2849485054	0.562643994	0.9999537652	1.0396202231
-1.1327613453	0.6815177044	0.9999537652	-1.1481564361
1.0047371945	0.9889418886	0.9999537652	-1.2309930785
1.4630570633	0.4300756164	0.9999537652	1.2490640241
1.0520250133	0.7738111329	0.9999537652	-1.2032167281
13.3324809638	0.3189346423	0.9999537652	1.0057660328
-1.1681084276	0.5904905481	0.9999537652	1.0100011738
-1.2402457461	0.6563399383	0.9999537652	-1.9356832214
1.4803117167	0.6260913293	0.9999537652	-1.0327071416
0	0	0	6.908559683
-1.0759873204	0.761246365	0.9999537652	1.0656479607
1.0159569092	0.9217857373	0.9999537652	1.0095540101
55.4875632021	0.1159952502	0.9999537652	1.2646703209
1.8453597646	0.0653890279	0.9999537652	-1.2720427236
1.1951394496	0.2587389114	0.9999537652	-1.0793834675
-1.0963287703	0.4868547234	0.9999537652	-1.3643027023
-1.7111605567	0.0842292393	0.9999537652	1.0773138365
-1.2231043212	0.7362276127	0.9999537652	-1.3960345991
1.9232789128	0.0611466815	0.9999537652	-1.0366979705

5.0103795884	0.1210739592	0.9999537652	-1.3101103507
-1.2266851314	0.1450589395	0.9999537652	-1.0705475559
1.231849859	0.7189619079	0.9999537652	-1.9794191391
-1.369107775	0.7698226973	0.9999537652	-1.5412153521
1.3974052255	0.6321454276	0.9999537652	-1.8808194447
-1.0856837069	0.8004074795	0.9999537652	-1.6268402151
-1.4750790135	0.6599462029	0.9999537652	1.7812319687
-1.4010002881	0.6116338218	0.9999537652	1.4660883411
1.6241136236	0.0275915967	0.9999537652	-1.1302308369
2.4296591599	0.0795045123	0.9999537652	1.4787198019
4.9036720728	0.0655346047	0.9999537652	-1.4802387149
-1.0195332935	0.9239511993	0.9999537652	-1.0232755734
1.0671731396	0.7541551974	0.9999537652	-1.1747023109
-1.8560826477	0.3459363187	0.9999537652	-2.5905422285
-1.1548350992	0.4122764219	0.9999537652	-1.0453770166
-1.1785142458	0.7485871462	0.9999537652	-1.483227323
2.3447569417	0.211893034	0.9999537652	-1.1281171991
1.600024502	0.0559755806	0.9999537652	-1.6107187353
-1.2741168816	0.3853552221	0.9999537652	1.30571158
15.0880352532	0.2955600906	0.9999537652	-1.0276776463
-2.4917271213	0.1783393312	0.9999537652	-1.5507301391
-1.0855442704	0.694693508	0.9999537652	1.1963188704
1.4951917803	0.2991770236	0.9999537652	-1.0963303551
1.0576655399	0.7194098387	0.9999537652	1.0738183103
-1.3957712427	0.2187335414	0.9999537652	-1.5016583053
1.0917681293	0.5456829517	0.9999537652	-1.1276070041
0	0	0	-9.7565542884
-1.0740254072	0.7022667466	0.9999537652	-1.0203384344
1.8863176825	0.0301314684	0.9999537652	-1.0706120454
-1.6055270944	0.4698831675	0.9999537652	-2.1778575467
-1.0095045255	0.9511063252	0.9999537652	1.0041787085
-3.5027997556	0.1273933372	0.9999537652	1.2705806872
6.3130691196	0.479799964	0.9999537652	0
2.7933974408	0.6948713406	0.9999537652	0
-1.0804946554	0.658974673	0.9999537652	-1.2668288827
2.6592735212	0.0727705408	0.9999537652	-1.2099923075
2.4790046106	0.1844937023	0.9999537652	2.4531065135
-1.4431547835	0.5561860446	0.9999537652	1.9342142049
0	0	0	2.996475627
-1.0826230524	0.6666958115	0.9999537652	-1.3007591385
1.0665844857	0.7084999517	0.9999537652	1.0770915898
1.116787235	0.7667984927	0.9999537652	1.1264776792
-1.1402367979	0.4901071377	0.9999537652	-1.1704618356
-1.3755764767	0.5062039731	0.9999537652	-1.0094872375
-7.7746186387	0.1018018517	0.9999537652	8.8639046058
-1.1562864818	0.4035291097	0.9999537652	-1.2073873292
4.5505976667	0.5564151234	0.9999537652	0
1.3996890452	0.632749889	0.9999537652	1.5316961476
1.0301390511	0.9310250303	0.9999537652	-1.2050386107

1.009623329	0.9567392518	0.9999537652	1.0165083298
-1.2078980119	0.4861840134	0.9999537652	-1.1544563199
-1.668044468	0.6126052646	0.9999537652	-2.0479626835
1.3662536087	0.1148607852	0.9999537652	-1.0954655284
9.8205505243	0.3807567972	0.9999537652	-1.5811963396
3.8622037496	0.6196794023	0.9999537652	-5.337186734
-2.6501620673	0.0926256257	0.9999537652	-1.0247191477
2.7630567166	0.08160757	0.9999537652	-1.1977486937
2.1680013163	0.413998897	0.9999537652	1.1438154581
1.3772588142	0.5212922706	0.9999537652	-2.0786680745
-50.755668691	0.0155594238	0.9999537652	1.6789724392
1.0464592107	0.8636451492	0.9999537652	-1.2617601037
1.0514999876	0.8741497758	0.9999537652	1.1009329645
1.6152065649	0.510513336	0.9999537652	-1.016338285
1.0789562771	0.6435735229	0.9999537652	1.1981993159
-1.7852186777	0.1739996562	0.9999537652	-1.3016934637
-1.0496273246	0.7863986757	0.9999537652	1.1261132019
1.507205829	0.0953040282	0.9999537652	-1.0310224425
-1.1182593331	0.3319595477	0.9999537652	-1.0987925845
15.0880352532	0.2955199564	0.9999537652	-5.3583704783
0	0	0	1.5920908625
-2.0683719388	0.0530594441	0.9999537652	-1.0085837439
-1.4609312109	0.3012801124	0.9999537652	-1.3070546205
-1.3634739279	0.1221888448	0.9999537652	1.1740249651
1.0699971675	0.7380353785	0.9999537652	-1.0751659649
-1.5732704371	0.0190437976	0.9999537652	1.0318671678
1.598158932	0.1453509655	0.9999537652	1.2470860643
1.0024679426	0.9952977543	0.9999537652	-1.2113577925
1.0363099496	0.966677396	0.9999537652	2.3290926981
1.0936518072	0.5410906016	0.9999537652	1.1615393661
1.0635937345	0.6839506103	0.9999537652	1.382616823
1.0756524821	0.8533867135	0.9999537652	1.0891191519
-1.1795068457	0.3620421941	0.9999537652	1.4317855033
-1.0528998347	0.8661375089	0.9999537652	1.3277698325
1.0211288116	0.9491113539	0.9999537652	1.0984583154
1.1352411167	0.6761924187	0.9999537652	-1.0904472788
-6.9736190158	0.2250321147	0.9999537652	33.3754495834
1.3501637095	0.4315723369	0.9999537652	1.3991327592
1.1463129501	0.6626928205	0.9999537652	1.0897949512
2.7933974408	0.6948713406	0.9999537652	2.996475627
-1.0332846416	0.8837205328	0.9999537652	1.1813173288
1.0856276127	0.5825951275	0.9999537652	-1.0311500697
-1.1453192235	0.9086853641	0.9999537652	9.7116702472
-1.820792799	0.2644391974	0.9999537652	1.4041776103
1.7191537344	0.0267261684	0.9999537652	1.5772587999
1.0015043352	0.9959331681	0.9999537652	-1.1283021243
-1.1802121107	0.5965296325	0.9999537652	1.0286673153
1.0240838787	0.9687325862	0.9999537652	2.251432921
-1.0846500161	0.8176478469	0.9999537652	1.482012642

1.3505284097	0.1646632732	0.9999537652	1.2641313492
29.1472433471	0.1877726381	0.9999537652	1.900054964
1.0690140949	0.8633591762	0.9999537652	1.2697899088
1.3670587466	0.4856641668	0.9999537652	1.0949825209
-1.0158172782	0.9495523606	0.9999537652	1.4531489341
1.5124661612	0.6687868988	0.9999537652	1.7125218959
-1.0378653972	0.8795136902	0.9999537652	1.0313696213
-1.0976720675	0.6312026761	0.9999537652	1.1618212163
1.1286899667	0.7985722824	0.9999537652	-1.4619975468
0	0	0	2.9985540909
4.5545995984	0.5696683115	0.9999537652	-1.859280336
1.2095848585	0.2063690438	0.9999537652	1.2312613508
-1.0736458315	0.7406031379	0.9999537652	1.6080621847
4.5544554512	0.5691453516	0.9999537652	1.5210253981
1.3114512769	0.3771622845	0.9999537652	1.4339703196
1.6018473574	0.6931423568	0.9999537652	1.372315235
1.1072783692	0.6434680238	0.9999537652	1.5355739057
0	0	0	2.9985540909
-1.4666797245	0.5200574061	0.9999537652	-1.1317444999
1.0817078961	0.766093447	0.9999537652	1.1604822459
-1.0753395146	0.7634871323	0.9999537652	1.3936406129
-1.4337007867	0.1081440698	0.9999537652	-1.6515493947
1.1166829721	0.570827938	0.9999537652	-1.0094428686
1.1617402047	0.3642226376	0.9999537652	1.0141235504
-1.548632686	0.021702788	0.9999537652	-1.0294756193
1.5744786829	0.3168739475	0.9999537652	-1.1614572515
1.1845873495	0.3842763811	0.9999537652	1.0204915949
-1.1062047527	0.6455117653	0.9999537652	-1.2173196102
-1.3834317179	0.4349621548	0.9999537652	-1.2778114429
1.1232387835	0.8893374972	0.9999537652	-2.5494282815
2.7933974408	0.6948713406	0.9999537652	-2.0477227478
1.0923445629	0.6286873067	0.9999537652	1.2364199616
-3.7261776128	0.1087236379	0.9999537652	0
1.0227578616	0.9399987838	0.9999537652	-1.2160210349
-1.0140895429	0.9272405751	0.9999537652	-1.1074043328
1.7745951127	0.5416997886	0.9999537652	-1.9769471573
1.5898914244	0.1238882968	0.9999537652	-1.1081625653
-1.0560883693	0.7572902809	0.9999537652	-1.0949187445
2.2637293736	0.080991002	0.9999537652	-1.4437589708
-2.6345204407	0.2430372092	0.9999537652	-4.739411962
-1.1095165555	0.8082433599	0.9999537652	1.0846813027
1.1286060605	0.866816283	0.9999537652	1.0136522632
4.5545995984	0.5696662339	0.9999537652	1.0682244185
-1.037914349	0.8957161684	0.9999537652	-1.0046999324
1.0109046375	0.962223729	0.9999537652	-1.3139605064
-1.0675553957	0.7538023248	0.9999537652	-1.1690697673
1.0032481056	0.9907056763	0.9999537652	-1.2601852059
1.0469217539	0.8461962137	0.9999537652	1.4343588247
13.3324809638	0.3190079485	0.9999537652	2.9985540909

-1.2616259486	0.4479022907	0.9999537652	-1.2875982219
6.3075231393	0.4862681283	0.9999537652	6.908559683
2.8816679996	0.0400107684	0.9999537652	1.0661970817
2.7933974408	0.6948713406	0.9999537652	0
1.3095754617	0.0877227163	0.9999537652	1.0065666535
-1.1058199357	0.6669196392	0.9999537652	1.0546700183
2.7933974408	0.6948713406	0.9999537652	1.2400291296
-1.1890004485	0.246389918	0.9999537652	1.0649335629
-1.2609714585	0.2279981695	0.9999537652	1.006775724
2.7933974408	0.6948713406	0.9999537652	3.9269887543
-1.1946935256	0.3814918957	0.9999537652	-1.0332654698
0	0	0	2.9985540909
-1.2111814081	0.6467568602	0.9999537652	1.4112067783
4.5505976667	0.5563619862	0.9999537652	4.3937380937
-1.3639673362	0.5907306055	0.9999537652	-1.405957898
-1.5481079555	0.0840867137	0.9999537652	-1.2780476548
1.2687506623	0.7467517407	0.9999537652	1.0415785031
1.4586470775	0.3940631694	0.9999537652	-1.0742973591
1.0009605121	0.9972645157	0.9999537652	-1.1572065783
1.1825768591	0.4614877039	0.9999537652	1.0441213776
1.337664844	0.0707673575	0.9999537652	1.1754041321
-1.0833161037	0.914876947	0.9999537652	-1.0254529169
-1.2027291607	0.1946461226	0.9999537652	1.0670616049
-1.0691633897	0.6437424374	0.9999537652	-1.0597808712
0	0	0	2.9985540909
0	0	0	-5.3589986948
-1.2659472121	0.0806765858	0.9999537652	-1.0838379999
-1.0376846293	0.8579295013	0.9999537652	1.0676458229
0	0	0	6.908559683
0	0	0	6.908559683
1.4515847663	0.0869404399	0.9999537652	1.065203236
2.7933974408	0.6948713406	0.9999537652	-2.7915177751
0	0	0	-1.1254032646
-3.0110183514	0.1138533899	0.9999537652	-1.2187323585
-1.0226571002	0.901094485	0.9999537652	1.0897990373
-1.2052254439	0.6721566646	0.9999537652	-2.0131438349
1.0612749977	0.8740271148	0.9999537652	1.0059581563
-1.246467954	0.1309427953	0.9999537652	1.0334418698
-1.03892764	0.8717901581	0.9999537652	1.0265208468
-1.48121844	0.5565015108	0.9999537652	0
1.0635656281	0.7811427267	0.9999537652	1.0673691188
1.0004886944	0.9974822022	0.9999537652	1.1406433644
1.8169036893	0.0896755479	0.9999537652	-1.254583778
1.4755590332	0.5146767055	0.9999537652	1.2076110237
-1.0677148635	0.8741103012	0.9999537652	-1.2743617254
0	0	0	4.9527501949
1.1636836978	0.5121444977	0.9999537652	-1.1484397129
-1.1421973186	0.5529506241	0.9999537652	1.1087985085
-1.4844123711	0.00662	0.9999537652	-1.3163544464

-1.1578795329	0.5914802508	0.9999537652	1.0661666444
1.5127636079	0.0216998541	0.9999537652	1.3003119651
2.7933974408	0.6948713406	0.9999537652	-1.8594983184
1.0765769989	0.8446830992	0.9999537652	-1.0682552474
-1.0188463246	0.9373302005	0.9999537652	1.0467757178
3.8622037496	0.61964876	0.9999537652	23.4460379037
1.1050578775	0.4538444757	0.9999537652	1.2666576723
2.7933974408	0.6948713406	0.9999537652	8.8710369702
1.0457737398	0.7996206691	0.9999537652	1.0459149273
-1.6746616296	0.3278246771	0.9999537652	-1.0004315405
2.7933974408	0.6948713406	0.9999537652	0
-1.5570528664	0.4131140951	0.9999537652	1.039747066
-2.1334087267	0.5175577046	0.9999537652	-1.1514942629
4.2566373836	0.0885984513	0.9999537652	-1.2219141927
0	0	0	4.9527501949
0	0	0	1.438718359
-1.1016476186	0.5821501123	0.9999537652	-1.0428418294
-1.1287383113	0.4935577196	0.9999537652	-1.2685780432
2.7933974408	0.6948713406	0.9999537652	0
1.8464669901	0.5922227875	0.9999537652	2.6421916756
-1.1017337822	0.8478287174	0.9999537652	-1.927029328
-1.0020289441	0.9900933782	0.9999537652	-1.3371155224
-1.6089495277	0.1284887215	0.9999537652	1.271682445
8.064174035	0.4238771103	0.9999537652	1.5655879817
-1.8209982108	0.2153860755	0.9999537652	-1.171774559
1.223681156	0.5781187171	0.9999537652	1.0843122041
1.1668597755	0.7359621063	0.9999537652	1.0819688052
-2.9497018331	0.0588564237	0.9999537652	1.2438563437
1.229695584	0.2556650593	0.9999537652	1.2243668844
1.1704029766	0.4610561196	0.9999537652	1.1128827805
1.1435182	0.4181017698	0.9999537652	1.1441005545
1.1263324535	0.8145382651	0.9999537652	-1.1015031859
-6.288827244	0.0293435012	0.9999537652	3.9571433256
-1.1509628501	0.6136349518	0.9999537652	1.2209759256
1.0725249281	0.7997717423	0.9999537652	-1.1711687769
-1.1641924398	0.6558513434	0.9999537652	1.0385141526
1.8388612766	0.4315161138	0.9999537652	3.0008703834
1.021212418	0.935518301	0.9999537652	-1.0359193237
-1.254909014	0.4853647431	0.9999537652	-1.023007165
1.1806772154	0.7495727328	0.9999537652	-1.1278975984
11.5868260078	0.3461772737	0.9999537652	-1.2898906004
-6.9736190158	0.2250321147	0.9999537652	3.8534174358
-1.0538695212	0.9091950398	0.9999537652	-1.3749993982
1.0865066769	0.7974904048	0.9999537652	1.0870661597
2.0354228963	0.4861596009	0.9999537652	-1.5086090391
-1.2756389503	0.1833985031	0.9999537652	-1.0340256646
1.0479097225	0.9147663298	0.9999537652	-1.2240318791
0	0	0	2.996475627
15.1008094413	0.2915612232	0.9999537652	-1.01313437

1.2153735694	0.8151665331	0.9999537652	-1.4240498189
53.6412760632	0.1197313309	0.9999537652	1.3012351799
4.5505976667	0.5563328301	0.9999537652	0
15.1919090446	0.3045457497	0.9999537652	2.9988886918
1.1535167817	0.7551108836	0.9999537652	-1.0509912817
-1.4010359172	0.6071843271	0.9999537652	1.1234461737
1.2526375214	0.8109284803	0.9999537652	-3.8998793135
-1.1568399326	0.5421961816	0.9999537652	1.210836571
-2.9163669278	0.006007	0.9999537652	-1.4551559136
20.3530564257	0.2434848478	0.9999537652	-1.3789635973
1.0509548006	0.9032023597	0.9999537652	1.2901207628
-1.2611741857	0.4399681334	0.9999537652	-1.9291724354
1.2443472443	0.386778837	0.9999537652	-1.660034509
-1.1611298266	0.674847426	0.9999537652	1.8021226464
1.0174541181	0.9233412317	0.9999537652	-1.2092420544
-2.5861438062	0.4672283668	0.9999537652	0
-1.1615474484	0.4773204886	0.9999537652	-1.1672696386
-2.3882871517	0.0007052	0.9999537652	-2.5201094407
-1.4976958482	0.0645417815	0.9999537652	-1.1966726182
1.1515210251	0.5210981896	0.9999537652	-1.3302470611
0	0	0	3.2466971401
6.3075231393	0.4862237795	0.9999537652	-1.4669967683
1.0434219265	0.7671692197	0.9999537652	1.2458202433
1.1031767962	0.7815083378	0.9999537652	1.1567313963
1.1158583321	0.9296681264	0.9999537652	9.7116702472
1.3147542662	0.4058335228	0.9999537652	1.4980847037
1.1378846384	0.472466636	0.9999537652	1.0036504876
-1.5269106325	0.4137696294	0.9999537652	-1.0699391644
1.3750192721	0.4273948217	0.9999537652	-1.4577791134
0	0	0	1.6439796197
-1.1866539258	0.5374691994	0.9999537652	1.2924138922
1.188489195	0.1920217629	0.9999537652	1.0143999945
2.7933974408	0.6948713406	0.9999537652	4.9527501949
1.2437817305	0.326058723	0.9999537652	1.0153595794
-1.6062238006	0.4515764407	0.9999537652	2.0954037025
-1.3458534658	0.4020030831	0.9999537652	-1.0131114019
-1.1179872155	0.6193269101	0.9999537652	1.047013589
-1.3180319268	0.2605711556	0.9999537652	-1.0637928876
0	0	0	6.908559683
-1.2044052565	0.3546926418	0.9999537652	-1.1611494096
-1.2269838739	0.1477034055	0.9999537652	-1.0714700564
1.1628464825	0.3789673533	0.9999537652	1.077508691
-1.0878810912	0.6161405332	0.9999537652	-1.0787609747
1.0633715935	0.6621042142	0.9999537652	-1.1551527277
1.2380436037	0.5087171542	0.9999537652	-1.04155603
-1.0094714013	0.9758225653	0.9999537652	-1.0944955585
1.030307896	0.9667510464	0.9999537652	1.5539805427
-1.0370202165	0.8577820542	0.9999537652	-1.0812845764
-1.0597883439	0.7127534379	0.9999537652	-1.005060256

18.9318466825	0.2781664069	0.9999537652	0
-1.1626817313	0.4257088267	0.9999537652	-1.0637726403
-1.1111459599	0.5071111552	0.9999537652	-1.0311822316
-1.0196283351	0.8797351657	0.9999537652	-1.0969027356
1.3561507449	0.629398347	0.9999537652	1.4542066119
3.2110135335	0.1652033293	0.9999537652	1.2800255125
1.0068767682	0.9786578183	0.9999537652	-1.1344181147
1.0357179724	0.8418230468	0.9999537652	1.0671621789
1.35645026	0.5176296782	0.9999537652	1.1229679312
0	0	0	-2.2358187773
6.3128691894	0.4814674928	0.9999537652	-5.3589986948
0	0	0	-5.3608259368
15.2205810263	0.3051907401	0.9999537652	-1.1248877899
-1.3245325997	0.422692457	0.9999537652	-1.0100398867
1.2494505312	0.2603502817	0.9999537652	-1.5119983952
1.066111387	0.7388063229	0.9999537652	1.105018084
-1.0810971762	0.849058129	0.9999537652	1.0284042522
1.2917344047	0.3133937127	0.9999537652	-1.0457065855
-1.1814667754	0.3423587163	0.9999537652	-1.1276417639
4.5505976667	0.5562991171	0.9999537652	0
2.7933974408	0.6948713406	0.9999537652	2.0626766153
0	0	0	4.9527501949
1.3594099105	0.7971568125	0.9999537652	1.4521160839
1.0819921721	0.7095507835	0.9999537652	-1.134031787
4.5505976667	0.5562571852	0.9999537652	-4.9774159092
1.0064665989	0.9768981976	0.9999537652	1.1714076481
2.7933974408	0.6948713406	0.9999537652	2.9985540909
2.7933974408	0.6948713406	0.9999537652	-6.3473723183
1.0511544362	0.7969562445	0.9999537652	1.1933507799
-1.0960739249	0.4602576868	0.9999537652	-1.033388119
-1.2286854188	0.1466160924	0.9999537652	-1.0232625697
0	0	0	-2.0467835026
1.4008305856	0.6299599604	0.9999537652	-2.4599066101
-1.1233419277	0.6945016606	0.9999537652	-1.1643220052
6.3075231393	0.4861796293	0.9999537652	1.6470100299
-2.0025825252	0.456359036	0.9999537652	1.5919129963
1.0994820275	0.5779163587	0.9999537652	1.4370261445
1.1469711324	0.344959478	0.9999537652	1.1666741801
1.1459153227	0.6017347741	0.9999537652	1.1973645778
-1.4776048529	0.4065393083	0.9999537652	-1.6364712014
3.0602334741	0.3285759071	0.9999537652	0
-1.3596770852	0.7810203465	0.9999537652	-1.0958850627
-2.3181698201	0.1836451987	0.9999537652	-1.2386335486
2.3808533718	0.153601287	0.9999537652	-1.2344071947
1.3256146221	0.0700660036	0.9999537652	-1.1073780404
1.1736749605	0.2414473385	0.9999537652	1.1764158184
1.7022866271	0.1750107412	0.9999537652	1.4970505639
1.6054143334	0.5414357928	0.9999537652	1.0338956323
4.5505976667	0.5561869329	0.9999537652	1.1226213608

1.0287239533	0.8546577586	0.9999537652	-1.0104170195
-1.0020339672	0.9880688053	0.9999537652	-1.049171694
1.953521676	0.2100934579	0.9999537652	-1.1676567136
2.7933974408	0.6948713406	0.9999537652	2.996475627
-1.0000938499	0.9996105876	0.9999537652	-1.0191372757
-1.0557017092	0.7665945745	0.9999537652	-1.1032338365
4.5505976667	0.5561580072	0.9999537652	6.908559683
0	0	0	0
0	0	0	6.908559683
1.4391923523	0.3770548623	0.9999537652	-1.1146602513
-1.251751213	0.6788034812	0.9999537652	-1.0998368348
1.3203746378	0.1870717086	0.9999537652	-1.3081460647
1.1822500266	0.5915448618	0.9999537652	-1.2758565713
2.7957660672	0.7054573471	0.9999537652	12.7732027325
1.5721822191	0.1606232785	0.9999537652	1.0236515308
-2.223376117	0.5879069091	0.9999537652	2.046451628
-1.1035861323	0.6503800652	0.9999537652	1.2373775498
-1.0869970173	0.8275762782	0.9999537652	-1.4494702798
-1.0653719869	0.7788560702	0.9999537652	1.1292924556
1.1945489934	0.3486330812	0.9999537652	-1.1242721237
1.2637355251	0.7540419027	0.9999537652	-1.4566656456
-1.121196012	0.66498757	0.9999537652	1.1253364549
1.4325240009	0.2069964187	0.9999537652	-1.1733158479
-1.0085355311	0.9930346205	0.9999537652	1.2398837679
1.0362702605	0.9655851499	0.9999537652	1.6418498278
-1.6994504307	0.5419668182	0.9999537652	1.3547781079
1.3317767424	0.1992633387	0.9999537652	-1.7747551607
-1.0517171444	0.8251395003	0.9999537652	1.0105238852
6.2299503085	0.0751870433	0.9999537652	1.1395888107
3.4578336419	0.004319	0.9999537652	1.3630456425
2.5552811191	0.2419329475	0.9999537652	-4.3692038192
-1.1038391045	0.6717451347	0.9999537652	-1.0318603456
1.3360869003	0.3330402362	0.9999537652	-1.1064766672
3.2736636389	0.0978249605	0.9999537652	1.127103216
1.5021571103	0.2069953103	0.9999537652	1.2468284343
1.1293956656	0.3532611304	0.9999537652	-1.374673629
-1.8908250111	0.5555239461	0.9999537652	-4.9108225367
2.7933974408	0.6948713406	0.9999537652	-2.5071004691
2.0813661621	0.3172021396	0.9999537652	1.0164363995
1.1832039436	0.4341537957	0.9999537652	1.0829567717
1.3923292999	0.713350935	0.9999537652	-1.1412590179
1.4162196156	0.1286446983	0.9999537652	-1.0078804636
1.1070641608	0.5584639263	0.9999537652	-1.0783708852
-1.0415260168	0.7842709791	0.9999537652	1.1305699783
1.1124051152	0.5950574551	0.9999537652	1.5524058421
2.7933974408	0.6948713406	0.9999537652	1.9292743022
1.0523610038	0.7493744866	0.9999537652	1.0571043664
0	0	0	2.9985540909
-1.2836417843	0.2574505269	0.9999537652	-1.9426625187

-1.223172313	0.3180919263	0.9999537652	-1.547840244
0	0	0	2.996475627
1.1397030765	0.5073985606	0.9999537652	1.0597429399
-2.0400392671	0.0865915992	0.9999537652	-1.0861980088
1.263979281	0.1005763855	0.9999537652	1.0167207218
-1.1765350641	0.5848332895	0.9999537652	1.0584041868
-1.0142452712	0.9342523881	0.9999537652	1.387787332
1.0037516139	0.9871352156	0.9999537652	1.1001295429
0	0	0	-1.9403006466
1.2351468843	0.1112755147	0.9999537652	-1.0280863348
2.7933974408	0.6948713406	0.9999537652	0
1.2246548654	0.6157074648	0.9999537652	-1.0130248265
-1.2431005095	0.4816230389	0.9999537652	-1.0430145029
-1.1356228087	0.6491735257	0.9999537652	-1.0655924886
1.0282459458	0.9687926839	0.9999537652	-1.5462126468
1.0913024785	0.686817292	0.9999537652	1.1655150126
1.0431845699	0.7474082564	0.9999537652	1.4737622543
-1.253267676	0.1034114353	0.9999537652	1.010286565
2.3273576668	0.167482631	0.9999537652	-1.715175694
1.0275436744	0.9019313437	0.9999537652	1.2298834542
-1.1997001219	0.3037766307	0.9999537652	-1.1841176034
-1.0581474895	0.6957958183	0.9999537652	1.0937817986
-1.0464981522	0.8171357685	0.9999537652	1.2279497699
1.0395710976	0.8463473807	0.9999537652	1.4866411378
2.7933974408	0.6948713406	0.9999537652	1.2698746349
-1.1979632149	0.429775155	0.9999537652	-1.1193617104
1.6462932404	0.5843296474	0.9999537652	-2.7463406416
-1.0024905434	0.9933822734	0.9999537652	-1.1447899711
-1.6124601893	0.0384199601	0.9999537652	-1.0931172792
2.189053179	0.0153209584	0.9999537652	1.7028255667
3.5444921315	0.2673911736	0.9999537652	1.1120558165
0	0	0	16.0748987101
8.064174035	0.4238757759	0.9999537652	-3.7211335265
1.2242429853	0.3120906136	0.9999537652	-1.5626631862
1.1598817343	0.4692331533	0.9999537652	1.2997348813
1.1691438373	0.3737364535	0.9999537652	1.1769727091
1.2180425134	0.1944775843	0.9999537652	-1.0041810378
1.5303596704	0.0207133392	0.9999537652	1.3463368731
-1.3669830632	0.109907973	0.9999537652	1.0673610223
1.0573525321	0.7135563237	0.9999537652	-1.1469809416
1.2115978667	0.312891221	0.9999537652	1.2276273407
1.0191377156	0.9602924614	0.9999537652	-1.0102528506
1.2668413654	0.207412658	0.9999537652	1.2523444325
-1.0256289156	0.9086093884	0.9999537652	1.2458120923
-1.2279344853	0.3577981705	0.9999537652	-1.180160306
1.3515500654	0.3003658593	0.9999537652	1.0382087646
-1.0982242123	0.6078529122	0.9999537652	-1.1937344346
2.0032022457	0.3619618067	0.9999537652	1.2273613936
-1.9924022667	0.0906760315	0.9999537652	1.1303568222

-1.3100198367	0.0370012693	0.9999537652	-1.1389167008
-1.0467643843	0.7403727899	0.9999537652	-1.1201074723
-1.276202567	0.2481802831	0.9999537652	1.213143819
1.2078564839	0.5676101056	0.9999537652	1.0051370783
-1.9751371463	0.1529716611	0.9999537652	-1.6408646877
4.5505976667	0.556132005	0.9999537652	0
4.5505976667	0.5561082901	0.9999537652	-5.1739447884
2.7933974408	0.6948713406	0.9999537652	2.2955915087
-1.4002029808	0.0580088814	0.9999537652	-1.1009278353
1.3801211037	0.2826357677	0.9999537652	-1.1938750561
1.0280692272	0.8647011676	0.9999537652	-1.3357442966
-1.2620409198	0.6225761548	0.9999537652	-1.0641213227
-1.2788125598	0.1432637523	0.9999537652	-1.381729217
-1.2348596062	0.5229224908	0.9999537652	1.0858980158
8.064174035	0.4238744414	0.9999537652	-2.0448962242
-1.0122239484	0.9560546715	0.9999537652	-1.2875781996
1.0200819327	0.9466776035	0.9999537652	1.15141394
0	0	0	10.818785458
1.0109184602	0.9303589463	0.9999537652	-1.087377481
-1.0973160202	0.6306645382	0.9999537652	-1.0301650575
-1.0604024991	0.79561619	0.9999537652	-1.0591944152
-1.1423160089	0.6824108333	0.9999537652	1.1069621951
-1.2978773206	0.2129267365	0.9999537652	1.1265041036
-1.092139102	0.6399378859	0.9999537652	1.0464983022
1.1571172166	0.3601129168	0.9999537652	1.1458014355
-2.236788418	0.3266929719	0.9999537652	-1.1200409105
-1.4178743856	0.1287345727	0.9999537652	-1.1600137412
-1.5554675182	0.0163030502	0.9999537652	-1.1247692664
4.5505976667	0.5560863595	0.9999537652	-4.6920174118
-1.0272368189	0.8797232709	0.9999537652	-1.2751749469
1.0719128465	0.584535402	0.9999537652	1.453161514
-1.3245004224	0.0307969155	0.9999537652	1.1886705827
4.5544554512	0.5691433571	0.9999537652	-4.3722641122
1.1241226732	0.8926902791	0.9999537652	-1.1708308502
1.7647165815	0.0597468529	0.9999537652	1.2825358522
0	0	0	-5.3583704783
0	0	0	2.996475627
-1.0392708541	0.8458751301	0.9999537652	1.253018982
1.3612474801	0.3750878438	0.9999537652	-1.3542976344
1.2323865332	0.2800221673	0.9999537652	1.3827196778
-1.1680863849	0.8568284088	0.9999537652	1.3387790497
-1.5208575531	0.0746295657	0.9999537652	1.1380775944
-1.3329960572	0.1193763663	0.9999537652	1.3819189474
1.2498120241	0.1170317475	0.9999537652	1.0266614246
0	0	0	10.8262784008
1.4834137528	0.2112356808	0.9999537652	-1.0013772798
-1.1512865013	0.3785281582	0.9999537652	1.010529564
-1.233588922	0.1636553884	0.9999537652	-1.0599868972
1.0594228089	0.6268420638	0.9999537652	1.0811375866

1.2702545883	0.4401717939	0.9999537652	1.1037062644
1.1681770476	0.2600413715	0.9999537652	1.051679934
1.0037640145	0.980765678	0.9999537652	1.0867500154
-1.0662897099	0.7266335214	0.9999537652	1.2130735705
-1.5326271468	0.2938516578	0.9999537652	-1.0785343651
-1.2155605447	0.1891699723	0.9999537652	-1.3132139096
4.5544554512	0.5691413627	0.9999537652	1.5908108123
-1.0591089595	0.7028910207	0.9999537652	1.0013146358
-1.842677928	0.4680134363	0.9999537652	-1.1242401582
2.7933974408	0.6948713406	0.9999537652	-1.8601323346
-1.5288655916	0.5023053785	0.9999537652	-3.0224955488
1.1140457014	0.6784957406	0.9999537652	-1.2041068002
-1.2583336287	0.8351114694	0.9999537652	1.440358797
-1.202939207	0.3143302746	0.9999537652	-1.1779462793
2.7957660672	0.7054596996	0.9999537652	0
1.0722775422	0.6556950068	0.9999537652	1.1543073472
-1.1786859467	0.3667046044	0.9999537652	-1.0901705567
-1.802154181	0.1425967086	0.9999537652	1.1095113765
-1.1457555741	0.9107285115	0.9999537652	1.0606581302
8.064174035	0.4238731068	0.9999537652	-7.959096917
-1.1358678147	0.4180873187	0.9999537652	1.3202505372
-1.5881570201	0.7110208511	0.9999537652	1.9416545697
-2.0390556158	0.0556320943	0.9999537652	-1.2495268798
1.0459683271	0.8555993664	0.9999537652	1.188497982
-1.505965599	0.529615042	0.9999537652	-1.1297098826
1.4652402235	0.3490070957	0.9999537652	1.2082749083
-1.9289169832	0.1905518369	0.9999537652	-1.3574402742
1.0852187741	0.5125551742	0.9999537652	1.1498771274
2.7933974408	0.6948713406	0.9999537652	-3.3826461929
-1.1427037524	0.4763009816	0.9999537652	1.3430349324
1.1704229895	0.4645420883	0.9999537652	1.147577427
1.0625660793	0.7893754683	0.9999537652	-1.1412760187
-2.5871290907	0.4991362671	0.9999537652	1.8445572683
4.5544554512	0.5691393684	0.9999537652	12.7732027325
4.5505976667	0.5560657957	0.9999537652	6.908559683
-1.096920501	0.6892125686	0.9999537652	-1.0570735447
1.0559411056	0.7551192743	0.9999537652	1.0592181781
1.2893973291	0.2926371768	0.9999537652	1.5726447983
-1.5875118025	0.720251633	0.9999537652	3.7001724876
0	0	0	0
1.204998764	0.3010833591	0.9999537652	1.417651017
-1.392339597	0.6755381597	0.9999537652	2.4345137645
0	0	0	2.9985540909
-1.2173029028	0.0902143968	0.9999537652	1.0370998287
-1.131171467	0.7648067589	0.9999537652	1.5264438305
-1.2930181341	0.0956381807	0.9999537652	-1.0896796757
-1.2572750762	0.4052101402	0.9999537652	1.4040927879
-1.0991276914	0.5516200718	0.9999537652	1.3293577153
1.0741436455	0.6128534745	0.9999537652	1.2375014729

1.3026120258	0.7423968732	0.9999537652	-1.4281216211
1.3823751836	0.5608616322	0.9999537652	1.0286352509
0	0	0	2.996475627
1.1193074263	0.6072481997	0.9999537652	1.0590215726
13.3324809638	0.3193640946	0.9999537652	2.9940379764
3.8622037496	0.6196181007	0.9999537652	0
-1.3842138473	0.5497909944	0.9999537652	-1.7724539785
1.0971336136	0.721275385	0.9999537652	1.3570002307
1.1879709748	0.4662950507	0.9999537652	1.4611233905
4.5505976667	0.5560462223	0.9999537652	-1.1520337027
-1.1208016402	0.473577852	0.9999537652	1.0247423279
1.0392068921	0.7682938924	0.9999537652	1.2969007265
-1.0461124926	0.8234169422	0.9999537652	1.2341202131
2.7933974408	0.6948713406	0.9999537652	2.996475627
1.092409221	0.8382741486	0.9999537652	1.245516455
-1.7956491197	0.1167857588	0.9999537652	1.1088050775
1.1399992826	0.6331216595	0.9999537652	1.3380476217
-2.0695649939	0.2615065035	0.9999537652	1.8007532435
1.1742141824	0.3744016908	0.9999537652	1.3402133879
11.5766527773	0.3455050172	0.9999537652	1.2593062028
0	0	0	2.9985540909
2.7933974408	0.6948713406	0.9999537652	4.9527501949
1.8984965231	0.3733514763	0.9999537652	1.1041736379
-1.1068338728	0.615198139	0.9999537652	1.1687219536
-1.1203230916	0.4426459321	0.9999537652	1.1049617158
6.3075231393	0.4861356763	0.9999537652	-1.358587248
0	0	0	1.590997316
2.8674152405	0.0540694617	0.9999537652	-1.095747511
-1.0792445099	0.8001475681	0.9999537652	1.1543048025
-1.1798309713	0.3777268943	0.9999537652	1.3203855356
1.3966050591	0.1303197189	0.9999537652	1.0347583869
2.7933974408	0.6948713406	0.9999537652	0
-1.1836454075	0.6002404187	0.9999537652	1.2625261472
6.3075231393	0.4860919192	0.9999537652	10.818785458
11.5766527773	0.345492614	0.9999537652	4.9527501949
2.0707589375	0.069546204	0.9999537652	1.2182278002
-1.1984339434	0.5246452917	0.9999537652	1.0695058583
1.4714168225	0.4767765058	0.9999537652	1.2400674234
4.5505976667	0.5560272763	0.9999537652	0
0	0	0	0
2.2819357465	0.3099613476	0.9999537652	1.2947771782
-1.0546187585	0.8496444247	0.9999537652	1.4420993255
1.332233652	0.6933826263	0.9999537652	1.0260196848
-1.4364779627	0.0997278458	0.9999537652	1.5245109895
0	0	0	22.5851407947
1.9925935006	0.0619633288	0.9999537652	1.304595304
1.3480788142	0.7449357005	0.9999537652	3.63273905
-1.1212262383	0.5153069526	0.9999537652	-1.0658168347
-1.125291708	0.6343934928	0.9999537652	1.2532564108

-1.0511738413	0.843379991	0.9999537652	1.3041555449
1.2406255197	0.5479456661	0.9999537652	3.8452636367
-1.5087575773	0.0263021649	0.9999537652	1.106995005
1.0079285648	0.9591159418	0.9999537652	1.3150992382
-1.1314980094	0.8432613978	0.9999537652	1.2022796328
1.0519812542	0.6921062325	0.9999537652	1.2166961959
-1.0249860165	0.9391489357	0.9999537652	1.1340447181
1.0290698449	0.828839208	0.9999537652	-1.2183943128
2.0852096246	0.0005036	0.9999537652	1.0619816487
-1.0588471763	0.635560031	0.9999537652	1.0048277686
4.5505976667	0.5560085526	0.9999537652	0
4.5505976667	0.5559558646	0.9999537652	-1.9771482009
-1.0032839687	0.9813634982	0.9999537652	1.2160179625
0	0	0	6.9133480437
1.156559555	0.5167778306	0.9999537652	1.7661304954
-1.1411681157	0.6272034293	0.9999537652	-1.3865388198
-1.2447511482	0.4525099718	0.9999537652	1.5677035527
-1.0748325078	0.7892516418	0.9999537652	1.0949964284
1.1671034383	0.5180434207	0.9999537652	-1.0960325565
-1.0359450606	0.756812221	0.9999537652	1.136315332
0	0	0	3.0002604593
-1.2430627591	0.6053999059	0.9999537652	1.3478082381
-1.0247494686	0.8689405362	0.9999537652	1.152265706
1.9989289019	0.2187844789	0.9999537652	-1.4683302163
1.0638984996	0.8108554386	0.9999537652	1.0760100827
1.1332113201	0.6534093832	0.9999537652	1.2460833343
1.0789600992	0.5241258421	0.9999537652	1.4156396796
-1.0347070303	0.7634790813	0.9999537652	1.1334731983
1.0543450169	0.6867875909	0.9999537652	1.043364822
1.1162834585	0.9271775215	0.9999537652	1.8396312969
1.1757527805	0.5136398178	0.9999537652	-1.1070711108
-1.5293553469	0.1726570613	0.9999537652	1.1305594983
-1.0317755932	0.8669683763	0.9999537652	1.105575701
-1.294675136	0.2289338838	0.9999537652	1.0815198275
2.7933974408	0.6948713406	0.9999537652	1.590997316
-1.4848828492	0.5184831105	0.9999537652	-1.1668348393
1.0144764616	0.9174454092	0.9999537652	1.6042374236
1.3177384511	0.2167116969	0.9999537652	-1.1973886373
4.5505976667	0.5559322269	0.9999537652	24.4899985757
-2.770555493	0.2451776615	0.9999537652	1.1519219883
1.3763731265	0.0862569354	0.9999537652	1.8254317785
-1.0377743758	0.8141039036	0.9999537652	1.1601852501
5.743746418	0.0979203093	0.9999537652	1.0675791322
2.7933974408	0.6948713406	0.9999537652	0
1.1162834585	0.9271695543	0.9999537652	1.1692101602
2.0903962787	0.5155815843	0.9999537652	-1.0342584907
3.8622037496	0.6195874233	0.9999537652	0
9.8205505243	0.3807299055	0.9999537652	-2.0479626835
-1.0008217171	0.9952338996	0.9999537652	1.4218960869

-1.1554181492	0.5064426488	0.9999537652	1.2531440909
0	0	0	1.4524478337
1.118812399	0.6352956877	0.9999537652	1.471276757
-1.026165804	0.8651524742	0.9999537652	-1.0986846103
6.3075231393	0.4860483568	0.9999537652	6.908559683
-1.0299520986	0.9189704341	0.9999537652	1.4105733886
-2.5871290907	0.5001526277	0.9999537652	1.0010497037
0	0	0	14.7271569203
-1.0440298828	0.7467999051	0.9999537652	1.2748936662
1.2244062091	0.4469566494	0.9999537652	1.2161760926
1.0998914838	0.4759839691	0.9999537652	1.2223179451
2.7933974408	0.6948713406	0.9999537652	-5.3589986948
1.3853136987	0.2104107263	0.9999537652	1.2120683062
-2.956038745	0.3205445351	0.9999537652	1.2407431382
0	0	0	-1.1248877899
0	0	0	2.9985540909
1.1004172696	0.7633299299	0.9999537652	1.3076031106
-1.4848615221	0.0895956076	0.9999537652	-1.0953182414
-1.0211615507	0.921903176	0.9999537652	1.2048793088
-1.0578723963	0.8950454676	0.9999537652	-1.1996932824
-1.5875118025	0.7201624284	0.9999537652	2.996475627
1.0020230375	0.9880315615	0.9999537652	1.3560981859
1.9109421781	0.1038210115	0.9999537652	-1.1149624023
1.2472630397	0.1768121081	0.9999537652	1.0691555635
-1.4882495476	0.3279660734	0.9999537652	-1.0604946197
-1.3441341749	0.0403719052	0.9999537652	-1.0737421053
1.0930933484	0.7610281373	0.9999537652	1.2564317896
1.1153938333	0.5645442986	0.9999537652	-1.1471673829
-3.8398244263	0.1426434157	0.9999537652	-1.6150741373
-1.0527716198	0.7810666485	0.9999537652	1.3010135507
-1.0694171877	0.7207843454	0.9999537652	1.0008115873
1.1200130315	0.7178601348	0.9999537652	1.2497045511
4.0084683418	0.0108545582	0.9999537652	-1.1303199936
4.5505976667	0.5559322269	0.9999537652	0
-1.0931432807	0.6353510769	0.9999537652	1.1612153826
0	0	0	-1.0338466263
1.0403528193	0.7881957561	0.9999537652	1.1473384245
3.9737398722	0.006509	0.9999537652	1.4776466071
-1.028698786	0.8823688146	0.9999537652	1.0824540407
-1.1000448055	0.5056013103	0.9999537652	-1.0263852321
1.1289026269	0.3134861446	0.9999537652	1.0666040466
2.6183639153	0.1357369211	0.9999537652	1.4132245309
1.1762411247	0.247933662	0.9999537652	1.1687659061
1.1844918384	0.3708548278	0.9999537652	1.0484333805
1.0497214602	0.7910595133	0.9999537652	-1.1124285129
1.815284058	0.3121519927	0.9999537652	1.1897884937
-1.0188647301	0.9230170338	0.9999537652	1.4848968124
6.3075231393	0.4860366635	0.9999537652	0
1.0593319107	0.6710588382	0.9999537652	1.0339039494

1.4673678633	0.0900579715	0.9999537652	1.0516310446
-1.1961569164	0.3289804901	0.9999537652	-1.1002693383
0	0	0	2.9985540909
-1.2520757133	0.7407098251	0.9999537652	1.2398837679
2.7933974408	0.6948713406	0.9999537652	6.9133480437
1.0469036191	0.9171043411	0.9999537652	1.0650049364
-1.1442445428	0.5576090232	0.9999537652	1.0743030847
1.3869904012	0.44401117	0.9999537652	1.0901934502
1.4922545969	0.0236609616	0.9999537652	1.3611385554
6.3075231393	0.4860250059	0.9999537652	0
1.1469777922	0.3037710611	0.9999537652	-1.0891166814
-1.014058902	0.9221479262	0.9999537652	-1.2977693598
-1.9778106374	0.0339813794	0.9999537652	-1.5076686263
-1.7379985875	0.4182179352	0.9999537652	-1.8659789582
7.0692834839	0.00156	0.9999537652	-1.0685193139
-1.3950582798	0.0732387555	0.9999537652	-1.142810852
-1.0574046063	0.842697829	0.9999537652	-1.0429569403
-1.1545729319	0.396872709	0.9999537652	-1.0676728044
1.0477127772	0.7469147306	0.9999537652	-1.2437031779
-1.2945287741	0.1500995226	0.9999537652	-1.3494181631
0	0	0	2.996475627
-1.0625772242	0.8570383349	0.9999537652	-1.4208519446
1.093033967	0.7004447746	0.9999537652	1.1511588411
-1.0127601346	0.9849704122	0.9999537652	1.4392443705
-1.2105448354	0.628782535	0.9999537652	1.0763998677
34.3811030879	0.1695345472	0.9999537652	-1.2057326182
6.3075231393	0.4860133837	0.9999537652	0
-1.1609777944	0.5414065951	0.9999537652	-1.1547648734
-1.504262006	0.5482599376	0.9999537652	-1.230881042
-1.1582067332	0.3579625886	0.9999537652	-1.0510331066
0	0	0	2.9985540909
0	0	0	4.9527501949
-1.7955716613	0.0978281245	0.9999537652	-1.0099746571
-2.6334517422	0.2822155674	0.9999537652	1.4854894758
1.0326847566	0.8992595069	0.9999537652	1.039177202
-1.0466032624	0.7933392625	0.9999537652	-1.1075571388
1.0766281756	0.7603579762	0.9999537652	1.0273873026
1.9493977999	0.0009916	0.9999537652	-1.0169382921
1.2782678456	0.604721955	0.9999537652	-1.1385829569
-1.2306946963	0.359160313	0.9999537652	1.2016520959
-1.090368761	0.5389862364	0.9999537652	-1.2888330094
-1.0906176713	0.8531565178	0.9999537652	-1.2954034602
-1.136183371	0.8057549443	0.9999537652	-2.1111702588
1.5211587958	0.0523829531	0.9999537652	-1.070741648
1.4238667314	0.6195250225	0.9999537652	1.2047455501
-1.2389914222	0.6635972442	0.9999537652	1.8306186492
1.2619151399	0.3652198033	0.9999537652	-1.5168094574
1.0252520669	0.8760344029	0.9999537652	-1.5150083371
-1.1512781114	0.7854255457	0.9999537652	1.0544109503

-1.1794425288	0.4031272578	0.9999537652	-1.1037921198
-1.0312044602	0.9596472616	0.9999537652	1.589769722
1.0842093679	0.5412480899	0.9999537652	-1.4272870349
-2.7335321369	0.2490565315	0.9999537652	-1.3380993941
-1.6073007454	0.0202466001	0.9999537652	-1.3506333614
1.3495521868	0.0874030743	0.9999537652	-1.1286733321
-1.2450467244	0.0947016616	0.9999537652	-1.2368045235
1.2659104578	0.4785111008	0.9999537652	1.008280716
-1.0108515467	0.9508273126	0.9999537652	-1.1431987952
8.064174035	0.4238717721	0.9999537652	2.8664963163
-1.1865676628	0.5690576636	0.9999537652	1.3496298223
-1.1300516051	0.6663988981	0.9999537652	-1.2363877643
1.0878907018	0.5982231518	0.9999537652	-1.3063999561
-15.9195842901	0.1126921344	0.9999537652	-1.2400060274
1.2234935363	0.2381893954	0.9999537652	-1.1656804666
-1.7534510083	0.0213841044	0.9999537652	-1.4211325518
0	0	0	-5.3583704783
-4.082259813	0.0865030055	0.9999537652	1.8579562448
1.2753356422	0.5434358999	0.9999537652	1.5756519876
1.1521671675	0.3673954124	0.9999537652	1.1350579548
-1.0555448873	0.9030301231	0.9999537652	-1.1726476675
-1.4779519502	0.2659872162	0.9999537652	-1.5241488866
1.0203497736	0.9264745777	0.9999537652	-1.1535949179
1.2652283139	0.2289003875	0.9999537652	-1.2572718305
-1.3063736302	0.3626238901	0.9999537652	-1.0449589771
-1.1787484764	0.4365212228	0.9999537652	-1.6772522202
2.7957660672	0.7054620531	0.9999537652	-3.384595918
-1.573893529	0.2356637856	0.9999537652	-1.2586486911
1.2753463182	0.5162378043	0.9999537652	-1.714471873
1.3956459719	0.1865325055	0.9999537652	-1.1059237123
-1.0245499429	0.8788103643	0.9999537652	-1.0248500214
-1.4822156075	0.2061356705	0.9999537652	-2.5095924535
1.597327124	0.1280737435	0.9999537652	1.0912384513
-1.8640511245	0.1065080695	0.9999537652	-1.9005143595
2.7957660672	0.7054644074	0.9999537652	-1.3480816769
-1.4512821826	0.2370805236	0.9999537652	1.2932948092
2.4268643346	0.3294885846	0.9999537652	-3.1966011202
1.0950595181	0.4211459173	0.9999537652	-1.043473629
1.2910557943	0.704145754	0.9999537652	1.5870215946
-1.4352458527	0.2366549231	0.9999537652	-1.1244734224
1.0119574961	0.9473068488	0.9999537652	1.0034723554
1.0537087738	0.8868394946	0.9999537652	1.1580671904
1.4611545842	0.2881040191	0.9999537652	1.4529814156
0	0	0	2.9940379764
-1.2636369764	0.1957768282	0.9999537652	-1.0192254213
2.5744732225	0.3974207695	0.9999537652	12.7732027325
1.0639635919	0.7892627316	0.9999537652	-1.0134503259
27.993011587	0.2236144496	0.9999537652	15.9659633994
-1.1502960954	0.4127922158	0.9999537652	1.4008042313

-1.2503735004	0.3271451624	0.9999537652	1.0307693933
1.846361394	0.5888000048	0.9999537652	3.7228836277
1.1025078533	0.6905610816	0.9999537652	1.2033064354
2.7933974408	0.6948713406	0.9999537652	1.6002511223
-1.4188646248	0.0622726091	0.9999537652	-1.0351980454
-1.1166060857	0.7845538891	0.9999537652	1.0633590063
1.2268003525	0.2413132626	0.9999537652	1.098599093
-1.8115085429	0.2692387521	0.9999537652	-1.0185380321
2.7933974408	0.6948713406	0.9999537652	-3.3865434896
-1.7495393231	0.2946621704	0.9999537652	2.0660460667
1.1111689845	0.7746968261	0.9999537652	1.2874037577
1.0498311458	0.8306714879	0.9999537652	1.0788718564
-1.2877681807	0.3773172709	0.9999537652	-1.4292611129
2.1181846932	0.2873564908	0.9999537652	1.0974312001
-3.810840035	0.0393067864	0.9999537652	-1.0118063622
6.3075231393	0.485991399	0.9999537652	-5.3589986948
-1.1253908775	0.7699914311	0.9999537652	1.2289396219
2.7933974408	0.6948713406	0.9999537652	-2.9680235826
0	0	0	0
-1.507422654	0.2480063086	0.9999537652	1.1764844331
-1.1790661357	0.4941561938	0.9999537652	-1.0048754531
1.0763637626	0.7989155489	0.9999537652	1.2006482502
-4.1032936321	0.1311326696	0.9999537652	1.3191741898
1.0903220495	0.7194056375	0.9999537652	1.3785947705
1.6413775565	0.0435328636	0.9999537652	1.1577567294
2.7933974408	0.6948713406	0.9999537652	-2.2219162835
-1.2891893615	0.1195691436	0.9999537652	-1.167270675
1.3070686944	0.1888778973	0.9999537652	1.0473379371
-1.7985323208	0.1473844049	0.9999537652	-1.2556088491
1.3594099105	0.7971167452	0.9999537652	1.2518582418
0	0	0	2.996475627
2.0903298609	0.5114050435	0.9999537652	1.0987070368
-1.576892299	0.0477677988	0.9999537652	1.1261796796
1.3395236905	0.1215756857	0.9999537652	1.2017641724
-1.8528058048	0.3376652504	0.9999537652	1.4371102393
-1.3309434275	0.3375674134	0.9999537652	-1.1984855355
-1.0145301364	0.9514000841	0.9999537652	-1.0186730775
2.7933974408	0.6948713406	0.9999537652	-2.9683711557
-2.5871290907	0.5001433169	0.9999537652	63.4727107264
2.2818443854	0.1549935697	0.9999537652	-1.4331129499
-1.1401584779	0.5056831895	0.9999537652	-1.2342067931
-1.1152158598	0.7252519352	0.9999537652	1.0542313768
1.2244113984	0.2244684982	0.9999537652	-1.167532326
1.146608954	0.6076381521	0.9999537652	1.0764068009
-1.0009808156	0.992937265	0.9999537652	1.1503546051
1.0565915369	0.9283513222	0.9999537652	-1.0393789722
1.9280222804	0.3895230563	0.9999537652	-1.069803044
-1.926212649	0.1054881872	0.9999537652	-1.1839994128
-1.0279795459	0.8967156292	0.9999537652	1.0064885048

-1.3942756209	0.3552817278	0.9999537652	1.6896352024
1.0269306994	0.9011584917	0.9999537652	-1.0841658469
1.0217259552	0.9233721563	0.9999537652	1.2499588597
1.2925565279	0.0918533018	0.9999537652	1.1228198908
3.3414463545	0.157992645	0.9999537652	-1.3978714673
-1.0289093964	0.9379985024	0.9999537652	1.2121282152
1.0522322073	0.8774307068	0.9999537652	-1.0154986484
1.2731181768	0.6677270521	0.9999537652	1.1245963244
2.7933974408	0.6948713406	0.9999537652	1.3564862013
1.1664424503	0.8015890884	0.9999537652	1.4223160585
2.7933974408	0.6948713406	0.9999537652	0
-1.0545936768	0.8933447844	0.9999537652	1.0815742139
1.044334363	0.8636652493	0.9999537652	-1.0195416024
1.3307714562	0.5811271186	0.9999537652	-1.1383960977
-1.3401808496	0.2027921056	0.9999537652	-1.2541169899
1.0754769463	0.7144143202	0.9999537652	1.5372642173
9.8205505243	0.3807094839	0.9999537652	1.9445634487
-1.1218098909	0.5510443206	0.9999537652	-1.1723859301
1.0086997665	0.9452979865	0.9999537652	1.0878462848
-1.168257259	0.3873156049	0.9999537652	-1.0272218532
1.1568397069	0.3320958117	0.9999537652	-1.1572145347
-4.815549291	0.1726839831	0.9999537652	12.7909013375
1.0155688328	0.9216307978	0.9999537652	1.0018024777
-1.0763806691	0.7034134232	0.9999537652	1.2490382867
1.1113887432	0.5961317206	0.9999537652	1.0301623611
-3.1138217608	0.2426045889	0.9999537652	-1.0951294003
-1.2360491754	0.3323501145	0.9999537652	1.235598801
1.0630168449	0.6667778791	0.9999537652	-1.5330591469
-1.3897388782	0.1920912332	0.9999537652	-1.1992680067
1.1473113052	0.4128970649	0.9999537652	1.3837522387
-1.1570244643	0.411176445	0.9999537652	-1.0716331651
-1.1685311166	0.8553789326	0.9999537652	-1.8145313785
1.2110976757	0.469937239	0.9999537652	1.4937060641
1.0596691157	0.9162025465	0.9999537652	1.0527870164
1.2037990876	0.6685036296	0.9999537652	1.0697707471
2.193145556	0.2487130696	0.9999537652	-1.5933224346
-3.1061883819	0.0592126439	0.9999537652	1.8941069248
-1.0020447937	0.9928147199	0.9999537652	-1.1896693447
-1.22580301	0.2650886501	0.9999537652	-1.4632960373
9.8205505243	0.3806891435	0.9999537652	1.3244668493
-1.1289485505	0.5936017327	0.9999537652	-1.4951366118
1.1006672117	0.8627356357	0.9999537652	1.0210230038
1.4808896861	0.3575772774	0.9999537652	-1.2605642381
0	0	0	2.9985540909
-1.0574320632	0.9501087726	0.9999537652	1.9327676833
-2.0033956173	0.4335773407	0.9999537652	-1.2571784464
0	0	0	2.996475627
-1.6247284074	0.4493301657	0.9999537652	1.2471219592
1.0497795381	0.711217781	0.9999537652	-1.0045056863

-1.3534502364	0.1832207657	0.9999537652	-1.7148677428
-1.4414944684	0.4705294826	0.9999537652	-1.2889369249
-1.0364095484	0.7905543985	0.9999537652	-1.1901727524
2.8246852305	0.2266224563	0.9999537652	-1.4682504234
-1.5432214039	0.2661914512	0.9999537652	-1.0957832685
-1.0228241935	0.9602350711	0.9999537652	-1.1700961977
15.0880352532	0.2955018616	0.9999537652	1.591370391
1.2658068701	0.6067999709	0.9999537652	-1.0240583004
1.253220623	0.8068932382	0.9999537652	-1.4512547171
-1.5875118025	0.7200732777	0.9999537652	0
-1.4448063006	0.2143597854	0.9999537652	-1.1081185463
2.1143085312	0.1114830517	0.9999537652	-1.0479589688
-4.5982967254	0.0124434125	0.9999537652	-1.9999284011
1.0810578719	0.678556242	0.9999537652	-1.0880880289
-2.0320138873	0.0930608594	0.9999537652	-1.0817072662
2.6954181122	0.2367403917	0.9999537652	1.1267022694
-2.5861438062	0.4675226193	0.9999537652	-1.6597328868
-1.3173381128	0.3391587294	0.9999537652	-1.125215004
-1.1447781567	0.9152681835	0.9999537652	-1.3811818004
1.0915963427	0.6802608014	0.9999537652	-1.1119809211
4.0453524072	0.2056884022	0.9999537652	1.1706814519
1.009463014	0.9792213211	0.9999537652	1.2086552941
1.3725069946	0.0832907945	0.9999537652	1.2569250149
1.1264410515	0.3561063146	0.9999537652	-1.0213636867
-2.0532004703	0.0154317799	0.9999537652	-1.0385191309
-1.7189596186	0.1058617469	0.9999537652	1.0076725025
-1.0976433342	0.6342565354	0.9999537652	-1.001185251
-1.8600491373	0.2257549986	0.9999537652	-1.1078871114
-1.212605086	0.3802659823	0.9999537652	-1.3068264348
1.0566204755	0.7478124161	0.9999537652	-1.0374470196
-1.2429321377	0.5164974185	0.9999537652	-1.3708072673
1.04061447	0.8736794877	0.9999537652	1.1447998
1.1035001105	0.676398495	0.9999537652	1.0605789544
-1.0719079882	0.5798726295	0.9999537652	1.1259718757
1.5699684466	0.5805343008	0.9999537652	1.3761875318
1.0085798906	0.9777182187	0.9999537652	-1.2486117439
-1.0407362297	0.8441974483	0.9999537652	1.7779445197
1.1460770273	0.37607337	0.9999537652	1.4457781222
1.0581484739	0.9223492423	0.9999537652	-1.7727302113
1.219049643	0.6885407647	0.9999537652	1.2266238482
1.1033131165	0.6314903479	0.9999537652	-1.1885843862
0	0	0	0
0	0	0	18.6465640159
0	0	0	2.996475627
-1.1007046215	0.8698864199	0.9999537652	-1.0681555454
-1.2497070439	0.6734562488	0.9999537652	1.7307122593
3.7966717237	0.2366051744	0.9999537652	1.4955638825
0	0	0	2.996475627
3.6149509319	0.1140712314	0.9999537652	1.1390495699

1.3588921917	0.7999455877	0.9999537652	1.9336600182
23.8818846228	0.2167826056	0.9999537652	-1.1425907537
0	0	0	2.0298295761
-12.9800088947	0.1122844628	0.9999537652	-2.6251364651
2.7933974408	0.6948713406	0.9999537652	1.590997316
-1.0475589205	0.7527919323	0.9999537652	1.3065869813
15.1008094413	0.2915777161	0.9999537652	1.0016199422
4.5505976667	0.5559322269	0.9999537652	-1.2372152196
1.3743046583	0.2409983979	0.9999537652	-1.304906384
-1.4112989661	0.3404876228	0.9999537652	1.0535612495
-1.0566429381	0.9038839394	0.9999537652	-1.1955905928
-1.3357204881	0.1896387441	0.9999537652	-1.1278623083
1.0823298706	0.7111396715	0.9999537652	1.0276174696
1.1621179655	0.5798447438	0.9999537652	1.3531114857
2.7933974408	0.6948713406	0.9999537652	0
1.091399163	0.4805486489	0.9999537652	1.0341130834
1.295601917	0.1753415098	0.9999537652	1.2500381549
1.092276421	0.8341616745	0.9999537652	1.1982824004
0	0	0	-1.5938156117
-1.6313316116	0.2422214045	0.9999537652	1.7472081796
1.5678164322	0.1556384442	0.9999537652	-1.0425779404
1.0605311687	0.8895960801	0.9999537652	-1.0490519171
30.8757261591	0.1832943425	0.9999537652	-1.4616280167
-1.2708533643	0.09547662	0.9999537652	-1.1013958298
8.064174035	0.4238704373	0.9999537652	1.068847327
-4.106901512	0.1170163158	0.9999537652	-1.9284645673
1.3279637572	0.2942346659	0.9999537652	1.0681631963
-1.5388920234	0.3158148164	0.9999537652	-1.2404363517
-1.038726441	0.844375877	0.9999537652	1.0094346068
1.0240877952	0.8961172321	0.9999537652	1.1803447211
1.3457288891	0.5101850067	0.9999537652	1.1600311244
1.2769191345	0.238090548	0.9999537652	1.1939839096
-1.061093121	0.9294961056	0.9999537652	-1.7783606855
-1.0808186081	0.6197637795	0.9999537652	1.0017925203
1.0391365057	0.8834706393	0.9999537652	1.1891245151
3.8642518105	0.1099984244	0.9999537652	-1.1570571525
-1.0986633164	0.7169141894	0.9999537652	1.3295985143
1.0145970138	0.9398449132	0.9999537652	1.4191004167
1.447287437	0.1674448088	0.9999537652	1.3007438884
-1.0461188494	0.7576998821	0.9999537652	-1.2173661349
-1.0308384324	0.9014046532	0.9999537652	-1.2872956686
1.2052118164	0.34705998	0.9999537652	-1.0732024144
1.2013528007	0.7147127837	0.9999537652	-1.218964211
-1.0220191719	0.8809019342	0.9999537652	1.0936175418
1.1706663721	0.822718875	0.9999537652	1.1600958952
-3.6992054516	0.044078034	0.9999537652	-1.3139186076
1.3960272491	0.6396304397	0.9999537652	1.0849152546
-1.1678729089	0.2035507808	0.9999537652	-1.2169606565
-1.004428369	0.9890608458	0.9999537652	1.1243694001

11.5864583426	0.3448419367	0.9999537652	1.6497435219
0	0	0	2.9988886918
1.1106251637	0.577611259	0.9999537652	1.0590561925
1.2841195985	0.183965972	0.9999537652	1.6647317967
-1.8333841838	0.001901	0.9999537652	-1.2619277852
8.064174035	0.4238691023	0.9999537652	-2.1280158161
-1.076467528	0.7579663242	0.9999537652	-1.0346836915
-1.0018529039	0.9940583616	0.9999537652	1.1077664645
3.5489539037	0.2511918332	0.9999537652	1.2598894344
1.0387687032	0.7650831154	0.9999537652	1.3689652967
0	0	0	0
3.5960636654	0.1474944966	0.9999537652	-1.2953919252
1.1552997158	0.4348268248	0.9999537652	1.1014453572
2.7933974408	0.6948713406	0.9999537652	22.5383525416
1.0737250852	0.8693777771	0.9999537652	-1.2860626306
-1.4077513013	0.5501177934	0.9999537652	1.4508492545
-1.1848011449	0.3829665343	0.9999537652	1.5054409163
-1.0105176837	0.9686074788	0.9999537652	1.1383936692
-1.1137354681	0.6002069911	0.9999537652	-1.2740358222
1.048251778	0.7563389677	0.9999537652	-1.0673659507
1.4356941257	0.2367973614	0.9999537652	1.0465092838
-1.006733681	0.9782568669	0.9999537652	1.128710771
-1.5875118025	0.7199841812	0.9999537652	1.2408817092
-1.0403101673	0.8408340842	0.9999537652	-1.0297007448
1.1614847191	0.3197575454	0.9999537652	1.4621666189
1.0718891308	0.7408486934	0.9999537652	1.0121009571
-1.0589827385	0.7852811984	0.9999537652	1.0355300217
-2.6323048902	0.2848454558	0.9999537652	16.6806485101
0	0	0	2.9988886918
-1.7230380909	0.4807061583	0.9999537652	-1.0084773122
1.1256551335	0.8550518516	0.9999537652	-1.1318456363
-1.25597146	0.7318578767	0.9999537652	-2.0695691574
1.0339887618	0.9113568278	0.9999537652	-1.1720042837
1.1220972931	0.9044459033	0.9999537652	-1.4815080994
1.7063326825	0.2289866938	0.9999537652	-1.2434072884
1.2384773505	0.3311233539	0.9999537652	-1.1141726526
-1.7116772754	0.2997934832	0.9999537652	-1.3907876095
0	0	0	6.908559683
-1.1168538378	0.6458542468	0.9999537652	1.1630421116
1.0958696246	0.5045933177	0.9999537652	-1.077447239
-1.1488518303	0.6360611566	0.9999537652	1.4926264968
-1.3916562724	0.6735294436	0.9999537652	-1.2614130545
-1.6939279844	0.3358654739	0.9999537652	-1.3201193671
-1.6288497936	0.3018033233	0.9999537652	1.1704511594
0	0	0	0
-1.1108685222	0.7869336239	0.9999537652	-1.7525097836
1.1171887656	0.5243188342	0.9999537652	1.0577120806
2.7933974408	0.6948713406	0.9999537652	-2.1742127776
-1.0949580351	0.7514253296	0.9999537652	-1.0930242284

-1.5986721877	0.2447369266	0.9999537652	1.3741257856
1.1598292034	0.7461209495	0.9999537652	-1.0312306261
1.1798769658	0.5765123279	0.9999537652	1.2333945589
0	0	0	1.2400291296
2.8186104398	0.3599094617	0.9999537652	-1.4681855619
1.2999552563	0.2689069769	0.9999537652	1.2473603292
-1.5268522869	0.3929657622	0.9999537652	1.1758749769
4.6478057571	0.0671942008	0.9999537652	-1.1850833597
2.0696758576	0.1134780009	0.9999537652	1.3426877329
-1.5250111891	0.4794794888	0.9999537652	1.2031505617
1.4410568594	0.4982444019	0.9999537652	-1.2524825119
1.5130423174	0.6605360433	0.9999537652	1.5332372248
18.9318466825	0.2781659383	0.9999537652	1.3519637633
-1.9819824477	0.0276984416	0.9999537652	1.2745666404
-2.5350664449	0.2508352746	0.9999537652	-1.4082032739
-1.2104022412	0.633281577	0.9999537652	1.0260595922
1.8339360555	0.4052989868	0.9999537652	-1.3066863121
1.0308307773	0.8690367741	0.9999537652	1.12602269
-1.4783387508	0.1034739872	0.9999537652	1.0171727476
1.029151863	0.8060412711	0.9999537652	1.1374080322
-1.0854731597	0.6068533113	0.9999537652	1.0443905908
1.1220094372	0.5633418191	0.9999537652	1.0281111761
1.1023960321	0.6689542414	0.9999537652	1.0469897944
1.0063516441	0.9778275755	0.9999537652	-1.0594502797
-1.7350961946	0.173713048	0.9999537652	-1.3416256734
1.0877650195	0.6873262057	0.9999537652	1.0930709454
-1.1693119757	0.8274881304	0.9999537652	5.1102118714
-1.1030763619	0.4414850722	0.9999537652	1.1376795205
-1.0304384993	0.8969704243	0.9999537652	1.4307888251
1.0412174444	0.7741667929	0.9999537652	1.0349889941
2.7933974408	0.6948713406	0.9999537652	1.6084809026
1.344859198	0.3329254107	0.9999537652	1.0947777858
1.4739980805	0.0914015398	0.9999537652	1.3704533304
1.2986471813	0.4244327623	0.9999537652	1.183499942
1.0635879733	0.7428145126	0.9999537652	-1.2658071704
-1.1111315061	0.6326504051	0.9999537652	1.2519081987
-1.0942315875	0.8474463318	0.9999537652	1.0173729967
2.7933974408	0.6948713406	0.9999537652	0
1.8455489546	0.5921514806	0.9999537652	-2.1287636007
-1.0796202809	0.714464129	0.9999537652	1.3863297551
-1.0418464933	0.9422936924	0.9999537652	1.1533206359
0	0	0	6.908559683
-1.1060987006	0.6297382056	0.9999537652	1.0313370881
2.0381854033	0.4031762039	0.9999537652	1.2770715033
1.0578565016	0.694881995	0.9999537652	1.2905866165
1.1641828182	0.345711248	0.9999537652	1.2492547373
1.0999313655	0.8886048549	0.9999537652	1.1092933382
2.7933974408	0.6948713406	0.9999537652	1.4153476291
-1.270073807	0.1682105864	0.9999537652	1.086495382

-1.3993360498	0.414411543	0.9999537652	-1.0181367411
-1.0821537024	0.7728961148	0.9999537652	-1.2731019667
2.7933974408	0.6948713406	0.9999537652	2.996475627
-1.1053878178	0.6114781005	0.9999537652	1.4257087606
0	0	0	9.7116702472
1.0706404744	0.6042819245	0.9999537652	1.4373529563
-1.4184453654	0.589065749	0.9999537652	-1.1918339622
-1.0951850746	0.6701207609	0.9999537652	-1.0761290116
-1.1645364823	0.4456261933	0.9999537652	1.2566704155
1.1190584955	0.7579520687	0.9999537652	1.0282292718
-1.0708660819	0.7392447224	0.9999537652	1.2156986192
0	0	0	8.8639046058
1.9639779619	0.052618044	0.9999537652	-1.3268664848
-1.1455442151	0.370616596	0.9999537652	1.0370579544
2.7933974408	0.6948713406	0.9999537652	-1.1248877899
-1.0465958371	0.7614841456	0.9999537652	1.5775453375
1.5107816438	0.5277967288	0.9999537652	-1.6188562449
-1.0941791399	0.8326624322	0.9999537652	1.153202049
0	0	0	2.9985540909
-1.2551248324	0.7718555006	0.9999537652	3.5599780551
-1.0888109777	0.64212423	0.9999537652	1.1411285374
2.7933974408	0.6948713406	0.9999537652	-5.3589986948
-1.2356160979	0.5925386194	0.9999537652	1.3608233581
40.1750348981	0.1540444008	0.9999537652	-1.043334644
1.1616785196	0.3334451874	0.9999537652	1.4277516352
1.5850121741	0.614255278	0.9999537652	-1.103904595
1.449543962	0.2689705894	0.9999537652	1.3348814661
-1.8291042549	0.3288463316	0.9999537652	1.7973499494
1.1512427029	0.402377106	0.9999537652	1.2196166457
-1.0404439214	0.8601749669	0.9999537652	1.1688052862
-1.0785200063	0.7616358561	0.9999537652	1.1177868022
1.0129847243	0.972796173	0.9999537652	-1.7278744164
2.8258092867	0.0936218368	0.9999537652	-1.0961030106
8.064174035	0.4238677672	0.9999537652	9.7116702472
0	0	0	2.996475627
1.1606463124	0.7515893335	0.9999537652	1.296751669
1.1781241293	0.6326897435	0.9999537652	1.3359680858
1.4584356027	0.0670056386	0.9999537652	1.1707478655
-1.1611368092	0.634725221	0.9999537652	1.4042658999
-1.2481294663	0.377436053	0.9999537652	1.1439442108
0	0	0	-2.5811097865
2.5553461028	0.2410261947	0.9999537652	-1.012946855
-1.3909491861	0.3568755199	0.9999537652	-1.064948932
-1.1006122314	0.9019770859	0.9999537652	-1.5460290334
1.6550710735	0.1780854368	0.9999537652	1.3611868973
-1.0857729511	0.5335592033	0.9999537652	1.0324929133
1.1158299481	0.929746046	0.9999537652	9.7116702472
-1.6674095463	0.621582587	0.9999537652	-3.2376885507
-1.3059708017	0.4653832042	0.9999537652	-1.2883867929

3.1603610665	0.1649230892	0.9999537652	-1.1286538667
4.5505976667	0.5559322269	0.9999537652	0
0	0	0	-2.2835167025
-1.1482113318	0.4245226284	0.9999537652	1.1902838709
1.7745951127	0.5409014838	0.9999537652	1.8687602882
1.0833861825	0.5729949902	0.9999537652	1.2357168875
1.4157529189	0.286445013	0.9999537652	-1.2984561635
20.3702769952	0.2388209961	0.9999537652	-1.1441751338
8.064174035	0.423866432	0.9999537652	2.7698355275
0	0	0	1.3590242562
1.5455319377	0.1539990451	0.9999537652	1.3426714575
1.014581964	0.9760622325	0.9999537652	-1.3548721631
-1.4167537021	0.2264839923	0.9999537652	-1.2412346122
2.7933974408	0.6948713406	0.9999537652	0
1.147612444	0.5407701274	0.9999537652	-1.2217589246
-1.0820588842	0.7373639026	0.9999537652	1.2789637166
-1.2829786572	0.1133567415	0.9999537652	1.0388610836
1.2764410314	0.2924118055	0.9999537652	1.028952031
1.1532607844	0.4237974371	0.9999537652	-1.0267349849
-1.4525887343	0.6066334026	0.9999537652	1.6794517025
-1.0940903919	0.8717737776	0.9999537652	1.1612416843
-1.2010406794	0.3369651085	0.9999537652	1.0722677181
-1.0953113997	0.5711100319	0.9999537652	-1.1010527962
-1.0411448639	0.9135167724	0.9999537652	1.0384366776
1.1111013903	0.5218915104	0.9999537652	1.1207263412
2.3306483975	0.4663384686	0.9999537652	-1.1609385688
1.0893367869	0.8043651126	0.9999537652	-1.1202346993
-1.0158906208	0.9314642622	0.9999537652	1.0009789548
2.7933974408	0.6948713406	0.9999537652	0
1.4071312909	0.1778004688	0.9999537652	1.3520653248
-1.1754324206	0.4895897462	0.9999537652	-1.3254583465
1.1636085994	0.4726357022	0.9999537652	-1.1405278035
0	0	0	8.8639046058
-1.0708192035	0.7824938013	0.9999537652	1.1936270727
1.1482020948	0.7418551716	0.9999537652	-1.0893265677
1.6801292393	0.3282897238	0.9999537652	-1.1137192611
-1.5875118025	0.7198951394	0.9999537652	-1.7599945182
-1.1119337941	0.6866871992	0.9999537652	-1.1963869325
-1.0901170013	0.6643147754	0.9999537652	1.0783336115
3.8622037496	0.6195567277	0.9999537652	0
-1.2523216303	0.2408153777	0.9999537652	-1.1756772207
-1.2151457163	0.6220485729	0.9999537652	1.2626933414
1.2344006896	0.1573453413	0.9999537652	-1.1062185136
-1.0126987429	0.9216957817	0.9999537652	-1.0140335333
-3.1189360224	0.2743918909	0.9999537652	1.9436832203
-1.4219873594	0.2320822619	0.9999537652	1.0757511929
2.4587508136	0.2702720672	0.9999537652	1.3541249348
-6.9764533263	0.2592694791	0.9999537652	-1.4669967683
1.0207027716	0.8695666741	0.9999537652	1.2182628762

-1.1974527407	0.257502758	0.9999537652	-1.0550211508
-1.6046546692	0.260603418	0.9999537652	-1.2231446266
-1.0881591482	0.7915221762	0.9999537652	-1.2146980268
1.0089010158	0.9591236466	0.9999537652	-1.0730989171
1.156844776	0.4347248191	0.9999537652	1.317456715
1.0585140261	0.6394322503	0.9999537652	-1.132134325
-1.0605621337	0.8252935405	0.9999537652	-1.0463899295
1.3229080008	0.1988401024	0.9999537652	-1.1463205742
-1.2167526382	0.3409430204	0.9999537652	-1.0698093515
1.1270659728	0.5478299287	0.9999537652	-1.0889557914
-1.3928314061	0.1648584757	0.9999537652	-1.3150453135
1.3016944098	0.4900993804	0.9999537652	-1.0036472585
2.6411140541	0.2007194274	0.9999537652	-1.0087487564
2.742474689	0.3003314193	0.9999537652	-1.039502375
-1.5347274833	0.5806692429	0.9999537652	-1.7447180438
1.0606078301	0.6295335762	0.9999537652	1.1318091391
-1.4642045296	0.4189758615	0.9999537652	-1.3350390061
1.3711214614	0.0726913575	0.9999537652	1.0357712178
1.0822804758	0.7437741173	0.9999537652	-1.1894510845
1.0158628881	0.9716510127	0.9999537652	-1.2902916507
0	0	0	2.996475627
1.0185610549	0.952371563	0.9999537652	1.0440513158
1.9248811335	0.3952618035	0.9999537652	-1.1166512338
1.5910953944	0.2540128413	0.9999537652	-1.0754604452
1.1110392875	0.6047586363	0.9999537652	-1.5791252518
-1.0374819907	0.8524690098	0.9999537652	-1.0081027032
-2.6433596742	0.1901298008	0.9999537652	-1.0722259357
-1.2251734364	0.5974753695	0.9999537652	1.3124962221
1.9990747151	0.074391473	0.9999537652	-1.5719257782
2.1100562021	0.31657602	0.9999537652	-1.7745242306
-1.371793711	0.2890631759	0.9999537652	-1.5792661484
1.4571151306	0.3543625551	0.9999537652	1.0882451886
-1.435761442	0.3168623884	0.9999537652	1.0579309345
-1.0514662236	0.8406139757	0.9999537652	-1.4865846104
-1.1652153337	0.5891544167	0.9999537652	-1.1542236544
-1.2896983043	0.3641559989	0.9999537652	1.0663827807
1.0598994939	0.8207454928	0.9999537652	1.1549746327
2.619441909	0.133660075	0.9999537652	-1.522993704
1.6467991421	0.0519032617	0.9999537652	-1.6107934925
-1.0761052163	0.8648282534	0.9999537652	1.2490906314
-1.2008401404	0.4275212993	0.9999537652	1.1972164375
1.2559979691	0.3943660374	0.9999537652	1.0139152933
-1.1165524262	0.7681066337	0.9999537652	1.1168533891
-1.1271921681	0.514220206	0.9999537652	-1.6047619248
-1.0565061249	0.8673474745	0.9999537652	-1.1097224503
-1.1069002784	0.7019939324	0.9999537652	1.1125646572
-1.1103196421	0.5089352844	0.9999537652	-1.1768202822
-1.3031004414	0.3674264822	0.9999537652	-1.2454695162
1.1284049452	0.5012112146	0.9999537652	1.112108123

-1.0887279855	0.4501810693	0.9999537652	-1.059544632
-1.1253818319	0.7781121163	0.9999537652	-1.7571611943
1.1022331828	0.7156102858	0.9999537652	1.3137723608
1.2862555804	0.5083174217	0.9999537652	1.1236804887
-1.2641819991	0.4794356482	0.9999537652	1.1259524955
1.0152567708	0.9379784134	0.9999537652	-1.1091810613
1.7065721245	0.2107842254	0.9999537652	1.014412612
2.1626092017	0.1210414063	0.9999537652	-1.4863113326
1.1066835057	0.70311146	0.9999537652	-1.5253353008
0	0	0	2.996475627
1.0040836887	0.993035111	0.9999537652	1.3289914258
1.4506359903	0.0564139672	0.9999537652	1.0269653906
1.788517704	0.1948077929	0.9999537652	1.1748799717
1.425492918	0.2660452955	0.9999537652	-1.3747487991
1.8890496501	0.3349073956	0.9999537652	-1.5939286483
-2.174440844	0.0636193077	0.9999537652	1.0999253806
-1.1929460989	0.4619645944	0.9999537652	-1.1398616187
1.3043818864	0.2632416019	0.9999537652	1.2395197325
-1.5995099286	0.5378398129	0.9999537652	2.111849768
1.0230076711	0.9100952272	0.9999537652	1.0065284046
2.7933974408	0.6948713406	0.9999537652	-1.1250196696
57.2359413924	0.1117674303	0.9999537652	1.1248329925
-1.3081518616	0.3275169635	0.9999537652	-1.1499642333
1.2329064426	0.2620198184	0.9999537652	1.0104511416
-1.1877134683	0.207395582	0.9999537652	-1.0753494029
1.2158337748	0.4219218799	0.9999537652	-1.2006901719
-1.1087164534	0.4225259939	0.9999537652	-1.095960283
1.1585745804	0.5500519141	0.9999537652	1.0829819728
1.2037205439	0.5771569664	0.9999537652	1.0823152594
2.7933974408	0.6948713406	0.9999537652	-2.7895993146
1.3028917047	0.4170923226	0.9999537652	1.1420012663
1.2965726617	0.198338116	0.9999537652	1.7389445271
-3.1203910316	0.2340291776	0.9999537652	1.1343531566
-1.3295600488	0.1644345473	0.9999537652	1.021463623
-1.0451567036	0.8125759175	0.9999537652	-1.003083654
8.064174035	0.4238650967	0.9999537652	1.9445739043
1.2654175061	0.5989933662	0.9999537652	-1.2167237019
2.7933974408	0.6948713406	0.9999537652	0
-1.5334315236	0.1001742286	0.9999537652	-1.0387746769
1.1627056682	0.5680227559	0.9999537652	-1.510363508
-1.2354566191	0.4171502442	0.9999537652	-1.1854456149
3.8622037496	0.6195260146	0.9999537652	-11.020251207
2.056870118	0.1288674367	0.9999537652	1.054088584
3.8622037496	0.6194952821	0.9999537652	2.996475627
1.0829272869	0.6775905493	0.9999537652	-1.0442754885
-1.2360773048	0.3711161069	0.9999537652	-1.1148506659
-1.4699883509	0.1885375407	0.9999537652	1.0014873875
-1.134616971	0.6213710471	0.9999537652	-1.140872062
-1.0002388629	0.9991329261	0.9999537652	1.0443058412

9.8205505243	0.3806688837	0.9999537652	1.2594537582
-1.0749307647	0.7323675029	0.9999537652	1.1081541973
-1.0254490023	0.8837746619	0.9999537652	1.0826092289
-2.1307903719	0.4720123032	0.9999537652	1.4383632232
1.0745938652	0.9014687322	0.9999537652	1.6861441184
1.6019886574	0.2060696122	0.9999537652	1.0031742143
1.0676282763	0.7269570757	0.9999537652	-1.2976395421
1.1220972931	0.9043779706	0.9999537652	-2.571072022
1.2219253713	0.1750134259	0.9999537652	1.0899821364
-1.4000115989	0.6273829366	0.9999537652	-1.2516975794
-1.0511078004	0.7830639643	0.9999537652	1.0281125392
1.1538095722	0.6857883874	0.9999537652	-1.7411139195
1.0803905107	0.4817877791	0.9999537652	1.0145691859
1.7016447663	0.1432214776	0.9999537652	1.1615051618
1.0282705766	0.9421894502	0.9999537652	1.0663266754
-1.0212229596	0.8763574417	0.9999537652	-1.1896778076
18.6298281873	0.2553876104	0.9999537652	1.6121286294
-1.0851943957	0.6887850228	0.9999537652	-1.1639282742
-1.1457555741	0.9107288121	0.9999537652	-2.1244020028
1.1292505485	0.8261096965	0.9999537652	-1.7259376803
-1.6665793788	0.615338868	0.9999537652	3.3805014133
0	0	0	2.996475627
0	0	0	9.7116702472
-1.0118967847	0.9656726138	0.9999537652	-1.2001396971
-1.0212400919	0.9088694736	0.9999537652	-1.4505596806
-1.0492606289	0.7631740095	0.9999537652	-1.1369679014
-1.1289722653	0.3904933134	0.9999537652	-1.2431883019
2.7933974408	0.6948713406	0.9999537652	-4.9096724685
1.039301144	0.9050882099	0.9999537652	-1.4530997693
2.0346255682	0.4403503446	0.9999537652	1.7773414401
-1.3632719965	0.5614381347	0.9999537652	1.4719994882
-1.3162005453	0.0676402926	0.9999537652	-1.104455951
0	0	0	2.996475627
4.5505976667	0.5559322269	0.9999537652	0
-1.3410082413	0.2349450075	0.9999537652	1.0295440894
1.2107601343	0.2219062987	0.9999537652	1.0890781806
1.0676107444	0.7626809375	0.9999537652	-1.2174384006
-1.163602196	0.3108059748	0.9999537652	-2.0464238628
-3.0158565881	0.0779952364	0.9999537652	-2.1027358968
6.3075231393	0.4859694426	0.9999537652	0
1.0853991643	0.8004228275	0.9999537652	1.2472184753
0	0	0	6.908559683
0	0	0	2.996475627
1.2694197581	0.395852337	0.9999537652	1.1341074152
-1.538941015	0.4082459815	0.9999537652	-1.4374227175
1.1176897722	0.7958229846	0.9999537652	-1.5010016565
1.1432003108	0.5789067491	0.9999537652	-1.654661594
-1.0965268463	0.6409837436	0.9999537652	-1.1986188038
-1.3188875	0.4132715104	0.9999537652	-1.5751482835

1.0944487429	0.6634349848	0.9999537652	-1.3034694167
-1.2563315302	0.2230536694	0.9999537652	1.3754908429
1.8701557858	0.2822176734	0.9999537652	1.0824224094
-1.1890534012	0.3901858811	0.9999537652	1.0024089826
1.3801180903	0.155505533	0.9999537652	1.2888818387
2.7933974408	0.6948713406	0.9999537652	3.8529287492
0	0	0	-1.3028722362
1.3491087878	0.1331782059	0.9999537652	-1.1292991519
1.6163318864	0.369388035	0.9999537652	1.6232125136
-1.139089678	0.3955054731	0.9999537652	-1.0605908685
-1.3512997623	0.1668158066	0.9999537652	-1.305422755
2.8197560167	0.3544979278	0.9999537652	-2.2620268691
1.0055167866	0.9726019633	0.9999537652	1.0399116487
-1.5838701797	0.1344105548	0.9999537652	-1.2955667527
-1.0963620756	0.6582927364	0.9999537652	-1.091742223
1.3820692738	0.7413995037	0.9999537652	-1.3935735413
-1.4405954658	0.2710961575	0.9999537652	-1.7352578558
2.7933974408	0.6948713406	0.9999537652	0
1.3867633649	0.729366044	0.9999537652	1.1929411067
2.0885615936	0.5185910451	0.9999537652	-1.2016883181
-2.2048955514	0.009441	0.9999537652	1.3858998812
-1.2513614036	0.4594351232	0.9999537652	-1.032460741
0	0	0	22.5383525416
1.3024294952	0.0759416517	0.9999537652	1.1561718903
-1.215331919	0.4542432368	0.9999537652	-1.2181494227
-1.0050652613	0.9834027609	0.9999537652	-1.309677357
1.1800813261	0.3120312452	0.9999537652	1.1716308631
2.7957660672	0.7054667628	0.9999537652	1.0004741777
2.7933974408	0.6948713406	0.9999537652	8.8639046058
1.1164747255	0.4603069422	0.9999537652	-1.3054881918
-1.5693576097	0.0886871753	0.9999537652	1.2408328152
1.1626253702	0.5401018226	0.9999537652	-1.0416062523
-1.0428136733	0.9314271245	0.9999537652	1.1917166734
-2.4387448463	0.3485651706	0.9999537652	-9.4780054324
4.5544554512	0.5691373742	0.9999537652	4.7454169068
0	0	0	6.908559683
-1.0846416503	0.5092354065	0.9999537652	-1.372762448
8.064174035	0.4238637612	0.9999537652	-1.2237070081
-1.2154453731	0.1819855523	0.9999537652	-1.3384500142
1.1058106324	0.639797151	0.9999537652	1.1608341375
-1.2002678735	0.5020519077	0.9999537652	-1.5290373432
-1.2954797492	0.4721370518	0.9999537652	-1.3377156923
3.805366741	0.0556202137	0.9999537652	1.0959591624
-1.0688853093	0.8346067582	0.9999537652	-1.1842238291
2.2953539498	0.3493867201	0.9999537652	-1.6167032484
-1.1615123054	0.3507972791	0.9999537652	1.0846668876
1.3598134657	0.1325174384	0.9999537652	1.1251755294
1.1287258111	0.6271472623	0.9999537652	1.2080475268
-1.4726528313	0.1813130955	0.9999537652	-1.3478140877

-1.245744448	0.2994658374	0.9999537652	-1.1313370293
-1.1624006362	0.6690673847	0.9999537652	1.1124073272
-1.0221298433	0.900002509	0.9999537652	1.0651244454
-1.0085355311	0.9930350584	0.9999537652	1.4048704932
-1.4390360156	0.0315297573	0.9999537652	-1.297070476
-1.2590130964	0.1064008215	0.9999537652	1.3362064066
1.1026647921	0.8029550943	0.9999537652	-1.3211476307
1.0546895876	0.7099874082	0.9999537652	1.7778818184
-1.0576001765	0.8520942831	0.9999537652	1.0164603399
1.8357201582	0.2612683768	0.9999537652	-1.7597223057
16.8433158146	0.2735193915	0.9999537652	2.3074450707
-1.0012547714	0.9946659375	0.9999537652	-1.0237264886
-1.0272985149	0.8841848917	0.9999537652	-1.0186915418
-1.2518477289	0.2603834682	0.9999537652	-1.0387899273
0	0	0	0
8.064174035	0.4238624257	0.9999537652	2.996475627
15.0880352532	0.2955455083	0.9999537652	4.0583010578
-1.2755409506	0.2251135518	0.9999537652	1.3164769935
1.3732671858	0.3072769239	0.9999537652	-1.2683976535
-1.1758878956	0.530086972	0.9999537652	-1.0204649236
-1.0436077749	0.7399851693	0.9999537652	1.0327520313
1.6304649079	0.1664505331	0.9999537652	-1.0869018319
-1.3699107418	0.396262722	0.9999537652	-1.2493820533
-1.0920094712	0.864296452	0.9999537652	-1.0693685302
-2.587194901	0.5007129884	0.9999537652	12.7732027325
-1.0874839212	0.6490775161	0.9999537652	1.0698424135
-1.1684806514	0.3976482294	0.9999537652	1.2357540451
-1.0042266312	0.989734773	0.9999537652	1.0532066797
-1.2840992906	0.255428276	0.9999537652	-1.0630620986
1.2273488568	0.543381251	0.9999537652	1.042245821
1.1333548398	0.4740079546	0.9999537652	1.1585263912
-1.2418696748	0.4039874247	0.9999537652	1.0130335143
0	0	0	2.996475627
1.0035792769	0.9776690134	0.9999537652	1.0482762038
-1.1026914183	0.8465221463	0.9999537652	-1.5939521358
-1.586167124	0.7107512635	0.9999537652	-3.2324675423
0	0	0	-1.4868621173
1.1435785632	0.7491532359	0.9999537652	1.0357004166
-1.3475836462	0.1455669338	0.9999537652	-1.1640857245
1.1547207742	0.6000385449	0.9999537652	-1.1185952314
1.2168577454	0.5614103567	0.9999537652	1.1838092576
-1.1528061049	0.5195061817	0.9999537652	-1.0748267717
1.0446178272	0.7894504274	0.9999537652	1.0763986179
-1.1693039948	0.565907182	0.9999537652	1.1635318451
2.7933974408	0.6948713406	0.9999537652	12.7820459093
1.4049428145	0.1175643402	0.9999537652	-1.0752873441
-1.2963709085	0.2407431275	0.9999537652	1.460144486
-1.0539580642	0.8732019188	0.9999537652	-1.2242110047
-1.0642209066	0.7053681004	0.9999537652	1.3973405727

0	0	0	4.9527501949
-1.0005546727	0.9984975432	0.9999537652	-1.0292619808
-1.6533121528	0.4727785747	0.9999537652	1.7805692956
-1.1619124208	0.2916893882	0.9999537652	-1.0068448899
2.7933974408	0.6948713406	0.9999537652	-4.1147445994
1.1789135104	0.3228883288	0.9999537652	1.0527034979
1.3703758797	0.5584653486	0.9999537652	2.191689061
-1.0475667213	0.7932577381	0.9999537652	1.046257844
8.064174035	0.42386109	0.9999537652	-7.5938938218
-1.0271839745	0.9301494863	0.9999537652	-1.4794014745
1.0129071158	0.9595136548	0.9999537652	1.1502256127
-1.036065864	0.9311263257	0.9999537652	1.2382459797
1.1082980069	0.7051440002	0.9999537652	1.2653659748
1.2221484578	0.7268243913	0.9999537652	1.3237138559
1.0608805067	0.6856390815	0.9999537652	1.2203688867
-1.6400934886	0.2993572224	0.9999537652	-1.1136140278
1.810938637	0.0662692265	0.9999537652	1.630642421
-1.2982596652	0.3439343602	0.9999537652	1.4833674591
1.1222763882	0.6938345701	0.9999537652	1.3056043564
0	0	0	2.9988886918
2.7933974408	0.6948713406	0.9999537652	2.9985540909
-1.3509517562	0.4401965018	0.9999537652	-1.2073222441
-1.0938933423	0.8347800059	0.9999537652	-1.042636332
-1.2826950936	0.407151505	0.9999537652	1.024124714
11.5766527773	0.3454613255	0.9999537652	-2.0931946048
2.7933974408	0.6948713406	0.9999537652	4.9527501949
-1.5753956458	0.1311730696	0.9999537652	-1.0878695743
1.0247747451	0.8935151706	0.9999537652	1.3029291222
1.1938444774	0.1690282715	0.9999537652	-1.0249770519
67.3679493299	0.1028771372	0.9999537652	-5.0778270227
1.0273911124	0.8358511828	0.9999537652	1.2749466445
-1.0069658003	0.9769681719	0.9999537652	1.0707892536
-1.001828667	0.9919310936	0.9999537652	-1.0514611088
-1.5861169239	0.7128681534	0.9999537652	2.9957580451
1.5619956449	0.0948722527	0.9999537652	1.0141229612
-1.6050134504	0.4757254636	0.9999537652	-1.5446056776
-1.8682422788	0.3275403123	0.9999537652	1.039905587
-1.3811511599	0.1454242764	0.9999537652	-1.0267481182
1.2547599433	0.8138366009	0.9999537652	1.2738997544
6.3075231393	0.4859256145	0.9999537652	3.9269887543
1.0174686303	0.9217625864	0.9999537652	-1.4714835237
1.9416585923	0.1335241641	0.9999537652	1.5320730211
1.1329612377	0.5043927977	0.9999537652	1.2131818071
1.1297615884	0.707570459	0.9999537652	-1.1224417065
1.0717966888	0.6188062913	0.9999537652	1.2609005158
1.3195866646	0.4814139981	0.9999537652	1.4848434056
1.2109021666	0.5262919175	0.9999537652	1.3347974597
-1.0117921347	0.9419769677	0.9999537652	1.1096622562
20.3888260912	0.2437331429	0.9999537652	0

23.8617041368	0.2201079761	0.9999537652	-1.5807630514
-1.1576464446	0.5788150279	0.9999537652	1.0769117024
1.0715997868	0.6112170917	0.9999537652	-1.0625987566
1.3857529974	0.1798595595	0.9999537652	1.2143594491
-1.577011236	0.0307774968	0.9999537652	1.2211677395
-1.0950953076	0.8492594781	0.9999537652	-1.5365812802
-1.1229879161	0.6105105193	0.9999537652	1.3160688149
-1.0065573028	0.9745287714	0.9999537652	1.0886217538
-1.1579609705	0.3841237465	0.9999537652	-1.0187390657
-2.6527850164	0.1212889734	0.9999537652	2.2155898126
1.0006941223	0.9982326477	0.9999537652	-1.1318963538
-1.1111645146	0.4379274766	0.9999537652	1.099736946
1.1478933566	0.4971537616	0.9999537652	-1.1603037568
1.163695605	0.6435938542	0.9999537652	-1.1666745609
1.0446357697	0.7528137897	0.9999537652	-1.0046334623
1.2969735684	0.6703765071	0.9999537652	1.2760891251
9.8205505243	0.3806487041	0.9999537652	8.8700459102
-2.7750113771	0.2108651016	0.9999537652	1.0691759863
-1.0569269529	0.8537995955	0.9999537652	2.0172653658
1.3570059574	0.3154784907	0.9999537652	-1.0632972084
1.2309640129	0.1867046242	0.9999537652	1.1316806538
0	0	0	-1.8577842586
1.0789731544	0.8018443088	0.9999537652	1.0144843107
1.1510341203	0.6487660506	0.9999537652	-1.2416069402
1.0857901371	0.6691006808	0.9999537652	1.1203497392
-1.1565394511	0.4147635069	0.9999537652	1.161097447
-1.7043575086	0.1781022047	0.9999537652	-1.1669506304
1.0676138738	0.7599465362	0.9999537652	-1.2067495424
2.7933974408	0.6948713406	0.9999537652	-1.144309199
-1.1765647297	0.3710578576	0.9999537652	1.1270844352
8.0710074692	0.4203544835	0.9999537652	-2.1294907556
1.1144134358	0.3852903689	0.9999537652	1.1700548017
-1.5764197779	0.5243565684	0.9999537652	-1.1409720983
-1.3734234034	0.7176110162	0.9999537652	-1.2800522501
1.2565136616	0.2676040844	0.9999537652	-1.1738191879
6.3130691196	0.4798093448	0.9999537652	1.1089257718
1.1953956413	0.3666069872	0.9999537652	-1.3085633241
-1.6674095463	0.6213810348	0.9999537652	1.3993098199
-1.266144156	0.3101608069	0.9999537652	1.1397712861
2.5539148516	0.2217371432	0.9999537652	1.5445547046
1.3650039897	0.2214565666	0.9999537652	-1.1406814698
0	0	0	0
-1.4276209478	0.4180243489	0.9999537652	-1.2006774845
1.1793006604	0.3680148579	0.9999537652	-1.0930814734
1.0915702994	0.4983809035	0.9999537652	-1.0202528275
0	0	0	0
-1.0761238566	0.9086825963	0.9999537652	-2.6795343993
-2.9570895726	0.3896766718	0.9999537652	-2.2394303886
1.113704751	0.6849583912	0.9999537652	1.2233404731

-2.1885668332	0.2435777519	0.9999537652	-1.634520667
6.3075231393	0.4859037428	0.9999537652	0
-1.0040545829	0.9865051117	0.9999537652	-1.2278647161
-1.3385641172	0.1735909755	0.9999537652	1.0921562182
-6.3038745744	0.1139519388	0.9999537652	1.7251428509
0	0	0	2.996475627
-1.1113356286	0.6074706298	0.9999537652	1.1853940771
-1.0955076328	0.7841971966	0.9999537652	1.0105552642
4.3467773692	0.0673462832	0.9999537652	-4.4703372671
0	0	0	2.9985540909
6.3075231393	0.4858818993	0.9999537652	0
1.3594099105	0.7970662522	0.9999537652	1.2801191671
-1.0048792563	0.9908938539	0.9999537652	-1.1925183839
1.2639004223	0.3996167215	0.9999537652	-1.3460629855
1.1597199062	0.7028182189	0.9999537652	-1.0852357393
1.0006990062	0.9976372662	0.9999537652	-1.0044369005
-1.0212402807	0.9590351587	0.9999537652	1.2379270009
22.1449503561	0.2295716029	0.9999537652	1.2684830807
-1.3060742329	0.2587590986	0.9999537652	1.6275105309
-1.2396428094	0.230178226	0.9999537652	1.0574725337
-1.3443523415	0.3689394867	0.9999537652	1.0563275471
1.3840729079	0.1109745055	0.9999537652	1.4026074125
-1.0155800036	0.9597684715	0.9999537652	-1.2830241381
-1.092657348	0.5884466162	0.9999537652	1.293138654
1.818286487	0.1964195738	0.9999537652	1.1710990071
2.7933974408	0.6948713406	0.9999537652	0
1.3009616064	0.54913202	0.9999537652	1.1038691503
1.2257351007	0.2637844861	0.9999537652	-1.0518619644
1.1913178689	0.1614046946	0.9999537652	1.3952440532
1.8206313854	0.5988534815	0.9999537652	-1.1365331554
2.7957660672	0.7054691191	0.9999537652	0
-2.587194901	0.5007035754	0.9999537652	4.9561842702
-1.1969229065	0.3820913358	0.9999537652	1.2960340598
2.1974038942	0.2946197239	0.9999537652	-4.2387878395
1.167995342	0.5273750092	0.9999537652	1.1077642929
1.3589992143	0.3718181318	0.9999537652	1.6019209384
2.7933974408	0.6948713406	0.9999537652	-5.3583704783
-2.1283838037	0.3362356889	0.9999537652	1.3060086369
1.1341637742	0.6703154468	0.9999537652	1.0071658635
-1.1767472282	0.4582170488	0.9999537652	1.1303148419
16.8433158146	0.2735542479	0.9999537652	-1.1241083699
-1.6980553762	0.5271226607	0.9999537652	-3.1944206015
-1.1417631068	0.4876767788	0.9999537652	-1.0149720939
-1.0188478837	0.966084823	0.9999537652	1.1710846984
-1.3361026929	0.2930825977	0.9999537652	1.1144917184
1.3216805319	0.496272292	0.9999537652	-1.425776575
-2.0984085497	0.010798083	0.9999537652	1.071563557
1.0056468924	0.9656377788	0.9999537652	1.1330933982
1.3959372098	0.666362328	0.9999537652	-1.939200616

1.0736533958	0.712426287	0.9999537652	1.0196583691
-1.2634569391	0.0514299564	0.9999537652	1.1381452304
1.0517529507	0.9033218549	0.9999537652	1.529869622
1.004601693	0.9672273473	0.9999537652	1.2227969234
1.0578173942	0.715384528	0.9999537652	1.115678269
1.0233172702	0.9456582211	0.9999537652	-1.0409672658
-1.5867714314	0.7160997034	0.9999537652	4.7486936474
1.0274942246	0.8650940633	0.9999537652	-1.2482403885
-1.7256502905	0.4947510749	0.9999537652	-1.9869457057
3.3386254244	0.1676165176	0.9999537652	-1.056751263
0	0	0	2.9985540909
1.2527115433	0.8123164945	0.9999537652	-1.3426678013
1.4737523755	0.2987734418	0.9999537652	-1.147633542
1.0549201187	0.7119897323	0.9999537652	-1.0292305634
1.0035498141	0.9781159802	0.9999537652	1.296709659
1.8222329048	0.2466093308	0.9999537652	-1.0760516321
-1.3423111503	0.0416840044	0.9999537652	1.0316461212
2.7933974408	0.6948713406	0.9999537652	6.908559683
1.5108760492	0.5271608029	0.9999537652	-2.6216240642
2.7933974408	0.6948713406	0.9999537652	0
18.6310147587	0.2581002427	0.9999537652	1.1562656392
-1.5587684445	0.1886260818	0.9999537652	1.0733574289
1.084985329	0.6668810927	0.9999537652	-1.4079773046
-1.5462358792	0.2329640599	0.9999537652	1.3134190914
6.6960780981	0.0826914313	0.9999537652	-2.0013472884
1.8259740378	0.6336148297	0.9999537652	1.5932034817
1.1298260652	0.5921353139	0.9999537652	1.1592797979
-1.0003821381	0.9976141232	0.9999537652	-1.4178967317
0	0	0	2.996475627
1.2321761132	0.4085661547	0.9999537652	1.1453665421
-1.1004181912	0.618196992	0.9999537652	1.1480633927
1.0894645231	0.6047534975	0.9999537652	-1.2384278976
1.5913841917	0.1619632532	0.9999537652	1.2651347039
-1.0692150516	0.77823473	0.9999537652	-1.0555202004
-1.483866215	0.0422730952	0.9999537652	1.0480245844
1.582154632	0.4526798258	0.9999537652	3.5481057558
1.0533757214	0.7688068226	0.9999537652	1.1894738143
1.1558796531	0.7482223541	0.9999537652	-1.1869442514
1.527204761	0.2377096856	0.9999537652	1.1735922103
-1.0533280245	0.8722013693	0.9999537652	1.0208349995
-1.1885359807	0.2299149989	0.9999537652	-1.2241671362
1.0917375443	0.5495066697	0.9999537652	-1.1570429423
1.1962825574	0.7550432886	0.9999537652	-1.4035206294
1.1318726633	0.477037499	0.9999537652	1.1828599125
2.7933974408	0.6948713406	0.9999537652	1.067171975
1.0435514625	0.8304428519	0.9999537652	-1.0827113432
1.6380746162	0.0656004594	0.9999537652	-1.1000722403
1.2990400075	0.7578358812	0.9999537652	2.488271085
-1.1475589774	0.5773714107	0.9999537652	-1.1781946077

1.0131896226	0.9694602283	0.9999537652	1.1797462473
-1.1117455455	0.4541391175	0.9999537652	1.1233748327
-1.4285873355	0.4428723042	0.9999537652	-1.3363898156
1.1873139295	0.5683578041	0.9999537652	1.1293134239
1.3640571596	0.1440051915	0.9999537652	1.20762297
-1.0580014703	0.7948883319	0.9999537652	1.1533013759
0	0	0	26.4411849241
1.1206203515	0.6211767423	0.9999537652	1.1036417695
-1.4032564076	0.0748251115	0.9999537652	1.1562766256
-1.1541146099	0.3140595719	0.9999537652	1.1138478762
-1.1978530251	0.6479976791	0.9999537652	1.7788094936
1.0999421972	0.7755602905	0.9999537652	-1.0767359799
4.5505976667	0.5559322269	0.9999537652	0
-1.4104128618	0.4813729915	0.9999537652	-1.046235579
1.040281569	0.7717415595	0.9999537652	1.1262364093
1.0933258334	0.6623162228	0.9999537652	-1.1309441773
-1.0363788903	0.8541371186	0.9999537652	1.2809675336
1.2072800896	0.2868797883	0.9999537652	-1.0528733751
-2.4941151816	0.4484109485	0.9999537652	2.0650565302
-1.2132060652	0.4376724878	0.9999537652	-1.0816359856
1.2084842145	0.5532965621	0.9999537652	-1.1369054259
1.1529343388	0.2175435991	0.9999537652	1.1506986909
1.0510635703	0.8105905001	0.9999537652	1.2375999683
-1.199133825	0.1601378376	0.9999537652	-1.2375603839
-1.1086651739	0.7456900635	0.9999537652	-1.0105082838
1.7180264605	0.0320375034	0.9999537652	1.4303847056
2.7957660672	0.7054714763	0.9999537652	0
2.5375363294	0.0601863413	0.9999537652	-1.0087239788
2.3407960637	0.2629337289	0.9999537652	1.4144911607
-1.0702036118	0.6448920995	0.9999537652	-1.292096712
5.9693523789	0.102919379	0.9999537652	2.3749818327
-1.3031268977	0.0931774419	0.9999537652	1.2140734742
2.9108875149	0.4212749072	0.9999537652	14.7271569203
1.0588673556	0.6599676411	0.9999537652	1.2601331836
0	0	0	-1.8582093966
-1.1400524203	0.6925582319	0.9999537652	1.079045435
4.5505976667	0.5559322269	0.9999537652	6.9133480437
-1.0641924796	0.7180478335	0.9999537652	1.0015309038
1.1216701798	0.9025855133	0.9999537652	-1.3140344109
1.275282393	0.6534106613	0.9999537652	1.027333951
-1.0974096956	0.6455469974	0.9999537652	1.3238635508
1.0226902024	0.896031783	0.9999537652	1.020298758
-1.6674095463	0.6211804098	0.9999537652	-5.3589986948
1.7438374005	0.1731885952	0.9999537652	1.5617427513
8.064174035	0.4238597542	0.9999537652	-1.6597328868
-1.1154820314	0.586544898	0.9999537652	1.2643983124
1.3025788871	0.7332127736	0.9999537652	-2.0448877535
1.0676074109	0.6430257067	0.9999537652	1.3876185589
-1.208510781	0.6367113156	0.9999537652	1.0559626811

-3.1179374904	0.2575007764	0.9999537652	2.9985540909
0	0	0	0
1.3001586758	0.7288997037	0.9999537652	-2.8215558128
-1.1338812575	0.7219653432	0.9999537652	1.7839566788
1.2057613658	0.7517614146	0.9999537652	1.0176694598
-1.1423326412	0.7403754276	0.9999537652	1.0651798039
1.0305243997	0.8819815336	0.9999537652	1.1070215658
1.9987268311	0.2978721628	0.9999537652	-1.4616100759
1.3979934472	0.455177279	0.9999537652	1.7206577206
8.1372627563	0.0117647212	0.9999537652	2.2447949745
5.9768568161	0.0923150692	0.9999537652	1.4519459565
-1.501078988	0.1905055824	0.9999537652	-1.2427586638
-1.0597710429	0.7545630442	0.9999537652	1.4479778851
0	0	0	0
-1.2040232458	0.5712705945	0.9999537652	-1.2217930755
0	0	0	4.9527501949
1.115591456	0.6802342081	0.9999537652	1.2692838438
1.1612439625	0.5084913285	0.9999537652	1.5218344384
-1.1042647991	0.826840717	0.9999537652	1.0268360662
1.1789008325	0.5674901112	0.9999537652	1.0468029632
-1.5312189421	0.1829520875	0.9999537652	-1.0217750814
-1.107708211	0.6272875538	0.9999537652	1.1213051659
1.4946040872	0.1614007054	0.9999537652	1.3286410706
1.0746305754	0.7400616157	0.9999537652	1.2336525653
-1.239744256	0.3440573251	0.9999537652	1.050575786
-1.5581362771	0.0844734608	0.9999537652	-1.1205799622
0	0	0	4.9527501949
1.1261405279	0.8542542807	0.9999537652	1.0387126023
-1.135379807	0.5269209686	0.9999537652	1.0693425341
1.3366571197	0.386755597	0.9999537652	-1.074393129
-1.9598615693	0.1611947422	0.9999537652	1.0126931411
-1.0995135404	0.6425324968	0.9999537652	1.5617682385
4.5544554512	0.56913538	0.9999537652	4.9527501949
1.0367599971	0.8640604535	0.9999537652	1.3636664298
0	0	0	2.9985540909
2.7933974408	0.6948713406	0.9999537652	2.996475627
0	0	0	0
2.7933974408	0.6948713406	0.9999537652	0
-1.1858184564	0.2698575895	0.9999537652	1.1676215024
1.0822052743	0.7233600657	0.9999537652	-1.1161410024
1.1401644591	0.5857501715	0.9999537652	-1.0899350295
0	0	0	2.9936870021
1.603906631	0.6805260368	0.9999537652	-1.4365753027
1.827667131	0.0256572699	0.9999537652	1.1926821055
1.0637894227	0.7691452458	0.9999537652	1.0929704504
-1.2053946388	0.7439060599	0.9999537652	-1.3178205855
29.1226294179	0.1908912554	0.9999537652	1.5563914108
2.7933974408	0.6948713406	0.9999537652	1.8589547811
-1.2242387651	0.1255177788	0.9999537652	-1.1063754375

-1.057635161	0.7385740007	0.9999537652	-1.2230162314
-1.0998297781	0.6789652972	0.9999537652	1.1254295224
1.7847110014	0.3579393046	0.9999537652	-1.1287447286
3.7917931577	0.2238250833	0.9999537652	-1.6103932565
-1.1516964145	0.4122310289	0.9999537652	-1.013115112
-1.2308837349	0.5339858947	0.9999537652	-1.3139663928
-1.0473752886	0.7816109512	0.9999537652	-1.185074891
1.3377809008	0.2080360016	0.9999537652	-1.367649661
-1.7141251245	0.5217296346	0.9999537652	-1.5413176099
1.0181666844	0.8827655577	0.9999537652	1.135308509
1.3326456572	0.2667891461	0.9999537652	-1.3834464205
-1.2089981555	0.5336352155	0.9999537652	1.0062326897
1.2467380137	0.1813207875	0.9999537652	-1.3319030788
1.1463368891	0.4874612654	0.9999537652	1.0146647071
4.5544554512	0.569133386	0.9999537652	3.0014231918
9.8205505243	0.3806255368	0.9999537652	-1.8582093966
1.1714652592	0.5584838141	0.9999537652	-1.0846414195
-1.3183497373	0.6678653454	0.9999537652	1.4370312972
4.5505976667	0.5559322269	0.9999537652	-4.8186929849
-1.2711474135	0.2128653532	0.9999537652	-1.2062856952
17.3472938331	0.2975561866	0.9999537652	8.8639046058
1.2204962623	0.5811143316	0.9999537652	1.0494835949
-1.0033878781	0.9931124794	0.9999537652	-1.0719699378
0	0	0	9.7116702472
1.0954599379	0.6004848573	0.9999537652	-1.1999649533
1.0356595745	0.8092380975	0.9999537652	-1.1567566972
-2.956038745	0.3205427223	0.9999537652	2.226401313
-1.0745696932	0.7142976782	0.9999537652	1.0590138039
1.0070737681	0.9823908948	0.9999537652	-1.009204357
2.0363455658	0.4191792472	0.9999537652	1.8159392373
-1.2653006465	0.1746380865	0.9999537652	-1.3587366354
1.1478914847	0.499518718	0.9999537652	1.1067607569
1.3670871454	0.4772640638	0.9999537652	1.5578544735
1.6512577839	0.1957128114	0.9999537652	1.2080900011
0	0	0	2.996475627
1.0442905078	0.7655283163	0.9999537652	1.1066304142
-1.6217625998	0.0628059593	0.9999537652	1.3592808127
-1.0806024826	0.7649858096	0.9999537652	-1.155288339
-1.0942188966	0.627498364	0.9999537652	1.0201860177
-1.1026115964	0.4138929342	0.9999537652	1.0420067715
2.1937664026	0.2878393251	0.9999537652	1.0004317752
-1.1780987613	0.6880432174	0.9999537652	-2.0406098627
1.2579508034	0.0750652361	0.9999537652	1.0928037111
-1.0919905267	0.63727033	0.9999537652	-1.2420607509
0	0	0	20.5862458385
-1.1902317392	0.5163879322	0.9999537652	-1.2207647944
1.2879691289	0.092152991	0.9999537652	1.1263895777
-1.0281465907	0.8875102938	0.9999537652	-1.2693078548
-1.3095088252	0.5260063579	0.9999537652	1.1632391783

1.5313852055	0.5216372602	0.9999537652	1.6984478315
-1.1813031634	0.4835621879	0.9999537652	-1.1660328546
1.0794889877	0.7293482269	0.9999537652	-1.6752775289
1.4071079349	0.2722577121	0.9999537652	1.0878127162
1.170178844	0.6928356326	0.9999537652	-1.0190639033
-1.101183384	0.8491448233	0.9999537652	-1.0927247289
1.6152058699	0.0792962622	0.9999537652	1.0708335229
-1.3304241061	0.0176016343	0.9999537652	-1.5482134169
-1.2668558963	0.098952339	0.9999537652	1.0178462262
4.5505976667	0.5559322269	0.9999537652	1.1554536166
1.1464431389	0.6035377383	0.9999537652	-1.0017159662
1.2607159044	0.1863536795	0.9999537652	-1.1712914944
1.4089461712	0.3760047958	0.9999537652	-1.1534884442
-1.1242717848	0.6044713935	0.9999537652	-1.2345256645
-1.0982503828	0.4968893589	0.9999537652	-1.1025862244
1.1413431861	0.7393987324	0.9999537652	-1.2115914115
4.5505976667	0.5557790267	0.9999537652	0
1.2884369719	0.2827133749	0.9999537652	1.1714801535
-1.0622050082	0.9006742497	0.9999537652	1.0074664906
-1.6674095463	0.6211992247	0.9999537652	-3.383799441
1.0998091457	0.7325809517	0.9999537652	-1.2756099766
1.3616604306	0.2379741322	0.9999537652	-1.1551584353
-2.956038745	0.3205409092	0.9999537652	14.759428837
-1.6818365992	0.2867264494	0.9999537652	-1.1105052602
-1.4356824791	0.2646209381	0.9999537652	-1.2202715628
1.1434339733	0.6327686631	0.9999537652	1.0551440262
-1.052220172	0.8298448144	0.9999537652	-1.0371440844
-1.361720732	0.1555506708	0.9999537652	1.0149559728
-1.1101170285	0.7725636825	0.9999537652	-1.1824957367
1.0047743529	0.9776543066	0.9999537652	-1.2331665558
1.0785588582	0.9501492552	0.9999537652	1.1361852165
1.4194596463	0.112338496	0.9999537652	1.0563023374
2.330589113	0.4664509828	0.9999537652	-1.3019789167
-1.1762648374	0.5443130387	0.9999537652	-1.1773596301
-1.1362981735	0.4738861131	0.9999537652	1.0847698528
-18.9917210433	0.0490947718	0.9999537652	6.908559683
1.1094201658	0.6714636741	0.9999537652	1.7672763476
1.695813492	0.054888381	0.9999537652	1.0108673835
1.0040159103	0.9754157117	0.9999537652	1.2534187913
-1.2047793594	0.314102676	0.9999537652	-1.1322125239
-1.1326255481	0.7004223503	0.9999537652	-1.3139335181
2.7957660672	0.7054761936	0.9999537652	0
1.3989923272	0.6140065661	0.9999537652	1.0688925511
-1.3139750984	0.1563628748	0.9999537652	-1.1191460601
-1.1953747446	0.5870574125	0.9999537652	-1.0421476012
1.1395594238	0.3632852134	0.9999537652	1.0307453602
-1.3679958882	0.7463109286	0.9999537652	28.0550019808
2.2535802584	0.1210621813	0.9999537652	1.6102150583
6.3075231393	0.4858600839	0.9999537652	-1.4601750349

1.0206341456	0.8892512256	0.9999537652	1.0186785036
1.1431700605	0.7331157731	0.9999537652	1.3794708826
1.4373613941	0.1377940692	0.9999537652	-1.5026553489
18.6146543426	0.2564041956	0.9999537652	1.1103074297
11.5868260078	0.3461944637	0.9999537652	-1.2800515331
1.0541531647	0.74765511	0.9999537652	-1.0581891681
1.2170565251	0.3327737042	0.9999537652	1.4487726995
0	0	0	-9.235035142
-1.0882761374	0.6427155507	0.9999537652	1.1321537748
-1.0488508938	0.7535619601	0.9999537652	1.1645070833
1.1716043256	0.7139429453	0.9999537652	-1.1091443276
1.1129205313	0.4757461081	0.9999537652	1.3211181481
18.5983228161	0.2580172642	0.9999537652	-3.0053232
1.0817075216	0.651137536	0.9999537652	1.2999016271
1.0448451532	0.8921609784	0.9999537652	-1.2200625877
1.1033108693	0.5628923532	0.9999537652	1.0299122555
1.0156369479	0.9527773212	0.9999537652	-1.2274391494
1.1211860098	0.9130175902	0.9999537652	-2.0741723538
1.2441716671	0.2449727246	0.9999537652	1.1106239848
1.2486503609	0.4200857934	0.9999537652	1.0659305893
1.0118450101	0.9704482605	0.9999537652	1.0192311602
6.3075231393	0.4858382966	0.9999537652	16.6806485101
-1.4206868771	0.36708337	0.9999537652	1.4141596021
1.1504832506	0.7836895831	0.9999537652	1.0876005687
1.2677054258	0.4416059492	0.9999537652	1.3037891306
1.5130423174	0.6604211772	0.9999537652	-1.1574174841
1.4619130493	0.4058424493	0.9999537652	1.2941134052
0	0	0	-5.3583704783
2.7933974408	0.6948713406	0.9999537652	0
-1.1314771982	0.6015570579	0.9999537652	-1.2080818548
-1.0512102387	0.8469507909	0.9999537652	-1.0469954452
-1.2194299046	0.300401329	0.9999537652	1.193100327
0	0	0	2.9985540909
-4.3234865661	0.1504192763	0.9999537652	18.6594677739
1.1158583321	0.9296598684	0.9999537652	0
-1.0494046033	0.8623624356	0.9999537652	1.5307245327
-1.170046239	0.4580758095	0.9999537652	1.0959183498
0	0	0	2.9988886918
-1.3951142566	0.3557604348	0.9999537652	1.8688548069
1.2735141212	0.2148657585	0.9999537652	1.0686226752
-1.0895214978	0.6746398157	0.9999537652	1.0398471179
-1.0413817275	0.775616009	0.9999537652	1.0535259259
1.1220518419	0.6490142141	0.9999537652	1.1982798319
1.0817709416	0.6138071626	0.9999537652	-1.3108942584
1.0229607066	0.8708518377	0.9999537652	1.516796602
-1.1977369949	0.6648609941	0.9999537652	-1.0504978146
1.0564691958	0.7658738235	0.9999537652	1.136316392
1.2417456347	0.1625214344	0.9999537652	1.1780957443
1.1781413431	0.720601728	0.9999537652	-1.0143339149

1.0811893466	0.8294479986	0.9999537652	1.0755583371
1.0703944827	0.6994561201	0.9999537652	1.2993964164
1.0994968002	0.6752103993	0.9999537652	-1.0348995782
0	0	0	8.8639046058
-1.3429809541	0.3881069219	0.9999537652	1.418735717
-1.0602621373	0.8170639411	0.9999537652	1.1861614471
1.2098822613	0.4281127395	0.9999537652	1.4163597087
-2.5861438062	0.4681199623	0.9999537652	-3.7211335265
0	0	0	4.9561842702
-2.4652347606	0.1804051	0.9999537652	1.0203837698
-1.1718543992	0.8302025312	0.9999537652	1.0824394533
0	0	0	4.9527501949
1.5298977121	0.3526169069	0.9999537652	1.4479895097
-1.6249640116	0.2755372466	0.9999537652	1.3602858602
1.0462261817	0.9444120098	0.9999537652	1.6930547015
1.9579430518	0.0196436142	0.9999537652	1.4930660957
-1.21815506	0.3842988946	0.9999537652	-1.0119304616
-1.3714431973	0.0163889198	0.9999537652	-1.4272116474
4.5505976667	0.5557790267	0.9999537652	0
1.087701826	0.6402075148	0.9999537652	1.7759163954
1.0472633739	0.9151780431	0.9999537652	1.2494841749
0	0	0	-1.2590868438
1.2177824976	0.4004620562	0.9999537652	-1.4276032567
1.0576409478	0.7953233081	0.9999537652	1.2560455907
1.2683633881	0.4504040727	0.9999537652	-1.160926583
-1.0180629367	0.9535611799	0.9999537652	1.2002708548
1.2873325466	0.1977813971	0.9999537652	-1.2257853903
2.7933974408	0.6948713406	0.9999537652	4.9527501949
1.261710384	0.4957689909	0.9999537652	1.0448392655
1.0522316591	0.7805385813	0.9999537652	-1.1110122708
-1.0948072995	0.5427091485	0.9999537652	1.1294282135
3.0590680122	0.3357191982	0.9999537652	-1.1322464845
0	0	0	4.9527501949
6.3130691196	0.4798187332	0.9999537652	1.5908108123
1.2357313159	0.3667894967	0.9999537652	1.164264678
-1.0784119283	0.5865033119	0.9999537652	-1.1233592427
0	0	0	2.996475627
1.0320276904	0.9160923415	0.9999537652	-1.2510297534
1.1574744424	0.3690516452	0.9999537652	1.1485223264
-1.1142215085	0.5859968392	0.9999537652	-1.2128413625
1.5666957508	0.0953541449	0.9999537652	1.1950963994
1.3725931849	0.063533509	0.9999537652	-1.1753659791
-2.1121758979	0.3451523316	0.9999537652	-1.4474064009
1.1189859225	0.6384529674	0.9999537652	-1.0986220743
6.3075231393	0.4858165373	0.9999537652	-9.7565542884
-1.3702762656	0.7549819127	0.9999537652	16.6921882404
-1.2766380543	0.5456278554	0.9999537652	1.0955482217
-1.1443444191	0.5835726082	0.9999537652	-1.3837886538
3.4552788346	0.0790731748	0.9999537652	1.0071225085

3.3249364486	0.0383976571	0.9999537652	-1.0424478359
1.2793527168	0.2325080685	0.9999537652	-1.062738616
-1.0600785361	0.7422583466	0.9999537652	-1.1892100792
6.3075231393	0.4857948061	0.9999537652	2.9985540909
1.3350618227	0.4408872704	0.9999537652	-1.2259965384
1.21207844	0.2366915329	0.9999537652	-1.1990482093
-1.1456095562	0.6377567175	0.9999537652	1.0082282879
16.8575730017	0.2720678674	0.9999537652	-2.7895842989
6.3075231393	0.485772013	0.9999537652	1.6755843638
0	0	0	4.9527501949
1.1887778888	0.3630892764	0.9999537652	-1.0721096281
1.3516764085	0.0973390111	0.9999537652	1.0231425108
-1.1154984609	0.769374728	0.9999537652	1.0685248398
-1.0860203881	0.5216212366	0.9999537652	-1.3477391635
2.7933974408	0.6948713406	0.9999537652	-2.2683615775
1.6539935729	0.2846857903	0.9999537652	-1.4621772559
1.0359217103	0.7858907891	0.9999537652	-1.3516611555
-1.1250888319	0.4856502038	0.9999537652	-1.3191935704
-1.5881570201	0.709587026	0.9999537652	1.9432271302
-1.2373495548	0.3656485584	0.9999537652	-1.3998623464
0	0	0	-1.2732083991
1.0015539757	0.9944860413	0.9999537652	-1.0531191994
1.2059832928	0.2076238796	0.9999537652	1.1068014826
-7.2100360446	0.1268521202	0.9999537652	4.4079008263
-1.5243343774	0.07402306	0.9999537652	-1.746687833
1.2359285705	0.761332809	0.9999537652	-1.4429435179
1.0134399309	0.9638557854	0.9999537652	-1.1535880637
-1.1664808483	0.4108594489	0.9999537652	-1.3149373974
2.9527141659	0.0925620315	0.9999537652	1.7231940929
0	0	0	2.996475627
0	0	0	1.2043966325
-1.090373429	0.6172043238	0.9999537652	-1.3812927695
-1.029574592	0.9055146177	0.9999537652	-1.2932473294
13.355081045	0.3178957903	0.9999537652	-14.149476906
1.211821937	0.1248525386	0.9999537652	1.0731678115
1.0830180545	0.7393428987	0.9999537652	1.0757038205
1.671787244	0.1572450681	0.9999537652	1.2462690465
11.5766527773	0.3454301478	0.9999537652	3.7700212082
1.9021910848	0.2909239508	0.9999537652	1.135702533
1.1338209709	0.2892004555	0.9999537652	-1.0832718725
1.1818857488	0.4815842885	0.9999537652	1.2205861525
-1.4310703425	0.3727322015	0.9999537652	-1.2145671933
1.0141365239	0.9420252643	0.9999537652	-1.1242826323
1.0950284989	0.5014780399	0.9999537652	1.0081225511
-1.2664954471	0.4993698719	0.9999537652	-1.1791482154
-1.0560213696	0.702268602	0.9999537652	-1.159581567
-1.2274688783	0.3690940286	0.9999537652	-1.2005920631
2.7933974408	0.6948713406	0.9999537652	0
0	0	0	4.9527501949

2.7933974408	0.6948713406	0.9999537652	-4.2377976271
1.5360039748	0.0262141626	0.9999537652	-1.0073240153
0	0	0	2.9936870021
1.698659425	0.1139921075	0.9999537652	-1.1958875504
-1.1453192235	0.9086509514	0.9999537652	-1.859280336
5.4939478721	0.1042652041	0.9999537652	-1.610613801
1.1193342077	0.3320228204	0.9999537652	-1.5561498901
1.9330420755	0.3731032652	0.9999537652	-1.6009476582
-1.1529188894	0.4669409468	0.9999537652	-1.3141911837
0	0	0	4.9561842702
1.3829044638	0.1887349303	0.9999537652	1.3546802486
9.8205505243	0.3806024432	0.9999537652	1.2557686318
1.2239099077	0.5212192095	0.9999537652	-1.1299298918
11.5864583426	0.3448229864	0.9999537652	2.4647648096
1.1974158141	0.5624993542	0.9999537652	-1.3271516535
-1.0163188705	0.9406390613	0.9999537652	1.0383885303
0	0	0	8.8639046058
1.0017929882	0.9924908703	0.9999537652	1.1246913783
1.7003370297	0.1768923331	0.9999537652	1.0606784486
4.2795716544	0.1764467898	0.9999537652	4.0080058676
2.0177191975	0.1476193755	0.9999537652	1.0575324125
1.1088914292	0.5732352172	0.9999537652	-1.1899818251
-1.1925736325	0.1557065228	0.9999537652	-1.5514463085
-1.0068955241	0.9692178285	0.9999537652	-1.3966090852
1.1073681766	0.670428013	0.9999537652	-1.5444051283
1.3249062187	0.3582319533	0.9999537652	1.3224980685
-1.5861169239	0.7133179947	0.9999537652	-1.4281141693
0	0	0	8.8639046058
-1.4743450296	0.64499366	0.9999537652	2.0633900066
-1.3219599047	0.1719399484	0.9999537652	1.1305881595
1.0955901317	0.7962107869	0.9999537652	1.0553953832
-1.0049649224	0.9748129406	0.9999537652	-1.0122406022
-1.1030525978	0.5716164103	0.9999537652	-1.2696773997
-1.0716117245	0.5871358438	0.9999537652	1.0767997954
-1.2416211531	0.588348796	0.9999537652	1.2494843587
-2.0846733449	0.2553367603	0.9999537652	-14.159275343
1.3671081636	0.4876913826	0.9999537652	1.3682078064
-1.5881570201	0.708069387	0.9999537652	1.0023136832
1.3346379155	0.5901172668	0.9999537652	1.5153496654
25.6156185703	0.2096817295	0.9999537652	-1.0403091835
1.542581106	0.1595200882	0.9999537652	-1.0557556442
1.6018473574	0.6931691698	0.9999537652	-2.0941712051
-1.7149555905	0.0414629273	0.9999537652	-1.8646014246
4.5505976667	0.5557790267	0.9999537652	-2.1294907556
4.5505976667	0.5557790267	0.9999537652	-1.1241083699
-1.586167124	0.7107802378	0.9999537652	-1.9205861372
1.4964917328	0.422854103	0.9999537652	-1.3717250396
1.2788907567	0.2392539024	0.9999537652	-1.073611821
1.202277345	0.6367563718	0.9999537652	-1.3815630528

-1.3452181228	0.2595387621	0.9999537652	-1.6696939021
1.0318886281	0.8614279811	0.9999537652	-1.4465061341
-1.2247725479	0.5480855007	0.9999537652	-1.0551473674
1.0340314084	0.8490308378	0.9999537652	-1.027620123
4.5505976667	0.5557790267	0.9999537652	1.0024362396
1.1312357379	0.4727872663	0.9999537652	1.0583470253
2.7933974408	0.6948713406	0.9999537652	4.9527501949
4.5505976667	0.5557790267	0.9999537652	-2.1128552243
-5.855397775	0.1784713162	0.9999537652	1.0948274935
1.226418828	0.6030418361	0.9999537652	1.0388350981
0	0	0	2.996475627
1.2277185342	0.1824188169	0.9999537652	1.1514036385
2.7933974408	0.6948713406	0.9999537652	0
-3.2098360912	0.2194561564	0.9999537652	-3.2100972269
1.5666754327	0.2073705481	0.9999537652	1.0000053717
0	0	0	1.6293799659
1.4541620227	0.4030079103	0.9999537652	-1.0396375821
1.2282270925	0.1069261984	0.9999537652	-1.2059614783
1.1216701798	0.9024905083	0.9999537652	-2.5242480573
1.4966853826	0.0406323051	0.9999537652	1.4904357197
2.7933974408	0.6948713406	0.9999537652	-4.9085166009
1.0630169038	0.7980352334	0.9999537652	-1.0290824579
1.1657570779	0.6380548572	0.9999537652	-1.0194787956
2.7933974408	0.6948713406	0.9999537652	-1.4678409108
1.0953696186	0.6994629566	0.9999537652	-1.0086132003
-1.1066536636	0.637159935	0.9999537652	1.1294841325
1.0780589231	0.6102839093	0.9999537652	1.2824743169
-1.1914891517	0.6440303917	0.9999537652	1.2894061787
1.0341662743	0.8682172256	0.9999537652	-1.235209575
1.0958036305	0.6962249736	0.9999537652	1.1250879518
1.1648845007	0.6782502087	0.9999537652	-1.0059845002
2.3176808751	0.1511361171	0.9999537652	-1.1004010026
1.2296514654	0.3854503751	0.9999537652	1.0183366278
1.0502031099	0.7081473763	0.9999537652	1.182052162
-1.1660606425	0.4806484571	0.9999537652	1.0640205517
-1.257252732	0.5578208053	0.9999537652	1.1641030491
1.019709227	0.9120835078	0.9999537652	1.2810625488
-1.181058058	0.1602913154	0.9999537652	-1.4777514356
-1.0775866517	0.7626560154	0.9999537652	1.5384267179
1.0482883457	0.9108513709	0.9999537652	1.9089640068
-1.1247627666	0.5129949217	0.9999537652	-1.1916722484
-1.2937681179	0.3523178786	0.9999537652	-1.2552645749
9.8288704632	0.3801446171	0.9999537652	20.6004768562
8.064174035	0.4238584183	0.9999537652	2.996475627
-1.1973885425	0.6223115057	0.9999537652	-1.1245623719
-1.0320811722	0.9163865328	0.9999537652	1.6864724442
1.2243477866	0.3989164383	0.9999537652	1.4400275153
-1.3595888537	0.286470943	0.9999537652	1.2337887463
-1.1453192235	0.90863368	0.9999537652	2.996475627

1.0097610764	0.9489873233	0.9999537652	1.1942304835
1.0671227974	0.6142070784	0.9999537652	1.1407309908
4.8358890185	0.1515497563	0.9999537652	1.2122205562
1.0147943183	0.9628263527	0.9999537652	1.0097637779
-4.8113470503	0.1909570135	0.9999537652	2.996475627
0	0	0	2.996475627
4.5505976667	0.5557790267	0.9999537652	2.996475627
1.2386230889	0.1255224854	0.9999537652	1.2641887055
1.093864445	0.4587785786	0.9999537652	1.0640091118
-1.1282172077	0.319867596	0.9999537652	1.172517253
1.0721415024	0.797889512	0.9999537652	1.0480093295
1.1599951194	0.2441759415	0.9999537652	1.2409294568
-1.024945222	0.961405319	0.9999537652	1.2662202225
-2.7401415473	0.0335946413	0.9999537652	-1.0288598893
0	0	0	2.9985540909
-1.5977194628	0.0619682956	0.9999537652	-1.1394805455
-1.0200960129	0.9264959218	0.9999537652	-1.072360123
0	0	0	8.8700459102
0	0	0	2.996475627
-1.1591181112	0.7315349917	0.9999537652	1.43688214
-1.1470525509	0.4967696843	0.9999537652	1.2511869557
-1.0120874203	0.9143754091	0.9999537652	1.2144656448
-3.7889870009	0.1877974708	0.9999537652	-1.7179436304
-1.0945545484	0.7276537763	0.9999537652	-1.005141972
-1.1479586872	0.6540800855	0.9999537652	1.0718275142
-1.0649032174	0.7950463902	0.9999537652	-1.1037874173
0	0	0	2.996475627
0	0	0	6.9133480437
1.4121692786	0.4598497303	0.9999537652	1.251589115
-1.2503937844	0.4855207819	0.9999537652	1.3295911183
-1.0277332158	0.9045850826	0.9999537652	1.0360637903
1.072315984	0.7130219035	0.9999537652	1.0366931672
-1.0935358825	0.7047760561	0.9999537652	1.0083136811
-1.3999679996	0.619842988	0.9999537652	1.8828397305
1.2873581838	0.6012741898	0.9999537652	-1.060449365
-1.1457555741	0.9107036899	0.9999537652	-3.384595918
4.5505976667	0.5557790267	0.9999537652	2.2926838869
-1.0291509029	0.8649364559	0.9999537652	1.3985036623
2.7957660672	0.7054785537	0.9999537652	4.9527501949
-1.3534658719	0.3649693136	0.9999537652	1.2428621427
1.3284240629	0.2412215355	0.9999537652	1.190883589
-1.090127928	0.6367899306	0.9999537652	1.505970108
8.064174035	0.4238570822	0.9999537652	0
0	0	0	2.9988886918
1.0426434256	0.9178859138	0.9999537652	-3.9149299287
-1.1117491132	0.5960620239	0.9999537652	1.0664326982
-1.1667189197	0.5734744125	0.9999537652	1.5156054808
-1.4939074423	0.5814653819	0.9999537652	1.9724641275
1.581938964	0.1725918725	0.9999537652	-1.0167228594

-1.3265913034	0.25642191	0.9999537652	1.0226245303
1.274835312	0.0825801787	0.9999537652	1.3572240672
5.830173951	0.0008693	0.9999537652	7.0047613637
-1.2128930081	0.5810114982	0.9999537652	1.7792401468
1.0128253901	0.9544725541	0.9999537652	-1.0394286676
1.4970843977	0.3800284172	0.9999537652	-1.2380128721
-1.0963785573	0.6561807771	0.9999537652	1.1726758889
-1.1472577241	0.56832836	0.9999537652	1.1669034067
-1.5781637505	0.0544758363	0.9999537652	-1.4700185722
-1.1701353796	0.4270914334	0.9999537652	1.0184828998
-1.1554329234	0.4236991902	0.9999537652	1.0455699803
-1.0225240938	0.9033163355	0.9999537652	-1.3573965955
-1.5897551395	0.5041519522	0.9999537652	-1.03536236
1.1301085744	0.3428300293	0.9999537652	1.2781979416
-1.7296889495	0.0899361334	0.9999537652	1.4608079493
-1.3971220169	0.1464404855	0.9999537652	1.1648647615
1.1721379156	0.3124550372	0.9999537652	1.0068375466
2.7933974408	0.6948713406	0.9999537652	-2.0477227478
-1.5772041867	0.5821342937	0.9999537652	-4.9096724685
1.0525005316	0.7321242631	0.9999537652	1.071049518
-1.2746701478	0.3290082184	0.9999537652	1.6100948227
1.5416925639	0.1359844447	0.9999537652	1.1813723778
1.8724948748	0.1823530709	0.9999537652	-1.3782542405
-1.0593100271	0.8983578218	0.9999537652	-1.3186193604
2.3853098311	0.0925239217	0.9999537652	1.1914259774
-1.0345674465	0.8450774607	0.9999537652	-1.0221789233
-1.1915933216	0.4198020976	0.9999537652	1.0617305525
1.0622651212	0.8967139571	0.9999537652	-1.0250247976
-1.4554833063	0.0878411101	0.9999537652	-1.3414801701
11.5966493128	0.3442703122	0.9999537652	1.2444904498
-1.208950374	0.6757330523	0.9999537652	-1.4864452136
1.3009698924	0.3538392612	0.9999537652	-1.4662129266
6.3130691196	0.479828129	0.9999537652	-1.1237570386
1.9210510653	0.0007126	0.9999537652	1.0070932263
-1.0459972533	0.8893730994	0.9999537652	-1.3435578132
-1.2134586322	0.4514421661	0.9999537652	-1.3303007737
2.3345973606	0.4597015139	0.9999537652	1.9439006421
1.2526375214	0.8109072595	0.9999537652	-1.5161065424
-1.0735266303	0.7655820726	0.9999537652	-1.2092469414
1.3599527681	0.427986488	0.9999537652	-1.5424785877
-1.0573424428	0.947401199	0.9999537652	-2.0477227478
-1.407159314	0.5131905984	0.9999537652	1.1840049923
3.1464506577	0.007356	0.9999537652	-1.1851503263
-7.0341510274	0.112856345	0.9999537652	1.2418880989
1.5817184657	0.0333022222	0.9999537652	-1.5568004726
3.1789916386	0.1149607365	0.9999537652	-1.4858477425
1.0363156376	0.7462348157	0.9999537652	1.0216825372
-1.4738309326	0.6574410295	0.9999537652	1.4032791701
-1.3628220697	0.2264860216	0.9999537652	-1.4938691548

-1.2985700899	0.1496637568	0.9999537652	-1.3084929511
1.4988051422	0.4265082377	0.9999537652	1.3667968135
2.7957660672	0.7054809147	0.9999537652	-1.8594983184
-1.1540270862	0.4719495155	0.9999537652	1.1544734553
2.7933974408	0.6948713406	0.9999537652	1.0119943569
-1.2934488892	0.5817496794	0.9999537652	-1.0012214379
-1.3568049148	0.178599515	0.9999537652	-1.2578852935
-1.2075832828	0.4309250967	0.9999537652	-1.0028055611
1.0881828735	0.6583465298	0.9999537652	-1.2907676159
-1.1527550849	0.7616373463	0.9999537652	1.093627361
1.4900947652	0.5701339147	0.9999537652	1.2360867785
1.2150491088	0.6025413176	0.9999537652	-1.1554641156
-1.7008315477	0.5854966472	0.9999537652	-1.346567469
1.4584001952	0.0877356483	0.9999537652	1.0996443532
1.4539789531	0.3227438796	0.9999537652	-1.5668184312
-1.3095454841	0.1184677885	0.9999537652	-1.117737687
0	0	0	2.9940379764
-1.0284544651	0.8873635267	0.9999537652	-1.2831222052
-1.2958290233	0.1153035106	0.9999537652	-1.0958692865
-2.8772495082	0.1550334452	0.9999537652	-5.4365958443
-1.2341761556	0.1341893058	0.9999537652	-1.2730833474
1.8447991669	0.5983745009	0.9999537652	-1.525284329
0	0	0	10.818785458
1.0276173005	0.8508859163	0.9999537652	-1.1515558926
-1.1457555741	0.9106870632	0.9999537652	-2.2425086765
-1.5741996781	0.0237035578	0.9999537652	-1.1296531448
1.0725856843	0.6949095112	0.9999537652	1.265458727
-1.6171481755	0.1412947611	0.9999537652	1.0520866134
-1.3799140705	0.5648061566	0.9999537652	-1.3121459758
1.0335265875	0.8121779914	0.9999537652	-1.3453573596
0	0	0	6.908559683
-2.4387448463	0.3504473173	0.9999537652	1.8126139092
1.165055936	0.2640011957	0.9999537652	1.2998936869
-1.251442232	0.598584382	0.9999537652	-1.0472475037
-1.034919673	0.8191217985	0.9999537652	1.0666477789
-1.1923384106	0.312266677	0.9999537652	1.0016431758
1.0739475427	0.7639988624	0.9999537652	1.0219576039
1.0769451168	0.8200466101	0.9999537652	1.1995024763
-1.4213039175	0.0436527595	0.9999537652	-1.4438451596
1.5422457228	0.0864568713	0.9999537652	-1.5958934884
-1.1605962485	0.3000556362	0.9999537652	-1.1120629771
1.0635603534	0.6249434992	0.9999537652	1.1230512765
-3.8493430216	0.0701681674	0.9999537652	-1.146612601
-1.804850317	0.1863408573	0.9999537652	-1.112478096
2.7933974408	0.6948713406	0.9999537652	-1.1248877899
1.0223047989	0.9006774765	0.9999537652	-1.0194559099
-1.1201877812	0.4768445197	0.9999537652	-1.0604886123
2.7933974408	0.6948713406	0.9999537652	0
1.3005825753	0.1004791369	0.9999537652	1.0837383107

1.0679551318	0.8780915913	0.9999537652	-1.1527470941
0	0	0	2.9988886918
1.2092841404	0.6107975243	0.9999537652	-1.1007348853
-1.4577546906	0.0943985855	0.9999537652	1.0189001421
1.0582294747	0.6900225722	0.9999537652	1.0683064326
-1.0230998976	0.8775689152	0.9999537652	1.0206674925
-1.5875118025	0.719861829	0.9999537652	6.9133480437
1.6018881045	0.6925672651	0.9999537652	0
1.081716247	0.7850048533	0.9999537652	1.2537202461
0	0	0	1.2398837679
1.8388612766	0.4313999445	0.9999537652	1.6020963591
0	0	0	4.9527501949
9.8205505243	0.3805794228	0.9999537652	1.2610511029
-1.359568862	0.2013058479	0.9999537652	-1.0224986581
2.7957660672	0.7054832767	0.9999537652	0
-1.4590969081	0.2785047582	0.9999537652	-1.219770326
2.3721824376	0.1382318609	0.9999537652	2.3873555128
9.8205505243	0.3805564752	0.9999537652	0
-1.0426167916	0.7869568888	0.9999537652	1.0237213529
1.0080615151	0.9554696698	0.9999537652	-1.3476380248
0	0	0	6.908559683
20.3530564257	0.2434367	0.9999537652	-1.8961334203
2.8173722589	0.3578436447	0.9999537652	1.2776901668
1.6032957979	0.6855260959	0.9999537652	-2.78893139
1.1559682557	0.5290365509	0.9999537652	-1.0749183568
-6.6831020251	0.0205660771	0.9999537652	1.1594663864
-1.2360019544	0.0998451874	0.9999537652	-1.5170309296
1.0622188426	0.8508685919	0.9999537652	-1.3165243928
-1.0714436087	0.7269613943	0.9999537652	-1.1648210908
1.328316327	0.2183756282	0.9999537652	-1.0821626065
1.0181583546	0.9622829065	0.9999537652	1.7993897083
-1.2901307454	0.268381033	0.9999537652	-1.0876534639
1.0162977585	0.9413699657	0.9999537652	1.1130722193
1.3594099105	0.7970234108	0.9999537652	-1.5186976953
1.3588576256	0.8008310861	0.9999537652	-1.8649865122
-1.3836247056	0.4729035472	0.9999537652	-2.4343626033
-1.0352915919	0.8211469486	0.9999537652	-1.0920870391
-1.2934004406	0.124654583	0.9999537652	1.0089312664
1.0170091418	0.9342706739	0.9999537652	-1.0671527287
-1.0667613964	0.7116011844	0.9999537652	-1.2343447624
1.803487669	0.1804835454	0.9999537652	1.0523155062
-1.5251975786	0.3791708341	0.9999537652	-2.2366824481
9.8205505243	0.3805494011	0.9999537652	-1.3582733839
1.7849205255	0.001992	0.9999537652	1.1368261123
-1.2470641064	0.1720962981	0.9999537652	-1.1491488305
0	0	0	2.9985540909
1.487629049	0.1165691285	0.9999537652	1.2021067438
-1.262385007	0.3200298605	0.9999537652	-1.3239437915
-1.0632333232	0.5983990699	0.9999537652	1.0078447486

-1.3131980863	0.4572652733	0.9999537652	-1.276629097
-1.1990671425	0.3559144233	0.9999537652	-1.0735665224
1.3590426899	0.3761537995	0.9999537652	-1.2980233679
6.4674395546	0.070478346	0.9999537652	1.8680940039
-1.0726072054	0.8100611604	0.9999537652	-1.1744467349
1.3150458572	0.1022277307	0.9999537652	-1.1286400813
-1.3186076808	0.4265578473	0.9999537652	-2.8193076484
-1.1396208367	0.544703296	0.9999537652	1.2787018343
23.8818846228	0.2167390634	0.9999537652	1.7563703072
-1.5211372542	0.3665068344	0.9999537652	-1.4506341495
-1.1335575491	0.4941690353	0.9999537652	1.12339959
-1.0182486212	0.9147780646	0.9999537652	-1.1241980094
6.3075231393	0.4857492078	0.9999537652	0
1.7158683406	0.3360161316	0.9999537652	-1.0497978916
1.0920240419	0.62215233	0.9999537652	1.1422894243
1.4469375885	0.4025877709	0.9999537652	1.1218054119
1.1216416516	0.901210313	0.9999537652	-1.5838380262
1.0893117919	0.6576163512	0.9999537652	-1.2865437113
-1.3154333204	0.6439373177	0.9999537652	1.3233841675
-1.3894082558	0.1666911894	0.9999537652	-1.3309752956
1.1693375575	0.4579439293	0.9999537652	-1.1578602813
-1.0693351968	0.7865455634	0.9999537652	-1.6695905654
1.2406947549	0.6664970513	0.9999537652	-1.7505240547
1.4884660645	0.025441515	0.9999537652	-1.5205260412
1.1698595601	0.3888229725	0.9999537652	-1.3292850387
6.3075231393	0.4857263905	0.9999537652	2.996475627
-1.1165792233	0.5418866877	0.9999537652	-1.025219763
1.3600088454	0.7955072923	0.9999537652	-2.1727063722
1.32927784	0.0995955705	0.9999537652	-1.0906901074
1.1399586414	0.3576059174	0.9999537652	1.1838622933
1.4222577311	0.1942158082	0.9999537652	1.5004387955
1.3366365121	0.3625361118	0.9999537652	1.0847390348
-1.1066010742	0.7141809449	0.9999537652	-1.0567885824
2.7933974408	0.6948713406	0.9999537652	-9.7554110723
1.8642987684	0.2497673143	0.9999537652	1.7999600779
1.0117578102	0.9281820982	0.9999537652	-1.1964733969
1.0717622344	0.720230473	0.9999537652	-1.0306774712
1.1059891928	0.6371327211	0.9999537652	1.0680966286
9.1237371271	0.0400749476	0.9999537652	-1.47512839
1.6012712676	0.002076	0.9999537652	-1.1473529684
-1.3040401656	0.1204523164	0.9999537652	-1.4761737229
3.7983224081	0.0271273739	0.9999537652	2.2286307361
1.1224886231	0.6927056118	0.9999537652	-1.2345037938
5.3460178673	0.0491827768	0.9999537652	1.0024930783
13.3324809638	0.3194026856	0.9999537652	1.0485154464
-1.0623931073	0.7766317106	0.9999537652	-1.0302681103
1.2127056579	0.1569705905	0.9999537652	-1.2231189272
-1.3357532129	0.2566369982	0.9999537652	-1.773248014
-1.2470493566	0.4600096806	0.9999537652	-1.5713887581

-1.1761764882	0.796154256	0.9999537652	1.0417527966
0	0	0	9.7116702472
15.2205810263	0.3049327708	0.9999537652	-18.5461680391
-1.719523697	0.1597657386	0.9999537652	-1.4430717011
-1.0459359664	0.7393024577	0.9999537652	-1.2587157308
1.1304253447	0.5593153775	0.9999537652	-1.105511541
-1.4523386343	0.2552905152	0.9999537652	-1.1224285761
-1.0235715085	0.9403831093	0.9999537652	-1.2798035639
1.1331001403	0.4993180178	0.9999537652	-1.0167621156
15.0880352532	0.2955892438	0.9999537652	1.2661429145
-1.4845108143	0.004804	0.9999537652	-1.0570591659
2.551628948	0.0212107716	0.9999537652	-1.2359445222
1.6380420288	0.0162382303	0.9999537652	-1.1069850529
1.0546898215	0.6857088037	0.9999537652	1.1628932732
1.104017262	0.7319083332	0.9999537652	-1.7981194791
2.9491984565	0.2037967334	0.9999537652	1.8700278501
0	0	0	-5.3608259368
20.3530564257	0.2433991055	0.9999537652	1.5320004571
-1.0405986745	0.8795818362	0.9999537652	1.0923007845
1.0472330956	0.8774829467	0.9999537652	1.2640989948
-1.4521928607	0.040412784	0.9999537652	-1.0797982511
4.5505976667	0.5557790267	0.9999537652	-1.9282391141
-1.0835233486	0.6348122059	0.9999537652	-1.1591135058
-1.7980431703	0.0721008648	0.9999537652	-1.4046391392
0	0	0	-1.4683576122
1.0863607976	0.7979072626	0.9999537652	-1.20600614
4.3306479182	0.1259977229	0.9999537652	-1.4734213639
-1.0288673451	0.9635854798	0.9999537652	1.3864346485
1.0448267999	0.7163906599	0.9999537652	-1.2959607117
1.0939814099	0.6994922539	0.9999537652	1.287732095
1.1034701615	0.766625649	0.9999537652	1.2532707225
1.1070542251	0.6067476999	0.9999537652	1.0580775672
-1.1453192235	0.9086163647	0.9999537652	1.6033828985
-1.1866217015	0.453124199	0.9999537652	-1.1553644184
-1.4738309326	0.657352028	0.9999537652	-7.059407508
1.5527095675	0.0578129006	0.9999537652	-1.3741477036
1.1236950163	0.8892660819	0.9999537652	-1.239649121
5.2421556375	0.1294811357	0.9999537652	-1.4903968027
-1.3368210118	0.1112946434	0.9999537652	-1.7405947593
-1.4311420977	0.4698846268	0.9999537652	-1.0565165766
-1.0495538285	0.8734987538	0.9999537652	-1.0540403849
2.2292907561	0.1350976065	0.9999537652	-1.3886635535
-1.0623578347	0.8174313755	0.9999537652	1.927155001
1.2161583754	0.4521825624	0.9999537652	-1.1494026898
4.4194285245	0.0343611582	0.9999537652	1.0034864765
1.0377531271	0.8857993072	0.9999537652	1.2124048895
-1.8043832832	0.1834550445	0.9999537652	1.1771673362
-1.286542464	0.552696716	0.9999537652	-1.0145744028
-1.25679892	0.1932593429	0.9999537652	-1.0238543449

-1.7061631951	0.512245813	0.9999537652	2.015179835
1.1137797095	0.4690510281	0.9999537652	-1.0131434984
-1.0578275344	0.7694535403	0.9999537652	-1.0725094354
1.3600088454	0.7960314898	0.9999537652	-4.7721736557
0	0	0	1.8383591193
1.1477192382	0.2576752983	0.9999537652	1.0643932097
1.0431215243	0.8584058197	0.9999537652	-1.2314243146
9.8291821523	0.3810792211	0.9999537652	1.1113292551
0	0	0	-1.6173574324
-1.2291604513	0.2864529338	0.9999537652	-1.0996644369
1.0374523201	0.9211744551	0.9999537652	1.2410887995
2.4311635089	0.061472287	0.9999537652	1.2829621228
-1.0295564388	0.9447503079	0.9999537652	-1.1722893862
1.1936037065	0.4672240491	0.9999537652	-1.1334585605
0	0	0	0
-1.2403103001	0.6468437841	0.9999537652	-1.6164179914
-1.3533560557	0.1822975248	0.9999537652	1.2060550334
1.2004094194	0.3690636639	0.9999537652	1.3335506904
0	0	0	1.266587837
-1.0490138295	0.7628811996	0.9999537652	-1.1003804534
1.1242072872	0.8664818374	0.9999537652	1.1334361866
1.1251860686	0.8659175242	0.9999537652	-1.1703505369
1.145590572	0.411043483	0.9999537652	-1.0439053235
6.3128691894	0.481370604	0.9999537652	21.8277395785
-1.3136730568	0.3696628686	0.9999537652	-1.8047914737
0	0	0	0
1.1762937518	0.6378251229	0.9999537652	1.1216960639
-1.0520168673	0.8514154958	0.9999537652	-1.4206729006
-1.0129021342	0.9710579648	0.9999537652	1.0244662873
0	0	0	22.9814455074
-4.3267800353	0.196360431	0.9999537652	-4.1081049688
1.2376801656	0.2302393292	0.9999537652	1.1417793181
-1.5170894827	0.2534339765	0.9999537652	-1.042069914
1.3637911166	0.1707045677	0.9999537652	1.2241968907
1.3036454842	0.7494629913	0.9999537652	4.8885934785
1.9936796515	0.0714485827	0.9999537652	-1.38196012
0	0	0	2.996475627
-1.5073464892	0.3210055756	0.9999537652	1.0302658554
1.1650335588	0.5902805278	0.9999537652	-1.1055173815
1.6807324819	0.4288357994	0.9999537652	1.0352991504
-1.1946343414	0.263872796	0.9999537652	-1.5006053242
-1.2354060772	0.3580659043	0.9999537652	1.0784520948
4.20605194	0.2970301065	0.9999537652	-1.2130610766
-2.0240465997	0.1687931397	0.9999537652	1.5123239872
-1.0086708751	0.9900522166	0.9999537652	-1.4126903528
1.2883472386	0.7181160133	0.9999537652	-1.4102941558
-1.3306718218	0.1051930339	0.9999537652	-1.2347059899
-4.3214691297	0.1498731208	0.9999537652	4.0161574689
-1.3685167982	0.7411237957	0.9999537652	2.6421916756

-1.120333337	0.5927805309	0.9999537652	-1.0710698321
1.0470307226	0.743670398	0.9999537652	-1.463306182
-1.2682791024	0.3070792828	0.9999537652	-1.3630524875
55.3905770121	0.1165454776	0.9999537652	1.6447327969
-1.311903025	0.380659926	0.9999537652	-1.106327684
-1.1300927121	0.8385744323	0.9999537652	1.0085110121
1.9875802815	0.1403839349	0.9999537652	-1.1791410574
-1.1383391077	0.3801249348	0.9999537652	1.0578530355
-1.3272048102	0.2123362942	0.9999537652	1.1137995697
0	0	0	2.9985540909
1.1281050728	0.4115895632	0.9999537652	-1.2412266682
4.5505976667	0.5557790267	0.9999537652	4.9527501949
1.0358891855	0.9686167954	0.9999537652	1.08782678
0	0	0	2.996475627
1.0004930433	0.998993079	0.9999537652	-1.2559840346
1.0167789469	0.9443360871	0.9999537652	-1.5497537206
3.3087541388	0.2716723318	0.9999537652	1.5500113516
-1.0515537329	0.8044856143	0.9999537652	-1.1308884522
1.2614216143	0.0601372921	0.9999537652	-1.293448118
-1.2746614738	0.1606763397	0.9999537652	-1.343514666
-1.4083859417	0.1219964968	0.9999537652	-1.0150048583
0	0	0	4.9527501949
1.1843584263	0.4939920952	0.9999537652	-1.0187531996
1.1662839454	0.5812564947	0.9999537652	-1.1073929618
1.8511995871	0.001362	0.9999537652	1.1387724476
-1.2885166979	0.2097972531	0.9999537652	-1.5979015063
1.1485985637	0.463638587	0.9999537652	-1.2338173939
1.2474504541	0.2814024272	0.9999537652	1.0274012556
1.4033976605	0.1585237062	0.9999537652	-1.0264917348
-1.1791115305	0.6931894239	0.9999537652	1.6457190488
-1.0959611736	0.544670553	0.9999537652	-1.268436564
1.3640825792	0.4394251463	0.9999537652	-1.0725835009
-1.2702086188	0.5106842361	0.9999537652	1.0639511794
1.0517213946	0.7251277381	0.9999537652	1.1020028167
1.6071434688	0.0321082374	0.9999537652	1.4847501504
1.1719379916	0.7833002549	0.9999537652	-1.2480287389
-1.3012101317	0.3635409955	0.9999537652	-1.2585399598
2.6895092879	0.2385949016	0.9999537652	1.4524556074
1.8705325496	0.4019540183	0.9999537652	-1.892635601
1.179616791	0.509223424	0.9999537652	1.0724299226
8.064174035	0.423855746	0.9999537652	0
1.1200919063	0.4592957806	0.9999537652	-1.3477369094
-1.2595693104	0.287632038	0.9999537652	-1.161452869
1.9059187914	0.4672046374	0.9999537652	-1.1140665736
-1.1457555741	0.9106704818	0.9999537652	-1.2697820089
1.0282505514	0.8757687512	0.9999537652	-1.0851625246
-1.3137997922	0.2059123605	0.9999537652	1.0281362136
0	0	0	0
3.3080470333	0.0608573319	0.9999537652	-2.3074879621

0	0	0	2.9972756496
9.8372048419	0.3843062409	0.9999537652	4.0566323706
-1.1301523119	0.6636493577	0.9999537652	-1.1704766606
-1.7345363658	0.4193767725	0.9999537652	1.1376282384
-1.0586229118	0.7711485084	0.9999537652	-1.8139138372
2.6488226131	0.1941596219	0.9999537652	2.207345736
1.3473071656	0.0748946907	0.9999537652	-1.0057254017
1.0826140142	0.8001200143	0.9999537652	1.5016244389
2.7933974408	0.6948713406	0.9999537652	0
0	0	0	12.7732027325
1.0459041815	0.7933892395	0.9999537652	-1.58328359
0	0	0	2.9985540909
1.5326933747	0.4164263986	0.9999537652	-1.2236066266
1.6046040403	0.6917707948	0.9999537652	-2.1769706842
1.122660888	0.4718890446	0.9999537652	-1.4579593921
39.7041008618	0.1475991552	0.9999537652	-2.1312910006
1.3028815963	0.1823035632	0.9999537652	-1.5651416058
1.3723866226	0.2145749054	0.9999537652	-1.0285542219
-1.2614698103	0.5023397603	0.9999537652	-1.1135315466
-1.0132266269	0.9585522246	0.9999537652	-1.0098569271
1.1283570835	0.8121235041	0.9999537652	-1.0581769348
1.2923449892	0.4693123496	0.9999537652	-1.1155336866
-1.2521622905	0.5918397809	0.9999537652	-1.0139659426
2.7957660672	0.7054856396	0.9999537652	0
1.1396036817	0.5664677148	0.9999537652	-1.0620047471
1.1881984225	0.4683083382	0.9999537652	-1.057108781
-1.2976669841	0.1058410483	0.9999537652	-1.0947358968
1.0554825372	0.8714353228	0.9999537652	1.2517152158
-3.2071184374	0.2125790761	0.9999537652	-2.2054639418
-1.6888941245	0.0595993437	0.9999537652	-1.5459255313
-4.1003030613	0.1495902562	0.9999537652	16.7153157555
1.1010789506	0.849421635	0.9999537652	-1.0951465634
1.7563124292	0.0750090928	0.9999537652	-1.1621660762
1.244365816	0.627586769	0.9999537652	1.4081574407
1.2944710075	0.3033174	0.9999537652	1.239900045
2.1937105913	0.315831261	0.9999537652	1.2253415563
1.1658942226	0.8143072371	0.9999537652	1.1001459919
-1.3633704643	0.1651451195	0.9999537652	-1.008350153
2.7933974408	0.6948713406	0.9999537652	8.8639046058
1.1176349168	0.6745296837	0.9999537652	-1.1535515591
-1.2924310654	0.421361771	0.9999537652	-1.0035198717
-1.1756105772	0.2821831644	0.9999537652	-1.0321947226
1.2838351741	0.6937459471	0.9999537652	-1.0646292152
-1.5875118025	0.7198285331	0.9999537652	1.2400291296
-1.2364669196	0.1702432465	0.9999537652	-1.3865126451
1.2657827949	0.0458097196	0.9999537652	-1.3449928239
2.8188879029	0.1115304891	0.9999537652	-1.1485760228
-1.1082002784	0.8140595727	0.9999537652	-1.2161300368
8.064174035	0.4238544098	0.9999537652	-1.3188442921

-1.0726198358	0.8440422844	0.9999537652	-1.2263715504
-1.214909249	0.2806375652	0.9999537652	-1.1537940212
1.1608701596	0.3962550227	0.9999537652	1.9530523656
1.3965847999	0.6273554772	0.9999537652	1.3160484734
-1.4285065007	0.1823650738	0.9999537652	1.185016327
2.7933974408	0.6948713406	0.9999537652	0
1.1426607954	0.7138442235	0.9999537652	-1.2979840495
-1.1423811041	0.6040181185	0.9999537652	1.2963173426
1.3588921917	0.7999082067	0.9999537652	1.2307342269
-1.3819104686	0.4575578992	0.9999537652	1.0632770748
-1.0961023794	0.6309564832	0.9999537652	-1.2428521456
2.3808946632	0.1196842596	0.9999537652	1.0591380176
1.1212430665	0.9123543124	0.9999537652	1.2197594041
-1.1688282056	0.3046970341	0.9999537652	-1.1669492991
-1.0165452602	0.9322961596	0.9999537652	1.2216942647
1.0719363028	0.8021190726	0.9999537652	1.0149268118
4.5135922652	0.0252168205	0.9999537652	-1.1395652107
8.885375379	0.0450586252	0.9999537652	-1.9069868573
1.325537838	0.0311560868	0.9999537652	1.2043603349
-1.0846227732	0.69933287	0.9999537652	-1.6060557994
-2.2639689735	0.1175515285	0.9999537652	-1.2555829581
2.0591268911	0.0081	0.9999537652	-1.3581570118
-1.0659362063	0.8861765174	0.9999537652	-1.2044940791
-1.1075535652	0.7166205661	0.9999537652	1.5088721074
1.0525803912	0.7827430016	0.9999537652	1.0227684358
1.4472430402	0.1968863502	0.9999537652	1.5302836452
2.0146502341	0.3727411799	0.9999537652	2.5921936947
-1.0712832442	0.7331085269	0.9999537652	1.4854659655
-1.2927168086	0.5077315697	0.9999537652	-1.3287293093
1.0168454914	0.9421374219	0.9999537652	1.3552929062
1.3619023403	0.0975713433	0.9999537652	1.2869908174
1.1486721533	0.5292648487	0.9999537652	1.5763814567
-1.1608398396	0.4630036979	0.9999537652	1.1509275924
6.3075231393	0.4857035612	0.9999537652	-2.8188010609
-1.0809832252	0.6392738664	0.9999537652	1.0181345881
-1.0567421345	0.7525498655	0.9999537652	-1.0801969844
1.4947645522	0.2206917176	0.9999537652	1.421428663
0	0	0	2.6434024479
-1.6658819164	0.4333457971	0.9999537652	1.1687933144
9.8205505243	0.3805423413	0.9999537652	1.5337522997
-1.0654590622	0.848148443	0.9999537652	-1.2353970623
18.5983228161	0.2579893867	0.9999537652	1.0281495543
-1.0025611851	0.9887195812	0.9999537652	1.5395556125
6.3075231393	0.4856807199	0.9999537652	2.8230593868
0	0	0	1.6293799659
2.7933974408	0.6948713406	0.9999537652	2.9985540909
2.6385886072	0.234634825	0.9999537652	-5.1602685354
-1.0025576653	0.9898298049	0.9999537652	2.1215014107
15.0880352532	0.2953123792	0.9999537652	-1.1241083699

1.4165391913	0.4168444074	0.9999537652	1.1963816205
-1.0966804838	0.7781248117	0.9999537652	1.0738289762
-1.0030849643	0.990485157	0.9999537652	1.1828444467
-1.2840126527	0.2064140148	0.9999537652	1.5502778448
1.7731057898	0.2467864476	0.9999537652	1.826300294
-1.301307353	0.3526377344	0.9999537652	-1.1743862213
0	0	0	2.996475627
1.0202654252	0.9471846565	0.9999537652	-1.1004435788
1.0997561902	0.7065568568	0.9999537652	1.1430204839
1.4922045073	0.1721897354	0.9999537652	1.2682682166
1.1162834585	0.9272419326	0.9999537652	-2.2257756135
1.0233315722	0.8984237065	0.9999537652	-1.0066325084
4.5122462652	0.0159171321	0.9999537652	1.0531903154
-1.7073157204	0.1543298723	0.9999537652	-1.1032944191
1.254032753	0.2421399241	0.9999537652	1.2295953012
-1.4927489467	0.0499075931	0.9999537652	1.2796089419
1.168006364	0.6600949886	0.9999537652	-1.4624495673
1.193935423	0.7914697934	0.9999537652	1.2273656619
-1.2552570644	0.3679937521	0.9999537652	1.5137526073
-1.1530470734	0.4376346138	0.9999537652	1.1240373259
1.0970727976	0.7737662306	0.9999537652	1.4164151306
-1.0097465734	0.9844020815	0.9999537652	-1.175553458
1.2765905664	0.5564131676	0.9999537652	1.1886228911
1.4514413853	0.5194901015	0.9999537652	1.2946642925
-1.5881570201	0.7077577411	0.9999537652	2.9985540909
-1.586167124	0.7108092302	0.9999537652	-3.3826461929
2.7933974408	0.6948713406	0.9999537652	6.908559683
0	0	0	2.9985540909
2.7933974408	0.6948713406	0.9999537652	2.996475627
1.2707369491	0.5336904993	0.9999537652	-1.1280160395
-1.1892662586	0.3612950794	0.9999537652	1.0213588522
-1.1575124287	0.3546859617	0.9999537652	1.3582638358
1.8447991669	0.5971013514	0.9999537652	4.9527501949
8.064174035	0.4238530733	0.9999537652	0
-1.5739807169	0.4890192501	0.9999537652	15.9061931258
9.8205505243	0.3804768107	0.9999537652	1.2398837679
2.6785550839	0.0002617	0.9999537652	1.6052999973
5.0040817115	0.1271426994	0.9999537652	2.6071338396
-1.1153600471	0.4629429261	0.9999537652	1.0742892442
-1.477038777	0.3702340459	0.9999537652	1.2827232261
1.4689054779	0.3802040161	0.9999537652	-1.4259261512
-1.0506650712	0.9120194616	0.9999537652	2.4592702872
1.2893688746	0.7076926618	0.9999537652	-1.019441536
1.0512666838	0.8891775199	0.9999537652	1.3192582085
1.6571181455	0.5555300284	0.9999537652	1.3055636456
-1.0338647544	0.8794896999	0.9999537652	1.5598415203
1.3368672143	0.3757451387	0.9999537652	1.0195338947
-1.5118183191	0.131020967	0.9999537652	-1.1685149838
2.4930331421	0.1076191604	0.9999537652	2.6831117316

-1.1189442889	0.7151141203	0.9999537652	1.7164978104
2.7933974408	0.6948713406	0.9999537652	4.9527501949
1.128245903	0.5500772122	0.9999537652	1.2959250467
-1.4276018648	0.0584723328	0.9999537652	-1.1164029876
1.0952012099	0.7716644543	0.9999537652	1.0927825172
1.2329510555	0.3608389314	0.9999537652	1.0458716648
-1.2920422915	0.5687702546	0.9999537652	1.2098319596
-2.0574409626	0.3046858164	0.9999537652	1.1527804599
-1.0038011802	0.9850343491	0.9999537652	1.0217813369
1.3475167769	0.4483815813	0.9999537652	1.0191573106
-1.1169034937	0.5605936878	0.9999537652	1.5926577405
-1.0837104855	0.6274159951	0.9999537652	1.4286121917
4.5505976667	0.5557790267	0.9999537652	1.1414910892
1.1258459376	0.8229052205	0.9999537652	-1.0290718605
-1.1853870477	0.3570105659	0.9999537652	1.0335562564
-1.0867616237	0.8051038887	0.9999537652	-1.2748562641
-1.6015602529	0.2157162929	0.9999537652	-1.002102582
1.5116429066	0.2807435933	0.9999537652	1.4665139098
0	0	0	2.996475627
0	0	0	2.9985540909
6.3075231393	0.4856578666	0.9999537652	1.26863809
2.375760489	0.2568951151	0.9999537652	2.0817128167
-1.2001894462	0.3218514376	0.9999537652	1.3357143938
1.0985261247	0.6780779652	0.9999537652	1.3199288051
1.0734694883	0.5939941365	0.9999537652	1.088456562
-1.0973034006	0.705984831	0.9999537652	1.3851160313
2.7149220237	0.3431876162	0.9999537652	-1.0439154738
-4.8095156671	0.2070002691	0.9999537652	0
18.5983228161	0.2580226347	0.9999537652	-1.6622355843
1.2217604276	0.4192622078	0.9999537652	1.3874055683
0	0	0	2.996475627
2.5569228903	0.2910472058	0.9999537652	1.1434777493
1.1241786901	0.8746946646	0.9999537652	-1.2129116771
-6.9764533263	0.2593113237	0.9999537652	12.7732027325
1.1453538527	0.5868607512	0.9999537652	1.1715936728
1.7223668512	0.2043971082	0.9999537652	-1.1449270845
0	0	0	1.9432271302
1.3014784288	0.7466097159	0.9999537652	-1.3375799456
1.277202723	0.6580279331	0.9999537652	-1.099788524
1.384485804	0.3194854382	0.9999537652	1.4777590678
-1.054141025	0.791556286	0.9999537652	1.0967061151
8.0781087093	0.4256693294	0.9999537652	-3.3826461929
1.1036431645	0.7504142811	0.9999537652	-1.3360055468
-1.3374992129	0.2467680636	0.9999537652	-1.0330152676
1.0564364664	0.8361101924	0.9999537652	1.1511899953
-1.3016661117	0.0427920737	0.9999537652	-1.1656863606
1.4282553643	0.3752054851	0.9999537652	-1.205290762
3.5458425332	0.2619565568	0.9999537652	-1.0582880019
1.0525913883	0.8841841245	0.9999537652	1.3646401747

1.2603880587	0.5336691305	0.9999537652	-1.1308094393
2.087766184	0.5276959119	0.9999537652	1.1729977978
1.233187847	0.6143792904	0.9999537652	-1.4705270114
6.3075231393	0.4856350014	0.9999537652	0
9.8288704632	0.3801346658	0.9999537652	2.6930891478
1.2451745823	0.4931042106	0.9999537652	-1.1251704249
0	0	0	16.6921882404
2.7933974408	0.6948713406	0.9999537652	0
4.5505976667	0.5557790267	0.9999537652	-5.3583704783
4.5505976667	0.5557790267	0.9999537652	0
1.5096749162	0.0906418615	0.9999537652	-1.2300293279
-1.1535860236	0.2573459755	0.9999537652	-1.0528838308
-1.8046806307	0.005882	0.9999537652	-1.0477872221
-1.3759108811	0.4737600891	0.9999537652	-1.3213877873
41.1815769185	0.1509716372	0.9999537652	2.6746613342
1.1334417217	0.6264088654	0.9999537652	1.2138833754
-2.587194901	0.5006847496	0.9999537652	-2.0714095881
1.0101987241	0.9392972628	0.9999537652	-1.0053744114
1.3240315559	0.5504599042	0.9999537652	-2.3359348394
1.7498891159	0.456032818	0.9999537652	-1.9178478095
2.7933974408	0.6948713406	0.9999537652	0
0	0	0	6.9133480437
-1.0907974023	0.8464360189	0.9999537652	1.2631811856
-1.0669022223	0.671507895	0.9999537652	1.4759401581
-1.2034385477	0.2859629469	0.9999537652	-1.1601433853
-1.2764424044	0.3707513532	0.9999537652	-1.0200578279
1.367060712	0.0617729359	0.9999537652	1.1255925107
-1.0470651298	0.9033337993	0.9999537652	1.1840172299
1.5401242945	0.0662567996	0.9999537652	-1.0677041408
-1.0959123468	0.5978645791	0.9999537652	-1.1482094461
1.2979308881	0.3638972584	0.9999537652	1.0354676769
1.0717588054	0.8565093837	0.9999537652	-1.3403308597
1.4647892141	0.4952985584	0.9999537652	1.0252934474
-1.3073678212	0.5824949814	0.9999537652	-1.3425058873
1.0798498775	0.7188689027	0.9999537652	-1.3442141121
-1.3365524418	0.3160480326	0.9999537652	-1.2530812186
-2.1293015853	0.3365392143	0.9999537652	1.1239199258
-1.2455119556	0.4837074263	0.9999537652	1.0808254817
-1.0962031519	0.6348632801	0.9999537652	-1.2451599711
2.7933974408	0.6948713406	0.9999537652	0
-1.0941301256	0.8501844533	0.9999537652	1.389359736
-1.0077008142	0.9743676258	0.9999537652	1.1676660251
1.2013224142	0.3416661239	0.9999537652	1.0038000571
1.0428108136	0.7744666013	0.9999537652	-1.1274505155
3.0602334741	0.3286200346	0.9999537652	0
2.614036232	0.1497598131	0.9999537652	1.3464022699
1.1365752596	0.2585398368	0.9999537652	1.3206932155
1.1339005076	0.574440775	0.9999537652	-1.2869957595
-2.5861438062	0.4687294343	0.9999537652	4.9527501949

1.091863293	0.8475944358	0.9999537652	-1.3306765214
1.7057258576	0.2354668158	0.9999537652	1.0563444999
0	0	0	0
1.0694044834	0.801499651	0.9999537652	-1.0204507389
1.0111288402	0.9822893092	0.9999537652	-1.2323826996
2.2962279822	0.3617210307	0.9999537652	1.1248592401
1.9889507548	0.0244790145	0.9999537652	-1.2090681484
-1.7505776912	0.4855268788	0.9999537652	1.840905247
16.8581089812	0.2737927009	0.9999537652	-2.0456148517
-1.1512122706	0.7742336184	0.9999537652	-1.0320395109
1.0918330848	0.873110139	0.9999537652	-1.4306553732
2.3253723069	0.1077908736	0.9999537652	-1.0461084318
1.089873767	0.7935337567	0.9999537652	-1.2936428674
-1.0610809001	0.7869883638	0.9999537652	1.061315052
9.8205505243	0.3804117652	0.9999537652	-1.8438619278
2.7933974408	0.6948713406	0.9999537652	-1.3092040421
-1.0824588844	0.5438907228	0.9999537652	-1.095329875
1.0288019279	0.857141477	0.9999537652	1.119630364
-1.0026864056	0.9909828381	0.9999537652	1.0696324998
5.5687238427	0.0418949521	0.9999537652	-4.1578342229
2.7933974408	0.6948713406	0.9999537652	0
1.1202036844	0.8945170799	0.9999537652	-1.1230580054
-1.2314081917	0.326162262	0.9999537652	1.0664169111
-2.2171247351	0.0896581548	0.9999537652	1.1914030435
0	0	0	4.0583010578
-1.3769796102	0.2384897627	0.9999537652	1.1205589412
3.0654152135	0.31441189	0.9999537652	-1.1698173577
-1.1485172546	0.4578692467	0.9999537652	1.2301513321
1.3656652675	0.2506135958	0.9999537652	-1.2687612391
1.1256336023	0.8815811281	0.9999537652	1.1021486217
1.075295779	0.7274758405	0.9999537652	-1.1283010443
-1.1414561101	0.4911634641	0.9999537652	1.0764623523
1.0719638578	0.7272870224	0.9999537652	1.6039923611
1.0036584035	0.9834417829	0.9999537652	1.1641662271
3.857717517	0.1263654983	0.9999537652	1.0441854119
1.0948276309	0.8697849337	0.9999537652	1.7255265328
-1.4178951235	0.1833840602	0.9999537652	-1.0735789564
2.7933974408	0.6948713406	0.9999537652	0
1.1240347019	0.8993767546	0.9999537652	1.6011715681
1.361463763	0.2709169206	0.9999537652	-1.4730549859
1.8996613299	0.1525785602	0.9999537652	1.186876449
1.20323409	0.1586628689	0.9999537652	1.0393344568
0	0	0	14.7373490367
-1.0155063475	0.9297137652	0.9999537652	1.0574108242
-1.0241779203	0.9450431611	0.9999537652	-1.0286221032
1.2547148158	0.480972848	0.9999537652	1.1526808356
-1.0652308948	0.740151293	0.9999537652	1.0429885079
1.3577288541	0.0928306361	0.9999537652	-1.3057352681
1.0669316331	0.8444982579	0.9999537652	-1.182886298

-2.0921616211	0.4099979478	0.9999537652	-1.3783320433
-4.8135938501	0.1785433054	0.9999537652	2.996475627
0	0	0	2.996475627
-1.3487089989	0.1851800659	0.9999537652	-1.0623795456
1.9758702874	0.0909947869	0.9999537652	1.7216300277
3.3101061658	0.3001735128	0.9999537652	-2.2790859482
-1.308886814	0.3794972507	0.9999537652	1.2334916476
-1.1300770692	0.5036448098	0.9999537652	-1.1079122308
-1.1233545595	0.6912390092	0.9999537652	-1.0337037416
-1.2254908816	0.3804892175	0.9999537652	-1.0144695311
-1.0220720069	0.929956001	0.9999537652	1.2543863681
8.064174035	0.4238517368	0.9999537652	-9.7542681481
-1.0014866836	0.9922884507	0.9999537652	-1.0406740712
1.0551311209	0.835728015	0.9999537652	-1.043378998
1.2266013804	0.7027208519	0.9999537652	-1.2363407481
-1.6062758565	0.3081292775	0.9999537652	-1.5876369064
1.118076533	0.4769363522	0.9999537652	-1.1376943547
-1.5701247492	0.2556389836	0.9999537652	-1.0210562873
-1.7280698657	0.4641170891	0.9999537652	-1.1617246056
0	0	0	-1.859280336
-1.7184794315	0.007938	0.9999537652	-1.2409760363
-1.2169981524	0.5097245994	0.9999537652	1.2216468823
1.637293124	0.2381534342	0.9999537652	-1.2846156591
4.5505976667	0.5557790267	0.9999537652	1.2400291296
-1.0197370871	0.8655374487	0.9999537652	1.069599718
-1.7329433723	0.1811641231	0.9999537652	-1.1815807982
1.3045611542	0.498670192	0.9999537652	1.0387679491
-1.2812453468	0.1580630315	0.9999537652	-1.1068047103
1.1123641806	0.7591573151	0.9999537652	-1.3543574793
1.0275816656	0.9173327923	0.9999537652	1.055996581
1.1425966877	0.494547478	0.9999537652	1.2039997795
1.8094157661	0.0600136193	0.9999537652	1.1667470542
9.8205505243	0.380401755	0.9999537652	-1.8677891537
-1.082876609	0.6760678528	0.9999537652	-1.3763058259
9.8205505243	0.3804054104	0.9999537652	-1.4769135829
9.8288704632	0.3801247182	0.9999537652	-1.1248877899
-2.1684921399	0.00255	0.9999537652	-1.0472178763
4.5505976667	0.5557790267	0.9999537652	-1.5096535101
-1.3778945252	0.0493259228	0.9999537652	-1.4099440408
1.2354125676	0.2570531061	0.9999537652	1.2309295889
2.8612976293	0.0720659659	0.9999537652	1.014511427
-1.0383470747	0.9549513126	0.9999537652	-4.2646994388
1.7487353185	0.2407377046	0.9999537652	-1.4384357174
-1.1403285666	0.3312190561	0.9999537652	-1.1037515255
-1.2894344198	0.2654411135	0.9999537652	1.014648994
1.0290346229	0.8076122934	0.9999537652	-1.1508101262
1.106085976	0.7481836812	0.9999537652	-1.1933853856
1.1276320247	0.5426791216	0.9999537652	1.1587921414
-1.1470586676	0.4175020133	0.9999537652	-1.0842399285

2.7933974408	0.6948713406	0.9999537652	2.996475627
1.4788179842	0.3420423134	0.9999537652	-1.267857559
-1.3740558324	0.5049392843	0.9999537652	1.2380305836
2.7933974408	0.6948713406	0.9999537652	-2.1267898759
-1.117698697	0.5690507809	0.9999537652	1.1937717607
-1.0873223526	0.6927162132	0.9999537652	-1.1296675489
1.3082061	0.5362974685	0.9999537652	1.2211966497
-1.0849522591	0.7365466994	0.9999537652	1.1294786692
-1.4929695772	0.5413232483	0.9999537652	2.4125708505
2.7933974408	0.6948713406	0.9999537652	2.9985540909
-1.2331744635	0.7458071816	0.9999537652	1.3937797673
1.5126786731	0.0819851339	0.9999537652	-1.2386555983
-1.0620863923	0.8376757312	0.9999537652	-1.0609190868
-1.0043322012	0.9724442932	0.9999537652	-1.2184562983
1.6311623189	0.2678415774	0.9999537652	-1.4330613149
-1.956097916	0.1350849269	0.9999537652	1.1796488974
-1.310422014	0.5460323993	0.9999537652	-1.2791063678
-3.0591167494	0.0163456428	0.9999537652	1.1132302063
-1.2673036399	0.1437885922	0.9999537652	-1.2430462331
-1.2710069817	0.2652799692	0.9999537652	-1.231625545
1.0644333303	0.6165930774	0.9999537652	-1.2591569783
-1.1656652265	0.4213411688	0.9999537652	1.2646064717
1.1615706976	0.5039451499	0.9999537652	-1.1779260488
-2.0016488794	0.4274993691	0.9999537652	-1.8939673643
1.5569214008	0.382177694	0.9999537652	1.1495705254
8.064174035	0.4238504002	0.9999537652	0
-1.3697544738	0.7454504647	0.9999537652	-3.8916278711
-1.2078348623	0.7082024422	0.9999537652	-1.0000250282
-1.2883540559	0.4246241569	0.9999537652	1.0881665884
-2.4710767924	0.5445143636	0.9999537652	15.9659633994
-1.5875118025	0.7196666679	0.9999537652	8.8639046058
1.6428427448	0.6033101941	0.9999537652	1.2133621807
1.3515265142	0.2122719189	0.9999537652	1.0531083219
-1.4239425349	0.4362473181	0.9999537652	-1.3191372433
1.1887590105	0.7346304275	0.9999537652	1.3072984098
-1.3477987541	0.357201161	0.9999537652	-1.1067590415
0	0	0	1.8236985283
1.6441361883	0.5981320653	0.9999537652	1.8779844073
2.7957660672	0.7054880037	0.9999537652	-1.859280336
18.5983228161	0.2584132187	0.9999537652	14.7373490367
4.5505976667	0.5557790267	0.9999537652	2.996475627
0	0	0	2.996475627
-1.2821668115	0.6635126359	0.9999537652	-1.168696175
-3.0642309448	0.0992832356	0.9999537652	-1.4298575296
-1.3117714304	0.1662613683	0.9999537652	-1.0322634167
4.3889698241	0.074209006	0.9999537652	1.3699481043
15.0880352532	0.2953064644	0.9999537652	1.2204357003
1.0353671495	0.9657588963	0.9999537652	1.2577820161
15.0880352532	0.2953005413	0.9999537652	1.5332902179

2.9491038969	0.1957929575	0.9999537652	-1.3958000578
-1.8702016042	0.0201329983	0.9999537652	1.2600569355
1.0690438635	0.6392806216	0.9999537652	1.2269577252
1.0946757017	0.5953256372	0.9999537652	1.5987313339
-3.9015340979	0.1462907409	0.9999537652	-2.2622910849
1.1085725316	0.4299362639	0.9999537652	1.1112629289
6.3075231393	0.4856121243	0.9999537652	-1.2751409182
1.0521655196	0.7728725581	0.9999537652	1.0493568883
1.0189249425	0.9205782109	0.9999537652	1.0743829913
1.6492074161	0.2360295935	0.9999537652	1.2700924697
-1.0584956639	0.9291524755	0.9999537652	1.0888652996
2.7933974408	0.6948713406	0.9999537652	2.996475627
8.0710074692	0.4203803387	0.9999537652	-1.8601323346
20.3530564257	0.2433615517	0.9999537652	1.590997316
9.8205505243	0.3808303912	0.9999537652	2.2945277226
5.4106480174	0.0151664645	0.9999537652	1.148750386
-1.0492751378	0.8162375214	0.9999537652	1.3064153756
0	0	0	2.996475627
-1.7148207449	0.4983920312	0.9999537652	-1.5570092418
1.17668772	0.8040260077	0.9999537652	-1.0609851856
1.1168392827	0.9281799885	0.9999537652	-1.8579915692
-2.9295608969	0.1082219532	0.9999537652	-1.0038186338
-2.6456414501	0.164437227	0.9999537652	1.0903282479
1.1442822167	0.5478726398	0.9999537652	1.4567424265
0	0	0	0
0	0	0	8.8639046058
-1.4127809275	0.2019263621	0.9999537652	-1.1698269913
-2.9571648132	0.3435095545	0.9999537652	2.0875246791
1.3588875466	0.0289937107	0.9999537652	-1.1281762101
2.7933974408	0.6948713406	0.9999537652	4.9527501949
1.4951656018	0.5018182406	0.9999537652	1.5954154423
1.0156213372	0.9431218968	0.9999537652	1.1243927921
-1.9223741003	0.0844857395	0.9999537652	1.0532522203
-1.171238167	0.8105875596	0.9999537652	1.1329884493
1.112445692	0.4744461319	0.9999537652	1.0235590997
1.138525766	0.6089528842	0.9999537652	-1.0600875003
-1.3718565692	0.2709999251	0.9999537652	1.193923766
1.4230726096	0.4330574073	0.9999537652	-1.1111137588
0	0	0	1.7042814026
-1.0294339927	0.889138731	0.9999537652	1.3822694431
29.1718985747	0.1883076951	0.9999537652	1.2544258963
-1.0128493486	0.9596399182	0.9999537652	1.4239148743
-6.9736190158	0.2250321147	0.9999537652	6.908559683
-1.3806066288	0.2113251362	0.9999537652	-1.1106373648
1.042993087	0.9384910311	0.9999537652	-1.2161032218
1.034946767	0.9678218098	0.9999537652	-2.5413313201
-1.0632421733	0.7797828587	0.9999537652	1.1131026493
0	0	0	33.3754495834
0	0	0	6.908559683

1.3076730811	0.7003855501	0.9999537652	1.3035051167
-1.0540886962	0.6469794344	0.9999537652	1.0713034019
0	0	0	2.2916337562
0	0	0	6.908559683
9.8288704632	0.3801147743	0.9999537652	-2.4308917764
1.2495745759	0.1538558033	0.9999537652	-1.0243446133
-1.4284000021	0.2092816681	0.9999537652	1.3479636082
0	0	0	1.2408886006
-1.0419869759	0.7838499062	0.9999537652	1.1318848164
1.5849862528	0.0524436549	0.9999537652	-1.224508663
-1.3250439852	0.2948037819	0.9999537652	-1.0989763394
1.7327292703	0.0607831301	0.9999537652	1.1255907232
0	0	0	0
6.3128691894	0.481274382	0.9999537652	-5.3589986948
-1.3024238841	0.1869568499	0.9999537652	1.2996440128
2.9775254962	0.169925526	0.9999537652	-2.3208801577
1.072825034	0.8033885682	0.9999537652	1.178401873
0	0	0	8.8700459102
-1.1400073919	0.2631404661	0.9999537652	1.1253673007
-1.1453192235	0.9085990052	0.9999537652	-2.0477227478
-1.4297107459	0.0501835856	0.9999537652	1.0967066077
0	0	0	4.9527501949
-1.4091072755	0.3233553755	0.9999537652	1.4120950102
1.2562592993	0.1948358925	0.9999537652	1.3230566745
4.5505976667	0.5557790267	0.9999537652	2.9940379764
-1.1911871978	0.2471919701	0.9999537652	1.1837766398
1.3352461464	0.5281449701	0.9999537652	1.0166578596
1.6018473574	0.6931959937	0.9999537652	-1.647337401
2.3712151217	0.0508543821	0.9999537652	-1.024486205
1.0285771544	0.8650263558	0.9999537652	-1.2876243038
2.7933974408	0.6948713406	0.9999537652	2.9985540909
1.2654778301	0.7439324913	0.9999537652	1.042293684
8.064174035	0.4238490634	0.9999537652	2.2926838869
-1.2998132716	0.2489585701	0.9999537652	-1.0721200822
-1.2003975041	0.4923755249	0.9999537652	1.6971277165
1.2793831669	0.2483831017	0.9999537652	1.3855836392
-1.1543560772	0.3255422625	0.9999537652	-1.1144600223
1.0533573211	0.8143680358	0.9999537652	1.1872501192
0	0	0	-1.8580020618
-1.1223548879	0.7204905026	0.9999537652	1.3996422181
-1.1306967923	0.7366957589	0.9999537652	-1.131004236
4.5505976667	0.5557790267	0.9999537652	4.0435942789
-1.4287639136	0.4229136721	0.9999537652	-1.119768208
6.3075231393	0.4855892354	0.9999537652	0
1.0689462721	0.6915874293	0.9999537652	1.0786632952
-1.0966119323	0.6229844159	0.9999537652	-1.2395964149
-1.3349844652	0.189647185	0.9999537652	-1.1366945333
1.2266035464	0.5164652771	0.9999537652	1.4555414363
-1.1085504689	0.6120702808	0.9999537652	1.1382013097

-1.004056509	0.9838261743	0.9999537652	1.1428178105
-1.9565971794	0.316289567	0.9999537652	-1.2860103139
1.4427365624	0.2644898552	0.9999537652	-1.7893016924
0	0	0	-4.5547427608
1.0244936248	0.9070447078	0.9999537652	-1.0976858444
-2.587194901	0.5006659243	0.9999537652	1.2400291296
-1.0582641688	0.9461292521	0.9999537652	-3.4826854224
-1.047053245	0.9219428316	0.9999537652	1.3890960004
4.5505976667	0.5557790267	0.9999537652	-1.1441751338
1.3950834493	0.2742635434	0.9999537652	1.0094745326
-1.2940353524	0.3153248544	0.9999537652	-1.007270923
1.1706394892	0.4637956757	0.9999537652	1.0038322139
-1.0255608151	0.8754939508	0.9999537652	1.1110728548
1.0536947701	0.8410262914	0.9999537652	1.2995231007
2.7933974408	0.6948713406	0.9999537652	0
-1.2320855018	0.7463975051	0.9999537652	2.4996798995
-1.7594313246	0.0810843924	0.9999537652	1.1310888701
-2.0621799935	0.0729633202	0.9999537652	-1.1563505327
-1.6059557345	0.1831838661	0.9999537652	1.7621960242
1.2754968928	0.5717023483	0.9999537652	-1.0501821802
8.064174035	0.4238477265	0.9999537652	6.9133480437
2.087766184	0.5276475732	0.9999537652	-5.3583704783
2.7933974408	0.6948713406	0.9999537652	-1.8579915692
-1.088499798	0.7883941811	0.9999537652	1.151003842
-1.1112656296	0.6269553922	0.9999537652	1.1855393277
1.1275648389	0.5704676811	0.9999537652	1.176068578
1.3722203124	0.1057161897	0.9999537652	1.1650509656
1.0490364922	0.7655056393	0.9999537652	1.1651703861
-1.0569400349	0.9456846761	0.9999537652	-2.1621926629
2.7933974408	0.6948713406	0.9999537652	2.996475627
-1.3756816316	0.324618767	0.9999537652	-1.1003610056
-1.0498950284	0.8801245528	0.9999537652	-1.3554279662
-1.2492485229	0.5079679933	0.9999537652	-1.0330045512
-1.0594879394	0.9344088818	0.9999537652	-1.2917079836
0	0	0	8.8761957907
-1.1145013669	0.7128146604	0.9999537652	1.528902924
-1.5455376716	0.0689078381	0.9999537652	1.0408824842
1.375774979	0.0236515179	0.9999537652	1.1583227683
-1.0690784395	0.7002528458	0.9999537652	-1.0632210722
1.0161963097	0.9147921241	0.9999537652	-1.0737041693
4.5505976667	0.5557790267	0.9999537652	-1.3091649629
0	0	0	0
-1.2321666424	0.7531198898	0.9999537652	3.0021266318
1.0003414108	0.998543502	0.9999537652	1.1630962963
-2.5838710423	0.5186063303	0.9999537652	18.6465640159
6.3130691196	0.4798375324	0.9999537652	-1.1675888632
-1.1131075046	0.760750158	0.9999537652	1.1570205107
-1.0108355311	0.9496326115	0.9999537652	-1.1503674174
-1.1311962502	0.5191300878	0.9999537652	-1.3138199896

1.3529236751	0.1103307464	0.9999537652	1.0268048798
-1.1648887085	0.5560267957	0.9999537652	1.173346655
-1.6988038983	0.5663993685	0.9999537652	4.1474746992
1.3811117964	0.201195657	0.9999537652	-1.2057598588
1.1267368185	0.8331796727	0.9999537652	-3.0819949543
4.5505976667	0.5557790267	0.9999537652	-1.9294790029
0	0	0	6.908559683
1.0380810992	0.8450469696	0.9999537652	1.128168511
1.0421780655	0.898547273	0.9999537652	1.0778269598
-1.4481035132	0.3122006393	0.9999537652	-1.4137017696
1.2840920884	0.1423998664	0.9999537652	-1.0536972927
-1.3023449467	0.7650510272	0.9999537652	1.4210023469
11.5864583426	0.3448040633	0.9999537652	22.5383525416
-1.0767130032	0.9147691222	0.9999537652	1.6825587615
-1.267384496	0.0886053391	0.9999537652	-1.2408505123
2.134585765	0.1895772602	0.9999537652	1.6532738136
0	0	0	2.996475627
22.1075168104	0.2294704866	0.9999537652	2.9985540909
1.2642357763	0.2445884501	0.9999537652	-1.3348312138
1.1310632311	0.6231638158	0.9999537652	-1.0512959964
-1.0741528283	0.8255087862	0.9999537652	-1.0087419526
2.0363406704	0.2085629925	0.9999537652	1.1245508853
-1.2129541886	0.1035138234	0.9999537652	-1.2743037316
-1.2004233957	0.2463642315	0.9999537652	-1.1402840221
-1.1317480949	0.317941875	0.9999537652	-1.2055539496
-2.587194901	0.5006565118	0.9999537652	-1.2587469952
1.0499984748	0.890047509	0.9999537652	1.1503195285
4.5505976667	0.5557790267	0.9999537652	-3.4533081742
-1.0979377978	0.7368350662	0.9999537652	-1.0012516009
-1.4174042178	0.158124906	0.9999537652	1.1783662659
1.3271639342	0.7054760304	0.9999537652	-1.0768314905
1.6078341906	0.3434634415	0.9999537652	-2.3512245222
1.6590470498	0.3449488471	0.9999537652	-1.304203355
1.1803715905	0.1931987383	0.9999537652	1.2859548321
-1.1713292595	0.8400442214	0.9999537652	1.2777403726
-2.1727943578	0.2333624615	0.9999537652	-1.3961350135
2.7933974408	0.6948713406	0.9999537652	-5.3583704783
-1.5875118025	0.7195054915	0.9999537652	1.4527805491
1.2327920787	0.2869515815	0.9999537652	1.4249281136
1.2438594783	0.4591142321	0.9999537652	-1.21395642
-1.2448621872	0.5070532887	0.9999537652	-1.771137395
-1.2452657089	0.2060281587	0.9999537652	1.008517653
-1.0434490867	0.8583724218	0.9999537652	1.0036670813
1.0286450495	0.8999889121	0.9999537652	-1.1074016197
-1.2272193196	0.2945943695	0.9999537652	1.01960193
-1.1362180401	0.6651493904	0.9999537652	-1.3109359951
-1.1046564951	0.6076603089	0.9999537652	1.0600874595
0	0	0	2.9985540909
-1.1156222656	0.7371852264	0.9999537652	1.6329466628

23.8818846228	0.2166833328	0.9999537652	-1.4343305899
1.6104718537	0.13869931	0.9999537652	1.1701719039
-1.0617218639	0.8060529643	0.9999537652	1.0465406692
-1.241468104	0.6742428708	0.9999537652	2.8841694169
1.0868129703	0.6625358094	0.9999537652	-1.0275312004
-1.3145475858	0.505971823	0.9999537652	1.1964580069
-1.0169075041	0.8942007642	0.9999537652	1.1101812288
-1.6281541418	0.4632033365	0.9999537652	-3.0221818452
1.0611950459	0.755334131	0.9999537652	-1.0810770921
4.7740047566	0.0654749582	0.9999537652	1.6822227324
-1.0475891277	0.8023459609	0.9999537652	1.011720277
-1.4577150681	0.4289542046	0.9999537652	-2.1142207014
-1.1798199945	0.7411459637	0.9999537652	-1.5435055503
3.0602334741	0.3287128565	0.9999537652	16.6921882404
34.4101428749	0.1668302055	0.9999537652	2.1977572987
-1.026712666	0.9294622645	0.9999537652	-1.2057338269
6.3075231393	0.4855663346	0.9999537652	-1.1501702336
-1.0405404381	0.9251368905	0.9999537652	-1.2609802176
-1.1271224648	0.4737585822	0.9999537652	-1.3875253667
15.0880352532	0.2952932902	0.9999537652	0
3.3416364815	0.1499628427	0.9999537652	2.8026883955
-1.2426004229	0.1979303413	0.9999537652	1.1097581076
-2.1299772349	0.2680379039	0.9999537652	8.8761957907
5.0144569961	0.1273718317	0.9999537652	-1.0197861124
64.4037956254	0.1028474157	0.9999537652	-1.1595553882
4.5505976667	0.5557790267	0.9999537652	-5.3589986948
22.1075168104	0.2294964054	0.9999537652	1.0666207935
2.7933974408	0.6948713406	0.9999537652	0
4.5505976667	0.5557790267	0.9999537652	0
2.0885615936	0.5186564193	0.9999537652	8.8639046058
1.2026698536	0.1883299928	0.9999537652	1.1596263765
8.064174035	0.4238463895	0.9999537652	2.2220493756
-1.1252718821	0.7338087055	0.9999537652	-1.5294284081
-1.314797679	0.6945494065	0.9999537652	-2.4566346185
1.643510367	0.5934426812	0.9999537652	18.6336779883
22.1075168104	0.2295064027	0.9999537652	-2.5486515888
1.3449903122	0.4727310305	0.9999537652	-1.2575300799
2.2912132526	0.0863481466	0.9999537652	1.4280854913
-2.5690229276	0.0275845055	0.9999537652	1.0581757898
1.1561298099	0.4594618891	0.9999537652	1.3176576144
-1.5238876418	0.219922476	0.9999537652	-1.8502779685
1.0850113403	0.5852836289	0.9999537652	-1.1363029532
-1.1959441372	0.6778579601	0.9999537652	1.2014543902
-1.634512723	0.15573538	0.9999537652	-1.623448388
-1.2190746751	0.4398547275	0.9999537652	1.1363723895
1.0400230889	0.78302448	0.9999537652	-1.2136329964
1.1890672481	0.1737416104	0.9999537652	1.1989776832
27.3284152947	0.2046641326	0.9999537652	1.3341676396
2.0867996709	0.0536616897	0.9999537652	1.0143682105

-1.0586941874	0.951323742	0.9999537652	2.996475627
-1.5204346711	0.1685758205	0.9999537652	-1.0816512457
-1.0086225336	0.9454264467	0.9999537652	-1.248061277
4.0297652987	0.2233339799	0.9999537652	-1.6203746953
2.7933974408	0.6948713406	0.9999537652	-1.6578228316
1.3786208238	0.3785569584	0.9999537652	1.0414885467
3.5458425332	0.2599220283	0.9999537652	-1.3268884013
0	0	0	-1.1242401582
-7.4805414887	0.00433	0.9999537652	1.5226566128
1.543329787	0.2931995739	0.9999537652	1.0541995306
-1.3685516055	0.7424185057	0.9999537652	-1.5732602273
3.9579785606	0.009576	0.9999537652	-1.1217609558
-1.013887414	0.9667781909	0.9999537652	-1.219812684
1.1124880746	0.6047242813	0.9999537652	-1.4958717391
-1.2194928882	0.1102687733	0.9999537652	1.1563416585
-1.1436175851	0.565637718	0.9999537652	-1.0402675051
1.2533792806	0.2496521796	0.9999537652	-1.0117747809
-1.0314801103	0.9251565668	0.9999537652	1.5539400434
1.3588921917	0.7998708465	0.9999537652	-3.0826861089
-1.0846468307	0.6943718498	0.9999537652	-1.1710879165
-1.1267770838	0.567546349	0.9999537652	1.1210320192
-1.1947641352	0.1595766842	0.9999537652	-1.1875683811
-1.0517525283	0.8931964324	0.9999537652	-1.4800500403
6.3075231393	0.4855434221	0.9999537652	-1.2808959382
1.798150741	0.3208122302	0.9999537652	-1.3473061746
-1.0141273035	0.9393866092	0.9999537652	1.0577806092
1.0349118986	0.9686744453	0.9999537652	-10.0604019451
1.0328743166	0.7998167006	0.9999537652	-1.1270267627
1.448697666	0.3132607351	0.9999537652	1.0391386154
1.0693056355	0.6657084771	0.9999537652	-1.2229503236
-1.066453755	0.8087817707	0.9999537652	-1.2004980257
-1.1732329994	0.7434360732	0.9999537652	-1.2635192949
1.1282390015	0.8381743829	0.9999537652	-1.0530499944
1.2494524772	0.4023406698	0.9999537652	-1.0511706383
1.4052010358	0.4979007365	0.9999537652	1.0263559879
0	0	0	1.4660736959
1.1369014154	0.5510298796	0.9999537652	-1.7082347022
1.004248794	0.9831048067	0.9999537652	-1.3232780297
1.3588576256	0.801333561	0.9999537652	-1.3911859456
3.0602334741	0.3283646902	0.9999537652	1.225766291
-1.6578780139	0.0293951705	0.9999537652	1.0030081543
1.0007212244	0.995152847	0.9999537652	-1.1278473391
-1.0534933443	0.6594171605	0.9999537652	-1.0373683498
1.5444112962	0.4900009618	0.9999537652	-1.937154584
-1.2204596224	0.287426221	0.9999537652	-1.1467661453
0	0	0	4.9527501949
-1.3802230046	0.2198866406	0.9999537652	-1.2758594071
1.1800112458	0.2222136777	0.9999537652	1.2478356974
1.0044600286	0.9866590041	0.9999537652	1.2100862909

1.0152588784	0.9625737593	0.9999537652	1.650829719
-1.2976100564	0.1441133758	0.9999537652	1.0942912258
-1.0463856551	0.8654047423	0.9999537652	-1.0487768568
1.0774268281	0.8745501458	0.9999537652	-2.3585094137
1.0539822683	0.7347968499	0.9999537652	1.0027323621
-1.0674298248	0.6934955123	0.9999537652	1.2370956292
-1.4084943164	0.4738527555	0.9999537652	-1.5804454321
1.3278895178	0.7144850349	0.9999537652	1.1309268147
1.1381955878	0.6772976558	0.9999537652	1.0651851045
-1.2296385908	0.3146892754	0.9999537652	-1.3380741914
-1.6304332292	0.2224171118	0.9999537652	-1.41474025
-1.0562596574	0.9403962757	0.9999537652	-1.2222893883
1.4125564959	0.2658861148	0.9999537652	1.1247802543
-1.0142202953	0.9060087045	0.9999537652	1.0288953166
-1.0660011546	0.6157656914	0.9999537652	1.1168594444
1.0264331092	0.8347672192	0.9999537652	1.0040677072
-1.0192531122	0.9587621597	0.9999537652	-1.1397943437
3.0768203874	0.1975714249	0.9999537652	1.430803866
2.7933974408	0.6948713406	0.9999537652	-2.9683711557
0	0	0	14.7271569203
1.7624255387	0.2485580472	0.9999537652	1.4885956851
2.7933974408	0.6948713406	0.9999537652	1.6447327969
4.5505976667	0.5557790267	0.9999537652	6.908559683
-1.624667929	0.0572377131	0.9999537652	1.1351714646
1.1167126147	0.7285233563	0.9999537652	-1.9781707549
1.1010872447	0.6617123439	0.9999537652	-1.1726406249
2.7933974408	0.6948713406	0.9999537652	-1.1444432957
-1.4407619125	0.5746492808	0.9999537652	-1.0946293968
-1.0115911681	0.9478124883	0.9999537652	-1.0265730725
-2.1325963601	0.4603116315	0.9999537652	-1.1241146656
1.6442324412	0.5883483586	0.9999537652	1.8286071118
-1.4741597973	0.1986069956	0.9999537652	-1.1330519708
-1.0089195645	0.9925509949	0.9999537652	5.0955150726
1.9042601795	0.4860487474	0.9999537652	1.1109046993
-1.2081320805	0.3686231979	0.9999537652	-1.2164041945
1.2092357838	0.2707930716	0.9999537652	-1.0038680276
-1.2399772406	0.6419157246	0.9999537652	-1.1206822297
-1.1105747506	0.819543664	0.9999537652	-1.5534881544
-1.050760015	0.7505836271	0.9999537652	-1.3416287432
0	0	0	2.996475627
-1.2403707278	0.3735240934	0.9999537652	-1.2787752906
0	0	0	0
1.9292309639	0.1737350981	0.9999537652	2.4776447593
1.1309621929	0.7935518966	0.9999537652	2.2620415671
1.3632564722	0.0883427924	0.9999537652	1.0099167801
-1.2093406112	0.6157336263	0.9999537652	-1.1745635889
2.0057180274	0.3367799306	0.9999537652	-1.6211394957
-1.0101239827	0.9473414413	0.9999537652	1.0314024837
1.0808920899	0.7990051241	0.9999537652	-1.340205331

1.1261854259	0.8630528928	0.9999537652	-1.6496636245
-1.3707945296	0.3287138139	0.9999537652	-1.4588926513
1.1547555504	0.6485578592	0.9999537652	1.2251321024
-1.2954004139	0.265103961	0.9999537652	-1.235560175
-1.0771708963	0.9336949682	0.9999537652	1.6178683885
-1.0826054966	0.6941613103	0.9999537652	-1.1907002272
1.4278875705	0.2168362539	0.9999537652	1.0336326672
1.3485163287	0.3195688868	0.9999537652	-1.0918387842
1.1197784385	0.5377572468	0.9999537652	1.3232123774
-1.0830409898	0.7708933966	0.9999537652	1.0071986158
-1.1394164215	0.7434932528	0.9999537652	-1.3448684038
1.1165478033	0.8009628298	0.9999537652	-1.3958347093
-1.2815515061	0.693910851	0.9999537652	-1.0338518335
-1.3794059984	0.4715813603	0.9999537652	1.397936197
1.0013465097	0.9941381854	0.9999537652	1.4220544204
-1.762709422	0.2480356749	0.9999537652	1.151609159
-1.0711456263	0.7658346104	0.9999537652	1.2725231676
-1.1508399632	0.4242552298	0.9999537652	-1.0503787856
1.7746986793	0.5317345774	0.9999537652	1.1670818081
0	0	0	2.996475627
1.4876413039	0.1180594066	0.9999537652	1.6167217615
1.1537490598	0.414228245	0.9999537652	1.1326271949
8.064174035	0.4230822908	0.9999537652	0
0	0	0	2.996475627
-1.1082079634	0.4909291308	0.9999537652	1.1079480482
1.2607334053	0.1503900462	0.9999537652	1.2369525042
1.0679308554	0.7080485759	0.9999537652	1.0720842872
-1.2407940756	0.7394963901	0.9999537652	1.0768907588
-1.3518561167	0.4071500171	0.9999537652	1.0949409705
1.0274043788	0.8572208762	0.9999537652	1.0409909572
-1.283216297	0.3614019816	0.9999537652	1.0255901679
1.132483306	0.6859277074	0.9999537652	-1.2593349746
-1.3174384883	0.0301448463	0.9999537652	-1.1902269235
-1.0102340433	0.9658227431	0.9999537652	1.1821339936
-1.0350037612	0.895520583	0.9999537652	-1.36884812
1.2390581437	0.2951079932	0.9999537652	1.0360320951
1.0966528316	0.7796804844	0.9999537652	-1.170069719
-1.0854549056	0.5954311189	0.9999537652	1.1283577779
1.2423197598	0.2289392579	0.9999537652	1.6260534691
1.0166980697	0.9264862972	0.9999537652	1.0940871915
1.0257818063	0.9518450434	0.9999537652	-1.3106272376
1.0548459601	0.6802568136	0.9999537652	1.1693797239
0	0	0	0
1.5037044015	0.2051142352	0.9999537652	1.1178065896
2.0885615936	0.518797066	0.9999537652	-3.3853891979
-1.0452744086	0.8077719723	0.9999537652	-1.044331215
18.5983228161	0.2583766021	0.9999537652	1.0203343598
-1.0104631551	0.9794834163	0.9999537652	-1.0658053623
0	0	0	8.9512754559

1.3239809969	0.3592204012	0.9999537652	-1.1078196195
2.0294079422	0.2451825376	0.9999537652	-1.0225109693
-1.6871513697	0.1997833382	0.9999537652	2.5761588337
1.1004912796	0.5992325848	0.9999537652	1.2579034211
1.0799672643	0.6808785732	0.9999537652	1.1699608222
0	0	0	12.7732027325
4.5544554512	0.569131392	0.9999537652	-1.6223462933
-1.6661685285	0.0287119198	0.9999537652	-1.4427483555
-1.1531088649	0.4420758648	0.9999537652	1.3894342228
2.6809644128	0.0341068952	0.9999537652	-1.1032473644
1.0746073568	0.8608106679	0.9999537652	1.303743597
-1.0655087673	0.6015098561	0.9999537652	1.2030600629
-1.3446989983	0.6591269618	0.9999537652	3.0555055251
-1.0454457482	0.8089870806	0.9999537652	1.0920644179
1.3309330294	0.5164433806	0.9999537652	1.043939591
4.5505976667	0.5557790267	0.9999537652	-1.7799057205
0	0	0	-1.859280336
4.5505976667	0.5557790267	0.9999537652	0
-1.1631691103	0.4671441117	0.9999537652	1.0505402061
-6.9764533263	0.2593448532	0.9999537652	-9.3613041355
0	0	0	8.8639046058
4.5505976667	0.5557790267	0.9999537652	1.590997316
0	0	0	2.996475627
1.1265982105	0.5421478705	0.9999537652	1.4280025521
-1.6342415317	0.3450846104	0.9999537652	-1.0689684185
-2.0160710885	0.0353183263	0.9999537652	-1.0157605017
6.3075231393	0.4855204978	0.9999537652	-1.144309199
-1.5875118025	0.7193450002	0.9999537652	-3.2026060843
4.5505976667	0.5557790267	0.9999537652	-5.3583704783
1.2509256148	0.4312690751	0.9999537652	1.0272792754
-1.0064087013	0.9870883774	0.9999537652	1.0245179551
1.7061363611	0.1789216697	0.9999537652	1.5391423279
-1.0379579947	0.810440888	0.9999537652	1.2918323555
-1.4867681804	0.1193185681	0.9999537652	-1.0053902956
2.4568497219	0.1117023542	0.9999537652	5.5152075473
-1.1593805716	0.446115096	0.9999537652	-1.1405917756
2.7933974408	0.6948713406	0.9999537652	0
1.9420462371	0.001995	0.9999537652	1.0803534395
0	0	0	8.8639046058
6.3075231393	0.4854975618	0.9999537652	2.9985540909
1.0417868618	0.8617129038	0.9999537652	1.2152490113
1.0887847388	0.5281571983	0.9999537652	1.0659933659
1.0192651553	0.9025108916	0.9999537652	1.1246350243
-1.1089217961	0.5827326393	0.9999537652	1.3272444605
-1.3918702637	0.514375824	0.9999537652	1.5456239127
1.4291786189	0.4117526825	0.9999537652	1.0942284709
1.5498541366	0.1631715231	0.9999537652	1.1423141742
-1.0209534506	0.9315434961	0.9999537652	1.356879688
1.0652606154	0.6596537803	0.9999537652	1.0439114393

1.538652413	0.242278366	0.9999537652	1.1107108221
1.1991686279	0.7044214231	0.9999537652	1.1671637511
1.173142651	0.535301655	0.9999537652	1.2479693568
-1.0567980975	0.6519866831	0.9999537652	1.0001891078
1.0685224318	0.7199837631	0.9999537652	-1.0266244299
0	0	0	-5.3608259368
-1.0276200717	0.84682801	0.9999537652	1.2386456352
1.2516839175	0.8280427789	0.9999537652	1.5920996292
-4.815549291	0.1727012834	0.9999537652	2.1977572987
2.1668004892	0.367901064	0.9999537652	1.2415266768
-1.1458640337	0.4954787361	0.9999537652	1.0051154334
-1.2415015851	0.2299812511	0.9999537652	1.2475593172
-1.4789398964	0.2682894628	0.9999537652	1.3774512377
1.5506685089	0.0618684239	0.9999537652	-1.0204024776
1.0744385771	0.5605973839	0.9999537652	1.3416696856
1.0552907781	0.6670060077	0.9999537652	1.2630976334
1.2230661395	0.2842104379	0.9999537652	1.295446323
-1.7127500346	0.1372780796	0.9999537652	1.3341508472
4.0102736869	0.1096386666	0.9999537652	1.3829372939
-1.2711633669	0.3058401175	0.9999537652	1.0117841599
1.4557577282	0.1916362645	0.9999537652	1.1903405418
18.6298281873	0.2553727058	0.9999537652	-2.0711873351
-1.1297167925	0.5970954469	0.9999537652	1.3064385098
2.7933974408	0.6948713406	0.9999537652	0
1.4946456017	0.4821253923	0.9999537652	1.5789238278
-1.1437750147	0.6016572077	0.9999537652	-1.413049955
1.056477578	0.7694882132	0.9999537652	1.0841796687
-1.1304205482	0.5963441401	0.9999537652	-1.150996288
62.6505509376	0.103692165	0.9999537652	2.5408539857
1.0337578361	0.8877987527	0.9999537652	-1.0479370285
1.6118933969	0.047463019	0.9999537652	-1.0846800531
1.5614986311	0.299657901	0.9999537652	1.0586805782
-1.2355073246	0.3424270136	0.9999537652	1.1910527014
-1.3088126937	0.4809509356	0.9999537652	-1.1657167669
-1.2592873974	0.1933788344	0.9999537652	1.0424240632
0	0	0	0
16.8718544959	0.273791704	0.9999537652	1.0328466942
-1.2515854966	0.562744126	0.9999537652	-1.0753748677
1.16869238	0.3343981204	0.9999537652	1.0651517019
1.1666718464	0.6233652523	0.9999537652	1.0876825954
-1.2429397535	0.1865254158	0.9999537652	1.1251392141
-1.7565904216	0.3101139973	0.9999537652	1.4455555239
1.3001100676	0.5447669592	0.9999537652	1.3487781182
-1.0747394431	0.7182856785	0.9999537652	1.2554072967
1.0467462673	0.7431890982	0.9999537652	1.1355191417
-1.0363967531	0.8703711937	0.9999537652	1.082871836
-2.1848178259	0.233224138	0.9999537652	1.1150142848
1.0972497395	0.7349194724	0.9999537652	1.0322038001
0	0	0	10.818785458

-1.0220081257	0.9301172406	0.9999537652	1.3777480177
1.0669763156	0.8490335031	0.9999537652	-1.0394306235
-1.0993782959	0.6431045274	0.9999537652	1.1108473335
-1.0706629432	0.6634078	0.9999537652	-1.0247731042
6.3075231393	0.4854746142	0.9999537652	-1.644923008
-1.0790444073	0.7958185359	0.9999537652	1.0556136998
0	0	0	10.818785458
-1.7876739331	0.1247089462	0.9999537652	-1.1509528927
1.3587238604	0.3404096172	0.9999537652	1.4780545859
-1.1796427531	0.7653407601	0.9999537652	-1.1587803058
-1.1295198141	0.603071559	0.9999537652	1.0766404706
1.1048689607	0.8505313588	0.9999537652	1.2221663107
1.0150539717	0.9384518653	0.9999537652	1.0484235394
-1.0622324628	0.82995295	0.9999537652	1.3945451975
4.5505976667	0.5557790267	0.9999537652	0
1.1158299481	0.9297544265	0.9999537652	-5.3583704783
-1.0128503736	0.9497058067	0.9999537652	1.1173731884
1.1648707746	0.2979450526	0.9999537652	1.5225268256
-1.1905004323	0.3217563702	0.9999537652	-1.0924134472
1.5540529377	0.0279148803	0.9999537652	1.0214143824
-1.0835619942	0.8462909724	0.9999537652	1.3384261804
-1.1202797683	0.8316176596	0.9999537652	-1.3738644509
1.1309455813	0.6969503953	0.9999537652	-1.3023084422
-1.1512918491	0.4686967453	0.9999537652	1.2736300536
-1.1635825669	0.6493943547	0.9999537652	-1.2327461774
1.1162834585	0.9273215157	0.9999537652	-1.5836401754
2.7957660672	0.7054927342	0.9999537652	-3.3826461929
-1.3824661695	0.1349791714	0.9999537652	-1.1227668843
-1.0830484303	0.7807021881	0.9999537652	-1.1770282279
-1.0375253331	0.8443322405	0.9999537652	1.218295698
1.745558548	0.0542390908	0.9999537652	1.0154090202
-1.034876915	0.9178545218	0.9999537652	1.6131054446
1.0781948674	0.7137832768	0.9999537652	1.1266282556
1.04354676	0.8863017949	0.9999537652	-1.0745724722
-1.1782938534	0.4960141001	0.9999537652	-1.1348631108
1.1323539498	0.5147386672	0.9999537652	-1.1405072718
1.3576258306	0.5361849952	0.9999537652	1.7383233151
1.5221533183	0.4489640482	0.9999537652	1.1188264007
-1.0717148199	0.6391404241	0.9999537652	-1.1478648967
1.209185672	0.3515169797	0.9999537652	1.3349795879
1.4213868285	0.008038	0.9999537652	1.3883810677
1.0944874443	0.6923275418	0.9999537652	1.4182092655
-1.2060533122	0.2015882754	0.9999537652	1.1293585279
1.2509474775	0.2403779911	0.9999537652	1.066375584
-1.0018516575	0.9932470045	0.9999537652	1.7570094758
-1.6715479992	0.4280290348	0.9999537652	-1.0386110167
-1.2359990247	0.7441983326	0.9999537652	1.3961512712
2.937871377	0.1850196146	0.9999537652	-7.2920271422
1.0709201901	0.5905464157	0.9999537652	1.420401534

0	0	0	8.8639046058
-1.338866394	0.3029460418	0.9999537652	1.046744
-1.0680335458	0.8908708817	0.9999537652	-1.1655607995
1.0558829496	0.820916409	0.9999537652	-1.3381489201
-1.229329195	0.2522684767	0.9999537652	-1.4093158561
-1.4311207211	0.2770849861	0.9999537652	1.5596372938
-1.3820084251	0.4313420627	0.9999537652	-1.2511030064
2.8173722589	0.3577970311	0.9999537652	-2.1857541833
1.0802249044	0.727447586	0.9999537652	-1.0763868485
1.1863520868	0.7087842864	0.9999537652	-1.4509645408
1.081122909	0.5379086846	0.9999537652	-1.0198725621
-1.0481861033	0.8345290903	0.9999537652	-1.119814806
-2.3806508789	0.009308	0.9999537652	-1.7856288627
-1.2817155393	0.4040066693	0.9999537652	1.0422816754
1.0854457506	0.492498634	0.9999537652	1.2032809525
1.0251691845	0.9018014232	0.9999537652	-1.0212221562
2.2773894756	0.2153016587	0.9999537652	-1.5790810514
1.1877419666	0.5848380683	0.9999537652	1.5767164782
4.6455089981	0.0683620362	0.9999537652	-1.9880884458
4.5505976667	0.5557790267	0.9999537652	2.2924151284
-1.1546110864	0.3900088611	0.9999537652	-1.1776300108
2.1327820026	0.1200348994	0.9999537652	-1.4023913842
1.4349404381	0.1376994937	0.9999537652	-1.3961347544
-1.8643763152	0.3172154085	0.9999537652	2.8690209402
-1.12188679	0.7748143959	0.9999537652	-1.4032646471
-1.1896169234	0.4632072937	0.9999537652	-1.1036267434
-1.8300986908	0.0295515815	0.9999537652	1.1213854033
-1.0312044602	0.9596494834	0.9999537652	-1.4834735767
1.1448177787	0.6335810666	0.9999537652	-1.103850241
-1.3148677635	0.1795795964	0.9999537652	1.1138600596
-1.6048757093	0.4641308228	0.9999537652	1.0396888355
-1.1580927871	0.3846371341	0.9999537652	1.0801528659
2.4666232931	0.1125361989	0.9999537652	-1.0757422118
-1.3321793584	0.5610167475	0.9999537652	1.2807032682
-1.0026391872	0.9930050146	0.9999537652	-1.2204291542
1.0512601692	0.6973919727	0.9999537652	-1.0949041184
1.2565162416	0.5438276699	0.9999537652	1.6450139622
0	0	0	24.4899985757
-1.0733599254	0.7084793963	0.9999537652	1.0215576509
-1.1741964346	0.7426649952	0.9999537652	-1.1556892707
1.4537616364	0.2586164218	0.9999537652	1.1129507202
-1.1157067995	0.3432303171	0.9999537652	1.0544170741
-1.0797696345	0.5396684314	0.9999537652	-1.2168962231
-1.6484285107	0.0975063659	0.9999537652	1.0084493247
2.7933974408	0.6948713406	0.9999537652	0
0	0	0	2.9985540909
1.1846865074	0.4912512751	0.9999537652	1.3346182716
-1.6388132474	0.0848115375	0.9999537652	-1.440812958
-1.8448851181	0.178493205	0.9999537652	-1.4975431842

1.0209353182	0.9024932957	0.9999537652	-1.0677626705
-1.3390154077	0.0980515113	0.9999537652	-1.3305629498
1.2126112857	0.0989912877	0.9999537652	1.0984966068
1.2783179019	0.2827663672	0.9999537652	-1.1614810519
-1.1711631892	0.8483989027	0.9999537652	-1.4612021112
0	0	0	9.7116702472
0	0	0	2.996475627
1.1404558615	0.3429807867	0.9999537652	1.1142776485
1.1169155339	0.488606985	0.9999537652	-1.1106993851
-1.4796889536	0.2264786651	0.9999537652	-1.6741145203
1.0267652648	0.8421715857	0.9999537652	-1.0306931434
-1.1024469748	0.4062594339	0.9999537652	1.0424777432
1.5086084868	0.0934143043	0.9999537652	-1.1080454004
1.1878746775	0.2602436572	0.9999537652	-1.1508122759
1.2821370521	0.2908481204	0.9999537652	-1.0082575728
-2.0008873798	0.4500303958	0.9999537652	2.0652983605
1.1803952659	0.2679343967	0.9999537652	-1.2118892371
1.4170626427	0.3518691427	0.9999537652	-1.1853570569
1.0512003843	0.8055335044	0.9999537652	1.1774011007
-1.6477115577	0.0213987352	0.9999537652	-1.1319145048
-4.8095156671	0.2070202161	0.9999537652	-2.0161248892
1.285893423	0.4443181069	0.9999537652	1.2321043478
25.6372777234	0.2064852657	0.9999537652	1.0276899286
1.5682964138	0.3323734601	0.9999537652	1.0746656978
-1.5432722588	0.1414819278	0.9999537652	-1.146838775
1.0892812652	0.6385055809	0.9999537652	-1.3366127715
1.0934204149	0.5200798167	0.9999537652	-1.026803823
-1.1808999578	0.3792454212	0.9999537652	-1.2147492053
-1.0180216044	0.9118720303	0.9999537652	-1.1329786063
-1.1055798886	0.6052894442	0.9999537652	1.0428533419
16.8718544959	0.2737628052	0.9999537652	1.9345442849
-1.3594371598	0.2798749983	0.9999537652	-1.2331972257
1.5185950608	0.0641564166	0.9999537652	-1.0949068645
-1.19568794	0.3859153684	0.9999537652	-1.2008199468
-1.1693119757	0.8275585751	0.9999537652	1.9684343355
1.1268458263	0.4237910281	0.9999537652	-1.201390655
-1.0095897015	0.9888202879	0.9999537652	-1.6589722521
-1.0278408125	0.9199837187	0.9999537652	1.0176587704
-1.3793873385	0.1587678539	0.9999537652	-1.2159132428
2.3335098933	0.4417206775	0.9999537652	1.6652336982
1.1757625339	0.4751544317	0.9999537652	1.0485705888
1.0729136782	0.9152272869	0.9999537652	-1.4136515635
-1.2940180539	0.3632567577	0.9999537652	1.1423389803
-1.0673171705	0.7880248873	0.9999537652	1.170939493
1.063316061	0.8482216421	0.9999537652	-1.2529808255
-1.0956944243	0.6631790322	0.9999537652	1.144403713
1.2555709875	0.4837661996	0.9999537652	-1.1078295401
6.3075231393	0.485451655	0.9999537652	-5.3589986948
1.0236618924	0.8667492069	0.9999537652	1.0435443979

2.0354520887	0.4477839505	0.9999537652	-1.4741963843
-1.4954848333	0.3220237192	0.9999537652	-1.4080908716
-1.2947942833	0.4876979208	0.9999537652	-1.1796174106
1.1844825577	0.7200917106	0.9999537652	-1.0673606747
1.4740649528	0.2400143728	0.9999537652	-1.1800734146
-1.1266715125	0.5004467097	0.9999537652	1.1720587512
-1.5774890195	0.3221730134	0.9999537652	-1.0839550431
-1.2771433826	0.2857400526	0.9999537652	-1.1327104837
1.0586498351	0.8053405735	0.9999537652	1.0501301114
1.3614856847	0.1297233268	0.9999537652	1.346270077
-1.1751153768	0.2857504191	0.9999537652	1.0806789664
1.8398140613	0.1602465916	0.9999537652	-1.6800294987
-1.1614465172	0.2807127974	0.9999537652	-1.1871333144
-1.0118448434	0.9747685652	0.9999537652	-1.4235410716
-1.5305812311	0.5675869828	0.9999537652	-1.1248044011
1.0894712817	0.5860976452	0.9999537652	1.0588171768
-1.0851687587	0.6866542388	0.9999537652	-1.0414462143
1.0525931057	0.7825188426	0.9999537652	1.0561233115
1.1148751653	0.6499725722	0.9999537652	1.3845512285
-1.0840217463	0.7015254254	0.9999537652	1.044661462
-1.0666211761	0.8838656895	0.9999537652	-1.3466975077
-1.2168919617	0.5346917371	0.9999537652	1.1095080702
-1.0202735182	0.884604801	0.9999537652	-1.0187378039
1.1408589403	0.7384613792	0.9999537652	-1.1723473927
-1.3107841951	0.1542406985	0.9999537652	-1.104755264
-1.0427069939	0.838677402	0.9999537652	1.1534174888
1.1614433121	0.3640398309	0.9999537652	-1.1456664312
1.0899709362	0.7207030753	0.9999537652	-1.0503446426
-1.0400531458	0.8012921758	0.9999537652	-1.2164433954
-1.0758055436	0.8083040755	0.9999537652	-1.208198232
4.517455539	0.0856145041	0.9999537652	-1.1533365725
0	0	0	2.9988886918
2.7933974408	0.6948713406	0.9999537652	0
4.5505976667	0.5557790267	0.9999537652	8.8639046058
1.1033573704	0.6527281908	0.9999537652	1.1387938929
4.5505976667	0.5557790267	0.9999537652	-1.123982892
16.8433158146	0.2735409335	0.9999537652	-1.3095105362
0	0	0	2.996475627
-1.2772694893	0.4578141867	0.9999537652	1.0662961519
-1.138183167	0.2560100613	0.9999537652	-1.0266970013
-1.1190894018	0.5289090516	0.9999537652	-1.0901198025
-1.9430018564	0.4087251328	0.9999537652	-1.7195161403
1.4745789114	0.0939880414	0.9999537652	-1.1232796119
1.0055567739	0.9749202939	0.9999537652	-1.3455722467
1.0424767751	0.8581046647	0.9999537652	-1.0292777891
-1.0225224938	0.916925319	0.9999537652	1.0296299222
-1.2000893308	0.6151023203	0.9999537652	1.1151943287
1.1923737832	0.8182828534	0.9999537652	-1.1923833604
0	0	0	-1.3598304575

-1.1073785092	0.6543001307	0.9999537652	-1.1058459266
0	0	0	0
6.3075231393	0.4854286841	0.9999537652	0
-1.1611015106	0.6273847581	0.9999537652	-1.371131225
-1.3036560105	0.2257855002	0.9999537652	-1.2496770151
1.0705253491	0.7088324696	0.9999537652	1.3539301635
-1.1427689666	0.4837608878	0.9999537652	-1.0514115431
-1.3894979293	0.0496627431	0.9999537652	-1.0318761021
-1.2278711531	0.6688413342	0.9999537652	1.9055543429
-1.3381928736	0.4309516453	0.9999537652	-1.2369910878
-1.2682216478	0.2792008333	0.9999537652	-1.1226690661
2.4905122517	0.0478835288	0.9999537652	1.528295012
34.3811030879	0.1695019447	0.9999537652	1.2594537582
1.2164420184	0.8053222086	0.9999537652	-4.1152258145
1.124746803	0.8556409972	0.9999537652	1.2803733205
11.5766527773	0.3453990803	0.9999537652	3.7052803395
1.1177441048	0.5172746255	0.9999537652	-1.0015936301
1.0236878871	0.8958923763	0.9999537652	1.0722987225
-1.4375831168	0.3579012916	0.9999537652	-1.1243689986
-1.1860795505	0.4719125662	0.9999537652	-1.0330433139
3.9744797554	0.2836205177	0.9999537652	-1.7268642166
-1.0355492915	0.9338307841	0.9999537652	1.6088862186
15.0880352532	0.2950942573	0.9999537652	0
1.4817259234	0.1642098823	0.9999537652	-1.0376791732
1.3571423233	0.512346602	0.9999537652	1.640937024
1.180297178	0.6635480006	0.9999537652	-1.0946237578
-1.4286094458	0.2443124005	0.9999537652	-1.011994059
-1.3893577805	0.2415178869	0.9999537652	1.1658742097
-1.2035404978	0.5419977471	0.9999537652	-1.0635714687
-1.095292568	0.4860024217	0.9999537652	1.1222777452
-1.3605514786	0.1864663462	0.9999537652	1.3116128299
-1.0626175961	0.6743609828	0.9999537652	1.0398862079
0	0	0	-1.3593668272
1.149120046	0.4510631356	0.9999537652	-1.0975474177
-1.2704982263	0.1752926778	0.9999537652	1.3251084227
0	0	0	6.908559683
-4.8135938501	0.1785914488	0.9999537652	2.996475627
-1.1323079957	0.5214140509	0.9999537652	-1.0084975503
-3.2699928362	0.1576323278	0.9999537652	1.2694869116
-1.1256611836	0.6992458483	0.9999537652	-1.3226887462
9.8291821523	0.3810402025	0.9999537652	-2.2391686371
-1.3567700241	0.473666366	0.9999537652	-1.3886093898
-1.1527571892	0.5558245207	0.9999537652	1.3395347413
2.7933974408	0.6948713406	0.9999537652	2.996475627
4.5505976667	0.5557790267	0.9999537652	8.8639046058
-1.5941542392	0.0427012111	0.9999537652	-1.087340296
-1.0316109055	0.8817592361	0.9999537652	1.0955409103
1.1093756627	0.5468701266	0.9999537652	1.1673522149
1.1012619464	0.6696795862	0.9999537652	1.247117995

1.2905235246	0.7076099484	0.9999537652	-1.2144502384
1.6025907098	0.1040205866	0.9999537652	1.0023957238
1.2589114534	0.3234820826	0.9999537652	1.4813495611
-1.0964929497	0.7502190531	0.9999537652	-1.0536964612
2.7933974408	0.6948713406	0.9999537652	-1.14351692
0	0	0	4.9527501949
1.0073836995	0.9696680003	0.9999537652	-1.0639745363
1.1492445044	0.2962706452	0.9999537652	1.1594244152
4.5505976667	0.5557790267	0.9999537652	2.2942711679
-1.0439086777	0.7695204926	0.9999537652	1.004009655
1.1529146634	0.5295431579	0.9999537652	-1.0820034817
-1.0944022099	0.7299593215	0.9999537652	1.0602051576
1.1756050889	0.2743302296	0.9999537652	1.1513338266
0	0	0	0
0	0	0	-1.1250196696
1.124070454	0.906249133	0.9999537652	1.2132629184
1.0877342563	0.6628109982	0.9999537652	1.1046539493
1.7747100951	0.5596991323	0.9999537652	34.3398315517
1.2147901705	0.1277642646	0.9999537652	1.1334330353
2.7933974408	0.6948713406	0.9999537652	0
16.8581089812	0.2738184187	0.9999537652	1.5894490894
-1.7140366854	0.003483	0.9999537652	-1.1395921295
-1.0889509227	0.6923877787	0.9999537652	1.1527555071
-1.0993038336	0.8296585186	0.9999537652	1.0162973402
-1.1668466258	0.1699008283	0.9999537652	1.0324186039
-1.0216541814	0.9525307393	0.9999537652	-1.1047225461
1.2594687841	0.1415731094	0.9999537652	-1.1082165781
0	0	0	-2.0460754901
-1.1038659669	0.500230957	0.9999537652	1.0033619365
1.007459388	0.9856189388	0.9999537652	-1.1913063346
1.2338638161	0.4980471597	0.9999537652	1.2109312597
2.7933974408	0.6948713406	0.9999537652	0
-1.1087694416	0.7962939824	0.9999537652	1.4990238903
2.006385497	0.0827066108	0.9999537652	1.1119771722
1.4668223311	0.0369563131	0.9999537652	-1.0118582916
1.141683967	0.5250382192	0.9999537652	1.1044345067
-1.5875118025	0.7191851908	0.9999537652	0
-1.7126738456	0.5348532116	0.9999537652	-1.9220666281
-1.2521293745	0.1831818193	0.9999537652	-1.0134922782
2.0354520887	0.4478716725	0.9999537652	1.4539570206
4.5505976667	0.5557790267	0.9999537652	-1.2784318804
1.4677303914	0.6422486858	0.9999537652	1.1165417891
-1.7150903924	0.5020348074	0.9999537652	-1.3741916786
1.2508079663	0.1466628114	0.9999537652	1.4236733801
1.2416511195	0.3610062985	0.9999537652	-1.1162347094
1.2029614	0.609061269	0.9999537652	-1.4860455187
-3.8978654675	0.0774219164	0.9999537652	1.1738496605
1.2680916436	0.1631572271	0.9999537652	1.2362264076
1.3661459224	0.0901914307	0.9999537652	-1.1900631644

1.26726803	0.632118024	0.9999537652	1.1825900946
1.0310457355	0.8680004628	0.9999537652	1.5392411207
1.2516860341	0.3525443409	0.9999537652	1.2617972731
1.7099471466	0.2534456358	0.9999537652	1.2941181437
1.2982841569	0.628969465	0.9999537652	-1.5836391682
1.5238592065	0.3266642932	0.9999537652	1.1199539848
1.1112880642	0.7359754549	0.9999537652	-1.2198910671
-1.2421080709	0.3379924788	0.9999537652	1.3037080462
-1.1233792384	0.7039295413	0.9999537652	-1.062454328
-1.0676775145	0.7434664942	0.9999537652	1.4330129612
-1.1464182068	0.5671317924	0.9999537652	1.1973787615
1.0297943064	0.8876284578	0.9999537652	1.6862654468
3.5302237986	0.0121905177	0.9999537652	-1.1330944388
-1.0772256662	0.8716325634	0.9999537652	1.0137498297
-1.1075691371	0.7576132878	0.9999537652	1.1569523817
-1.0159785761	0.964462702	0.9999537652	1.5766577618
-1.328502012	0.3365850117	0.9999537652	1.2248634774
1.0884461353	0.7726432075	0.9999537652	-1.2361179311
1.2496769766	0.4952852787	0.9999537652	1.1168570711
0	0	0	2.996475627
-1.0189614409	0.9618229175	0.9999537652	1.3452097851
1.6018473574	0.693917909	0.9999537652	8.8639046058
1.3952070747	0.6767646104	0.9999537652	2.0089640792
-1.16505023	0.6108144604	0.9999537652	-1.014166157
2.7933974408	0.6948713406	0.9999537652	0
-1.181565353	0.488518357	0.9999537652	1.2863765844
1.0114183383	0.9633928542	0.9999537652	-1.0603336318
1.2396186463	0.2701981128	0.9999537652	-1.0167170124
1.194447843	0.2734036408	0.9999537652	1.266549411
1.0538310802	0.6446619435	0.9999537652	1.1177040158
-1.5200323825	0.1349310678	0.9999537652	1.1373076228
-1.0929237384	0.6672455936	0.9999537652	-1.1521827216
-1.0186716739	0.9019037425	0.9999537652	1.2278441686
9.8205505243	0.3808440684	0.9999537652	1.7769408362
1.0985653498	0.4301505877	0.9999537652	1.026693314
-1.9370249802	0.3050917988	0.9999537652	-1.1368943321
-1.3184660385	0.2700227623	0.9999537652	1.1329284071
1.0809755048	0.5490760881	0.9999537652	1.3359047412
1.0623709753	0.7626878612	0.9999537652	1.2378963413
1.0336048792	0.8720139777	0.9999537652	1.2352826647
-1.0797577441	0.5842578716	0.9999537652	1.2374267913
1.0707806375	0.6205358691	0.9999537652	1.5370034658
-1.1516975413	0.2717871967	0.9999537652	1.0391274319
1.0097571943	0.9447296052	0.9999537652	1.5122308347
0	0	0	4.9527501949
-1.2839015029	0.1805765691	0.9999537652	-1.1427614548
1.0182947114	0.939739388	0.9999537652	1.0387785861
1.6869826229	0.0143497632	0.9999537652	1.173749672
1.1168637133	0.6259076265	0.9999537652	1.0815119824

0	0	0	36.3443360834
1.3990463717	0.0897346325	0.9999537652	1.0126625903
1.1369777985	0.5987380087	0.9999537652	1.1374388297
1.3845416171	0.4033059594	0.9999537652	1.0315740338
1.070345413	0.6077962166	0.9999537652	1.3422463993
-1.0421333985	0.7939308726	0.9999537652	1.253430513
-1.026203725	0.885839766	0.9999537652	1.2459959983
-1.0472308498	0.8153839882	0.9999537652	1.0829484399
-2.0879819015	0.1714065322	0.9999537652	-1.5174351876
-1.2143283383	0.538169455	0.9999537652	-1.4505459521
1.0100880939	0.9490736331	0.9999537652	1.2933245531
-1.3970752264	0.4291431202	0.9999537652	-1.3216462151
18.5983228161	0.2583489815	0.9999537652	4.9178905533
1.1093446212	0.5429038998	0.9999537652	1.2446208157
1.017707045	0.9171895947	0.9999537652	-1.198899296
-1.4194804336	0.3044234307	0.9999537652	1.8974404564
0	0	0	1.7042814026
-4.3884604323	0.0126324025	0.9999537652	-1.181329099
-3.140785573	0.001264	0.9999537652	-1.444469972
1.04912148	0.7961039067	0.9999537652	1.3016380803
1.1270986227	0.8600806696	0.9999537652	-1.2785952114
-1.166524873	0.860779544	0.9999537652	1.576587674
1.3227096637	0.400671865	0.9999537652	-1.5735506893
-1.4288788366	0.5987652662	0.9999537652	-2.2203993938
1.0012832251	0.9942563745	0.9999537652	1.2829118309
1.0283302552	0.879513622	0.9999537652	1.2780404681
1.1188037212	0.6282817788	0.9999537652	-1.027034897
-1.047199372	0.7719144965	0.9999537652	1.2284220271
0	0	0	-1.1248877899
0	0	0	2.996475627
-1.1429576332	0.4380556582	0.9999537652	1.0096768151
1.1302581055	0.6202606752	0.9999537652	1.2488862235
-1.0091741698	0.949578719	0.9999537652	1.3181271265
-1.1954252144	0.4940107287	0.9999537652	-1.0565164767
-1.035202297	0.8128492469	0.9999537652	1.1231014417
-1.0820310994	0.6640628498	0.9999537652	-1.3459739525
-1.2237499106	0.7441372579	0.9999537652	2.3524535037
4.5505976667	0.5557790267	0.9999537652	-3.3841961096
-1.4498385823	0.1105566022	0.9999537652	-1.1619056393
1.0058544808	0.9636617449	0.9999537652	1.1582769028
1.0474354718	0.742956304	0.9999537652	1.3200009309
-1.0721992114	0.7659324767	0.9999537652	-1.0344306034
2.7933974408	0.6948713406	0.9999537652	0
0	0	0	8.8639046058
-1.075820225	0.6999965317	0.9999537652	1.0495168842
1.2762043335	0.1271720537	0.9999537652	1.2978543044
-1.0380856761	0.7799799112	0.9999537652	1.0992341518
2.7933974408	0.6948713406	0.9999537652	2.2926838869
1.0900886499	0.8895462733	0.9999537652	1.1127752407

-2.0411599594	0.2377865367	0.9999537652	-1.1307727859
1.3505325057	0.6953045765	0.9999537652	2.9988886918
-1.1854717225	0.1940370141	0.9999537652	-1.0627580213
-1.0427400921	0.7632897574	0.9999537652	1.3438184196
5.0059881963	0.1255547862	0.9999537652	12.801795161
1.2694556548	0.3949727424	0.9999537652	1.4702705073
15.0880352532	0.2948920595	0.9999537652	-1.5255688471
6.3075231393	0.4854057018	0.9999537652	-1.3601489831
1.7423697864	0.1004776416	0.9999537652	1.3010908179
1.0327732145	0.8872965433	0.9999537652	1.0340505057
-1.0597204291	0.7505854911	0.9999537652	1.4994133365
8.0712632467	0.4228920926	0.9999537652	3.8090921162
3.7984577094	0.2220478677	0.9999537652	2.0131819221
1.0222993647	0.8935020036	0.9999537652	-1.0281449369
-1.0647587572	0.8154719438	0.9999537652	-1.0389380136
4.5505976667	0.5557790267	0.9999537652	-1.5100069453
2.8162276504	0.3669573302	0.9999537652	-1.1451545078
-1.2605653319	0.2427719424	0.9999537652	-1.0718841671
-1.1112163725	0.4887193022	0.9999537652	1.3003759484
-5.692811393	0.076858607	0.9999537652	2.6107368452
-1.0823609808	0.5743824003	0.9999537652	-1.0446859488
9.8205505243	0.3809193616	0.9999537652	2.996475627
1.1397146838	0.6935032981	0.9999537652	1.5864914288
-1.149802149	0.343812271	0.9999537652	1.0067476133
1.5869633341	0.2897162818	0.9999537652	1.0472842668
1.5325809924	0.2261985587	0.9999537652	1.0333966667
1.044127911	0.7646311458	0.9999537652	1.205305723
-1.2159646883	0.4730642701	0.9999537652	-1.1563072121
-1.1774067813	0.3927347504	0.9999537652	-1.0380361536
1.5021386016	0.001302	0.9999537652	1.3466271437
1.0925853437	0.7457817768	0.9999537652	-1.0736265352
18.6140621435	0.2552147836	0.9999537652	1.2603257066
6.3075231393	0.4853827079	0.9999537652	-1.4683576122
1.1769892637	0.4082943246	0.9999537652	1.0281442147
1.3896688665	0.0785759688	0.9999537652	1.5067871232
4.5544554512	0.5691293981	0.9999537652	-14.149476906
1.2079525956	0.4204395495	0.9999537652	-1.1108688054
1.4643107975	0.586147239	0.9999537652	2.767147042
-1.0296811672	0.824512758	0.9999537652	-1.2185767031
4.7592049098	0.1554062325	0.9999537652	-1.0728752675
1.312793133	0.5878123954	0.9999537652	1.851411927
1.0193705161	0.9477470752	0.9999537652	1.4358215607
1.143668494	0.3131867028	0.9999537652	1.0820291482
-1.3565263824	0.6051338741	0.9999537652	-1.1987244464
-1.1638388551	0.2395762942	0.9999537652	-1.0312762123
-2.8699603703	0.1713714843	0.9999537652	-2.1379338358
1.3118119729	0.1785752981	0.9999537652	-1.0412639393
-1.0929209539	0.5829118826	0.9999537652	-1.0169992171
-1.2040857698	0.5455875109	0.9999537652	1.2014730137

-1.1075595053	0.5086236562	0.9999537652	-1.31003991
-1.6674095463	0.6210110914	0.9999537652	1.5902685827
1.0537889709	0.8103712791	0.9999537652	1.2075530448
17.3472938331	0.2975749715	0.9999537652	1.9416545697
1.0814547753	0.6892352385	0.9999537652	1.164383578
1.1445045483	0.5861189161	0.9999537652	-1.2184354277
27.7226916631	0.2128547262	0.9999537652	-1.582751021
0	0	0	-4.5547427608
1.1364349707	0.3859972904	0.9999537652	-1.0092656345
0	0	0	0
1.1180519408	0.6268002499	0.9999537652	-1.0913554344
4.5505976667	0.5557790267	0.9999537652	-3.2022480197
11.5766527773	0.3453898319	0.9999537652	-1.2898805803
16.8433158146	0.2735276478	0.9999537652	8.8639046058
3.0590680122	0.3355942233	0.9999537652	12.7820459093
1.1949607999	0.4285244635	0.9999537652	-1.0349120057
-1.2453533991	0.1431774087	0.9999537652	-1.023401472
-1.1047735058	0.4346912562	0.9999537652	-1.0500406904
1.0244986373	0.9705256529	0.9999537652	1.0401486537
1.8447991669	0.5967896241	0.9999537652	-2.9707609366
-1.174661995	0.7906572339	0.9999537652	1.2181218538
-1.1007256046	0.8228118355	0.9999537652	1.3811522028
-1.0743517271	0.7630609126	0.9999537652	1.1643351917
-1.0804386955	0.5862978887	0.9999537652	-1.0673268355
0	0	0	-2.1292399642
-6.9736190158	0.2250321147	0.9999537652	-3.1664209003
1.0142654541	0.9685053513	0.9999537652	1.0565165854
-1.0600956368	0.9084150813	0.9999537652	1.181478472
2.7957660672	0.7054951009	0.9999537652	0
-1.2192482365	0.2320886472	0.9999537652	-1.0212113906
0	0	0	1.0667457716
-1.0835637166	0.6958731352	0.9999537652	1.2070098446
1.1071476644	0.5655334153	0.9999537652	1.101474769
1.0043259449	0.9818605619	0.9999537652	1.0098069768
1.7036365097	0.2379812747	0.9999537652	1.5744428501
-1.1298056631	0.6416280193	0.9999537652	1.1274721957
1.464469179	0.2485461267	0.9999537652	-1.1036344046
1.0894889315	0.8408469235	0.9999537652	1.0307841522
2.7933974408	0.6948713406	0.9999537652	0
-1.0306164867	0.9232654324	0.9999537652	-1.1793380639
-1.0979527679	0.6282465591	0.9999537652	1.2861463346
1.2500688203	0.4447039177	0.9999537652	1.0912702922
1.1465309051	0.2744730672	0.9999537652	1.0946903038
-1.5875118025	0.7196340801	0.9999537652	1.5217746204
13.3324809638	0.3193939983	0.9999537652	-9.7542681481
-1.1022674273	0.8986954579	0.9999537652	1.0104093403
-6.9736190158	0.2250321147	0.9999537652	2.6430925766
0	0	0	6.9133480437
1.343460578	0.2052263881	0.9999537652	-1.129135943

-1.0577155866	0.6856516963	0.9999537652	1.1611084543
1.0427158284	0.8522246407	0.9999537652	-1.1818136925
0	0	0	6.908559683
1.5203463218	0.3459694307	0.9999537652	1.1823781632
1.5788290836	0.3215338341	0.9999537652	-1.7454312517
0	0	0	6.914120153
-1.0482281847	0.6865803834	0.9999537652	1.1464280239
-2.2458766151	0.19216844	0.9999537652	2.8313672951
-1.0209798996	0.8850227394	0.9999537652	1.0311852217
-1.1583321592	0.5392011826	0.9999537652	-1.386949342
1.1131573272	0.8148101776	0.9999537652	-1.2483010586
1.496328291	0.0743668191	0.9999537652	-1.1091387947
-2.6929317699	0.0112540573	0.9999537652	1.1319496294
1.1456471223	0.4867548765	0.9999537652	-1.0886608589
-2.1263939687	0.204551414	0.9999537652	-1.3804393286
1.1077115801	0.6003899004	0.9999537652	-1.0736581053
2.237850947	0.0886800987	0.9999537652	1.564098443
-1.0196623059	0.8917567879	0.9999537652	1.5107441105
11.5864583426	0.3448177155	0.9999537652	10.818785458
1.2604470262	0.3031533674	0.9999537652	1.3406360957
0	0	0	-1.6597328868
-1.0801223135	0.814623867	0.9999537652	1.2176057957
1.1470270058	0.461487662	0.9999537652	-1.1741455177
-1.0789960003	0.7197221612	0.9999537652	1.038129561
-1.022384353	0.961989132	0.9999537652	-1.0024134507
-1.5567356743	0.4801670548	0.9999537652	-2.3154224155
1.0134901939	0.9262237882	0.9999537652	-1.1395113775
1.1212145381	0.9050256353	0.9999537652	-1.0737434018
4.5505976667	0.5557790267	0.9999537652	0
-1.4166339314	0.0849724764	0.9999537652	1.0236954255
1.1112993539	0.5472571239	0.9999537652	1.0304297882
-1.1453231638	0.4050469004	0.9999537652	-1.7470191562
1.4318390876	0.4198837708	0.9999537652	1.448689926
3.3030568913	0.2925089316	0.9999537652	-2.2485377414
1.7591019097	0.2579019412	0.9999537652	1.1303443867
-1.0222492834	0.9518980533	0.9999537652	-1.0171224643
1.1639684027	0.8286960727	0.9999537652	1.366248638
13.355081045	0.3179414131	0.9999537652	-3.2847784345
1.1639417098	0.2671624503	0.9999537652	-1.0083642169
-1.1141622118	0.5109920465	0.9999537652	-1.1646942619
-4.5432040502	0.0756781999	0.9999537652	1.3313172299
3.0628221549	0.312101358	0.9999537652	1.5870799612
1.5016491002	0.2721995741	0.9999537652	-1.1198849425
1.6692870837	0.1423457017	0.9999537652	-1.0606229546
-1.5844901692	0.1002629122	0.9999537652	-1.6056492038
6.3075231393	0.4853597026	0.9999537652	1.2431398771
-1.5547694709	0.4954473292	0.9999537652	-1.1279694059
1.1800404199	0.4974495111	0.9999537652	-1.1253404591
-1.2852554392	0.1075441737	0.9999537652	-1.2096082349

0	0	0	1.4803181279
-1.1347286286	0.4161906141	0.9999537652	1.3453385346
-1.0662583978	0.8671585953	0.9999537652	-1.7936363489
1.3024576124	0.1407146023	0.9999537652	1.1651630734
-1.6650408433	0.3908878953	0.9999537652	-1.4137706238
1.6024984014	0.6863056391	0.9999537652	-1.3092040421
-1.6261259292	0.5848320959	0.9999537652	3.1670230084
-1.3746425094	0.4007011966	0.9999537652	-8.0515593985
-1.1188315859	0.6842166205	0.9999537652	-1.2144594074
0	0	0	4.9527501949
-1.1750986341	0.4841420198	0.9999537652	-1.2306339465
2.1610197243	0.2916162694	0.9999537652	1.3312219541
-2.5905607418	0.0973772776	0.9999537652	43.2296245206
1.270421642	0.102669523	0.9999537652	-1.3189983627
1.386423469	0.1103996374	0.9999537652	1.1609931314
1.0041291717	0.9884729546	0.9999537652	1.0284012339
20.3709255077	0.240489183	0.9999537652	14.7475552392
1.4093534306	0.3580348173	0.9999537652	-1.5693334717
1.1722488518	0.3536499462	0.9999537652	-1.6454014177
-1.2009907539	0.5731856972	0.9999537652	1.3816017983
-1.0085126764	0.9674741655	0.9999537652	1.1390330146
1.1976693016	0.2878494987	0.9999537652	-1.1142365549
8.4228148819	0.0442145594	0.9999537652	-2.6201438026
-1.4756243598	0.5433632425	0.9999537652	1.080343006
1.0625902878	0.8863953164	0.9999537652	-1.4535543675
-3.8981079135	0.1326283676	0.9999537652	-1.0941138562
1.1914602592	0.3627065558	0.9999537652	-1.1336367724
1.8447991669	0.5967824527	0.9999537652	-1.0581652744
1.229529627	0.1625618535	0.9999537652	1.0358171438
13.3324809638	0.3193853126	0.9999537652	4.9527501949
0	0	0	0
-6.9736190158	0.2250321147	0.9999537652	4.9527501949
-1.1272725815	0.563741911	0.9999537652	-1.1227862798
-1.1955728427	0.3572090417	0.9999537652	1.4100211527
6.3075231393	0.4853366858	0.9999537652	-1.4680135518
-1.049396749	0.8455164725	0.9999537652	-1.6460911629
1.4797261816	0.4834765546	0.9999537652	1.0132811597
-1.2923745811	0.1893206296	0.9999537652	-1.0319453723
1.0188399558	0.9623852445	0.9999537652	1.2633273242
1.0471365914	0.8092486596	0.9999537652	1.3899103597
1.2672497572	0.4238195182	0.9999537652	-1.0103219526
1.6220063926	0.2336785013	0.9999537652	1.3628564202
-1.2414433923	0.7398211739	0.9999537652	1.1346215583
-1.1567926223	0.4880813222	0.9999537652	1.3328463923
1.322335606	0.3730068283	0.9999537652	1.2417108583
1.2038538633	0.1875600249	0.9999537652	1.273542703
1.4224320209	0.417595321	0.9999537652	-1.4180425176
-1.0027836106	0.9879458144	0.9999537652	1.8492853581
-1.3981186001	0.0789106088	0.9999537652	1.1432867529

-1.2263960989	0.3995316732	0.9999537652	1.2093557034
1.1485389377	0.4477027142	0.9999537652	1.173531842
1.1955604706	0.464322743	0.9999537652	1.0742605181
1.221805472	0.1667195927	0.9999537652	1.3859930266
1.0864708467	0.5840330441	0.9999537652	-1.1043921972
-1.0029694647	0.99247297	0.9999537652	1.1467698557
-1.205655428	0.553875664	0.9999537652	1.630299082
-1.5225759426	0.2877456912	0.9999537652	1.0408043491
1.0063316484	0.9742185617	0.9999537652	1.1618705071
-1.1294640322	0.7719368238	0.9999537652	1.5265536804
1.9158642511	0.1390056266	0.9999537652	2.0229936606
-1.1110929768	0.4721393467	0.9999537652	1.0436023954
0	0	0	2.9985540909
1.3661434872	0.2282654102	0.9999537652	1.0880269019
1.338277756	0.1431578718	0.9999537652	-1.0500557846
-1.5875118025	0.7200716995	0.9999537652	0
5.9843348305	0.0892649912	0.9999537652	3.1668299586
1.0635210123	0.8716494314	0.9999537652	1.5179334301
-1.2366111617	0.6802445842	0.9999537652	1.1660774039
1.2620661248	0.238329277	0.9999537652	1.0471036173
1.0601682245	0.7494128605	0.9999537652	-1.0692399349
1.6153845695	0.3526556207	0.9999537652	1.0343427953
1.1076354351	0.5859625956	0.9999537652	1.1459832143
0	0	0	2.996475627
1.0278927117	0.8062560011	0.9999537652	1.1031580594
1.2181904606	0.1979556593	0.9999537652	1.1894915044
-1.0367108132	0.7748942372	0.9999537652	1.1357612808
1.1712788023	0.448544156	0.9999537652	1.1190533384
1.1847522967	0.529325654	0.9999537652	1.7838137732
-1.2630572068	0.3963821605	0.9999537652	-1.0374832999
-1.7273247423	0.4757529256	0.9999537652	1.899154088
-1.0373643103	0.8911391489	0.9999537652	1.9268931499
-1.1161406883	0.6856540102	0.9999537652	1.1411752257
-1.0664931696	0.6157412751	0.9999537652	1.1435636088
-1.5800919873	0.2165924087	0.9999537652	1.4589382486
1.0970578367	0.8131231029	0.9999537652	-1.421780825
-1.2703108722	0.450818258	0.9999537652	1.3293906638
-1.1930284735	0.4993493788	0.9999537652	2.2968992123
1.0508109119	0.9163795976	0.9999537652	-1.2032318277
-1.0629755097	0.8356483978	0.9999537652	1.4002849445
1.5707075507	0.5527715905	0.9999537652	2.3843565867
1.1047381258	0.7904938338	0.9999537652	2.2223039899
-1.1337429989	0.5096982934	0.9999537652	1.0395829044
1.6175850298	0.6135732969	0.9999537652	-2.225399492
1.0517703627	0.7809389694	0.9999537652	1.1215434217
-1.4336299357	0.4561288407	0.9999537652	-1.044632029
1.2409028678	0.1354022209	0.9999537652	1.2722886121
3.8622037496	0.6194645299	0.9999537652	2.719037262
1.346591354	0.1118672443	0.9999537652	1.3432864832

-1.0731179781	0.6628058369	0.9999537652	1.081187033
-3.7721528137	0.034595666	0.9999537652	4.5410297837
1.0469822222	0.8546887483	0.9999537652	1.143743146
1.0349597892	0.9371831907	0.9999537652	-1.071202464
1.9042601795	0.4857971403	0.9999537652	-1.2779826415
1.108582374	0.4736578487	0.9999537652	1.0746868286
1.1769915264	0.2672716477	0.9999537652	1.3445445725
-1.0191824441	0.9511598066	0.9999537652	1.0413611366
-1.4123278392	0.0557332298	0.9999537652	1.1040275
-1.0313543946	0.9267516344	0.9999537652	-1.0434166215
-1.0060206103	0.9725821843	0.9999537652	1.3529064741
1.307470285	0.0601549708	0.9999537652	1.459239867
-1.0780766494	0.7342025656	0.9999537652	1.2372933796
1.5950355194	0.2370865165	0.9999537652	-1.1878985118
1.8447991669	0.5962138556	0.9999537652	-1.0597351848
-1.0506933818	0.7848519371	0.9999537652	-1.3227504982
-1.5066460521	0.2338319023	0.9999537652	1.207216514
1.13469086	0.6367260158	0.9999537652	-1.0917463776
-1.0325789148	0.8328090182	0.9999537652	1.2341242213
-1.0168813118	0.9347955731	0.9999537652	-1.0997922981
-1.0690801504	0.8105778621	0.9999537652	1.5416487068
-1.0951686159	0.81806383	0.9999537652	1.2463798261
1.3081855134	0.5433589281	0.9999537652	1.3903161209
0	0	0	0
1.2890103725	0.0829647326	0.9999537652	1.2550870371
-1.2135779811	0.6138261942	0.9999537652	-1.0050453798
-1.6044090876	0.2248180855	0.9999537652	1.1169595848
1.3144208192	0.1577335197	0.9999537652	1.3263114485
1.5956389477	0.0392846676	0.9999537652	1.1558040718
-1.0104578462	0.966204329	0.9999537652	1.0767467946
2.7957660672	0.7054974687	0.9999537652	2.996475627
1.3998044119	0.0563819532	0.9999537652	1.1545606783
-1.0343543478	0.8134596441	0.9999537652	-1.0521291579
1.2386449094	0.3781672837	0.9999537652	-1.0145827841
1.049401216	0.9114587428	0.9999537652	1.8296666597
-1.0121961358	0.9760891688	0.9999537652	1.1710513718
1.0037567808	0.9885811225	0.9999537652	1.0525990101
-1.854982839	0.6694971359	0.9999537652	-1.8582093966
1.1108134048	0.8092673189	0.9999537652	1.6323443946
-2.0049727571	0.4873925634	0.9999537652	-1.1597631554
-1.0335985921	0.943810092	0.9999537652	-1.3017482893
1.1020291412	0.7628199992	0.9999537652	1.1981087661
2.7933974408	0.6948713406	0.9999537652	12.7909013375
-1.3942835591	0.2026501885	0.9999537652	1.153944849
1.1169355694	0.6462479617	0.9999537652	1.1607139985
1.0049860966	0.9823780311	0.9999537652	1.1533018321
0	0	0	8.8639046058
1.4298393692	0.4055482656	0.9999537652	1.2028167091
1.0809311942	0.822193759	0.9999537652	-1.2968981054

1.1723002888	0.7755492984	0.9999537652	1.2584485193
0	0	0	4.9527501949
-1.1146306381	0.7224277023	0.9999537652	1.1314939616
1.2310686261	0.6065656306	0.9999537652	1.3482609657
0	0	0	2.2924151284
2.7933974408	0.6948713406	0.9999537652	0
11.5766527773	0.345362513	0.9999537652	0
-1.0311616413	0.8737358736	0.9999537652	1.2836234747
1.008818647	0.9641574001	0.9999537652	-1.0925167214
-2.1004794521	0.1140863462	0.9999537652	1.2123204254
2.1881200327	0.2125230807	0.9999537652	1.0483600032
1.0620987388	0.695432061	0.9999537652	1.226991701
-1.0637461542	0.7935483252	0.9999537652	-1.0272174654
-2.1334630094	0.4839493564	0.9999537652	-14.149476906
0	0	0	4.9527501949
-1.5799022448	0.5967477828	0.9999537652	-1.2021592749
-1.3164847373	0.5305101405	0.9999537652	-1.153587748
1.1093907672	0.5761776071	0.9999537652	1.0012044607
-1.4476378234	0.515261142	0.9999537652	-1.6746323835
4.5505976667	0.5557790267	0.9999537652	0
-17.0527724669	0.1063772664	0.9999537652	1.0490376413
-1.0071249919	0.960616034	0.9999537652	-1.1683296871
-1.2613224469	0.598119673	0.9999537652	-1.0010455891
-1.1246002998	0.6069520815	0.9999537652	1.024409277
-1.2162776307	0.536136465	0.9999537652	1.2507550533
1.0616364134	0.7706767992	0.9999537652	-1.231484353
1.1934306586	0.2079402082	0.9999537652	1.1638087086
1.5700030029	0.0702062927	0.9999537652	-1.0900414669
-4.1203521956	0.03258327	0.9999537652	2.0679121385
1.3279689902	0.4163517252	0.9999537652	1.3429089002
-1.0453550858	0.8306909358	0.9999537652	1.0436755596
1.0521854961	0.8190653786	0.9999537652	-1.0174301226
1.0116229213	0.9428882902	0.9999537652	1.1650180223
0	0	0	0
-1.0418583109	0.8414265238	0.9999537652	-1.234243132
1.3767654965	0.2401189146	0.9999537652	1.0250709488
2.8256295203	0.0876726021	0.9999537652	-1.0605276563
1.2019298597	0.1711736834	0.9999537652	-1.1479730021
-1.8105080737	0.0211818987	0.9999537652	-1.0163270948
-1.2938717281	0.687251086	0.9999537652	2.0225373915
-3.4222690496	0.2994818546	0.9999537652	4.1560398863
1.1566482891	0.2518375702	0.9999537652	-1.1466207482
1.2692032851	0.3283732647	0.9999537652	1.0555183433
1.1039120407	0.7054029048	0.9999537652	1.2021853784
1.0585637429	0.667752531	0.9999537652	-1.132745483
-1.1711577976	0.1943509975	0.9999537652	-1.0667380786
0	0	0	1.260030389
1.0170424609	0.941452401	0.9999537652	-1.1806607509
0	0	0	6.908559683

8.0710074692	0.4204062628	0.9999537652	-1.420807078
1.2404356038	0.3941941101	0.9999537652	1.1978546347
1.3228796398	0.2634363733	0.9999537652	-1.116176739
-3.0771439936	0.0405164484	0.9999537652	2.2026102564
1.1103672411	0.632890972	0.9999537652	1.0456058944
-1.1392831724	0.6706716072	0.9999537652	-1.5572884298
2.7933974408	0.6948713406	0.9999537652	0
1.0819131344	0.8560233292	0.9999537652	1.0227701733
1.5635972403	0.3004749494	0.9999537652	-1.5327178342
-1.122875753	0.4807521998	0.9999537652	1.0325514248
1.0778072992	0.7285317151	0.9999537652	-1.2836897126
1.2303693658	0.6754356443	0.9999537652	-1.0854573451
-1.3633757889	0.1496354067	0.9999537652	-1.028651198
1.0807141003	0.90751728	0.9999537652	-1.7221998916
1.4556800965	0.0920771429	0.9999537652	1.4551228482
0	0	0	12.7732027325
0	0	0	2.996475627
1.1755982974	0.7552796319	0.9999537652	1.3437952164
1.0445159967	0.9279536782	0.9999537652	1.1823907463
-1.1593391779	0.5518276892	0.9999537652	1.021371221
-1.0484216212	0.9381883126	0.9999537652	1.1963620661
-2.0288012188	0.2226599793	0.9999537652	-1.5746344992
1.8993989353	0.0845766511	0.9999537652	-1.1521524629
1.7949800932	0.2806698722	0.9999537652	-2.0756169608
-1.4127194569	0.4928967744	0.9999537652	1.0570586743
-1.7413885129	0.3946726132	0.9999537652	1.8448324843
1.3837390216	0.4604357121	0.9999537652	1.2982922721
-1.6255754634	0.0426127442	0.9999537652	-1.3990777792
-1.025896251	0.9477053077	0.9999537652	-1.1655552762
11.5766527773	0.3453729851	0.9999537652	-1.0931530667
-1.1029785928	0.5788676233	0.9999537652	-1.1356617337
-1.1634848613	0.5710168884	0.9999537652	-1.0940235868
-2.4084168057	0.2472137932	0.9999537652	1.7585914753
1.0030120122	0.9914984035	0.9999537652	-1.1885298623
1.0454929694	0.7720673323	0.9999537652	1.2249918822
-1.0358108118	0.8680303025	0.9999537652	1.2271603724
-1.0404315745	0.956800981	0.9999537652	-1.5200437261
1.8997719676	0.0165238457	0.9999537652	-1.1186551763
0	0	0	4.9527501949
4.5505976667	0.5557790267	0.9999537652	-2.2049479182
1.141719395	0.5709505107	0.9999537652	-1.0810232217
-1.1923561156	0.5378501573	0.9999537652	1.3408938512
3.3030568913	0.2923090227	0.9999537652	1.5762331035
-1.0953312734	0.6445886497	0.9999537652	1.0558976974
-1.2097854419	0.1895125059	0.9999537652	1.1141685225
1.0909697259	0.7877855123	0.9999537652	-1.0303647856
1.0524126307	0.7821868917	0.9999537652	1.0656804465
-1.1344589861	0.4229985513	0.9999537652	-1.1969739175
-1.1017238568	0.4614971942	0.9999537652	1.1185268517

-1.0892590523	0.6466924552	0.9999537652	1.1152295502
1.0713018552	0.8306544224	0.9999537652	-1.4398459869
1.0349118986	0.96866929	0.9999537652	1.70049381
-1.1708143936	0.8368412765	0.9999537652	1.3560121951
-1.0167048523	0.9313869939	0.9999537652	1.0493948245
16.8581089812	0.2738049063	0.9999537652	-2.1752099359
1.2905180359	0.3931371891	0.9999537652	-1.5866582948
-1.0143489742	0.9768810865	0.9999537652	1.1246972007
0	0	0	4.9527501949
-1.4540659285	0.1175072252	0.9999537652	-1.6380064068
1.7777127261	0.5426913471	0.9999537652	-2.0451275073
13.3441949841	0.3177230898	0.9999537652	4.398765428
-1.5732382151	0.0653873188	0.9999537652	1.5248238359
0	0	0	0
1.2225264535	0.7082255851	0.9999537652	1.0134225724
1.6560510752	0.1363434874	0.9999537652	-1.5269385376
1.2022733572	0.7147355263	0.9999537652	1.259119136
-1.1354041114	0.545249206	0.9999537652	-1.0772316405
0	0	0	4.9527501949
-1.3045064545	0.7717955884	0.9999537652	1.1490503071
1.7775859945	0.2286185605	0.9999537652	-1.1666805438
1.3264454653	0.0996002051	0.9999537652	-1.2923929199
-1.0084662653	0.958163175	0.9999537652	1.0319762474
1.7330527012	0.2137272843	0.9999537652	1.3178864572
-1.0888977827	0.4909023804	0.9999537652	-1.0929777658
-1.1698447291	0.4025330229	0.9999537652	-1.1606639723
-2.9523355565	0.3410275525	0.9999537652	3.6975953223
1.0882534344	0.8474813457	0.9999537652	-1.0245128851
0	0	0	0
-1.1692788994	0.6710113129	0.9999537652	1.0729673733
6.5591783221	0.1221769227	0.9999537652	1.0468043088
-1.5554802511	0.0202089415	0.9999537652	-1.2094762063
1.1123900346	0.5430608154	0.9999537652	1.1928207466
2.7933974408	0.6948713406	0.9999537652	0
1.2901571405	0.5274507438	0.9999537652	-1.0116744335
-2.4076384939	0.261806788	0.9999537652	1.3454879954
-2.9548384089	0.3320364738	0.9999537652	1.1075715118
4.5505976667	0.5557790267	0.9999537652	0
-1.1897399587	0.4908028593	0.9999537652	1.2188167332
1.0012418124	0.9979944043	0.9999537652	6.4574967663
1.4381425725	0.4704268886	0.9999537652	-1.1614254091
-1.0369407296	0.8158419845	0.9999537652	-1.039504396
1.6102122309	0.2510982009	0.9999537652	-1.1551840315
-1.2192096094	0.4000317338	0.9999537652	-1.314799552
1.0139488168	0.9186419636	0.9999537652	-1.0968945811
-2.606596754	0.004068	0.9999537652	-1.3800523585
-1.0825892839	0.5698532677	0.9999537652	-1.0588478572
-1.217629373	0.129732958	0.9999537652	-1.077886714
1.1155246223	0.6647937502	0.9999537652	-1.0446292135

-1.4315318565	0.1232567893	0.9999537652	-1.2045274738
-1.3032274429	0.2599483305	0.9999537652	1.7602086529
-1.2730640585	0.2581881774	0.9999537652	-1.2737942327
1.0497175022	0.7907591056	0.9999537652	1.1565049455
8.064174035	0.4222712946	0.9999537652	-1.2567496987
-1.0279577398	0.9591477357	0.9999537652	1.1962074888
-1.0397128837	0.8711281519	0.9999537652	-1.579571179
1.1577629587	0.5279802488	0.9999537652	1.0590887738
-1.0752763322	0.5783875662	0.9999537652	1.0707166822
-1.035257715	0.8402878745	0.9999537652	-1.1270095525
-1.5881570201	0.7074470251	0.9999537652	1.4836175613
-1.0196035013	0.9499469485	0.9999537652	-1.4578345401
0	0	0	-2.1294907556
1.4215568538	0.4385950049	0.9999537652	1.16387152
1.9164640685	0.0141726048	0.9999537652	-1.0661164068
-1.2333517567	0.3787896165	0.9999537652	-1.1381043479
-1.0442260389	0.9103650259	0.9999537652	1.3556242168
-1.4162966763	0.1182381061	0.9999537652	1.7452237084
1.4517602532	0.0334107145	0.9999537652	1.2595647504
-1.4116799472	0.0604303746	0.9999537652	-1.1915942714
1.181347916	0.4744948084	0.9999537652	-1.0009216603
-1.0008213246	0.9964135212	0.9999537652	-1.1612819369
1.4663278119	0.156134944	0.9999537652	-1.6435212058
-1.2660139623	0.4542647454	0.9999537652	-1.8328251143
1.1278195782	0.4727355824	0.9999537652	-1.2061965622
1.3690635889	0.1573380805	0.9999537652	-1.1752471352
3.7950012782	0.2233498707	0.9999537652	1.6777099281
1.2185752416	0.2837202748	0.9999537652	-1.1421709287
-1.1107449644	0.885408535	0.9999537652	1.6944665328
-1.0216657614	0.969455364	0.9999537652	1.0811651952
1.2236077574	0.3909402183	0.9999537652	1.0658180481
1.3549988344	0.4899782337	0.9999537652	1.0866621179
1.0821298481	0.9047336486	0.9999537652	-1.3319334118
0	0	0	2.9985540909
-1.3372457995	0.0437376303	0.9999537652	-1.0364107525
-1.1531996456	0.6700293704	0.9999537652	-1.1423667136
-1.0345660389	0.8884461592	0.9999537652	1.7831051404
4.5505976667	0.5557790267	0.9999537652	-1.5100069453
-1.0668050857	0.6138377127	0.9999537652	1.0710860867
-1.1244953179	0.4456959952	0.9999537652	1.2170575577
11.5766527773	0.3453834666	0.9999537652	1.3574283852
-1.1227231237	0.494563338	0.9999537652	1.0559602783
-1.6001912718	0.0338141264	0.9999537652	-1.1883124001
1.154207681	0.6248566768	0.9999537652	-1.4118498049
4.5505976667	0.5557790267	0.9999537652	2.2825222368
1.1262065255	0.7043547904	0.9999537652	1.0808569045
-7.8342489948	0.0136573932	0.9999537652	1.5842650109
1.0629048358	0.7460915252	0.9999537652	1.0661001028
0	0	0	1.1700186664

1.0869907045	0.5825344441	0.9999537652	1.1379604044
1.7391982989	0.0257888344	0.9999537652	-1.5369179689
-1.0933042691	0.7082032224	0.9999537652	-1.0678348956
-1.1066761544	0.6643157542	0.9999537652	1.0315091038
1.0349994282	0.9675313124	0.9999537652	-1.3269334757
1.221981633	0.7018225786	0.9999537652	-1.6284361991
0	0	0	2.996475627
1.109848328	0.5394180148	0.9999537652	1.1130275869
-1.0234269768	0.8790014282	0.9999537652	-1.2856167514
1.002632797	0.9812588525	0.9999537652	1.1126210608
1.0952316116	0.7158993795	0.9999537652	-1.4723183875
1.9491018051	0.3202018509	0.9999537652	1.7141474805
-1.2644984285	0.4652834071	0.9999537652	-1.972375129
-1.2717055773	0.4875279292	0.9999537652	1.0859797898
1.1482262978	0.6957543752	0.9999537652	1.1685800277
1.5327931967	0.2345172935	0.9999537652	-1.3469064259
-1.0251835228	0.8944765121	0.9999537652	1.033414963
-1.2336678355	0.3267077319	0.9999537652	1.0413508965
-2.3052484641	0.2776253318	0.9999537652	1.2507078744
-1.3027577835	0.1401717335	0.9999537652	-1.0314141135
-2.587194901	0.5006470995	0.9999537652	-1.8582093966
1.0095657304	0.9439216259	0.9999537652	-1.0205323068
1.0407237678	0.7801667772	0.9999537652	-1.1523577522
2.330589113	0.4664892188	0.9999537652	-1.5705467174
-1.0001753882	0.999358114	0.9999537652	-1.1367806427
-1.0401261452	0.8278088685	0.9999537652	-1.2423304861
1.0734173562	0.7186913917	0.9999537652	1.2275169558
-1.0678286543	0.6722408651	0.9999537652	-1.1856729112
-1.8319526048	0.0618747103	0.9999537652	1.0869205685
1.1833286525	0.3788698012	0.9999537652	-1.0070319458
-2.6228880748	0.0202922465	0.9999537652	-1.5057213597
1.0839546126	0.580310108	0.9999537652	-1.0120983611
1.2521963851	0.5775405818	0.9999537652	1.0711962509
-1.5863269394	0.0464677276	0.9999537652	-1.0959859932
2.7933974408	0.6948713406	0.9999537652	1.5756933516
1.0182112369	0.9060000298	0.9999537652	-1.0904500673
-1.2738619992	0.2045482531	0.9999537652	-1.1058163778
1.4309845342	0.4068681391	0.9999537652	-1.0027200464
0	0	0	0
4.5505976667	0.5557790267	0.9999537652	1.2603189694
-1.4963644574	0.6151081976	0.9999537652	1.897906693
1.0283194864	0.8471230877	0.9999537652	-1.0350044613
-1.3718088005	0.3061485935	0.9999537652	-1.3356371379
1.3826309223	0.7329052215	0.9999537652	-1.5384306948
1.776015977	0.0778270529	0.9999537652	-1.237305892
1.0013845877	0.9959051746	0.9999537652	1.1018310916
-1.0200504634	0.9176229043	0.9999537652	-1.3618678517
1.0841638385	0.6911425732	0.9999537652	-1.1098137413
1.0475940189	0.9393601759	0.9999537652	1.079650584

-1.0343284833	0.8467169807	0.9999537652	1.1445304789
-6.9764533263	0.2593700317	0.9999537652	8.8639046058
-1.2618517205	0.5872110544	0.9999537652	1.03963227
-1.268795569	0.3429782844	0.9999537652	-1.0812747137
2.258038479	0.0776225326	0.9999537652	1.5262115505
3.8622037496	0.6194337597	0.9999537652	0
-6.9762758667	0.257419656	0.9999537652	-5.3583704783
1.0549349563	0.7350195196	0.9999537652	1.1084324612
-1.1340167542	0.4922289578	0.9999537652	1.1427608641
1.225993467	0.3843798377	0.9999537652	1.1091584351
2.108863471	0.0002225	0.9999537652	1.4861586109
1.8696685518	0.0114358782	0.9999537652	1.1592213224
-1.0417605612	0.7824791725	0.9999537652	1.0070508378
-1.5875118025	0.7203177862	0.9999537652	-1.1248877899
1.8366107518	0.4261058267	0.9999537652	-1.3519310275
-6.9736190158	0.2250321147	0.9999537652	16.6921882404
-1.0518570978	0.7745174959	0.9999537652	-1.0804291815
9.8205505243	0.380994976	0.9999537652	-1.1567178131
2.4449452442	0.0215423507	0.9999537652	-1.0522487344
1.1266438642	0.8181125702	0.9999537652	1.0595894297
-1.6686798736	0.6491343639	0.9999537652	1.5517968762
-1.1833028368	0.4789037669	0.9999537652	1.4410760795
1.3261654664	0.0872907158	0.9999537652	1.4328268746
1.0912415555	0.6737259844	0.9999537652	1.3999637636
3.899881088	0.0395998217	0.9999537652	1.1223406437
1.203940792	0.8610436641	0.9999537652	2.0098769534
-1.5487651399	0.3334051318	0.9999537652	1.0241324626
0	0	0	0
1.7703819676	0.0132340253	0.9999537652	1.088456437
-4.0807853644	0.001071	0.9999537652	-1.1190444854
0	0	0	0
0	0	0	4.7454169068
1.0563236658	0.7258605568	0.9999537652	1.4528650933
1.8618500038	0.2598939419	0.9999537652	1.7266774322
58.8883703784	0.1122314687	0.9999537652	-7.8899638767
-4.7800108543	0.0395153498	0.9999537652	1.4019884908
15.0880352532	0.2942883058	0.9999537652	0
-1.1969567826	0.3780434493	0.9999537652	1.340017756
1.1072800056	0.4275938339	0.9999537652	1.1490828701
1.2127039295	0.5586538638	0.9999537652	-1.2838220281
-2.5861438062	0.4709545055	0.9999537652	23.7230095966
-1.6952918056	0.270858941	0.9999537652	-1.7028105906
1.322151751	0.2360467642	0.9999537652	-1.8831980752
1.0084102709	0.9528638825	0.9999537652	1.0375158742
4.5505976667	0.5557790267	0.9999537652	1.029347285
-1.0262533566	0.9035952524	0.9999537652	-1.2346007941
1.0965971874	0.522694675	0.9999537652	-1.0721736523
-1.176304255	0.6774066005	0.9999537652	1.0835576938
-1.1082147911	0.4372816266	0.9999537652	1.1018351959

0	0	0	6.908559683
-2.3117555798	0.1522936756	0.9999537652	4.1809571314
-1.0561176895	0.8168034366	0.9999537652	1.0264222519
1.1571624393	0.5980152062	0.9999537652	-1.1642035708
-1.0288870077	0.8865309765	0.9999537652	1.1596997932
-1.1502223204	0.3250046162	0.9999537652	1.197561258
1.0989601209	0.764622033	0.9999537652	-1.1322601057
-1.7490652112	0.0437536281	0.9999537652	-1.5603081777
1.3152430872	0.4651088869	0.9999537652	1.1660875283
1.6810466894	0.2926172386	0.9999537652	-1.0144415291
1.1172067747	0.5476592311	0.9999537652	1.2532169093
1.3019739475	0.7507211223	0.9999537652	-1.0841536928
-6.9736190158	0.2250321147	0.9999537652	0
0	0	0	-1.3582894632
-1.7136183137	0.5421628711	0.9999537652	-1.69406917
1.0615648937	0.755256969	0.9999537652	1.1436958289
1.0582141412	0.6343580778	0.9999537652	1.1066544677
1.1535052054	0.6496127013	0.9999537652	-1.0494973352
-1.209246437	0.4901255202	0.9999537652	1.1566072698
1.0951169398	0.7674747484	0.9999537652	1.0938062629
-1.509769767	0.4059804021	0.9999537652	-1.3682707861
-1.3717433714	0.1270905411	0.9999537652	-1.2640603123
-1.2326413295	0.2714532241	0.9999537652	-1.016319789
1.8360797651	0.4045793112	0.9999537652	-1.4560556818
1.7489762908	0.4445069032	0.9999537652	-1.4786786349
1.2597970535	0.2145553382	0.9999537652	1.1343796609
1.2827647554	0.3226362006	0.9999537652	-1.0107226662
-1.1010106894	0.5496910579	0.9999537652	1.0307400844
1.3868418181	0.3165465187	0.9999537652	1.1679263073
-1.3878172573	0.1604975778	0.9999537652	-1.0817782228
-1.2386169288	0.2432868612	0.9999537652	-1.3545773447
-1.2221310789	0.2623640485	0.9999537652	1.0632928544
-1.2781903416	0.1262303852	0.9999537652	1.0055383814
1.3654685218	0.1234162627	0.9999537652	1.1746727176
-1.2962040907	0.199680731	0.9999537652	-1.2946693566
1.7357216427	0.2492998457	0.9999537652	-1.2398497886
-25.0060995147	0.0343971431	0.9999537652	20.7721997036
-1.1165787947	0.6401239183	0.9999537652	-1.0746148657
0	0	0	4.9527501949
-1.4282571996	0.5267196929	0.9999537652	-1.187405001
-1.1585090632	0.6996725321	0.9999537652	-1.2937703852
-1.2639097136	0.3072641927	0.9999537652	1.489517965
0	0	0	-5.3583704783
-1.1426437232	0.7806691147	0.9999537652	-1.0223056695
-1.1391240281	0.483221687	0.9999537652	-1.1486155181
-1.0370090734	0.8439378658	0.9999537652	1.2620746671
1.1031473388	0.4938834645	0.9999537652	1.1866843141
-1.0330311243	0.8109069692	0.9999537652	1.1948141904
1.0028853506	0.9887016687	0.9999537652	1.0662845969

1.0097518039	0.9504063144	0.9999537652	-1.225855303
1.1995078124	0.4707496883	0.9999537652	1.1617347337
-1.1475384184	0.598530024	0.9999537652	-1.2262105064
1.2673362162	0.1436364791	0.9999537652	1.0481369425
-1.0111076367	0.9709130324	0.9999537652	1.5826570617
1.1782560174	0.7375192819	0.9999537652	-1.2503926932
1.5640503221	0.0446669114	0.9999537652	-1.0130902147
1.2516955386	0.6520906508	0.9999537652	-1.1899815703
6.3075231393	0.4853175467	0.9999537652	20.6004768562
1.1291914808	0.7776980625	0.9999537652	-1.0371777014
4.5505976667	0.5557790267	0.9999537652	1.638122109
0	0	0	2.996475627
1.1787197499	0.492011671	0.9999537652	-1.1853831376
-1.2246495388	0.183827773	0.9999537652	-1.0005561089
1.3159568783	0.4587586164	0.9999537652	-1.2779765113
2.105568983	0.3166905972	0.9999537652	-1.3659900625
1.0206943064	0.9003158103	0.9999537652	-1.0630506289
1.404100928	0.5399065217	0.9999537652	-1.4923018149
-1.1584416479	0.4151841532	0.9999537652	1.5163406239
-1.0734104392	0.6731296183	0.9999537652	1.1336496883
-1.7539180866	0.0979197859	0.9999537652	-1.08115216
-1.9360854345	0.0799603983	0.9999537652	-1.3503849507
-1.1940646118	0.6014726154	0.9999537652	1.0242826737
-1.1377318032	0.5681149026	0.9999537652	-1.0498149866
1.0285673021	0.9106824305	0.9999537652	-1.2379061598
-1.2873988025	0.1637503722	0.9999537652	1.1398926157
0	0	0	2.9985540909
1.5466895784	0.078383864	0.9999537652	1.0127880788
0	0	0	4.9527501949
-1.3496643058	0.3674543041	0.9999537652	-1.1549830464
1.1024663542	0.7910956373	0.9999537652	1.3727647869
1.0706053958	0.8178919106	0.9999537652	1.1859144042
-1.0011881979	0.9946789894	0.9999537652	-1.0899245251
-1.0258778433	0.9636814017	0.9999537652	-1.1865010949
1.6052664222	0.6760285368	0.9999537652	1.7791803399
1.3766983443	0.5400571722	0.9999537652	-1.0743277914
-1.0879481565	0.831282769	0.9999537652	-1.0785973397
1.2600240288	0.7499892903	0.9999537652	2.2942711679
1.7069561144	0.001074	0.9999537652	-1.4889190041
1.2335124016	0.647802239	0.9999537652	-1.4464184743
1.0059559074	0.9585892938	0.9999537652	-1.6048774281
0	0	0	-1.4671767304
-1.2288827784	0.1669511509	0.9999537652	-1.0917155773
-1.1636046933	0.372816801	0.9999537652	1.1182547331
-1.0361672725	0.8602886397	0.9999537652	-1.0408898903
-1.8651323557	0.3127928859	0.9999537652	-1.0127291587
-1.4796663775	0.0497421794	0.9999537652	1.30273875
1.2314285161	0.3424146738	0.9999537652	1.0461096814
-1.1729715571	0.2271969892	0.9999537652	1.0761216497

-1.2310998671	0.3172221803	0.9999537652	1.1740418899
1.1262528854	0.8590423635	0.9999537652	1.9614046585
-1.1573470393	0.2221345091	0.9999537652	1.1171416851
1.2134129531	0.1733906627	0.9999537652	-1.3860873617
0	0	0	8.8700459102
16.8433158146	0.2736437241	0.9999537652	6.5010535319
9.8288704632	0.3801048341	0.9999537652	1.4360713397
0	0	0	8.8639046058
6.3075231393	0.4853079883	0.9999537652	8.8639046058
1.0412916276	0.9039008571	0.9999537652	1.0581464647
4.5204235356	0.1568737007	0.9999537652	-2.7911910562
4.5505976667	0.5557790267	0.9999537652	4.4018286016
16.8433158146	0.2736906085	0.9999537652	6.1460359069
-1.2891641163	0.1475777902	0.9999537652	1.108808396
1.0330362255	0.8074522675	0.9999537652	1.0690440539
-1.5081216981	0.0568957111	0.9999537652	-1.3608350649
2.7933974408	0.6948713406	0.9999537652	0
11.5766527773	0.3453939576	0.9999537652	15.7262184124
1.1749178842	0.2009720376	0.9999537652	1.4546797915
1.8486773713	0.597508024	0.9999537652	-1.4884129525
1.216076899	0.3069245115	0.9999537652	1.0982195965
1.3129275726	0.0364746685	0.9999537652	1.1838181617
-1.2325232486	0.4298024991	0.9999537652	1.0902899402
1.156027552	0.3062320368	0.9999537652	1.4292753205
1.1450658693	0.4301942297	0.9999537652	1.1893173501
-1.4712611897	0.5100694128	0.9999537652	-1.1386107712
1.2159527821	0.4864124209	0.9999537652	1.4973186614
1.0403016984	0.7873473631	0.9999537652	1.0249232295
-1.134323779	0.7470397286	0.9999537652	1.6751022027
2.7933974408	0.6948713406	0.9999537652	9.7116702472
1.1423122415	0.4728638022	0.9999537652	-1.0480617211
1.1901748577	0.3118724281	0.9999537652	1.0701405656
1.0883303231	0.8915836888	0.9999537652	-1.6149407038
-1.0137948708	0.9203764222	0.9999537652	1.0006690711
1.0313582086	0.8740827071	0.9999537652	1.2616895519
-1.5007506109	0.0250431019	0.9999537652	-1.0386324788
1.8528871235	0.6236356389	0.9999537652	-1.859280336
2.7933974408	0.6948713406	0.9999537652	16.6921882404
1.1197165763	0.4388440347	0.9999537652	1.3207729249
13.3324809638	0.3193766287	0.9999537652	0
1.0858146959	0.5883987354	0.9999537652	1.287777361
1.2232587176	0.4107987945	0.9999537652	1.6561638413
1.0093087785	0.9536238723	0.9999537652	1.2808469015
1.0861679403	0.6878299526	0.9999537652	1.1673314828
-1.0302490502	0.8312355715	0.9999537652	1.146732109
-1.0155812503	0.9531816976	0.9999537652	-1.1569322354
1.2601694192	0.7476173472	0.9999537652	2.3697912034
1.1684700032	0.5149452709	0.9999537652	-1.3326501422
1.1063683457	0.4990587707	0.9999537652	1.4798701681

2.0853648548	0.0823910824	0.9999537652	1.1795011917
-1.0670702872	0.909462178	0.9999537652	1.0057694371
1.2741030728	0.8384358611	0.9999537652	-3.3853891979
1.0189640423	0.8839145995	0.9999537652	1.124765166
-1.0282707113	0.8368056055	0.9999537652	1.1617344316
-1.0025725449	0.9888566129	0.9999537652	1.1697201952
1.119365539	0.8084500976	0.9999537652	2.0676325407
-1.0406519715	0.8174761295	0.9999537652	1.0397483482
1.0812231057	0.9007032907	0.9999537652	2.9644643681
1.6179314394	0.4722136221	0.9999537652	-1.4439334837
2.7933974408	0.6948713406	0.9999537652	0
1.9066445334	0.474201645	0.9999537652	-1.1626441795
-1.133504523	0.5795744495	0.9999537652	1.1355210512
-1.2584520901	0.2543569632	0.9999537652	-1.0946926513
1.4451058543	0.6003003127	0.9999537652	1.3716738948
-1.3204799705	0.5579101894	0.9999537652	-1.1081534871
1.8049120379	0.0503598636	0.9999537652	1.1008357584
-1.257961459	0.1243358169	0.9999537652	1.0691522129
4.5225226171	0.1563080352	0.9999537652	-1.6798913749
-1.0087761577	0.9636760491	0.9999537652	-1.0729288431
-1.1457555741	0.910637459	0.9999537652	1.9418822056
6.3075231393	0.4852984374	0.9999537652	4.9527501949
7.6803712917	0.054587146	0.9999537652	2.9959081515
4.5545995984	0.5696641564	0.9999537652	-1.4636323788
1.04961577	0.7749579842	0.9999537652	1.1791889236
1.2501272441	0.2435927604	0.9999537652	1.0457081085
-1.2783158092	0.0567139491	0.9999537652	-1.0239170922
-1.191441802	0.6261704539	0.9999537652	-1.0781736232
1.0907959547	0.5153641616	0.9999537652	-1.0205163277
1.4717207227	0.3853329745	0.9999537652	-1.1419570229
-1.739818287	0.1178543278	0.9999537652	1.029751722
1.00113172	0.9940597631	0.9999537652	1.4186025385
-1.085804469	0.5575951198	0.9999537652	1.0683421565
1.0190376465	0.8805015969	0.9999537652	1.0282617854
-1.3773314892	0.0903210022	0.9999537652	1.052258108
2.2149242497	0.2789824949	0.9999537652	1.3889218671
1.0737726678	0.743192988	0.9999537652	1.2145355146
0	0	0	4.9561842702
-1.0556202517	0.8726793543	0.9999537652	-1.2512643744
-1.2369993804	0.2999961627	0.9999537652	1.1579741585
1.1518919277	0.4709444805	0.9999537652	1.4374095615
1.0371154324	0.8027753852	0.9999537652	1.2393289546
-1.0346176087	0.8514213405	0.9999537652	1.127784666
1.1412238519	0.5729779811	0.9999537652	-1.1054368451
0	0	0	0
-1.0509333268	0.8082713418	0.9999537652	1.0629854896
-1.189834497	0.5590894187	0.9999537652	-1.0234766648
1.0142015787	0.9502130125	0.9999537652	1.0679898826
1.4001944622	0.6032346081	0.9999537652	-1.1826872882

1.0952324259	0.509838899	0.9999537652	1.2645066367
1.0368295037	0.8109061463	0.9999537652	1.1798228814
1.037193117	0.8457949755	0.9999537652	1.036693899
0	0	0	3.8462080177
-1.2370238115	0.3063886647	0.9999537652	-1.3644004332
1.001916372	0.9876381379	0.9999537652	1.2504847939
-1.1858870587	0.4590701844	0.9999537652	-1.0709649066
-1.1790190163	0.3849271386	0.9999537652	-1.200390781
1.2607522837	0.2247859503	0.9999537652	-1.0484652732
0	0	0	0
1.5101610281	0.0817550697	0.9999537652	1.223564641
1.1558590933	0.4533574964	0.9999537652	-1.5430149474
1.4609539587	0.2305983618	0.9999537652	1.4419663777
-1.2808770493	0.0779807784	0.9999537652	-1.0889296854
-1.0205125782	0.9221898151	0.9999537652	-1.2142507046
2.7933974408	0.6948713406	0.9999537652	0
-1.4014416871	0.6352188282	0.9999537652	-1.2525304726
1.1649166714	0.4022488339	0.9999537652	1.2937604524
1.1123661974	0.5142449221	0.9999537652	1.2400663375
1.0107914197	0.9696120089	0.9999537652	1.1526272851
9.8205505243	0.3811471738	0.9999537652	0
-2.1299793114	0.480283682	0.9999537652	-1.1511079519
1.0429903551	0.7895968919	0.9999537652	1.0576421303
-1.0182908362	0.9015594701	0.9999537652	1.1231024891
-1.0601416404	0.7813744265	0.9999537652	-1.1228478116
1.2921049422	0.486219074	0.9999537652	1.2263795356
-1.2599085795	0.2888474391	0.9999537652	-1.0155895798
-1.1581909615	0.3343729654	0.9999537652	1.118292821
6.3075231393	0.4852888938	0.9999537652	-1.4300917892
1.1839827621	0.3239695423	0.9999537652	1.0502090721
1.0301143907	0.8696522723	0.9999537652	1.2372278502
-1.3419168111	0.4064397771	0.9999537652	1.3665428263
-1.1514009361	0.4170346049	0.9999537652	1.4287525201
1.0408060511	0.7595131532	0.9999537652	1.1617337459
-1.0358883782	0.9062269986	0.9999537652	1.5700225911
-1.3204010827	0.0492185365	0.9999537652	1.1096429173
1.2254893424	0.5201549692	0.9999537652	1.2693924856
1.1869733253	0.4714744076	0.9999537652	-1.2172887378
-1.3668636763	0.0284117852	0.9999537652	-1.5135978667
1.5862555348	0.3505256261	0.9999537652	-1.2628336924
1.1805131425	0.2398295273	0.9999537652	1.3628917046
-1.1422124923	0.4747343514	0.9999537652	-1.3797540929
2.608128111	0.1517316786	0.9999537652	-1.007834096
-1.2351432639	0.2033373895	0.9999537652	-1.0091761166
-1.0170434563	0.9341478144	0.9999537652	1.2045720219
1.0788786704	0.6935874055	0.9999537652	1.1921238009
1.0183109303	0.9033082635	0.9999537652	1.1357876156
1.392975024	0.6538178206	0.9999537652	1.0399499187
-2.462078324	0.1593858769	0.9999537652	-1.3326161485

-1.0758793808	0.6117621954	0.9999537652	1.0219298287
4.5505976667	0.5557790267	0.9999537652	8.8639046058
1.1162834585	0.9273004279	0.9999537652	1.4067915718
-1.496681452	0.3663965517	0.9999537652	1.0441887571
-1.9991287888	0.4535404311	0.9999537652	2.4613556948
-1.0773189521	0.6249833341	0.9999537652	1.196120886
1.0902237227	0.4593817391	0.9999537652	-1.0280400571
1.0536087347	0.7226105676	0.9999537652	-1.1948776026
1.0425056794	0.8447769683	0.9999537652	-1.1741182728
2.7933974408	0.6948713406	0.9999537652	-3.89071692
4.5505976667	0.5557790267	0.9999537652	2.9926666006
15.0880352532	0.2938366616	0.9999537652	1.5358840667
-1.2792892569	0.1084352128	0.9999537652	1.224686176
-1.0046076372	0.9854240271	0.9999537652	-1.0486446503
-4.3180711894	0.1805808777	0.9999537652	1.1717762073
0	0	0	2.996475627
1.0936437502	0.4311215515	0.9999537652	1.0182812038
1.0644387145	0.8010036853	0.9999537652	-1.0444671123
-1.17745997	0.7906672588	0.9999537652	-1.0844141669
-1.0800754572	0.6280606658	0.9999537652	-1.2136942245
-1.0027327021	0.9863721284	0.9999537652	-1.1411363747
2.7933974408	0.6948713406	0.9999537652	28.4311330788
-1.0384172509	0.9041286727	0.9999537652	1.1034777787
-1.0754234039	0.6682237042	0.9999537652	1.1513396866
-1.2176930367	0.535300904	0.9999537652	-1.3069515244
4.5505976667	0.5557790267	0.9999537652	-2.0482026754
3.0669619516	0.3204959524	0.9999537652	1.2765689895
1.1033812008	0.4762483444	0.9999537652	1.4641257906
1.2464313046	0.6241137858	0.9999537652	1.003137417
-1.0238030823	0.9488510949	0.9999537652	-1.1881433652
-1.2481677054	0.2785206721	0.9999537652	-1.0750450585
4.5505976667	0.5557790267	0.9999537652	-2.0482026754
1.7776560069	0.5215716221	0.9999537652	-2.2414820712
1.4808270106	0.6341451449	0.9999537652	2.9940379764
1.279335829	0.5184088515	0.9999537652	1.2831155513
1.3170135462	0.3959600609	0.9999537652	-1.2399123282
1.4439128375	0.3433176638	0.9999537652	1.0461823352
1.3408411056	0.3366347447	0.9999537652	1.0898533637
1.4414670849	0.1240803287	0.9999537652	-1.2071277023
1.2053536902	0.3079633689	0.9999537652	1.0848956048
1.1849377253	0.377771704	0.9999537652	-1.1321345132
0	0	0	12.7732027325
1.080827404	0.6106914443	0.9999537652	-1.6158166159
-1.0650190283	0.6874634849	0.9999537652	-1.436698616
1.0724482025	0.7828197758	0.9999537652	-1.1726098556
1.4477315328	0.1115638052	0.9999537652	-1.2524137015
1.0564719306	0.6431833498	0.9999537652	1.2904210235
1.1496313829	0.5150173415	0.9999537652	-1.2919083016
-1.2331909292	0.5002820743	0.9999537652	-1.4260185799

-1.0065576531	0.9840958227	0.9999537652	-1.2749165084
-1.3143326454	0.1587908787	0.9999537652	-1.1102358527
0	0	0	3.6950602847
1.1208663544	0.5363983716	0.9999537652	1.0895719508
1.1919115821	0.8106792912	0.9999537652	-1.4212516787
-1.620580577	0.2071852641	0.9999537652	-1.2274746622
-1.4768937254	0.0363728585	0.9999537652	-1.2431811024
-1.1283833943	0.5320557533	0.9999537652	-1.1484194329
1.1355024823	0.4638317706	0.9999537652	1.0174361434
-1.1653900061	0.2019984449	0.9999537652	-1.2240867834
-1.9573783405	0.3068429304	0.9999537652	-1.0194881681
-1.176033766	0.3708136688	0.9999537652	-1.012661497
-1.2861044151	0.1512273023	0.9999537652	1.3201863476
-1.5788392377	0.2074983811	0.9999537652	1.0249738366
-1.1021300171	0.889345452	0.9999537652	-1.0552131742
1.1221358527	0.3626763492	0.9999537652	1.133132161
-3.2687501303	0.1999864487	0.9999537652	2.4267255252
1.2144087327	0.4392190551	0.9999537652	-1.0030325481
1.1514307313	0.3560100412	0.9999537652	1.6160368437
-1.4475713225	0.0801206975	0.9999537652	-1.7002491637
1.070695326	0.5858369091	0.9999537652	1.2493779425
-1.1885556815	0.4444202248	0.9999537652	1.0426272218
-1.1142715925	0.6083026473	0.9999537652	-1.0603883324
0	0	0	2.2924151284
1.0476021643	0.8011595106	0.9999537652	-1.2117215963
1.9930986705	0.172670644	0.9999537652	-1.4621109275
-1.1114526659	0.5716308958	0.9999537652	-1.0033620637
2.0921011564	0.5214604309	0.9999537652	-1.201159017
-1.420925217	0.3569845371	0.9999537652	1.0836096588
1.061466329	0.6665607231	0.9999537652	1.0950060094
1.0274746218	0.851801736	0.9999537652	-1.0421745334
11.5868260078	0.3462116853	0.9999537652	1.444719916
1.1734634941	0.199484792	0.9999537652	1.2479336915
1.5010626463	0.0959575148	0.9999537652	-1.4261946974
-1.0604045157	0.9242752064	0.9999537652	1.491272219
0	0	0	1.4852334646
-2.390262974	0.1348411085	0.9999537652	-1.1641740884
-1.0696248962	0.5777242672	0.9999537652	-1.338743615
1.276455514	0.2192436583	0.9999537652	-1.1022170303
-2.9543499299	0.1230730753	0.9999537652	-1.0958923887
2.7933974408	0.6948713406	0.9999537652	4.9527501949
1.2480242908	0.0933434972	0.9999537652	-1.0646658001
-1.5516840448	0.3133003026	0.9999537652	-1.185643086
1.1395128077	0.3854036518	0.9999537652	1.3974587146
163.3929613609	0.0594282054	0.9999537652	-1.6566762511
1.0032252867	0.9874475114	0.9999537652	-1.0445372312
1.0072200856	0.9531689866	0.9999537652	1.1109014696
1.0106558672	0.9413529666	0.9999537652	1.1593674353
-1.0207830004	0.8786705405	0.9999537652	-1.1194294931

2.3315363418	0.4563813059	0.9999537652	-4.7721736557
-1.2738098317	0.0921966616	0.9999537652	-1.1403928992
1.3187056891	0.6037270261	0.9999537652	1.2658484952
-1.0271529872	0.8322865268	0.9999537652	-1.045640536
-1.3209007499	0.0634732078	0.9999537652	-1.1761276231
0	0	0	-1.1441751338
1.0668323481	0.7310445531	0.9999537652	-1.0450284483
1.7949800932	0.281175186	0.9999537652	1.1851182321
1.5088458739	0.5301360532	0.9999537652	1.078186748
1.0034913121	0.9950884065	0.9999537652	1.1053359433
-1.0702652931	0.785381122	0.9999537652	1.0802537033
0	0	0	1.5330591177
4.5544554512	0.5691274043	0.9999537652	18.6336779883
2.2288449002	0.1532415988	0.9999537652	-1.3046729605
1.0766432389	0.5198885541	0.9999537652	1.1496877388
-1.1453192235	0.908573838	0.9999537652	4.0953564288
-1.0113497253	0.9679593465	0.9999537652	1.3930303767
1.281913786	0.4072643493	0.9999537652	-1.13545812
1.2267996097	0.6446976357	0.9999537652	-1.0969319908
-1.3659192566	0.1132507936	0.9999537652	1.2490883855
-1.0148871416	0.9503655321	0.9999537652	-1.0249961351
-2.0778932133	0.0167958493	0.9999537652	1.6479171331
1.2225112655	0.482732512	0.9999537652	1.3116947587
1.7976377322	0.2367499927	0.9999537652	1.0438412248
0	0	0	1.2398837679
0	0	0	2.030756821
0	0	0	3.346511156
-2.5423404596	0.2378375033	0.9999537652	1.2515306702
0	0	0	2.1335943382
-1.1712303027	0.8330358523	0.9999537652	-1.8405281286
-1.0961218072	0.806364673	0.9999537652	1.0481594651
1.1027768967	0.7076901306	0.9999537652	1.0729973941
1.2234272733	0.2613933662	0.9999537652	-1.0205609736
-1.1454518545	0.345376664	0.9999537652	-1.0699973383
-1.1993148188	0.3097456646	0.9999537652	-1.6846525605
-1.5875118025	0.7201667036	0.9999537652	-1.8579915692
1.4913242644	0.328940737	0.9999537652	-1.2063557801
4.5505976667	0.5557790267	0.9999537652	22.5383525416
1.0930960888	0.6154668543	0.9999537652	-1.0831407003
-1.2982700168	0.3959742743	0.9999537652	1.1499777115
1.0065992309	0.977603212	0.9999537652	-1.1276577668
1.122276038	0.5473335858	0.9999537652	1.1274089389
-1.301951197	0.1649974471	0.9999537652	-1.1725946021
1.7352723816	0.4566627418	0.9999537652	-1.6653783823
1.238780517	0.1345003182	0.9999537652	-1.4539417389
3.978377481	0.04964647	0.9999537652	1.0479736847
0	0	0	12.7732027325
0	0	0	-3.3849925178
1.3362904775	0.3985053506	0.9999537652	-1.4404670446

-1.0552675813	0.7356897401	0.9999537652	1.332986139
-1.0881540529	0.7657828591	0.9999537652	-1.2394469978
1.0146182305	0.9282096046	0.9999537652	-1.0667450714
-1.2388965854	0.4753773976	0.9999537652	-1.303725964
-3.0628606717	0.0688520139	0.9999537652	3.3549728814
0	0	0	-1.4680135518
-2.5861438062	0.4715339839	0.9999537652	-1.1533929684
4.5505976667	0.5557790267	0.9999537652	1.3742147816
4.5505976667	0.5557790267	0.9999537652	-1.8882538771
-1.3668368923	0.752155156	0.9999537652	1.4660688402
1.0023346655	0.9871842868	0.9999537652	-1.1616033336
-1.1153765637	0.7406784552	0.9999537652	-1.2877952779
86.7434081177	0.1021564713	0.9999537652	46.2964317397
-1.1206475287	0.660887213	0.9999537652	-1.3867524638
-1.011714451	0.9298183269	0.9999537652	-1.1878966615
1.403141125	0.4756625939	0.9999537652	-1.26159545
1.1793849535	0.2605442279	0.9999537652	-1.0008406411
9.8205505243	0.381195962	0.9999537652	-1.7821643145
1.1709835065	0.3131703811	0.9999537652	1.088898973
6.3128691894	0.4811788216	0.9999537652	1.0017424929
-1.0197828113	0.8984853793	0.9999537652	-1.089488292
-1.2121682788	0.1127446768	0.9999537652	1.0972380172
-1.0975717723	0.6653579502	0.9999537652	-1.1322962376
1.3102067608	0.126632553	0.9999537652	-1.170805725
1.1595709269	0.5868398847	0.9999537652	-1.5254591311
1.3430439783	0.2080054387	0.9999537652	1.2716612353
1.2001615273	0.3722857192	0.9999537652	-1.1508310948
1.3935918612	0.1376433155	0.9999537652	-1.2621074912
1.0002829396	0.9989758165	0.9999537652	-1.0845502872
1.0642219571	0.6689493875	0.9999537652	-1.2619727673
-1.1421477292	0.7764694598	0.9999537652	-1.5319065574
-1.1811567651	0.5738484929	0.9999537652	-1.2700203908
-1.029366635	0.8714707725	0.9999537652	-1.2851424412
0	0	0	15.7262184124
-1.5784038767	0.5480897108	0.9999537652	-1.2549309907
1.1623934731	0.4806897707	0.9999537652	-1.1356712697
6.3075231393	0.4852793576	0.9999537652	1.2652798573
-1.0622467421	0.9027296244	0.9999537652	1.0250681692
1.5418133296	0.1461429737	0.9999537652	1.2316690629
1.0382351538	0.8424447844	0.9999537652	1.0957510336
1.2766807249	0.4771435226	0.9999537652	1.4675935119
-1.179223437	0.5506992706	0.9999537652	-1.0665617039
1.3959912071	0.2818483116	0.9999537652	-1.2810036924
-1.1441984695	0.7215879425	0.9999537652	1.334661317
11.5970171551	0.3502565327	0.9999537652	-4.9942401165
-1.467251024	0.3090767033	0.9999537652	1.4427656689
-1.1144290208	0.7296363678	0.9999537652	-1.0045981937
-1.0089195645	0.9925492641	0.9999537652	-1.1562874686
1.1416604346	0.7698209476	0.9999537652	-1.0699489612

-1.063259948	0.664569217	0.9999537652	-1.0124206043
1.0310504732	0.8519609457	0.9999537652	-1.2795683456
-1.2962696481	0.0721442438	0.9999537652	-1.1310783008
1.0746051196	0.6996006238	0.9999537652	1.1890296401
-1.0366685029	0.8987350354	0.9999537652	1.07289715
-1.1238061404	0.8691664385	0.9999537652	-1.1839629195
-1.1146022967	0.4579055839	0.9999537652	-1.2098771003
-1.0966013797	0.5917026805	0.9999537652	-1.4176928308
1.1162834585	0.9273200307	0.9999537652	-1.3582894632
1.0839946656	0.5978934935	0.9999537652	-1.0238367717
-1.1996097823	0.5603170819	0.9999537652	-1.1968632117
-1.115339507	0.5721341751	0.9999537652	-1.3550695459
-1.0432557936	0.9172425976	0.9999537652	-1.4211405633
0	0	0	1.590997316
0	0	0	2.996475627
-1.0089918016	0.9630493776	0.9999537652	-1.2844973678
-1.0307006845	0.8912048702	0.9999537652	1.085702139
-1.0153746423	0.9541483597	0.9999537652	-1.0538386981
1.3646178857	0.0896101281	0.9999537652	-1.0334460543
1.7025203179	0.0553956047	0.9999537652	-1.2266672543
-1.3358438138	0.4174168903	0.9999537652	-1.5249679375
1.2146174525	0.4825614304	0.9999537652	-1.2881620534
-2.1317846121	0.4677383323	0.9999537652	1.3513892501
-1.5939280118	0.30764496	0.9999537652	-1.2396413123
1.0847631898	0.7578361709	0.9999537652	-1.4871304102
-1.5875118025	0.7201623664	0.9999537652	-2.6399253151
-1.060373497	0.7464753048	0.9999537652	1.0328223823
1.2408466135	0.3265428068	0.9999537652	-1.0882611118
1.4313016951	0.0779835604	0.9999537652	-1.6246443134
1.0190507135	0.9188778209	0.9999537652	-1.2937368251
-1.524960239	0.5499517931	0.9999537652	-1.5037428056
1.1629956299	0.762629821	0.9999537652	1.1886566977
1.0980661343	0.8282987727	0.9999537652	-2.8371299626
3.2200264641	0.0825417824	0.9999537652	1.3384228545
1.6506720326	0.2190550538	0.9999537652	1.5532508978
2.2720949076	0.0905094517	0.9999537652	1.0704248589
-1.108086402	0.7401774572	0.9999537652	1.2180576921
0	0	0	2.9985540909
1.4350458055	0.2349488621	0.9999537652	1.3094226511
-1.0605920868	0.8801062604	0.9999537652	-1.493436693
1.0267233535	0.9172140989	0.9999537652	1.0023670513
1.1085999121	0.6955649555	0.9999537652	-1.4896479148
13.3324809638	0.3193679464	0.9999537652	-2.1341400956
-2.3043460049	0.0681501233	0.9999537652	1.1518828513
8.064174035	0.4213973314	0.9999537652	-1.5377740926
2.7933974408	0.6948713406	0.9999537652	0
0	0	0	0
1.3011715182	0.0422767619	0.9999537652	1.0299919617
-1.3009825858	0.0538006183	0.9999537652	-1.1529378602

-1.0224729808	0.8835265774	0.9999537652	-1.0818694527
-1.0651939987	0.7288641112	0.9999537652	-1.0231595854
1.1332589726	0.7367023341	0.9999537652	1.3258994869
-1.3256856079	0.2914585742	0.9999537652	-1.0034265665
1.6725583551	0.478550357	0.9999537652	1.4443863998
1.1743806988	0.3674200402	0.9999537652	-1.4191417118
-1.4217598579	0.1864941783	0.9999537652	-1.4416838718
-2.746603376	0.022292897	0.9999537652	-1.4956854776
2.7933974408	0.6948713406	0.9999537652	-1.3607721321
-1.2085881694	0.5110035563	0.9999537652	1.0629368176
-1.0782708489	0.6312734216	0.9999537652	-1.1147629222
-1.1254842338	0.6972783045	0.9999537652	1.0265146123
-1.2061735959	0.8458929999	0.9999537652	-1.5210032613
-1.0077148126	0.9569884028	0.9999537652	1.0207724382
0	0	0	-1.859280336
1.455540275	0.1369661356	0.9999537652	-1.2306475908
1.1991419323	0.3354179737	0.9999537652	-1.0758646414
1.0149585581	0.9597758053	0.9999537652	-1.4626267296
-1.0996816679	0.7125321622	0.9999537652	1.1008365997
0	0	0	10.818785458
0	0	0	2.6434024479
1.0323451371	0.8160553632	0.9999537652	-1.013676705
0	0	0	2.996475627
1.1023938338	0.518033941	0.9999537652	1.0098568406
1.01941503	0.8636952273	0.9999537652	1.0101975904
-1.2178409995	0.6365481041	0.9999537652	-1.3381435203
-7.0403382421	0.1005520082	0.9999537652	2.996475627
1.04021894	0.9359842482	0.9999537652	1.2393612499
-1.3920087874	0.0118500244	0.9999537652	-1.2220602595
2.7933974408	0.6948713406	0.9999537652	2.996475627
1.0609526506	0.8048598116	0.9999537652	1.005993897
1.1310210172	0.7846483202	0.9999537652	-1.1639181098
-1.2665504101	0.4992310607	0.9999537652	-1.3128330878
-1.3398763835	0.3523885417	0.9999537652	-1.1217575351
-1.4406913797	0.1216484179	0.9999537652	-1.2024502442
2.216996837	0.1055252038	0.9999537652	-2.2858367088
1.1043648176	0.5043500236	0.9999537652	1.1880243167
-1.1495672	0.5834173794	0.9999537652	-1.3278807112
1.3588576256	0.8014139255	0.9999537652	-7.9636855069
1.3334492853	0.1174231658	0.9999537652	-1.0236343959
-1.3033801392	0.0744993417	0.9999537652	-1.1974406179
-1.5914616654	0.1349086511	0.9999537652	-1.6692265356
-1.0688445283	0.7450211495	0.9999537652	-1.3609191796
-1.0549588096	0.8562923255	0.9999537652	-1.0024446427
-1.182724755	0.4392620349	0.9999537652	1.4362682927
1.0028190338	0.9820715817	0.9999537652	-1.2998889922
1.7913882976	0.0580516687	0.9999537652	-1.0557446667
1.7114119638	0.0725190321	0.9999537652	1.1241622155
-1.1936903692	0.4614611172	0.9999537652	-1.0261834446

-1.1816673994	0.46975339	0.9999537652	1.2114753243
1.6018473574	0.6946381432	0.9999537652	-1.7691190357
-1.2906676756	0.1177595692	0.9999537652	-1.4193791171
1.0648765994	0.6592635019	0.9999537652	1.030012374
6.3075231393	0.4852698288	0.9999537652	-3.384595918
6.3128691894	0.4810839179	0.9999537652	1.9991325461
0	0	0	2.996475627
-1.229535285	0.7400584302	0.9999537652	-1.4851550121
2.0880127507	0.0992959266	0.9999537652	-1.3207364426
-1.2434792443	0.51523504	0.9999537652	-1.0356316362
-1.1113919897	0.5523724823	0.9999537652	-1.1832784401
1.6374475495	0.5960750779	0.9999537652	-3.1881123249
-1.5881570201	0.7075125057	0.9999537652	-1.4727509169
1.0604339708	0.8279524541	0.9999537652	-1.1925380155
1.1642522676	0.6217144243	0.9999537652	-1.0268092547
3.7338262809	0.117354566	0.9999537652	4.7163908587
4.5505976667	0.5557790267	0.9999537652	4.9561842702
1.0622483758	0.652141234	0.9999537652	1.0441035748
1.1719648111	0.243966056	0.9999537652	1.1209781731
1.8366741697	0.2506314126	0.9999537652	1.4128084833
1.2516202231	0.8303779394	0.9999537652	-1.4292703376
-1.185242554	0.6483593386	0.9999537652	1.1080863171
11.5766527773	0.3453712281	0.9999537652	1.1331826089
1.1029209479	0.813679922	0.9999537652	-1.1293435299
2.7602715418	0.0419577259	0.9999537652	-1.4934746286
-1.0065650977	0.9831861734	0.9999537652	1.1577816025
1.5807620463	0.292709029	0.9999537652	1.2303360356
-1.317283144	0.5030840096	0.9999537652	-1.0107054234
2.1474619702	0.2589394284	0.9999537652	1.139398705
-1.1887732262	0.5053289167	0.9999537652	-1.3283731709
1.0382517226	0.9389806192	0.9999537652	1.1235729759
1.3273898611	0.2133726424	0.9999537652	-1.1090925543
-1.3509716065	0.5573946263	0.9999537652	1.1409731457
1.1682520285	0.3080899486	0.9999537652	-1.1663582912
-1.1749245553	0.749565706	0.9999537652	-1.7095238164
-1.4768386434	0.045964824	0.9999537652	1.0816888936
-1.674537298	0.1547306803	0.9999537652	-1.2992982656
-1.1547091666	0.5900948974	0.9999537652	1.7334646473
1.2312715047	0.6447041921	0.9999537652	1.061274526
-1.0026462193	0.9911034792	0.9999537652	-1.3983026337
120.6208104207	0.0659056338	0.9999537652	-7.9984306033
-1.7153371014	0.0345016687	0.9999537652	1.3746297624
-1.0814913541	0.6531431544	0.9999537652	1.0398887409
1.0669322604	0.8122686818	0.9999537652	1.334517701
-1.1920634676	0.3479499331	0.9999537652	-1.2695498817
-1.5850657366	0.167170501	0.9999537652	-1.0991019002
1.2011626653	0.4592169276	0.9999537652	1.6390895238
1.0105955832	0.9775822554	0.9999537652	-1.0391495954
1.3356053654	0.3430918386	0.9999537652	1.2299044262

-1.216643898	0.2117024219	0.9999537652	-1.2373214694
2.3799893397	0.1267530901	0.9999537652	1.2203978783
2.0225469564	0.1497012721	0.9999537652	-1.199039625
18.5983228161	0.2588461094	0.9999537652	6.9133480437
2.7933974408	0.6948713406	0.9999537652	0
-1.2537514618	0.3674217225	0.9999537652	-1.0749180509
-1.3149356864	0.1384091604	0.9999537652	1.6025092553
1.0732956812	0.6241685609	0.9999537652	-1.1340129937
2.7933974408	0.6948713406	0.9999537652	4.9527501949
1.1416508574	0.6535294448	0.9999537652	1.0809739446
-6.9762758667	0.2573781562	0.9999537652	8.8639046058
41.9731433602	0.1493503109	0.9999537652	1.2196199025
-1.2507415237	0.3257180448	0.9999537652	-1.3449449566
0	0	0	4.9527501949
1.6666137657	0.3753116338	0.9999537652	-1.2266184861
-1.2645490621	0.6880962475	0.9999537652	-1.3762789529
-1.0994028975	0.7496066711	0.9999537652	-1.0899302059
1.17556746	0.546263497	0.9999537652	-1.5591088157
1.5728474279	0.0238177211	0.9999537652	-1.1519498403
-1.0519973377	0.7314473492	0.9999537652	-1.1496597598
-1.1213192715	0.5474489997	0.9999537652	-1.0360642734
1.0043858916	0.9847648623	0.9999537652	1.0161826057
-1.1989647349	0.4332987735	0.9999537652	1.019045855
0	0	0	18.6723895103
-1.347364702	0.2159324122	0.9999537652	-1.1541963532
0	0	0	1.0376348451
1.7745951127	0.5408978315	0.9999537652	-1.4239685954
1.098835743	0.5479559891	0.9999537652	-1.1536379666
1.0374892852	0.8987215732	0.9999537652	1.3895637335
6.3075231393	0.4852603074	0.9999537652	-1.8692819149
1.8557422296	0.3218735126	0.9999537652	1.1059318362
-1.1299110903	0.5199862739	0.9999537652	1.064996907
-1.1476861652	0.77577207	0.9999537652	-1.8922915264
-1.0535876328	0.6865203557	0.9999537652	1.2054761848
1.0842927461	0.7273563016	0.9999537652	1.3672566925
1.2504025925	0.5370461157	0.9999537652	1.2152158153
1.4500051737	0.1038829653	0.9999537652	1.1818304912
1.2505200867	0.1011303288	0.9999537652	1.2699948056
1.065744814	0.6348848711	0.9999537652	1.0883030932
1.4267642224	0.4092481147	0.9999537652	1.0062187336
1.1669563645	0.5995411504	0.9999537652	1.102566814
-1.0108880557	0.9883912163	0.9999537652	1.110588605
1.6560364947	0.3027477056	0.9999537652	-1.4818633664
1.8920098155	0.1680897107	0.9999537652	1.4664814702
1.1172132586	0.6411663374	0.9999537652	-1.2152334541
1.0576665918	0.7819331153	0.9999537652	-1.0906933583
-1.1656372969	0.862770482	0.9999537652	1.7984806365
1.6168475804	0.0184634012	0.9999537652	1.1185341135
1.7291298651	0.3372900039	0.9999537652	-1.4802940734

1.0074940527	0.9577649925	0.9999537652	1.0798445923
1.6470167447	0.0641387979	0.9999537652	-1.222806565
-1.2341931486	0.1852023319	0.9999537652	1.3248324547
6.3075231393	0.4852507934	0.9999537652	-1.4206411224
1.6056477681	0.3928289718	0.9999537652	-1.3840469295
-1.4604704138	0.2849420459	0.9999537652	-1.0680493131
-1.0293826085	0.8747053315	0.9999537652	1.1078828286
-1.5699952994	0.4903036441	0.9999537652	-1.265426292
1.1016736637	0.5712695439	0.9999537652	1.1423039461
-1.944524113	0.0270179523	0.9999537652	1.1037273925
2.3729118478	0.3017562155	0.9999537652	5.8001127949
1.7846532622	0.3772603986	0.9999537652	2.1070894252
-1.4750076826	0.1067363279	0.9999537652	-1.2546737464
-2.4710767924	0.5445105149	0.9999537652	1.2915363004
-1.1625858476	0.2663644869	0.9999537652	-1.2232876672
2.8173722589	0.3577504083	0.9999537652	-7.4219176587
1.0999141952	0.3999280148	0.9999537652	1.1017037515
1.0373288779	0.8152836268	0.9999537652	1.6047165983
-1.0736334663	0.7094059054	0.9999537652	1.0195324497
-1.2155863658	0.6382431193	0.9999537652	2.115793706
2.1077739145	0.2653502674	0.9999537652	-1.3953149115
8.064174035	0.4204298929	0.9999537652	-1.1427246333
-1.6288558071	0.4355686581	0.9999537652	2.7242954019
1.2225946172	0.2087631903	0.9999537652	1.0024361165
1.1219535594	0.6819914903	0.9999537652	-1.0149234793
1.0446300955	0.8579968748	0.9999537652	-1.3072752469
-1.0466090104	0.8079593721	0.9999537652	1.152710562
-1.2700754765	0.7499062125	0.9999537652	-2.1715390799
-4.1037783439	0.1457766854	0.9999537652	1.5128265653
0	0	0	2.996475627
0	0	0	4.9527501949
-1.083525836	0.7322417554	0.9999537652	1.1793779674
2.7957660672	0.7054998373	0.9999537652	-1.9814915788
1.086270844	0.7919651292	0.9999537652	1.3490826275
2.0265793637	0.0748231802	0.9999537652	-1.296615928
-2.3726635459	0.054564454	0.9999537652	1.0772757219
-1.2203912745	0.5227693207	0.9999537652	1.3391630313
1.7398053282	0.2210854143	0.9999537652	1.1750198675
2.7933974408	0.6948713406	0.9999537652	0
144.262103935	0.0663267197	0.9999537652	-1.7465907299
-1.1081430555	0.5067063754	0.9999537652	1.2815258762
6.0689445799	0.0357017211	0.9999537652	-1.8676422841
1.087219878	0.5420142803	0.9999537652	1.0210939364
0	0	0	16.6806485101
-1.3230608984	0.4066449336	0.9999537652	-1.3644127141
2.7933974408	0.6948713406	0.9999537652	3.7005385664
0	0	0	2.996475627
-1.0480960432	0.8302340345	0.9999537652	-1.1183978181
1.0372981855	0.8717814736	0.9999537652	-1.26429544

-1.0967589153	0.705261475	0.9999537652	1.1564774017
-1.9445493603	0.1943987684	0.9999537652	4.0965603855
1.6024984014	0.6862231608	0.9999537652	3.6551704795
1.2050101121	0.5495819501	0.9999537652	1.1446861974
1.1592231455	0.6330590559	0.9999537652	-1.1442222517
1.1145776833	0.8149442952	0.9999537652	1.0533536468
-1.0834038168	0.701873703	0.9999537652	1.005989477
-1.1201924633	0.7219318388	0.9999537652	-1.5326892303
1.3129587976	0.2996248014	0.9999537652	-1.0791404304
1.0205020034	0.8851024489	0.9999537652	1.1858288221
1.0170015816	0.9679978787	0.9999537652	1.2503965669
-1.1164535624	0.6135616211	0.9999537652	-1.7039376089
1.1604970644	0.4267951748	0.9999537652	-1.2288978001
1.0216567631	0.8717761847	0.9999537652	-1.0117599554
1.2168974893	0.1554291357	0.9999537652	1.0053116326
-1.1452962001	0.5084154124	0.9999537652	-1.2313167565
-2.955963562	0.318889966	0.9999537652	2.1230226627
-1.033080645	0.9154758944	0.9999537652	-1.2507143103
1.430204995	0.2099873154	0.9999537652	-1.0357021453
-1.4741942417	0.5298175828	0.9999537652	1.1899349411
-4.8154268017	0.171205961	0.9999537652	8.8639046058
1.2910574909	0.7068915691	0.9999537652	-1.3513401684
1.0135240894	0.9417247475	0.9999537652	-1.1924526816
1.3145865926	0.1456307322	0.9999537652	-1.2442361461
1.7286784756	0.4071607084	0.9999537652	-1.1025258849
-1.7180507824	0.0863407843	0.9999537652	-1.0731035797
1.1833138015	0.8256586683	0.9999537652	-1.6576187027
8.064174035	0.4192990295	0.9999537652	-1.9821566108
-1.0677326935	0.7451562835	0.9999537652	-1.1364621289
2.2592667537	0.1577237699	0.9999537652	-1.4377981263
-18.9844959609	0.0576182292	0.9999537652	-2.6856242212
-1.6863530946	0.0148886783	0.9999537652	-1.2503763775
-1.0606604912	0.7818516508	0.9999537652	-1.1775397349
1.3577059233	0.2512336728	0.9999537652	1.351884749
1.8455489546	0.5920717501	0.9999537652	-9.7565542884
-6.9762758667	0.2573366588	0.9999537652	1.2439599102
-1.44703975	0.3739847266	0.9999537652	-1.1001301261
8.064174035	0.4193050961	0.9999537652	1.5185018078
1.3487618019	0.0373581617	0.9999537652	1.1575893131
0	0	0	14.7271569203
2.1416353779	0.0676610987	0.9999537652	-1.0637216495
15.2790678492	0.007011	0.9999537652	-1.3415412517
1.1176600764	0.6752130527	0.9999537652	-1.0750180715
1.2329089224	0.1099236272	0.9999537652	-1.1853444352
-1.0553653563	0.8151604788	0.9999537652	-1.2473979577
-1.1163043415	0.8073292412	0.9999537652	1.6199771783
2.3981407703	0.0306761543	0.9999537652	1.000000614
1.0471237689	0.9400144864	0.9999537652	-1.283870632
-1.2551604504	0.2263184232	0.9999537652	1.0216483988

1.3297177015	0.430919211	0.9999537652	-1.1976936839
1.4549753547	0.122361653	0.9999537652	-1.3767581111
3.1966312975	0.0550392658	0.9999537652	1.1103578518
-1.2549682299	0.7046813256	0.9999537652	-1.6681632674
1.1361581687	0.4747101408	0.9999537652	1.9067922888
1.2967522927	0.2381812178	0.9999537652	1.0055563268
1.1681724717	0.5980109893	0.9999537652	-1.0032494006
-2.0933030754	0.4311045288	0.9999537652	2.5555207059
1.2923978463	0.0757224131	0.9999537652	-1.0625136382
-1.001352875	0.9939349192	0.9999537652	1.027873894
1.1376235531	0.7266645076	0.9999537652	-1.3775874542
-1.5098119052	0.0239249045	0.9999537652	-1.0564896624
-1.1808819591	0.2991964767	0.9999537652	-1.1639347019
-2.5839527681	0.5182093244	0.9999537652	-7.9600406939
-1.2772426758	0.2173335418	0.9999537652	1.0632855752
1.2904993165	0.7067137628	0.9999537652	-1.3344270248
-1.083958991	0.7150263865	0.9999537652	-1.3273707385
1.5186643114	0.126521503	0.9999537652	-1.0960387582
2.063730313	0.2434517488	0.9999537652	1.184649269
-1.3938591781	0.4112724619	0.9999537652	1.0791122804
-1.0311247665	0.8835681629	0.9999537652	-1.0682180894
1.0905362733	0.8214881044	0.9999537652	1.0523237315
1.3138925203	0.3987977175	0.9999537652	-1.1787733872
1.4562834313	0.4031274025	0.9999537652	1.1000534772
1.2520825157	0.3686714579	0.9999537652	-1.1998053226
9.8205505243	0.3812413623	0.9999537652	1.1896492559
1.2782066758	0.0579197463	0.9999537652	-1.1769873521
-1.2269614418	0.5097569439	0.9999537652	-1.2419776884
13.3324809638	0.3193592658	0.9999537652	-2.1478925545
2.1927369676	0.3100396102	0.9999537652	-1.4227651232
-1.4752476881	0.3249836121	0.9999537652	1.1338579197
1.2277912236	0.4827626121	0.9999537652	-1.0715795887
1.1181639381	0.3712795204	0.9999537652	1.0313640661
-1.1540709157	0.5083055084	0.9999537652	-1.1775945909
1.2278372524	0.4547177853	0.9999537652	-1.1823374258
1.3104819349	0.5977979388	0.9999537652	-1.6828571806
-3.0420574223	0.0200169586	0.9999537652	1.1628701098
1.1548620047	0.4365212256	0.9999537652	1.1659094826
-1.2578696146	0.4794039733	0.9999537652	-1.5848247986
-1.703673415	0.2500601166	0.9999537652	-2.016360435
2.5757525888	0.4024060953	0.9999537652	1.3226394782
-1.1697024363	0.5690672907	0.9999537652	-1.3625971749
-1.4112279528	0.452090802	0.9999537652	1.0491203044
-1.1028662803	0.5116989564	0.9999537652	1.0033204842
1.2078320701	0.2040808514	0.9999537652	1.3252215312
1.4119895969	0.1351538328	0.9999537652	-1.1087349309
1.234880122	0.3371095467	0.9999537652	-1.0235070363
1.1820042162	0.6881403305	0.9999537652	1.0949608177
1.2625201345	0.7166896632	0.9999537652	-1.2869062722

-1.5474820895	0.4986612739	0.9999537652	-1.3645986872
1.1883762882	0.7489324124	0.9999537652	1.3519574459
1.0634737655	0.8098414562	0.9999537652	-1.0468073545
-1.3662673861	0.4330921379	0.9999537652	-1.6884599034
-1.4797035191	0.1926787505	0.9999537652	-1.2304213428
1.9050337421	0.4949709275	0.9999537652	-1.0946813431
-1.7633461612	0.0828048363	0.9999537652	-1.6947161979
2.3275623066	0.1421970963	0.9999537652	1.1210043915
-3.0095759667	0.0133438297	0.9999537652	-1.0851531775
1.5853476499	0.2340964662	0.9999537652	-1.0713435126
1.3681812834	0.1597829151	0.9999537652	-1.0999016363
1.0216203554	0.9617310435	0.9999537652	-1.3544732753
1.1796703259	0.4674396698	0.9999537652	-1.161603061
2.7933974408	0.6948713406	0.9999537652	1.1360578751
-1.008016201	0.9679791712	0.9999537652	-1.0340513362
1.301673237	0.2878222209	0.9999537652	-1.2157198662
-1.0590929665	0.8395130035	0.9999537652	-1.0050191535
1.7357769655	0.4589461362	0.9999537652	-1.8319376845
1.0938187138	0.6619564189	0.9999537652	-1.3433466815
1.258269974	0.5405133883	0.9999537652	-1.2276245095
1.6164264405	0.2885611186	0.9999537652	-1.4107422155
16.8433158146	0.2737407497	0.9999537652	1.5887280788
1.131919818	0.5175381532	0.9999537652	1.00310556
-1.3646799224	0.247198007	0.9999537652	-1.2449300023
-1.0325602145	0.8861054924	0.9999537652	-1.023933547
20.3530564257	0.243041495	0.9999537652	-1.0585261291
-1.0230076016	0.8580295474	0.9999537652	-1.0469732088
-1.7700702233	0.006406	0.9999537652	-1.21365965
1.438522744	0.33551139	0.9999537652	1.6627054478
2.9980508928	0.0772277407	0.9999537652	-1.1386918785
6.3128691894	0.4809896669	0.9999537652	-1.4680135518
-1.2121435816	0.3236440173	0.9999537652	1.0323700801
-3.414177408	0.0821008145	0.9999537652	1.326842897
1.4869359056	0.192161553	0.9999537652	-1.5665347228
-2.3629710095	0.4005336112	0.9999537652	-1.1838291283
1.0435402836	0.9225519067	0.9999537652	1.4821423524
1.3007249239	0.4220272917	0.9999537652	1.5307690468
1.2207004539	0.3401405687	0.9999537652	1.1564218995
-1.0696482273	0.6590707059	0.9999537652	-1.0550947353
1.0825557215	0.6859748582	0.9999537652	1.0726541443
-1.3647999079	0.431033001	0.9999537652	1.2733598154
1.078475595	0.6424906223	0.9999537652	-1.1703720322
1.0773330201	0.7038892221	0.9999537652	1.3338616035
1.0762510692	0.7508030278	0.9999537652	1.0529602153
-1.2117665196	0.3085119919	0.9999537652	1.1541777298
1.1184146532	0.5963791382	0.9999537652	-1.0624029909
8.064174035	0.419311172	0.9999537652	-1.1393408151
-1.0157803324	0.9440684254	0.9999537652	-1.1242781646
-1.6076590535	0.0694281927	0.9999537652	1.0983259251

-1.0099323877	0.9704815666	0.9999537652	-1.3750266236
-1.1341438159	0.8230593928	0.9999537652	2.2750730938
1.2041125841	0.3233374273	0.9999537652	1.0878277235
-1.1421017849	0.5166545102	0.9999537652	-1.1481724641
-1.141788231	0.5457253625	0.9999537652	-1.2539258591
-1.0078760438	0.9608345575	0.9999537652	1.2337773377
1.7564675364	0.3066856983	0.9999537652	-1.6498312408
-1.0535827059	0.9227654106	0.9999537652	1.0539138314
-1.5115068889	0.3922956888	0.9999537652	-1.0553376913
-1.0577205428	0.8175032406	0.9999537652	-1.2855289153
-1.1731722061	0.5162539436	0.9999537652	-1.0714926283
-4.5414764708	0.0895972986	0.9999537652	-14.149476906
1.3705463739	0.3942624787	0.9999537652	1.3564311356
-1.0067597911	0.9859314309	0.9999537652	2.3110468829
1.2646652142	0.5932615042	0.9999537652	-1.4894136055
-1.0521480798	0.8160056237	0.9999537652	-1.0008534458
-1.1419238385	0.5555155639	0.9999537652	-1.1499512687
1.0262167194	0.8831107799	0.9999537652	1.2752038196
-1.5935758472	0.2820162251	0.9999537652	1.8169692298
1.2044284456	0.6620473463	0.9999537652	1.027221941
-1.0351324722	0.859210317	0.9999537652	1.112950593
1.2015997872	0.2626275289	0.9999537652	-1.1611403584
1.05723955	0.8679302659	0.9999537652	1.4394000994
-1.1603390474	0.3691974075	0.9999537652	1.2906927264
1.1274281612	0.5934347976	0.9999537652	1.1066493571
1.1063168623	0.7106320016	0.9999537652	1.1251025723
-1.418833986	0.006299	0.9999537652	-1.1130761234
-1.0997522348	0.5656251359	0.9999537652	1.1701654835
-1.0610145345	0.7837553448	0.9999537652	-1.112646512
-1.3178027369	0.0682141086	0.9999537652	-1.0930913189
1.1779948772	0.534737708	0.9999537652	-1.1511919005
1.0913090539	0.5422445268	0.9999537652	-1.2545732017
-1.7774568645	0.001742	0.9999537652	1.0778027527
11.5766527773	0.3453485698	0.9999537652	-2.2833428939
1.0915254028	0.6970863701	0.9999537652	-1.472049282
-1.2077069657	0.3958757779	0.9999537652	-1.4026183768
1.3000853227	0.1643933317	0.9999537652	-1.0900508808
1.141365169	0.7160474507	0.9999537652	-1.0386549784
1.1352638971	0.5805056095	0.9999537652	-1.0555960324
0	0	0	-5.3589986948
0	0	0	2.9985540909
1.036534056	0.8580524046	0.9999537652	-1.103061949
1.7364753052	0.0871315304	0.9999537652	-1.3851369611
1.0869018671	0.6078320992	0.9999537652	-1.0247322805
1.8541209205	0.1889969472	0.9999537652	1.1593389183
1.9053972733	0.2675957788	0.9999537652	-1.1633625896
1.2894430397	0.7031643746	0.9999537652	-3.4537504656
16.8433158146	0.2737706906	0.9999537652	-3.3853891979
1.4681234004	0.5952711777	0.9999537652	1.0949809192

-1.0333474347	0.9033506267	0.9999537652	-1.2251517201
2.0894806265	0.51754937	0.9999537652	-2.9704130837
0	0	0	2.3208796036
-1.1832445801	0.6579223784	0.9999537652	2.0756676815
-1.5311983595	0.0526218433	0.9999537652	-1.2764452543
1.0452318965	0.9282077995	0.9999537652	1.7397796038
-1.5174748936	0.0195303105	0.9999537652	-1.0388425823
-1.044149981	0.8118945345	0.9999537652	1.0480642143
-3.2699928362	0.157555617	0.9999537652	2.1856030494
-1.6988038983	0.5664473099	0.9999537652	3.0768011901
-1.0265103462	0.9037288313	0.9999537652	-1.0813960616
1.1268373502	0.8033495904	0.9999537652	-2.1500229021
-1.582236944	0.0325235808	0.9999537652	1.1783122745
-1.3121336108	0.1453815808	0.9999537652	-1.119345894
-1.0773179943	0.8652964544	0.9999537652	-1.0444522523
1.0310185201	0.8327062783	0.9999537652	1.1022102698
2.7933974408	0.6948713406	0.9999537652	1.0451154953
1.0068522111	0.9856706857	0.9999537652	-1.2255966104
-1.0409774945	0.8005864942	0.9999537652	-1.0701630811
-1.0191717191	0.9559911016	0.9999537652	-1.2273917056
3.2097096617	0.1617537627	0.9999537652	2.9985540909
-1.4493251305	0.3160794559	0.9999537652	-1.1898078549
-1.0820757546	0.6173418949	0.9999537652	1.1338001755
-1.124952368	0.6095409709	0.9999537652	-1.1118872995
-1.1457847193	0.9108654146	0.9999537652	0
1.149287899	0.3479711581	0.9999537652	1.1404333934
-1.1208614982	0.5414584093	0.9999537652	-1.0330868012
-6.9736190158	0.2250321147	0.9999537652	4.9527501949
-1.0089529604	0.9628142328	0.9999537652	1.0364739072
1.3140592355	0.3492277092	0.9999537652	1.1751789433
1.0990733552	0.8707054707	0.9999537652	-1.2363104227
0	0	0	0
-1.028889346	0.8779122485	0.9999537652	1.452138451
-1.3823233029	0.5185918578	0.9999537652	1.5179413665
4.2373079572	0.0230508341	0.9999537652	-1.7352264768
-1.5681585861	0.211200835	0.9999537652	1.1343479444
2.0877130777	0.5300403745	0.9999537652	-6.4360166533
1.2293721557	0.6959236393	0.9999537652	1.0395649746
45.0425653048	0.1352731676	0.9999537652	-2.3907756185
2.7933974408	0.6948713406	0.9999537652	8.8639046058
-1.0909523832	0.5793684984	0.9999537652	-1.0375097263
-1.3012163181	0.728507528	0.9999537652	-3.8919672371
0	0	0	-2.4271384939
4.5505976667	0.5557790267	0.9999537652	2.9985540909
-1.4471406585	0.0426840013	0.9999537652	1.159041731
1.040479317	0.784806039	0.9999537652	1.0149923097
-2.1326506013	0.4633398779	0.9999537652	-2.4941652249
1.3499426524	0.3196502706	0.9999537652	1.0634879949
1.6018881045	0.6925466852	0.9999537652	2.7192563323

0	0	0	-3.3818725041
0	0	0	4.9527501949
1.1750291162	0.5665007466	0.9999537652	-1.1140069997
4.5505976667	0.5557790267	0.9999537652	-5.3583704783
-1.1698447024	0.6168365705	0.9999537652	-1.1359253675
4.5505976667	0.5557790267	0.9999537652	2.9926666006
-1.054860168	0.7801687934	0.9999537652	1.1204924391
0	0	0	23.4460379037
1.2090200984	0.5476080637	0.9999537652	1.2201694775
-2.4065148764	0.2679247363	0.9999537652	-1.1659848296
1.196795406	0.666702689	0.9999537652	-1.503716555
4.5505976667	0.5557790267	0.9999537652	-2.0739144097
1.066664157	0.8089606899	0.9999537652	1.0463856997
-1.0547082242	0.8398104898	0.9999537652	1.3844485411
1.2807758528	0.09940768	0.9999537652	-1.0550311545
1.1059787706	0.800710731	0.9999537652	-1.6084213994
-1.0783617518	0.6159981459	0.9999537652	1.0225263947
1.3828243429	0.0738805779	0.9999537652	1.4979416122
-1.0202039148	0.9078109579	0.9999537652	-1.3554312836
-2.1334087267	0.5175748664	0.9999537652	-1.163826152
-1.2749274555	0.455207495	0.9999537652	-1.3518525677
1.5544407955	0.4128654436	0.9999537652	1.4831017011
1.3224892896	0.0877943224	0.9999537652	-1.0231727389
4.5545995984	0.5696620789	0.9999537652	-2.5434338259
-1.8808773671	0.4103619195	0.9999537652	-1.3567146076
2.5558846201	0.2829688636	0.9999537652	-1.5298940734
1.0627685541	0.7270799872	0.9999537652	-1.4150565051
-1.9462569054	0.1354184304	0.9999537652	-1.0225687453
-1.3451090191	0.1421091587	0.9999537652	-1.111161498
1.3021826629	0.2447494622	0.9999537652	-1.2376004066
-1.9661648673	0.0750283716	0.9999537652	-1.2862490202
-1.0285793351	0.9460080061	0.9999537652	1.0653364133
-1.0509965722	0.8456837929	0.9999537652	-1.0066055205
1.1665046315	0.6050333634	0.9999537652	1.2168600965
2.6885716491	0.2434112801	0.9999537652	-2.1542132939
1.0644272711	0.8963168609	0.9999537652	-1.4373790352
-7.0430176546	0.0829686738	0.9999537652	1.6681061489
4.5544554512	0.5691254106	0.9999537652	1.0677352434
1.2641867543	0.6656874377	0.9999537652	1.3511424844
1.2525465648	0.1724654616	0.9999537652	-1.2957631809
0	0	0	-4.03997453
-1.2657442334	0.2930423311	0.9999537652	-1.4856263691
1.2311598771	0.7102269607	0.9999537652	-1.0970546077
-1.0435087147	0.8100922284	0.9999537652	-1.1774188036
1.0085464844	0.9655318649	0.9999537652	-1.1454528563
-2.7379437534	0.0490319505	0.9999537652	-1.9579945106
1.041242241	0.8291282997	0.9999537652	1.3481756778
1.5601465308	0.5281513061	0.9999537652	-1.6896885141
1.0208805207	0.9118801733	0.9999537652	-1.0881509333

1.3258696851	0.1773089864	0.9999537652	1.0468359481
-1.3314639657	0.2965478945	0.9999537652	-1.0232789571
1.2098037704	0.3496633119	0.9999537652	1.0920564403
1.0492989766	0.7555626724	0.9999537652	1.1017664314
-1.3510764862	0.099041564	0.9999537652	-1.1481053473
1.039650522	0.8757422377	0.9999537652	-1.1642788485
0	0	0	2.9985540909
-1.0191902011	0.9579402026	0.9999537652	1.6349942807
-1.0599491199	0.7435217596	0.9999537652	1.3341478871
-1.3390129686	0.2472838794	0.9999537652	-1.3416941806
1.1585815174	0.8070043188	0.9999537652	1.2674474087
-1.1688076322	0.4064516576	0.9999537652	1.0064794219
1.4268563215	0.1629241824	0.9999537652	-1.0272175701
1.1505026035	0.7779726307	0.9999537652	-1.3167013681
1.5565153735	0.3314984628	0.9999537652	-1.6300855198
-1.1334978809	0.5320079577	0.9999537652	-1.0886989388
11.5864583426	0.3448001076	0.9999537652	-4.3749565696
1.6032449232	0.6828897941	0.9999537652	12.7820459093
-1.2498624805	0.5553175663	0.9999537652	-1.1741762696
1.5699684466	0.580659408	0.9999537652	1.4842910408
-1.1260678553	0.6737426764	0.9999537652	1.1702446062
-1.9568017681	0.2893502982	0.9999537652	1.634708435
0	0	0	4.9527501949
-2.1334087267	0.5175920343	0.9999537652	6.908559683
-2.4479441787	0.0367044062	0.9999537652	-1.5447133649
-1.1607129013	0.4045394488	0.9999537652	1.1175385527
1.3896916952	0.5611214357	0.9999537652	1.7414311168
-1.3361116832	0.5027236386	0.9999537652	-1.2442071513
1.2314060088	0.2559164327	0.9999537652	-1.123540661
0	0	0	-2.0460754901
1.6610722823	0.0897734859	0.9999537652	-1.1214982781
1.1504236229	0.7212534383	0.9999537652	1.1867348035
3.0834316129	0.137675855	0.9999537652	22.5564546887
0	0	0	4.9527501949
1.1615082715	0.4539279899	0.9999537652	-1.079940441
22.1644218026	0.2281090536	0.9999537652	-1.7614958461
2.3315363418	0.4569932801	0.9999537652	1.2149795761
1.4047998333	0.0931232281	0.9999537652	1.1530409265
-1.0370298509	0.7981590775	0.9999537652	-1.1384650592
4.5545995984	0.5696600016	0.9999537652	-1.6826577865
8.064174035	0.4193172572	0.9999537652	6.908559683
-1.2100887496	0.2178547784	0.9999537652	-1.1269152667
1.0570789238	0.7791166667	0.9999537652	-1.2021564776
1.1691161268	0.6180153317	0.9999537652	-1.0026289606
-2.5871290907	0.5001060748	0.9999537652	-1.8601323346
-1.067068821	0.8485929164	0.9999537652	-1.0767938797
1.1545761986	0.6071043638	0.9999537652	1.0962904631
0	0	0	-2.2697679462
-1.2449724804	0.6767191729	0.9999537652	-1.039597507

-1.0619428552	0.7844663098	0.9999537652	-1.1150022829
-1.2600796688	0.3597958047	0.9999537652	1.1116180045
16.8433158146	0.2737918885	0.9999537652	24.5435744105
-1.0198297051	0.9636339738	0.9999537652	-1.1106100071
-1.1192700412	0.5531385307	0.9999537652	1.0969084742
1.2907686534	0.4444380185	0.9999537652	-1.0048258875
1.2575084836	0.107962923	0.9999537652	1.2492845102
1.0273907875	0.8583568005	0.9999537652	1.0293785551
1.0236395102	0.9367361632	0.9999537652	-1.1680054244
-1.5378473775	0.4463996633	0.9999537652	1.0054017569
1.0428454802	0.8235387148	0.9999537652	-1.1018746942
9.8205505243	0.3812562769	0.9999537652	-1.8601323346
1.7193609644	0.3057486486	0.9999537652	-1.0676303356
-2.5871290907	0.5000967645	0.9999537652	1.4007535733
-1.1770676473	0.4819150643	0.9999537652	-1.2732049116
2.7933974408	0.6948713406	0.9999537652	0
1.032981085	0.8989489842	0.9999537652	1.0292637479
-1.2470248275	0.3876231124	0.9999537652	-1.0424269105
-1.1467684078	0.5770087193	0.9999537652	1.4589831901
1.2815359084	0.1931513171	0.9999537652	1.3429042969
-1.5778038045	0.5472260021	0.9999537652	1.2773413674
1.2264638687	0.1579185969	0.9999537652	1.2790406341
-1.9290774189	0.6162844244	0.9999537652	12.7732027325
0	0	0	0
1.1857008507	0.7275479954	0.9999537652	1.0722232032
-1.3038112768	0.2521719475	0.9999537652	1.0594211181
-1.4647167555	0.0532515948	0.9999537652	-1.3558857238
2.7933974408	0.6948713406	0.9999537652	0
-1.0308401072	0.8401489039	0.9999537652	1.05872405
-1.0859522881	0.5263838918	0.9999537652	-1.0354184459
1.4709947928	0.1546581448	0.9999537652	1.0396699623
-1.5881166222	0.7196088008	0.9999537652	-4.7557826338
1.2048594181	0.6745238692	0.9999537652	-1.3946366495
-1.5867714314	0.7161394982	0.9999537652	-2.5808051651
-1.0965736598	0.6284010207	0.9999537652	-1.0217300038
1.0234232714	0.934991721	0.9999537652	1.0862156339
0	0	0	1.2398837679
1.2131929555	0.3553808445	0.9999537652	-1.109124632
0	0	0	2.996475627
2.481077056	0.1991577997	0.9999537652	1.1597107703
-1.8837873194	0.3490819291	0.9999537652	-1.2222390237
1.0464198275	0.9215490832	0.9999537652	-1.8186334012
-1.2651992128	0.1316702361	0.9999537652	-1.3104582613
-1.0349920834	0.8755716003	0.9999537652	-1.0081220711
-1.0425976033	0.7787105059	0.9999537652	1.2433218081
-1.3906298771	0.0353257539	0.9999537652	-1.2707902954
-1.0877321833	0.8925972361	0.9999537652	-1.1891771074
-1.0158491591	0.9406491422	0.9999537652	1.0415100212
1.1943767543	0.3294204005	0.9999537652	-1.2247525511

1.5014581463	0.0293894415	0.9999537652	-1.3031882664
1.2231451982	0.2310652934	0.9999537652	1.1646150158
-1.0345852564	0.7798145022	0.9999537652	1.0660679311
1.3064100778	0.4689158932	0.9999537652	-1.0747216812
2.1077739145	0.2653322096	0.9999537652	-1.0355383115
-1.4793459569	0.2178485357	0.9999537652	-1.0690524939
0	0	0	9.7116702472
1.8816342439	0.216566109	0.9999537652	1.228767494
1.0530778882	0.8456091488	0.9999537652	-1.3774005225
1.2209655098	0.213487925	0.9999537652	-1.036112813
1.0435945408	0.87820214	0.9999537652	-1.3109588781
-1.0849393848	0.7154511402	0.9999537652	-1.0485371829
-2.9548384089	0.3320356365	0.9999537652	0
-1.0156822469	0.9490469311	0.9999537652	-1.9543770932
8.064174035	0.4193233519	0.9999537652	-2.1302390584
1.0441365929	0.8774567076	0.9999537652	1.263520793
0	0	0	9.7116702472
-1.2742713741	0.741750854	0.9999537652	-1.0595590983
2.2827468184	0.2949241459	0.9999537652	-4.0137283111
1.3766897199	0.1378843191	0.9999537652	1.2162449357
2.1527660067	0.001218	0.9999537652	1.0279068361
-1.2041079789	0.323304076	0.9999537652	-1.7669610497
-1.6479062862	0.2504451174	0.9999537652	-1.1149655531
1.2792732586	0.2044816439	0.9999537652	1.1272415695
1.1907196182	0.4883574483	0.9999537652	1.9569263205
-1.1353647453	0.6163608574	0.9999537652	-1.3315481694
1.2955970728	0.1914148741	0.9999537652	-1.1989476149
1.2984791627	0.1331481461	0.9999537652	1.1777778501
-1.207347084	0.4103218259	0.9999537652	-1.3236435604
-1.0315434734	0.8371232326	0.9999537652	-1.3110157555
-6.9762758667	0.2572951647	0.9999537652	6.908559683
1.1316185042	0.7110613521	0.9999537652	-1.2399398533
-1.1457847193	0.9113769398	0.9999537652	9.7116702472
1.2096399056	0.5646164549	0.9999537652	1.0727372764
6.3075231393	0.4852412867	0.9999537652	-4.8059548358
-1.4102132064	0.4886247623	0.9999537652	-1.3938181653
-1.0606362831	0.6956012866	0.9999537652	-1.1298364197
2.1650257591	0.3957180379	0.9999537652	2.9985540909
1.2479349758	0.2949638421	0.9999537652	-1.0380379978
1.0499381996	0.6673164377	0.9999537652	1.0912322027
-1.0225789549	0.9445092791	0.9999537652	-1.0222204389
1.043101863	0.8638792068	0.9999537652	1.0222708069
-1.3369663034	0.1304068318	0.9999537652	-1.25017036
-1.0044497915	0.9907702844	0.9999537652	1.1823257365
-1.0455798076	0.8420779	0.9999537652	-1.6023426764
1.8686704283	0.4506424113	0.9999537652	1.4195464433
-1.5880958314	0.3400877239	0.9999537652	1.0132271448
-1.270562122	0.2753244788	0.9999537652	-1.1795331513
-1.3204269266	0.4200643299	0.9999537652	-1.5265388131

-3.7785697044	0.0502111918	0.9999537652	2.0230057879
3.8622037496	0.6194029705	0.9999537652	0
-1.2196401939	0.1320431036	0.9999537652	-1.1539340775
-1.2607293934	0.6736917417	0.9999537652	-1.0763957721
-2.4377475547	0.0160115123	0.9999537652	1.1330996428
1.2498870213	0.2745204609	0.9999537652	-1.2776170875
2.4143254621	0.1247846832	0.9999537652	1.8081208762
-1.7293265403	0.4355204193	0.9999537652	-1.2809257219
3.0189841269	0.1037183763	0.9999537652	-1.5470541076
1.0066570173	0.9923896849	0.9999537652	-1.5719718188
-17.0527724669	0.1063660528	0.9999537652	-1.859280336
-1.2356571081	0.0969574853	0.9999537652	-1.0685616283
-1.1418633071	0.5237493171	0.9999537652	1.1783204451
1.0102613121	0.9643936247	0.9999537652	-1.1448643895
1.1611759353	0.4829989044	0.9999537652	-1.0937265607
-1.0268470296	0.8868303152	0.9999537652	-1.0211322999
1.334958699	0.3352230142	0.9999537652	-1.378565536
1.3417664133	0.2180465002	0.9999537652	1.0041849578
-1.3120680583	0.3374807224	0.9999537652	1.0386943992
-1.0132111758	0.9430237477	0.9999537652	-1.2018986942
0	0	0	4.9527501949
-1.0199370498	0.8752249717	0.9999537652	1.1711170835
-1.1451054031	0.5255906467	0.9999537652	1.0156013297
1.5616557332	0.3263259699	0.9999537652	-1.2363599305
-1.010378892	0.9810184155	0.9999537652	1.1279243293
-1.164088214	0.3256578925	0.9999537652	-1.2346462956
1.392424055	0.0917604727	0.9999537652	1.0757666433
-1.2110226476	0.6911343772	0.9999537652	1.6764330611
-1.3044732667	0.7563770174	0.9999537652	1.1591954369
1.0590049173	0.6984952954	0.9999537652	-1.0510870327
4.5505976667	0.5557790267	0.9999537652	6.908559683
-2.4799918249	0.0124168696	0.9999537652	-1.5771444742
16.8433158146	0.2737488288	0.9999537652	1.2753124274
-2.0066854809	0.0726506836	0.9999537652	-1.10558782
-1.2543756335	0.0844492408	0.9999537652	-1.3557101564
-3.7442675911	0.0497470251	0.9999537652	1.4856749753
9.8205505243	0.381261333	0.9999537652	-15.5945848976
-1.1284709041	0.5380490346	0.9999537652	-1.0869786006
-2.6399157167	0.3173634304	0.9999537652	-1.8673517833
1.1771203451	0.395979467	0.9999537652	-1.1316576448
0	0	0	-2.222435925
-1.0085765745	0.9587260141	0.9999537652	-1.0768202987
-1.2577124827	0.2581963069	0.9999537652	-1.8162844319
1.0927010352	0.6995298987	0.9999537652	-1.1068774857
1.1001460438	0.7460478559	0.9999537652	1.2671511582
1.1029820449	0.6932844392	0.9999537652	-1.1847361501
-1.0157131754	0.9230344106	0.9999537652	-1.0550119014
-1.015161324	0.9178886146	0.9999537652	-1.7219734033
1.4098308678	0.3478319002	0.9999537652	-1.0411656692

0	0	0	10.818785458
0	0	0	4.9527501949
0	0	0	-5.3583704783
1.3765758161	0.544052857	0.9999537652	-1.1256227609
-1.1532512476	0.7985494227	0.9999537652	2.0636487513
1.8761040358	0.2582301484	0.9999537652	-1.393553444
-1.0481751604	0.8867066185	0.9999537652	-1.2237436856
-1.1104845875	0.4697769365	0.9999537652	1.0523998732
1.345923943	0.3844384625	0.9999537652	-1.1753043794
1.1135180875	0.5339761643	0.9999537652	1.1416410478
1.8373097308	0.4508328576	0.9999537652	-2.5401817516
-1.2895081652	0.193663793	0.9999537652	1.0959337861
-1.0330319854	0.9253703569	0.9999537652	1.0366893556
-1.0406514179	0.8530789912	0.9999537652	1.081990147
-1.144585995	0.4545167067	0.9999537652	-1.045769983
-1.214822317	0.6581178543	0.9999537652	1.2066463783
1.6642583336	0.2097999134	0.9999537652	-1.1332909573
-1.1361797731	0.4710123443	0.9999537652	1.0772114024
-1.2411090088	0.1521071615	0.9999537652	1.081070195
-1.0593151771	0.8215478746	0.9999537652	-1.567703606
-1.5407794824	0.1503315494	0.9999537652	-1.2997421261
1.208704874	0.3307084104	0.9999537652	-1.0711267175
0	0	0	2.9985540909
0	0	0	1.2400291296
1.1025279052	0.7411250533	0.9999537652	-1.092829959
-1.1039276934	0.571996538	0.9999537652	-1.4673057872
1.1264249507	0.7874515889	0.9999537652	1.5283555043
-2.3929677766	0.1709044729	0.9999537652	-1.2860377464
3.7985791816	0.2499436608	0.9999537652	-2.1744673066
0	0	0	6.908559683
-1.0670788264	0.6665896935	0.9999537652	-1.2411495764
4.5505976667	0.5557790267	0.9999537652	-1.3587293308
-1.713314385	0.4942542716	0.9999537652	-1.1192843301
-4.5374012562	0.0439028396	0.9999537652	-1.1726268366
-2.9548384089	0.3320347989	0.9999537652	3.6971811836
2.7933974408	0.6948713406	0.9999537652	2.9985540909
1.1250543471	0.6041301734	0.9999537652	1.0756585411
1.4899610928	0.1469978645	0.9999537652	-1.4322353
1.029417669	0.8207619124	0.9999537652	-1.1229442873
1.9257237851	0.4119406519	0.9999537652	-1.4602762239
-1.1573970448	0.3884279219	0.9999537652	-1.1712273957
1.1394226192	0.7235795986	0.9999537652	-1.2385059546
2.3256471815	0.1785262566	0.9999537652	-1.1070345832
-1.2228679769	0.1931591571	0.9999537652	-1.3114339991
1.2043597123	0.4011839477	0.9999537652	-1.0129665659
1.1259226368	0.5733641573	0.9999537652	-1.1523081806
1.1922709584	0.290010515	0.9999537652	1.0738134296
1.0317763013	0.8426344875	0.9999537652	1.6562393951
-1.2114213602	0.4043430392	0.9999537652	1.0162490856

1.9839653328	0.0219418958	0.9999537652	1.0052110797
1.2349787261	0.3828078129	0.9999537652	1.3086932652
0	0	0	1.1354046367
2.7933974408	0.6948713406	0.9999537652	1.3101765491
-1.0455686368	0.875573689	0.9999537652	-1.1125800587
2.7957660672	0.705502207	0.9999537652	-2.6248262166
1.4029703146	0.5259625967	0.9999537652	1.5010337023
1.1225627191	0.4037246196	0.9999537652	1.2631810827
1.1811480828	0.5805774794	0.9999537652	-1.6102876173
8.064174035	0.4193294559	0.9999537652	1.4357352596
1.3588921917	0.799737259	0.9999537652	10.818785458
-1.2202318239	0.7324022135	0.9999537652	-1.0461018666
1.1236237983	0.4259764678	0.9999537652	-1.2619607446
-1.0488824431	0.8053778574	0.9999537652	-1.1917520062
2.7933974408	0.6948713406	0.9999537652	-9.7554110723
2.7933974408	0.6948713406	0.9999537652	0
6.3128691894	0.4808960613	0.9999537652	0
11.5766527773	0.3453604812	0.9999537652	-2.1294907556
1.2319279711	0.37693661	0.9999537652	-1.6832496079
1.3154794667	0.1498239394	0.9999537652	-1.2384697077
1.7065609388	0.0826933768	0.9999537652	-1.3153603421
-1.1318243716	0.5119257692	0.9999537652	1.0466384416
-1.2393765322	0.1751305958	0.9999537652	-1.3043577061
-1.1002525041	0.5207087799	0.9999537652	-1.2353178773
-1.4772849924	0.1422745918	0.9999537652	-1.1062512672
-1.7155763775	0.5413192404	0.9999537652	2.2942711679
4.5505976667	0.5557790267	0.9999537652	-1.9072087437
-1.0659181843	0.6261836128	0.9999537652	-1.1438406256
1.1763867606	0.5431205597	0.9999537652	1.1258456249
1.7385979073	0.3109535202	0.9999537652	-1.8122170016
1.0174927746	0.9415196219	0.9999537652	-1.1587235065
1.1162834585	0.9273396232	0.9999537652	10.8337816869
8.064174035	0.4193355693	0.9999537652	1.590997316
-1.5473884214	0.1316467169	0.9999537652	-1.4665734572
1.4051186348	0.0768603959	0.9999537652	1.2093502298
-1.3137642683	0.6668299424	0.9999537652	-1.2102268229
4.5505976667	0.5557790267	0.9999537652	33.3754495834
-1.5783503792	0.5297238837	0.9999537652	-1.616488444
-2.065175426	0.2150273517	0.9999537652	-1.0466447002
-1.9702891638	0.0798160463	0.9999537652	1.0039139339
-1.1011409245	0.5697092134	0.9999537652	-1.2278578401
0	0	0	1.7042814026
1.8357580406	0.0278021424	0.9999537652	1.2575807613
7.6841730958	0.0528755818	0.9999537652	-1.1618546036
-1.1139243258	0.3756516541	0.9999537652	-1.1283332336
1.2342967253	0.7628312814	0.9999537652	-1.1088878398
6.3130691196	0.4798469433	0.9999537652	-2.1294907556
1.17757233	0.3600702569	0.9999537652	1.1092867724
4.5545995984	0.5696579243	0.9999537652	9.7116702472

1.3154484946	0.2316375261	0.9999537652	1.0199059729
4.5505976667	0.5557790267	0.9999537652	-3.3822687648
1.5797041712	0.0206424265	0.9999537652	1.0316867166
-1.5749738312	0.284495083	0.9999537652	-1.8436572032
2.3077154233	0.2685512486	0.9999537652	1.2283827455
0	0	0	1.4514511122
-1.4431747638	0.3202592949	0.9999537652	-1.4159530023
-1.3129493191	0.1060663274	0.9999537652	-1.1002063844
-1.3070344795	0.3699780667	0.9999537652	-1.0828575686
1.1041992656	0.5426916107	0.9999537652	-1.0814952784
-2.5861438062	0.4721076244	0.9999537652	-2.6399253151
1.6200274653	0.1445362676	0.9999537652	1.0032699964
1.0220442395	0.9630983943	0.9999537652	-1.366228589
0	0	0	0
-1.0089195645	0.9925494358	0.9999537652	1.3561873216
-1.8237103933	0.5664269327	0.9999537652	-1.649233751
-1.7569468604	0.0933338279	0.9999537652	-1.3266223117
-1.5437484553	0.2083675897	0.9999537652	1.0706429242
-1.003882893	0.9883992714	0.9999537652	-1.0846844381
-1.4574462276	0.3272496935	0.9999537652	-1.7240293712
1.2386374862	0.1880530826	0.9999537652	-1.2275731672
-1.3611809183	0.660749763	0.9999537652	-1.0296106127
-1.0048668959	0.9812028488	0.9999537652	1.1431506756
1.3347485961	0.0698637007	0.9999537652	1.0928060342
-1.3917747917	0.6970489516	0.9999537652	-1.4706174584
2.0885615936	0.5188097351	0.9999537652	1.4527805491
1.1812385199	0.1425927911	0.9999537652	1.2212962458
-1.040046278	0.8633741762	0.9999537652	-1.0293235933
3.6560415165	0.0350336184	0.9999537652	1.0075827047
0	0	0	0
1.3832036905	0.7447508225	0.9999537652	-1.1204482161
1.1168981342	0.6024939848	0.9999537652	1.0371481598
-1.2125935268	0.7139683215	0.9999537652	1.5070950561
1.1057535685	0.6980353007	0.9999537652	1.0965873212
2.7933974408	0.6948713406	0.9999537652	-2.2275543816
0	0	0	6.908559683
0	0	0	2.996475627
-1.070697391	0.7101742083	0.9999537652	-1.2380109474
0	0	0	12.7820459093
1.8900501345	0.0637304322	0.9999537652	1.7863340122
1.2152319459	0.2921953334	0.9999537652	-1.021906891
-1.261684088	0.6951476887	0.9999537652	1.3066084358
27.4155731128	0.1963476416	0.9999537652	-1.3876714084
9.8205505243	0.3812663801	0.9999537652	0
-4.3214691297	0.1498150591	0.9999537652	12.7820459093
1.5618236382	0.4052027396	0.9999537652	1.0090123198
-1.0274047275	0.9108363187	0.9999537652	-1.0773432321
3.0602334741	0.3280640279	0.9999537652	3.1847673229
1.1350793029	0.5036453881	0.9999537652	1.046744879

-1.1342259121	0.6632388034	0.9999537652	-1.1325438164
2.7933974408	0.6948713406	0.9999537652	0
3.0590680122	0.3354937932	0.9999537652	22.424363668
1.0785304702	0.7400926007	0.9999537652	1.1072320158
1.8615850356	0.0007879	0.9999537652	-1.2057380758
2.3315363418	0.4571079906	0.9999537652	-1.5289664635
1.1225440058	0.5933175895	0.9999537652	-1.3082795106
-1.1378669652	0.4633405051	0.9999537652	-1.017279228
1.0358174347	0.8707262662	0.9999537652	1.1132979619
1.1271822705	0.696000932	0.9999537652	1.2273233034
-1.2874099099	0.2156395149	0.9999537652	-1.0958479888
-1.0025664452	0.9828788022	0.9999537652	1.0179357948
1.1132252094	0.7570251326	0.9999537652	-1.0764252542
2.0539087089	0.1034438612	0.9999537652	1.0520849078
0	0	0	10.818785458
1.6783510795	0.0331886958	0.9999537652	1.0493849037
1.0217691486	0.9051516279	0.9999537652	-1.366962936
1.1007449551	0.6637639136	0.9999537652	1.1286045855
1.3278551105	0.1755529553	0.9999537652	-1.1212516947
-1.4479386307	0.044979365	0.9999537652	1.0662499599
1.1059703615	0.7632206757	0.9999537652	-1.1425153632
-1.1343361967	0.5506079848	0.9999537652	-1.3697880257
1.0455120441	0.8935559769	0.9999537652	1.1412925518
-1.3632333972	0.1976959166	0.9999537652	-1.000491132
-1.0518507437	0.8714302116	0.9999537652	-1.0162859083
1.3473247044	0.5156250212	0.9999537652	-1.4097915498
-1.8462877751	0.3514459803	0.9999537652	-1.106512183
-1.7135297713	0.2033277488	0.9999537652	-1.3261191407
2.8279434874	0.0884588012	0.9999537652	1.0014601474
1.0794086068	0.8103717937	0.9999537652	-1.3406927006
6.3075231393	0.4852317874	0.9999537652	4.0510214676
-1.1004355672	0.6384500706	0.9999537652	1.1344998886
-1.0513856881	0.8410598082	0.9999537652	-1.0595443462
-1.1880775569	0.5391318769	0.9999537652	1.0122145576
-1.4531411845	0.6040252215	0.9999537652	1.3988325769
-1.0272270355	0.9188219939	0.9999537652	1.189863323
-1.0911433338	0.7392100051	0.9999537652	1.4941039635
1.0184031377	0.9544259811	0.9999537652	1.3274266497
1.6754456997	0.4292795127	0.9999537652	-1.3590484476
-1.0681648186	0.8667077161	0.9999537652	-2.7352741074
-1.5069259724	0.1708941601	0.9999537652	-1.6119944413
-1.1457847193	0.9115501622	0.9999537652	2.996475627
-2.0058375128	0.1046722166	0.9999537652	1.7148712287
-1.5881166222	0.7196052286	0.9999537652	-2.2809262314
-1.0465915848	0.8435670874	0.9999537652	1.3985782511
-1.0619267211	0.9009311065	0.9999537652	1.2811802077
2.0354520887	0.4471243048	0.9999537652	-2.0467835026
2.2186454522	0.2011907431	0.9999537652	-1.2524890471
-1.0469169629	0.9634396732	0.9999537652	-1.3307652929

-2.1307903719	0.4719402534	0.9999537652	1.4127221338
4.5505976667	0.5557790267	0.9999537652	4.9561842702
0	0	0	2.996475627
0	0	0	2.9988886918
-1.346787305	0.4562749508	0.9999537652	1.1800101661
-1.968160983	0.0310227399	0.9999537652	1.1300568683
0	0	0	33.3754495834
1.1162834585	0.9274601222	0.9999537652	0
-1.0478369715	0.7479754904	0.9999537652	-1.2742820457
-1.7432752692	0.4032918419	0.9999537652	-1.2500975743
1.054362661	0.8383353599	0.9999537652	1.0405332398
1.1683700158	0.4441430442	0.9999537652	-1.0709738031
46.6807036647	0.1309531656	0.9999537652	1.6303647068
1.1418371148	0.4430620805	0.9999537652	-1.1583004765
2.2760937505	0.2561300108	0.9999537652	1.2071735423
-1.2265853195	0.1045778743	0.9999537652	-1.1376221827
1.3407294023	0.1134917926	0.9999537652	1.2958251832
-1.953078177	0.1020444953	0.9999537652	-1.4078044981
-1.0045532804	0.9813349354	0.9999537652	-1.322048517
3.3058503755	0.2779758097	0.9999537652	-1.3069793515
2.0642641853	0.0476652242	0.9999537652	1.007250981
0	0	0	0
-1.1285043416	0.4844812468	0.9999537652	-1.2549749233
-2.0435859602	0.2729222297	0.9999537652	1.1119016458
1.3057267165	0.6334500042	0.9999537652	-1.8758407561
1.0947414544	0.6152839268	0.9999537652	-1.1005854725
-1.1511107936	0.6526280022	0.9999537652	1.0183472457
1.6017137321	0.5052569949	0.9999537652	-2.1800718368
1.0345456238	0.9701489939	0.9999537652	-1.4229595386
-1.1052756593	0.6403761198	0.9999537652	-1.3662487148
-1.2732611382	0.1456586417	0.9999537652	-1.2342315982
1.8464200217	0.5964582209	0.9999537652	1.1256010339
-1.1948881021	0.4745291684	0.9999537652	-1.2907313712
1.4650376402	0.5859643184	0.9999537652	-1.5052536245
1.0970968203	0.6482464744	0.9999537652	1.1803377277
-1.1337697354	0.5702200452	0.9999537652	-1.0483733223
-1.2101741221	0.4250470731	0.9999537652	-1.4170877758
3.6274675349	0.062661038	0.9999537652	-1.3365115701
-1.1170265702	0.5481868017	0.9999537652	1.3486513305
1.1242358844	0.8721387441	0.9999537652	1.191154938
-3.2724810761	0.1892285141	0.9999537652	-1.047728856
1.0450406427	0.8917247236	0.9999537652	-1.3244605112
1.0402380815	0.9149863326	0.9999537652	-1.0168547654
-1.0222894065	0.9447608703	0.9999537652	-1.0558263916
1.3498440135	0.1933889798	0.9999537652	1.0542836883
1.0513712786	0.8884915418	0.9999537652	1.1368268503
-1.7856811462	0.0816335863	0.9999537652	-1.1526541247
0	0	0	20.6004768562
-2.4619996563	0.1413460529	0.9999537652	-1.4090385426

1.63824064	0.1288061846	0.9999537652	-1.3224177195
2.7933974408	0.6948713406	0.9999537652	0
-1.0213574992	0.8912072113	0.9999537652	-1.018355381
-1.0113316251	0.9513295426	0.9999537652	-1.0940162591
2.0123068682	0.095779085	0.9999537652	1.2866242178
1.1537725945	0.7050030658	0.9999537652	-1.1828579189
-1.0681293201	0.8042453782	0.9999537652	-1.0309690345
-1.7562484372	0.2082375399	0.9999537652	1.0088782704
1.383157405	0.7358556793	0.9999537652	1.0666207935
1.0190248587	0.9233308807	0.9999537652	-1.0724420454
1.1384004922	0.5270511873	0.9999537652	1.2020665003
2.7933974408	0.6948713406	0.9999537652	-2.7911910562
-1.0540356878	0.7082080483	0.9999537652	1.0131547836
1.0469035417	0.8210274951	0.9999537652	-1.0209863603
1.1222280161	0.9093250984	0.9999537652	-1.7642364482
1.2300465216	0.408886445	0.9999537652	1.1981906556
-1.01501261	0.9505199001	0.9999537652	-1.0553460161
-1.0904097914	0.7306392535	0.9999537652	1.0800630199
-1.02571069	0.9167627597	0.9999537652	-1.147805551
2.9503019035	0.2026001842	0.9999537652	-1.6731571458
-1.3377354311	0.3157103065	0.9999537652	1.017263506
2.1324144008	0.1810798079	0.9999537652	1.1498623554
-1.1427778392	0.6932194056	0.9999537652	1.2895122884
1.1543555604	0.5186048315	0.9999537652	-1.7172902327
-1.046888968	0.7477368737	0.9999537652	-1.0870376858
-1.1053764341	0.599254403	0.9999537652	-1.0932685909
1.0887865237	0.5546575928	0.9999537652	1.1228074594
0	0	0	2.996475627
1.0199396468	0.9460299257	0.9999537652	-1.0800440071
0	0	0	0
1.3159538127	0.1190906353	0.9999537652	1.0950264229
4.5505976667	0.5557790267	0.9999537652	-1.6624136632
1.2754757452	0.6085797021	0.9999537652	1.5893507858
1.1134440428	0.3954590699	0.9999537652	1.0177269216
-1.3806680149	0.0765388777	0.9999537652	-1.3130578669
1.1086291888	0.673056313	0.9999537652	-1.2945775116
11.5864583426	0.3447824979	0.9999537652	-1.9360727542
0	0	0	-1.1242401582
2.701471957	0.2702272836	0.9999537652	-1.4346156636
1.0273120224	0.8612440592	0.9999537652	2.0203657024
3.1209897832	0.0161429559	0.9999537652	1.9326334757
15.1008094413	0.2915801219	0.9999537652	8.8639046058
2.9458943806	0.0139292031	0.9999537652	1.4181845008
1.6962523591	0.4352556217	0.9999537652	-1.2846638916
-1.1298702821	0.650858824	0.9999537652	1.0571239172
-1.1050841704	0.738800059	0.9999537652	1.089851134
1.3747748923	0.1479652789	0.9999537652	1.1059389326
2.4994474338	0.1264476835	0.9999537652	-1.2389082909
-1.2717832802	0.7774354067	0.9999537652	2.4881108915

-1.6844643939	0.1141540222	0.9999537652	1.1588655246
18.5983228161	0.2588999519	0.9999537652	1.2125664034
1.7371865656	0.4486353557	0.9999537652	1.2776901668
1.0457666265	0.8411081061	0.9999537652	-1.0463267697
1.3588921917	0.7996041584	0.9999537652	-2.7864308214
-1.4841110134	0.3734004535	0.9999537652	-1.2414239924
-1.5990709095	0.3460143768	0.9999537652	1.0257700707
1.80267916	0.4093718283	0.9999537652	2.1548787182
-1.5102607004	0.0351109537	0.9999537652	-1.1135364169
11.5766527773	0.3453724053	0.9999537652	-1.6210814782
1.1612717392	0.3350069669	0.9999537652	1.03514426
1.3069608346	0.73779073	0.9999537652	2.4810553188
1.06078406	0.7024707728	0.9999537652	-1.0229287615
1.9156018173	0.300588126	0.9999537652	1.1126092251
9.8205505243	0.3816478295	0.9999537652	-1.221605188
2.7957660672	0.7055045776	0.9999537652	2.996475627
2.5705379464	0.0188934062	0.9999537652	1.1927603811
5.6954555782	0.003474	0.9999537652	3.3172145292
4.2651728822	0.001788	0.9999537652	1.3615461777
2.8162276504	0.366827699	0.9999537652	-2.9704130837
-6.9762758667	0.2572121796	0.9999537652	2.2926838869
-1.1273035988	0.4250441749	0.9999537652	-1.0273124493
1.1600132444	0.6069677958	0.9999537652	-1.0168943843
-1.03975212	0.8525472231	0.9999537652	1.0044624578
1.0990780746	0.5780759642	0.9999537652	1.221395586
1.737981082	0.161613865	0.9999537652	1.0430641248
-1.8590077338	0.0870649945	0.9999537652	-1.2120560827
1.0599363666	0.7130139276	0.9999537652	-1.1992998236
-1.0860286715	0.7018497481	0.9999537652	1.1177445677
-1.3306509057	0.2407273136	0.9999537652	-1.0255575343
1.4465266046	0.2016454056	0.9999537652	-1.2372447455
1.002202007	0.9922829549	0.9999537652	1.2057107994
-1.1440835562	0.5228302509	0.9999537652	1.0730330732
0	0	0	2.996475627
-2.0042093694	0.4615758655	0.9999537652	-1.1636261403
1.1621704408	0.7343927337	0.9999537652	-1.3287663901
0	0	0	16.7037438866
1.1041811368	0.6349566373	0.9999537652	1.1627002836
1.0067443913	0.9758270117	0.9999537652	-1.1546099183
-1.5419520715	0.3264857969	0.9999537652	-1.2001233495
20.3530564257	0.2430051447	0.9999537652	-1.0008261553
-1.5159463026	0.0205505456	0.9999537652	-1.1626627647
1.8004822351	0.2735802704	0.9999537652	-1.5633885455
-1.0532663664	0.8158126567	0.9999537652	-1.0796574466
-1.2514084948	0.2267340049	0.9999537652	-1.0485520336
16.296263041	0.2962347823	0.9999537652	-1.466151735
-1.1763671393	0.4339658541	0.9999537652	1.3306437674
1.0466181378	0.8120309901	0.9999537652	1.2876608444
-1.0155937669	0.9480379185	0.9999537652	1.063304543

0	0	0	1.1333153721
1.1543216269	0.2541522921	0.9999537652	1.1655530138
-1.0899107906	0.5111774664	0.9999537652	-1.3047129738
2.3306483975	0.4661126935	0.9999537652	1.0666207935
-1.1457555741	0.9106210146	0.9999537652	-1.8290769175
0	0	0	4.9527501949
16.8433158146	0.2737655673	0.9999537652	-1.4683576122
1.0536604232	0.6576492353	0.9999537652	1.1142435764
2.0056634044	0.1423428125	0.9999537652	1.1490567045
-1.3751395195	0.0774366041	0.9999537652	1.0148236347
-1.7787002326	0.001825	0.9999537652	-1.0315227857
0	0	0	2.996475627
1.4063986972	0.0606552986	0.9999537652	-1.3119566425
2.3335098933	0.4401826908	0.9999537652	-1.1241083699
1.839484446	0.2645089588	0.9999537652	-1.1387671852
1.0876049784	0.7711459915	0.9999537652	-1.0759801931
0	0	0	2.9985540909
6.3075231393	0.4852222955	0.9999537652	-1.7809308717
2.8162992884	0.3667120061	0.9999537652	-1.0942329492
1.1677464601	0.6392231997	0.9999537652	-1.3407836056
-2.5861438062	0.4732359327	0.9999537652	44.0115004274
0	0	0	0
1.0557507578	0.6751093692	0.9999537652	-1.2821701016
4.5505976667	0.5557790267	0.9999537652	0
1.5969197685	0.0121216223	0.9999537652	-1.4763747302
-1.1114910845	0.5807251282	0.9999537652	1.069876727
-1.2177113287	0.1859984715	0.9999537652	1.0253324604
-1.0908276674	0.6718033531	0.9999537652	-1.1396370742
8.064174035	0.4193416922	0.9999537652	22.5383525416
1.0194909176	0.9152168645	0.9999537652	-1.0471259868
1.0369656959	0.8340166371	0.9999537652	-1.0000012186
-1.2377843894	0.6093826736	0.9999537652	-1.3350229505
-1.0230277198	0.857115768	0.9999537652	-1.0057990478
1.2911318369	0.6968318206	0.9999537652	1.1159520636
4.5505976667	0.5557790267	0.9999537652	3.7228836277
-1.3697196358	0.7436595474	0.9999537652	2.996475627
-1.1848732952	0.281527617	0.9999537652	-1.0519660874
0	0	0	6.908559683
2.7957660672	0.7055069491	0.9999537652	0
-1.0543047174	0.7599053367	0.9999537652	-1.1702006067
-2.4409066979	0.0157049425	0.9999537652	1.1014119819
0	0	0	2.996475627
-1.2689832482	0.3359471239	0.9999537652	3.2731217557
-2.8606942226	0.1090460506	0.9999537652	-12.0476907974
1.0524988558	0.6770562046	0.9999537652	1.1106426934
1.2154426353	0.5667666207	0.9999537652	-1.16496205
-1.0899406675	0.6910372511	0.9999537652	-1.136175307
0	0	0	1.2277336482
1.6692396298	0.1212130209	0.9999537652	1.2003199654

2.7933974408	0.6948713406	0.9999537652	4.9567375629
-1.5989325897	0.1096827162	0.9999537652	1.0456781294
-1.2217199882	0.1245797247	0.9999537652	-1.1576683914
-3.1190154029	0.2770687298	0.9999537652	-1.1599986346
-1.0301002711	0.8433618254	0.9999537652	-1.1797958323
-1.0052760915	0.9894664973	0.9999537652	-1.2585898004
-1.0656346646	0.8147681085	0.9999537652	-1.1695895831
2.7933974408	0.6948713406	0.9999537652	2.996475627
1.5259976099	0.0680113431	0.9999537652	-1.546339139
0	0	0	2.996475627
1.4210838424	0.0348187149	0.9999537652	-1.7060576568
-1.5875118025	0.7201580292	0.9999537652	20.6027844127
2.7933974408	0.6948713406	0.9999537652	-1.1606649125
1.1812620667	0.6386954592	0.9999537652	-1.1010701503
-1.7239935755	0.0972538552	0.9999537652	1.1045138456
1.1028881939	0.6981415636	0.9999537652	-1.223676494
1.2559353002	0.35037501	0.9999537652	-1.1473970482
1.1162834585	0.9277159244	0.9999537652	-1.8594983184
-1.1039491595	0.6726255246	0.9999537652	-1.1470625326
3.1875904576	0.0323072714	0.9999537652	-1.1088528593
-1.190387193	0.3312486124	0.9999537652	-1.0978619391
1.2949104594	0.365808996	0.9999537652	1.5963482859
0	0	0	-2.4096087603
-1.5650679954	0.1541124997	0.9999537652	-1.1641581635
1.2992021391	0.2371721661	0.9999537652	1.1745119525
3.8622037496	0.619341334	0.9999537652	0
-1.387029126	0.5046458487	0.9999537652	1.0506525768
-1.161430603	0.8783984966	0.9999537652	1.3961707382
2.3326360255	0.4521370209	0.9999537652	-1.316866097
2.2674233494	0.1898486642	0.9999537652	1.0948982875
-1.0121266589	0.9503324809	0.9999537652	1.0097969468
-4.3217318489	0.1708182022	0.9999537652	-1.158117308
1.0438066479	0.8664921502	0.9999537652	-1.2028165095
-1.0195651651	0.9279617129	0.9999537652	-1.2738053713
1.5659221626	0.3961885671	0.9999537652	1.314719452
1.058142182	0.7209152455	0.9999537652	-1.0776164224
-17.0527724669	0.1063548552	0.9999537652	-1.3590484476
-2.1326506013	0.4631645182	0.9999537652	-1.0338466263
0	0	0	4.9561842702
1.0168611786	0.93290619	0.9999537652	-1.1013467357
22.1075168104	0.2295008652	0.9999537652	-1.9620219724
-1.7176229898	0.5174147387	0.9999537652	3.4611114442
-1.2649652647	0.4310966757	0.9999537652	-1.4932385963
1.0367300283	0.7764224752	0.9999537652	1.0282268827
11.5766527773	0.3453843422	0.9999537652	-1.4286012664
-1.0937621337	0.4891450831	0.9999537652	-1.2078102484
-1.0299628598	0.8629764745	0.9999537652	1.3082566609
1.0080937575	0.9697128826	0.9999537652	1.1600397245
-1.0754922775	0.8699612824	0.9999537652	1.2696468831

-1.0663320497	0.8897806168	0.9999537652	-1.2235634669
-1.2438524431	0.5002728272	0.9999537652	-1.3512838194
-1.3047656289	0.4847595586	0.9999537652	1.5875171767
1.4218452648	0.1483491389	0.9999537652	-1.0152102617
0	0	0	2.9985540909
18.5983228161	0.2588803694	0.9999537652	-1.3445118518
-1.8012851692	0.2031318419	0.9999537652	-1.2595893966
1.0900268301	0.7331241983	0.9999537652	1.0557461212
-1.3451228325	0.3171990737	0.9999537652	1.0091017354
-1.211949769	0.6069438586	0.9999537652	1.085874827
-1.468246697	0.2989237541	0.9999537652	1.7249496821
-1.6322470252	0.3724814928	0.9999537652	-1.9313985451
2.2885357703	0.2771135587	0.9999537652	1.3106201703
1.118417687	0.7504042898	0.9999537652	-1.1199226122
-1.1060323543	0.6099505415	0.9999537652	-1.811449099
1.2531151052	0.1265882593	0.9999537652	-1.2157291683
13.3324809638	0.3193505869	0.9999537652	2.6452318482
1.2393646862	0.2826186877	0.9999537652	1.0792247985
1.2565549207	0.2408121205	0.9999537652	-1.2193996967
-1.2922465961	0.3812300655	0.9999537652	-1.1845953322
1.1224466517	0.5808666328	0.9999537652	-1.0001468923
-1.6006511709	0.3244533117	0.9999537652	-1.3860350062
1.3025788871	0.7330718572	0.9999537652	1.4012418682
-1.2054828867	0.3685891572	0.9999537652	1.4459939108
-1.1778378824	0.6248772099	0.9999537652	-1.5801387112
-6.5296045739	0.1275936128	0.9999537652	-1.4371079004
0	0	0	-1.1124408841
-1.884506258	0.3396400481	0.9999537652	-1.7284443593
-1.1930553883	0.3393180569	0.9999537652	1.0254753524
-1.1594602839	0.8874951963	0.9999537652	1.6659944801
0	0	0	2.996475627
-1.014102456	0.9466434524	0.9999537652	-1.0893504391
4.5505976667	0.5557790267	0.9999537652	3.3469034948
1.4796250031	0.6369429881	0.9999537652	1.4118490176
1.0643403978	0.8614155696	0.9999537652	-1.0805393413
-1.1890288123	0.2979574003	0.9999537652	-1.1160985872
-1.1317263623	0.7724208764	0.9999537652	1.4601248216
5.0059881963	0.1255295171	0.9999537652	-1.3850093291
-1.3236385671	0.2274943763	0.9999537652	-1.1393812462
23.8818846228	0.2166969769	0.9999537652	-1.0450563444
-1.1709862367	0.5865384144	0.9999537652	-1.1051436399
2.7933974408	0.6948713406	0.9999537652	0
-1.1376883721	0.821009146	0.9999537652	1.0061446883
-1.1638905218	0.495493245	0.9999537652	1.0809047125
-1.1960205762	0.6317515885	0.9999537652	-1.2345870015
-1.4154799939	0.2528628	0.9999537652	-1.3519777413
1.1020620886	0.7426996882	0.9999537652	1.354993556
-1.1966477219	0.3992123191	0.9999537652	1.1559025492
-1.8175467381	0.1636194653	0.9999537652	-1.0287589877

-1.4228647442	0.4770381833	0.9999537652	-1.3267336889
1.2603303963	0.3595705509	0.9999537652	-1.3585846682
130.8067674395	0.0542346844	0.9999537652	1.6972074939
-1.0218604174	0.8501676115	0.9999537652	1.1071593411
2.0877130777	0.5308629928	0.9999537652	-1.6574346079
1.0898819289	0.7438062779	0.9999537652	-1.0293650637
0	0	0	2.996475627
1.1594390706	0.4799729835	0.9999537652	-1.1888758789
1.6018881045	0.6925261145	0.9999537652	4.9567375629
-1.4137205962	0.384366144	0.9999537652	1.1975499493
-1.0604282475	0.6707851286	0.9999537652	-1.2925926736
-1.0731789005	0.5605700415	0.9999537652	-1.17764975
1.8986710575	0.1957800346	0.9999537652	1.0261034196
0	0	0	2.996475627
1.1991423283	0.6692854827	0.9999537652	1.0795303638
-1.069299774	0.7851731037	0.9999537652	-1.2190727012
9.8205505243	0.3816603866	0.9999537652	6.908559683
1.4851550397	0.2509385523	0.9999537652	-1.1787998522
1.0202390615	0.9535426111	0.9999537652	-1.3563068967
1.0953279185	0.6561554564	0.9999537652	1.0898749356
1.1065498649	0.5965777103	0.9999537652	-1.4429187773
1.1412291911	0.5355483972	0.9999537652	1.1668477171
-1.0117579392	0.9514941669	0.9999537652	1.0132655071
1.1041551707	0.7432042715	0.9999537652	-1.1141218681
9.8205505243	0.3816729323	0.9999537652	1.1330498456
-6.9736190158	0.2250321147	0.9999537652	4.9567375629
0	0	0	1.0666207935
2.7933974408	0.6948713406	0.9999537652	-5.3589986948
2.7933974408	0.6948713406	0.9999537652	-2.0477227478
1.0011405924	0.9955618512	0.9999537652	1.1656591249
-1.0538156238	0.6787151398	0.9999537652	-1.2151614857
1.1146826657	0.6206701915	0.9999537652	-1.0985877053
-1.3101654085	0.1512173926	0.9999537652	-1.0148378174
0	0	0	1.2408886006
-3.1179374904	0.2575217728	0.9999537652	2.4147444764
0	0	0	2.996475627
9.8205505243	0.3816873261	0.9999537652	10.818785458
-1.4346235745	0.5723253943	0.9999537652	-1.4943570592
1.1162834585	0.9279619253	0.9999537652	0
-1.1330468105	0.3518649723	0.9999537652	-1.1613274798
-1.0001811849	0.9989670012	0.9999537652	-1.0793454031
1.3366597161	0.0983213379	0.9999537652	-1.0888440415
1.1366182375	0.4864976137	0.9999537652	-1.3416467371
1.3920227785	0.3377244458	0.9999537652	1.1178573431
-1.5101047647	0.0626453437	0.9999537652	1.2449438635
2.7933974408	0.6948713406	0.9999537652	0
0	0	0	-2.0477227478
-1.7495015986	0.2655124854	0.9999537652	-1.0247784186
-1.5783001323	0.5359474909	0.9999537652	1.7460170926

-1.5351801594	0.3581393837	0.9999537652	-1.160895632
9.8205505243	0.3819497662	0.9999537652	-1.1242401582
-1.7293496257	0.1449292804	0.9999537652	-1.2089304571
2.7933974408	0.6948713406	0.9999537652	-1.3598304575
4.5505976667	0.5557790267	0.9999537652	5.0961124596
1.1246813911	0.8864180307	0.9999537652	2.9979638378
1.0428711823	0.8923404686	0.9999537652	-1.24274376
1.1091496219	0.5107663111	0.9999537652	-1.0344127523
-1.0831716006	0.7284964134	0.9999537652	1.1968509925
-1.1109620866	0.6210817043	0.9999537652	1.0525092926
1.2949597239	0.5716948647	0.9999537652	-1.5882223759
-1.0065930739	0.9741559938	0.9999537652	-1.26094063
0	0	0	1.6646597813
1.3180723625	0.5517009827	0.9999537652	-1.7185362769
1.9015109615	0.6235981582	0.9999537652	0
-1.3249168377	0.4171919463	0.9999537652	1.1293016654
-1.0057145646	0.9666187499	0.9999537652	1.0145992937
1.8771369955	0.1724053618	0.9999537652	1.3807731526
-1.0339643056	0.8538999336	0.9999537652	1.1656438395
1.577312308	0.1838344341	0.9999537652	-1.4394758639
-1.043681335	0.7500802766	0.9999537652	-1.0419488348
-1.093713204	0.4448763622	0.9999537652	-1.1140643091
1.5471365957	0.2334479395	0.9999537652	1.1063191396
-1.1603883193	0.431085157	0.9999537652	1.3591083287
2.7933974408	0.6948713406	0.9999537652	0
1.0969178476	0.6111636108	0.9999537652	1.3784304799
-1.108777061	0.8167940475	0.9999537652	1.2973624736
-1.0578614036	0.9471368289	0.9999537652	-2.5802143578
-1.2729458826	0.5000478898	0.9999537652	-1.1058541929
0	0	0	-1.8582093966
1.179144353	0.46942354	0.9999537652	1.2997451959
-1.3702762656	0.7548902156	0.9999537652	0
1.8979370239	0.310236851	0.9999537652	1.0304724424
1.0408170904	0.934351684	0.9999537652	1.3367792425
1.787649606	0.055313161	0.9999537652	1.1341696424
-1.1006929295	0.6262047786	0.9999537652	-1.180850851
-1.1779033249	0.3662004469	0.9999537652	-1.2007516509
-1.0620447609	0.8977311612	0.9999537652	1.1175283869
-1.1866782852	0.3418936627	0.9999537652	-1.035551104
-1.0604409414	0.812932722	0.9999537652	1.1873582987
1.0025315868	0.9858919404	0.9999537652	1.3325167164
1.2844507971	0.5727032843	0.9999537652	-1.5377004024
-1.1268404068	0.515711227	0.9999537652	1.0101204026
-1.0942202955	0.516972304	0.9999537652	1.0555222416
1.6258890912	0.2063538032	0.9999537652	-1.1100608804
18.5983228161	0.2585399858	0.9999537652	1.811806509
1.0885861526	0.8530176513	0.9999537652	-1.1401632482
-5.3600263521	0.1948035092	0.9999537652	1.1353985409
1.3594099105	0.7968739818	0.9999537652	-3.3853891979

6.3075231393	0.4852128108	0.9999537652	-1.6597328868
-1.0233993854	0.8621432498	0.9999537652	-1.0017388608
1.5752480734	0.1685669564	0.9999537652	1.1790408691
0	0	0	14.7475552392
1.4188013253	0.0515677387	0.9999537652	-1.0750340929
13.3324809638	0.3193419097	0.9999537652	-3.6852522302
-1.0470886977	0.831074909	0.9999537652	1.1881344766
-2.8969127737	0.3395250449	0.9999537652	-1.1883486959
-1.186831757	0.5027649271	0.9999537652	-1.0430599337
1.0047906714	0.9792737114	0.9999537652	1.2633335371
1.1091391926	0.45096475	0.9999537652	1.0320865728
-1.1699825231	0.4717546029	0.9999537652	-1.3261010268
1.5156531293	0.6413221364	0.9999537652	1.0918431289
1.0003373048	0.9995270402	0.9999537652	-1.1354697632
1.0636295337	0.8133816572	0.9999537652	-1.006844365
-1.081586986	0.5320533455	0.9999537652	1.048929634
-1.105155594	0.6264372286	0.9999537652	-1.1521768735
1.1130728543	0.6344712728	0.9999537652	1.1376625313
-1.4657664736	0.083114812	0.9999537652	-1.1770987899
1.6367259986	0.0374841545	0.9999537652	1.3059307161
1.4334654888	0.4505777825	0.9999537652	1.345112118
1.6009138193	0.1460147534	0.9999537652	-1.2057336972
-1.0228649836	0.9003864998	0.9999537652	1.1613307944
1.3020070582	0.7501040856	0.9999537652	-1.19176092
-1.26535817	0.4535473157	0.9999537652	1.0592249325
1.2710188429	0.4230682784	0.9999537652	1.0492733557
1.6154121672	0.0004657	0.9999537652	1.7735800293
0	0	0	2.996475627
-1.1056546381	0.4934039425	0.9999537652	1.010776676
1.0160348504	0.9553901325	0.9999537652	1.3010502522
1.3275131029	0.5399991186	0.9999537652	1.0351854796
-1.0637253167	0.7677919161	0.9999537652	-1.1386820616
1.1162834585	0.9279861599	0.9999537652	5.3812467488
1.1661684552	0.6964289986	0.9999537652	1.2503386741
1.068067219	0.7334770771	0.9999537652	-1.159070428
-1.0206955143	0.9094414725	0.9999537652	-1.1553282619
1.9235077106	0.2042999889	0.9999537652	-1.2032064744
2.7933974408	0.6948713406	0.9999537652	8.9512754559
-1.0286637554	0.8445033628	0.9999537652	-1.1861786165
1.0382033736	0.8833349349	0.9999537652	1.2304326688
2.7762553073	0.0445748132	0.9999537652	-1.1929975499
-1.1111932047	0.5774848728	0.9999537652	-1.2231115995
-1.5077326167	0.3876487356	0.9999537652	-1.516837928
-1.4432991877	0.0783940072	0.9999537652	1.3363618797
-1.0829983138	0.715997839	0.9999537652	1.0640214169
-1.3320202714	0.1954118996	0.9999537652	1.0953594386
-1.0136562041	0.9396461931	0.9999537652	1.0088289106
1.2015200298	0.3685698079	0.9999537652	-1.4257010169
1.9936126395	0.5572200094	0.9999537652	-1.1267047693

-2.5875654693	0.0710707148	0.9999537652	1.0593796655
1.5191296774	0.3035382768	0.9999537652	-1.1679838252
1.0582401461	0.8873948484	0.9999537652	-1.5468046935
1.1029763937	0.6445973007	0.9999537652	-1.1028656347
1.8651774762	0.0316594066	0.9999537652	-1.0931641773
1.4630658602	0.5984399874	0.9999537652	-1.6434190706
1.4953186627	0.4274120244	0.9999537652	1.1445467728
1.0982265381	0.4707024162	0.9999537652	1.3768668301
-1.0463589702	0.8977149277	0.9999537652	1.2586681921
-1.1075405885	0.6839215187	0.9999537652	1.1422341192
-1.0578962542	0.8600898611	0.9999537652	-1.2064904094
1.1352054359	0.4792593869	0.9999537652	1.3679992151
2.0885615936	0.5186626856	0.9999537652	5.3165033614
3.3071401144	0.3245208179	0.9999537652	1.2400291296
20.3530564257	0.2429739469	0.9999537652	-16.5082395846
0	0	0	4.9527501949
-1.0412515922	0.8307617642	0.9999537652	1.0937406329
1.0167791595	0.9175341461	0.9999537652	1.0849073187
1.0833826809	0.6390380444	0.9999537652	-1.0859817795
-1.0714934623	0.645128514	0.9999537652	1.0974142947
-1.460992866	0.0384207436	0.9999537652	-1.1151679694
1.481754914	0.1795419133	0.9999537652	1.3160700047
-1.1167430974	0.382155197	0.9999537652	-1.0473051514
1.308402725	0.1300234957	0.9999537652	1.2814245214
-1.2307312453	0.7053299484	0.9999537652	-1.1079134319
-1.6560320481	0.3939888644	0.9999537652	8.6426265779
-1.0728063956	0.6522624829	0.9999537652	1.1137553525
1.7090181294	0.2900528811	0.9999537652	1.0051882092
2.7933974408	0.6948713406	0.9999537652	-1.1541475958
9.8205505243	0.3822070427	0.9999537652	2.996475627
8.064174035	0.4193478245	0.9999537652	0
37.9506346044	0.1516488587	0.9999537652	-1.321583129
-1.1204523193	0.4985095794	0.9999537652	-1.1264119773
-1.1457555741	0.9106046164	0.9999537652	-9.7542681481
249.5637219222	0.028511351	0.9999537652	-2.5964422649
-1.1375146148	0.5318922948	0.9999537652	1.2107812615
-1.3706824165	0.637970541	0.9999537652	1.0287051083
-1.3385478755	0.7110003247	0.9999537652	-1.1246693829
2.1598265017	0.1702870737	0.9999537652	-1.1757242868
0	0	0	1.0667457716
1.1875125807	0.1336346302	0.9999537652	1.2155856694
2.6862171381	0.2568232491	0.9999537652	1.8624192744
0	0	0	0
-1.1998967319	0.5490387246	0.9999537652	-1.4586789856
1.166715855	0.55486977	0.9999537652	1.1432838632
1.1550777531	0.7907732768	0.9999537652	2.6071338396
1.2379410704	0.5719919778	0.9999537652	1.1689131253
-1.5875118025	0.7201536922	0.9999537652	2.9985540909
2.7933974408	0.6948713406	0.9999537652	0

1.2145433703	0.3691555874	0.9999537652	-1.0176923122
-1.1739092263	0.4658680995	0.9999537652	-1.3510363071
1.0900370194	0.5290219369	0.9999537652	1.0885681808
-1.3306046939	0.4984053163	0.9999537652	1.0165393545
1.1839179914	0.4719483696	0.9999537652	-1.04004813
-1.3039767968	0.7557538898	0.9999537652	-2.5918501576
1.1318814021	0.6903968921	0.9999537652	-1.688975049
-1.4878781745	0.166091953	0.9999537652	-1.2673951642
1.0302248064	0.8689379041	0.9999537652	-1.0881380904
-1.253858095	0.4634060959	0.9999537652	-1.0337605262
1.2911522073	0.1920092053	0.9999537652	1.3705116092
-1.0391283302	0.9365673212	0.9999537652	-1.077387845
-1.021220323	0.9608697004	0.9999537652	-1.1994448092
-1.5256790713	0.0836425367	0.9999537652	1.28358692
1.0513272264	0.7063721572	0.9999537652	1.0460781052
-1.0215098318	0.8801195233	0.9999537652	1.2327952832
1.298973715	0.2575337718	0.9999537652	-1.0381339434
1.366474448	0.1009585378	0.9999537652	-1.0725450289
-1.0357309229	0.8205681392	0.9999537652	1.0625298633
1.5915109622	0.068814288	0.9999537652	-1.1260856715
1.162565591	0.5672224773	0.9999537652	-1.2450889704
1.2117865278	0.1122545043	0.9999537652	1.8213282266
-1.1171876296	0.7058825058	0.9999537652	-1.3241170447
1.2173850991	0.7722437285	0.9999537652	-2.0184868898
-1.2683659373	0.2106354716	0.9999537652	-1.0855365705
2.1097000422	0.1161793699	0.9999537652	-1.7088382807
-1.1231451824	0.8623471835	0.9999537652	-1.3967867798
-1.2830789759	0.7153793686	0.9999537652	1.0737757585
4.5505976667	0.5557790267	0.9999537652	4.048242769
16.296263041	0.2962447536	0.9999537652	1.0301713759
-1.8932786066	0.007712	0.9999537652	1.1648018121
1.5137073316	0.6728341611	0.9999537652	1.1969806455
1.1018875973	0.6112305696	0.9999537652	-1.1018557963
-1.1471330499	0.2378934886	0.9999537652	-1.1474313514
-1.4615349616	0.1225041063	0.9999537652	1.102047612
-1.1265831123	0.5968698212	0.9999537652	-1.342678224
1.1463819113	0.4860299451	0.9999537652	1.0020840939
0	0	0	8.8639046058
1.0535076598	0.7744756521	0.9999537652	1.2081305367
-1.091840392	0.6915627046	0.9999537652	-1.4874014749
1.3041775569	0.7306542437	0.9999537652	-2.123711531
-1.0806523375	0.7074681211	0.9999537652	-1.1907578771
-1.0545652153	0.8978771021	0.9999537652	-1.2129588215
1.4043101181	0.5331759938	0.9999537652	-1.4649193108
-1.0846660344	0.6678563399	0.9999537652	1.0481972831
4.5505976667	0.5557790267	0.9999537652	2.8664963163
1.0916880155	0.7561540434	0.9999537652	-1.3295338654
-1.0872008538	0.8714047984	0.9999537652	-1.3338035496
-1.6994504307	0.5420730382	0.9999537652	-1.156904861

1.0606444597	0.70348711	0.9999537652	1.035991718
-1.1453192235	0.9085486745	0.9999537652	3.3469034948
-1.0316180608	0.9045644615	0.9999537652	-1.2528263223
1.0546059095	0.8686883958	0.9999537652	-1.2870901931
-1.0519015254	0.8269683051	0.9999537652	1.0295092211
2.7933974408	0.6948713406	0.9999537652	-5.3583704783
1.2557228731	0.3836584167	0.9999537652	1.2551410583
11.5766527773	0.345396292	0.9999537652	1.1693470523
-1.369198268	0.7571957905	0.9999537652	1.6086304467
1.3923627432	0.6684487346	0.9999537652	1.4776337576
3.4895363975	0.0659379287	0.9999537652	1.1779489798
1.3251114985	0.2629720175	0.9999537652	1.1404164435
1.1024976244	0.7133188087	0.9999537652	1.2660124944
-1.1165838431	0.3768395667	0.9999537652	-1.1235660869
-1.8551455303	0.0891583013	0.9999537652	1.031274069
4.5505976667	0.5557790267	0.9999537652	1.2394611522
-1.6004705264	0.1875727412	0.9999537652	1.0240173192
1.1041313795	0.5540151932	0.9999537652	1.0166388614
-1.1394705898	0.6493510759	0.9999537652	-1.0756807151
1.3733524417	0.1191731845	0.9999537652	-1.1943457761
-1.445711632	0.1134836418	0.9999537652	-1.1738169746
-7.0431967789	0.0842904904	0.9999537652	1.7206366659
-1.1951129036	0.2862695494	0.9999537652	-1.1089455233
1.0434932533	0.8399646648	0.9999537652	-1.0643543443
1.5700882646	0.1942977849	0.9999537652	1.4377975579
1.1658912736	0.3767258147	0.9999537652	-1.0790187384
-2.5838710423	0.5185078317	0.9999537652	18.6336779883
1.0325262642	0.9172727503	0.9999537652	-1.1740530274
-3.0678798337	0.0757237567	0.9999537652	-1.5197744168
-1.042035903	0.9013750862	0.9999537652	1.263050618
-1.0612709526	0.8294984142	0.9999537652	-1.2941225636
1.0420401189	0.8310753722	0.9999537652	-1.1285233218
1.1162834585	0.9279846283	0.9999537652	-1.859280336
-1.0302089517	0.9260647018	0.9999537652	-1.0486995939
-1.3172130908	0.620143246	0.9999537652	-1.7412494823
-1.1453192235	0.9085235142	0.9999537652	1.2707518347
1.2702608995	0.2018156466	0.9999537652	-1.1257580444
-1.2789806116	0.7307715535	0.9999537652	-1.595141759
-1.1261857203	0.5400832343	0.9999537652	1.0514331165
-1.2698571935	0.3534367721	0.9999537652	-1.1620773296
20.3709255077	0.2404319414	0.9999537652	-1.1060913839
-1.1384613128	0.5705852226	0.9999537652	-1.3661551712
-1.0243262586	0.8600566589	0.9999537652	-1.1107166296
0	0	0	2.996475627
-1.0833231221	0.9095558675	0.9999537652	-1.1625772141
1.1366232902	0.5726422419	0.9999537652	-1.1547175111
1.4621934254	0.3643637361	0.9999537652	-1.2334713253
1.045679119	0.8441753272	0.9999537652	-1.2349830164
2.1113088034	0.3083341341	0.9999537652	1.7344154021

-1.5907083418	0.1219693751	0.9999537652	-1.8457250557
-1.2503888487	0.7722447878	0.9999537652	-1.4900431113
6.3075231393	0.4852033336	0.9999537652	2.2924151284
-1.7570926671	0.2361343164	0.9999537652	-1.3862454905
-1.4608166475	0.1148087149	0.9999537652	-2.0291577155
-1.0073447772	0.9625281972	0.9999537652	1.0017775406
-1.1109153622	0.4289138107	0.9999537652	-1.207194092
-1.1604800797	0.8777239625	0.9999537652	1.7013955871
1.2835061594	0.6333964541	0.9999537652	-1.2687078362
-1.4235027739	0.0357163621	0.9999537652	-1.3455779303
-1.118569044	0.3752532761	0.9999537652	-1.0878914258
18.9318466825	0.2781654697	0.9999537652	1.1932077424
1.4644578187	0.2488858225	0.9999537652	-1.1851156955
1.284971304	0.2932571328	0.9999537652	-1.2613335294
1.2059010171	0.7416994671	0.9999537652	1.2208336453
2.1642013532	0.4347886977	0.9999537652	1.3742147816
1.305406083	0.3160370004	0.9999537652	-1.1874311862
1.2135155632	0.442327709	0.9999537652	1.31124185
-1.3322384505	0.2752819251	0.9999537652	1.3295579631
1.1126605832	0.6639006367	0.9999537652	-1.290457686
1.210568601	0.2896311487	0.9999537652	1.0852405163
-1.0840753248	0.6981678725	0.9999537652	-1.2937795303
2.7933974408	0.6948713406	0.9999537652	0
1.1337133962	0.5334844757	0.9999537652	-1.4329560052
1.9460988524	0.3011783504	0.9999537652	-1.1661979027
1.1770305358	0.1437481902	0.9999537652	1.0156602461
1.1358528676	0.4017677189	0.9999537652	1.0181585099
-1.1821041638	0.3154368339	0.9999537652	2.1226998443
-1.0498296191	0.7186400677	0.9999537652	-1.086234982
-1.0465508746	0.8324754989	0.9999537652	-1.2306736155
1.4223548157	0.0758640097	0.9999537652	-1.1955589914
-1.0257428536	0.8855254371	0.9999537652	-1.1263156728
-1.5881166222	0.7196016486	0.9999537652	0
0	0	0	4.9527501949
-1.0755225977	0.863470302	0.9999537652	1.1270777255
1.222021874	0.3218208175	0.9999537652	-1.2953708004
1.4355993469	0.4794033111	0.9999537652	-1.4089567536
2.5098097939	0.0673873726	0.9999537652	1.3211861918
-1.2541628693	0.2603365557	0.9999537652	-1.3838679458
-1.137995001	0.7709791043	0.9999537652	-1.3106989976
-1.0818260504	0.7401925756	0.9999537652	-1.2904701699
-1.0768309153	0.6486691656	0.9999537652	-1.1431252668
-1.1093901949	0.4517532237	0.9999537652	-1.3209835976
-1.3396553218	0.2651248864	0.9999537652	-1.326290048
1.0915569585	0.5745927661	0.9999537652	1.0981070904
1.1033726586	0.5796693699	0.9999537652	-1.1681120152
1.4317593632	0.4965345408	0.9999537652	-1.4608953882
-1.2808524125	0.3359447726	0.9999537652	-1.3153546415
-1.0423082512	0.9232268939	0.9999537652	-1.4492133135

1.131572734	0.675060469	0.9999537652	-1.255807597
-1.05521984	0.7934638153	0.9999537652	-1.0818027296
-1.1640062011	0.3865162115	0.9999537652	-1.068716784
1.0816388297	0.6814303659	0.9999537652	-1.1903179823
-1.1170938003	0.8003732321	0.9999537652	1.0178100145
1.1320671704	0.6040319583	0.9999537652	1.0666018029
2.7933974408	0.6948713406	0.9999537652	0
1.4071828944	0.4614613059	0.9999537652	-1.2018945272
-1.1453192235	0.9084983564	0.9999537652	0
1.1119690798	0.5609410252	0.9999537652	1.0829165078
1.1651448872	0.4540678169	0.9999537652	1.0204043333
1.0521689726	0.736278478	0.9999537652	1.3437074937
1.0432030485	0.8634497154	0.9999537652	-1.0967444021
4.5544554512	0.569123417	0.9999537652	2.9985540909
-3.424263505	0.0465987074	0.9999537652	-2.3110607932
1.0913731451	0.6764752229	0.9999537652	-1.0015868663
-1.3927039579	0.0132439592	0.9999537652	1.0787984963
1.2682896538	0.2019142095	0.9999537652	1.1115301823
-1.6410956547	0.1571233863	0.9999537652	-1.366295336
1.4672515657	0.2995099256	0.9999537652	-1.0493288913
-1.2698360685	0.241014012	0.9999537652	1.0561548556
1.025986051	0.8973852058	0.9999537652	1.1142755139
1.1218124863	0.6562977421	0.9999537652	1.117205035
1.2473203314	0.4642442354	0.9999537652	-1.0192052879
-1.1064153886	0.5385122363	0.9999537652	1.2251928726
1.0109738374	0.9472051686	0.9999537652	1.2678122748
-1.5191908506	0.3897159669	0.9999537652	-1.2471775178
-1.865641096	0.3849939666	0.9999537652	1.4370974628
-1.5088679904	0.2014484135	0.9999537652	1.1240370032
-1.2250402764	0.2986874549	0.9999537652	1.0519472581
6.3075231393	0.4851938636	0.9999537652	0
0	0	0	1.9429993325
1.6748770634	0.0234879429	0.9999537652	1.2183004394
-2.0616778935	0.3664784239	0.9999537652	-1.5929518064
1.0075604803	0.9674390732	0.9999537652	1.1540236101
1.0904523195	0.6563168241	0.9999537652	1.6664365568
1.0947945491	0.7484234679	0.9999537652	1.3488251881
-1.1378168294	0.3699761982	0.9999537652	-1.0084322429
-1.6525426056	0.0104542961	0.9999537652	1.0551841528
1.0956548455	0.5800393441	0.9999537652	-1.1268760924
9.8205505243	0.3829068144	0.9999537652	-5.3589986948
-1.2287947425	0.106419117	0.9999537652	1.0216749559
2.7957660672	0.7055093216	0.9999537652	18.6465640159
1.037646073	0.871825649	0.9999537652	1.2494719046
-1.6547859728	0.428407077	0.9999537652	-1.0620681059
-1.0427054911	0.9119865299	0.9999537652	1.1845895774
-1.3761467853	0.3162957501	0.9999537652	1.0057495425
-1.1435642915	0.3094494395	0.9999537652	-1.1379883492
-1.3697544738	0.7454660002	0.9999537652	1.9418822056

1.0577003822	0.6572666087	0.9999537652	1.0240003973
1.5957586408	0.29268491	0.9999537652	-1.0537536839
1.1827519787	0.7608843561	0.9999537652	-1.474672455
-1.0158202826	0.9718679118	0.9999537652	1.2914350297
1.452030427	0.0412905405	0.9999537652	1.3304209264
-1.2863564238	0.4587775529	0.9999537652	1.1673138225
1.078010753	0.6883812638	0.9999537652	1.1554331924
-1.2824570106	0.1569812672	0.9999537652	1.1309397348
1.0570433333	0.8666098854	0.9999537652	-1.0530204883
-1.001808101	0.9945267834	0.9999537652	-1.0992927125
1.0534549393	0.7850132192	0.9999537652	1.3902250691
-1.1471526808	0.4689631683	0.9999537652	-1.0314138285
2.1104313265	0.1376727097	0.9999537652	1.0108534739
4.5544554512	0.5691214234	0.9999537652	-1.8579915692
1.1074829774	0.5320456439	0.9999537652	1.2444713338
1.1764388862	0.5267520452	0.9999537652	-1.0568255457
1.1398761875	0.2929886175	0.9999537652	-1.0096106204
1.2498624507	0.4695516531	0.9999537652	1.3852494711
-1.1903487778	0.302195048	0.9999537652	1.2857916302
-1.1619717093	0.8743568181	0.9999537652	-1.0324473817
9.8291821523	0.3810532719	0.9999537652	-3.8929589845
-1.0852237286	0.6950456669	0.9999537652	1.1941255412
0	0	0	6.908559683
1.793573223	0.2310303635	0.9999537652	-1.5747310857
-1.0032978663	0.9886196825	0.9999537652	-1.7730500579
1.434690516	0.1880357941	0.9999537652	-1.0840269184
2.1419513341	0.0103498366	0.9999537652	1.2392317369
-1.4279215627	0.3036384895	0.9999537652	-1.0539750881
0	0	0	2.996475627
17.3472938331	0.297593761	0.9999537652	10.8337816869
1.445516321	0.0879057859	0.9999537652	-1.0578711082
-1.1343868507	0.6436410492	0.9999537652	1.1115686962
-1.1658955302	0.700708192	0.9999537652	1.2112783616
1.0848793797	0.6628822603	0.9999537652	1.3174707932
-1.206383623	0.679279939	0.9999537652	2.4850917429
1.0933195059	0.780591948	0.9999537652	1.3857265732
-1.2088847843	0.6168540632	0.9999537652	1.29058679
6.3075231393	0.485184401	0.9999537652	2.9988886918
2.7933974408	0.6948713406	0.9999537652	0
2.5603529545	0.2533448675	0.9999537652	-3.2960765574
-1.1273264967	0.6734540551	0.9999537652	1.5152163339
-1.0552900301	0.8299308523	0.9999537652	-1.2669986972
1.117210085	0.56850067	0.9999537652	1.4991880385
1.9059311107	0.4748393818	0.9999537652	1.2775595037
9.8205505243	0.3834204237	0.9999537652	0
1.431746495	0.2482106738	0.9999537652	-1.1855556174
2.382293359	0.0341921925	0.9999537652	1.1606269066
1.0349547516	0.9587074483	0.9999537652	-1.0025721281
1.0120824699	0.9497876596	0.9999537652	-1.1650277482

0	0	0	15.9061931258
1.0980537363	0.7761736383	0.9999537652	1.1486103331
1.0084917111	0.9588856549	0.9999537652	1.020535448
1.0507500623	0.8518208642	0.9999537652	1.3058568606
-1.1971512532	0.3909110707	0.9999537652	1.089320051
-1.5405413639	0.0235519751	0.9999537652	1.0777048372
1.0715941098	0.744095118	0.9999537652	-1.0357292731
-1.1599754231	0.8809246774	0.9999537652	-1.5335264323
1.0209446956	0.9069626208	0.9999537652	-1.057812912
-1.2124534043	0.6001277472	0.9999537652	-1.4326822578
-1.0183766977	0.9482440068	0.9999537652	1.2899072685
-1.8806546573	0.2161173175	0.9999537652	-1.0494562787
1.0358561766	0.966566142	0.9999537652	-1.0162949722
-1.1000361821	0.5938461815	0.9999537652	1.0570774796
-2.4156082583	0.3145552024	0.9999537652	0
-1.6167995859	0.0100472812	0.9999537652	1.1469168166
-1.0141188946	0.905982832	0.9999537652	1.0483600866
-1.0275168639	0.9170862329	0.9999537652	1.1055823132
-1.0342452756	0.9023586959	0.9999537652	-1.1970335872
1.2221260278	0.695659622	0.9999537652	-1.1212533501
0	0	0	2.9985540909
1.1579497658	0.5432283642	0.9999537652	-1.2315692271
1.5954008	0.3509687722	0.9999537652	-1.1700467391
-1.0831077593	0.7647360585	0.9999537652	-1.1082536133
1.0174830237	0.905877466	0.9999537652	1.0234883342
-1.2243823421	0.0903323627	0.9999537652	-1.1496406173
-1.668044468	0.6115623291	0.9999537652	-1.7689121852
-1.1717020419	0.7816799213	0.9999537652	1.2025112422
-1.0432294284	0.942614894	0.9999537652	1.6428693505
1.2058113856	0.315642274	0.9999537652	1.1271831837
1.7832225176	0.3957663894	0.9999537652	-137.3174062836
-1.8162185293	0.3282390241	0.9999537652	2.1803770037
-1.0579303901	0.6732502445	0.9999537652	1.1009642278
24.3870295947	0.2290259672	0.9999537652	0
1.4067138366	0.1158823064	0.9999537652	1.2561081415
4.5505976667	0.5557790267	0.9999537652	1.7534505922
55.4857722906	0.1140953786	0.9999537652	1.2076073882
1.1555817374	0.5110236142	0.9999537652	1.1042430807
1.2247772049	0.4431972123	0.9999537652	-1.259435987
-1.3386471893	0.1200329769	0.9999537652	-1.3388648871
4.5544554512	0.5691194299	0.9999537652	0
-1.0659183645	0.7277037495	0.9999537652	1.4828053352
1.2480005421	0.6196435355	0.9999537652	-1.2127671684
1.1387802751	0.4553588377	0.9999537652	1.2510988699
1.0872153021	0.7043579325	0.9999537652	1.2281780708
1.2741030728	0.8382550595	0.9999537652	-4.2406870258
1.0039655521	0.9837517676	0.9999537652	1.5304376115
1.1874655834	0.6879300426	0.9999537652	1.3515664869
-1.1453192235	0.9084480465	0.9999537652	0

-1.3342296605	0.4152711407	0.9999537652	1.4245270644
1.1951212749	0.3946343301	0.9999537652	-1.1844520008
0	0	0	4.9527501949
1.0041643949	0.9886306612	0.9999537652	1.0799804531
-1.0200823636	0.9202562835	0.9999537652	1.5712644739
-1.030324962	0.920956554	0.9999537652	-1.3574022302
4.5505976667	0.5557790267	0.9999537652	-3.384595918
-1.5512850017	0.2538545585	0.9999537652	1.0957553385
1.3129421727	0.1997029771	0.9999537652	1.3190749324
1.0364864658	0.8045989142	0.9999537652	1.1341170545
-1.4830986381	0.507101195	0.9999537652	1.3089286093
1.1791813282	0.7395802764	0.9999537652	1.080055544
-1.2601749205	0.4466249394	0.9999537652	1.2057134772
-12.9852817769	0.1145723811	0.9999537652	18.6336779883
1.3519214764	0.2291674868	0.9999537652	1.2297879018
2.7933974408	0.6948713406	0.9999537652	0
-1.0988764368	0.5557819776	0.9999537652	1.4382201485
-1.1577729869	0.4790174805	0.9999537652	1.185358789
-1.0335865813	0.91920921	0.9999537652	1.2468505536
0	0	0	-1.3592076145
1.2442439145	0.4821989253	0.9999537652	-1.1940834369
-1.0101874558	0.9739407161	0.9999537652	1.1056624323
3.0642477781	0.3208985299	0.9999537652	-1.5086083432
-1.0550219132	0.8424033721	0.9999537652	-1.1531118541
1.3242201799	0.5571784961	0.9999537652	-1.1316682221
1.1490921787	0.544016473	0.9999537652	-1.0638934517
-1.4427833345	0.1596014047	0.9999537652	-1.009168882
1.1899003621	0.334224868	0.9999537652	1.2343873872
3.8622037496	0.619279619	0.9999537652	0
-6.9762758667	0.257129201	0.9999537652	0
1.2368337997	0.1867771456	0.9999537652	1.531463071
-1.0488806479	0.9448345571	0.9999537652	2.0176725665
4.5505976667	0.5557790267	0.9999537652	16.7037438866
1.3553237329	0.4450800821	0.9999537652	1.4085994299
-1.4113237029	0.108512766	0.9999537652	1.24781523
1.3626371084	0.6287186377	0.9999537652	-1.0313954446
4.5505976667	0.5557790267	0.9999537652	-5.3583704783
-3.1203116482	0.2603638239	0.9999537652	16.6921882404
-1.1251425411	0.7582654172	0.9999537652	-1.3544123384
-1.0739359402	0.8138175097	0.9999537652	1.3186573404
0	0	0	2.996475627
4.5505976667	0.5557790267	0.9999537652	1.438718359
-1.107412991	0.6087562491	0.9999537652	1.3203319014
1.6373443238	0.3525638553	0.9999537652	1.1416445229
-1.127938319	0.5493274252	0.9999537652	1.2983882762
0	0	0	12.7732027325
-1.6937362394	0.0394929285	0.9999537652	-1.3823718316
1.0164849475	0.944218865	0.9999537652	1.1720170648
1.043043508	0.8826961489	0.9999537652	1.1816476191

0	0	0	2.996475627
-1.0821575717	0.7610075121	0.9999537652	-1.1122593874
1.1168038901	0.9276262293	0.9999537652	6.9133480437
-2.0284297556	0.0527920993	0.9999537652	1.0340558087
1.1038218769	0.4563641756	0.9999537652	-1.2487588538
-1.9182044808	0.3932826021	0.9999537652	1.089503369
1.0628622838	0.8591795761	0.9999537652	1.0744087558
2.7933974408	0.6948713406	0.9999537652	1.4809662823
-1.7329739058	0.083202991	0.9999537652	-1.0526731925
1.0549469795	0.8470773317	0.9999537652	-1.2223015012
0	0	0	-1.8594983184
-1.2017692044	0.673867422	0.9999537652	1.3615494687
1.1095500384	0.4622691028	0.9999537652	-1.3111951279
1.9475083768	0.253088899	0.9999537652	1.2260009445
-1.1105845616	0.4113894703	0.9999537652	-1.0374441436
-1.2355728683	0.3192775803	0.9999537652	-1.8654886106
-1.4611854053	0.1221374522	0.9999537652	1.0437952688
-1.4700854183	0.2876580917	0.9999537652	-1.078184316
0	0	0	6.908559683
2.7933974408	0.6948713406	0.9999537652	-4.9124967738
-1.1332131568	0.7706845748	0.9999537652	-1.4143284498
0	0	0	1.590997316
-2.5861438062	0.4737924651	0.9999537652	-1.3540113423
8.064174035	0.4193539664	0.9999537652	-2.0479626835
1.1239887064	0.8172081433	0.9999537652	2.0814691733
1.076325972	0.6218672132	0.9999537652	-1.3047161295
-1.0924021824	0.5849504373	0.9999537652	1.2225706684
-1.7140706619	0.5280304655	0.9999537652	1.1108484856
1.1791841738	0.6266625135	0.9999537652	-1.0357036706
1.4049480419	0.0281712698	0.9999537652	1.1500886859
-1.060194812	0.8870212164	0.9999537652	-1.5109779539
1.1221352547	0.9083204107	0.9999537652	1.8731367028
1.4538216685	0.4524110102	0.9999537652	1.0474919068
-1.0299913959	0.8216556834	0.9999537652	-1.2439754529
1.3456038051	0.6620606422	0.9999537652	-1.0745375958
1.3254494764	0.4007581604	0.9999537652	1.7115999035
1.5293196826	0.3631019115	0.9999537652	-1.2255202261
1.6852529786	0.0393589994	0.9999537652	-1.0517316153
1.574033549	0.0988894235	0.9999537652	1.0584205644
1.2679783476	0.583214252	0.9999537652	1.5561795548
1.1647323104	0.6316243108	0.9999537652	-1.247228855
-1.012173367	0.9337569888	0.9999537652	1.1991308885
1.2443382777	0.2032815398	0.9999537652	-1.2682668164
-2.2584811166	0.2304468642	0.9999537652	-1.3904070108
1.1102031215	0.4697882284	0.9999537652	-1.1607173895
1.1819055871	0.2616148325	0.9999537652	1.0084096069
-1.5359824943	0.1723885928	0.9999537652	-1.1720381847
-1.0255285461	0.8505252435	0.9999537652	-1.630840201
1.3988806434	0.009168	0.9999537652	-1.0551735063

-1.0235213228	0.8668677782	0.9999537652	1.1681414929
1.1857677205	0.4890078628	0.9999537652	1.0505029141
2.3346865743	0.4712405372	0.9999537652	1.3417940918
-1.259045832	0.2862559366	0.9999537652	-2.2592526017
1.2966439193	0.4492315966	0.9999537652	-1.4074073802
-1.4749363527	0.0299002497	0.9999537652	1.2055394099
-1.1159649051	0.6176142688	0.9999537652	-1.5774951867
-1.1816462225	0.2130860698	0.9999537652	-1.3006247567
-1.2412985235	0.2067028541	0.9999537652	-1.2416710747
1.0213036022	0.9341192925	0.9999537652	1.1228726229
1.1776698887	0.4463078782	0.9999537652	1.319640004
1.319104928	0.1757534243	0.9999537652	-1.0313678912
-1.2225352262	0.1216022462	0.9999537652	-1.4331020139
1.1268989761	0.2924369112	0.9999537652	1.040921958
1.2484788586	0.2707464202	0.9999537652	-1.0182502969
1.9990319965	0.0371575639	0.9999537652	1.1768538732
1.1242358844	0.8720335654	0.9999537652	-1.1077831536
1.5353839104	0.4124099276	0.9999537652	-1.1277926517
4.5505976667	0.5557790267	0.9999537652	1.41016296
-2.5871290907	0.5000874544	0.9999537652	-1.1566816905
1.3397973953	0.118935355	0.9999537652	1.2727136518
-1.2446995364	0.2015562286	0.9999537652	-1.5807004474
-1.2106520858	0.1763623154	0.9999537652	-1.0142536285
-1.0554656727	0.6836848426	0.9999537652	-1.1560170508
-1.1996181251	0.7035874525	0.9999537652	-1.0295962437
-1.0412679261	0.9263938144	0.9999537652	-1.1530500699
-1.2019245538	0.3474947059	0.9999537652	-1.2953335051
1.702429257	0.00172	0.9999537652	1.933921747
-20.3937810938	0.005648	0.9999537652	0
2.8204370028	0.1259364259	0.9999537652	1.0473264101
-1.0553982292	0.7475491601	0.9999537652	1.1247693461
1.3788650953	0.2326408704	0.9999537652	1.1762737506
1.1558667666	0.4682730715	0.9999537652	1.047296754
-1.0641993194	0.7875772109	0.9999537652	1.1504131496
1.906929619	0.2287373946	0.9999537652	-1.8076940847
1.2185613789	0.3171824383	0.9999537652	1.1110018251
-1.2868191341	0.3743962017	0.9999537652	-1.2050129896
-1.1416409003	0.416250177	0.9999537652	-1.120534752
1.2476907627	0.7138964939	0.9999537652	-1.013822428
-1.1124710649	0.8695624557	0.9999537652	1.8032167839
2.7933974408	0.6948713406	0.9999537652	-3.3826461929
-1.0743494351	0.55228298	0.9999537652	1.1245661604
2.3335098933	0.4402988569	0.9999537652	-1.4609057566
-1.0782624345	0.8291134416	0.9999537652	-1.1084308424
-1.0063461039	0.9676709442	0.9999537652	1.2055330357
1.3653240897	0.1135225939	0.9999537652	-1.5081863564
-1.3441069402	0.6689428982	0.9999537652	-1.3565062967
-1.0516577135	0.6809218519	0.9999537652	-1.2517600711
1.0151744851	0.9265961412	0.9999537652	-1.0470911945

1.4475824847	0.0476381873	0.9999537652	-1.4763839802
1.7463566065	0.0830853989	0.9999537652	1.0494342308
1.3177878897	0.3820750638	0.9999537652	-1.2054035958
-1.9559533151	0.2734561958	0.9999537652	1.5313660668
1.4196256229	0.2526398297	0.9999537652	-1.26713777
-1.2639957703	0.208777656	0.9999537652	1.0987073359
1.3666903992	0.0254134835	0.9999537652	1.0213041724
-1.2925019483	0.5775165415	0.9999537652	-1.1850676391
1.1823716234	0.7716266879	0.9999537652	1.9394623409
2.7933974408	0.6948713406	0.9999537652	-1.7829804346
18.5983228161	0.2580521535	0.9999537652	-1.7622084838
1.7482661688	0.4628829324	0.9999537652	1.2242146434
1.0484510524	0.9375957026	0.9999537652	1.8970550837
-1.1755560667	0.8214953964	0.9999537652	1.0012045069
1.2458528047	0.1553644419	0.9999537652	1.1270686261
1.2690129842	0.5886625791	0.9999537652	1.0253827829
1.2916350336	0.1743918669	0.9999537652	-1.3227652287
1.1722727737	0.4102416559	0.9999537652	1.0954701246
-1.1927418657	0.2338604041	0.9999537652	-1.2908309235
-4.8154268017	0.1711980838	0.9999537652	-1.4688582794
-1.2985839753	0.1002733076	0.9999537652	-1.1255768705
1.4320838073	0.3460680139	0.9999537652	-1.6583240028
-1.7472607057	0.0402400421	0.9999537652	1.2411684383
1.0985483363	0.7720239733	0.9999537652	-1.5442375212
1.0310635756	0.8353097527	0.9999537652	-1.0166134526
1.1242912857	0.4668436377	0.9999537652	-1.5054888918
4.5544554512	0.5691174365	0.9999537652	-3.1944206015
-1.0068830088	0.9621735952	0.9999537652	1.0046624951
1.1111136264	0.7647554886	0.9999537652	1.5070956666
-1.1752136988	0.6250345101	0.9999537652	-1.4957532113
9.8205505243	0.3837505727	0.9999537652	1.1910156039
-1.0151329301	0.9615319037	0.9999537652	1.0102007223
1.0216665371	0.8987596314	0.9999537652	1.0164888743
2.7345456454	0.1996468189	0.9999537652	2.0314603002
4.2722366566	0.2006421097	0.9999537652	-1.3794335919
2.7933974408	0.6948713406	0.9999537652	0
-1.2954243326	0.2628456139	0.9999537652	-1.5710435718
1.1023024147	0.3873336606	0.9999537652	1.003950646
1.0290098953	0.9055964291	0.9999537652	1.1297657117
-1.1015308523	0.5702507305	0.9999537652	1.044204672
1.1540124325	0.3110621586	0.9999537652	-1.1730916061
2.7933974408	0.6948713406	0.9999537652	0
1.1092861353	0.6160730988	0.9999537652	1.0331513001
4.5505976667	0.5557790267	0.9999537652	-1.2375740166
0	0	0	20.5862458385
1.0190257003	0.9522403606	0.9999537652	-1.2875554414
0	0	0	4.9527501949
0	0	0	1.2400291296
3.8622037496	0.6192487316	0.9999537652	1.066768139

1.5080610755	0.2870358661	0.9999537652	1.2085064706
1.0051455705	0.9789580781	0.9999537652	-1.2493646909
-1.3134657905	0.078067684	0.9999537652	-1.2279633221
1.4971426362	0.007404	0.9999537652	-1.0638698069
1.4232493793	0.0584489606	0.9999537652	1.0105417796
-2.5838710423	0.5184586067	0.9999537652	1.0679531163
2.1462288065	0.5693449697	0.9999537652	-1.0281427069
1.0369596019	0.8354600279	0.9999537652	-1.2835066516
-1.3160085985	0.6486525711	0.9999537652	-1.2892848175
-1.0973540167	0.6866493543	0.9999537652	-1.0685467739
1.2303320746	0.1199768546	0.9999537652	1.063137466
1.1204342736	0.6700261411	0.9999537652	-1.0695890564
1.1851804944	0.5281572802	0.9999537652	1.1480253285
-1.3665054091	0.3354449374	0.9999537652	-1.2765901538
1.0736659873	0.5838239078	0.9999537652	-1.1067404018
0	0	0	-1.7811487064
1.3040940818	0.1427049689	0.9999537652	1.4520261279
1.2521099466	0.2337016339	0.9999537652	-1.1559341711
-1.5867212121	0.7194021882	0.9999537652	-1.1432552104
-1.06423776	0.7463907327	0.9999537652	-1.2261460066
1.032915085	0.8612954169	0.9999537652	1.0272469439
29.8698312703	0.2177863823	0.9999537652	1.0729559622
-1.2252227094	0.2868897168	0.9999537652	-1.1347568438
-1.4420309206	0.5598383811	0.9999537652	-1.1124754531
-1.1045488064	0.40026188	0.9999537652	1.1002203095
-1.0630634165	0.6777411347	0.9999537652	-1.4084144008
-1.6687223317	0.6215950954	0.9999537652	0
-1.6973662086	0.5474133501	0.9999537652	0
-1.0280584149	0.9214690687	0.9999537652	-1.3682891857
-1.8650254067	0.3015269972	0.9999537652	-1.0569502624
0	0	0	-1.8588429871
-1.5875118025	0.7201493553	0.9999537652	9.7116702472
-1.0293606601	0.829107166	0.9999537652	1.1793600402
-1.0085355311	0.9930374939	0.9999537652	2.2253832076
1.0690082471	0.8628931491	0.9999537652	1.9620588819
1.2571062983	0.2923318567	0.9999537652	1.3068231642
1.4509590208	0.189029105	0.9999537652	-1.0060823977
2.0259070874	0.1623554539	0.9999537652	1.7292704494
-1.1380592839	0.4477371455	0.9999537652	1.0500446226
2.7933974408	0.6948713406	0.9999537652	2.7983193256
-1.0010583562	0.9949979653	0.9999537652	-1.1393596982
-1.436108247	0.2084486858	0.9999537652	1.0852330837
0	0	0	2.9988886918
1.1576166872	0.4582439296	0.9999537652	-1.0389598578
-1.0018962327	0.987862453	0.9999537652	-1.4583331983
1.2065683536	0.7159980484	0.9999537652	-1.2538296023
1.0815467883	0.6905557318	0.9999537652	-1.0221428959
-17.0527724669	0.1064209713	0.9999537652	18.6465640159
-1.0036777173	0.990590593	0.9999537652	1.1326136241

-3.2640787972	0.0865176329	0.9999537652	1.0091255776
1.8267262185	0.065227085	0.9999537652	-1.1938784617
1.7550025421	0.0848264417	0.9999537652	-1.1364730368
10.3257158514	0.0003955	0.9999537652	-1.3273865216
-1.0139376956	0.9494443371	0.9999537652	-1.2606363892
2.0363455658	0.4192599304	0.9999537652	-1.0648363021
-1.4267571933	0.5004994825	0.9999537652	1.207055345
-1.2732762498	0.1088317454	0.9999537652	-1.0881085736
1.0525190758	0.9170460597	0.9999537652	-1.1193114299
0	0	0	4.9527501949
-1.9467268757	0.00405	0.9999537652	-1.1931768843
1.1950571358	0.5081226889	0.9999537652	-1.1943853152
0	0	0	5.7130160159
-1.0979248749	0.6095719606	0.9999537652	-1.1212242689
2.6872400047	0.26362364	0.9999537652	-1.1601340508
2.7933974408	0.6948713406	0.9999537652	-1.658786914
0	0	0	6.908559683
1.7166879814	0.3015433011	0.9999537652	-1.3820107225
-2.4397350298	0.3123038017	0.9999537652	-1.379117529
1.1535037484	0.3675774058	0.9999537652	1.2094013101
-3.1179374904	0.2574432025	0.9999537652	3.7631108754
1.0614263706	0.8889007518	0.9999537652	1.3462221558
1.1207661674	0.5416247967	0.9999537652	-1.0835401821
1.0782959684	0.5770187908	0.9999537652	-1.1044786833
-1.0719128329	0.6735832334	0.9999537652	1.3061091306
-1.1761764882	0.7959563116	0.9999537652	-1.0330277526
0	0	0	9.7116702472
6.2167132833	0.0849912995	0.9999537652	2.8014102743
0	0	0	-1.0084773122
-1.93731553	0.1230901383	0.9999537652	-1.0229163268
1.6024984014	0.686210146	0.9999537652	2.329086775
1.0973831338	0.6560755214	0.9999537652	1.2786506033
-1.1453192235	0.9084228918	0.9999537652	-1.5933216452
1.1402754382	0.6505659793	0.9999537652	-1.534652146
-1.0709734459	0.8932928065	0.9999537652	1.9907660386
1.0927017526	0.7268980104	0.9999537652	1.4039691797
-1.0719888271	0.6203771366	0.9999537652	-1.2377895624
0	0	0	-1.8577842586
2.0351796303	0.1185950445	0.9999537652	1.048864024
-1.1641919713	0.5551718631	0.9999537652	-1.1611543717
1.177090287	0.3509968983	0.9999537652	1.1192150025
1.1879887589	0.1531499514	0.9999537652	-1.3964218786
-1.7540719944	0.2121022615	0.9999537652	-1.1485866433
1.603906631	0.6805398061	0.9999537652	1.1360578751
-1.0929418997	0.5728940492	0.9999537652	-1.033696669
1.471195557	0.4657860859	0.9999537652	-1.3065878424
-1.2276362296	0.1224673987	0.9999537652	-1.1158007982
-1.1266634304	0.5275220039	0.9999537652	-1.2141472478
1.0909679987	0.8809229433	0.9999537652	1.0561583085

1.4709411218	0.018013412	0.9999537652	-1.3422603268
-1.3510151174	0.5756497845	0.9999537652	1.0144898042
-1.1894962702	0.6595292809	0.9999537652	-1.2764153756
2.7933974408	0.6948713406	0.9999537652	-1.0601741034
0	0	0	-1.3808714259
1.1281416946	0.7374502938	0.9999537652	-1.2102430893
3.8622037496	0.619217824	0.9999537652	1.115697704
9.8205505243	0.383964091	0.9999537652	1.1257491784
-1.2666655769	0.4597281813	0.9999537652	-1.0338148364
0	0	0	1.9432165124
2.7933974408	0.6948713406	0.9999537652	0
-1.07307118	0.8883734348	0.9999537652	1.0096073434
2.6239526374	0.1313100907	0.9999537652	-1.0003629227
1.6476882037	0.596576655	0.9999537652	1.2747352577
-1.0894773671	0.8938770878	0.9999537652	-1.493761877
1.0138193575	0.9706121908	0.9999537652	1.0689012739
-1.0873687295	0.5648046501	0.9999537652	1.1418789822
-1.0787763667	0.7008960567	0.9999537652	-1.1010982524
0	0	0	0
2.7933974408	0.6948713406	0.9999537652	0
1.5987470131	0.3780539628	0.9999537652	-1.5314170113
-1.1635738407	0.6665308741	0.9999537652	1.1110286957
1.2655060468	0.2766468799	0.9999537652	1.5327504982
1.1449704735	0.4554874515	0.9999537652	1.1247855206
-1.3888213571	0.0120867815	0.9999537652	-1.3035805716
1.1861573905	0.7272933958	0.9999537652	-1.3288093709
1.1442220107	0.3872763964	0.9999537652	-1.21328781
-1.0818367621	0.8541655733	0.9999537652	-1.3164998192
1.055285734	0.8870423753	0.9999537652	1.153500395
4.5544554512	0.5691154432	0.9999537652	-1.5002047937
-1.1718047967	0.8443104731	0.9999537652	-2.9687025352
1.2669793002	0.3655763652	0.9999537652	1.2205689728
1.5345406962	0.2685594143	0.9999537652	1.3428761373
1.1929726634	0.2563076318	0.9999537652	-1.1142674056
1.1399460543	0.575533989	0.9999537652	-1.3603715413
1.0263927084	0.8361054328	0.9999537652	-1.4364937229
2.8162992884	0.3665312	0.9999537652	-1.2384158616
-1.2462075393	0.1511559927	0.9999537652	-1.0718958087
1.3322071085	0.6205680367	0.9999537652	-1.5371931092
-1.1426563294	0.5050162828	0.9999537652	-1.7663278549
-1.16596854	0.5348960718	0.9999537652	-1.3948278289
15.1008094413	0.2915047567	0.9999537652	-5.0960079851
-1.4504451984	0.1404386498	0.9999537652	-1.364605108
-1.1128693633	0.3943221213	0.9999537652	1.1276960565
2.0372378093	0.4352795618	0.9999537652	2.1074774138
1.1224138006	0.5624427143	0.9999537652	-1.1394339584
1.1234480071	0.5927726365	0.9999537652	1.0429606645
1.1414955553	0.688348978	0.9999537652	-1.0129551596
18.6298281873	0.2553026637	0.9999537652	-1.3123873953

1.1268507337	0.353393727	0.9999537652	-1.0449177474
-1.4162636112	0.169824119	0.9999537652	1.1490009265
-1.233298793	0.2094907544	0.9999537652	-1.1592534993
-1.2099540125	0.273001395	0.9999537652	-1.1264145318
1.4366050279	0.0856662037	0.9999537652	-1.2273667388
-1.2132688221	0.5852036512	0.9999537652	-1.1208182977
2.5976061076	0.1040264891	0.9999537652	-1.3345403183
-1.0685380045	0.9372568069	0.9999537652	-1.6770957387
-1.4580288226	0.1251923031	0.9999537652	-1.1139850457
1.1966118699	0.3664719812	0.9999537652	1.0200838671
-2.0049217286	0.4846480013	0.9999537652	-1.0005722444
-1.3806165014	0.3922009695	0.9999537652	-1.572077592
-1.1132667401	0.744901039	0.9999537652	-1.3595718051
-1.6568408305	0.4210319385	0.9999537652	-1.338149107
0	0	0	6.908559683
2.2227236654	0.5576798218	0.9999537652	-2.0477227478
1.1332615238	0.7051276106	0.9999537652	-1.0229460566
2.7933974408	0.6948713406	0.9999537652	-2.0482026754
1.3820692738	0.7418794899	0.9999537652	-1.2790173198
-1.1236546327	0.5159860458	0.9999537652	-1.1870418109
-1.0219825914	0.924742867	0.9999537652	1.2396646562
1.7039322442	0.2510657094	0.9999537652	-1.0657726067
0	0	0	2.996475627
0	0	0	2.996475627
-3.0586831592	0.0112522695	0.9999537652	-1.1235708981
0	0	0	21.234349681
1.5800944083	0.4969428548	0.9999537652	-1.289762344
-1.1371645363	0.674019683	0.9999537652	1.3100732482
2.7957660672	0.7055116951	0.9999537652	4.3967731856
2.7933974408	0.6948713406	0.9999537652	-5.3583704783
0	0	0	1.2410271878
-1.2459100936	0.6630945225	0.9999537652	1.2801203073
0	0	0	2.996475627
1.009065489	0.9845214742	0.9999537652	1.8000199784
57.187733295	0.1123556626	0.9999537652	-3.4230273045
0	0	0	-1.8580020618
-1.467185214	0.4062869535	0.9999537652	1.3662600626
1.2610885254	0.7418548403	0.9999537652	-1.7236068244
-1.2148704852	0.4850438186	0.9999537652	-1.1797502881
1.3336520419	0.0819193623	0.9999537652	-1.2540981758
5.5083463532	0.0701864284	0.9999537652	1.8715612428
1.2096219116	0.2042829636	0.9999537652	-1.4630395478
1.0025878864	0.9912681226	0.9999537652	-1.1857382596
-1.1938303351	0.5794445479	0.9999537652	-1.4178250262
-1.0370189999	0.8057155715	0.9999537652	-1.0451859915
-1.026119763	0.8848562237	0.9999537652	-1.1396437199
1.395501518	0.1314970435	0.9999537652	1.2400867901
1.3076659969	0.4808092242	0.9999537652	-1.0971436643
1.2354572624	0.3340771775	0.9999537652	-1.1628088682

-1.0862235581	0.6449499584	0.9999537652	-1.0494848713
-1.1500823788	0.4379295734	0.9999537652	-1.1066781983
2.7933974408	0.6948713406	0.9999537652	-1.1250196696
1.1470858769	0.7815227573	0.9999537652	1.1223813354
1.001038249	0.9947860259	0.9999537652	1.1695399364
2.0057237411	0.2464624472	0.9999537652	1.3630917942
-1.1873725306	0.3392017972	0.9999537652	1.0951827936
16.8433158146	0.2737823561	0.9999537652	1.7462448558
1.0712193836	0.667849089	0.9999537652	-1.3737945697
1.2184218866	0.087700567	0.9999537652	-1.1081969004
1.0340248443	0.8575274954	0.9999537652	-1.3565382948
-1.1745894864	0.3095283838	0.9999537652	-1.0865234307
4.5544554512	0.56911345	0.9999537652	2.9302599816
1.1057812314	0.6081386661	0.9999537652	1.3263697254
-1.1649408325	0.2855274374	0.9999537652	-1.1535871194
1.0257391298	0.8955622162	0.9999537652	-1.1491347225
1.8723258494	0.009106	0.9999537652	1.3227579242
-1.0525632343	0.7536432774	0.9999537652	-1.3327032796
1.1338604475	0.3796128015	0.9999537652	-1.3234144172
11.5766527773	0.3454082547	0.9999537652	-1.2402444264
1.1410834531	0.7558440573	0.9999537652	-1.0441383663
-1.0605156096	0.7671190227	0.9999537652	1.1358557182
1.131099688	0.4455767943	0.9999537652	-1.0723378931
-3.0930401431	0.1225336416	0.9999537652	-1.0418572178
0	0	0	2.9988886918
-1.6267638816	0.4445153888	0.9999537652	-1.861834348
-1.4218047209	0.5194881691	0.9999537652	-1.4905881959
-1.5763646825	0.5009531945	0.9999537652	1.2536187606
4.0347077664	0.2015377033	0.9999537652	-1.5805867929
-1.2365301525	0.6187436842	0.9999537652	1.1979151226
0	0	0	0
1.1263768786	0.4953153322	0.9999537652	1.1994469857
2.5734927579	0.4060790046	0.9999537652	-1.3436666544
2.7933974408	0.6948713406	0.9999537652	1.2398837679
1.0587017639	0.9181531174	0.9999537652	-1.4054675939
1.1949616849	0.3671841622	0.9999537652	-1.3913625158
1.4755277565	0.007468	0.9999537652	1.2707775723
-1.035281337	0.8453948225	0.9999537652	-1.1040211784
-1.077841221	0.6360926516	0.9999537652	-1.0783846538
1.0929080844	0.5775855807	0.9999537652	1.07672512
-1.1772887308	0.7533021757	0.9999537652	-1.303877636
-1.0223357772	0.9373761128	0.9999537652	-1.4638354216
1.053261896	0.7312630396	0.9999537652	-1.2437038296
-1.1346812318	0.7474540794	0.9999537652	1.5931238094
1.0291290148	0.8366993142	0.9999537652	1.1088192833
4.5505976667	0.5557790267	0.9999537652	16.6806485101
1.7916349996	0.3105003687	0.9999537652	-1.0172517022
1.1454331778	0.6836879932	0.9999537652	-1.1028188337
1.6719641693	0.1808932908	0.9999537652	1.1231736035

2.7933974408	0.6948713406	0.9999537652	0
0	0	0	8.9512754559
1.1542967725	0.4986015926	0.9999537652	-1.2428113881
-7.0403382421	0.1005442596	0.9999537652	14.7373490367
-1.9290774189	0.6162740647	0.9999537652	0
-1.1309592246	0.4183687682	0.9999537652	-1.2271183541
-1.2779195599	0.1841567712	0.9999537652	-1.4064907524
1.1046260832	0.7418420231	0.9999537652	1.0143323519
-1.4986982513	0.0797381862	0.9999537652	-1.4022422059
-1.0879166553	0.5777995377	0.9999537652	-1.1096927001
6.3075231393	0.4851654977	0.9999537652	-1.4680135518
18.5983228161	0.2580535817	0.9999537652	5.2143761525
0	0	0	0
4.5505976667	0.5557790267	0.9999537652	1.9432271302
0	0	0	2.996475627
1.2523812163	0.2185934968	0.9999537652	1.1631657223
-1.0375894658	0.8586634434	0.9999537652	1.2787147993
1.1506613189	0.5388169716	0.9999537652	1.0188388352
-7.0458783659	0.0842665862	0.9999537652	1.4239170092
1.0433630444	0.7561994395	0.9999537652	-1.2416065854
1.0003654271	0.9982515557	0.9999537652	-1.5099916317
4.5505976667	0.5557790267	0.9999537652	1.5740434553
1.0569659205	0.8362623647	0.9999537652	-1.0586187108
0	0	0	6.908559683
1.3164896658	0.349665885	0.9999537652	2.5975288819
-1.0913409447	0.8107263823	0.9999537652	-1.1743577437
-1.085408551	0.652071967	0.9999537652	-1.0476977684
1.0321393073	0.8626602276	0.9999537652	-1.160044071
1.263371817	0.2035082129	0.9999537652	1.0027886176
1.2995518875	0.4519143484	0.9999537652	-1.0337214812
1.2578299656	0.4954577645	0.9999537652	-1.4092705524
-1.0040890011	0.9829905955	0.9999537652	-1.3401748293
1.2689653878	0.3053835969	0.9999537652	1.0972667659
-1.6631765169	0.4183763937	0.9999537652	-1.1962298278
1.2363480637	0.3202040232	0.9999537652	-1.0254072819
-1.1503649434	0.3798858903	0.9999537652	-1.2407701073
1.1177376122	0.7209603172	0.9999537652	-1.0359795774
1.2401278409	0.6359609104	0.9999537652	-1.0450950344
-1.209275812	0.2579939967	0.9999537652	-1.1168873591
0	0	0	1.2394611522
-1.0468894492	0.7509938438	0.9999537652	-1.2967486027
-1.1280813984	0.5824659127	0.9999537652	1.1019241375
-1.2206505016	0.6585729013	0.9999537652	1.2982156351
-1.1292691121	0.4051571655	0.9999537652	-1.1207564135
1.0226734087	0.9062388152	0.9999537652	-1.0133870332
2.6517750321	0.1137899357	0.9999537652	1.38408839
1.058939053	0.7565235258	0.9999537652	1.0264611055
1.1223268592	0.4311710026	0.9999537652	-1.1980028151
-1.011363348	0.9614125468	0.9999537652	-1.0204186667

2.7933974408	0.6948713406	0.9999537652	6.908559683
1.5373481529	0.2488226969	0.9999537652	-1.0443588544
-1.1767280502	0.3867672578	0.9999537652	-1.1829846796
2.7933974408	0.6948713406	0.9999537652	12.7909013375
-1.096363092	0.4381520923	0.9999537652	-1.5078135119
2.7933974408	0.6948713406	0.9999537652	12.7732027325
-1.228774061	0.4245989417	0.9999537652	-1.4253833756
1.0578046988	0.8627365462	0.9999537652	-1.373482463
9.8205505243	0.3838938042	0.9999537652	0
2.6478843491	0.4841251341	0.9999537652	1.2400291296
1.0075921245	0.9753178566	0.9999537652	-1.2147172582
1.1340072463	0.2962197846	0.9999537652	1.1229859791
0	0	0	8.8639046058
-1.0158784018	0.9580962615	0.9999537652	-1.5051103498
2.7933974408	0.6948713406	0.9999537652	10.8262784008
-1.0193890362	0.9159097666	0.9999537652	-1.1582453594
1.182633619	0.6391293015	0.9999537652	1.1188571753
1.0124323008	0.970400032	0.9999537652	1.393395795
1.2866053758	0.3178883358	0.9999537652	-1.0079769229
-1.0455899786	0.7167943151	0.9999537652	1.1167550107
1.2976630662	0.3484942625	0.9999537652	2.5451612408
1.6024984014	0.6868416379	0.9999537652	1.6354495038
4.5505976667	0.5557790267	0.9999537652	2.6979916739
-1.8031291404	0.005258	0.9999537652	-1.3594899797
1.075052797	0.5793106644	0.9999537652	1.0242667762
-1.5000103367	0.0378717179	0.9999537652	-1.1129408141
2.3232499608	0.0759825089	0.9999537652	2.2894864996
2.7933974408	0.6948713406	0.9999537652	1.109951066
1.0742663477	0.6986155493	0.9999537652	-1.4030457436
-1.0857698111	0.7650941046	0.9999537652	-1.1308726791
-1.5992043474	0.0292797251	0.9999537652	-1.045857399
1.0498805782	0.9153925553	0.9999537652	-1.0711605254
-7.3545033058	0.0461451899	0.9999537652	1.0962558674
1.1367523693	0.2583196913	0.9999537652	1.1221138119
-1.0775719903	0.7233708187	0.9999537652	-1.0011542077
4.5505976667	0.5557790267	0.9999537652	-1.1241083699
-1.5881570201	0.7077423276	0.9999537652	-1.6580718273
-1.7095355717	0.1246906798	0.9999537652	1.0934734729
1.1104162823	0.7391088992	0.9999537652	-1.3877206036
-1.0465422522	0.8167737468	0.9999537652	-1.242747704
1.0582372718	0.9167360602	0.9999537652	1.1746426066
1.1705908586	0.7466496761	0.9999537652	-1.4381901012
-1.0031946337	0.9866699521	0.9999537652	-1.3424261703
1.1258393082	0.5475827881	0.9999537652	1.1353958728
1.0064785949	0.9665446248	0.9999537652	-1.4493212981
1.0556465585	0.7757672672	0.9999537652	-1.5364602838
1.1228311004	0.5454087816	0.9999537652	1.083947944
1.0235618423	0.95161708	0.9999537652	1.0982353058
1.2889858652	0.4360421881	0.9999537652	-1.0034674522

14.82452558	0.0121449648	0.9999537652	-2.5782436629
-1.6741932226	0.2456607751	0.9999537652	2.1358250889
1.2291170595	0.5661657579	0.9999537652	-1.2036944082
-1.3118702523	0.5274168221	0.9999537652	-1.9495375127
1.1094173088	0.5540296474	0.9999537652	-1.4131486317
6.3075231393	0.485156057	0.9999537652	1.2657113394
1.1871337559	0.6251749007	0.9999537652	-1.161938699
-1.1463369772	0.478542075	0.9999537652	-1.3309772441
-1.1859740966	0.286882304	0.9999537652	-1.3719877269
0	0	0	4.9527501949
-1.022961404	0.8709906034	0.9999537652	-1.0010809753
-2.8761557559	0.1800622315	0.9999537652	-1.5146989318
3.5995952878	0.0123621257	0.9999537652	-1.5535897272
-2.4056861539	0.2979252185	0.9999537652	-1.6233324417
-1.4139821599	0.4696351681	0.9999537652	-1.5391150511
1.1482117586	0.6705865546	0.9999537652	1.0348962827
-1.041369009	0.8972527596	0.9999537652	1.2165094772
-1.0315532783	0.8858593104	0.9999537652	1.1730053244
2.7933974408	0.6948713406	0.9999537652	-5.3608259368
-1.1814159946	0.5917033152	0.9999537652	1.2648504152
1.5136569565	0.663621509	0.9999537652	1.0672344538
1.3594099105	0.7967251991	0.9999537652	1.782653648
0	0	0	4.9561842702
1.4405757815	0.1855593607	0.9999537652	1.2232242095
1.6316072287	0.2591807405	0.9999537652	-1.0284201167
1.6520799496	0.3222463328	0.9999537652	1.1550303116
-1.1225725447	0.7345970144	0.9999537652	1.1249691142
2.7933974408	0.6948713406	0.9999537652	0
0	0	0	2.9985540909
-1.3526707021	0.434671712	0.9999537652	1.4528714662
2.7933974408	0.6948713406	0.9999537652	0
2.7933974408	0.6948713406	0.9999537652	0
4.5505976667	0.5557790267	0.9999537652	-1.859280336
-1.109326109	0.7652812065	0.9999537652	1.3398488739
1.6117727667	0.2758460632	0.9999537652	1.193363803
-1.1294930875	0.3154847956	0.9999537652	1.1191816572
-2.587194901	0.5006282751	0.9999537652	2.996475627
1.3490258238	0.0786177907	0.9999537652	-1.0697205926
1.5967362629	0.0209120421	0.9999537652	1.2693740616
-1.0675527747	0.7757677745	0.9999537652	1.1312572492
-1.5573027356	0.1096838255	0.9999537652	-1.4632233352
-1.1624006169	0.3172283933	0.9999537652	-1.0558039249
1.1719869208	0.6294469485	0.9999537652	1.5611255112
-1.5452790953	0.0718196266	0.9999537652	-1.0516392973
-1.1640194144	0.6179363249	0.9999537652	1.1958631106
-1.0504458159	0.8434366637	0.9999537652	1.4551075882
1.1237069365	0.4081851676	0.9999537652	1.2750825638
1.0223194374	0.8948396545	0.9999537652	-1.01323961
1.2935952465	0.7175409253	0.9999537652	1.1540898154

1.239470163	0.2268006772	0.9999537652	1.5232282048
0	0	0	-1.8582093966
-1.7864429677	0.1444320758	0.9999537652	-1.0329950039
1.0902790324	0.8273722316	0.9999537652	-1.410084982
-1.7323125616	0.4238959165	0.9999537652	1.4892730691
1.7090046434	0.1760065813	0.9999537652	-1.1450397395
-2.3113168502	0.1942861307	0.9999537652	1.2798415061
1.0632120177	0.8024801714	0.9999537652	-1.2104646259
-1.0100849333	0.9325857124	0.9999537652	-1.2798197453
-1.0166484871	0.9250288939	0.9999537652	1.2009468591
-1.0493327012	0.7997777324	0.9999537652	1.3067000269
-1.096028251	0.7632270094	0.9999537652	1.2184805412
-1.1761637688	0.3459219346	0.9999537652	1.049184107
2.6239154224	0.1607521775	0.9999537652	-1.077264645
1.0726209006	0.5690020452	0.9999537652	1.0316130586
-1.1703501368	0.455105873	0.9999537652	-1.0841726273
-1.0096470273	0.9423582173	0.9999537652	1.1124971368
-1.9398321751	0.4439852018	0.9999537652	-1.0369932741
1.1287178186	0.7940283367	0.9999537652	1.3310925361
1.2629828152	0.7087132151	0.9999537652	1.9043397705
0	0	0	5.0196635897
-1.1471550932	0.5877606531	0.9999537652	-1.0553164576
-4.8135938501	0.1786396346	0.9999537652	-3.1776407877
0	0	0	1.4531210393
-6.9736190158	0.2250321147	0.9999537652	12.7732027325
-1.1030814387	0.5669685688	0.9999537652	1.3807147261
1.5364576902	0.4988590638	0.9999537652	-2.2200956212
1.0042861411	0.9760134219	0.9999537652	-1.2722745309
2.0844878286	0.1186410286	0.9999537652	1.2896809982
-6.9762758667	0.2571161994	0.9999537652	0
-1.8034168983	0.1925407177	0.9999537652	1.2398105102
0	0	0	0
-2.3755481719	0.0888315432	0.9999537652	1.9125410629
1.1504905576	0.3148087744	0.9999537652	1.026249466
0	0	0	2.9985540909
-1.4503791503	0.0970538506	0.9999537652	1.1107027101
1.0237370269	0.8921997631	0.9999537652	-1.1485374493
1.5143610053	0.6457699058	0.9999537652	-2.6631453939
-1.0020094292	0.9886481287	0.9999537652	-1.0078073856
-6.9762758667	0.257123944	0.9999537652	8.8639046058
-4.8154268017	0.1712411542	0.9999537652	-1.7606748791
1.0284143981	0.912840579	0.9999537652	-1.259337601
1.1611327203	0.6749962601	0.9999537652	-1.2401750591
1.3954064265	0.6466040402	0.9999537652	-1.3416682102
1.0293152054	0.9071517752	0.9999537652	1.0635387767
1.0366976783	0.9655652907	0.9999537652	1.106644687
2.7933974408	0.6948713406	0.9999537652	6.908559683
-1.5875118025	0.7201406819	0.9999537652	-1.8393168198
-1.8767924498	0.2022052213	0.9999537652	-1.1925389909

-1.1222971227	0.7908495522	0.9999537652	-1.2559844064
-1.4768396305	0.6824825744	0.9999537652	-1.3628769604
-1.1202018389	0.7482467981	0.9999537652	1.1685462677
-1.016035328	0.9109619303	0.9999537652	1.0058733591
1.8244386635	0.20278243	0.9999537652	1.146043406
-1.1746456801	0.7610213283	0.9999537652	1.1787001477
1.2886625128	0.2982560495	0.9999537652	1.0943595005
6.3075231393	0.4851466235	0.9999537652	-1.859280336
1.7856493071	0.1634627446	0.9999537652	1.1762778563
-1.7978430642	0.3905726788	0.9999537652	-1.4066818868
1.6565751183	0.6570178588	0.9999537652	-6.0629655178
1.7064255976	0.2607080521	0.9999537652	1.0048788457
-1.1744739978	0.5334841777	0.9999537652	-1.0074490986
1.1162834585	0.928075067	0.9999537652	2.996475627
-1.2295894174	0.2793843153	0.9999537652	-1.1939503566
1.1708542901	0.3156639245	0.9999537652	-1.1420555567
1.0175079716	0.9001862145	0.9999537652	-1.4401746456
2.7933974408	0.6948713406	0.9999537652	0
1.2199723318	0.7302383051	0.9999537652	-1.411296211
-1.3575061074	0.2360726128	0.9999537652	1.3268929248
-1.2277171428	0.6123856021	0.9999537652	1.3706605491
2.7933974408	0.6948713406	0.9999537652	4.9527501949
1.1900847293	0.7146819542	0.9999537652	1.1435880799
1.0415427222	0.8657156359	0.9999537652	1.3165446469
1.0203895018	0.9508330417	0.9999537652	-1.1433807021
2.1966379029	0.3073143036	0.9999537652	-1.1689146499
-1.0438016879	0.8671588789	0.9999537652	1.0548551032
-1.3891045615	0.3098036488	0.9999537652	-1.0010964566
26.2649513705	0.2127166708	0.9999537652	-2.0338104344
2.7933974408	0.6948713406	0.9999537652	1.5908108123
-2.955963562	0.3188880279	0.9999537652	7.9160069202
2.7933974408	0.6948713406	0.9999537652	-1.1554448798
0	0	0	-1.4680135518
1.1120467753	0.7967618205	0.9999537652	1.0500298609
1.5667419971	0.4960075137	0.9999537652	1.5848797111
4.5505976667	0.5557790267	0.9999537652	-2.0463039075
-1.0724904296	0.8773014093	0.9999537652	1.2941465606
-2.0380285376	0.1282113529	0.9999537652	-1.2062349717
-1.3666719636	0.5113910877	0.9999537652	1.4109581402
1.0240467077	0.9668185573	0.9999537652	1.61720377
-1.7365671102	0.4033500878	0.9999537652	2.4865368686
2.7933974408	0.6948713406	0.9999537652	-2.2056994995
4.5505976667	0.5557790267	0.9999537652	-2.8891109792
-2.587194901	0.500618863	0.9999537652	-3.382249888
0	0	0	2.996475627
2.7933974408	0.6948713406	0.9999537652	0
-1.7778717557	0.0493320958	0.9999537652	-1.1598130679
-1.8860846276	0.0565457086	0.9999537652	1.0617640928
-1.7353594011	0.4459321111	0.9999537652	1.9673405098

1.3616660934	0.2159349163	0.9999537652	-1.0067872208
0	0	0	-5.3608259368
1.1096169563	0.669335692	0.9999537652	-1.0092471301
-1.7000974549	0.5642783408	0.9999537652	1.088279459
1.0609220037	0.6401979008	0.9999537652	1.084279428
-3.2110570455	0.1789959947	0.9999537652	-1.2370590979
1.9005950367	0.0676868115	0.9999537652	-1.1034362219
-1.1074481279	0.8575875992	0.9999537652	-1.4326840837
4.5545995984	0.5696558471	0.9999537652	-1.6201500558
1.7104175053	0.1048652315	0.9999537652	-1.1337878997
-1.04162631	0.7623370174	0.9999537652	1.0097261542
1.0504091041	0.7977776268	0.9999537652	1.0022104146
1.0480590319	0.7770635761	0.9999537652	-1.5833562462
2.7933974408	0.6948713406	0.9999537652	-5.3583704783
1.1319366373	0.4016992381	0.9999537652	-1.1185267595
1.0807983733	0.6595218413	0.9999537652	-1.3133215998
1.1469125055	0.6615525102	0.9999537652	-1.3494901439
1.0090502171	0.9637981776	0.9999537652	-1.0885622665
1.5652133061	0.0441764519	0.9999537652	-1.1202116347
1.7746986793	0.5317851422	0.9999537652	-3.4525384698
1.667743132	0.1421869887	0.9999537652	-1.1762989642
1.0044022912	0.9785734695	0.9999537652	-1.0958918134
2.7933974408	0.6948713406	0.9999537652	-3.8965260884
0	0	0	2.996475627
1.2390654071	0.82520923	0.9999537652	-1.2071759626
1.1212615905	0.8764301599	0.9999537652	-2.0441288174
1.2511402761	0.084499103	0.9999537652	1.1897330799
1.0576704962	0.7970535945	0.9999537652	-1.0545433632
1.0345591585	0.8953535086	0.9999537652	-1.1228954974
-1.3033478886	0.1811560729	0.9999537652	-1.0126558696
-1.0183719031	0.9694454293	0.9999537652	1.4616084642
1.5829254006	0.3506506439	0.9999537652	-1.2468159874
1.1217848564	0.3227532836	0.9999537652	1.0473343274
1.1235175252	0.5041935744	0.9999537652	-1.3338143901
-1.1314791281	0.5485688363	0.9999537652	1.0761231729
1.3222882774	0.2424777075	0.9999537652	-1.3392239908
-1.4936192846	0.5621758208	0.9999537652	-1.1122457742
1.1203334982	0.5531498837	0.9999537652	1.3008388436
0	0	0	-1.8594983184
-1.6973123489	0.5505433209	0.9999537652	1.2398837679
1.1590274252	0.258582843	0.9999537652	1.0120533717
0	0	0	0
1.1022883385	0.5764268934	0.9999537652	-1.2667201877
-1.7453178439	0.3376090411	0.9999537652	1.2762114499
-2.0093413255	0.0262037283	0.9999537652	-1.0760758998
-1.1384406097	0.7514674975	0.9999537652	-1.027217425
-1.370241401	0.7724693238	0.9999537652	1.9173151254
0	0	0	-1.3168181252
1.1235032197	0.3723083576	0.9999537652	-1.7925361789

1.0664480948	0.722310812	0.9999537652	1.0120021558
1.07253336	0.78580353	0.9999537652	1.0528558492
0	0	0	-5.3583704783
1.1888190984	0.7349761105	0.9999537652	1.0923689292
1.6112484302	0.1204637404	0.9999537652	-1.304117013
-1.4626255649	0.5298368492	0.9999537652	-2.1669390485
1.0695794985	0.7348037136	0.9999537652	-1.0855762624
13.3437712784	0.3162651917	0.9999537652	-1.4244561168
1.0151793847	0.9341432929	0.9999537652	-1.1920154691
1.3137838062	0.5886536884	0.9999537652	1.037533173
-1.173197598	0.4643320777	0.9999537652	-1.1359129716
-1.9766940606	0.358620074	0.9999537652	1.1401665501
-1.0406410007	0.9183476414	0.9999537652	-1.2003308242
1.5277506613	0.4225277507	0.9999537652	-1.0180890767
1.2452683054	0.1555964138	0.9999537652	-1.1620149005
2.7933974408	0.6948713406	0.9999537652	1.2398837679
1.8268037885	0.1322238752	0.9999537652	-1.2335121207
1.2178405026	0.3203198634	0.9999537652	-1.2853404188
2.7933974408	0.6948713406	0.9999537652	2.996475627
0	0	0	-1.1441751338
1.3266178201	0.6219441398	0.9999537652	1.5481620213
0	0	0	9.7116702472
-1.0475131665	0.7377653509	0.9999537652	-1.20410622
1.6709804649	0.120079398	0.9999537652	-1.5652617164
1.1273856946	0.6700853015	0.9999537652	-1.1091176957
-1.1510763739	0.6303626785	0.9999537652	1.1316339569
-2.2948394972	0.1846483075	0.9999537652	-1.2197693063
1.3514036244	0.1486181482	0.9999537652	-1.0159562467
1.2015914834	0.2277435442	0.9999537652	-1.2086785014
-2.3829585738	0.098072766	0.9999537652	5.8490850601
-1.0499779563	0.8284179728	0.9999537652	-1.3051684383
1.096886693	0.7642162676	0.9999537652	1.4399278316
-1.0186547096	0.9468371288	0.9999537652	-1.0090980532
4.7632247429	0.1405638546	0.9999537652	8.8700459102
1.3093074791	0.0287623083	0.9999537652	1.3319353735
-2.437607402	0.3171545822	0.9999537652	2.9985540909
2.7933974408	0.6948713406	0.9999537652	-1.1248877899
-1.0833990771	0.4754774016	0.9999537652	-1.0746473234
-1.3899054936	0.2338941641	0.9999537652	1.1359236223
1.0765801366	0.7958610846	0.9999537652	-1.2479152962
1.2883472386	0.71816335	0.9999537652	-1.2289559961
1.2374309422	0.3306815259	0.9999537652	-1.010237061
2.7933974408	0.6948713406	0.9999537652	6.908559683
-1.2352812073	0.5583451453	0.9999537652	-1.2873276747
1.39402103	0.2962550526	0.9999537652	1.5268412227
1.2880427605	0.2057183098	0.9999537652	1.0495946676
1.0853741222	0.8439081188	0.9999537652	-1.4331260173
1.1189781175	0.5423272372	0.9999537652	-1.2053580476
1.2669247997	0.3880510013	0.9999537652	1.4682927717

-1.7880842793	0.1137152489	0.9999537652	-1.3071773515
1.0085080113	0.962533812	0.9999537652	-1.2140724594
-2.6364591566	0.3036774825	0.9999537652	2.8094823151
-2.587194901	0.5006094511	0.9999537652	2.2220493756
-1.3845834311	0.4624602056	0.9999537652	-1.3828467394
-1.388693665	0.4732958489	0.9999537652	-1.1348003577
1.1807437876	0.8018084931	0.9999537652	-1.2943066969
8.064174035	0.4193601177	0.9999537652	1.569740882
-1.3012009203	0.7634629305	0.9999537652	2.9953398646
0	0	0	8.8639046058
1.4845631328	0.4657901969	0.9999537652	1.4194142339
-2.9548384089	0.3320339612	0.9999537652	1.4237450586
1.1783722105	0.8234975571	0.9999537652	-1.3274509342
1.1957414578	0.2930410887	0.9999537652	1.0707406718
-1.2652441392	0.6240302144	0.9999537652	1.248996885
-2.7990579126	0.1560453052	0.9999537652	-1.144718392
5.9744286098	0.0963038725	0.9999537652	2.6071338396
1.2963066726	0.5604264013	0.9999537652	-1.4531353517
-1.1096937514	0.6678099544	0.9999537652	1.076133164
1.3575806046	0.027183372	0.9999537652	-1.2967010744
-1.3921860315	0.6958702853	0.9999537652	1.5101393617
1.6596734241	0.0364216404	0.9999537652	1.0348982307
-1.144260977	0.6721072073	0.9999537652	-1.1490768115
1.0434844666	0.8212904973	0.9999537652	-1.1367935523
0	0	0	2.996475627
0	0	0	2.9985540909
-1.7646548859	0.1000204027	0.9999537652	-1.1422619559
0	0	0	1.6447327969
-1.1470703565	0.8069994196	0.9999537652	-1.000766043
2.228825771	0.1229087545	0.9999537652	1.0739898918
2.7933974408	0.6948713406	0.9999537652	0
0	0	0	2.9985540909
1.1300026147	0.8581974193	0.9999537652	-1.1766397111
1.3446238497	0.6536590721	0.9999537652	1.0363829803
1.0145384288	0.9641826977	0.9999537652	1.3582219674
-1.3541337391	0.3176787827	0.9999537652	1.266896433
-1.1013365972	0.8919881044	0.9999537652	-1.0988634516
8.1275610652	0.0739418969	0.9999537652	1.5197241324
2.7933974408	0.6948713406	0.9999537652	0
1.2179411049	0.5346476273	0.9999537652	1.4550617181
-1.5875118025	0.7199976905	0.9999537652	0
-1.4230926939	0.1010844731	0.9999537652	1.0074617456
1.3941100335	0.6692274679	0.9999537652	-2.9208553059
2.6834556635	0.0388636945	0.9999537652	1.0981765084
0	0	0	4.9527501949
-1.0505287927	0.9048217003	0.9999537652	-1.192855091
-1.2496138974	0.1472685176	0.9999537652	1.2032028901
1.3825957569	0.7338372779	0.9999537652	-1.1522914685
2.7933974408	0.6948713406	0.9999537652	0

-1.1257976117	0.8135004768	0.9999537652	1.0342570616
-1.4360591638	0.4556670856	0.9999537652	1.4946811679
-1.269726979	0.2358504639	0.9999537652	-1.0706078211
1.7140555608	0.0620104312	0.9999537652	-1.11911955
-1.1767198936	0.7061023112	0.9999537652	-1.6239558842
-1.0774949743	0.8364460793	0.9999537652	1.01754714
1.3057186386	0.56524369	0.9999537652	-1.8138060591
2.7933974408	0.6948713406	0.9999537652	-1.028941223
1.26183282	0.1475013716	0.9999537652	-1.1777037613
1.2078192865	0.7219347201	0.9999537652	-2.2828512392
1.4047673854	0.3095935217	0.9999537652	-1.4401421966
-1.1200597572	0.8287560869	0.9999537652	-1.6240518608
-1.0502073271	0.911448515	0.9999537652	1.1903887318
1.0038911202	0.9939709317	0.9999537652	1.1582963538
1.1420308345	0.4067854983	0.9999537652	-1.2004190879
-1.0295620502	0.9261914817	0.9999537652	-1.155215744
-1.946370632	0.1857990005	0.9999537652	1.7624065711
-1.1749547935	0.7201653657	0.9999537652	-1.3254236684
1.8358183613	0.4505177299	0.9999537652	1.5010966111
-1.0454265369	0.7503460939	0.9999537652	-1.2179168766
-1.0320459637	0.8910176413	0.9999537652	-1.4584524744
2.7933974408	0.6948713406	0.9999537652	2.996475627
1.5634126039	0.0298508151	0.9999537652	-1.3081825729
22.1262177703	0.2270345719	0.9999537652	-1.2304078955
1.0125121839	0.9161591969	0.9999537652	-1.0603586012
-2.0776099901	0.1202710567	0.9999537652	-1.1908228585
-1.5540946092	0.1255678557	0.9999537652	-1.3153942783
0	0	0	-1.1248877899
1.8455489546	0.5920200474	0.9999537652	-2.5258795711
6.3075231393	0.4851371974	0.9999537652	-2.856452037
1.0103673638	0.98726175	0.9999537652	2.4547373682
-1.0546659807	0.8124345292	0.9999537652	-1.3852550781
4.5505976667	0.5557790267	0.9999537652	-5.3583704783
1.0627195518	0.8345296817	0.9999537652	-1.1874473805
0	0	0	2.996475627
1.403089636	0.5620043494	0.9999537652	1.1862018982
2.7933974408	0.6948713406	0.9999537652	-5.3589986948
2.3191715895	0.0182156275	0.9999537652	1.263054002
1.8746911302	0.4092150565	0.9999537652	-2.4225156659
1.0114023889	0.9273043938	0.9999537652	-1.2273960838
-6.9736190158	0.2250321147	0.9999537652	1.4832922388
0	0	0	1.2398837679
1.9050337421	0.494542025	0.9999537652	-1.3110578867
1.214708255	0.5522071292	0.9999537652	-1.0398068364
0	0	0	1.6293799659
1.2159455323	0.3959793839	0.9999537652	-1.1942166979
-1.2137025022	0.7120570405	0.9999537652	-1.0924895181
-1.2416227775	0.0982055852	0.9999537652	-1.2488202409
1.0833905814	0.6304476746	0.9999537652	1.0574714368

1.0104602496	0.9584720489	0.9999537652	-1.0245370182
1.3359180976	0.5596858167	0.9999537652	-1.1468148148
1.0111888127	0.9635469083	0.9999537652	1.208889498
1.0134049843	0.9405641432	0.9999537652	-1.0203406468
-1.0616337161	0.6410566288	0.9999537652	-1.3507383662
-1.2059865214	0.4434670841	0.9999537652	-1.098463088
-1.1575686555	0.7320651848	0.9999537652	1.7404963396
1.1528946509	0.8113509344	0.9999537652	-3.0025255606
15.1008094413	0.2914268618	0.9999537652	-1.1716875133
3.8622037496	0.6191868959	0.9999537652	1.2398837679
1.2351282506	0.4944373567	0.9999537652	-1.0001951152
4.0296627938	0.2233024732	0.9999537652	-1.0970754137
8.064174035	0.4193662786	0.9999537652	1.9432271302
-1.0906160707	0.8545465731	0.9999537652	-1.0791142638
1.1536178917	0.5387970744	0.9999537652	-1.5573397007
1.3462952915	0.5705306309	0.9999537652	1.0674845818
-1.8528879937	0.3353298801	0.9999537652	1.2077225187
-1.0556971843	0.9068945786	0.9999537652	-1.3282920366
-1.1418666168	0.6684034137	0.9999537652	-1.1739369598
1.191852872	0.2616196642	0.9999537652	-1.3773643697
0	0	0	2.996475627
0	0	0	2.996475627
0	0	0	2.9985540909
8.064174035	0.419372449	0.9999537652	6.9133480437
1.5446975432	0.004168	0.9999537652	-1.6739771507
-1.0034984703	0.9930568932	0.9999537652	-1.6840977205
-1.1358958929	0.4695770407	0.9999537652	1.0213832943
-1.0135764785	0.9800334332	0.9999537652	2.0963273302
11.5766527773	0.3454202303	0.9999537652	-1.973750035
-1.2714855078	0.1777945064	0.9999537652	-1.2468811214
2.0174668837	0.3674430785	0.9999537652	-1.1503912392
8.064174035	0.419378629	0.9999537652	1.8398374193
1.3379068959	0.0223798189	0.9999537652	-1.2951345953
1.5345316606	0.5327294726	0.9999537652	-1.1238239289
-1.2630168054	0.1547334515	0.9999537652	-1.1448861352
1.2263937571	0.4663331051	0.9999537652	1.0598382063
1.0151088999	0.9515109293	0.9999537652	-1.221851512
1.8876737135	0.3715147966	0.9999537652	1.1995439467
8.064174035	0.4193848186	0.9999537652	-1.8666954137
-2.8713019239	0.220522412	0.9999537652	2.7467800943
1.0703740035	0.5463030357	0.9999537652	-1.1430760337
-1.2028952075	0.6594774492	0.9999537652	1.06377116
-1.372650131	0.3151606867	0.9999537652	-1.3457634457
1.6884354984	0.1767018446	0.9999537652	-1.4624787082
-1.5881166222	0.7193210141	0.9999537652	-1.3534814645
22.1449503561	0.2294584327	0.9999537652	1.006929937
-3.2106792237	0.2294793867	0.9999537652	2.4534858631
16.8575730017	0.2723402166	0.9999537652	-1.5524433849
0	0	0	-1.4681855619

0	0	0	2.996475627
1.1808340954	0.4745945147	0.9999537652	-1.1855155006
-2.6706739834	0.3784198081	0.9999537652	-1.3740676439
2.3649792899	0.2792305176	0.9999537652	1.1256490283
1.2660358196	0.2182619771	0.9999537652	1.0356929584
17.3472938331	0.2976125552	0.9999537652	-3.8938703542
1.6342376016	0.0965907016	0.9999537652	-1.3176835538
4.5545995984	0.56965377	0.9999537652	6.908559683
-1.1735894744	0.7690124446	0.9999537652	1.0018943605
1.0431844248	0.7228243872	0.9999537652	1.0599028768
-1.0625536321	0.8030798572	0.9999537652	-1.0219813262
1.1482250997	0.4167131705	0.9999537652	-1.1333440103
0	0	0	0
-1.2274037453	0.1116278091	0.9999537652	-1.3500171451
-1.9081560885	0.2284524764	0.9999537652	-1.4813754993
-3.9058478335	0.1063239579	0.9999537652	2.4178802295
1.1146344094	0.8260558799	0.9999537652	1.0465029305
1.349774274	0.2177212472	0.9999537652	1.2184911367
-1.0833031695	0.8538565791	0.9999537652	-1.4499747611
1.0811396674	0.7124205788	0.9999537652	-1.118580505
1.5149858802	0.654746019	0.9999537652	-1.5745581423
3.3030568913	0.292043189	0.9999537652	-1.2941221818
16.8575730017	0.2723530244	0.9999537652	-1.6606876536
4.5505976667	0.5557790267	0.9999537652	1.2701719397
-1.087032714	0.6489355241	0.9999537652	-1.0010582136
-1.0925649741	0.7196320232	0.9999537652	-1.0656730143
1.2041884279	0.7892803866	0.9999537652	1.0684139388
1.0619058455	0.5899662472	0.9999537652	1.0214091119
1.0988575367	0.410735665	0.9999537652	-1.0211450882
1.472645704	0.0506770442	0.9999537652	-1.2159228596
1.6024984014	0.6872458504	0.9999537652	6.9133480437
1.0791547746	0.7194642476	0.9999537652	-1.1138592499
1.034946767	0.9678872064	0.9999537652	1.7671414969
2.1152390369	0.1043860678	0.9999537652	1.1251537086
-1.053450369	0.8857010664	0.9999537652	-1.228582504
3.0628221549	0.3120234743	0.9999537652	-1.986027526
-1.3341494051	0.1741476301	0.9999537652	-1.4612467315
1.3786410676	0.035938852	0.9999537652	-1.2724516274
1.2760040591	0.15144539	0.9999537652	-1.5292905358
1.2416309862	0.7533444088	0.9999537652	1.0056391713
6.3075231393	0.4851277785	0.9999537652	-1.1629386999
2.0482638049	0.2100977941	0.9999537652	1.1177297064
-1.4750790135	0.6603393192	0.9999537652	1.01694521
1.2147136981	0.4773590211	0.9999537652	1.0506234898
1.0895583843	0.6845624392	0.9999537652	-1.2154636547
-1.6580866023	0.2005178564	0.9999537652	-1.0905508353
1.8131227004	0.33088475	0.9999537652	1.5599463705
-1.1794522573	0.7359752204	0.9999537652	1.6316059346
1.0162548412	0.9686288465	0.9999537652	1.7603231865

1.0042347517	0.9748071447	0.9999537652	-1.0696040602
-1.3457716089	0.1051554557	0.9999537652	-1.0749133662
1.1473122003	0.5354836739	0.9999537652	-1.2368306743
1.1072399635	0.7041248674	0.9999537652	1.0368273147
4.5505976667	0.5557790267	0.9999537652	10.818785458
2.7933974408	0.6948713406	0.9999537652	26.4594426731
1.5232415062	0.2494938683	0.9999537652	-1.286466914
-1.058716223	0.8021355877	0.9999537652	1.3182158476
2.7933974408	0.6948713406	0.9999537652	1.4539570206
-1.0821001117	0.7554446584	0.9999537652	1.1238234418
-1.2591700515	0.6875137367	0.9999537652	2.4197893354
4.5545995984	0.569651693	0.9999537652	-5.3589986948
1.1158299481	0.9297712578	0.9999537652	-1.0368675441
-1.368560196	0.7386883633	0.9999537652	-1.9748749398
2.4609030837	0.2530777529	0.9999537652	2.8349310674
-1.0671293958	0.8608734643	0.9999537652	1.2547898049
1.0124541275	0.9326895418	0.9999537652	-1.2597507555
34.4101428749	0.1668536206	0.9999537652	2.3408023448
-1.1444309547	0.4575339	0.9999537652	1.2058662082
0	0	0	1.0031259015
9.8205505243	0.3838237526	0.9999537652	2.030756821
1.6740429925	0.425398107	0.9999537652	-1.1330359562
-3.9820556893	0.0983103654	0.9999537652	4.9561842702
1.2815750993	0.6476708528	0.9999537652	-1.0251004176
-1.0699381068	0.720306221	0.9999537652	1.1022438407
6.3075231393	0.4851183669	0.9999537652	-3.3826461929
0	0	0	-1.1558452152
1.6681177207	0.454544333	0.9999537652	1.716905782
1.0678877326	0.6533519617	0.9999537652	1.0955843554
2.0765723329	0.1342061663	0.9999537652	-1.5303069064
1.0328500538	0.8353845588	0.9999537652	1.0580628577
-1.351636497	0.4106203445	0.9999537652	-1.0589605593
8.064174035	0.4193910179	0.9999537652	-3.384595918
-1.1374032486	0.6752078934	0.9999537652	1.0890568721
1.418815938	0.1040220559	0.9999537652	1.3021853207
-1.8466138227	0.3718389628	0.9999537652	1.278693586
1.3681518085	0.0921915721	0.9999537652	-1.0152952664
-1.1643625881	0.5267268735	0.9999537652	1.5139994866
1.0148519638	0.9659559366	0.9999537652	1.2343188059
-2.5871290907	0.5000781443	0.9999537652	2.9940379764
2.0389656363	0.4520656635	0.9999537652	-1.7020755295
1.1128663284	0.6957180392	0.9999537652	1.3128693803
4.5505976667	0.5557790267	0.9999537652	-1.1632104493
1.050720514	0.9329313325	0.9999537652	-1.5282263687
-1.2396734609	0.7406910918	0.9999537652	2.9972756496
-1.010418368	0.9884526747	0.9999537652	1.4002700172
1.5170591203	0.3314241187	0.9999537652	2.2232514773
-1.2793488238	0.6762444968	0.9999537652	1.5101339254
2.6814021431	0.1540617049	0.9999537652	-1.2307795458

-1.0290366928	0.8498063274	0.9999537652	-1.2681670258
1.6837314654	0.2969691608	0.9999537652	-1.0216214608
-1.1045603102	0.6255779581	0.9999537652	1.2064761473
0	0	0	2.9985540909
11.5766527773	0.3454186666	0.9999537652	-5.3583704783
4.5505976667	0.5557790267	0.9999537652	1.2676116495
4.5545995984	0.5696496161	0.9999537652	-2.5900575084
1.1701996185	0.4989884039	0.9999537652	-1.1769058092
-1.2436434669	0.606668197	0.9999537652	-1.222629269
1.2215679218	0.759946732	0.9999537652	-1.0363551544
-1.258552828	0.6727634901	0.9999537652	1.8167613353
2.7933974408	0.6948713406	0.9999537652	-3.384595918
-1.1091397827	0.7635448436	0.9999537652	-1.8842864578
-1.3197821904	0.6507559826	0.9999537652	-1.7033379786
5.8024455297	0.2158134709	0.9999537652	0
1.2350972138	0.6116303428	0.9999537652	1.1637628085
0	0	0	-2.2890929548
0	0	0	1.0682244185
-1.586167124	0.7108672696	0.9999537652	6.1545167512
2.7933974408	0.6948713406	0.9999537652	-5.3589986948
1.1272401212	0.8389811038	0.9999537652	1.2815412708
1.4696819851	0.3458444478	0.9999537652	1.3052581491
2.7933974408	0.6948713406	0.9999537652	0
0	0	0	12.3846842502
1.1305291038	0.8399998973	0.9999537652	3.6976146343
1.2100129772	0.212838484	0.9999537652	-1.0124907198
6.3075231393	0.4851089626	0.9999537652	0
-1.1134398134	0.7427416875	0.9999537652	1.0634834426
1.037711622	0.8338821169	0.9999537652	-1.1947369696
1.3575279261	0.1970311382	0.9999537652	-1.2021903268
-1.1617983164	0.3785074407	0.9999537652	-1.1148147701
1.9664475558	0.1117751963	0.9999537652	-1.2107413166
-1.0409763036	0.9352214459	0.9999537652	1.1836307135
-1.7140706619	0.5271586641	0.9999537652	-1.0625358683
-1.034717661	0.8544063116	0.9999537652	-1.1623795131
1.180187422	0.2983911583	0.9999537652	-1.1314673151
-1.0197466857	0.882047514	0.9999537652	-1.2037086373
15.0880352532	0.2935756102	0.9999537652	-1.1503049253
-1.5428233137	0.4119504765	0.9999537652	-1.373027903
0	0	0	6.908559683
0	0	0	2.996475627
1.3826895979	0.0539450536	0.9999537652	-1.1488026002
-1.393435122	0.6849930345	0.9999537652	-1.647673557
3.3045913726	0.283082629	0.9999537652	1.0165948208
-1.1878405995	0.6481791441	0.9999537652	-1.4369273505
1.3594099105	0.7965814627	0.9999537652	1.3448856344
-1.4026939728	0.0530130199	0.9999537652	-1.2313618149
-1.5875118025	0.7198552203	0.9999537652	-2.839009059
2.1973466799	0.2904785797	0.9999537652	1.1103092415

8.064174035	0.4193972267	0.9999537652	6.9133480437
3.8622037496	0.6191559473	0.9999537652	0
2.7933974408	0.6948713406	0.9999537652	4.9527501949
4.5505976667	0.5557790267	0.9999537652	0
-3.8114682973	0.0471558261	0.9999537652	3.1880366645
-1.1443156703	0.3480868913	0.9999537652	1.1260203733
-20.4075039392	0.0218955863	0.9999537652	24.9357983742
1.234311755	0.4790701567	0.9999537652	-1.0006916416
-1.2301913722	0.7670414376	0.9999537652	-1.9665272784
1.1958895821	0.8153214677	0.9999537652	-1.5275589953
-1.3187888541	0.1348769593	0.9999537652	-1.5827577202
-1.6424310402	0.4233747791	0.9999537652	-1.230245554
1.2529672008	0.2358517993	0.9999537652	-1.279781972
1.0188924992	0.9296854887	0.9999537652	1.099545523
-1.1708143936	0.8340608861	0.9999537652	1.3324118025
1.5426605882	0.2026257795	0.9999537652	1.8627466464
0	0	0	-1.1102514825
-3.1203910316	0.2339919831	0.9999537652	-1.4978192929
-1.0575035506	0.8875768674	0.9999537652	-1.4887243938
-1.0534540998	0.6781889754	0.9999537652	-1.3790135508
1.0443783564	0.8441914576	0.9999537652	1.3213255487
16.8433158146	0.2737991955	0.9999537652	39.4416233421
-1.2619381918	0.6777884501	0.9999537652	-1.3086774402
-1.1460379741	0.5969353735	0.9999537652	-1.4044603147
0	0	0	8.8761957907
-1.6994504307	0.5419238448	0.9999537652	2.4596721034
-1.0583261614	0.9255582372	0.9999537652	1.6669149077
1.0828767608	0.5112206895	0.9999537652	1.0774153077
-1.3444413857	0.0519313115	0.9999537652	-1.1574012797
1.0702722079	0.8551624179	0.9999537652	1.1318700978
1.143529603	0.7243918451	0.9999537652	-1.3121044357
2.044883541	0.1864114465	0.9999537652	-2.2138120489
0	0	0	33.3754495834
1.2302887969	0.5310426177	0.9999537652	-1.4416799564
-1.343917123	0.3988303718	0.9999537652	-1.5448580467
-1.4662362166	0.1433122262	0.9999537652	-1.5492751053
1.7682213732	0.3139689864	0.9999537652	1.6362770216
1.6180778276	0.2487375003	0.9999537652	1.0790227481
-1.1447852916	0.9152929442	0.9999537652	18.6486518258
-1.0463517569	0.8554793608	0.9999537652	1.0651198214
1.3007488027	0.3993948277	0.9999537652	-1.1411165095
1.9058703168	0.4679276633	0.9999537652	1.9844330535
1.0468975627	0.7464121219	0.9999537652	-1.2154533569
1.0631479972	0.7851098446	0.9999537652	1.0571125433
-1.3685950045	0.7393763031	0.9999537652	1.2398837679
-1.5861169239	0.7137687045	0.9999537652	0
1.1451126686	0.5005121455	0.9999537652	-1.1039793627
-1.250682192	0.3186708398	0.9999537652	1.0416843349
1.3241821346	0.1993293329	0.9999537652	1.0036840783

1.6626752525	0.5077518681	0.9999537652	-2.0129022119
0	0	0	15.7262184124
0	0	0	2.996475627
1.1162834585	0.9281644509	0.9999537652	12.7909013375
-1.7819050662	0.0779101934	0.9999537652	1.2384480575
2.7933974408	0.6948713406	0.9999537652	2.996475627
-1.5875118025	0.7201250889	0.9999537652	1.1034199328
-1.2429919493	0.5566467392	0.9999537652	1.0393772297
-1.3116175838	0.1950619506	0.9999537652	-1.3102788073
-1.4622790761	0.3932923345	0.9999537652	1.0897767381
1.0561264997	0.8875920417	0.9999537652	-1.5045819864
-1.0228110582	0.9555325496	0.9999537652	1.1982721806
1.1141578549	0.7726968874	0.9999537652	-1.1926011489
2.7933974408	0.6948713406	0.9999537652	0
8.064174035	0.4194034453	0.9999537652	10.8337816869
1.5031431015	0.2194430396	0.9999537652	1.0670865011
1.438563165	0.2491635166	0.9999537652	1.0491812593
-1.8846957778	0.1771403868	0.9999537652	-2.0193672047
-1.4227986927	0.2954201233	0.9999537652	-1.1060533884
-1.6056235628	0.4468273523	0.9999537652	1.2740027272
-1.4962412698	0.3576504131	0.9999537652	1.0183769815
-1.1589122199	0.71798473	0.9999537652	-1.2095148632
-1.5287863393	0.4981532473	0.9999537652	-1.1682882678
1.2777749361	0.677384887	0.9999537652	1.5744318196
1.1260260935	0.8880845748	0.9999537652	-1.7076891067
1.7936180972	0.6488788917	0.9999537652	-5.7442173985
2.3466011022	0.2311108724	0.9999537652	1.0930472557
1.6024984014	0.6872276041	0.9999537652	1.590997316
-1.7164821341	0.1942706	0.9999537652	1.4244023721
-1.0552135611	0.8731965347	0.9999537652	2.5459274337
1.0619782594	0.8731530096	0.9999537652	-1.0249966481
-1.0102815366	0.9857996617	0.9999537652	1.1454461625
-2.1299793114	0.4794574753	0.9999537652	-5.3583704783
2.7957660672	0.7055140695	0.9999537652	0
4.5505976667	0.5557790267	0.9999537652	14.7475552392
0	0	0	0
6.3128691894	0.4808030983	0.9999537652	0
1.1871343615	0.4870818116	0.9999537652	1.3924284007
1.6827116179	0.2817949364	0.9999537652	-1.5267058412
1.1230829931	0.9008178848	0.9999537652	-1.6856836243
2.7558710195	0.1132540706	0.9999537652	1.2176277036
1.4900223999	0.2338840004	0.9999537652	-1.5686003931
-1.1276081398	0.8489218316	0.9999537652	1.0967635494
0	0	0	-2.4867043709
1.0123914647	0.915927378	0.9999537652	1.0132432847
0	0	0	2.996475627
-1.2204550169	0.4715208525	0.9999537652	-1.3859533801
-4.815549291	0.1727185641	0.9999537652	-1.313384656
-2.3372359995	0.2552173964	0.9999537652	2.8644035913

3.5991848458	0.0150765676	0.9999537652	-1.0503184456
1.022832683	0.9685671225	0.9999537652	-1.1440555758
-1.0985917701	0.7321182608	0.9999537652	-1.2190139265
1.1526842441	0.6560572327	0.9999537652	-1.3018392326
1.0996862317	0.5283122123	0.9999537652	-1.0151132918
-1.1253017056	0.4644358313	0.9999537652	-1.1764330945
1.1403316133	0.4457765861	0.9999537652	1.2664733768
1.9075275807	0.1947299963	0.9999537652	-1.1016246679
1.1264016939	0.7479180542	0.9999537652	-1.0503566672
1.0974854953	0.5252282463	0.9999537652	1.1020293836
1.1970988109	0.3704267018	0.9999537652	-1.2146438781
8.0710074692	0.4204322566	0.9999537652	6.908559683
0	0	0	1.6293799659
-1.0574860508	0.7014151972	0.9999537652	-1.0843215192
1.2196782492	0.1926009716	0.9999537652	1.0426769517
1.0516386376	0.7615483051	0.9999537652	1.0441694229
1.1278567131	0.5110405008	0.9999537652	1.0063939625
1.2462745639	0.2399338997	0.9999537652	1.0730767346
2.7957660672	0.7055164449	0.9999537652	0
1.3199290799	0.3353047644	0.9999537652	1.0242896905
9.8205505243	0.3838105254	0.9999537652	1.3346184872
1.1198932801	0.4470728356	0.9999537652	1.0507527637
8.064174035	0.4194096735	0.9999537652	-3.466758885
-15.9195842901	0.1124602651	0.9999537652	1.591370391
17.3472938331	0.297631354	0.9999537652	-1.8594983184
1.0763924171	0.8875330403	0.9999537652	1.0150552869
-1.0455811674	0.7204028955	0.9999537652	-1.1559676665
1.0650141675	0.6777655347	0.9999537652	-1.0939699307
1.6032041415	0.6834392951	0.9999537652	1.4861911385
1.1286287893	0.6240548072	0.9999537652	-1.11180564
1.1582396193	0.6922576739	0.9999537652	-3.2663345239
1.5385036419	0.4656340502	0.9999537652	-1.4597659263
-2.2763224034	0.2504844353	0.9999537652	-1.1460915129
-1.0276953307	0.8515367388	0.9999537652	-1.0805217072
1.3588921917	0.7999684864	0.9999537652	1.0931805911
-1.1447836674	0.4429012246	0.9999537652	-1.1317327102
1.6240583657	0.2682060611	0.9999537652	-1.3452041151
1.063401246	0.8425790836	0.9999537652	2.0339270487
9.8205505243	0.3837972826	0.9999537652	0
1.1746478082	0.2217223423	0.9999537652	-1.0537513329
-1.6215746016	0.1442955364	0.9999537652	-1.2351710175
-1.0901760975	0.4968462079	0.9999537652	-1.0196289272
-1.0770850968	0.6209982721	0.9999537652	-1.224794066
1.9363887703	0.3827482325	0.9999537652	-1.2625853971
2.7933974408	0.6948713406	0.9999537652	0
2.503478678	0.001207	0.9999537652	-1.5857625604
-1.2130974397	0.6180421296	0.9999537652	-1.5062569855
-1.0761575667	0.7038195067	0.9999537652	-1.0644282598
-6.9762758667	0.2571471921	0.9999537652	-1.2195867056

-1.0074268848	0.9707523111	0.9999537652	-1.128215671
-1.9189881436	0.0576963724	0.9999537652	-1.4176445132
1.2223195987	0.2488037169	0.9999537652	1.4528456691
-1.5756849726	0.5089496494	0.9999537652	-1.6404518061
-1.3969646988	0.0744918133	0.9999537652	-1.2880112974
1.0000079996	0.9999537652	0.9999537652	-1.4941378965
1.7202565997	0.3014390102	0.9999537652	-2.6671091064
-1.2817623199	0.5548950806	0.9999537652	-1.2983560702
-1.5092651006	0.0305964462	0.9999537652	-1.0820260997
9.8035423414	0.0452593727	0.9999537652	1.1350054982
-1.3027991995	0.755731467	0.9999537652	-1.8225907301
1.0635479941	0.7178648941	0.9999537652	-1.2017437107
2.7957660672	0.7055188213	0.9999537652	2.996475627
1.2067548981	0.2185227984	0.9999537652	-1.0061288284
-1.0748561648	0.6285007671	0.9999537652	-1.1042055875
1.8100377663	0.0773764102	0.9999537652	1.0199227096
1.0230551597	0.8875242695	0.9999537652	-1.0649719987
1.2092843852	0.1475182035	0.9999537652	1.2365590694
-1.1553756775	0.402176897	0.9999537652	-1.2651583243
-1.1300185227	0.7420936806	0.9999537652	-1.1417751321
0	0	0	0
1.1540990034	0.3339886906	0.9999537652	1.1509487343
-1.1101003444	0.429584814	0.9999537652	-1.0976867685
-1.5884256519	0.0315694733	0.9999537652	1.0427096745
1.0746557004	0.5757493135	0.9999537652	1.0859390291
1.0826469763	0.556653833	0.9999537652	1.3791914575
1.0018197503	0.9926551943	0.9999537652	-1.1782355431
1.0858476403	0.6543052954	0.9999537652	-1.1853810054
-1.3057742179	0.3886432643	0.9999537652	1.014076988
1.1172293767	0.9254364973	0.9999537652	1.4542901762
-1.4276367323	0.0931856606	0.9999537652	-1.121967289
-1.0300139697	0.8909425105	0.9999537652	1.1532207259
1.235610588	0.1352873566	0.9999537652	1.2708917827
1.0671862706	0.814487559	0.9999537652	1.0695119091
-1.0836475261	0.6480208417	0.9999537652	1.1046062877
-1.032302797	0.883744751	0.9999537652	-1.1501726585
-2.5861438062	0.4748939165	0.9999537652	1.2593062028
2.2179001574	0.0375169104	0.9999537652	-1.1771623374
0	0	0	0
0	0	0	2.996475627
-1.2011850916	0.1966889454	0.9999537652	1.0606288651
1.2995020274	0.4206388496	0.9999537652	1.253332609
1.2992150871	0.506780862	0.9999537652	-1.2679466736
8.0710074692	0.4204583208	0.9999537652	0
0	0	0	1.4542901762
-1.5121416779	0.3398933898	0.9999537652	-1.1888310928
-1.369198268	0.7561307866	0.9999537652	15.9061931258
1.0290859244	0.8704116947	0.9999537652	1.363360292
-1.0487872257	0.9298568754	0.9999537652	1.4604703382

0	0	0	-1.1242401582
-2.4110939284	0.0688889082	0.9999537652	-1.3806498298
-1.1494001102	0.775929489	0.9999537652	1.111601668
-1.1351150408	0.3332621682	0.9999537652	1.1186276713
0	0	0	8.8639046058
-1.1118908168	0.8887540616	0.9999537652	1.5233400822
-1.0681040278	0.6401932637	0.9999537652	1.1447909569
1.1329956281	0.7164308121	0.9999537652	1.0463220163
1.4607525075	0.0414051065	0.9999537652	-1.0725439861
-1.2589896649	0.3160518102	0.9999537652	1.0108182692
0	0	0	4.9527501949
-2.7245308968	0.0490546101	0.9999537652	1.5015099363
-1.3002840432	0.5211566511	0.9999537652	-1.3926400799
2.0602460006	0.2287587571	0.9999537652	-1.0705926724
23.8818846228	0.2167080871	0.9999537652	-1.1002339347
-1.4015977922	0.3500050829	0.9999537652	1.9672458041
1.5203380125	0.4686723305	0.9999537652	-1.3247783493
1.1035024722	0.59597711	0.9999537652	1.2672161823
-1.2707862961	0.1236107298	0.9999537652	-1.0881618889
0	0	0	2.996475627
-1.0490673243	0.922034677	0.9999537652	-1.044213841
-3.4472269618	0.076553139	0.9999537652	-1.0624502046
1.0932997774	0.5445347426	0.9999537652	1.162616109
1.6351118749	0.0866004483	0.9999537652	-1.131412753
-1.0404989372	0.953295809	0.9999537652	1.411790598
9.8288704632	0.3801114215	0.9999537652	4.0976538475
-1.3248480218	0.1669545853	0.9999537652	-1.0383220698
1.577928447	0.0757006282	0.9999537652	1.0518861416
-1.063334962	0.7829991275	0.9999537652	1.2034762313
3.20757305	0.0408121082	0.9999537652	2.4244866223
-1.0137378958	0.9116254718	0.9999537652	-1.0162274382
-1.1217386794	0.43260323	0.9999537652	1.0436080625
-1.0033742971	0.9905676432	0.9999537652	1.1051263322
-1.0713263371	0.906127381	0.9999537652	-1.35243166
1.2234698515	0.3239584882	0.9999537652	-1.3790493137
-1.2999419354	0.8180884024	0.9999537652	-5.6211326582
4.5545995984	0.5696475393	0.9999537652	8.9512754559
1.1035339639	0.4296512037	0.9999537652	1.012733769
-1.074051266	0.5664675074	0.9999537652	-1.3345315783
-1.1317616859	0.4327208937	0.9999537652	-1.4438309328
-1.216923946	0.3694527347	0.9999537652	-1.1913658023
1.3773829257	0.5420393207	0.9999537652	1.0603226543
1.0975231782	0.5621739622	0.9999537652	-1.3701244318
-1.1407223098	0.3640964219	0.9999537652	1.0288423649
8.064174035	0.4194159115	0.9999537652	2.0149727286
-1.4368650953	0.0914893129	0.9999537652	-1.2626240279
1.7226500049	0.202782067	0.9999537652	-1.6367727885
-1.1099831564	0.4985317761	0.9999537652	1.2152624187
2.0382505185	0.4245727514	0.9999537652	-2.0725678067

2.5912735708	0.1170578798	0.9999537652	-1.7801628221
6.3128691894	0.4807107715	0.9999537652	0
-1.4297904902	0.1796939141	0.9999537652	1.5193693651
-1.0109448648	0.936742971	0.9999537652	-1.0673048431
-3.8588403224	0.1581097392	0.9999537652	8.8639046058
0	0	0	2.996475627
1.2526375214	0.8108859998	0.9999537652	-3.0936259592
-1.1134750962	0.5236329628	0.9999537652	-1.496047969
0	0	0	2.9985540909
-1.5881570201	0.7078287578	0.9999537652	-5.0090574362
1.2482257223	0.2179162198	0.9999537652	1.1009373405
1.1948778953	0.5569393931	0.9999537652	-1.0167790123
-1.0723427307	0.7541048601	0.9999537652	-1.0449644252
-1.6869835841	0.004008	0.9999537652	-1.1410506349
1.0989597165	0.7507606093	0.9999537652	-1.0704217672
-1.1484450308	0.4246489156	0.9999537652	-1.2171499378
1.0552506149	0.8690707636	0.9999537652	-1.1257715842
1.0010482845	0.9942844235	0.9999537652	-1.0308539013
-1.8701378137	0.0784795006	0.9999537652	-1.2507755863
1.0205474525	0.8942602419	0.9999537652	-1.0708880499
6.3075231393	0.4850995655	0.9999537652	-3.3853891979
1.5753996545	0.0407950023	0.9999537652	-1.0224425695
-1.0690912086	0.8763022403	0.9999537652	-1.6361884696
-1.1766116099	0.5390035096	0.9999537652	-1.0800969431
-1.2326976539	0.7443243936	0.9999537652	1.0595374479
-2.5839527681	0.5181115426	0.9999537652	1.7042814026
1.1601436399	0.4882118628	0.9999537652	-1.4476027836
-1.2961143902	0.6360027832	0.9999537652	-1.3668316439
1.276248929	0.1140034765	0.9999537652	1.4988080172
1.0858800109	0.5718292522	0.9999537652	-1.0573232475
1.0903986127	0.6587323632	0.9999537652	-1.383011595
-1.0433941465	0.7256760764	0.9999537652	-1.0366553349
1.2014276949	0.5011945733	0.9999537652	1.2969643111
-1.1241198887	0.3991304618	0.9999537652	1.0845090638
1.164250034	0.6797559362	0.9999537652	-1.0970976532
0	0	0	-1.0727505572
-1.1620890089	0.4695622473	0.9999537652	-1.1906504097
4.5545995984	0.5696454625	0.9999537652	1.8468806494
-1.2975978325	0.4275321118	0.9999537652	-1.4397922351
-1.7044043661	0.1989076531	0.9999537652	1.2371045246
-1.0338564951	0.8646373666	0.9999537652	1.1907047903
1.1699151473	0.6464590602	0.9999537652	1.0386707371
-1.0819818974	0.7771607755	0.9999537652	1.2395491944
-1.1029907237	0.6497798995	0.9999537652	-1.0176857649
-1.0040388734	0.9904739495	0.9999537652	1.0176928704
-1.3363021608	0.3522081585	0.9999537652	-1.0339503721
1.2951200919	0.4931517382	0.9999537652	1.0825176271
1.0583880499	0.7068505961	0.9999537652	-1.1565164157
-1.0786812601	0.72602947	0.9999537652	-1.0449164364

2.7933974408	0.6948713406	0.9999537652	8.8700459102
1.0804595421	0.7378009485	0.9999537652	-1.0979409177
1.0795517636	0.6744844578	0.9999537652	-1.1121025595
1.6046550712	0.6810120677	0.9999537652	-1.2749878971
1.1212392628	0.5421465795	0.9999537652	-1.0023651245
-1.5394092355	0.0271733208	0.9999537652	-1.6158088087
-1.1390108139	0.6186677603	0.9999537652	-1.275526239
-1.007454716	0.9530442058	0.9999537652	-1.071930311
-1.0224441726	0.8606037291	0.9999537652	1.0578255187
-1.1184676139	0.5109886106	0.9999537652	-1.0140341426
1.1170153023	0.6233778091	0.9999537652	-1.0005956804
0	0	0	6.908559683
-1.7491145644	0.2767222645	0.9999537652	1.1338940997
1.1883473308	0.4316586356	0.9999537652	1.196382233
1.0541171147	0.8781912007	0.9999537652	-1.0711004689
-1.1264949047	0.6473203794	0.9999537652	-1.4706253601
-1.1387882964	0.3425324947	0.9999537652	1.1119624704
1.6368695344	0.2654456881	0.9999537652	1.1553495621
1.0285188052	0.8789907155	0.9999537652	-1.2387413167
-1.0143615881	0.9367861037	0.9999537652	1.0320238442
-1.103045968	0.8197984048	0.9999537652	1.2541909535
2.4683706953	0.2710146482	0.9999537652	-1.3712612654
1.1221638072	0.9024799169	0.9999537652	2.2717261431
-1.0537456786	0.7911024955	0.9999537652	-1.0435090838
1.4030593221	0.5131397053	0.9999537652	-1.3213352774
1.0331225636	0.823902041	0.9999537652	1.1432365567
1.2384590172	0.6360278263	0.9999537652	1.2690896005
-1.2168566013	0.3846209613	0.9999537652	1.3330102335
-1.2457534506	0.3163792637	0.9999537652	-1.1473129108
0	0	0	4.9527501949
1.4785821667	0.2954671978	0.9999537652	-1.1001091216
-1.2272625392	0.5431415494	0.9999537652	1.4343081104
6.3075231393	0.4850901757	0.9999537652	-1.859280336
4.5505976667	0.5557790267	0.9999537652	14.7271569203
1.1249057005	0.754500404	0.9999537652	1.2179128543
0	0	0	1.590997316
1.4268022358	0.2422845296	0.9999537652	-1.2652214082
-1.2182878375	0.5590720069	0.9999537652	1.2350889974
-1.2296455092	0.7706709362	0.9999537652	-4.7716407405
-1.2262293166	0.2877521498	0.9999537652	-1.0866535397
1.1854845115	0.6763969274	0.9999537652	-1.2421176396
2.204523133	0.0938901638	0.9999537652	-1.0956458214
1.0551815701	0.7465789438	0.9999537652	1.289175658
1.3136596018	0.4583011223	0.9999537652	-1.3277209335
-1.0435274445	0.8922441435	0.9999537652	-1.1127572418
1.4742536511	0.376944677	0.9999537652	-1.0420566599
-1.010457517	0.9368049184	0.9999537652	-1.1573771318
1.6386722747	0.3726583339	0.9999537652	-1.1249021223
16.8433158146	0.2738688772	0.9999537652	-1.9435868636

-1.1758790375	0.5376095513	0.9999537652	1.0474221133
1.237653776	0.1451258348	0.9999537652	-1.6910220418
-2.2765895442	0.2466976634	0.9999537652	-1.4504059134
15.0880352532	0.2934043377	0.9999537652	-2.0131031796
-1.0933012256	0.777593082	0.9999537652	-1.0690661743
4.5505976667	0.5557790267	0.9999537652	26.4777255918
-1.0085355311	0.9930378932	0.9999537652	1.9432271302
29.8698312703	0.2178162544	0.9999537652	-2.0477227478
-1.5381739117	0.237480494	0.9999537652	-1.4741003796
-1.2383574185	0.2519178174	0.9999537652	-1.1485322747
1.5389507255	0.4921199992	0.9999537652	-1.2721024789
1.132735359	0.4839367451	0.9999537652	1.0004381978
0	0	0	1.5908108123
1.0846676983	0.4905809627	0.9999537652	1.1825084201
-1.668764792	0.6521049629	0.9999537652	1.4380982837
-1.0556403821	0.7527330003	0.9999537652	-1.1992166791
-1.0317298804	0.8696656408	0.9999537652	-1.1374369046
1.4163115192	0.2636769146	0.9999537652	1.0062753509
1.9066445334	0.4740584396	0.9999537652	1.5537663683
1.3400712069	0.1854412337	0.9999537652	-1.3590216973
-1.4270565985	0.2158159237	0.9999537652	1.0926621754
-1.1447490371	0.9152039999	0.9999537652	-17.3329126105
4.5166078038	0.1766694351	0.9999537652	2.996475627
1.1209420802	0.4093111056	0.9999537652	-1.1120096618
2.7933974408	0.6948713406	0.9999537652	0
0	0	0	2.996475627
0	0	0	1.590997316
1.8844843324	0.005516	0.9999537652	-1.0394786074
4.5115277415	0.079829615	0.9999537652	1.1904248789
-1.1123231904	0.7564494424	0.9999537652	-1.3226713131
1.8585605296	0.1812392074	0.9999537652	1.2565167478
1.4956507458	0.4398248268	0.9999537652	-1.4244126558
1.276812955	0.4812003383	0.9999537652	-1.3176374119
2.7933974408	0.6948713406	0.9999537652	-1.1503049253
-1.1048877431	0.5739081598	0.9999537652	1.2346315359
2.7520178873	0.1481642386	0.9999537652	-1.2587979171
-2.5871290907	0.5000688344	0.9999537652	3.3445905597
-1.7652571835	0.0659506084	0.9999537652	-1.176385356
-1.0653664483	0.8465143199	0.9999537652	-1.0018310437
4.6654312047	0.0004305	0.9999537652	1.594530159
1.3208497255	0.518052141	0.9999537652	-1.0846843115
-1.4149785476	0.1218778756	0.9999537652	-1.3994497245
0	0	0	2.996475627
2.7957660672	0.7055235769	0.9999537652	-1.2214576418
1.0408601071	0.8297347817	0.9999537652	-1.1990449332
1.0566150785	0.8640192865	0.9999537652	1.0499867029
1.7738745164	0.5343515944	0.9999537652	18.6336779883
-1.0391372056	0.9528707401	0.9999537652	1.3234054731
1.2491784013	0.3297271378	0.9999537652	1.2046928615

2.0885615936	0.5185160385	0.9999537652	-1.0601718512
-1.0245769117	0.9195673649	0.9999537652	-1.097279382
-1.3912856256	0.4180925669	0.9999537652	1.007010439
85.6500119782	0.0835799918	0.9999537652	2.5233586594
1.3172108461	0.1425652383	0.9999537652	-1.1218893772
2.3461886365	0.008532	0.9999537652	-1.3304834327
-1.2410216542	0.1174367276	0.9999537652	-1.090670444
-1.11819709	0.4550548529	0.9999537652	1.0494952079
1.1916669781	0.2585188334	0.9999537652	-1.3234395348
1.3024093443	0.2726204517	0.9999537652	1.0119529652
0	0	0	2.9985540909
-1.4034442924	0.0328407811	0.9999537652	-1.0236311959
-1.0103773406	0.9733064539	0.9999537652	-1.4859171843
-1.2477568887	0.5447656874	0.9999537652	-1.3326624714
-1.0362145677	0.8452332041	0.9999537652	-1.1686962495
1.4205746494	0.476038906	0.9999537652	-1.0425181343
1.1317396891	0.5333938925	0.9999537652	1.132326217
-1.8332325169	0.2547506675	0.9999537652	-1.0432965625
2.2198211934	0.0917975693	0.9999537652	1.2049119316
-1.0466832537	0.8303413964	0.9999537652	1.1808107261
1.1167754821	0.9277512034	0.9999537652	-1.5087935345
1.1939731432	0.2851455618	0.9999537652	-1.4982330779
-2.7740269098	0.2151947459	0.9999537652	-2.5763095902
1.6710714369	0.4504946823	0.9999537652	1.2268431205
1.8455489546	0.5920264881	0.9999537652	-1.0953721119
-1.0887021823	0.5646330745	0.9999537652	-1.0636099664
-1.0542443527	0.7967303187	0.9999537652	1.5211085816
1.2316932874	0.6543414355	0.9999537652	1.2806145297
-1.0820712914	0.6980781435	0.9999537652	-1.1421430704
1.1902735921	0.6836860152	0.9999537652	-1.5242622678
2.7933974408	0.6948713406	0.9999537652	2.996475627
1.0484842868	0.8777552251	0.9999537652	-1.1450238802
-1.1605169044	0.8770226491	0.9999537652	1.6464401882
1.5385690594	0.0558287068	0.9999537652	1.35499568
2.7933974408	0.6948713406	0.9999537652	0
1.3215409268	0.1721006838	0.9999537652	1.3363100178
1.416057543	0.1690519695	0.9999537652	1.067283092
1.0774360859	0.7019347639	0.9999537652	-1.015007029
-1.4354957421	0.2144268943	0.9999537652	-1.1506170694
11.5766527773	0.3454083239	0.9999537652	1.2698746349
-6.6829003845	8.30E-05	0.6382149566	-2.0277560291
1.7785996228	0.003753	0.9999537652	1.0415475608
11.5966493128	0.3442508969	0.9999537652	3.9662067717
0	0	0	-2.6269410807
30.8757261591	0.1832471885	0.9999537652	-1.8686445933
-1.0102577328	0.9453702519	0.9999537652	-1.1344574504
-1.1222924563	0.3584395646	0.9999537652	-1.0222572477
0	0	0	6.908559683
-1.0942568408	0.8334457746	0.9999537652	-1.7104086043

-1.1154970392	0.6175770483	0.9999537652	1.1882547662
-1.1365734422	0.5062325472	0.9999537652	-1.1544245831
1.1736492426	0.5731223124	0.9999537652	-1.2093051167
1.034927222	0.8565418249	0.9999537652	-1.3219532138
-1.037288508	0.8107260933	0.9999537652	1.5944967176
1.0633911734	0.7359435553	0.9999537652	1.047425354
-1.0112420483	0.9844098963	0.9999537652	1.130545974
-1.1457555741	0.9105882642	0.9999537652	1.3730979101
-1.1207926898	0.3352555101	0.9999537652	1.0579882161
-1.1579031467	0.4778520844	0.9999537652	1.0277782216
1.8471118167	0.5851526856	0.9999537652	-5.3589986948
-1.0399132936	0.8308670093	0.9999537652	-1.1159147497
1.1484783125	0.7717381669	0.9999537652	-1.0614166346
1.0597727521	0.7466420066	0.9999537652	1.0411535348
-1.1017436422	0.8220930162	0.9999537652	1.0863300677
13.3324809638	0.3193495606	0.9999537652	-1.9860002794
1.1116123432	0.7564539241	0.9999537652	-1.0542036433
0	0	0	-1.3581071904
-1.777015156	0.0644868647	0.9999537652	-1.1514320004
-1.1025616656	0.5850605966	0.9999537652	1.2196308063
1.0792696037	0.5596759373	0.9999537652	1.1052620368
1.2919142292	0.4960480957	0.9999537652	-1.1790000723
1.6355379708	0.2094580072	0.9999537652	-1.0094237983
1.7087130193	0.1839423524	0.9999537652	1.0826144763
1.0187259402	0.8969119777	0.9999537652	-1.0199712567
-1.4016400111	0.6413973278	0.9999537652	-1.3906070606
-1.0241857312	0.8449569374	0.9999537652	1.0143769034
-1.07837959	0.8616430252	0.9999537652	-1.0414679626
-1.0452438	0.8442350749	0.9999537652	-1.320279003
1.3588921917	0.7998497015	0.9999537652	3.6030199429
1.3271976949	0.6908059814	0.9999537652	-1.8695625541
1.1158299481	0.9297797086	0.9999537652	-2.0477227478
1.1457972302	0.5094256825	0.9999537652	1.1168954247
1.2830731733	0.2308670626	0.9999537652	-1.2832244625
-1.4169132951	0.2396679734	0.9999537652	1.0881070491
-1.1422351649	0.4611257055	0.9999537652	-1.1942345597
2.7957660672	0.7055259561	0.9999537652	0
-1.1024234553	0.6583122988	0.9999537652	1.0973577609
-1.1670800354	0.4041696507	0.9999537652	-1.2126523643
1.0688368103	0.7053589304	0.9999537652	-1.3292742281
1.032552319	0.8461585508	0.9999537652	-1.0103944828
1.0662554746	0.7453543402	0.9999537652	1.1185365673
-1.5643710553	0.3804847966	0.9999537652	5.1481919969
-1.1161721852	0.6100510396	0.9999537652	-1.4090632666
0	0	0	4.9527501949
1.3924641813	0.0563094902	0.9999537652	1.1513599417
1.671629292	0.465334768	0.9999537652	1.125794308
0	0	0	2.996475627
1.7048355104	0.16358517	0.9999537652	-1.3161992606

1.1503991893	0.543535535	0.9999537652	-1.170947106
1.2276521349	0.2247827111	0.9999537652	-1.1465581773
1.0794343821	0.9113761966	0.9999537652	1.5247400379
1.343879998	0.6998249381	0.9999537652	-1.071402865
-1.0092422658	0.9697902258	0.9999537652	1.0518885104
-5.1764559054	0.0358969429	0.9999537652	1.0701803436
1.0772326823	0.8240633841	0.9999537652	1.2382065883
1.101152236	0.4919868085	0.9999537652	-1.2818319516
-1.1776184739	0.746468579	0.9999537652	-1.6302923146
-1.1457555741	0.910571956	0.9999537652	-2.6181175393
1.2281238208	0.1437525755	0.9999537652	-1.1795906443
-1.1608106207	0.3085970267	0.9999537652	-1.082740225
-1.0534822785	0.8385756062	0.9999537652	1.0693662477
0	0	0	2.2217890137
-1.3886329778	0.3101969502	0.9999537652	-1.5130485783
1.121795781	0.5205782298	0.9999537652	-1.102257063
1.5256842953	0.4013624821	0.9999537652	-1.3769722583
1.0584154638	0.7651746629	0.9999537652	1.0056528517
-1.0656835295	0.8749174417	0.9999537652	-1.6422376232
1.0397361931	0.9294787113	0.9999537652	-1.4266768545
-1.1777127504	0.5425376769	0.9999537652	1.1234612537
-1.7936922955	0.4660695729	0.9999537652	-3.1688506665
1.253661757	0.1699072903	0.9999537652	-1.2739212131
1.2609036704	0.3550098983	0.9999537652	-1.2303135515
-1.2435663335	0.2408037564	0.9999537652	1.1915912749
3.0643454819	0.3120649314	0.9999537652	1.0138718302
0	0	0	-5.3583704783
0	0	0	0
2.4307668446	0.2876836765	0.9999537652	-1.0751015612
-1.1772599164	0.4482316707	0.9999537652	-1.061072143
0	0	0	2.5938834885
-1.0824631274	0.8358621057	0.9999537652	1.2086543503
27.369260276	0.2000494026	0.9999537652	-1.1411969817
-1.7438320327	0.5074107466	0.9999537652	1.399968965
-6.5577136368	0.001583	0.9999537652	-1.0462859193
2.7933974408	0.6948713406	0.9999537652	10.818785458
2.7933974408	0.6948713406	0.9999537652	0
27.7226916631	0.2128734045	0.9999537652	1.3467382728
-1.2397660874	0.3458735515	0.9999537652	1.1039377613
1.2157816924	0.3316256424	0.9999537652	1.1277834379
-6.9736190158	0.2250321147	0.9999537652	12.3846842502
1.2289869031	0.6574568792	0.9999537652	-2.0852514066
1.6483580263	0.0402941117	0.9999537652	-1.0712719774
1.1177865052	0.9286373457	0.9999537652	1.9418822056
1.0113931203	0.972066166	0.9999537652	1.0050125339
1.0791087817	0.5539625938	0.9999537652	1.1981755059
-1.122767557	0.6951389334	0.9999537652	1.1299525771
-1.034157361	0.8405519423	0.9999537652	1.0747380576
-1.1457555741	0.9105755498	0.9999537652	2.996475627

1.6746256188	0.4287305743	0.9999537652	3.18554147
-1.2154673509	0.3712458678	0.9999537652	1.1843346827
1.1880119602	0.6701998571	0.9999537652	-1.0864953517
-1.1145487473	0.5130493877	0.9999537652	1.0987144999
1.0492483407	0.6945871742	0.9999537652	1.1150380916
-1.586811795	0.716742255	0.9999537652	1.9305383734
-1.1828551405	0.5710218691	0.9999537652	1.1952558649
-2.8782708378	0.1779564275	0.9999537652	1.0674845818
1.1216701798	0.9024721268	0.9999537652	-1.6635832525
1.015350556	0.8996277398	0.9999537652	1.1343325924
-1.3636173358	0.3399330952	0.9999537652	1.5942285498
1.2902250022	0.0840760414	0.9999537652	1.3442549415
-1.2134962926	0.484998831	0.9999537652	-1.0206294124
1.0558087725	0.8867881084	0.9999537652	-1.2301065239
9.8372048419	0.3843085791	0.9999537652	1.1001085314
43.2519505013	0.1383053945	0.9999537652	-1.4354930872
1.0213667383	0.9002253275	0.9999537652	-1.1173924018
1.1893183402	0.6003368428	0.9999537652	-1.1894568553
-1.0039312271	0.9889525391	0.9999537652	1.1224258875
-1.5881570201	0.7104954826	0.9999537652	1.4367288239
1.7927701543	0.003629	0.9999537652	-1.0771653991
-1.078505798	0.6839680568	0.9999537652	-1.0847921732
1.3153276664	0.0828340704	0.9999537652	1.2159413622
-1.1385017487	0.4777225355	0.9999537652	1.0154229768
1.1391083023	0.5752644496	0.9999537652	-1.0280150033
1.0204515276	0.9024349976	0.9999537652	-1.0215679756
1.0077278019	0.9569012455	0.9999537652	-1.1315347652
1.1036803303	0.4874676167	0.9999537652	1.1789544506
1.3946258785	0.107662583	0.9999537652	1.1868450122
1.338776268	0.4097951244	0.9999537652	-1.0253718085
-1.0597738355	0.8149616692	0.9999537652	1.0342991795
1.1053606867	0.4206638598	0.9999537652	1.081129869
-1.0646509197	0.6601503787	0.9999537652	1.0057982041
-2.7739563827	0.2141457904	0.9999537652	-6.262381099
1.9992276705	0.0188216276	0.9999537652	1.6105563021
4.5544554512	0.5691114569	0.9999537652	-1.1441751338
2.5560713637	0.2244365447	0.9999537652	1.0102910746
1.3970678716	0.0562074453	0.9999537652	-1.021132696
1.0245802971	0.885707349	0.9999537652	-1.2063358888
1.2683954833	0.5238426255	0.9999537652	-1.7820707453
-1.25001277	0.3023648867	0.9999537652	1.1171763061
13.3324809638	0.3193572283	0.9999537652	-14.4562307049
1.0466162917	0.8062307767	0.9999537652	1.2334460931
-1.3823836963	0.2093859926	0.9999537652	1.6238763338
1.074999585	0.7083689571	0.9999537652	1.0671453893
0	0	0	4.9527501949
-1.6666745839	0.6134564792	0.9999537652	1.532480552
1.0542464528	0.851462818	0.9999537652	-1.0062619915
1.3843118609	0.2871718928	0.9999537652	-1.1031146131

1.0197104916	0.8870177396	0.9999537652	1.1071579151
1.0217638108	0.9275922459	0.9999537652	1.0177837522
-1.087734772	0.6378202589	0.9999537652	1.199923851
-18.664869065	0.0419427805	0.9999537652	9.7116702472
-6.9736190158	0.2250321147	0.9999537652	24.4899985757
1.3911069039	0.6824958828	0.9999537652	-4.4401050878
-2.0766225707	0.0650572093	0.9999537652	-1.3591196342
-1.1905930305	0.2551984552	0.9999537652	-1.2646849194
1.246980349	0.1272828037	0.9999537652	1.3236358918
1.2173697394	0.3380293835	0.9999537652	1.1577041964
-1.1371386432	0.263782006	0.9999537652	1.0784877285
-1.102162793	0.5633734177	0.9999537652	1.2213281979
1.7476005214	0.4809986477	0.9999537652	-1.7402957735
-1.5875118025	0.7210671248	0.9999537652	-3.0249801457
0	0	0	0
1.3826309223	0.7329507267	0.9999537652	3.9589389566
1.0021669913	0.9912472332	0.9999537652	-1.1013804027
8.064174035	0.4194221592	0.9999537652	1.2674637396
0	0	0	2.9985540909
-1.4017655923	0.402386176	0.9999537652	-1.0755112179
-1.0162042674	0.9541742894	0.9999537652	-1.2073356628
-1.4685406098	0.1384957579	0.9999537652	1.273494796
2.6532911141	0.1038819751	0.9999537652	-1.0137170537
-1.17513703	0.7528126867	0.9999537652	2.0848300342
1.0318295738	0.8802273096	0.9999537652	1.0729654707
-1.2721916713	0.7569555928	0.9999537652	-1.5440473325
1.1167754821	0.9277522878	0.9999537652	-2.1285142809
-1.1013045071	0.7504155434	0.9999537652	-1.7565101817
1.0563667559	0.9343995892	0.9999537652	1.0254869434
-1.271448481	0.305182213	0.9999537652	-1.130130951
-1.1734992811	0.2832060493	0.9999537652	-1.222879627
-1.3125418099	0.2025065884	0.9999537652	-1.0587156129
0	0	0	-1.0736853543
-1.0537663165	0.7875406635	0.9999537652	-1.1785874642
1.6026102837	0.0959362612	0.9999537652	-1.179973074
-1.2803144096	0.1954813188	0.9999537652	-1.9594929259
-1.6062706965	0.4071429856	0.9999537652	-1.1640071036
2.6206366172	0.1455366619	0.9999537652	1.5580155169
-1.4428391683	0.0204007428	0.9999537652	-1.1816805323
-1.0165584389	0.9006997208	0.9999537652	-1.466780655
1.2122901119	0.1142854112	0.9999537652	1.1214755013
-1.0475832478	0.8254290455	0.9999537652	-1.1907308559
-1.0317350572	0.8223023426	0.9999537652	-1.028943166
1.0785123831	0.7675217623	0.9999537652	1.0727703548
-1.2749535851	0.1742982058	0.9999537652	-1.0418246644
1.6081277835	0.181921397	0.9999537652	2.10295626
-1.9429524323	0.4335406868	0.9999537652	-2.0718935183
1.1070112236	0.4822276274	0.9999537652	-1.3220035147
1.2218501334	0.33400344	0.9999537652	-1.2258814027

-1.1528956697	0.4420305933	0.9999537652	1.0236279578
-1.4041359274	0.5989398136	0.9999537652	1.6026084528
2.6791577165	0.1814540818	0.9999537652	-1.323124154
0	0	0	2.996475627
-1.1916821274	0.1758406931	0.9999537652	-1.2194501867
1.0990335097	0.4861099775	0.9999537652	-1.2532832654
1.0317023194	0.8110996527	0.9999537652	-1.1478004577
-1.1709147062	0.1762768936	0.9999537652	-1.425451599
1.0714411381	0.7391351348	0.9999537652	1.0673294167
11.5766527773	0.3453979996	0.9999537652	-1.619960364
1.118472422	0.6064879063	0.9999537652	-1.2909867644
-1.2710840668	0.4888601359	0.9999537652	-1.073933606
-2.4387448463	0.350320906	0.9999537652	1.8775765352
-1.044265872	0.7915801916	0.9999537652	-1.3597456187
1.0969890895	0.7618913834	0.9999537652	-1.7689304763
15.0880352532	0.2933962091	0.9999537652	-2.2267949004
-1.6267789228	0.4332034012	0.9999537652	-1.1146012341
1.2179071343	0.3245571665	0.9999537652	-1.5631120287
1.0748593979	0.6945648695	0.9999537652	-1.4137569598
-1.3181038637	0.660883906	0.9999537652	-1.3338590027
1.7770533563	0.1559966928	0.9999537652	-1.4713629668
-1.4374007791	0.1368363757	0.9999537652	1.235105271
-1.0752136643	0.7409241885	0.9999537652	1.1247584868
1.1561297187	0.6994753453	0.9999537652	1.4123528595
1.0771240962	0.6043945373	0.9999537652	-1.3676352739
0	0	0	-1.1248877899
1.0063866681	0.9646263483	0.9999537652	1.0673656124
-2.1848874448	0.2485164924	0.9999537652	2.2266622154
-1.3727891389	0.1434621015	0.9999537652	-1.1077010752
1.0929869337	0.7496665471	0.9999537652	-1.3360782943
1.8447991669	0.5955682701	0.9999537652	-3.2520699103
4.2332151529	0.1683009855	0.9999537652	-1.1345231394
8.064174035	0.4194284167	0.9999537652	0
1.0588714969	0.6950353352	0.9999537652	-1.1870673991
-1.356320743	0.1186748243	0.9999537652	-1.0146697206
-1.0350598224	0.8240623864	0.9999537652	-1.0778236326
-1.4144052759	0.4358234851	0.9999537652	-1.2705771923
1.0563036173	0.7793106249	0.9999537652	1.1171219355
-1.7169693606	0.5214600757	0.9999537652	-6.7496343228
-1.0662991483	0.8072802465	0.9999537652	-1.8034001629
2.7933974408	0.6948713406	0.9999537652	14.7271569203
1.4271180501	0.6554148992	0.9999537652	2.9666211993
1.1059952251	0.8326433367	0.9999537652	1.3336511346
8.064174035	0.419434684	0.9999537652	1.6778989432
-1.0756344936	0.7540173594	0.9999537652	-1.1078604286
1.1574430789	0.7666234673	0.9999537652	-1.6569452998
-1.6246276659	0.0119974418	0.9999537652	-1.3875954909
1.2031295302	0.4088581153	0.9999537652	-1.019488001
-1.9866409174	0.2172263406	0.9999537652	-1.0929796937

1.0084785312	0.9732918891	0.9999537652	-1.043895419
-1.1384365954	0.4711946492	0.9999537652	-1.0279632609
1.1334661355	0.5297372697	0.9999537652	1.2676452832
-1.0735894262	0.7648240361	0.9999537652	-1.2213622781
-1.1559908397	0.6622902392	0.9999537652	-1.7612825391
9.8205505243	0.383784024	0.9999537652	-1.6175548097
6.3075231393	0.4850807931	0.9999537652	-9.4802220615
-1.084779055	0.9219811384	0.9999537652	-1.7016325867
-1.1918212237	0.4648087453	0.9999537652	-1.0702105065
-1.1907399532	0.7138687965	0.9999537652	-1.0862742596
-1.412233084	0.341777579	0.9999537652	-1.3404035038
1.1141052312	0.5882125836	0.9999537652	1.0088356667
1.5571829811	0.2769052371	0.9999537652	3.0459701107
-1.281885198	0.5548494491	0.9999537652	-1.1080340654
1.9939828875	0.0379051981	0.9999537652	-1.0082432596
1.2082216971	0.4695881418	0.9999537652	1.2342699425
-1.030324871	0.8465714442	0.9999537652	-1.0072952154
-1.1452457414	0.663730053	0.9999537652	-1.1802054568
-2.5871290907	0.5000502148	0.9999537652	0
1.4696851973	0.1313032563	0.9999537652	1.2191300171
-1.2009321264	0.473658251	0.9999537652	-1.0943385619
6.3075231393	0.4850714177	0.9999537652	-1.3710897962
1.0705399723	0.8375389707	0.9999537652	-1.0486878052
-2.2386052964	0.3387938717	0.9999537652	-1.4377224945
-1.2072938767	0.5135982983	0.9999537652	-1.1257733271
-1.7355837304	0.0925576965	0.9999537652	-1.0731192448
1.1285974849	0.4411537941	0.9999537652	-1.123174573
1.372384133	0.2506231166	0.9999537652	1.2303622764
-1.1347432124	0.6059977813	0.9999537652	-1.078956744
13.3437712784	0.3162721485	0.9999537652	1.135271392
1.4534338257	0.4590033119	0.9999537652	-1.188556735
-1.4016425072	0.1302699873	0.9999537652	1.1448141725
0	0	0	-1.8579915692
2.1697122316	0.3609426919	0.9999537652	-2.3138943259
-1.4625285412	0.099940496	0.9999537652	-1.4958846435
1.3594099105	0.7964383549	0.9999537652	-4.5809105665
0	0	0	24.561035655
1.1060299692	0.5257268307	0.9999537652	-1.0271786283
-1.0560157548	0.791967046	0.9999537652	-1.37088054
-2.223376117	0.5879030156	0.9999537652	-4.9102474202
1.1128931333	0.5557622729	0.9999537652	1.0906180392
1.3505011552	0.5370674842	0.9999537652	-1.2994393007
-1.0862101524	0.7720871917	0.9999537652	1.0272036295
1.125498098	0.8624041203	0.9999537652	-1.3946376339
1.0563636447	0.7820543877	0.9999537652	1.2182563991
-1.2441281711	0.2224801575	0.9999537652	-1.2938241057
1.6310488281	0.392878117	0.9999537652	1.4433324361
-1.3968764843	0.4599725671	0.9999537652	1.0805292111
0	0	0	2.9985540909

1.0880792593	0.8210100698	0.9999537652	1.1227859182
1.3881051374	0.3623642669	0.9999537652	-1.194038978
1.0333223267	0.9629933284	0.9999537652	-1.2914682514
0	0	0	-5.3583704783
-1.6583999534	0.1153597364	0.9999537652	1.0537539781
2.3315363418	0.45714549	0.9999537652	-1.4119507574
1.8487361328	0.5872310539	0.9999537652	-1.3813421161
1.1560468193	0.5544445762	0.9999537652	1.2406010386
-1.0075959136	0.9942997012	0.9999537652	2.1744801732
-9.2663780788	0.0598432968	0.9999537652	1.0260351662
0	0	0	4.9527501949
1.0794355559	0.6145634596	0.9999537652	1.0299533271
0	0	0	4.9527501949
1.3627340806	0.1910210818	0.9999537652	1.2484105522
1.2989158155	0.5406470413	0.9999537652	-1.3744760131
3.8032158502	0.0183179672	0.9999537652	1.0881165141
1.0616945401	0.6240458428	0.9999537652	1.2609642525
0	0	0	-1.5263022474
1.0235529464	0.85345965	0.9999537652	-1.1195832156
1.1012443766	0.6167391889	0.9999537652	-1.1394805828
0	0	0	2.996475627
1.1935114622	0.8110705715	0.9999537652	-3.3143315952
-1.0396471216	0.8668130759	0.9999537652	-1.1017901241
-1.4615661169	0.068051964	0.9999537652	-1.1576052068
-1.0793000367	0.804628345	0.9999537652	1.3066335416
1.3784816619	0.0267768282	0.9999537652	-1.1327215861
1.0438980096	0.7414770303	0.9999537652	-1.0216080367
1.7982279591	0.2054164982	0.9999537652	-1.2272441027
5.9743887745	0.0914295122	0.9999537652	-1.2486442955
3.7902526731	0.2291175146	0.9999537652	-2.7991461852
0	0	0	2.996475627
30.9018162087	0.1808215217	0.9999537652	1.037875072
-1.4490984494	0.618297102	0.9999537652	-2.1425023034
1.0658546113	0.8991248444	0.9999537652	1.7903935568
-1.176697247	0.5491582032	0.9999537652	-1.6193623533
0	0	0	1.5932034817
0	0	0	4.9527501949
1.2635663613	0.6025959559	0.9999537652	-1.1747970282
-1.162773861	0.485479953	0.9999537652	-1.1066950409
-1.5284995321	0.0167114835	0.9999537652	-1.5148592367
1.7574703403	0.5562381998	0.9999537652	-2.051809064
1.0459602339	0.7600439303	0.9999537652	1.314784731
9.8205505243	0.383743098	0.9999537652	2.996475627
2.7933974408	0.6948713406	0.9999537652	2.996475627
0	0	0	32.2919870527
1.0164767859	0.9161627851	0.9999537652	-1.0450307281
1.2138837193	0.8081441496	0.9999537652	-1.2403739705
-1.3750557215	0.0797287469	0.9999537652	-1.2625249227
1.2404371515	0.5072041498	0.9999537652	1.4875701876

1.0265362329	0.8899256977	0.9999537652	-1.0779250193
1.0231930422	0.885460746	0.9999537652	-1.1752732696
1.0011937922	0.9957661729	0.9999537652	-1.0026228014
1.2509843358	0.6510319379	0.9999537652	1.3452580731
-1.1928398557	0.6315069795	0.9999537652	-1.0527538873
1.0144029994	0.960122021	0.9999537652	1.012267099
-1.2969402738	0.0951873738	0.9999537652	1.1484021494
-1.1790596572	0.6860832106	0.9999537652	1.4766739315
1.0492503333	0.770530771	0.9999537652	-1.2343301
-1.5881570201	0.7105610915	0.9999537652	-2.0477227478
-1.0471386134	0.8788781686	0.9999537652	1.0567957067
1.4010597526	0.2399335576	0.9999537652	-1.247118865
-1.2203312105	0.3464417067	0.9999537652	1.0644238673
1.3281567871	0.428635688	0.9999537652	-1.0172198358
-1.7001839609	0.5686067077	0.9999537652	16.6921882404
-1.212625285	0.2164154523	0.9999537652	-1.0419225158
1.1663478342	0.630998264	0.9999537652	1.3207646882
-1.7000524918	0.1034609448	0.9999537652	-1.2278753683
0	0	0	4.9527501949
5.9490049147	0.0348114593	0.9999537652	-1.780940241
1.1717813462	0.8080329042	0.9999537652	-1.0197524619
-1.5047828104	0.4916459184	0.9999537652	-1.004326321
-1.1510363467	0.306701964	0.9999537652	1.0820230352
-5.0913719412	0.0687285425	0.9999537652	1.0700419808
0	0	0	-1.1250196696
3.3773576537	0.1414725456	0.9999537652	1.2651833566
1.1678481523	0.3624616199	0.9999537652	-1.1305258256
2.7933974408	0.6948713406	0.9999537652	8.8639046058
0	0	0	0
-1.2081688639	0.505103209	0.9999537652	-1.3986503759
12.8276037884	0.0241645172	0.9999537652	-1.3027260822
-1.6639105903	0.4552489134	0.9999537652	5.2702039024
2.2243819093	0.147497346	0.9999537652	-2.779971155
1.7747552523	0.5382375344	0.9999537652	1.5461710487
8.064174035	0.419440961	0.9999537652	1.4285818179
1.1617842091	0.7301827238	0.9999537652	-1.1921032166
-1.4762397703	0.6421324097	0.9999537652	2.1094903549
-1.0753058266	0.9061169492	0.9999537652	-1.3845395584
1.136825298	0.6782332913	0.9999537652	-1.271456937
-1.2012183	0.3356836914	0.9999537652	-1.2648386692
1.5618741434	0.2429377662	0.9999537652	1.2384884805
1.2781742471	0.4320247114	0.9999537652	-1.1615529137
1.3489110337	0.1045940494	0.9999537652	-1.0040453842
-1.4135960926	0.1838760885	0.9999537652	-1.3620064009
1.3171799933	0.5237054096	0.9999537652	-1.3753110137
-1.267587539	0.3074902029	0.9999537652	-1.1068007261
1.055665269	0.8573598545	0.9999537652	-1.0416175866
-1.3254846731	0.3831951394	0.9999537652	-1.424438756
-4.8154268017	0.1712842889	0.9999537652	1.6293799659

1.0301371532	0.8432304465	0.9999537652	1.1108422422
4.5505976667	0.5557790267	0.9999537652	10.818785458
-2.4105941946	0.2544345395	0.9999537652	3.346511156
-1.806116959	0.2388346274	0.9999537652	1.0791114358
-1.1801563466	0.2804392575	0.9999537652	-1.0938523839
-1.0212577444	0.8905414794	0.9999537652	-1.1624157212
2.4298416015	0.2822957211	0.9999537652	1.069558106
0	0	0	0
18.5983228161	0.2580166151	0.9999537652	-5.4378051094
-1.3569909963	0.1435217181	0.9999537652	1.0189906049
1.2890025947	0.4471845627	0.9999537652	-1.2879677397
1.0673376449	0.8286744568	0.9999537652	1.4482720026
1.0048337711	0.9757705383	0.9999537652	-1.0405154475
-2.4366028299	0.3448880053	0.9999537652	-1.27586727
9.8205505243	0.3834791406	0.9999537652	12.7820459093
-1.1250701949	0.5765240371	0.9999537652	1.3309170284
0	0	0	-1.783797305
1.2918684994	0.3065640001	0.9999537652	1.9231337463
-1.2938353722	0.6925444678	0.9999537652	-1.2542924597
4.5505976667	0.5557790267	0.9999537652	8.9512754559
-1.078780888	0.5875836863	0.9999537652	-1.0338521436
1.145033321	0.5212725013	0.9999537652	-1.2390817472
-1.2520819365	0.2343634681	0.9999537652	1.0978733519
1.3071135433	0.0363668756	0.9999537652	-1.250560334
1.2019322441	0.5447644618	0.9999537652	-1.0257987911
-1.0168784051	0.9033265967	0.9999537652	-1.1360957679
1.210538481	0.5108700744	0.9999537652	1.4099628362
-1.0852976242	0.6591075975	0.9999537652	-1.2215101234
-1.2205009789	0.2341121108	0.9999537652	-1.053799295
1.5402842257	0.2785541672	0.9999537652	1.773442696
25.6372777234	0.2064866588	0.9999537652	2.0501017816
1.0530578414	0.7028038341	0.9999537652	-1.0349191979
1.1123436412	0.6973014257	0.9999537652	-1.0407860647
6.3075231393	0.4850526886	0.9999537652	-1.4789049904
1.1558054485	0.3223017618	0.9999537652	1.0327059119
0	0	0	-1.8577842586
-1.045486109	0.8043288179	0.9999537652	-1.5171560264
1.118603212	0.5386021884	0.9999537652	1.1279073823
1.444159585	0.0912430859	0.9999537652	-1.4274858485
-1.0860596516	0.5596414582	0.9999537652	1.0280001368
1.4024244579	0.0842961447	0.9999537652	-1.1258731094
1.0273242406	0.8864750818	0.9999537652	-1.1927378547
1.1158299481	0.9297700246	0.9999537652	-18.5505103377
-1.1372178321	0.5542245606	0.9999537652	1.3268195762
-1.1624491312	0.4689861471	0.9999537652	-1.2828888516
0	0	0	12.7732027325
-1.8932679072	0.0375664401	0.9999537652	-1.286185604
-1.0077665341	0.9548072135	0.9999537652	-1.0278867501
2.0551347362	0.0771231753	0.9999537652	-1.1415967226

-1.0340663196	0.9432285308	0.9999537652	1.3625539832
-1.2743531835	0.1740886959	0.9999537652	-1.1046940635
-1.1453192235	0.9083725834	0.9999537652	-1.6585837467
-2.4710767924	0.5445066715	0.9999537652	2.8664963163
-2.9531134474	0.1065868873	0.9999537652	-1.292009265
1.1548920694	0.7899023326	0.9999537652	-1.2387664314
-1.3362100218	0.2798665508	0.9999537652	1.4496745044
1.1170046682	0.6375265172	0.9999537652	1.1161019094
1.1755664601	0.7676777761	0.9999537652	-1.3401925228
0	0	0	9.7116702472
1.3911422814	0.6812316109	0.9999537652	-1.1172627069
-1.0992971271	0.4642693738	0.9999537652	-1.1273488767
1.1965287697	0.6460277568	0.9999537652	-1.0408241761
4.5505976667	0.5557790267	0.9999537652	-1.3465212658
1.404315049	0.5321816099	0.9999537652	-1.5222523012
-1.0172976377	0.8918401746	0.9999537652	-1.1441166703
-1.0121114565	0.9688055823	0.9999537652	-1.0537061719
1.373254683	0.1805985577	0.9999537652	-1.2176158191
-1.5521182934	0.0143472286	0.9999537652	-1.5105895643
-1.0695394047	0.8362497034	0.9999537652	-1.0679530314
1.3022993228	0.0808330785	0.9999537652	-1.4368336078
1.0166077861	0.9023734741	0.9999537652	-1.299134679
6.3128691894	0.4806190757	0.9999537652	0
1.134419041	0.5085529512	0.9999537652	1.4976518284
1.2186845019	0.411706872	0.9999537652	1.1424378048
1.5372566733	0.5059855362	0.9999537652	1.0593994383
1.0466770734	0.9466484957	0.9999537652	-1.7594595112
1.7165647286	0.3113521217	0.9999537652	1.529843838
15.1136055058	0.2931870683	0.9999537652	1.6443474089
-1.1324485602	0.68086442	0.9999537652	1.1818790531
2.7933974408	0.6948713406	0.9999537652	0
1.0502013436	0.6725169898	0.9999537652	-1.025340689
1.5343375215	0.0603198468	0.9999537652	-1.059950513
1.8209565278	0.0379128593	0.9999537652	1.1935454681
-1.26502287	0.6550082736	0.9999537652	1.2833153249
-1.0694258459	0.6620659212	0.9999537652	-1.0274939451
1.2254792116	0.1304833389	0.9999537652	-1.2161943526
-1.1259191861	0.4291303433	0.9999537652	1.0598989714
-3.8100204382	0.0432887753	0.9999537652	1.9418822056
1.0345181807	0.9675034178	0.9999537652	2.3314895061
1.0563012871	0.7939397975	0.9999537652	-1.0048660842
-1.0378596797	0.9605695181	0.9999537652	-1.6695388296
-1.0497523855	0.8199846321	0.9999537652	-1.2214344583
1.0748985771	0.6226264326	0.9999537652	1.0706384193
1.7445826814	0.1454413654	0.9999537652	1.1561486813
1.0206733293	0.9422085254	0.9999537652	-1.1559190362
1.3016203124	0.284268227	0.9999537652	-1.0407730845
1.1559268405	0.3401589682	0.9999537652	1.1318754747
1.4423222949	0.2736078997	0.9999537652	1.0740134352

1.1246252399	0.4254114348	0.9999537652	-1.0481332576
1.1030675442	0.7174529418	0.9999537652	1.2121788465
-1.9032106646	0.1955507858	0.9999537652	2.5726413682
-1.0482410655	0.9007698246	0.9999537652	-2.3345897784
1.0346377397	0.8290283341	0.9999537652	-1.3614604753
-1.1887908385	0.3785554058	0.9999537652	-1.0182023229
5.4845415866	0.1224906324	0.9999537652	-1.061752755
0	0	0	8.8639046058
-1.0239893699	0.9270020461	0.9999537652	-1.0188866158
1.2031525807	0.2594995301	0.9999537652	-1.0076098181
1.1494558174	0.7273537161	0.9999537652	-1.176113658
-1.1382157344	0.3660248158	0.9999537652	-1.3328321118
0	0	0	0
1.1527994666	0.5497651792	0.9999537652	-1.1403723214
-1.0446110432	0.8852149608	0.9999537652	1.0537363514
1.086292769	0.5172187702	0.9999537652	-1.0999393553
1.02058654	0.8744485888	0.9999537652	-1.1123752535
1.3600088454	0.7959330601	0.9999537652	-2.1459038996
-1.0127495209	0.9797283576	0.9999537652	1.081313546
1.9681797508	0.1647896757	0.9999537652	-1.1215245158
1.0924809279	0.653854552	0.9999537652	-1.177014691
1.2185229758	0.4179645716	0.9999537652	-1.272283359
-6.9762758667	0.2572015215	0.9999537652	10.818785458
1.2067917127	0.3099352323	0.9999537652	1.0999951056
1.0185684971	0.9403162359	0.9999537652	1.073816778
-2.2202518712	0.2391289597	0.9999537652	1.3789201524
-1.1063899099	0.6642680603	0.9999537652	-1.0689461864
-1.0214729369	0.9186883513	0.9999537652	-1.146641679
1.1840785337	0.1912944118	0.9999537652	1.0868676621
1.3605615982	0.7922944487	0.9999537652	10.818785458
1.536236782	0.7286300615	0.9999537652	18.6723895103
-1.6202196427	0.0895636446	0.9999537652	-1.361786319
1.2058527351	0.5650362783	0.9999537652	-1.1157108887
-1.0236791058	0.9044592128	0.9999537652	-1.165006881
1.220427997	0.6251875197	0.9999537652	1.128463338
1.1594020585	0.4001454866	0.9999537652	-1.3320609556
-1.1009292492	0.662447975	0.9999537652	-1.0206310658
2.7957660672	0.7055283363	0.9999537652	0
1.1049558507	0.6738593205	0.9999537652	1.7820635533
1.1252402684	0.4875709804	0.9999537652	-1.0483672394
3.0589901989	0.3336403716	0.9999537652	-1.2052463201
1.011645593	0.9769609383	0.9999537652	1.6939652145
1.1426624059	0.744225699	0.9999537652	-1.7978773859
1.1192151057	0.4186828696	0.9999537652	-1.0385522696
1.1242920327	0.6488434153	0.9999537652	-1.6950361864
1.3819288708	0.4745008631	0.9999537652	1.5518947271
-1.1523079009	0.5700685447	0.9999537652	-1.243328708
-1.0786021403	0.8322874244	0.9999537652	-1.0415387195
1.158407971	0.5065510149	0.9999537652	-1.1590769815

-1.4257970267	0.2494606856	0.9999537652	1.1878951287
1.5715463549	0.3301591465	0.9999537652	-1.2436835366
6.3075231393	0.4850433349	0.9999537652	-3.1575868765
0	0	0	1.5746361775
1.1173553606	0.5920244542	0.9999537652	-1.1970299896
1.0908127517	0.5354841444	0.9999537652	-1.2004474802
1.8627733224	0.2522587133	0.9999537652	-1.0060504437
1.0829303747	0.8614820266	0.9999537652	-1.1319899092
5.729217422	0.1066646443	0.9999537652	9.3351613897
1.251864346	0.200701416	0.9999537652	1.2919866582
2.7933974408	0.6948713406	0.9999537652	0
1.1512845273	0.7192823605	0.9999537652	-1.2151679374
1.526526342	0.4270015733	0.9999537652	-1.6785389006
0	0	0	0
-1.1758575684	0.6694227587	0.9999537652	-1.3483185415
-1.3055155248	0.2682253761	0.9999537652	1.2453709858
1.234944745	0.6149935156	0.9999537652	-1.3984039662
2.8173722589	0.3575164131	0.9999537652	-3.0470159237
-1.030714772	0.9316625803	0.9999537652	1.5183919491
-1.1955637999	0.4569734941	0.9999537652	1.0339015345
1.0082473734	0.9835181967	0.9999537652	-1.2485901673
2.7933974408	0.6948713406	0.9999537652	0
-4.8180649265	0.003264	0.9999537652	1.0214404941
-2.5065115073	0.1191177972	0.9999537652	-1.0532424745
-1.1982698859	0.6101677384	0.9999537652	-3.6847346575
1.1867487184	0.5533270761	0.9999537652	1.5633351639
1.2931157656	0.1091851041	0.9999537652	1.1472425825
-1.0805508967	0.6296727196	0.9999537652	-1.2609591373
-1.2414129464	0.1462939016	0.9999537652	-1.0799107093
2.7933974408	0.6948713406	0.9999537652	-1.0283826769
-4.8095156671	0.2071263266	0.9999537652	-2.0465550316
1.0864783405	0.6723037584	0.9999537652	-1.2003120059
1.0485779015	0.8812930874	0.9999537652	-1.5255527176
3.4279118306	0.0486421236	0.9999537652	-2.0658636651
1.5306759421	0.5559626481	0.9999537652	1.1387229202
1.010638708	0.9675956833	0.9999537652	-1.6248908522
2.1450382405	0.1065054933	0.9999537652	-1.0635163472
3.6225051582	0.0828145505	0.9999537652	-1.0699611471
1.0035261346	0.9881345005	0.9999537652	-1.2524040825
1.4927666537	0.1325807218	0.9999537652	-1.2782739264
18.5983228161	0.2580245272	0.9999537652	1.0317044844
-1.480037784	0.306583018	0.9999537652	-1.3711714557
1.2424936042	0.4744106804	0.9999537652	-1.0659471312
-1.1344408063	0.4986598315	0.9999537652	-1.1244329736
1.3009829077	0.7685772033	0.9999537652	-1.3464555531
15.0880352532	0.2933880806	0.9999537652	-1.417057339
0	0	0	-1.1507047539
1.7536570027	0.2260547586	0.9999537652	-1.1683224195
1.4581289787	0.4553578527	0.9999537652	-1.4211366135

-1.0565379405	0.9482502772	0.9999537652	3.7898935296
1.4233225829	0.4490143599	0.9999537652	1.4461950822
1.1986398919	0.4657517974	0.9999537652	-1.3637989963
-1.1558483247	0.623544999	0.9999537652	-1.2709502879
1.2033945198	0.5688300182	0.9999537652	-1.1010539694
-1.1764906816	0.7338307591	0.9999537652	1.0396988814
2.7933974408	0.6948713406	0.9999537652	-2.9500562616
1.308800742	0.4843659154	0.9999537652	1.2080202757
-1.9836846783	0.2996519325	0.9999537652	-1.5335805844
1.3386370254	0.0292941635	0.9999537652	-1.0791586371
0	0	0	4.9527501949
-1.1935357527	0.6034810118	0.9999537652	-1.2889115685
-1.0609413011	0.7317991207	0.9999537652	-1.1384679068
-1.4951938362	0.126579483	0.9999537652	-1.2970744973
2.7933974408	0.6948713406	0.9999537652	-1.6177358982
1.1186563498	0.7686555885	0.9999537652	-1.0859278153
-1.5403203254	0.1725270786	0.9999537652	1.2012722187
1.0170309763	0.9231621064	0.9999537652	1.1564702098
-1.1581330352	0.6041721781	0.9999537652	1.199592174
-6.9764533263	0.2594457297	0.9999537652	-1.1250196696
0	0	0	8.8700459102
0	0	0	4.9527501949
-1.1416943838	0.474426536	0.9999537652	-1.1480784159
0	0	0	-1.5094768143
4.5505976667	0.5557790267	0.9999537652	-1.8638252835
-1.1377112569	0.5047532425	0.9999537652	-1.2091405908
-1.2012337684	0.6512216385	0.9999537652	-1.1124839237
-12.9800088947	0.1122149734	0.9999537652	1.0071007236
4.5505976667	0.5557790267	0.9999537652	-2.0486608969
11.5766527773	0.3453876938	0.9999537652	-1.8099040113
-1.7140208703	0.492732186	0.9999537652	-1.380387871
2.7933974408	0.6948713406	0.9999537652	-2.1287636007
1.0924427605	0.6397859973	0.9999537652	-1.2123245883
-1.722233171	0.4472985861	0.9999537652	-1.2574134143
1.0794100206	0.7487065438	0.9999537652	1.0176022321
3.8622037496	0.6191249779	0.9999537652	16.6806485101
-1.3167984204	0.1196188046	0.9999537652	-1.0487482802
0	0	0	-5.3589986948
-1.0258142164	0.9592317531	0.9999537652	-1.1534670955
-1.0141055286	0.9409898378	0.9999537652	1.2777132603
1.1228403073	0.6852984785	0.9999537652	1.0864517801
6.3075231393	0.4850339883	0.9999537652	2.996475627
-1.1565935919	0.4010721525	0.9999537652	1.1278955887
0	0	0	2.996475627
-1.0110308297	0.9357535516	0.9999537652	1.0470438807
9.8205505243	0.3832114642	0.9999537652	4.9527501949
-3.2084199394	0.1755607384	0.9999537652	1.4645753428
1.2510834545	0.7543523389	0.9999537652	1.1395310869
-1.0029687624	0.9930033276	0.9999537652	1.205308698

1.4293980703	0.1772713472	0.9999537652	1.1786059235
1.6794784813	0.0633133448	0.9999537652	1.0315507091
-2.6346039855	0.2373772989	0.9999537652	1.1286185336
1.3049036967	0.3083795	0.9999537652	-1.0190699707
-1.0395856478	0.9559926493	0.9999537652	-1.5213869822
-1.1830030697	0.424900887	0.9999537652	-1.401061249
1.057547026	0.8624918725	0.9999537652	1.6035971241
-1.2348617235	0.4429230787	0.9999537652	1.1893335829
-1.1614998763	0.8771943156	0.9999537652	1.590997316
-1.2648530332	0.3838028312	0.9999537652	1.300634885
0	0	0	6.908559683
-1.4305171946	0.216227946	0.9999537652	-1.4772967103
-1.0391547537	0.937290952	0.9999537652	-1.2847094215
-1.0892345993	0.7810756218	0.9999537652	-1.0243291121
1.184711193	0.5664053213	0.9999537652	1.3986726513
-1.0466702521	0.7806818862	0.9999537652	1.1821646714
1.0950484463	0.8377363143	0.9999537652	1.0875791768
0	0	0	0
-1.2267866072	0.3654564023	0.9999537652	1.2305504307
-1.0573270666	0.7510154222	0.9999537652	1.0934442567
-1.054993213	0.6888434304	0.9999537652	-1.0226480527
-1.0091800042	0.9894803742	0.9999537652	1.2978163757
1.2516520703	0.8155283669	0.9999537652	1.3122322072
-1.0294245193	0.9274764095	0.9999537652	-1.0338450591
0	0	0	2.996475627
1.3381879427	0.4696927068	0.9999537652	1.4776267496
-1.3193843665	0.6368582348	0.9999537652	-1.1109452766
-1.3691163547	0.7485465961	0.9999537652	26.4836696502
1.0326264856	0.8582294438	0.9999537652	-1.1589555837
1.1923348135	0.8094831065	0.9999537652	-1.1138754971
1.1508419291	0.329386538	0.9999537652	-1.1051170833
-1.0110479264	0.981462493	0.9999537652	-1.1873903297
-1.066225041	0.6087240011	0.9999537652	-1.0926434679
1.6078998043	0.2129207506	0.9999537652	1.0329513553
6.3075231393	0.485024649	0.9999537652	1.1113570682
-1.04443861	0.7348901094	0.9999537652	1.2101040626
-1.2333718185	0.2249906788	0.9999537652	1.1141662159
-1.0809880189	0.7808653855	0.9999537652	1.0599674536
1.8105142317	0.2118058633	0.9999537652	5.9093030807
1.0135429035	0.9204590547	0.9999537652	1.2181348458
1.7636638943	0.2360964154	0.9999537652	-1.1386266738
1.0650909735	0.7360574537	0.9999537652	1.2135849789
1.5282990302	0.0992296747	0.9999537652	-1.4640290775
1.1623893453	0.3614432423	0.9999537652	-1.0714554815
1.0839352039	0.5181761681	0.9999537652	1.2494185662
2.7933974408	0.6948713406	0.9999537652	-2.0482026754
-1.5204530206	0.2241585596	0.9999537652	1.5825303983
-1.0577236681	0.6404184192	0.9999537652	1.1610759594
-1.2452728949	0.2086214036	0.9999537652	-1.0923730245

-1.0525508171	0.7802100798	0.9999537652	1.0719033462
1.3594099105	0.7963857293	0.9999537652	1.03027813
-1.1169422023	0.3787224548	0.9999537652	-1.0414571035
18.6146543426	0.2564103496	0.9999537652	1.7647064373
2.5559496274	0.2821600258	0.9999537652	-1.4614455774
3.0847820696	0.0845495183	0.9999537652	1.1818535
1.5043500252	0.0762851799	0.9999537652	1.1092263452
1.1038300958	0.7595932124	0.9999537652	1.0162474349
1.2434999203	0.4606656211	0.9999537652	1.1233092449
4.5505976667	0.5557790267	0.9999537652	2.3306887369
2.0488142106	0.0864135954	0.9999537652	1.1214479884
4.2863366418	0.009618	0.9999537652	1.234596048
-1.2658340445	0.5071918883	0.9999537652	1.1051794994
-6.9764533263	0.2594541557	0.9999537652	12.7732027325
1.1162834585	0.9283005435	0.9999537652	-1.8319388746
1.3987841508	0.2942672787	0.9999537652	-1.118893955
1.6316572466	0.0112249733	0.9999537652	1.2298525014
-1.1309194187	0.4483743129	0.9999537652	1.1821615247
0	0	0	0
1.2429956471	0.0947007811	0.9999537652	1.1846917128
-1.3265546466	0.3201852035	0.9999537652	1.2627285698
-1.0022969468	0.9916830218	0.9999537652	1.1609174153
2.1063705725	0.3260900467	0.9999537652	2.7252039655
1.0386897236	0.8503483764	0.9999537652	1.0270180789
1.0862402122	0.5593188864	0.9999537652	1.0722439628
1.1358328801	0.6883537094	0.9999537652	-1.0949246265
1.0173947989	0.9798950047	0.9999537652	-2.2049479182
4.5096948998	0.0955483879	0.9999537652	1.2593062028
2.7933974408	0.6948713406	0.9999537652	1.6293799659
0	0	0	1.590997316
1.4019107883	0.2853816598	0.9999537652	1.7110593438
-1.2859315764	0.4086132609	0.9999537652	-1.1641384423
2.4081512873	0.015889705	0.9999537652	1.1358029341
1.8514811731	0.2419713149	0.9999537652	1.0887530024
1.9568571513	0.13604572	0.9999537652	1.5478811278
-1.4598916326	0.3905566585	0.9999537652	1.8932975474
-1.1961439546	0.4131510851	0.9999537652	1.4842686482
1.1531921971	0.7307403067	0.9999537652	-1.0539493995
0	0	0	-1.1242401582
-1.0329924429	0.8105179622	0.9999537652	-1.1135262281
-1.1215690563	0.5562090087	0.9999537652	-1.1702633418
-1.0473609764	0.7692406106	0.9999537652	-1.4120905621
1.5290360293	0.0823151218	0.9999537652	-1.2989424385
-1.1778371941	0.6194565994	0.9999537652	-1.4140486124
-2.587194901	0.5006000393	0.9999537652	2.9985540909
1.3251991125	0.4160211183	0.9999537652	1.025082739
-1.4977878883	0.1004715367	0.9999537652	-1.2856784969
8.064174035	0.419447248	0.9999537652	2.996475627
1.812364286	0.001195	0.9999537652	1.0569154473

3.2873517405	0.0641098434	0.9999537652	1.5553899964
-1.3904208954	0.0789278164	0.9999537652	-1.2582030796
-1.0223205347	0.9114483754	0.9999537652	1.1367478743
-1.8747432512	0.1776903576	0.9999537652	-1.0817301948
1.718615226	0.2109332789	0.9999537652	-1.1750887277
1.1478931101	0.4509326238	0.9999537652	1.2462134155
1.9436827332	0.0309632824	0.9999537652	-1.2182511759
1.105252454	0.4447350908	0.9999537652	1.1402788272
-1.2935404951	0.383049112	0.9999537652	-1.2716154218
1.0311519106	0.810892828	0.9999537652	1.3435096171
-1.1193092232	0.6692193614	0.9999537652	-1.6504736728
1.0235019997	0.9049780812	0.9999537652	1.1235723754
1.0720230937	0.7720032263	0.9999537652	-1.3030832063
1.1521390299	0.6154937469	0.9999537652	-1.0924083554
3.8622037496	0.6190939875	0.9999537652	0
-1.0725217753	0.7699070756	0.9999537652	-1.1874510179
-1.0922108267	0.662918313	0.9999537652	1.0229090235
-1.1494578057	0.602570806	0.9999537652	-1.4050945683
-1.0277153659	0.8773118627	0.9999537652	-1.2168712931
1.1849524885	0.3624634567	0.9999537652	-1.0508470223
-3.9806836591	0.1367776348	0.9999537652	-1.4037934311
-1.0010472983	0.9952860046	0.9999537652	-1.4369823081
1.2580167088	0.359952157	0.9999537652	-1.0771964528
-1.2342136154	0.6438732923	0.9999537652	-1.1078460497
-1.0810587326	0.8357773496	0.9999537652	1.4754630712
-1.0811804713	0.730017097	0.9999537652	-1.0635320465
4.5505976667	0.5557790267	0.9999537652	10.8262784008
1.0501731349	0.7158974838	0.9999537652	1.0109157302
-1.0594179852	0.9284605946	0.9999537652	-1.4780730704
-1.104077235	0.8400353081	0.9999537652	-1.7588602022
0	0	0	2.9985540909
1.2109003063	0.446889576	0.9999537652	-1.0090949702
29.8698312703	0.2178461767	0.9999537652	4.6668957481
1.1660180084	0.7597234221	0.9999537652	1.2398622434
18.5983228161	0.2580324448	0.9999537652	-5.3583704783
0	0	0	0
0	0	0	0
-1.3202757318	0.4464955961	0.9999537652	1.0141839657
-1.1643105413	0.675404011	0.9999537652	-1.3323208868
-1.576381917	0.1182924784	0.9999537652	-1.064823008
-1.1080920104	0.4918357272	0.9999537652	-1.4182031104
-1.0735852446	0.6438749396	0.9999537652	4.7906608684
-1.2213734706	0.3482951064	0.9999537652	-1.1207717034
2.7933974408	0.6948713406	0.9999537652	-1.8601323346
-1.1572314773	0.7103575172	0.9999537652	-1.1615957223
1.2771987714	0.3803432774	0.9999537652	1.006149901
1.7132142193	0.3174288178	0.9999537652	-1.7987039554
-1.008890327	0.9505146367	0.9999537652	1.0839832223
-2.5861438062	0.4759835951	0.9999537652	2.0298295761

-1.0227354494	0.8796437018	0.9999537652	-1.117798263
1.2186578499	0.2230578661	0.9999537652	1.3781625467
1.0781818837	0.7410022047	0.9999537652	-1.1363978585
-1.087028354	0.7860508775	0.9999537652	1.1489603668
0	0	0	-2.6884253381
-1.2666659704	0.3747691866	0.9999537652	-1.3471614313
1.0797723979	0.7757195769	0.9999537652	1.1393691335
-1.203878637	0.2498597283	0.9999537652	1.1017265882
1.1135349126	0.5839624546	0.9999537652	1.0174114462
4.5505976667	0.5557790267	0.9999537652	2.6434024479
-1.0318054181	0.8868974491	0.9999537652	-1.6895485678
2.7933974408	0.6948713406	0.9999537652	-2.2519168774
0	0	0	6.0076339951
1.0694330635	0.8359191578	0.9999537652	1.3369382744
-1.6283093982	0.2088898022	0.9999537652	-1.0249803451
-1.1017949057	0.7991396757	0.9999537652	1.0968353938
-1.988371896	0.3509003474	0.9999537652	-1.4377113732
1.2497824691	0.6297590601	0.9999537652	-1.4668835758
-1.2575819565	0.2227733406	0.9999537652	1.7301041123
1.0970003946	0.7660480017	0.9999537652	-1.146501125
2.3437339069	0.2413758611	0.9999537652	1.4957399208
-1.0844560842	0.8245079197	0.9999537652	1.4630083089
50.353918894	0.1257610872	0.9999537652	1.5704032366
1.1021665157	0.5613410312	0.9999537652	-1.220146069
1.100014683	0.8441380715	0.9999537652	-1.6486130817
-1.4964533459	0.240918103	0.9999537652	1.243911249
0	0	0	4.9527501949
-1.0646727325	0.8903564567	0.9999537652	3.1950411467
-1.2433492998	0.2105056105	0.9999537652	-1.4366725925
1.0007280239	0.995642041	0.9999537652	1.0274992894
6.952600375	0.0636929868	0.9999537652	2.4627764693
1.2457924947	0.3641242111	0.9999537652	1.3412167313
1.0191212541	0.9509178499	0.9999537652	1.6325280088
-1.0843974846	0.8008114114	0.9999537652	1.4500952851
1.2559996159	0.6354174253	0.9999537652	-1.1191567661
1.8384783622	0.3350415769	0.9999537652	32.2919870527
1.3305963343	0.0873031979	0.9999537652	1.0689989011
0	0	0	8.8639046058
1.3611966861	0.805568733	0.9999537652	-4.1128151248
2.7933974408	0.6948713406	0.9999537652	-3.4501471538
1.0757937148	0.8109443621	0.9999537652	1.1159516421
-1.2628687322	0.1299836875	0.9999537652	-1.0612391732
-7.0431967789	0.0843517916	0.9999537652	1.1696208368
1.0375972768	0.8229954645	0.9999537652	1.0421521756
1.5237790209	0.3179726548	0.9999537652	1.4262322601
-2.6365262476	0.2553458373	0.9999537652	1.7067971001
1.1247725537	0.4826707244	0.9999537652	-1.1349212475
1.1422707648	0.7240415672	0.9999537652	-1.2687374905
2.0750906843	0.2910129601	0.9999537652	1.5872627531

-1.1169753243	0.5540566302	0.9999537652	-1.2394105526
1.1322498903	0.3300484125	0.9999537652	1.1858160467
8.064174035	0.4194535447	0.9999537652	-1.260546903
1.3744821631	0.0330168603	0.9999537652	1.0041009675
-1.2487542434	0.2385973341	0.9999537652	-1.0794081971
1.9050337421	0.4943882978	0.9999537652	-1.0061411197
-2.0040326722	0.1036249358	0.9999537652	8.9512754559
0	0	0	6.9133480437
2.3027622606	0.0302704266	0.9999537652	2.1992263377
0	0	0	-4.4136565172
-1.3487785294	0.0782859655	0.9999537652	-1.4052923717
-1.01865071	0.9117438789	0.9999537652	1.3553611791
-1.1387126306	0.5414619043	0.9999537652	-1.1983103027
1.2096348278	0.1052795573	0.9999537652	-1.0346466493
-3.1022113581	0.1973670708	0.9999537652	2.4838166037
1.0831090224	0.7054776263	0.9999537652	1.0188192245
-1.5881166222	0.7191987762	0.9999537652	3.6950602847
-1.0561997538	0.70305254	0.9999537652	1.0362603879
-1.1211441156	0.6561588418	0.9999537652	-1.0635596181
-1.5366091256	0.1730146082	0.9999537652	1.2800845618
9.8205505243	0.3829399466	0.9999537652	-2.2617017545
1.2180613568	0.2105186041	0.9999537652	1.3082623578
-1.5875118025	0.720994624	0.9999537652	-1.2438634229
-1.0364965506	0.7968992663	0.9999537652	1.2010246808
-1.0096132336	0.9497448591	0.9999537652	1.2231330123
1.1203537902	0.3814406257	0.9999537652	1.2586473613
-1.0985513382	0.8319975091	0.9999537652	1.2160325514
-2.0427176335	0.2138588308	0.9999537652	-3.7217691978
-1.0695859759	0.5538396605	0.9999537652	1.1636059612
1.0299297453	0.8767442686	0.9999537652	-1.0795901453
-1.0970387217	0.7672191647	0.9999537652	1.1702978149
2.4256002084	0.3111574991	0.9999537652	1.7794382074
-2.9572400577	0.3878857582	0.9999537652	0
1.0791464579	0.8141688455	0.9999537652	1.1707410023
1.1927285864	0.5049995088	0.9999537652	1.9889272489
1.5054915967	0.007358	0.9999537652	1.3072022281
1.4538097815	0.2477994009	0.9999537652	-1.1010504481
-1.2100472527	0.4874443214	0.9999537652	1.0521458817
-1.0242721031	0.9264006896	0.9999537652	1.1039865205
4.5505976667	0.5557790267	0.9999537652	-9.7554110723
-1.3018440751	0.4781047576	0.9999537652	1.0496990134
-1.1316156217	0.4775419005	0.9999537652	1.0099235848
9.8205505243	0.3829094471	0.9999537652	1.1085489246
-6.9762758667	0.2572248416	0.9999537652	2.996475627
3.8622037496	0.6190629756	0.9999537652	0
-1.0088218013	0.9694672454	0.9999537652	-1.2160514908
1.0340900316	0.9148846869	0.9999537652	-1.0911447612
-2.852289864	0.046902447	0.9999537652	1.0644477251
1.1158299481	0.9297603423	0.9999537652	-2.9737541292

-1.0199742319	0.9369845466	0.9999537652	1.3018095446
1.0059913159	0.9800285316	0.9999537652	-1.0728637588
-4.3217318489	0.1760789903	0.9999537652	-4.7001646681
1.3588921917	0.7998744661	0.9999537652	-1.1248877899
1.1043826186	0.6101279153	0.9999537652	1.0891406389
-1.6026225251	0.0904292434	0.9999537652	1.2070459566
1.0151750387	0.9023195996	0.9999537652	1.1230752933
3.8118921289	0.0144439053	0.9999537652	3.7181029888
-1.190347565	0.4340579067	0.9999537652	-1.2289660045
1.1015849128	0.5388692572	0.9999537652	1.1231048904
25.6156185703	0.2096981958	0.9999537652	-1.2506145038
0	0	0	0
1.3586480118	0.3496560499	0.9999537652	2.0243635915
-1.1761465577	0.7993710594	0.9999537652	1.1503685294
0	0	0	1.4537866576
8.0781087093	0.4256311478	0.9999537652	-2.5509215507
-1.9066285216	0.4111062421	0.9999537652	-1.7161531777
-1.0185885583	0.8795100617	0.9999537652	1.0710413422
1.2002190147	0.2761338964	0.9999537652	-1.1130577991
1.3190010683	0.4531182914	0.9999537652	1.1271017689
1.0103587518	0.9339506655	0.9999537652	1.0265557581
6.3075231393	0.4850153168	0.9999537652	0
1.2971957717	0.4539322691	0.9999537652	1.1920992849
1.1805207632	0.4057274343	0.9999537652	1.2149219869
0	0	0	2.9988886918
1.7749775399	0.0313955292	0.9999537652	1.8681634265
2.7933974408	0.6948713406	0.9999537652	0
-1.2302888673	0.4998259205	0.9999537652	-1.3025324162
-1.1453192235	0.9083222695	0.9999537652	2.6430925766
2.7933974408	0.6948713406	0.9999537652	2.9988886918
-1.1538633603	0.2689735591	0.9999537652	-1.1249474547
1.6070119312	0.003297	0.9999537652	-1.3557332248
0	0	0	4.9527501949
1.0993526806	0.6083911617	0.9999537652	1.44181622
1.546724015	0.1843755706	0.9999537652	1.4025537326
0	0	0	4.9567375629
1.1406129562	0.5212537832	0.9999537652	1.0245837828
2.8234781609	0.3571958561	0.9999537652	-1.9178632647
-1.1741413282	0.3305162079	0.9999537652	1.0881178296
-6.9736190158	0.2254964479	0.9999537652	8.8639046058
-2.0110266953	0.0651236858	0.9999537652	-1.2353900146
-1.4997668867	0.0226380024	0.9999537652	-1.2392981261
1.1836698478	0.3479135599	0.9999537652	1.2341637126
2.7957660672	0.7055307175	0.9999537652	2.9985540909
-1.7705309934	0.0150085158	0.9999537652	1.1261916202
2.7933974408	0.6948713406	0.9999537652	-1.859280336
4.5505976667	0.5557790267	0.9999537652	0
0	0	0	4.9527501949
0	0	0	2.9985540909

2.7957660672	0.7055330996	0.9999537652	0
8.064174035	0.4194598514	0.9999537652	-1.1248877899
1.2646874733	0.5420003543	0.9999537652	-1.2118554381
-1.1914319791	0.2747370425	0.9999537652	1.3083925536
1.2627085493	0.5224240771	0.9999537652	1.1038342177
-1.0658619679	0.8195425626	0.9999537652	1.0240496877
0	0	0	0
1.0259251803	0.8572350105	0.9999537652	1.5047155275
-6.9762758667	0.2572326198	0.9999537652	1.135271392
-1.2189036438	0.4522662017	0.9999537652	1.0843189304
1.0242314081	0.8571543901	0.9999537652	1.4181281865
1.2725088739	0.1592831115	0.9999537652	1.0492433912
1.2221011652	0.1391762293	0.9999537652	1.3735903825
-1.0202208064	0.908381675	0.9999537652	-1.0683469055
1.0455504623	0.8155550973	0.9999537652	-1.023263208
1.1956521695	0.5535803264	0.9999537652	-1.714633041
1.062424187	0.619663238	0.9999537652	1.1234947518
1.0101032125	0.9536084731	0.9999537652	-1.0658960682
1.2649711727	0.3810131816	0.9999537652	-1.1692845124
-1.2444974294	0.0727550052	0.9999537652	1.130548589
-1.0887934452	0.7574178257	0.9999537652	1.1141791921
1.0106051247	0.9586312195	0.9999537652	1.0371546229
4.5505976667	0.5557790267	0.9999537652	-1.038722389
-1.1923137678	0.3646740422	0.9999537652	1.1510564855
1.0926627905	0.6662972764	0.9999537652	-1.0031410517
18.5983228161	0.2581151183	0.9999537652	-1.2173989672
-1.1819800889	0.2671767659	0.9999537652	-1.2131018739
-1.0516197387	0.7866504625	0.9999537652	-1.1687738634
1.0715889046	0.6588098112	0.9999537652	1.1672366879
-1.2506369357	0.2184955976	0.9999537652	1.4683307042
-2.5861438062	0.4765251449	0.9999537652	0
0	0	0	2.996475627
-1.0326333469	0.8215401045	0.9999537652	1.0393627243
-1.3908389207	0.1165262648	0.9999537652	1.3327427712
-1.0232004376	0.8806709779	0.9999537652	1.2327716394
0	0	0	1.2400291296
-2.5871290907	0.5000409052	0.9999537652	2.9985540909
0	0	0	6.908559683
-1.1004235189	0.7427304309	0.9999537652	1.227584173
1.017643433	0.9691037721	0.9999537652	1.131914333
-1.2686505191	0.3624838625	0.9999537652	1.0529721961
1.7671539795	0.2255620168	0.9999537652	1.0929207328
1.1848870568	0.453215803	0.9999537652	1.4646192864
-1.3448588931	0.4231443981	0.9999537652	-1.2783396637
-1.1615920107	0.5714238967	0.9999537652	-1.293213934
-1.0425902303	0.8999243487	0.9999537652	1.0379497197
1.378012146	0.0680404732	0.9999537652	1.0833667551
-1.8122989227	0.2782924859	0.9999537652	1.0709421595
1.8027371112	0.4190916331	0.9999537652	1.233775198

1.0153502276	0.9500399995	0.9999537652	1.1087992865
-1.1041574634	0.7684901006	0.9999537652	-1.3887172398
1.0966499769	0.6858960062	0.9999537652	1.5279350235
-1.4456334055	0.1795798304	0.9999537652	1.8672258446
1.593132356	0.1211717136	0.9999537652	1.866659274
4.5544554512	0.5691094638	0.9999537652	-9.7598809705
0	0	0	2.9988886918
15.0880352532	0.2933799524	0.9999537652	-3.384595918
-1.2835526175	0.1136805105	0.9999537652	1.2097061402
-1.0558461694	0.777060492	0.9999537652	1.1366901367
1.2557671782	0.2109364175	0.9999537652	1.6438635573
9.8205505243	0.3828660831	0.9999537652	-3.8938703542
1.5275932178	0.0326822203	0.9999537652	1.4378724457
-1.4437979634	0.5749443101	0.9999537652	-1.3853788118
-1.0294818433	0.9279215917	0.9999537652	1.0795020561
1.2633691172	0.5669080621	0.9999537652	1.2217787389
-1.5884336587	0.3201788247	0.9999537652	1.1747922332
-1.2143676553	0.5233401533	0.9999537652	1.1079584868
-1.2350179012	0.3000777022	0.9999537652	1.0411610528
-1.9893749934	0.0237469629	0.9999537652	-1.1145736255
13.3324809638	0.3193649127	0.9999537652	-1.1446993292
-1.0830481592	0.6881189843	0.9999537652	1.2978699048
-1.2080529024	0.2043157132	0.9999537652	1.2594668575
0	0	0	2.996475627
1.0740170126	0.6904840167	0.9999537652	1.0302075238
1.0489179834	0.7067076026	0.9999537652	1.0077437993
1.0954049491	0.7605116269	0.9999537652	1.0524499689
1.0211054721	0.9148878452	0.9999537652	-1.0756176935
-1.1837761897	0.2392677938	0.9999537652	-1.0575660145
-1.0622491751	0.7799125861	0.9999537652	-1.1166815526
-1.2225269604	0.1919387424	0.9999537652	-1.1392479275
59.0891363577	0.1085514315	0.9999537652	2.6488015607
0	0	0	8.8710369702
1.9569338339	0.0288546728	0.9999537652	1.0973153205
-2.3908639205	0.0117484481	0.9999537652	-3.3722286371
0	0	0	6.908559683
-2.0677479284	0.0926159638	0.9999537652	-1.1381418077
-1.2677335371	0.4116307622	0.9999537652	1.1027421869
-1.1391927021	0.5074026462	0.9999537652	1.0482609427
1.0167565324	0.9246484751	0.9999537652	-1.1340411268
1.0925830294	0.6196738349	0.9999537652	1.3124275979
-1.2697946181	0.0523828467	0.9999537652	-1.0366395676
1.0253551658	0.9160412453	0.9999537652	-1.1287794271
1.5745683148	0.007292	0.9999537652	1.1345386682
2.417047608	0.1687344085	0.9999537652	-1.0081408879
15.1008094413	0.2914548718	0.9999537652	1.1381128106
2.5670947518	0.0290037763	0.9999537652	1.5324445939
6.3075231393	0.4850059918	0.9999537652	0
-1.2455951775	0.2013552927	0.9999537652	-1.3257163779

-1.197576325	0.6623620233	0.9999537652	-1.1865364646
2.7933974408	0.6948713406	0.9999537652	-5.3589986948
-1.1959606133	0.3530227076	0.9999537652	1.0692639063
-1.6671991184	0.0448815724	0.9999537652	-1.2640384552
-1.1077836192	0.5143282245	0.9999537652	-1.1763800783
2.7933974408	0.6948713406	0.9999537652	0
0	0	0	6.908559683
-1.0857945282	0.8711902386	0.9999537652	1.5123051406
-1.0130402092	0.9187989799	0.9999537652	-1.1249871506
1.0988560272	0.818465598	0.9999537652	-1.0094721103
4.5505976667	0.5557790267	0.9999537652	-3.20894012
0	0	0	0
-1.0825391702	0.7172610732	0.9999537652	-1.3173815964
1.0975512742	0.5165143285	0.9999537652	1.103672028
11.5766527773	0.345372235	0.9999537652	33.3754495834
1.2185387303	0.2116757194	0.9999537652	-1.3716623699
-1.1254592709	0.4217652485	0.9999537652	1.1872285251
2.0570832917	0.1751146452	0.9999537652	2.4808300066
-1.4021492508	0.0389210208	0.9999537652	-1.2995589273
1.4462181475	0.2775525965	0.9999537652	-1.3205774937
1.0397146958	0.7360116622	0.9999537652	1.0398318525
1.0285866397	0.8754624479	0.9999537652	-1.1562757244
0	0	0	2.996475627
2.7933974408	0.6948713406	0.9999537652	2.0426682045
0	0	0	-1.5087935345
1.3079063983	0.0501264509	0.9999537652	-1.1940289567
-1.158439708	0.2835288814	0.9999537652	-1.4005988103
-2.8760826119	0.1553805544	0.9999537652	-2.2511269746
1.0829616751	0.5108588824	0.9999537652	1.0468957885
2.9313230887	0.0632300451	0.9999537652	1.7346310769
-1.0108728944	0.9635058103	0.9999537652	-1.1572161701
1.2356088368	0.1875473958	0.9999537652	1.0019706692
1.1391698979	0.6399915339	0.9999537652	-1.1659764659
1.1919080759	0.4569336392	0.9999537652	1.1204931101
5.496040989	0.1012496507	0.9999537652	-1.283611481
1.0028347771	0.9851256821	0.9999537652	-1.1084524378
1.0434550088	0.7835112812	0.9999537652	-1.2061968396
-1.418412925	0.3259607511	0.9999537652	-1.3914488087
1.0809853157	0.7527577596	0.9999537652	1.0881480925
-1.2465935171	0.2873358005	0.9999537652	-1.3073503178
-1.1270828252	0.5697084709	0.9999537652	1.1620755807
2.7933974408	0.6948713406	0.9999537652	0
0	0	0	6.908559683
4.4401862342	0.0345830526	0.9999537652	-1.3580827111
-1.1880058961	0.1894267359	0.9999537652	-1.4114785674
1.0248488197	0.9101584263	0.9999537652	-1.1847961128
1.0003784265	0.9992134816	0.9999537652	-1.0969187291
-1.0075197723	0.9684314815	0.9999537652	-1.0656795983
1.0336868444	0.8314678021	0.9999537652	-1.2094196508

1.0338424695	0.887545736	0.9999537652	-1.1570730669
-1.0412872224	0.7600874351	0.9999537652	1.0260560303
1.136013751	0.3160499214	0.9999537652	1.2627590312
0	0	0	1.6601562553
-1.6994504307	0.542122934	0.9999537652	1.791511063
0	0	0	2.996475627
1.2379676245	0.4612616912	0.9999537652	1.1838852129
1.7141897291	0.009923	0.9999537652	1.3480408936
6.3075231393	0.484996674	0.9999537652	-1.6396378201
1.0676357032	0.8385135872	0.9999537652	-1.5109058578
-1.2028374646	0.5527646301	0.9999537652	-1.2918748817
1.1943111976	0.7386871802	0.9999537652	-1.1105035059
2.7933974408	0.6948713406	0.9999537652	1.0675791322
1.0146958614	0.971023082	0.9999537652	1.0969125818
-1.1572711259	0.8283974137	0.9999537652	-1.1845093957
1.1277902631	0.3932117799	0.9999537652	-1.0636997129
0	0	0	2.996475627
-1.3062078421	0.4273097615	0.9999537652	-1.0058955941
1.1119208683	0.6351603186	0.9999537652	-1.2197776484
-1.1282426787	0.6001528702	0.9999537652	-1.1398488343
-1.0491282329	0.8974337407	0.9999537652	-1.0780915189
-1.2508804687	0.5573146363	0.9999537652	-1.0256555556
1.4666339935	0.0313181681	0.9999537652	1.0826045061
1.5847055173	0.4398070576	0.9999537652	1.4302724714
0	0	0	0
-1.0908430523	0.6839889892	0.9999537652	-1.1721388968
1.2117886498	0.3825788676	0.9999537652	1.3031671303
6.3075231393	0.4850126447	0.9999537652	-1.0834689998

LZ vs. C 24H - P-value	LZ vs. C 24H - FDR p-value	C1_U937_0H_R1_001 (GE) - TPM
0.4506277405	0.9999986525	0
0.9634256918	0.9999986525	1.4585853856
0.2259641952	0.9999986525	0
0.1185994695	0.9999986525	0
0.4130863814	0.9999986525	17.4915275307
0.1272623854	0.9999986525	37.6100539265
0.0674732874	0.9999986525	0
0.571066625	0.9999986525	0
0.1330005216	0.9999986525	1475.2491124668
0.0486760051	0.9999986525	10.264240868
0.5151446629	0.9999986525	0
0.211727368	0.9999986525	6.1151992388
0.6312664098	0.9999986525	0
0.9157910673	0.9999986525	107.9967984839
0.8468849379	0.9999986525	132.3657098404
0.2161354344	0.9999986525	0
0.0862692722	0.9999986525	103.2814927958
0.7351839838	0.9999986525	4.5470441865
0.5429585224	0.9999986525	6.7962833903
0.5569763241	0.9999986525	84.7463386173
0.3115315717	0.9999986525	36.276063805
0.6050312545	0.9999986525	25.5078088512
0.4729816619	0.9999986525	0
0.0233257409	0.9999986525	19.0457692846
0.2248214915	0.9999986525	0
0.4814039658	0.9999986525	370.8408535493
0.0398367704	0.9999986525	46.5047709653
0.838654829	0.9999986525	311.9315952827
0	0	0
0.8560159159	0.9999986525	5.598401296
0.3835753132	0.9999986525	16.3982003808
0.2266986865	0.9999986525	9.4597739972
0.5778832954	0.9999986525	31.8060332588
0.6751120977	0.9999986525	25.2819139427
0.8418079538	0.9999986525	0
0.5694449709	0.9999986525	0
0.7780577599	0.9999986525	12.8741167367
0.2834629282	0.9999986525	3.3717826939
0.5998253505	0.9999986525	2.3808284825
0.1387183735	0.9999986525	29.9483517019
0.4147841635	0.9999986525	25.2499694419
0.7311715355	0.9999986525	13.4738294493
0	0	0
0.6312664098	0.9999986525	0
0.9068866046	0.9999986525	0
0.4874497034	0.9999986525	0
0.0330299741	0.9999986525	0
0.3299717062	0.9999986525	0

0.6622446636	0.9999986525	7.4751662328
0.7918445591	0.9999986525	3.8872349926
0.7377822442	0.9999986525	0.749144218
0.8163586565	0.9999986525	59.7992139
0.7449896062	0.9999986525	0
0.6312664098	0.9999986525	0
0.0189955297	0.9999986525	0
0.2662910164	0.9999986525	21.4342200594
0.3341139676	0.9999986525	22.3787789095
0.5073272766	0.9999986525	0
0.9446520117	0.9999986525	0
0.6148601643	0.9999986525	30.825244101
0.6024143212	0.9999986525	21.8453723081
0.72751791	0.9999986525	0.6314659568
0.7023495176	0.9999986525	0
0.537350719	0.9999986525	55.132736251
0.2332801701	0.9999986525	2.3313800616
0.3897704795	0.9999986525	11.2024431491
0.3502688179	0.9999986525	9.4640118675
0.177195026	0.9999986525	1.2219221282
0.46598719	0.9999986525	11.6398585571
0.3371199317	0.9999986525	0
0.5316072528	0.9999986525	604.1044071092
0.6718319089	0.9999986525	0
0.3408234741	0.9999986525	53.5565693209
0.7852254468	0.9999986525	3.8748289234
0.3293281315	0.9999986525	7.3412141633
0.7999534346	0.9999986525	31.7658596333
0.6312664098	0.9999986525	0
0.3644843651	0.9999986525	0
0.6868158828	0.9999986525	47.7168871039
0.0345378896	0.9999986525	40.8697374551
0.3646074104	0.9999986525	48.2671524642
0.4627647269	0.9999986525	0
0.505065311	0.9999986525	0.1073035967
0.3936513486	0.9999986525	15.5885236795
0.2395309877	0.9999986525	4.3363316934
0.2064416691	0.9999986525	0
0.4564667208	0.9999986525	0
0.9234882051	0.9999986525	183.2807691632
0.8875281597	0.9999986525	0
0.4482922011	0.9999986525	1.783414253
0.3482502561	0.9999986525	878.6265067219
0.7624875438	0.9999986525	0
0.5615640939	0.9999986525	0
0.2243977175	0.9999986525	3.7412235979
0.5772687741	0.9999986525	0
0.9264203483	0.9999986525	5.7765900004
0.6307937915	0.9999986525	0

0.1212827366	0.9999986525	2.4391602922
0.0694075232	0.9999986525	10.1428688739
0.7018160942	0.9999986525	0
0.249589136	0.9999986525	30.8710142213
0.2611516293	0.9999986525	6.0828963677
0.8218483791	0.9999986525	9.3631134541
0.8355935438	0.9999986525	4.9555635211
0.0399138881	0.9999986525	0.9813612469
0.4729816619	0.9999986525	0
0.5288006053	0.9999986525	0.7124644128
0.408166978	0.9999986525	1.2361506449
0.5285116491	0.9999986525	78.5337073997
0.0224847831	0.9999986525	8.3079940579
0.8147085498	0.9999986525	0
0.6839962716	0.9999986525	0
0.3127086061	0.9999986525	26.3498677331
0.9287501179	0.9999986525	0.3389570718
0.4284809222	0.9999986525	0.6598646663
0.9355755708	0.9999986525	112.0751042944
0.2920446467	0.9999986525	0
0.934330816	0.9999986525	386.773476575
0.3580399981	0.9999986525	4.7582964346
0.5843089519	0.9999986525	2.7080415188
0.5183818903	0.9999986525	6.0059040476
0.8371741487	0.9999986525	0
0.6902524954	0.9999986525	0
0.0759865039	0.9999986525	0
0.6754746348	0.9999986525	24.4099223416
0.30753857	0.9999986525	1.5032995055
0.0728228538	0.9999986525	39.555600224
0.0127526944	0.9999986525	7.776664115
0.0757593414	0.9999986525	0
0.4743912191	0.9999986525	262.4869492318
0.3465642132	0.9999986525	15.504624198
0.4713682857	0.9999986525	9.5060299792
0.6522807607	0.9999986525	12.3705305027
0.2876054532	0.9999986525	67.4618943636
0.9834089352	0.9999986525	1.4982193682
0.4615238273	0.9999986525	0
0.3283924407	0.9999986525	38.2709552365
0.4564100194	0.9999986525	1.6739089014
0.7136040962	0.9999986525	0.1725012068
0.3588599846	0.9999986525	0
0.6312664098	0.9999986525	0
0.4769688016	0.9999986525	20.6617118593
0.3075114912	0.9999986525	0
0.887686936	0.9999986525	0
0.9997772097	0.9999986525	0
0.9195725661	0.9999986525	3.1519406915

0.3680775443	0.9999986525	0
0.5400503825	0.9999986525	0
0.5507284732	0.9999986525	1.2113283997
0.8624853065	0.9999986525	0
0.667486048	0.9999986525	4.9577499086
0.4870574405	0.9999986525	0
0.6312664098	0.9999986525	0
0.3047359984	0.9999986525	0
0.0157178781	0.9999986525	883.5042082468
0.219536839	0.9999986525	1.7111819021
0.9413685068	0.9999986525	1.541382157
0.573738744	0.9999986525	14.2317214299
0.2754251837	0.9999986525	0
0.1204457024	0.9999986525	0
0.8629812278	0.9999986525	1.4996569367
0.7828576125	0.9999986525	6.7886395098
0.3166916357	0.9999986525	24.4773296275
0	0	0
0.4126362447	0.9999986525	1.0319450991
0.6098761915	0.9999986525	0
0.3360834124	0.9999986525	10.7269052963
0.1065378049	0.9999986525	0
0.3523102098	0.9999986525	0
0.008737	0.9999986525	13.4087072706
0.8094366416	0.9999986525	1.0022947683
0.1880955406	0.9999986525	0
0.4729816619	0.9999986525	0
0.5020686701	0.9999986525	105.6278364526
0.0223511299	0.9999986525	1.1889737837
0.0477135024	0.9999986525	2.9442889006
0.4729816619	0.9999986525	0
0.6017159452	0.9999986525	10.1014826062
0.2475265649	0.9999986525	1.0080338638
0.1930727657	0.9999986525	8.5541835763
0.3393823594	0.9999986525	17.6556446043
0.1788520882	0.9999986525	18.5355351754
0.9503183601	0.9999986525	0
0.1994606277	0.9999986525	123.0963238668
0.2281222615	0.9999986525	2.5932396262
0.7957967009	0.9999986525	22.2951264735
0.7756127722	0.9999986525	67.5349235958
0.4071957235	0.9999986525	0
0	0	3.4682110734
0.4170868729	0.9999986525	0.4469203475
0.465462121	0.9999986525	1.6908569946
0.6098761915	0.9999986525	0
0.9268829827	0.9999986525	0
0.4730788793	0.9999986525	4.0767208202
0.750799056	0.9999986525	0

0.858220382	0.9999986525	0
0.3187440932	0.9999986525	5.8448338011
0.7686138325	0.9999986525	3.5258640915
0.4222684215	0.9999986525	11.6993782414
0.8625459366	0.9999986525	81.0195104719
0.4986738748	0.9999986525	0
0.8105190729	0.9999986525	24.5738657468
0.8131244057	0.9999986525	0.8540666885
0.5389206123	0.9999986525	2.0582999426
0.9823558256	0.9999986525	1.0974092637
0.7252613376	0.9999986525	0
0.5618134639	0.9999986525	5.5159186549
0.007867	0.9999986525	0
0.0629929562	0.9999986525	0
0	0	0
0.248556393	0.9999986525	44.5388600267
0.5556468149	0.9999986525	0.5161641734
0.9516558556	0.9999986525	0
0.6343729656	0.9999986525	0
0.640379247	0.9999986525	0
0.3460793044	0.9999986525	0
0.1750541701	0.9999986525	0.8220193034
0.6920704993	0.9999986525	2.2523847759
0.1141394891	0.9999986525	29.0745489823
0.7958425602	0.9999986525	0
0.8508147212	0.9999986525	2.3563440884
0.6292476793	0.9999986525	0
0.4689847935	0.9999986525	22.719217101
0.6646523436	0.9999986525	0
0.8136369685	0.9999986525	1500.2016797661
0.4130623505	0.9999986525	11.3007207075
0.9398460025	0.9999986525	42.9076211852
0.2826233433	0.9999986525	222.3275867241
0.0856348148	0.9999986525	50.7240858877
0.4873057326	0.9999986525	0
0.5573912876	0.9999986525	47.6148320419
0.9250293643	0.9999986525	3.7017371777
0.0539059284	0.9999986525	85.9961450533
0.6842953402	0.9999986525	79.6301764464
0	0	0
0.6312664098	0.9999986525	0
0.4072384421	0.9999986525	0
0.8415546651	0.9999986525	0
0.1113097011	0.9999986525	0
0.2612097461	0.9999986525	1.7715657529
0.8755585025	0.9999986525	0
0.6748018463	0.9999986525	0
0.5225600898	0.9999986525	27.9161774569
0.4059433862	0.9999986525	0.533172329

0.243973771	0.9999986525	26.5485513872
0.1040132575	0.9999986525	77.3112885132
0.242846765	0.9999986525	3.8502929407
0.0541537344	0.9999986525	10.9736365995
0.8223960475	0.9999986525	0
0.1931476407	0.9999986525	3.046136707
0.5201550731	0.9999986525	7.8959746179
0.3736891372	0.9999986525	0
0.368278062	0.9999986525	8.7793957267
0.482222045	0.9999986525	0
0.1124512159	0.9999986525	3.834597998
0.0382475638	0.9999986525	0
0.9772740273	0.9999986525	698.2273694647
0.5678846271	0.9999986525	1.375326638
0.535957178	0.9999986525	0
0.0157103331	0.9999986525	0
0.7747697331	0.9999986525	667.0405719519
0.3075114913	0.9999986525	0
0.6403713668	0.9999986525	0
0.9790695191	0.9999986525	2.3652612399
0.1797579448	0.9999986525	410.5468379613
0.4552680037	0.9999986525	0
0.1203660109	0.9999986525	385.3550100998
0.4072698906	0.9999986525	0
0.3622138515	0.9999986525	4.060006526
0.4582577267	0.9999986525	3007.5978952128
0.0882024129	0.9999986525	41.9972843291
0.9377380765	0.9999986525	4.9083567125
0.5995967067	0.9999986525	0
0.5665597555	0.9999986525	5.4614372024
0.6098761915	0.9999986525	0
0.16828652	0.9999986525	9.605150173
0.8907647941	0.9999986525	5.2898555916
0.1499152499	0.9999986525	17.7307245612
0	0	0
0.8841889965	0.9999986525	0
0.1582566073	0.9999986525	1.8754276562
0.4186354209	0.9999986525	0.7125653448
0.5266496436	0.9999986525	3.7046800103
0.0617216597	0.9999986525	4.4229058057
0.4517787603	0.9999986525	0
0	0	0
0	0	0
0.0674244865	0.9999986525	15.3596700363
0.5660525099	0.9999986525	0.7551965886
0.5982406329	0.9999986525	6.8079643655
0.1072110811	0.9999986525	154.5019548307
0.6237611602	0.9999986525	1.429837784
0.9364804364	0.9999986525	4.1204539283

0.3370539183	0.9999986525	17.6618300967
0.2200976837	0.9999986525	14.9244558746
0.4468279444	0.9999986525	27.2306874072
0.7618760922	0.9999986525	0
0.425216001	0.9999986525	0
0.5559263079	0.9999986525	2.2316745921
0.3446318237	0.9999986525	11.2920224554
0.0893537083	0.9999986525	38.6986028403
0.8952792282	0.9999986525	969.7841063888
0.6639819905	0.9999986525	4.5845415127
0.6973159579	0.9999986525	7.5816707187
0.3005072878	0.9999986525	2.0189424075
0.6229053388	0.9999986525	36.5285415049
0.9221338831	0.9999986525	20.687813164
0.5046863539	0.9999986525	4.6315810073
0.4520587775	0.9999986525	3.3800186594
0.2438062878	0.9999986525	19.115277295
0.6403634807	0.9999986525	0
0.7093091139	0.9999986525	0.6801359685
0.376427148	0.9999986525	70.6795282007
0.3033692161	0.9999986525	12.2865925849
0.8043208143	0.9999986525	5.515525897
0.6158538344	0.9999986525	0
0.0538342385	0.9999986525	0
0.9034463357	0.9999986525	120.7270428064
0.405427986	0.9999986525	3.1480891858
0.3370713275	0.9999986525	0
0.0192835558	0.9999986525	4.2557548933
0.6141191168	0.9999986525	3.0713355467
0.4329422654	0.9999986525	37.7026829143
0.3653766082	0.9999986525	6.3083992148
0.4858717369	0.9999986525	0
0	0	0
0.6974381111	0.9999986525	20.608969131
0.3047473685	0.9999986525	0
0.9318415258	0.9999986525	0
0.8978638139	0.9999986525	4.1212577625
0.4145250353	0.9999986525	99.1283227572
0.1342951761	0.9999986525	31.4899641616
0.4551008589	0.9999986525	3.4254714117
0.8736424672	0.9999986525	6.165708575
0.3162242032	0.9999986525	2.8368651354
0.8816504087	0.9999986525	116.0745455523
0.0949940599	0.9999986525	0
0.7080007137	0.9999986525	0
0.6397342202	0.9999986525	0
0.3387051196	0.9999986525	47.4329172774
0.30755387	0.9999986525	0
0.4112661233	0.9999986525	34.3393486517

0.6831591816	0.9999986525	240.9593946451
0.1062507322	0.9999986525	0
0.3825614801	0.9999986525	0
0.6908209882	0.9999986525	1.7705561107
0.3110228613	0.9999986525	0.7905565103
0.383385698	0.9999986525	5.2474582592
0.3176196955	0.9999986525	0
0.007017	0.9999986525	18.4866044984
0.7747537202	0.9999986525	0
0.6924349683	0.9999986525	22.2135369678
0.116702404	0.9999986525	5.0164004679
0.4073013259	0.9999986525	0
0.7533613327	0.9999986525	7.7202043892
0.284274112	0.9999986525	4.8351110708
0.3355987706	0.9999986525	116.667498498
0.5439839741	0.9999986525	0
0.0856550969	0.9999986525	2.6293895363
0.7269323251	0.9999986525	1.1891544869
0.6312664098	0.9999986525	0
0.1177570616	0.9999986525	5.9012711832
0.0707117235	0.9999986525	0
0.532794195	0.9999986525	0
0.9245462844	0.9999986525	61.418025213
0.30755387	0.9999986525	0
0.1022579231	0.9999986525	13.81839828
0.2745679642	0.9999986525	1.153041617
0.7094206677	0.9999986525	1.8638123348
0.8641842077	0.9999986525	393.3821819979
0.6419319204	0.9999986525	9.6496509601
0.7498330569	0.9999986525	4.678276874
0.3570275522	0.9999986525	5.8760478667
0.2668367678	0.9999986525	6.4604181317
0.9555576649	0.9999986525	31.210948271
0.1661533338	0.9999986525	9.9386372274
0.8179323728	0.9999986525	7.4654677326
0.2713237761	0.9999986525	2.1015864478
0.9959190878	0.9999986525	2.6493947574
0.4521095247	0.9999986525	1.4386793306
0.3522627872	0.9999986525	187.0991001916
0.6403555884	0.9999986525	0
0.0597950727	0.9999986525	113.8406243523
0.6192182591	0.9999986525	14.856235788
0.8703432821	0.9999986525	18.921300973
0.6233722125	0.9999986525	0
0.1462408097	0.9999986525	0
0.7844005642	0.9999986525	10.2642092042
0.3663674881	0.9999986525	151.2292812648
0.2804437999	0.9999986525	0
0.6719097504	0.9999986525	6.0852538572

0.7307116682	0.9999986525	9.1733299374
0.4919359015	0.9999986525	0
0.7400241978	0.9999986525	42.8510492708
0.727695324	0.9999986525	42.489073392
0.2035119645	0.9999986525	2.6544993077
0.6312664098	0.9999986525	0
0.7171034182	0.9999986525	9.0515387376
0.934373702	0.9999986525	14.7154968588
0.2321752268	0.9999986525	125.0286187008
0.3551554379	0.9999986525	0
0.246602392	0.9999986525	33.8941844605
0.9972987842	0.9999986525	60.3357786287
0.4399946605	0.9999986525	191.586160434
0.5281129983	0.9999986525	0
0.3607544229	0.9999986525	3.0564317592
0.4034495718	0.9999986525	43.034035252
0.2487253046	0.9999986525	24.1407465324
0.3700012234	0.9999986525	65.3961345051
0.30755387	0.9999986525	0
0.6256058169	0.9999986525	58.6088702747
0.2053839859	0.9999986525	43.6518747394
0.1643863901	0.9999986525	0
0.1930391795	0.9999986525	0
0.1203824212	0.9999986525	24.9424792595
0.5212598977	0.9999986525	367.2029797562
0.6922058901	0.9999986525	1.8348541714
0.500591718	0.9999986525	0
0.9076415054	0.9999986525	72.4515932518
0.4069532021	0.9999986525	0
0.8624143192	0.9999986525	0
0.1066403629	0.9999986525	27.3865551233
0.3075114913	0.9999986525	0
0	0	0
0.198214479	0.9999986525	4.9729449143
0.56576422	0.9999986525	0
0.3054290442	0.9999986525	7.1096342769
0.6984869757	0.9999986525	64.8421341023
0.7101248559	0.9999986525	51.1907450196
0.6048968113	0.9999986525	4.6471839853
0.9597323569	0.9999986525	2.9231447752
0.3946922803	0.9999986525	3.0764791883
0.9513392532	0.9999986525	9.5791149722
0.782217035	0.9999986525	0
0.7926335687	0.9999986525	2.7616564645
0.9296354279	0.9999986525	13.4763761741
0.4613241911	0.9999986525	3.1397609076
0.8851573384	0.9999986525	0
0.261436769	0.9999986525	1.3197870462
0.4729816619	0.9999986525	0

0.7001891865	0.9999986525	122.7279277141
0.8054555569	0.9999986525	15.8757682467
0.30755387	0.9999986525	0
0.273765018	0.9999986525	2.2112574331
0.8810479534	0.9999986525	4.876406281
0.2448357755	0.9999986525	0.2991967903
0.44347263	0.9999986525	1.6572815891
0.904996925	0.9999986525	0
0.7773454679	0.9999986525	0
0.4640361131	0.9999986525	3.0992264671
0.1985906326	0.9999986525	0
0.064490339	0.9999986525	58.4070926722
0.1007612426	0.9999986525	154.2619893485
0.1465190801	0.9999986525	7.6357472134
0.9611923278	0.9999986525	6.2148644653
0.7804070177	0.9999986525	0
0.9396104387	0.9999986525	0
0.9361376845	0.9999986525	3.7877088411
0.3033010284	0.9999986525	0
0.374126427	0.9999986525	14.0620455605
0.3060346534	0.9999986525	75.4754101126
0.3410865114	0.9999986525	0
0	0	0
0	0	0
0.7033868751	0.9999986525	0
0.2278685735	0.9999986525	87.0081025145
0.2449368409	0.9999986525	33.8695499527
0.4763504063	0.9999986525	98.3832515647
0.2903516429	0.9999986525	0
0.007049	0.9999986525	116.2342079259
0.3087536661	0.9999986525	1.5620028065
0.2593850616	0.9999986525	8.5797235436
0.5546980174	0.9999986525	0.708376014
0.5440740112	0.9999986525	2.48922648
0.0878994065	0.9999986525	46.6966562567
0.5693285527	0.9999986525	1272.2761576554
0.1276143382	0.9999986525	35.2975226241
0.4843644183	0.9999986525	19.9976971701
0	0	0
0.2186993699	0.9999986525	1.1580221945
0.2056017556	0.9999986525	0
0.3860963202	0.9999986525	1.5738452414
0.6181946527	0.9999986525	5.351236157
0.6269897914	0.9999986525	0
0.5518060966	0.9999986525	13.4898800877
0.4187680111	0.9999986525	37.6381971396
0.556099491	0.9999986525	6787.2638832043
0.3234421057	0.9999986525	0
0.1828504385	0.9999986525	18.3032120001

0	0	0
0.4729816619	0.9999986525	0
0.1857285465	0.9999986525	0
0.9991381713	0.9999986525	1.1632936225
0.8570359306	0.9999986525	0
0.2652809746	0.9999986525	268.7731207446
0.1835087902	0.9999986525	0
0.2701757466	0.9999986525	0
0.9625339662	0.9999986525	0
0.5235343277	0.9999986525	35.0379820166
0.8319106678	0.9999986525	0
0.9966126035	0.9999986525	0
0.4923631264	0.9999986525	445.4615235013
0.9148634706	0.9999986525	30.1433663929
0.4206886897	0.9999986525	60.4987751382
0.4753063306	0.9999986525	8.8930285961
0.85793303	0.9999986525	42.1701678588
0.5529206873	0.9999986525	0.9349250881
0.0009277	0.9999986525	0.2119908572
0.1206685035	0.9999986525	1.1619712714
0.1390495836	0.9999986525	0
0.186815333	0.9999986525	1.6700053194
0.7706346193	0.9999986525	8.5784228675
0.9541020489	0.9999986525	4.2306979794
0.9931675261	0.9999986525	577.6835719399
0.9124875608	0.9999986525	0
0.2514646832	0.9999986525	0
0.3355210908	0.9999986525	0
0.9270581493	0.9999986525	5.3096391027
0.6143104157	0.9999986525	0
0.3894274584	0.9999986525	1.5817759809
0.7189290499	0.9999986525	0
0.6463390272	0.9999986525	4.811544502
0.1544292614	0.9999986525	0
0.4786564218	0.9999986525	6.5354660677
0.6388357637	0.9999986525	12.8977443885
0	0	0
0.7949282995	0.9999986525	11.2970948078
0.8623434941	0.9999986525	0
0.5546077001	0.9999986525	0
0.6587773694	0.9999986525	0
0.9384108176	0.9999986525	0
0.8888177542	0.9999986525	0.2035210586
0.7557287784	0.9999986525	35.5469750808
0.0449470888	0.9999986525	1.4206837452
0.607944535	0.9999986525	0
0.2163416833	0.9999986525	1.5995299053
0.0982087439	0.9999986525	3.6772946208
0.5124952471	0.9999986525	4.707857128

0.7054169577	0.9999986525	0
0.5776538437	0.9999986525	19.0499968631
0.5934498605	0.9999986525	31.1402819731
0.4415361606	0.9999986525	16.8236157908
0.658865423	0.9999986525	0.2555545147
0.3688572167	0.9999986525	5.9158687456
0.1212939141	0.9999986525	4.7919717723
0.375749911	0.9999986525	31.318789223
0.4069913918	0.9999986525	0.405671126
0.0948808174	0.9999986525	0
0.757756129	0.9999986525	0
0.2128048764	0.9999986525	12.0401641329
0.6715356757	0.9999986525	1.0143842932
0.6899524879	0.9999986525	0
4.54E-06	0.0407058325	0.8491734515
0.3047587416	0.9999986525	0
0.7395121592	0.9999986525	2.9722504489
0.2223344251	0.9999986525	56.5200164477
0.3661245927	0.9999986525	58.5869900966
0.5025755316	0.9999986525	11.0816511794
0.2116622849	0.9999986525	0
0.8265626471	0.9999986525	13.0060406342
0.9985712836	0.9999986525	1.3863376267
0.4654335785	0.9999986525	0
0.1537643425	0.9999986525	14.938476951
0.7165071559	0.9999986525	33.4688962144
0.1817955974	0.9999986525	19.63853725
0.779299769	0.9999986525	2.7006043171
0.4860110914	0.9999986525	3.8080552474
0.3663491982	0.9999986525	60.4827085481
0.2549654881	0.9999986525	3.6510746504
0.67714376	0.9999986525	0
0.9623721001	0.9999986525	3.6801559631
0.3256808776	0.9999986525	9.8401248745
0.4818048973	0.9999986525	6.8983696743
0.6397203658	0.9999986525	0
0.9751629192	0.9999986525	0
0.0565350529	0.9999986525	221.8010493795
0.4081785001	0.9999986525	0
0.7891405437	0.9999986525	0.3544177687
0.3715804129	0.9999986525	0.622526677
0.6312664098	0.9999986525	0
0.6082681169	0.9999986525	0
0.7042490002	0.9999986525	0
0.9309463343	0.9999986525	0.799230009
0.0193968536	0.9999986525	2.3290154224
0.0939207155	0.9999986525	190.7213417742
0.3351267446	0.9999986525	0.1132553583
0	0	0

0.6674082526	0.9999986525	0.4108274142
0.6871652938	0.9999986525	9.5209533274
0.8979104298	0.9999986525	0
0.2461578326	0.9999986525	1.9032042604
0.9599061563	0.9999986525	88.5853734523
0.8985579538	0.9999986525	0.4520907387
0.650543462	0.9999986525	0
0.2486417829	0.9999986525	0.7441409864
0.4294688278	0.9999986525	3.829500524
0.3189578376	0.9999986525	0.8504656719
0.7191730753	0.9999986525	9.4634465987
0.6312664098	0.9999986525	0
0.7079684115	0.9999986525	0
0.0105637372	0.9999986525	0
0.1825681224	0.9999986525	1.3511773181
0.7648639571	0.9999986525	0
0.7719352838	0.9999986525	1.8683287897
0.8576692991	0.9999986525	0
0.1632502447	0.9999986525	10.1826320488
0.9542873749	0.9999986525	0.1814693017
0.7034331269	0.9999986525	0
0.182104656	0.9999986525	0
0.1199022358	0.9999986525	2.4863358914
0.3954779182	0.9999986525	1.9182042722
0.6437008393	0.9999986525	0
0.4065652646	0.9999986525	305.7144309861
0.6831720888	0.9999986525	0
0.0823935333	0.9999986525	1.1974678014
0	0	0
0.5960726121	0.9999986525	0
0.5649852868	0.9999986525	0
0.0482337233	0.9999986525	2.6026537884
0.8045402245	0.9999986525	33.0521510728
0.9267415074	0.9999986525	1.1005651043
0.786101268	0.9999986525	0
0.114994151	0.9999986525	5.1555952974
0	0	0
0.8634275808	0.9999986525	41.0797741635
0.8328280138	0.9999986525	0.060022023
0.8229458629	0.9999986525	0
0.7026058656	0.9999986525	0
0	0	0
0.4073327479	0.9999986525	0
0.2468302822	0.9999986525	0
0.6473371618	0.9999986525	4.7246683966
0.1453477485	0.9999986525	6.7136905685
0.002527	0.9999986525	0
0.6928329146	0.9999986525	0
0.833088655	0.9999986525	0.0946151954

0.007505	0.9999986525	21.4547561145
0.9941409674	0.9999986525	0
0.6066338885	0.9999986525	2.5992167441
0.5404797186	0.9999986525	6.6127955458
0.3193766118	0.9999986525	0
0.840139363	0.9999986525	0
0.6441042217	0.9999986525	20.863285981
0.5059410814	0.9999986525	0
0.6312664098	0.9999986525	0
0.6312664098	0.9999986525	3.4018626877
0.64034769	0.9999986525	0
0.9479756671	0.9999986525	0
0.5661134227	0.9999986525	0
0.6406654988	0.9999986525	1.0072648566
0.7119565445	0.9999986525	11.0524051954
0.2294506921	0.9999986525	95.9438989215
0.7789728056	0.9999986525	1.0521748825
0.0447793403	0.9999986525	4.9451234294
0.3231952833	0.9999986525	0
0.4729816619	0.9999986525	0
0.5786112196	0.9999986525	0
0.8839728645	0.9999986525	38.034714772
0.4029305119	0.9999986525	18.9824488189
0.1533102981	0.9999986525	0
0.2089832458	0.9999986525	7.1581761255
0.30755387	0.9999986525	0
0.9096023179	0.9999986525	1.0664738546
0.6312664098	0.9999986525	0
0.6186450893	0.9999986525	2.4218236032
0.0188212649	0.9999986525	9.1443875344
0.7075933834	0.9999986525	1.3517975251
0.2199274035	0.9999986525	7.7454905494
0.9904358696	0.9999986525	1.0416124611
0.0925444959	0.9999986525	2.687252562
0.3474737978	0.9999986525	2.7705419787
0.1954151179	0.9999986525	3.9106936858
0.6386618606	0.9999986525	0
0	0	0
0.2417500713	0.9999986525	0
0.3637326082	0.9999986525	3.7962850939
0.0518873822	0.9999986525	2.1059580605
0.6602663737	0.9999986525	1.0183351952
0.0473638989	0.9999986525	1.8303370581
0.9356814824	0.9999986525	3.9182371264
0.0216432315	0.9999986525	1.9748688713
0.3995448932	0.9999986525	0.3586854263
0.6055575796	0.9999986525	4.2334109436
0.2916818947	0.9999986525	0.2340420022
0.9297314549	0.9999986525	0

0	0	0
0.6023669343	0.9999986525	0
0.6312664098	0.9999986525	0
0.3951852412	0.9999986525	2.1540106643
0.3955611155	0.9999986525	0.5920112792
0.9138366331	0.9999986525	110.7129990287
0.870890766	0.9999986525	0
0.5535758736	0.9999986525	849.2529326439
0.2566437612	0.9999986525	0
0.6532321245	0.9999986525	6.1757250319
0.2449526119	0.9999986525	15.3588362426
0.8145494387	0.9999986525	2.1288209209
0.0637381866	0.9999986525	9.5034641722
0.3783674557	0.9999986525	0
0.0679421649	0.9999986525	0
0.8185476769	0.9999986525	10.8347355065
0.0480360382	0.9999986525	2.1645048453
0.5487824559	0.9999986525	1.9771903677
0.0647379053	0.9999986525	0
0	0	0
0.404245426	0.9999986525	0.5851892857
0.3370229016	0.9999986525	0
0.00593	0.9999986525	0
0.6967305144	0.9999986525	0.5651872034
0.515079977	0.9999986525	0.2505056496
0.2418267268	0.9999986525	0
0.1265334072	0.9999986525	0
0.0165928699	0.9999986525	0.9167014081
0.7617799981	0.9999986525	1.1658701595
0.0324370337	0.9999986525	4.1862649197
0.0977684646	0.9999986525	0
0.0578672091	0.9999986525	0
0.6467343928	0.9999986525	5.87352728
0.2929181318	0.9999986525	3.9691806874
0.3505327708	0.9999986525	0
0.8942355544	0.9999986525	4.5845415127
0.357534671	0.9999986525	2.6187507798
0.6220866265	0.9999986525	0
0	0	0
0	0	0
0	0	0
0.4058804319	0.9999986525	0
0.4456832924	0.9999986525	0
0.6312664098	0.9999986525	0
0.7433189725	0.9999986525	0
0.30755387	0.9999986525	0
0.334467179	0.9999986525	0.4023774512
0.0778827212	0.9999986525	8.0463638232
0.9199698486	0.9999986525	0.6687083684

0.8529886324	0.9999986525	7.9785358753
0.2112605292	0.9999986525	33.3140779392
0.6403397854	0.9999986525	0
0.1132335663	0.9999986525	0.8401297758
0	0	0
0.8115531521	0.9999986525	0.8953636155
0.9311735919	0.9999986525	17.0908772796
3.75E-06	0.0404027443	1.4666866124
0.1330296611	0.9999986525	0
0.6817644178	0.9999986525	8.5266254805
0.1465448262	0.9999986525	15.2201493448
0.9438011139	0.9999986525	0
0.2500243367	0.9999986525	0
0.3369746529	0.9999986525	0
0.008085	0.9999986525	3.847167703
0.0447520527	0.9999986525	0
0.2962808969	0.9999986525	6.9382446435
0	0	0
0.9029030402	0.9999986525	14.5215934209
0.1327505984	0.9999986525	0
0.2425974947	0.9999986525	51.7656635396
0.6098761915	0.9999986525	0
0.6312664098	0.9999986525	0
0.4519536033	0.9999986525	66.3525458461
0.7294663952	0.9999986525	18.3182894347
0.5750463239	0.9999986525	0
0.6892948998	0.9999986525	2.2025377374
0.1729574571	0.9999986525	64.5910129144
0.5638746735	0.9999986525	1.8136647743
0.1249991371	0.9999986525	87.0733064283
0.9480606913	0.9999986525	0
0.3155612349	0.9999986525	0
0.7001255887	0.9999986525	4.8591331517
0.5762735484	0.9999986525	2.3077962957
0.890077009	0.9999986525	5.8067901999
0.8100369647	0.9999986525	14.0290915971
0.3047701176	0.9999986525	0
0.4031083728	0.9999986525	0
0.5523075659	0.9999986525	0
0.9554647406	0.9999986525	16.8215049027
0.8851221672	0.9999986525	445.6870736397
0.6384876182	0.9999986525	10.0159147025
0.3422339338	0.9999986525	0
0	0	0
0.3982360831	0.9999986525	4.2378755175
0	0	0
0.234247603	0.9999986525	0
0.3042706813	0.9999986525	8.7949905456
0.3900990534	0.9999986525	0

0.8842513052	0.9999986525	0
0.258162169	0.9999986525	1.0215952072
0.4993119259	0.9999986525	28.3472254555
0.0893279	0.9999986525	11.2906545057
0.7045613178	0.9999986525	8.4138789591
0.1387580276	0.9999986525	0
0.3170977094	0.9999986525	0
0.4426572746	0.9999986525	18.986287362
0.3875832304	0.9999986525	1.7875452514
0.8679074176	0.9999986525	0.3575151005
0.9940924505	0.9999986525	1.6850038517
0.705666328	0.9999986525	0
0.0617222822	0.9999986525	1.8227094693
0.3892758046	0.9999986525	0
0.9493511461	0.9999986525	28.7840411807
0.4391490329	0.9999986525	5.5781493691
0.6207895474	0.9999986525	4.340305157
0.6305365023	0.9999986525	18.4406139058
0.9050847504	0.9999986525	2.6767427132
0.1462769727	0.9999986525	18.7442923858
0.2433871874	0.9999986525	0
0.8524412373	0.9999986525	29.7399587792
0.4501914175	0.9999986525	130.9499649736
0.3820287698	0.9999986525	18.8469687666
0.1078278587	0.9999986525	0
0.0283859267	0.9999986525	2.5458011726
0.8247478143	0.9999986525	5.6312821538
0.3330716111	0.9999986525	1.6935318007
0.4992899402	0.9999986525	0
0.4372153018	0.9999986525	31.753194301
0.453427394	0.9999986525	1.2581632376
0.4995890981	0.9999986525	0
0.9297102699	0.9999986525	0
0.7470079268	0.9999986525	0
0.5889932685	0.9999986525	5.5987277092
0.3402152744	0.9999986525	0
0.8407626413	0.9999986525	2.001715092
0.1042893014	0.9999986525	21.2123020366
0.3676274705	0.9999986525	2.2694189681
0.5002028914	0.9999986525	10.3293405489
0.2409078862	0.9999986525	0.2280777775
0	0	0
0.309050792	0.9999986525	0.4823805621
0.4453279771	0.9999986525	0
0.3863757695	0.9999986525	21.1320694657
0.8093903492	0.9999986525	2.6097540153
0.2103611826	0.9999986525	15.3944379589
0.7740588541	0.9999986525	0
0.3555793722	0.9999986525	0

0.0357995102	0.9999986525	0
0.215229913	0.9999986525	5.7633521458
0.7479445006	0.9999986525	0
0.6300503422	0.9999986525	0
0.6318611188	0.9999986525	2.9122645838
0.1264408692	0.9999986525	0
0.7132822282	0.9999986525	0
0.7351597178	0.9999986525	35.6646894677
0.603003061	0.9999986525	1.7786485044
0.1179761067	0.9999986525	6.9611069232
0.3521223914	0.9999986525	0
0.1933826462	0.9999986525	0
0.4738263135	0.9999986525	4.160869631
0.6264316288	0.9999986525	0
0.0518777466	0.9999986525	1.2803312039
0.3630310715	0.9999986525	0
0.3341083308	0.9999986525	0
0.3124784153	0.9999986525	0
0.2244967319	0.9999986525	3.4662762594
0.9407364286	0.9999986525	4.1903835592
0.2360184628	0.9999986525	0
0.173144363	0.9999986525	2.643736208
0.6252812617	0.9999986525	2.6507688329
0.5359618573	0.9999986525	2.488874563
0.0811921772	0.9999986525	119.7292856516
0.5414763012	0.9999986525	16.4128818231
0.3420264268	0.9999986525	0
0.9915675797	0.9999986525	27.8811763106
0.4862396544	0.9999986525	538.6472181583
0.8112152279	0.9999986525	25.7461414818
0.7539450509	0.9999986525	7.9972619967
0.3566945135	0.9999986525	0
0.9443362153	0.9999986525	62.784020716
0.7089408035	0.9999986525	0
0.2469025541	0.9999986525	0
0.3257710105	0.9999986525	0
0.0675764563	0.9999986525	2.7531626037
0.5573188289	0.9999986525	9.0620999016
0.9102396415	0.9999986525	6.8721704222
0.0435147719	0.9999986525	0
0.2979398545	0.9999986525	2.4326627056
0.8955782234	0.9999986525	2.1064480298
0.6043888669	0.9999986525	0
0.9352659065	0.9999986525	4.8483636834
0.2088281818	0.9999986525	0
0.253760786	0.9999986525	0
0.9039201331	0.9999986525	0.4344839865
0.4555145	0.9999986525	0
0.2714389319	0.9999986525	0

0.2292341018	0.9999986525	0
0.1161091319	0.9999986525	0.5981597643
0.4942972795	0.9999986525	0
0.4020224867	0.9999986525	0
0.719607977	0.9999986525	16.0985322779
0.5480387591	0.9999986525	0
0	0	115.5665606703
0	0	0
0.4729816619	0.9999986525	0
0.4640687041	0.9999986525	296.7074057658
0.1634764402	0.9999986525	0
0.4729816619	0.9999986525	0
0.6403318746	0.9999986525	133.3684803695
0.4008551543	0.9999986525	0
0.6210566055	0.9999986525	120.8556481151
0.3350762033	0.9999986525	0
0.5957680215	0.9999986525	0
0.3609789227	0.9999986525	42.7989612855
0.6051435013	0.9999986525	0
0.1208765209	0.9999986525	3.7490181457
0.9636729116	0.9999986525	0.6767978244
0.6428882271	0.9999986525	134.1211241639
0.2812086487	0.9999986525	175.6494461867
0.4465268932	0.9999986525	0
0.3367340473	0.9999986525	412.6820106478
0.7527485916	0.9999986525	0
0.6209341691	0.9999986525	0
0.6733033719	0.9999986525	45.3547983183
0.0248140912	0.9999986525	11.6440134672
0.5954200674	0.9999986525	5.1660480587
0.7286665691	0.9999986525	2.6086100082
0.127060821	0.9999986525	0
0.6408676262	0.9999986525	0
0	0	0
0.0870828969	0.9999986525	661.7009800906
0.3255355584	0.9999986525	112.6696922161
0.5353412149	0.9999986525	7.2529511807
0.0267054372	0.9999986525	4.4304500497
0.1990608982	0.9999986525	0
0.4202778218	0.9999986525	8.6626670119
0.0756329515	0.9999986525	8.2309542938
0.2532083734	0.9999986525	16.1165450797
0.0710252116	0.9999986525	0
0.1370143484	0.9999986525	0
0.810905397	0.9999986525	11.597683613
0.8455826455	0.9999986525	0
0.9656037028	0.9999986525	174.2075367553
0.1996872809	0.9999986525	6.0852538572
0.3256485882	0.9999986525	0

0.7863950567	0.9999986525	3.8156210112
0.8282674635	0.9999986525	0
0.001328	0.9999986525	0
0.5779101282	0.9999986525	0
0.0480938665	0.9999986525	0
0.6767591993	0.9999986525	74.1074624317
0.1894920339	0.9999986525	0
0.4156117	0.9999986525	49.3446124006
0.5117127747	0.9999986525	9.2235274583
0.6056852955	0.9999986525	107.446156016
0.7541187257	0.9999986525	40.8184084446
0.067757672	0.9999986525	1.4703615977
0.7648350374	0.9999986525	15.8125503672
0.4729816619	0.9999986525	0
0.801668901	0.9999986525	489.8424118798
0.2284490699	0.9999986525	6.515410588
0.3305037841	0.9999986525	1.8974605959
0.7528918336	0.9999986525	0
0.8114801862	0.9999986525	4.8532725204
0.6312664098	0.9999986525	0
0.3519887641	0.9999986525	197.7123752038
0.1305451842	0.9999986525	0
0.9972177788	0.9999986525	0
0.6312664098	0.9999986525	0
0	0	0
0.8832925677	0.9999986525	9.4745624701
0.8341821637	0.9999986525	13.9855022225
0.346785013	0.9999986525	466.3480638085
0.335034503	0.9999986525	0
0.3420313646	0.9999986525	0
0.0944131067	0.9999986525	2.820502976
0.5666890955	0.9999986525	532.4654641594
0.4729816619	0.9999986525	0
0.1831689936	0.9999986525	14.6020294924
0.9169990031	0.9999986525	3600.2184061757
0.2994182121	0.9999986525	0
0.2812654717	0.9999986525	6541.3948077377
0.6221171804	0.9999986525	0
0.6382889573	0.9999986525	0
0.8622728307	0.9999986525	0
0.6593552957	0.9999986525	0
0.9972261752	0.9999986525	0
0.0811501642	0.9999986525	59.9914689792
0.1120728721	0.9999986525	181.604595339
0.2285315232	0.9999986525	114.3034698113
0.0952462998	0.9999986525	1.1294920385
0.8803635686	0.9999986525	43.414647124
0.6054485315	0.9999986525	4.0196299738
0.2966255536	0.9999986525	33.8136953896

0.0425712523	0.9999986525	0
0.4763061874	0.9999986525	40.6886889263
0.5672041143	0.9999986525	0
0.9715942792	0.9999986525	1424.7078021664
0.6015773991	0.9999986525	52.6501178596
0.2666639544	0.9999986525	3222.6726843046
0.9166343292	0.9999986525	0.3249387407
0.9815084842	0.9999986525	140.5235660887
0.1973664543	0.9999986525	0
0.9861705847	0.9999986525	112.3939523863
0.810161631	0.9999986525	11.3160190204
0.8613872445	0.9999986525	350.7111986051
0.5202336508	0.9999986525	6.1490183055
0.8105701263	0.9999986525	31.2323585017
0.8529920687	0.9999986525	0
0.6312664098	0.9999986525	0
0.416596058	0.9999986525	20.9787162766
0.6403239576	0.9999986525	0
0.4445750527	0.9999986525	48.2633717473
0.2759028692	0.9999986525	0
0.1249702835	0.9999986525	61.8947643704
0.5533677611	0.9999986525	13.1116976729
0.2422464287	0.9999986525	55.5117912826
0.6457950968	0.9999986525	228.6316806334
0.3202754017	0.9999986525	0
0.829045351	0.9999986525	962.7537176671
0.2130237439	0.9999986525	43.0343599318
0.6044304734	0.9999986525	3.3220479448
0.1636473192	0.9999986525	3.4588983055
0.489092423	0.9999986525	0
0.1847119089	0.9999986525	183.6207500263
0.8142657574	0.9999986525	0
0.8588610868	0.9999986525	0
0.888505362	0.9999986525	0
0.255561553	0.9999986525	29.2922452725
0.3353073152	0.9999986525	2474.4441921551
0.3474584626	0.9999986525	228.981537834
0.5158067125	0.9999986525	0
0.3999046673	0.9999986525	14.8939419702
0.2541657083	0.9999986525	0
0.5307568397	0.9999986525	51.3654135638
0.3023797833	0.9999986525	25.3547375066
0.5132025384	0.9999986525	12.7585259636
0.0799545238	0.9999986525	102.0938196196
0.6286261041	0.9999986525	24.7673598885
0.8133012253	0.9999986525	0
0.025339143	0.9999986525	57.6039044334
0.9257760266	0.9999986525	28.640953485
0.5029300237	0.9999986525	30.8552589443

0.8407280613	0.9999986525	2.3174163329
0.918860048	0.9999986525	88.0598886641
0.2120985064	0.9999986525	24.9871533631
0.8180987111	0.9999986525	22.8240949374
0.9576821281	0.9999986525	2.8875054387
0.3004012757	0.9999986525	0
0.0167559588	0.9999986525	11.6544137314
0.9970764644	0.9999986525	9.8717604161
0.6160519669	0.9999986525	3.5664998239
0.891461767	0.9999986525	163.5927144725
0.6556425067	0.9999986525	22.7990149909
0.1016890845	0.9999986525	97.6743533908
0.6241710589	0.9999986525	5.2887961372
0.3153474239	0.9999986525	23.8628158259
0.7845787814	0.9999986525	9.4045226373
0.256917669	0.9999986525	8.3014646693
0.9270830015	0.9999986525	19.4957247052
0.6473030989	0.9999986525	0
0.6433495833	0.9999986525	0
0.052246338	0.9999986525	8.7450603805
0.6340069719	0.9999986525	0
0.0541927982	0.9999986525	123.1093664949
0.357559696	0.9999986525	91.4449101864
0.7039703373	0.9999986525	0
0.4589309121	0.9999986525	0
0.5170354479	0.9999986525	9.958984634
0.2871153574	0.9999986525	1.1189389455
0.4003935642	0.9999986525	117.9906327508
0.0834840512	0.9999986525	0
0.3301098732	0.9999986525	0
0.9422505447	0.9999986525	0
0.0789954175	0.9999986525	0
0.4665969982	0.9999986525	149.5698980921
0.2230152133	0.9999986525	12.4414412783
0.6864593209	0.9999986525	279.6500965084
0.9662041	0.9999986525	536.2078334715
0.6786700859	0.9999986525	3.1683915188
0.7599378019	0.9999986525	24.8746788933
0.1902447646	0.9999986525	0
0.4083142083	0.9999986525	0
0.8622023284	0.9999986525	0
0.2674981673	0.9999986525	0
0.074906931	0.9999986525	0
0	0	0
0	0	0
0.1427959265	0.9999986525	0
0	0	0
0.6098761915	0.9999986525	0
0.4274397564	0.9999986525	0

0	0	0
0.6988889454	0.9999986525	46.6785997692
0.102510938	0.9999986525	0
0.2000626373	0.9999986525	17.2256745683
0.5257323429	0.9999986525	2.2676325611
0.2574863455	0.9999986525	131.9523253624
0.6403160343	0.9999986525	0
0.8035396371	0.9999986525	4.8685396595
0.2096048762	0.9999986525	0
0.7593428708	0.9999986525	0
0.1026070483	0.9999986525	1967.3823761011
0.6403081047	0.9999986525	0
0.7813096581	0.9999986525	48.8791469008
0.350459501	0.9999986525	6.5170185373
0.335166057	0.9999986525	0
0.6891496274	0.9999986525	21.0037455662
0.2850480166	0.9999986525	11.273944035
0.563076021	0.9999986525	31.6516350391
0.9316346287	0.9999986525	15.3701970174
0.575694419	0.9999986525	9.9667707542
0.3352558168	0.9999986525	9.1405189038
0.6923773878	0.9999986525	0
0	0	0
0.2007315439	0.9999986525	4.7858924267
0.527285758	0.9999986525	0
0.663587728	0.9999986525	0
0.0652049094	0.9999986525	3.2259669686
0.807449067	0.9999986525	0
0.472558091	0.9999986525	24.2515983131
0.4030513474	0.9999986525	0
0.7090970231	0.9999986525	0
0.7115871701	0.9999986525	18.3461287803
0.6312664098	0.9999986525	0
0.9998761962	0.9999986525	84.4009879765
0.6978227218	0.9999986525	35.8060462551
0.4281512695	0.9999986525	0
0.7384622321	0.9999986525	62.7219654835
0.6448427365	0.9999986525	0
0.0339443063	0.9999986525	63.1242050514
0.3094057404	0.9999986525	0
0.4073641567	0.9999986525	0
0.4464961756	0.9999986525	257.5683185136
0.6629903015	0.9999986525	240.5492226907
0.5985528881	0.9999986525	0
0.0613958316	0.9999986525	29.4786326336
0.2537470584	0.9999986525	0
0.9839115973	0.9999986525	24.8341308274
0.6538354735	0.9999986525	35.6454109039
0.442457911	0.9999986525	0

0.6932350948	0.9999986525	14.1713851632
0.2733388544	0.9999986525	0
0.3368786822	0.9999986525	5.9575767881
0	0	0
0.2620969569	0.9999986525	0
0.8507698661	0.9999986525	7.1553879185
0.0169602151	0.9999986525	0
0.7551133781	0.9999986525	42.4037882186
0.6061251423	0.9999986525	1.8687915582
0.2863172618	0.9999986525	0
0.1731144663	0.9999986525	0
0.8116660277	0.9999986525	0
0.449978893	0.9999986525	4.8817400382
0.160015244	0.9999986525	2.7504384036
0.2733641246	0.9999986525	0
0.3371604181	0.9999986525	12.4885122313
0.6312664098	0.9999986525	0
0.805481544	0.9999986525	135.8812810604
0.9800742498	0.9999986525	26.0795162974
0.9908388129	0.9999986525	20.1164323769
0.5161148466	0.9999986525	8.7893449571
0.7141503441	0.9999986525	0
0.5721469803	0.9999986525	37.4102484337
0.0628764823	0.9999986525	7.9586977436
0.8512615133	0.9999986525	1.5344185193
0.3983256703	0.9999986525	0
0.003081	0.9999986525	16.4953285629
0.4716051222	0.9999986525	14.1278442213
0.9070426844	0.9999986525	5.2836562467
0.8524830549	0.9999986525	0
0.0445044323	0.9999986525	46.6119405773
0.4742661212	0.9999986525	3.4049819411
0.6372216083	0.9999986525	0
0.245091171	0.9999986525	40.8530523452
0.5625565014	0.9999986525	0
0.6020353216	0.9999986525	0
0.1812552361	0.9999986525	2.0302113564
0.2153252607	0.9999986525	0.2241877172
0.9171112969	0.9999986525	0
0.3137846545	0.9999986525	0
0.2925473203	0.9999986525	0
0.8095536552	0.9999986525	0
0.8373645272	0.9999986525	0
0.8819498609	0.9999986525	4.449286299
0.5848518692	0.9999986525	6.4103896473
0.233479945	0.9999986525	0.3442597317
0.244297269	0.9999986525	0
0.6403001687	0.9999986525	0
0.6402922262	0.9999986525	0

0.4555576695	0.9999986525	0
0.1702696067	0.9999986525	34.0076743247
0.8276730606	0.9999986525	15.6462504003
0	0	0
0.6880105509	0.9999986525	0
0.7112066796	0.9999986525	8.7071628532
0.0525586479	0.9999986525	0
0.407977789	0.9999986525	1.050605097
0.4439305308	0.9999986525	200.5084215122
0.2130990224	0.9999986525	3.9867083017
0	0	0
0.3651754684	0.9999986525	0
0.7786970695	0.9999986525	105.9872715474
0.6728680225	0.9999986525	0
0.355775883	0.9999986525	0
0.4591844069	0.9999986525	1.468787269
0.8223608363	0.9999986525	6.3149364161
0.4104587167	0.9999986525	7.7928817545
0.2396959147	0.9999986525	0
0.4027121381	0.9999986525	2.55596565
0.9423976894	0.9999986525	0.1263649579
0.1886534088	0.9999986525	192.9198950536
0.6802701349	0.9999986525	55.3394075528
0.8918416691	0.9999986525	0.6584537674
0.2034737987	0.9999986525	0.1248786259
0.7257447906	0.9999986525	1.0061557116
0.2237017344	0.9999986525	0
0.2004334085	0.9999986525	0
0.5396226041	0.9999986525	0.27379904
0.4729816619	0.9999986525	0
0.1336759182	0.9999986525	0
0.2482829048	0.9999986525	9.3621410655
0.9455265072	0.9999986525	94.176031074
0.6348338519	0.9999986525	4.8617424225
0.2732566303	0.9999986525	2.4388036184
0.2416762507	0.9999986525	0
0	0	0
0.9955703328	0.9999986525	19.1315166417
0.0867216339	0.9999986525	8.3311470543
0.6452772173	0.9999986525	1.6493216525
0.1385663282	0.9999986525	110.0239026056
0.2900481445	0.9999986525	3.1305794131
0.3680252162	0.9999986525	1.9681808247
0.6088857229	0.9999986525	0.2044911071
0.7053624616	0.9999986525	0
0.5214475827	0.9999986525	2.8768884533
0.7993309361	0.9999986525	3.6984536573
0.4146047929	0.9999986525	383.3631980561
0.6280620131	0.9999986525	0

0.8710287649	0.9999986525	0
0.7393554165	0.9999986525	138.4522441812
0.881957567	0.9999986525	0
0.4026834602	0.9999986525	3.3798744545
0.497646772	0.9999986525	91.1999969372
0.4795526219	0.9999986525	1.2901582525
0.7696306011	0.9999986525	22.7837934788
0.1450622162	0.9999986525	0
0.7869860509	0.9999986525	2.1621477498
0.336830958	0.9999986525	0
0.7303097298	0.9999986525	0
0.1290361262	0.9999986525	10.9183360474
0.904650437	0.9999986525	8.9616377081
0.0942666597	0.9999986525	157.1036648439
0.5126534258	0.9999986525	0
0.4112815837	0.9999986525	0
0.2330628872	0.9999986525	10.4146639798
0.088546908	0.9999986525	2.1823392076
0.009774	0.9999986525	4.919252827
0.3143393042	0.9999986525	5.4504119663
0.2557318937	0.9999986525	2.7704450897
0.4426345019	0.9999986525	31.1354928796
0.5181383129	0.9999986525	0
0.3657684939	0.9999986525	44.4045434901
0	0	0
0.008566	0.9999986525	0
0.5976164309	0.9999986525	7.4649820027
0.848824863	0.9999986525	1.1577429573
0.1829737027	0.9999986525	18.3892472933
0.1583893584	0.9999986525	1.3121714871
0.9322356637	0.9999986525	9.1975058734
0.0861524929	0.9999986525	12.2737434874
0	0	0
0.9592048558	0.9999986525	0
0.1942838943	0.9999986525	11.054718624
0.4973172685	0.9999986525	11.4259311282
0.0456202291	0.9999986525	20.9766331948
0.2863819254	0.9999986525	2.3851293062
0.6397064999	0.9999986525	0
0.4713262799	0.9999986525	7.5969387552
0.1322610391	0.9999986525	0
0.1899322775	0.9999986525	0
0.5013402711	0.9999986525	0.1903952119
0.8995657924	0.9999986525	0
0.273086892	0.9999986525	1.5961363801
0.739793467	0.9999986525	5.3478387187
0.7242297459	0.9999986525	9.4001227487
0.1757662562	0.9999986525	8.3837665126
0.3106695852	0.9999986525	0

0.2561562745	0.9999986525	0
0.493440265	0.9999986525	8.5656924504
0.5358667631	0.9999986525	3.8894536114
0.5996807121	0.9999986525	0
0.30755387	0.9999986525	0
0.1597306321	0.9999986525	8.5981144854
0.325406586	0.9999986525	0
0.9281527853	0.9999986525	0
0.6547951782	0.9999986525	31.1586538208
0.3556623687	0.9999986525	0
0.592847263	0.9999986525	1.4781145076
0.3826235796	0.9999986525	9.5790184504
0.4029945366	0.9999986525	9.8995160687
0.7583116531	0.9999986525	0
0.3665552566	0.9999986525	73.1060762476
0.1669998051	0.9999986525	44.9288968323
0.6312664098	0.9999986525	0
0.3367834063	0.9999986525	0
0.0276934687	0.9999986525	358.6130249935
0.0737447542	0.9999986525	39.8540920964
0.6312664098	0.9999986525	0
0.3743723527	0.9999986525	56.348506352
0.9074697241	0.9999986525	11.4493127377
0.5605873726	0.9999986525	0
0.2005620503	0.9999986525	12.5320737077
0.739618158	0.9999986525	28.8705821208
0.3302234128	0.9999986525	23.9110658033
0.1301505133	0.9999986525	5.9515346209
0.2442982102	0.9999986525	9.773115882
0.6823326083	0.9999986525	15.1052277275
0.9359106179	0.9999986525	0
0.3139335438	0.9999986525	6.598440558
0.2214197736	0.9999986525	161.181846062
0.3501041357	0.9999986525	7.7360360665
0.5107486803	0.9999986525	51.9438585948
0.6745404679	0.9999986525	4.052391526
0.9372608894	0.9999986525	4.0489051078
0.8436490951	0.9999986525	4.5069222954
0.6339125286	0.9999986525	17.9639177641
0.2152989418	0.9999986525	193.1947259533
0.8623905352	0.9999986525	1.9328765271
0.6312664098	0.9999986525	0
0.3420507072	0.9999986525	0
0.0526661864	0.9999986525	0
0.0415094285	0.9999986525	16.0078557934
0.5254024089	0.9999986525	8.0480023895
0.7169234279	0.9999986525	2.5304929436
0	0	0
0.5357625584	0.9999986525	0

0.3558315282	0.9999986525	39.6500887585
0.9285971793	0.9999986525	11.7753503473
0.3760848427	0.9999986525	7.6427822564
0.5357495767	0.9999986525	4.6497264803
0.4151866428	0.9999986525	90.4261892965
0.6416830087	0.9999986525	0
0.6421916577	0.9999986525	0
0.6155838911	0.9999986525	0
0.5928112764	0.9999986525	0
0.390335954	0.9999986525	6.9946033321
0.818755852	0.9999986525	107.2478433495
0.5641793685	0.9999986525	35.1998921796
0.442801036	0.9999986525	4.282151719
0.5271398503	0.9999986525	0
0.6665420151	0.9999986525	15.622851735
0.6999859506	0.9999986525	19.7605111783
0.6958726301	0.9999986525	0
0.3834770345	0.9999986525	0
0.1004881053	0.9999986525	2.2260445264
0.030829513	0.9999986525	35.6743668596
0.0716729021	0.9999986525	0
0.8818349347	0.9999986525	66.876685427
0.4955363401	0.9999986525	10.7657174186
0.1125519425	0.9999986525	65.249909098
0.6286029672	0.9999986525	0
0.6098761915	0.9999986525	0
0.2299239591	0.9999986525	0
0.6769317461	0.9999986525	14.0822094247
0.2858880917	0.9999986525	0
0.8356568867	0.9999986525	0
0.0878648143	0.9999986525	8.0233829436
0.8343800232	0.9999986525	0
0.011771458	0.9999986525	4.8587978773
0.4073955524	0.9999986525	0
0.336823401	0.9999986525	0
0	0	0
0.9500390055	0.9999986525	0
0.4729816619	0.9999986525	0
0.7116709906	0.9999986525	0
0.3810611079	0.9999986525	219.9384608374
0.4072754761	0.9999986525	3.56862855
0.3250505084	0.9999986525	0
0.6837244543	0.9999986525	0.1515785797
0.7269656123	0.9999986525	0.9259264403
0.6780636109	0.9999986525	0
0.6115964775	0.9999986525	14.2011073478
0.0654599187	0.9999986525	12.1585998794
0.7333254341	0.9999986525	4.8388765611
0.9930975242	0.9999986525	3.3239645176

0.4544042258	0.9999986525	33.4582947268
0.4518840872	0.9999986525	13.1469132325
0.2029191476	0.9999986525	4.1394273127
0.8340380864	0.9999986525	101.7684631945
0.9154110355	0.9999986525	1.9051265503
0.617355369	0.9999986525	0
0.2111596803	0.9999986525	5.0818912903
0.4999700131	0.9999986525	2.1127257471
0.1046372315	0.9999986525	5.1389153934
0.7803585972	0.9999986525	0
0.9559024345	0.9999986525	4.4579993438
0.5322073549	0.9999986525	0
0	0	0
0.1401403229	0.9999986525	8.5011583002
0.6793289897	0.9999986525	0
0.9364877517	0.9999986525	30.1085959201
0.491700142	0.9999986525	3.8665313415
0.6175997957	0.9999986525	0
0.9299591526	0.9999986525	0
0.568344124	0.9999986525	0.7754550667
0.1835143875	0.9999986525	0
0.7454571034	0.9999986525	4.0075819119
0.2627804206	0.9999986525	0
0.1820610837	0.9999986525	2.1050627058
0	0	0
0.4103779563	0.9999986525	12.8552049898
0.4167826258	0.9999986525	4.8849007146
0.1302007952	0.9999986525	22.5244324798
0.9081683546	0.9999986525	10.6279114869
0.4591241284	0.9999986525	0.3875539771
0.4870351814	0.9999986525	0
0.5768253012	0.9999986525	65.3291089564
0.2658932005	0.9999986525	0
0.4911975377	0.9999986525	0
0.8034755738	0.9999986525	1.6404385223
0.6680176829	0.9999986525	1.0515882808
0.8578081725	0.9999986525	8.3059408579
0.1007812073	0.9999986525	2.3323581623
0.1525822971	0.9999986525	0
0.7085253963	0.9999986525	0.275816621
0.2518623644	0.9999986525	7.8057815883
0.2370112181	0.9999986525	5.5939328418
0.8114276142	0.9999986525	0
0.9401584986	0.9999986525	3.3035540268
0.0335092083	0.9999986525	5.1606330102
0.4507498627	0.9999986525	1.540243367
0.8713699993	0.9999986525	62.9792329348
0.9402413663	0.9999986525	3.2231778725
0.883119105	0.9999986525	0.8639465775

0.4452391258	0.9999986525	0
0.2373267649	0.9999986525	119.7069141636
0.3803307162	0.9999986525	0.3049055979
0.003284	0.9999986525	66.7790968564
0.6828934338	0.9999986525	10.4169463957
0.1551721283	0.9999986525	1.4201489211
0.4932880138	0.9999986525	307.6079466586
0.5501062497	0.9999986525	9.508026565
0.3567262747	0.9999986525	1147.6346258299
0.9023511392	0.9999986525	12.5615826816
0.9926344783	0.9999986525	2.6763583868
0.6508871974	0.9999986525	73.5228559995
0.9430517405	0.9999986525	0
0.9861911039	0.9999986525	10.342691177
0.2673480246	0.9999986525	26.2935681007
0.7252620589	0.9999986525	0
0.3249336053	0.9999986525	0
0.0202667207	0.9999986525	2.9858615008
0.5868592284	0.9999986525	74.8249136618
0	0	0
0.6312664098	0.9999986525	0
0.2631973682	0.9999986525	0
0.562440646	0.9999986525	191.4356970687
0.1892578266	0.9999986525	193.0333268505
0.0885044215	0.9999986525	79.2406875113
0.7128243654	0.9999986525	102.0777974953
0.9298208943	0.9999986525	0
0.7125328266	0.9999986525	3.2105725366
0.4648704373	0.9999986525	0.3045451665
0.1859193043	0.9999986525	0
0.032222513	0.9999986525	219.85812159
0.7695804736	0.9999986525	0
0.367547977	0.9999986525	0
0.5586440061	0.9999986525	179.5984523679
0.0916676751	0.9999986525	0
0.6041285217	0.9999986525	41.0010578829
0.362533399	0.9999986525	0
0.7539880367	0.9999986525	12.1296283929
0.1342279442	0.9999986525	188.1172510287
0.3302168005	0.9999986525	9.6301077132
0.1895095998	0.9999986525	0
0.5717878512	0.9999986525	7.0896452239
0.1224964435	0.9999986525	2.5081406766
0.8537978587	0.9999986525	0
0.8144485154	0.9999986525	4.6621084207
0.7903750074	0.9999986525	2.3541698936
0.6176563209	0.9999986525	0
0.9430746306	0.9999986525	2.0498318737
0.6402763203	0.9999986525	0

0.2808699766	0.9999986525	3.2529899434
0.5747218245	0.9999986525	0
0.9856976962	0.9999986525	2.4386534712
0.8730716598	0.9999986525	4.9609166096
0	0	0
0.7818882525	0.9999986525	15.1546393763
0.623848747	0.9999986525	4.3053654705
0.2858524976	0.9999986525	10.2710848359
0.6037469751	0.9999986525	0
0.599198204	0.9999986525	1.7232981442
0.0525008676	0.9999986525	32.0743588721
0.2972125256	0.9999986525	0.7645732725
0.8782512169	0.9999986525	0
0.7632282998	0.9999986525	29.0452345883
0.9132867326	0.9999986525	9.4818524643
0.8706173979	0.9999986525	0.1694899576
0.4745335078	0.9999986525	1.8396613625
0.068766282	0.9999986525	0.1258052348
0.001266	0.9999986525	54.7397626015
0.8622359358	0.9999986525	0.6011076385
0.5711313291	0.9999986525	492.2735387273
0.287601432	0.9999986525	113.1669080917
0.4900516676	0.9999986525	15.6182317157
0.1738160143	0.9999986525	2.2913330828
0.4163345187	0.9999986525	9.8441599676
0.0941199656	0.9999986525	0
0.5008989401	0.9999986525	11.5841770879
0.3820454685	0.9999986525	0
0.4432791036	0.9999986525	2.6089380203
0.2521777785	0.9999986525	0
0.5556705964	0.9999986525	0
0.6962754943	0.9999986525	0
0.1267306016	0.9999986525	1.1693679378
0.7779015821	0.9999986525	37.9759536257
0.5803482044	0.9999986525	6.6567770601
0.3420700529	0.9999986525	0
0.9924472143	0.9999986525	1.4446083506
0.2139629809	0.9999986525	0
0.6933155805	0.9999986525	0
0.9947188185	0.9999986525	0
0.6858415324	0.9999986525	0.2721515861
0.3687639701	0.9999986525	35.6385250526
0.1409166936	0.9999986525	0
0.7445253282	0.9999986525	8.9420390648
0.0710601937	0.9999986525	7.4733151587
0.5658617507	0.9999986525	3.7748494194
0.3345458639	0.9999986525	0
0.5919770381	0.9999986525	2.8111948808
0.514651096	0.9999986525	1.9419658204

0.0433879111	0.9999986525	0.145554237
0.8712829565	0.9999986525	6.1857482111
0	0	0
0.4380106515	0.9999986525	19.362158688
0.0998921416	0.9999986525	0
0.4095133158	0.9999986525	10.3765713557
0.9542889748	0.9999986525	54.1237120581
0.847431982	0.9999986525	313.172185252
0.3990876431	0.9999986525	1.018983566
0.1046366913	0.9999986525	0
0.7932841877	0.9999986525	0.696750781
0.0513011803	0.9999986525	6.1643292487
0.1837408903	0.9999986525	1.0330249724
0.3543891552	0.9999986525	38.3560751496
0.7686938273	0.9999986525	1.1372383636
0.7207049893	0.9999986525	1.1955341866
0.5318981456	0.9999986525	0
0.0665371228	0.9999986525	3.1434361297
0.701872499	0.9999986525	0
0.6220080861	0.9999986525	0
0.7179273913	0.9999986525	9.6162264242
0.4729816619	0.9999986525	0
0.0739229291	0.9999986525	0
0.6402683596	0.9999986525	0
0.6396926222	0.9999986525	0
0.0932476764	0.9999986525	63.3500281755
0.7153552678	0.9999986525	4.6573120129
0.232478635	0.9999986525	5.6000337426
0.0515640592	0.9999986525	0
0.4514339034	0.9999986525	1.1301688007
0.3572973655	0.9999986525	55.1170223756
0.5544967195	0.9999986525	0
0.4068997721	0.9999986525	0
0.1185635055	0.9999986525	11.1574771789
0	0	0
0.4729816619	0.9999986525	0
0.4729816619	0.9999986525	0
0.2274646749	0.9999986525	3.3547734098
0.5718576373	0.9999986525	3.6849245725
0.9383787239	0.9999986525	73.1460942149
0.5041031226	0.9999986525	54.8512503834
0.2760898598	0.9999986525	1699.1573479209
0.7482891501	0.9999986525	0
0	0	0
0.5272524189	0.9999986525	4.1804660804
0.7299164391	0.9999986525	0
0.9837815677	0.9999986525	0.3311321247
0.2910664892	0.9999986525	555.7109971202
0.4617984186	0.9999986525	4.3700435238

0.4074269349	0.9999986525	0
0.9375364213	0.9999986525	38.3800624374
0.3610331654	0.9999986525	9.5881967168
0.555739782	0.9999986525	10.5719459985
0.6607591252	0.9999986525	21.9616798149
0.1424311344	0.9999986525	71.7808992861
0.7641717345	0.9999986525	0.5805322146
0.3730663846	0.9999986525	0
0.6402603911	0.9999986525	0
0.778169528	0.9999986525	238.6530421433
0.6073690266	0.9999986525	3.7537078158
0.1035480477	0.9999986525	19.3590150797
0.3092438634	0.9999986525	271.0391697831
0.3319426782	0.9999986525	3.986758459
0.623340901	0.9999986525	0
0.6603244793	0.9999986525	84.1740869747
0.2332149223	0.9999986525	0
0.4047091174	0.9999986525	4.49422782
0.8985697102	0.9999986525	2.2318120096
0.7074677131	0.9999986525	26.6455579732
0.8847506732	0.9999986525	0.4859893279
0.9672327112	0.9999986525	4.5812988733
0.1632811133	0.9999986525	18.3785819138
0.8680582842	0.9999986525	0.3750442459
0.0953370249	0.9999986525	8.0241956233
0.9586830788	0.9999986525	0.4536966379
0	0	0
0.1748141985	0.9999986525	0
0.0796905399	0.9999986525	2.3467637514
0.5815636575	0.9999986525	1.6384537515
0.935861932	0.9999986525	61.9154961621
0.3368634269	0.9999986525	0
0.4954020587	0.9999986525	19.7214033705
0.4882441653	0.9999986525	136.2658502149
0.7515047793	0.9999986525	12.2193155191
0.285588017	0.9999986525	0
0.0214397484	0.9999986525	5.9401550136
0.7144973367	0.9999986525	1.8980606869
0	0	0
0	0	0
0.8486854513	0.9999986525	1.1180624982
0.5609261256	0.9999986525	0
0.8781438649	0.9999986525	0
0.2889176024	0.9999986525	11.6773905757
0.1394324843	0.9999986525	0
0.2455250806	0.9999986525	119.2053227092
0.1863324762	0.9999986525	1.1386604479
0	0	0
0.1740801304	0.9999986525	0

0.6166995041	0.9999986525	4.6897170241
0.8004712694	0.9999986525	0.7581317011
0.9276921644	0.9999986525	203.3912126816
0.003937	0.9999986525	16.2437722656
0.0263642021	0.9999986525	3.6658511992
0.7360792634	0.9999986525	0
0.4528894085	0.9999986525	1.8050794889
0.5178565957	0.9999986525	0.4817469509
0.264277852	0.9999986525	0.3563632073
0.4690650058	0.9999986525	1.8245922775
0.8117658766	0.9999986525	4.7164548596
0.0462360421	0.9999986525	8.1569833643
0.1835283788	0.9999986525	21.6192058235
0.3395278632	0.9999986525	8.7873486659
0.2501854383	0.9999986525	20.7937908061
0.2715420473	0.9999986525	0
0.982017077	0.9999986525	4.2415707526
0.6098761915	0.9999986525	0
0	0	0
0.319141334	0.9999986525	40.487140992
0.9383286056	0.9999986525	30.7916967271
0.1632111141	0.9999986525	0
0.2247769315	0.9999986525	0
0.4406069417	0.9999986525	30.0352128221
0.8204902933	0.9999986525	13.7536245381
0.9645795307	0.9999986525	559.1049975098
0.1075453136	0.9999986525	0
0.2031184247	0.9999986525	0
0.7345862433	0.9999986525	0
0.4664629713	0.9999986525	161.7165140892
0.3584199631	0.9999986525	141.1581809366
0.544048751	0.9999986525	112.8502526203
0.00155	0.9999986525	61.6985026008
0.2321091095	0.9999986525	0
0.8839977518	0.9999986525	50.491317616
0.9244094697	0.9999986525	15.5209516498
0.3020736155	0.9999986525	5.0805410979
0.6603600781	0.9999986525	25.9485349949
0.3779807311	0.9999986525	90.0493064387
0.4029379392	0.9999986525	0
0.0854263045	0.9999986525	140.9112791013
0.1121465848	0.9999986525	91.2472671498
0.972951525	0.9999986525	0
0.4870129515	0.9999986525	0
0.30755387	0.9999986525	0
0.6876964891	0.9999986525	6.5612242187
0.5414986626	0.9999986525	0
0.6089129592	0.9999986525	30.9284624056
0.9922152531	0.9999986525	12.1579001442

0.4324843803	0.9999986525	10.056601376
0.6582421856	0.9999986525	118.4611249936
0.6411270632	0.9999986525	0.4843847113
0.7410162964	0.9999986525	1.1781273654
0.7708594424	0.9999986525	8.6037172316
0.9169923191	0.9999986525	0
0.2365552834	0.9999986525	22.6965835364
0.776003589	0.9999986525	0
0.4769044793	0.9999986525	10.3106873398
0.2500800109	0.9999986525	21.6862555709
0.9098775993	0.9999986525	0.7445606167
0.2346063186	0.9999986525	0.6131314661
0.3294364203	0.9999986525	0
0.3016110819	0.9999986525	30.2528530582
0.9079198662	0.9999986525	1.852924893
0.4295023236	0.9999986525	0.5361819109
0.1919613537	0.9999986525	0.2678652416
0.8542018053	0.9999986525	0.9254964598
0.9298432236	0.9999986525	0
0	0	0
0.7002747706	0.9999986525	1.4684299371
0.1791770317	0.9999986525	7.4934617234
0.4893720127	0.9999986525	1.6839919934
0.9589897771	0.9999986525	5.3740038218
0.5764377773	0.9999986525	0
0.1109544764	0.9999986525	1.7580612572
0.391273335	0.9999986525	2.4190227171
0.4529242107	0.9999986525	1.0944075226
0.4271736524	0.9999986525	0.3502396314
0.4062080027	0.9999986525	0
0.6648192233	0.9999986525	26.9081229022
0.6812204228	0.9999986525	0
0.6826057102	0.9999986525	9.1948237429
0.8030174764	0.9999986525	0.4471587353
0.7304505256	0.9999986525	3.2775684261
0.967851867	0.9999986525	1.0216841396
0.578256546	0.9999986525	17.8497763371
0.9412722154	0.9999986525	6.3899583031
0.1017980406	0.9999986525	43.4810264727
0.757715678	0.9999986525	3.5245699374
0.3198238669	0.9999986525	6.0255468598
0.3881085074	0.9999986525	0
0.8779852649	0.9999986525	27.5664739225
0.6312664098	0.9999986525	0
0.3047814966	0.9999986525	0
0.6049711272	0.9999986525	2.4964332735
0.9296678184	0.9999986525	0
0.7457045837	0.9999986525	0.7669975635
0.3612705579	0.9999986525	387.9441815003

0.8886969685	0.9999986525	13.9682407369
0.7683403102	0.9999986525	0.675758662
0.6481766509	0.9999986525	0
0.7620766958	0.9999986525	3.3144268155
0.4919424505	0.9999986525	0
0.321213027	0.9999986525	0.2808548832
0.7048108694	0.9999986525	0
0.0876000027	0.9999986525	2.4441269975
0.864146911	0.9999986525	0
0.3285455631	0.9999986525	5.8246719159
0.4648698926	0.9999986525	55.3488977429
0.3467677428	0.9999986525	0
0.3366665081	0.9999986525	0.6038659903
0.0736833116	0.9999986525	0
0.3846361408	0.9999986525	0
0.5804123148	0.9999986525	1142.9409176205
0.9431602288	0.9999986525	0.4338362061
0.0422594126	0.9999986525	0
0.4555638821	0.9999986525	5.7729593077
0.3204017756	0.9999986525	11.4333309119
0.9701879642	0.9999986525	5.9668653094
0.4226392316	0.9999986525	0.8958561279
0.3600786575	0.9999986525	2.3724223877
0.2486488642	0.9999986525	0
0.6833215979	0.9999986525	0.3936372651
0.0745295985	0.9999986525	0.5914080113
0.6726805141	0.9999986525	5.1571055782
0.0006092	0.9999986525	21.2354921913
0.4991563621	0.9999986525	0
0.4514739311	0.9999986525	8.1220943062
0.8889454948	0.9999986525	1.6548565143
0.4836077737	0.9999986525	7.9248614354
0.6047145229	0.9999986525	2.6373003871
0.7147986222	0.9999986525	0
0.9077560703	0.9999986525	238.1404796915
0.0130110768	0.9999986525	4.5779278149
0.4074667026	0.9999986525	0
0.8167265398	0.9999986525	59.7342406212
0.7783182187	0.9999986525	3.2049225212
0.7338676699	0.9999986525	4.0842240648
0.2625771148	0.9999986525	0
0.0104471159	0.9999986525	0
0.4729816619	0.9999986525	0
0.6465834634	0.9999986525	2.2564264815
0.9464515504	0.9999986525	0
0.5535716608	0.9999986525	0
0.4396775402	0.9999986525	3.9612419291
0.7936781248	0.9999986525	6.4302258691
0.1072265688	0.9999986525	0.1579066092

0.571627785	0.9999986525	2.9459722899
0.9786769726	0.9999986525	0
0.7539435317	0.9999986525	21.3621450763
0.9835985842	0.9999986525	0
0.8890129954	0.9999986525	17992.2890236935
0.3002008416	0.9999986525	7.8291556563
0.6069461537	0.9999986525	5.9968114257
0.3196045074	0.9999986525	0
0.6393618846	0.9999986525	12.741415858
0.0542108683	0.9999986525	2.6474026647
0.3075114913	0.9999986525	0
0.1276114079	0.9999986525	0.4120598595
0.2996721301	0.9999986525	7.1389454212
0.6859256077	0.9999986525	4.5009304778
0.4444232944	0.9999986525	15.9992130029
0.6799162358	0.9999986525	0
0.7058292614	0.9999986525	13.5777153355
0.3226688543	0.9999986525	7.268091634
0.1351543022	0.9999986525	1.5391361609
0.4162601179	0.9999986525	0
0.2809718268	0.9999986525	30.6323023135
0.827501959	0.9999986525	0.7458012882
0.2128166536	0.9999986525	21.7918756814
0.1555414922	0.9999986525	0
0.2518924647	0.9999986525	272.4884561688
0.6112767204	0.9999986525	1.9216863045
0.6062640353	0.9999986525	16.6168594315
0.9574867041	0.9999986525	0
0.8896056888	0.9999986525	0.4251906136
0.763929942	0.9999986525	0
0.7357261785	0.9999986525	0.2200362946
0.3067582631	0.9999986525	88.0752845777
0.0686776571	0.9999986525	7.3043966488
0.7304931084	0.9999986525	0.8346859409
0.2912615633	0.9999986525	6.1500028502
0.7204634501	0.9999986525	3.5365180321
0.5217078676	0.9999986525	46.2220565488
0.7355101883	0.9999986525	0
0.2747662473	0.9999986525	44.9759476702
0.0240211501	0.9999986525	60.623428254
0.6450887636	0.9999986525	1.2657619707
0.976009037	0.9999986525	3.4533697375
0.5265052212	0.9999986525	4.0309813942
0.002364	0.9999986525	22.8214549662
0.4499949908	0.9999986525	5.780014398
0.8853638964	0.9999986525	14.5999663367
0.4987791544	0.9999986525	43.0180021663
0.9494796008	0.9999986525	1.4719187934
0.759312863	0.9999986525	32.7386053969

0.7467754899	0.9999986525	0
0.3820994228	0.9999986525	0
0.9774898819	0.9999986525	3.1780332298
0.1252613693	0.9999986525	0
0.3688349367	0.9999986525	3.1303629331
0.3591003786	0.9999986525	10.0571599298
0.675198663	0.9999986525	3.4100812948
0.045900331	0.9999986525	0.5782345308
0.1416762428	0.9999986525	17.3357650914
0.3737338608	0.9999986525	32.7339673795
0.5130317271	0.9999986525	3.7852655959
0.9817435518	0.9999986525	0
0.049778994	0.9999986525	2684.4310234961
0.9561446739	0.9999986525	0
0.1977142212	0.9999986525	8.0529481412
0.9839518709	0.9999986525	25.163646229
0.9961040937	0.9999986525	429.831244227
0.5863549166	0.9999986525	60.4772497275
0.3308674799	0.9999986525	0
0.4028998823	0.9999986525	0
0.7338279081	0.9999986525	0
0.4047727846	0.9999986525	0
0.2755763759	0.9999986525	59.1156461006
0.26420985	0.9999986525	14.9149726705
0.0148998376	0.9999986525	2.5621655376
0.5177690125	0.9999986525	13.3442615156
0.3660899479	0.9999986525	3.3317090208
0.897669336	0.9999986525	1.356469611
0.9686345848	0.9999986525	0
0.6312664098	0.9999986525	0
0.8864512353	0.9999986525	1.2169199048
0.0502600666	0.9999986525	45.3029548072
0	0	0
0.5341368	0.9999986525	8.131473168
0.1927901652	0.9999986525	60.6707848665
0.3348484403	0.9999986525	215.6550356321
0.9911482746	0.9999986525	0
0.2573229324	0.9999986525	0.235606027
0.0662643275	0.9999986525	35.8790205342
0.6305852333	0.9999986525	5.8812826533
0.8822866751	0.9999986525	7.0818287525
0.5928480632	0.9999986525	0
0.7841214995	0.9999986525	11.5041519661
0.8457198223	0.9999986525	1.6747581905
0.7439252537	0.9999986525	29.5474286752
0.2188002815	0.9999986525	14.0196218272
0.5749127555	0.9999986525	72.581269073
0.2391349529	0.9999986525	1.2459443144
0.0550341526	0.9999986525	29.0242648816

0.9149876743	0.9999986525	0
0.8215212407	0.9999986525	0
0	0	0
0.6381789318	0.9999986525	17.9761464351
0.887704547	0.9999986525	63.5801583784
0.9614520608	0.9999986525	72.7798597515
0.3257672725	0.9999986525	307.7734162372
0.9339905179	0.9999986525	15.2362491277
0.290995031	0.9999986525	13.7381214497
0.2130469037	0.9999986525	0
0.7270574796	0.9999986525	0
0.6312664098	0.9999986525	0
0.0763050049	0.9999986525	162.5043637733
0.4109187201	0.9999986525	63.6005758987
0.2415250718	0.9999986525	0
0.5788620653	0.9999986525	99.0415719199
0.3274006339	0.9999986525	0
0	0	0
0.686700488	0.9999986525	11.3110533269
0.5927164383	0.9999986525	47.6444909574
0.7482936882	0.9999986525	0.3450604561
0.3356953908	0.9999986525	30.8132545078
0.3060469822	0.9999986525	1.0938635149
0.6959553527	0.9999986525	0
0.3569154611	0.9999986525	0
0.2862266804	0.9999986525	26.4510441868
0.303146489	0.9999986525	0
0.7044811495	0.9999986525	0
0.4125877146	0.9999986525	1426.0267216361
0.9823790916	0.9999986525	2.9883957577
0.1271661593	0.9999986525	0
0.4175030136	0.9999986525	143.8651338006
0.3331310407	0.9999986525	33.1537465325
0.6832852788	0.9999986525	1.4136866144
0.1991001882	0.9999986525	238.241536687
0.9452615649	0.9999986525	0
0.2275957366	0.9999986525	452.475263243
0.4867280906	0.9999986525	3.1408812504
0.7350523989	0.9999986525	406.8868892628
0.1563639472	0.9999986525	0
0.3236135809	0.9999986525	346.1788986567
0.8117411193	0.9999986525	0
0.1435852523	0.9999986525	4.548708587
0.8879494597	0.9999986525	30.9713638624
0.9385840532	0.9999986525	0
0.1818029411	0.9999986525	3.1760128827
0.9308394777	0.9999986525	1.9847892303
0.4658078916	0.9999986525	19.0844156366
0.6330117631	0.9999986525	18.3385198031

0.1651338486	0.9999986525	29.259788491
0.7671689308	0.9999986525	11.1571557613
0.7768406911	0.9999986525	10.5508260424
0.6595914375	0.9999986525	0.2426073334
0.9953925618	0.9999986525	2.5781751636
0.0697242568	0.9999986525	10.9741306422
0	0	0
0.3047928786	0.9999986525	0
0.2359621579	0.9999986525	0
0.5829227479	0.9999986525	1.5818707349
0.4772343571	0.9999986525	0
0.2093921927	0.9999986525	7.3868202701
0.2718504771	0.9999986525	1.4911393262
0.9206951349	0.9999986525	15.1462028045
0.595513197	0.9999986525	6.7056777839
0.2193419182	0.9999986525	0
0.1137345703	0.9999986525	44.6879238206
0.8700472958	0.9999986525	0
0.6375920911	0.9999986525	0
0.3420894015	0.9999986525	0
0.4366694034	0.9999986525	11.3075759834
0.3499316066	0.9999986525	0.7398306154
0.1643371827	0.9999986525	0
0.3755390941	0.9999986525	0
0.3528820973	0.9999986525	0
0.0399166461	0.9999986525	22.9326609754
0.6964981329	0.9999986525	204.7563112826
0.9120267575	0.9999986525	18.1065944436
0.3555668591	0.9999986525	98.6070168527
0.4418719607	0.9999986525	39.9785747119
0.9586851194	0.9999986525	10.2166931643
0.458420163	0.9999986525	23.2222002251
0.6694725155	0.9999986525	11.6780360921
0.7053778279	0.9999986525	0
0.8777245229	0.9999986525	12.3671509721
0.3762127543	0.9999986525	3.1073103487
0.8698421695	0.9999986525	22.4040942702
0.6004258064	0.9999986525	0
0.8310129869	0.9999986525	42.1037713812
0.5877241541	0.9999986525	61.2581135744
0.8997079313	0.9999986525	0.4770086275
0.826466209	0.9999986525	11.2012551912
0.5978030894	0.9999986525	0.6979689991
0.1105647143	0.9999986525	4.3297638504
0.371797558	0.9999986525	2001.8377989383
0.2298912173	0.9999986525	174.7519353076
0.3075673608	0.9999986525	0
0.9507566392	0.9999986525	1.7615869526
0	0	0

0.4512516202	0.9999986525	3.8865978518
0.4266236515	0.9999986525	28.715701515
0.0610679402	0.9999986525	21.2381293762
0.6122142588	0.9999986525	0.4714644417
0.7966433457	0.9999986525	2.5966274343
0.8522177168	0.9999986525	0
0.0689532768	0.9999986525	0
0.0001594	0.6597988509	0
0.6407724408	0.9999986525	8.0644248322
0.8768648504	0.9999986525	28.5805066434
0.2323279398	0.9999986525	0
0.1868028974	0.9999986525	12.2359643736
0.8025558853	0.9999986525	73.0552648969
0.6478078777	0.9999986525	2.356475509
0.5362452139	0.9999986525	0
0.6098761915	0.9999986525	0
0.6929768369	0.9999986525	6.1390117664
0.251353868	0.9999986525	7.2761477201
0.9298655101	0.9999986525	0
0.3607185031	0.9999986525	925.3397639422
0.5342418312	0.9999986525	0.2795984913
0.4154259415	0.9999986525	145.4094341438
0.3350332156	0.9999986525	8.5901897411
0.9627639712	0.9999986525	0
0.2074792601	0.9999986525	0.6785281647
0.9422684686	0.9999986525	8.5794331799
0.929646552	0.9999986525	0
0	0	0
0.6263790473	0.9999986525	6.1467282217
0.750962754	0.9999986525	0
0	0	0
0.3849240223	0.9999986525	0
0.8721989101	0.9999986525	4.7445463977
0.5044099171	0.9999986525	1.4816918508
0.6053820367	0.9999986525	8.5271761538
0.9875191267	0.9999986525	7.2337923882
0.4889054872	0.9999986525	4.6564907623
0.5930845808	0.9999986525	0
0.4127252809	0.9999986525	2.8237829375
0.2926310095	0.9999986525	0
0.9953972547	0.9999986525	8.3634974477
0	0	0
0.1511460907	0.9999986525	0
0.2143943011	0.9999986525	0
0.5789485442	0.9999986525	2.6043222111
0.3864321976	0.9999986525	11.0538509748
0.8002085379	0.9999986525	1.2699542881
0.0392719541	0.9999986525	2.2881245238
0.640252416	0.9999986525	0

0.0805498198	0.9999986525	0.1407627925
0.0111422342	0.9999986525	0.7391627581
0.116796008	0.9999986525	34.0442102877
0.384484247	0.9999986525	0
0	0	0
0	0	0
0.6280995674	0.9999986525	0
0.3034512409	0.9999986525	0
0.0655466818	0.9999986525	0
0.1518417986	0.9999986525	5.2278068029
0.725251546	0.9999986525	0.273623852
0.6354443696	0.9999986525	12.2223575799
0.478765912	0.9999986525	6.7107901177
0.007492	0.9999986525	1.866638329
0.3662454128	0.9999986525	5.7678705003
0.7128567224	0.9999986525	1.0438667884
0.1960615814	0.9999986525	4.0202419294
0.002239	0.9999986525	0.8192014616
0.1403656745	0.9999986525	0
0.9139697916	0.9999986525	13.5902798286
0.3815199535	0.9999986525	5.9949394962
0.3618159443	0.9999986525	0
0.8528888429	0.9999986525	88.1750540814
0.4425266535	0.9999986525	0
0.9111489541	0.9999986525	9.321528862
0.9949052332	0.9999986525	260.9015014708
0.6503012672	0.9999986525	0
0	0	0
0	0	0
0.6312664098	0.9999986525	0
0.9904500416	0.9999986525	18.2894787809
0.5099502919	0.9999986525	11.5236233611
0.2020935816	0.9999986525	0
0.2120950449	0.9999986525	0
0.1052392806	0.9999986525	25.5510005933
0.4721134215	0.9999986525	16.3197324721
0.0165720581	0.9999986525	3.9015212829
0.2897072739	0.9999986525	29.4963310908
0.862585264	0.9999986525	59.6801155165
0.6334523602	0.9999986525	7.7781911968
0.8214123454	0.9999986525	1.6055140008
0.1766796199	0.9999986525	4.887010107
0.2925313503	0.9999986525	0
0.0287478505	0.9999986525	2.4463912836
0.0256674561	0.9999986525	1.6335632986
0.3308141064	0.9999986525	1069.7915205756
0	0	0
0	0	0
0.8072961307	0.9999986525	71.5635748324

0.2442015783	0.9999986525	0.7033446887
0.7064531854	0.9999986525	0
0.7240095775	0.9999986525	88.9422671376
0.3079532715	0.9999986525	17.3977989106
0.2048042856	0.9999986525	0
0.70415309	0.9999986525	0
0.247655751	0.9999986525	2.8804418653
0.2199270684	0.9999986525	105.8077095589
0.2805344758	0.9999986525	68.6507995468
0.6883328714	0.9999986525	0
0.2833893365	0.9999986525	175.0050062726
0.6675323582	0.9999986525	1.7789052587
0.4757386327	0.9999986525	7.1588795817
0.5567143681	0.9999986525	4.7408198403
0.488063005	0.9999986525	0
0.2140827264	0.9999986525	13.7536245381
0.115190485	0.9999986525	12.1786464572
0.189518388	0.9999986525	0
0.3634854441	0.9999986525	37.576604928
0.9305654787	0.9999986525	3.9504024703
0.5902926637	0.9999986525	22.2093852928
0.3355852802	0.9999986525	5.4865902998
0.8602222333	0.9999986525	0
0.6493035308	0.9999986525	7.9029079842
0.2188114417	0.9999986525	5.5594662146
0.2180059989	0.9999986525	10.9370288883
0.6444393339	0.9999986525	0
0.5158940821	0.9999986525	0
0.3202203529	0.9999986525	121.7471605033
0.6169422637	0.9999986525	236.4259424732
0	0	0
0.9322177019	0.9999986525	133.4527308309
0.9278158468	0.9999986525	165.7051643844
0.8743914872	0.9999986525	19.0536219958
0.3146464501	0.9999986525	1.3156027128
0.9947401083	0.9999986525	11.5837778225
0.7254520306	0.9999986525	10.5613754619
0.7084239142	0.9999986525	43.2074596888
0.5187127862	0.9999986525	59.7130750515
0.4978796723	0.9999986525	0
0.205123654	0.9999986525	3.5276696504
0.4117685501	0.9999986525	5.8384074526
0.7031419873	0.9999986525	2.1908208152
0.9617266459	0.9999986525	0
0.4772918281	0.9999986525	1.3028892398
0.8182676445	0.9999986525	2.9977006461
0.483565296	0.9999986525	1.1524503459
0.6217087436	0.9999986525	2.1796687292
0	0	0

0.4056943048	0.9999986525	10.9408508237
0.9160131338	0.9999986525	0
0.5635830527	0.9999986525	61.6248367451
0.0455176629	0.9999986525	19.1054016486
0.948445741	0.9999986525	957.2262562688
0.2469458927	0.9999986525	0.0579004115
0.0410848007	0.9999986525	1.0098842807
0.3182065304	0.9999986525	5.1737772557
0.6402444342	0.9999986525	0
0.9822925771	0.9999986525	0.7573190832
0.9804176403	0.9999986525	0.4090867549
0.748826278	0.9999986525	4.2711800767
0.1390087064	0.9999986525	1.2238050359
0.4024148491	0.9999986525	3.4742951206
0.6595189924	0.9999986525	0.212996708
0.9920116389	0.9999986525	2.9399865412
0.351330502	0.9999986525	26.1391202609
0.6381940782	0.9999986525	0
0.0524785925	0.9999986525	40.6618376137
0.053636078	0.9999986525	0
0.1437867028	0.9999986525	35.2961616771
0.2321581877	0.9999986525	0
0.5125367423	0.9999986525	8.7670081438
0.4626077076	0.9999986525	0.2119729895
0.2465375659	0.9999986525	42.2050926618
0.8068242776	0.9999986525	0.5929306958
0.2714622506	0.9999986525	1.7844951421
0.9106463321	0.9999986525	0.5035251924
0.6413187541	0.9999986525	18.2898459566
0.0751489872	0.9999986525	2.4360101578
0.2981426416	0.9999986525	0
0.3421087532	0.9999986525	0
0.0254350892	0.9999986525	5.1515722031
0.529374271	0.9999986525	0
0.6312664098	0.9999986525	0
0.6312664098	0.9999986525	0
0.0619197912	0.9999986525	1.6866989725
0.6466041563	0.9999986525	0
0.2704113639	0.9999986525	0
0.6967999652	0.9999986525	1.8815913864
0.3934232485	0.9999986525	0
0.6940820034	0.9999986525	1.0354249069
0.1041417498	0.9999986525	0.5319586453
0.6754996766	0.9999986525	4.7048584662
0.3252098327	0.9999986525	1.7825677814
0.7351208838	0.9999986525	0
0.2236215253	0.9999986525	0.5208370469
0.6312664098	0.9999986525	0
0.9896541348	0.9999986525	2.382160078

0.0649190897	0.9999986525	2.6614016159
0.7192738394	0.9999986525	16.8484991877
0	0	0
0.4620965616	0.9999986525	0.7249480896
0.4632550118	0.9999986525	8.1834465584
0.800620942	0.9999986525	0.9995442338
0.0133449111	0.9999986525	5.3040359762
0.6312664098	0.9999986525	0
0.334517053	0.9999986525	0
0.3118284891	0.9999986525	1.0878252982
0.1897924239	0.9999986525	0.6562610215
0.7767837355	0.9999986525	0
0.8692981566	0.9999986525	2.6984793491
0.7985966442	0.9999986525	2.6499209486
0.1226100411	0.9999986525	0.6702188833
0.5813281068	0.9999986525	16.6349598728
0.8225212909	0.9999986525	7.2514218551
0.284552576	0.9999986525	0
0.0914600105	0.9999986525	0
0.4966435242	0.9999986525	3.189053694
0.4072217228	0.9999986525	3.4411692261
0.119664874	0.9999986525	17.5839705311
0.7173090477	0.9999986525	7.2796579223
0.2749198624	0.9999986525	0.9195744298
0.0434354648	0.9999986525	5.8548770275
0.1215348395	0.9999986525	119.5516144865
0.245406672	0.9999986525	52.112405081
0.3815865922	0.9999986525	0.6172999259
0.7631395035	0.9999986525	0
0.478284036	0.9999986525	0
0.9304791276	0.9999986525	1.1016210885
0.6741431531	0.9999986525	17.7420263823
0.5575983614	0.9999986525	1.3176467798
0.4169588609	0.9999986525	1.7743929253
0.6344424305	0.9999986525	0.9287759958
0.5736417582	0.9999986525	4.4150181035
0.6170071093	0.9999986525	58.9159247146
0.216280995	0.9999986525	4.7622151292
0.0339102582	0.9999986525	16.3798838807
0.899570655	0.9999986525	1.2798099747
0.0986431745	0.9999986525	390.2816364757
0.0460650664	0.9999986525	47.8242543552
0.6140687057	0.9999986525	40.7277820005
0.8894781004	0.9999986525	122.4705714811
0.5205009143	0.9999986525	0
0.7528323068	0.9999986525	1.0970046626
0.4257363109	0.9999986525	88.1642598596
0.3021965208	0.9999986525	8.9146442216
0.2604497209	0.9999986525	5.3955680374

0.9954052811	0.9999986525	6.6619751368
0.0299343025	0.9999986525	3.0756729596
0.4859671558	0.9999986525	0.8459566277
0.8987539146	0.9999986525	12.8473926568
0.6056937452	0.9999986525	0
0.2974469855	0.9999986525	3.3023490663
0.8007351851	0.9999986525	4681.7787637492
0.1354609348	0.9999986525	6.0137305673
0.9319019276	0.9999986525	0
0.0206870937	0.9999986525	6.1562755287
0.8201020831	0.9999986525	0
0.191402431	0.9999986525	389.0959713105
0.6298555339	0.9999986525	7.5818884014
0.6953717586	0.9999986525	1.3981738051
0.8529902776	0.9999986525	0
0.30755387	0.9999986525	0
0.6359590311	0.9999986525	0
0.9776710939	0.9999986525	0
0.2762077418	0.9999986525	31.9648468611
0.8716423226	0.9999986525	19.5625217988
0.8829491567	0.9999986525	3.2571025733
0.3772403883	0.9999986525	1.4705253578
0.7775782203	0.9999986525	0
0.7523416556	0.9999986525	0.3239481711
0.5853818646	0.9999986525	5.0714344369
0.5684803832	0.9999986525	9.5392822546
0.643368529	0.9999986525	14.1631971059
0.3312766572	0.9999986525	0.4385226861
0.4557424349	0.9999986525	0
0.8416280353	0.9999986525	0
0.2683091781	0.9999986525	9.0795202077
0.2701626553	0.9999986525	0
0.3138100846	0.9999986525	1.195020211
0.4875000033	0.9999986525	0
0.1246560888	0.9999986525	0.154824134
0.7041524778	0.9999986525	0
0.4266849286	0.9999986525	143.3923827037
0.9329737585	0.9999986525	0
0	0	0
0.4028619226	0.9999986525	0
0.3932646681	0.9999986525	21.6640742798
0.5352051999	0.9999986525	0
0.04058154	0.9999986525	11.1976928286
0.8803082107	0.9999986525	1.1899314669
0.2151884411	0.9999986525	3.1932957389
0.7094193785	0.9999986525	34.7784129241
0.8677985727	0.9999986525	1.6937490478
0.7862039431	0.9999986525	0.4890087056
0.3119892835	0.9999986525	5.9489562346

0.3863468213	0.9999986525	3.7461993351
0.0430931527	0.9999986525	0
0.2964169703	0.9999986525	1.7255052054
0.9455476117	0.9999986525	1.2028587293
0.5162485151	0.9999986525	17.4525528766
0.8040005502	0.9999986525	25.6973615167
0.6312664098	0.9999986525	0
0.857377728	0.9999986525	30.3208792802
0.8380716544	0.9999986525	0.6530152976
0.0406846295	0.9999986525	0
0.8108693696	0.9999986525	0
0.2731294157	0.9999986525	11.0744219388
0.1868642777	0.9999986525	0
0.3803395633	0.9999986525	1.1232582542
0.4639259143	0.9999986525	5.7504878707
0.506351628	0.9999986525	0.5634806912
0.8802082716	0.9999986525	1.2264515886
0.2035987904	0.9999986525	28.1706892209
0.7289584846	0.9999986525	14.4781609795
0.1995741636	0.9999986525	516.9136690034
0.487440191	0.9999986525	974.9324391358
0.1567294689	0.9999986525	27.7027555017
0.298845877	0.9999986525	0
0.8007927381	0.9999986525	136.867944869
0.7990979912	0.9999986525	0.5235139366
0.2722846561	0.9999986525	12.4136604128
0.21164553	0.9999986525	5.9062758025
0.4126276672	0.9999986525	29.9772495325
0.0899852328	0.9999986525	0.2994274858
0.0447868135	0.9999986525	0
0.1411059579	0.9999986525	5.7174936508
0.3927596983	0.9999986525	60.0840935453
0.9422033231	0.9999986525	13.5004903441
0.388353111	0.9999986525	2.8661159291
0.6145050447	0.9999986525	7.2318113414
0.3098139308	0.9999986525	2.8955528186
0.9493318581	0.9999986525	0.539170613
0.0104448203	0.9999986525	37.6919199446
0.3727733943	0.9999986525	13.0464561381
0.3385522482	0.9999986525	0
0.506169185	0.9999986525	9.7318760667
0.9036159699	0.9999986525	0
0.6104618156	0.9999986525	2.129879751
0.7268083006	0.9999986525	3.6194989018
0.3418933198	0.9999986525	0
0.2378852355	0.9999986525	2.262225573
0.9296252584	0.9999986525	0
0.149937576	0.9999986525	0
0.6767703994	0.9999986525	0

0.2025467388	0.9999986525	9.7743216952
0.6899427013	0.9999986525	19.4854491954
0.4383530836	0.9999986525	0.5517485006
0.9309974588	0.9999986525	0
0.365362511	0.9999986525	1.0687182449
0.2871194658	0.9999986525	4.4923801264
0.1671045143	0.9999986525	0
0.5747135679	0.9999986525	15.8012112221
0.5268268215	0.9999986525	5.4851250383
0.4017679844	0.9999986525	1.5190964015
0.4894667681	0.9999986525	0.4565360657
0.5071389258	0.9999986525	2.2847837263
0.0287895344	0.9999986525	12.6587995429
0.226230859	0.9999986525	2469.7156005109
0.9105249294	0.9999986525	1.3270495559
0.7506187165	0.9999986525	0
0.6189487284	0.9999986525	12.0023838577
0.1986526311	0.9999986525	0
0.4433654219	0.9999986525	0
0.1647516519	0.9999986525	11.6592163509
0.7508461819	0.9999986525	4.7329600741
0.505174295	0.9999986525	15.4562242395
0.0589227122	0.9999986525	0.2584002888
0.430647704	0.9999986525	53.045643934
0.4185496395	0.9999986525	1.7115146227
0.2701403448	0.9999986525	0
0.8559043163	0.9999986525	3.9726141056
0.8277471336	0.9999986525	0
0.0738227452	0.9999986525	4.5558695904
0.6384392345	0.9999986525	0
0.5044143924	0.9999986525	0
0.7530723097	0.9999986525	3.1411801436
0.3631855443	0.9999986525	0
0.5382547781	0.9999986525	27.6281221104
0.623820945	0.9999986525	0.6974159918
0.2020850842	0.9999986525	0.743512427
0.8501568443	0.9999986525	0
0	0	0
0.7207684542	0.9999986525	23.9575698419
0.4729816619	0.9999986525	0.4626063646
0.008531	0.9999986525	1.495297798
0.7880595897	0.9999986525	3598.7020281499
0.2473648789	0.9999986525	70.1757273405
0	0	0
0.4866915687	0.9999986525	0.2949427204
0.923503451	0.9999986525	8.8288061228
0.512496811	0.9999986525	82.2227152631
0.7476472381	0.9999986525	0
0.8864418026	0.9999986525	425.5684908879

0.5340427193	0.9999986525	11.0602169004
0.9041470937	0.9999986525	0
0.1839489771	0.9999986525	0
0.2431637491	0.9999986525	0
0.5124905588	0.9999986525	97.2376190384
0.5237281467	0.9999986525	0
0.474153324	0.9999986525	1.7881353352
0.8564435533	0.9999986525	11.3753637993
0.6740643902	0.9999986525	0
0.7967595684	0.9999986525	0
0.5989515587	0.9999986525	55.7697130162
0.3380400809	0.9999986525	0
0.1016579924	0.9999986525	1.7207714788
0.3271605724	0.9999986525	7.9249180618
0.0573496857	0.9999986525	0
0.0212216359	0.9999986525	0
0.5722926921	0.9999986525	0
0.3307151646	0.9999986525	0
0.4847010514	0.9999986525	0
0	0	0
0.1856588125	0.9999986525	84.1253874264
0.4851969382	0.9999986525	21.7460547185
0.236141343	0.9999986525	294.6383164648
0.6579582619	0.9999986525	0
0.0545228247	0.9999986525	30.738530348
0.6526757145	0.9999986525	15.2795944529
0.3098441517	0.9999986525	0
0.6854718342	0.9999986525	65.0818462431
0.2690045097	0.9999986525	668.5306104596
0.9798115605	0.9999986525	6.9647788773
0.2396154249	0.9999986525	0
0.2933242188	0.9999986525	9.4330782
0.6396787323	0.9999986525	0
0	0	0
0.6379896282	0.9999986525	0
0.015575885	0.9999986525	45.462054548
0.8999905877	0.9999986525	0
0.5831733149	0.9999986525	43.838072817
0.2658851386	0.9999986525	0
0	0	0
0.5809361901	0.9999986525	2.8393053183
0.5826903655	0.9999986525	0
0.4004284377	0.9999986525	16.1713974761
0.1956051656	0.9999986525	5.1283117179
0.3609594602	0.9999986525	1.2838699993
0.8054896004	0.9999986525	0.7539998319
0.0760775287	0.9999986525	4.312924603
0.8321280831	0.9999986525	9.0389765448
0.5003758129	0.9999986525	1.8330528331

0.7658269443	0.9999986525	0.1902518668
0.3307033456	0.9999986525	0
0.1057075396	0.9999986525	0.7827299141
0.3868027149	0.9999986525	0.2653339072
0.0938901856	0.9999986525	0
0.2163232473	0.9999986525	0
0.2974218863	0.9999986525	9.6947778669
0.3732732783	0.9999986525	1.7791649413
0.1549174167	0.9999986525	85.1150978667
0.6312664098	0.9999986525	0
0.30755387	0.9999986525	0
0.51815559	0.9999986525	1.6566681585
0.2249541274	0.9999986525	0
0.6085975006	0.9999986525	0.8763759164
0.8272302533	0.9999986525	0
0.0792444571	0.9999986525	0
0.3116021851	0.9999986525	1.6319858545
0.2176872221	0.9999986525	1.4638815407
0.4890391389	0.9999986525	10.809971664
0.5225915892	0.9999986525	6.8523396275
0.9550830263	0.9999986525	6.3760723601
0.1786179964	0.9999986525	36.5631923214
0.4074583044	0.9999986525	0
0.9379466272	0.9999986525	547.2664143693
0.6873410114	0.9999986525	3273.8944724701
0.9412897335	0.9999986525	2.159945943
0.6186389753	0.9999986525	5.7441813682
0.989610685	0.9999986525	0.4143503023
0.6312664098	0.9999986525	0
0.7530578338	0.9999986525	0
0.411444301	0.9999986525	33.5488757298
0.8621319865	0.9999986525	0
0.6047843933	0.9999986525	1.9408200337
0.651050696	0.9999986525	0
0.3682136659	0.9999986525	12.8520995839
0.9057033829	0.9999986525	0
0.341183732	0.9999986525	0
0.5257839836	0.9999986525	3.1438994794
0.3489498941	0.9999986525	0.5786494968
0.6312664098	0.9999986525	0
0.9319105682	0.9999986525	20.6927923197
0	0	0
0.8573536375	0.9999986525	315.8569741917
0.3075114913	0.9999986525	0
0.8956936127	0.9999986525	0
0.3075114913	0.9999986525	0
0.3583390589	0.9999986525	2.3354522962
0.4928527741	0.9999986525	27.5770123515
0.7938869082	0.9999986525	0

0.7390940963	0.9999986525	1.5328598072
0.5664485855	0.9999986525	3.1427502664
0.1403942771	0.9999986525	16.7959215014
0.9470965805	0.9999986525	0
0.9706968387	0.9999986525	10.4460662649
0.3984746622	0.9999986525	9.6050253984
0.6048370941	0.9999986525	0
0.196344736	0.9999986525	0
0.3421550737	0.9999986525	16.0317469067
0.4381563375	0.9999986525	23.7943405237
0.002533	0.9999986525	2643.5384257518
0.8602743846	0.9999986525	2.2930171812
0.0564368571	0.9999986525	1.9703745048
0.1001084326	0.9999986525	10.3516107852
0.1056095389	0.9999986525	0
0.2321844724	0.9999986525	0
0.3140836434	0.9999986525	5.4342494978
0.593073845	0.9999986525	41.2005212882
0.7221439096	0.9999986525	3.2620313286
0.8316954308	0.9999986525	0.5710909312
0.1328762934	0.9999986525	13.3334809963
0.3218074329	0.9999986525	41.3942731273
0.6879300813	0.9999986525	4.2612488483
0.8009792394	0.9999986525	4.0858671071
0.2332001744	0.9999986525	0
0.1217213949	0.9999986525	0
0.6635760155	0.9999986525	1.6927537893
0.0838851056	0.9999986525	0
0.1223637333	0.9999986525	6.1991804949
0.6399542429	0.9999986525	0
0.276647005	0.9999986525	4.1736464352
0.6801971683	0.9999986525	0
0.2450146115	0.9999986525	9.5182262965
0.3645567655	0.9999986525	0.3810692976
0.3474050342	0.9999986525	59.7601577374
0.7895214822	0.9999986525	3.9755297033
0.0124371952	0.9999986525	8.7425787496
0.1120268044	0.9999986525	75.2670073591
0.2751094723	0.9999986525	3.7311139687
0.1319160057	0.9999986525	4.6276580022
0.7328109259	0.9999986525	0
0.6312664098	0.9999986525	0
0.4729816619	0.9999986525	0
0.2213241584	0.9999986525	0
0.5725962674	0.9999986525	3.9829500925
0.0864367801	0.9999986525	15.2211241206
0.6098761915	0.9999986525	0
0.3199999237	0.9999986525	0.8546553904
0.6625636537	0.9999986525	3.0447318244

0.2911088539	0.9999986525	0.893906253
0.7679611904	0.9999986525	9.153192067
0.9268145983	0.9999986525	19.803419024
0.2074405066	0.9999986525	4.9065327226
0.9050342072	0.9999986525	0
0.4029074108	0.9999986525	0
0.6042459725	0.9999986525	0
0.9053690899	0.9999986525	3.4423654524
0.4172075807	0.9999986525	0
0.5514829093	0.9999986525	8.8206961547
0.0890530489	0.9999986525	25.601178433
0.1085918974	0.9999986525	0
0.5889230582	0.9999986525	14.9580599939
0.5747710117	0.9999986525	0
0.6855615347	0.9999986525	38.9124466578
0.3608824411	0.9999986525	16.5461456992
0	0	0
0.3197354476	0.9999986525	4.0476638739
0.1163375812	0.9999986525	159.0454042815
0.3450290083	0.9999986525	0
0.0568614886	0.9999986525	10.0037728201
0.0816344006	0.9999986525	154.0762606278
0.4075210043	0.9999986525	0
0.6225004472	0.9999986525	140.3505666391
0.6722196461	0.9999986525	0
0.0876881127	0.9999986525	3.1904022125
0.1599078476	0.9999986525	13.6282259314
0.862110046	0.9999986525	0
0.7310613716	0.9999986525	23.9227943952
0.0607002902	0.9999986525	11.2898499842
0.4256013894	0.9999986525	1.3980997797
0.4282139732	0.9999986525	13.5499716898
0.1920464287	0.9999986525	219.6385440013
0.6934740044	0.9999986525	114.0878924796
0.544483723	0.9999986525	0.6696749918
0.2724400104	0.9999986525	38.726979451
0.6507223095	0.9999986525	0
0.5983358728	0.9999986525	52.1361482984
0.8763292496	0.9999986525	0
0.4729816619	0.9999986525	0
0.8384331653	0.9999986525	2.5117390426
0.8419074595	0.9999986525	3.2514100714
0.3489167702	0.9999986525	3.2066349058
0.4866907299	0.9999986525	1.6897209568
0	0	0
0.9925989497	0.9999986525	1.153110848
0.3082608235	0.9999986525	1.9137904526
0.9541468754	0.9999986525	0
0.2927117016	0.9999986525	9.8902468589

0.7400301486	0.9999986525	1.965754205
0.6312664098	0.9999986525	0
0.380484263	0.9999986525	0
0.188059993	0.9999986525	1957.2497565038
0.8840718967	0.9999986525	0.2736784412
0.7586455575	0.9999986525	26.8915137477
0.5175057302	0.9999986525	2.0116522521
0	0	0
0.3821153699	0.9999986525	49.5323969297
0.2684475617	0.9999986525	14.090474955
0.697557198	0.9999986525	6.3012489681
0.4880330522	0.9999986525	80.664776845
0.8658807962	0.9999986525	4.8691007192
0.1635694897	0.9999986525	2.4560043818
0.887625862	0.9999986525	0
0.3406974235	0.9999986525	0
0.8554328194	0.9999986525	2.0773252921
0.7430335412	0.9999986525	50.2212463818
0.3954411234	0.9999986525	1.8152231733
0.3469230127	0.9999986525	2.262274024
0.4825633681	0.9999986525	2.4270544439
0.6875191774	0.9999986525	5.7027078809
0.7403952036	0.9999986525	17.5379950277
0.6853391589	0.9999986525	0.7374288698
0.116870352	0.9999986525	10.2678399429
0.9035144217	0.9999986525	2.0174154179
0.7103644028	0.9999986525	5.2106639161
0.6402364457	0.9999986525	0
0.0682480435	0.9999986525	46.6259840565
0.5896324016	0.9999986525	6.9366499066
0.9815844464	0.9999986525	0
0.5611157653	0.9999986525	0.1834351624
0.5722582572	0.9999986525	0.6313376315
0.7184781373	0.9999986525	0.1723967548
0.2195935511	0.9999986525	8.9603557833
0.2904313318	0.9999986525	15.2497138936
0.7909325438	0.9999986525	0
0.1066431599	0.9999986525	1.385307325
0.9209837385	0.9999986525	13.4186573113
0.3886154996	0.9999986525	0
0.347877458	0.9999986525	0.2723777948
0.0623700467	0.9999986525	1.8477603522
0.1652431071	0.9999986525	71.0303142152
0.3975462285	0.9999986525	2053.4063665532
0.2805761639	0.9999986525	1.4936485259
0.1236353934	0.9999986525	0
0.2699501675	0.9999986525	53.5765968402
0.3661127807	0.9999986525	6.685682233
0.2816028513	0.9999986525	0

0.8352233675	0.9999986525	0.2014837128
0.2049888825	0.9999986525	2.3640966082
0	0	0
0.1244728026	0.9999986525	2.5988863275
0.479115223	0.9999986525	17.7512515979
0.8397728092	0.9999986525	0
0.9405799556	0.9999986525	3.6152043781
0.3224218507	0.9999986525	11.0529308896
0.862124168	0.9999986525	0
0.1769762104	0.9999986525	6.7922409101
0.408808695	0.9999986525	0.8023565782
0.6065120229	0.9999986525	10.7516824351
0.7207208076	0.9999986525	119.8093515319
0.1195562579	0.9999986525	7.0569105059
0.8008474417	0.9999986525	0.5952138286
0.797837979	0.9999986525	0
0.6862765083	0.9999986525	16.2709466525
0.4473238863	0.9999986525	82.7755401583
0.0526475576	0.9999986525	0
0.8026012283	0.9999986525	18.257163973
0.726657638	0.9999986525	2.8533668542
0.0545259477	0.9999986525	11.136381537
0.2583941678	0.9999986525	0.6304144745
0.7848076987	0.9999986525	42.5975950205
0.3199443832	0.9999986525	3.6742034003
0.8543838607	0.9999986525	2.5515613675
0.6503976174	0.9999986525	7.6858130317
0.2910039747	0.9999986525	0
0.9199542703	0.9999986525	0.1437234657
0.8029214661	0.9999986525	2.5906531284
0.044176545	0.9999986525	13.7428879069
0.5767441608	0.9999986525	0
0.2295743333	0.9999986525	0
0.7377862736	0.9999986525	32.7066169433
0.9785656	0.9999986525	46.4808961288
0.9352198022	0.9999986525	5.2558135301
0.4918162454	0.9999986525	0
0.8607118864	0.9999986525	3.9753800814
0.2370501614	0.9999986525	2.7192831957
0.5777078448	0.9999986525	8.7501107105
0.4679333154	0.9999986525	0
0.6328691481	0.9999986525	0
0.6032233915	0.9999986525	0
0.3672245978	0.9999986525	1.1818895902
0.4607003034	0.9999986525	8.9781416449
0.7680320963	0.9999986525	0
0.6559132507	0.9999986525	8.2832368611
0.6312664098	0.9999986525	0
0.223055804	0.9999986525	2.8951824485

0.2695186919	0.9999986525	67.8793665172
0.6256429045	0.9999986525	2183.2880442035
0.4290520649	0.9999986525	0
0.2866949103	0.9999986525	115.5244292227
0.5638078667	0.9999986525	0
0.7500842004	0.9999986525	634.0580617605
0.4389505442	0.9999986525	4.0239175791
0.3381491972	0.9999986525	0
0.2120317452	0.9999986525	0
0.6187861001	0.9999986525	33.4933868453
0.2914354243	0.9999986525	0
0.7435754042	0.9999986525	1.9921417226
0.3372404885	0.9999986525	3.9774009283
0.4731492401	0.9999986525	56.1892890925
0.4733134029	0.9999986525	7.688095069
0.8039027841	0.9999986525	15.2015307552
0.6043899202	0.9999986525	0
0.0146070496	0.9999986525	36.6288502587
0.2248985095	0.9999986525	0
0.0612380154	0.9999986525	1.3464522996
0.7729579035	0.9999986525	15.7277898232
0.8411123684	0.9999986525	4.0857406723
0.3464940326	0.9999986525	0.3247379461
0.9893271704	0.9999986525	3.31714463
0.6312664098	0.9999986525	0
0.4158758995	0.9999986525	2.9616665205
0.1999993251	0.9999986525	2.7869406204
0.1742810695	0.9999986525	0
0.1864612853	0.9999986525	0
0.4497516829	0.9999986525	6.7551376968
0.700908611	0.9999986525	0
0.4931431269	0.9999986525	110.2160915219
0.9524663685	0.9999986525	4.0377172331
0.517727176	0.9999986525	28.0999830946
0.4403485792	0.9999986525	1.3200179512
0.4921534662	0.9999986525	31.2046784203
0.5442485017	0.9999986525	23.1926085253
0.2801521536	0.9999986525	0
0.9578706979	0.9999986525	0
0.4122659121	0.9999986525	795.4759065824
0.8199891111	0.9999986525	0.9885187025
0.9969512288	0.9999986525	1.2129558749
0.4593973331	0.9999986525	39.4800136247
0.3369034842	0.9999986525	0
0.5184192152	0.9999986525	2.2782541536
0.97746975	0.9999986525	0.6777445041
0.3130310307	0.9999986525	15.924594671
0.8227542088	0.9999986525	0.9429709725
0.246989225	0.9999986525	0

0.9000029518	0.9999986525	0
0.6339387232	0.9999986525	1.0727994765
0.7245398346	0.9999986525	0
0	0	0
0.4259135275	0.9999986525	0
0.7679182435	0.9999986525	7.4122485698
0.5341316916	0.9999986525	7.0366955201
0.99295094	0.9999986525	3.5777424316
0.1005804162	0.9999986525	4.8168543857
0.5769907651	0.9999986525	2.4765424243
0.0821162656	0.9999986525	6.5081846243
0.8384734968	0.9999986525	18.31446362
0.3242106037	0.9999986525	26.3017521047
0.1616312564	0.9999986525	2.9336176319
0.3139366892	0.9999986525	20.2065208387
0.4094767299	0.9999986525	24.5708467471
0.8412137595	0.9999986525	3.3203771046
0.1203897433	0.9999986525	20.1317308445
0.781674993	0.9999986525	0
0.3641892685	0.9999986525	4.6230670716
0.2494929326	0.9999986525	4.0969605326
0.1748192544	0.9999986525	0
0.7034794153	0.9999986525	0
0.5621564548	0.9999986525	0
0.1610937921	0.9999986525	36.4661452919
0.3742622288	0.9999986525	1521.1066225014
0.174867894	0.9999986525	2.7876025666
0.6374828126	0.9999986525	149.0515095757
0.0595790601	0.9999986525	62.7565530249
0.2460302368	0.9999986525	1.1815194234
0.5193727467	0.9999986525	1.691127705
0.2433158402	0.9999986525	84.1695518747
0	0	0
0.6863325541	0.9999986525	21.6206808827
0.2732873873	0.9999986525	1.9965190423
0.3344514563	0.9999986525	9.546984495
0.2709365199	0.9999986525	32.4720087469
0.5094757829	0.9999986525	28.1333220576
0.0956389819	0.9999986525	2.5107042013
0.7943973561	0.9999986525	0
0.2564871489	0.9999986525	27.7043124894
0.6213309744	0.9999986525	0
0.3477924387	0.9999986525	0
0.5044583564	0.9999986525	97.01955586
0.4710839257	0.9999986525	174.8226356382
0.5694011191	0.9999986525	204.4032957524
0.8336841471	0.9999986525	4.0835293292
0.5482396104	0.9999986525	86.1283728413
0.6155816848	0.9999986525	36.8669250009

0.8097510705	0.9999986525	110.6630030934
0.8872066994	0.9999986525	9.8386583879
0.4729816619	0.9999986525	0
0.5417415433	0.9999986525	0
0.1106967109	0.9999986525	18.6863727655
0.5481913378	0.9999986525	5.871941353
0.8964599972	0.9999986525	0
0.6268290412	0.9999986525	4509.0496541482
0.5581243912	0.9999986525	155.5638239361
0.1260420109	0.9999986525	24.731406334
0.0291434562	0.9999986525	0
0.7358710469	0.9999986525	22.6604340216
0.3649428668	0.9999986525	1.5785139048
0.2580361021	0.9999986525	187.7692874386
0.7246095901	0.9999986525	0
0.3025533294	0.9999986525	9.6678618958
0.9725896724	0.9999986525	35.5409947687
0.776247363	0.9999986525	0
0.6130727425	0.9999986525	4.2423543863
0.6312664098	0.9999986525	0
0.1334423998	0.9999986525	111.1287074725
0.3114750242	0.9999986525	0
0.965501452	0.9999986525	5.6606557584
0.4400830676	0.9999986525	0
0.6312664098	0.9999986525	0
0.0903210908	0.9999986525	12.4891028723
0.0361650911	0.9999986525	1.9245371479
0.0646428402	0.9999986525	31.628257247
0.1592772996	0.9999986525	0
0.2667328841	0.9999986525	88.9204855802
0.3421281079	0.9999986525	0
0.2287154669	0.9999986525	130.2974956241
0.9760573865	0.9999986525	0
0.280547	0.9999986525	87.4402619641
0.5975123224	0.9999986525	0
0.5058680394	0.9999986525	62.6128254017
0.5254178171	0.9999986525	111.0202485238
0.2962110604	0.9999986525	47.3624950465
0.8029003861	0.9999986525	0
0.8548712358	0.9999986525	44.3938791479
0.4815257734	0.9999986525	15.6485683634
0.5248231721	0.9999986525	122.0489706911
0.2379190473	0.9999986525	0
0.6179959085	0.9999986525	0
0.9113539391	0.9999986525	2.6540991118
0.9751104279	0.9999986525	19.6407282359
0.2627009244	0.9999986525	26.8047987579
0.8462197083	0.9999986525	431.5484786577
0.4762041896	0.9999986525	87.4797621577

0.8396087526	0.9999986525	5.7744320819
0.2882391242	0.9999986525	4.0632967292
0.5745681101	0.9999986525	3.1864561963
0.8478124718	0.9999986525	31.0086415138
0.7903854133	0.9999986525	24.0573572243
0.2045674417	0.9999986525	5.2745349272
0.08452164	0.9999986525	69.0265227594
0.630566946	0.9999986525	25.6585837537
0.148354904	0.9999986525	0
0.0178438652	0.9999986525	71.3822175723
0.7366171597	0.9999986525	2600.7717830667
0.8656545245	0.9999986525	42.0995761071
0.5660871394	0.9999986525	26.587086625
0.1294244094	0.9999986525	32.5108761011
0.0978944206	0.9999986525	0
0.7853055575	0.9999986525	70.0317157765
0.1400238533	0.9999986525	43.3344970062
0.7423968055	0.9999986525	47.9690447105
0.5025458882	0.9999986525	0
0.1975830717	0.9999986525	0.603670426
0.8161963326	0.9999986525	87.0367802016
0.8680368489	0.9999986525	36.1904419093
0.873982947	0.9999986525	8.9697551335
0.2956317759	0.9999986525	11.2474736007
0.4639019509	0.9999986525	0
0.922900878	0.9999986525	195.8204165425
0.7176793474	0.9999986525	4.8838467012
0.1909344452	0.9999986525	98.5104560062
0.3261700738	0.9999986525	18.2116959401
0.5307791649	0.9999986525	563.769408906
0.3923872746	0.9999986525	3.3525196449
0.4611449974	0.9999986525	5.963631236
0.4163819221	0.9999986525	0
0.8764278413	0.9999986525	17.365573349
0.2725434572	0.9999986525	1.0745675034
0.0514199524	0.9999986525	0
0.9880249568	0.9999986525	18.6970832744
0.1645062503	0.9999986525	11.7323292686
0.940120378	0.9999986525	80.7140879669
0.3544576774	0.9999986525	4.1129131864
0.2920077405	0.9999986525	0
0.6167852831	0.9999986525	7.9090378215
0.0190502035	0.9999986525	2.7641135828
0.080722219	0.9999986525	46.8485377525
0.5443356336	0.9999986525	0.832183542
0.4729816619	0.9999986525	0
0.734515319	0.9999986525	7.3451279347
0.5199958159	0.9999986525	0.8368417269
0.2435014321	0.9999986525	0.7504300796

0.4889185709	0.9999986525	3.313918443
0.1098042291	0.9999986525	0
0.597216584	0.9999986525	2.3095624019
0.5011328367	0.9999986525	10.6923752331
0.7735374203	0.9999986525	10.0112182124
0.5948500298	0.9999986525	58.6237322423
0.031923136	0.9999986525	0
0.4286731759	0.9999986525	0
0.2071773087	0.9999986525	4.8347864796
0	0	0
0.6312664098	0.9999986525	0
0.7536648131	0.9999986525	0.9227556255
0.6463823723	0.9999986525	12.8830145692
0.7426760496	0.9999986525	15.2903258858
0.1454666034	0.9999986525	152.7640250801
0.7945212519	0.9999986525	0
0.9776240297	0.9999986525	1.6469865664
0.6312664098	0.9999986525	0
0.1533092014	0.9999986525	0
0.6312664098	0.9999986525	0
0.0844539369	0.9999986525	0
0.7102440155	0.9999986525	1.435080878
0.7968557811	0.9999986525	0
0	0	0
0.109683566	0.9999986525	13.8035004675
0.3058711981	0.9999986525	1.1406401068
0.094292042	0.9999986525	35.3086661742
0.300464568	0.9999986525	0
0.4231571972	0.9999986525	0
0.6569545214	0.9999986525	0
0.9985266795	0.9999986525	0.645862736
0.4740001081	0.9999986525	0.4317402249
0.8493543873	0.9999986525	8.1831295671
0.1754184371	0.9999986525	1.4044285388
0.7235819191	0.9999986525	1.3629398252
0.1955853451	0.9999986525	1.6215012811
0.0624014975	0.9999986525	22.6545429921
0.1503417234	0.9999986525	24.9711197288
0.901272575	0.9999986525	0.7087993878
0.1875212784	0.9999986525	0
0.4022339735	0.9999986525	1.2589210035
0.5071689719	0.9999986525	2.7964956767
0.1992645381	0.9999986525	15.5885236795
0.7552063759	0.9999986525	6.5988985858
0.9975592913	0.9999986525	4.2592298895
0.4743018806	0.9999986525	0.8326790573
0.5104777699	0.9999986525	10.2043204456
0.6312664098	0.9999986525	0
0.4305836387	0.9999986525	0

0.5013448755	0.9999986525	0
0.2636687656	0.9999986525	2.9114511648
0.7442831809	0.9999986525	0
0.7855636976	0.9999986525	14.9371189214
0.6312664098	0.9999986525	0
0.7387217527	0.9999986525	0
0.8821895497	0.9999986525	4.3149947998
0.7479543767	0.9999986525	1.8542476673
0.2836540982	0.9999986525	19.1980800532
0.4729816619	0.9999986525	0
0.9889422913	0.9999986525	1.0452613103
0.0369854788	0.9999986525	0
0.1190952279	0.9999986525	9.2090528729
0.7104970854	0.9999986525	1.6562317557
0.2730303261	0.9999986525	0
0.5096498109	0.9999986525	1.3814061055
0.2006715808	0.9999986525	0
0.3635151487	0.9999986525	5.171919526
0	0	0
0.2739330327	0.9999986525	13.9093806232
0.2078163277	0.9999986525	12.0140851288
0.6065998814	0.9999986525	3.3211078904
0.7215997553	0.9999986525	0.4995641149
0.3421474656	0.9999986525	0.3339736068
0.3153100896	0.9999986525	2.4884675674
0.3025449122	0.9999986525	3.2785348703
0.0551899558	0.9999986525	20.1994237133
0.7752023115	0.9999986525	61.2496679483
0.0759070723	0.9999986525	0.9579192191
0.9720816252	0.9999986525	0
0.4083083226	0.9999986525	0
0.1834205627	0.9999986525	33.2715440898
0.6312664098	0.9999986525	0.5387526087
0.348168911	0.9999986525	0.5587207632
0.0181727	0.9999986525	80.1772777237
0.990788078	0.9999986525	954.0311165954
0.1964806418	0.9999986525	1.9062267461
0.08819886	0.9999986525	562.8481516125
0.0423567863	0.9999986525	0
0.4296214442	0.9999986525	3.4099161666
0.2513249291	0.9999986525	12.9216265769
0.4522868367	0.9999986525	3.1973555047
0.7226634084	0.9999986525	0.2512543624
0.5888474023	0.9999986525	0.2466879576
0.748397815	0.9999986525	1.2819178887
0.7461614398	0.9999986525	8.2671913008
0.2207505486	0.9999986525	0.8551614862
0.8698111861	0.9999986525	0.3123346673
0.006379	0.9999986525	2.4064847801

0.1898724759	0.9999986525	0
0.4075523348	0.9999986525	0
0.63966483	0.9999986525	0
0.3369435736	0.9999986525	0
0.6396509149	0.9999986525	0
0.6402284505	0.9999986525	0
0.5440709492	0.9999986525	2.3945157473
0.1204530446	0.9999986525	0
0	0	0
0.6880575644	0.9999986525	14.5763936318
0.4496410712	0.9999986525	0
0.336983693	0.9999986525	0
0.1043446725	0.9999986525	10.7574942919
0.7652522307	0.9999986525	6.9702940776
0.5205228879	0.9999986525	15.2026247053
0.1982604702	0.9999986525	18.3275598241
0.3248174816	0.9999986525	0
0.8857941866	0.9999986525	0.6674280868
0.3479065373	0.9999986525	1.4575387947
0.9362981606	0.9999986525	0
0.6898210256	0.9999986525	1.0940674544
0.4324287494	0.9999986525	28.318454813
0.8639959446	0.9999986525	8.9322432425
0.3040887331	0.9999986525	1.7141810525
0.1599035211	0.9999986525	0
0.8149253012	0.9999986525	6.6667404981
0	0	0
0.9098354548	0.9999986525	0
0.9299395264	0.9999986525	0
0.4444917421	0.9999986525	0
0.8434841888	0.9999986525	0.421876843
0.892743037	0.9999986525	9.1260406462
0.6492362319	0.9999986525	8.1974185288
0.7092335903	0.9999986525	30.7522523735
0.6717845605	0.9999986525	0
0.1532072713	0.9999986525	174.436213654
0.6312664098	0.9999986525	0
0.6904490321	0.9999986525	0.9669443133
0.3421668262	0.9999986525	0
0.46630262	0.9999986525	8.5837906995
0.916788802	0.9999986525	43.0712104276
0.9011794336	0.9999986525	0
0.8138811388	0.9999986525	4.7276006791
0.8193314785	0.9999986525	5.2259962623
0.2800420966	0.9999986525	0.1022417928
0.097315241	0.9999986525	2.7654403149
0.2414833665	0.9999986525	0
0.733331793	0.9999986525	0
0.0880271947	0.9999986525	14.2977004437

0.8384460798	0.9999986525	1.2181736415
0.0635869631	0.9999986525	5.6598758031
0.007123	0.9999986525	5.1428436156
0.946499737	0.9999986525	0
0.3053863099	0.9999986525	0
0.3353454842	0.9999986525	0
0.4875988974	0.9999986525	0
0.2468537739	0.9999986525	31.4256326469
0.8662139466	0.9999986525	15.6023392101
0.5502894667	0.9999986525	38.6855486449
0.88375122	0.9999986525	12.6258470539
0.2199712614	0.9999986525	1.1353070911
0.6563030618	0.9999986525	5.7457957654
0.9751499193	0.9999986525	14.5814241376
0.1164988471	0.9999986525	5.2692724959
0.4544341077	0.9999986525	0.9810695712
0.8574261761	0.9999986525	0
0.1733909888	0.9999986525	0.5553630805
0.7571935859	0.9999986525	2.925573645
0.30755387	0.9999986525	0
0.0419748884	0.9999986525	0.5645769806
0.6186133179	0.9999986525	0
0.9321171422	0.9999986525	1.4268247528
0.6724232991	0.9999986525	0
0.2657499032	0.9999986525	44.6912783806
0.8143018505	0.9999986525	0.7573842458
0.6551284047	0.9999986525	1.1704045081
0.2344229483	0.9999986525	0.4058550544
0.8040420391	0.9999986525	4.2339765824
0.7145514961	0.9999986525	8.614359173
0.3975477066	0.9999986525	0
0.072509454	0.9999986525	0
0.1353388074	0.9999986525	0.818046781
0.1041972454	0.9999986525	3.328818091
0.0603001158	0.9999986525	0
0.6007705189	0.9999986525	3.5784211821
0.7508280551	0.9999986525	0
0.4075836524	0.9999986525	0
0.419931398	0.9999986525	20.5283297503
0.1198134726	0.9999986525	1.4426484806
0.6072361761	0.9999986525	8.3372845022
0.178368634	0.9999986525	0
0.2690136485	0.9999986525	2.8721167157
0.5347680086	0.9999986525	0
0.8911597328	0.9999986525	0.824570056
0.6639461952	0.9999986525	1.9109859329
0.1112778812	0.9999986525	18.8838450613
0.2797391073	0.9999986525	100.8903753493
0.4739265164	0.9999986525	0

0.6113865004	0.9999986525	6.6025650989
0.5948410704	0.9999986525	6.2972575369
0.2940683231	0.9999986525	43.749556905
0.6245155327	0.9999986525	12.4194291653
0.5155937021	0.9999986525	0.7745736674
0.1921302228	0.9999986525	0
0.5908226309	0.9999986525	35.884824886
0.8924210752	0.9999986525	20.8484614543
0.3973174829	0.9999986525	14.1360673319
0.1469361871	0.9999986525	0.432029827
0.7168353078	0.9999986525	0
0.0821735053	0.9999986525	0
0.1039778705	0.9999986525	5.0188350518
0.0734699944	0.9999986525	10.9329418183
0.2704383531	0.9999986525	49.0440644707
0.7003317177	0.9999986525	3.7458007891
0.4027066871	0.9999986525	9.9694046788
0.1669154086	0.9999986525	1436.8750147835
0.8397106565	0.9999986525	0
0.5624614017	0.9999986525	45.7461396503
0.276985505	0.9999986525	2.6252071889
0.4729816619	0.9999986525	0
0	0	4.4114532431
0.9262827591	0.9999986525	7.1748292659
0.0793357692	0.9999986525	0.6445595234
0.730598634	0.9999986525	3.1892462697
0.4076149571	0.9999986525	0
0.7045488625	0.9999986525	0.7776015287
0.5179828437	0.9999986525	2.859735785
0.8280370806	0.9999986525	0
0.7819359687	0.9999986525	0
0.3297625125	0.9999986525	0
0.4159643785	0.9999986525	37.5245890965
0.4397957059	0.9999986525	0
0.7969796785	0.9999986525	0
0.278856124	0.9999986525	0.4346609898
0.5597662107	0.9999986525	0
0.087645632	0.9999986525	6.6960423751
0.4695648409	0.9999986525	2.523902331
0.4729816619	0.9999986525	0
0.3816922075	0.9999986525	218.0565077558
0.3165335081	0.9999986525	5.1295569373
0.8157777671	0.9999986525	35.2468754847
0.4179903467	0.9999986525	0
0.6198935583	0.9999986525	1.1283578649
0.5512847257	0.9999986525	38.0869602594
0.4489020364	0.9999986525	2.3661884713
0.4729816619	0.9999986525	0
0.5994635789	0.9999986525	0

0.4227970536	0.9999986525	0
0.8071510605	0.9999986525	7.6410604465
0.5781741608	0.9999986525	2.6457559195
0.1805918766	0.9999986525	0.1269022347
0.8371135758	0.9999986525	1.4322806917
0.7730666607	0.9999986525	0.5907398882
0.2895021255	0.9999986525	0
0.3685129153	0.9999986525	0
0.9567214113	0.9999986525	3.782690032
0.4282477163	0.9999986525	0
0.0597979199	0.9999986525	0.7507415632
0.848464512	0.9999986525	0
0.8282919605	0.9999986525	0
0.6534144009	0.9999986525	0
0.8621383263	0.9999986525	0
0.7235703246	0.9999986525	3.1878287569
0.5524894377	0.9999986525	0
0.8706719585	0.9999986525	21.6920394053
0	0	0
0.583892405	0.9999986525	0.8961308248
0.5989145388	0.9999986525	0
0.3628436093	0.9999986525	8.3119166236
0.0641687803	0.9999986525	0.837266257
0.7363465655	0.9999986525	0
0.3334212837	0.9999986525	1.2347682277
0.005492	0.9999986525	36.8683547828
0.390308787	0.9999986525	31.7435847733
0.8225618436	0.9999986525	1.0299276652
0.3067801289	0.9999986525	1.5515024229
0.6593769335	0.9999986525	1.4934373438
0.8722978992	0.9999986525	0.8150799158
0.4095118833	0.9999986525	0
0.1443580291	0.9999986525	0
0.0229516241	0.9999986525	2.9610853457
0.9192094596	0.9999986525	16.9498403145
0.1334340453	0.9999986525	62.2952562235
0.037220037	0.9999986525	0.1810835344
0.0572320378	0.9999986525	2.5673967345
0.270118021	0.9999986525	0
0.6707045418	0.9999986525	1.780404471
0.3354350609	0.9999986525	0
0.9367571956	0.9999986525	16.0209889275
0.0007786	0.9999986525	0
0.4284635889	0.9999986525	4.8438693258
0	0	0
0.226168082	0.9999986525	0
0.0309253425	0.9999986525	0.7633944499
0.8482551812	0.9999986525	30.1148500221
0.6402204484	0.9999986525	0

0.6854492929	0.9999986525	1.4135352682
0.0235571304	0.9999986525	0
0.7360355875	0.9999986525	495.3239692972
0.8209728892	0.9999986525	1.0185512271
0.452405527	0.9999986525	37.9800997296
0.3630270237	0.9999986525	8.0551999125
0.8927356408	0.9999986525	62.2049167523
0.1895447745	0.9999986525	0
0.7079984933	0.9999986525	25.9887760799
0.1944166885	0.9999986525	0.1504614308
0	0	0
0.3113178116	0.9999986525	0
0.1483358952	0.9999986525	0.8570163498
0.6098761915	0.9999986525	0
0.9800852517	0.9999986525	91.4151814066
0.4098531013	0.9999986525	9.5106661972
0.7232431763	0.9999986525	62.7765579773
0.095197367	0.9999986525	1.0815875123
0.30755387	0.9999986525	0
0.9761026242	0.9999986525	1.209220584
0.6314459079	0.9999986525	1.8896532336
0.4450314324	0.9999986525	4.9790899338
0.2353428903	0.9999986525	0
0.3558684967	0.9999986525	239.6337036086
0.4641863378	0.9999986525	4.8969789732
0.6859660336	0.9999986525	0.4748769802
0.0823414023	0.9999986525	14.8156022796
0.8450237622	0.9999986525	0
0.6652789507	0.9999986525	0
0.9499686477	0.9999986525	5.1768200575
0.2929390438	0.9999986525	0.1214716232
0	0	0
0.938875334	0.9999986525	9.8869553215
0.4095900097	0.9999986525	15.2015307552
0.5776223461	0.9999986525	0.1692428322
0.2250388788	0.9999986525	0
0.1126632057	0.9999986525	61.4591755931
0.2844607647	0.9999986525	0.370501787
0.2058901968	0.9999986525	185.1353032415
0.004552	0.9999986525	22.1040394362
0.3930318658	0.9999986525	3.7406803855
0.4559372552	0.9999986525	0.3266409271
0.7223940716	0.9999986525	13.0926015869
0.5817209682	0.9999986525	0
0.2306037001	0.9999986525	3.0587211328
0.8009379691	0.9999986525	0.5852930484
0.0553855258	0.9999986525	2.4211574587
0.9842608338	0.9999986525	2.3663658489
0.6098761915	0.9999986525	0

0.0807323966	0.9999986525	0
0.2011866943	0.9999986525	0
0.6312664098	0.9999986525	0
0.1770112877	0.9999986525	35.1178608939
0.0212823117	0.9999986525	6.5889736419
0.0830722241	0.9999986525	4.4852584481
0.6947822424	0.9999986525	0
0.1653944385	0.9999986525	2.6965137459
0.9542598521	0.9999986525	363.0834214741
0.9432368715	0.9999986525	0
0.3379817236	0.9999986525	0.9196567808
0.4018206955	0.9999986525	1.256929574
0	0	0
0.8716351978	0.9999986525	1.4626814916
0.1067879654	0.9999986525	2.9341065681
0.7045355922	0.9999986525	0
0.729265519	0.9999986525	2.6880047957
0.9499679505	0.9999986525	0
0.0001033	0.4631572217	0
0.431854887	0.9999986525	3.560428862
0.887314885	0.9999986525	0
0.7464010622	0.9999986525	2.8709457614
0.7333333469	0.9999986525	0
0.2074920061	0.9999986525	8.3658986577
0.0193910308	0.9999986525	1.117683919
0.3060975032	0.9999986525	15.2267315054
0.004374	0.9999986525	10.3451222726
0.7050032593	0.9999986525	13.0549791685
0.007869	0.9999986525	26.7900569272
0.1008314136	0.9999986525	0.3876051742
0.6634823787	0.9999986525	0
0.0495812278	0.9999986525	6.5130001512
0.5476103862	0.9999986525	6.0094348554
0.8277776401	0.9999986525	52.2392860794
0.1509395628	0.9999986525	0
0.3919764697	0.9999986525	2.1911306751
0.1268241014	0.9999986525	1.8779394889
0	0	0
0.0584182415	0.9999986525	0
0.578601822	0.9999986525	10.4659733914
0.3895137294	0.9999986525	0
0.8984782526	0.9999986525	4.2761709391
0.7927767378	0.9999986525	1.3798061002
0.9665248656	0.9999986525	0
0.9304800992	0.9999986525	10.8482740575
0.2256970871	0.9999986525	1.6154832905
0.5792961955	0.9999986525	82.1207669214
0.8984423846	0.9999986525	20.1976304406
0.2164490723	0.9999986525	131.3779060356

0.6936779789	0.9999986525	6.6533028756
0.3688101855	0.9999986525	51.3682683874
0.765311989	0.9999986525	3.6528358427
0.16955775	0.9999986525	70.4498686878
0.4316930762	0.9999986525	185.6469958743
0.176698876	0.9999986525	106.3163614602
0.3911909225	0.9999986525	0
0.5191149283	0.9999986525	115.0462929705
0.3421861899	0.9999986525	0
0.7507377807	0.9999986525	0
0.704532793	0.9999986525	125.5979030352
0.375403928	0.9999986525	0.6588617449
0	0	0
0.9702354136	0.9999986525	0
0.2768214732	0.9999986525	0.7685489927
0.9991011507	0.9999986525	16.7539129406
0.2652920118	0.9999986525	3.5308141614
0.7690187394	0.9999986525	0.582587842
0.5266543701	0.9999986525	69.465461624
0.7033768324	0.9999986525	22.7080672672
0.8870843936	0.9999986525	0.7368218732
0.5146637233	0.9999986525	8.8972234209
0.8156611794	0.9999986525	4.3153914472
0.5235221104	0.9999986525	34.9182930422
0.6312664098	0.9999986525	0
0	0	0
0.2010518249	0.9999986525	150.2767195817
0.194229574	0.9999986525	6.2858745806
0.8958861397	0.9999986525	4.9887987745
0.9316158321	0.9999986525	0
0.9328972871	0.9999986525	4.3380769827
0.853998785	0.9999986525	0.2148007444
0.6312664098	0.9999986525	0
0.6312664098	0.9999986525	0
0.6312664098	0.9999986525	0
0.3252043939	0.9999986525	1.1068464912
0.6312664098	0.9999986525	0
0.2608115176	0.9999986525	0
0.3365476807	0.9999986525	0.8704610452
0.1833732319	0.9999986525	0.2889812169
0.9096714452	0.9999986525	1.5137554427
0.0420852605	0.9999986525	106.8674994462
0.1230924507	0.9999986525	5.6362902308
0.8806643307	0.9999986525	0
0.5186227357	0.9999986525	9.9994796263
0.1396762839	0.9999986525	218.3087239879
0.9342980695	0.9999986525	0.7809205666
0.2652476463	0.9999986525	3.4490005432
0.0418827068	0.9999986525	7.2897057593

0.5534676122	0.9999986525	0
0.0663515347	0.9999986525	3.4710983367
0.5087542927	0.9999986525	0
0.3763936588	0.9999986525	59.4353359199
0.2789238807	0.9999986525	3.1215744563
0.9512557043	0.9999986525	0
0.445800429	0.9999986525	9.7529026123
0.006992	0.9999986525	7.101031022
0.3025997336	0.9999986525	5.9416586389
0.7795088692	0.9999986525	4.528718764
0	0	0
0.8914734043	0.9999986525	7.3582609859
0.6397230378	0.9999986525	13.0688701936
0.9392712365	0.9999986525	18.9390535573
0.6355637525	0.9999986525	17.5403248842
0.1100983411	0.9999986525	6.7039754032
0.6876490605	0.9999986525	7.3992190723
0.4625864655	0.9999986525	3.1597193034
0.9408620992	0.9999986525	14.684809739
0.5982237227	0.9999986525	2.2893891467
0.0967195774	0.9999986525	18.8103850932
0.4844436018	0.9999986525	0.8229254962
0.6697727713	0.9999986525	11.6365001364
0.8605012547	0.9999986525	1.9894995502
0.4716445927	0.9999986525	13.6219769598
0.788870377	0.9999986525	0.5362254622
0.5193006987	0.9999986525	1.4888693559
0.4030261235	0.9999986525	1434.1991547966
0.6162842046	0.9999986525	0
0.2798618676	0.9999986525	0
0	0	0
0.6402124395	0.9999986525	0
0.9693206949	0.9999986525	72.4796143036
0.7770706918	0.9999986525	0.8923078703
0.3315847795	0.9999986525	3.8908146623
0.4005836465	0.9999986525	0
0.236984625	0.9999986525	610.5655286279
0.0267731459	0.9999986525	13.4815362416
0.0830810005	0.9999986525	0
0.2591624927	0.9999986525	17.0001882703
0.4729816619	0.9999986525	0
0.3041133887	0.9999986525	12.0730598958
0.7383451545	0.9999986525	2.0104270356
0.471607397	0.9999986525	3.5984137239
0.9700242336	0.9999986525	3.6758016583
0.2343640409	0.9999986525	0
0.5560058303	0.9999986525	7.0058921836
0.620813172	0.9999986525	16.3351082996
0.4745374291	0.9999986525	46.1731349883

0.7239396665	0.9999986525	7.3501417071
0.9521182426	0.9999986525	0
0.516527052	0.9999986525	0
0.5318359699	0.9999986525	0.9579974102
0.0568935189	0.9999986525	21.1139546155
0.3831942481	0.9999986525	68.3865015879
0.9756907128	0.9999986525	71.0222467323
0.4729816619	0.9999986525	0
0.9988033578	0.9999986525	25.9533650736
0.913082918	0.9999986525	5.7172873381
0.6992617668	0.9999986525	17.4373698242
0.8680123087	0.9999986525	6.3166987473
0.8483577081	0.9999986525	5.5140862629
0.4852789058	0.9999986525	0
0.1545247053	0.9999986525	0
0.3435114756	0.9999986525	10.7724880799
0.1715764081	0.9999986525	1.0625545199
0.5076229825	0.9999986525	0
0.2096528404	0.9999986525	1.1286954656
0.4112940087	0.9999986525	0.3875170098
0.003046	0.9999986525	3.5886334325
0.6965107156	0.9999986525	8.7887703501
0.1990483812	0.9999986525	7.3399388351
0.206152538	0.9999986525	663.2445544699
0.7697763739	0.9999986525	61.9615011845
0.9414377398	0.9999986525	0
0.4334277537	0.9999986525	1.8700598365
0.5263431386	0.9999986525	0
0.5780944872	0.9999986525	1.0783045318
0.9101794364	0.9999986525	107.8170712208
0.5992315295	0.9999986525	0
0.1487517609	0.9999986525	0
0.2228800971	0.9999986525	18.8041900312
0.0231290255	0.9999986525	0
0.407646249	0.9999986525	0
0.9232032489	0.9999986525	0.8561804351
0.7536479445	0.9999986525	12.3628085736
0.2523220433	0.9999986525	0
0.2805171312	0.9999986525	0
0	0	0
0.1525907332	0.9999986525	0
0.6312664098	0.9999986525	0
0.6728785301	0.9999986525	0.4346413156
0.527133231	0.9999986525	8.9458256576
0.3317059432	0.9999986525	1253.2116189294
0.9168981113	0.9999986525	1268.041253933
0.0721317499	0.9999986525	484.753723968
0.9871280039	0.9999986525	204.9699284333
0.9319593894	0.9999986525	0.5206368059

0.6894635128	0.9999986525	4.6974174287
0.0872461572	0.9999986525	0
0.5873962289	0.9999986525	0
0.2856173862	0.9999986525	96.3757631867
0.0540394442	0.9999986525	0.6718949452
0	0	0
0.4915465875	0.9999986525	0
0.9338215657	0.9999986525	1.7893318277
0.1895579799	0.9999986525	0
0.3285693438	0.9999986525	2.1277917178
0.2360439607	0.9999986525	0
0	0	0
0.6312664098	0.9999986525	0
0.4503748411	0.9999986525	0.7849752267
0.4626177266	0.9999986525	18.5768266712
0.8986637475	0.9999986525	0
0.4076775281	0.9999986525	0
0.5556920494	0.9999986525	0.5533526979
0.4077087944	0.9999986525	0
0.0524268178	0.9999986525	3.5815759003
0.6239791298	0.9999986525	0.7800823926
0.717317951	0.9999986525	0
0.8144615874	0.9999986525	0
0.8430737802	0.9999986525	3.4222750983
0.8001443175	0.9999986525	0
0.2026594927	0.9999986525	1.8039388676
0.3580771924	0.9999986525	3.5219140177
0.2688605205	0.9999986525	52.8667850113
0.889908796	0.9999986525	0
0.5492270709	0.9999986525	7.9979886462
0.8437637684	0.9999986525	12.2623446079
0.2055209223	0.9999986525	1.7223427546
0.336203994	0.9999986525	105.6182347949
0.1102635265	0.9999986525	0
0.2133199066	0.9999986525	37.9470911733
0.3621145674	0.9999986525	4.6527987161
0.707110302	0.9999986525	1.7402671735
0.0980482412	0.9999986525	0
0.5412309668	0.9999986525	0
0.5623612163	0.9999986525	0.3877930129
0.251691686	0.9999986525	17.5744237329
0.2963045737	0.9999986525	0
0.0939109382	0.9999986525	1.3015406926
0.2279734612	0.9999986525	1.1689926343
0.8499939008	0.9999986525	0.8743591912
0.5473350299	0.9999986525	3.8388717728
0.062139791	0.9999986525	0.9092600991
0.43762035	0.9999986525	0
0.9371907325	0.9999986525	3.4996319731

0.7674747231	0.9999986525	0
0.986223577	0.9999986525	0.6413698165
0.6402044238	0.9999986525	0
0.3876191428	0.9999986525	0
0.407740048	0.9999986525	0
0.5290796118	0.9999986525	0
0.8034115639	0.9999986525	0.5525740048
0.3167668427	0.9999986525	11.6461684669
0.1366430046	0.9999986525	1.7030430075
0.845212751	0.9999986525	0
0.2341989814	0.9999986525	4.2432178543
0.6312664098	0.9999986525	0
0.9033670037	0.9999986525	0.6769973623
0.6209908391	0.9999986525	0
0.8998918472	0.9999986525	3.2234401701
0.5146519711	0.9999986525	0.5107383274
0.6453583151	0.9999986525	8.0291768806
0.9296039376	0.9999986525	1.1820218488
0.2522302334	0.9999986525	733.374615785
0.0306992015	0.9999986525	2.8345812702
0.4880570237	0.9999986525	0
0.7789139524	0.9999986525	0.5759298404
0.6815114273	0.9999986525	22.4344954648
0.526645818	0.9999986525	8.8537394992
0.8970023618	0.9999986525	0
0.7869085846	0.9999986525	0.982511408
0.4486262039	0.9999986525	0.7629974173
0.9301263448	0.9999986525	0.8696798549
0.0930674853	0.9999986525	0.6493173911
0	0	0
0.4673058806	0.9999986525	32.895620197
0.6467435598	0.9999986525	11.0674598127
0.3969401806	0.9999986525	1.6323599588
0.3418718889	0.9999986525	0.4246140345
0.4729816619	0.9999986525	0
0.2653121842	0.9999986525	0
0.5304715882	0.9999986525	0
0	0	0
0.0530639824	0.9999986525	0
0.8896978238	0.9999986525	0
0.344751813	0.9999986525	0
0.8837798298	0.9999986525	0.783182594
0.8128373253	0.9999986525	0.7661964054
0.5978086352	0.9999986525	0
0.1912104049	0.9999986525	1.2468022103
0.6745455413	0.9999986525	1.1199275821
0.6000498194	0.9999986525	72.0774838507
0.4871611084	0.9999986525	0
0.6004639995	0.9999986525	0

0.2417986326	0.9999986525	0
0.318900713	0.9999986525	0.6425962918
0.8599669833	0.9999986525	5.5894316091
0.8049247515	0.9999986525	0.2718709274
0.9818232926	0.9999986525	0.7731053404
0.4239878518	0.9999986525	0
0	0	0
0.3965994611	0.9999986525	9.7850665554
0.9481452127	0.9999986525	4.5937129883
0.9529032794	0.9999986525	0
0.1690559584	0.9999986525	3.0787508439
0.5333735815	0.9999986525	1.2492056915
0.4319569566	0.9999986525	2.1129370577
0.5247728369	0.9999986525	11.2436490606
0.3657134283	0.9999986525	59.7815623951
0.2814449443	0.9999986525	31.56924877
0.3554704444	0.9999986525	0
0.4794073011	0.9999986525	5.6642983941
0.8528228421	0.9999986525	11.0666867835
0.7530544483	0.9999986525	17.0941608409
0.0750602447	0.9999986525	1.9976266113
0.0365995987	0.9999986525	0
0.3693602974	0.9999986525	1.9545341468
0.4167454375	0.9999986525	0
0.0815697826	0.9999986525	0
0.1243482727	0.9999986525	3.0917515441
0	0	0
0.9945511043	0.9999986525	0
0.6747228694	0.9999986525	0
0.3400584376	0.9999986525	10.6372443558
0.3451220914	0.9999986525	0
0	0	0
0.4009346331	0.9999986525	203.8163752101
0.4711796353	0.9999986525	1.0115767863
0.4026805036	0.9999986525	36.4989068599
0.996225501	0.9999986525	0
0.002949	0.9999986525	1.4133839545
0.9285887586	0.9999986525	0
0.5504548264	0.9999986525	10.517907241
0.2248421472	0.9999986525	4.8485319983
0.0274826208	0.9999986525	131.5824807881
0.4729816619	0.9999986525	0
0	0	0
0.9398258172	0.9999986525	30.1449304945
0	0	0
0.6312664098	0.9999986525	0
0.5455442171	0.9999986525	0
0.2479042708	0.9999986525	0.5874087223
0.1149432075	0.9999986525	4.3985940057

0.9914455128	0.9999986525	0
0	0	0
0.6401964011	0.9999986525	0
0.1680315942	0.9999986525	5.813878494
0.6751501549	0.9999986525	2.3533516721
0.9207765693	0.9999986525	3.47929235
0.4644564078	0.9999986525	2.5727119968
0.7799232056	0.9999986525	0
0.2969132395	0.9999986525	65.4447561664
0.4729816619	0.9999986525	0
0.6295451873	0.9999986525	1.5599113396
0.5642301847	0.9999986525	3.8296625076
0.6920274438	0.9999986525	0
0.7296430576	0.9999986525	0
0.9047109196	0.9999986525	41.3821102655
0.0762202891	0.9999986525	0.9701129338
0.5574057876	0.9999986525	0
0.8197386407	0.9999986525	0.5839599101
0.9416704986	0.9999986525	62.2222952872
0.389097026	0.9999986525	8.2155896751
0.2166860532	0.9999986525	0
0.0685579249	0.9999986525	1.9770867453
0.9822470153	0.9999986525	5.8142056262
0.0700360895	0.9999986525	0.9761334684
0.5976749661	0.9999986525	0.7545492217
0.5325281103	0.9999986525	13.0803066688
0.3266061153	0.9999986525	3.4009266985
0.9535921607	0.9999986525	2.1512046573
0.7468351218	0.9999986525	2.5255316673
0.407771289	0.9999986525	0
0.8773486045	0.9999986525	125.121210322
0.8914649446	0.9999986525	599.6881865947
0.0346587507	0.9999986525	5.1616417344
0	0	0
0.1236384677	0.9999986525	0.3119159362
0.1203619048	0.9999986525	1.2871554349
0.6334530009	0.9999986525	0
0.0870748437	0.9999986525	2.9889369561
0.0177695255	0.9999986525	2.5931759618
0.2604393944	0.9999986525	4.2745269901
0.0185317633	0.9999986525	5.405428386
0.1556995537	0.9999986525	1.2370627088
0.8577403587	0.9999986525	0
0.3392251546	0.9999986525	118.5395264057
0.5487921102	0.9999986525	0
0.5417083513	0.9999986525	0.7518851716
0.1248326356	0.9999986525	0.7717158588
0.4426695906	0.9999986525	0.1779114996
0.0458532823	0.9999986525	0.1001135804

0.3370238446	0.9999986525	0
0.6290281078	0.9999986525	0
0.7743139697	0.9999986525	5.3825582872
0.3985799625	0.9999986525	2.6186122811
0.5287011691	0.9999986525	0
0.0937568526	0.9999986525	1.9281485972
0.1042349905	0.9999986525	2.6773080996
0	0	0
0.1589546151	0.9999986525	347.0123459858
0.8338056859	0.9999986525	0
0.8213767655	0.9999986525	1.9400476886
0.4091039983	0.9999986525	0.8185412453
0.947346974	0.9999986525	3.1965169302
0.7573768895	0.9999986525	56.1054372093
0.8300786706	0.9999986525	149.6144992247
0.7159173173	0.9999986525	0
0.8759488519	0.9999986525	0
0.5827379899	0.9999986525	50.660832831
0.7540747041	0.9999986525	0
0.1416226127	0.9999986525	0.5729643305
0.8647829937	0.9999986525	0
0.3597388735	0.9999986525	0
0.4729816619	0.9999986525	0
0.9293179079	0.9999986525	1.2204481706
0.5342591604	0.9999986525	0.1365734735
0.0567241169	0.9999986525	0
0.6545604761	0.9999986525	2.3153082747
0.5615816343	0.9999986525	0.5609699485
0.053148337	0.9999986525	10.1214868199
0.0287546044	0.9999986525	3.9311865055
0.9175641583	0.9999986525	32.6176576548
0.9239951149	0.9999986525	0
0.9313834372	0.9999986525	0.9521682843
0.4090986638	0.9999986525	6.5166164756
0.0843780722	0.9999986525	1.0462552393
0.5288486808	0.9999986525	6866.5076583347
0.4729816619	0.9999986525	0
0.5993979306	0.9999986525	1.4668631351
0.2174128014	0.9999986525	0
0.3216613238	0.9999986525	1.6773778259
0.8508358386	0.9999986525	3.1877612877
0.6843895102	0.9999986525	2.4468068579
0.8946305028	0.9999986525	65.2102438139
0.7558818131	0.9999986525	19.9465070934
0.9808580833	0.9999986525	0
0.4633745435	0.9999986525	0.8900372137
0.1426504439	0.9999986525	4.7473184923
0.5653206405	0.9999986525	3.9922403739
0.3353901781	0.9999986525	0

0.465027245	0.9999986525	0.3943426609
0.3995761129	0.9999986525	4.0241475304
0.9295825896	0.9999986525	0
0.9791552432	0.9999986525	4.0473536843
0.5778480876	0.9999986525	1.137854981
0.30755387	0.9999986525	0
0.5033477409	0.9999986525	0.1938922354
0.9917440899	0.9999986525	9.4265679842
0.6312664098	0.9999986525	0
0.6464189418	0.9999986525	3.9635954315
0.6234591257	0.9999986525	0.5778138379
0.9343730525	0.9999986525	0
0.7218727761	0.9999986525	13.9405881553
0.4078025173	0.9999986525	0
0.0590979714	0.9999986525	1149.1695715607
0.2535268951	0.9999986525	39.8295009248
0.6889322894	0.9999986525	1.4843014163
0.9557923879	0.9999986525	0.8218721956
0.2671495831	0.9999986525	10.6730720565
0.1983415344	0.9999986525	10.5112783812
0.8480070439	0.9999986525	51.5090229775
0.2630739786	0.9999986525	0
0.6401883714	0.9999986525	2.188361574
0.4106484708	0.9999986525	0
0.7463571633	0.9999986525	1.2089714599
0.3441254844	0.9999986525	3.8285751662
0.6380995433	0.9999986525	5.1385403995
0.3442901522	0.9999986525	1.3524777553
0.3934337293	0.9999986525	0.5407771441
0.5916401166	0.9999986525	12.8008915183
0.708606037	0.9999986525	0.4676156843
0.6445212261	0.9999986525	0
0.5660835127	0.9999986525	0
0.5017639653	0.9999986525	0
0.2117161571	0.9999986525	0.723428782
0.0946993004	0.9999986525	5.889809103
0.1002593351	0.9999986525	2.2791930946
0.1469254	0.9999986525	0.2508259794
0	0	0
0.2109677488	0.9999986525	0
0.3127990667	0.9999986525	13.1590677031
0.7355497083	0.9999986525	0
0.01075055	0.9999986525	42.6090506061
0.0567871762	0.9999986525	9.1071041223
0.5009241657	0.9999986525	20.1490118495
0.0396349319	0.9999986525	91.6663467212
0.660751586	0.9999986525	0
0.4729816619	0.9999986525	0
0.8664363829	0.9999986525	0.501969027

0.5545353365	0.9999986525	0
0.1694308529	0.9999986525	0
0.846066832	0.9999986525	5.2185947327
0.8332486335	0.9999986525	0.2664805741
0.1554496043	0.9999986525	0
0.2973767577	0.9999986525	0
0.6098761915	0.9999986525	0
0.6312664098	0.9999986525	0
0.6312664098	0.9999986525	0
0.2553963621	0.9999986525	3.0322039821
0.3001182767	0.9999986525	0
0.8651700082	0.9999986525	1.0961792907
0.0384185282	0.9999986525	2.8373223502
0.2724589569	0.9999986525	0.9763660573
0.707134123	0.9999986525	45.8243561398
0.5750283271	0.9999986525	5.6354597053
0.4105174559	0.9999986525	0.9672790217
0.3403728153	0.9999986525	5.5549743073
0.7158957402	0.9999986525	10.114379488
0.697574777	0.9999986525	0
0.5136676341	0.9999986525	2.2097503902
0.3143112668	0.9999986525	0
0.008559	0.9999986525	54.3396071976
0.7424113489	0.9999986525	5.3218973749
0.4925506896	0.9999986525	0
0.5904035797	0.9999986525	5.2088156578
0.7463404315	0.9999986525	52.3434222378
0.7062876975	0.9999986525	0.1206653999
0.5620421723	0.9999986525	2.1079866141
0.4990239626	0.9999986525	0
0.3658650425	0.9999986525	3.5654965891
0.8939814383	0.9999986525	0
0.4785897153	0.9999986525	10.9998441182
0.67842572	0.9999986525	0.4490019476
0.2743725125	0.9999986525	8.1339778571
0.6226451458	0.9999986525	0
0.4230688526	0.9999986525	14.3949626074
0.3075114913	0.9999986525	0
0.5636404013	0.9999986525	0
0.4231349984	0.9999986525	0
0.0708632788	0.9999986525	18.4306684588
0.5992214711	0.9999986525	3.4169021928
0.3369929686	0.9999986525	0
0.3968474474	0.9999986525	0
0	0	0
0.0913256639	0.9999986525	11.0189689602
0.3320885215	0.9999986525	0.3935932617
0.3457795072	0.9999986525	0
0.1168396065	0.9999986525	1.0865943468

0.349401901	0.9999986525	0
0.2700956838	0.9999986525	0
0.6401722909	0.9999986525	0
0.625888406	0.9999986525	0
0.2135261465	0.9999986525	2193.6489656991
0.0313250251	0.9999986525	0
0.6598145893	0.9999986525	1.1575526455
0.6066443031	0.9999986525	0.7374940871
0.1793333931	0.9999986525	0.9770946168
0.4077258632	0.9999986525	0
0.2720388534	0.9999986525	1.2005346023
0.0667901054	0.9999986525	0
0.1052023056	0.9999986525	3.748379553
0.7765259997	0.9999986525	1.541895162
0.4914278533	0.9999986525	5.964529238
0.2385277028	0.9999986525	0
0.3048042636	0.9999986525	0
0.4705685641	0.9999986525	13.6850212415
0.6396369867	0.9999986525	0
0	0	0
0.6312664098	0.9999986525	0
0.6207182181	0.9999986525	9.4795832643
0.3512447805	0.9999986525	1.7735289374
0	0	0
0	0	0
0.001422	0.9999986525	0
0.9298877536	0.9999986525	0
0.5942626249	0.9999986525	58.9441051633
0.4564298853	0.9999986525	0
0.5941475786	0.9999986525	14.8000331305
0.1054783199	0.9999986525	5.3172452477
0.6887134913	0.9999986525	0
0.6815169832	0.9999986525	0
0.6780593293	0.9999986525	0
0.2570043813	0.9999986525	2.3054289118
0.6352625631	0.9999986525	6.0894636488
0.2993081989	0.9999986525	0
0.1005454516	0.9999986525	0
0	0	0
0.1015107468	0.9999986525	1.4337582318
0.2622164696	0.9999986525	0
0.0552157428	0.9999986525	10.2227082185
0.4832812877	0.9999986525	0
0.8641127168	0.9999986525	0
0.8973785469	0.9999986525	0
0.906715496	0.9999986525	0
0.6624040143	0.9999986525	2.735669228
0.9539230583	0.9999986525	0.8959458654
0.4738730589	0.9999986525	0

0.3811153447	0.9999986525	3.0865176674
0.0242728729	0.9999986525	11.1237936377
0.3154750705	0.9999986525	1.2157620273
0.9859858413	0.9999986525	0
0.4235705412	0.9999986525	1.3610519145
0.004539	0.9999986525	344.1767235341
0.2736182781	0.9999986525	24.0391070671
0.3252834078	0.9999986525	0
0	0	0
0.3458178621	0.9999986525	0
0.5148208292	0.9999986525	2.084252579
0.64016424	0.9999986525	0
0.6312664098	0.9999986525	0
0.3466303828	0.9999986525	334.727035155
0.6426098434	0.9999986525	3.9782997421
0.5456838608	0.9999986525	54.0295838632
0.1623781174	0.9999986525	0.2270472642
0.1269726797	0.9999986525	0
0.4662248344	0.9999986525	13.4990114866
0.8855851795	0.9999986525	0
0.0813315402	0.9999986525	0
0.4729816619	0.9999986525	0
0.2331531389	0.9999986525	23.6092616121
0.3734322625	0.9999986525	50.1012911755
0.7661041877	0.9999986525	16.1799085707
0.7046882025	0.9999986525	1.9442614573
0.4978717813	0.9999986525	0.232455626
0.9441523358	0.9999986525	0
0.3244532946	0.9999986525	1.216195973
0.0722973272	0.9999986525	4.8497629225
0.1012900469	0.9999986525	1.1646617908
0.5756269024	0.9999986525	11.8629645612
0.5312008499	0.9999986525	6.6948163219
0.678566024	0.9999986525	2.0879192815
0.5374224049	0.9999986525	0
0.5328629419	0.9999986525	0
0.2737402004	0.9999986525	0
0.9772414636	0.9999986525	1.9970475016
0.7909035916	0.9999986525	0
0.6401561819	0.9999986525	0
0.5240339034	0.9999986525	14.4405536528
0.9799955734	0.9999986525	0
0.6312664098	0.9999986525	0
0.6312664098	0.9999986525	0
0.3369622054	0.9999986525	0
0.4249670767	0.9999986525	58.9809682685
0.5915898874	0.9999986525	0
0.9248561608	0.9999986525	17.0023774176
0.8543436762	0.9999986525	24.3640347955

0.7498950781	0.9999986525	3.1503425826
0.3712488152	0.9999986525	16.5301778486
0.6318992309	0.9999986525	3.0208727465
0.6504562201	0.9999986525	0
0.7409728805	0.9999986525	0
0.8056218116	0.9999986525	0
0.3251115015	0.9999986525	18.155351745
0.6973233015	0.9999986525	7.9734166033
0.7689641145	0.9999986525	0
0.7293518013	0.9999986525	0
0.5232014694	0.9999986525	96.6332166471
0.8596159084	0.9999986525	10.1345969252
0.4890661022	0.9999986525	103.9644059573
0.9894901888	0.9999986525	158.4057533713
0.6373882077	0.9999986525	0
0.7217265067	0.9999986525	2.3451312081
0.6897991985	0.9999986525	24.0026896953
0.7938186689	0.9999986525	18.5735601288
0.412287192	0.9999986525	1.457297487
0.8325487663	0.9999986525	2.4509324652
0.3081697637	0.9999986525	0
0.0964214305	0.9999986525	0
0.0974497003	0.9999986525	3.2987051139
0.6576899294	0.9999986525	0
0.4571560206	0.9999986525	4.3284775008
0.3412354089	0.9999986525	201.7509609664
0.9886445251	0.9999986525	3.6528358427
0.2210890015	0.9999986525	0
0.6312664098	0.9999986525	0
0.2790016521	0.9999986525	2.2595639603
0.9876299413	0.9999986525	18.6676209512
0.6312664098	0.9999986525	0
0.3702883525	0.9999986525	0
0.8065259641	0.9999986525	0
0.7068233016	0.9999986525	4.7359097389
0.7089298194	0.9999986525	1.5814539022
0.5388924942	0.9999986525	0
0.279244827	0.9999986525	11.1054709945
0.1381259339	0.9999986525	2.93367874
0.2431680421	0.9999986525	0
0.9887906194	0.9999986525	7.0391574198
0.3021174273	0.9999986525	0
0.6932649208	0.9999986525	0.1914532775
0.30755387	0.9999986525	0
0.6401481166	0.9999986525	0
0.211122979	0.9999986525	0.8021433184
0.1838182513	0.9999986525	4.5979121775
0.7871558702	0.9999986525	0.1120018624
0.9085080089	0.9999986525	18.0982605948

0.28523072	0.9999986525	0
0.1119448789	0.9999986525	0.5842958571
0.1409195819	0.9999986525	33.6012868027
0.4076493308	0.9999986525	0
0.052324907	0.9999986525	30.8447471025
0.2630703198	0.9999986525	0
0.9319618931	0.9999986525	0
0.4515338104	0.9999986525	17.4963288019
0.160773871	0.9999986525	2.0785516245
0.34926081	0.9999986525	0
0.9338071492	0.9999986525	0
0.831250352	0.9999986525	30.4227639552
0.2925851165	0.9999986525	37.0884257207
0.6660053686	0.9999986525	5.3845965906
0.4729816619	0.9999986525	0
0.6587071082	0.9999986525	0
0.640140044	0.9999986525	0
0.744808918	0.9999986525	0
0.5198564268	0.9999986525	0.9896918939
0.6428190642	0.9999986525	12.9433188477
0.0634247586	0.9999986525	4.2513870299
0.2416030038	0.9999986525	0
0.55458796	0.9999986525	0
0.6153149235	0.9999986525	2.2347884853
0.4502433516	0.9999986525	0.610219855
0.2880286059	0.9999986525	1.9849135547
0.3811601262	0.9999986525	0.7072162337
0.6312664098	0.9999986525	0
0.1502765297	0.9999986525	0.7738189657
0.1597437977	0.9999986525	0.312006228
0.6401319642	0.9999986525	0
0.8656253785	0.9999986525	0
0.6955474227	0.9999986525	0.8657593598
0.1307874024	0.9999986525	1.163558454
0.958072439	0.9999986525	4.0817619775
0.3801616339	0.9999986525	1240.9536116844
0.9305149036	0.9999986525	0
0.3048156515	0.9999986525	0
0.6401238769	0.9999986525	0
0.1029543884	0.9999986525	0.3755656407
0.7986427631	0.9999986525	6.0971967474
0.4878856944	0.9999986525	0
0.8899148224	0.9999986525	0
0	0	0
0.3003406441	0.9999986525	667.4744799534
0.9655097321	0.9999986525	4.7599832567
0.8862785342	0.9999986525	0
0.8334650404	0.9999986525	22.307456917
0.2906376853	0.9999986525	1.3069840438

0.3158217601	0.9999986525	1.8380927236
0.3793723739	0.9999986525	5.0859228543
0.1354399289	0.9999986525	3.0767480252
0.003893	0.9999986525	40.0712581383
0.4954747799	0.9999986525	0
0.6057173799	0.9999986525	3.3633725626
0.514390578	0.9999986525	0
0.7457583584	0.9999986525	10.794505701
0.3684906551	0.9999986525	0
0	0	0
0.2575038881	0.9999986525	3.7472625391
0.6075927142	0.9999986525	7.7701807012
0.5557338238	0.9999986525	3.4425524379
0.8642701251	0.9999986525	0.3713632393
0.5242656507	0.9999986525	1.1798828968
0	0	0
0.5920268579	0.9999986525	0
0.2061282397	0.9999986525	2.053059077
0.8865660454	0.9999986525	3.5856488434
0.2046122862	0.9999986525	0
0.6500419882	0.9999986525	0
0.2105508754	0.9999986525	0
0.8173506822	0.9999986525	0
0.0447800905	0.9999986525	0
0.6892875342	0.9999986525	0.6513803432
0.5755448684	0.9999986525	2.6889627935
0.7172380133	0.9999986525	0
0.4017388179	0.9999986525	5.8130537154
0.3355477794	0.9999986525	0
0.4030840826	0.9999986525	21.4516320984
0.2357890584	0.9999986525	0
0.9820725853	0.9999986525	0.1390699076
0.1467412076	0.9999986525	4.091921416
0.9792455376	0.9999986525	0
0.4950828964	0.9999986525	0
0.615886604	0.9999986525	2.0321936319
0.3255128664	0.9999986525	55.3953411226
0.9932313978	0.9999986525	0.7407281659
0.5814327319	0.9999986525	1.9555157755
0.7968883856	0.9999986525	17.1284679984
0.7819041965	0.9999986525	0.4893711961
0.3618603554	0.9999986525	0
0	0	0
0.2414074257	0.9999986525	0
0.378837473	0.9999986525	7.2915748409
0.0710388961	0.9999986525	1.9024729795
0.5667713348	0.9999986525	0
0.0435838066	0.9999986525	1.4220991498
0.6807989836	0.9999986525	6.516482466

0.0388134419	0.9999986525	1.522848771
0.2834528827	0.9999986525	2.5510273017
0.9396799582	0.9999986525	0.9282862556
0.8558603359	0.9999986525	3.9454593027
0.8009121168	0.9999986525	2.051411005
0.1819348279	0.9999986525	12.0643991989
0.3248409387	0.9999986525	0
0.5192418976	0.9999986525	8.5891253432
0.1046613379	0.9999986525	4.2679063323
0.1909808473	0.9999986525	56.4050390455
0.4050808925	0.9999986525	5.0758210693
0.1348522624	0.9999986525	55.3114425958
0.7156842751	0.9999986525	2.4774330719
0.2412226728	0.9999986525	2.6414443205
0.001954	0.9999986525	34.6548019858
0.3906523031	0.9999986525	80.1577207001
0.5987285854	0.9999986525	5.8030895755
0.239153929	0.9999986525	0
0.1235908805	0.9999986525	11.2822701289
0.2748229099	0.9999986525	2.9574374637
0.0561749712	0.9999986525	1.8813065303
0.0002033	0.7627450395	0
0.9496559219	0.9999986525	0
0.1490345368	0.9999986525	0
0.327120852	0.9999986525	1.8651616834
0.4918897418	0.9999986525	0
0.8359325125	0.9999986525	0
0.0319540623	0.9999986525	0
0.8017304308	0.9999986525	1.291567526
0.2985612705	0.9999986525	2.9579757894
0.3357046437	0.9999986525	0
0.9476539081	0.9999986525	7.8553954268
0.1278134927	0.9999986525	1.354654583
0.9357387693	0.9999986525	10.2605824894
0.8219382485	0.9999986525	0
0.6404773781	0.9999986525	0
0.2737550012	0.9999986525	322.796555349
0.7415342472	0.9999986525	0
0.561803081	0.9999986525	5.9889911239
0.5297864684	0.9999986525	0
0.2735609655	0.9999986525	0
0.913801376	0.9999986525	15.5973474446
0.9397011518	0.9999986525	4.575803
0.8202824153	0.9999986525	142.5663066018
0.274951909	0.9999986525	0
0.8477026452	0.9999986525	0
0.43047794	0.9999986525	22.5455254769
0.1746262855	0.9999986525	1.4722060051
0.8393330229	0.9999986525	2.3280144681

0.2658409545	0.9999986525	0
0.1533625861	0.9999986525	0
0.8030652525	0.9999986525	0.1042770318
0.6937227417	0.9999986525	0.346459359
0.516481838	0.9999986525	0
0.4075729196	0.9999986525	0
0.146379629	0.9999986525	7.5773196881
0.5459381306	0.9999986525	5.0303760574
0.9273355307	0.9999986525	0
0.1201695966	0.9999986525	1.1113338431
0	0	0
0.7608596999	0.9999986525	69.4324515083
0.7610625239	0.9999986525	1.264225379
0.7579898833	0.9999986525	7.0279755119
0.2828193076	0.9999986525	0
0.3019191213	0.9999986525	6.3761123482
0.3782144051	0.9999986525	0.7504908626
0.3309845876	0.9999986525	0
0.4512325928	0.9999986525	0
0.4246361708	0.9999986525	0
0.1079120122	0.9999986525	0
0.8345210796	0.9999986525	0.7291920007
0.9062934251	0.9999986525	2.1928137109
0.8990252742	0.9999986525	0.9675891436
0.5327267496	0.9999986525	101.0142405914
0.0165924724	0.9999986525	2497.9555917849
0.3586897792	0.9999986525	0.2377206359
0.1114133531	0.9999986525	0
0	0	1.5965996018
0.161201617	0.9999986525	13.7001084893
0.5446031964	0.9999986525	0
0.3043052433	0.9999986525	0.9490542189
0.2428233285	0.9999986525	0.553060069
0.554105595	0.9999986525	4631.7091322661
0.799919427	0.9999986525	0.8044770484
0.2275574795	0.9999986525	34.3393486517
0.5078360649	0.9999986525	0.1802984321
0.3369315541	0.9999986525	0
0.9788380809	0.9999986525	2018.2461607911
0.2145237088	0.9999986525	1.276459039
0.6535403016	0.9999986525	9.5738091591
0.9166435085	0.9999986525	0
0.1592953438	0.9999986525	1.7049122178
0.2343787841	0.9999986525	57.7832803351
0.4250188673	0.9999986525	6.3761823284
0.1164782674	0.9999986525	2.5998138387
0.780937621	0.9999986525	18.8742470968
0.2009301372	0.9999986525	0
0.3388189177	0.9999986525	4.6833305158

0.451761582	0.9999986525	0.8508595884
0.2006156025	0.9999986525	0.1129096025
0.0601253596	0.9999986525	3.134096765
0.5611787308	0.9999986525	0.3636783687
0.8245034343	0.9999986525	0
0.2674148366	0.9999986525	0.5796656631
0.7439764253	0.9999986525	4.2255360117
0.2211316849	0.9999986525	0
0.2100793644	0.9999986525	0
0.6312664098	0.9999986525	0
0.2168820396	0.9999986525	40.9637038874
0.0626862108	0.9999986525	0
0.5132523822	0.9999986525	0
0.8440983964	0.9999986525	1.4065332825
0.8857502974	0.9999986525	0.2988243581
0.9620486344	0.9999986525	0
0.0203697157	0.9999986525	0
0.7156514658	0.9999986525	0
0.9987907069	0.9999986525	0
0.0731854378	0.9999986525	0
0.7715767406	0.9999986525	2.1465872929
0.7154064949	0.9999986525	0
0.009922	0.9999986525	1.0267690228
0.3113389925	0.9999986525	2.7407042079
0.1373370704	0.9999986525	2.5324748206
0.2415986349	0.9999986525	0.7645243896
0.7613130938	0.9999986525	0
0.1804180935	0.9999986525	3.17124524
0.8452883217	0.9999986525	0
0.1974741823	0.9999986525	0
0.1035065409	0.9999986525	0.4038795733
0.6636999573	0.9999986525	0
0.274736305	0.9999986525	4.2101813483
0.7121098598	0.9999986525	1.1719498109
0.3048270423	0.9999986525	0
0.8563428645	0.9999986525	9.7709461678
0.1305367552	0.9999986525	5.5113530277
0.0356369513	0.9999986525	9.564850871
0.6149203647	0.9999986525	2.5593408636
0.3528806933	0.9999986525	0.7608375395
0.2342350983	0.9999986525	0.7613640138
0.0766169933	0.9999986525	8.2801201283
0.8262271051	0.9999986525	4.5974118715
0.4117126917	0.9999986525	2.3222054358
0.4248009951	0.9999986525	0
0.3123085494	0.9999986525	2.5887589352
0.8400581978	0.9999986525	0
0.7652825623	0.9999986525	0
0.8725486876	0.9999986525	4.2156030576

0.0531353976	0.9999986525	5.3574678663
0.0256474446	0.9999986525	2.199480186
0.0762088556	0.9999986525	0
0.0154236748	0.9999986525	2.2151630831
0.000246	0.8275161028	20.3568910832
0.0526692147	0.9999986525	19.3372576986
0.5521356273	0.9999986525	6.5583815889
0.6359317515	0.9999986525	0
0.0555151235	0.9999986525	374.5246339777
0.009872	0.9999986525	0.9596877154
0.8995357772	0.9999986525	0.8040361451
0.9035632541	0.9999986525	33.2950967923
0.2664996153	0.9999986525	0
0.990658009	0.9999986525	5.4528200975
0.06130192	0.9999986525	2.3500792376
0.4729816619	0.9999986525	0
0.0745690378	0.9999986525	0.4644696787
0.30755387	0.9999986525	0
0.3422055565	0.9999986525	0
0.2367291597	0.9999986525	7.0957837198
0.1083164583	0.9999986525	0.2872600989
0	0	0
0.4002990937	0.9999986525	0.6710108023
0.3998440872	0.9999986525	1.5097311469
0.4729816619	0.9999986525	0
0.4068414848	0.9999986525	4.010472946
0.4029529639	0.9999986525	0
0.5768417186	0.9999986525	21.8607893582
0.2696944033	0.9999986525	0.9620664081
0.3244745913	0.9999986525	0.2279370154
0.092486174	0.9999986525	14.0908508915
0.739822201	0.9999986525	0.2206998614
0.088074177	0.9999986525	1.4961732486
0.4457838019	0.9999986525	5.4786222226
0.6312664098	0.9999986525	0
0.3722082826	0.9999986525	1253.7428659065
0.4794085293	0.9999986525	3.0605231783
0.9497290657	0.9999986525	0.404140725
0.3114510542	0.9999986525	1.3598691529
0.6004152944	0.9999986525	0
0.6312664098	0.9999986525	0
0.4258593473	0.9999986525	5.3001543691
0.2951570122	0.9999986525	4.0773502838
0.7827359258	0.9999986525	0
0.2046079482	0.9999986525	1.8066746639
0.4729816619	0.9999986525	0
0.4532983454	0.9999986525	0
0.0978568878	0.9999986525	0
0.0502937768	0.9999986525	13.4926068461

0.9295612144	0.9999986525	0
0.9161243593	0.9999986525	1000.0832125812
0.2719775265	0.9999986525	0
0.8270505589	0.9999986525	1.7946825549
0.6038059062	0.9999986525	7.1281058442
0.8799411139	0.9999986525	5.9735241547
0.8867079817	0.9999986525	23.989969669
0.8879179898	0.9999986525	8.5258512165
0.6312664098	0.9999986525	0
0.7118944851	0.9999986525	107.4545640413
0.7973660954	0.9999986525	28.3536117713
0.2562644528	0.9999986525	0
0.5053737405	0.9999986525	20.1839844174
0.956693996	0.9999986525	4.2045950343
0.3106401717	0.9999986525	4.4699221547
0.6299454035	0.9999986525	6.665268115
0.5965449856	0.9999986525	0
0.2108458666	0.9999986525	25.9728416175
0.1360211961	0.9999986525	9.4825072432
0.1120570879	0.9999986525	6.0628995783
0.8013107742	0.9999986525	11.372506078
0.3229921216	0.9999986525	107.4144042359
0.7541152566	0.9999986525	2.4114843261
0.4598814265	0.9999986525	16.1485761279
0.9409150273	0.9999986525	0.5088029733
0.9188764315	0.9999986525	37.8929603956
0.3369010142	0.9999986525	0
0.4689868422	0.9999986525	0
0.6401157822	0.9999986525	0
0.6558474016	0.9999986525	5.2930365029
0.4564352626	0.9999986525	0.1007837655
0.5828055134	0.9999986525	0
0.7754615365	0.9999986525	1352.833766498
0.8649826175	0.9999986525	0
0.6451843357	0.9999986525	0.9903600027
0.8999180697	0.9999986525	0
0.5549084083	0.9999986525	0
0.7610179686	0.9999986525	1.3575446799
0.5643889046	0.9999986525	23.504191467
0.295583973	0.9999986525	4.1106723402
0.6396230452	0.9999986525	0
0.1614640488	0.9999986525	11.9373720351
0.8680680942	0.9999986525	25.9847076144
0.5229916751	0.9999986525	1.2510403218
0.1065211837	0.9999986525	0
0.0108873439	0.9999986525	25.5496271789
0.6007726905	0.9999986525	43.2734051401
0.8316217084	0.9999986525	2.3259767233
0.9901399828	0.9999986525	0.4197293795

0.1945168272	0.9999986525	20.058457359
0.4132608238	0.9999986525	0
0.0839114668	0.9999986525	12.1705077143
0.1692807861	0.9999986525	44.9671504694
0.9191765082	0.9999986525	0
0.9551438997	0.9999986525	0
0.4274140088	0.9999986525	0
0.7401805232	0.9999986525	50.9014770183
0.4296751243	0.9999986525	19.6278814262
0.0794033294	0.9999986525	12.9916435903
0.8628978763	0.9999986525	0
0.1024690122	0.9999986525	0
0.8739146059	0.9999986525	59.8571041477
0.3517538236	0.9999986525	36.7913049295
0.4045086625	0.9999986525	0
0.3640783493	0.9999986525	10.0005697943
0.3484954967	0.9999986525	26.865593168
0.8906852437	0.9999986525	3.4433130549
0.3255656878	0.9999986525	0
0.8009139111	0.9999986525	27.6079028888
0.4692566675	0.9999986525	13.1590677031
0.3507215365	0.9999986525	0
0.3082649839	0.9999986525	81.4311514503
0.3894786461	0.9999986525	0
0.8640786018	0.9999986525	0
0.9690295288	0.9999986525	196.1722096726
0.8495360638	0.9999986525	32.3746944583
0.1203738306	0.9999986525	22.6644858819
0.322476351	0.9999986525	0
0.3058908329	0.9999986525	8.8892366853
0.6120327074	0.9999986525	6.691235047
0.42586574	0.9999986525	175.4337682304
0.6401076801	0.9999986525	0
0.9587394074	0.9999986525	19.0389034702
0	0	0
0	0	0
0.6312664098	0.9999986525	0
0.2470325519	0.9999986525	0
0	0	0
0.5925826907	0.9999986525	299.1638069464
0.2413704323	0.9999986525	0
0.1895976449	0.9999986525	0
0.1833991689	0.9999986525	0
0.0911333331	0.9999986525	0
0.0580611904	0.9999986525	10.6607611165
0.1088732698	0.9999986525	118.9725959669
0.4709465467	0.9999986525	0
0.0339667068	0.9999986525	0
0.3987123375	0.9999986525	1.8317177618

0.4338943948	0.9999986525	5.7837067542
0.8627729229	0.9999986525	3.4121926752
0.1843151824	0.9999986525	4.7609057964
0.1684415707	0.9999986525	89.5367952493
0.6430764004	0.9999986525	8.0923153679
0.9037202029	0.9999986525	0
0	0	0
0.4439895897	0.9999986525	0.3454637232
0.9798677291	0.9999986525	3.016367758
0.143128609	0.9999986525	16.8250774853
0.5806817339	0.9999986525	0.1676823962
0.4961996958	0.9999986525	0
0	0	0
0.820012529	0.9999986525	0
0.9041038105	0.9999986525	20.4405807189
0.483584649	0.9999986525	6.164521118
0.563494321	0.9999986525	9.8433954914
0.0120235524	0.9999986525	0.4428877242
0.9628979499	0.9999986525	0.535068317
0	0	0
0.4729816619	0.9999986525	0
0.500167256	0.9999986525	2.6899214743
0.6098761915	0.9999986525	0
0.9111724591	0.9999986525	129.3190945796
0.8656546451	0.9999986525	40.3159681117
0.4450443753	0.9999986525	18.1304216362
0.1888559741	0.9999986525	0
0.3200866848	0.9999986525	8.5399533595
0.3126650582	0.9999986525	0
0.4665921558	0.9999986525	0.4138697455
0.1750387815	0.9999986525	0
0.2123519788	0.9999986525	15.8410072664
0.0523534773	0.9999986525	0
0.5597803815	0.9999986525	5.0262589199
0.6418903261	0.9999986525	229.4269940592
0.6312664098	0.9999986525	0
0.3422249262	0.9999986525	0
0.3368705857	0.9999986525	0
0.8261457534	0.9999986525	6.1886475541
0.1268440672	0.9999986525	4.6737980731
0.2412548801	0.9999986525	4901.0407813021
0.2210825803	0.9999986525	45.2092798469
0.2629495218	0.9999986525	0
0.1048731431	0.9999986525	40.9489577254
0.8915375808	0.9999986525	3.1468699414
0.5025475507	0.9999986525	28.1059646779
0.7033416276	0.9999986525	0
0.1658550729	0.9999986525	0
0.4432928027	0.9999986525	9.1503375422

0.0192162689	0.9999986525	85.9033246768
0.570442617	0.9999986525	6.1351607995
0.30755387	0.9999986525	0
0.5360715775	0.9999986525	36.5431020421
0.819923128	0.9999986525	0
0.1094771314	0.9999986525	0.3642967286
0.2081758634	0.9999986525	0
0.6891698646	0.9999986525	36.704138267
0.6002015931	0.9999986525	25.4441007734
0.5986769327	0.9999986525	0
0.126375793	0.9999986525	1.0820750333
0.6640401546	0.9999986525	2.8554237795
0.158065926	0.9999986525	0.9175056369
0.5858002378	0.9999986525	31.4847495553
0.9956937972	0.9999986525	5.4792312066
0.0445591306	0.9999986525	0
0.2182395469	0.9999986525	11.2125509742
0.3108284719	0.9999986525	2.0792471891
0.6774889367	0.9999986525	13.0455353959
0.1815459149	0.9999986525	0
0.5355707751	0.9999986525	0
0.4729816619	0.9999986525	0
0.6884020113	0.9999986525	2.2564264815
0.6800170837	0.9999986525	0
0.8482660634	0.9999986525	2.8543876002
0	0	0
0.2840626648	0.9999986525	26.246853308
0.8319679722	0.9999986525	0
0.0463890224	0.9999986525	1.4384246381
0.3417122305	0.9999986525	0.5231068939
0.8002995394	0.9999986525	17.0545466777
0.0377817115	0.9999986525	6.9007732874
0.1096286357	0.9999986525	17.4897415235
0.2734960291	0.9999986525	12.1635002824
0.8995645681	0.9999986525	8.8967622576
0.1556380941	0.9999986525	0
0.9824224401	0.9999986525	1.7264246025
0.0526890107	0.9999986525	0
0.9417589119	0.9999986525	21.1002469941
0.3147141952	0.9999986525	144.6330997838
0.7040856192	0.9999986525	0.1441852899
0.2700733332	0.9999986525	0
0.1052341109	0.9999986525	35.4646241111
0.3887458042	0.9999986525	32.2134298422
0.7627592115	0.9999986525	1.680099196
0.9294003206	0.9999986525	12.9684268205
0.9500282014	0.9999986525	0
0.367423032	0.9999986525	27.3322356798
0.9524430709	0.9999986525	4.4035451058

0.3368402657	0.9999986525	0
0.7880442098	0.9999986525	22.4793220164
0.9483684886	0.9999986525	0.710284553
0.5114603678	0.9999986525	3.6418368657
0.4674805583	0.9999986525	13.8047840945
0.7890873647	0.9999986525	0
0.8395181269	0.9999986525	0
0.3422442988	0.9999986525	0
0.3533972588	0.9999986525	402.2921859989
0.6801958069	0.9999986525	0
0.1264171166	0.9999986525	0.3634193582
0.7268806457	0.9999986525	3.249576231
0.2375303082	0.9999986525	0
0.4596122643	0.9999986525	1.4244580321
0.8258107939	0.9999986525	0.8166490375
0.6017888796	0.9999986525	21.0100122233
0.041963544	0.9999986525	50.9295257727
0.3070429266	0.9999986525	0.4458149868
0.822660106	0.9999986525	29.5324799029
0.8432614305	0.9999986525	14.4588144857
0.0649075771	0.9999986525	1.2735451706
0.8451382378	0.9999986525	10.5913803723
0.6312664098	0.9999986525	0
0.6996982913	0.9999986525	21.2680633147
0.0926836426	0.9999986525	2.1804711367
0	0	0
0.3638439939	0.9999986525	9.2755442294
0.6650525365	0.9999986525	4.982915202
0.8894102602	0.9999986525	0
0.334397409	0.9999986525	4.9047751587
0	0	0
0.9444415618	0.9999986525	0.4906851823
0.876333893	0.9999986525	6.6831911707
0.0687223028	0.9999986525	1.0644104604
0.6429426533	0.9999986525	116.4001403505
0.4323674923	0.9999986525	491.0384959058
0.001444	0.9999986525	15.3020458437
0.6592082737	0.9999986525	0.8553969458
0.7981809663	0.9999986525	12.60625808
0.1003984416	0.9999986525	0
0	0	0
0.2717575636	0.9999986525	18.0993551153
0.8238584751	0.9999986525	0.7508376205
0.21482118	0.9999986525	0
0.8218256301	0.9999986525	0.8979210144
0.7738137113	0.9999986525	1.8052954444
0.8621525211	0.9999986525	0
0.6207479874	0.9999986525	0.6319911237
0.7881808183	0.9999986525	82.6832379277

0.6012087755	0.9999986525	776.1895424129
0.5945137046	0.9999986525	54.4313055925
0.1964936846	0.9999986525	142.9299563277
0.1199323801	0.9999986525	10.6115969914
0.6335850058	0.9999986525	0
0.3096934709	0.9999986525	21.2987975103
0.0760302919	0.9999986525	51.6707039025
0.7591711568	0.9999986525	39.2449698877
0.4473756357	0.9999986525	15.5616208942
0.7037915421	0.9999986525	3.7161496078
0.9613732343	0.9999986525	49.1063860775
0.0868047172	0.9999986525	137.7754388512
0.4008325526	0.9999986525	1.5177721708
0.1283949581	0.9999986525	1762.2713987218
0.9297453084	0.9999986525	46.4638576771
0.7708802216	0.9999986525	0
0.036496703	0.9999986525	0
0.1049625906	0.9999986525	11.7110523258
0.976342528	0.9999986525	410.9575673457
0.3184259492	0.9999986525	276.8439625462
0.4097668878	0.9999986525	2.1690384914
0.8143689091	0.9999986525	0.1496399338
0.1476887201	0.9999986525	2.1653923012
0	0	0
0.4551080169	0.9999986525	2.7533061321
0.2123195545	0.9999986525	0
0.336818305	0.9999986525	0
0.8139883232	0.9999986525	0
0.5202122082	0.9999986525	6.2159094018
0.7230255719	0.9999986525	238.3507737685
0.6982513209	0.9999986525	67.7471928352
0.969747201	0.9999986525	11.8851223589
0.5768478192	0.9999986525	20.8915815769
0.7596702259	0.9999986525	191.9274497322
0.7742695735	0.9999986525	39.9966563829
0.6466866522	0.9999986525	0
0.0298860014	0.9999986525	6.9209694963
0.0810324952	0.9999986525	2.6327975188
0.7156539194	0.9999986525	5.4188499839
0.8457245836	0.9999986525	6.9133923636
0.8465716157	0.9999986525	39.9595590276
0.7736199556	0.9999986525	0
0.6036127077	0.9999986525	0
0.3412808243	0.9999986525	2.9533030379
0.6312664098	0.9999986525	0
0.165586671	0.9999986525	0
0.4239714367	0.9999986525	21.0481102448
0.3318677571	0.9999986525	1036.7540904147
0.6384005035	0.9999986525	0

0	0	0
0.7764014086	0.9999986525	11.5782886211
0.6312664098	0.9999986525	0
0.445678097	0.9999986525	1.5234491133
0.335860788	0.9999986525	0
0.9684106664	0.9999986525	260.0917178005
0.4883606041	0.9999986525	0.9342560333
0.6112644115	0.9999986525	0
0.7182348945	0.9999986525	10.3531327079
0.575420113	0.9999986525	461.9451583902
0.6312664098	0.9999986525	0
0.8648300803	0.9999986525	8.7997531097
0.9304280452	0.9999986525	7.8979988375
0.774094233	0.9999986525	5.1475553827
0.6844757672	0.9999986525	0
0.4711323033	0.9999986525	40.7934486815
0.5031264478	0.9999986525	5.8791003591
0.1706366002	0.9999986525	11.5865732584
0.4729816619	0.9999986525	0
0.7455515425	0.9999986525	0
0.711681918	0.9999986525	18.5730935737
0.5205715091	0.9999986525	0.8846601861
0.2571055314	0.9999986525	10.0502223076
0.3276766108	0.9999986525	3.2158508328
0.0865171817	0.9999986525	0.7240564435
0.824618667	0.9999986525	0
0.122581746	0.9999986525	0.7315757733
0.8627206082	0.9999986525	44.3235046659
0.060165929	0.9999986525	0
0.6787706888	0.9999986525	11.2282442548
0.4012936766	0.9999986525	2.4261067677
0.6378787426	0.9999986525	7.1454650061
0.0537908357	0.9999986525	2.0773252921
0.9755804334	0.9999986525	10.838812059
0.7582564202	0.9999986525	4.6862394167
0.7582164464	0.9999986525	0
0.2603890577	0.9999986525	0.4886286682
0.1723092051	0.9999986525	0
0.0303087689	0.9999986525	2.7917284188
0.4829186361	0.9999986525	51.4316915168
0.5371391743	0.9999986525	39.5576950763
0.8553102866	0.9999986525	0.6928050979
0.0764535728	0.9999986525	0
0.2858296997	0.9999986525	2.362721703
0.0540439213	0.9999986525	0.7959857257
0.8152841669	0.9999986525	1.1659216351
0.2901193172	0.9999986525	0
0.5615505089	0.9999986525	0.3242027097
0.5984393818	0.9999986525	1.2966516468

0.6112731323	0.9999986525	0.0920343613
0.2649268485	0.9999986525	5.9588251118
0.8097121582	0.9999986525	0
0.4529215519	0.9999986525	4.4695060489
0.2916505459	0.9999986525	1.2205854391
0.5849654122	0.9999986525	2.5802534737
0.0179134606	0.9999986525	21.5642456594
0.5760734516	0.9999986525	1.7588028493
0.4769536998	0.9999986525	3.7704028718
0.2520920589	0.9999986525	8.0578627208
0.109330846	0.9999986525	7.4609980542
0.8964466585	0.9999986525	3.7654834823
0.1468219729	0.9999986525	24.0829540476
0.9574086261	0.9999986525	3.7081915553
0.7697732631	0.9999986525	0.4568885544
0.8311004902	0.9999986525	0
0.7210154348	0.9999986525	4.6194278165
0.3815805044	0.9999986525	5.1778351202
0.8384494899	0.9999986525	5.8998977724
0.4176057685	0.9999986525	0.7713777181
0.0255582586	0.9999986525	33.9891552942
0.7710820096	0.9999986525	0
0.3659191347	0.9999986525	4.5835965438
0.1878670551	0.9999986525	0.3783513831
0.6872736883	0.9999986525	64.9484544697
0.8828473767	0.9999986525	6.8038714596
0.4616758549	0.9999986525	0
0.7673814295	0.9999986525	6.2517955238
0.3413045023	0.9999986525	0
0.2566679808	0.9999986525	12.4922046541
0.3139074885	0.9999986525	4.1656830466
0.7632178126	0.9999986525	19.7079115217
0.2670846616	0.9999986525	56.7433985778
0.1252850474	0.9999986525	0
0.8305929454	0.9999986525	0
0.4729816619	0.9999986525	0
0.9448924112	0.9999986525	6.0522462538
0.3243619244	0.9999986525	0
0.3048384362	0.9999986525	0
0.2629837647	0.9999986525	0
0.4870143658	0.9999986525	0
0	0	0
0.1833516434	0.9999986525	0
0.2630990778	0.9999986525	216.4504845341
0.0674617366	0.9999986525	5823.0730352085
0.6400995704	0.9999986525	0
0.4729816619	0.9999986525	96.7287879603
0.5975326213	0.9999986525	0
0.8934875116	0.9999986525	0

0	0	0
0.5574487432	0.9999986525	42.0828033676
0	0	0
0.6400914532	0.9999986525	0
0.2671262295	0.9999986525	0
0.4074966293	0.9999986525	0
0.6400833283	0.9999986525	0
0.2410278045	0.9999986525	141.2136850971
0.9639059797	0.9999986525	3.051451844
0.5784344017	0.9999986525	4.4070359001
0.9417926013	0.9999986525	0
0.1017914574	0.9999986525	0
0.1563169283	0.9999986525	2.821557764
0.991323263	0.9999986525	30.7272039948
0.4894512186	0.9999986525	220.9076373687
0.3422566312	0.9999986525	0
0.6312664098	0.9999986525	0
0.2681120625	0.9999986525	0
0.0102590286	0.9999986525	6.1655286279
0	0	142.7403195306
0	0	0
0.8623129952	0.9999986525	0
0.5127389705	0.9999986525	0
0.6400751957	0.9999986525	0
0.6312664098	0.9999986525	0
0.3242500671	0.9999986525	0
0.6400670554	0.9999986525	0
0	0	0
0.083035169	0.9999986525	741.7685144145
0.0653642809	0.9999986525	0
0.6400589073	0.9999986525	0
0.1924179352	0.9999986525	0
0.4870157798	0.9999986525	0
0.3075114913	0.9999986525	0
0.6400507514	0.9999986525	0
0.675240439	0.9999986525	0
0.6098761915	0.9999986525	0
0	0	0
0.7477376201	0.9999986525	0
0.8237959601	0.9999986525	0
0.7572756953	0.9999986525	0
0.9038891727	0.9999986525	8.8867437702
0.8048070591	0.9999986525	12.0222896031
0.7827532512	0.9999986525	11.9922611776
0	0	0
0.4663675365	0.9999986525	0
0.1077411884	0.9999986525	2.384913896
0.9043314264	0.9999986525	1.5781365633
0.1705997813	0.9999986525	2.7414439775

0.6312664098	0.9999986525	0
0.6114011817	0.9999986525	47.8067863888
0.3367964154	0.9999986525	0
0.0375623163	0.9999986525	0
0	0	0
0.7795173107	0.9999986525	0
0	0	0
0.692934467	0.9999986525	479.42356223
0.4442132617	0.9999986525	129.5164207185
0.8041181558	0.9999986525	106.3877489451
0.9191561881	0.9999986525	0
0.3367745977	0.9999986525	0
0.8121565378	0.9999986525	5.4556075573
0.8989038215	0.9999986525	6.255349784
0.152518963	0.9999986525	0
0.5279430513	0.9999986525	244.2262114511
0.5696177683	0.9999986525	209.0550929791
0.1140175576	0.9999986525	39.0780009074
0.4842299487	0.9999986525	6.6116572642
0.7903359401	0.9999986525	70.8031674335
0.1165170815	0.9999986525	52.2105957861
0.4064562349	0.9999986525	243.130018305
0.5088664245	0.9999986525	0
0.6576109298	0.9999986525	0
0.3125369941	0.9999986525	10.5972246253
0.6660936419	0.9999986525	151.4607892125
0.5669277132	0.9999986525	15.6532063504
0.3348455007	0.9999986525	23.2378916407
0.243690603	0.9999986525	3203.5997087142
0.3034471939	0.9999986525	20.9260720701
0.5935462032	0.9999986525	1328.1606654545
0.3367979258	0.9999986525	0
0.4319661379	0.9999986525	80.5090216864
0.5293936932	0.9999986525	8.621272972
0	0	0
0.1128181129	0.9999986525	16.7132652615
0.9864280424	0.9999986525	92.7862231664
0.2450265474	0.9999986525	8.3079940579
0.9465694067	0.9999986525	1402.9671311594
0.9514095858	0.9999986525	3562.5956673369
0.9223465747	0.9999986525	6.8006590557
0.2969472546	0.9999986525	6.5025755019
0.3041262027	0.9999986525	12.926634142
0.4535601143	0.9999986525	0
0.5815644304	0.9999986525	0
0.4019178729	0.9999986525	0
0.7336417674	0.9999986525	57.1991894148
0.6977441357	0.9999986525	56.5459509918
0.2808442824	0.9999986525	96.6933691437

0.9295398119	0.9999986525	0
0.209000459	0.9999986525	610.3975138979
0.2099309777	0.9999986525	3.0870889775
0.165432723	0.9999986525	28.9748556995
0.1559593411	0.9999986525	0
0.6489277269	0.9999986525	85.3558274365
0.3693452273	0.9999986525	41.7914288635
0.4309005724	0.9999986525	88.2122232365
0.278845883	0.9999986525	0
0.1624662765	0.9999986525	51.4880996601
0.3422689655	0.9999986525	0
0.6396090899	0.9999986525	0
0.6312664098	0.9999986525	0
0.4729816619	0.9999986525	0
0.1396467944	0.9999986525	32.7122441786
0.875969564	0.9999986525	172.440813443
0.898776222	0.9999986525	4.6468627184
0.0484934732	0.9999986525	56.9422298936
0.6395951205	0.9999986525	0
0.4724616049	0.9999986525	22.9401317087
0.7020456398	0.9999986525	137.6366054058
0.1313463706	0.9999986525	9.7084408504
0.1254465051	0.9999986525	195.6876344326
0.5429239325	0.9999986525	62.8737121742
0.8997764996	0.9999986525	402.7442239022
0.8936749751	0.9999986525	272.1665458712
0.3341744274	0.9999986525	0
0.1643222828	0.9999986525	14.7122174568
0.9682951831	0.9999986525	2.2056345052
0.7986858854	0.9999986525	0
0.7198946882	0.9999986525	113.1565166969
0.3025496341	0.9999986525	19.289232369
0.422831259	0.9999986525	63.887804306
0.1248520745	0.9999986525	16.5422587888
0.7407721787	0.9999986525	0
0.9299099546	0.9999986525	0
0	0	0
0.2694039052	0.9999986525	0
0.1126708459	0.9999986525	36.639579437
0.4451595761	0.9999986525	66.6842401847
0.5663729164	0.9999986525	0
0.2799449745	0.9999986525	20.650603412
0.8301052909	0.9999986525	0
0.5210726387	0.9999986525	47.9835719803
0.4753884256	0.9999986525	153.4651006991
0.2773981242	0.9999986525	74.038203121
0.659470576	0.9999986525	0
0.1015055748	0.9999986525	0
0	0	0

0.4682443963	0.9999986525	30.4402986895
0.9907446101	0.9999986525	0
0.9824907806	0.9999986525	9.5435341934
0.3910664435	0.9999986525	0
0.8871936137	0.9999986525	0
0.3422813017	0.9999986525	0
0.9095301223	0.9999986525	12.3224260911
0.6320516478	0.9999986525	49.7335466789
0.8178267826	0.9999986525	438.1339506805
0.895403794	0.9999986525	96.4307533517
0.503925128	0.9999986525	55.6815163166
0.1441817869	0.9999986525	0
0.8509977861	0.9999986525	0
0.32673388	0.9999986525	65.7809973238
0.3255194095	0.9999986525	0
0.9099202824	0.9999986525	0
0.8902606355	0.9999986525	0
0.7877451832	0.9999986525	42.7469997785
0.4930282767	0.9999986525	102.3525547022
0.7046815568	0.9999986525	0
0.3989555586	0.9999986525	37.5988976457
0.866168519	0.9999986525	7.3034827454
0.7724720705	0.9999986525	2491.6724400223
0.5681924403	0.9999986525	59.3525958715
0.0879969158	0.9999986525	39.4722856699
0.4359177176	0.9999986525	20.4837433328
0.4033106751	0.9999986525	136.581522567
0.2362816933	0.9999986525	0
0.0479538054	0.9999986525	130.2877552118
0.1558353016	0.9999986525	115.7766479858
0.5616530502	0.9999986525	0
0.5555387546	0.9999986525	755.7801692374
0.7100912261	0.9999986525	0
0.9045688578	0.9999986525	0
0.6526020449	0.9999986525	54.5316656957
0.7907117964	0.9999986525	4.2115242731
0.1200594084	0.9999986525	28.1843336542
0.6182694603	0.9999986525	13.7405042649
0.6345291764	0.9999986525	0
0.0146967225	0.9999986525	2564.5435271045
0.3077084378	0.9999986525	154.8227723747
0.6054639115	0.9999986525	6.0080106432
0.1759958556	0.9999986525	0
0.5988905907	0.9999986525	1693.0661055021
0.4729816619	0.9999986525	0
0.5603810076	0.9999986525	6.7480889576
0.5156465887	0.9999986525	163.8227002964
0.2040876823	0.9999986525	1.2354421911
0.7737030665	0.9999986525	51.3055354831

0.7616491716	0.9999986525	13.9161109472
0.9427075729	0.9999986525	0
0.9266259698	0.9999986525	2.8927245366
0.4035844757	0.9999986525	11.0240290194
0.9215958751	0.9999986525	1.8502201775
0.7013652122	0.9999986525	21.4574423996
0.9581498735	0.9999986525	8717.2909966148
0.2358864528	0.9999986525	0
0.8421690438	0.9999986525	0
0.4451486533	0.9999986525	4.7703201644
0.0954067445	0.9999986525	0
0.8273992052	0.9999986525	0
0	0	0
0.4332664227	0.9999986525	6.590730263
0.6264550159	0.9999986525	0
0.2599620064	0.9999986525	9.4181958444
0.3958838451	0.9999986525	0
0.6961873218	0.9999986525	4.398960372
0.6279067352	0.9999986525	0
0.5133642284	0.9999986525	0
0.7110428847	0.9999986525	0
0.7067934683	0.9999986525	15.2840162715
0.766018097	0.9999986525	7.8775304523
0.445640383	0.9999986525	379.65692078
0.9221730054	0.9999986525	33.9795429765
0.9050518658	0.9999986525	19.4080997553
0.7114205502	0.9999986525	3.2114835957
0.6021342873	0.9999986525	10.666189546
0.331771886	0.9999986525	44.4561601232
0.4740950624	0.9999986525	0
0.6476223782	0.9999986525	103.2361978687
0.9724737277	0.9999986525	189.9421628431
0.2953037741	0.9999986525	0
0.8713327841	0.9999986525	2.1050627058
0.6509616039	0.9999986525	5.1820264652
0	0	0
0.2519797557	0.9999986525	7.4364852473
0.1508955705	0.9999986525	31.0578760519
0.1305728296	0.9999986525	1.6130573806
0.9081415075	0.9999986525	2.2062992315
0.0714034426	0.9999986525	0
0.1956472467	0.9999986525	197.6742866242
0.2613887988	0.9999986525	0
0.5519199731	0.9999986525	42.7297073028
0.6335300217	0.9999986525	6.5194319499
0	0	0
0.2700509692	0.9999986525	0
0.6312664098	0.9999986525	0
0.6201159739	0.9999986525	0

0.9332162408	0.9999986525	0
0.2462165674	0.9999986525	28.0031379779
0.2078415166	0.9999986525	1.7939510267
0.4074204595	0.9999986525	0
0.0817929279	0.9999986525	13.7955380652
0	0	0
0.4462943287	0.9999986525	8.1835522276
0.0963498628	0.9999986525	6.9182497022
0.8410216597	0.9999986525	1.8875933532
0.2315529376	0.9999986525	19.1933064209
0.0451799796	0.9999986525	0
0.428253236	0.9999986525	0
0.3996627299	0.9999986525	4110.6187372556
0.9394205604	0.9999986525	2.5038362233
0.1916318797	0.9999986525	23.4059783213
0.7801769099	0.9999986525	142.9818429139
0.6559060406	0.9999986525	45.880011797
0.20212796	0.9999986525	3.2240961007
0.6716427874	0.9999986525	0
0.4729816619	0.9999986525	0
0.5062089909	0.9999986525	4.5244511459
0.3473329164	0.9999986525	199.7105037819
0.9187276249	0.9999986525	2.7884365759
0.95666384	0.9999986525	0
0.8636351028	0.9999986525	2.0375582837
0.1832126967	0.9999986525	0
0.4645206821	0.9999986525	383.8685758423
0.8448306999	0.9999986525	5.6460855155
0.1607921167	0.9999986525	369.0184336662
0.8506696006	0.9999986525	0
0.6312664098	0.9999986525	0
0.006722	0.9999986525	124.7818505052
0.8432103149	0.9999986525	25.8362812537
0.9396674585	0.9999986525	6.8718909929
0.5250134956	0.9999986525	69.3721937132
0.6436816114	0.9999986525	1.8965747918
0.7090698258	0.9999986525	212.8409711733
0.5138011687	0.9999986525	139.4558383946
0.1838157636	0.9999986525	6.4667464462
0.8296976079	0.9999986525	126.7171988292
0.2489938165	0.9999986525	13.6241243973
0.191467057	0.9999986525	791.2778582673
0.201300466	0.9999986525	6.2254135304
0.8938757837	0.9999986525	755.4656703373
0.258785772	0.9999986525	0
0.6515402091	0.9999986525	50.4623717049
0.7007955928	0.9999986525	16.5959730469
0.362683073	0.9999986525	0
0.4094058881	0.9999986525	0

0.1108434937	0.9999986525	12.7332585102
0.9295183821	0.9999986525	0
0.1547620062	0.9999986525	112.5006004046
0.3480022535	0.9999986525	108.2323967089
6.95E-05	0.3402364001	0
0.7077215466	0.9999986525	15.4694576063
0.4276235625	0.9999986525	30.124297414
0.9622006496	0.9999986525	46.2833390819
0.7668600238	0.9999986525	0
0.701596014	0.9999986525	69.9269499867
0.3251539598	0.9999986525	19.4657847139
0.0759962877	0.9999986525	26.0777277997
0.4884984835	0.9999986525	218.2082388607
0.2677751326	0.9999986525	12.7958406335
0	0	0
0.1872702779	0.9999986525	137.0197919437
0.806158703	0.9999986525	6099.963104735
0.8212281066	0.9999986525	83.6104246327
0.1971129115	0.9999986525	83.1387929576
0.5852222	0.9999986525	0
0.9189723866	0.9999986525	0
0.9818326254	0.9999986525	13.6214890003
0.1536453212	0.9999986525	0
0.6242684492	0.9999986525	5.8765148795
0.7911442975	0.9999986525	0.0957950494
0.8282802719	0.9999986525	1.1006470182
0.30755387	0.9999986525	0
0	0	0
0.4729816619	0.9999986525	0
0.4729816619	0.9999986525	0
0.4202028298	0.9999986525	0.6732175682
0.0133870022	0.9999986525	0.7064556539
0.493637188	0.9999986525	0
0.3646889839	0.9999986525	0
0.0351452006	0.9999986525	8.0096330229
0.0564237847	0.9999986525	0
0.4729816619	0.9999986525	0
0.6312664098	0.9999986525	0
0.2385324977	0.9999986525	0
0.7174858682	0.9999986525	161.974830673
0.1589977413	0.9999986525	5.7846569799
0.5300704547	0.9999986525	0.6986892211
0.941648024	0.9999986525	16.574168129
0.0558197071	0.9999986525	30.6602118323
0	0	0
0.8828790939	0.9999986525	6.0590739662
0.8891028345	0.9999986525	1.7440120935
0.5084557729	0.9999986525	20.847437942
0.7222724768	0.9999986525	0.3850533554

0.2128564132	0.9999986525	19.3736708174
0.1402016068	0.9999986525	4.420592038
0.8027364336	0.9999986525	0.4074236338
0.4729816619	0.9999986525	0
0	0	0
0	0	0
0	0	0
0.6312664098	0.9999986525	0
0.4708228508	0.9999986525	0
0.5978527655	0.9999986525	0.7443297615
0.1484458587	0.9999986525	5.0867859267
0.1401178825	0.9999986525	22.9426230349
0.9415703124	0.9999986525	15.1280277873
0.8480178454	0.9999986525	4.0909309238
0.5502216301	0.9999986525	1.4951708016
0.2773439149	0.9999986525	0.3181101421
0.9170016869	0.9999986525	501.0808180864
0.1094290886	0.9999986525	27.5626912713
0.3961676117	0.9999986525	9.2747814901
0.0999018688	0.9999986525	0.0265764371
0.5373586799	0.9999986525	0
0.7111259648	0.9999986525	0
0.008644	0.9999986525	1.7400330528
0.7994482517	0.9999986525	0.3574038088
0.0448837175	0.9999986525	0
0.5091947972	0.9999986525	2.7189332146
0.3275329881	0.9999986525	12.7176647626
0.7362389705	0.9999986525	0
0.4834097331	0.9999986525	0.3661219719
0	0	0
0.8558447059	0.9999986525	1.5330155359
0.1213683924	0.9999986525	0
0.3527794056	0.9999986525	0
0.831785732	0.9999986525	2.6586417431
0.2979520136	0.9999986525	8907.2877417456
0.6232583387	0.9999986525	9.5808351417
0.5902208908	0.9999986525	0
0.5190685553	0.9999986525	304.0136002794
0.8715183435	0.9999986525	21.4590072493
0.2699644066	0.9999986525	0
0.3800263622	0.9999986525	2.2613150803
0.4029985819	0.9999986525	0
0.1137472132	0.9999986525	0
0.7333025124	0.9999986525	0.7750226462
0.885474691	0.9999986525	5.8732706749
0.4567908393	0.9999986525	1.3394009339
0.7212376384	0.9999986525	0.4407385284
0.5759063627	0.9999986525	90.7715581088
0.8762477976	0.9999986525	3.3721917445

0	0	0
0.8654204851	0.9999986525	1.1418731779
0.6894750864	0.9999986525	0
0.9161280232	0.9999986525	5.4514779342
0.0962919658	0.9999986525	0
0.0331786336	0.9999986525	0.4783197851
0.3697387786	0.9999986525	0
0.6312664098	0.9999986525	0
0.7134073247	0.9999986525	0.509545854
0.6007944363	0.9999986525	5.956837643
0.7957329055	0.9999986525	64.4090025211
0.1591596016	0.9999986525	83.0407519282
0.4561688235	0.9999986525	2.4310875433
0.6400425875	0.9999986525	0
0.1081991237	0.9999986525	5.3506831697
0.0437834568	0.9999986525	0
0.1245783066	0.9999986525	8.0396678766
0.587603424	0.9999986525	0
0.2134028242	0.9999986525	1.2036262956
0.2104674619	0.9999986525	11.5617158989
0.5541739852	0.9999986525	0.7463387913
0.0717034125	0.9999986525	35.5968893909
0.1822399813	0.9999986525	8.457484976
0.6312664098	0.9999986525	0
0.6540449529	0.9999986525	16.0693471955
0.7267806592	0.9999986525	5.3933579333
0.947663769	0.9999986525	0.2632049628
0.7869424838	0.9999986525	40.4270653906
0.7436391458	0.9999986525	3.1360999125
0.2730523585	0.9999986525	3.5575309221
0.3788472074	0.9999986525	0.1425919468
0.1977459506	0.9999986525	7.8795738514
0	0	0
0.012790629	0.9999986525	0
0.147169562	0.9999986525	0
0.6202596649	0.9999986525	0
0.795434514	0.9999986525	6.1547509878
0.1814821998	0.9999986525	0
0.1043683498	0.9999986525	0
0.8983822611	0.9999986525	3.3228003716
0.0218059143	0.9999986525	2.1276202806
0.3064933093	0.9999986525	25.1540918462
0.9717890975	0.9999986525	0.4875190916
0.7786863224	0.9999986525	1.2655344929
0.563488417	0.9999986525	17.8781082498
0.3663864948	0.9999986525	0
0.8310504755	0.9999986525	0.5660974302
0.0291500092	0.9999986525	0
0.2919709353	0.9999986525	0

0.9740736439	0.9999986525	0.4006305099
0.9299321133	0.9999986525	0
0.6400344157	0.9999986525	0
0.9184573176	0.9999986525	4.6269700098
0.3603303561	0.9999986525	0
0.0566478538	0.9999986525	0.3736525397
0.9488639996	0.9999986525	0.755772959
0.7452797789	0.9999986525	18.2623747798
0.9758614514	0.9999986525	1.3593616346
0.2745631932	0.9999986525	0
0.5204622894	0.9999986525	6.7666775434
0.3211738513	0.9999986525	0
0.0327023566	0.9999986525	1.0317670194
0.9226307129	0.9999986525	1.2339268069
0.7110379166	0.9999986525	0
0.8243067661	0.9999986525	5.2508783849
0.207833914	0.9999986525	1.5846235479
0.0489978395	0.9999986525	7.3874475978
0.8676599942	0.9999986525	0.2287198021
0.728544482	0.9999986525	5.4949773659
0.5289736622	0.9999986525	0.3710692707
0.1681874456	0.9999986525	0
0.2244601913	0.9999986525	0
0.762872261	0.9999986525	0
0.217316655	0.9999986525	0
0.8691196401	0.9999986525	39.496885125
0.5701636471	0.9999986525	0
0.5199398437	0.9999986525	49.5905335458
0.408775356	0.9999986525	7.2324715698
0.3100706595	0.9999986525	2.548688265
0.9203586528	0.9999986525	0
0.7912874304	0.9999986525	0.3934994205
0.0938953807	0.9999986525	0.5864433834
0.7918722432	0.9999986525	5.0978685547
0.4073444098	0.9999986525	0
0.3477510548	0.9999986525	0
0.5149632824	0.9999986525	1.014579161
0.3778809758	0.9999986525	0.7670718295
0.2013244533	0.9999986525	13.6767943318
0.5147778082	0.9999986525	124.547775858
0.1935417148	0.9999986525	4.5679865268
0.1737119261	0.9999986525	0.3782635847
0.5008482439	0.9999986525	0
0.2517634046	0.9999986525	1.429837784
0.8655934977	0.9999986525	5.1278635868
0.4298936585	0.9999986525	0.9912475678
0.6412039942	0.9999986525	3.126563949
0.9334755424	0.9999986525	0
0.0501284355	0.9999986525	240.8060104766

0.1645744785	0.9999986525	16.579711772
0.9495894789	0.9999986525	0
0.300130697	0.9999986525	0.729181933
0.6395831326	0.9999986525	0
0.4949566078	0.9999986525	12.7724106956
0.8452486826	0.9999986525	2.2580906729
0.8102760018	0.9999986525	80.3406247976
0.6312664098	0.9999986525	0
0.4741507213	0.9999986525	30.8783870898
0.6400262358	0.9999986525	0
0.2810466741	0.9999986525	1.5911161458
0.3607196289	0.9999986525	1.7378144262
0.9517498788	0.9999986525	0
0.3199216881	0.9999986525	0
0.4650088846	0.9999986525	11.2439150314
0.0482757707	0.9999986525	7.4862389049
0.0442335238	0.9999986525	1.9300934177
0.2790480516	0.9999986525	0.423646719
0.6312664098	0.9999986525	0
0.0993653075	0.9999986525	0
0.0113123707	0.9999986525	0
0.7133493916	0.9999986525	0
0.6400180479	0.9999986525	0
0.7671100876	0.9999986525	11.0130604899
0.0320070675	0.9999986525	7.4036473297
0.1862354901	0.9999986525	0
0	0	0
0.8617391408	0.9999986525	0
0.9260539133	0.9999986525	0
0.9301033979	0.9999986525	0
0.6376177446	0.9999986525	0
0.1777016672	0.9999986525	0
0.1235702735	0.9999986525	21.6878747336
0.7523032132	0.9999986525	2.7965512057
0.0661784037	0.9999986525	11.2226770562
0.0302943126	0.9999986525	2.9981220254
0.3355404835	0.9999986525	103.5567024045
0.336016224	0.9999986525	0
0.5735351357	0.9999986525	15.6957386255
0.4956967019	0.9999986525	10.5824092041
0.5725564692	0.9999986525	2.5167461628
0.0702662752	0.9999986525	0
0.6400098519	0.9999986525	0
0.1824997133	0.9999986525	0
0.4995666018	0.9999986525	1.1816868511
0.892855747	0.9999986525	0
0	0	0
0	0	0
0.1436477944	0.9999986525	0

0.0465410097	0.9999986525	2.9192938549
0.8668971272	0.9999986525	0
0.6400016476	0.9999986525	0
0.3954852269	0.9999986525	0
0.8279263705	0.9999986525	6.6402771007
0.6312664098	0.9999986525	0
0.4072684798	0.9999986525	0
0.7031949911	0.9999986525	4442.2921872597
0.7944008835	0.9999986525	0
0.1028170411	0.9999986525	1.49483225
0.8628550101	0.9999986525	0
0.9216786301	0.9999986525	0
0.2367416561	0.9999986525	1.6134762541
0.0482080241	0.9999986525	1.1175065483
0.1313048191	0.9999986525	72.5408422771
0.7894770578	0.9999986525	0.7410607598
0.819853337	0.9999986525	0
0.0611120615	0.9999986525	9.3352042822
0.5402289693	0.9999986525	51.5898322076
0.9236186666	0.9999986525	0.2530189869
0.9308750547	0.9999986525	1.7899003436
0.088838108	0.9999986525	0.5758482061
0.0506940381	0.9999986525	2.3095119042
0.0301215291	0.9999986525	3.1070666094
0.167842804	0.9999986525	0
0.3683594168	0.9999986525	119.9277987405
0.8990150598	0.9999986525	0.1951921405
0.354876457	0.9999986525	0
0.6826247084	0.9999986525	8.0008965651
0.1007774333	0.9999986525	0
0.7533921271	0.9999986525	4.387162714
0.293568253	0.9999986525	368.4439955601
0.4835619501	0.9999986525	5.7785809255
0.0665643683	0.9999986525	0
0.1543004219	0.9999986525	110.2126841117
0.0456801163	0.9999986525	0
0.419233124	0.9999986525	6.4418564793
0.4317058991	0.9999986525	2.1808725621
0.9234377919	0.9999986525	1.3006889709
0.2265898437	0.9999986525	15.1334698883
0.0171314522	0.9999986525	2.6515673374
0.9392570223	0.9999986525	9.2807963144
0.7289538604	0.9999986525	0
0.6676213708	0.9999986525	5.1687138605
0.4297875911	0.9999986525	1.6995629357
0.5099273713	0.9999986525	3368.0517729126
0.8214976662	0.9999986525	0
0.9502745756	0.9999986525	0.3718766246
0.3943562063	0.9999986525	0

0.5779190979	0.9999986525	1.5937810102
0.5263780442	0.9999986525	0
0	0	0
0.6800399336	0.9999986525	23.4415970823
0.2537183485	0.9999986525	0.2439464486
0.9223035161	0.9999986525	2.8349920945
0.3634944499	0.9999986525	0.2945479391
0.6575960336	0.9999986525	0
0.2634068591	0.9999986525	1.0906896221
0.9067447748	0.9999986525	2.8255580465
0.9513724691	0.9999986525	3.8079637252
0.4878342846	0.9999986525	2.1521564069
0.6995351654	0.9999986525	10.7293816808
0.1813401885	0.9999986525	0
0.7078735562	0.9999986525	16.6096427165
0.685395212	0.9999986525	33.3685876655
0.1679618187	0.9999986525	3.4755907315
0.1136596022	0.9999986525	0
0.2698779482	0.9999986525	0
0.1722780911	0.9999986525	0
0.6102093498	0.9999986525	0
0	0	0
0.3454940176	0.9999986525	4.9201999447
0.73679161	0.9999986525	0
0.7893078479	0.9999986525	1.5651284565
0.92995423	0.9999986525	0
0.0426685116	0.9999986525	3.8283438967
0.0625222522	0.9999986525	2.2274485239
0.4002783735	0.9999986525	2.9597578024
0	0	0
0.5070999324	0.9999986525	0
0.6399934351	0.9999986525	0
0.2435650824	0.9999986525	12.1105063578
0.7341491417	0.9999986525	15.8814969858
0.355485408	0.9999986525	0
0.5863659889	0.9999986525	1.5659858928
0.12044948	0.9999986525	0.5014566727
0.7381279711	0.9999986525	1.5707987219
0.7113265836	0.9999986525	5.4965421883
0.9986424141	0.9999986525	0
0.5917437099	0.9999986525	1.0979187635
0.4689936359	0.9999986525	2.9975548116
0.0255310718	0.9999986525	3.0271978484
0.4838081795	0.9999986525	0.2305962408
0.8192353834	0.9999986525	2.700995639
0.5963149222	0.9999986525	0.8985473608
0.9360845898	0.9999986525	6.808330043
0.976962578	0.9999986525	1.1331959267
0.1751266841	0.9999986525	1.6003571035

0.6603104399	0.9999986525	31.7658596333
0.6062408911	0.9999986525	0
0.301206545	0.9999986525	14.7568879628
0.2938785647	0.9999986525	1.0945662935
0.2064692091	0.9999986525	1.9161859889
0.2473403391	0.9999986525	19.7712375204
0.6613681845	0.9999986525	12.6551933066
0.2836168631	0.9999986525	57.0190750082
0.2442064603	0.9999986525	33.8629692521
0.6278787962	0.9999986525	433.5963302535
0.2920059191	0.9999986525	125.6844246368
0.3564747569	0.9999986525	0
0.9646122157	0.9999986525	7.7882412451
0.3980754827	0.9999986525	0
0.6312664098	0.9999986525	0
0.1042751976	0.9999986525	3.7764689472
0.5963220314	0.9999986525	0.7328701126
0.4850384193	0.9999986525	0
0.5981669036	0.9999986525	0
0.6485779051	0.9999986525	4.5086151807
0.3079313236	0.9999986525	0
0.1073911564	0.9999986525	0
0	0	0
0.4925841324	0.9999986525	32.3176211884
0.0241605753	0.9999986525	14.5935115298
0.3405115533	0.9999986525	0.3496159534
0.4467800107	0.9999986525	44.8550688381
0.8352267386	0.9999986525	2.4506481475
0.6312664098	0.9999986525	0
0.9677896046	0.9999986525	0
0.1289178185	0.9999986525	0.1915963542
0.0650469077	0.9999986525	87.0490764267
0.8075980241	0.9999986525	13.1064915193
0.2998264729	0.9999986525	0
0.1359265004	0.9999986525	0
0.497714726	0.9999986525	11.9003871623
0.9175410652	0.9999986525	0.9024472127
0.6484675983	0.9999986525	223.2717526862
0.1456156208	0.9999986525	2.8489544841
0.1243722829	0.9999986525	138.96875753
0.8241968538	0.9999986525	0
0.3442314228	0.9999986525	0
0.5024292345	0.9999986525	7.7855295457
0.7582961404	0.9999986525	2.2546925472
0.9439893233	0.9999986525	1.183558215
0.7733974871	0.9999986525	13.2522921661
0.1768875511	0.9999986525	3.5586495672
0.7938582725	0.9999986525	31.8780251856
0.073557232	0.9999986525	9.1837515174

0.4817015056	0.9999986525	58.9550715084
0.8954271926	0.9999986525	7.2706840187
0.9368258704	0.9999986525	0
0.3933638262	0.9999986525	0
0.6199748465	0.9999986525	0
0.6223452818	0.9999986525	0
0.0533197547	0.9999986525	6.1271416017
0.4599577615	0.9999986525	0
0.1688007831	0.9999986525	12.8688884567
0.2175163368	0.9999986525	1290.5289163038
0.6030327866	0.9999986525	1.290032199
0.3727849069	0.9999986525	3.418130764
0.6416011123	0.9999986525	33.3927151153
0.288462436	0.9999986525	65.100078495
0.0715465804	0.9999986525	3.5993596423
0.3552305115	0.9999986525	106.8128545369
0.2972607917	0.9999986525	8.4857993872
0.3841967155	0.9999986525	6.3443952461
0.7148845522	0.9999986525	14.7122174568
0.5420678191	0.9999986525	1.8942280088
0.7007998554	0.9999986525	41.821339888
0.2899021415	0.9999986525	0.6003969559
0.8411284826	0.9999986525	12.5966189159
0.6248607638	0.9999986525	0.163755117
0	0	0
0.847376904	0.9999986525	4.5833479325
0.1503080738	0.9999986525	44.9982795474
0.8307007428	0.9999986525	0
0.0180300407	0.9999986525	0
0.0261253817	0.9999986525	3.85460849
0.003287	0.9999986525	21.7779375074
0.284795121	0.9999986525	1.7744823515
0.4732702312	0.9999986525	6.643037327
0.2184003423	0.9999986525	2.668897259
0.9109050764	0.9999986525	7492.4203621575
0.0236084885	0.9999986525	0
0.5548807357	0.9999986525	4.0613594453
0.9165431016	0.9999986525	3.9196426416
0.3598499873	0.9999986525	0
0.0570921892	0.9999986525	19.370591684
0.5621762866	0.9999986525	5.5328603244
0.5089599221	0.9999986525	25.3321520888
0.765675698	0.9999986525	37.1295425975
0.3651390424	0.9999986525	3.8260789326
0.5317113576	0.9999986525	0
0.1566986199	0.9999986525	8.1040230515
0.4576965066	0.9999986525	211.449202684
0.7097096873	0.9999986525	9.5045229088
0.8461932056	0.9999986525	47.9338007932

0.6089582975	0.9999986525	0
0.1174616989	0.9999986525	22.7960627703
0.2521532582	0.9999986525	16.3687953592
0.8463498905	0.9999986525	0
0.0161785427	0.9999986525	88.1995962363
0.7174116871	0.9999986525	14.2717176205
0.3368211778	0.9999986525	0
0.0737240949	0.9999986525	28.9539407695
0.1813119101	0.9999986525	3.9946992078
0.4538206859	0.9999986525	2.4988842312
0.2771190428	0.9999986525	8.3683458705
0.753529848	0.9999986525	0.4427206584
0.6461572079	0.9999986525	2.6332788426
0.6685644226	0.9999986525	0.3532609042
0.3422936397	0.9999986525	0
0.4287973293	0.9999986525	288.6356959174
0.2465524274	0.9999986525	1.9676217136
0.3487206576	0.9999986525	0
0.2816379341	0.9999986525	4.0559012576
0.7088383495	0.9999986525	51.4254315738
0.824517722	0.9999986525	0
0.807270377	0.9999986525	1.147081322
0.4319549086	0.9999986525	11.3691163594
0.8640445658	0.9999986525	0
0.0103162315	0.9999986525	1.1118719627
0.917712536	0.9999986525	0.3526617491
0.2554233407	0.9999986525	0
0.1829236001	0.9999986525	62.2438635549
0.7822040976	0.9999986525	0.6383426589
0.8215988652	0.9999986525	2.4368812553
0.2882239396	0.9999986525	0
0.2806431955	0.9999986525	28.1511579406
0.6312664098	0.9999986525	0
0.4999196684	0.9999986525	423.710135462
0.3132964626	0.9999986525	0.4462594909
0.468890209	0.9999986525	1.5157248946
0.2364880554	0.9999986525	19.0124673481
0.411476053	0.9999986525	18.271551021
0.6779476705	0.9999986525	63.3962083972
0.8535800783	0.9999986525	1.6456420122
0.4611551913	0.9999986525	67.8045315062
0.967362207	0.9999986525	2.2295642615
0.4968881934	0.9999986525	0
0.6312664098	0.9999986525	0
0.6395711862	0.9999986525	0
0.281569868	0.9999986525	0.4374149479
0.3365342242	0.9999986525	0
0.8283849696	0.9999986525	0.5750581791
0.008366	0.9999986525	424.9667090181

0.3208092107	0.9999986525	0
0.6143638592	0.9999986525	0
0.2795304025	0.9999986525	4.5384479012
0.1007522785	0.9999986525	296.4616740411
0.7210715505	0.9999986525	362.1185003821
0.5297455039	0.9999986525	7.6879884846
0.30755387	0.9999986525	0
0.2414997354	0.9999986525	2.5152838581
0.3310382233	0.9999986525	0
0.1473764774	0.9999986525	0
0.8046033353	0.9999986525	0
0.7804730093	0.9999986525	0
0.6428688994	0.9999986525	0
0.6399769851	0.9999986525	0
0.3357235662	0.9999986525	17.8304923114
0.9721781619	0.9999986525	119.5271346861
0.4995790015	0.9999986525	281.9750038778
0.3727878338	0.9999986525	78.0501254576
0.7510586141	0.9999986525	16.3662591343
0.6245749788	0.9999986525	0
0.008664	0.9999986525	0.3839281068
0.5878588946	0.9999986525	24.5387971183
0.7409335523	0.9999986525	0.4523727742
0.488259013	0.9999986525	0.1002972388
0.3726436419	0.9999986525	1.3998971089
0.6495808848	0.9999986525	0
0.8384233905	0.9999986525	0
0.6960531945	0.9999986525	0
0.7190406122	0.9999986525	0
0.425113516	0.9999986525	0
0.8128759137	0.9999986525	1.0343197561
0.5871875893	0.9999986525	11.3863999051
0.3462753291	0.9999986525	3.1668336547
0.7256575535	0.9999986525	3.889525222
0.0108690837	0.9999986525	0
0.2332916707	0.9999986525	224.8494268241
0.7643172224	0.9999986525	24.1901963936
0.4729816619	0.9999986525	0
0.30755387	0.9999986525	0
0.4080896095	0.9999986525	170.2644955916
0.8918410791	0.9999986525	3.395811108
0.4005689208	0.9999986525	17.6200569586
0.9494628378	0.9999986525	1.0663446581
0.4528226283	0.9999986525	6.5088530216
0.7050098457	0.9999986525	25.3321520888
0.8705924279	0.9999986525	0
0.8418886527	0.9999986525	1.935266926
0.30755387	0.9999986525	0
0.0248096448	0.9999986525	3.3785231318

0.6769574328	0.9999986525	15.6447509078
0.4050550325	0.9999986525	1.0677027843
0.9993203995	0.9999986525	0.8005019738
0.4845669345	0.9999986525	4.1267966546
0.9422353826	0.9999986525	0
0.545454642	0.9999986525	0.2656172543
0.7903975421	0.9999986525	0
0.0504620273	0.9999986525	0
0.4729816619	0.9999986525	0
0.8746869024	0.9999986525	13.4639223036
0.6350421984	0.9999986525	10.066313084
0.8706308722	0.9999986525	4.2631980271
0.1930514496	0.9999986525	0.2130675998
0.2201148416	0.9999986525	1.140332255
0.9299198869	0.9999986525	0
0.6794138949	0.9999986525	1.1553116819
0.5483565878	0.9999986525	7.4412001728
0.7668961619	0.9999986525	2.5041482828
0.0869984651	0.9999986525	193.5588626095
0.2714182374	0.9999986525	0.0727583698
0.0463339364	0.9999986525	37.3800931139
0.2964671667	0.9999986525	0
0.1835029244	0.9999986525	27.1606676402
0.1901528641	0.9999986525	134.2201753438
0.0116114119	0.9999986525	47.856033188
0.2765428872	0.9999986525	0.1164785084
0.6866856173	0.9999986525	5.9626886249
0.4062528265	0.9999986525	2.1807712539
0.5758546106	0.9999986525	4.2946873939
0.2942813692	0.9999986525	1395.8847574033
0.5107856256	0.9999986525	0
0.1539579913	0.9999986525	3.1363425844
0.7969109485	0.9999986525	0
0.3539760949	0.9999986525	0
0.7553391059	0.9999986525	0
0.8672940747	0.9999986525	21.6488509448
0.9836589314	0.9999986525	18.4599504461
0.311693834	0.9999986525	0
0.2007590221	0.9999986525	299.6451028526
0	0	0
0.4231307756	0.9999986525	51.5180413403
0.3752153494	0.9999986525	45.7516202495
0.6312664098	0.9999986525	0
0.5929347068	0.9999986525	69.4767615343
0.6976901431	0.9999986525	155.9702264748
0.1596597116	0.9999986525	2.5750576908
0.0181882512	0.9999986525	19.803672173
0.5889435107	0.9999986525	3020.0313905612
0.6515897622	0.9999986525	179.0470907004

0.9960315213	0.9999986525	29.0409756001
0.183208467	0.9999986525	16.0577434559
0.650585018	0.9999986525	9.0004448821
0	0	0
0.0811658415	0.9999986525	58.9441051633
0.2503502727	0.9999986525	0
0.5997990923	0.9999986525	1.2026591659
0.6790737057	0.9999986525	0.2870009685
0.2083913121	0.9999986525	0
0.9410717474	0.9999986525	0
0.8735117859	0.9999986525	18.0993551153
0.3224119953	0.9999986525	0
0.5890232686	0.9999986525	17.6654292683
0.8090945968	0.9999986525	52.5551850078
0.3556026623	0.9999986525	0
0.9328345879	0.9999986525	2.8562099336
0.8748627852	0.9999986525	0
0.789585328	0.9999986525	203.7108274443
0.3359278293	0.9999986525	2.3199612663
0.8410253062	0.9999986525	4.5886907194
0	0	0
0.4071457558	0.9999986525	0
0.7627565222	0.9999986525	0
0.1058565746	0.9999986525	77.123128251
0.8490908098	0.9999986525	14.8328703663
0.1454805395	0.9999986525	6.4029288379
0.2128070945	0.9999986525	188.8510688304
0.9393028999	0.9999986525	0
0.565906017	0.9999986525	19.5259975696
0.5626190186	0.9999986525	7730.8990577869
0.4729816619	0.9999986525	0
0.6395592809	0.9999986525	0
0.2682369802	0.9999986525	0
0	0	0
0.4729816619	0.9999986525	0
0.6312664098	0.9999986525	0
0.8052584862	0.9999986525	1.2208770019
0.9503741735	0.9999986525	5.6702581248
0.5459106281	0.9999986525	4.0322124161
0.9371898735	0.9999986525	1.926671466
0.7941705448	0.9999986525	0
0.4255445408	0.9999986525	2.3060832341
0.0532634512	0.9999986525	0
0.1895370918	0.9999986525	0
0.8547965604	0.9999986525	4.6946463821
0.7813730876	0.9999986525	31.740600848
0.4557321667	0.9999986525	9.0254488567
0.5984456088	0.9999986525	10.6289488519
0.28631197	0.9999986525	1.3613588923

0.1195052767	0.9999986525	25.7646823879
0.4582713183	0.9999986525	3.2658134232
0.4661393733	0.9999986525	36.7784945866
0.001553	0.9999986525	40.116914718
0.6143057484	0.9999986525	1.7407642916
0.813739167	0.9999986525	0
0.7488100631	0.9999986525	0
0.2539233117	0.9999986525	0.4163034291
0.4661639393	0.9999986525	32.2277258752
0.3411417934	0.9999986525	21.7094480652
0.003683	0.9999986525	0
0.7535834202	0.9999986525	291.0086804032
0.3422387866	0.9999986525	0
0.8621667525	0.9999986525	0
0.5816804083	0.9999986525	18.0558199575
0.8079207606	0.9999986525	4.9398043517
0.3823797534	0.9999986525	16.2671206036
0.2070733565	0.9999986525	0
0.2072487387	0.9999986525	62.5263435986
0.8553102999	0.9999986525	50.4568619604
0.6587356268	0.9999986525	0
0.9980771668	0.9999986525	19.0389034702
0.6595366255	0.9999986525	0
0.3418474269	0.9999986525	0
0.0306644445	0.9999986525	1.3629749987
0.1630135946	0.9999986525	88.3098166241
0.507580262	0.9999986525	194.6523532596
0.1171804199	0.9999986525	23.0055835807
0.1886393725	0.9999986525	0
0.9028901657	0.9999986525	27.1687081764
0.6213911013	0.9999986525	13.7045522482
0.0247908661	0.9999986525	4.7637614982
0.5718301909	0.9999986525	1.5803087441
0.1102448693	0.9999986525	18.0703267887
0.0977807096	0.9999986525	0.9223205307
0.9968020164	0.9999986525	0
0.0129948158	0.9999986525	8.3580753365
0.4053143547	0.9999986525	0
0.0267199663	0.9999986525	3.85167554
0.3984911129	0.9999986525	0
0.6312664098	0.9999986525	0
0.0622330059	0.9999986525	5.3196936167
0.1522568123	0.9999986525	6.6243730529
0.141750896	0.9999986525	114.5491974799
0.0863747225	0.9999986525	1.9971230186
0.56221904	0.9999986525	260.1403873641
0.7250967773	0.9999986525	0
0.739828725	0.9999986525	0
0.5965587716	0.9999986525	1214.1130626738

0.536898178	0.9999986525	104.8577870788
0.4114962586	0.9999986525	0
0.50214658	0.9999986525	0
0.1440880612	0.9999986525	5.9554270569
0.1261602021	0.9999986525	0
0.7340149004	0.9999986525	0
0.6116455576	0.9999986525	39.6455096975
0.0195045238	0.9999986525	0
0.1896152973	0.9999986525	0
0.6121599926	0.9999986525	0
0.9037568328	0.9999986525	0
0.7289961201	0.9999986525	0.9995158589
0.9834057416	0.9999986525	5.6451444
0.463465638	0.9999986525	1.2668550031
0.1301583696	0.9999986525	0
0.8115974832	0.9999986525	2.6621092526
0.4265019426	0.9999986525	6.1658485411
0.4070845659	0.9999986525	0
0.6399687474	0.9999986525	0
0.4729816619	0.9999986525	0
0.7568250341	0.9999986525	0
0.1240148457	0.9999986525	1.2010806474
0.3327060685	0.9999986525	0
0.6312664098	0.9999986525	0
0	0	0
0.4570010397	0.9999986525	6.3055371697
0.4562049808	0.9999986525	4.7561538942
0.5985680651	0.9999986525	0.3930381975
0.9997635868	0.9999986525	67.5894561148
0.30755387	0.9999986525	0
0.1515714138	0.9999986525	0
0.9544632035	0.9999986525	0
0.6369450152	0.9999986525	2.3582379597
0.5126359087	0.9999986525	0
0.2470758724	0.9999986525	0
0.3510959979	0.9999986525	0.4296630005
0.8991768821	0.9999986525	0
0.0263742184	0.9999986525	0
0.0204051376	0.9999986525	2.4881856803
0.5767107736	0.9999986525	35.0581392837
0.8919490153	0.9999986525	5.5994400155
0.9386094755	0.9999986525	1.3580508422
0.5281025252	0.9999986525	12.5548141584
0.9294754406	0.9999986525	0
0.4301674295	0.9999986525	1.2723486046
0.1130841827	0.9999986525	0
0.4971577321	0.9999986525	3.5640529221
0.3155704634	0.9999986525	7.0659559919
0.197848822	0.9999986525	17.1186596812

0.7932016426	0.9999986525	0
0.4504777535	0.9999986525	0.4100968927
0.003111	0.9999986525	9.481001387
0.192017961	0.9999986525	0.427269418
0.4729816619	0.9999986525	0
0	0	0
0.9225036484	0.9999986525	1.7481684892
0.5899661918	0.9999986525	0.1563388409
0.8977374343	0.9999986525	2.6154826585
0.5570799434	0.9999986525	90.002416859
0.2362639757	0.9999986525	2.4311696104
0.7567204605	0.9999986525	0
0.8527009984	0.9999986525	0
0.6312664098	0.9999986525	0
0.656809971	0.9999986525	1.5417721297
0.5664525457	0.9999986525	2.8696198335
0.9149183542	0.9999986525	68.7208500932
0.7970635305	0.9999986525	12.5441860841
0.8241082904	0.9999986525	0
0.624295257	0.9999986525	0.3919283897
0.2570352428	0.9999986525	0
0.9530630304	0.9999986525	0
0.792133439	0.9999986525	13.0759886671
0.1416472026	0.9999986525	335.8762623823
0.504930701	0.9999986525	113.0919019835
0.3572793591	0.9999986525	76.8412013841
0.8411361276	0.9999986525	0
0	0	0
0.0826301229	0.9999986525	22.8904185616
0.0242336779	0.9999986525	124.7934891556
0.8858110438	0.9999986525	3.5728533504
0.5521813798	0.9999986525	7.6630757728
0.3718281029	0.9999986525	1.6096406152
0.6615099293	0.9999986525	6.2509075898
0.423162128	0.9999986525	19.7403581581
0.9936739479	0.9999986525	0
0.9648794129	0.9999986525	0.9718623967
0.2864271649	0.9999986525	0
0.9690914711	0.9999986525	0
0.4069625223	0.9999986525	0
0.7358865974	0.9999986525	3.6181351118
0.9595542334	0.9999986525	19.3961371691
0.5556539018	0.9999986525	0
0.3228635304	0.9999986525	14.1198583644
0.672318722	0.9999986525	211.8423131571
0.0812701961	0.9999986525	280.8946046987
0.7281141317	0.9999986525	0
0.2344511068	0.9999986525	8.2592725352
0.9087854602	0.9999986525	18.0037219111

0.7567051535	0.9999986525	0
0.7111738963	0.9999986525	47.7152074838
0.1595241698	0.9999986525	12.2765965194
0.4689159432	0.9999986525	0
0.1442034895	0.9999986525	0
0.0918670679	0.9999986525	5.057351166
0.4418668332	0.9999986525	0
0.4411038781	0.9999986525	0
0.4930187986	0.9999986525	26.8663741104
0.1001908339	0.9999986525	0
0.1528437244	0.9999986525	1.8587287332
0.9099817962	0.9999986525	89.0622567054
0.3833385795	0.9999986525	197.9532167403
0.0333837622	0.9999986525	0
0.8137034411	0.9999986525	37.2482770972
0.3445547933	0.9999986525	0
0.7686404212	0.9999986525	32.2036086746
0.0794931022	0.9999986525	52.04196245
0.326002344	0.9999986525	528.8947461862
0.9526936719	0.9999986525	0
0.2231631965	0.9999986525	8.9257931767
0.3007427974	0.9999986525	112.7470044477
0.7293179667	0.9999986525	2.3614539784
0.7316634432	0.9999986525	408.5054448444
0.0733292421	0.9999986525	21.445824943
0.5798732421	0.9999986525	2006.0535237894
0.1951227671	0.9999986525	0
0.9196155675	0.9999986525	19.0457692846
0.7823785524	0.9999986525	22.1689876422
0.1201081472	0.9999986525	0
0.9820494535	0.9999986525	96.4179900437
0.7787109233	0.9999986525	0
0	0	0
0	0	0
0.172690281	0.9999986525	50.5235187114
0.5324771632	0.9999986525	21.6805904049
0.0697390156	0.9999986525	7.6853780888
0.2648662305	0.9999986525	7.09102017
0.6312664098	0.9999986525	0
0.1668838817	0.9999986525	76.3856227537
0.6926533894	0.9999986525	105.8232023647
0.654821815	0.9999986525	31.3948094673
0.4481326231	0.9999986525	77.1146825717
0.9833766283	0.9999986525	0
0.3368674518	0.9999986525	0
0.2865802249	0.9999986525	1.8053918698
0	0	0
0.2766944735	0.9999986525	3.1487460935
0.4560629902	0.9999986525	3.4830784295

0.9317239415	0.9999986525	24.9421732994
0.5601175697	0.9999986525	6.1021280446
0.5267040439	0.9999986525	0
0.6276945538	0.9999986525	152.1756965843
0.4389528929	0.9999986525	0
0.1579307172	0.9999986525	0
0.9200732415	0.9999986525	15.2017495326
0.3537663061	0.9999986525	0
0.6974905102	0.9999986525	0
0.1198753067	0.9999986525	0
0.2259295023	0.9999986525	0
0.2301763719	0.9999986525	0.2001467289
0.7378014351	0.9999986525	0.9634646074
0.9589422865	0.9999986525	0
0.2658761836	0.9999986525	4.2188695312
0.2562613299	0.9999986525	3.1697860294
0.4568752578	0.9999986525	8.1274688724
0.9027691104	0.9999986525	6.4232926786
0.6685695693	0.9999986525	268.3913254668
0.30755387	0.9999986525	0
0.71531788	0.9999986525	0
0.9744761676	0.9999986525	1.9391454181
0.2448408099	0.9999986525	0.091073711
0.3370729722	0.9999986525	1.5860670565
0.6641525639	0.9999986525	2.0329678845
0.9138518348	0.9999986525	1.6060184956
0.3849321829	0.9999986525	0
0.3548863302	0.9999986525	0.1938417091
0.3264664501	0.9999986525	0.3307919893
0.3932441747	0.9999986525	26.0750942769
0.0587225987	0.9999986525	12.6929412529
0.8310485059	0.9999986525	11.9380466154
0.0455759267	0.9999986525	9.6682306082
0.3191669193	0.9999986525	0
0.5566379105	0.9999986525	7.8664717785
0.710337604	0.9999986525	0
0.1524887164	0.9999986525	0
0.2238221711	0.9999986525	7.2511729562
0.7094149473	0.9999986525	6.0928356783
0.6312664098	0.9999986525	1.8474479484
0.4234541747	0.9999986525	7.3690411925
0.8507194778	0.9999986525	24.571143665
0.3421840084	0.9999986525	0
0.1879819872	0.9999986525	4.5734255478
0.0285136737	0.9999986525	34.6320775254
0.5616557481	0.9999986525	31.0305042458
0.8860593522	0.9999986525	0.1173790003
0.3605294393	0.9999986525	0
0.0715214321	0.9999986525	1.1535872984

0.2753968619	0.9999986525	3.9078000907
0.3246525948	0.9999986525	0
0.4471562367	0.9999986525	0
0.6986464351	0.9999986525	0.4374511785
0.0892934459	0.9999986525	0.540223993
0.2738006077	0.9999986525	0
0.9304858619	0.9999986525	2.1871834276
0.5325745944	0.9999986525	4.6531266609
0.2876748507	0.9999986525	0.1908727533
0.6399605013	0.9999986525	0
0.6098761915	0.9999986525	0
0.2090127879	0.9999986525	15.8378403318
0.1004596157	0.9999986525	6.3623561289
0.735783533	0.9999986525	0
0.127021059	0.9999986525	3.3342821751
0.684141227	0.9999986525	0
0.0110116893	0.9999986525	10.6191468091
0.6399522465	0.9999986525	0
0.7842758518	0.9999986525	0
0.5468192984	0.9999986525	1.4725343843
0.130643658	0.9999986525	2.1153913535
0.0089	0.9999986525	95.5811912401
0.9719047343	0.9999986525	125.972375018
0.9359948725	0.9999986525	2.8775611591
0.890906284	0.9999986525	3.986758459
0.5876169979	0.9999986525	8.994961803
0.9157602696	0.9999986525	20.8727093261
0.4854370708	0.9999986525	183.5941537415
0.5517736115	0.9999986525	0
0.2102963838	0.9999986525	0
0.543712117	0.9999986525	0
0.2195048353	0.9999986525	10.8314024254
0	0	0
0.2919741185	0.9999986525	5.1569377255
0.5311375683	0.9999986525	814.655121968
0.3896935123	0.9999986525	9.3674917039
0.6099728088	0.9999986525	0
0.6444136691	0.9999986525	6.7982349144
0.1984250215	0.9999986525	4.2286655372
0.042833308	0.9999986525	0
0.773285972	0.9999986525	0.5147605554
0.9755064739	0.9999986525	0.9786335766
0.9503648516	0.9999986525	0
0.983371746	0.9999986525	1.4531174838
0.1264105631	0.9999986525	4.3208991505
0.4247417345	0.9999986525	12.7392916182
0.2558549354	0.9999986525	3.2395214517
0.189192496	0.9999986525	3.8847093287
0.6399439832	0.9999986525	0

0.3964508665	0.9999986525	3.3398346265
0.4069016698	0.9999986525	0
0.8159399992	0.9999986525	0.9749001955
0	0	0
0.9711882795	0.9999986525	2.595990227
0.8019222491	0.9999986525	8.4343328845
0.8621810206	0.9999986525	0
0.74806378	0.9999986525	3.7305868635
0.9679601841	0.9999986525	0.3249307442
0.2319726405	0.9999986525	0
0.8774671819	0.9999986525	6.8210801364
0.6399357111	0.9999986525	0
0.3808654804	0.9999986525	0
0.2205746486	0.9999986525	0.2760544971
0.4828992323	0.9999986525	0
0.3258594054	0.9999986525	2.0177494101
0.9298269027	0.9999986525	8.7046343632
0.7289084631	0.9999986525	0
0.4977035196	0.9999986525	0
0.8226109377	0.9999986525	2.9069748033
0.3189966154	0.9999986525	30.3092787525
0.9569093051	0.9999986525	0
0.7136976814	0.9999986525	36.4578742046
0.742975146	0.9999986525	29.1662993637
0.6399274303	0.9999986525	0
0.3079313236	0.9999986525	0
0.6994903007	0.9999986525	3.9960204632
0.686297574	0.9999986525	2.9081867765
0.4068409297	0.9999986525	0
0.4067803018	0.9999986525	0
0.7290190311	0.9999986525	0.8433023548
0.2857631491	0.9999986525	0
0.9320214295	0.9999986525	0
0.589030418	0.9999986525	0.3515069433
0.6057493603	0.9999986525	7.9776922489
0.0514940742	0.9999986525	0.9067856777
0.9790634322	0.9999986525	1.0577910057
0.8715523472	0.9999986525	17.7955175193
0.8982763539	0.9999986525	61.0800904699
0	0	0
0.705949684	0.9999986525	1.7089907666
0.4089408376	0.9999986525	3.4907042012
0.5867752109	0.9999986525	2.6421490933
0.6242051031	0.9999986525	0
0.4951533907	0.9999986525	0
0.4729816619	0.9999986525	0
0.3938780349	0.9999986525	17.9165195641
0.6163674508	0.9999986525	21.423593277
0.267536928	0.9999986525	90.0899668721

0.7234453455	0.9999986525	0.2570846025
0.1831740105	0.9999986525	29.9364687826
0.6391292475	0.9999986525	0
0.8418068752	0.9999986525	0
0.8198896713	0.9999986525	4.6505453464
0.1658997533	0.9999986525	0
0.2007431219	0.9999986525	112.7390701054
0.3409720412	0.9999986525	0
0.8792869546	0.9999986525	24.1846577815
0.9995379596	0.9999986525	0
0	0	0
0.9340755774	0.9999986525	0
0.878625452	0.9999986525	0
0.6033217694	0.9999986525	0
0.4729816619	0.9999986525	0
0.7106720935	0.9999986525	0
0.8153442425	0.9999986525	101.9037187317
0.2046166809	0.9999986525	54.2181687982
0	0	0
0.4360833008	0.9999986525	0
0.2220472467	0.9999986525	0
0.1043565325	0.9999986525	47.8290666167
0.5724646553	0.9999986525	17.5267427742
0.5864511964	0.9999986525	0
0.61076353	0.9999986525	0.4731370054
0.718898592	0.9999986525	1.0383971653
0.7794790015	0.9999986525	0.2747262213
0.496962418	0.9999986525	0
0.2738646449	0.9999986525	4.1047219629
0.6282571468	0.9999986525	4.0998759152
0.5406953062	0.9999986525	20.6006624123
0.8726019896	0.9999986525	0
0.138300169	0.9999986525	0
0.5018328273	0.9999986525	0.9326631859
0.4605668205	0.9999986525	13.723693306
0.8743251087	0.9999986525	0
0.1949701701	0.9999986525	4.6279283409
0.8867188532	0.9999986525	5.3527619824
0.9488383945	0.9999986525	2.1091820378
0.7807036528	0.9999986525	2.6903325468
0.7165721416	0.9999986525	0
0.2196906855	0.9999986525	0
0.1715729412	0.9999986525	1.8174094366
0.7711738546	0.9999986525	6.8182182063
0.6293176171	0.9999986525	0
0.8377389095	0.9999986525	81.7131277354
0.3895168493	0.9999986525	5.4686946131
0.6312664098	0.9999986525	0
0.9794213619	0.9999986525	0

0.5859790763	0.9999986525	0
0.5410902541	0.9999986525	0
0	0	0
0.6395474163	0.9999986525	0
0.9384070544	0.9999986525	1.4502545027
0.8400064021	0.9999986525	0
0.0557716644	0.9999986525	0
0.5242217432	0.9999986525	20.5242079962
0.4631112105	0.9999986525	0
0.6566453363	0.9999986525	0
0.3711957821	0.9999986525	12.7139909067
0.005403	0.9999986525	14.4174269017
0.0216048488	0.9999986525	173.4447232391
0.0454987436	0.9999986525	43.1310071264
0.245842887	0.9999986525	18.023382666
0	0	0
0.5734673091	0.9999986525	13.5156920428
0.0008276	0.9999986525	49.9895108626
0.3087594433	0.9999986525	6.8653699857
0.2571262155	0.9999986525	5.746420937
0.136531563	0.9999986525	0
0.7355239076	0.9999986525	0
0.2010278798	0.9999986525	19.5922757874
0.5078988933	0.9999986525	0
0.3421293051	0.9999986525	0
0.1613218852	0.9999986525	0
0.9837343672	0.9999986525	1.4003518047
0.8766929947	0.9999986525	0
0.188031312	0.9999986525	0.1696936302
0.6280800817	0.9999986525	0.0460731814
0.2870691614	0.9999986525	0
0.9330293765	0.9999986525	55.1028039279
0.4729816619	0.9999986525	0
0.9287239443	0.9999986525	2.6986789563
0.2668006089	0.9999986525	0
0.9642759867	0.9999986525	1.3880865808
0.830929826	0.9999986525	0.497463119
0.8328521325	0.9999986525	6.7536979829
0.4068058149	0.9999986525	0
0.4134605463	0.9999986525	6.479191937
0.6911753359	0.9999986525	13.1319106436
0.6870769918	0.9999986525	0.8561008379
0.7303619296	0.9999986525	8.6036004333
0.445113188	0.9999986525	25.7025486395
0.8796573497	0.9999986525	0.9877022007
0.7035024093	0.9999986525	1.4518591745
0.3083211076	0.9999986525	0
0.6396469928	0.9999986525	3.7109273627
0.9767105334	0.9999986525	4.5332668023

0	0	0
0.7369380543	0.9999986525	8.5756370322
0.8570181068	0.9999986525	5.4905830363
0.5868582631	0.9999986525	40.8212253723
0.3156333433	0.9999986525	0.5259617008
0.6380575653	0.9999986525	0.0256417702
0.5275686568	0.9999986525	0
0.717384407	0.9999986525	8.9435953871
0.5573600678	0.9999986525	0
0.2847272194	0.9999986525	0
0.3079812825	0.9999986525	0
0.3236980505	0.9999986525	0
0.9294539288	0.9999986525	0
0.9752221589	0.9999986525	0.7686049164
0.0616359926	0.9999986525	4.8394086322
0.6155040326	0.9999986525	0.5077333778
0.9068444929	0.9999986525	3.4319265856
0.7781702221	0.9999986525	0.6976186594
0.5198562946	0.9999986525	1.6915429626
0	0	0
0.4251873458	0.9999986525	1.0831624567
0.4729816619	0.9999986525	0
0.717555775	0.9999986525	7.7587657156
0.6367653312	0.9999986525	2.1142763405
0.194462142	0.9999986525	0
0.4548897097	0.9999986525	4.8390982432
0.6399191406	0.9999986525	0
0.122812834	0.9999986525	0
0.333408265	0.9999986525	23.8922950583
0.8451190675	0.9999986525	56.754101942
0.8977764661	0.9999986525	61.8261032032
0.5606488889	0.9999986525	0
0.1387327164	0.9999986525	0
0.5586821891	0.9999986525	6.2894302571
0.6102604878	0.9999986525	0
0.7079082536	0.9999986525	0
0.062097408	0.9999986525	5.2978909688
0.4058360077	0.9999986525	83.4702730588
0.390159987	0.9999986525	1.2191437824
0.373501383	0.9999986525	0.4524141087
0	0	0
0.9050426315	0.9999986525	0
0.6170342111	0.9999986525	0
0.5138625046	0.9999986525	0
0.5715498215	0.9999986525	9.8546299379
0.3675753853	0.9999986525	38.6443304095
0.206959101	0.9999986525	0.9914196885
0.9219037096	0.9999986525	0.0943768006
0.8611849804	0.9999986525	0

0.953901124	0.9999986525	21.1679030967
0.8049885224	0.9999986525	73.2808202618
0.7417866402	0.9999986525	0.9134669427
0.6312664098	0.9999986525	0
0.926892066	0.9999986525	76.3049386139
0.5517277566	0.9999986525	12.7603608357
0.4068313092	0.9999986525	0
0	0	0
0.4068567846	0.9999986525	0
0.6124460062	0.9999986525	3.4743713063
0.8295061845	0.9999986525	0
0.2090117399	0.9999986525	53.6454222715
0.2643435604	0.9999986525	5.5962360636
0.2697915952	0.9999986525	0
0.9043081336	0.9999986525	17.42458536
0.4541753567	0.9999986525	0
0.3382238514	0.9999986525	4.7380199064
0.2085871616	0.9999986525	1.051054623
0.5099123831	0.9999986525	15.581369699
0.5822799996	0.9999986525	41.0790133987
0.5127658181	0.9999986525	0
0.6620791434	0.9999986525	6.6684240185
0.5502620519	0.9999986525	3.25135447
0.8627706067	0.9999986525	0
0.6682536824	0.9999986525	0
0.6876434776	0.9999986525	0
0.0747641669	0.9999986525	8.0528662824
0.9574101076	0.9999986525	3.2298798222
0.8283489874	0.9999986525	0
0.1322652984	0.9999986525	5.5705008149
0.0382063458	0.9999986525	0
0.8787240734	0.9999986525	8.6278454955
0.7422415283	0.9999986525	12.9003219898
0.8408446287	0.9999986525	0.4921483718
0.3259400542	0.9999986525	3.6958655162
0.0908843187	0.9999986525	44.1351391893
0.1922087256	0.9999986525	0
0.4417414744	0.9999986525	0
0.9428927952	0.9999986525	2.4305728854
0.6446426908	0.9999986525	0.2286376193
0.7483847997	0.9999986525	0
0.9708301933	0.9999986525	3.4494101121
0.6685342021	0.9999986525	8.0112124727
0.4901563582	0.9999986525	15.6119477452
0.018731007	0.9999986525	12.2733360197
0.3429185534	0.9999986525	0
0.7467647162	0.9999986525	4.4016183541
0.6399108421	0.9999986525	0
0.0147064002	0.9999986525	9.9897704143

0.081865968	0.9999986525	4.2848152168
0.6312664098	0.9999986525	0
0.7780670381	0.9999986525	10.6689395942
0.8657603858	0.9999986525	0.5262289711
0.9299769722	0.9999986525	24.1662140443
0.8331727714	0.9999986525	2.8890059749
0.0757200086	0.9999986525	7.418987635
0.6183923695	0.9999986525	1.5699271195
0.4965728809	0.9999986525	0
0.8883215721	0.9999986525	40.0536125114
0	0	0
0.9716367649	0.9999986525	4.045648491
0.8845525089	0.9999986525	3.0162392414
0.7576158992	0.9999986525	1.5126859777
0.1573122727	0.9999986525	0.5386234885
0.5753496596	0.9999986525	8.9570781095
0.028265189	0.9999986525	59.4082319756
0.9552092238	0.9999986525	32.4616882316
0.4388784512	0.9999986525	0
0.299945741	0.9999986525	1.9315334172
0.302899849	0.9999986525	7.286688497
0.6410802102	0.9999986525	22.2222902233
0.4515416414	0.9999986525	2.7887801366
0.0413071435	0.9999986525	9.6988133519
0.7727756778	0.9999986525	0
0.5523628768	0.9999986525	0.3758997739
0.1482196169	0.9999986525	0
0.6060168261	0.9999986525	0.9579829294
0.8030244916	0.9999986525	0
0.0724854095	0.9999986525	10.4976979182
0.7775695099	0.9999986525	0
0.2321174487	0.9999986525	0
0.2988035893	0.9999986525	0
0.0497925676	0.9999986525	296.7768760083
0.1696461161	0.9999986525	17.419659218
0.3329624377	0.9999986525	23.4520063172
0.9806924321	0.9999986525	32.5153623591
0.0718719456	0.9999986525	96.2799304755
0.7065907312	0.9999986525	9.1125814046
0.4647866261	0.9999986525	10.61088633
0.316648859	0.9999986525	21.0557824117
0.9608313407	0.9999986525	0
0.2730725253	0.9999986525	15.7535923121
0.2033368579	0.9999986525	4.3155677583
0.4408925509	0.9999986525	8.6900729291
0.8538388036	0.9999986525	0
0.3485716609	0.9999986525	0.9850494396
0.6741551038	0.9999986525	0
0.6739769211	0.9999986525	1.8307335988

0.4770479016	0.9999986525	17.3447501845
0.4982711624	0.9999986525	13.8069903726
0.3979312878	0.9999986525	19.01605313
0.9832385482	0.9999986525	0.4877712349
0.0572850379	0.9999986525	0
0	0	0
0.1678279206	0.9999986525	0
0.4878255919	0.9999986525	0
0.6542644185	0.9999986525	46.5571540268
0.5840496156	0.9999986525	6.6680030587
0.0803305198	0.9999986525	21.4351357778
0.8684223182	0.9999986525	0.2168610986
0.0514494741	0.9999986525	8.1883380088
0.7261875957	0.9999986525	8.6874522798
0.5435148547	0.9999986525	0
0.2135229565	0.9999986525	3.4433379988
0.4974639145	0.9999986525	4.7491683556
0.3048498329	0.9999986525	0
0.6248608476	0.9999986525	169.7247616606
0.9001991773	0.9999986525	3.7798624597
0.7893319488	0.9999986525	0
0.7120156397	0.9999986525	23.7971995613
0.5479643824	0.9999986525	5.5445354763
0.7702011477	0.9999986525	3.9516586776
0.4407406961	0.9999986525	28.654550411
0.830198682	0.9999986525	0
0.4905477094	0.9999986525	4.2333091446
0.4974209181	0.9999986525	0.5557908907
0.1387825445	0.9999986525	0
0.1600987076	0.9999986525	3.1054389686
0.0222121572	0.9999986525	91.1700336906
0.3068697066	0.9999986525	33.5991845606
0.0968167509	0.9999986525	0
0.8094065336	0.9999986525	0
0.153696991	0.9999986525	0
0.30755387	0.9999986525	0
0.6312664098	0.9999986525	0
0.1743256927	0.9999986525	5.7400817557
0.2287840015	0.9999986525	6.0981354276
0.1445998942	0.9999986525	1.6534188489
0.6547943035	0.9999986525	0
0.5976437137	0.9999986525	6.6131947764
0.086997064	0.9999986525	9.4902011152
0.8718790038	0.9999986525	103.2064542566
0.2919342304	0.9999986525	0
0.9951536167	0.9999986525	4.5707868301
0.9577781652	0.9999986525	9.9248164442
0.733491312	0.9999986525	7.816005006
0.6466595264	0.9999986525	2969.8548205985

0.6077192254	0.9999986525	2.0950006238
0.7762307163	0.9999986525	132.7538790774
0.6400533129	0.9999986525	14.6937980208
0.237475479	0.9999986525	5.2492402262
0.7976550249	0.9999986525	1.7787255229
0.1921120265	0.9999986525	24.9050464508
0.7032729126	0.9999986525	0
0.9936081929	0.9999986525	15.8340317913
0.9300804343	0.9999986525	0
0.6597141694	0.9999986525	0
0.0501097273	0.9999986525	0.1188196717
0.4387498428	0.9999986525	0.4933920473
0.6087525604	0.9999986525	0
0.4410372471	0.9999986525	44.1747665787
0	0	0
0.4474270598	0.9999986525	24.9420305206
0.5873422352	0.9999986525	1.2438511123
0.6572947229	0.9999986525	2.0727434724
0.9195976952	0.9999986525	0
0.0675785384	0.9999986525	0
0.22038433	0.9999986525	650.1127097025
0.5991642757	0.9999986525	0
0.4299890388	0.9999986525	0
0.3932075131	0.9999986525	2.8575094401
0.6871093688	0.9999986525	0
0.3366447609	0.9999986525	1.0863093875
0.5548056842	0.9999986525	0.3530224139
0.400699983	0.9999986525	36.9871931265
0.3162406439	0.9999986525	0
0.1623623007	0.9999986525	19.2060588905
0.4664540439	0.9999986525	3.5419698061
0.5163379471	0.9999986525	1.5567387321
0.435027103	0.9999986525	0
0.2697334328	0.9999986525	0
0.4068822413	0.9999986525	0
0.78858353	0.9999986525	0.893246573
0.7278537637	0.9999986525	14.9860729318
0.0848508051	0.9999986525	1.6531730124
0.2418508356	0.9999986525	0
0	0	0
0.0438881598	0.9999986525	12.2219861674
0.184316768	0.9999986525	0
0.6399025346	0.9999986525	0
0.864979928	0.9999986525	1712.069444577
0.2783577595	0.9999986525	3.4424402442
0.606705507	0.9999986525	1.77476966
0.0886039499	0.9999986525	1.0689561849
0.1401837118	0.9999986525	29.178960346
0.2382094153	0.9999986525	143.1271496648

0.6486827223	0.9999986525	0
0.9697007488	0.9999986525	0
0.6312664098	0.9999986525	0
0.8045343905	0.9999986525	7.6221558993
0.3504668415	0.9999986525	0
0	0	0
0.4610683086	0.9999986525	0.407345074
0.167352207	0.9999986525	1.896166238
0.099607911	0.9999986525	1.5443569281
0.8792934625	0.9999986525	0
0.876066859	0.9999986525	8.0536365658
0.1827667308	0.9999986525	245.932098842
0.2949505569	0.9999986525	5.3820358938
0.6312664098	0.9999986525	0
0.475993444	0.9999986525	0
0.7118833661	0.9999986525	0
0.1483985533	0.9999986525	0
0.2722622148	0.9999986525	0
0.1237117385	0.9999986525	58.0372727762
0.8197845654	0.9999986525	0
0.639894218	0.9999986525	0
0.4729816619	0.9999986525	0
0.8262207127	0.9999986525	0.4210897468
0.4979464658	0.9999986525	6.7089430982
0.5905997776	0.9999986525	17.9501803811
0.7013481496	0.9999986525	0
0.7048516408	0.9999986525	0
0.7851998887	0.9999986525	1.5851467143
0.4365769985	0.9999986525	10.148326011
0.1687800025	0.9999986525	4.1101924765
0.8499512785	0.9999986525	3.127057604
0	0	0
0.3053039353	0.9999986525	4.4175415688
0.3048612327	0.9999986525	0
0.4729816619	0.9999986525	0
0.4077071439	0.9999986525	2.2615958988
0.8018928363	0.9999986525	25.0006713497
0.5621131896	0.9999986525	0.9057592863
0	0	0
0	0	0
0.3912508237	0.9999986525	0
0.0844808903	0.9999986525	13.3267520127
0.9522363178	0.9999986525	1.6693191171
0.177846988	0.9999986525	17.1957623919
0.163651345	0.9999986525	0
0.3502606661	0.9999986525	0
0.032424202	0.9999986525	5.7151734906
0.6906955386	0.9999986525	8.8368638084
0.313388752	0.9999986525	1.424688586

0.2895468483	0.9999986525	2.9597992701
0.0414455815	0.9999986525	0
0.5929768901	0.9999986525	2.3278349007
0.0945428834	0.9999986525	6.899270833
0.7192976287	0.9999986525	0
0.2900054443	0.9999986525	654.3525977364
0.7189712006	0.9999986525	2.3772919619
0.2557206298	0.9999986525	43.6076223537
0.7847086861	0.9999986525	1.0599308864
0.9777104068	0.9999986525	48.9646821507
0	0	0
0.369545403	0.9999986525	0
0.3131260816	0.9999986525	1510.5243295853
0.4030442649	0.9999986525	0
0.001621	0.9999986525	10.6680416563
0.3827395088	0.9999986525	7.4474960483
0.1304705834	0.9999986525	0.5001223294
0.623352637	0.9999986525	0.6157226507
0.609997693	0.9999986525	0.1819970924
0.4338493407	0.9999986525	332.9716442483
0.3464562211	0.9999986525	0
0.190981919	0.9999986525	3.0687924594
0.5160178016	0.9999986525	46.7104229596
0.3607190604	0.9999986525	0
0.7356128745	0.9999986525	0.6671793611
0.3225726815	0.9999986525	4.2221579475
0.03640233	0.9999986525	218.2719321209
0.4364254432	0.9999986525	184.5131676686
0.8141424463	0.9999986525	93.6105784448
0.5094571166	0.9999986525	0
0.656613775	0.9999986525	0.4819161908
0.6513113396	0.9999986525	3.0512402927
0.5723064597	0.9999986525	0.9875021327
0.7209110256	0.9999986525	29.6457581961
0.7046700602	0.9999986525	0
0.6183268671	0.9999986525	0
0.0170875374	0.9999986525	23.690245385
0.3560248882	0.9999986525	4.0596424326
0.1590124704	0.9999986525	0
0.7485816677	0.9999986525	0
0.0124868786	0.9999986525	15.8144443126
0.4125837767	0.9999986525	22.0517403868
0.9400166925	0.9999986525	5.9381513634
0	0	0
0.8612741097	0.9999986525	0
0.9667517541	0.9999986525	0.1094550414
0	0	0
0.5268056785	0.9999986525	0
0.0408404091	0.9999986525	42.6461728134

0.2023636652	0.9999986525	8.5412029291
0.7154182679	0.9999986525	0
0.0849060113	0.9999986525	1.6984333796
0.5782808921	0.9999986525	63.5328110645
0.4069076791	0.9999986525	0
0.1326636588	0.9999986525	2.0083628637
0.9991532756	0.9999986525	0
0.240680563	0.9999986525	0
0.2184349367	0.9999986525	8.0880351853
0.4194923764	0.9999986525	12.9097820157
0.2794134512	0.9999986525	74.8408615518
0.3085983118	0.9999986525	0
0.451768979	0.9999986525	8.0357942222
0.8647685969	0.9999986525	0
0.9294323897	0.9999986525	0
0.6398858924	0.9999986525	0
0.2683598949	0.9999986525	6.4985749017
0.7170993657	0.9999986525	5.1735238504
0.3490866283	0.9999986525	21.6155190558
0.3493883869	0.9999986525	0.8679457166
0.6312664098	0.9999986525	0
0.0930936768	0.9999986525	20.2458325861
0.6554532466	0.9999986525	1.3500835458
0.7376052436	0.9999986525	2.8669457103
0.7907332164	0.9999986525	0.2734106666
0.7018497102	0.9999986525	30.6847781903
0.3434297571	0.9999986525	0.4286530872
0.4469845176	0.9999986525	22.166919929
0.5003008606	0.9999986525	0
0.169942875	0.9999986525	10.1417002508
0.9959088092	0.9999986525	3.4231179904
0.3702382278	0.9999986525	1.0395826669
0.677257631	0.9999986525	4.8713462519
0	0	0
0.4224577832	0.9999986525	7.8635603774
0.9739374862	0.9999986525	0
0.4068627897	0.9999986525	11.9109436544
0.3086680007	0.9999986525	23.7900532551
0.6593072569	0.9999986525	22.8763723186
0.8782217932	0.9999986525	28.1841247576
0.6989438288	0.9999986525	320.2313829064
0.4773189296	0.9999986525	0.2971447762
0.3736883341	0.9999986525	22.1851290542
0.8235849838	0.9999986525	3.1269341756
0.5797542181	0.9999986525	4.0285508694
0.8262132576	0.9999986525	0
0.0483257019	0.9999986525	39.3693016968
0	0	0
0.8734477414	0.9999986525	17.5942021702

0.764908729	0.9999986525	1.0785366649
0.6436134927	0.9999986525	2.8924604934
0.6398775575	0.9999986525	0
0.8626429317	0.9999986525	0
0.4031904844	0.9999986525	0
0.8627989384	0.9999986525	0
0.7319694984	0.9999986525	3.595229287
0.7592719436	0.9999986525	0.1970962764
0.0707864227	0.9999986525	8.7186994506
0	0	0
0.6311445471	0.9999986525	40.2112027808
0.1645618061	0.9999986525	12.5739939828
0.2190607627	0.9999986525	0.673469386
0.1494340391	0.9999986525	0
0.8985950722	0.9999986525	0.3241579376
0.4880906547	0.9999986525	0.2167895885
0.849987592	0.9999986525	18.0375403778
0.6898496984	0.9999986525	2.1600961246
0.2212713801	0.9999986525	19.651836749
0.1187920488	0.9999986525	4.6989329752
0.6312664098	0.9999986525	0
0.3715177148	0.9999986525	2.6105441267
0.4814337289	0.9999986525	1.1048243463
0.5096284341	0.9999986525	0
0.7961450205	0.9999986525	8.5037438106
0.7105837129	0.9999986525	0
0	0	0
0.4642967325	0.9999986525	65.135356497
0.4862603763	0.9999986525	4.3843531651
0.7649013359	0.9999986525	242.1422639006
0.6398692135	0.9999986525	0
0.4729816619	0.9999986525	0
0.9878662278	0.9999986525	3.3247666494
0.545504561	0.9999986525	5.5622873329
0.911854226	0.9999986525	6.1669684991
0.5317459824	0.9999986525	12.5116875018
0.9199507532	0.9999986525	2.3561864031
0.9269613175	0.9999986525	49.1241488256
0.6910874009	0.9999986525	24.1159443956
0.3538734441	0.9999986525	73.4400928852
0.1353807337	0.9999986525	123.3764150122
0.56602673	0.9999986525	21.8963176726
0.268986823	0.9999986525	9.2623497415
0.7358006489	0.9999986525	87.6014124653
0.5918147342	0.9999986525	5.9616117199
0.3419366141	0.9999986525	4.7231191403
0.1023655029	0.9999986525	4.3183906972
0.0480904854	0.9999986525	80.9843107051
0.8786427525	0.9999986525	0

0.6686795107	0.9999986525	8.5661787112
0.4173882275	0.9999986525	17.0001882703
0.0687162472	0.9999986525	1148.1286570937
0.6171158189	0.9999986525	0
0.2089996067	0.9999986525	51.0371861823
0.4554044798	0.9999986525	308.4238828566
0.2238719595	0.9999986525	868.5802837059
0.9762686234	0.9999986525	18.7516130752
0.451542098	0.9999986525	174.2510799308
0.4257910135	0.9999986525	0
0.4312907055	0.9999986525	51.5257738793
0.337076529	0.9999986525	1.8543398982
0.2644332521	0.9999986525	19.7650154965
0.5312825543	0.9999986525	30.8492513004
0.3260717955	0.9999986525	36.5257268909
0.054957016	0.9999986525	51.3712424995
0.30755387	0.9999986525	0
0.5205358835	0.9999986525	22.1534891889
0.3143042905	0.9999986525	0
0.5870762626	0.9999986525	289.1272895601
0.7338030332	0.9999986525	5.4220951929
0.0992487681	0.9999986525	6.5323337324
0.5203354367	0.9999986525	47.1342420583
0.1693392999	0.9999986525	191.5475575405
0.8883525967	0.9999986525	6.7240331309
0.0191612185	0.9999986525	32.78127877
0.3602097011	0.9999986525	0
0.3388057848	0.9999986525	81.1273705473
0.0513394126	0.9999986525	10.9413545114
0.694189409	0.9999986525	50.3309258828
0.8936689295	0.9999986525	169.3529214769
0.0982440009	0.9999986525	14.6420621642
0.7063659384	0.9999986525	45.3727819814
0.0113159084	0.9999986525	18.4922682865
0.6937805563	0.9999986525	0
0.256902087	0.9999986525	4.5667028298
0.0380199695	0.9999986525	0
0.7919803464	0.9999986525	5927.289107427
0.2680018761	0.9999986525	0
0.6317177064	0.9999986525	7.7021902036
0.9858365275	0.9999986525	201.1664040264
0.5249044148	0.9999986525	4.1649712729
0.1027461815	0.9999986525	0
0.3501510933	0.9999986525	0
0.9242905064	0.9999986525	205.7876023291
0.2696753809	0.9999986525	0
0.9383228655	0.9999986525	32.9819010968
0.2267691656	0.9999986525	0
0.0540607086	0.9999986525	0

0.8702161007	0.9999986525	0.4159165726
0.339161753	0.9999986525	0
0.2994068291	0.9999986525	1.7233606249
0.7116783842	0.9999986525	0
0.8529894635	0.9999986525	3.8484184217
0.7809478687	0.9999986525	4.6152564719
0.5544405596	0.9999986525	21.1366640391
0.9332937337	0.9999986525	0
0.3378728042	0.9999986525	0
0.0770356906	0.9999986525	17.7168461007
0.3428860936	0.9999986525	1.8308287942
0.6730915763	0.9999986525	9.7538632528
0.1447114982	0.9999986525	4.6433900322
0.7335332555	0.9999986525	0.7381503337
0.9782832609	0.9999986525	0
0.3091866526	0.9999986525	0
0.5718715154	0.9999986525	0
0.3575599096	0.9999986525	0
0	0	0
0.5014490848	0.9999986525	0.3562802689
0.9773167614	0.9999986525	3.2000677549
0.3986743574	0.9999986525	0.4522320352
0.6911310044	0.9999986525	5.5846376469
0.1517965816	0.9999986525	22.7548118166
0.4099973746	0.9999986525	3.3744397048
0.3426284411	0.9999986525	0
0.3521531602	0.9999986525	0.8129041518
0.809091464	0.9999986525	31.8443884391
0.5280249505	0.9999986525	0
0.652277887	0.9999986525	0
0.6312664098	0.9999986525	0
0.8978742349	0.9999986525	0
0.4692214102	0.9999986525	2.3975811797
0.2842211412	0.9999986525	8.9984100569
0.356519626	0.9999986525	0
0.4280835565	0.9999986525	0.3434411246
0.9242289711	0.9999986525	0.7041311235
0.3213755118	0.9999986525	0
0.0762246345	0.9999986525	0
0.4900512299	0.9999986525	0.8679718678
0.2075825021	0.9999986525	351.66652572
0.7931274989	0.9999986525	5.3019634408
0.4501276024	0.9999986525	123.6795545475
0.7121847904	0.9999986525	1.8401549165
0.4123893117	0.9999986525	3408.6330949678
0.9317231264	0.9999986525	0
0.9074789761	0.9999986525	0.0659813555
0.5394715269	0.9999986525	0.1880308539
0.9693871621	0.9999986525	0.7820877755

0.0527524744	0.9999986525	3.7600682206
0.7033405507	0.9999986525	119.7051637042
0.2218974681	0.9999986525	6.6709508938
0.5210295095	0.9999986525	25.1568568424
0.460294576	0.9999986525	0
0.3977568139	0.9999986525	2.0256949304
0.9448079651	0.9999986525	3.5924577063
0.6440759525	0.9999986525	0.8869506214
0.7075908808	0.9999986525	0
0.4728797914	0.9999986525	0.2580770422
0	0	0
0.7587820318	0.9999986525	1.9330887678
0.9185732126	0.9999986525	6.3790703538
0.7042045085	0.9999986525	0
0.2414017569	0.9999986525	8.7404084777
0.0327960721	0.9999986525	16.1190044945
0.5780773363	0.9999986525	0
0.4002932538	0.9999986525	0.7477653404
0.6706396544	0.9999986525	96.9721721938
0.8765836621	0.9999986525	10.6036835676
0.4670259626	0.9999986525	23.2656333044
0.2650026715	0.9999986525	0
0.9922826766	0.9999986525	107.4239651499
0.345977696	0.9999986525	36.8662101516
0.8780373177	0.9999986525	1.3081332613
0.8987792644	0.9999986525	0
0.4540598555	0.9999986525	16.0858655985
0.0185515314	0.9999986525	19.9510152153
0.3947316573	0.9999986525	2.0750889235
0.1432412169	0.9999986525	20.921063891
0.718900735	0.9999986525	17.9592752303
0.3304414768	0.9999986525	0
0.0267841617	0.9999986525	3.7171958211
0.9028447761	0.9999986525	0
0.815506198	0.9999986525	7.2629729397
0.3127686522	0.9999986525	0
0.1916005796	0.9999986525	0
0.3638691525	0.9999986525	324.2106705114
0.031820719	0.9999986525	37.8377405261
0.6258688001	0.9999986525	0
0.0605202475	0.9999986525	136.0745075074
0.9419498707	0.9999986525	26.3411063473
0.3286650125	0.9999986525	10.8692978445
0.6398608601	0.9999986525	0
0.4371757463	0.9999986525	0
0.5904747806	0.9999986525	0
0.6312664098	0.9999986525	0
0.435199549	0.9999986525	0
0.97837326	0.9999986525	133.0245616495

0.0387098117	0.9999986525	68.3785961823
0.3835603041	0.9999986525	4.7554401428
0.4070928321	0.9999986525	540.4933151815
0.2836439151	0.9999986525	4.3183906972
0.7361406069	0.9999986525	0.4340322992
0.6065112158	0.9999986525	0.7146673644
0.7141650021	0.9999986525	0
0.9502271251	0.9999986525	0
0.4401619287	0.9999986525	1.4320088454
0	0	0
0.8541509155	0.9999986525	16.3916568052
0.8733809256	0.9999986525	26.6826801413
0.1125215984	0.9999986525	0
0.6323631054	0.9999986525	10.545007775
0.7327660479	0.9999986525	67.580189669
0.7750039706	0.9999986525	12.783746545
0.6307927463	0.9999986525	0
0.6387269284	0.9999986525	3.7201414568
0.6774815591	0.9999986525	0
0.5770431815	0.9999986525	101.2730934349
0.6801073256	0.9999986525	1.3303589064
0.3883474049	0.9999986525	0.6032360364
0.1899762161	0.9999986525	34.7231546524
0.9049473473	0.9999986525	141.9528145848
0.8521885278	0.9999986525	1.68691447
0.9730979603	0.9999986525	0.3497911623
0.9951848731	0.9999986525	2.5192191633
0.7244627322	0.9999986525	0
0.1756275412	0.9999986525	0.2219249361
0.8202860567	0.9999986525	27.9494175968
0.1627583842	0.9999986525	1.9339381972
0.4917825035	0.9999986525	5.9465088359
0.7151322454	0.9999986525	6.3497346831
0.5219520892	0.9999986525	362.3382135233
0.5041075335	0.9999986525	0
0.373386551	0.9999986525	11.6261927413
0.0170760223	0.9999986525	58.9112305926
0.0350192864	0.9999986525	5.5083352343
0.2056248422	0.9999986525	0
0.5626269935	0.9999986525	0
0.5124323193	0.9999986525	35.1975463021
0	0	0
0.1918417116	0.9999986525	0
0.6312664098	0.9999986525	0
0.8324023086	0.9999986525	0
0.2746270988	0.9999986525	0
0.6817342746	0.9999986525	0
0.6312664098	0.9999986525	0
0.7299918745	0.9999986525	0

0.4149230916	0.9999986525	0
0.9023685261	0.9999986525	0
0.4828932893	0.9999986525	0
0.2466180115	0.9999986525	0
0.7041998364	0.9999986525	0
0.1459773385	0.9999986525	7.0243728799
0.9985841367	0.9999986525	0
0.8507461597	0.9999986525	0
0.2545558254	0.9999986525	10.9379555196
0.8647617929	0.9999986525	0.1737528564
0.5641257468	0.9999986525	0
0.5723036299	0.9999986525	6.5388037918
0.8592488259	0.9999986525	2.0404341822
0.1307115615	0.9999986525	1.0452199376
0	0	0
0.8546564764	0.9999986525	10.6517981313
0.234455113	0.9999986525	2.7532487189
0.4317338855	0.9999986525	1.7568915946
0.6258340642	0.9999986525	0
0.142336853	0.9999986525	1.2258502263
0.8520867015	0.9999986525	2.0397380796
0.8510965796	0.9999986525	2.5506576947
0.5021351588	0.9999986525	0
0.6015918989	0.9999986525	68.7730975963
0.9228978443	0.9999986525	0
0.4123496102	0.9999986525	0
0.8286164921	0.9999986525	16.9792374944
0.4640666743	0.9999986525	0.2781641696
0.959844764	0.9999986525	50.2670541494
0.4648321648	0.9999986525	7.756486742
0.6688822966	0.9999986525	0
0.4676630026	0.9999986525	5.3963337311
0.2422406195	0.9999986525	0
0.992770849	0.9999986525	52.7435934351
0.3803837521	0.9999986525	1.6064825851
0.6137874426	0.9999986525	0
0.1414833857	0.9999986525	0.440044478
0.0307826867	0.9999986525	1.1729872439
0.3283686753	0.9999986525	3.739479695
0.2798975157	0.9999986525	44.7860235118
0.1519639079	0.9999986525	1.3619732635
0.6524578896	0.9999986525	0.8324520005
0.4686221523	0.9999986525	0
0.60892684	0.9999986525	31.7161298909
0.6359827147	0.9999986525	1.3470017528
0.4825520422	0.9999986525	0
0.8212094637	0.9999986525	0
0.3271622394	0.9999986525	1118.6570081006
0.5930344048	0.9999986525	2.2615474768

0.4033394695	0.9999986525	0
0.6395355923	0.9999986525	0
0.7139340357	0.9999986525	1.5985567815
0.005003	0.9999986525	5.5780184481
0.2508453488	0.9999986525	5.7049100851
0.470872166	0.9999986525	0
0.8765787203	0.9999986525	11.716898109
0.6133201956	0.9999986525	5.5372939053
0.8197822817	0.9999986525	0
0.1205104399	0.9999986525	5.2462420012
0	0	8.7541717597
0.5504798237	0.9999986525	6.3326041039
0.5324086143	0.9999986525	8.1857568077
0.1707194184	0.9999986525	0
0.401423877	0.9999986525	0
0.3485500105	0.9999986525	0
0.0302153696	0.9999986525	57.5210581517
0.512103345	0.9999986525	6.0715423432
0.1639715517	0.9999986525	6.1556925268
0.7180805998	0.9999986525	13.6285541609
0.8191721043	0.9999986525	0
0.5793093601	0.9999986525	9.8908642661
0.8693771448	0.9999986525	0
0.883061881	0.9999986525	6.8881457475
0.0624444511	0.9999986525	4.4248644834
0.9562193706	0.9999986525	3.7407598701
0.8717277808	0.9999986525	1.8230051716
0.2172206051	0.9999986525	0
0.6395238085	0.9999986525	0
0.9945527571	0.9999986525	0
0.8060334024	0.9999986525	0
0.0925974589	0.9999986525	1.8504578756
0.56824672	0.9999986525	0.9875636834
0.5385626271	0.9999986525	1.5918355002
0.3921019829	0.9999986525	1.7818861724
0.5928352088	0.9999986525	21.5278301744
0.4118711597	0.9999986525	12.3628085736
0.4069330981	0.9999986525	0
0.6146423746	0.9999986525	29.7836843233
0.707838028	0.9999986525	24.0021302899
0.1036480023	0.9999986525	1.5388521351
0.5910053919	0.9999986525	0
0.2774388625	0.9999986525	0
0.6648321212	0.9999986525	0
0	0	0
0.0965579354	0.9999986525	52.9197577418
0.7894634044	0.9999986525	3.0495137207
0.3790682952	0.9999986525	0
0.6891714805	0.9999986525	0

0.33506773	0.9999986525	0.4830313039
0.9091379889	0.9999986525	0.7138559449
0.4783248179	0.9999986525	0
0.8621953256	0.9999986525	0
0.7247972528	0.9999986525	0
0.2524675641	0.9999986525	6.4598254871
0.45754575	0.9999986525	3.8473545403
0.6445594043	0.9999986525	0
0.3144883577	0.9999986525	3.7507221239
0.6823651753	0.9999986525	2.2004882391
0.5451468814	0.9999986525	0
0.6104802026	0.9999986525	3.9748564933
0.5700342595	0.9999986525	0
0.5177842143	0.9999986525	0.6279447153
0.3164701903	0.9999986525	0
0.9291844004	0.9999986525	3.1435920494
0.511959707	0.9999986525	0
0.5469264813	0.9999986525	13.5067678901
0.9584444948	0.9999986525	5.6811558205
0.4378109456	0.9999986525	151.6482363464
0.7896835911	0.9999986525	27.9999566467
0.8883406633	0.9999986525	11.1410016299
0.8169173206	0.9999986525	2.2097873735
0.77426323	0.9999986525	5.5864097976
0.4550435389	0.9999986525	0
0.7061798235	0.9999986525	2.4551671162
0.1413304869	0.9999986525	0
0.4639657243	0.9999986525	34.749462695
0.0978781737	0.9999986525	57.1991894148
0.8623937078	0.9999986525	133.1123719098
0.6611175213	0.9999986525	0
0.6982499557	0.9999986525	1.5555236942
0.0664192057	0.9999986525	1.3217357782
0.3876844844	0.9999986525	1.0407144795
0.2409608932	0.9999986525	9.3378634665
0.3795294479	0.9999986525	2.1537361645
0.967493688	0.9999986525	0
0	0	0
0.4838543539	0.9999986525	0
0.221868534	0.9999986525	12.1817364148
0.724707554	0.9999986525	0
0.4069584982	0.9999986525	0
0.8571521079	0.9999986525	21.4865411824
0.6113522276	0.9999986525	0
0.1598773034	0.9999986525	14.4203338852
0.1998364512	0.9999986525	59.4607844928
0.6707142825	0.9999986525	0
0.5682048876	0.9999986525	0
0.7233414714	0.9999986525	7.0819139743

0.950331098	0.9999986525	0.4790334621
0.3763589764	0.9999986525	2.1593277685
0.6312664098	0.9999986525	0
0.0363985612	0.9999986525	4.5037268701
0.3420201226	0.9999986525	0
0.0460740423	0.9999986525	24.810966711
0.550923814	0.9999986525	0
0.702570583	0.9999986525	4.742842102
0.2686861065	0.9999986525	16.1480823783
0.9037840505	0.9999986525	0
0.2991280906	0.9999986525	1.0076203765
0.3368702551	0.9999986525	0
0.260848468	0.9999986525	1.6230260915
0.840652386	0.9999986525	5.1145302725
0.9294108232	0.9999986525	0
0.0770122044	0.9999986525	131.7363304421
0.5472709787	0.9999986525	0
0.7887972103	0.9999986525	0.2460661605
0.6398524973	0.9999986525	0
0.1276629786	0.9999986525	0.3181408018
0.46271102	0.9999986525	4.6831039998
0.3103158083	0.9999986525	0
0.2787982871	0.9999986525	2.2962573142
0.9641850171	0.9999986525	0
0.0423914691	0.9999986525	79.1356543271
0.7405431276	0.9999986525	0
0.24856022	0.9999986525	1.367917279
0.2081506145	0.9999986525	0.3919691126
0.2831170366	0.9999986525	4.0993985687
0.4279014293	0.9999986525	2.2686634619
0.5508838458	0.9999986525	1.1845403989
0.1080736279	0.9999986525	3.3424415054
0.7449108084	0.9999986525	0.422406589
0.3419656432	0.9999986525	0
0.6312664098	0.9999986525	0
0.3613378695	0.9999986525	8.0112124727
0.4184424298	0.9999986525	0.9376472362
0.4067357476	0.9999986525	4.9169247738
0.2922728399	0.9999986525	12.3080676361
0.542125351	0.9999986525	12.2311065832
0.2288621114	0.9999986525	0
0.9726790924	0.9999986525	1.9474876738
0.7636478773	0.9999986525	2.4335968218
0.4730970736	0.9999986525	0
0.2915108211	0.9999986525	3.5146015989
0.8525754148	0.9999986525	796.3407300126
0.3419112382	0.9999986525	0
0.0797447889	0.9999986525	0
0.2585419053	0.9999986525	0.8786991286

0.7046536483	0.9999986525	0
0	0	0
0.2184879093	0.9999986525	0
0.3448064682	0.9999986525	4.2337842482
0.282668908	0.9999986525	1.0212297592
0.2197686467	0.9999986525	170.3827454205
0.2555233538	0.9999986525	6.2094573126
0.905071528	0.9999986525	2.4571470283
0.3956910373	0.9999986525	0
0.6008253812	0.9999986525	0
0.1460357505	0.9999986525	0.2667868148
0.3618081293	0.9999986525	0
0.4093190019	0.9999986525	7.9318041941
0	0	0
0.268301557	0.9999986525	2.7607614257
0.3914129405	0.9999986525	0.4650340603
0.8339051841	0.9999986525	13.0240372654
0.8799134753	0.9999986525	11.8141370406
0.8802612271	0.9999986525	6.8416242278
0.5212433244	0.9999986525	0
0.5990864635	0.9999986525	0
0.7094228215	0.9999986525	1.5407676007
0.6618937306	0.9999986525	37.4863934636
0.8904180862	0.9999986525	0.4008251044
0.4624760823	0.9999986525	10.1185780681
0.8242290199	0.9999986525	0.241547685
0.7012231009	0.9999986525	0.3800478405
0.995578436	0.9999986525	30.8921068809
0	0	0
0.149630902	0.9999986525	12.5200912429
0.2650122493	0.9999986525	0.8972498085
0.4606009042	0.9999986525	8.1888391699
0.3368687627	0.9999986525	0
0.4394756567	0.9999986525	20.9595675158
0.7667774036	0.9999986525	0.9459604561
0.6919692517	0.9999986525	0
0.3932654765	0.9999986525	0
0.3533397775	0.9999986525	1.5243695803
0.6892627996	0.9999986525	1.4341086221
0	0	0
0.3547091322	0.9999986525	1.3431210528
0.4854103928	0.9999986525	0
0.5925097331	0.9999986525	14.479178737
0.9343801294	0.9999986525	34.9202639501
0.5862468318	0.9999986525	0
0.7082433124	0.9999986525	1.2473960752
0.5412895526	0.9999986525	1.3996374152
0.7356553307	0.9999986525	0
0.3515926008	0.9999986525	1448.7285920516

0.7908506525	0.9999986525	64.258737596
0.5000834963	0.9999986525	0
0.369644987	0.9999986525	1.0512742989
0.6685094608	0.9999986525	12.1783656297
0.1532284504	0.9999986525	6.2748588501
0.5070248526	0.9999986525	1356.6878076483
0.8227340813	0.9999986525	1.0233470563
0.5386690087	0.9999986525	0.5871670898
0.6853406139	0.9999986525	1.9201569979
0.6173309287	0.9999986525	3.8101473765
0.2906764722	0.9999986525	1.5470259301
0.9897285126	0.9999986525	2.0946267243
0.9842684804	0.9999986525	0.1712552432
0.914096047	0.9999986525	0
0.4146895838	0.9999986525	0
0.5029978495	0.9999986525	899.1994590274
0.3143353428	0.9999986525	1.0153398613
0.8599482279	0.9999986525	4.3013914668
0.3630288428	0.9999986525	3.2799601432
0.5226607261	0.9999986525	2.4348309541
0.6312664098	0.9999986525	0
0.8772026847	0.9999986525	27.3363965975
0.6293796193	0.9999986525	0
0.8526918986	0.9999986525	22.2047165131
0.0372694256	0.9999986525	49.8800635777
0.9709242289	0.9999986525	11.9542594446
0.8747404524	0.9999986525	0
0.3768513583	0.9999986525	2.4673636172
0.155112397	0.9999986525	28.8065442491
0.2126946451	0.9999986525	4.7696124109
0.1300424467	0.9999986525	46.7379807312
0.8196742132	0.9999986525	3.9658028304
0.8330409734	0.9999986525	8.6750851226
0.1802214546	0.9999986525	5.145173358
0.6169273234	0.9999986525	47.5801065102
0.8217222139	0.9999986525	35.1780094758
0.470283115	0.9999986525	6.8913487077
0.1706907406	0.9999986525	0
0.0719578231	0.9999986525	31.4072559946
0.3522218874	0.9999986525	15.5209516498
0.5249282638	0.9999986525	0
0.9569300453	0.9999986525	13.6740963734
0.8384628692	0.9999986525	0
0.0501065568	0.9999986525	71.4460194384
0.5498819539	0.9999986525	0
0.6178778387	0.9999986525	8.0128620136
0.4747316799	0.9999986525	50.5530724846
0.3134635335	0.9999986525	0.6511554745
0.5975281972	0.9999986525	18.2132660492

0.7179504586	0.9999986525	798.4209774282
0.1072144119	0.9999986525	2.8665826219
0.3506865148	0.9999986525	48.4531359874
0.6569332275	0.9999986525	6.1811149087
0.6416178419	0.9999986525	2.273913641
0.5954627677	0.9999986525	49.4165316737
0.9727466193	0.9999986525	11.5998063313
0.1919578757	0.9999986525	2.1291642099
0.0493733417	0.9999986525	4.4096116078
0.6312664098	0.9999986525	0
0.7644173067	0.9999986525	47.9255156319
0.9048210836	0.9999986525	12.4468866603
0.6788357278	0.9999986525	5.1370409713
0.2516395498	0.9999986525	4.4659156288
0.528870357	0.9999986525	11.3152476114
0.7165032115	0.9999986525	6.6025650989
0.5549117533	0.9999986525	6.9495655332
0.5030281013	0.9999986525	58.4655921324
0.1504839981	0.9999986525	1.8957578602
0.9770657501	0.9999986525	69.8042799713
0.9299983383	0.9999986525	0
0.8020604336	0.9999986525	0
0.5324969173	0.9999986525	31.8847610639
0.9628281789	0.9999986525	14.1201100329
0.7042468279	0.9999986525	375.8763065176
0.2888879889	0.9999986525	3.4130747206
0.5941735774	0.9999986525	12.358951223
0.6394896617	0.9999986525	6.1081974262
0.7687271314	0.9999986525	1.6318976077
0.2872021064	0.9999986525	0
0.4816657423	0.9999986525	2.8009078397
0.005653	0.9999986525	5.3904587718
0.8834636894	0.9999986525	0
0.926957038	0.9999986525	4.743055072
0.9876128769	0.9999986525	2.431090341
0.5818937218	0.9999986525	0
0.5060456771	0.9999986525	0
0	0	0
0.8544331852	0.9999986525	2.4202143812
0.030886599	0.9999986525	8.8565661722
0.3775725673	0.9999986525	3.7669337698
0.0276761258	0.9999986525	0
0.8536198172	0.9999986525	7.0606842549
0.6312664098	0.9999986525	0
0.8099584209	0.9999986525	2.9107665328
0.6239179613	0.9999986525	2.4030575502
0.9957009334	0.9999986525	1.9020390473
0.4869400389	0.9999986525	1.6627845842
0.8074907184	0.9999986525	5.0613900677

0.8282397234	0.9999986525	0
0.6872866611	0.9999986525	2.729154636
0.6529640647	0.9999986525	25.431042844
0.6338578233	0.9999986525	0.3446528813
0.1560385707	0.9999986525	4.4029944332
0.988996023	0.9999986525	1.9589005685
0.2014824137	0.9999986525	32.2626256728
0.3025326533	0.9999986525	0
0.7042274973	0.9999986525	18.4588751301
0.4910602497	0.9999986525	0
0.8841981505	0.9999986525	124.7421760432
0.0824745788	0.9999986525	43.671927971
0.9324609449	0.9999986525	6429.0915400022
0.5270387038	0.9999986525	45.1304577879
0.9013129023	0.9999986525	22.8582203966
0.3683279565	0.9999986525	54.4110423518
0.2641048877	0.9999986525	0
0.4641805431	0.9999986525	56.6719716832
0.1967586123	0.9999986525	0
0.3373383809	0.9999986525	0
0.1708834054	0.9999986525	0
0.6312664098	0.9999986525	0
0.3418569075	0.9999986525	0
0.4490098348	0.9999986525	1.6040256142
0.0742220521	0.9999986525	38.6824062972
0.4392757457	0.9999986525	45.6529300548
0.2369361196	0.9999986525	10.1810854945
0.223887471	0.9999986525	0
0.1699016321	0.9999986525	6.3053866077
0.285860054	0.9999986525	4.6966579125
0.3722153038	0.9999986525	11.1515874633
0.5447466164	0.9999986525	225.459629568
0.6312664098	0.9999986525	0
0	0	0
0.9020141505	0.9999986525	2.1324955626
0.6362639813	0.9999986525	29.7417531895
0.4206101865	0.9999986525	2.9754655391
0.00379	0.9999986525	441.6494903942
0.1520856173	0.9999986525	0
0	0	0
0.4797193973	0.9999986525	2.6735132869
0.4069838796	0.9999986525	0
0.6312664098	0.9999986525	0
0.7494795087	0.9999986525	55.2446843371
0.4019121666	0.9999986525	0
0.2439577842	0.9999986525	4.2851049271
0.0843040613	0.9999986525	66.922370751
0.3820004342	0.9999986525	32.0351312314
0.2170963883	0.9999986525	0

0.1535870111	0.9999986525	17.8196633465
0.2470471983	0.9999986525	5.142142601
0.8064493475	0.9999986525	7.6414552885
0.9891692847	0.9999986525	62.7112251063
0.1779670293	0.9999986525	39.1310829042
0.2443923954	0.9999986525	0
0.6809019917	0.9999986525	0
0.599465419	0.9999986525	185.4979566403
0.3007306145	0.9999986525	4.6311748708
0.750860685	0.9999986525	17.4638700321
0.1623565163	0.9999986525	142.2277869649
0.2582677415	0.9999986525	0
0.8376377266	0.9999986525	225.1126043272
0.3572929235	0.9999986525	1.1778304689
0.6364561056	0.9999986525	182.4525042364
0.4710672114	0.9999986525	0
0.1861784708	0.9999986525	33.8767916782
0	0	0
0.8263904841	0.9999986525	0
0.6963347182	0.9999986525	0
0.5840456511	0.9999986525	39.6054879837
0.8728450337	0.9999986525	46.8208494914
0.1709010412	0.9999986525	0
0.5289544668	0.9999986525	37.5010070246
0.3483775414	0.9999986525	29.9059559605
0.188474754	0.9999986525	11.8278953297
0.3485352324	0.9999986525	42.586145593
0.9995870086	0.9999986525	0
0.3368672726	0.9999986525	0
0.1560767576	0.9999986525	477.6838279372
0.4323294906	0.9999986525	5.8069178918
0.8495000475	0.9999986525	271.3624571679
0.7370535031	0.9999986525	3.8564379866
0.0513810461	0.9999986525	0
0.1861908144	0.9999986525	0
0.4070092421	0.9999986525	0
0.0508348485	0.9999986525	2738.8189525281
0.757374142	0.9999986525	0
0.0881605718	0.9999986525	92.6103010161
0.5075793495	0.9999986525	76.7963081812
0.3277469958	0.9999986525	40.3005862086
0.2343739446	0.9999986525	1.3615460705
0.8528695859	0.9999986525	0
0.5648490208	0.9999986525	17.9212481257
0.2822013198	0.9999986525	0
0.6778040912	0.9999986525	1511.1278462464
0.5796147661	0.9999986525	19.7747332877
0.4603702075	0.9999986525	18.846408312
0.2350847795	0.9999986525	27.16485867

0.5209590203	0.9999986525	1.459351153
0.6927933713	0.9999986525	1.7609628804
0.7409701399	0.9999986525	6.1463603316
0.3969874205	0.9999986525	0
0.2408810251	0.9999986525	6.760884665
0.0606979894	0.9999986525	39.5744618862
0.5400731111	0.9999986525	0
0.00277	0.9999986525	21.372264284
0.9302032194	0.9999986525	0.6998743504
0.2483243333	0.9999986525	0.0942829568
0.1586932153	0.9999986525	0
0.8832367934	0.9999986525	11.4264726226
0.9289741414	0.9999986525	7.5201364412
0.844419816	0.9999986525	62.5622550602
0	0	0
0.6312664098	0.9999986525	0
0.3153383123	0.9999986525	0
0.1402280567	0.9999986525	9.0850932312
0.4307141508	0.9999986525	3.8079179658
0.9171282003	0.9999986525	2.6747318744
0.8532154313	0.9999986525	51.3626501539
0.8066743063	0.9999986525	0
0.3137124787	0.9999986525	2.199480186
0.7588181541	0.9999986525	0
0.2697286363	0.9999986525	0
0.7125719905	0.9999986525	37.09624094
0.2593730198	0.9999986525	4.3271574466
0.8395449516	0.9999986525	1.4075528953
0.753509887	0.9999986525	0.8354550603
0.9092311386	0.9999986525	1.4248615504
0.3601710733	0.9999986525	21.4494540466
0.9515948985	0.9999986525	0.7995849445
0.6312664098	0.9999986525	0
0.7802057575	0.9999986525	1.5104173667
0.2346820651	0.9999986525	0.3491136847
0.085981805	0.9999986525	0
0.7421161922	0.9999986525	0
0.8872120102	0.9999986525	2.1584452756
0.4069662402	0.9999986525	2.5731715999
0.5892016238	0.9999986525	5.8345026763
0.5943692368	0.9999986525	0
0.785473975	0.9999986525	20.5655001692
0.660382378	0.9999986525	14.5016158706
0.4630417763	0.9999986525	0.4346538354
0.2629316312	0.9999986525	0
0.666141592	0.9999986525	13.8274429183
0.1002752261	0.9999986525	4.2628825979
0.2831013051	0.9999986525	2.364625844
0.0724422957	0.9999986525	15.397145024

0.4729816619	0.9999986525	0
0.9297723355	0.9999986525	2.2272606527
0.193088481	0.9999986525	0
0.9685306707	0.9999986525	2.2663292274
0.1133110731	0.9999986525	0
0.7753658221	0.9999986525	18.8722237721
0.2376937032	0.9999986525	0
0.8043441258	0.9999986525	2.8003137978
0.0413384418	0.9999986525	0
0.1714888816	0.9999986525	14.9234015898
0.4965007972	0.9999986525	1.6467041305
0.4218810247	0.9999986525	0
0.2584127591	0.9999986525	1.3753087308
0.5735424187	0.9999986525	0
0.2681911931	0.9999986525	2.5601444571
0.85303971	0.9999986525	0.8089499935
0.0226284571	0.9999986525	43.7427627923
0.159155566	0.9999986525	1.9263903643
0.2586737413	0.9999986525	0.7264637995
0.6395120645	0.9999986525	0
0.639844125	0.9999986525	0
0.4436508696	0.9999986525	0
0.8835969582	0.9999986525	0
0.9117553486	0.9999986525	2.3678062419
0.3068962215	0.9999986525	0
0.4729816619	0.9999986525	0
0.714376115	0.9999986525	0
0.1756238409	0.9999986525	1.9226734947
0.881418352	0.9999986525	335.5395058851
0.0006292	0.9999986525	0
0.1306981733	0.9999986525	364.9073758338
0.7137147449	0.9999986525	0
0.7747947712	0.9999986525	4.7014136778
0.336484017	0.9999986525	0
0.9392015858	0.9999986525	5.9753263946
0.4438442904	0.9999986525	0
0.9536518769	0.9999986525	0
0.9083786293	0.9999986525	1.4896777651
0.7007817031	0.9999986525	0
0.2319016736	0.9999986525	0
0.01815353	0.9999986525	4.0765320191
0.299455622	0.9999986525	4.2785959069
0.2302066493	0.9999986525	2.3064457878
0.6217165113	0.9999986525	0.3254012115
0.2048173524	0.9999986525	11.8173091887
0.0997519023	0.9999986525	6.3087759931
0.2169025797	0.9999986525	0
0.5713496927	0.9999986525	15.2625341659
0	0	0

0.4099843565	0.9999986525	0
0.7443432812	0.9999986525	0.6285525468
0.7086443461	0.9999986525	23.2950337871
0.4686800015	0.9999986525	116.3943101406
0.3326830721	0.9999986525	0.5665432615
0.2431215694	0.9999986525	0
0.1328120571	0.9999986525	6.6788808521
0.6695057466	0.9999986525	1.8988932595
0.9077103588	0.9999986525	0.8437941274
0.1298976836	0.9999986525	0
0.5628162533	0.9999986525	1.2831473747
0.5827855742	0.9999986525	21.1549615529
0.4415863365	0.9999986525	1.901782207
0.5018315189	0.9999986525	0.6045784546
0.98011302	0.9999986525	10.00815016
0.692542587	0.9999986525	0.671015065
0.3360669815	0.9999986525	0
0.9332567613	0.9999986525	0
0.0188034656	0.9999986525	1.7850228807
0.8210715438	0.9999986525	0.6132738594
0.4274858306	0.9999986525	1.6216008544
0.6746834632	0.9999986525	0
0.9448797969	0.9999986525	3.7282167321
0.412008428	0.9999986525	5.8500130955
0.5629330331	0.9999986525	6.3878789154
0.4147131084	0.9999986525	5.6619706141
0.5990952698	0.9999986525	0
0.3492154261	0.9999986525	22.1602494846
0.9000153205	0.9999986525	0
0.4555848647	0.9999986525	5.6348069122
0.4895090187	0.9999986525	0
0.4104452258	0.9999986525	223.484758913
0.7766538853	0.9999986525	0
0.755722215	0.9999986525	0
0.3925935806	0.9999986525	16.6119495736
0.8261020683	0.9999986525	0
0.1244286477	0.9999986525	46.8936010888
0.7278931336	0.9999986525	0
0.5217852133	0.9999986525	1.1291217739
0.317734465	0.9999986525	0
0.5879880153	0.9999986525	0.8027041299
0	0	0
0.7298011663	0.9999986525	3.2068685546
0.6468407562	0.9999986525	11.8151942341
0.8996965585	0.9999986525	65.9478434168
0	0	0
0.0167420138	0.9999986525	0.1050569569
0.3030454714	0.9999986525	0
0.326618375	0.9999986525	0.1008116602

0.6503511544	0.9999986525	0
0	0	0
0.3700734422	0.9999986525	1.193786672
0.6996850614	0.9999986525	0.8857175142
0.5481889872	0.9999986525	0
0.6312664098	0.9999986525	0
0.4093837052	0.9999986525	1.5599113396
0.9620927176	0.9999986525	0.6209092304
0.014805319	0.9999986525	0
0.6398273518	0.9999986525	0
0	0	0
0.6895643234	0.9999986525	0
0.545711	0.9999986525	0.4267687913
0.1826665978	0.9999986525	1.1566273537
0.7706448343	0.9999986525	3.603569748
0.9838689634	0.9999986525	1.6096406152
0.445249854	0.9999986525	0
0.7900921204	0.9999986525	0
0.0636750405	0.9999986525	38.5503052747
0.7825032158	0.9999986525	130.5336584931
0.8150931455	0.9999986525	2.0229792096
0.1335305643	0.9999986525	14.7717466604
0.4144172395	0.9999986525	0
0.1421705406	0.9999986525	26.2420093402
0.7160085406	0.9999986525	0.3316748824
0	0	0
0.7222020317	0.9999986525	0.890382329
0.749081834	0.9999986525	3.1354204697
0.0359451504	0.9999986525	63.9316284061
0.8752010282	0.9999986525	0
0	0	0
0.4870171935	0.9999986525	0
0.2634659854	0.9999986525	141.8204034004
0.0902974251	0.9999986525	7.3639038241
0.6558878915	0.9999986525	7.2729747385
0.0378468582	0.9999986525	0.2976647827
0.30755387	0.9999986525	0
0.3682328758	0.9999986525	0
0.9773880138	0.9999986525	7.8307808295
0.4762533447	0.9999986525	3.0975905118
0.929900234	0.9999986525	0
0.203402674	0.9999986525	0
0.9422691491	0.9999986525	114.2112512588
0.5545690148	0.9999986525	0.8281028933
0.6946409525	0.9999986525	1.8069013044
0.3842704358	0.9999986525	1.1103992226
0.7438862911	0.9999986525	1.1282212745
0.4589276008	0.9999986525	20.0181906409
0.3447733189	0.9999986525	0

0.9051987392	0.9999986525	11.5157085258
0.4546815676	0.9999986525	204.3444516305
0.1473678474	0.9999986525	1.8686593152
0.2195218831	0.9999986525	148.604788104
0.5449176458	0.9999986525	9.456574567
0.872115966	0.9999986525	3.6555748902
0.1651240443	0.9999986525	0
0.1476955947	0.9999986525	5.606445525
0.2614603286	0.9999986525	0
0.9294773649	0.9999986525	0
0.6398105398	0.9999986525	0
0.6471840166	0.9999986525	0
0.5474737213	0.9999986525	36.1739165934
0.8628483753	0.9999986525	44.1718296688
0.2196755313	0.9999986525	75.9291750354
0.7988412922	0.9999986525	0.631133928
0.8731218907	0.9999986525	36.6254634024
0.4069883814	0.9999986525	0
0.0942260233	0.9999986525	0.9610481075
0	0	0
0.7760191379	0.9999986525	0
0.8423732642	0.9999986525	6.4732855188
0.0608570783	0.9999986525	24.3494321007
0.2785181718	0.9999986525	1.1965634652
0.3358449838	0.9999986525	0
0.7154104474	0.9999986525	0
0.4819644665	0.9999986525	12.1761194758
0.0914353925	0.9999986525	203.1709731443
0.6312664098	0.9999986525	0
0.5084797278	0.9999986525	0.4386173759
0.4089071717	0.9999986525	31.9101263137
0.2587506737	0.9999986525	448.2086412417
0.460952246	0.9999986525	0
0.7628832858	0.9999986525	1.8361826738
0.8067559013	0.9999986525	1.8904165776
0.009269	0.9999986525	0
0.3296570674	0.9999986525	3.530420791
0.5813912451	0.9999986525	2.4617282664
0.5287608034	0.9999986525	1.5731537658
0.9290095376	0.9999986525	0.7128153947
0.2403359038	0.9999986525	98.2036411795
0.4091751074	0.9999986525	260.5724347702
0.3670064036	0.9999986525	0
0.3707240406	0.9999986525	16.25668895
0.9288192709	0.9999986525	0
0.6663713434	0.9999986525	4.4309208707
0.6980469521	0.9999986525	0.4098677456
0.2509366464	0.9999986525	2.2452987937
0.3927942307	0.9999986525	5.6562100057

0.4604708313	0.9999986525	0
0.5012581703	0.9999986525	594.5281601461
0.4070628564	0.9999986525	3.255697092
0.6862429433	0.9999986525	15.9703411631
0.2606315063	0.9999986525	5.9541226198
0.581406359	0.9999986525	19.0434801297
0.1483431508	0.9999986525	0
0.6235138653	0.9999986525	0.43273426
0.4773418832	0.9999986525	6.8524983913
0.5251906308	0.9999986525	0.9462841836
0.1188798494	0.9999986525	0
0.7739328907	0.9999986525	1.1141941779
0	0	0
0.9057589697	0.9999986525	0.6399204942
0.485863833	0.9999986525	59.5226126993
0.6166520002	0.9999986525	181.0467324114
0.2558135371	0.9999986525	77.9541228433
0.7687393831	0.9999986525	0.0469152623
0.3702663368	0.9999986525	0
0.6983594836	0.9999986525	2.7222867337
0.6034398973	0.9999986525	2.9129606317
0.4175022523	0.9999986525	279.48720498
0.2151293488	0.9999986525	1.0667969828
0.2759778169	0.9999986525	275.1074383533
0.9701355278	0.9999986525	1.668607119
0.1843056134	0.9999986525	123.7826208429
0	0	0
0.971713706	0.9999986525	3.7976499767
0.3741872446	0.9999986525	4.2160068832
0.1413969746	0.9999986525	19.3999892953
0.0616962481	0.9999986525	0
0.2306737203	0.9999986525	13.9055149283
0.240591648	0.9999986525	0.4860519444
0.1864474543	0.9999986525	145.801855371
0.6727734277	0.9999986525	0
0.7357274593	0.9999986525	0.5560413365
0.4033358126	0.9999986525	0
0.99334836	0.9999986525	7.321623118
0.6010100773	0.9999986525	0
0.9266527712	0.9999986525	0
0.1463014093	0.9999986525	3.5330186567
0.9127711543	0.9999986525	7.6233346926
0.3114112721	0.9999986525	0
0.1021759511	0.9999986525	2.0846227838
0.6319096759	0.9999986525	0
0.2188269454	0.9999986525	2.3516226919
0.2875824087	0.9999986525	0
0.0581202503	0.9999986525	18.4982339192
0.9113075435	0.9999986525	0.1296355653

0.6398021191	0.9999986525	0
0	0	0
0.3800156094	0.9999986525	0
0.1126264935	0.9999986525	0
0.9698350696	0.9999986525	0
0.8353110555	0.9999986525	1.0859687502
0.5857971278	0.9999986525	6.8641207957
0.6124931888	0.9999986525	0
0.1506841345	0.9999986525	16.0650701829
0.0410328732	0.9999986525	7.5762327107
0.7053374491	0.9999986525	0
0.5198686894	0.9999986525	3.1971619484
0.0867298508	0.9999986525	3.599934443
0	0	0
0.4964586203	0.9999986525	7.4690875727
0.4729816619	0.9999986525	0
0.2193181405	0.9999986525	1.7326121998
0.031070075	0.9999986525	2.3619116108
0.9318364431	0.9999986525	18.1241998031
0.8393152822	0.9999986525	0.9850586259
0.9261768828	0.9999986525	0
0.5126318135	0.9999986525	4.2047958844
0.3933216553	0.9999986525	0
0.3702586551	0.9999986525	2.5420638345
0.8263272532	0.9999986525	18.4406139058
0.6203888315	0.9999986525	0.2269965196
0.4729816619	0.9999986525	0
0.9545304697	0.9999986525	0.5998991143
0.7451917742	0.9999986525	3.2517882108
0.730325233	0.9999986525	9.1347220916
0.9809294333	0.9999986525	0.157437774
0.0470860324	0.9999986525	5.3255942549
0.4729816619	0.9999986525	0
0.1614666614	0.9999986525	3.7501228925
0.6397936884	0.9999986525	0
0.6312664098	0.9999986525	0
0	0	0
0	0	0
0.4951076992	0.9999986525	30.101089355
0.6093786801	0.9999986525	8.5881875612
0.7039041661	0.9999986525	0.6349124007
0.3548843914	0.9999986525	0
0.5218683824	0.9999986525	0
0.500083171	0.9999986525	2.9303622164
0.6083413344	0.9999986525	4.365628487
0.3012392684	0.9999986525	0
0.2418085103	0.9999986525	0
0.511533562	0.9999986525	0
0.6011985578	0.9999986525	49.0273235351

0.2526704012	0.9999986525	7.8881892784
0.5535886536	0.9999986525	1.9976215742
0.8099997624	0.9999986525	11.6202240322
0.3675420803	0.9999986525	0
0.9489443145	0.9999986525	8.2403526261
0.3753988602	0.9999986525	39.8445252556
0.3474914356	0.9999986525	191.6151228165
0.1847428013	0.9999986525	7.0938775321
0.4743046041	0.9999986525	0
0.4476782866	0.9999986525	324.6757268011
0.1431541562	0.9999986525	5.2244453681
0.9835158302	0.9999986525	4.6987471732
0.1619002377	0.9999986525	40.4544356457
0.9527492666	0.9999986525	13.1434214226
0.2499534443	0.9999986525	0
0.6727261991	0.9999986525	0
0.7340778099	0.9999986525	4.4320611676
0.460148864	0.9999986525	0
0.0963867944	0.9999986525	0
0.4047262285	0.9999986525	11.1014247754
0.3368825518	0.9999986525	0
0.9157372514	0.9999986525	0
0.7645294352	0.9999986525	0.957728139
0.341802651	0.9999986525	0
0.3667548929	0.9999986525	15.0148549779
0.4232641607	0.9999986525	41.1991821306
0.408733513	0.9999986525	0
0.7819136582	0.9999986525	7.7887444293
0.9729916178	0.9999986525	0
0.3076777365	0.9999986525	0
0.0829687483	0.9999986525	20.9164032579
0.5565636176	0.9999986525	8.8254866067
0.2260794894	0.9999986525	1.4847047742
0.4307264497	0.9999986525	1.1790312367
0.6312664098	0.9999986525	0
0.5885941009	0.9999986525	46.9874717316
0.1990657235	0.9999986525	7.6703098143
0.5848499328	0.9999986525	2.9850177034
0.9121243363	0.9999986525	31.4485556986
0.8196057245	0.9999986525	296.4243039025
0.9991673216	0.9999986525	0
0.0121632608	0.9999986525	4.7733484373
0.6165044973	0.9999986525	571.1916902603
0.3102833537	0.9999986525	35.1292621648
0.1831175483	0.9999986525	0
0.337934731	0.9999986525	3.8476581897
0.5029592461	0.9999986525	81.6878504222
0.2980452614	0.9999986525	61.4383227876
0.6869253521	0.9999986525	4.9028888067

0.3406082665	0.9999986525	0
0.4621159996	0.9999986525	47.5182709224
0.0316158863	0.9999986525	7.5437677798
0.7071709561	0.9999986525	0
0.9245795163	0.9999986525	11.2501689693
0.7493132806	0.9999986525	4.9539366125
0.7406416854	0.9999986525	1.8721258469
0.0770082518	0.9999986525	7837.3933222384
0.9169443683	0.9999986525	27.7424498227
0.7889894946	0.9999986525	0
0.993181156	0.9999986525	0.752195722
0.5685418027	0.9999986525	39.5313759179
0.6265984827	0.9999986525	3.4611650977
0.455929951	0.9999986525	6.0915707297
0.589696782	0.9999986525	149.0132781693
0.5575399347	0.9999986525	54.7521441285
0	0	0
0.3961119274	0.9999986525	1.5375007125
0.9794820657	0.9999986525	0
0.3469142262	0.9999986525	0
0.4069215553	0.9999986525	0
0.4357960148	0.9999986525	5.6053829576
0.2344884461	0.9999986525	0
0.7308299872	0.9999986525	0
0.5541258295	0.9999986525	11.2241080942
0.7821615841	0.9999986525	1.6178256464
0.9010265918	0.9999986525	0
0.9349870136	0.9999986525	7.7844967538
0.6637848712	0.9999986525	4.2812839029
0.219555539	0.9999986525	8.6156473452
0.8824915358	0.9999986525	0
0.8318566433	0.9999986525	34.3840613453
0.6842639858	0.9999986525	0
0.4069197331	0.9999986525	0.9884385332
0.6508918192	0.9999986525	6.0192517686
0.4069675129	0.9999986525	24.7952667729
0.052229941	0.9999986525	2.1851473231
0.9620381275	0.9999986525	7.5044642959
0.230362307	0.9999986525	219.3504106398
0.5515268143	0.9999986525	8.5044855339
0.2283522162	0.9999986525	18.7449576668
0	0	0
0.9234954322	0.9999986525	0
0.5315886155	0.9999986525	1.6161053321
0.8411284051	0.9999986525	0
0.8691062166	0.9999986525	16.1690022123
0.1584359313	0.9999986525	0
0.0996155513	0.9999986525	0
0.6328104448	0.9999986525	0

0.9082161299	0.9999986525	111.4301642933
0.3516094716	0.9999986525	1.4150501896
0.2162493868	0.9999986525	12.8376077361
0.8488759742	0.9999986525	0.0846184333
0.7694510823	0.9999986525	0
0.7492499617	0.9999986525	20.6627223108
0.0203147865	0.9999986525	2.2762003603
0.1001704454	0.9999986525	0
0.4414249448	0.9999986525	5.9593694166
0.3394509012	0.9999986525	39.8428553258
0.8188205626	0.9999986525	3.8612310445
0.1121112418	0.9999986525	21.4435029611
0.3669101718	0.9999986525	0
0.1314725521	0.9999986525	22.4644484161
0.3115785614	0.9999986525	0
0.8524565404	0.9999986525	2.8003731906
0.5033860854	0.9999986525	0.8059256276
0.1487789171	0.9999986525	0
0.5916318054	0.9999986525	13.2448697746
0.7586166697	0.9999986525	0.7172571902
0.9472968593	0.9999986525	1.2143363889
0.2171200074	0.9999986525	0
0.3200781835	0.9999986525	2.0411964994
0.833538562	0.9999986525	0
0.2750496379	0.9999986525	0
0.8434584851	0.9999986525	0
0.3962922064	0.9999986525	0
0.30755387	0.9999986525	0
0	0	0
0.3097287388	0.9999986525	0.9466459453
0.8191642191	0.9999986525	3.3818655695
0.4081334392	0.9999986525	1.3374675402
[truncated: 418,809 more chars]
